# Identification of miRNAs and their targets using high-throughput sequencing and degradome analysis in cytoplasmic male-sterile and its maintainer fertile lines of *brassica juncea*

**DOI:** 10.1186/1471-2164-14-9

**Published:** 2013-01-16

**Authors:** Jinghua Yang, Xunyan Liu, Baochen Xu, Na Zhao, Xiaodong Yang, Mingfang Zhang

**Affiliations:** 1 Laboratory Germplasm Innovation and Molecular Breeding, Institute of Vegetable Science, Zhejiang University, Hangzhou, 310058, China; 2 Key laboratory of Horticultural Plant Growth, Development & Quality Improvement, Ministry of Agriculture, Hangzhou, 310058, China; 3 College of Life and Environmental Sciences, Hangzhou Normal University, Hangzhou, 310036, China

**Keywords:** *Brassica juncea*, Cytoplasmic male sterility, Degradome analysis, High-throughput sequencing, MicroRNA, Nuclear-cytoplasmic incompatibility

## Abstract

**Background:**

Regulatory network of cytoplasmic male sterility (CMS) occurrence is still largely unknown in plants, although numerous researches have been attempted to isolate genes involved in CMS. Here, we employed high-throughput sequencing and degradome analysis to identify microRNAs and their targets using high-throughput sequencing in CMS and its maintainer fertile (MF) lines of *Brassica juncea*.

**Results:**

We identified 197 known and 78 new candidate microRNAs during reproductive development of *B*. *juncea*. A total of 47 differentially expressed microRNAs between CMS and its MF lines were discovered, according to their sequencing reads number. Different expression levels of selected microRNAs were confirmed by using real-time quantitative PCR between CMS and MF lines. Furthermore, we observed that the transcriptional patterns of these microRNAs could be mimicked by artificially inhibiting mitochondrial F_1_F_0_-ATPase activity and its function in MF line by using treatment with oligomycin. Targeted genes of the microRNAs were identified by high-throughput sequencing and degradome approaches, including auxin response factor, NAC (No Apical Meristem) domain transcription factor, GRAS family transcription factor, MYB transcription factor, squamosa promoter binding protein, AP2-type transcription factor, homeobox/homeobox-leucine zipper family and TCP family transcription factors, which were observed to be differentially expressed between CMS and MF.

**Conclusion:**

Taken together, from these findings we suggested microRNA might participate in the regulatory network of CMS by tuning fork in gene expressions in CMS *B*. *juncea*. The differential expression of miRNAs observed between CMS and MF lines suggested that biogenesis of miRNAs could be influenced in the CMS.

## Background

Cytoplasmic male sterility (CMS), as a maternally inherited trait that prevents the production of functional pollen, is widely used in hybrid breeding and currently, is observed in > 150 plant species. CMS has been associated with expression of mitochondrial novel open reading frames (ORFs) that arise from rearrangements of mitochondrial genomes. Such *orfs* are often located adjacent to genes encoding components of the ATPase complex, and co-transcribed with these genes [1]. Although numerous attempts have been tried to isolate genes involved in CMS, regulatory network of CMS occurrence is still largely unknown in plants. Commonly, mitochondrial mutations lead to embryo lethality in plants owing to the essential nature of mitochondrial functions in energy metabolism. CMS mutations are an exception and these are naturally occurring mitochondrial genomic variants that condition nuclear-cytoplasmic incompatibility to produce a male sterility phenotype [1,2].

In plant cells, the mitochondrion and chloroplast are two semi-autonomous organelles that encode limited genetic information, with the majority being derived and imported from the nucleus. The inter-organellar communication between mitochondria and the nucleus is extensive, multifaceted and highly regulated. The predominant role of the nucleus in the cell has directed attention in recent years to studies of signaling from the nucleus to organelles (anterograde regulation), focusing mainly on pentatricopeptide repeat proteins that regulate RNA editing in mitochondria/chloroplasts and male fertility restorer genes in CMS [3]. However, organelles are also engaged in regulation of organelle-to-nucleus signaling (retrograde regulation) that fine-tunes nuclear gene expression, and influences stress responses, growth and development [3-5]. Mitochondrial retrograde regulation (MRR) of nuclear gene expression was first defined in yeast [6] and was well described in yeast and mammals [7]. The RTG (retrograde) pathway in yeast revealed the nuclear target gene *CIT2* and key proteins of signal transduction (*Rtg1*, *Rtg2* and *Rtg3*) [7]. However, mitochondrial retrograde regulation of nuclear gene expression is poorly understood in plants. The few reviews available have presumed that there are similar mitochondrial retrograde regulation pathways to yeasts and mammals [8-11]. In plant, evidence of plastid retrograde regulation is relatively well described, in which the GUN1 gene integrates multiple signals in the plastid and leads to ABI4-mediated repression of nuclear gene expression [5]. More recently, ABI4-mediated repression of nuclear gene expression was also identified to be involved in a mitochondrial retrograde regulation pathway [12].

MicroRNAs (miRNAs) are endogenous non-coding RNAs of ~ 22 nucleotides (nt) in length in plants, which guide post-transcriptional gene regulation mainly via mRNA cleavage. In *Arabidopsis*, miRNAs have been shown to play key roles in various biological processes, including developmental regulation, hormone response and stress adaptation [13-17]. To date, hundreds of small RNAs (sRNAs) have been isolated by direct cloning or deep sequencing in plants [18,19]. MicroRNA targets can be identified by computational prediction, based on sequence complementarity between miRNAs and the target mRNA or sequence conservation among different species [20]. They have been implicated in degradation of their mRNA targets into fragments with a monophosphate at the end. Therefore, isolation and sequencing of target mRNA degradation fragments can be used to validate miRNA targets. Recently, degradome sequencing, which combines high-throughput deep sequencing with bioinformatics analysis, has been successfully implemented to identify miRNA targets in *Arabidopsis*[21-23]. This method has been used to confirm predicted miRNA targets, allowing large-scale discovery of miRNA targets in plants [21,22,24-26].

In the present study, we identified miRNAs and their targets using high-throughput sequencing methods during reproductive development of CMS and its MF lines of *B*. *juncea*. The differential expression of miRNAs observed between CMS and MF lines suggested that biogenesis of miRNAs could be influenced in the CMS.

## Results

### sRNA populations in reproductive development of *B. juncea*

**Figure 1:**
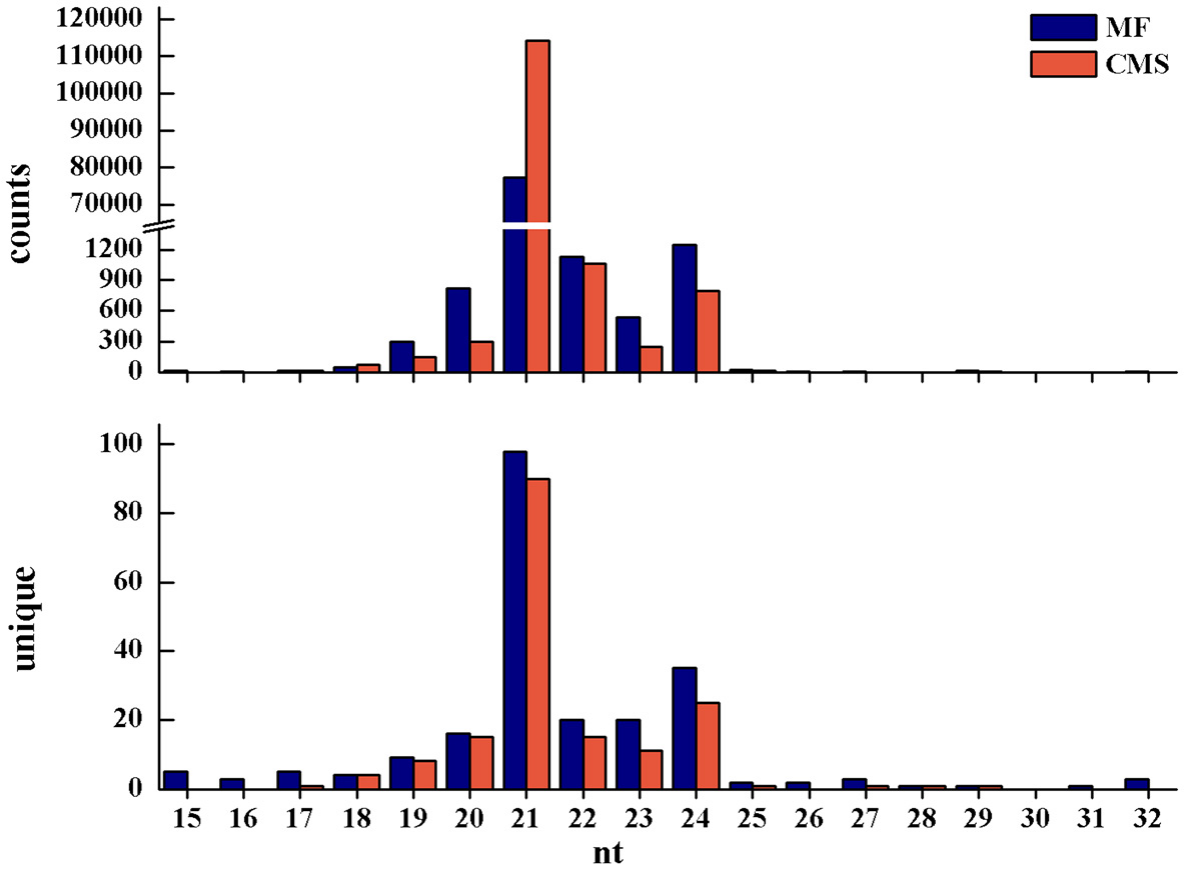
**Length distribution of counts of sequ-seqs and unique sRNAs during floral development in MF and CMS of *Brassica jucnea.***

To study the possible gene differences involved in the abortion of pollen development and the abnormal development of floral organ possibly caused by sRNA in CMS *B*. *juncea*, the sRNA libraries of reproductive development from CMS and MF *B*. *juncea* were constructed with RNAs from all floral buds of one intact inflorescence. Deep sequencing generated a total number of 11 845 753 from MF and 3 339 182 from CMS raw reads (Additional file 1: Table S1). After removal of corrupted adapter sequences, reads with length < 15 and > 32 nt and junk reads, there were 7 331 575 and 3 192 676 mappable reads obtained for MF and CMS libraries, respectively (Additional file 1: Tables S1 and S2). The majority of sRNAs were 21–24 nt for both libraries. This is within the typical size range for Dicer-derived products, in which 21-nt sRNAs were most abundant followed by 24 nt as the second largest percentage representing the class of endogenous sRNA families (Figure 1). We further compared the unique miRNAs between MF and CMS. There were 173 and 141 21–24-nt unique miRNAs in MF and CMS, respectively, of which there were 98 and 90 unique 21-nt miRNAs (Additional file 1: Table S3).

### Identification of known miRNAs in reproductive development from B. juncea

To identify the miRNAs from the reproductive development of *B*. *juncea*, sRNA sequences identified from *B*. *juncea* by deep sequencing were compared with the currently known mature plant miRNAs. In miRBase 18.0, there are 291 *Arabidopsis* pre-microRNAs (pre-miRNAs) reported that correspond to 328 mature miRNAs. In the present study, 99 pre-miRNAs corresponding to 94 miRNAs were detected, as well as 63 new mature 5^′^- or 3^′^-miRNAs corresponding to *Arabidopsis* pre-miRNAs detected for the first time (Additional file 1: Tables S4 and S5). There were four novel pre-miRNAs corresponding to five mature miRNAs originating from other species rather *Arabidopsis*. Among the five mature miRNAs, three new mature 5’- or 3’-miRNAs were detected in *B*. *juncea* for the first time. The other two pre-miRNAs corresponding to two mature miRNAs came from two other plant species (*Glycine max* and *Rehmannia glutinosa*) and could be mapped to the *Arabidopsis* genome (Additional file 1: Table S6). Five novel miRNAs originating from seven pre-miRNAs could not be mapped to the *Arabidopsis* genome; these were mapped to other plant species genomes and the extended sequences at the mapped positions of the genome potentially form hairpins (Additional file 1: Table S7). Another 17 novel miRNAs identified to originate from pre-miRNAs, that could not be mapped to the *Arabidopsis* genome but were mapped to other plant genomes, failed in hairpin structure prediction for extended sequences at the mapped positions (Additional file 1: Table S8). There were also 13 novel miRNAs conserved in other plant species, but not found in the *Arabidopsis* genome (Additional file 1: Table S9).

### Identification of new candidate miRNAs in reproductive development of *B. juncea*

**Figure 2:**
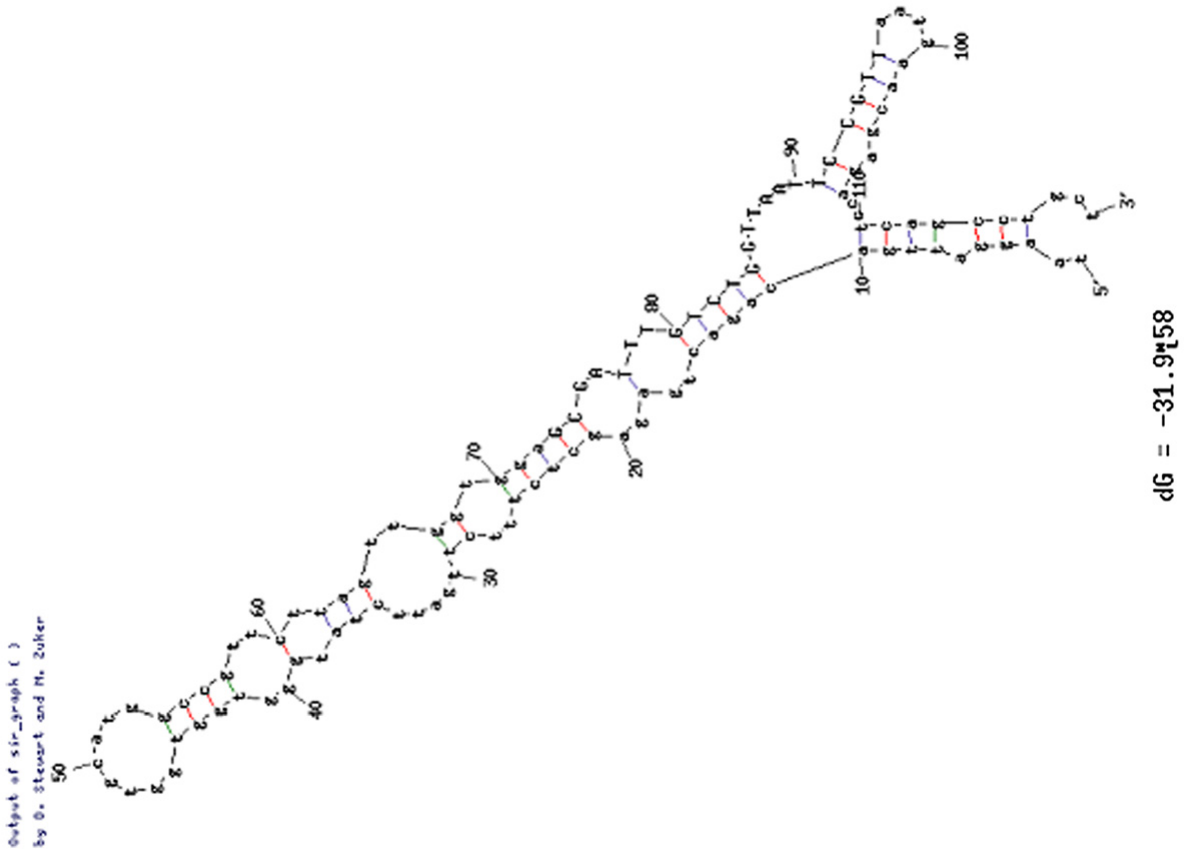
**Prediction of secondary structure of representative new candidate miRNA during floral development of *Brassica juncea*.**

To identify new miRNAs, we removed all reads with low abundance from sequence data, and the data were then used to query the mRNA (ftp://ftp.arabidopsis.org/Sequences/ATH cDNA EST sequences FASTA/), non-coding RNA sequences at the database (ftp://ftp.sanger.ac.uk/pub/databases/Rfam/10.1/) and the repeat-Repbase (http://www.girinst.org/repbase/update/index.html). The consensus surrounding the genomic regions of each miRNA was retrieved and secondary structure was predicted. All genomic loci-generating sRNAs that can be folded into a secondary structure were considered as miRNA candidates. In total, 78 pre-miRNAs corresponding to 93 unique mature miRNAs were first identified in the present study, and these candidate miRNAs originated from predicted RNA hairpins (Additional file 1: Table S10). The secondary hairpin structures of the representative miRNAs are shown in Figure 2 and all secondary hairpin structures of candidate miRNAs are listed in Additional file 2: Figure S1. Interestingly, some new candidate miRNAs were organelle-derived non-coding sRNAs: miRNAs PC-5p-13, PC-3p-14, PC-3p-39, PC-5p-40, PC-3p-54, PC-3p-72, PC-5p-75, PC-5p-88 and PC-3p-90 are derived from chloroplasts, and PC-5p-17, PC-3p-18, PC-3p-25 and PC-5p-26 from mitochondria. Strikingly, several pre-miRNA and mature sequences of new candidate miRNAs were the same, but were located by alignment to nuclear and mitochondrial genomes respectively, including pre-miRNA22/pre-miRNA23, pre-miRNA24/pre-miRNA25, pre-miRNA73/pre-miRNA74 and pre-miRNA75/pre-miRNA76 (Additional file 1: Table S10). Finally, 290 miRNAs, including known and new candidates, were identified in reproductive development of MF and CMS *B*. *juncea* (Additional file 1: Table S11).

### Differential expression of miRNAs during reproductive development of MF and CMS *B. juncea*

**Figure 3:**
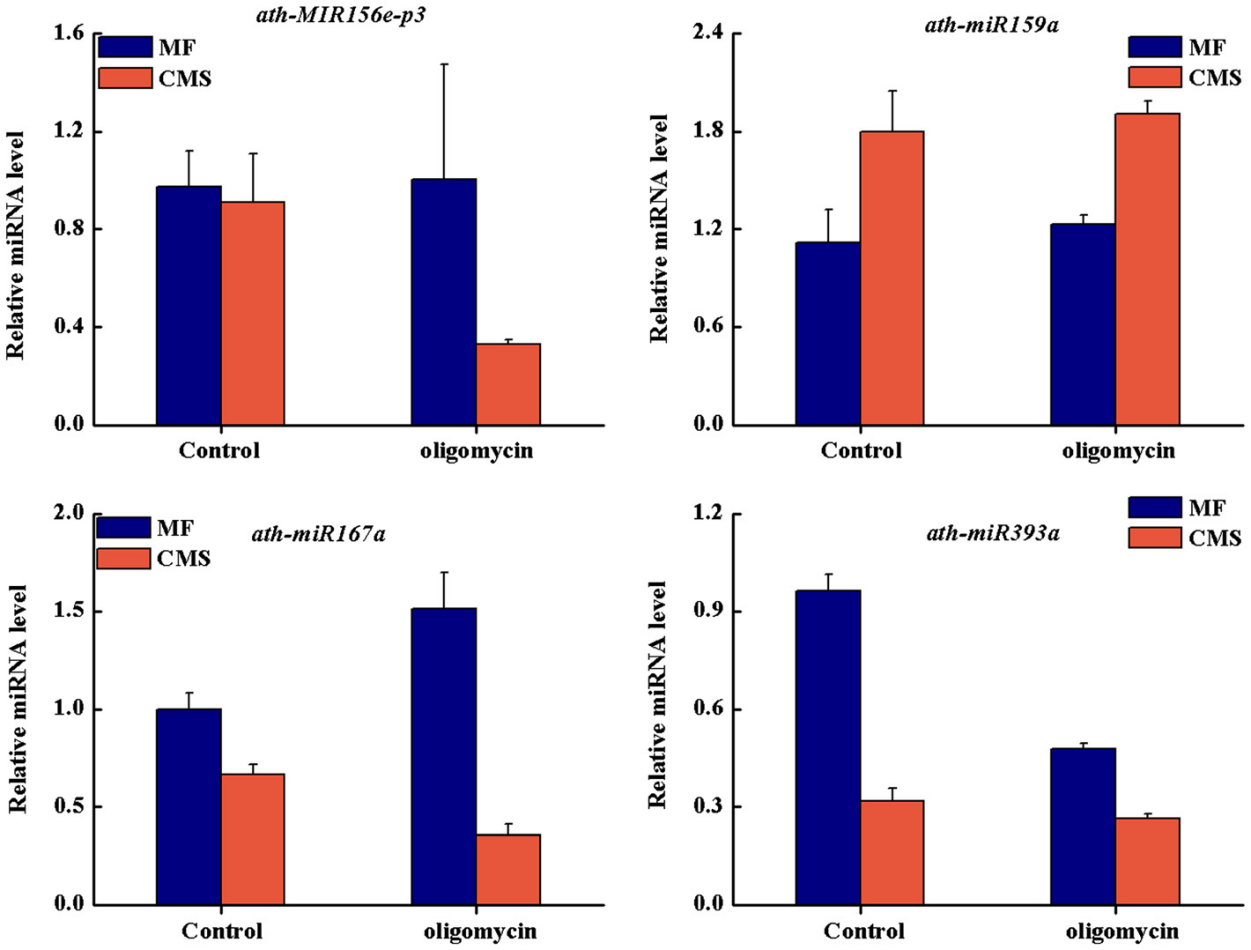
**Detection of selected miRNAs expression in MF and CMS floral buds and treated with oligomycin using q-PCR.** 5 s rRNA was chosen as an endogenous control. The results were obtained from three biological replicates, and the error bars indicate the standard error of the mean.

miRNAs in MF and CMS were used for differential expression analysis with IDEG6 (http://telethon.bio.unipd.it/bioinfo/IDEG6_form/). The selection methods of differential expression were Audic and Claverie, Fisher’s exact test and chi-squared 2 × 2, with the selection threshold of 0.01 (http://telethon.bio.unipd.it/bioinfo/IDEG6_form/detail.html#AC). Finally, 47 miRNAs were differentially expressed between MF and CMS: among these, 25 and 22 miRNAs were down- and up-regulated, respectively, according to sequencing reads (Table 1). The expressions of 8 selected miRNAs were tested using quantitative real-time RT-PCR (qRT-PCR) analysis, which further validated the differential expression data obtained from sequencing on the whole (Figure 3). The expression patterns of 6 of 8 miRNAs were consistent with the sequencing reads. The expressions of ath-miR396b and ath-miR159_R-2 showed no significant differences between CMS and MF, however, the expressions of these two miRNAs were down-regulated in CMS (Figure 3). To determine whether the expressions of miRNAs could be regulated by mitochondrial function, we studied the expressions of 4 selected miRNAs in MF, CMS and when treated with a mitochondrial-specific inhibitor (oligomycin). For the down-regulated miRNAs in CMS, ath-miR393a and ath-MIR156e-p3, their expressions were also decreased in MF treated with oligomycin, and relatively more reduced in CMS treated with oligomycin. For up-regulated miRNAs in CMS, the expression of ath-miR159a was also increased in MF treated with oligomycin, and relatively more increased in CMS treated with oligomycin (Figure 3).

**Table 1:** **Differentially-expressed miRNAs between CMS and MF of *Brassica juncea***

**UNIQID**	**Description**	**norm_MF**	**norm_CMS**	**AC 1 2**	**Fisher 1 2**	**Chi2x2 1 2**	**CMS/MF**
ath-MIR156a-p3	TGCTCACTGCTCTTTCTGTCAGA	50	0	0	0	0	down
ath-MIR156e-p3	GCTTACTCTCTCTCTGTCACC	91	9	0	0	0	down
ath-miR156h_L + 1	TTGACAGAAGAAAGAGAGCAC	14	47	0.000005	0.000027	0.000024	up
ath-miR158a	TCCCAAATGTAGACAAAGCA	31	1	0	0	0	down
ath-miR159a	TTTGGATTGAAGGGAGCTCTA	3375	5739	0	0	0	up
ath-MIR159a-p5	AGCTGCTAAGCTATGGATCCC	54	12	0	0	0	down
ath-miR159c_R-2	TTTGGATTGAAGGGAGCTC	77	17	0	0	0	down
ath-miR164a	TGGAGAAGCAGGGCACGTGCA	253	472	0	0	0	up
ath-miR164b	TGGAGAAGCAGGGCACGTGCA	253	472	0	0	0	up
ath-miR164c	TGGAGAAGCAGGGCACGTGCG	102	51	0.000006	0.000046	0.000037	down
ath-MIR164c-p3	CACGTGTTCTACTACTCCAAC	1	17	0.000034	0.000145	0.000162	up
ath-miR165a	TCGGACCAGGCTTCATCCCCC	3116	4420	0	0	0	up
ath-miR165b	TCGGACCAGGCTTCATCCCCC	3116	4420	0	0	0	up
ath-miR166a	TCGGACCAGGCTTCATTCCCC	2589	3591	0	0	0	up
ath-miR166b	TCGGACCAGGCTTCATTCCCC	2589	3591	0	0	0	up
ath-miR166c	TCGGACCAGGCTTCATTCCCC	2589	3591	0	0	0	up
ath-miR166d	TCGGACCAGGCTTCATTCCCC	2589	3591	0	0	0	up
ath-miR166e	TCGGACCAGGCTTCATTCCCC	2589	3591	0	0	0	up
ath-miR166f	TCGGACCAGGCTTCATTCCCC	2589	3591	0	0	0	up
ath-miR166g	TCGGACCAGGCTTCATTCCCC	2589	3591	0	0	0	up
ath-miR167a	TGAAGCTGCCAGCATGATCTA	6244	864	0	0	0	down
ath-MIR167a-p3	GATCATGTTCGCAGTTTCACC	568	83	0	0	0	down
ath-miR167b	TGAAGCTGCCAGCATGATCTA	6244	864	0	0	0	down
ath-miR167d_R-2	TGAAGCTGCCAGCATGATCT	359	42	0	0	0	down
ath-MIR169e-p3	GCAAGTTGACTTTGGCTCTGT	141	39	0	0	0	down
ath-miR319a	TTGGACTGAAGGGAGCTCCCT	45330	82597	0	0	0	up
ath-miR319b	TTGGACTGAAGGGAGCTCCCT	45330	82597	0	0	0	up
ath-MIR319b-p5	GAGCTTTCTTCGGTCCACTC	0	20	0	0.000002	0.000008	up
ath-miR390a	AAGCTCAGGAGGGATAGCGCC	289	527	0	0	0	up
ath-MIR390a-p3_1ss5AG	CGCTGTCCATCCTGAGTTTCA	52	102	0.000009	0.000069	0.000056	up
ath-miR390b	AAGCTCAGGAGGGATAGCGCC	289	527	0	0	0	up
ath-miR395a	CTGAAGTGTTTGGGGGAACTC	36	7	0.000002	0.000009	0.00001	down
ath-miR395d	CTGAAGTGTTTGGGGGAACTC	36	7	0.000002	0.000009	0.00001	down
ath-miR395c	CTGAAGTGTTTGGGGGAACTC	36	7	0.000002	0.000009	0.00001	down
ath-miR396b	TTCCACAGCTTTCTTGAACTT	260	171	0.000002	0.000021	0.000018	down
ath-miR408	ATGCACTGCCTCTTCCCTGGC	805	1272	0	0	0	up
ath-MIR408-p5	ACAGGGAACAAGCAGAGCATG	39	117	0	0	0	up
ath-miR845a	CGGCTCTGATACCAATTGATG	121	53	0	0	0	down
peu-MIR2916-p3	TCTCAACCATAAACGATGCCGACC	523	335	0	0	0	down
peu-MIR2916-p5	GTCTCAACCATAAACGATGCCGAC	316	228	0.000014	0.000185	0.000161	down
ahy-miR167-5p_1ss21TC	TGAAGCTGCCAGCATGATCTC	80	2	0	0	0	down
ahy-miR159_1ss7TC	TTTGGACTGAAGGGAGCTCTA	33	6	0.000003	0.000014	0.000015	down
rco-miR319d_2ss20TC21TA	TTGGACTGAAGGGAGCTCCCA	201	83	0	0	0	down
peu-MIR2911-p3_1ss6CT	GCGTGTCGGCCGGGGGACGGGCTG	19	0	0.000001	0.000004	0.000013	down
peu-MIR2911-p5_1ss3TA	GGAGGACTGCTCGAGCTGC	18	1	0.000018	0.000076	0.000096	down
smo-MIR1103-p3	GTGACCTCCCGGGAAGTCC	179	117	0.000034	0.000374	0.000313	down
PC-3p-77	GCGATTTGTCTGGTTAATTCCGTT	199	94	0	0	0	down

### Target genes of miRNAs by degradome sequencing and analysis

**Figure 4:**
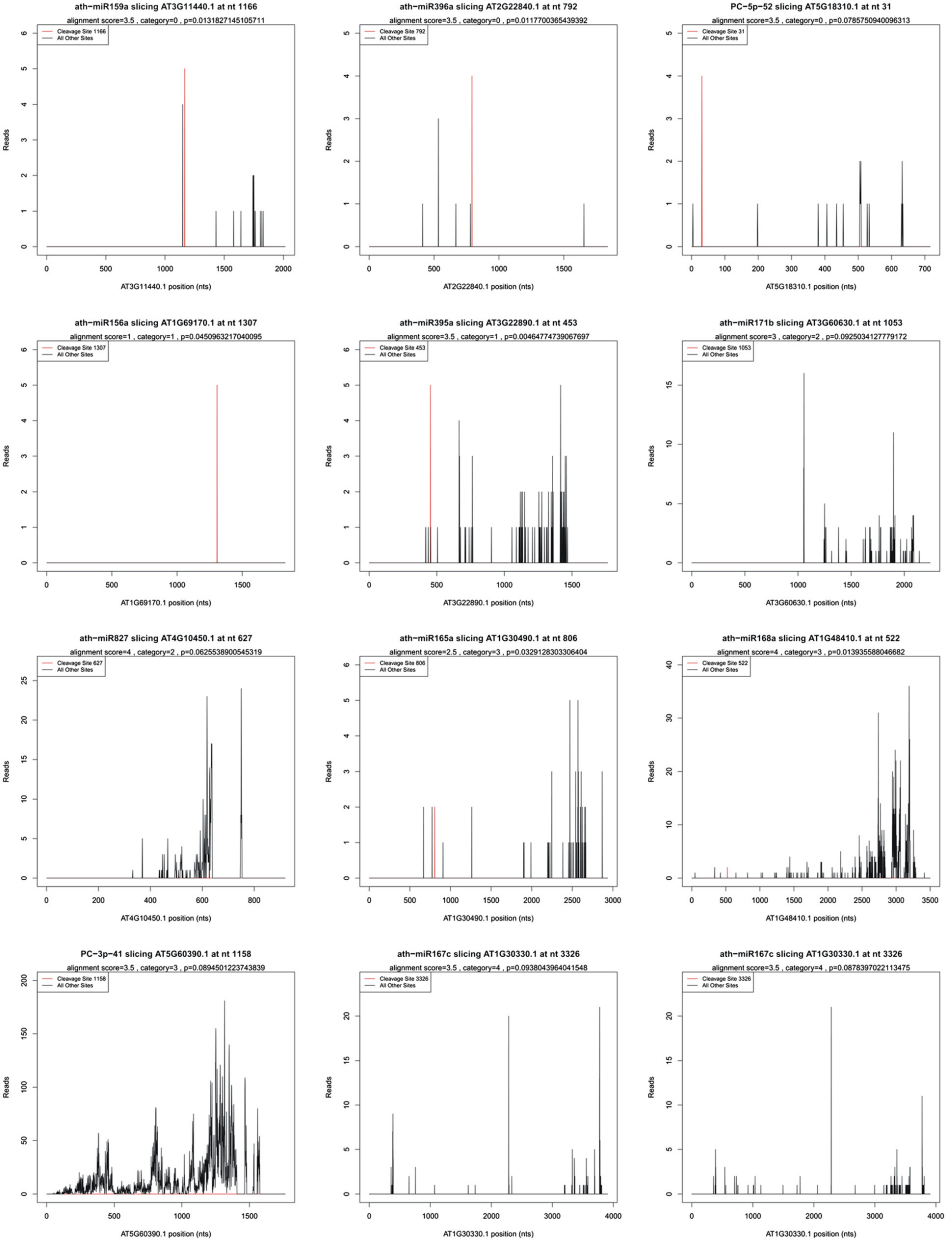
**Target plot (t-plot) of representative validated miRNAs target in MF and CMS of *Brassica juncea.*** The abundance of each signature is plotted as a function of its position in the transcript.

To date, limited targets for miRNAs have been identified in plants. Here, we performed a genome-wide sequencing of miRNA-cleaved mRNA and Cleveland analysis based on recently developed high-throughput degradome sequencing technology [21,22]. The targets for all miRNAs are listed (Additional file 1: Table S12). The abundance of transcripts was plotted for each transcript and the sliced-target transcripts were grouped into five categories according to the relative abundance of tags at the target sites (Additional file 3: Figure S2 and Additional file 4: Figure S3). Category ‘0’ is defined as > 1 raw read at the position, with abundance at a position equal to the maximum on the transcript, and with only one maximum on the transcript. Category ‘1’ is described as > 1 raw read at the position, with abundance at the position equal to the maximum on the transcript, and more than one maximum position on the transcript. Category ‘2’ includes > 1 raw read at the position, and abundance at the position less than the maximum but higher than the median for the transcript. Category ‘3’ comprised the transcripts with > 1 raw read at the position, and abundance at the position equal to or less than the median for the transcript; and category ‘4’ showed only one raw read at the position. The representative miRNAs and corresponding targets included all five categories, in which a red line indicates the cleavage site of each transcript (Figure 4). In total we identified 838 and 768 targets in reproductive development for MF and CMS *B*. *juncea*, respectively (Additional file 1: Tables S12-1 and S12-2), and 333 high assuring targets with different read abundance of cleavage (Additional file 1: Table S12-Table S3). These targets included auxin response factor, NAC (No Apical Meristem) domain transcription factor, GRAS family transcription factor, MYB transcription factor, squamosa promoter binding protein, AP2-type transcription factor, homeobox/homeobox-leucine zipper family and TCP family transcription factor, which have essential roles in gene regulation (Additional file 1: Table S12).

We also identified the targets of new candidate miRNAs by degradome sequencing and analysis. In total, 13 new candidate miRNAs were shown to target 102 transcripts (Table 2). Although some new candidate miRNAs were accumulated to a very low level according to sequencing reads, their targets were still identified by degradome sequencing and analysis. The abundance of transcripts was also plotted on each transcript and these sliced-target transcripts were grouped into five categories according to the relative abundance of the tags at the target sites for the new candidate miRNAs (Additional file 3: Figure S2 and Additional file 4: Figure S3). The new candidate miRNAs targeted different genes with a wide variety of predicted functions. Among the identified targets of new candidate miRNAs, PC-3p-41 targeted the GTP-binding elongation factor Tu family of proteins; and PC-3p-65 targeted several genes including MYB domain protein 47, eukaryotic translation initiation factor family protein and squamosa promoter binding protein-like gene, suggesting its multiple regulating functions (Table 2). In addition, several new candidate miRNAs targeted some genes of unknown function.

**Table 2:** **Targets of new candidate microRNAs in *Brassica juncea***

**miRNA**	**referred to*Arabidopsis***	**Category**	**Cleavage Site**	**MF-Rep Norm reads**	**CMS-Rep Norm reads**	**Annotation**
PC-3p-30	AT4G28000.1	4	77	1		P-loop containing nucleoside triphosphate hydrolases superfamily protein
	AT4G38850.1	4	272		0.111111	SAUR-like auxin-responsive protein family
PC-3p-41	AT1G07920.1	3	1202	1	3.361111	GTP binding Elongation factor Tu family protein
	AT1G07930.1	3	1214	1	3.361111	GTP binding Elongation factor Tu family protein
	AT1G07930.2	3	983	1	3.361111	GTP binding Elongation factor Tu family protein
	AT1G07940.1	3	1206	1	3.361111	GTP binding Elongation factor Tu family protein
	AT1G07940.2	3	1333	1	3.361111	GTP binding Elongation factor Tu family protein
	AT1G35550.1	4	303		0.111111	elongation factor Tu C-terminal domain-containing protein
	AT5G60390.1	3	1158	1	3.361111	GTP binding Elongation factor Tu family protein
	AT5G60390.2	3	1158	1	3.361111	GTP binding Elongation factor Tu family protein
	AT5G60390.3	3	1148	1	3.361111	GTP binding Elongation factor Tu family protein
PC-3p-65	AT1G18400.1	2	460	2		BR enhanced expression 1
	AT1G18710.1	4	792	0.333333		myb domain protein 47
	AT1G62750.1	3	82		0.4	Translation elongation factor EFG/EF2 protein
	AT1G76720.1	4	1493	0.2		eukaryotic translation initiation factor 2 (eIF-2) family protein
	AT1G76810.1	4	1225	0.2		eukaryotic translation initiation factor 2 (eIF-2) family protein
	AT1G76820.1	4	878	0.2		eukaryotic translation initiation factor 2 (eIF-2) family protein
	AT2G26630.1	4	761	0.25	0.2	transposable element gene
	AT2G27710.1	3	505	0.535714	0.142857	60S acidic ribosomal protein family
	AT2G27710.2	3	502	0.535714	0.142857	60S acidic ribosomal protein family
	AT2G27710.3	3	433	0.535714	0.142857	60S acidic ribosomal protein family
	AT2G27710.4	3	454	0.535714	0.142857	60S acidic ribosomal protein family
	AT2G30860.1	3	128		1	glutathione S-transferase PHI 9
	AT2G30860.2	3	128		1	glutathione S-transferase PHI 9
	AT2G33810.1	4	409	1		squamosa promoter binding protein-like 3
	AT3G06870.1	2	585	0.4		proline-rich family protein
	AT4G09255.1	4	62	0.5		transposable element gene
	AT4G11420.1	4	538		1	eukaryotic translation initiation factor 3A
	AT5G21274.1	4	63		1	calmodulin 6
	AT5G57290.2	2	348	3	0.333333	60S acidic ribosomal protein family
	AT5G57655.1	4	1553		0.5	xylose isomerase family protein
	AT5G57655.2	4	1592		0.5	xylose isomerase family protein
PC-5p-12	AT3G25100.1	3	823	0.5		cell division cycle 45
	AT5G28800.1	4	13	0.166667		unknown protein
	AT5G40340.1	2	380	0.85		Tudor/PWWP/MBT superfamily protein 6
	AT5G52830.1	4	1040		1	WRKY DNA-binding protein 27
	AT5G62750.1	2	212	2		unknown protein
PC-5p-20	AT2G28056.1	2	677	2		MIR172/MIR172A; miRNA
PC-5p-32	AT1G70700.1	4	958		0.5	TIFY domain/Divergent CCT motif family protein
	AT1G70700.2	4	886		0.5	TIFY domain/Divergent CCT motif family protein
	AT2G24270.1	2	1736	0.75		aldehyde dehydrogenase 11A3
	AT2G24270.2	2	1633	0.75		aldehyde dehydrogenase 11A3
	AT2G24270.3	2	1622	0.75		aldehyde dehydrogenase 11A3
	AT2G24270.4	2	1633	0.75		aldehyde dehydrogenase 11A3
	AT2G39720.1	4	1004		1	RING-H2 finger C2A
PC-5p-51	AT2G20585.1	4	375	0.333333		nuclear fusion defective 6
	AT2G20585.2	4	375	0.333333		nuclear fusion defective 6
	AT2G20585.3	4	375	0.333333		nuclear fusion defective 6
PC-5p-52	AT2G27760.1	4	42		0.5	tRNAisopentenyltransferase 2
	AT2G45960.1	4	148	0.166667		plasma membrane intrinsic protein 1B
	AT2G45960.2	4	148	0.166667		plasma membrane intrinsic protein 1B
	AT2G45960.3	4	148	0.166667		plasma membrane intrinsic protein 1B
	AT2G47700.1	4	32		0.5	RING/U-box superfamily protein
	AT3G03340.1	4	67	0.1	0.1	LUC7 related protein
	AT3G09980.1	4	71	0.333333		Family of unknown function (DUF662)
	AT3G57870.1	4	81	1		sumo conjugation enzyme 1
	AT5G18310.1	0	31	1.5		unknown protein
	AT5G18310.2	0	24	1.5		unknown protein
PC-5p-53	AT3G46040.1	3	131	1		ribosomal protein S15A D
	AT4G02660.1	2	8902	1		Beige/BEACH domain ;WD domain, G-beta repeat protein
	AT4G34230.1	2	178		1	cinnamyl alcohol dehydrogenase 5
	AT4G34230.2	2	175		1	cinnamyl alcohol dehydrogenase 5
	AT5G53530.1	4	993	1		vacuolar protein sorting 26A
	AT5G59850.1	2	122	2.333333	0.333333	Ribosomal protein S8 family protein
PC-5p-56	AT4G12800.1	2	385	4	4	photosystem I subunit l
	AT5G19290.1	4	387		1	alpha/beta-Hydrolases superfamily protein
PC-5p-64	AT1G20260.1	4	648		0.5	ATPase, V1 complex, subunit B protein
	AT1G50500.1	4	533	0.333333		Membrane trafficking VPS53 family protein
	AT1G50500.2	4	548	0.333333		Membrane trafficking VPS53 family protein
	AT1G67410.1	0	1638	6		Exostosin family protein
	AT1G71680.1	4	1781	1		Transmembrane amino acid transporter family protein
	AT1G76030.1	4	654		0.5	ATPase, V1 complex, subunit B protein
	AT2G38040.1	4	1257	0.5	2.5	carboxyltransferase alpha subunit
	AT2G38040.2	4	1232	0.5	2.5	carboxyltransferase alpha subunit
	AT4G25050.1	4	355		0.5	acyl carrier protein 4
	AT4G25050.2	4	416		0.5	acyl carrier protein 4
	AT4G35300.1	4	2370		0.2	tonoplast monosaccharide transporter2
	AT4G35300.2	4	2371		0.2	tonoplast monosaccharide transporter2
	AT4G35300.3	4	2331		0.2	tonoplast monosaccharide transporter2
	AT4G35300.4	4	2370		0.2	tonoplast monosaccharide transporter2
	AT4G35300.5	4	2251		0.2	tonoplast monosaccharide transporter2
	AT4G36640.1	1	281	1.5		Sec14p-like phosphatidylinositol transfer family protein
	AT4G36640.2	1	185	1.5		Sec14p-like phosphatidylinositol transfer family protein
	AT5G19770.1	4	558	0.5		tubulin alpha-3
	AT5G19780.1	4	591	0.5		tubulin alpha-5
	AT5G54960.1	4	1869	0.5	0.5	pyruvate decarboxylase-2
PC-5p-74	AT3G09800.1	2	689	3	2	SNARE-like superfamily protein
	AT3G17900.1	4	905		1	unknown protein
	AT3G53710.1	4	1391	0.5		ARF-GAP domain 6
	AT3G53710.2	4	1333	0.5		ARF-GAP domain 6
	AT3G56850.1	4	1331		1	ABA-responsive element binding protein 3
	AT4G09510.1	0	1861	6.5	3	cytosolic invertase 2
	AT4G09510.2	0	1916	6.5	3	cytosolic invertase 2
	AT5G27860.1	4	463	0.5		unknown protein
	AT5G27860.2	4	463	0.5		unknown protein
	AT5G39740.1	2	838	0.75	0.5	ribosomal protein L5 B
	AT5G39740.2	2	880	0.75	0.5	ribosomal protein L5 B
	AT5G47040.1	1	2243		4	lon protease 2
	ATMG01360.1	2	1453	13	2	cytochrome oxidase
	AT4G31480.1	4	2477	0.333333		Coatomer, beta subunit
	AT4G31480.2	4	2557	0.333333		Coatomer, beta subunit
	AT4G31490.1	4	2399	0.333333		Coatomer, beta subunit

### Expression of miRNA395a and APS1 gene in MF and CMS *B. juncea*

**Figure 5:**
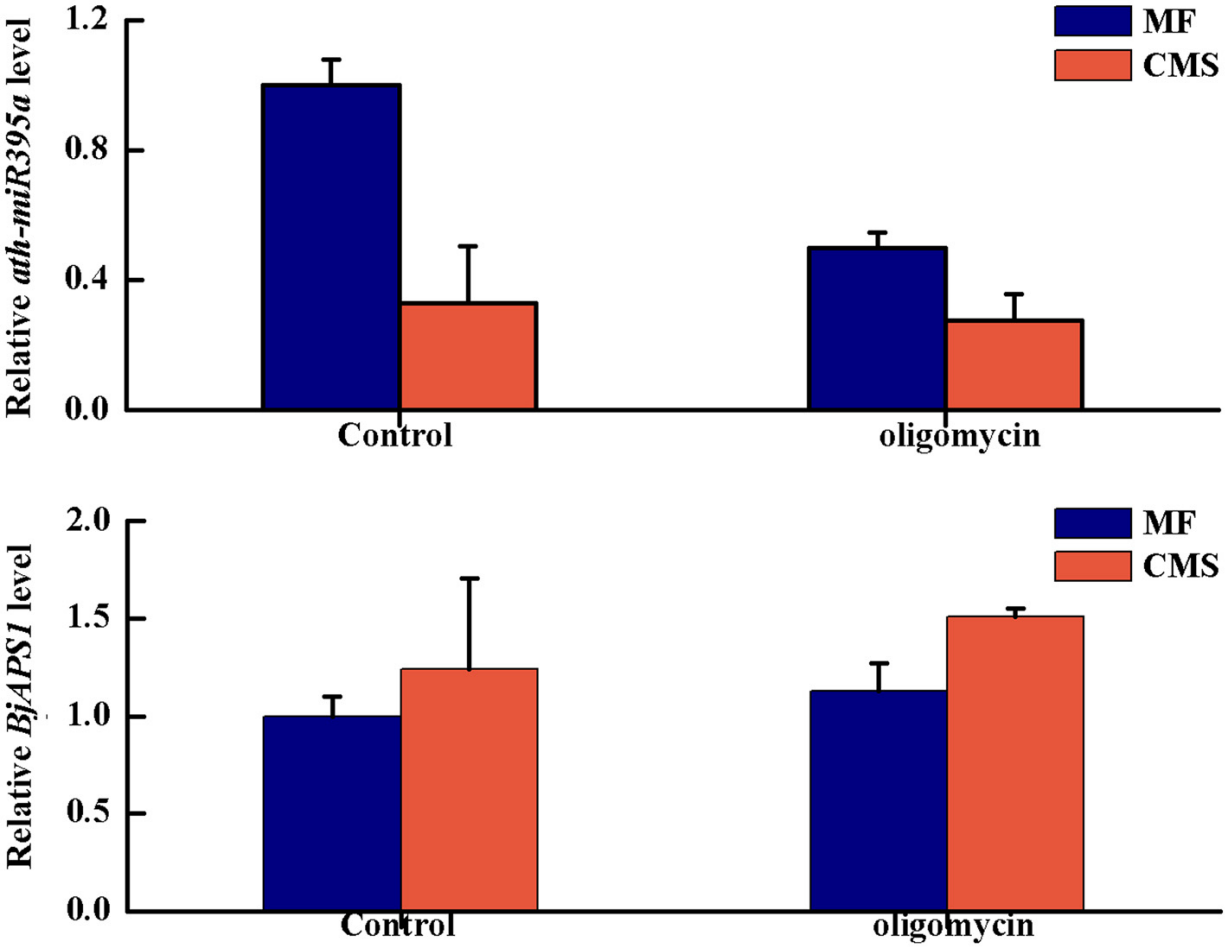
**Expression of miR395a and its targeted gene (*APS1*) in MF and CMS floral buds and treated with oligomycin using q-PCR.**

From degradome sequencing and analysis, *ATP Sulfurylase 1* (*APS1*) gene was identified to be the target gene of miRNA395. To confirm the causality of the miRNA expression patterns and its target gene, we studied the expression of *APS1* in MF and CMS. *APS1* expression level was higher in CMS than MF because of lower expression of miRNA395a and negative regulation of *APS1* expression in CMS (Figure 5). To determine whether the expression of miR395a and *APS1* could be regulated by mitochondrial function, we studied the expression of miR395a and *APS1* in MF and CMS, and treated with oligomycin. When we treated MF and CMS with oligomycin, *APS1* expression was increased, and its induction was greater in CMS (Figure 5).

## Discussion

Using high-throughput deep sequencing technology, we pyrosequenced sRNA populations from reproductive development of *B*. *juncea* at the genome-wide level. Based on the analysis from the newly updated miRBase 18.0, we identified a much wider range of sRNAs with 15–32 nt in length (Figure 1) than the range of sRNAs with 16–27 nt in length in previous publications. Several species in plants, including *Arabidopsis*, *Oryzasativa*, *Solanum lycopersicum* and *Medicago truncatula*, had been shown to contain substantially more 24-nt than 21-nt sRNAs (Rajagopalan et al., 2006; Morin et al., 2008; Moxon et al., 2008; Szittya et al., 2008). Additionally, more 21-nt than 24-nt sRNAs were reported in *Vitis vinifera* and *M*. *truncatula* treated with Hg^+^[26,27]. Here, we observed a high level of 21-nt compared with 24-nt sRNA in reproductive development of *B*. *juncea* (Figure 1). *Brassica juncea* is an allopolyploid species that originated from hybridization between ancestral parents of *B*. *rapa* and *B*. *nigra*. sRNAs serve as a genetic buffer against genomic shock in interspecific hybrids and allopolyploid *Arabidopsis*, in which allopolyploid species usually face reorganization of homological gene regions [28]. In our investigations, we found almost no recombination for the chromosome from *B*. *rapa* and *B*. *nigra* in allopolyploid *B*. *juncea* (unpublished data). The unusual sRNAs might play a role in regulating homologous gene expression.

Identification of miRNAs has previously been reported in model plants, including in developing pollen of *O*. *sativa*[29] and mature pollen of *Arabidopsis*[30,31] by using deep sequencing or miRNA arrays. The present study is the first to report comprehensive identification of miRNAs and their targets using high-throughput sequencing in reproductive development of *B*. *juncea*. Because whole genome sequences of *B*. *juncea* are not yet available, we mainly referred to genome information of *Arabidopsis* (which belongs to the *Cruciferae* family as does *B*. *juncea*) to analyze the miRNAs. The majority of these known miRNAs in *Arabidopsis* and other species were detected and had relatively high expression abundance in *B*. *juncea* (Table 1). Most known miRNAs had the canonical 21 nt length, suggesting DCL1 cleavage products, with few variations observed in *B*. *juncea*. We also identified many *B*. *juncea*-specific miRNAs with named miRNA* strands in formed miRBase, being an important prerequisite for new miRNA identification. Additionally, 93 new candidate mature miRNAs were sequenced in reproductive development of *B*. *juncea*, of which most represented a new class of miRNAs of 23–25 nt in length, termed long miRNAs (Additional file 1: Table S10). These long miRNAs were likely to be dependent on DCL3 and the hierarchical action of other DCLs according to the evolution of miRNAs [32]. Interestingly, some new candidate miRNAs were observed to be organelle-derived miRNAs (Additional file 1: Table S10), which suggested that these miRNAs were derived by alternative biogenesis pathways, not via Dicer proteins. Indeed, sRNAs can be generated from organelles including chloroplasts and mitochondria in animals, plants, fungi and humans [33-36]. New candidate miRNAs are considered to be young miRNAs that have evolved recently, and are often expressed at a lower level than conserved miRNAs, as reported from *Arabidopsis* and *Triticum*[37,38]. This observation was also true for most of the new miRNAs identified from *B*. *juncea* (Additional file 1: Table S10). Moreover, the miRNAs with the same sequence of pre-miRNA and mature miRNA for each group (like pre-miRNA22/pre-miRNA23) (Additional file 1: Table S10) are thought to be associated with nuclear-mitochondrial co-evolution or communications between nucleus and mitochondria.

miRNAs related to reproductive development, especially floral organ development, have been well studied in *Arabidopsis*; however, identification of the targets regulated by miRNAs related to reproductive development are largely unknown. For example, among these identified miRNAs and their corresponding targets, miRBL and miRFIS (that target the class C genes module) exert homeotic control over *Petunia hybrida* and *Antirrhinum majus* floral organ identity [39]. miR172 likely acts in cell-fate specification as a translational repressor of *APETALA2* gene in *Arabidopsis* flower development [40]. miR159 that targets MYB33 and MYB65 is essential for normal anther development in *Arabidopsis*[41]. *Arabidopsis* miR167 controls patterns of *ARF6* and *ARF8* gene expression, and regulates both female and male reproduction [42]. In previous cases, targets of miRNAs were identified by computational prediction *in silico*, and then using modified 5^′^-RACE-PCR to confirm the expression of target genes in pollen development [29-31]. The confirmation procedures were convincing but not high-throughput. Degradome sequencing has shown to be powerful in identifying target genes of miRNAs with greater throughput [21,22,24,26]. The present study is the first to report comprehensive identification of miRNA targets associated with reproductive development using high-throughput sequencing and degradome analysis in reproductive development of *B*. *juncea*. Of the identified targets of miRNAs, some were previously shown to be involved in floral organ or pollen development, e.g. MYB65/MYB33 [41], ARF6/AFR8 [42], AP2 [40] and SQUAMOSA PROMOTOTER BINDING PROTEIN-LIKE [43]. Some of them have not been shown to be associated with reproductive development as miRNA targets, such as homeobox-leucine zipper family protein, other genes in the PROMOTOTER BINDING PROTEIN-LIKE family and the GRAS family transcription factor genes. We identified 102 targets for new candidate miRNAs in reproductive development of *B*. *juncea* (Table 2). The newly identified miRNAs and their targets might offer useful information in potential future studies on miRNAs and their targets involved in reproductive development, which should be further investigated. It is noteworthy that PC-3p-30 targeted the GTP-binding elongation factor Tu family of proteins, which were likely associated with pollen development. The most obvious difference between the targets of conserved and new miRNAs was that most new miRNA targets belonged to categories ‘3’ and ‘4’ (Table 2), where cleavage abundance was below the median on target transcripts. The finding that new miRNA targets mainly fall into categories ‘3’ and ‘4’ may suggest that these new miRNAs are young and not fully stabilized evolutionarily.

The majority of mature miRNAs are generated from the processes of pri-miRNA by a Dicer-like enzyme and loaded into a ribo-nucleo-protein complex consisting of an ARGONAUTE (AGO) in various biological processes, including developmental regulation, hormone response and stress adaptation. A link between miRNA biogenesis and stresses has been well studied and documented in many cases [13,44-46]. In the case of miR398, down-regulated by multiple stresses, reduced expression of miR398 in transgenic lines causes enhanced tolerance to oxidative stress [15,47]. Intriguingly, sucrose can up-regulate miR398 expression, suggesting a possible link between cellular energy status and miRNA biogenesis [48]. Such a link is further supported by a recent study, in which miRNA biogenesis could be triggered by inhibition of mitochondrial respiration [49]. Here, we employed deep sequencing to identify miRNAs that might be related to abnormal reproductive development in CMS *B*. *juncea*. We detected differential expression of many miRNAs between CMS and MF. Interestingly, the expression patterns of these miRNAs could be mimicked by artificial inhibition of F_1_F_0_-ATPase activity after treatment with oligomycin, a specific inhibitor of the ATPase complex. Our studies suggest that miRNA biogenesis can be regulated by mitochondrial function inhibition, which enlarges the scope of induction of miRNA biogenesis. At least three kinds of mitochondrial retrograde regulation (MRR)pathways and mechanisms are seen in yeast [7,50]. The general process of MRR is conserved among yeast, mammals and plants, among which the mechanisms of signal molecules and signal transduction pathways are probably quite diverse [7]. In *Arabidopsis*, using the promoter of *AOX1a* gene as a mitochondrial marker, candidate mitochondrial retrograde regulation mutants were identified in response to distinct mitochondrial perturbations of the tricarboxylic acid cycle or mitochondrial electron transport chain [51]. The transcription factor ABI4, which has been identified in chloroplast retrograde regulation, also plays an important role in mediating mitochondrial retrograde regulation signals to induce the expression of *AOX1a* in *Arabidopsis*. Comparative studies between CMS and MF lines have shown that some nuclear candidate genes and transcription factor genes are involved in retrograde regulation signaling in plants [52-54]. With deep sequencing and degradome analysis in the present study, we observed that nuclear-cytoplasmic incompatibility can regulate miRNA biogenesis and its corresponding targeted gene expression. Of these TF genes, the TCP family was reported to be involved in nuclear–mitochondrial communication [12], and homeobox domain protein (PDH) that interacts with ABI4 plays an important role in chloroplast retrograde regulation in *Arabidopsis*[55]. In the present study, we observed the different miRNAs biogenesis in CMS, although we could not completely conclude that the difference on sRNA is cause or effect for CMS occurrence or whether miRNAs are involved in the retrograde regulation or not. However, we can propose that this difference may partially answer for how mitochondrial and nuclear transcriptome interacts.

## Conclusion

**Figure 6:**
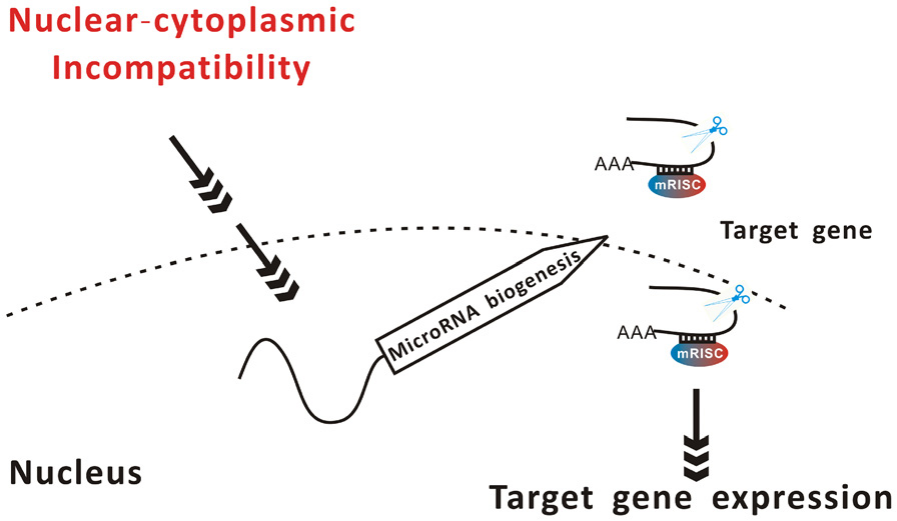
**Proposed model of retrograde regulation of gene expression by the cytoplasmic-nuclear incompatibility by microRNAs during floral development in CMS *Brassica jucnea*.**

In this study, we employed high-throughput sequencing approaches to identify known and new candidate miRNAs and their targets associated with reproductive development in *B*. *juncea*. Comparison of the expression of miRNAs between CMS and MF lines led to a proposal of that microRNA might participate the regulatory network of CMS by tuning fork in genes expressions in CMS *B*. *juncea* (Figure 6).

## Methods

### Plant materials

The CMS *B*. *juncea* was developed previously by interspecific hybridization between *B*. *rapa* as CMS cytoplasm donor and fertile *B*. *juncea*, followed by recurrent backcrossings to fertile *B*. *juncea*. Following 13 generations of backcrossings, we obtained the stable CMS *B*. *juncea* line with fertile *B*. *juncea* being self-crossed as its corresponding maintainer line. All floral buds of an inflorescence from CMS and MF lines of *Brassica juncea* were collected in this experiment. In each case, samples were harvested and pooled from six individual plants. Then samples were immediately frozen in liquid nitrogen and stored at −80°C.

### Total RNA isolation, small RNA library preparation and sequencing

Total RNAs were extracted using the Trizol reagent (Invitrogen, USA) according to the manufacturer’s protocol. Total RNA quantity and purity were assayed with the NanoDrop ND-1000 spectrophotometer (Nano Drop) at 260/280 nm (ratio > 2.0). Small RNA fractions between 10–40 nt were isolated from the total RNA pool with a Novex 15% TBE-Urea gel (Invitrogen).

Small RNAs were 5’ and 3’ RNA adapter-ligated by T4 RNA ligase. The adapter-ligated small RNAs were transcribed to cDNA by Super-Script II Reverse Transcriptase and PCR amplified, using primers that annealed to the ends of adapters. The developed cDNA libraries were subjected to Solexa/Illumina sequencing (LC Sciences).

### Analysis of sequencing data

Raw sequencing reads were processed into clean full-length reads by the BGI small RNA pipeline. Unique small RNAs were then used to query the mRNA (ftp://ftp.arabidopsis.org/Sequences/ATH cDNA EST sequences FASTA/), non-coding RNA sequences database (ftp://ftp.sanger.ac.uk/pub/databases/Rfam/10.1/) and the repeat-Repbase (http://www.girinst.org/repbase/update/index.html). New candidate miRNAs were identified by folding the flanking genome sequence of unique small RNAs using MIREAP (http://sourceforge.net/projects/mireap/), followed by the prediction of secondary structures by Mfold program. Differentially expressed miRNAs in MF and CMS were identified by the online service IDEG6 (http://telethon.bio.unipd.it/bioinfo/IDEG6_form/). The selection methods of differential expression were Audic and Claverie, Fisher’s exact test and chi-squared 2 × 2, with the selection threshold of 0.01 (http://telethon.bio.unipd.it/bioinfo/IDEG6_form/detail.html#AC). Finally, all data were submitted to the database (http://www.ncbi.nlm.nih.gov/geo).

### Degradome sequencing and analysis

Degradome cDNA libraries using sliced ends of polyadenylated transcripts from reproductive development in MF and CMS *B*. *juncea* were constructed based on the method described previously [21,22]. Identification and classification of categories of the sliced miRNA targets were processed according to the CleaveLand 3.0 pipeline [21].

### Real-time quantitative-PCR

The expression of 8 selected miRNAs was assayed in CMS and MF lines of *B*. *juncea* by Platinum SYBR Green-based q-PCR (Invitrogen, 11733–038) with the High-Specificity miRNA QuantiMir RT Kit (RA610A-1, System Biosciences) on ABI 7900. The primers of 8 selected miRNAs and 2 internal control genes (U6 snRNA and actin) are available in Additional file 1: Table S13.

The expression of selected target gene *APS1* was assayed in CMS and MF lines of *B*. *juncea* by Real-Time PCR. Real-time PCR reactions were performed according to a previously established method. Primers used are listed in Additional file 1: Table S13. All the gene expression data were obtained from three individual biological replicates and processed according to strict statistical methods.

## Competing interests

The authors have declared that no competing interests exist.

## Authors’ contributions

JY, XL and MZ conceived and designed the experiments. BX, NZ and XY performed the qPCR experiments. JY, XL and XY analyzed the data. JY wrote the paper. All authors read and approved the final manuscript.

## Supplementary Material

Additional file 1 **In this additional table, it includes 13 sub-tables.** All the annotations of the tables are followings, also in the additional table.
**Table S1.** Distribution of counts of sequ-seqs during standard filtering in two libraries.
** Table S2.** Distribution of counts of mappable reads in two libraries.
** Table S3.** Length distribution of mappable counts and unique sRNAs of sequ-seqs type in two libraries.
** Table S4.** Brassica juncea miRNA detection information referred to Arabidopsis (miRBase 18.0).
** Table S5.** Profile of known miRNAs in B. juncea referred to Arabidopsis (miRBase18.0).
** Table S6.** Profile of novel miRNAs originating from other plant pre-miRNAs (miRbase 18.0) that can be mapped to the Arabidopsis genome.
** Table S7.** Profile of novel miRNAs originating from pre-miRNAs that could not be mapped to the Arabidopsis genome; however, novel miRNAs were mapped to the genome and the extended sequences at the mapped positions of the genome were potentially from hairpins.
** Table S8.** Profile of novel miRNAs originating from pre-miRNAs that could not be mapped to the Arabidopsis genome; however, novel miRNAs were mapped to the genome and the extended sequences at the mapped positions of the genome were not potentially from hairpins.
** Table S9.** Profile of novel miRNAs originating from pre-miRNAs that could not be mapped to the Arabidopsis genome; additionally, novel miRNAs could not be mapped to the Arabidopsis genome.
** Table S10.** Profile of candidate miRNAs originating from predicted RNA hairpins.
** Table S11.** Profiles of all the microRNAs discovered in reproductive development of B. juncea.
** Table S12.** Overall information of miRNA targets by degradome sequencing and Cleveland analysis in B. juncea.
**Table S13.** Primers used in this study. Click here for file

Additional file 2 **Figure S1.** Prediction of secondary structure of all new candidate miRNAs during floral development of *Brassica juncea*. Click here for file

Additional file 3 **Figure S2.** Target plot (t-plot) of representative validated miRNAs target in MF of *Brassica juncea*. Click here for file

Additional file 4 **Figure S3.** Target plot (t-plot) of representative validated miRNAs target in CMS of *Brassica juncea*. Click here for file
