# Supplementary material for: Identification of miRNAs and their targets using high-throughput sequencing and degradome analysis in cytoplasmic male-sterile and its maintainer fertile lines of brassica juncea
Source: BMC Genomics. 2013 Jan 16;14:9. doi: 10.1186/1471-2164-14-9 (PMC3553062; doi:10.1186/1471-2164-14-9)

Secondary structure prediction of candidate pre-microRNAs based on Table S10

Pre-miRNA 1

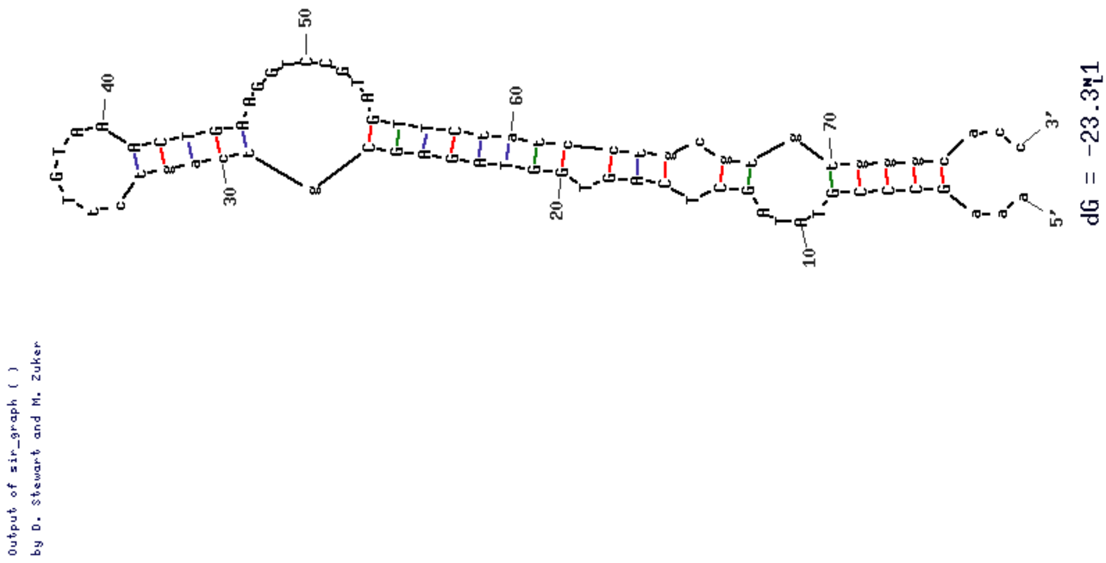

Pre-miRNA 2

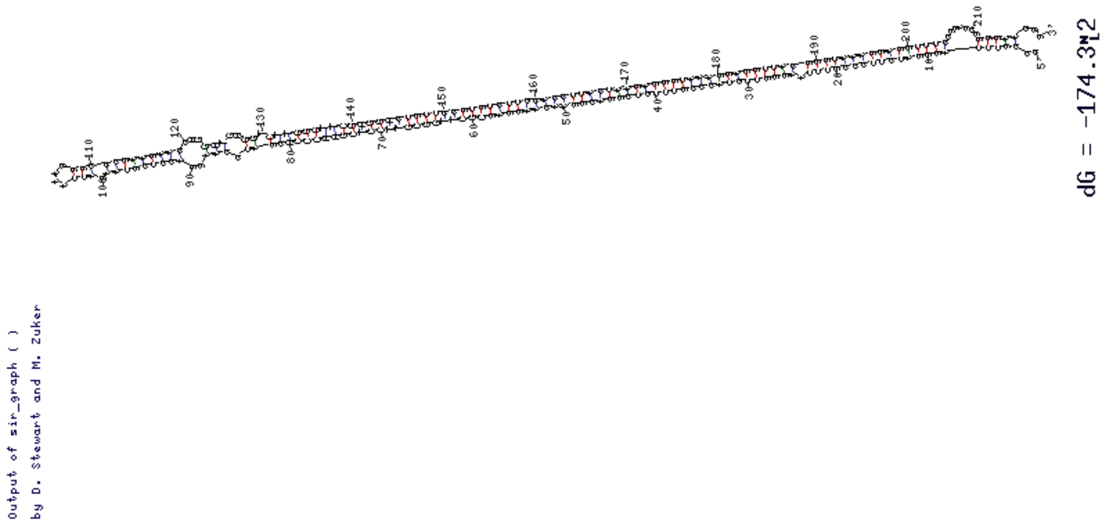

Pre-miRNA 3

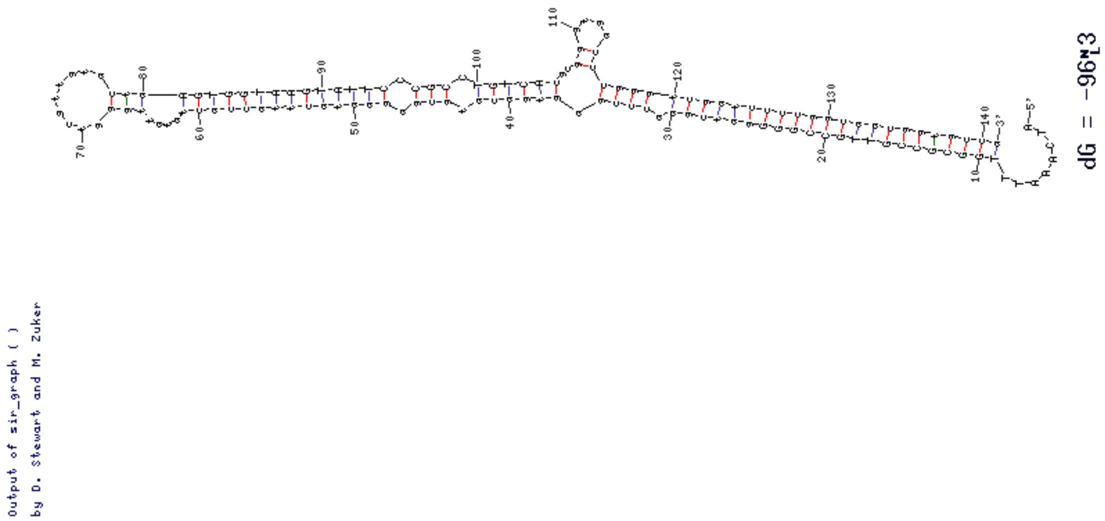

Pre-miRNA 4

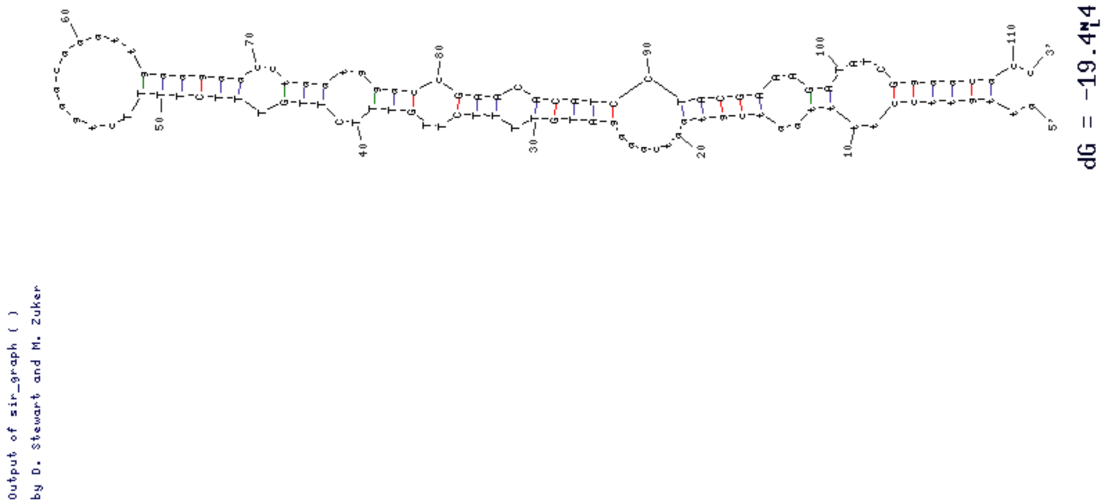

Pre-miRNA 5

Output of sir_graph ( )
by D. Stewart and M. Zuker

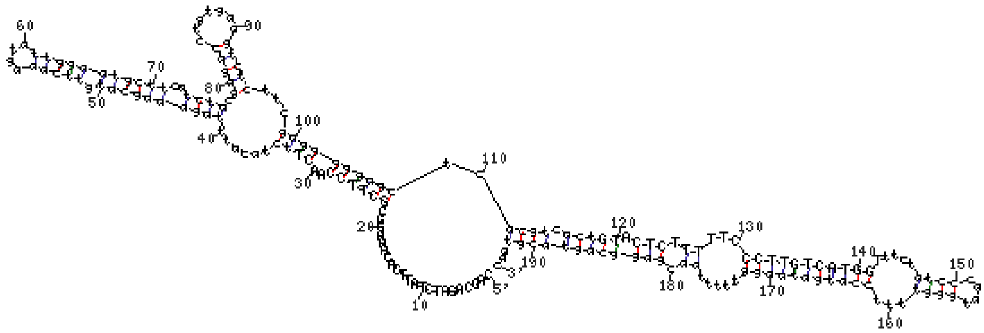

dG = -57.6[illegible]5

Pre-miRNA 6

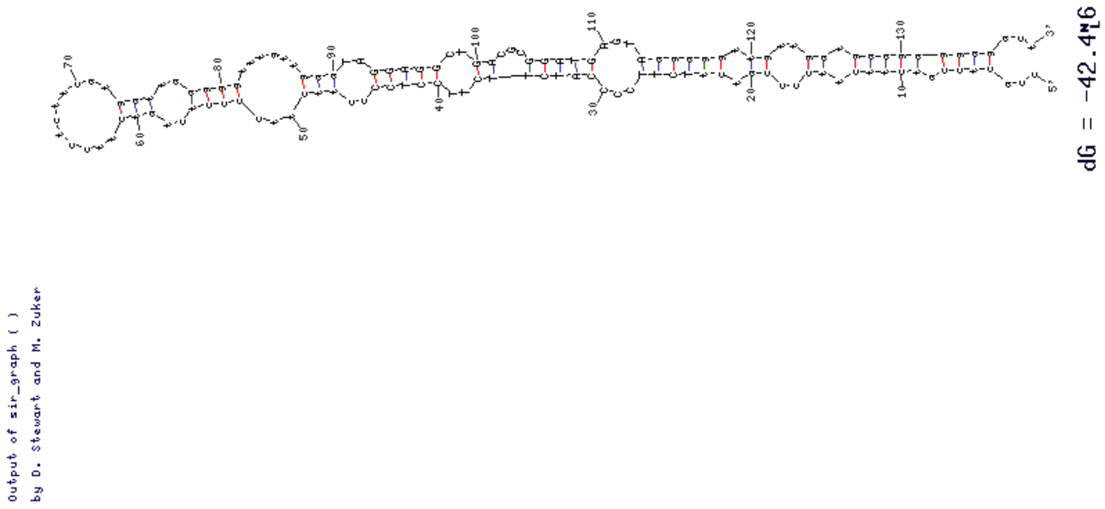

Pre-miRNA 7

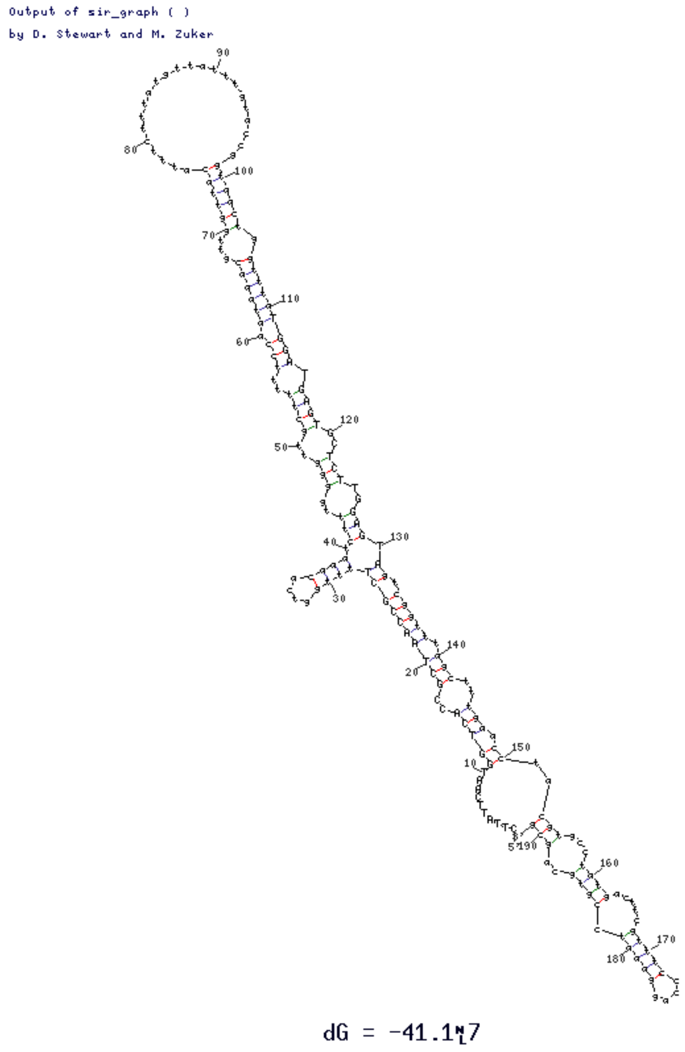

Pre-miRNA 8

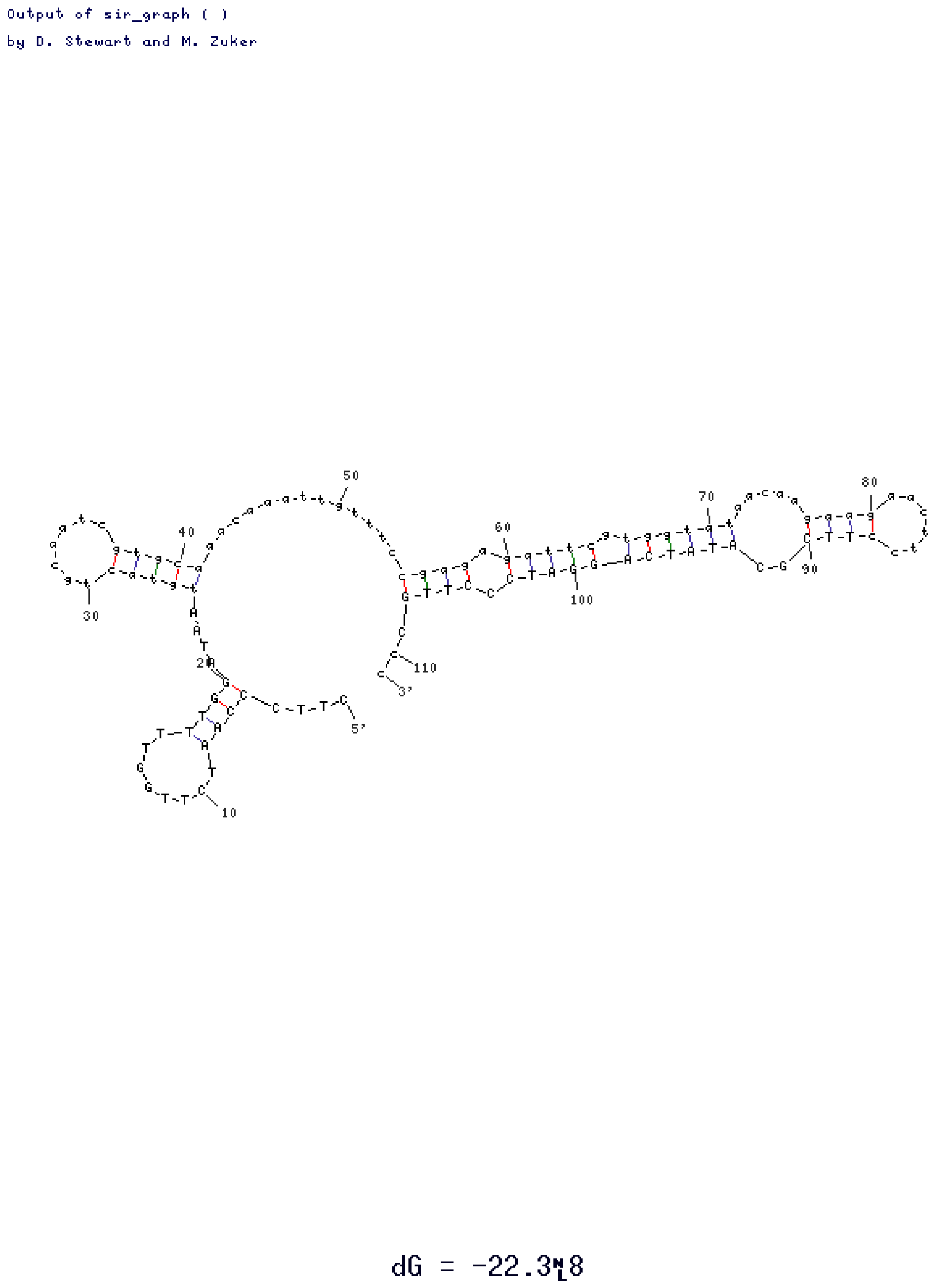

Pre-miRNA 9

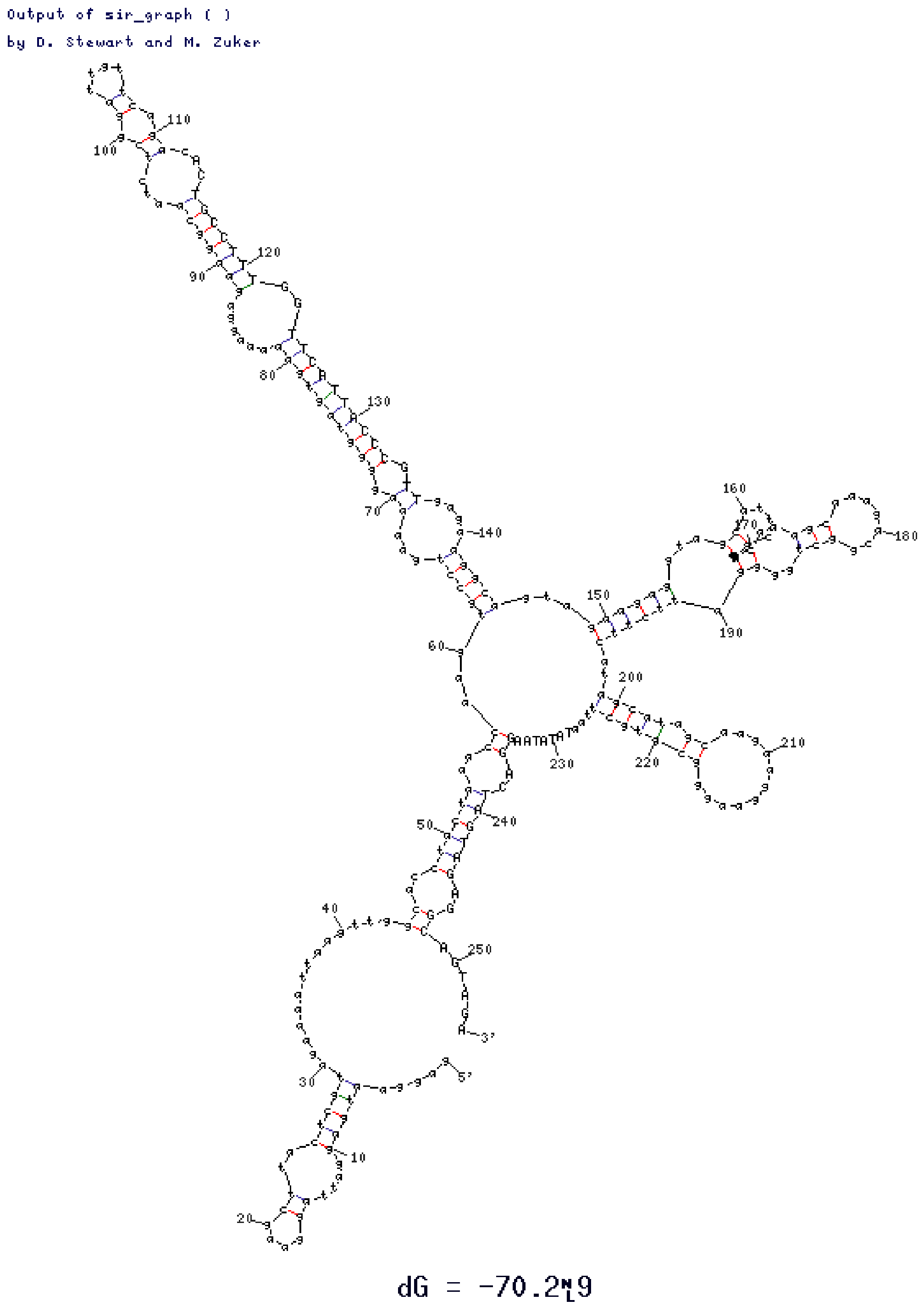
Output of sir_graph ( )
by D. Stewart and M. Zuker
5'
3'
10
20
30
40
50
60
70
80
90
100
110
120
130
140
150
160
170
180
190
200
210
220
230
240
250
dG = -70.2

Pre-miRNA 10

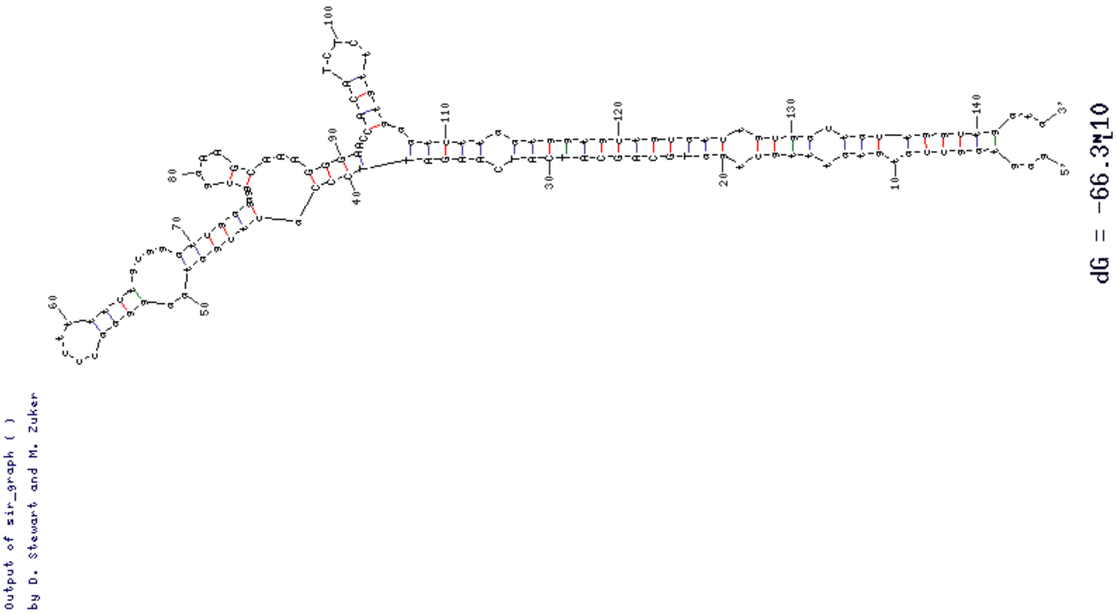

Pre-miRNA 11

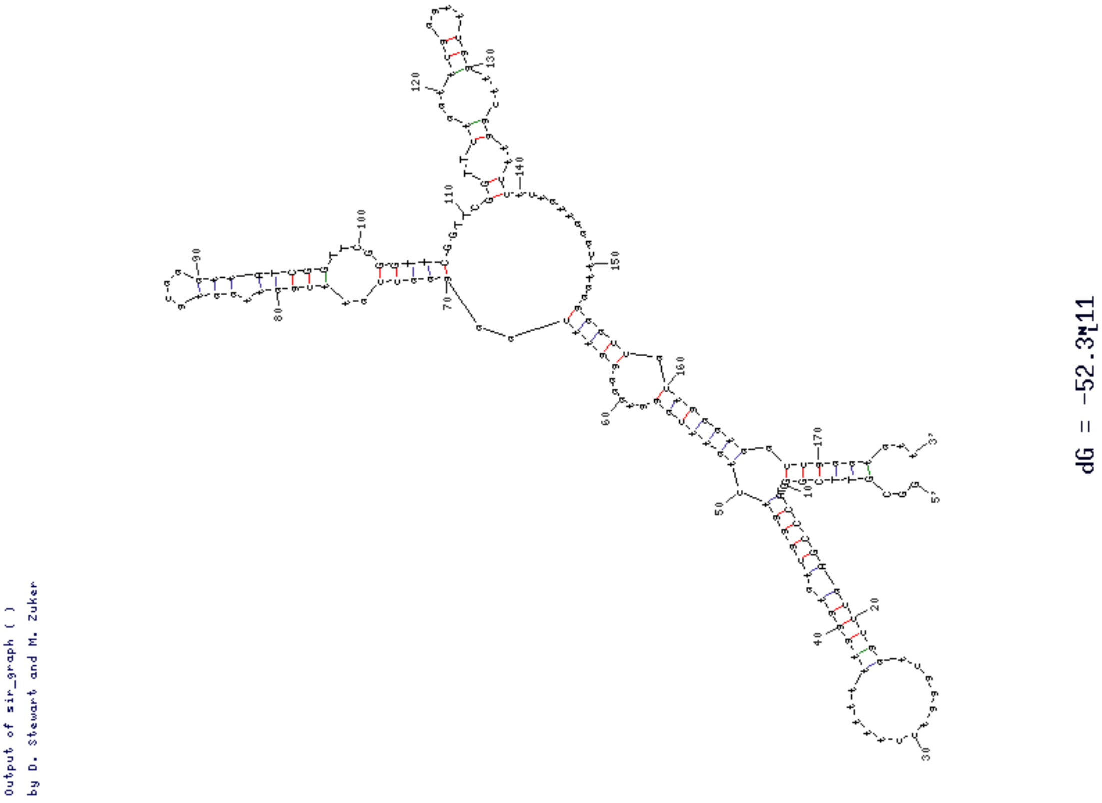

Pre-miRNA 12

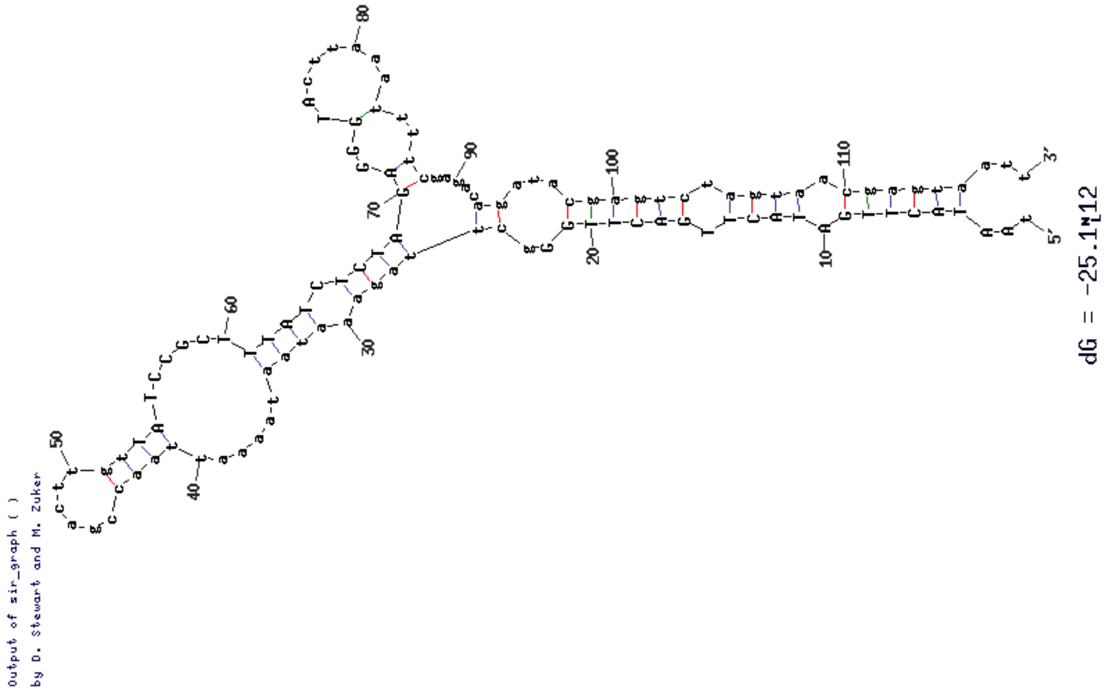

Pre-miRNA 13

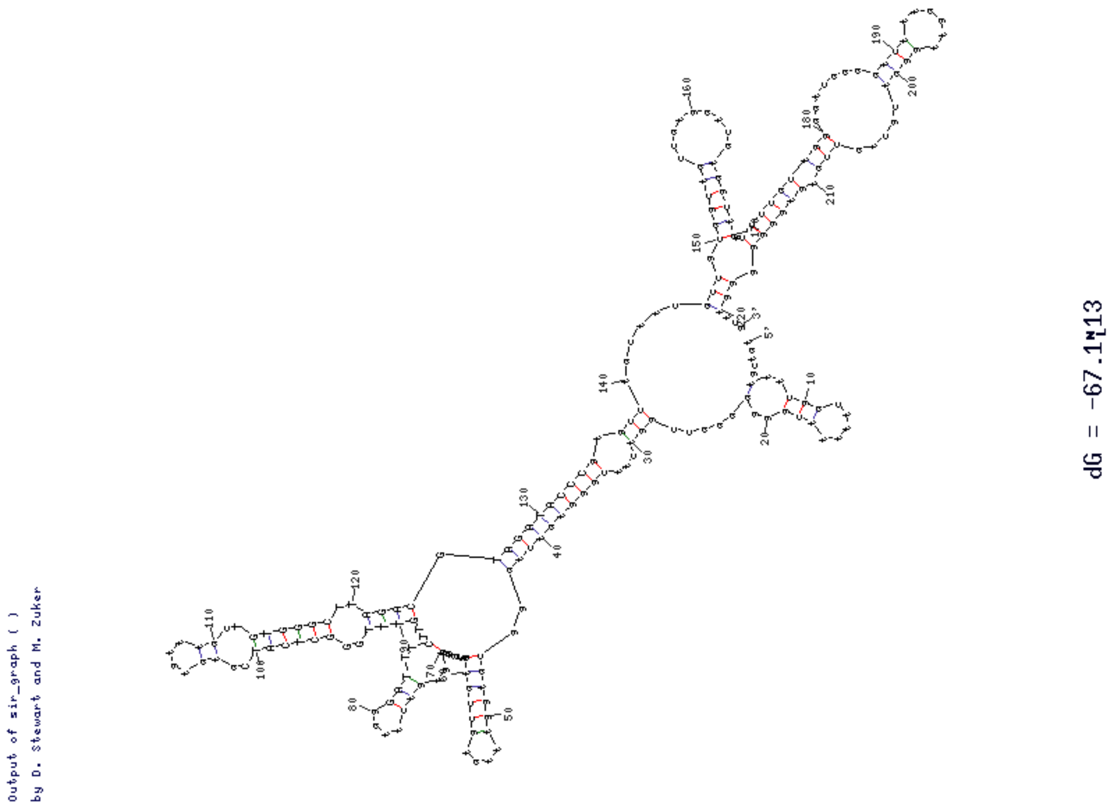

Pre-miRNA 14

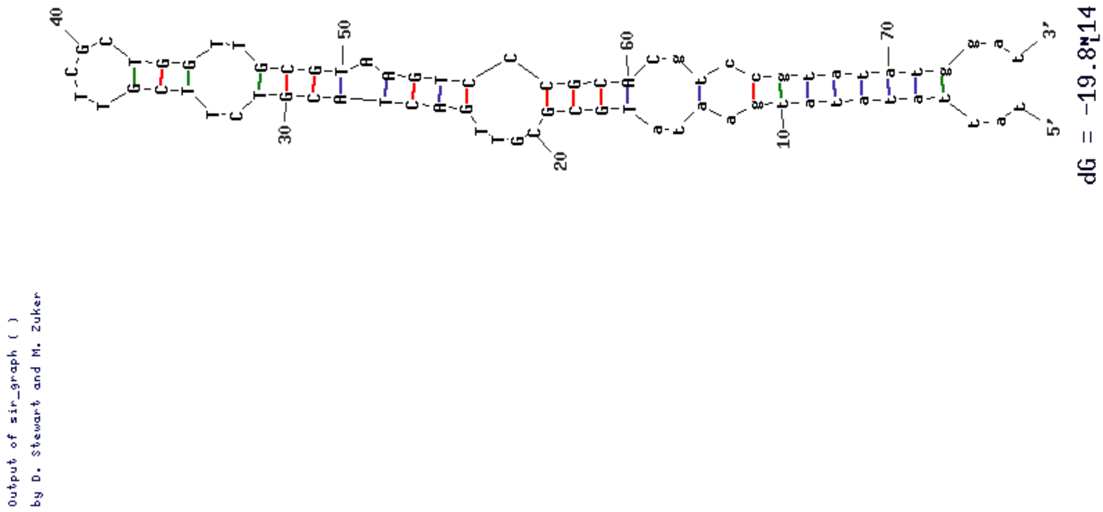

Pre-miRNA 15

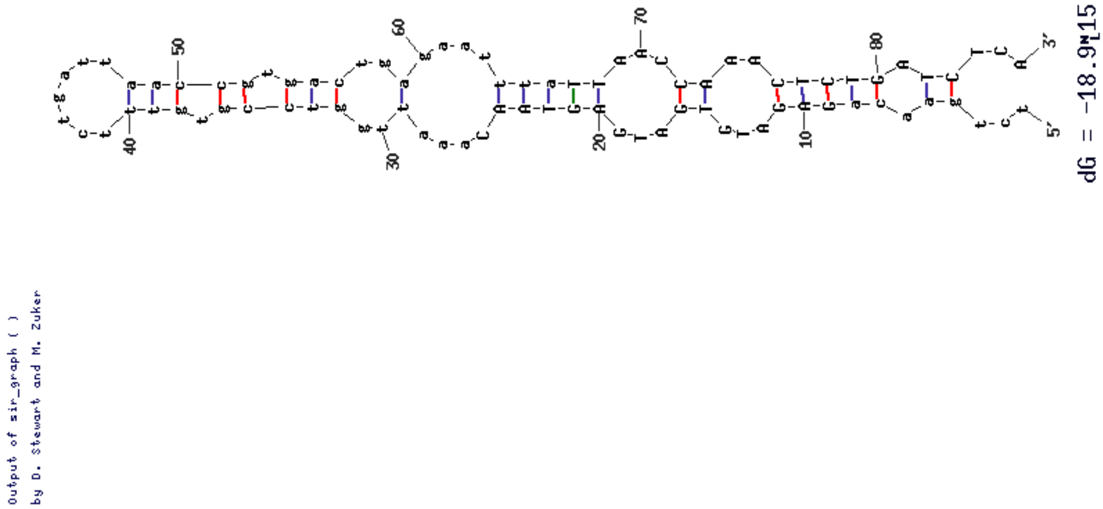

Pre-miRNA 16

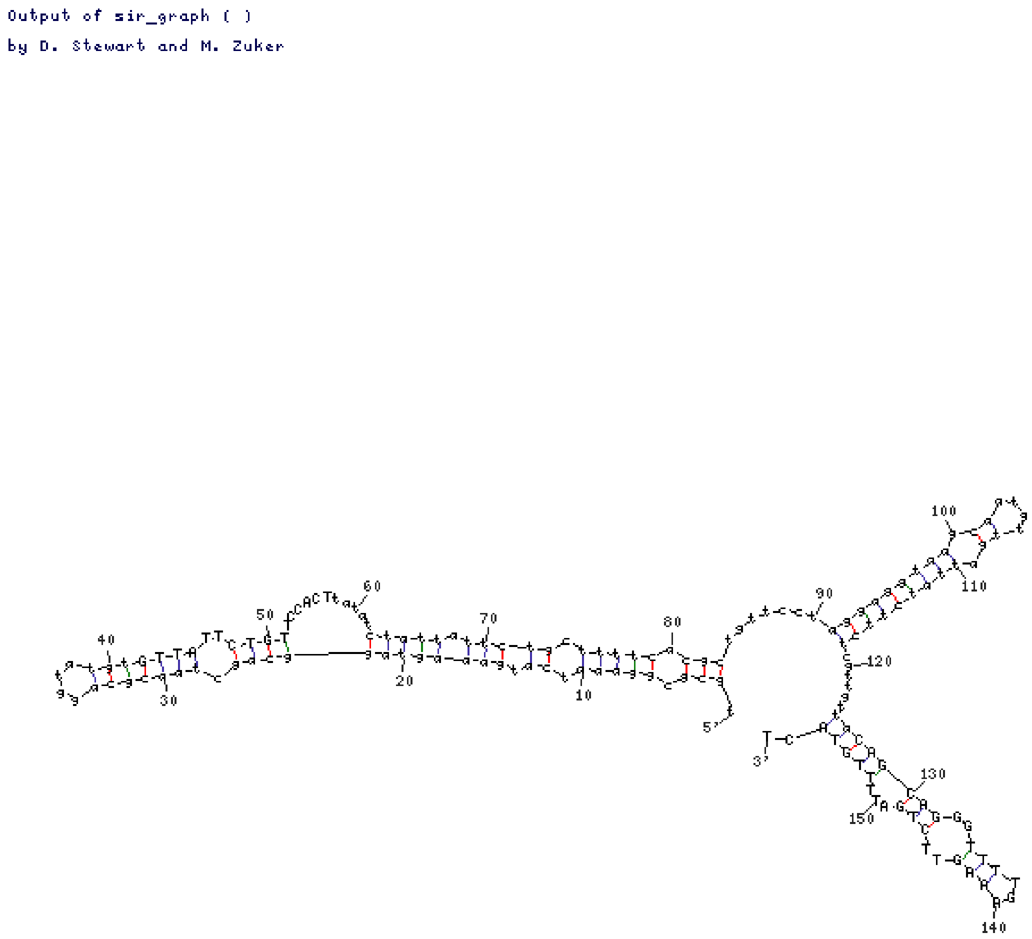

dG = -34.3 16

Pre-miRNA 17

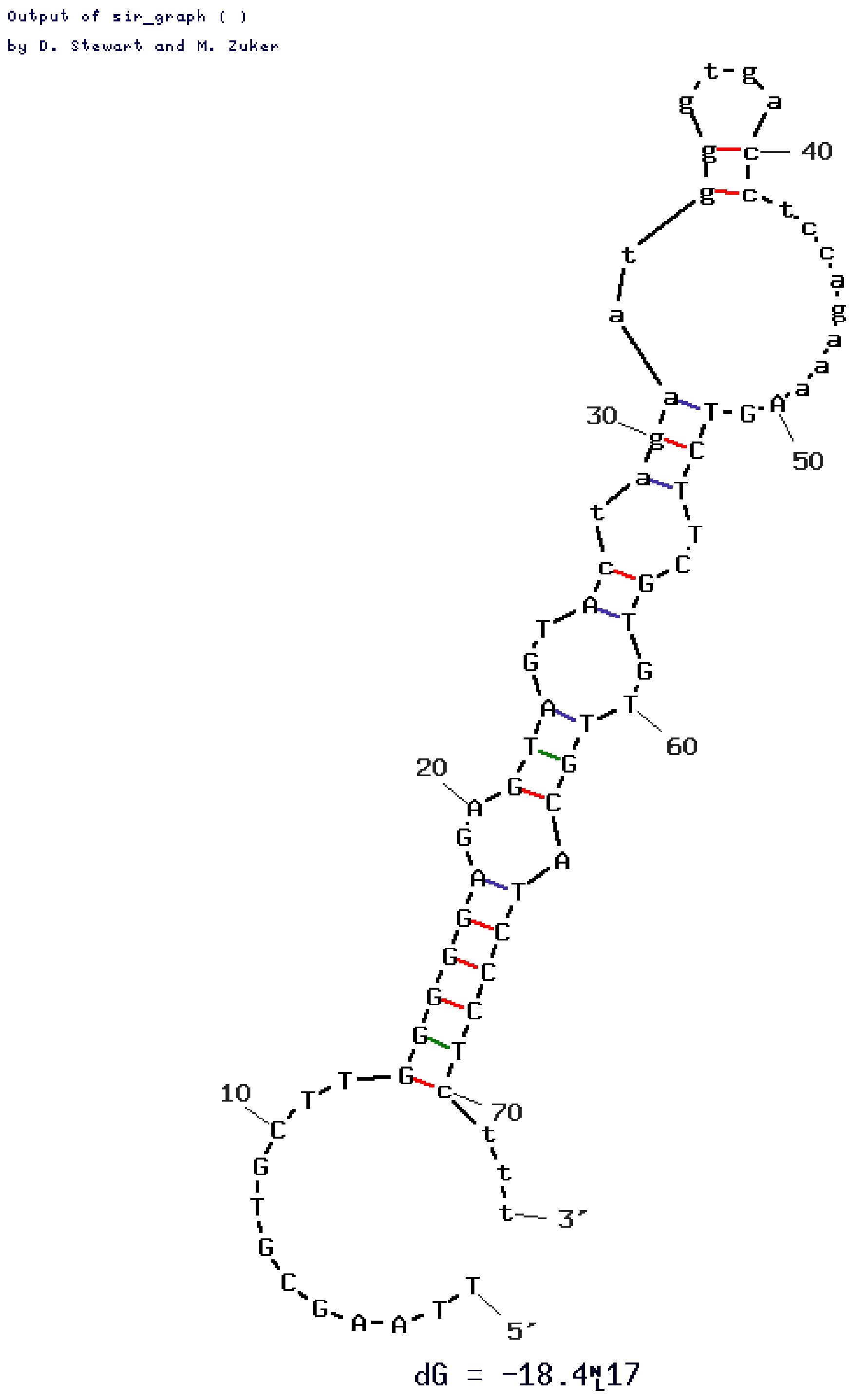
Output of sir_graph ( )
by D. Stewart and M. Zuker
5′
3′
10
20
30
40
50
60
70
dG = -18.4

Pre-miRNA 18

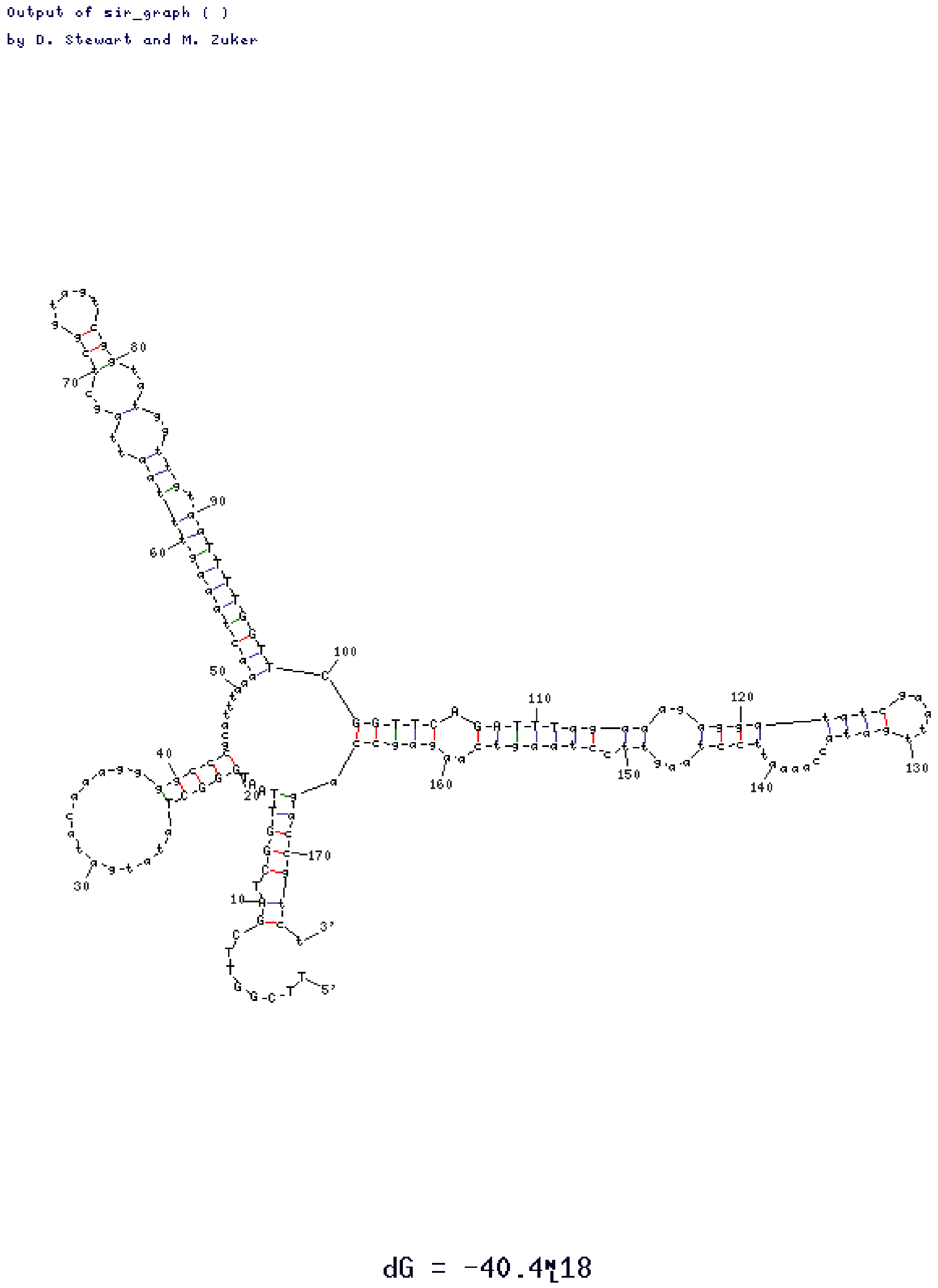

Pre-miRNA 19

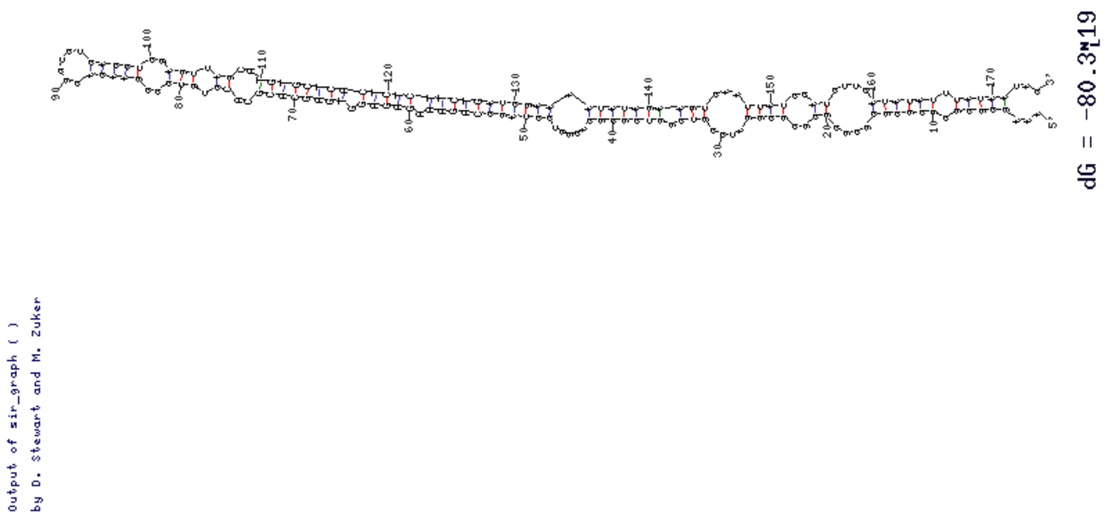

Pre-miRNA 20

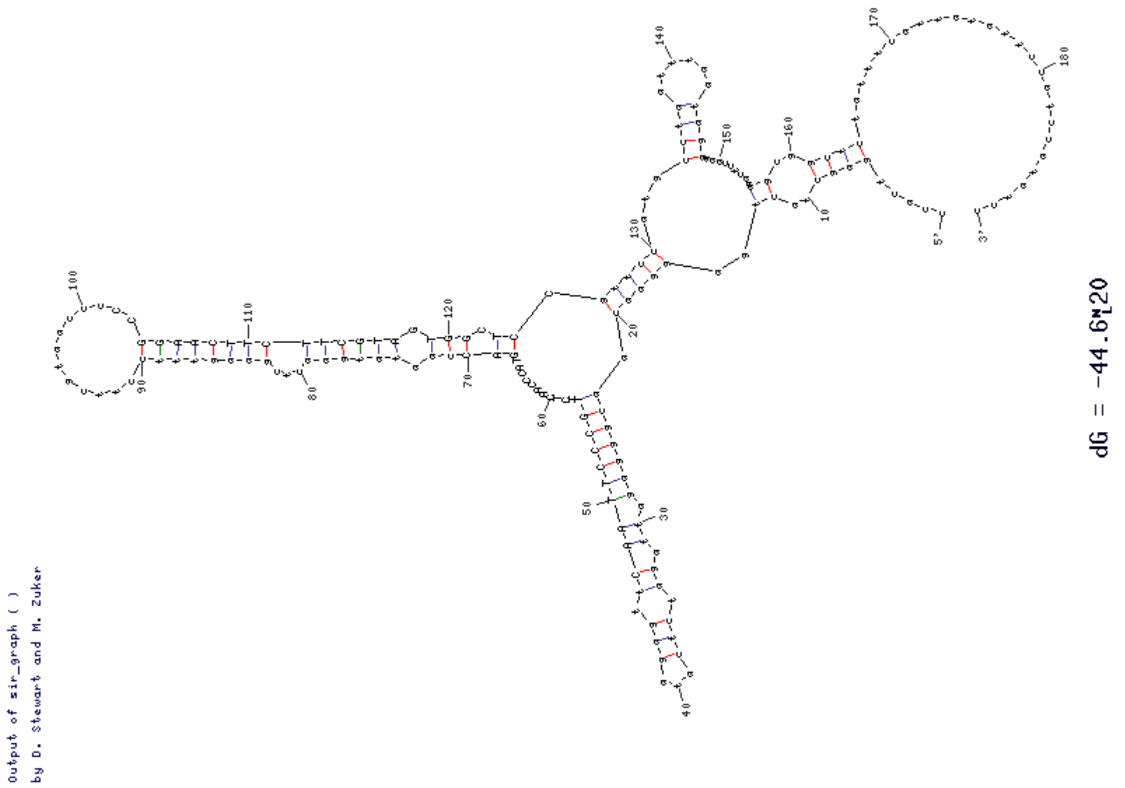

Pre-miRNA 21

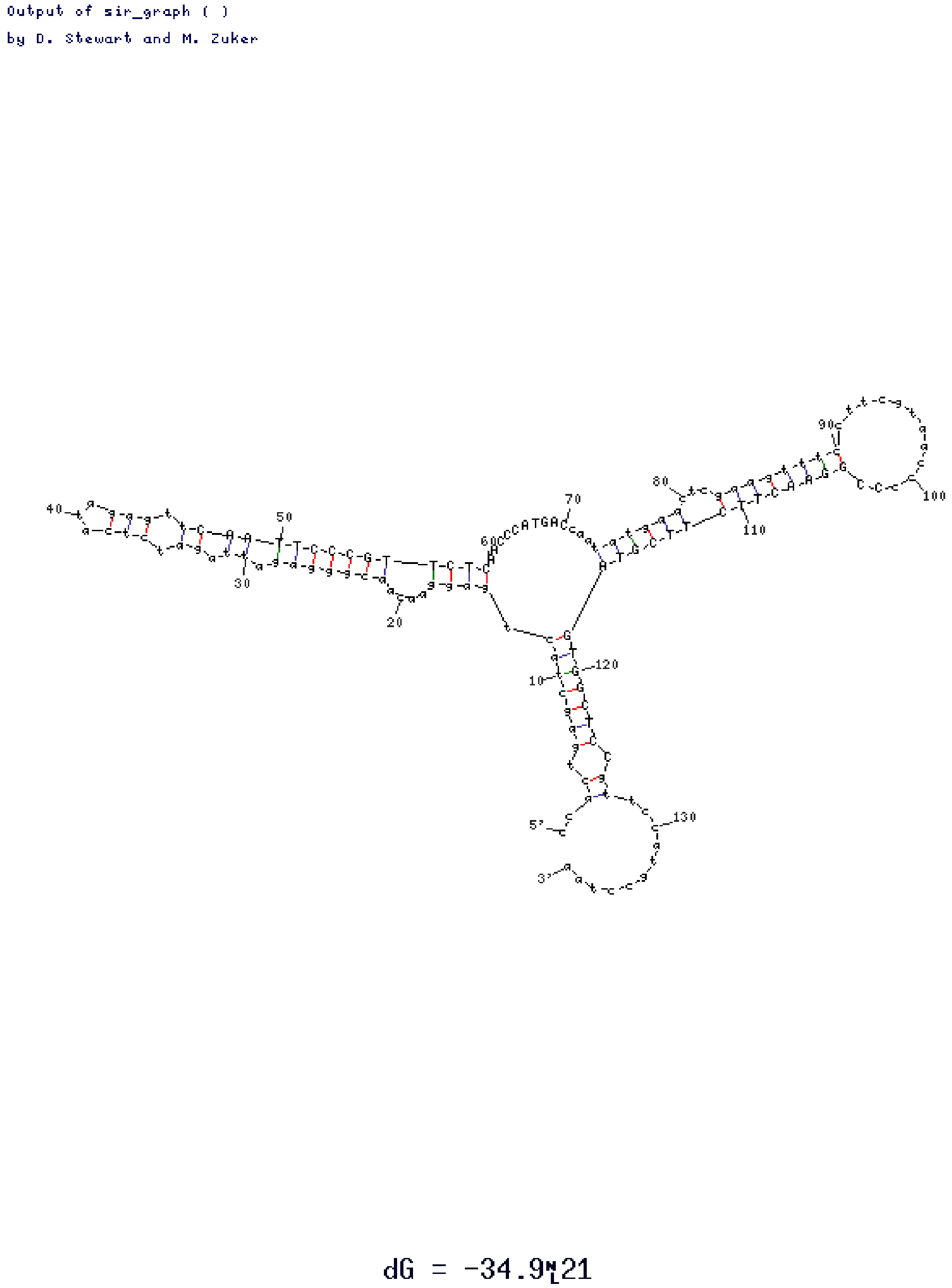
Output of sir_graph ( )
by D. Stewart and M. Zuker
40
50
30
20
60
70
80
90
100
110
120
10
130
5'
3'
dG = -34.9

Pre-miRNA 22/ Pre-miRNA 23

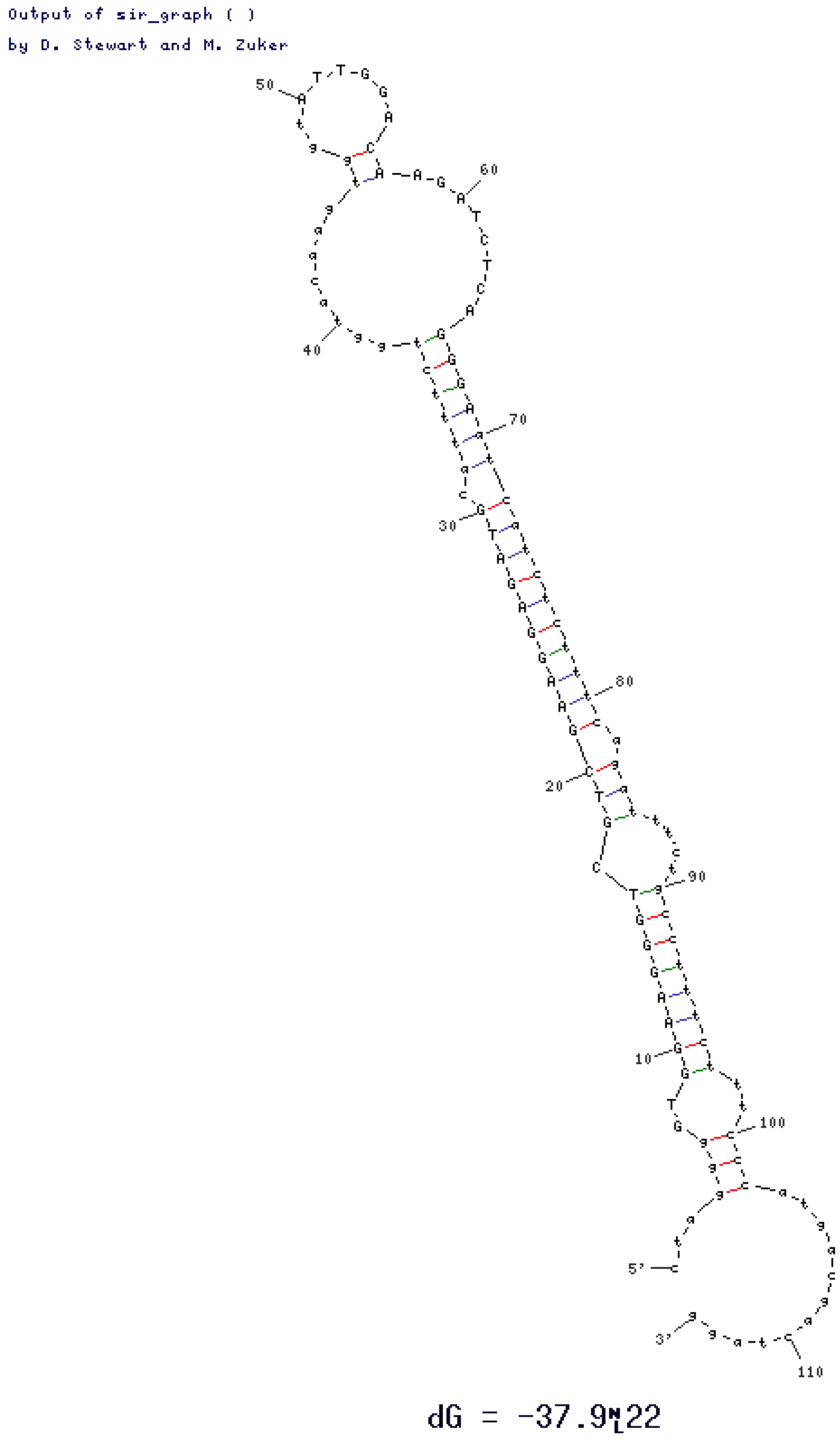

Pre-miRNA 24/ Pre-miRNA 25

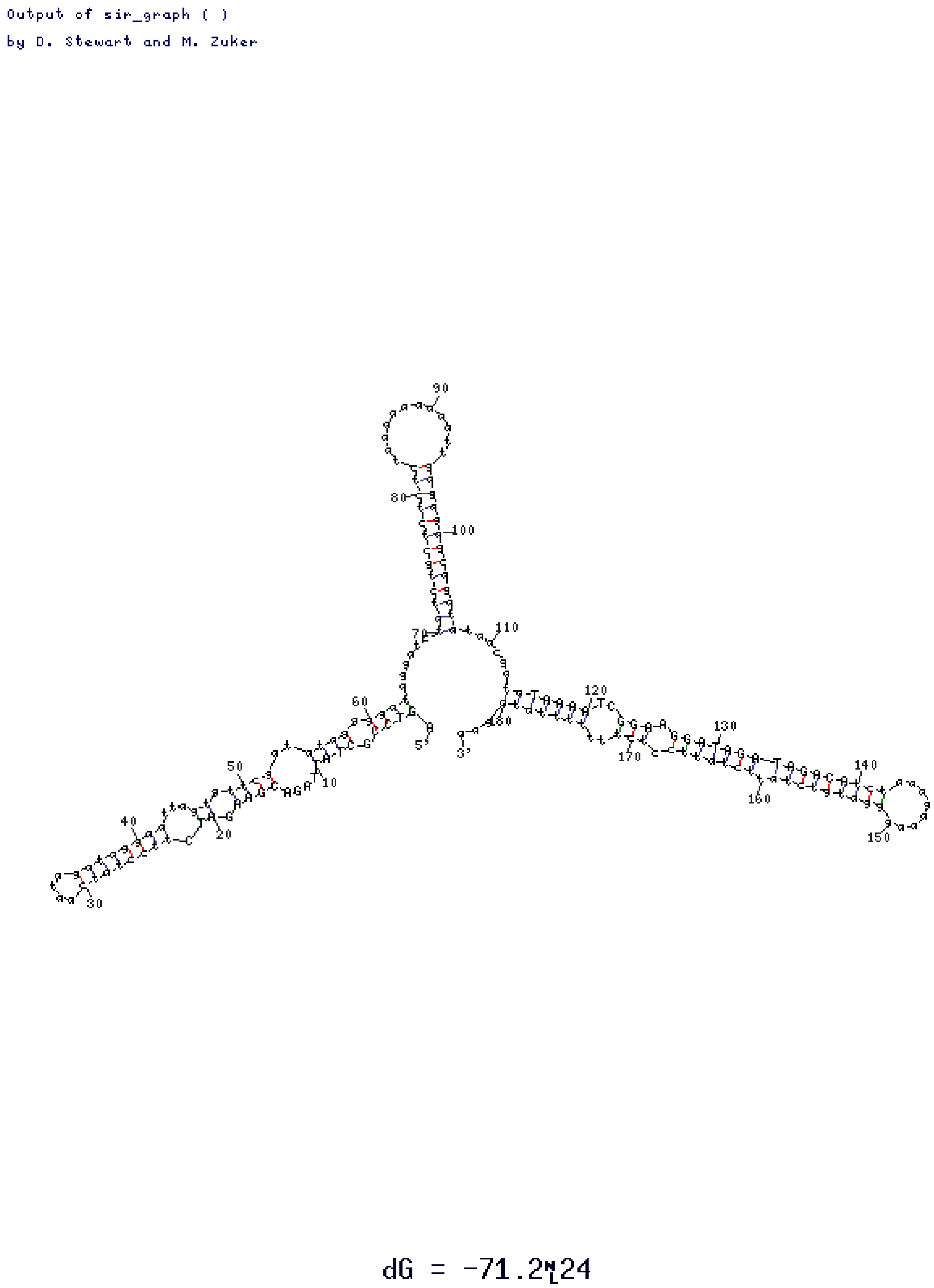

Pre-miRNA 26

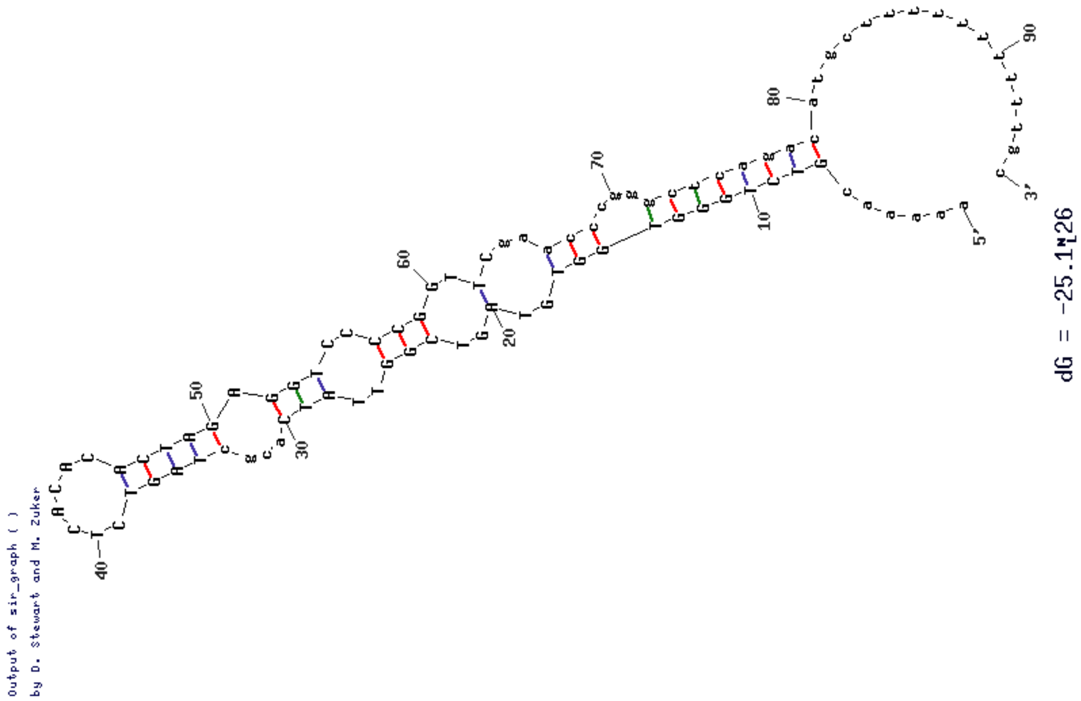

Pre-miRNA 27

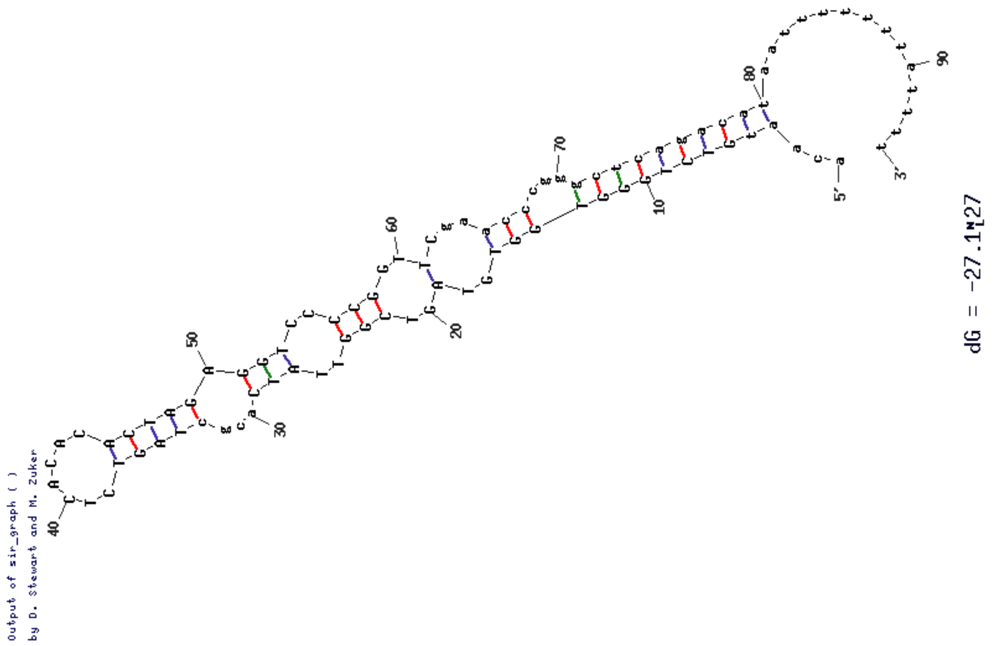

Pre-miRNA 28

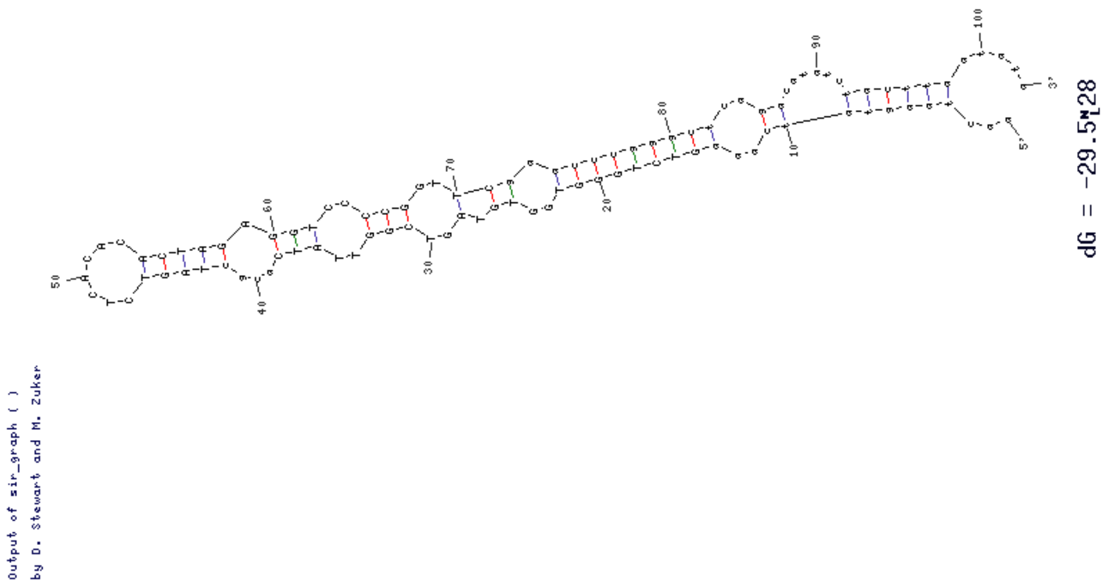

Pre-miRNA 29

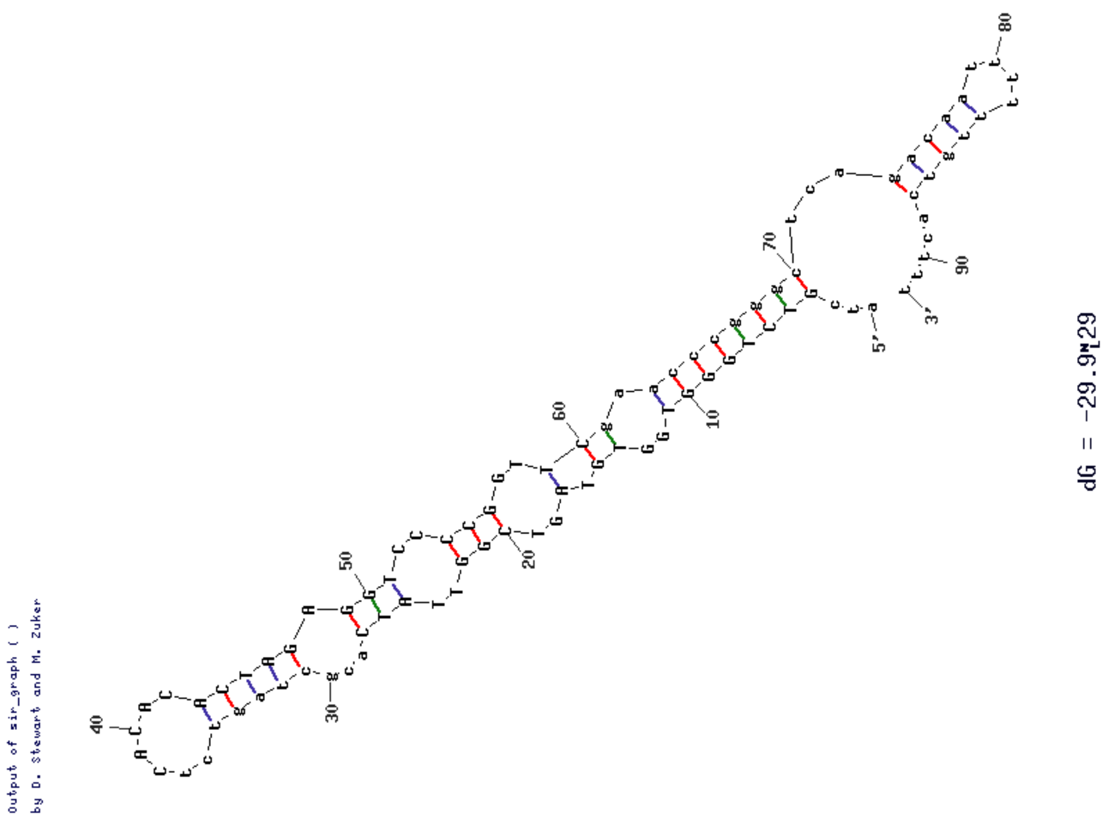

Pre-miRNA 30

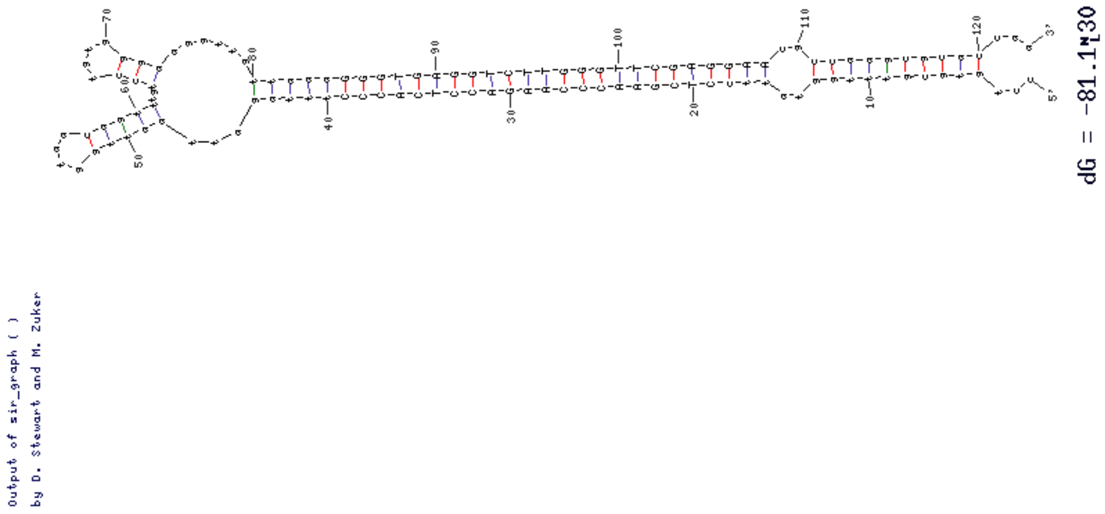

Pre-miRNA 31

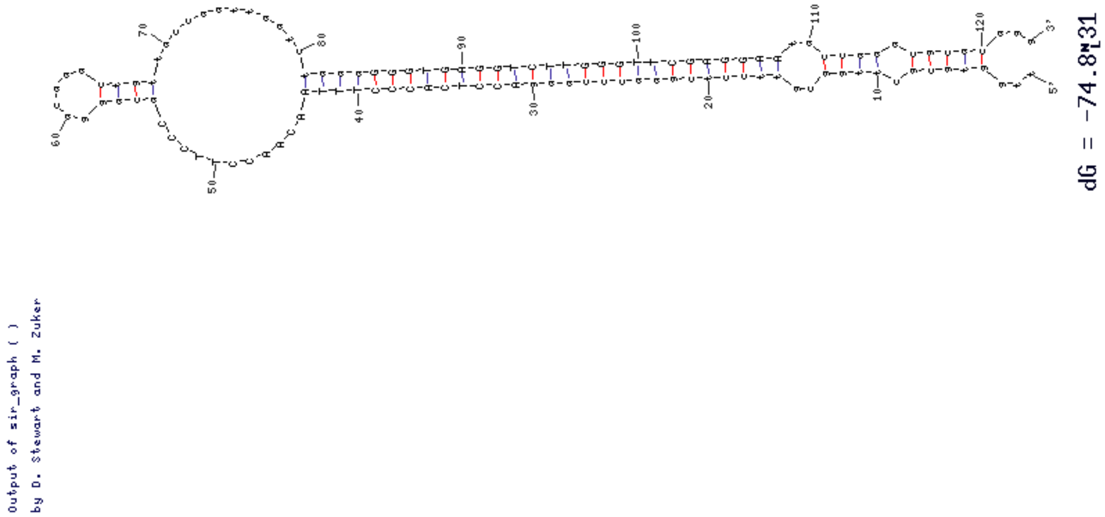

Pre-miRNA 32

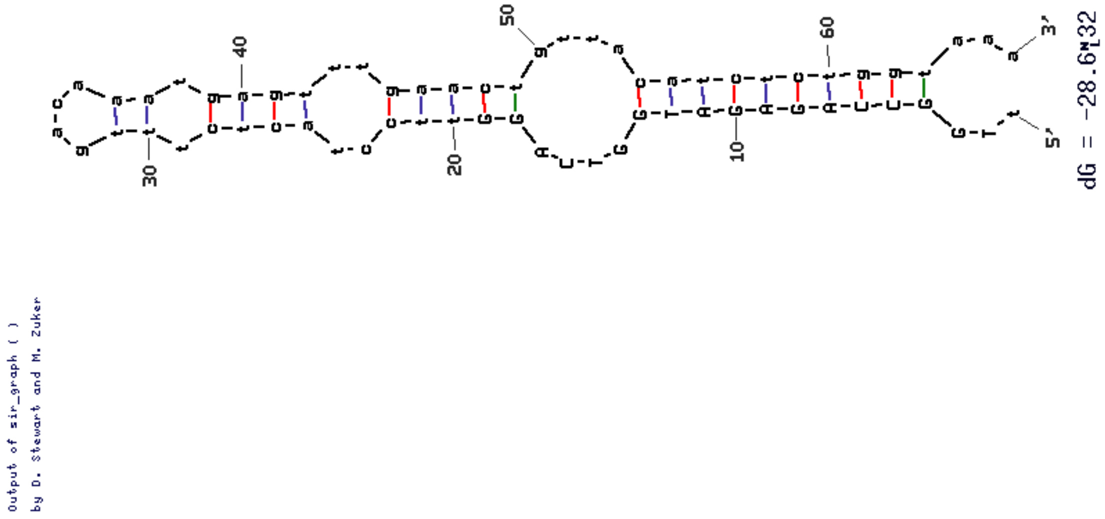

Pre-miRNA 33

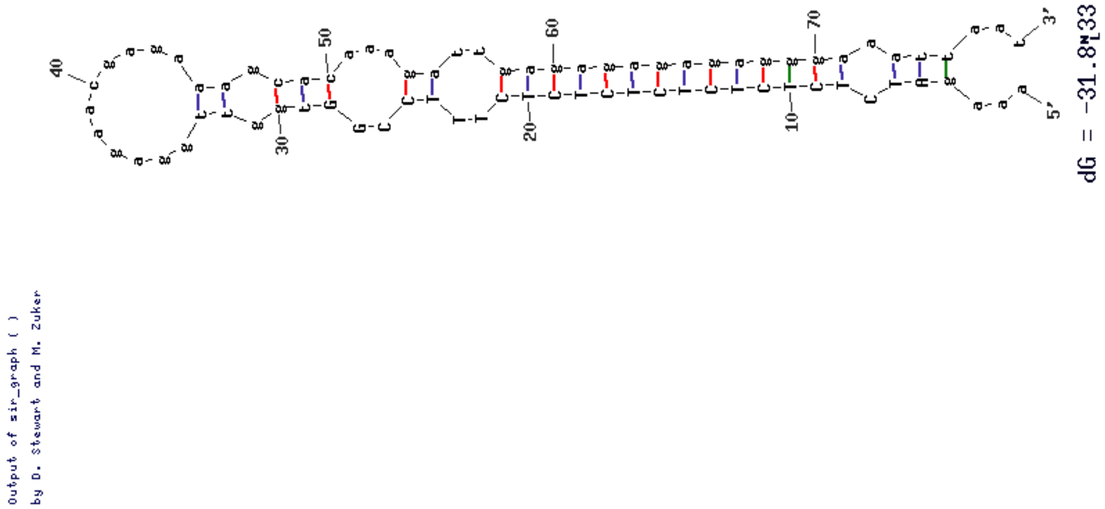

Pre-miRNA 34

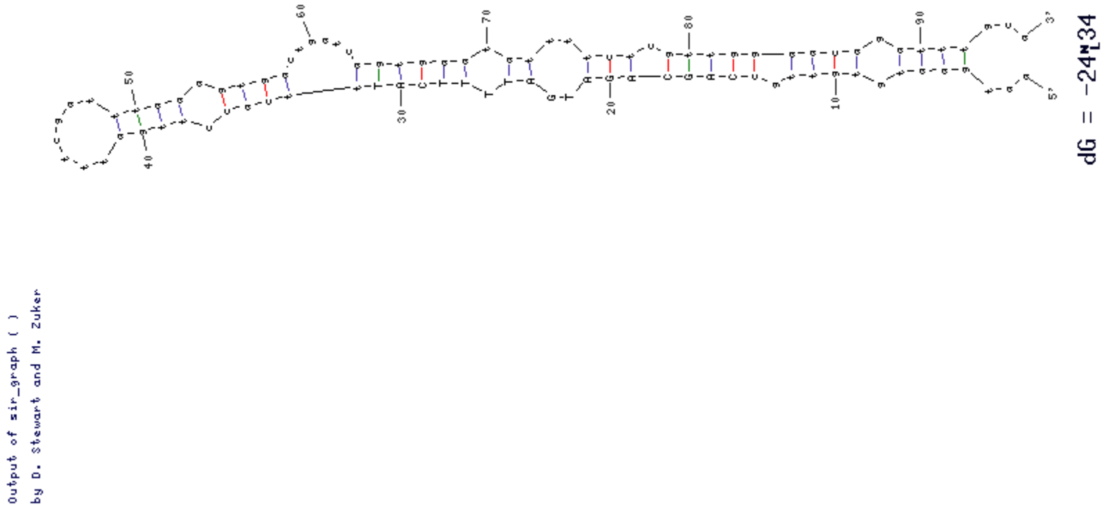

Pre-miRNA 35

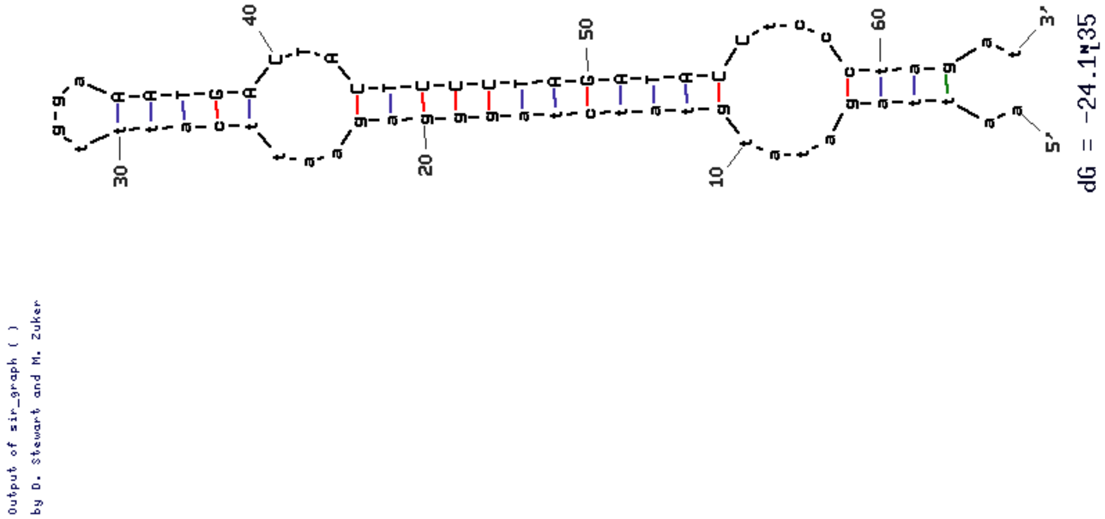

Pre-miRNA 36

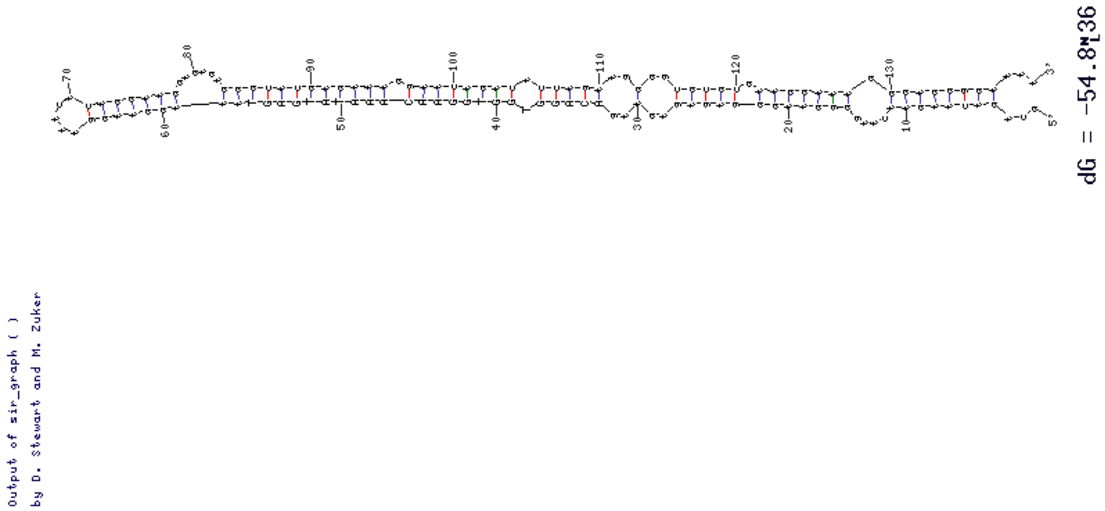

Pre-miRNA 37

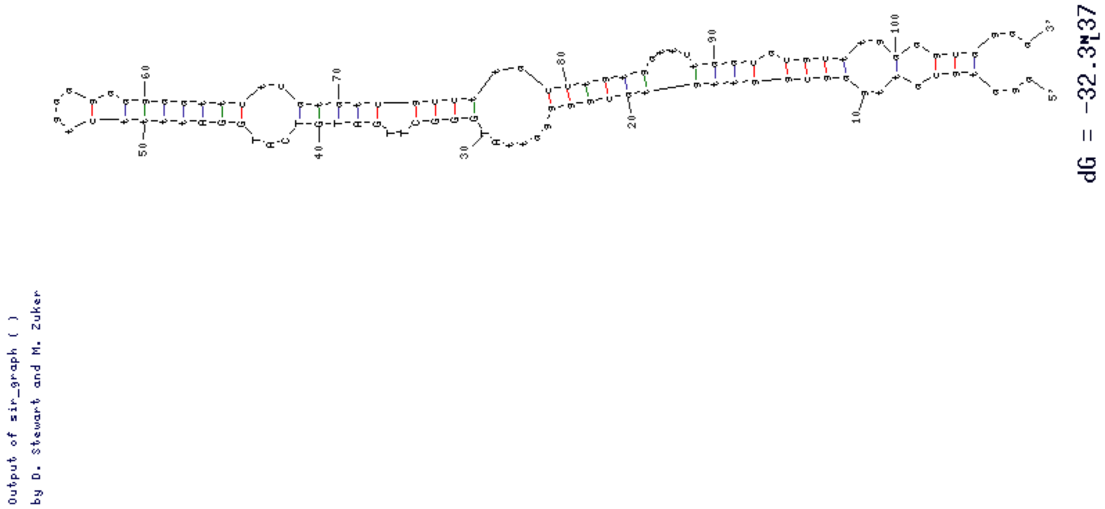

Pre-miRNA 38

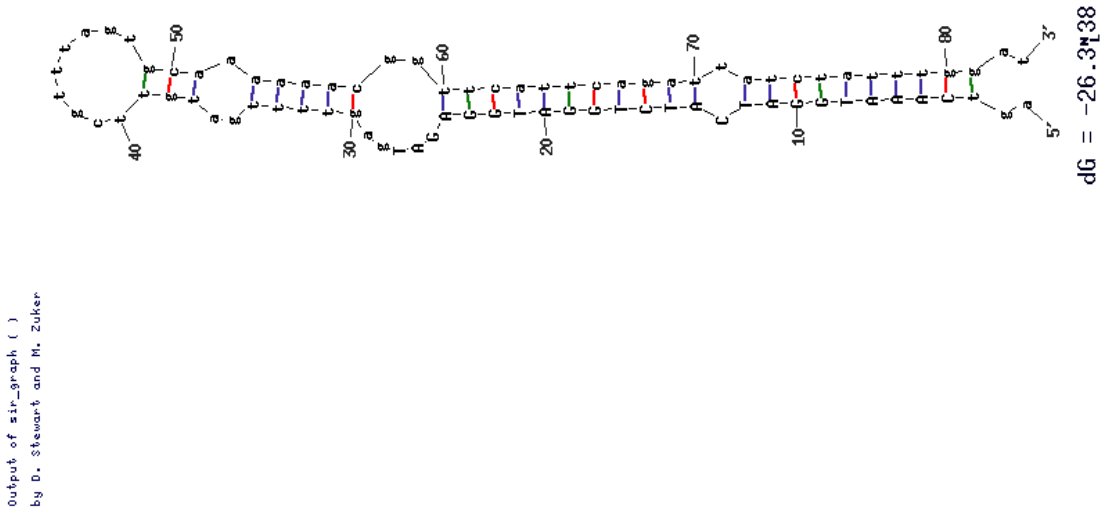

Pre-miRNA 39

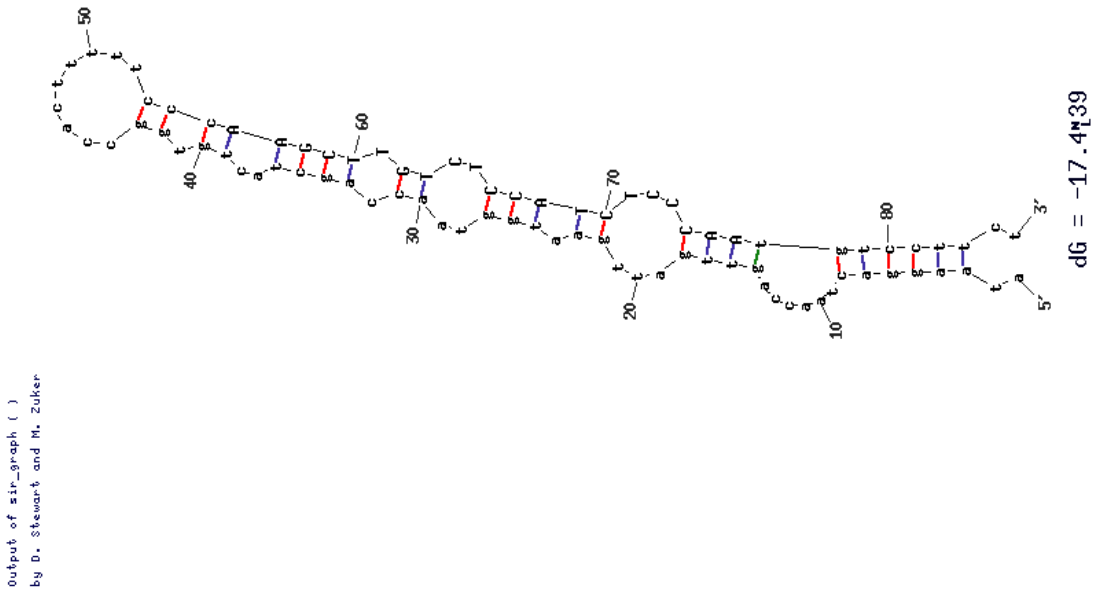

Pre-miRNA 40

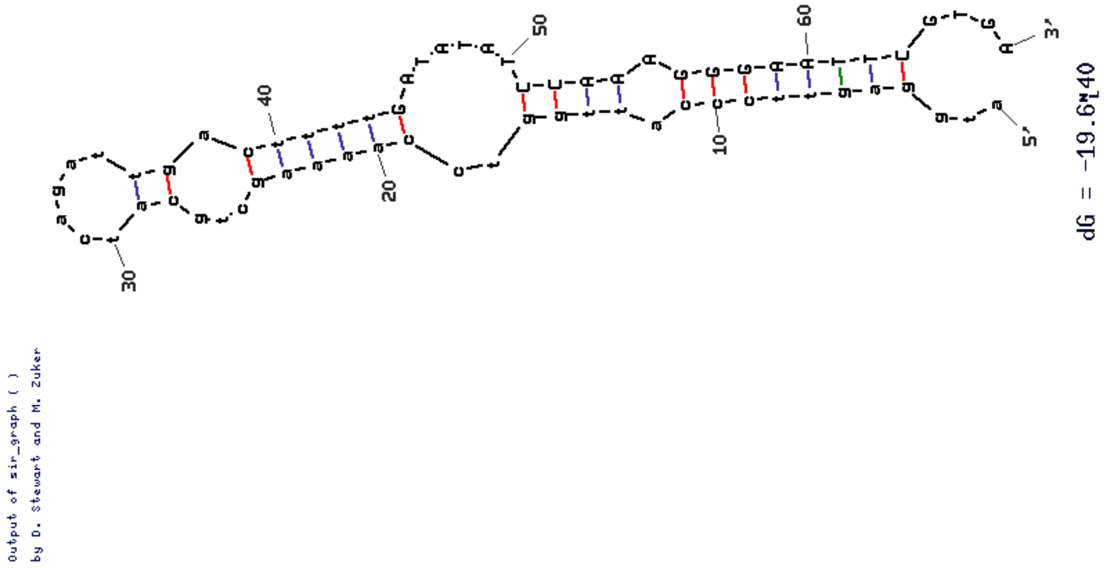

Pre-miRNA 41

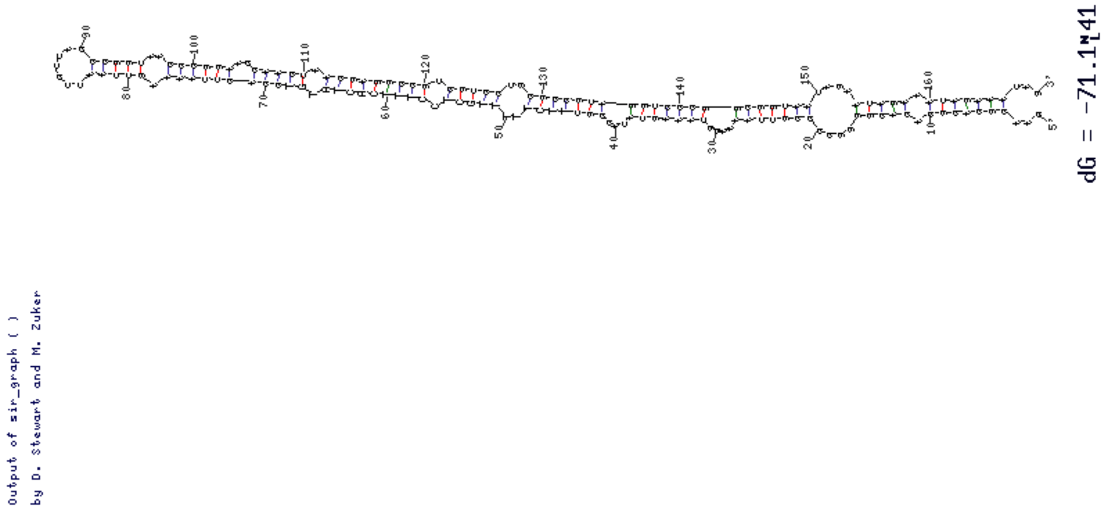

Pre-miRNA 42

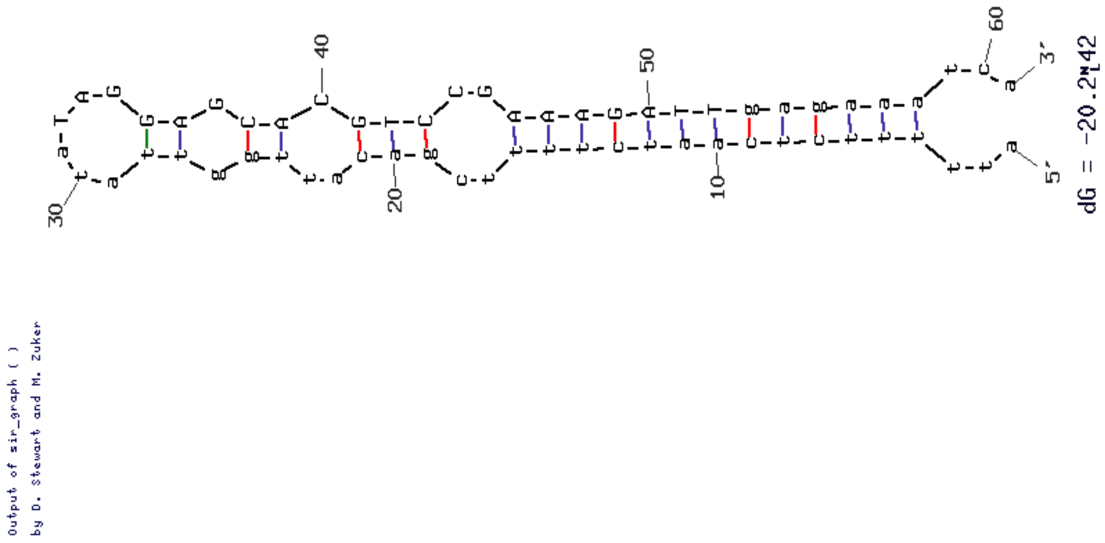

Pre-miRNA 43

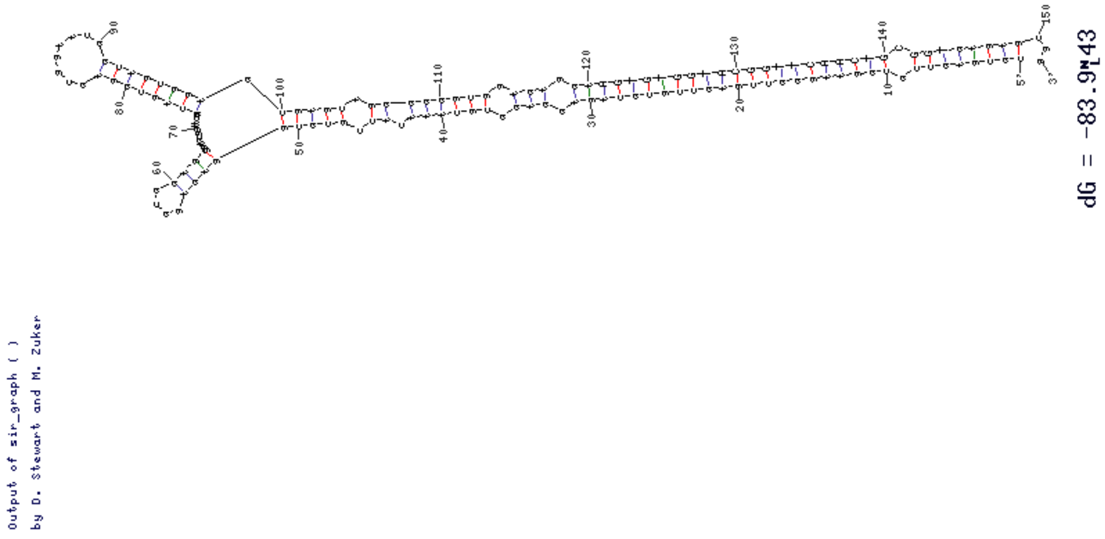

Pre-miRNA 44

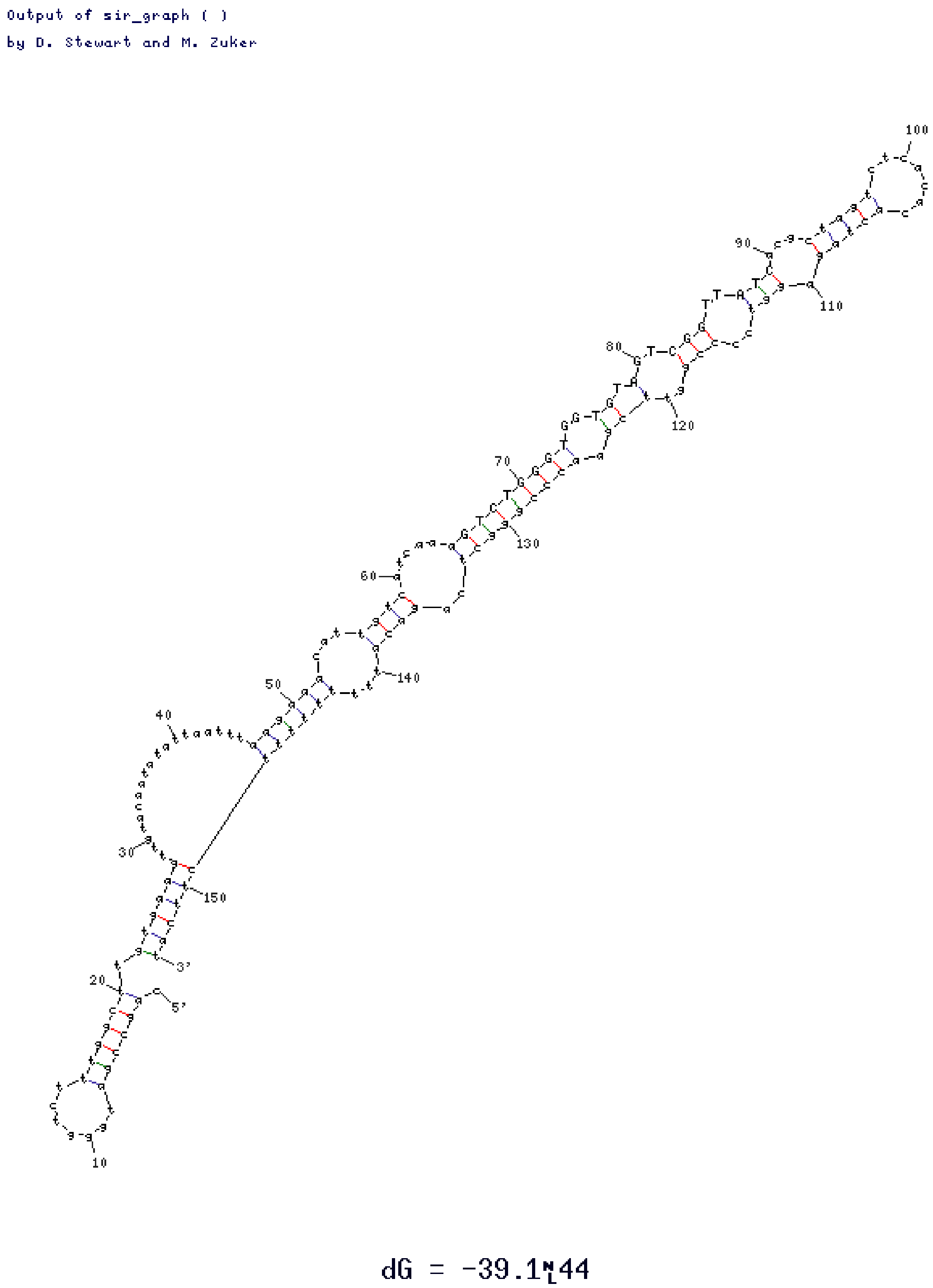
Output of sir_graph ( )
by D. Stewart and M. Zuker
5'
3'
10
20
30
40
50
60
70
80
90
100
110
120
130
140
150
dG = -39.1

Pre-miRNA 45

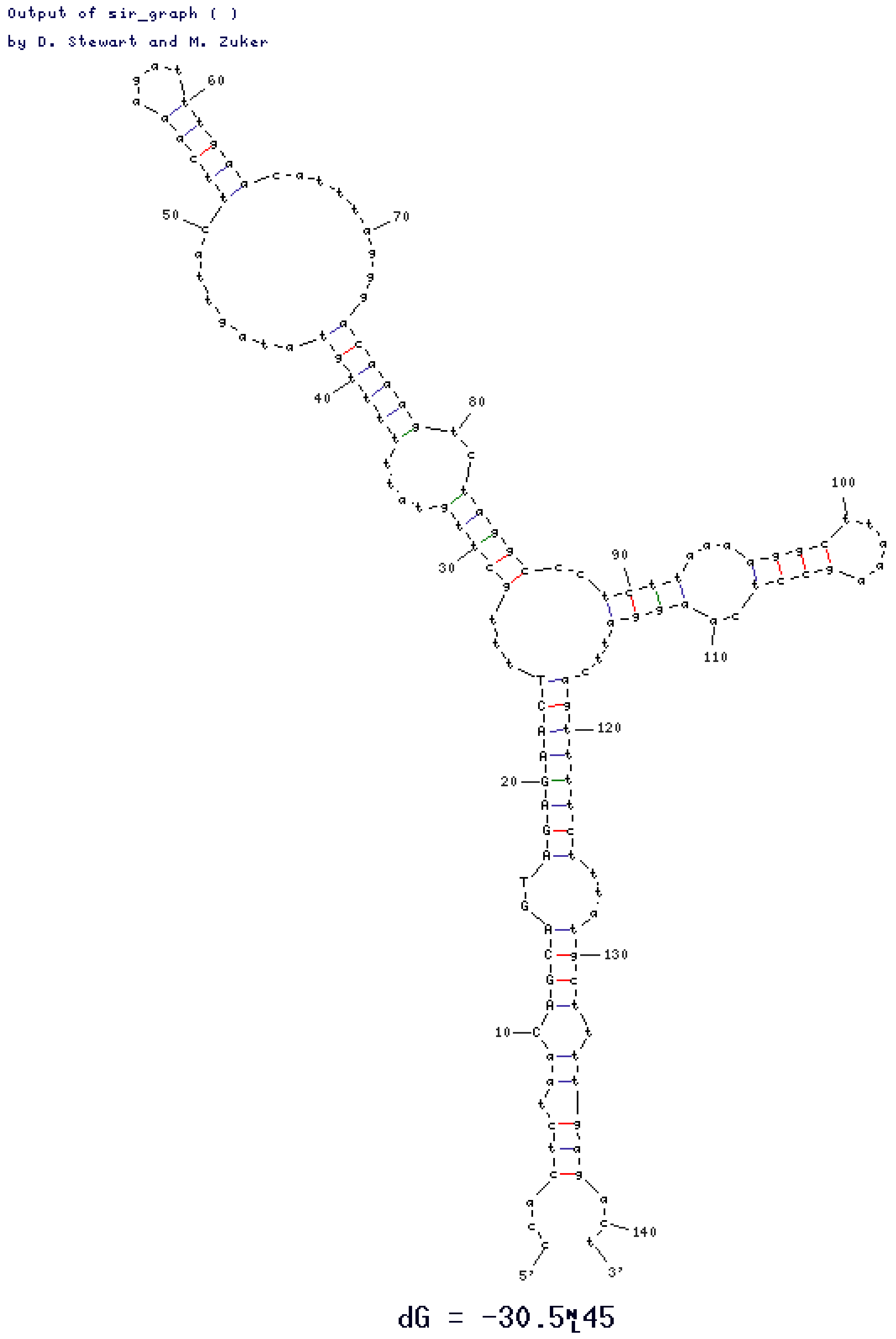

Pre-miRNA 46

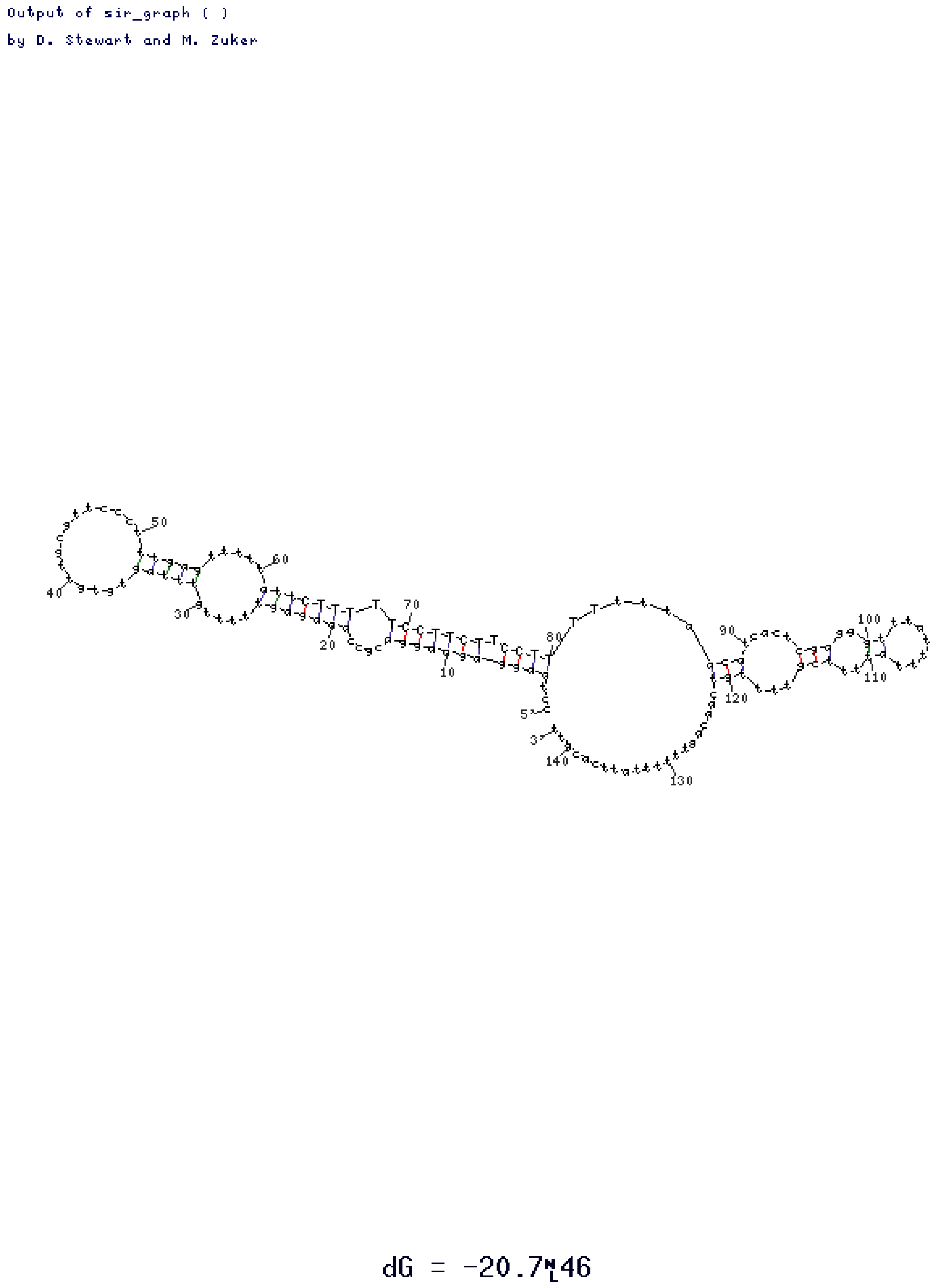

Pre-miRNA 47

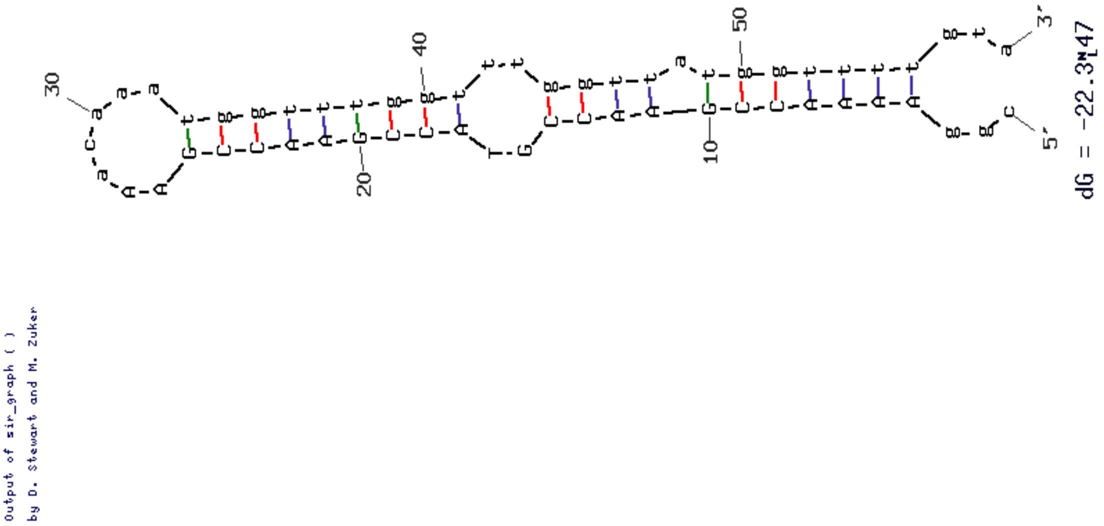

Pre-miRNA 48

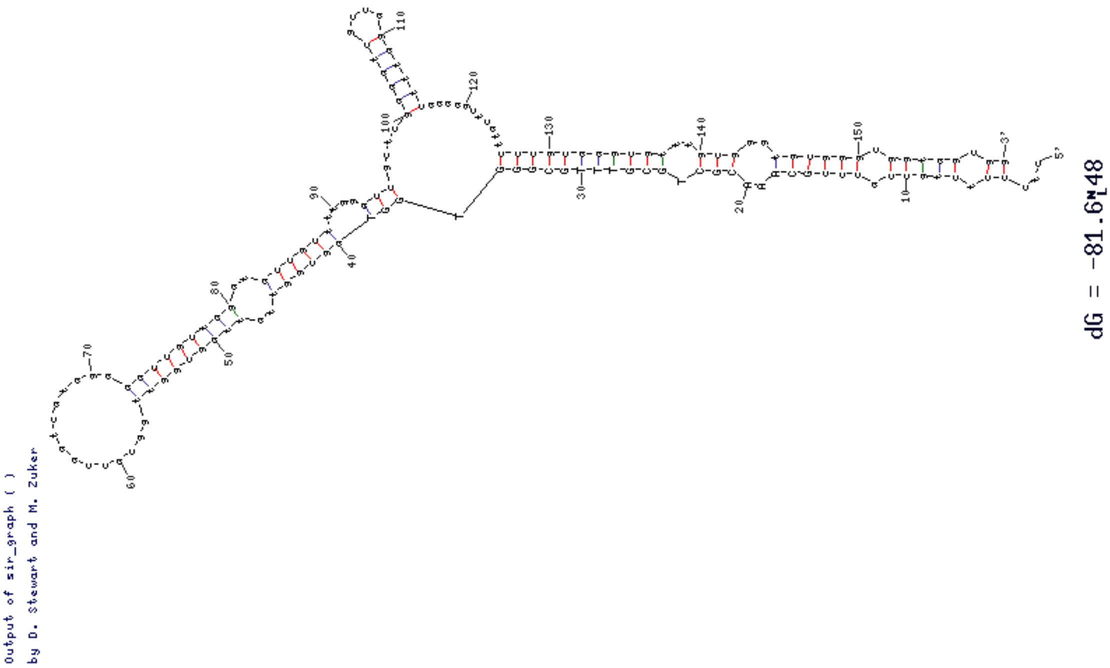

Pre-miRNA 49

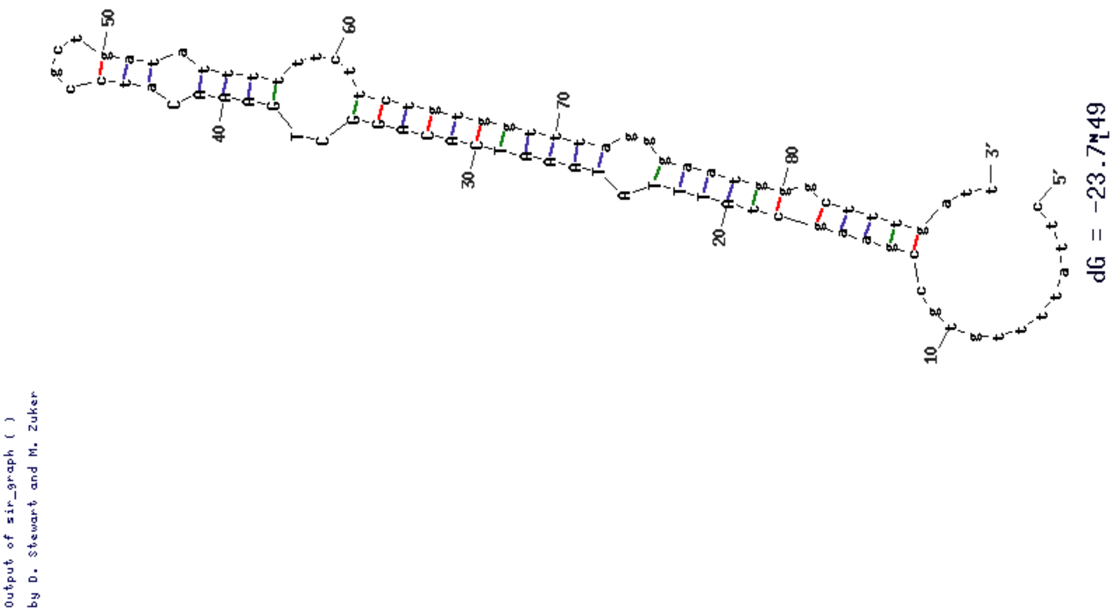

Pre-miRNA 50

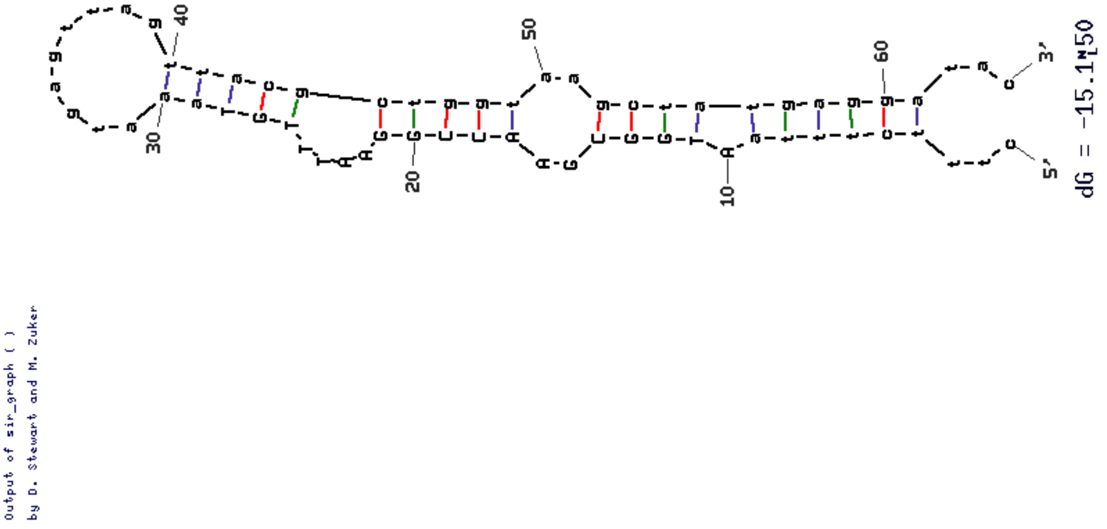

Pre-miRNA 51

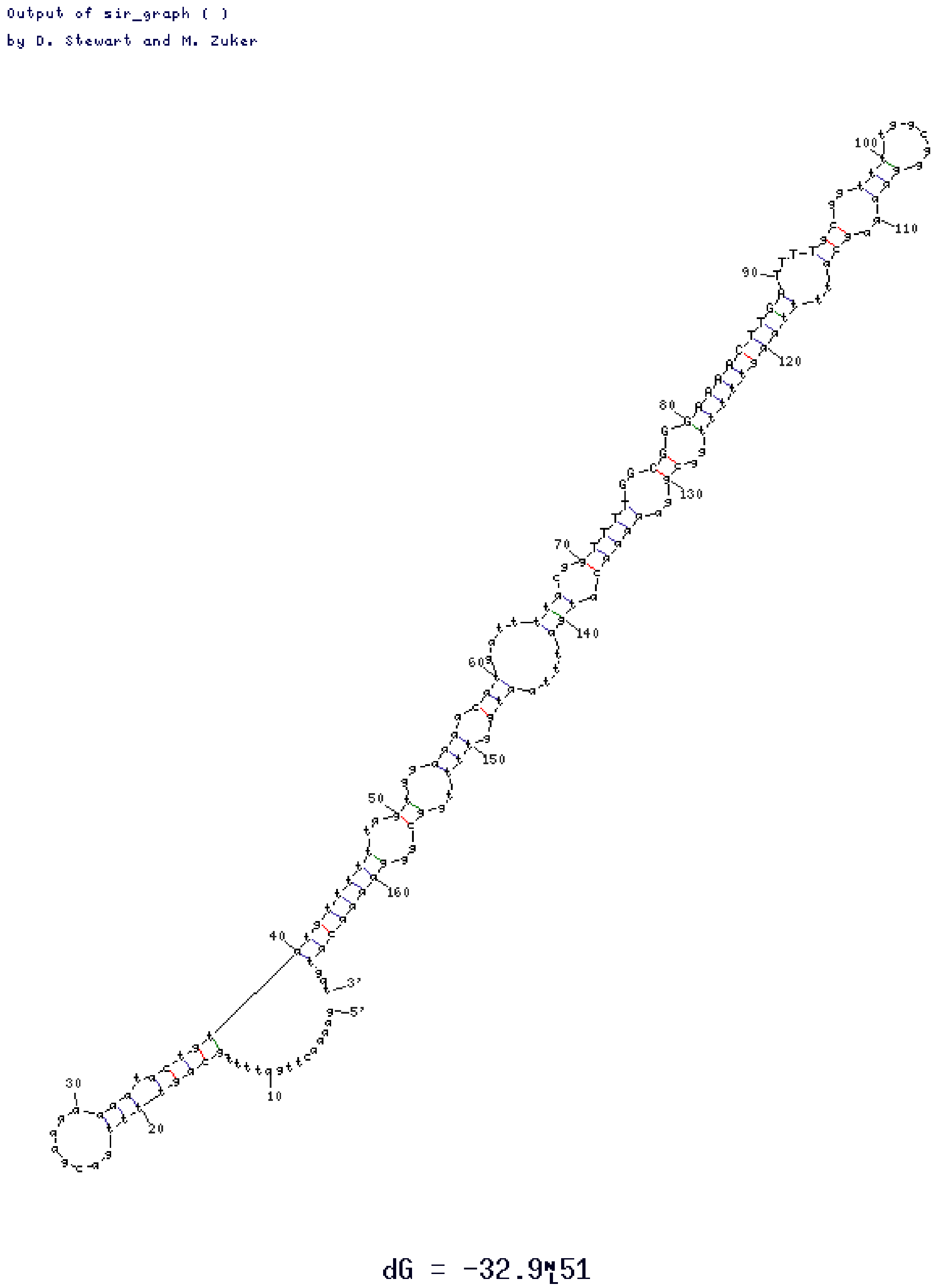

Pre-miRNA 52

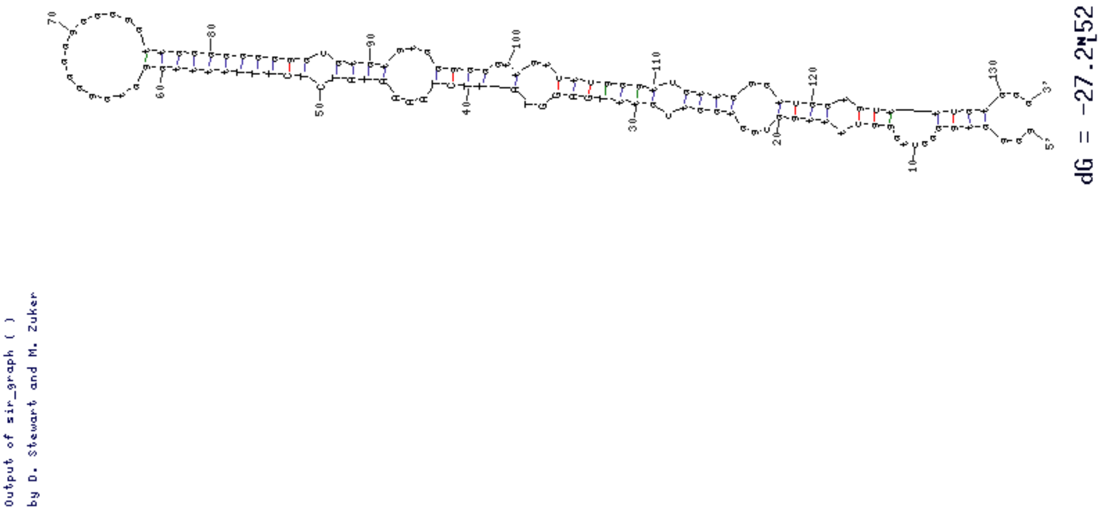

Pre-miRNA 53

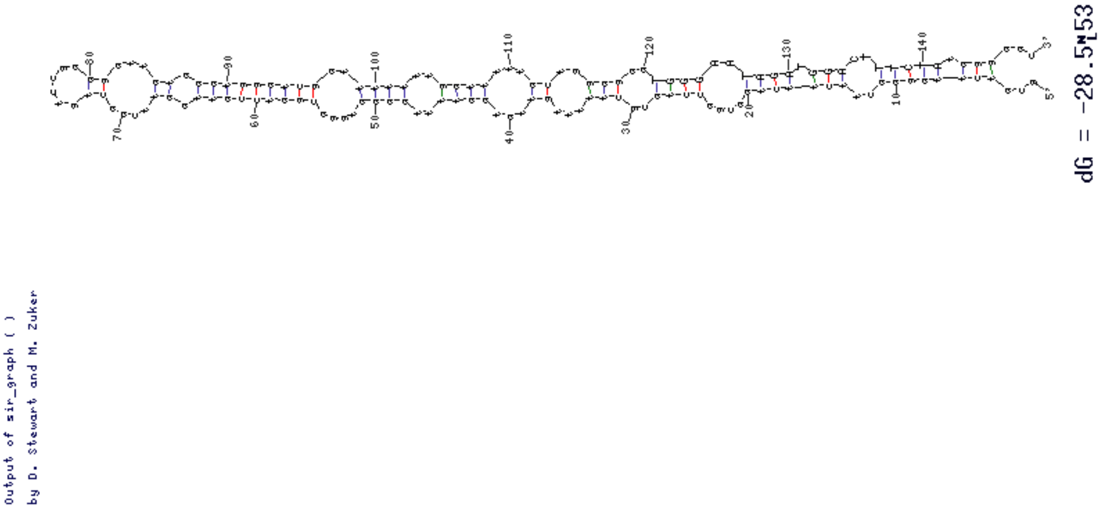

Pre-miRNA 54

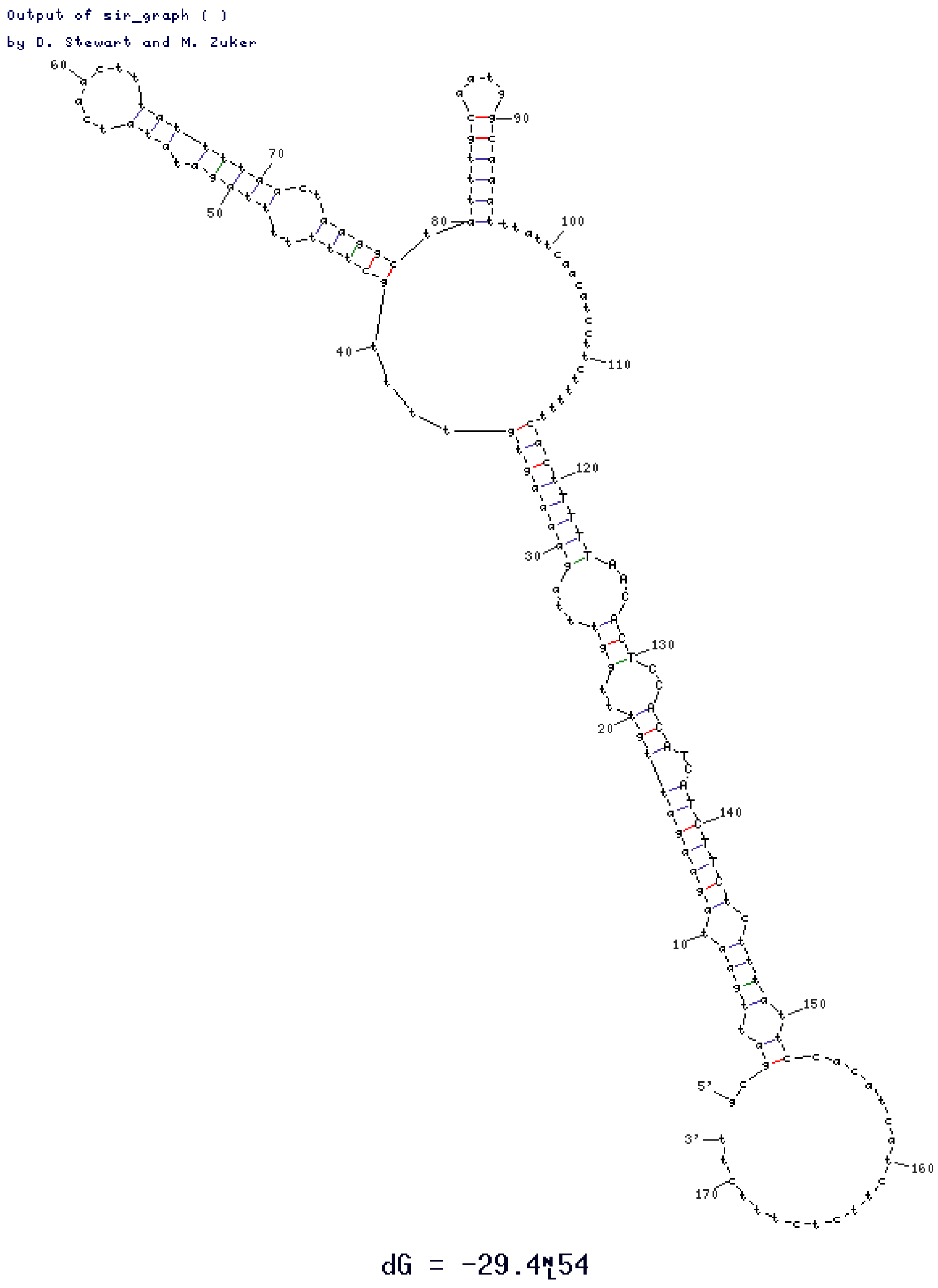

Pre-miRNA 55

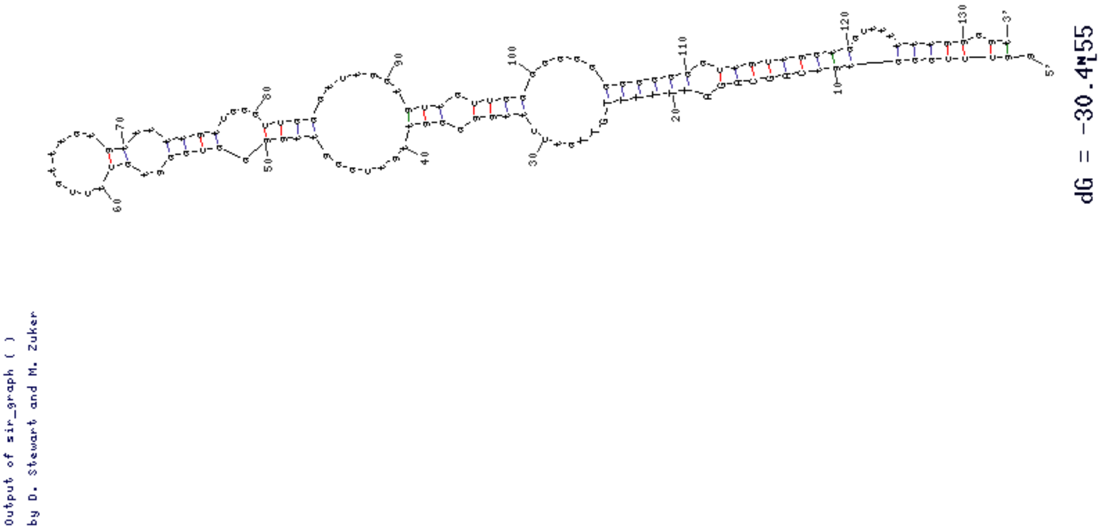

Pre-miRNA 56

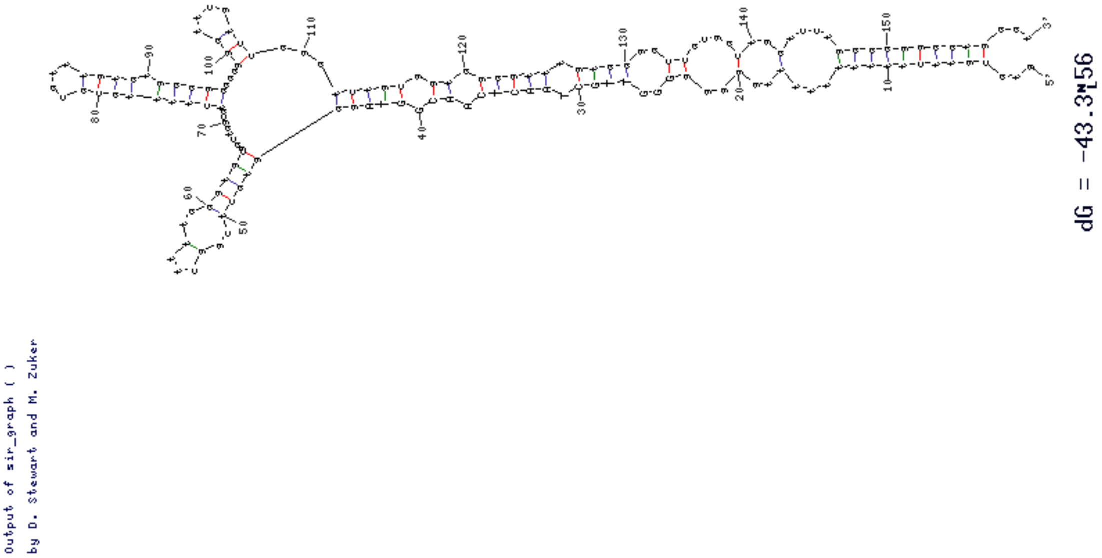

Pre-miRNA 57

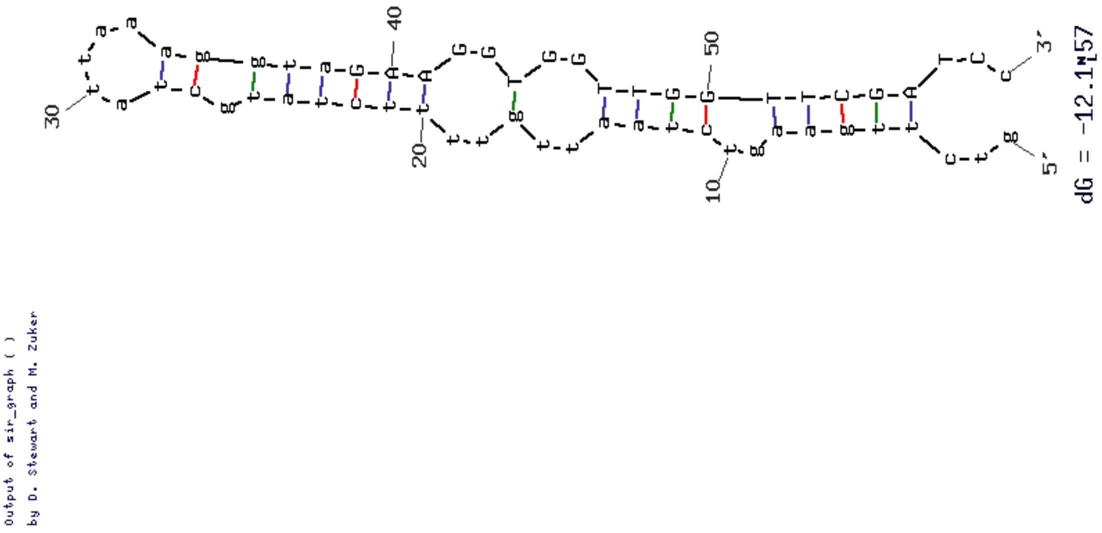

Pre-miRNA 58

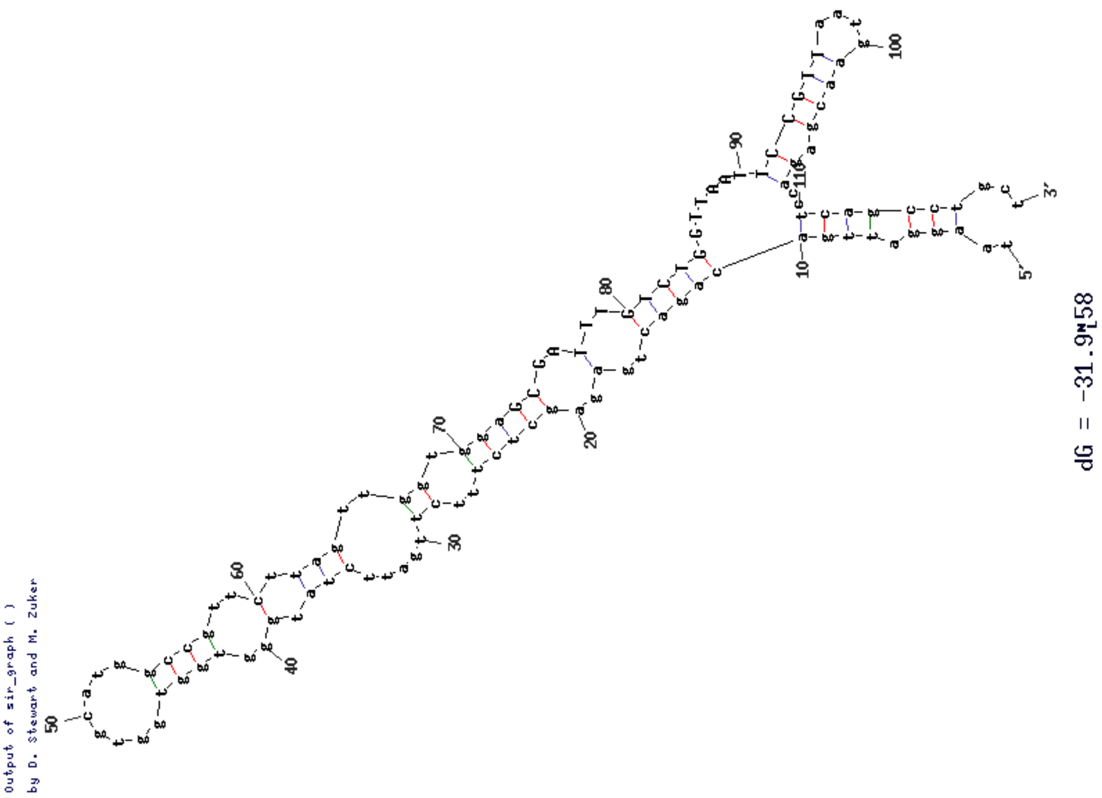

Pre-miRNA 59

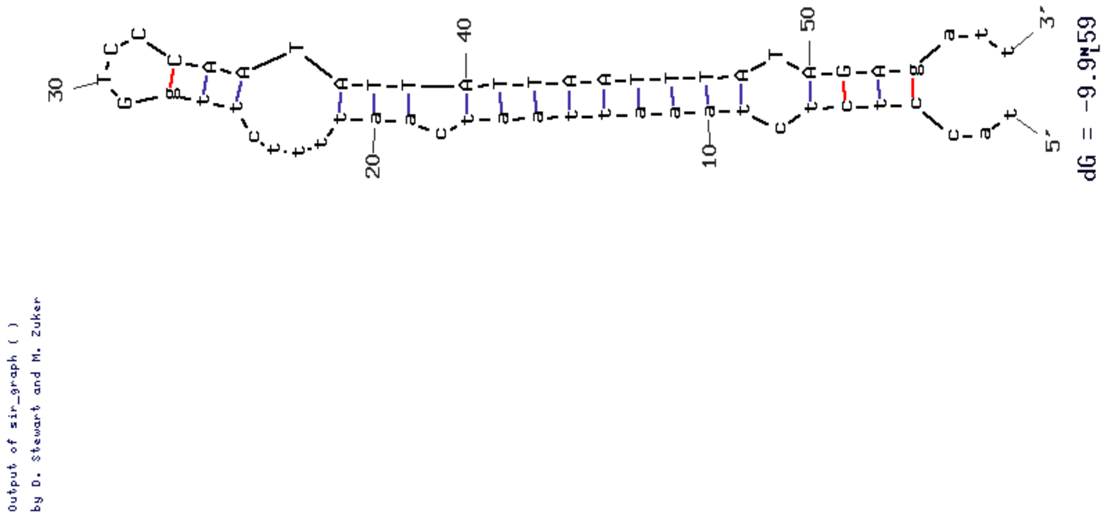

Pre-miRNA 60

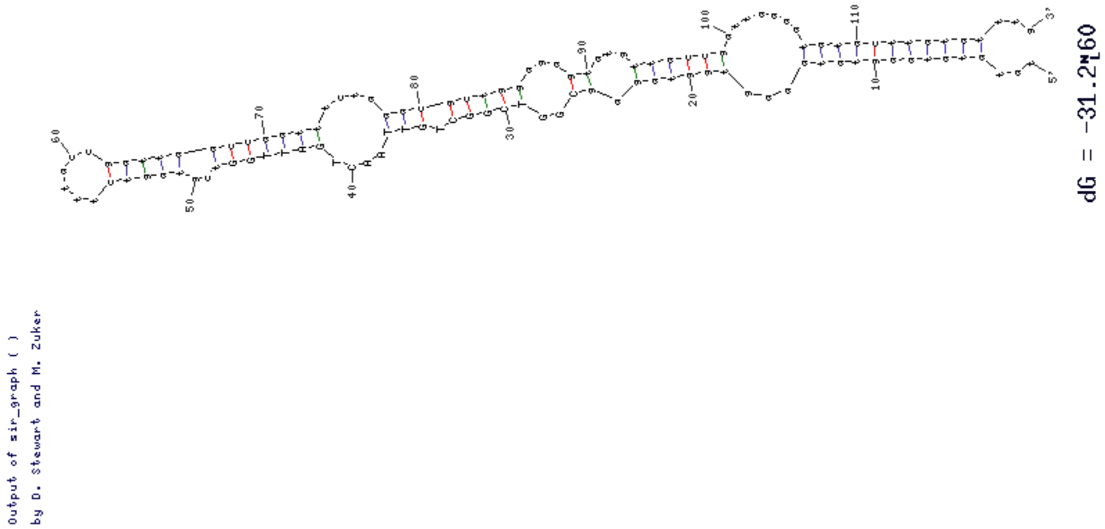

Pre-miRNA 61

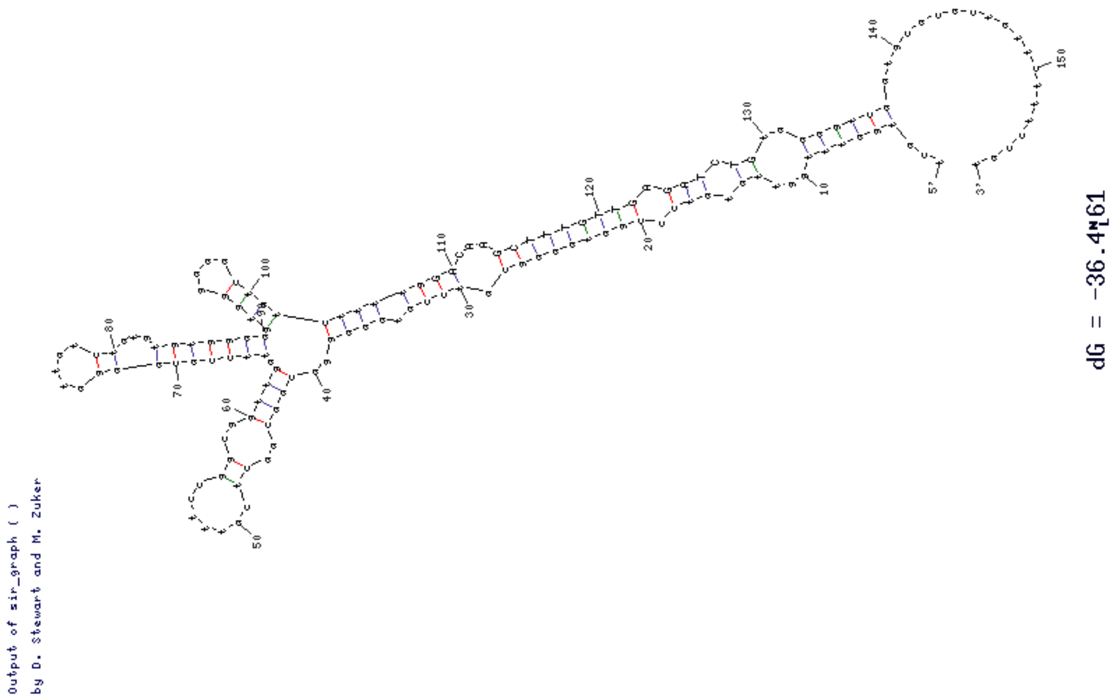

Pre-miRNA 62

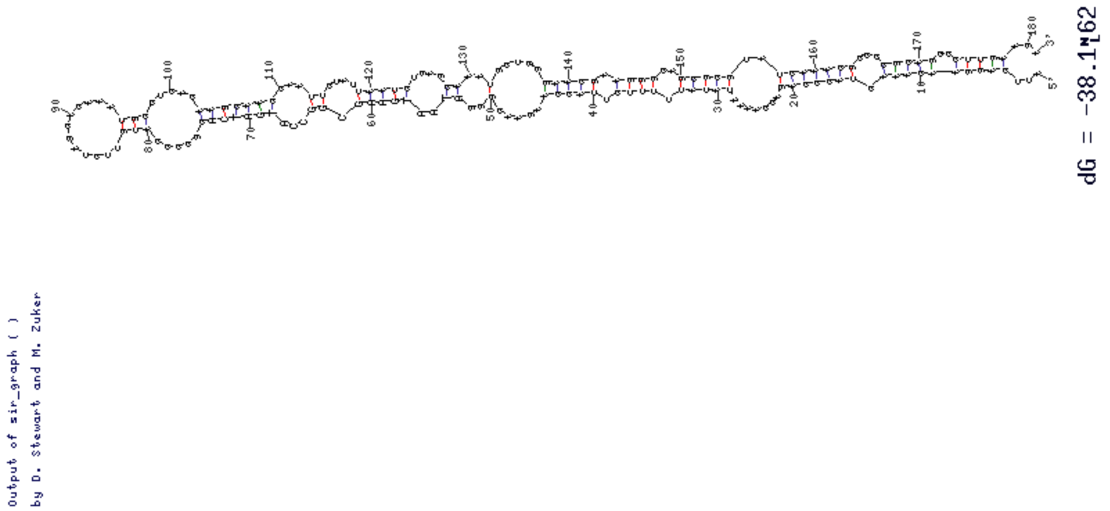

Pre-miRNA 63

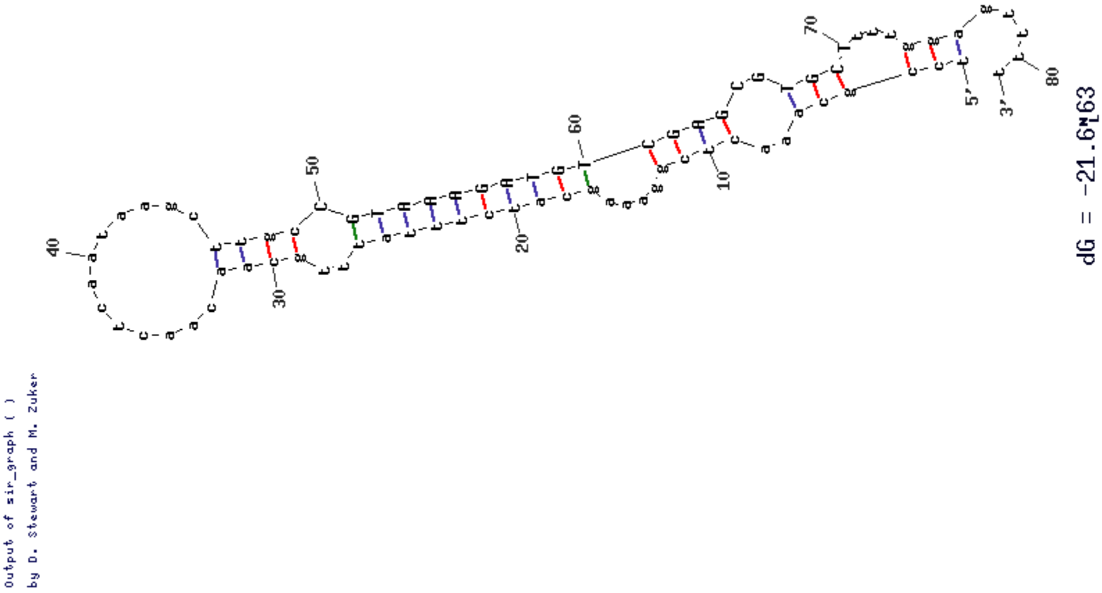

Pre-miRNA 64

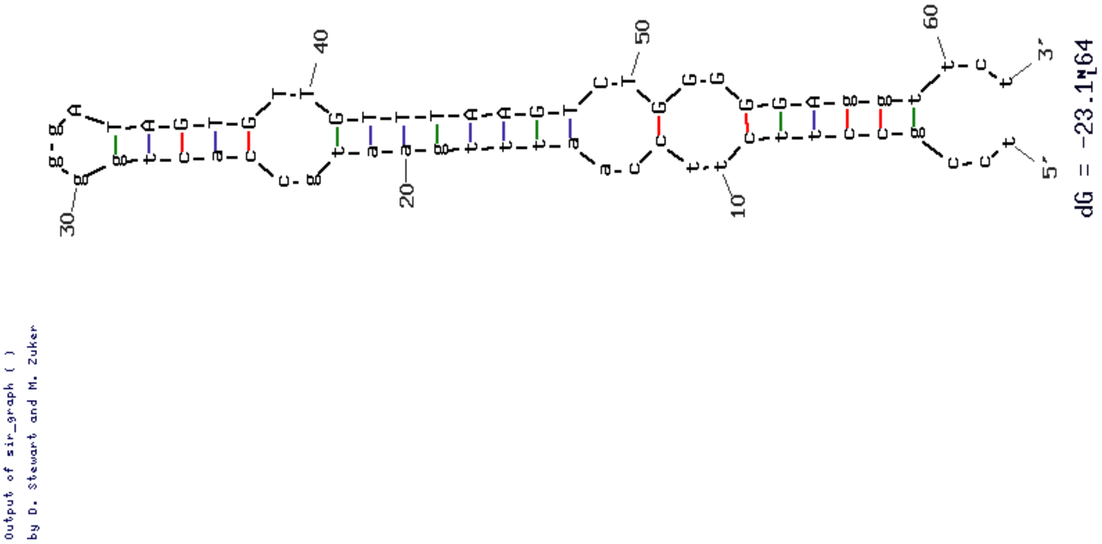

Pre-miRNA 65

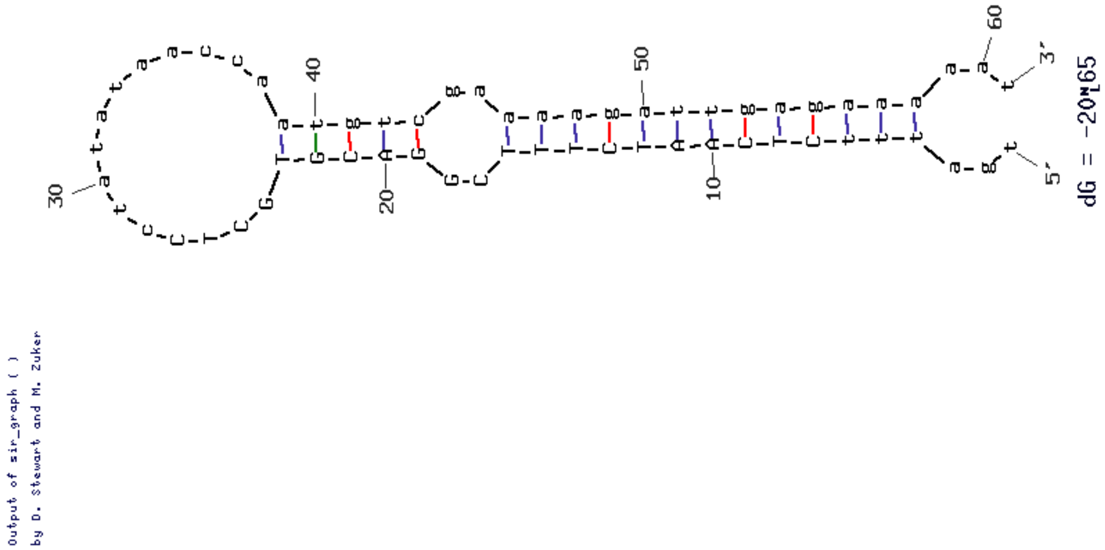

Pre-miRNA 66

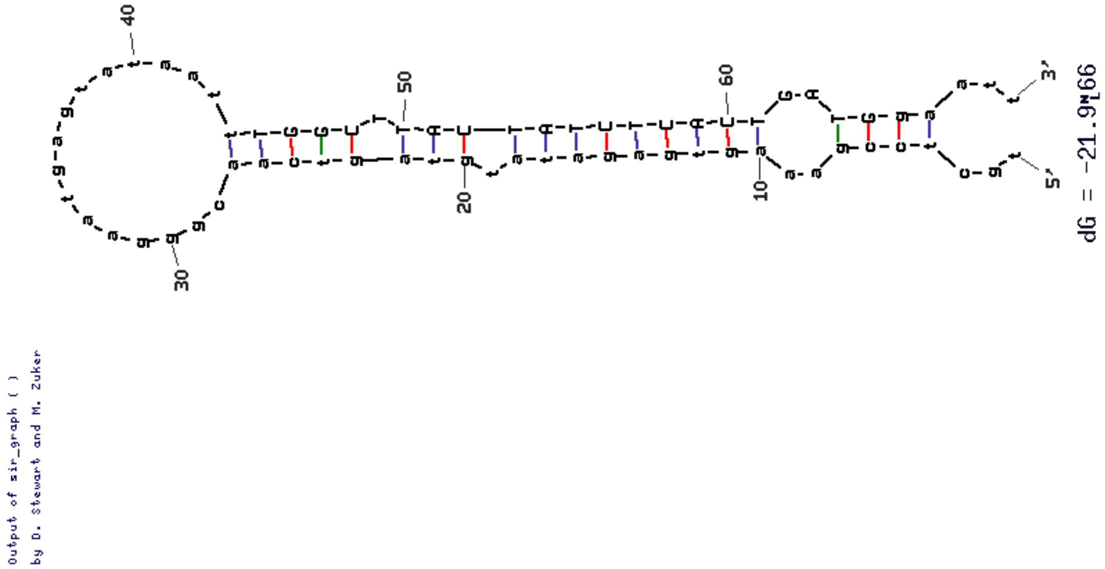

Pre-miRNA 67

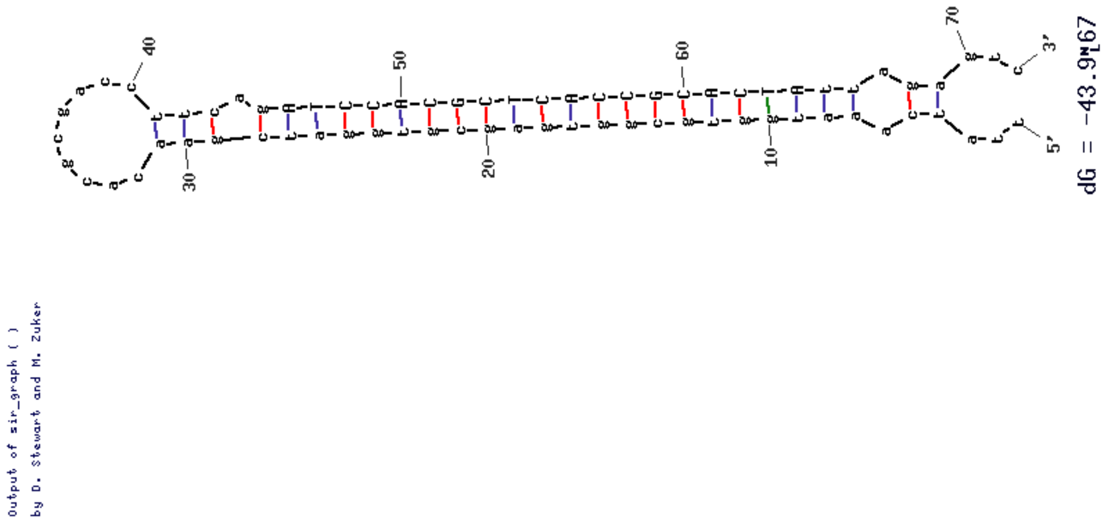

Pre-miRNA 68

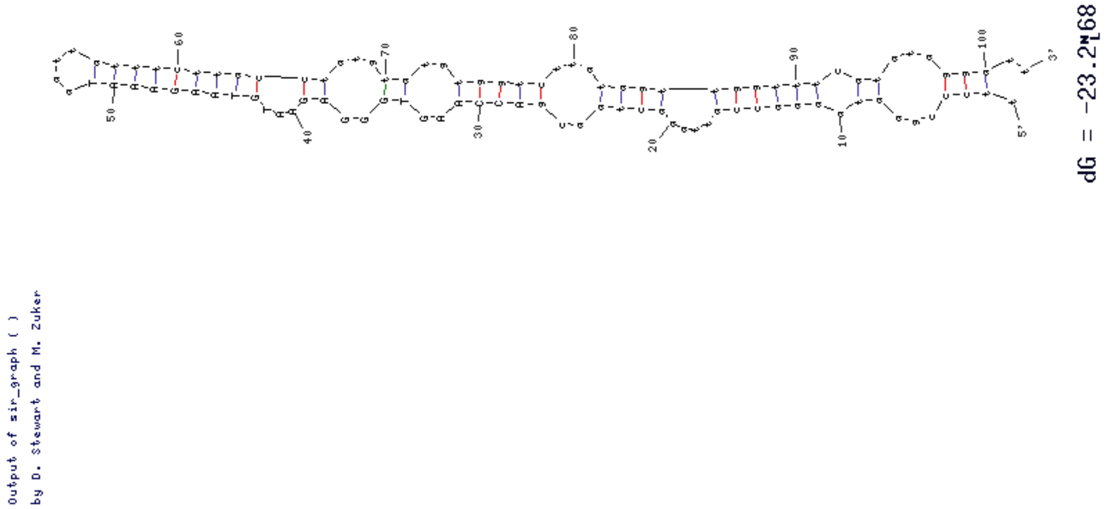

Pre-miRNA 69

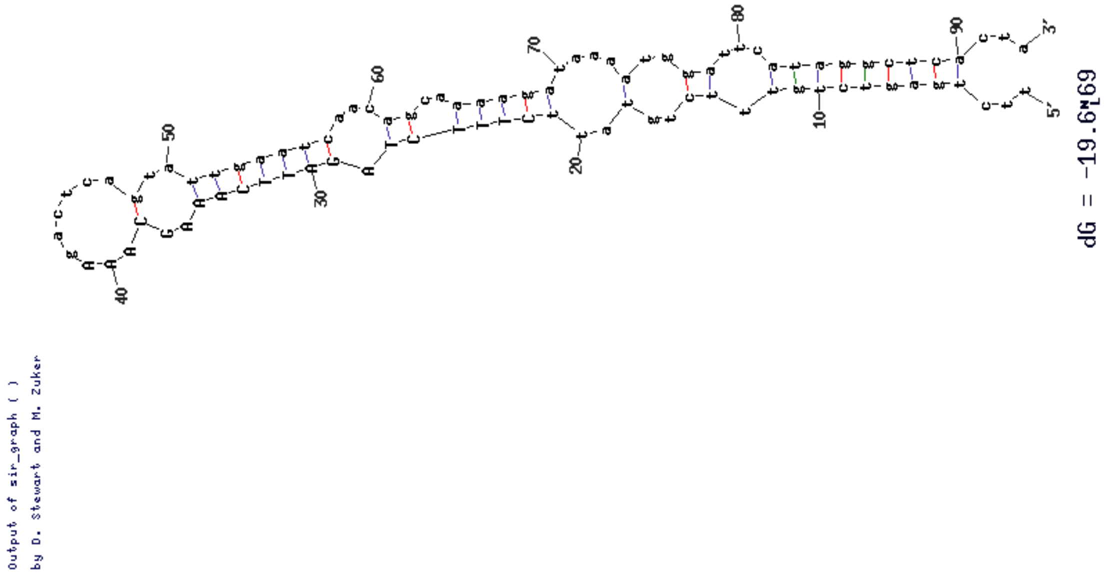

Pre-miRNA 70

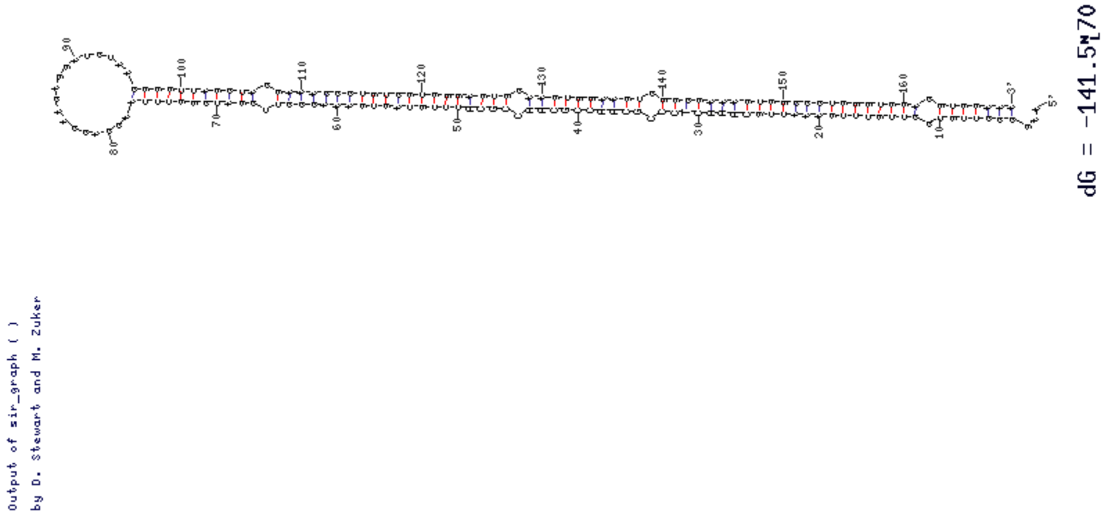

Pre-miRNA 71/ Pre-miRNA 72

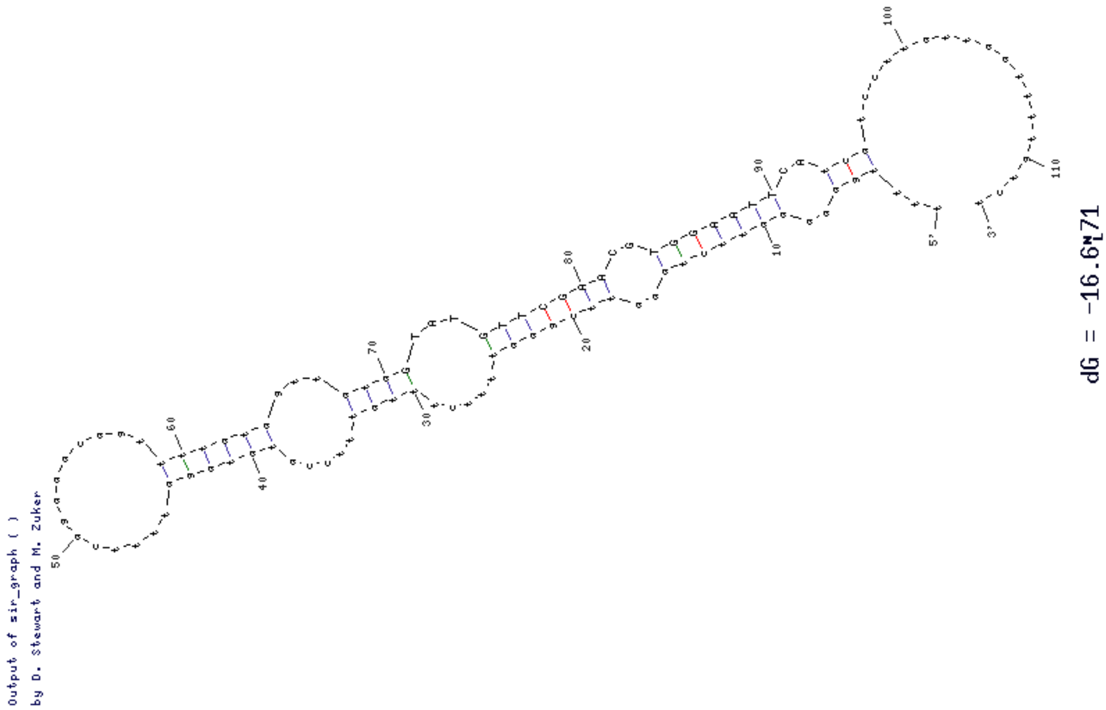

Pre-miRNA 73/ Pre-miRNA 74

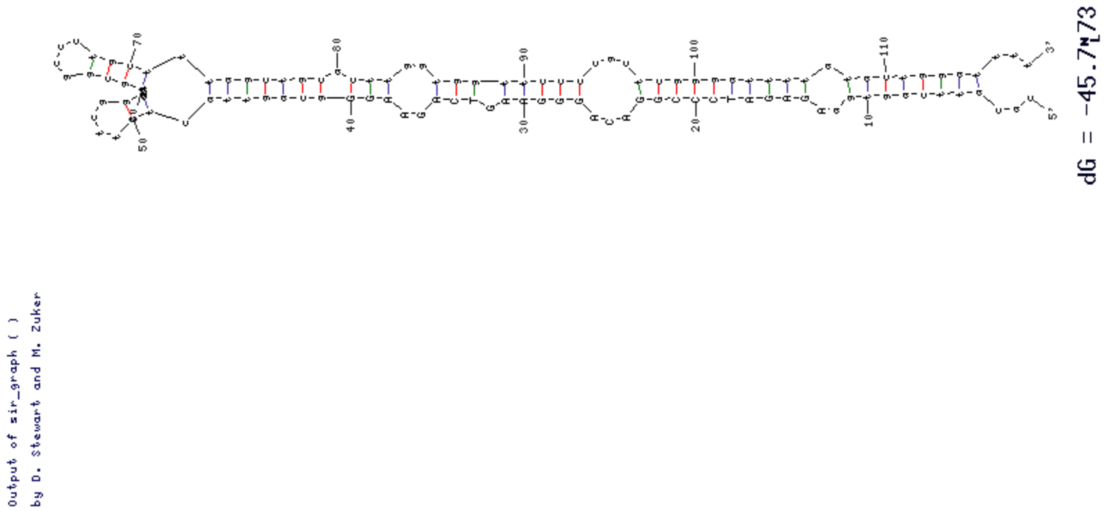

Pre-miRNA 75

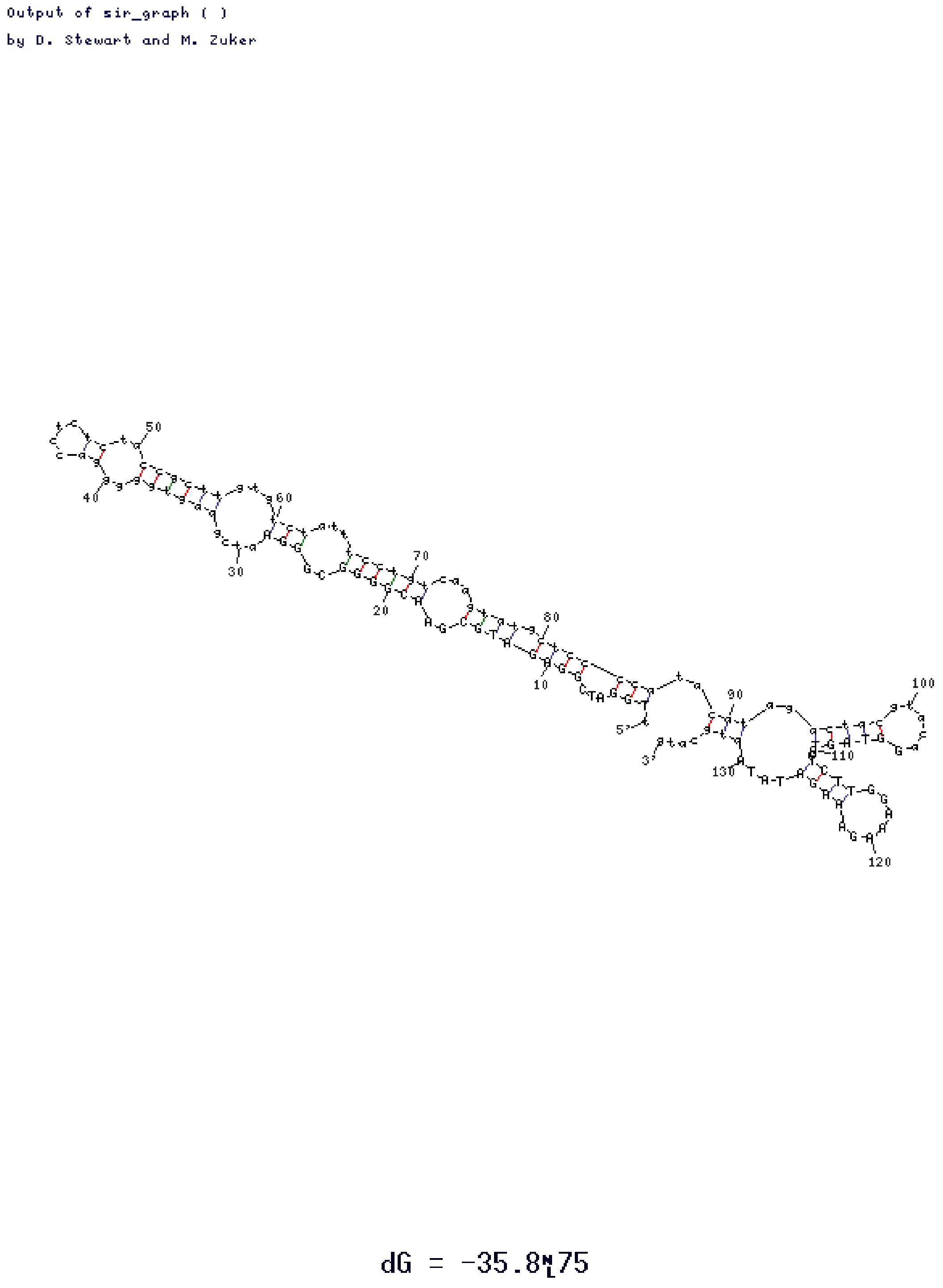

Pre-miRNA 76

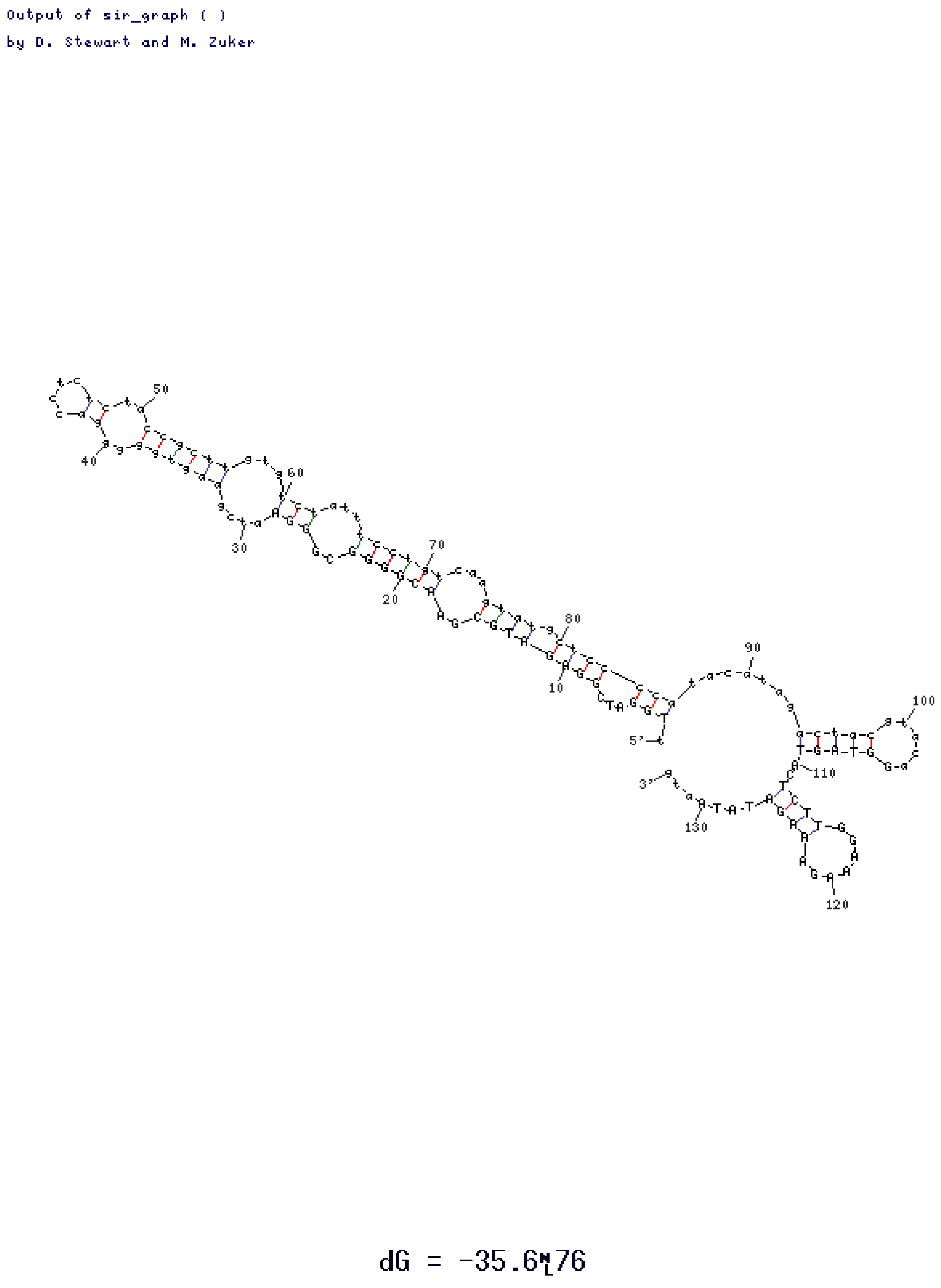

Output of sir_graph ( )
by D. Stewart and M. Zuker
5'
3'
10
20
30
40
50
60
70
80
90
100
110
120
130
dG = -35.6
76

Pre-miRNA 77

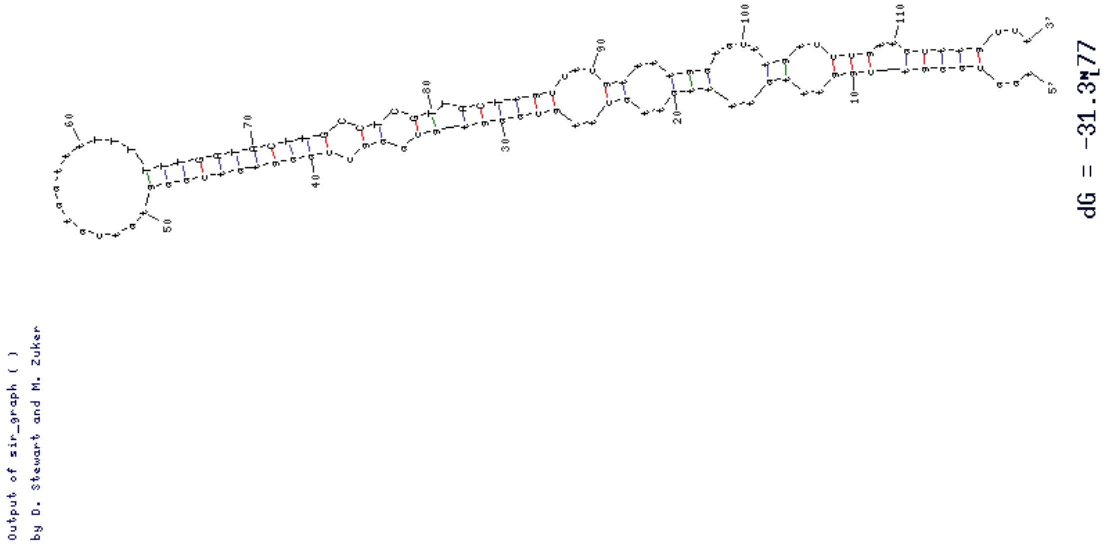

Pre-miRNA 78

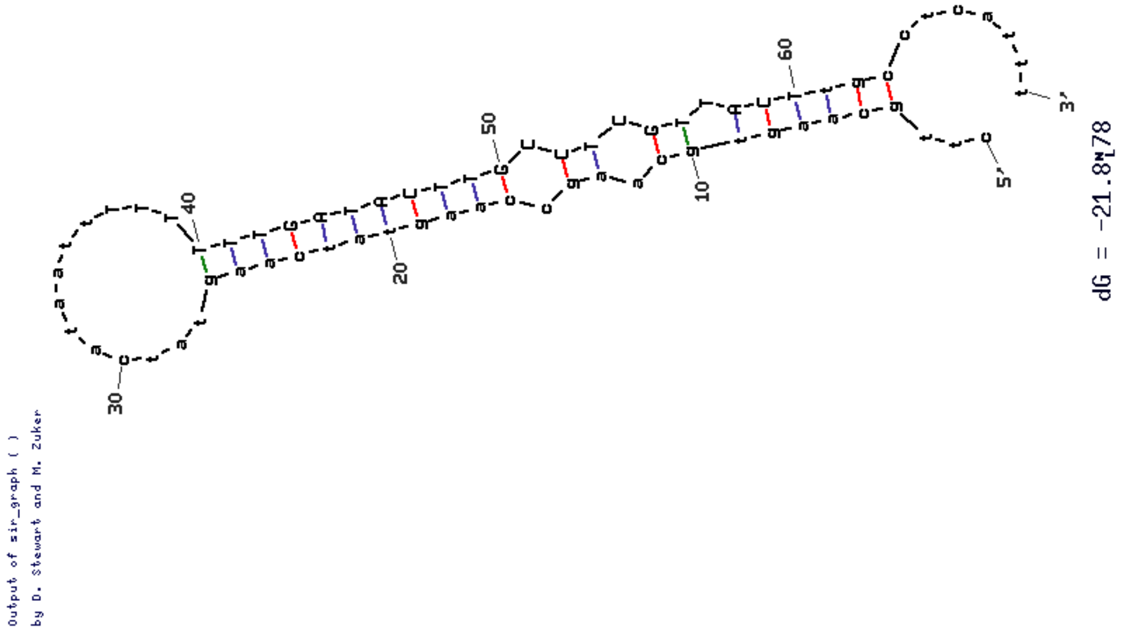

Supplement: Additional file 2 — Figure S1. Prediction of secondary structure of all new candidate miRNAs during floral development of Brassica juncea. [file 1471-2164-14-9-S2.pdf]
