# Supplementary material for: Identification of miRNAs and their targets using high-throughput sequencing and degradome analysis in cytoplasmic male-sterile and its maintainer fertile lines of brassica juncea
Source: BMC Genomics. 2013 Jan 16;14:9. doi: 10.1186/1471-2164-14-9 (PMC3553062; doi:10.1186/1471-2164-14-9)
Supplement: Additional file 3 — Figure S2. Target plot (t-plot) of representative validated miRNAs target in MF of Brassica juncea. [file 1471-2164-14-9-S3.pdf]

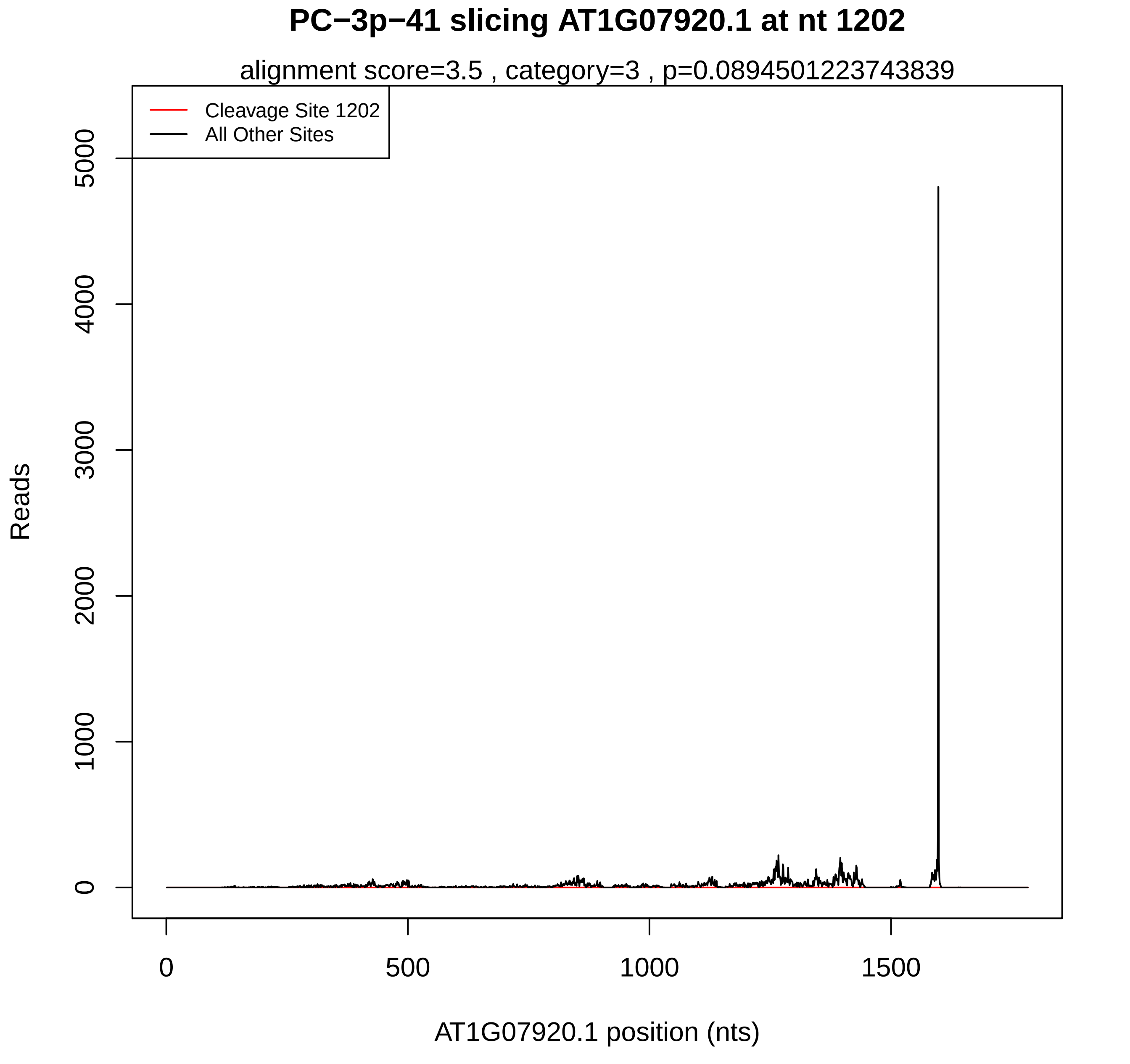
PC−3p−41 slicing AT1G07920.1 at nt 1202
alignment score=3.5 , category=3 , p=0.0894501223743839
Cleavage Site 1202
All Other Sites
Reads
0
1000
2000
3000
4000
5000
0
500
1000
1500
AT1G07920.1 position (nts)

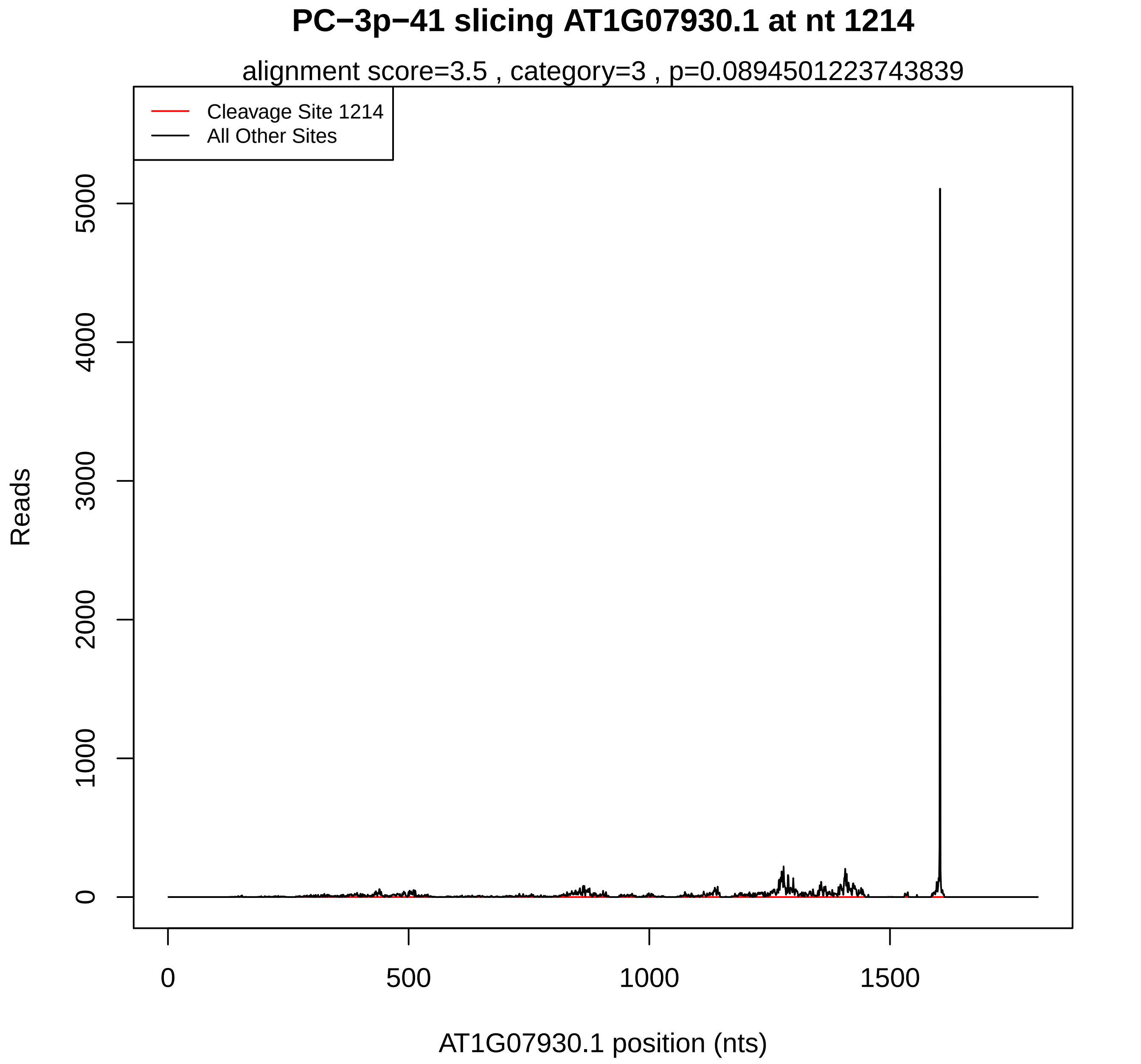

PC−3p−41 slicing AT1G07930.1 at nt 1214
alignment score=3.5 , category=3 , p=0.0894501223743839
Cleavage Site 1214
All Other Sites
Reads
0
1000
2000
3000
4000
5000
0
500
1000
1500
AT1G07930.1 position (nts)

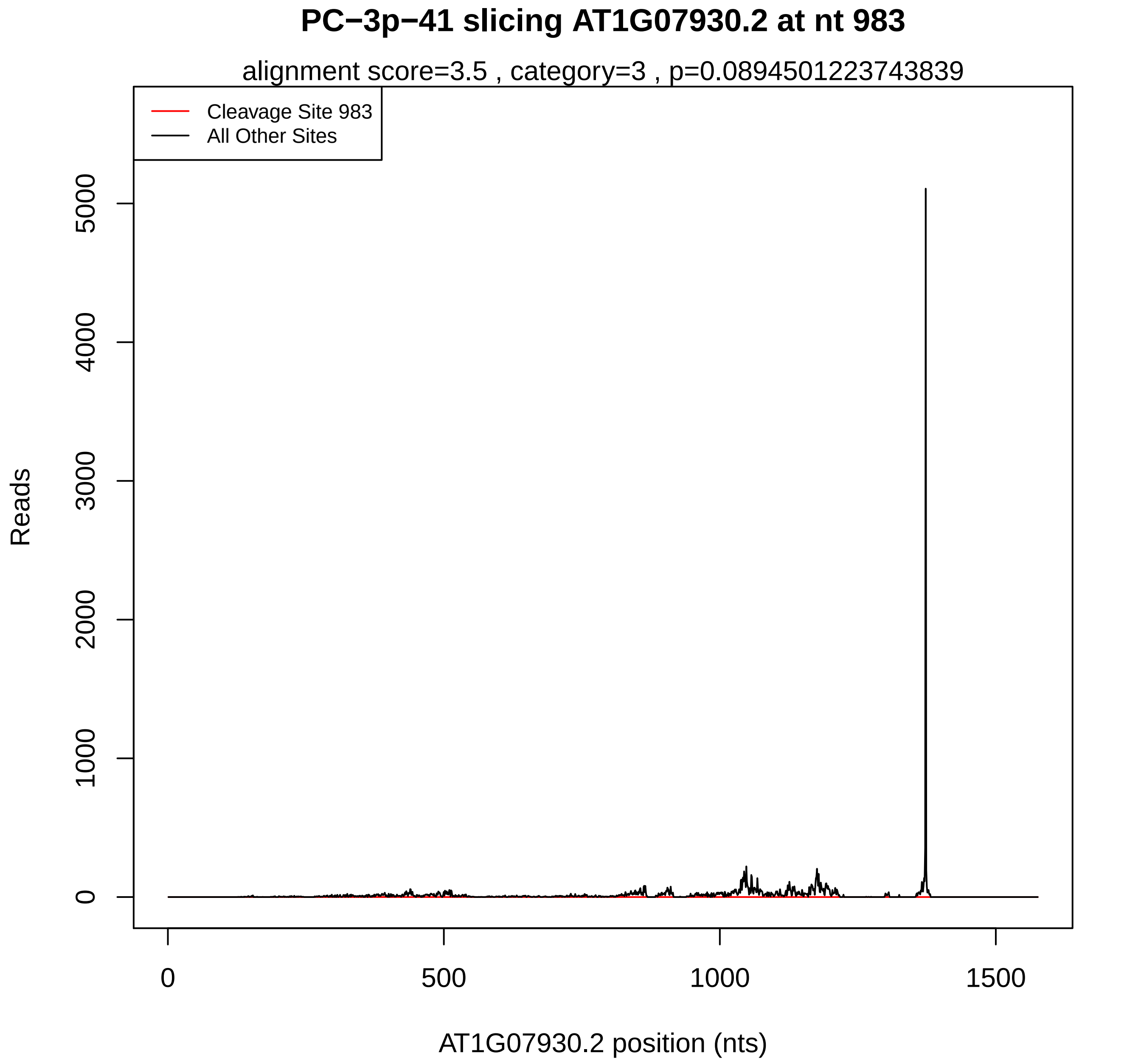
PC−3p−41 slicing AT1G07930.2 at nt 983
alignment score=3.5 , category=3 , p=0.0894501223743839
Cleavage Site 983
All Other Sites
Reads
0
1000
2000
3000
4000
5000
0
500
1000
1500
AT1G07930.2 position (nts)

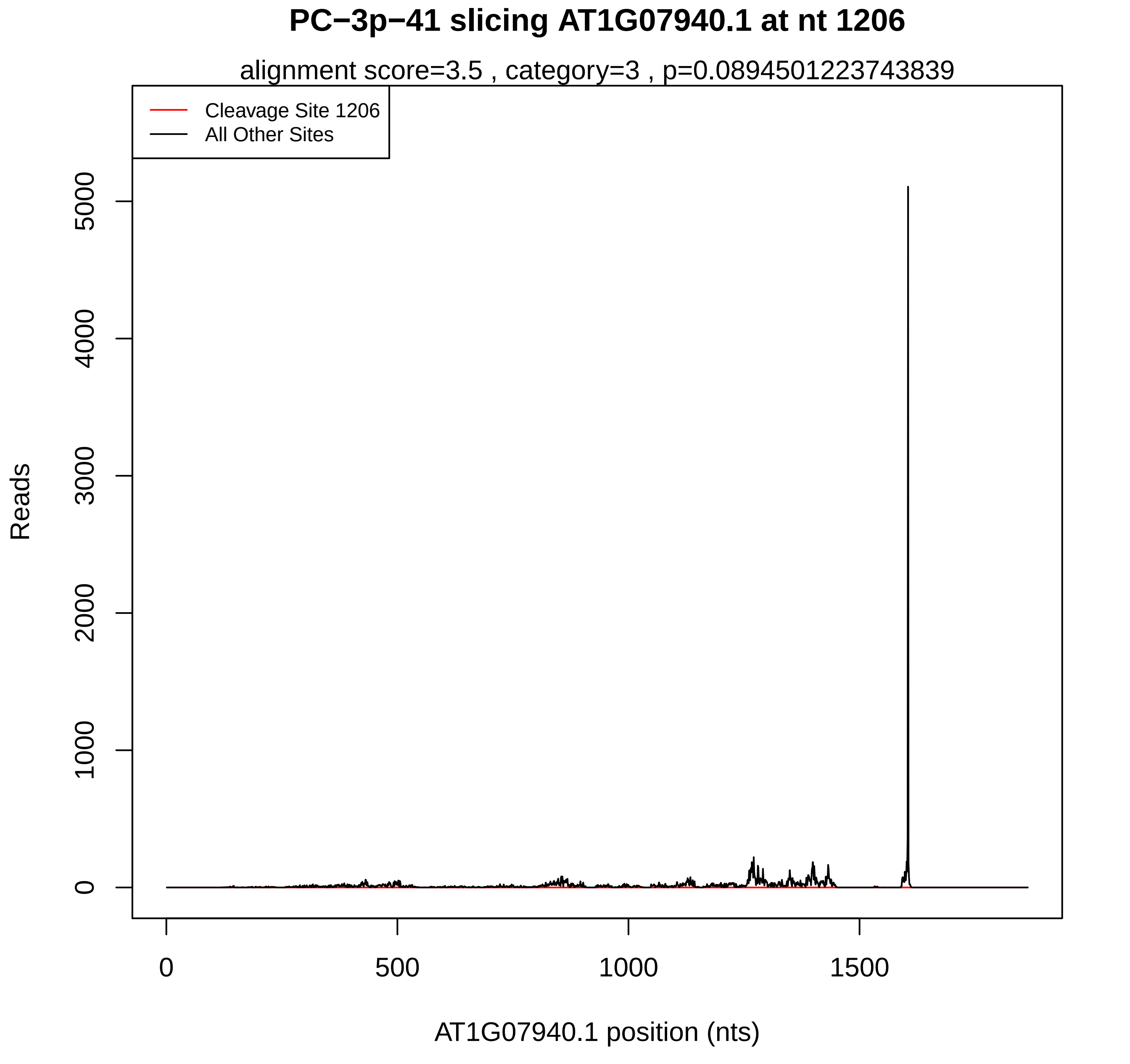
PC−3p−41 slicing AT1G07940.1 at nt 1206
alignment score=3.5 , category=3 , p=0.0894501223743839
Cleavage Site 1206
All Other Sites
Reads
0
1000
2000
3000
4000
5000
0
500
1000
1500
AT1G07940.1 position (nts)

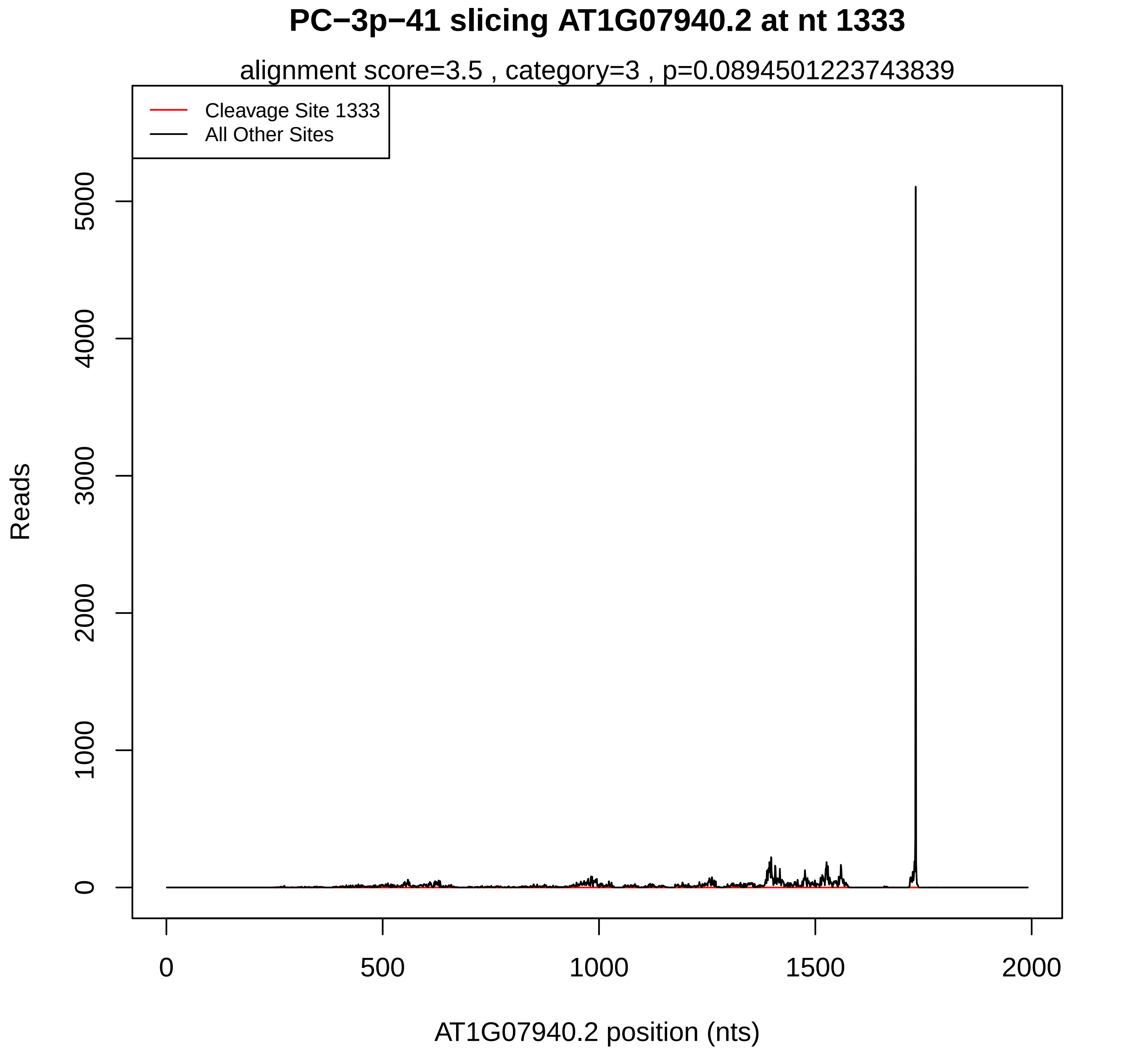
PC−3p−41 slicing AT1G07940.2 at nt 1333
alignment score=3.5 , category=3 , p=0.0894501223743839
Cleavage Site 1333
All Other Sites
Reads
0
1000
2000
3000
4000
5000
0
500
1000
1500
2000
AT1G07940.2 position (nts)

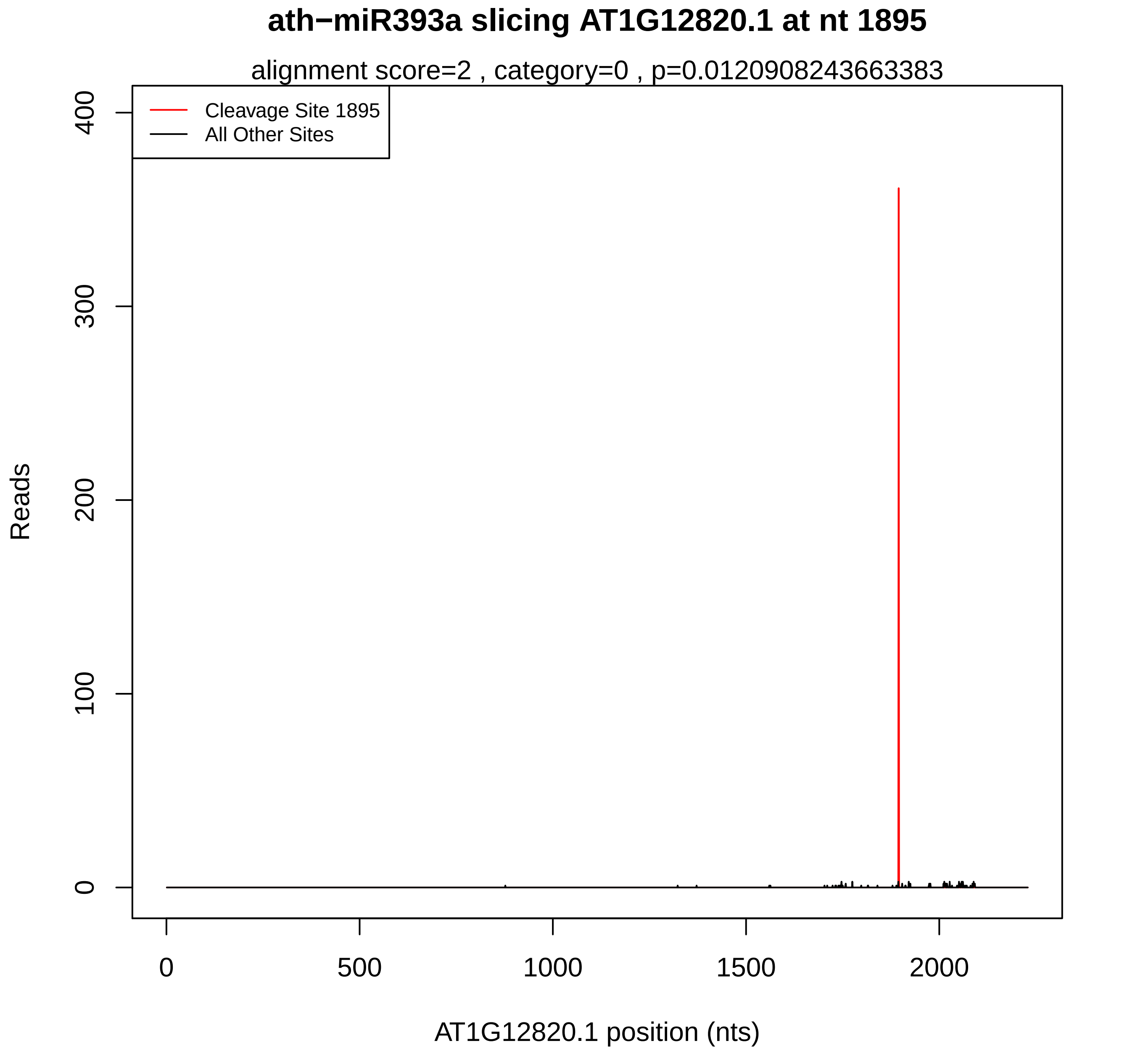

ath−miR393a slicing AT1G12820.1 at nt 1895
alignment score=2 , category=0 , p=0.0120908243663383
Cleavage Site 1895
All Other Sites
Reads
0
100
200
300
400
0
500
1000
1500
2000
AT1G12820.1 position (nts)

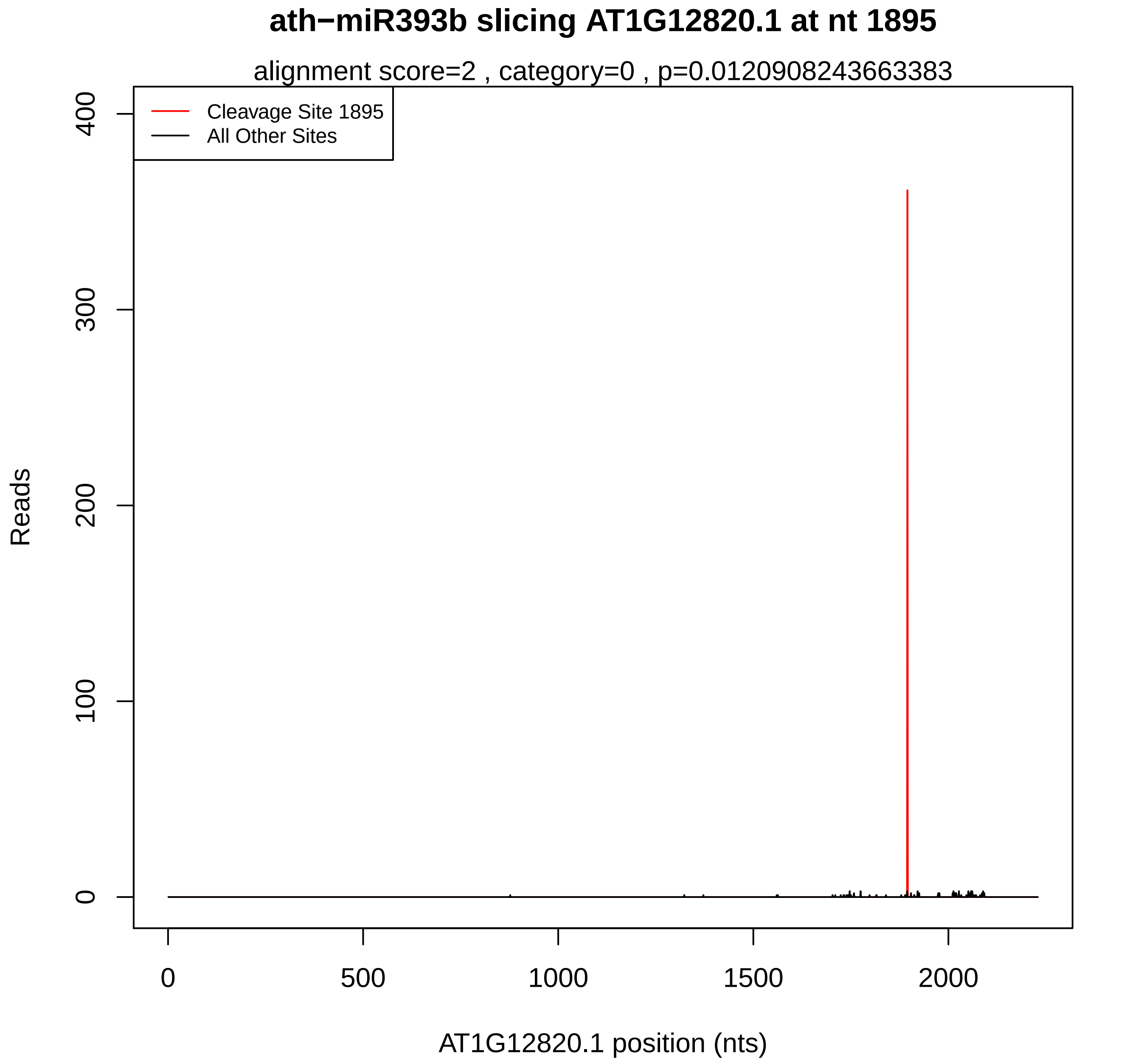
ath−miR393b slicing AT1G12820.1 at nt 1895
alignment score=2 , category=0 , p=0.0120908243663383
Cleavage Site 1895
All Other Sites
Reads
0
100
200
300
400
0
500
1000
1500
2000
AT1G12820.1 position (nts)

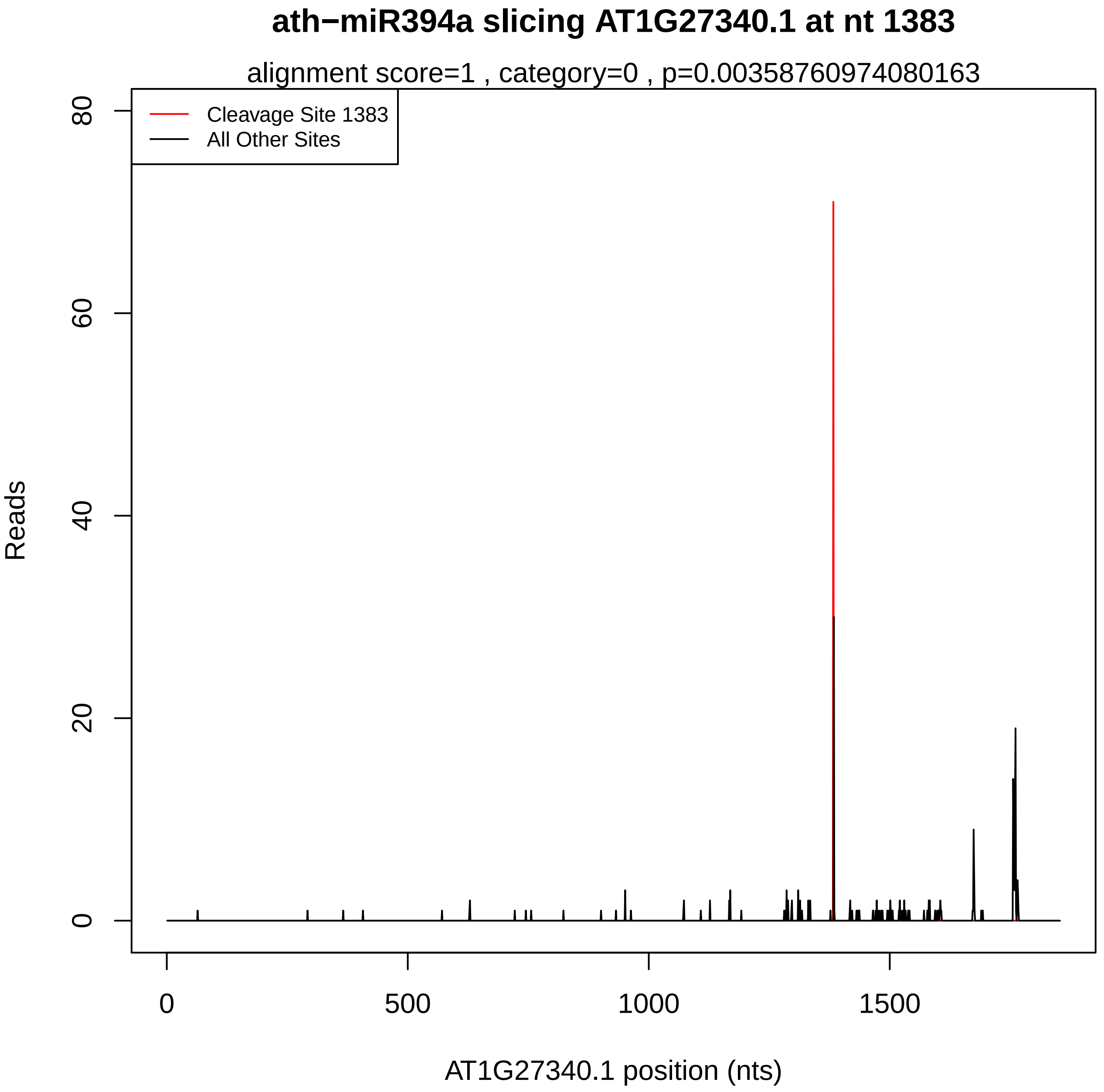
ath−miR394a slicing AT1G27340.1 at nt 1383
alignment score=1 , category=0 , p=0.00358760974080163
Cleavage Site 1383
All Other Sites
Reads
0
20
40
60
80
0
500
1000
1500
AT1G27340.1 position (nts)

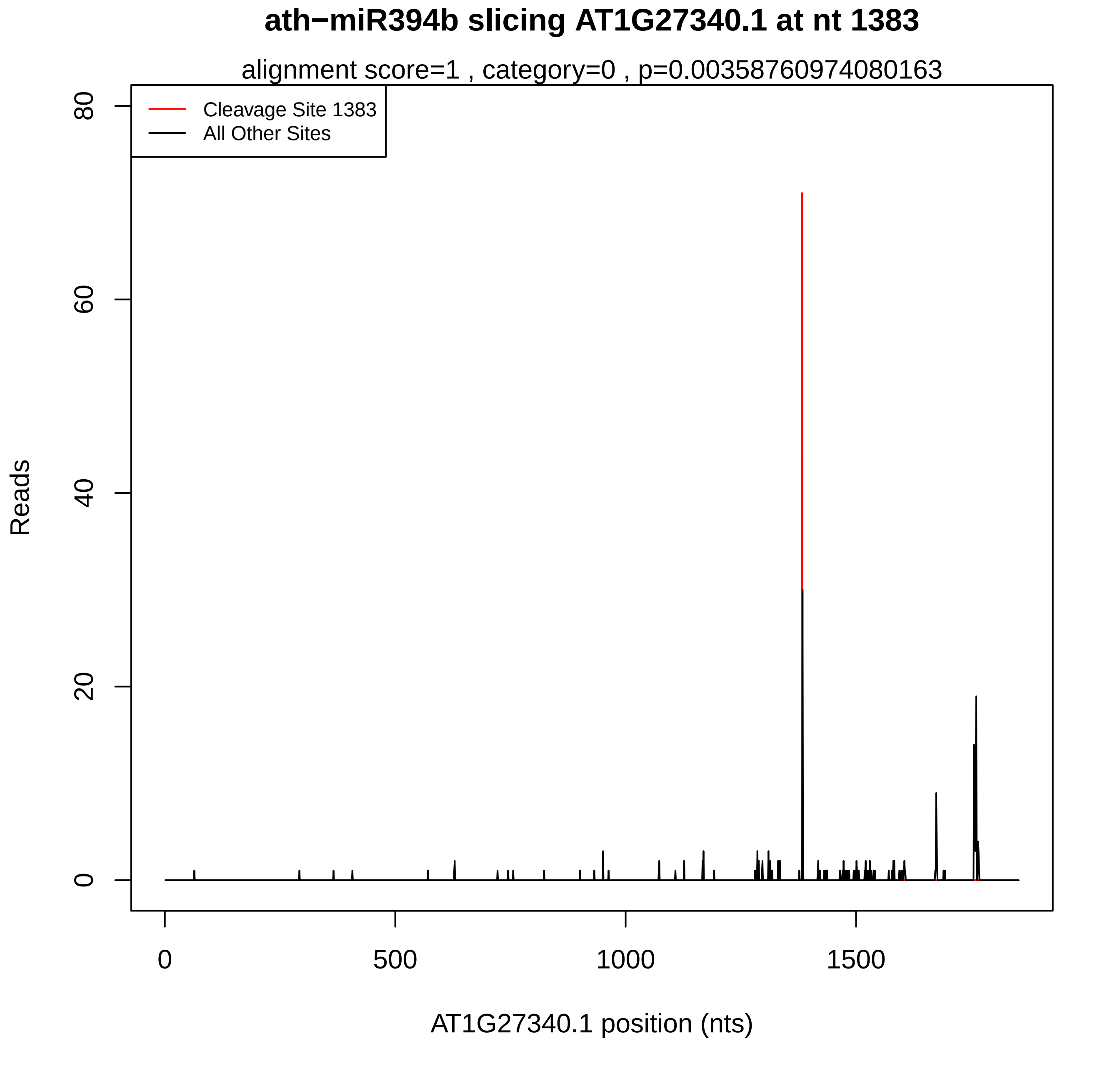

ath−miR394b slicing AT1G27340.1 at nt 1383
alignment score=1 , category=0 , p=0.00358760974080163
Cleavage Site 1383
All Other Sites
Reads
0
20
40
60
80
0
500
1000
1500
AT1G27340.1 position (nts)

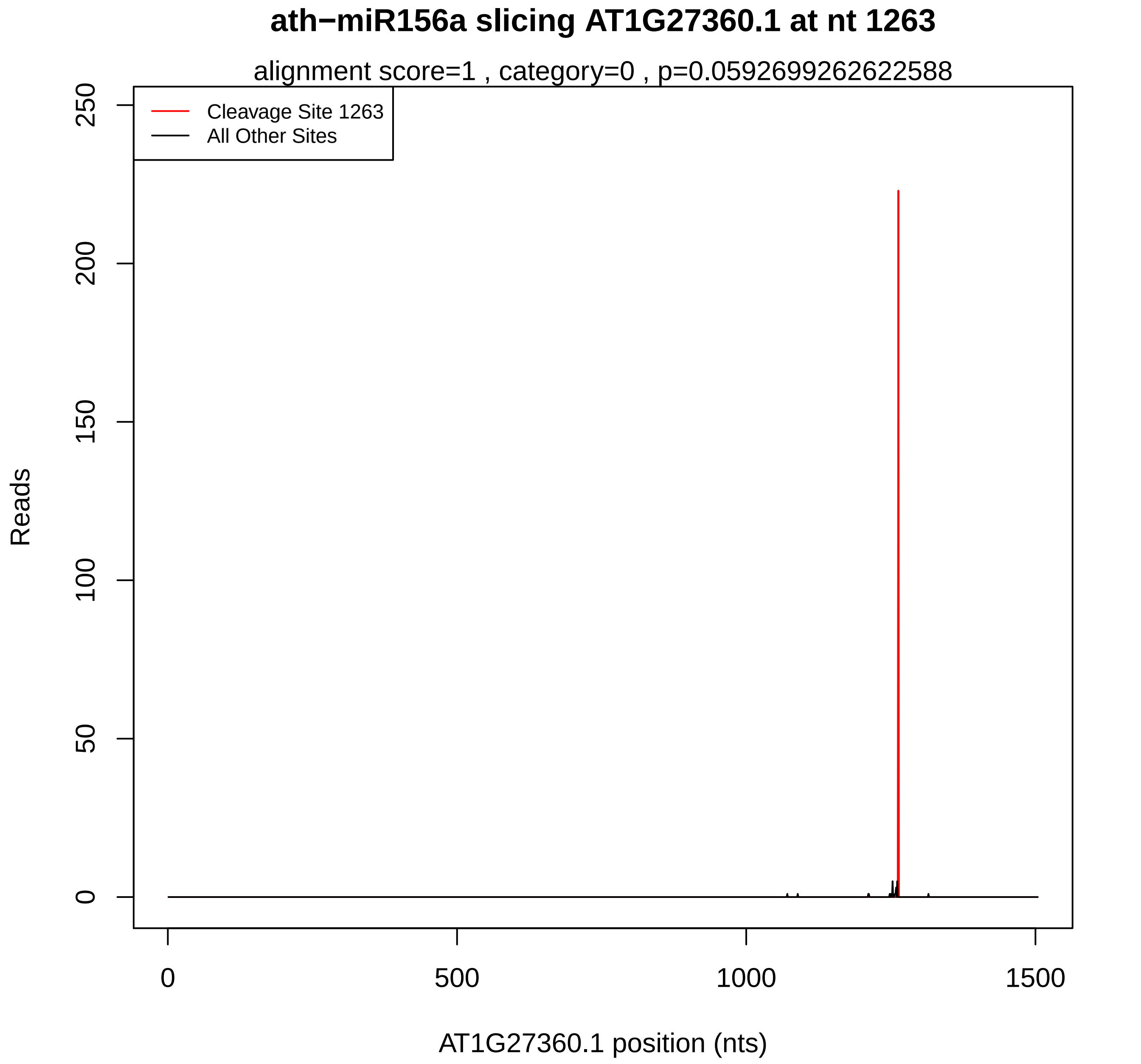

ath−miR156a slicing AT1G27360.1 at nt 1263
alignment score=1 , category=0 , p=0.0592699262622588
Cleavage Site 1263
All Other Sites
Reads
0
50
100
150
200
250
0
500
1000
1500
AT1G27360.1 position (nts)

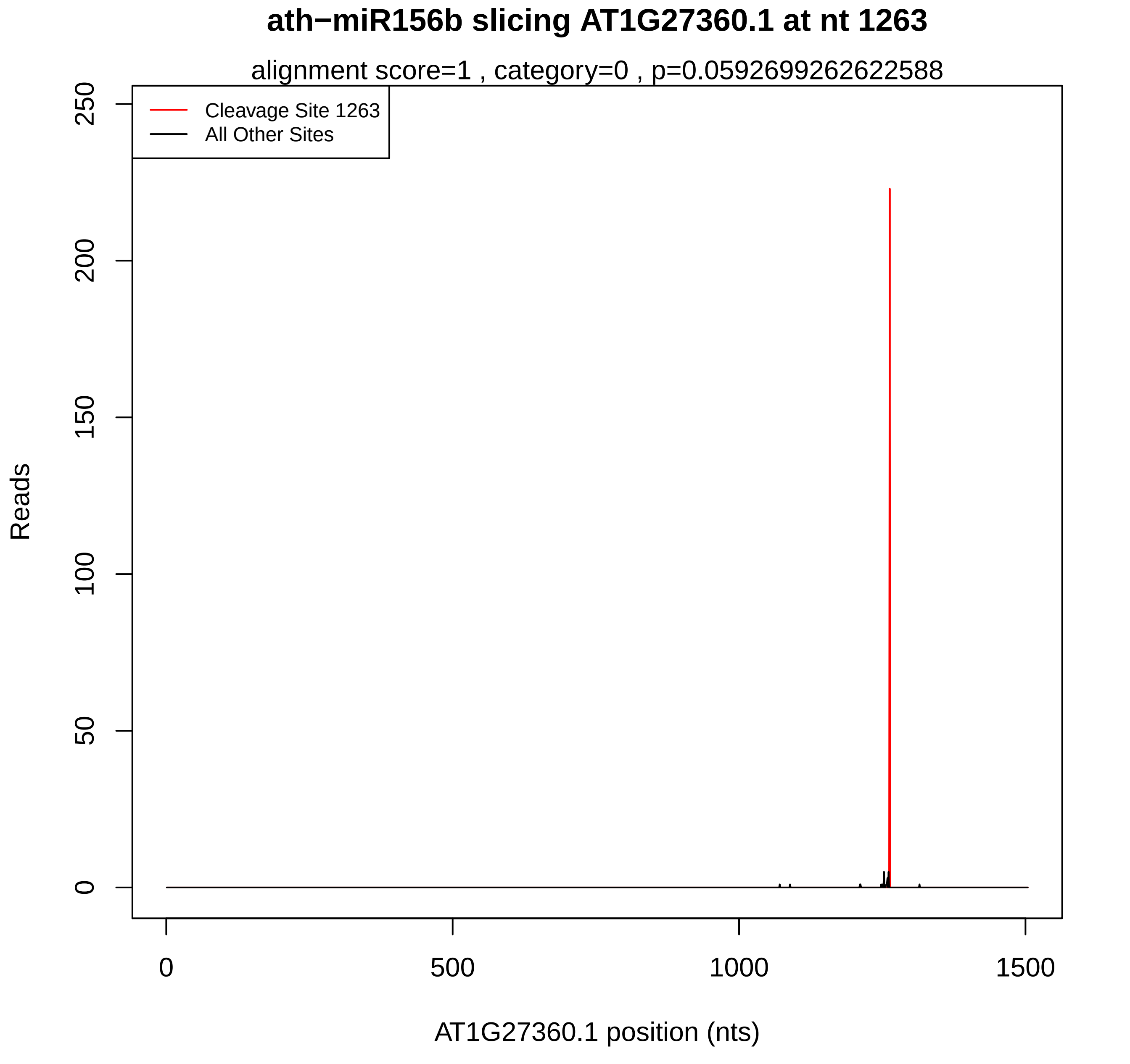
ath−miR156b slicing AT1G27360.1 at nt 1263
alignment score=1 , category=0 , p=0.0592699262622588
Cleavage Site 1263
All Other Sites
Reads
0
50
100
150
200
250
0
500
1000
1500
AT1G27360.1 position (nts)

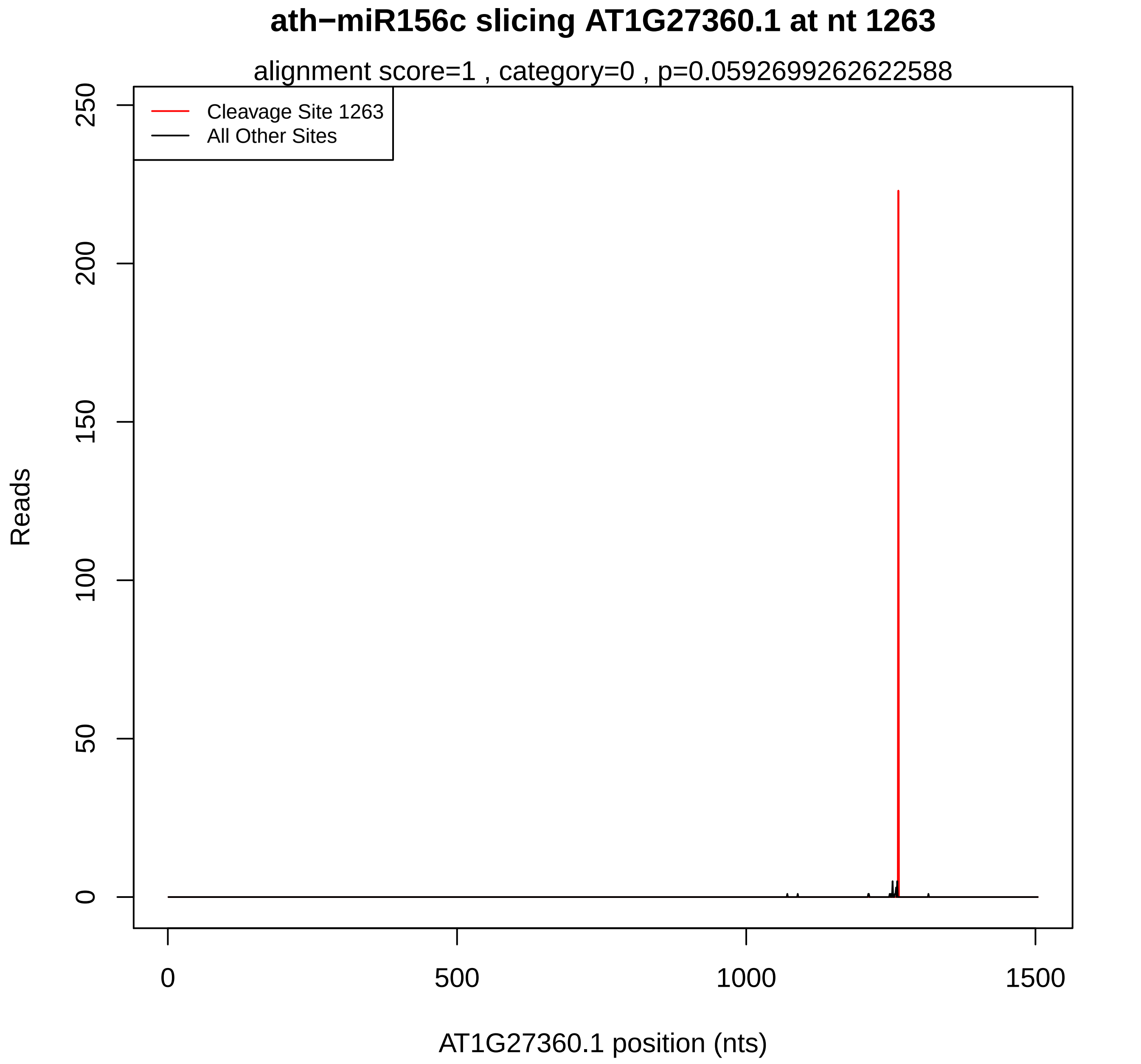
ath−miR156c slicing AT1G27360.1 at nt 1263
alignment score=1 , category=0 , p=0.0592699262622588
Cleavage Site 1263
All Other Sites
Reads
0
50
100
150
200
250
0
500
1000
1500
AT1G27360.1 position (nts)

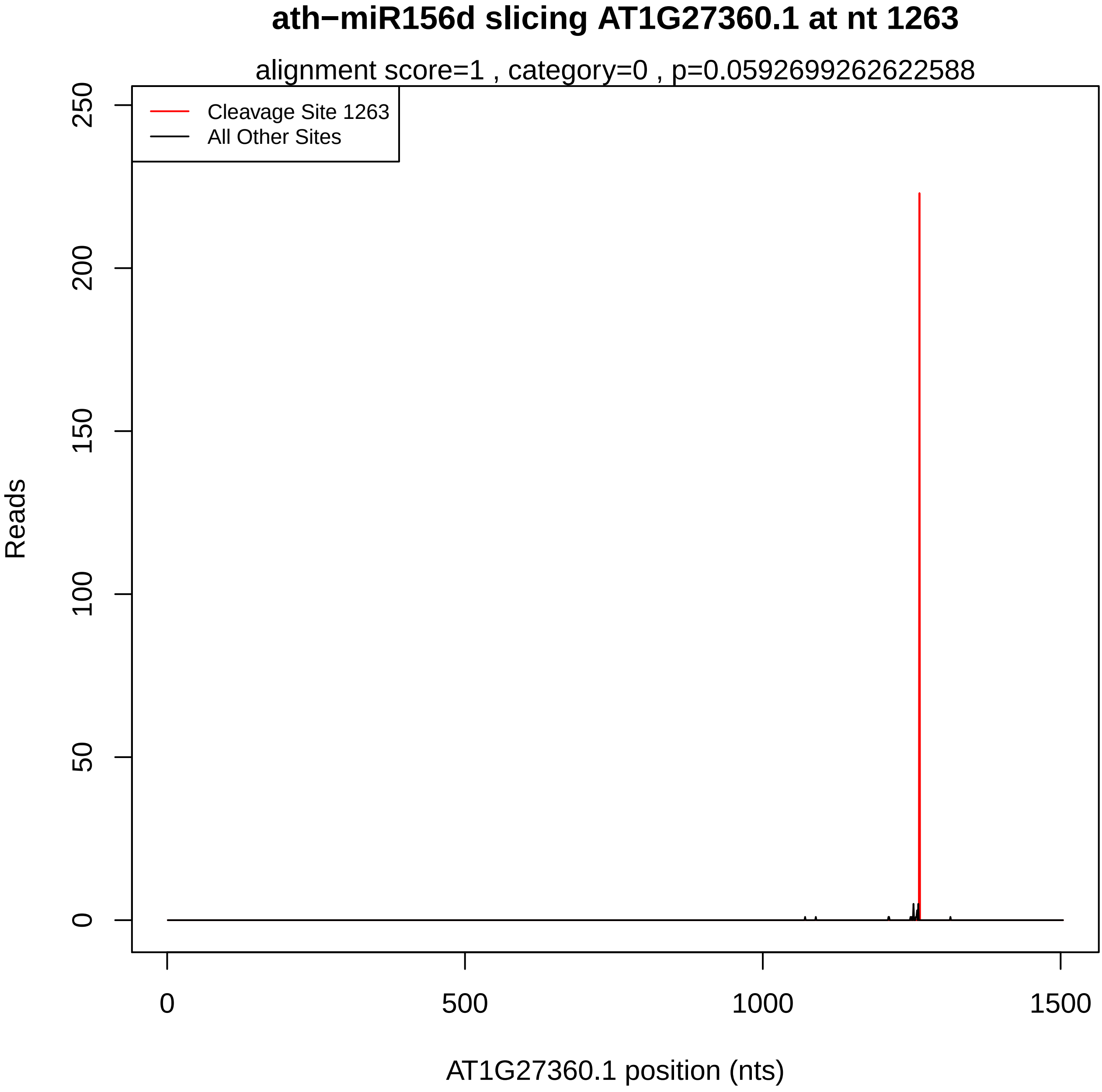
ath−miR156d slicing AT1G27360.1 at nt 1263
alignment score=1 , category=0 , p=0.0592699262622588
Cleavage Site 1263
All Other Sites
Reads
0
50
100
150
200
250
0
500
1000
1500
AT1G27360.1 position (nts)

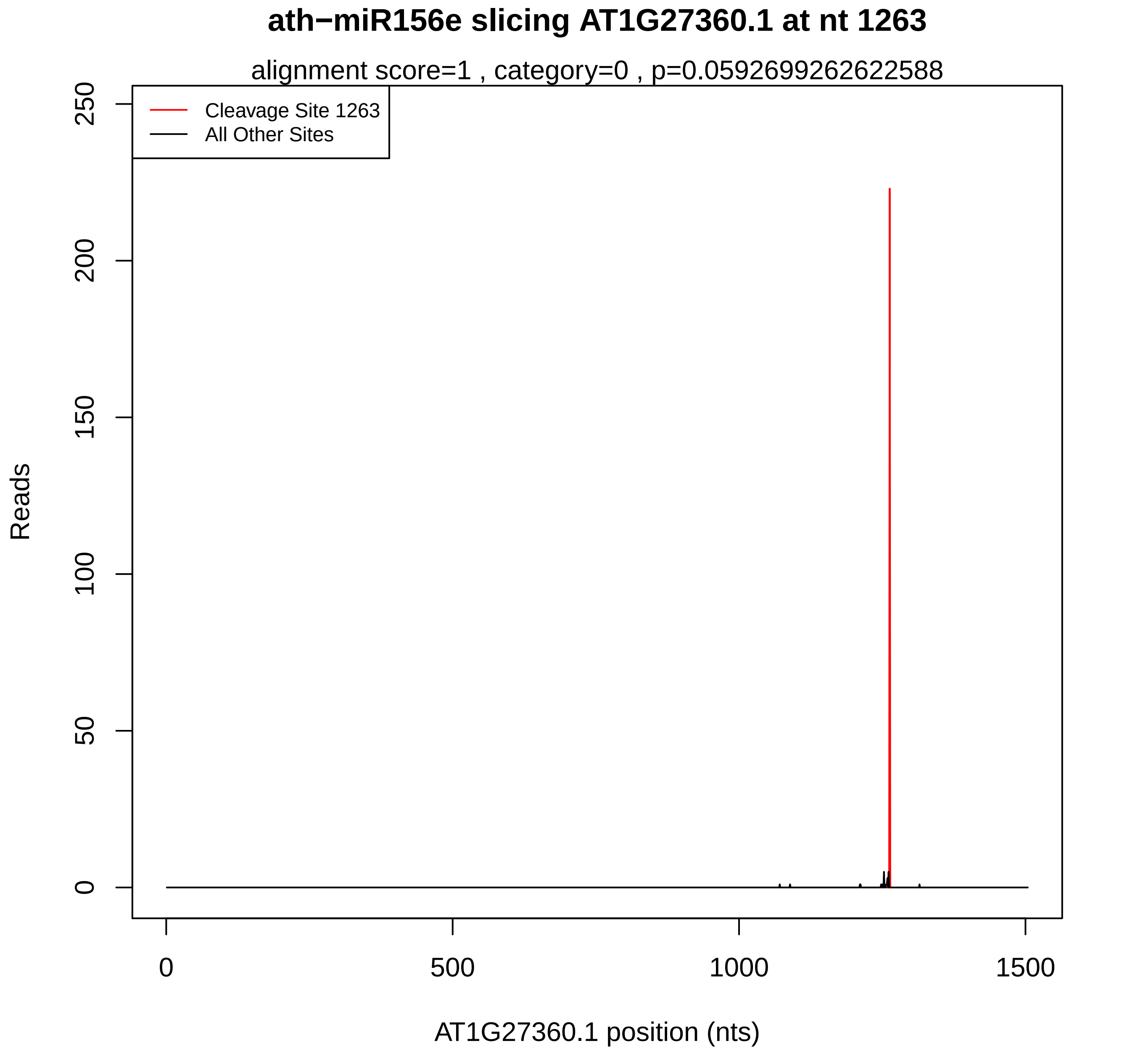
ath−miR156e slicing AT1G27360.1 at nt 1263
alignment score=1 , category=0 , p=0.0592699262622588
Cleavage Site 1263
All Other Sites
Reads
0
50
100
150
200
250
0
500
1000
1500
AT1G27360.1 position (nts)

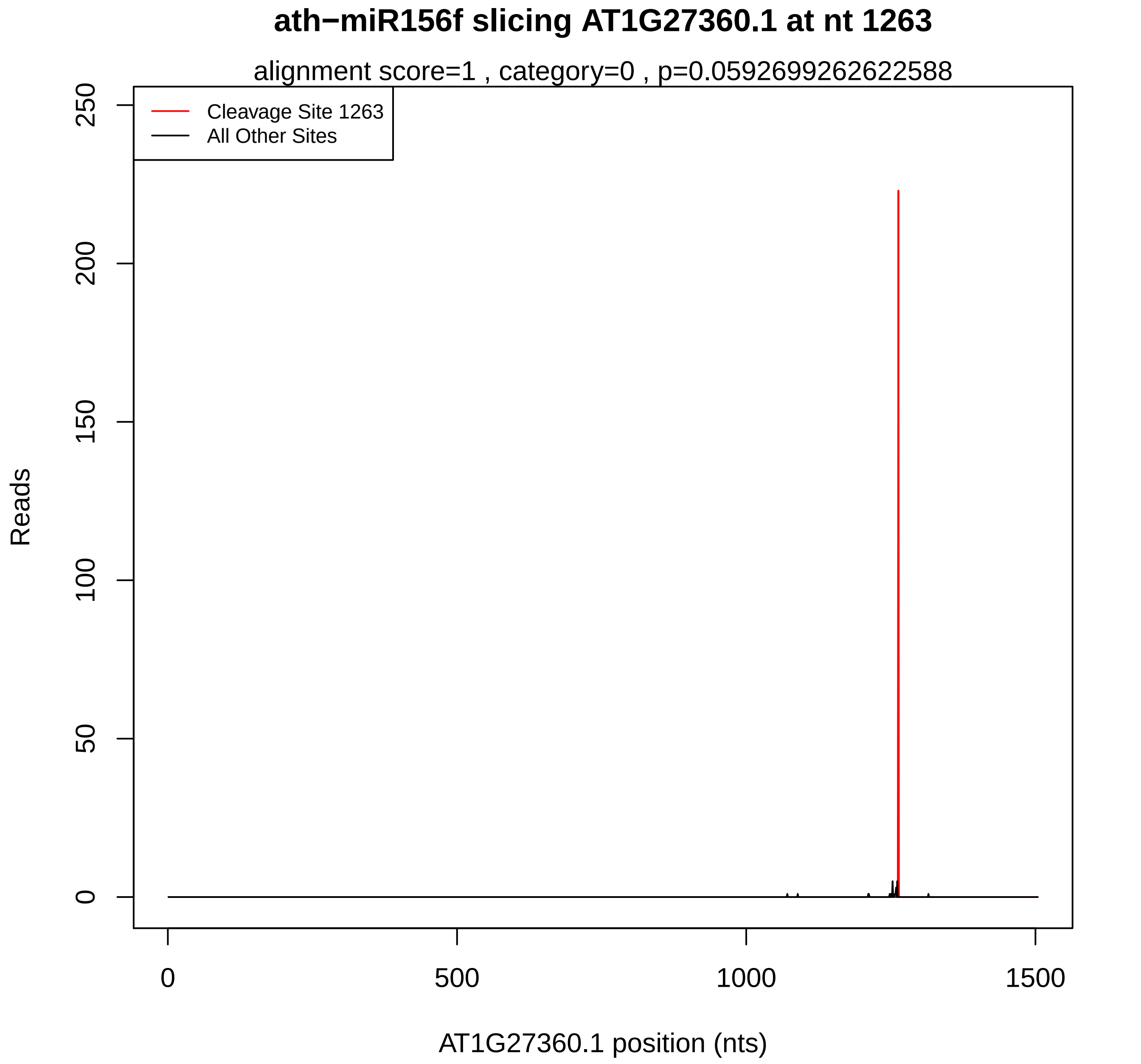
ath−miR156f slicing AT1G27360.1 at nt 1263
alignment score=1 , category=0 , p=0.0592699262622588
Cleavage Site 1263
All Other Sites
Reads
0
50
100
150
200
250
0
500
1000
1500
AT1G27360.1 position (nts)

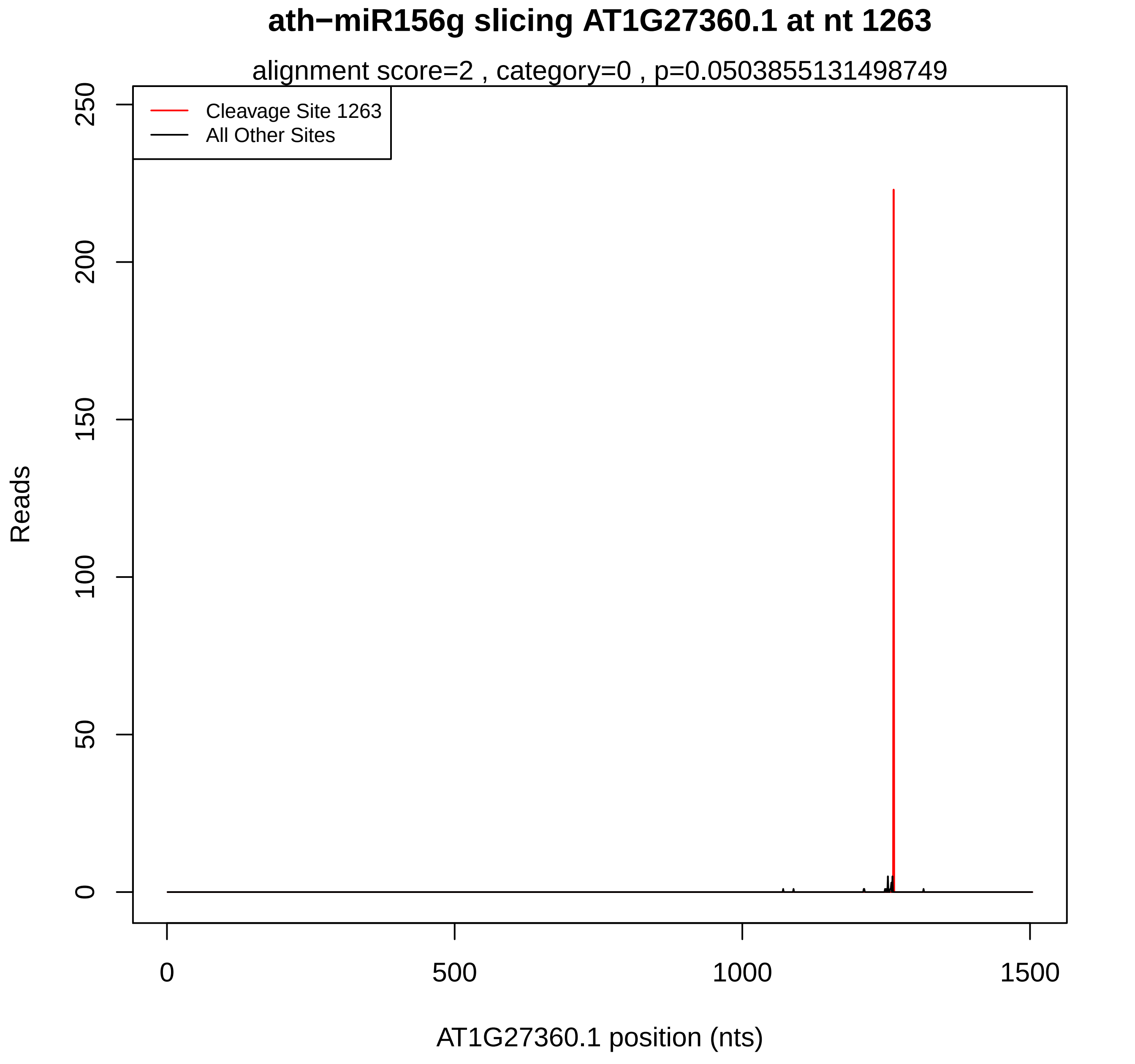
ath−miR156g slicing AT1G27360.1 at nt 1263
alignment score=2 , category=0 , p=0.0503855131498749
Cleavage Site 1263
All Other Sites
Reads
0
50
100
150
200
250
0
500
1000
1500
AT1G27360.1 position (nts)

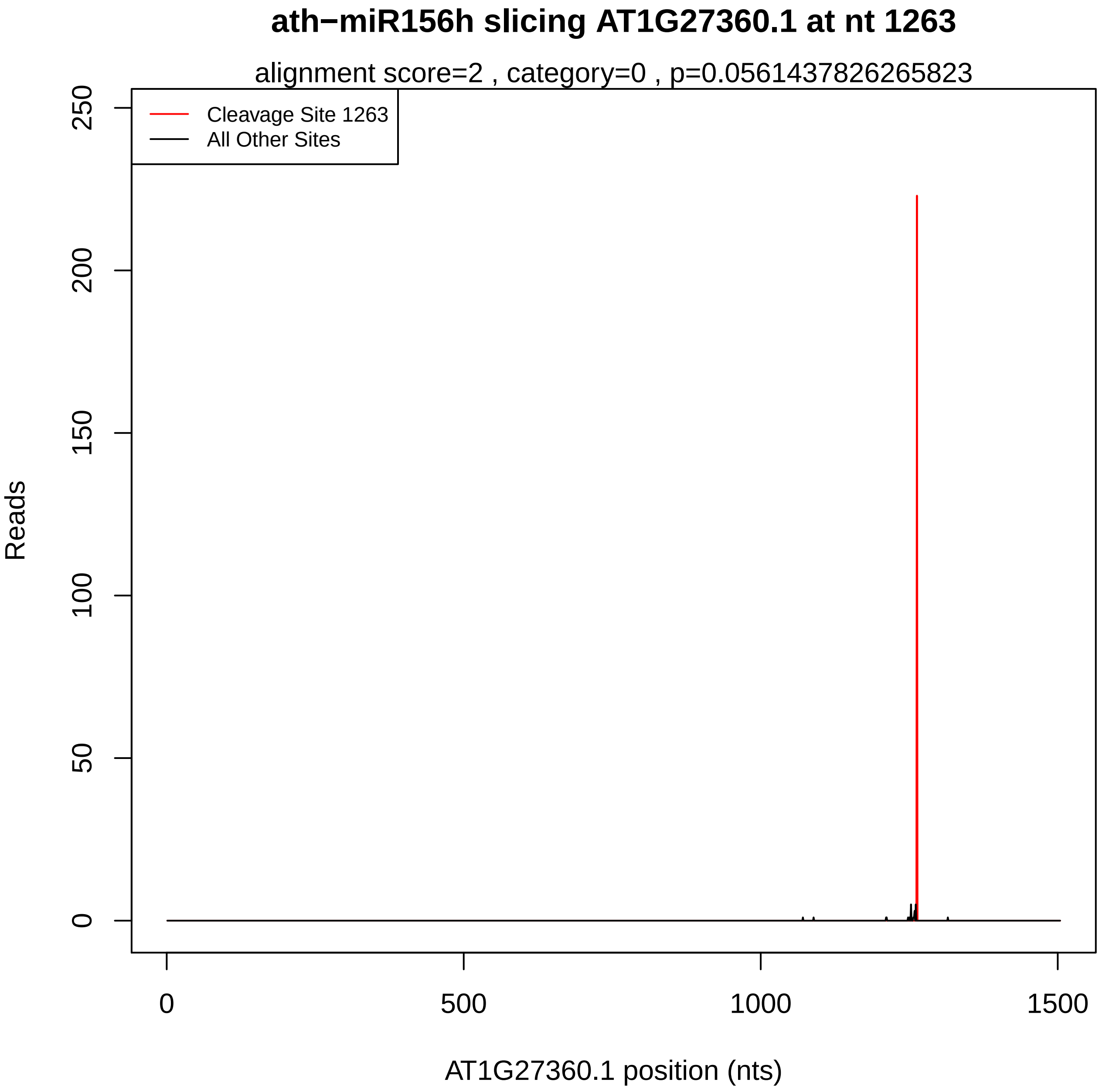

ath−miR156h slicing AT1G27360.1 at nt 1263
alignment score=2 , category=0 , p=0.0561437826265823
Cleavage Site 1263
All Other Sites
Reads
0
50
100
150
200
250
0
500
1000
1500
AT1G27360.1 position (nts)

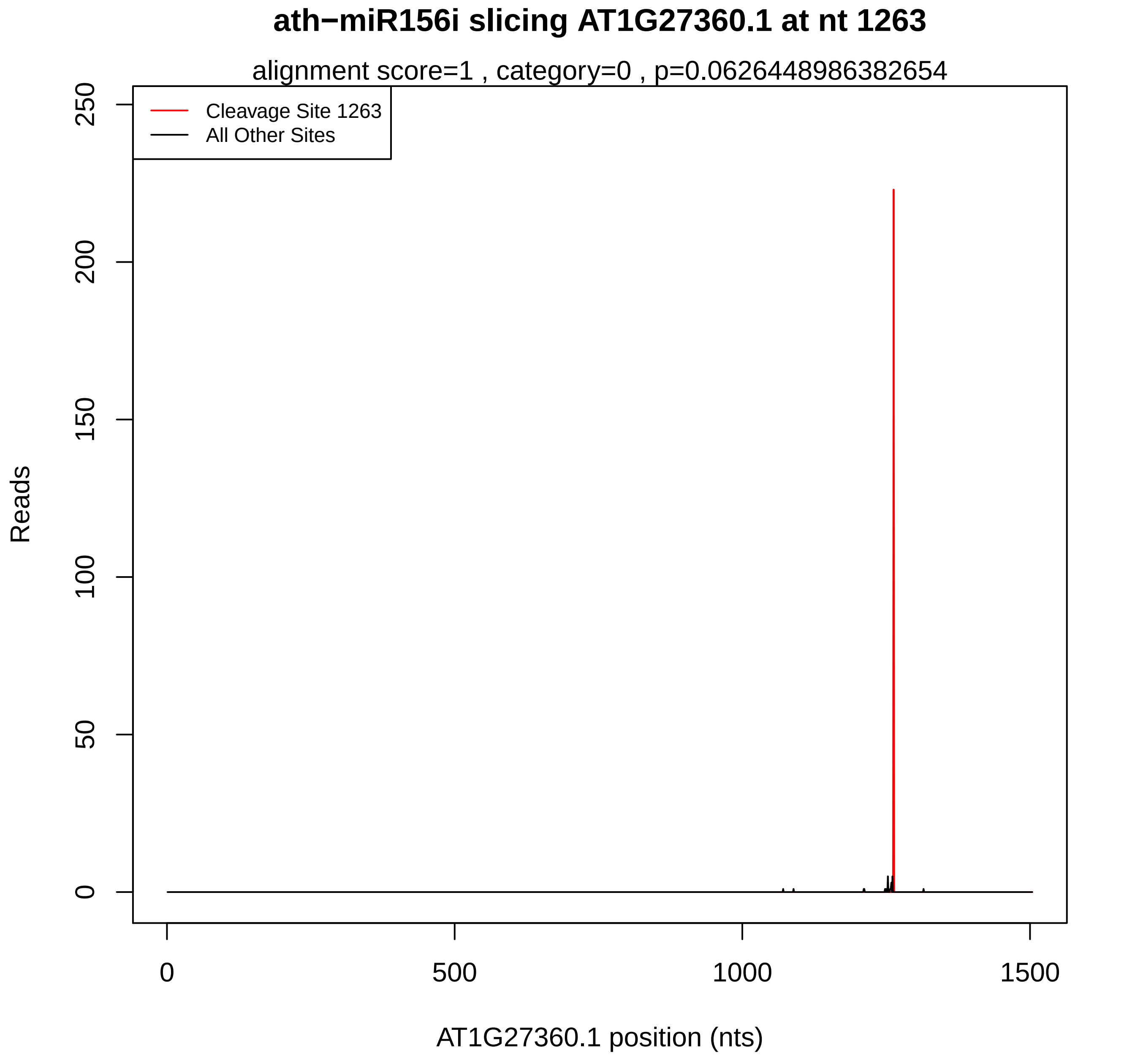
ath−miR156i slicing AT1G27360.1 at nt 1263
alignment score=1 , category=0 , p=0.0626448986382654
Cleavage Site 1263
All Other Sites
Reads
0
50
100
150
200
250
0
500
1000
1500
AT1G27360.1 position (nts)

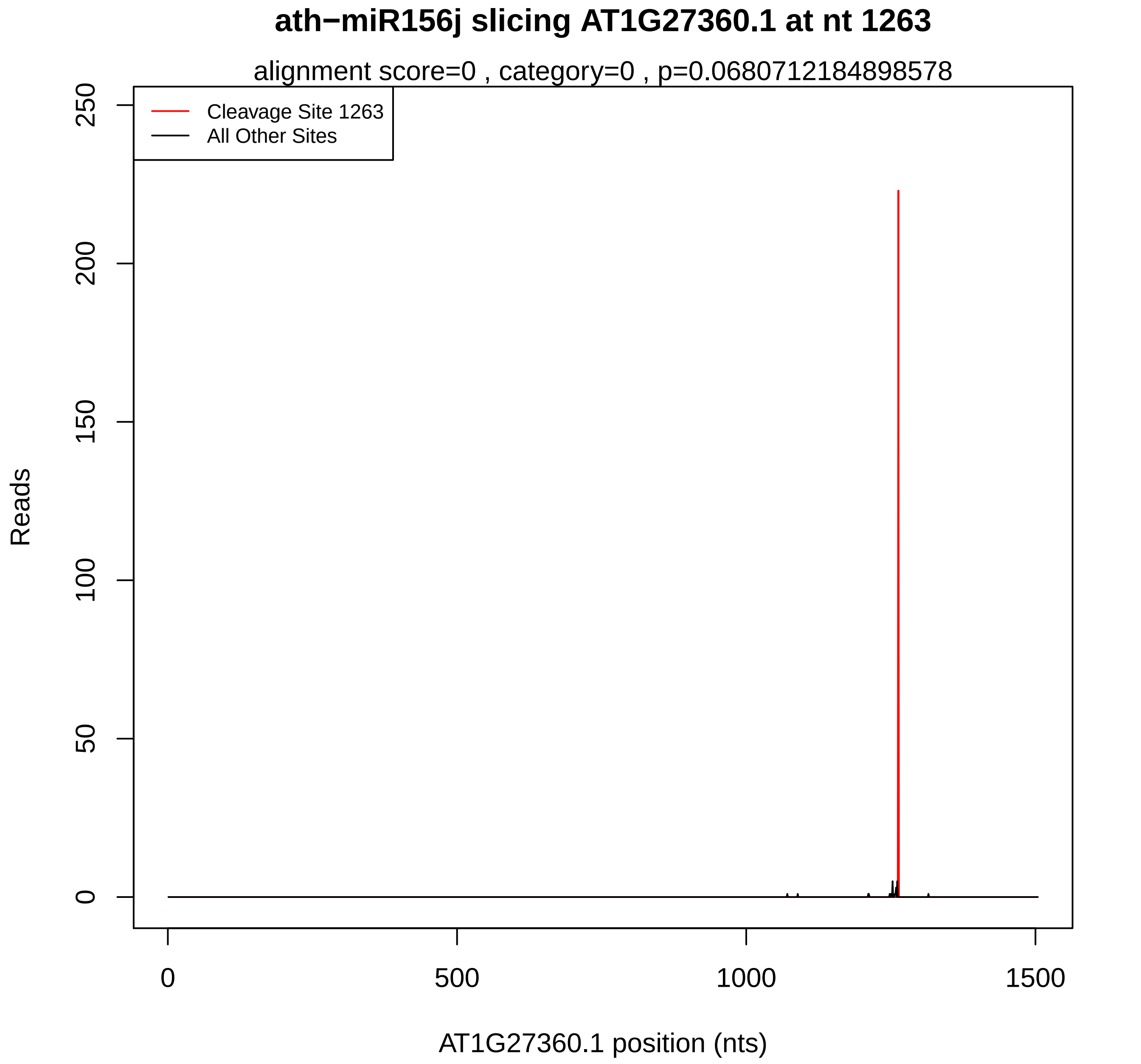

ath−miR156j slicing AT1G27360.1 at nt 1263
alignment score=0 , category=0 , p=0.0680712184898578
Cleavage Site 1263
All Other Sites
Reads
0
50
100
150
200
250
0
500
1000
1500
AT1G27360.1 position (nts)

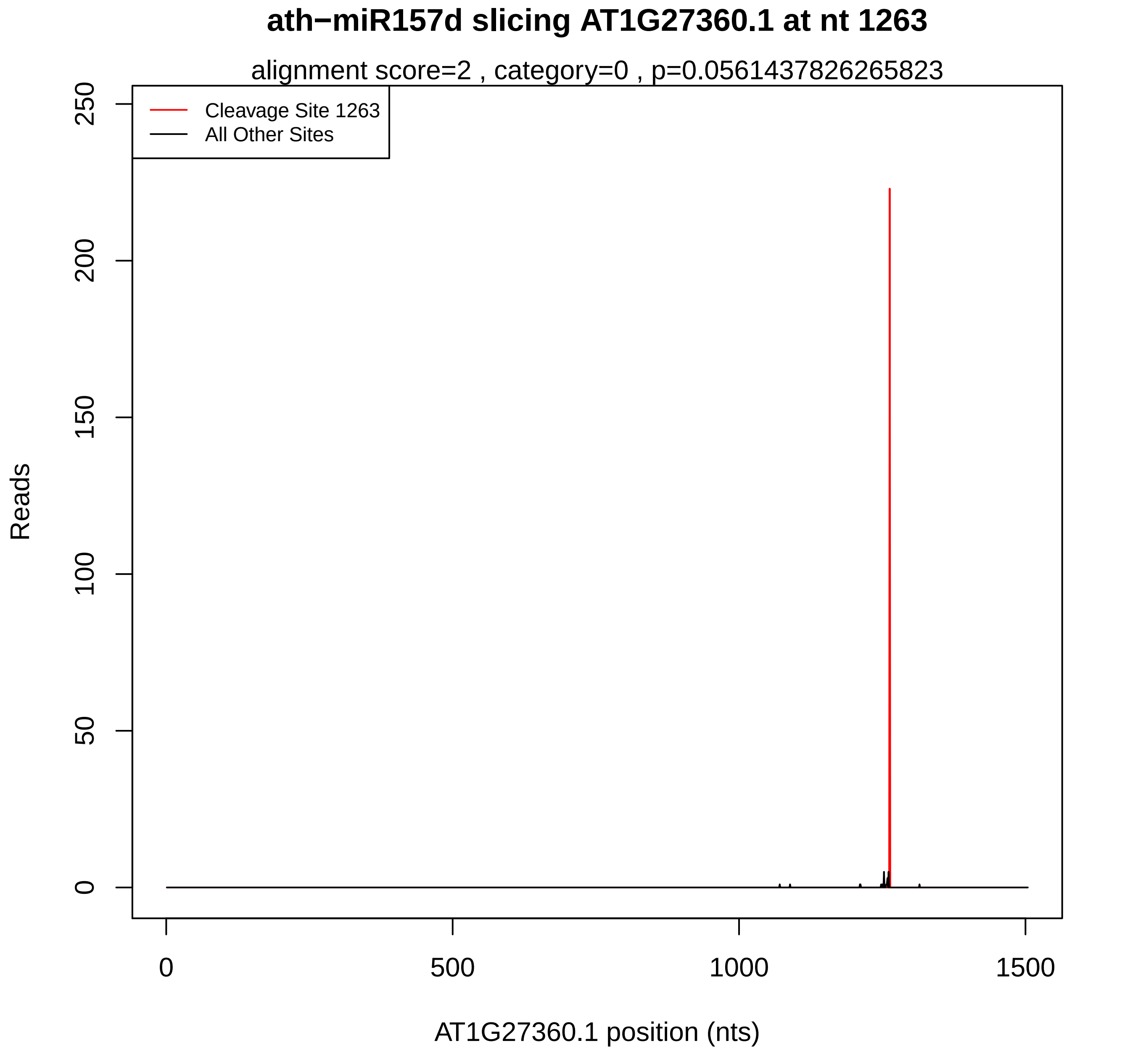

ath−miR157d slicing AT1G27360.1 at nt 1263
alignment score=2 , category=0 , p=0.0561437826265823
Cleavage Site 1263
All Other Sites
Reads
0
50
100
150
200
250
0
500
1000
1500
AT1G27360.1 position (nts)

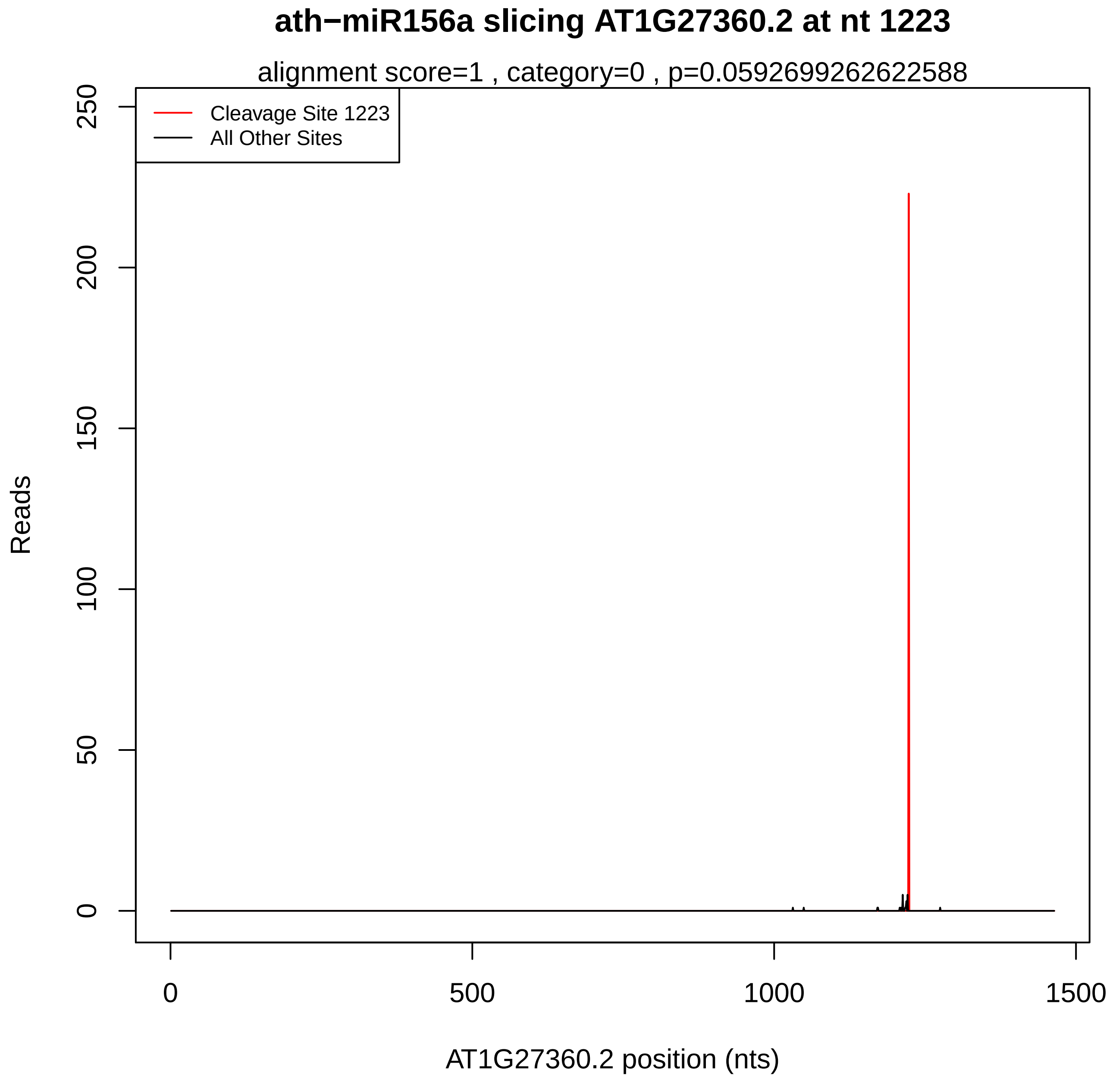
ath−miR156a slicing AT1G27360.2 at nt 1223
alignment score=1 , category=0 , p=0.0592699262622588
Cleavage Site 1223
All Other Sites
Reads
0
50
100
150
200
250
0
500
1000
1500
AT1G27360.2 position (nts)

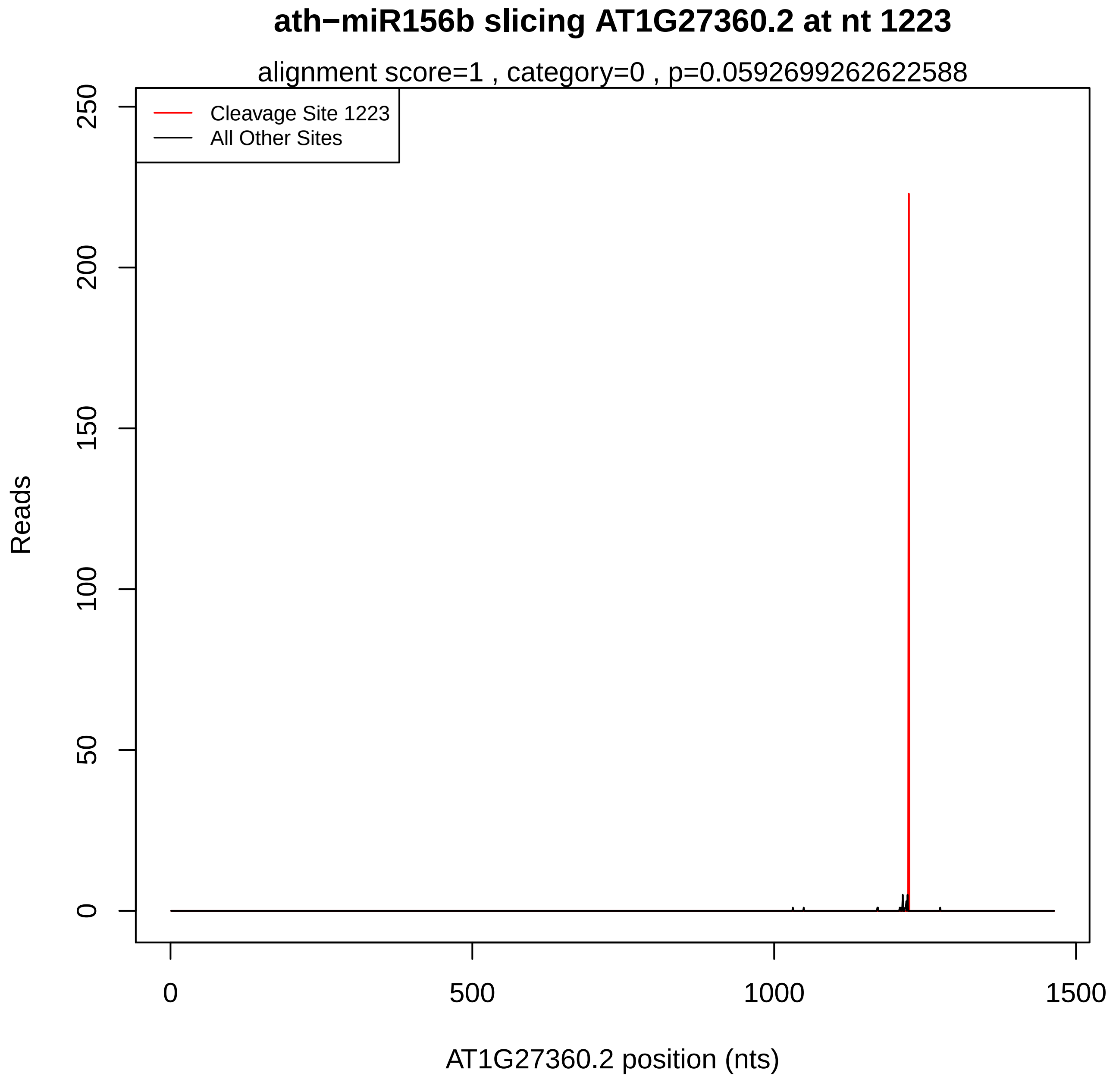
ath−miR156b slicing AT1G27360.2 at nt 1223
alignment score=1 , category=0 , p=0.0592699262622588
Cleavage Site 1223
All Other Sites
Reads
0
50
100
150
200
250
0
500
1000
1500
AT1G27360.2 position (nts)

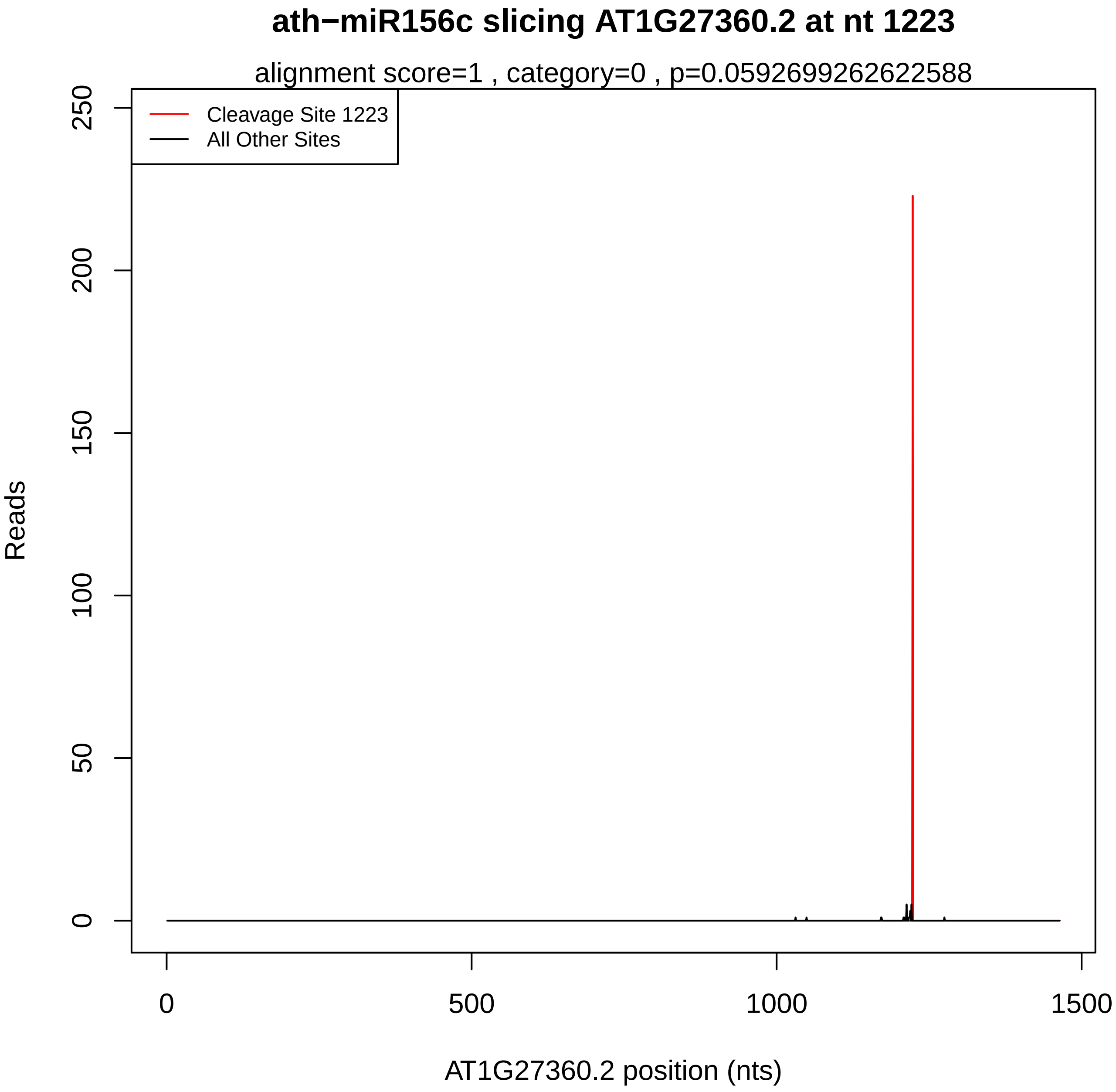
ath−miR156c slicing AT1G27360.2 at nt 1223
alignment score=1 , category=0 , p=0.0592699262622588
Cleavage Site 1223
All Other Sites
Reads
0
50
100
150
200
250
0
500
1000
1500
AT1G27360.2 position (nts)

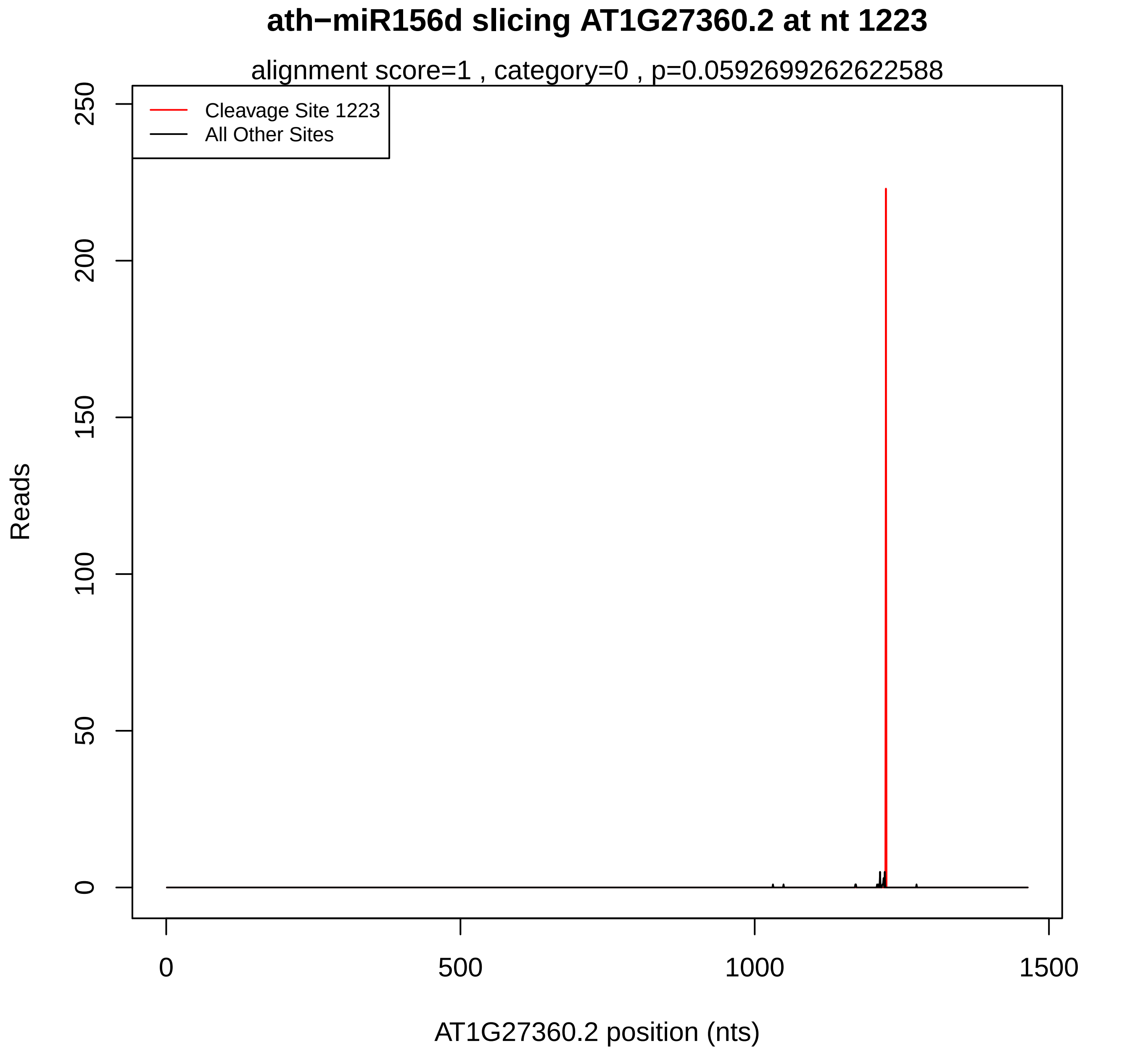
ath−miR156d slicing AT1G27360.2 at nt 1223
alignment score=1 , category=0 , p=0.0592699262622588
Cleavage Site 1223
All Other Sites
Reads
0
50
100
150
200
250
0
500
1000
1500
AT1G27360.2 position (nts)

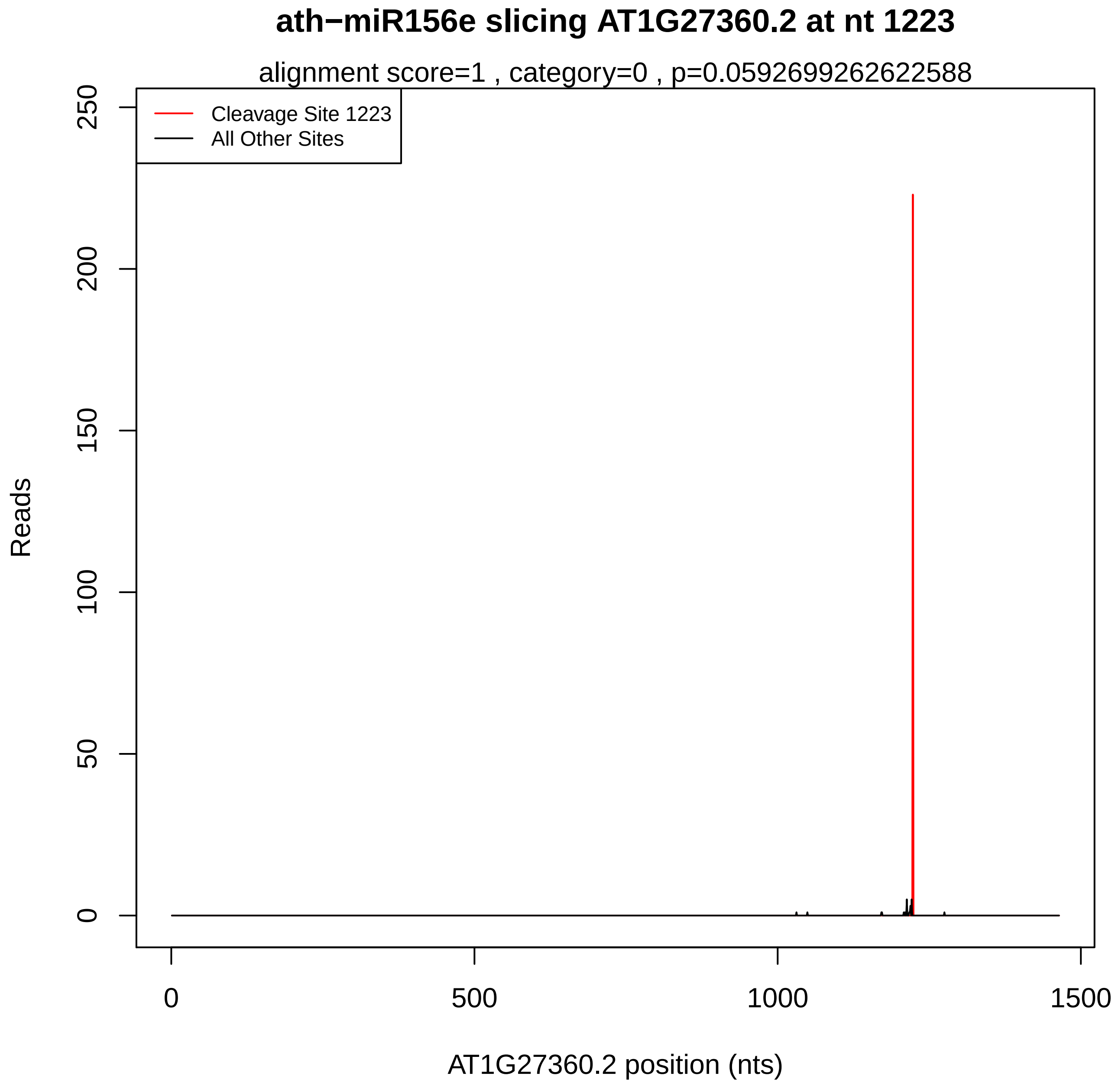

ath−miR156e slicing AT1G27360.2 at nt 1223
alignment score=1 , category=0 , p=0.0592699262622588
Cleavage Site 1223
All Other Sites
Reads
0
50
100
150
200
250
0
500
1000
1500
AT1G27360.2 position (nts)

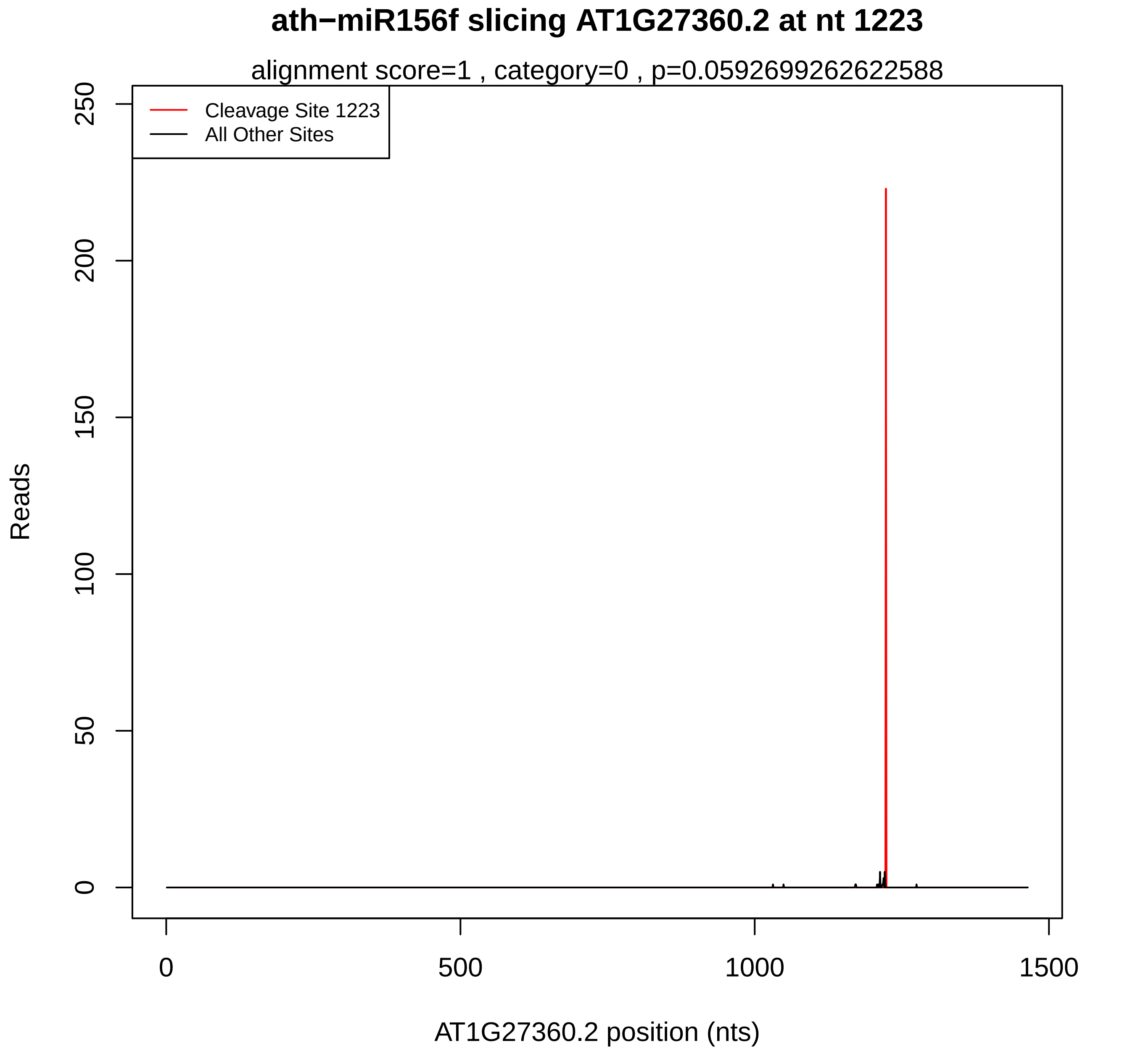

ath−miR156f slicing AT1G27360.2 at nt 1223
alignment score=1 , category=0 , p=0.0592699262622588
Cleavage Site 1223
All Other Sites
Reads
0
50
100
150
200
250
0
500
1000
1500
AT1G27360.2 position (nts)

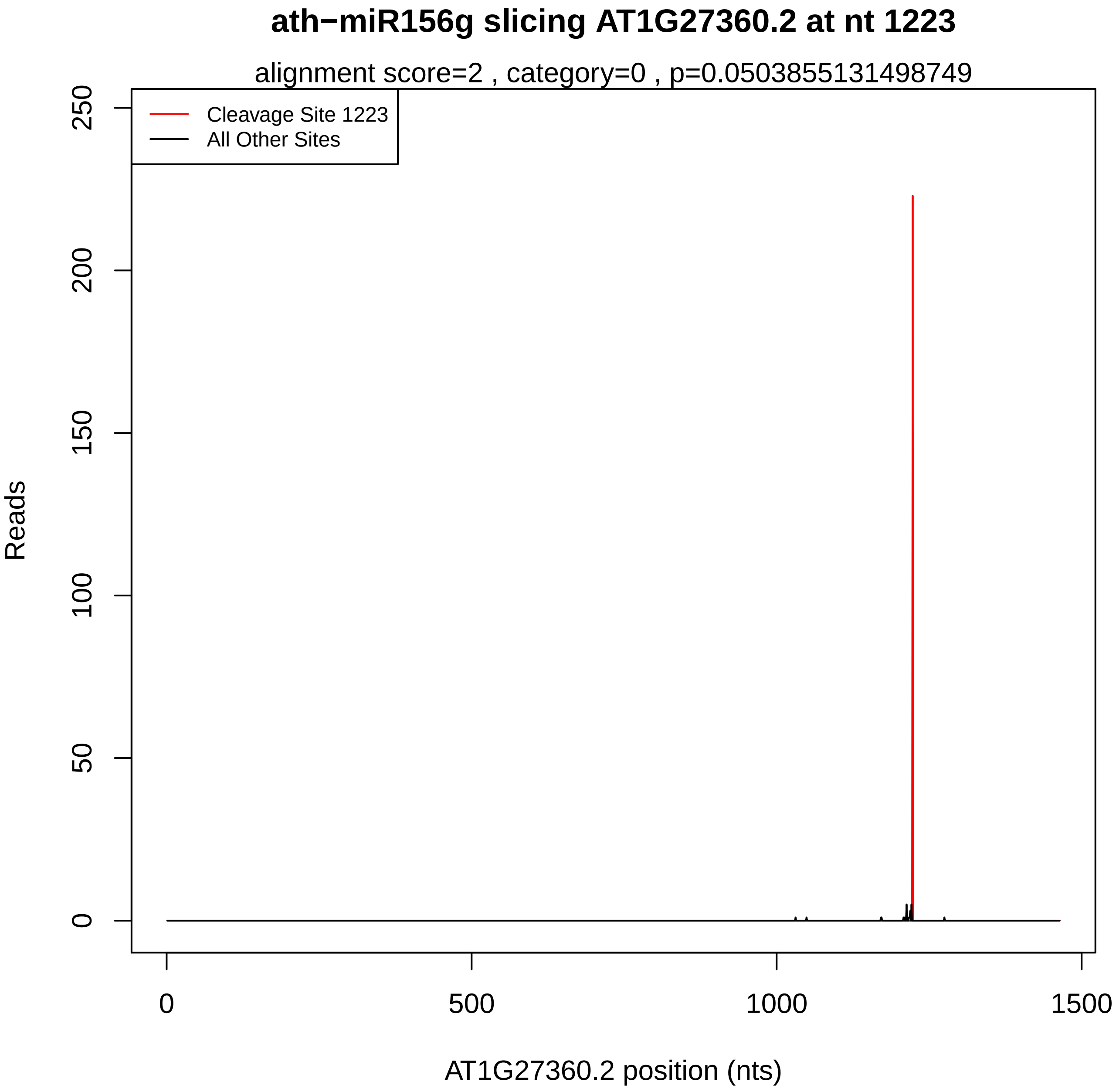
ath−miR156g slicing AT1G27360.2 at nt 1223
alignment score=2 , category=0 , p=0.0503855131498749
Cleavage Site 1223
All Other Sites
Reads
0
50
100
150
200
250
0
500
1000
1500
AT1G27360.2 position (nts)

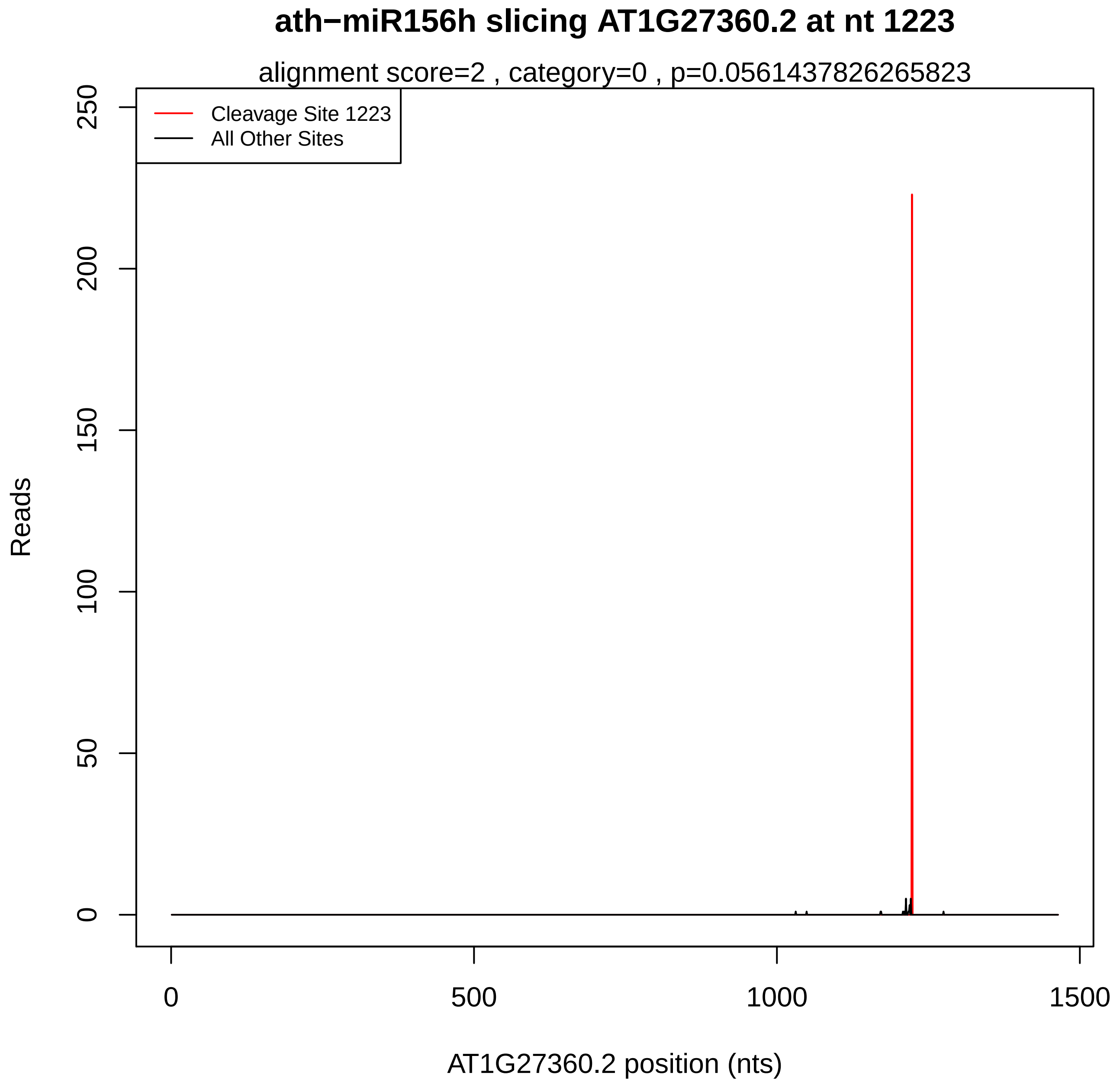
ath−miR156h slicing AT1G27360.2 at nt 1223
alignment score=2 , category=0 , p=0.0561437826265823
Cleavage Site 1223
All Other Sites
Reads
0
50
100
150
200
250
0
500
1000
1500
AT1G27360.2 position (nts)

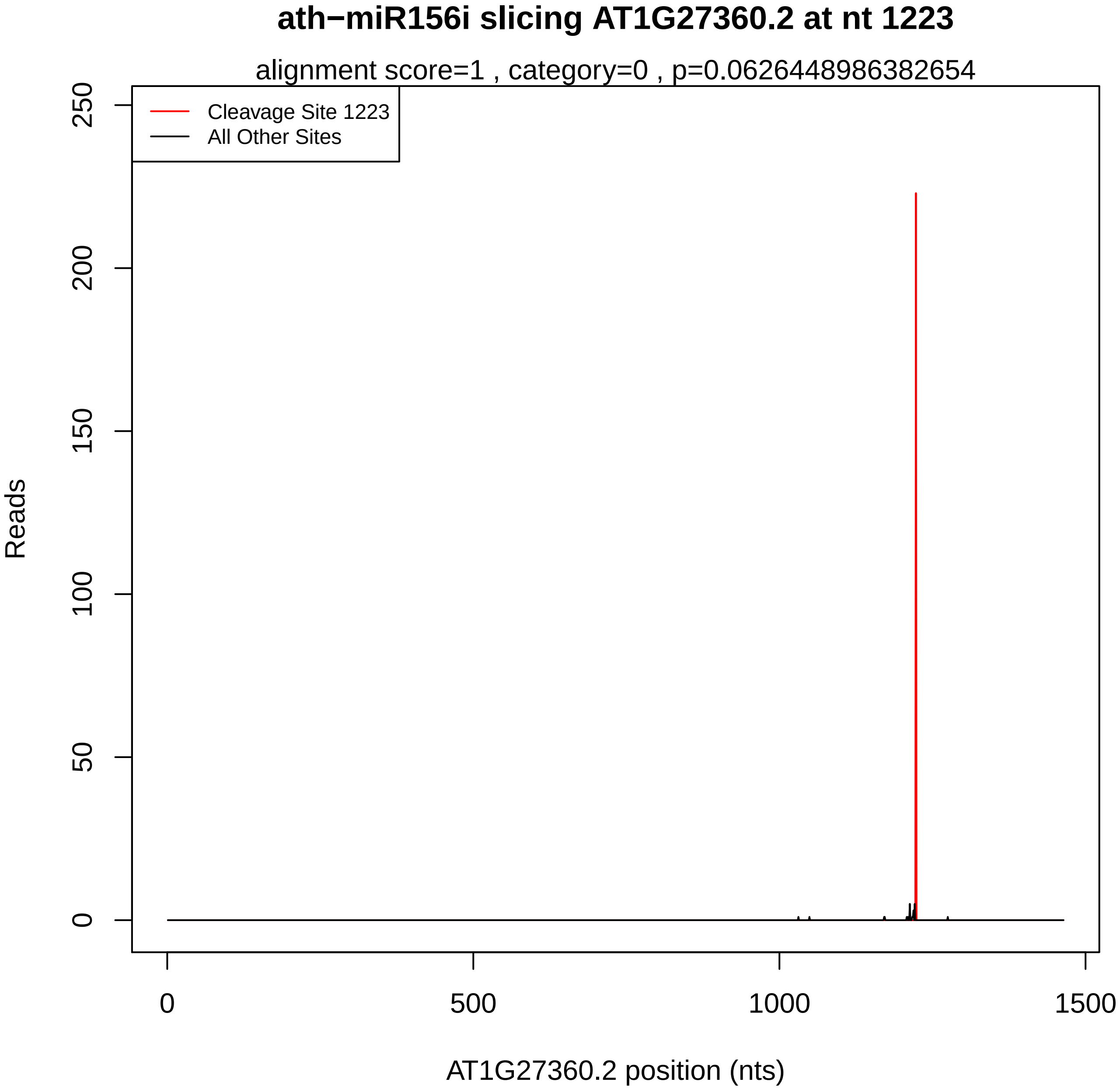
ath−miR156i slicing AT1G27360.2 at nt 1223
alignment score=1 , category=0 , p=0.0626448986382654
Cleavage Site 1223
All Other Sites
Reads
0
50
100
150
200
250
0
500
1000
1500
AT1G27360.2 position (nts)

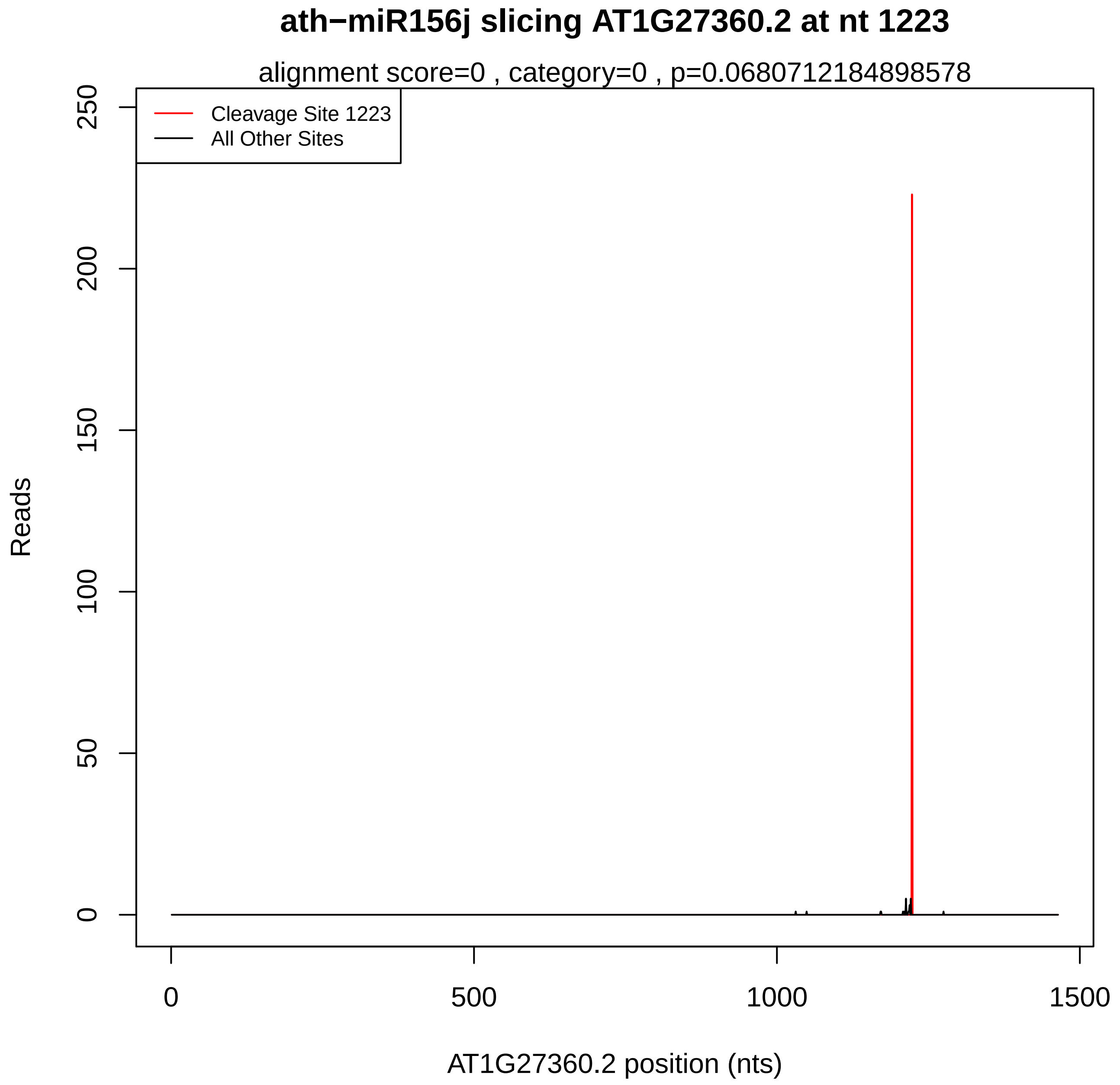
ath−miR156j slicing AT1G27360.2 at nt 1223
alignment score=0 , category=0 , p=0.0680712184898578
Cleavage Site 1223
All Other Sites
Reads
0
50
100
150
200
250
0
500
1000
1500
AT1G27360.2 position (nts)

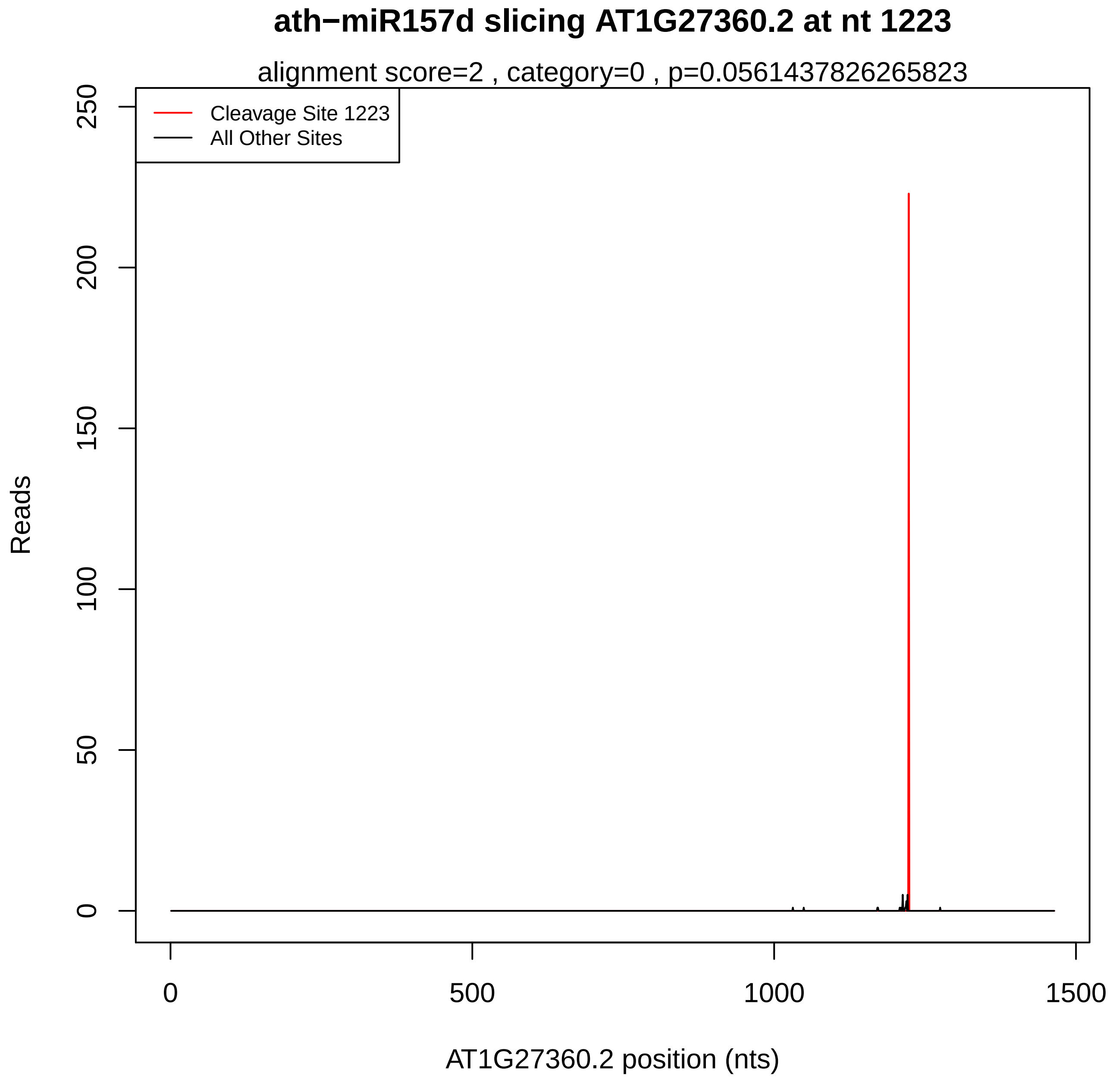

ath−miR157d slicing AT1G27360.2 at nt 1223
alignment score=2 , category=0 , p=0.0561437826265823
Cleavage Site 1223
All Other Sites
Reads
0
50
100
150
200
250
0
500
1000
1500
AT1G27360.2 position (nts)

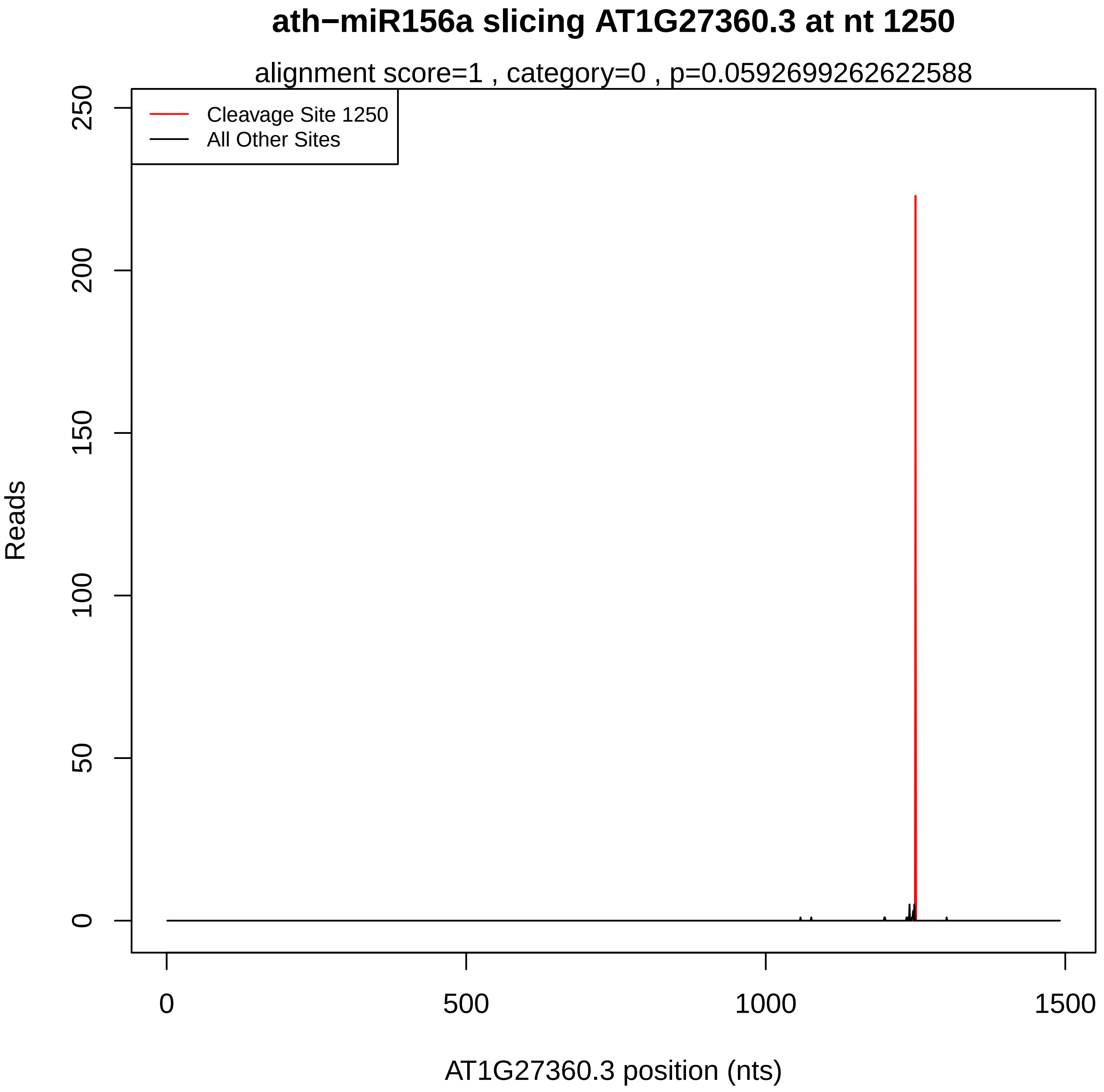
ath−miR156a slicing AT1G27360.3 at nt 1250
alignment score=1 , category=0 , p=0.0592699262622588
Cleavage Site 1250
All Other Sites
Reads
0
50
100
150
200
250
0
500
1000
1500
AT1G27360.3 position (nts)

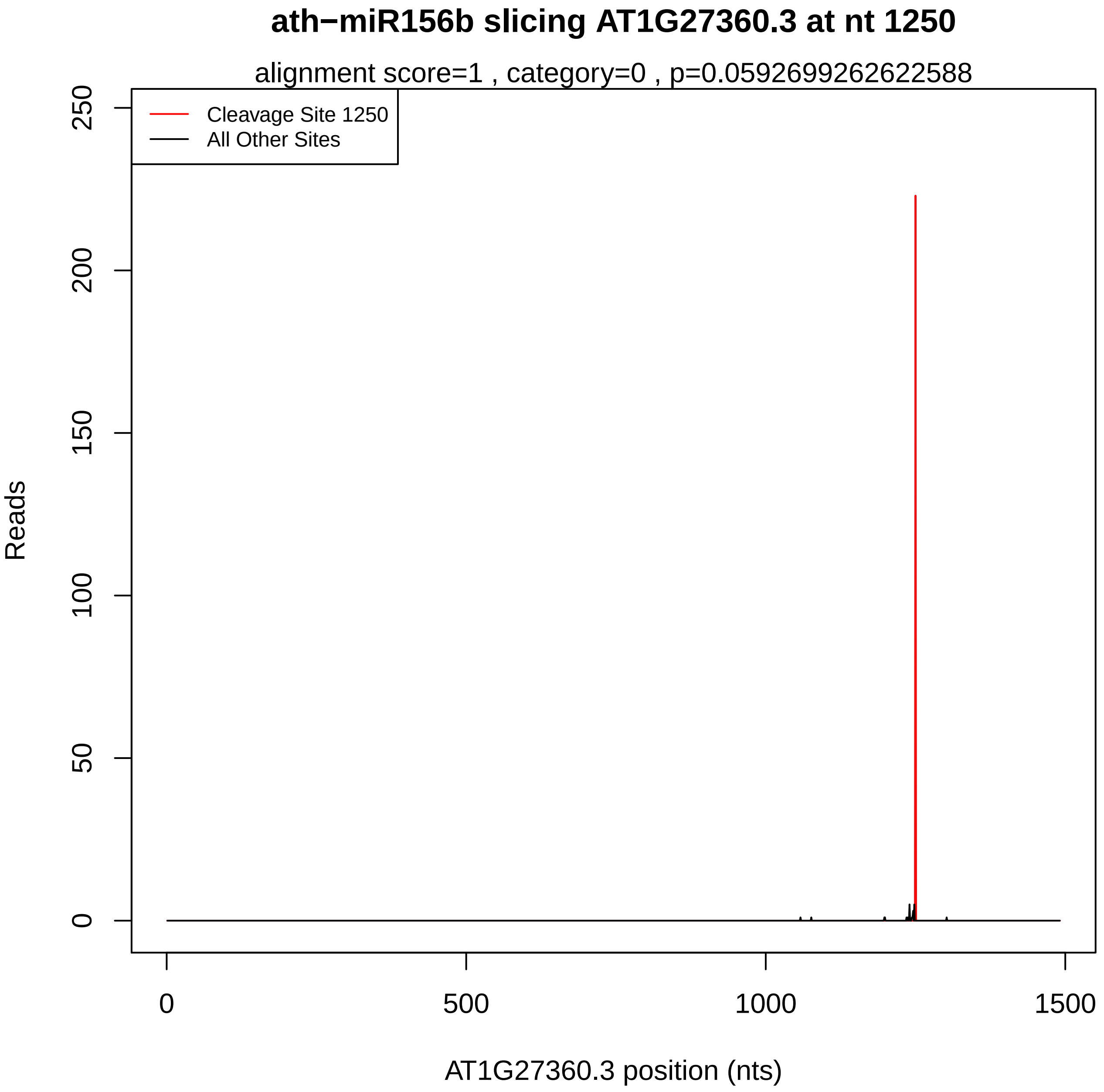
ath−miR156b slicing AT1G27360.3 at nt 1250
alignment score=1 , category=0 , p=0.0592699262622588
Cleavage Site 1250
All Other Sites
Reads
0
50
100
150
200
250
0
500
1000
1500
AT1G27360.3 position (nts)

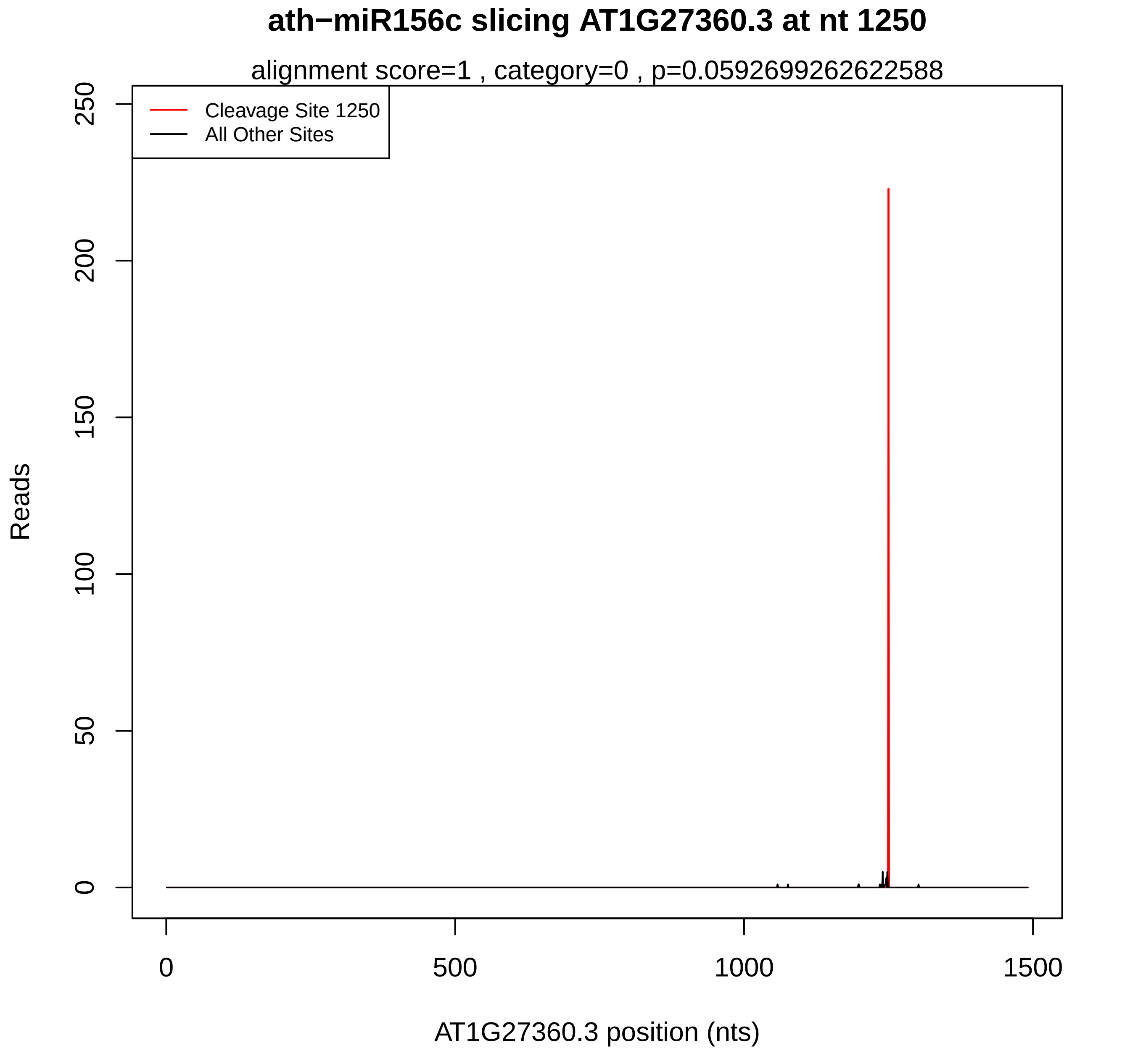

ath−miR156c slicing AT1G27360.3 at nt 1250
alignment score=1 , category=0 , p=0.0592699262622588
Cleavage Site 1250
All Other Sites
Reads
0
50
100
150
200
250
0
500
1000
1500
AT1G27360.3 position (nts)

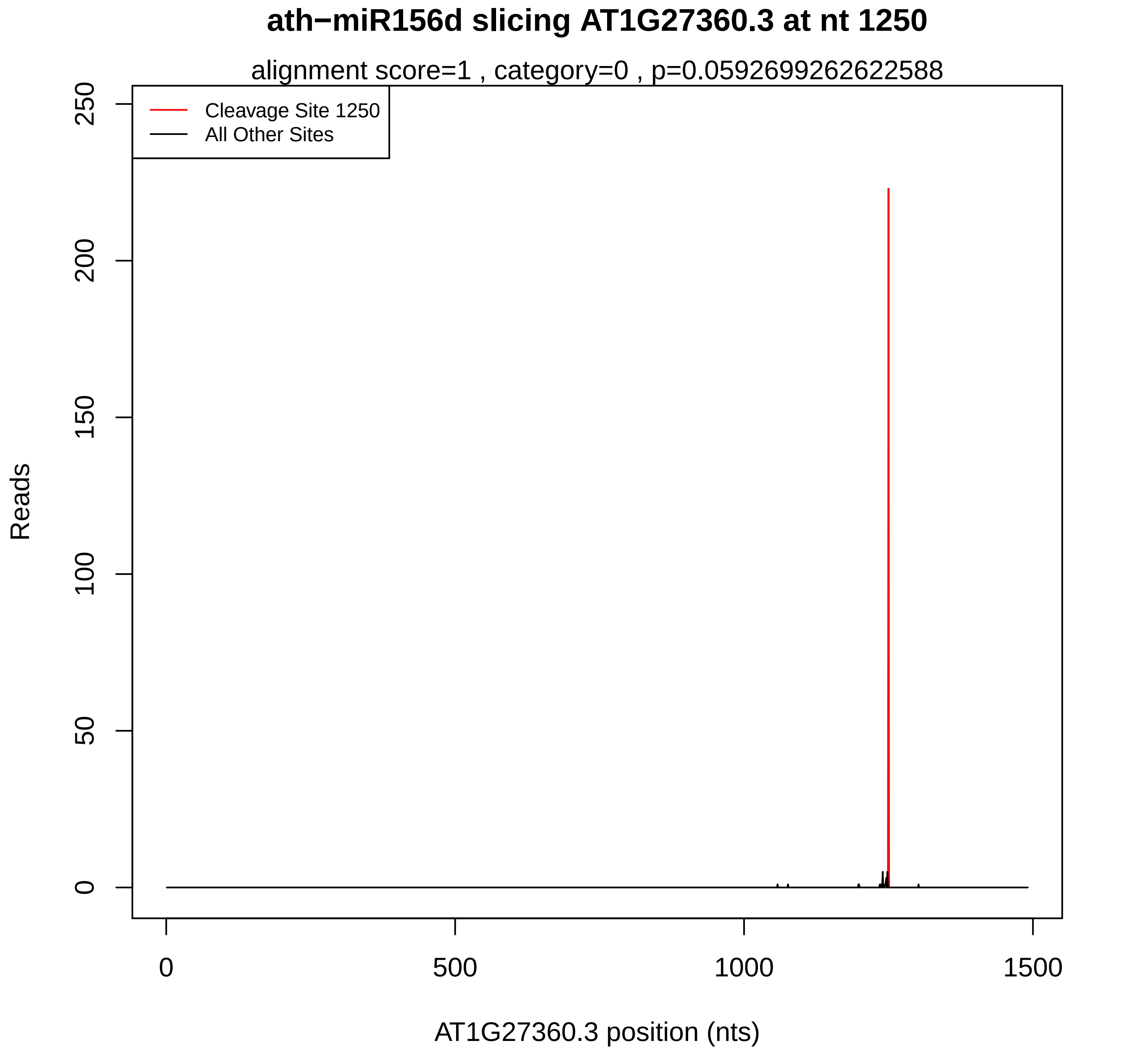
ath−miR156d slicing AT1G27360.3 at nt 1250
alignment score=1 , category=0 , p=0.0592699262622588
Cleavage Site 1250
All Other Sites
Reads
0
50
100
150
200
250
0
500
1000
1500
AT1G27360.3 position (nts)

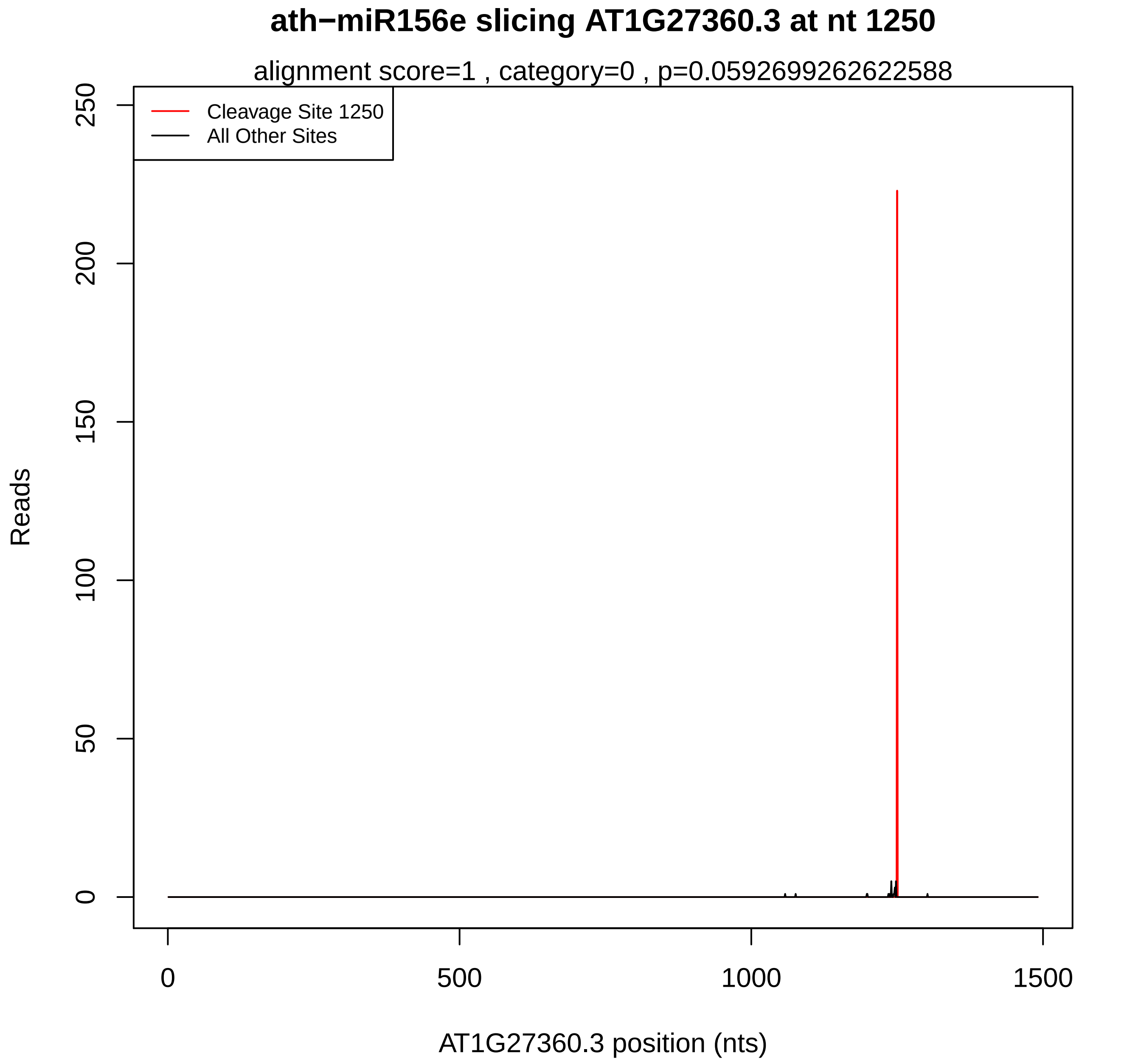
ath−miR156e slicing AT1G27360.3 at nt 1250
alignment score=1 , category=0 , p=0.0592699262622588
Cleavage Site 1250
All Other Sites
Reads
0
50
100
150
200
250
0
500
1000
1500
AT1G27360.3 position (nts)

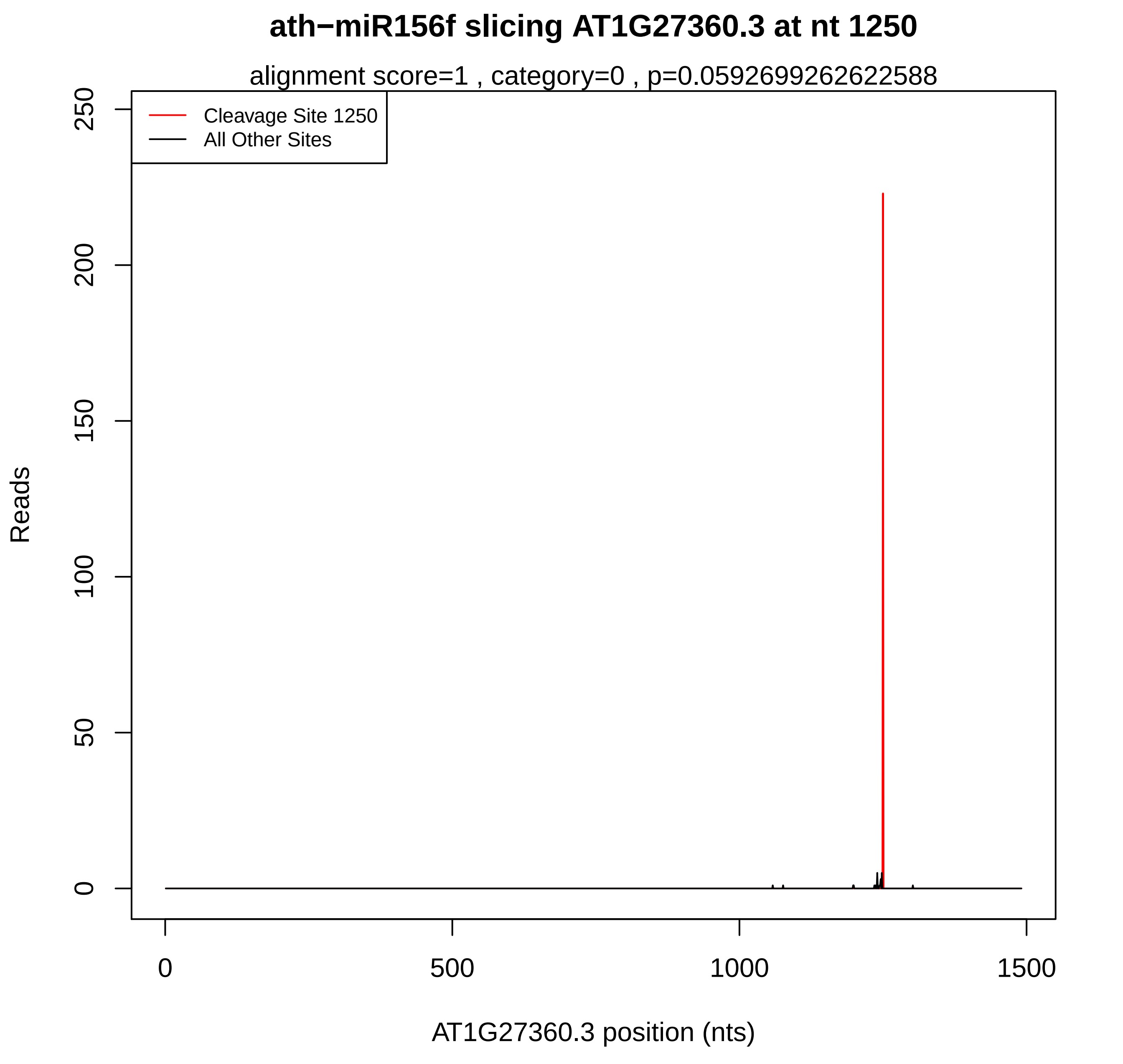

ath−miR156f slicing AT1G27360.3 at nt 1250
alignment score=1 , category=0 , p=0.0592699262622588
Cleavage Site 1250
All Other Sites
Reads
0
50
100
150
200
250
0
500
1000
1500
AT1G27360.3 position (nts)

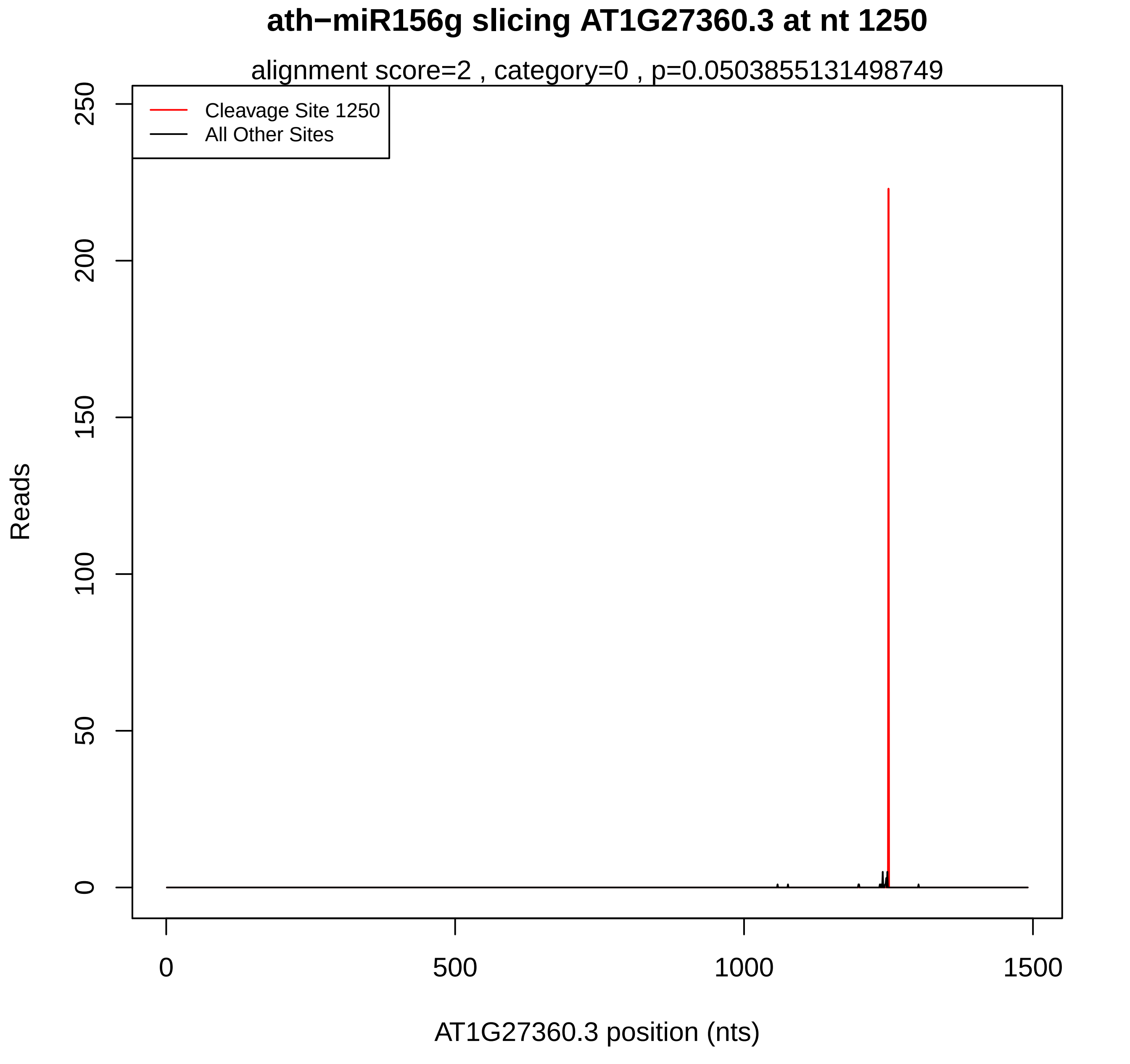
ath−miR156g slicing AT1G27360.3 at nt 1250
alignment score=2 , category=0 , p=0.0503855131498749
Cleavage Site 1250
All Other Sites
Reads
0
50
100
150
200
250
0
500
1000
1500
AT1G27360.3 position (nts)

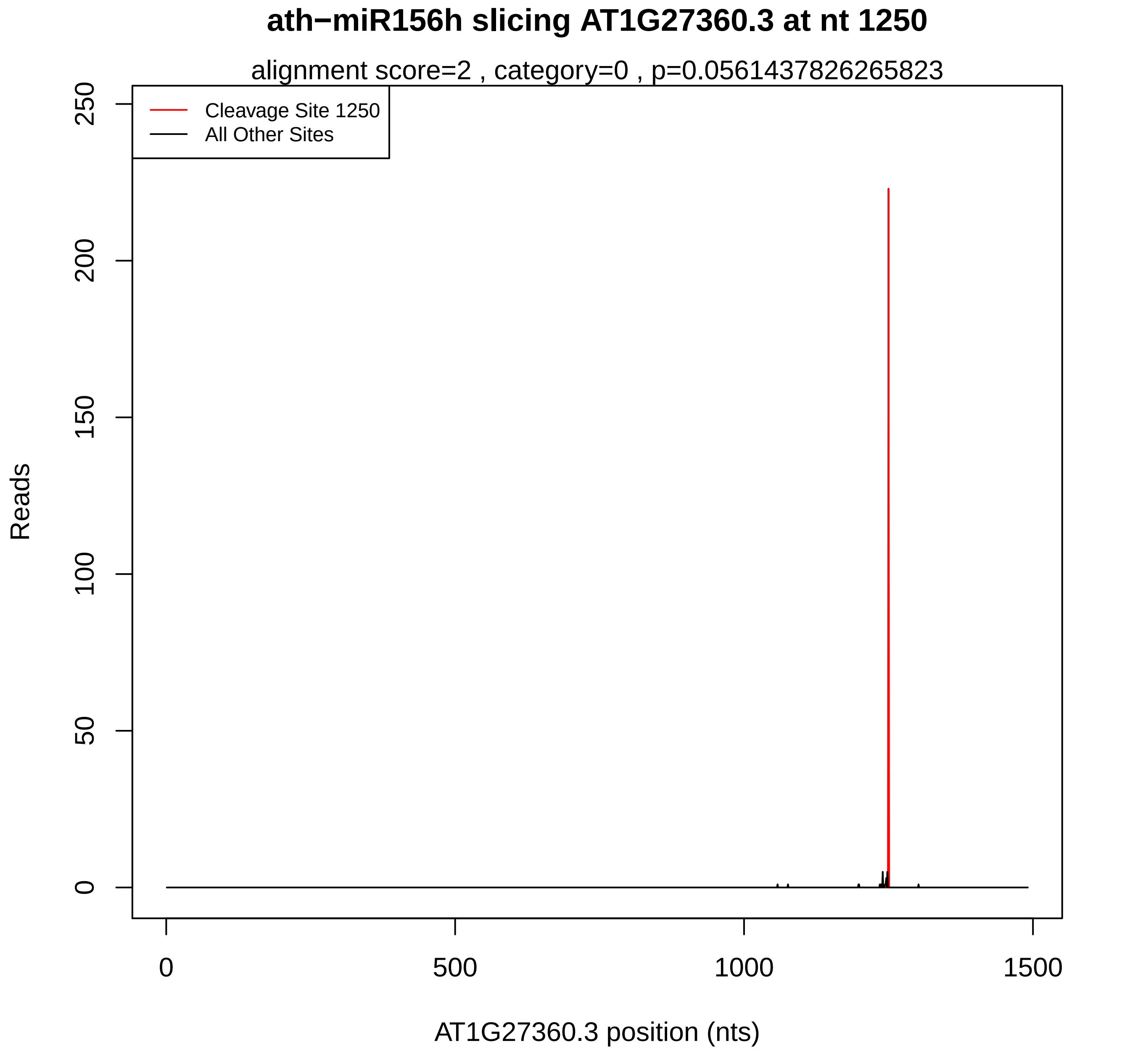

ath−miR156h slicing AT1G27360.3 at nt 1250
alignment score=2 , category=0 , p=0.0561437826265823
Cleavage Site 1250
All Other Sites
Reads
0
50
100
150
200
250
0
500
1000
1500
AT1G27360.3 position (nts)

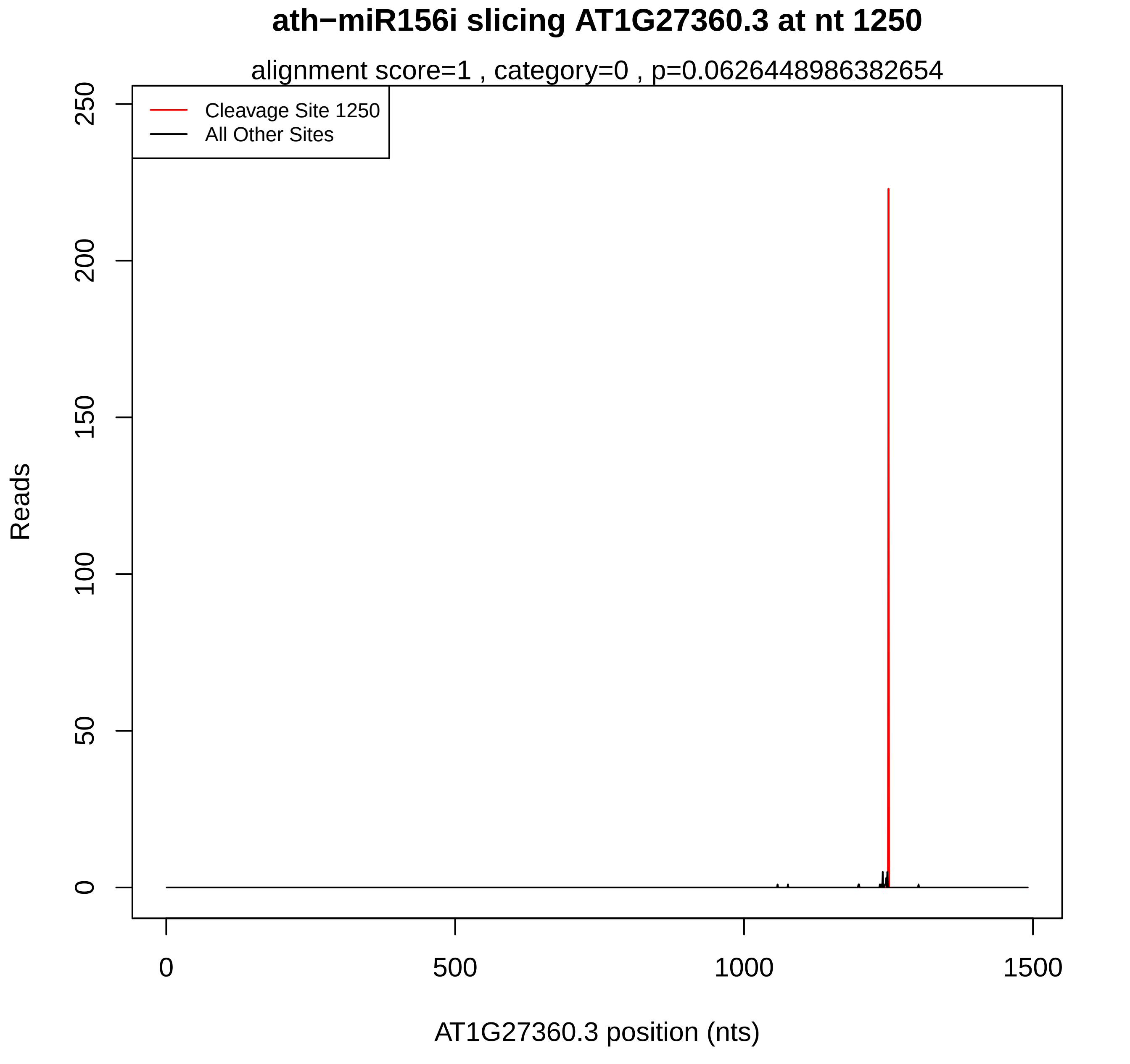
ath−miR156i slicing AT1G27360.3 at nt 1250
alignment score=1 , category=0 , p=0.0626448986382654
Cleavage Site 1250
All Other Sites
Reads
0
50
100
150
200
250
0
500
1000
1500
AT1G27360.3 position (nts)

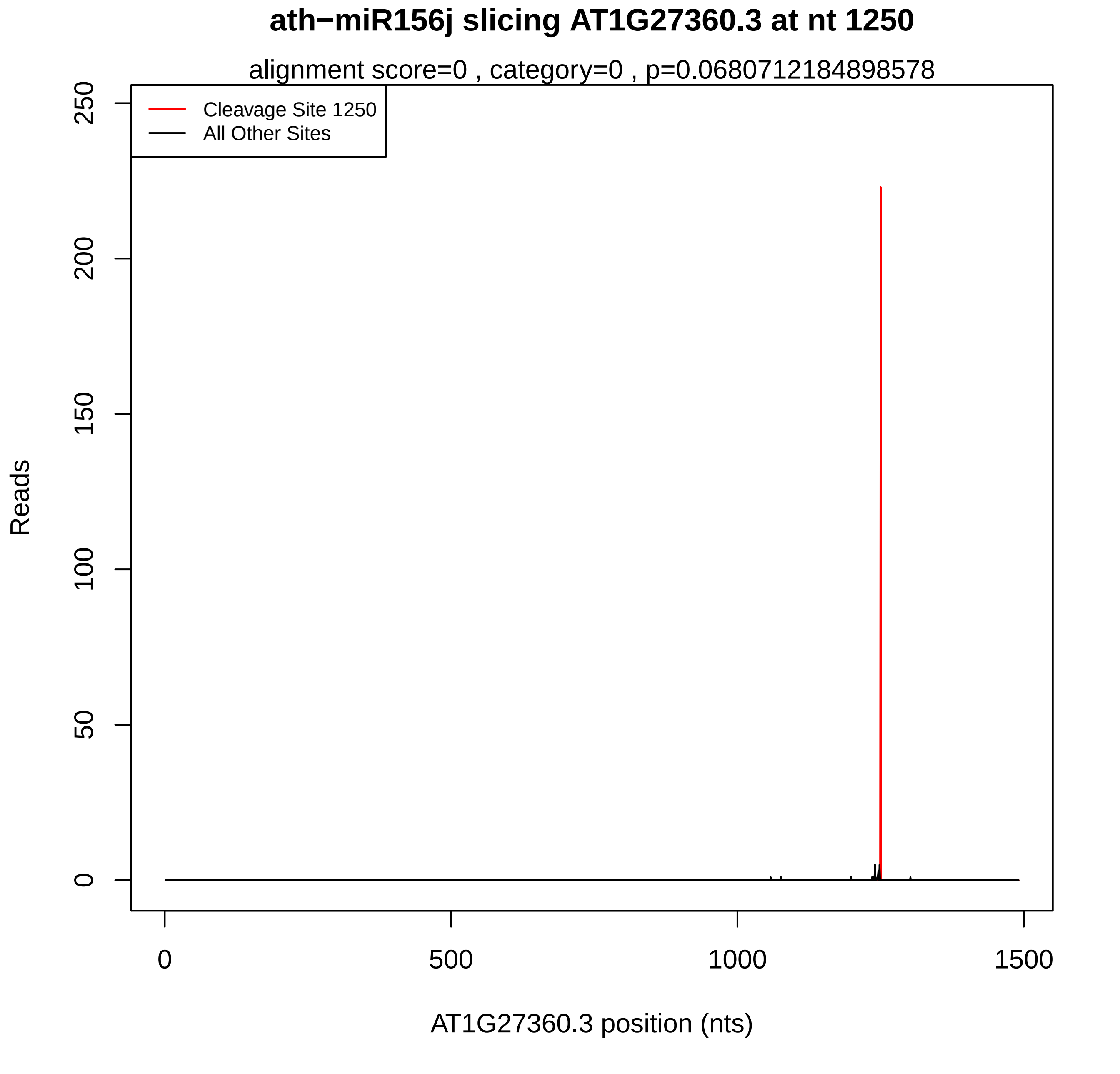
ath−miR156j slicing AT1G27360.3 at nt 1250
alignment score=0 , category=0 , p=0.0680712184898578
Cleavage Site 1250
All Other Sites
Reads
0
50
100
150
200
250
0
500
1000
1500
AT1G27360.3 position (nts)

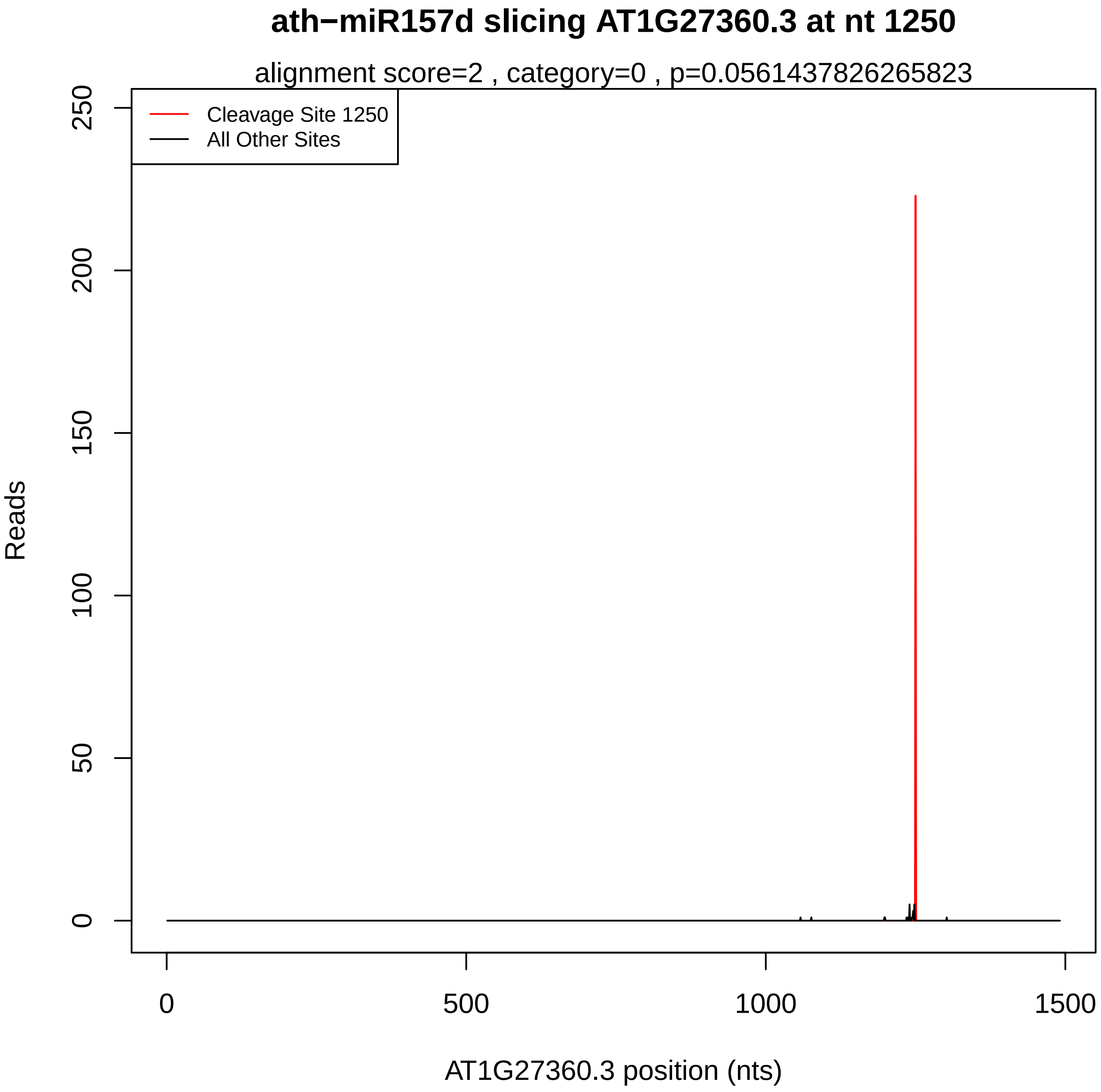

ath−miR157d slicing AT1G27360.3 at nt 1250
alignment score=2 , category=0 , p=0.0561437826265823
Cleavage Site 1250
All Other Sites
Reads
0
50
100
150
200
250
0
500
1000
1500
AT1G27360.3 position (nts)

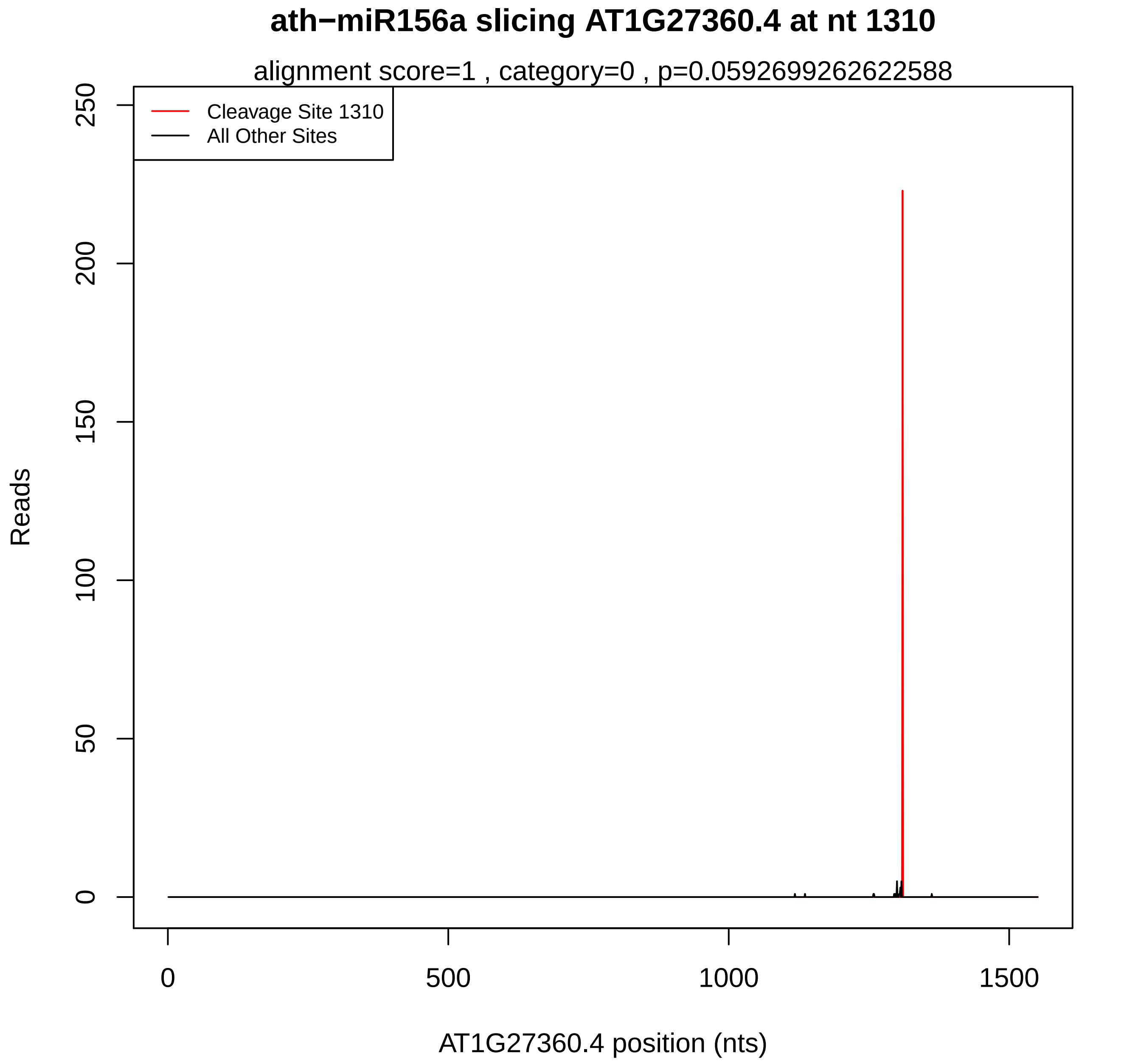
ath−miR156a slicing AT1G27360.4 at nt 1310
alignment score=1 , category=0 , p=0.0592699262622588
Cleavage Site 1310
All Other Sites
Reads
0
50
100
150
200
250
0
500
1000
1500
AT1G27360.4 position (nts)

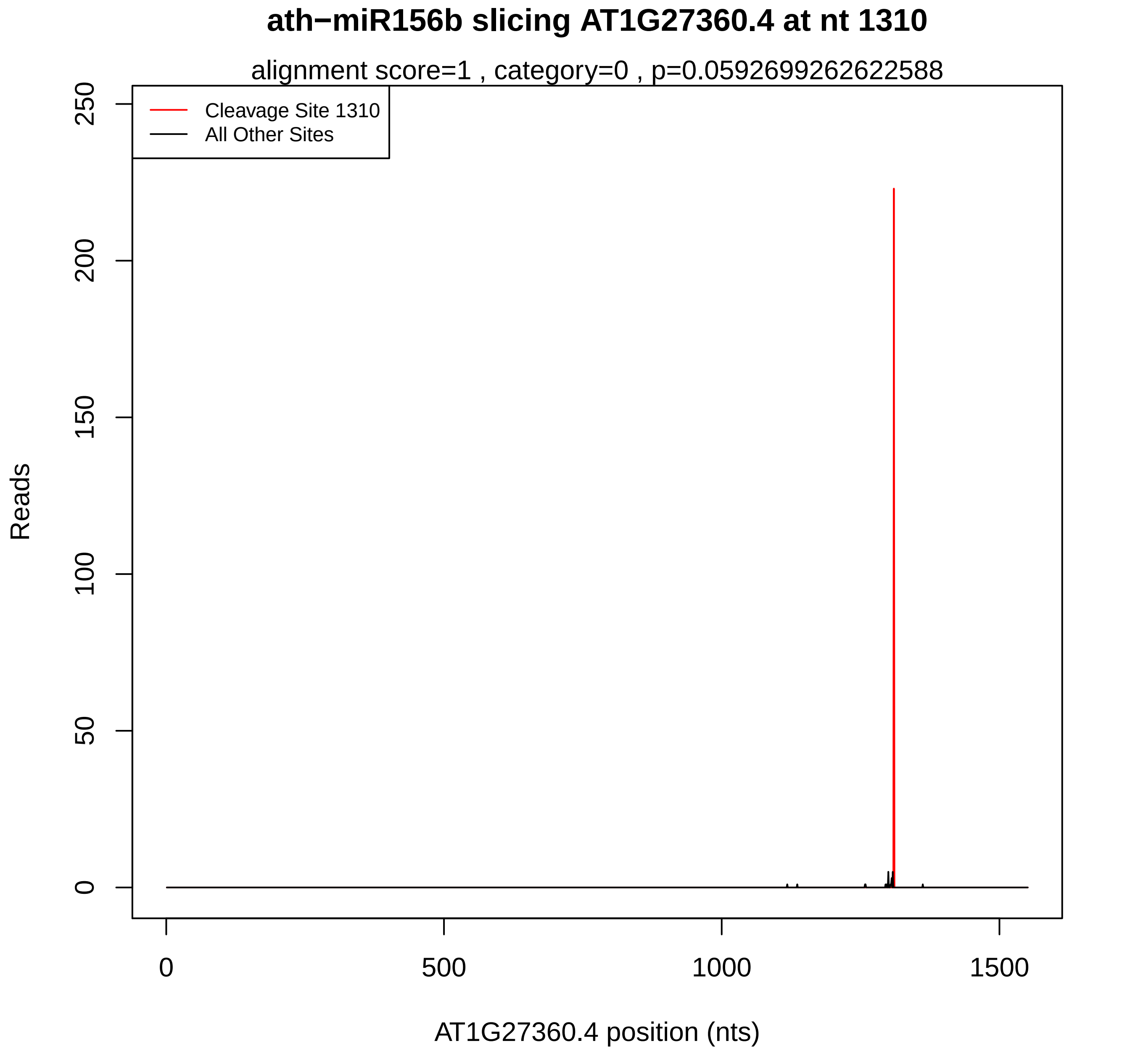
ath−miR156b slicing AT1G27360.4 at nt 1310
alignment score=1 , category=0 , p=0.0592699262622588
Cleavage Site 1310
All Other Sites
Reads
0
50
100
150
200
250
0
500
1000
1500
AT1G27360.4 position (nts)

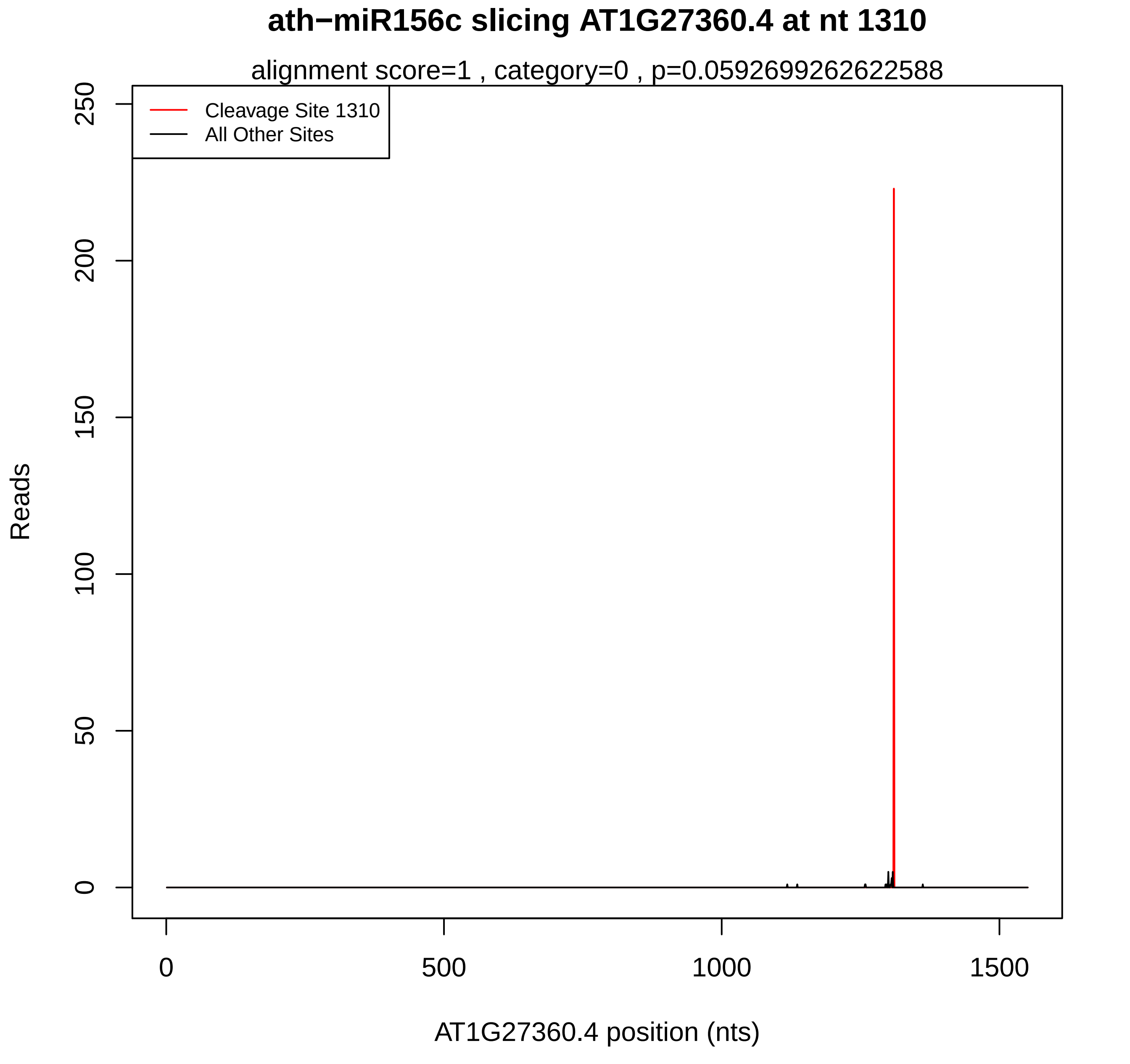

ath−miR156c slicing AT1G27360.4 at nt 1310
alignment score=1 , category=0 , p=0.0592699262622588
Cleavage Site 1310
All Other Sites
Reads
0
50
100
150
200
250
0
500
1000
1500
AT1G27360.4 position (nts)

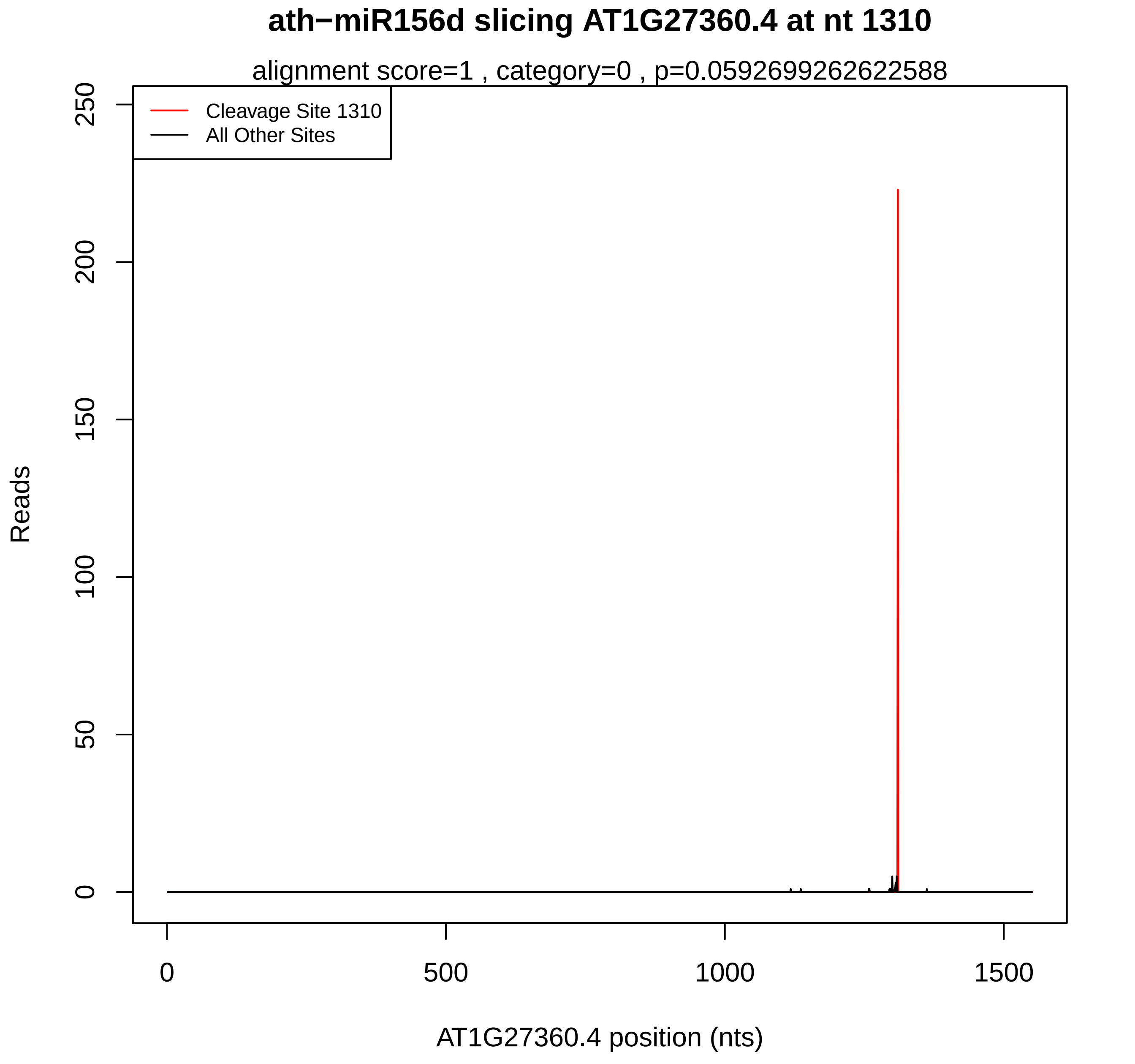
ath−miR156d slicing AT1G27360.4 at nt 1310
alignment score=1 , category=0 , p=0.0592699262622588
Cleavage Site 1310
All Other Sites
Reads
0
50
100
150
200
250
0
500
1000
1500
AT1G27360.4 position (nts)

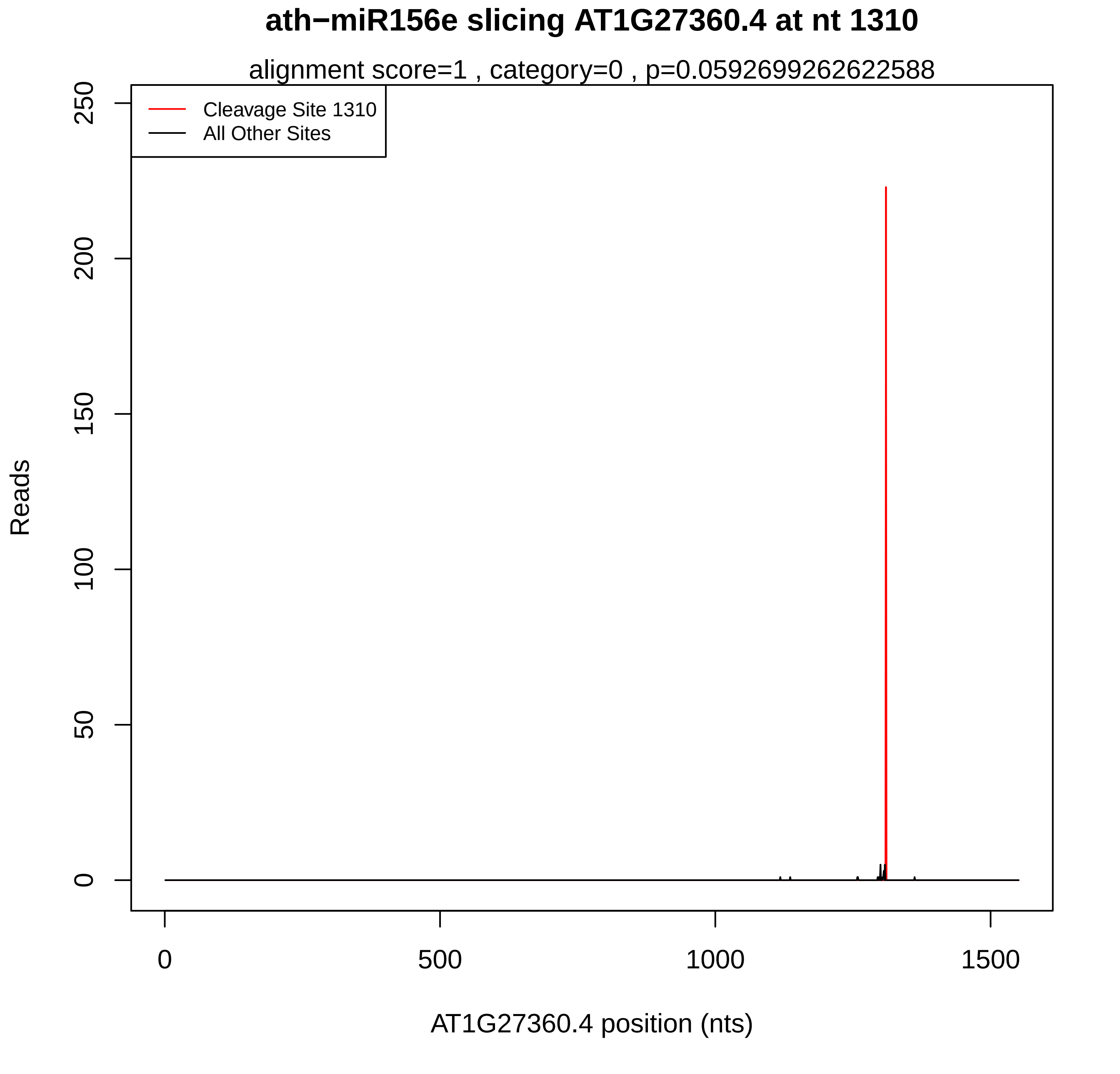
ath−miR156e slicing AT1G27360.4 at nt 1310
alignment score=1 , category=0 , p=0.0592699262622588
Cleavage Site 1310
All Other Sites
Reads
0
50
100
150
200
250
0
500
1000
1500
AT1G27360.4 position (nts)

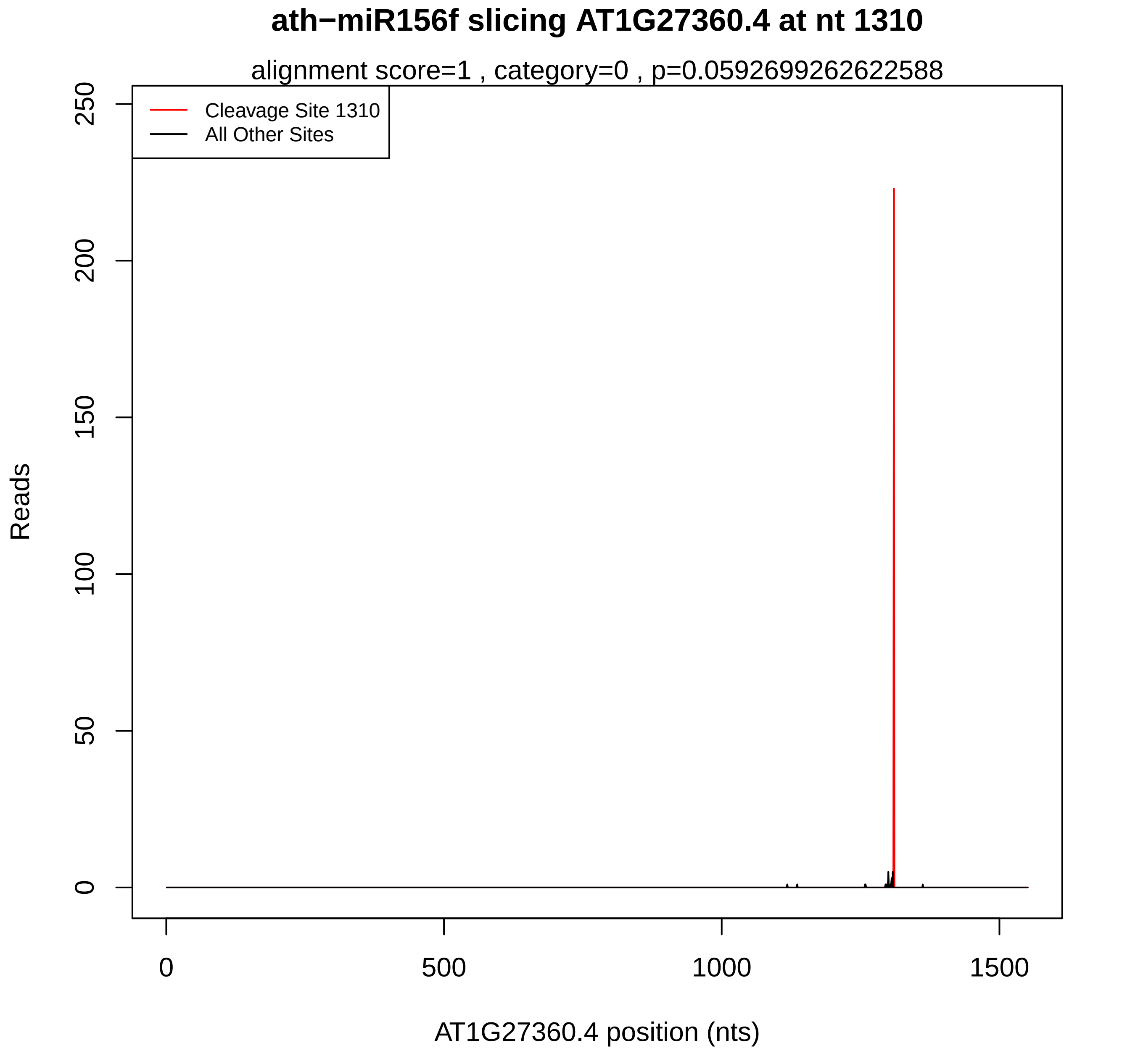
ath−miR156f slicing AT1G27360.4 at nt 1310
alignment score=1 , category=0 , p=0.0592699262622588
Cleavage Site 1310
All Other Sites
Reads
0
50
100
150
200
250
0
500
1000
1500
AT1G27360.4 position (nts)

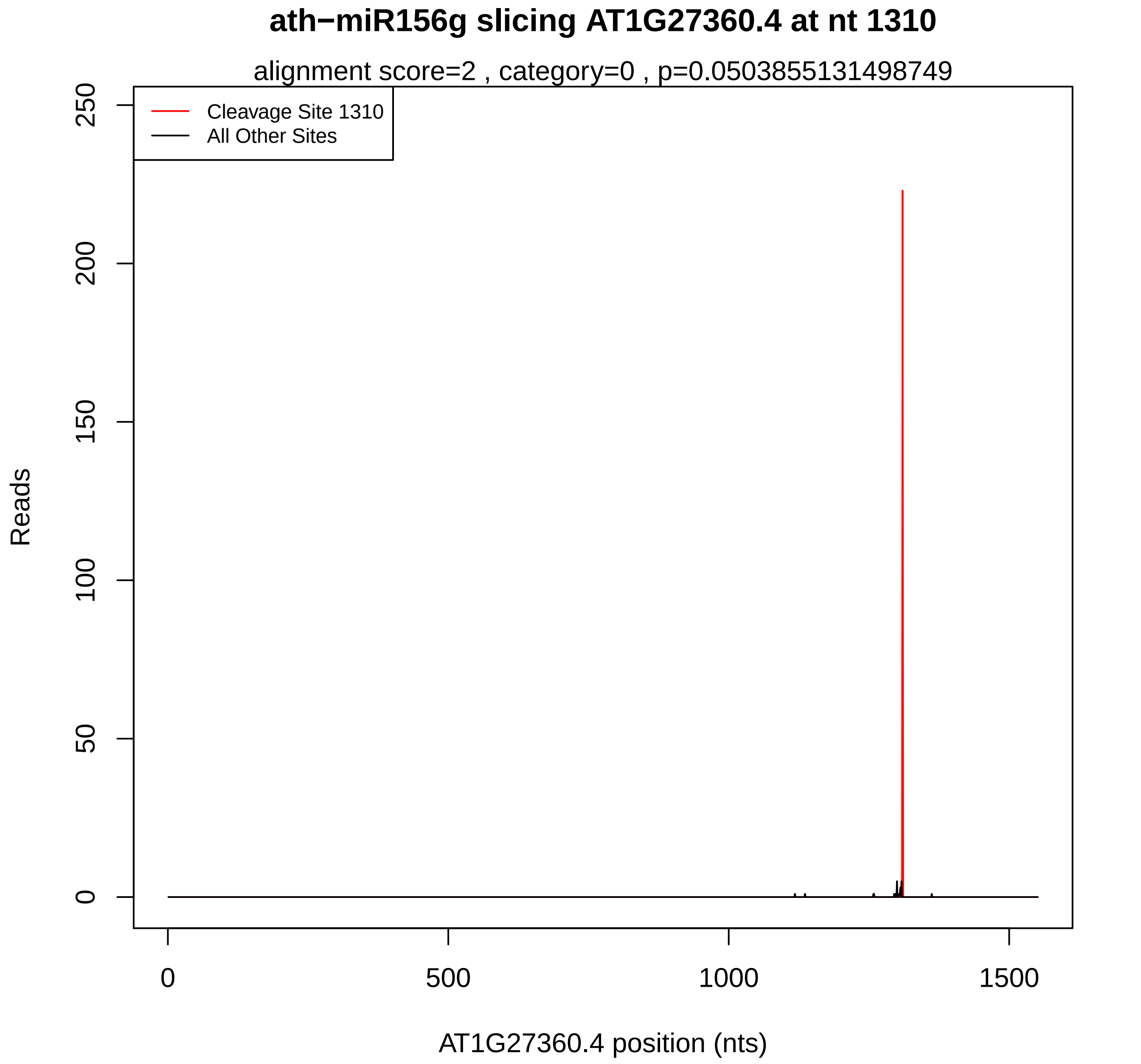

ath−miR156g slicing AT1G27360.4 at nt 1310
alignment score=2 , category=0 , p=0.0503855131498749
Cleavage Site 1310
All Other Sites
Reads
0
50
100
150
200
250
0
500
1000
1500
AT1G27360.4 position (nts)

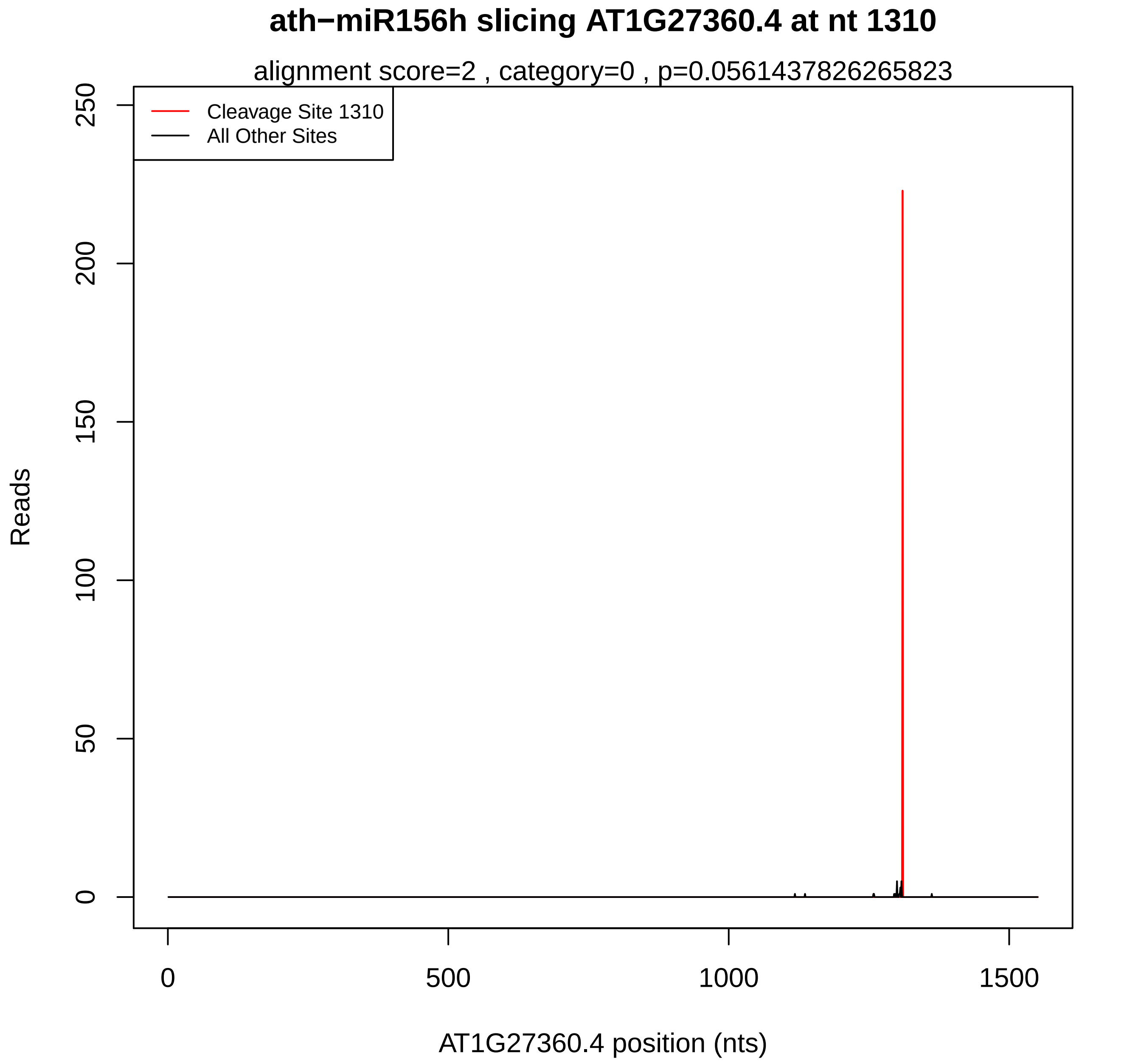
ath−miR156h slicing AT1G27360.4 at nt 1310
alignment score=2 , category=0 , p=0.0561437826265823
Cleavage Site 1310
All Other Sites
Reads
0
50
100
150
200
250
0
500
1000
1500
AT1G27360.4 position (nts)

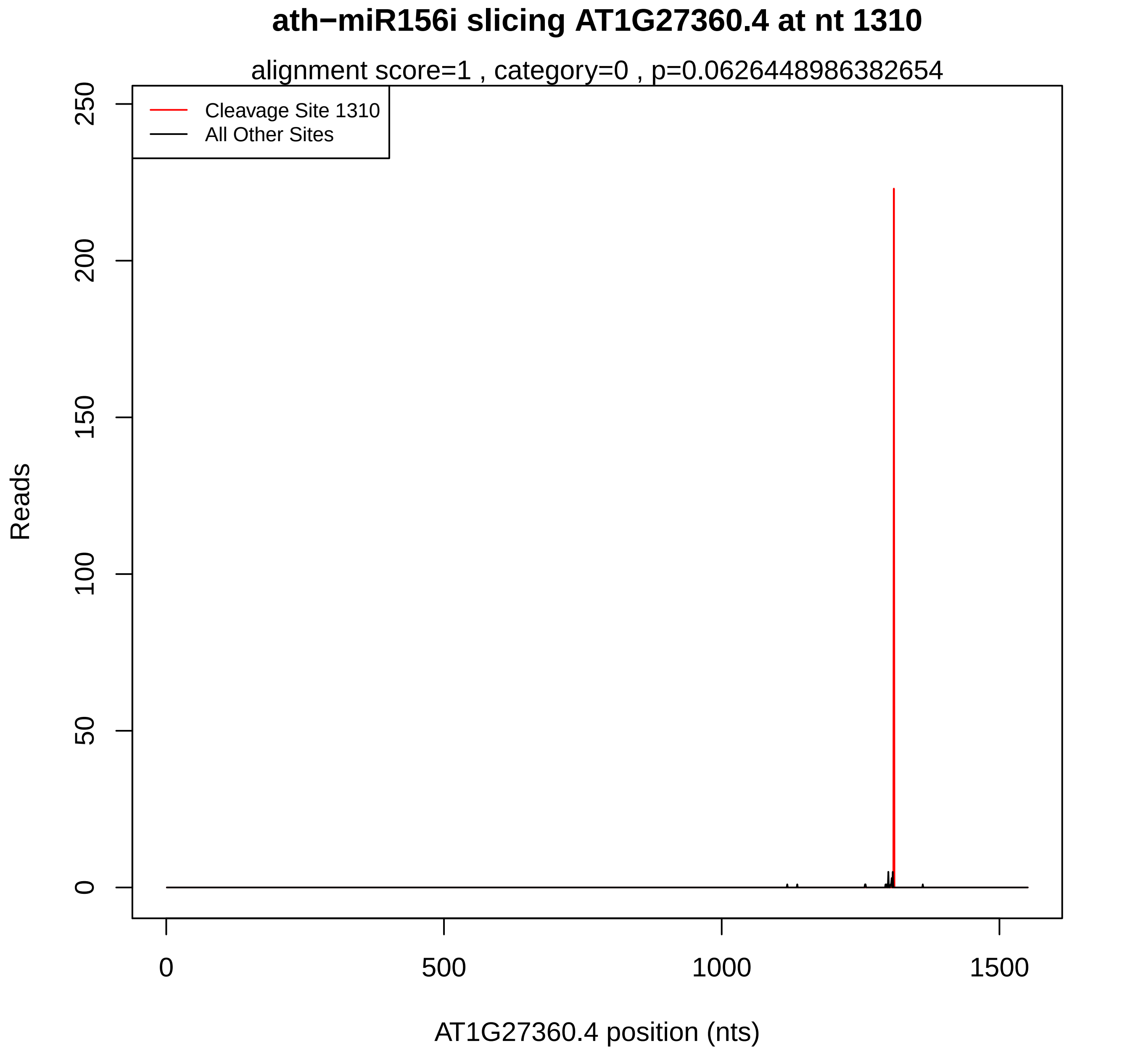
ath−miR156i slicing AT1G27360.4 at nt 1310
alignment score=1 , category=0 , p=0.0626448986382654
Cleavage Site 1310
All Other Sites
Reads
0
50
100
150
200
250
0
500
1000
1500
AT1G27360.4 position (nts)

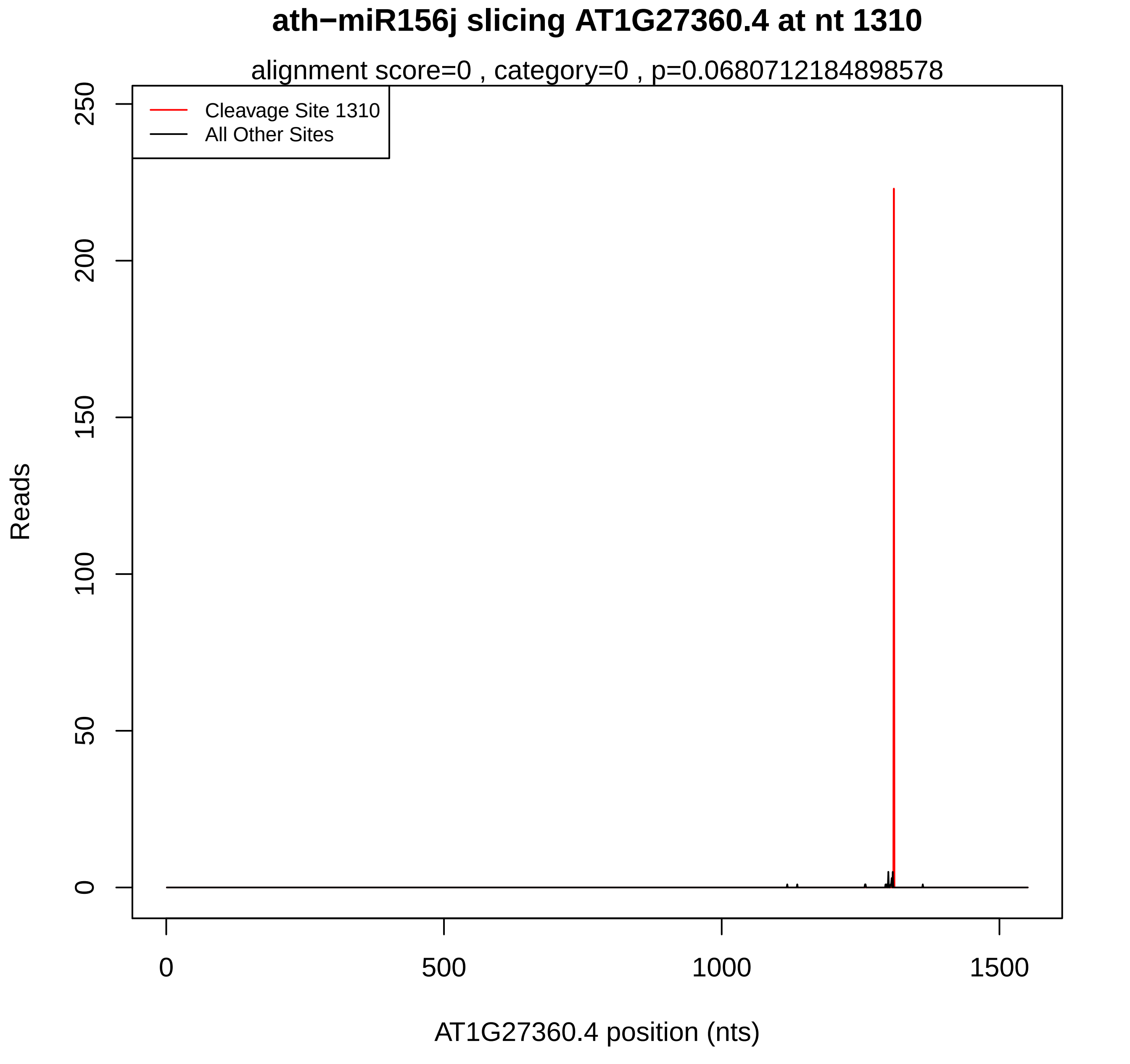

ath−miR156j slicing AT1G27360.4 at nt 1310
alignment score=0 , category=0 , p=0.0680712184898578
Cleavage Site 1310
All Other Sites
Reads
0
50
100
150
200
250
0
500
1000
1500
AT1G27360.4 position (nts)

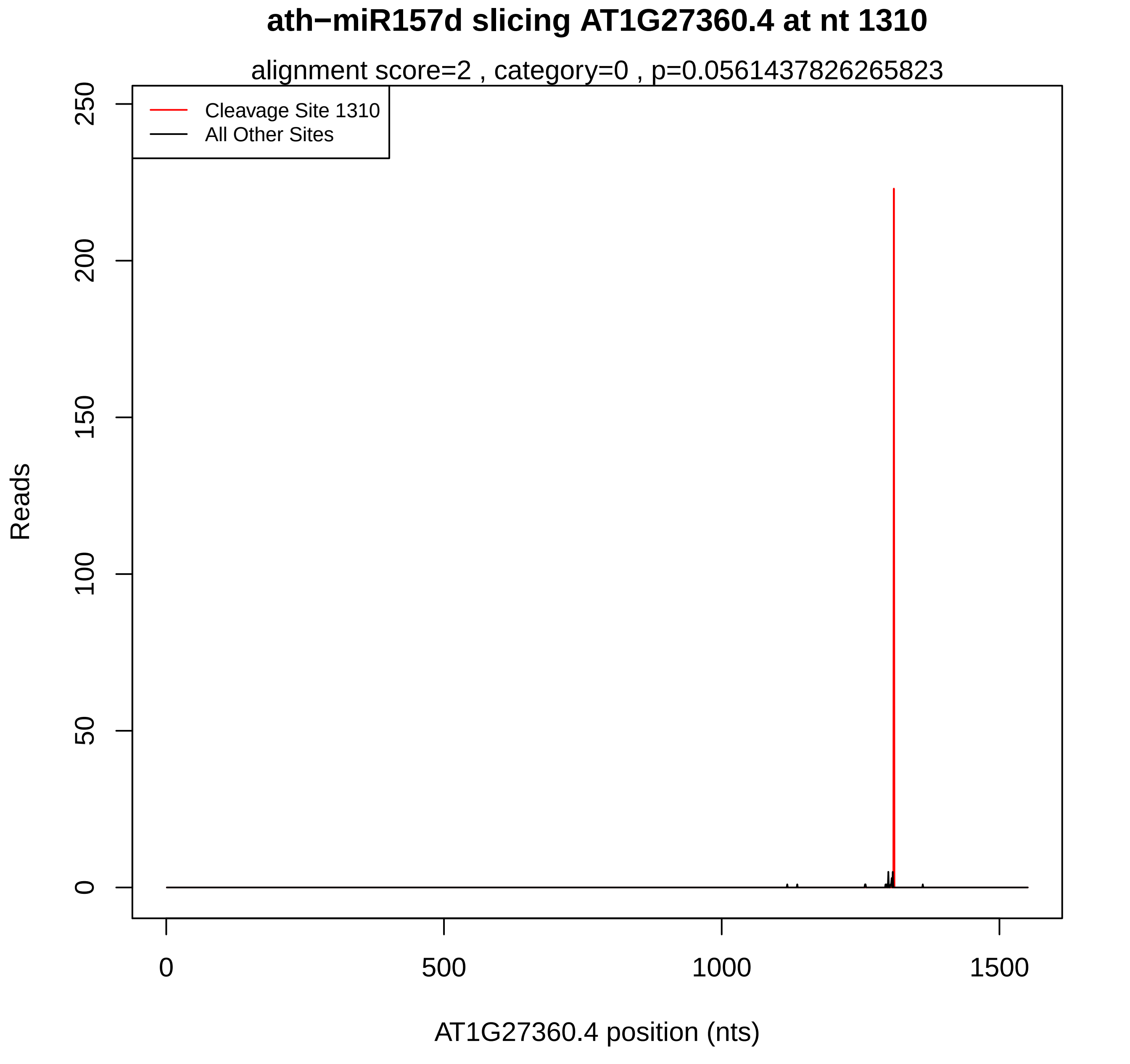
ath−miR157d slicing AT1G27360.4 at nt 1310
alignment score=2 , category=0 , p=0.0561437826265823
Cleavage Site 1310
All Other Sites
Reads
0
50
100
150
200
250
0
500
1000
1500
AT1G27360.4 position (nts)

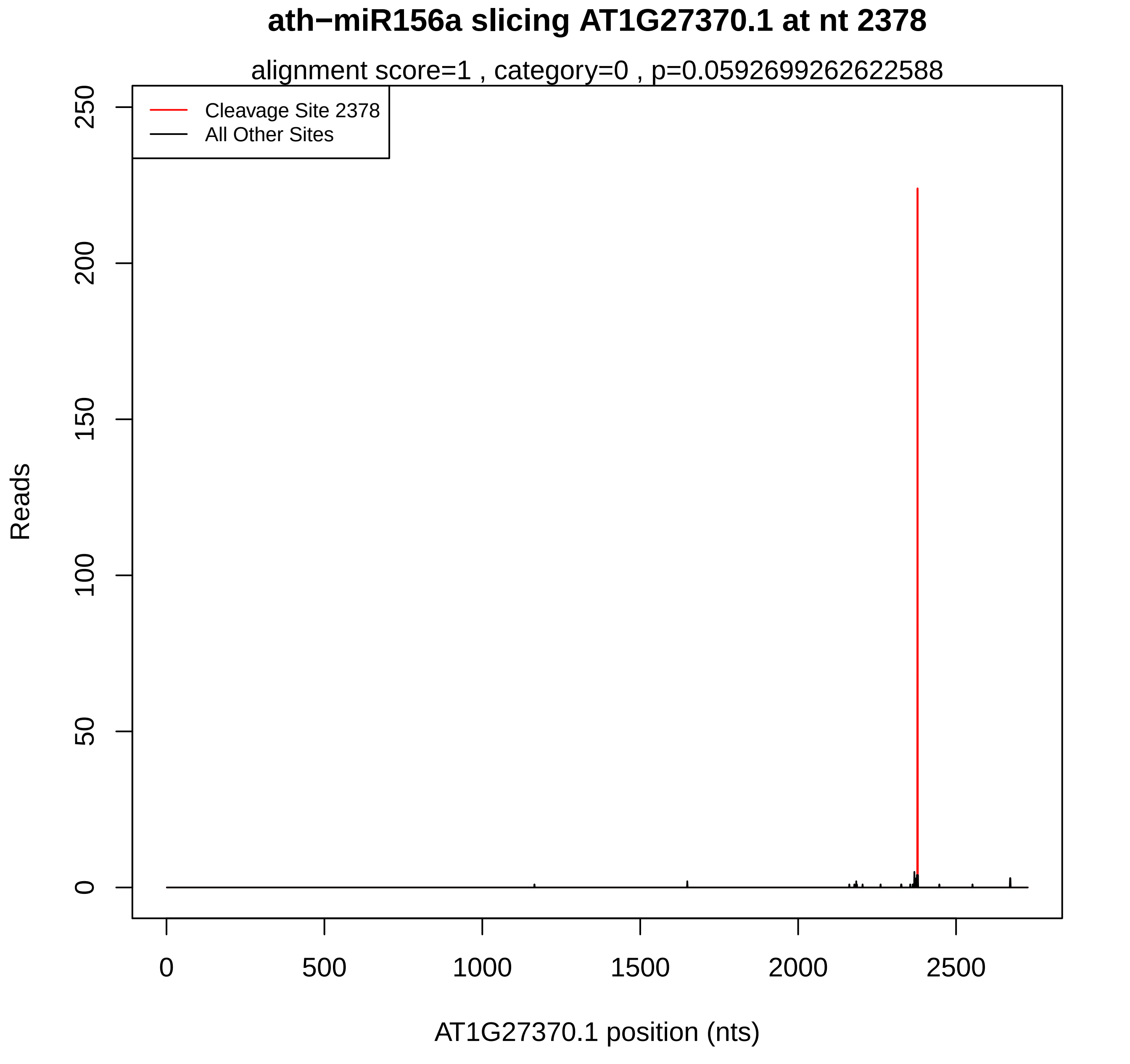

ath−miR156a slicing AT1G27370.1 at nt 2378
alignment score=1 , category=0 , p=0.0592699262622588
Cleavage Site 2378
All Other Sites
Reads
0
50
100
150
200
250
0
500
1000
1500
2000
2500
AT1G27370.1 position (nts)

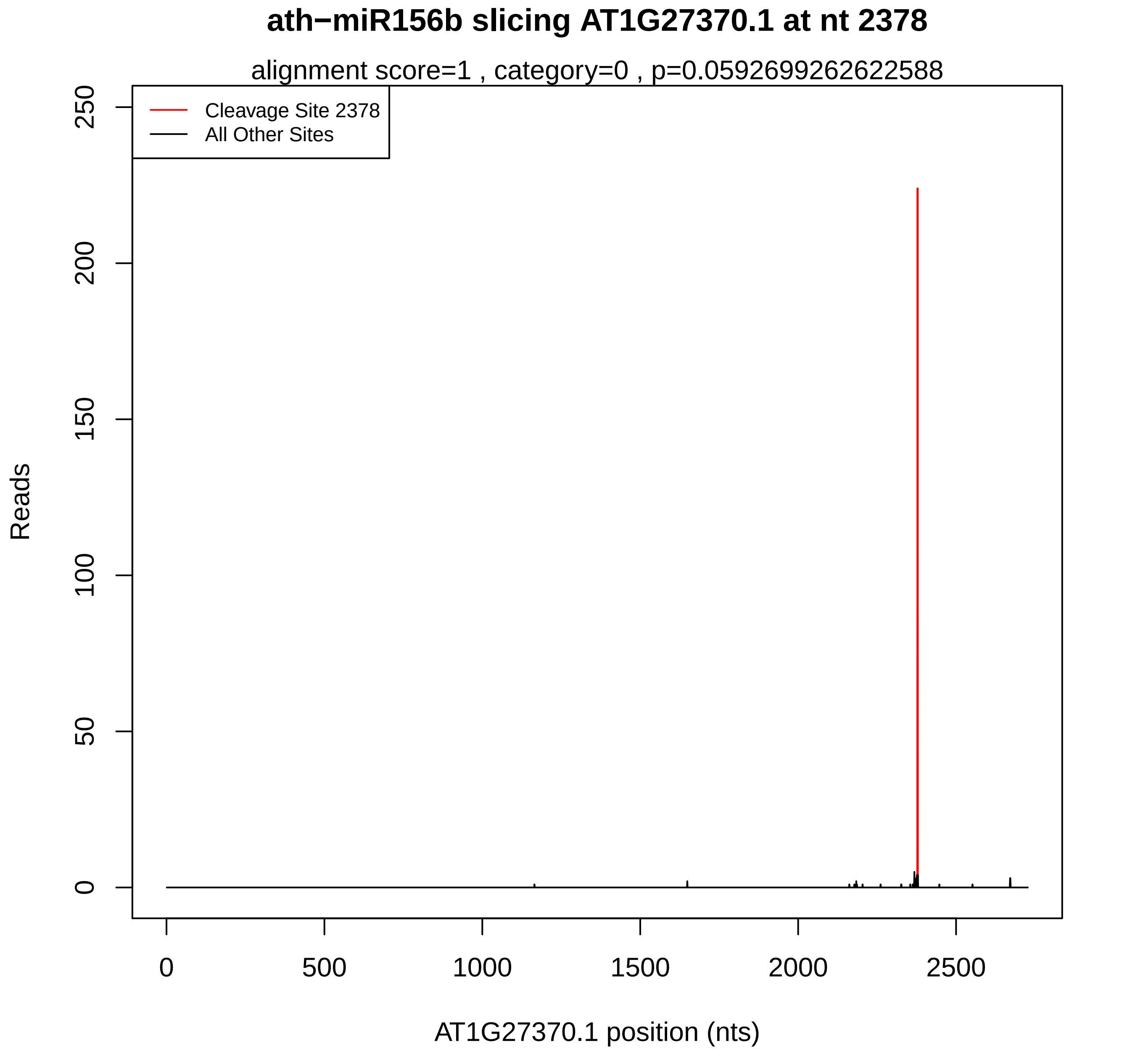

ath−miR156b slicing AT1G27370.1 at nt 2378
alignment score=1 , category=0 , p=0.0592699262622588
Cleavage Site 2378
All Other Sites
Reads
0
50
100
150
200
250
0
500
1000
1500
2000
2500
AT1G27370.1 position (nts)

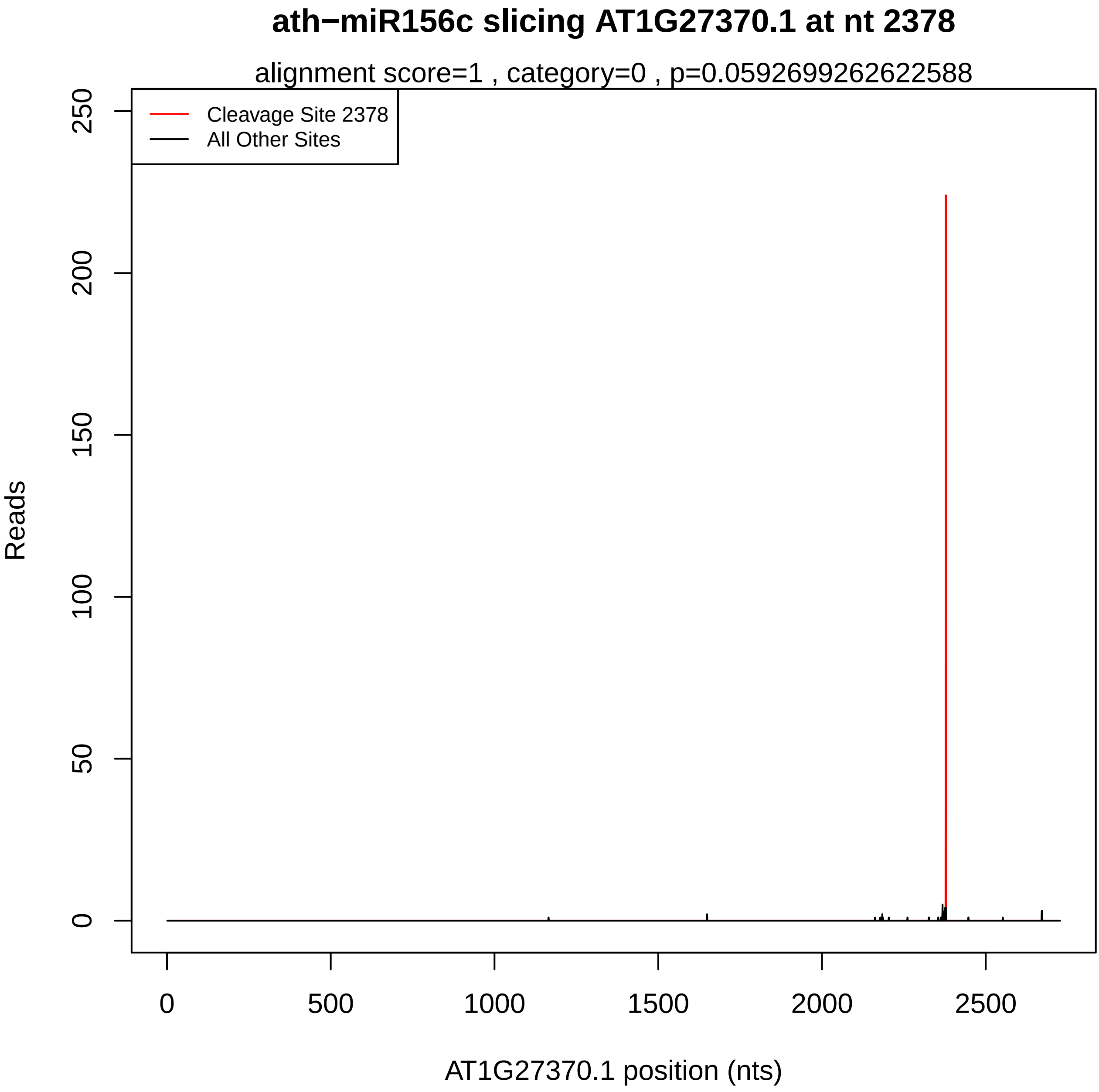
ath−miR156c slicing AT1G27370.1 at nt 2378
alignment score=1 , category=0 , p=0.0592699262622588
Cleavage Site 2378
All Other Sites
Reads
0
50
100
150
200
250
0
500
1000
1500
2000
2500
AT1G27370.1 position (nts)

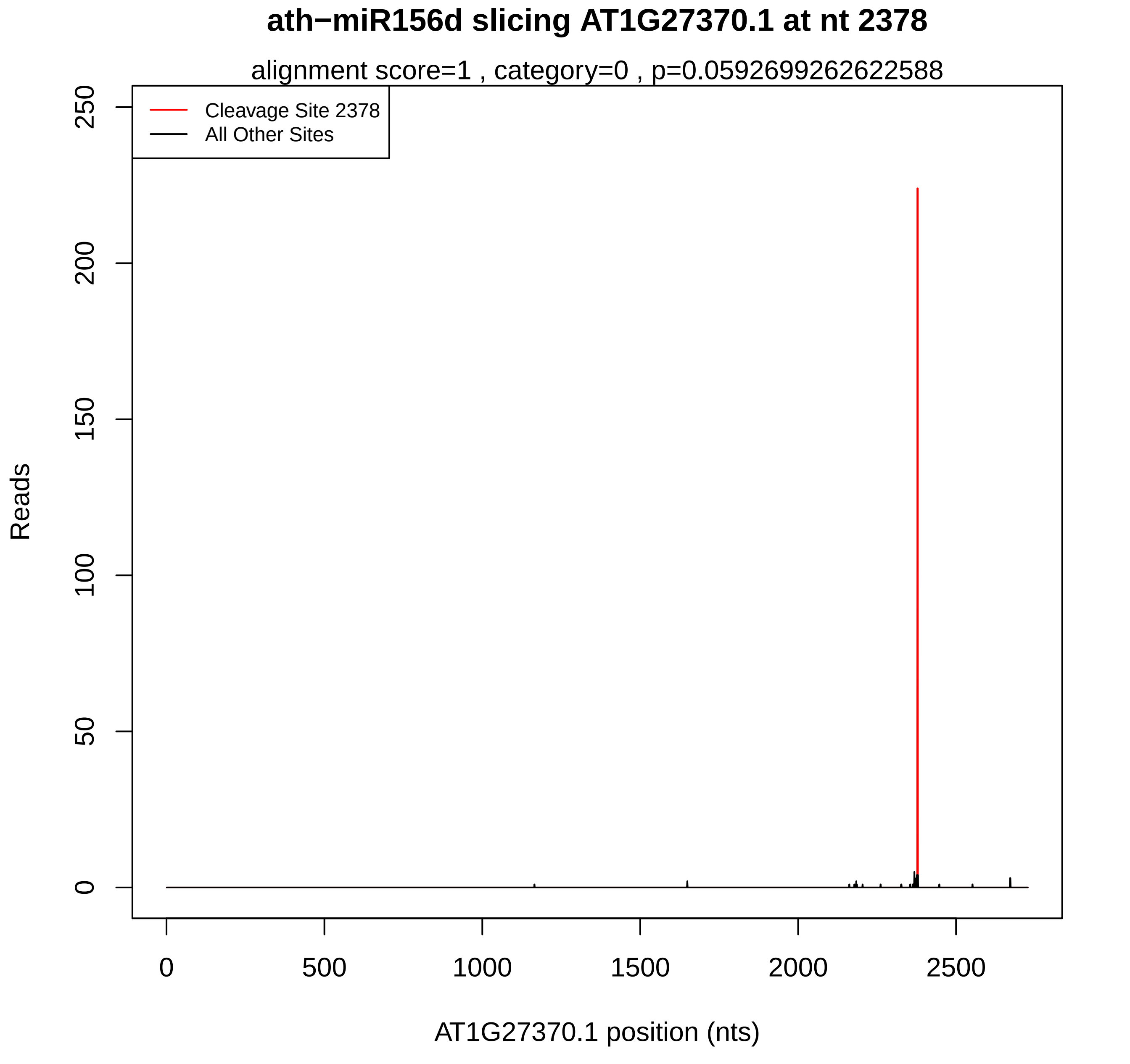
ath−miR156d slicing AT1G27370.1 at nt 2378
alignment score=1 , category=0 , p=0.0592699262622588
Cleavage Site 2378
All Other Sites
Reads
0
50
100
150
200
250
0
500
1000
1500
2000
2500
AT1G27370.1 position (nts)

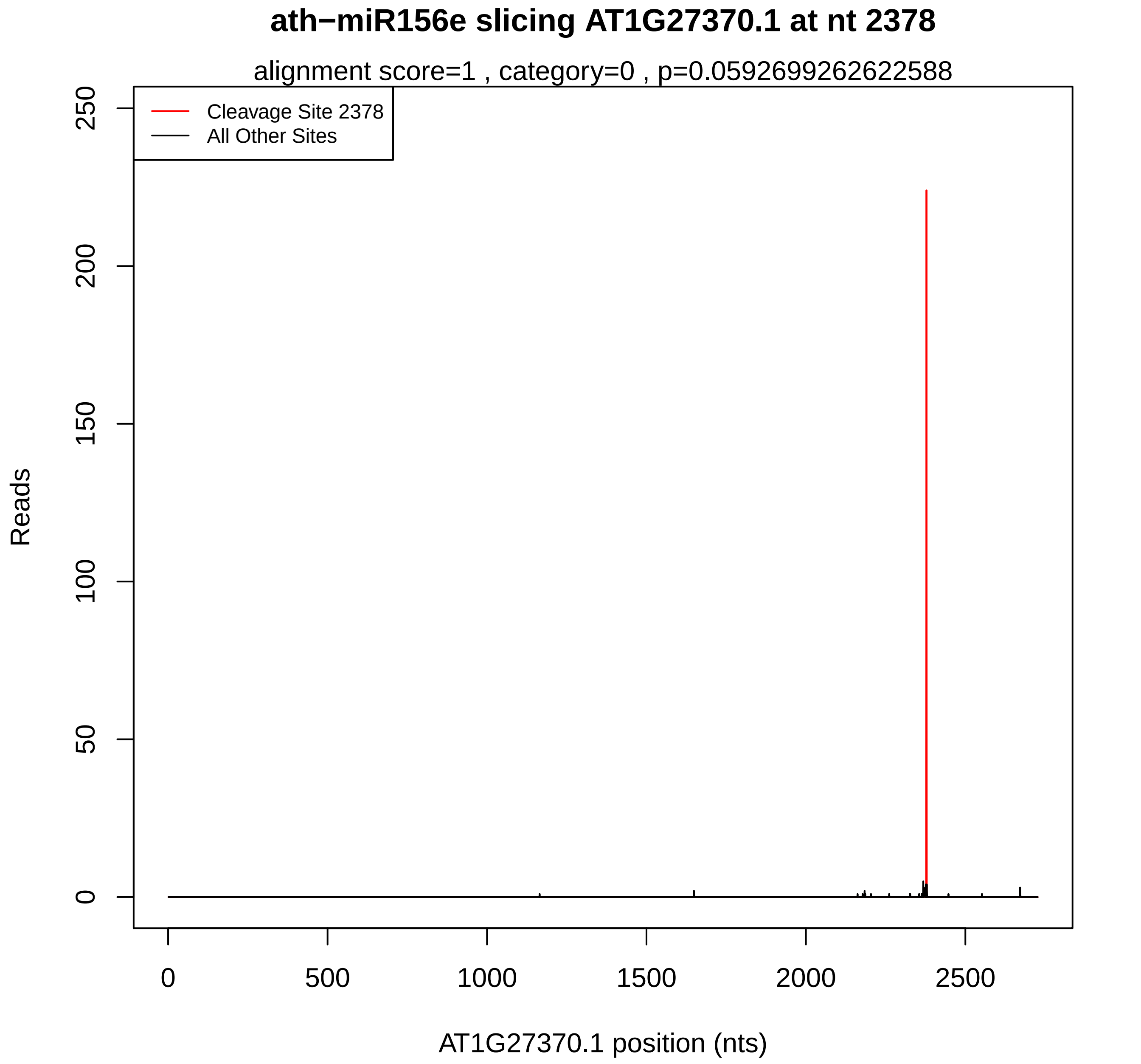
ath−miR156e slicing AT1G27370.1 at nt 2378
alignment score=1 , category=0 , p=0.0592699262622588
Cleavage Site 2378
All Other Sites
Reads
0
50
100
150
200
250
0
500
1000
1500
2000
2500
AT1G27370.1 position (nts)

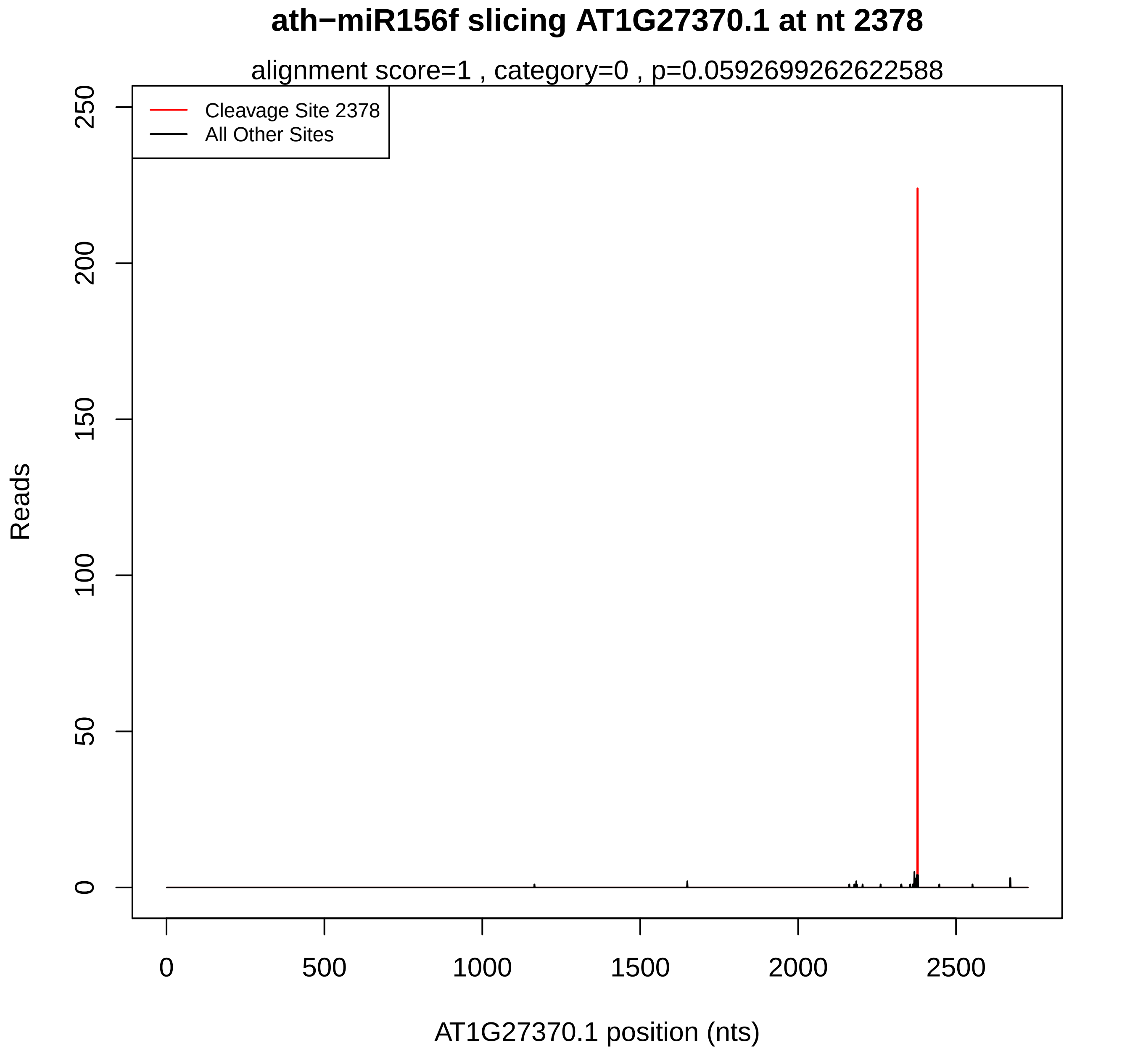

ath−miR156f slicing AT1G27370.1 at nt 2378
alignment score=1 , category=0 , p=0.0592699262622588
Cleavage Site 2378
All Other Sites
Reads
0
50
100
150
200
250
0
500
1000
1500
2000
2500
AT1G27370.1 position (nts)

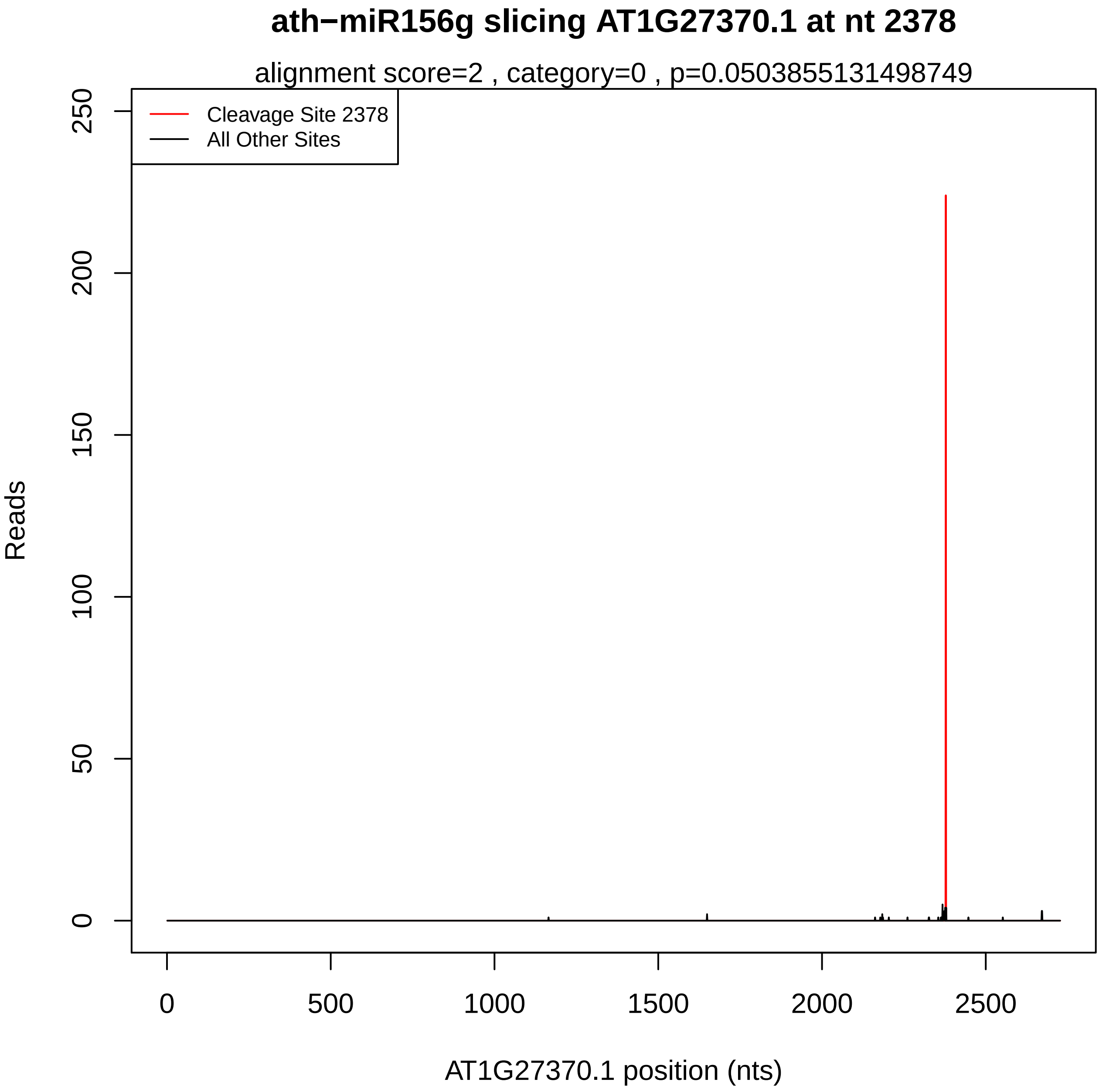
ath−miR156g slicing AT1G27370.1 at nt 2378
alignment score=2 , category=0 , p=0.0503855131498749
Cleavage Site 2378
All Other Sites
Reads
0
50
100
150
200
250
0
500
1000
1500
2000
2500
AT1G27370.1 position (nts)

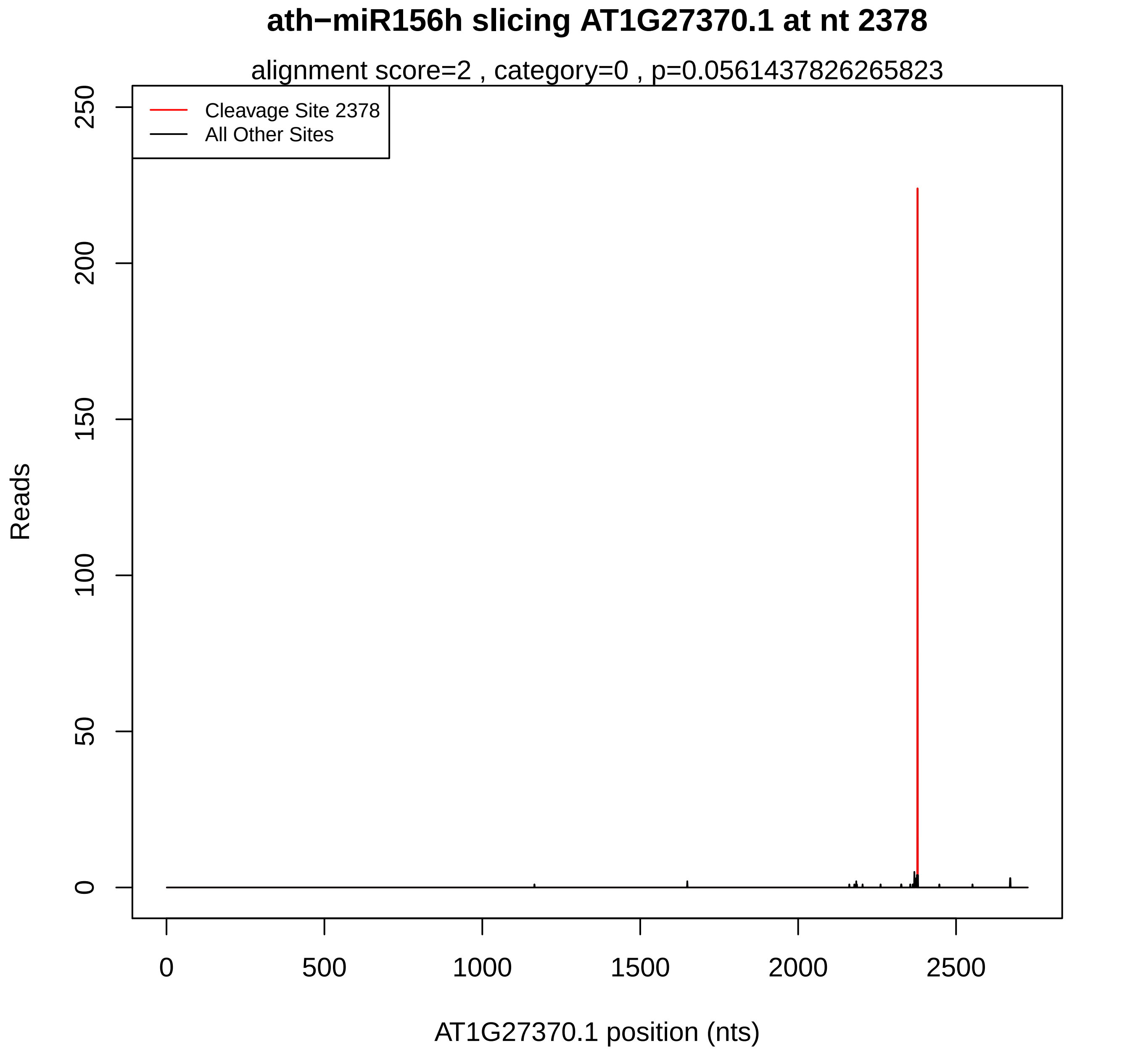

ath−miR156h slicing AT1G27370.1 at nt 2378
alignment score=2 , category=0 , p=0.0561437826265823
Cleavage Site 2378
All Other Sites
Reads
0
50
100
150
200
250
0
500
1000
1500
2000
2500
AT1G27370.1 position (nts)

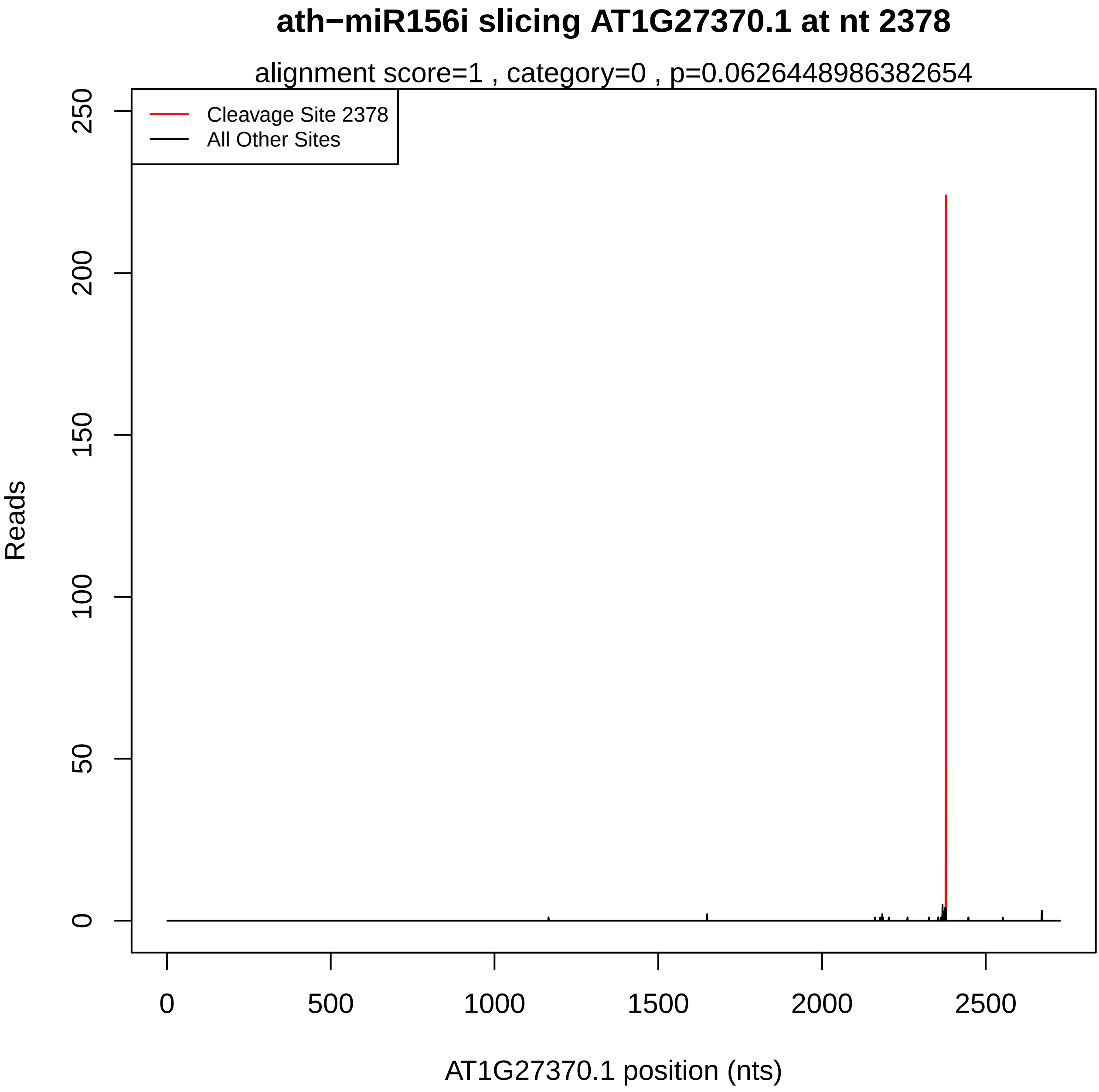
ath−miR156i slicing AT1G27370.1 at nt 2378
alignment score=1 , category=0 , p=0.0626448986382654
Cleavage Site 2378
All Other Sites
Reads
0
50
100
150
200
250
0
500
1000
1500
2000
2500
AT1G27370.1 position (nts)

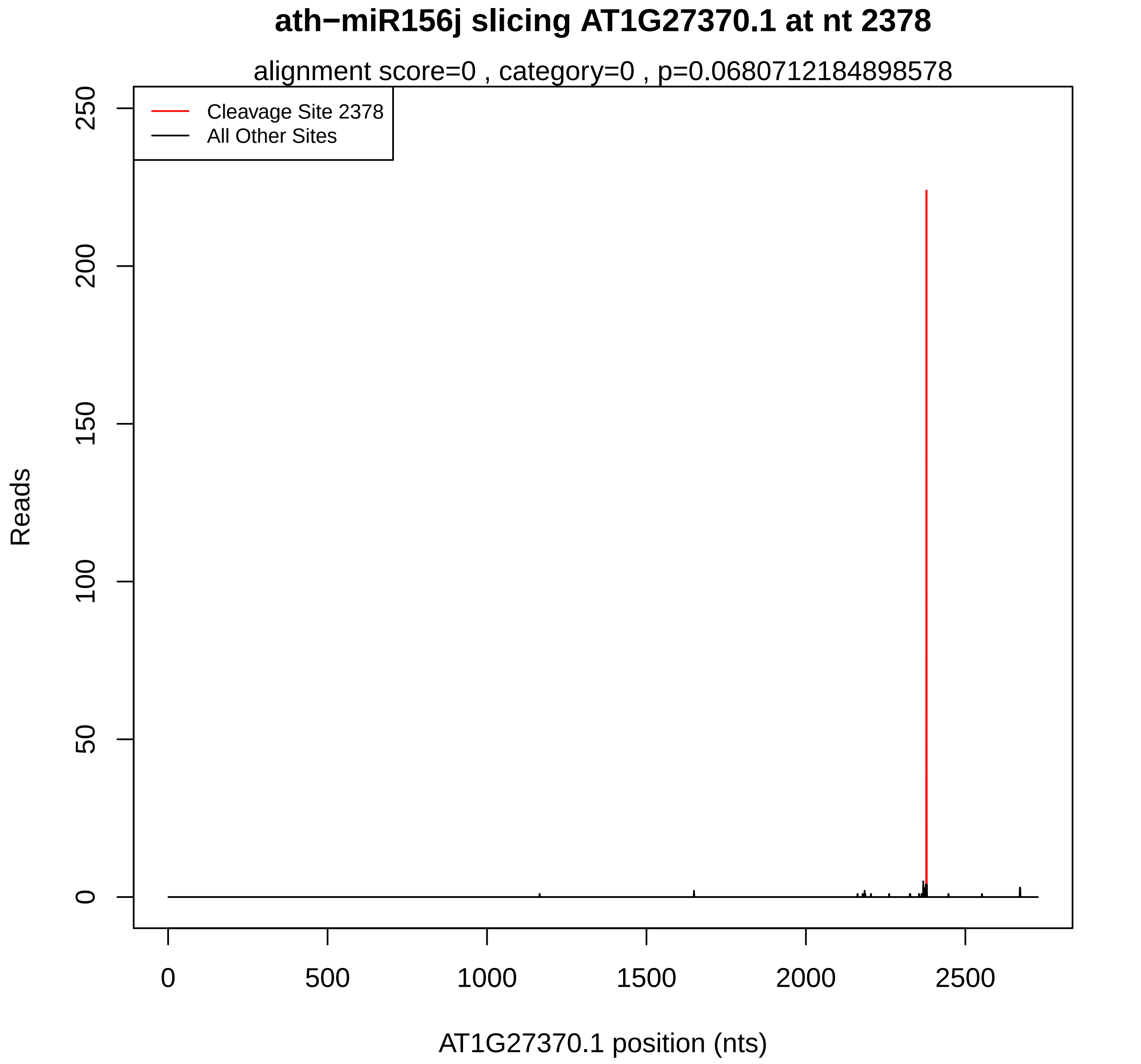

ath−miR156j slicing AT1G27370.1 at nt 2378
alignment score=0 , category=0 , p=0.0680712184898578
Cleavage Site 2378
All Other Sites
Reads
0
50
100
150
200
250
0
500
1000
1500
2000
2500
AT1G27370.1 position (nts)

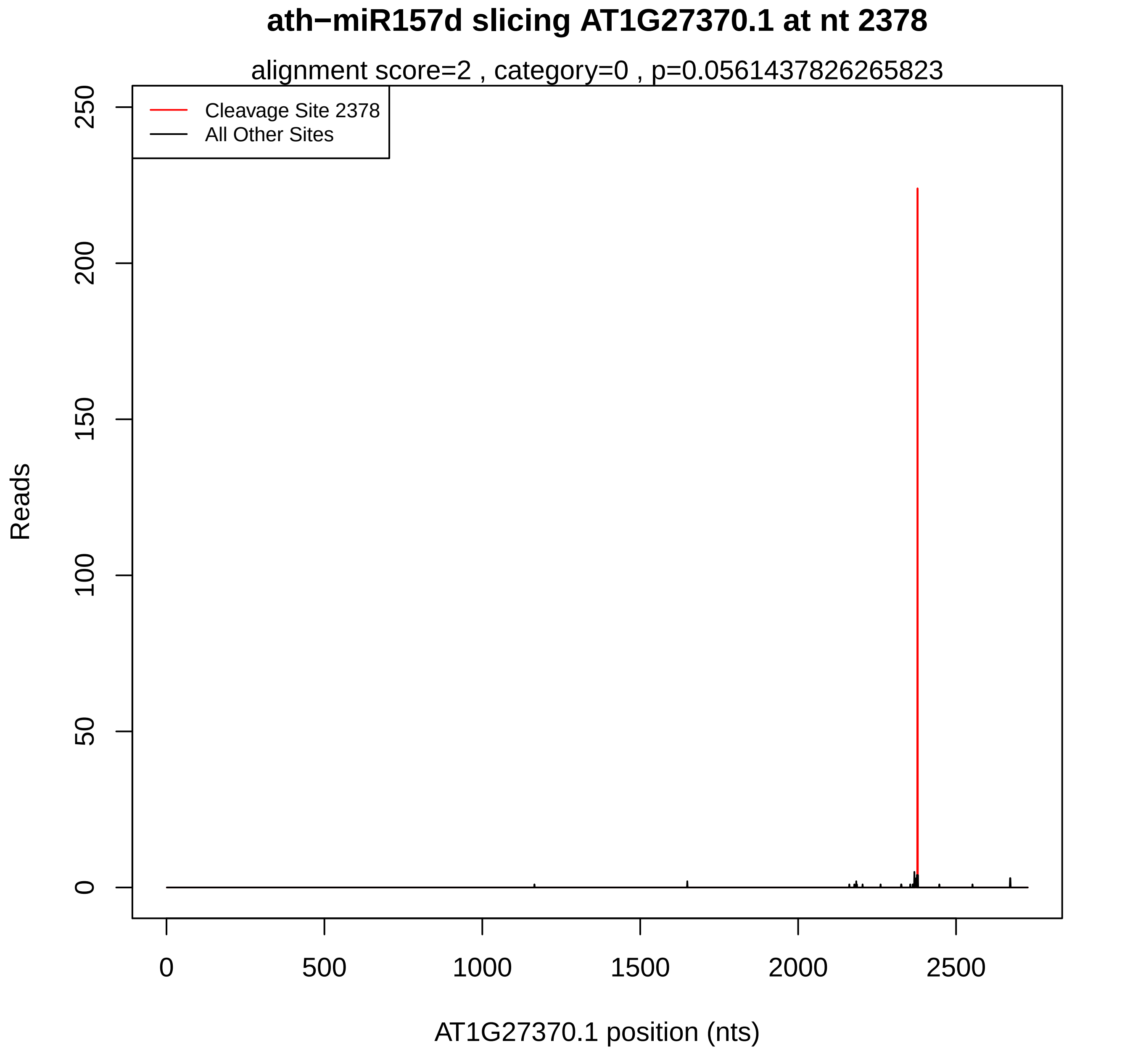
ath−miR157d slicing AT1G27370.1 at nt 2378
alignment score=2 , category=0 , p=0.0561437826265823
Cleavage Site 2378
All Other Sites
Reads
0
50
100
150
200
250
0
500
1000
1500
2000
2500
AT1G27370.1 position (nts)

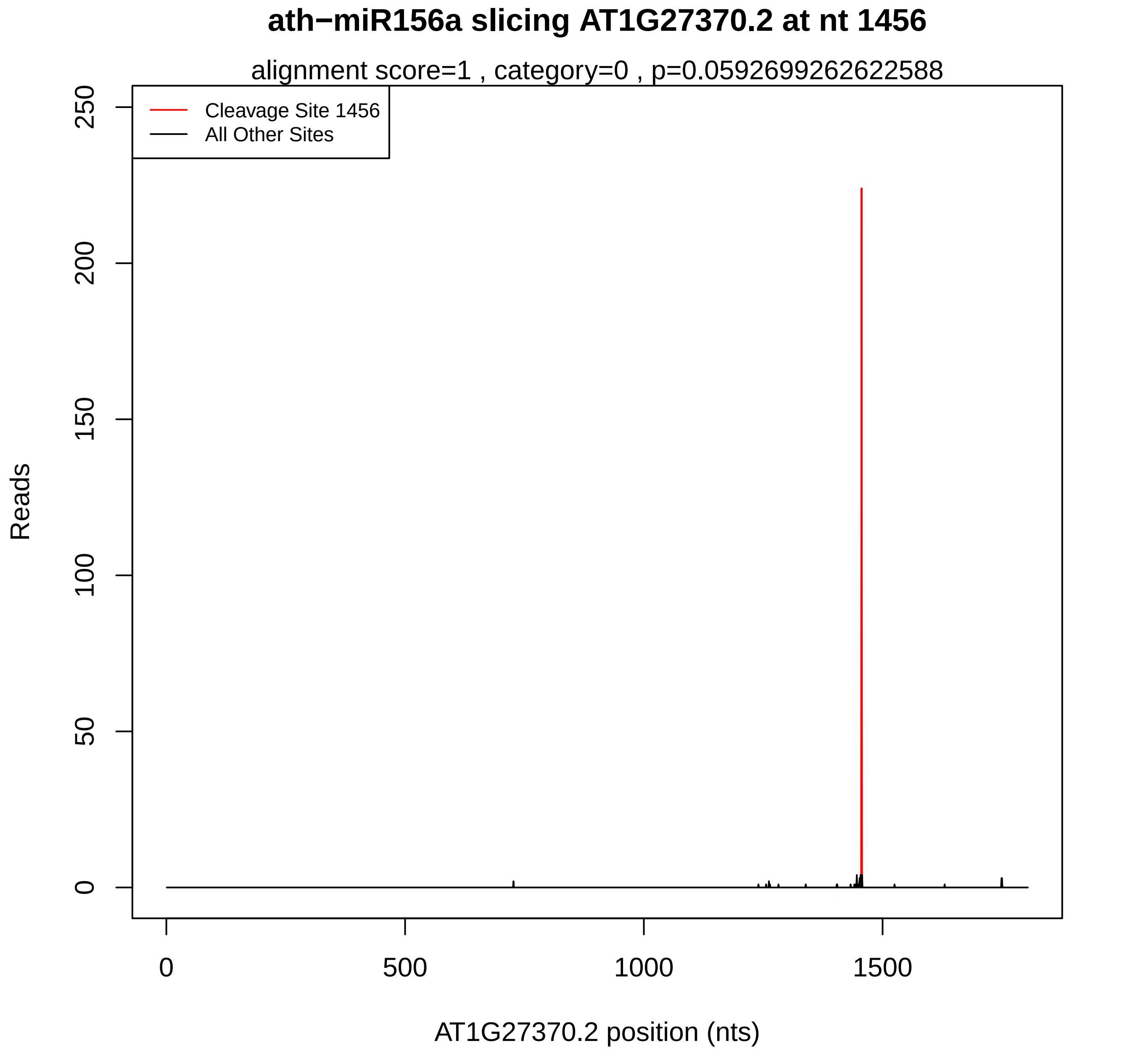

ath−miR156a slicing AT1G27370.2 at nt 1456
alignment score=1 , category=0 , p=0.0592699262622588
Cleavage Site 1456
All Other Sites
Reads
0
50
100
150
200
250
0
500
1000
1500
AT1G27370.2 position (nts)

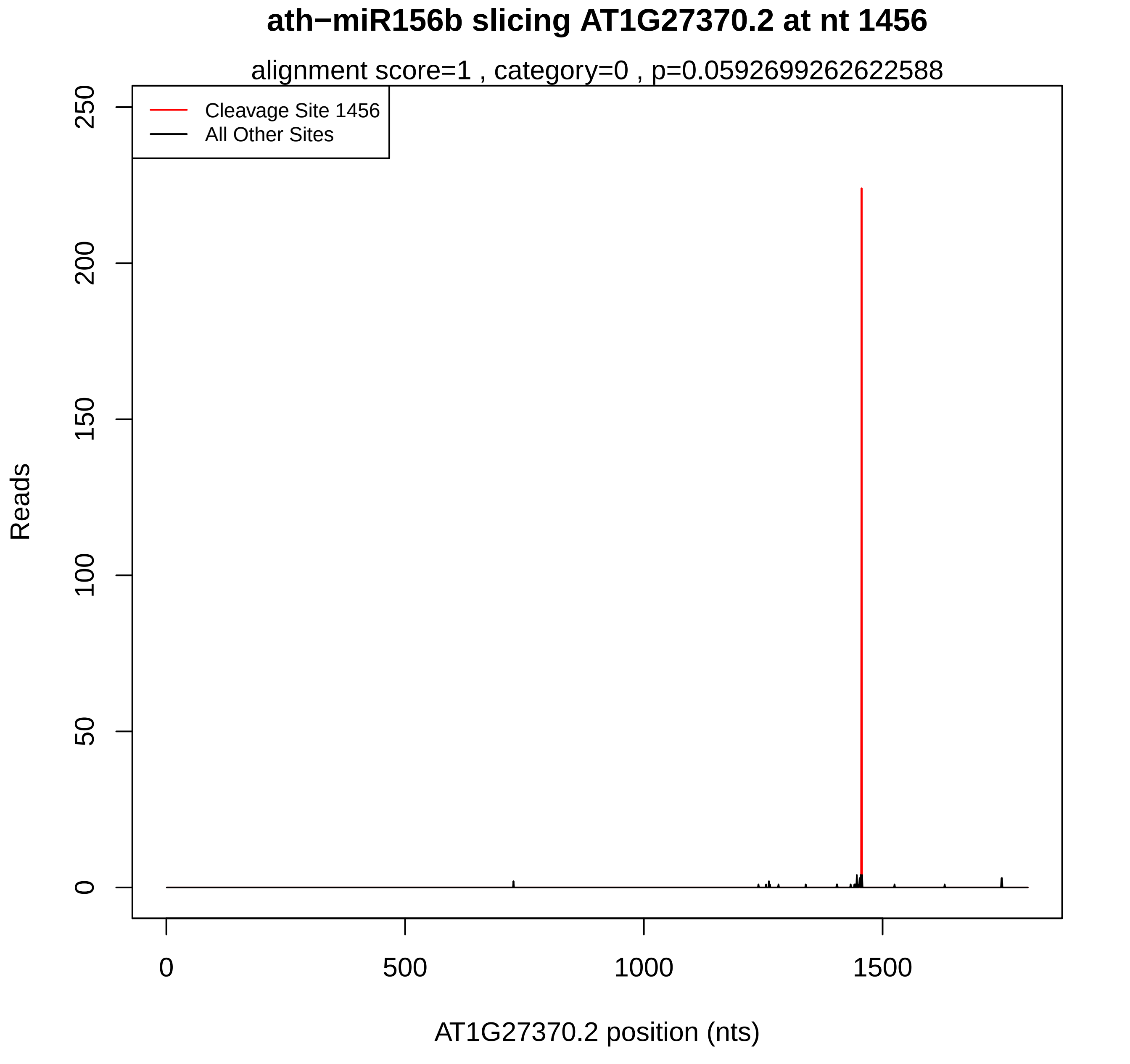
ath−miR156b slicing AT1G27370.2 at nt 1456
alignment score=1 , category=0 , p=0.0592699262622588
Cleavage Site 1456
All Other Sites
Reads
0
50
100
150
200
250
0
500
1000
1500
AT1G27370.2 position (nts)

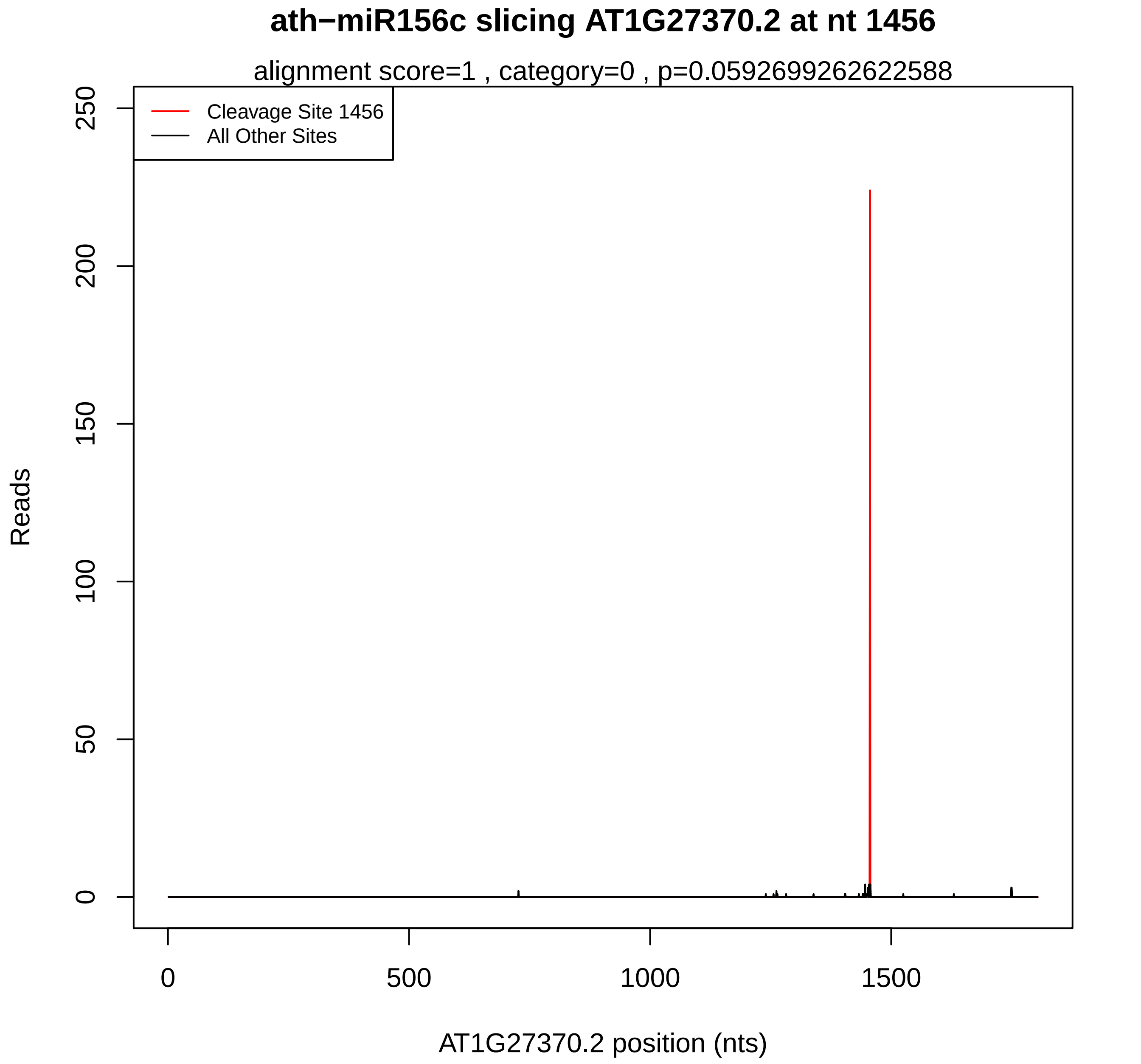
ath−miR156c slicing AT1G27370.2 at nt 1456
alignment score=1 , category=0 , p=0.0592699262622588
Cleavage Site 1456
All Other Sites
Reads
0
50
100
150
200
250
0
500
1000
1500
AT1G27370.2 position (nts)

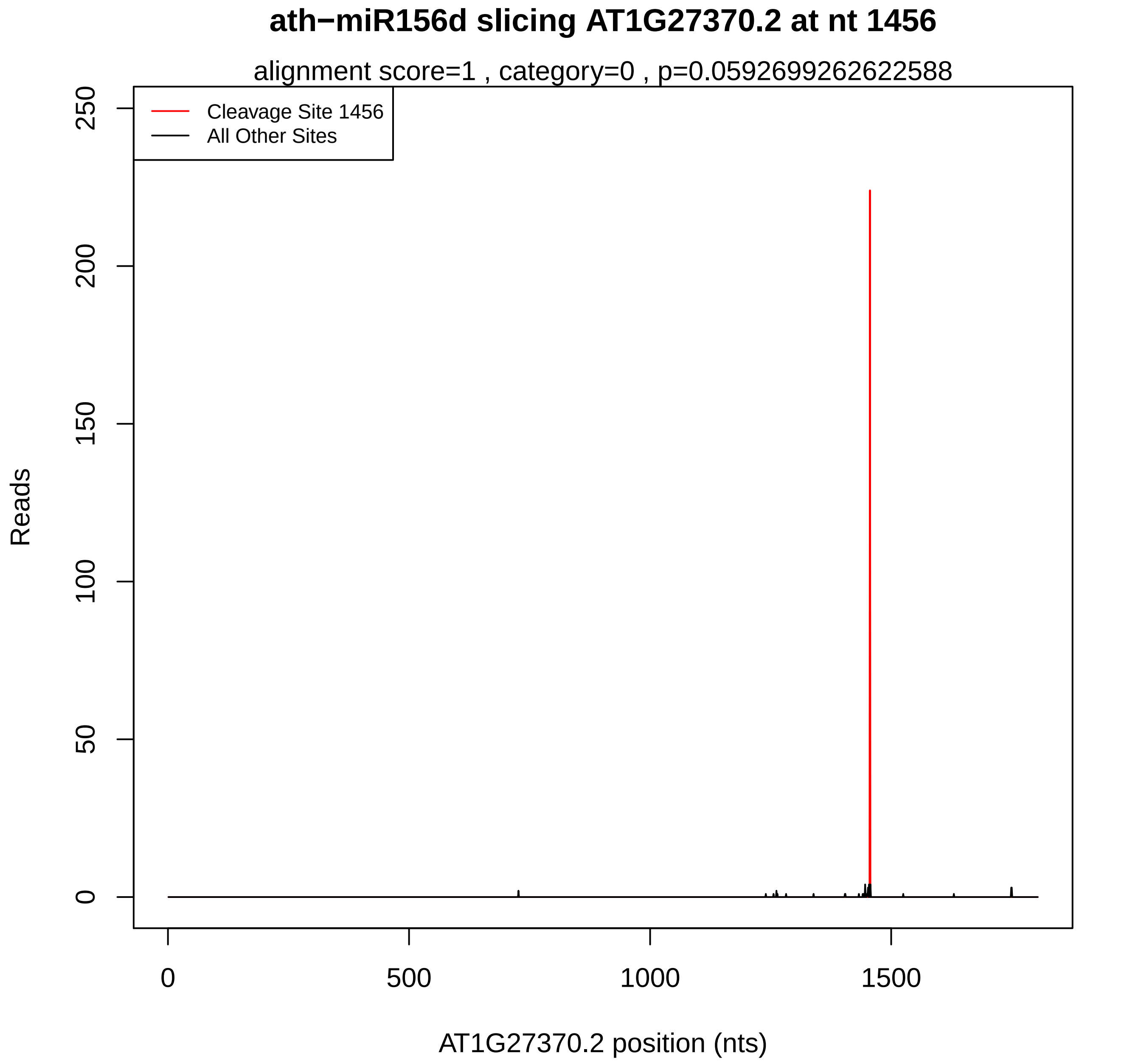
ath−miR156d slicing AT1G27370.2 at nt 1456
alignment score=1 , category=0 , p=0.0592699262622588
Cleavage Site 1456
All Other Sites
Reads
0
50
100
150
200
250
0
500
1000
1500
AT1G27370.2 position (nts)

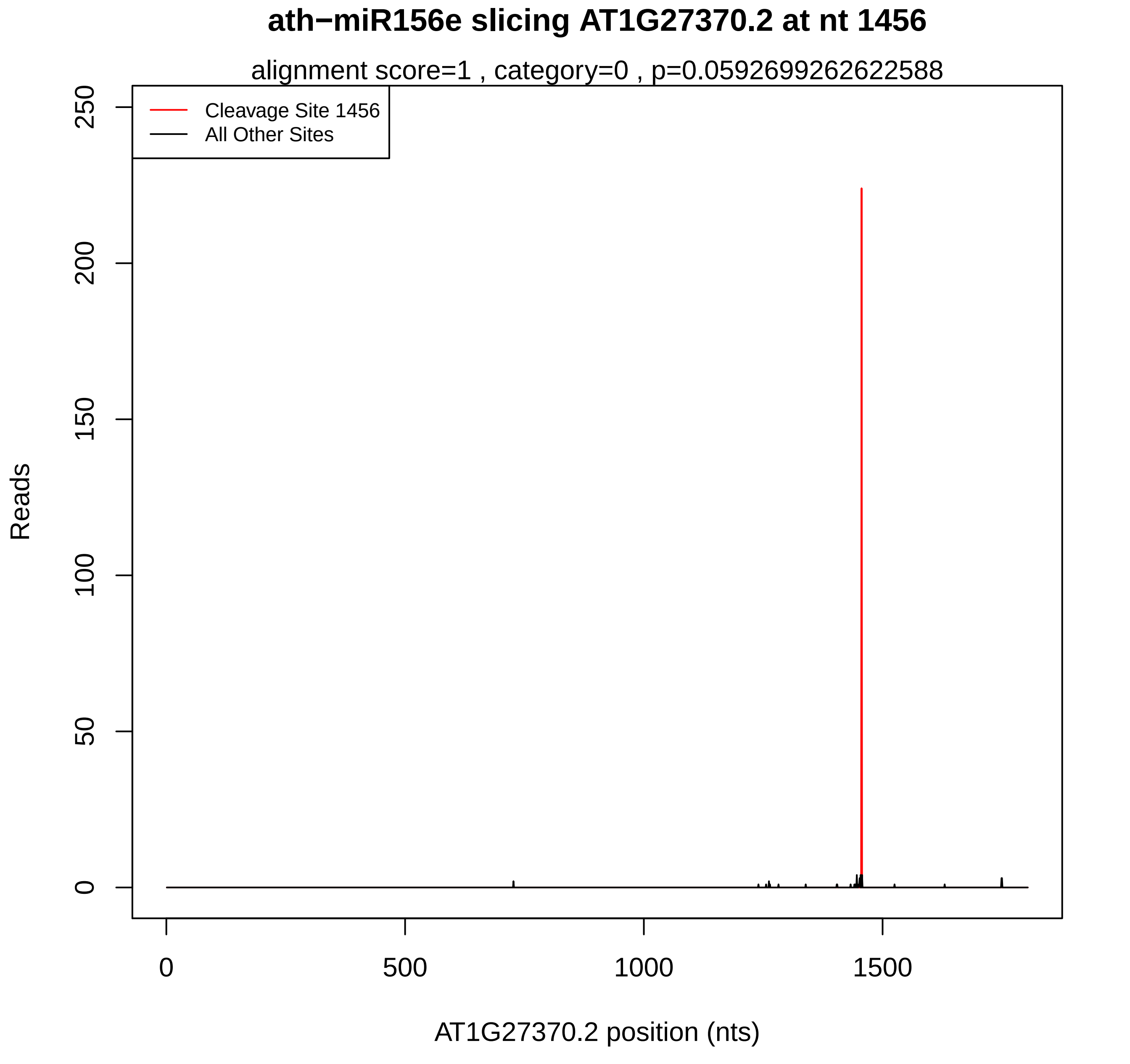

ath−miR156e slicing AT1G27370.2 at nt 1456
alignment score=1 , category=0 , p=0.0592699262622588
Cleavage Site 1456
All Other Sites
Reads
0
50
100
150
200
250
0
500
1000
1500
AT1G27370.2 position (nts)

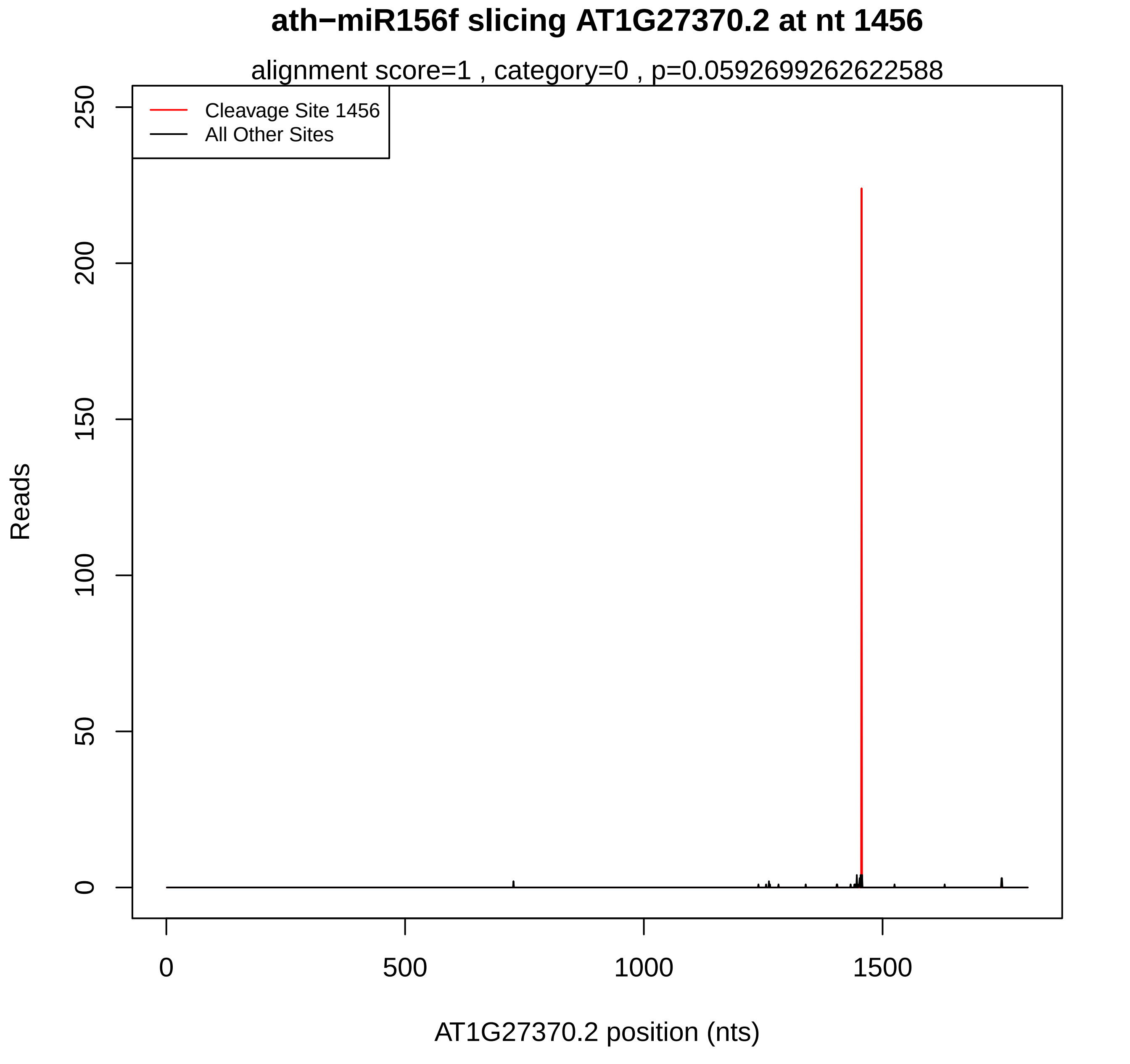

ath−miR156f slicing AT1G27370.2 at nt 1456
alignment score=1 , category=0 , p=0.0592699262622588
Cleavage Site 1456
All Other Sites
Reads
0
50
100
150
200
250
0
500
1000
1500
AT1G27370.2 position (nts)

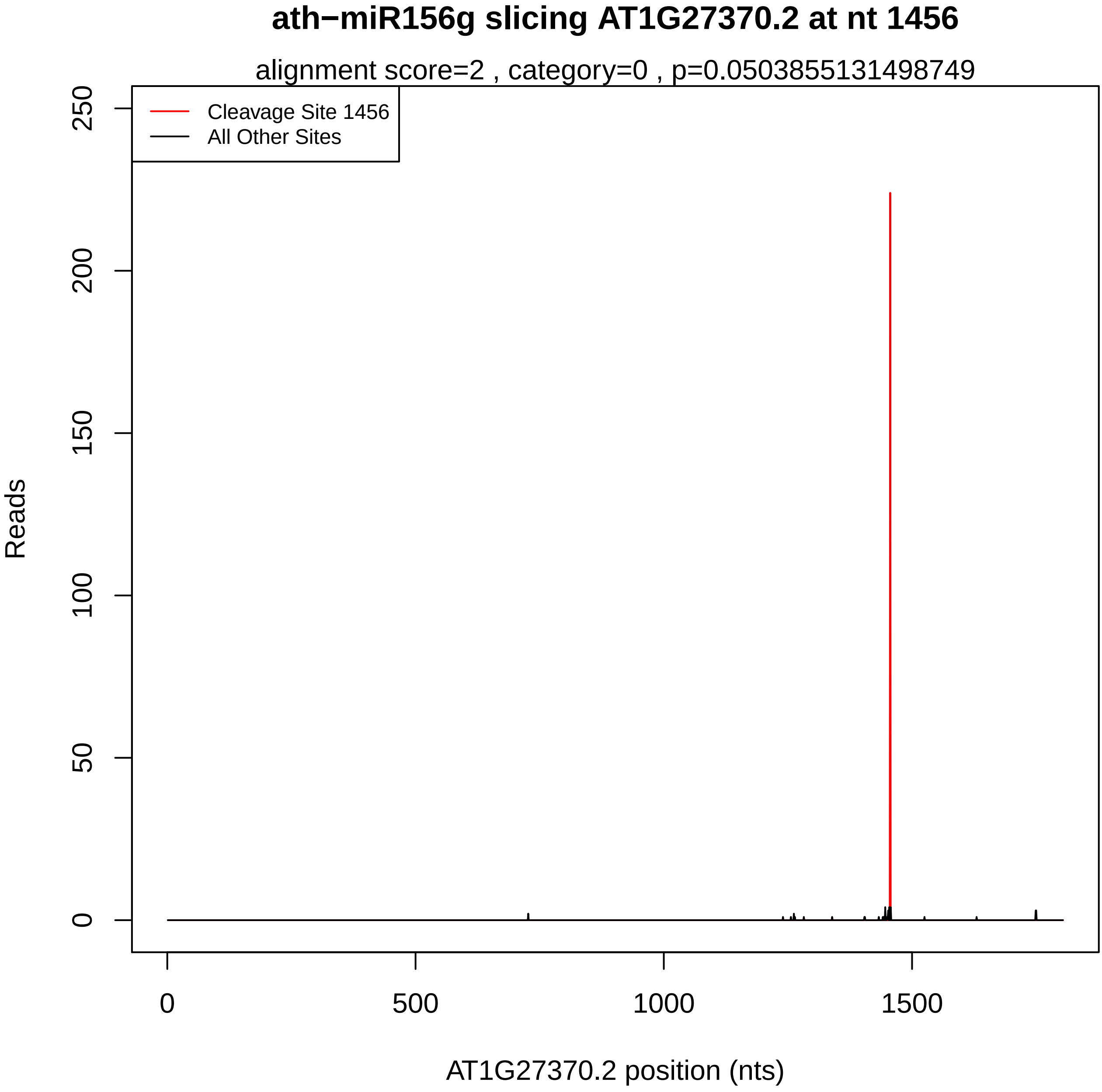
ath−miR156g slicing AT1G27370.2 at nt 1456
alignment score=2 , category=0 , p=0.0503855131498749
Cleavage Site 1456
All Other Sites
Reads
0
50
100
150
200
250
0
500
1000
1500
AT1G27370.2 position (nts)

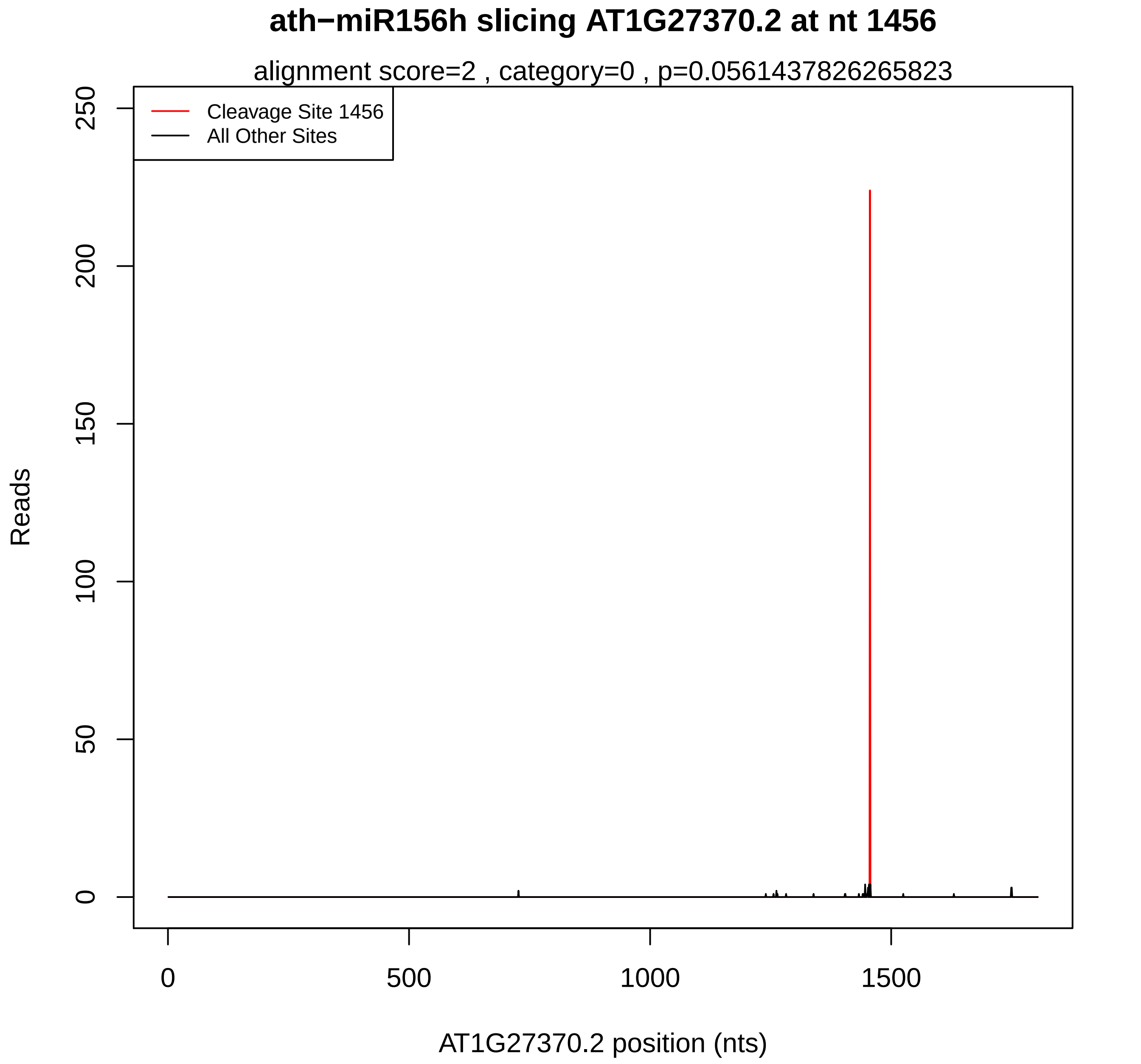
ath−miR156h slicing AT1G27370.2 at nt 1456
alignment score=2 , category=0 , p=0.0561437826265823
Cleavage Site 1456
All Other Sites
Reads
0
50
100
150
200
250
0
500
1000
1500
AT1G27370.2 position (nts)

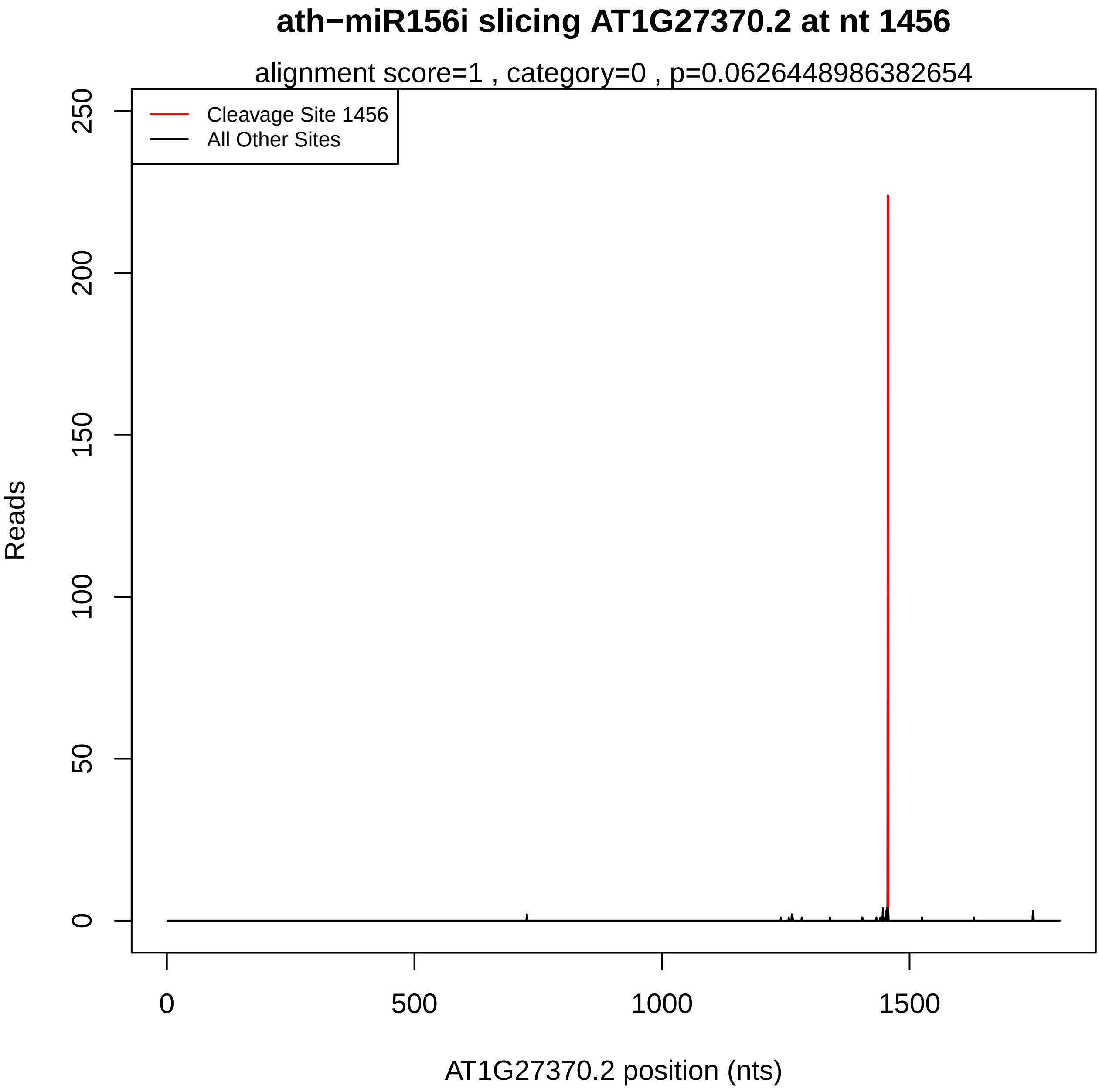
ath−miR156i slicing AT1G27370.2 at nt 1456
alignment score=1 , category=0 , p=0.0626448986382654
Cleavage Site 1456
All Other Sites
Reads
0
50
100
150
200
250
0
500
1000
1500
AT1G27370.2 position (nts)

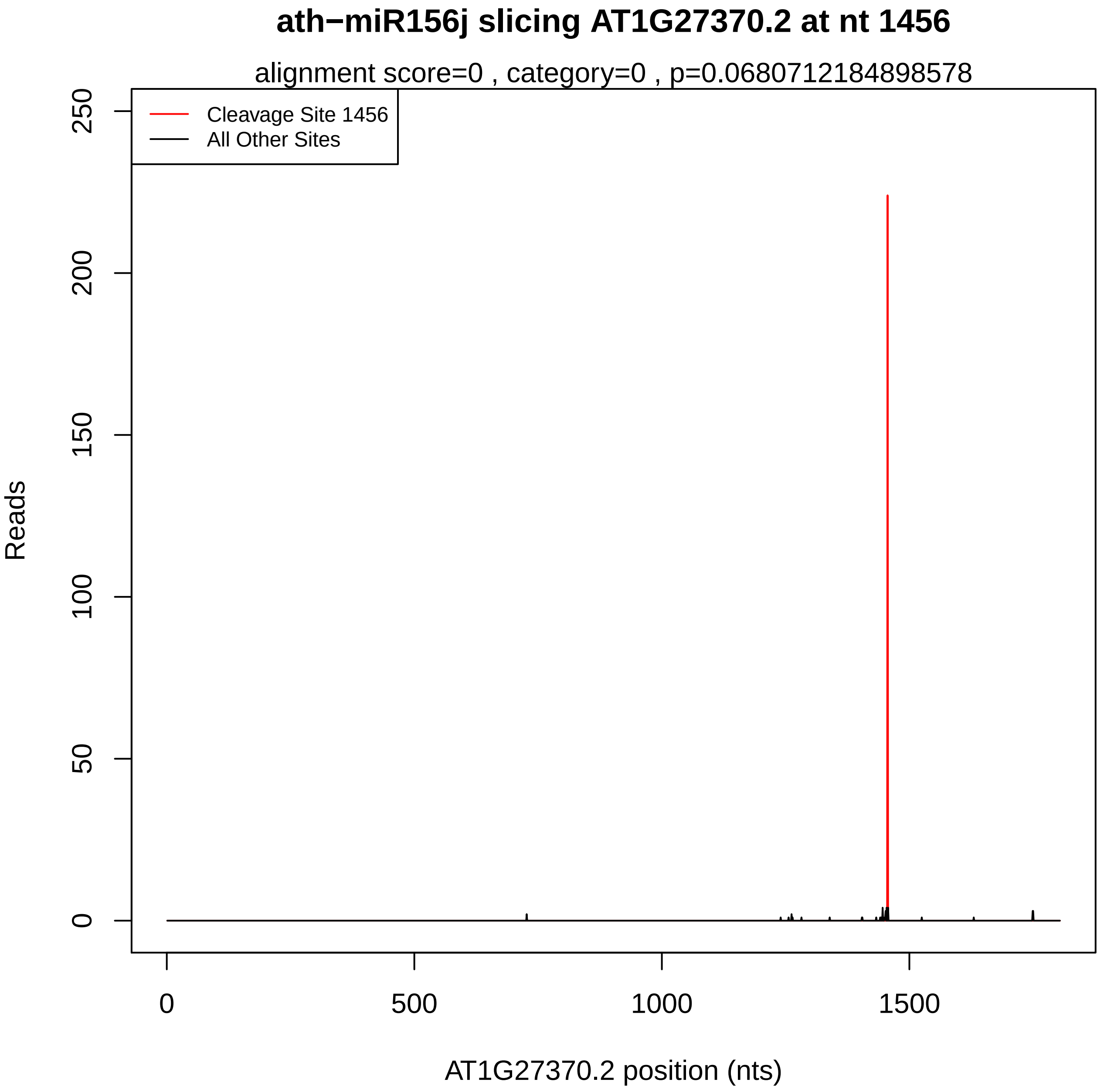
ath−miR156j slicing AT1G27370.2 at nt 1456
alignment score=0 , category=0 , p=0.0680712184898578
Cleavage Site 1456
All Other Sites
Reads
0
50
100
150
200
250
0
500
1000
1500
AT1G27370.2 position (nts)

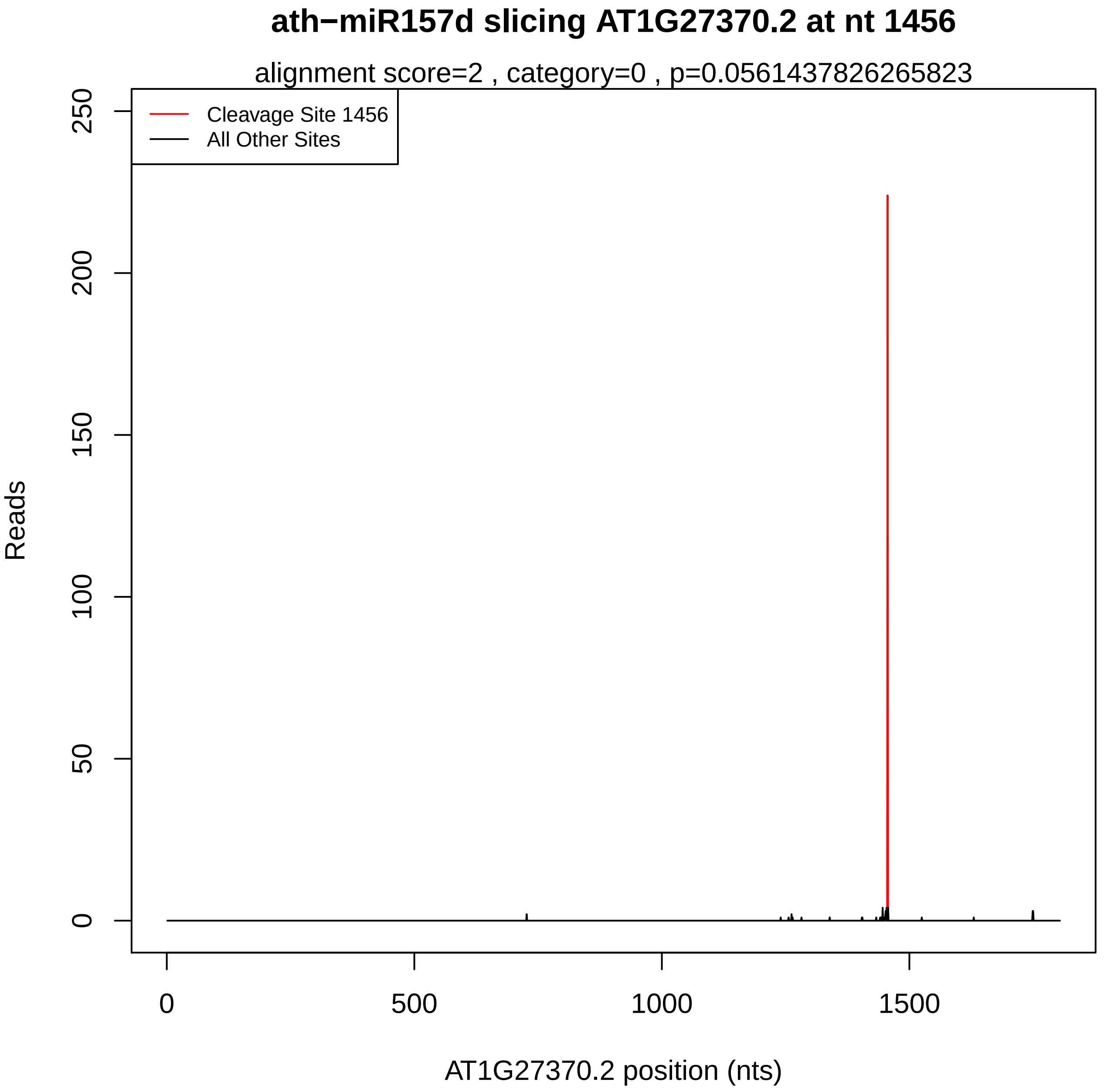
ath−miR157d slicing AT1G27370.2 at nt 1456
alignment score=2 , category=0 , p=0.0561437826265823
Cleavage Site 1456
All Other Sites
Reads
0
50
100
150
200
250
0
500
1000
1500
AT1G27370.2 position (nts)

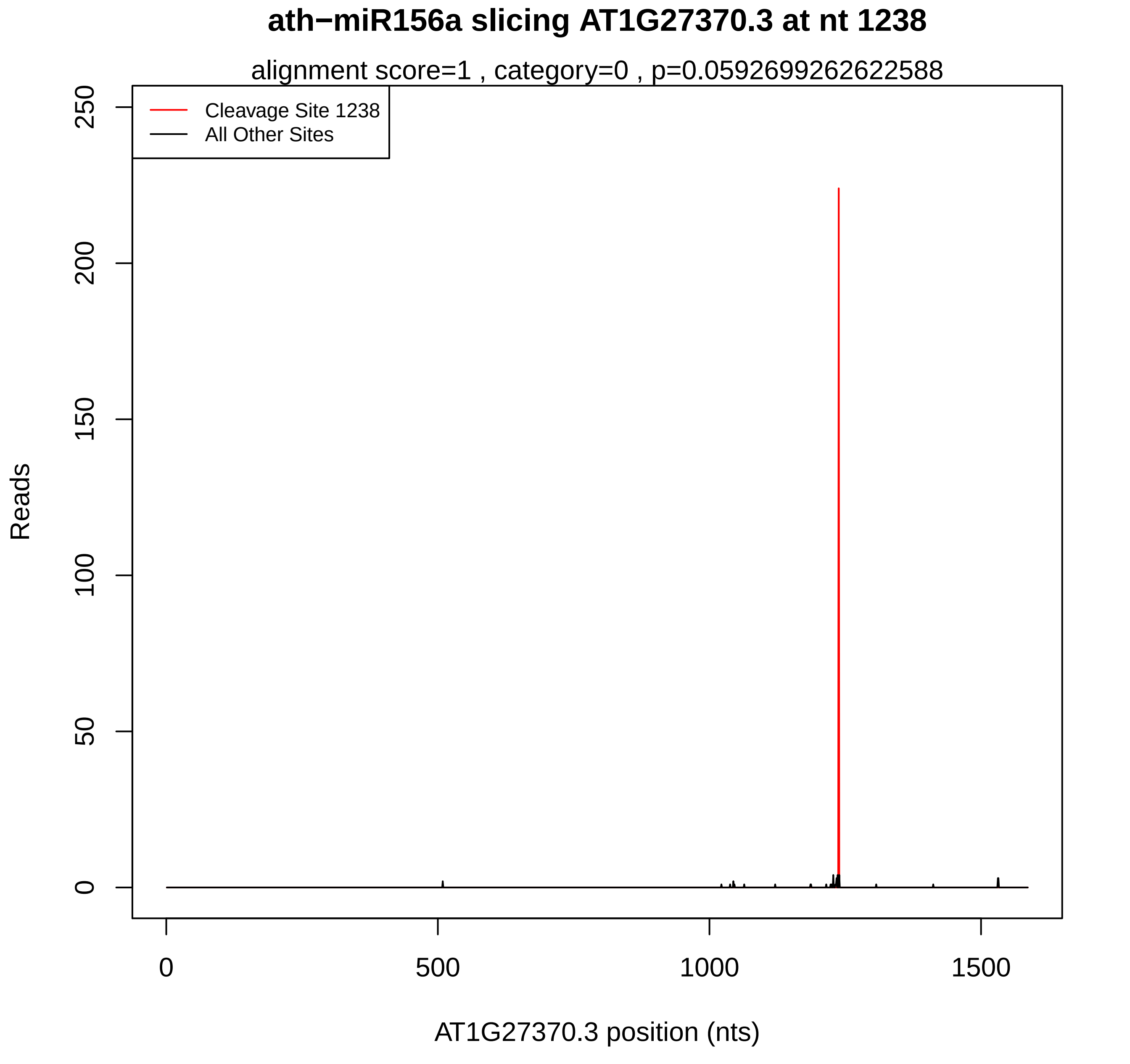
ath−miR156a slicing AT1G27370.3 at nt 1238
alignment score=1 , category=0 , p=0.0592699262622588
Cleavage Site 1238
All Other Sites
Reads
0
50
100
150
200
250
0
500
1000
1500
AT1G27370.3 position (nts)

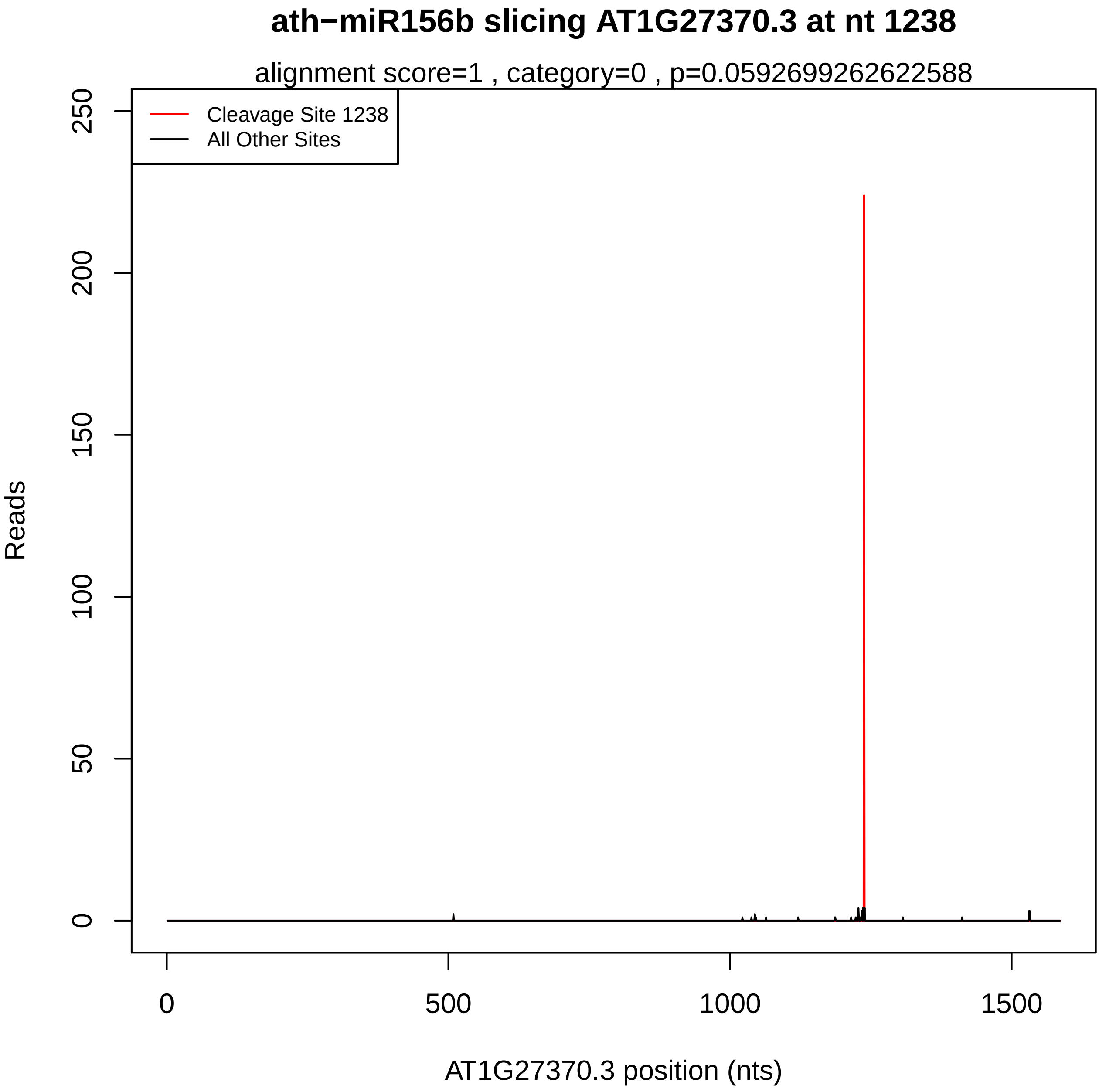
ath−miR156b slicing AT1G27370.3 at nt 1238
alignment score=1 , category=0 , p=0.0592699262622588
Cleavage Site 1238
All Other Sites
Reads
0
50
100
150
200
250
0
500
1000
1500
AT1G27370.3 position (nts)

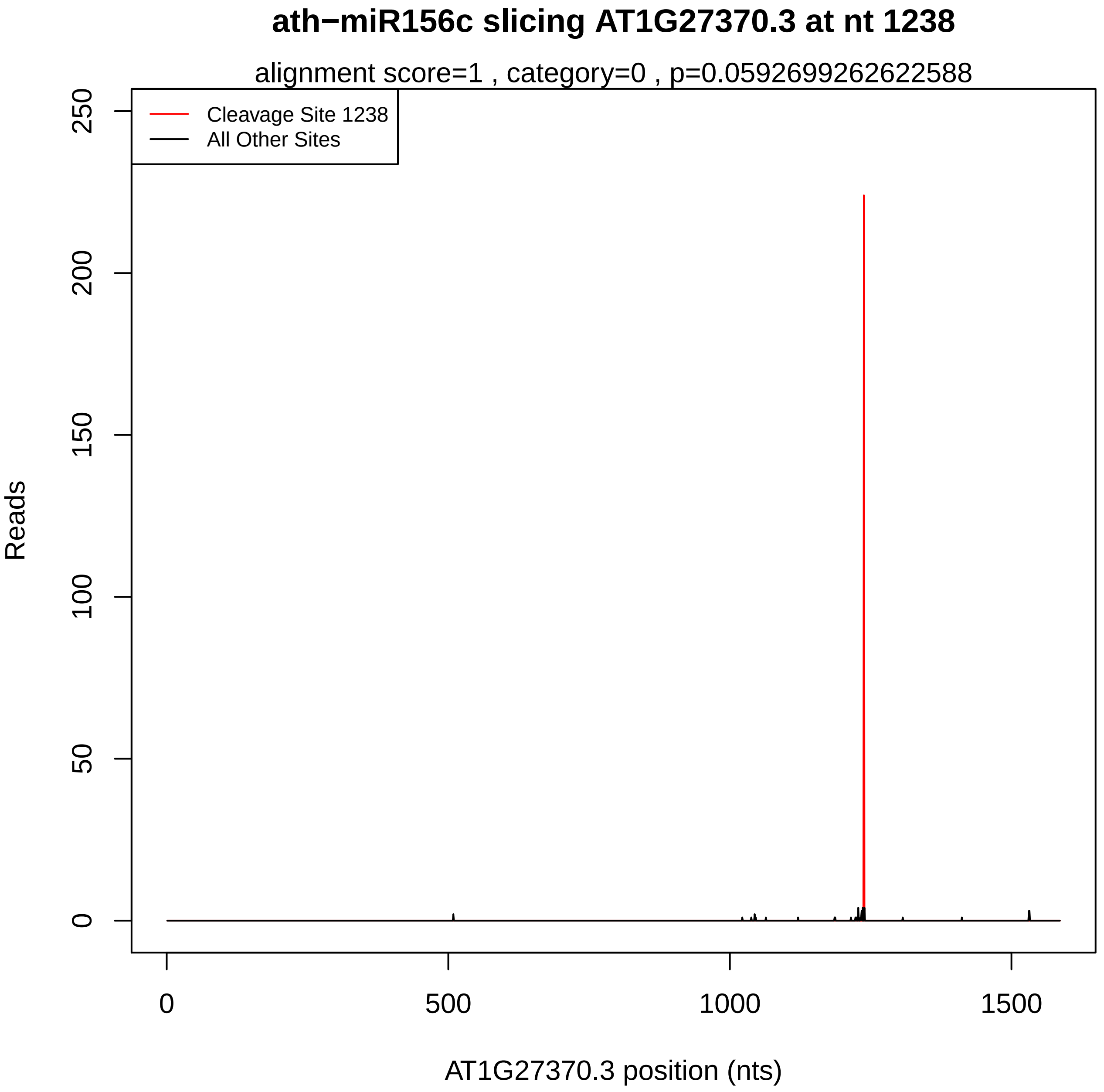

ath−miR156c slicing AT1G27370.3 at nt 1238
alignment score=1 , category=0 , p=0.0592699262622588
Cleavage Site 1238
All Other Sites
Reads
0
50
100
150
200
250
0
500
1000
1500
AT1G27370.3 position (nts)

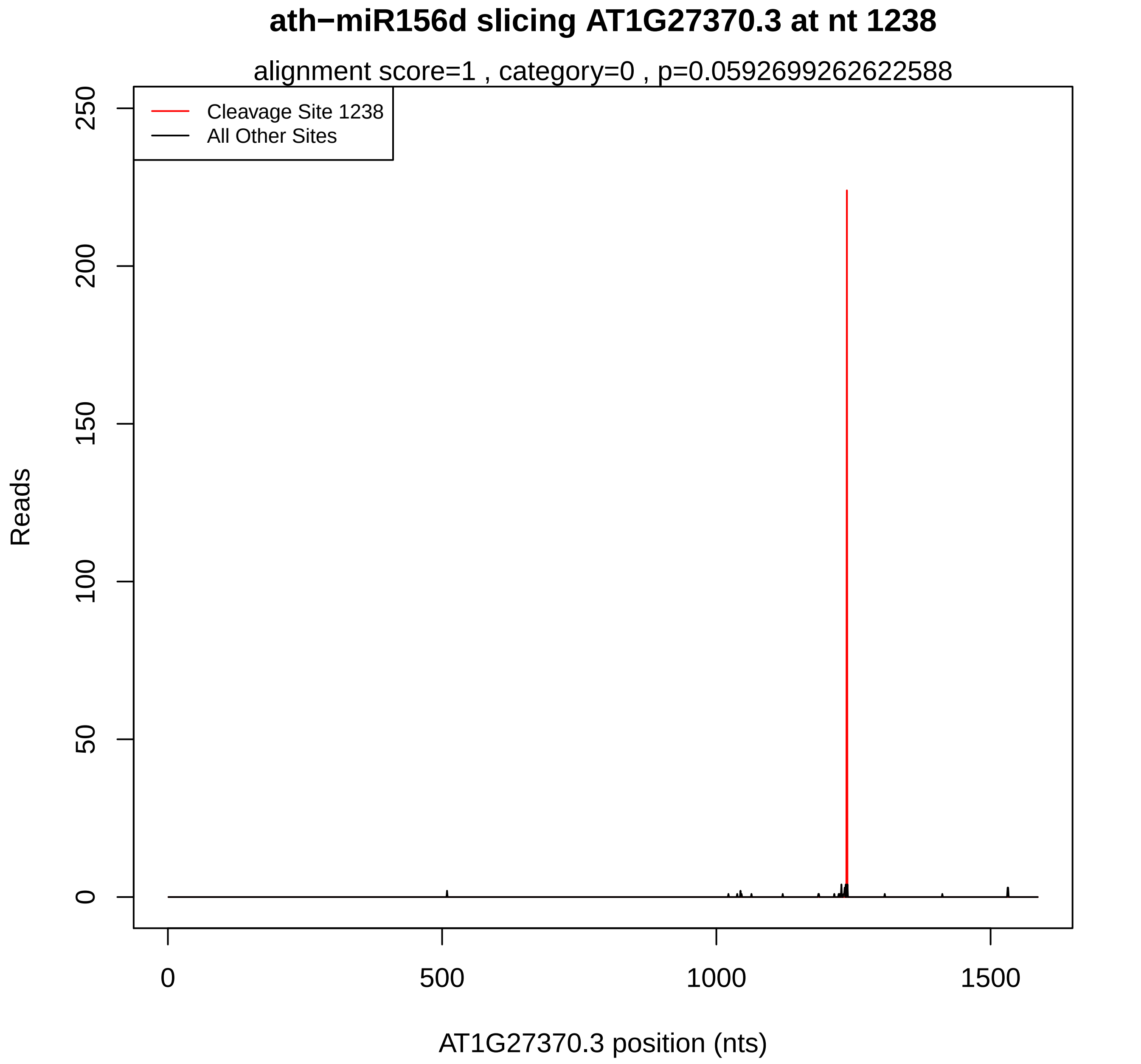
ath−miR156d slicing AT1G27370.3 at nt 1238
alignment score=1 , category=0 , p=0.0592699262622588
Cleavage Site 1238
All Other Sites
Reads
0
50
100
150
200
250
0
500
1000
1500
AT1G27370.3 position (nts)

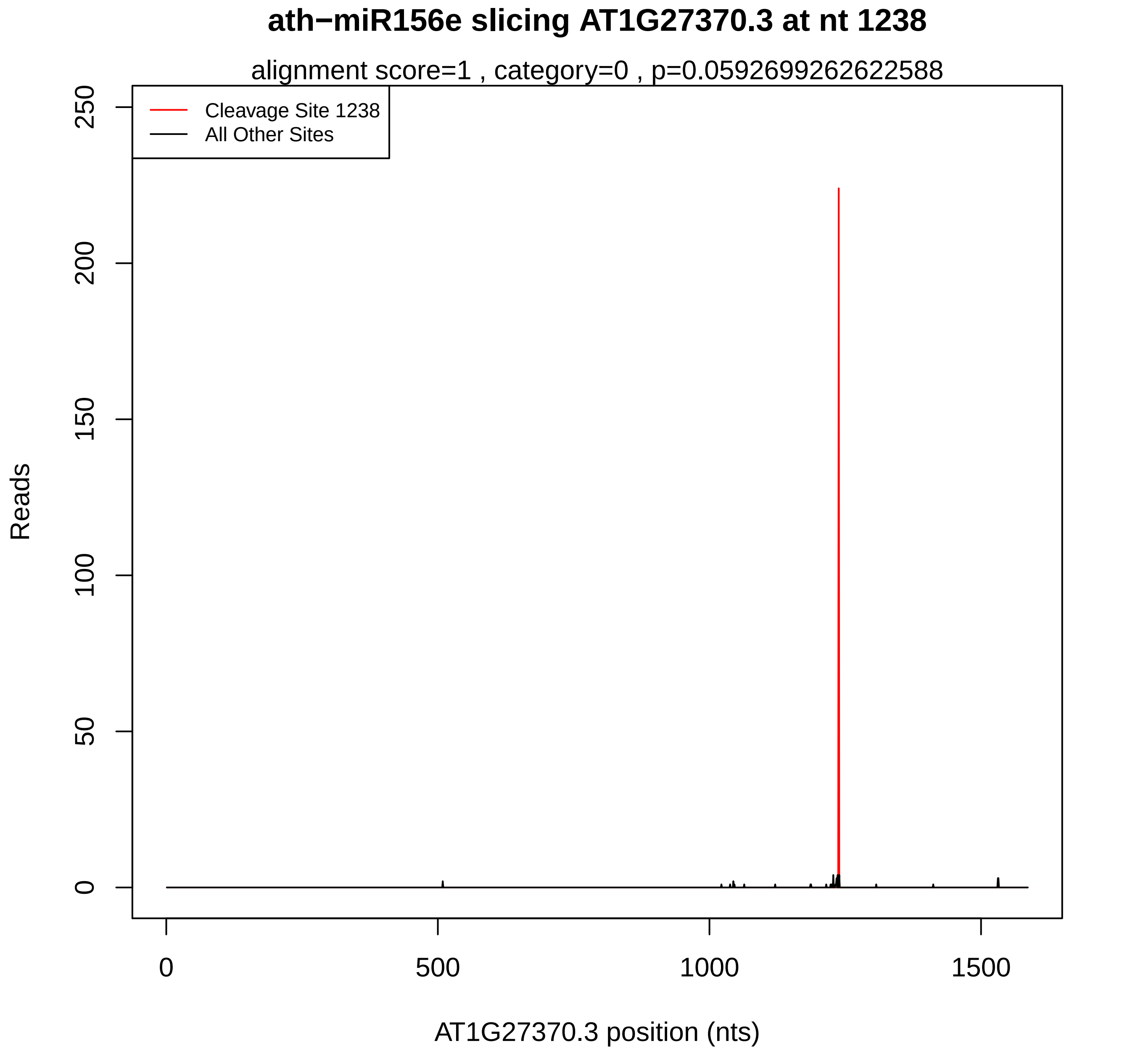
ath−miR156e slicing AT1G27370.3 at nt 1238
alignment score=1 , category=0 , p=0.0592699262622588
Cleavage Site 1238
All Other Sites
Reads
0
50
100
150
200
250
0
500
1000
1500
AT1G27370.3 position (nts)

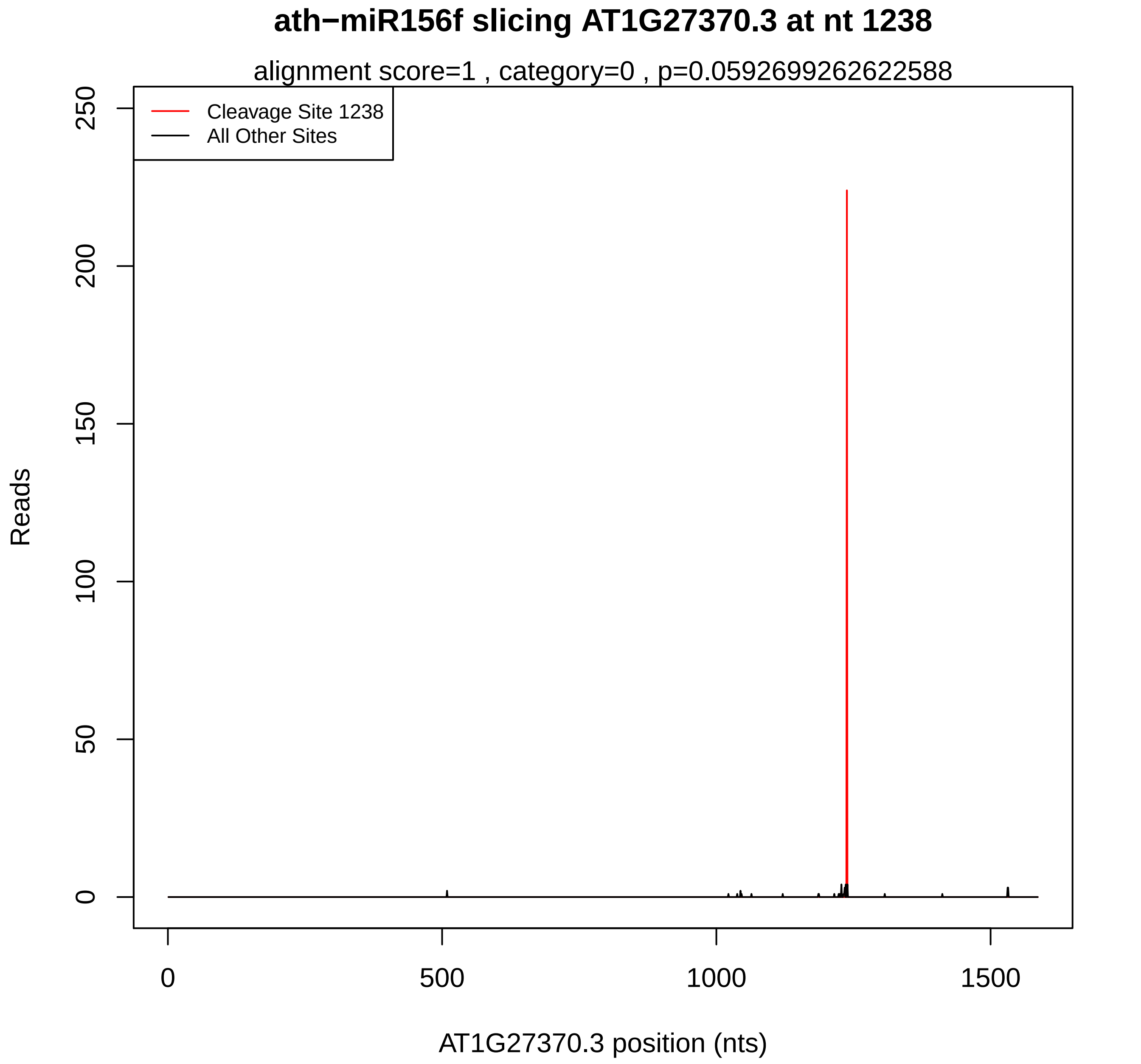
ath−miR156f slicing AT1G27370.3 at nt 1238
alignment score=1 , category=0 , p=0.0592699262622588
Cleavage Site 1238
All Other Sites
Reads
0
50
100
150
200
250
0
500
1000
1500
AT1G27370.3 position (nts)

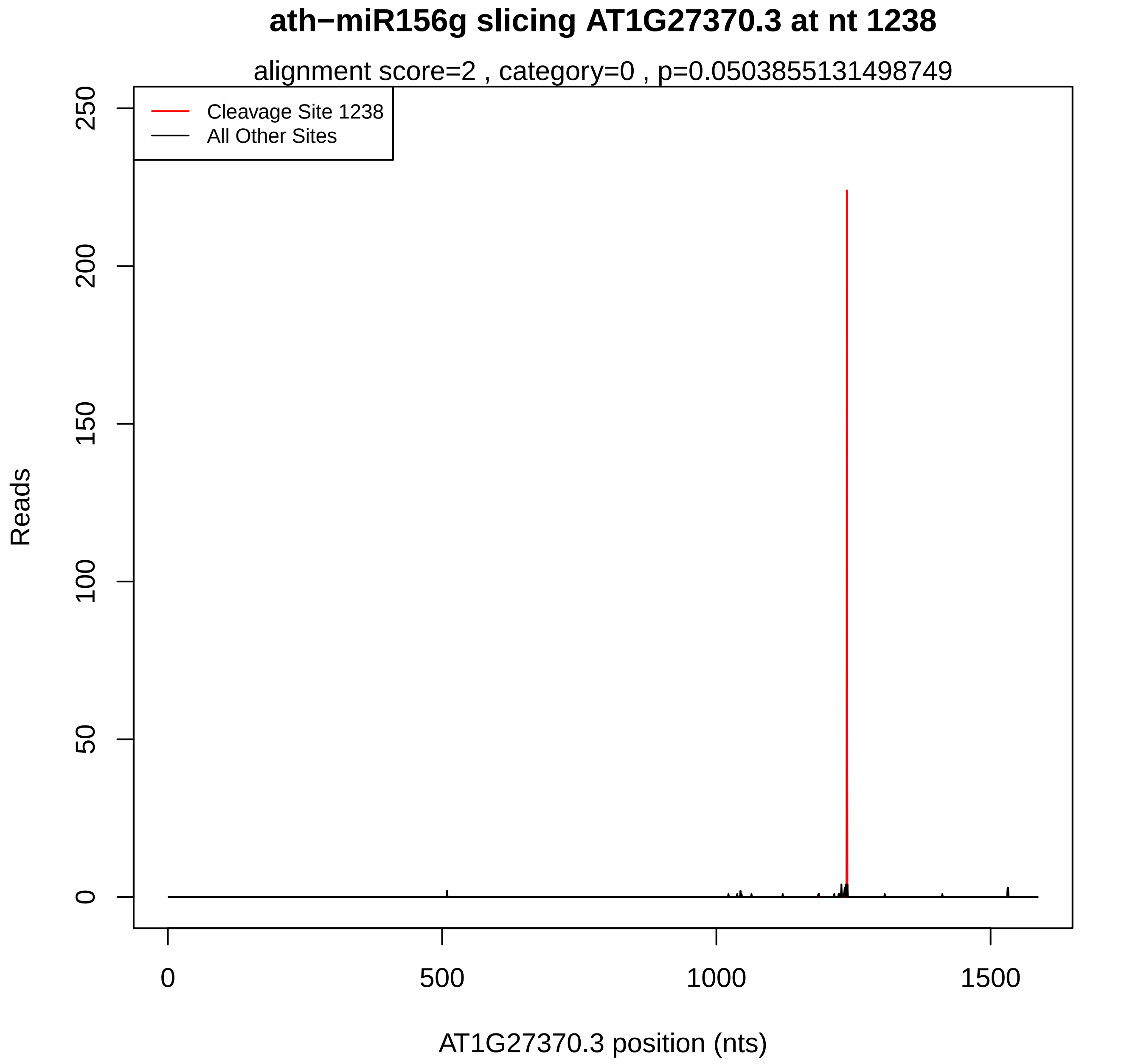
ath−miR156g slicing AT1G27370.3 at nt 1238
alignment score=2 , category=0 , p=0.0503855131498749
Cleavage Site 1238
All Other Sites
Reads
0
50
100
150
200
250
0
500
1000
1500
AT1G27370.3 position (nts)

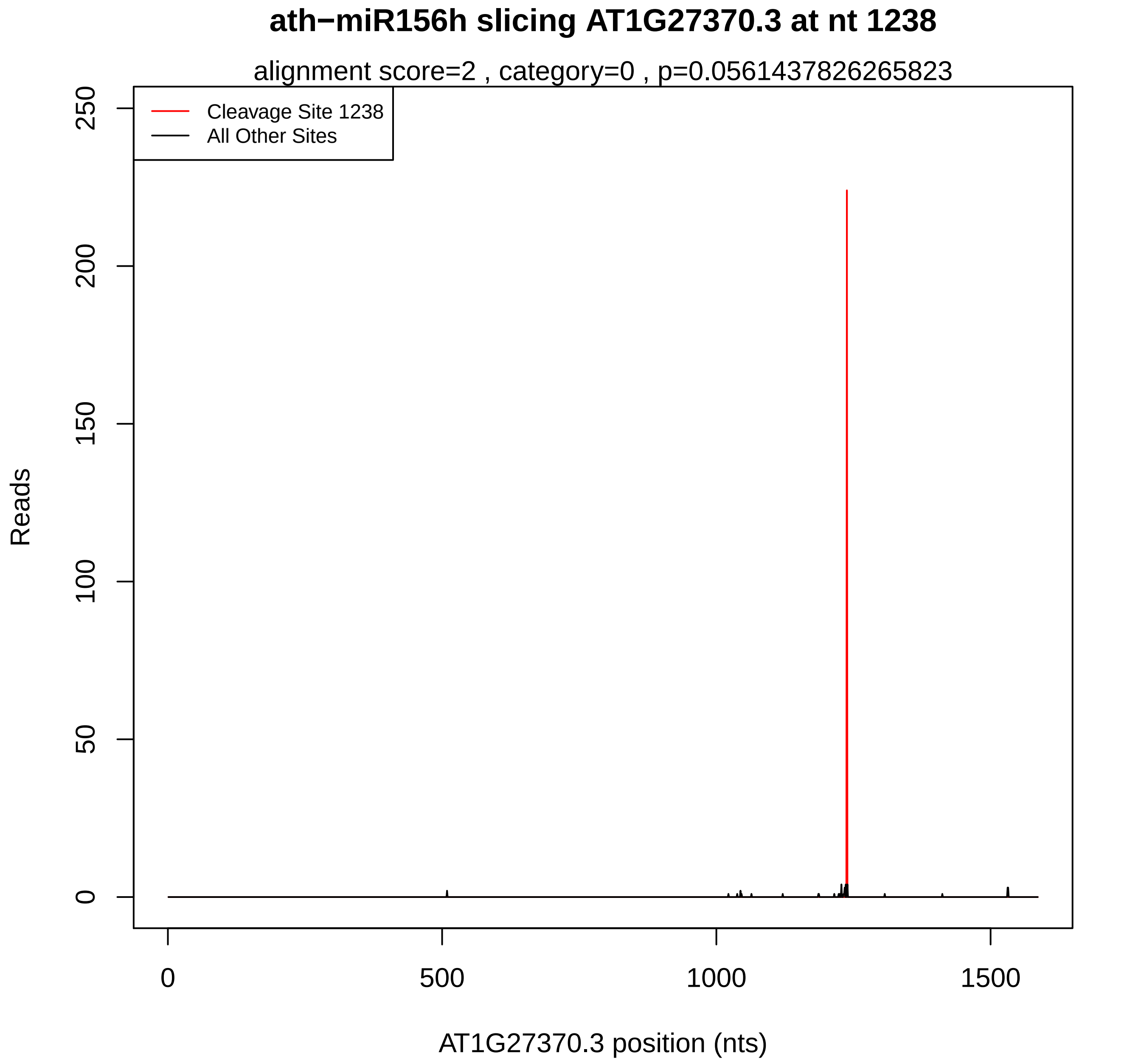

ath−miR156h slicing AT1G27370.3 at nt 1238
alignment score=2 , category=0 , p=0.0561437826265823
Cleavage Site 1238
All Other Sites
Reads
0
50
100
150
200
250
0
500
1000
1500
AT1G27370.3 position (nts)

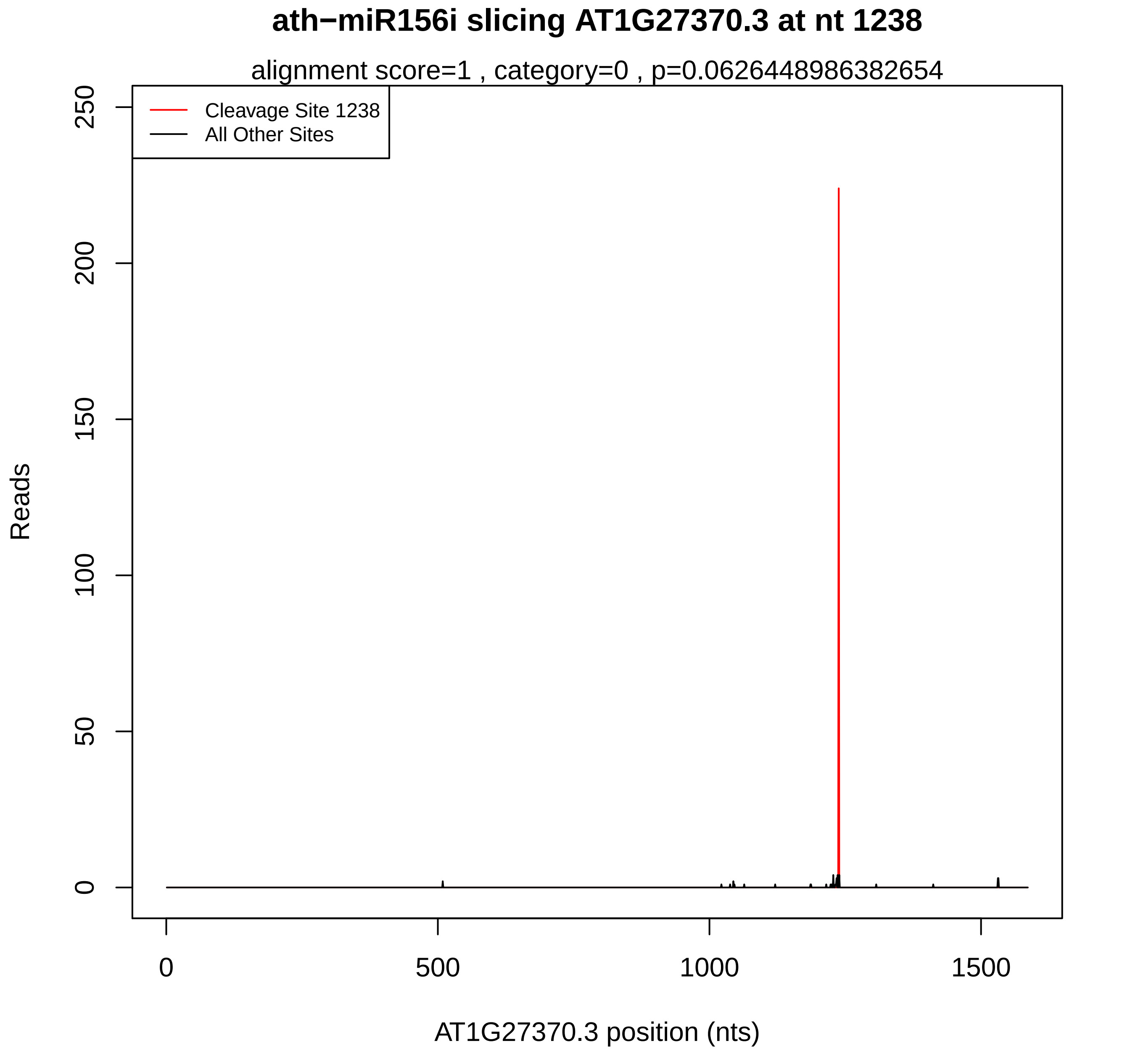
ath−miR156i slicing AT1G27370.3 at nt 1238
alignment score=1 , category=0 , p=0.0626448986382654
Cleavage Site 1238
All Other Sites
Reads
0
50
100
150
200
250
0
500
1000
1500
AT1G27370.3 position (nts)

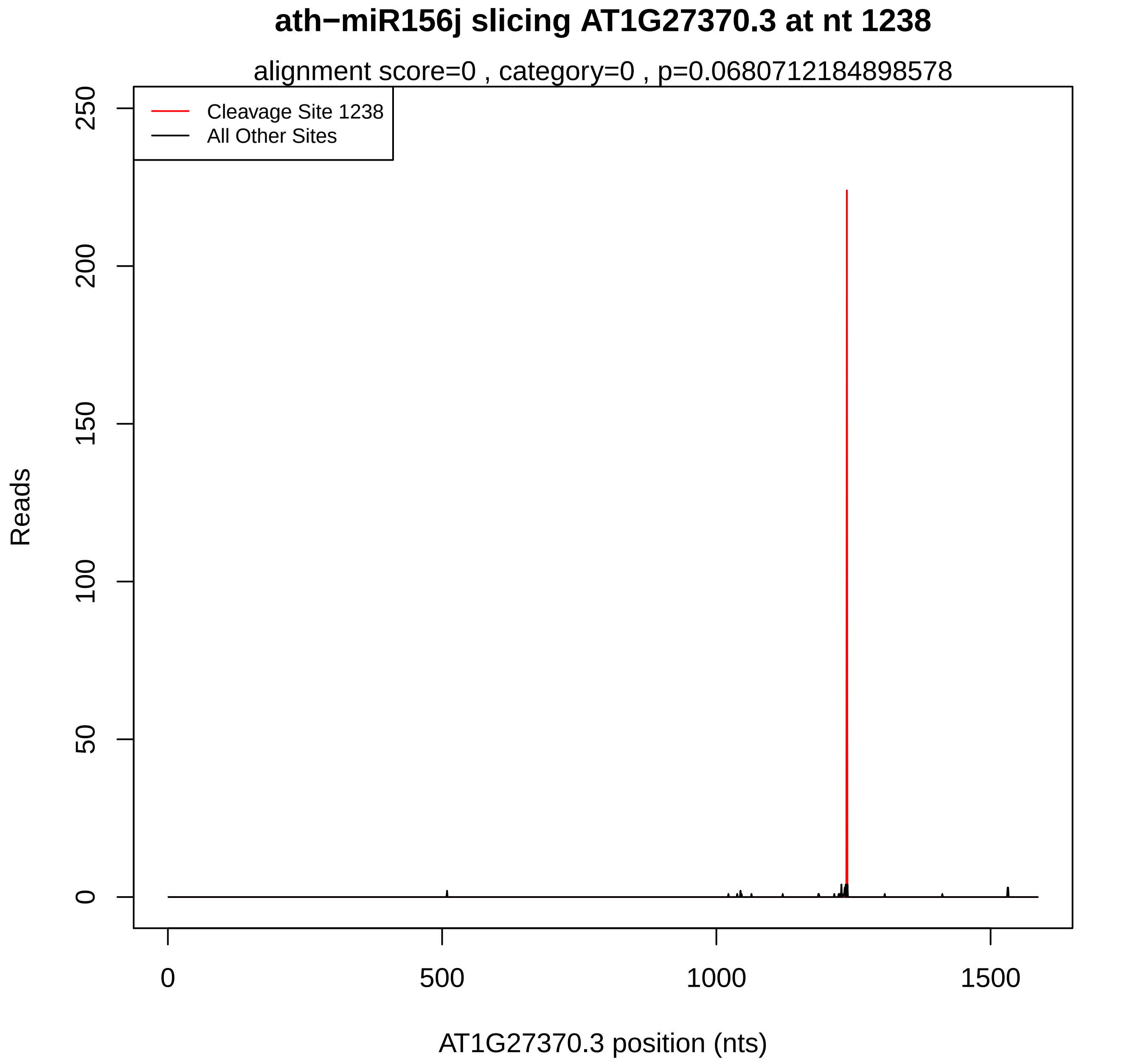
ath−miR156j slicing AT1G27370.3 at nt 1238
alignment score=0 , category=0 , p=0.0680712184898578
Cleavage Site 1238
All Other Sites
Reads
0
50
100
150
200
250
0
500
1000
1500
AT1G27370.3 position (nts)

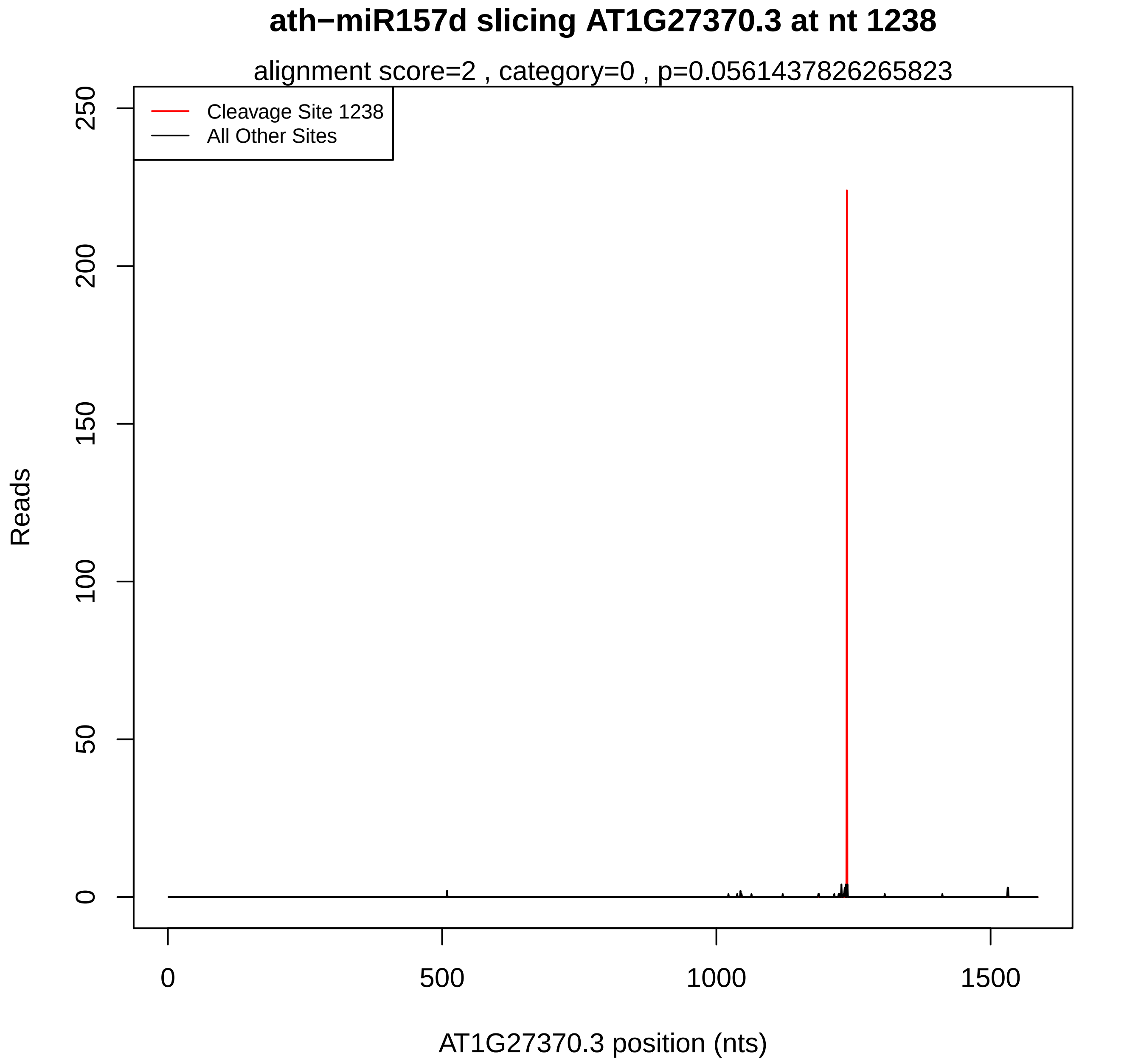

ath−miR157d slicing AT1G27370.3 at nt 1238
alignment score=2 , category=0 , p=0.0561437826265823
Cleavage Site 1238
All Other Sites
Reads
0
50
100
150
200
250
0
500
1000
1500
AT1G27370.3 position (nts)

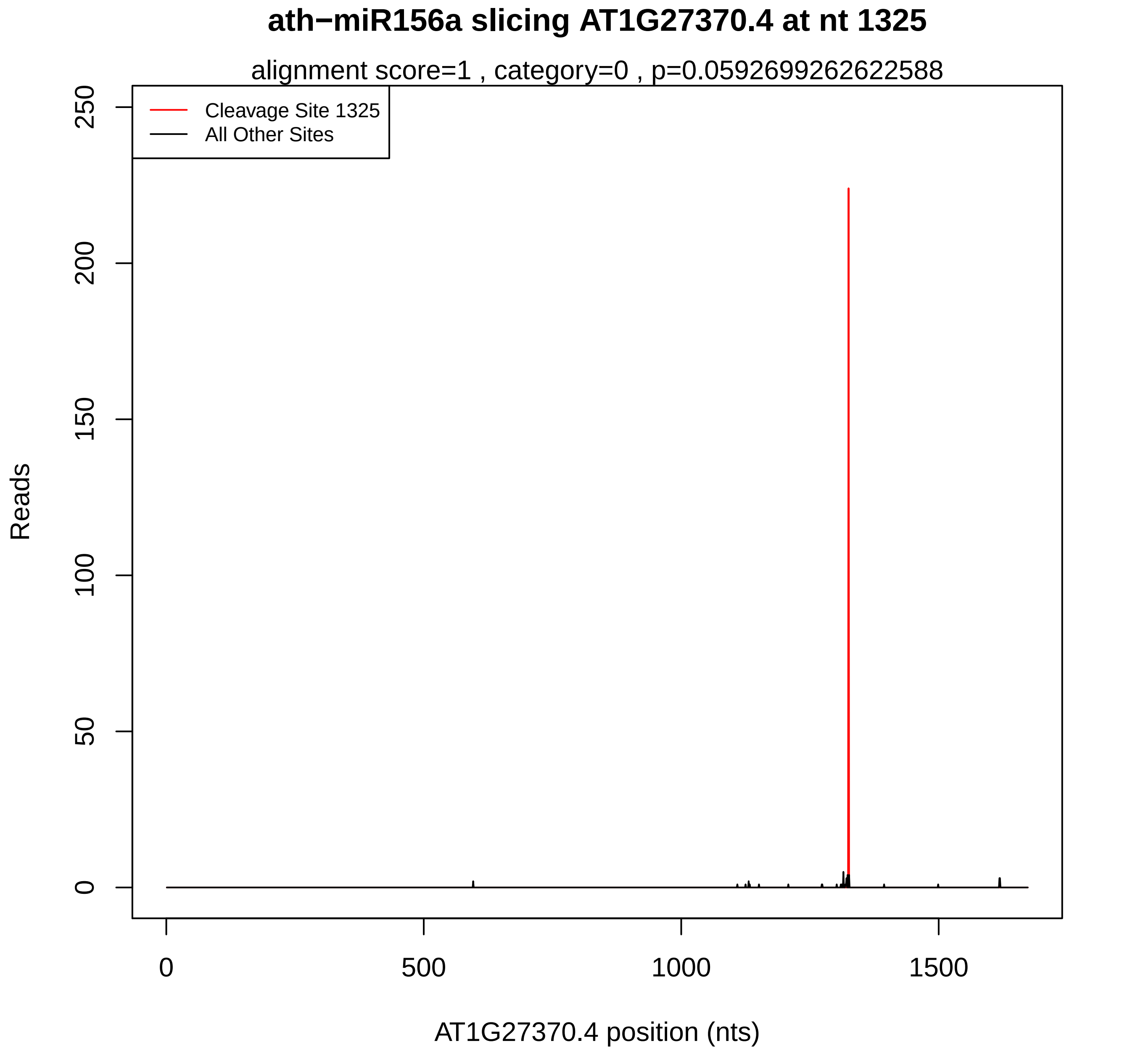

ath−miR156a slicing AT1G27370.4 at nt 1325
alignment score=1 , category=0 , p=0.0592699262622588
Cleavage Site 1325
All Other Sites
Reads
0
50
100
150
200
250
0
500
1000
1500
AT1G27370.4 position (nts)

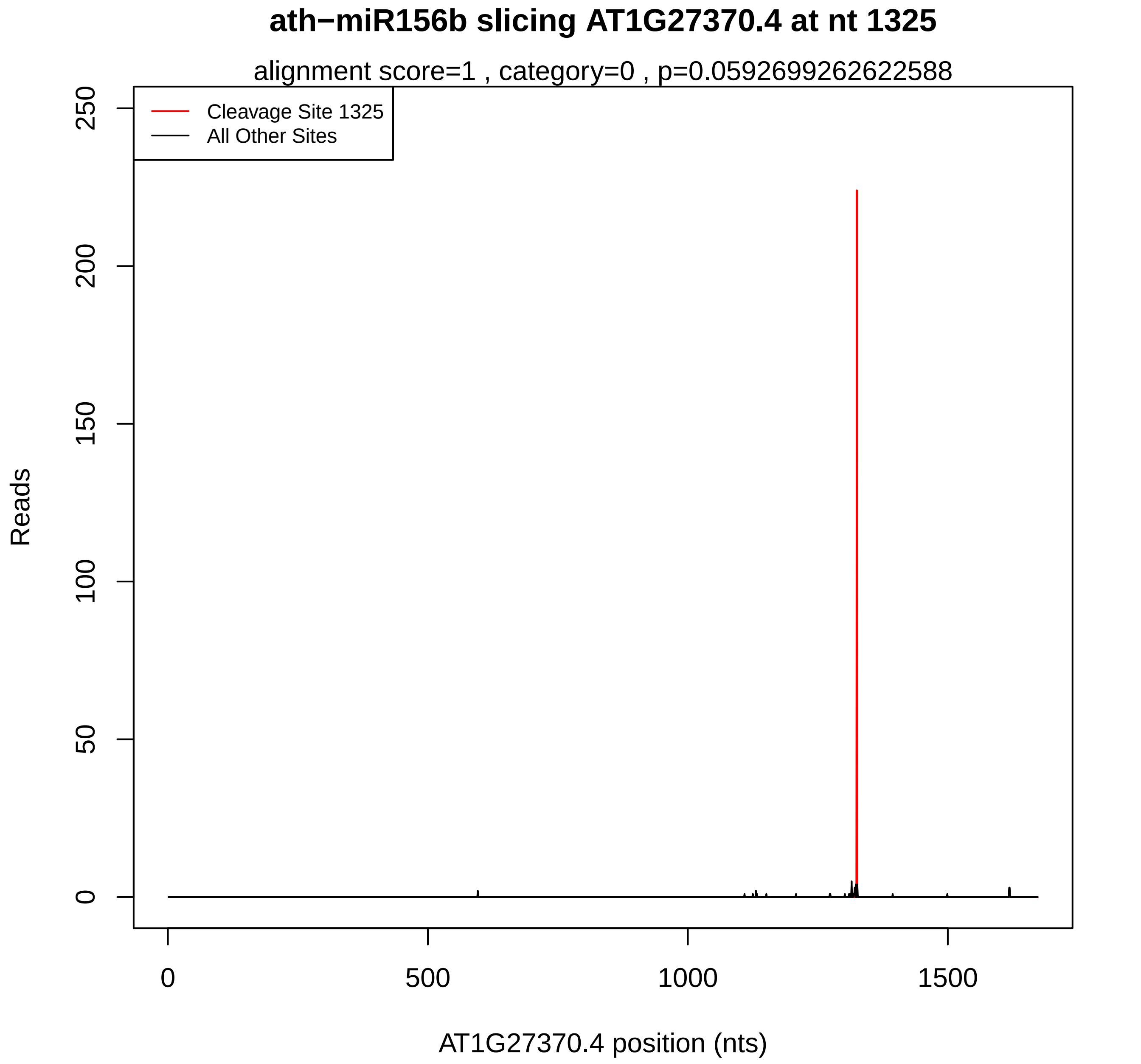
ath−miR156b slicing AT1G27370.4 at nt 1325
alignment score=1 , category=0 , p=0.0592699262622588
Cleavage Site 1325
All Other Sites
Reads
0
50
100
150
200
250
0
500
1000
1500
AT1G27370.4 position (nts)

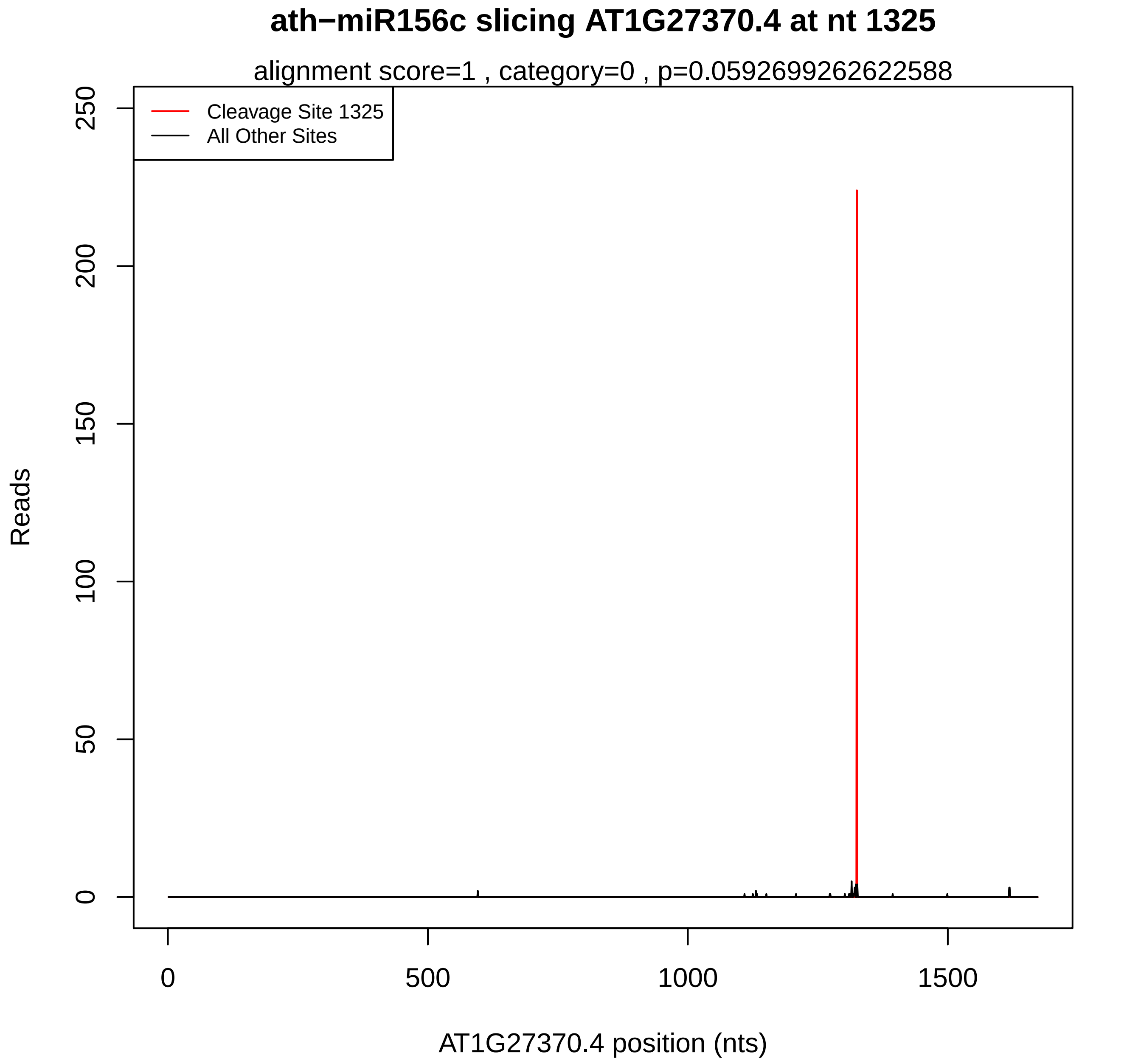
ath−miR156c slicing AT1G27370.4 at nt 1325
alignment score=1 , category=0 , p=0.0592699262622588
Cleavage Site 1325
All Other Sites
Reads
0
50
100
150
200
250
0
500
1000
1500
AT1G27370.4 position (nts)

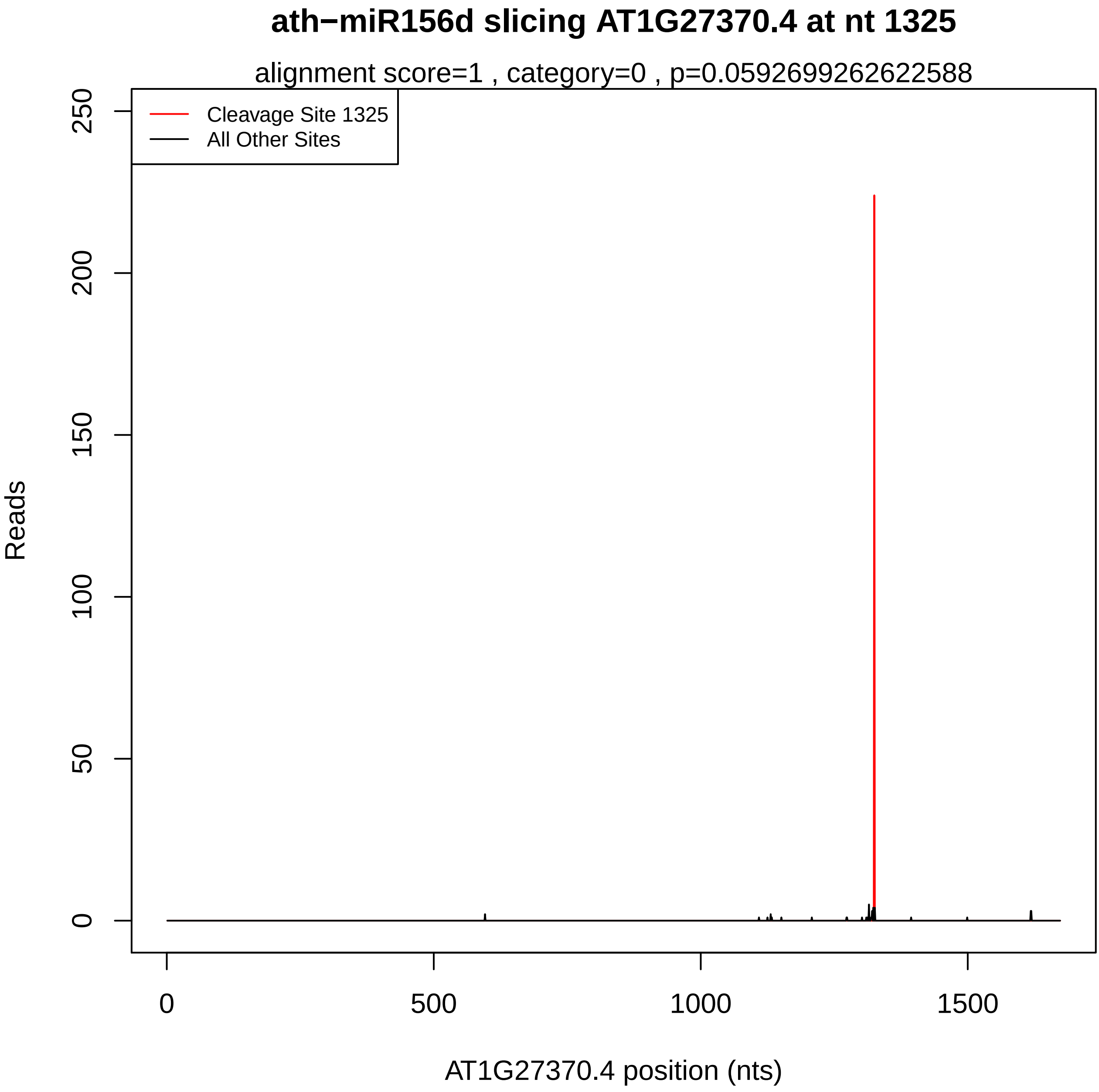

ath−miR156d slicing AT1G27370.4 at nt 1325
alignment score=1 , category=0 , p=0.0592699262622588
Cleavage Site 1325
All Other Sites
Reads
0
50
100
150
200
250
0
500
1000
1500
AT1G27370.4 position (nts)

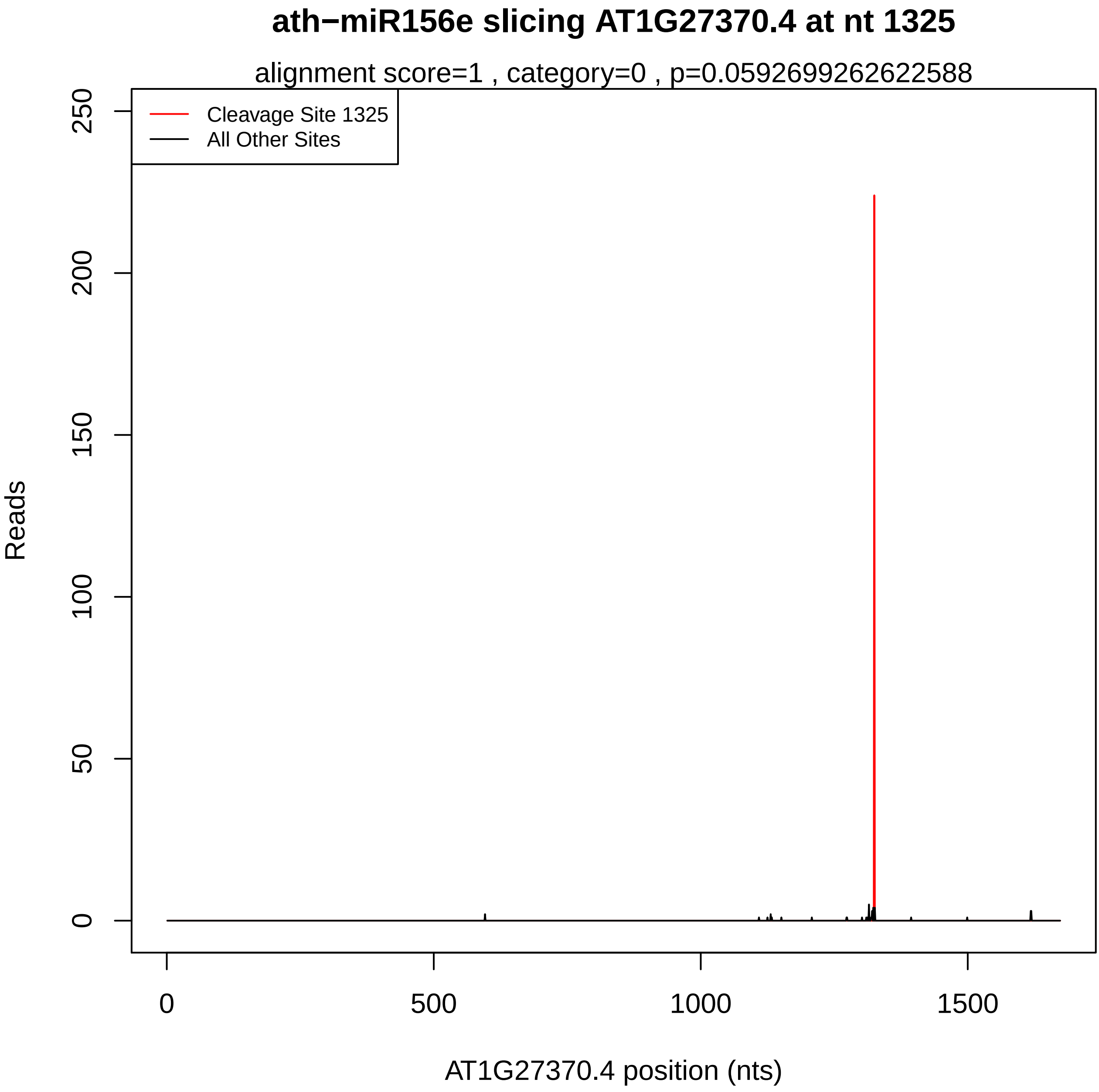

ath−miR156e slicing AT1G27370.4 at nt 1325
alignment score=1 , category=0 , p=0.0592699262622588
Cleavage Site 1325
All Other Sites
Reads
0
50
100
150
200
250
0
500
1000
1500
AT1G27370.4 position (nts)

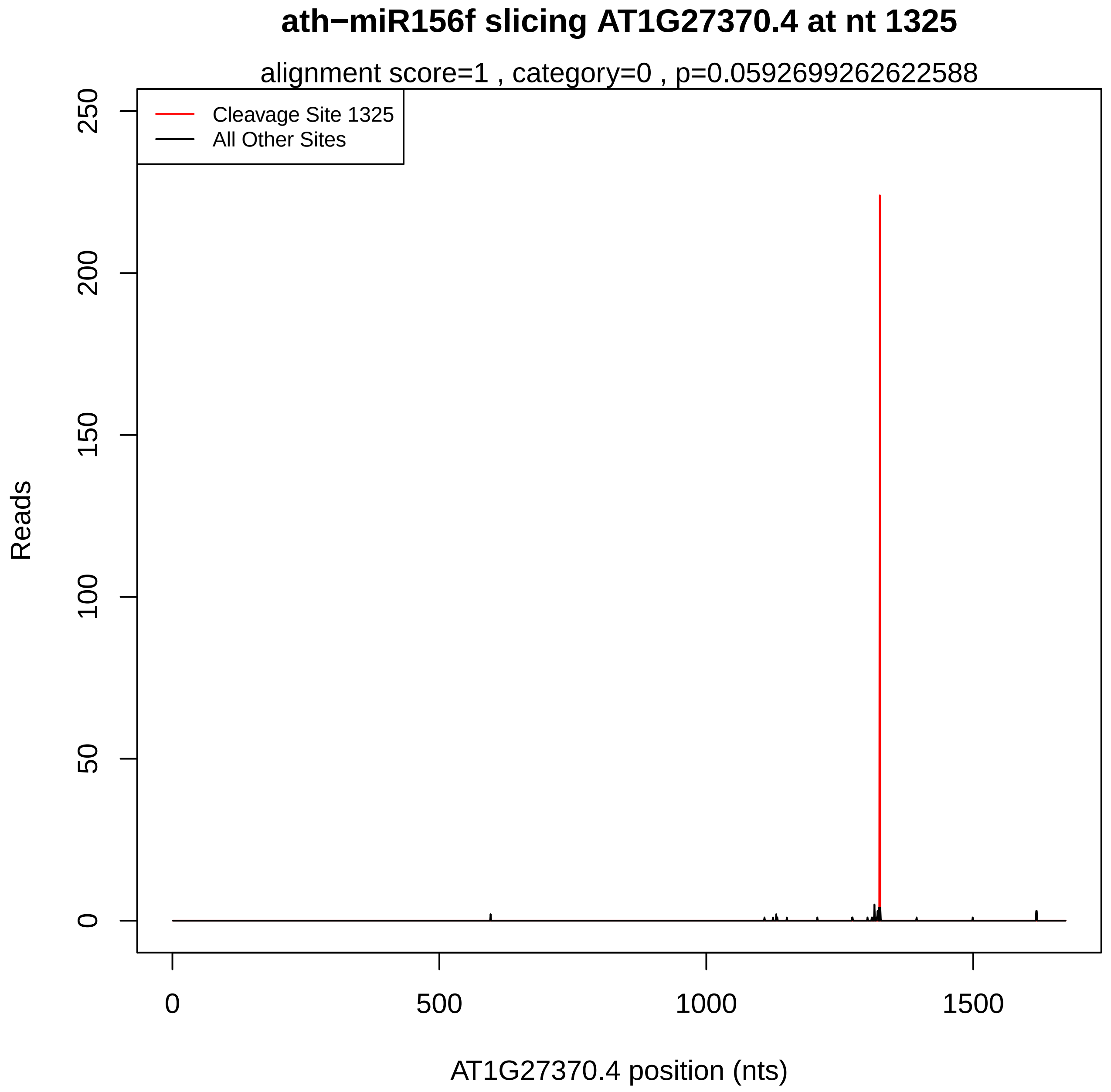

ath−miR156f slicing AT1G27370.4 at nt 1325
alignment score=1 , category=0 , p=0.0592699262622588
Cleavage Site 1325
All Other Sites
Reads
0
50
100
150
200
250
0
500
1000
1500
AT1G27370.4 position (nts)

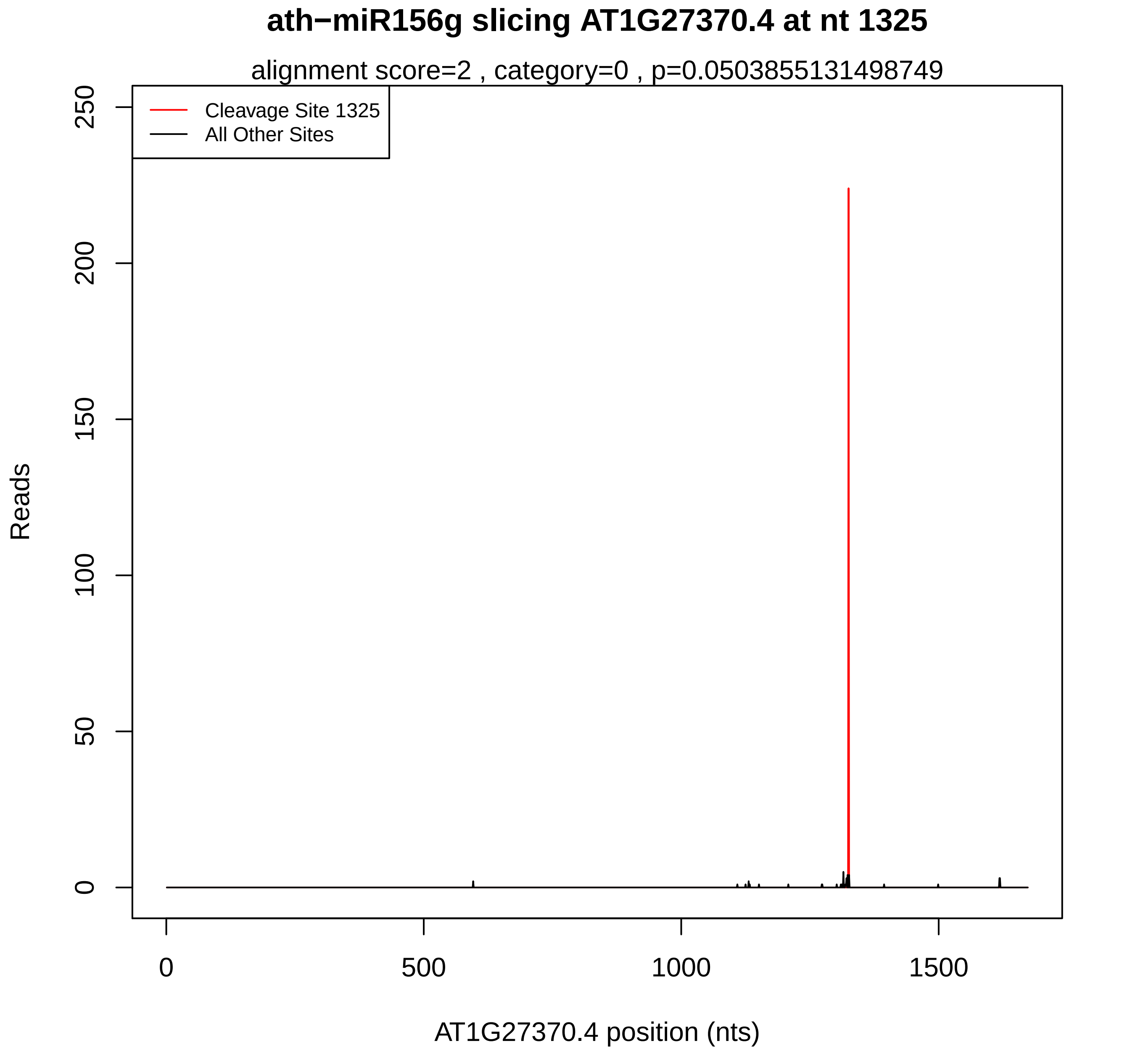

ath−miR156g slicing AT1G27370.4 at nt 1325
alignment score=2 , category=0 , p=0.0503855131498749
Cleavage Site 1325
All Other Sites
Reads
0
50
100
150
200
250
0
500
1000
1500
AT1G27370.4 position (nts)

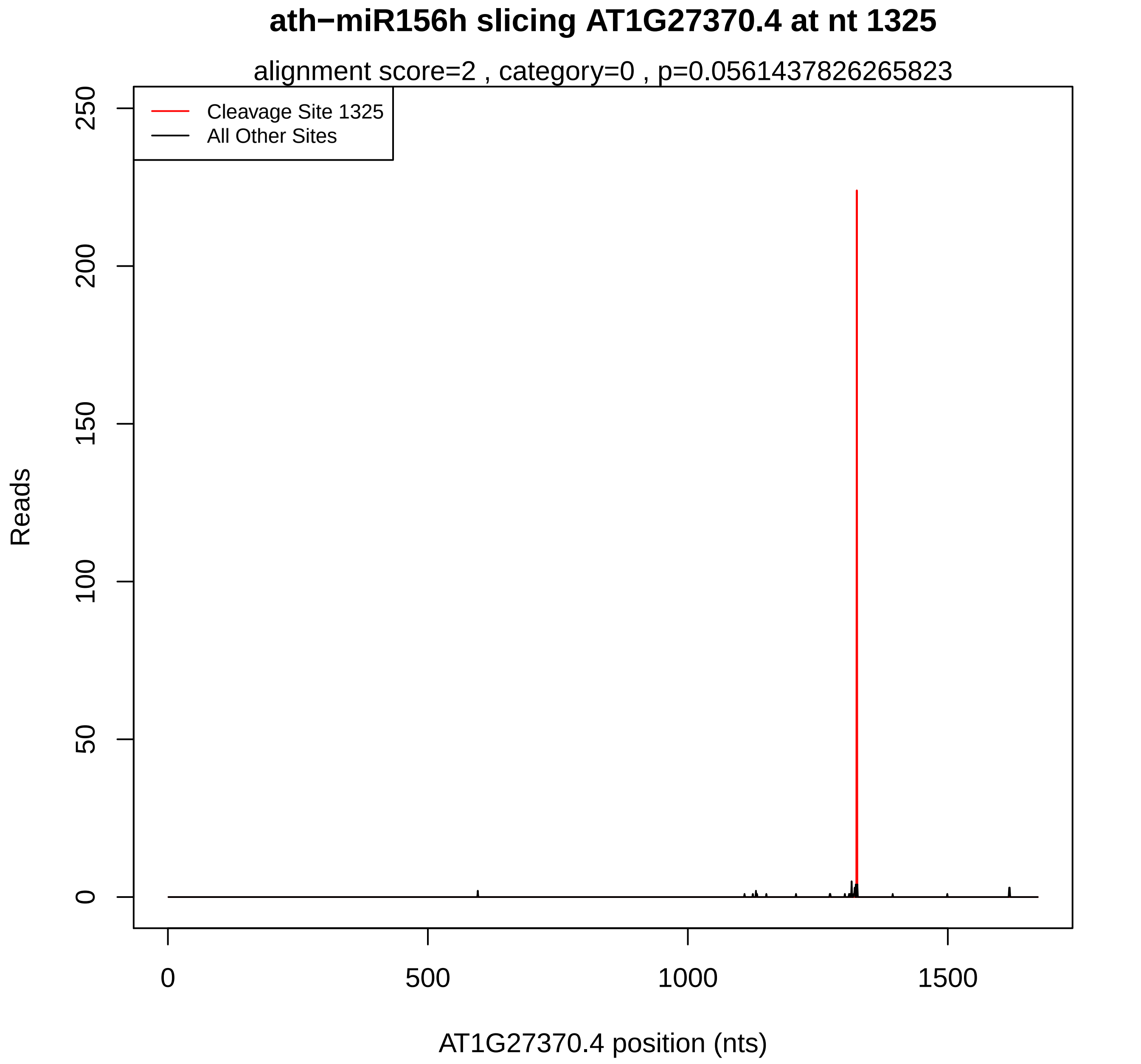

ath−miR156h slicing AT1G27370.4 at nt 1325
alignment score=2 , category=0 , p=0.0561437826265823
Cleavage Site 1325
All Other Sites
Reads
0
50
100
150
200
250
0
500
1000
1500
AT1G27370.4 position (nts)

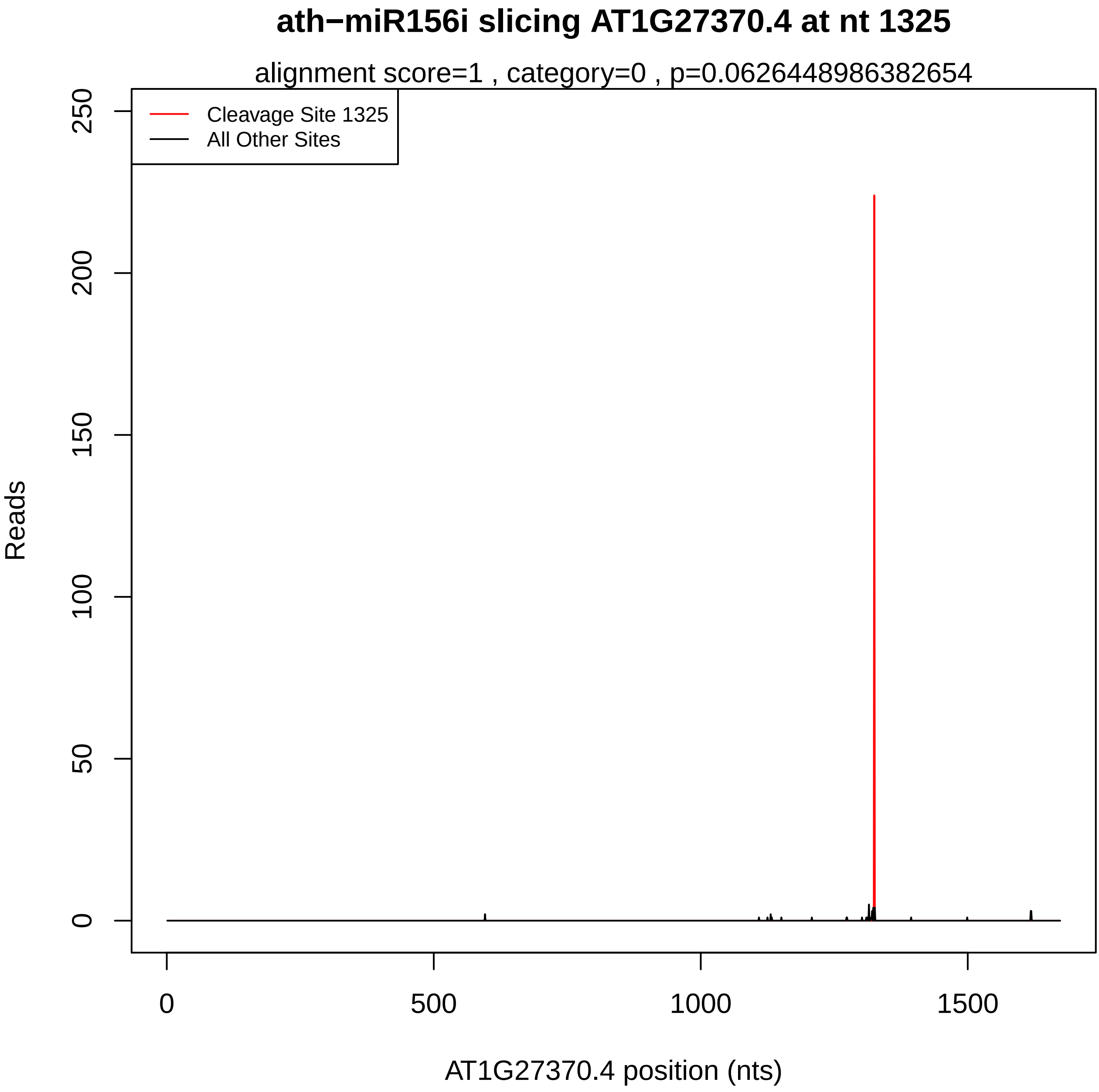

ath−miR156i slicing AT1G27370.4 at nt 1325
alignment score=1 , category=0 , p=0.0626448986382654
Cleavage Site 1325
All Other Sites
Reads
0
50
100
150
200
250
0
500
1000
1500
AT1G27370.4 position (nts)

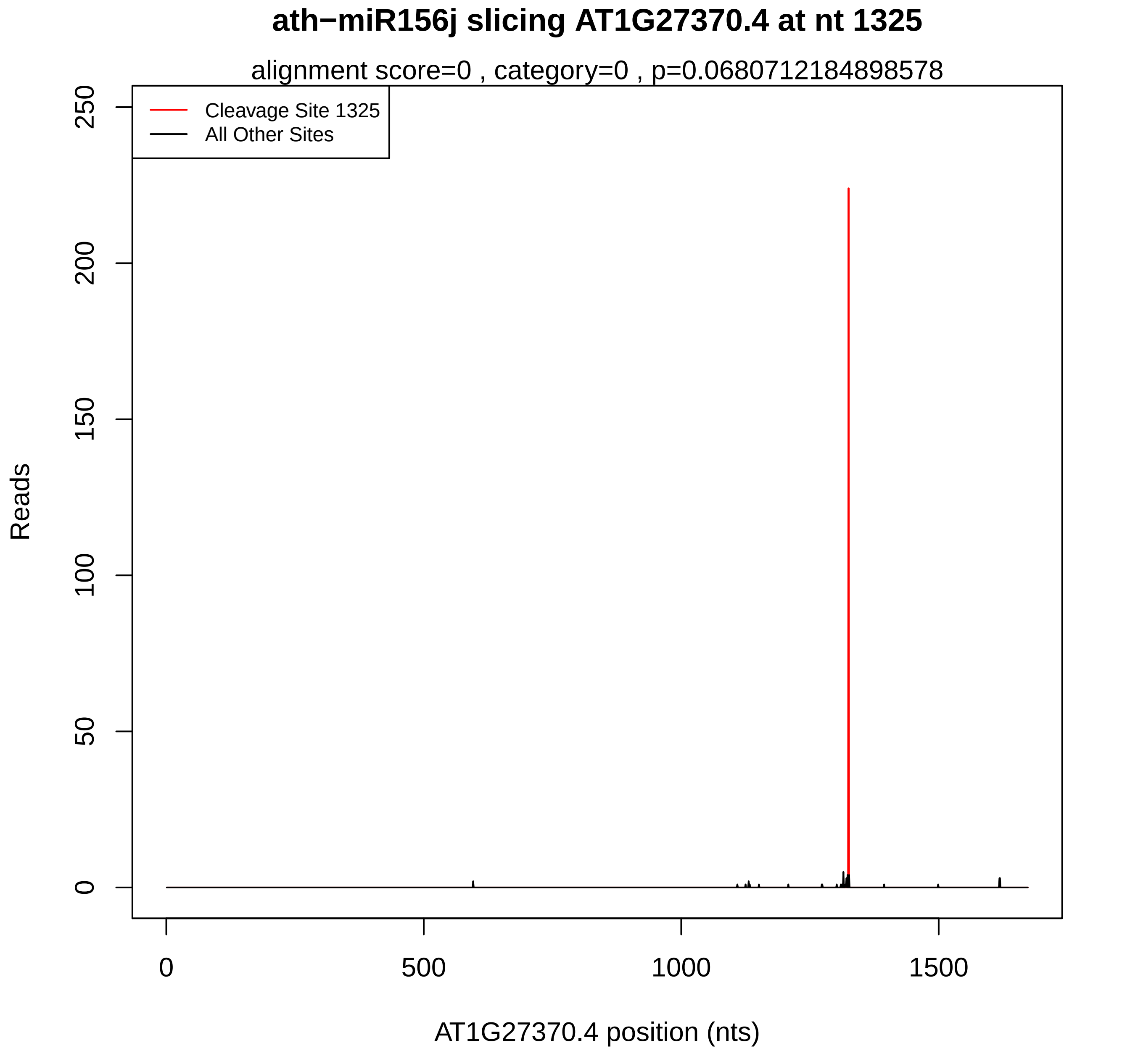
ath−miR156j slicing AT1G27370.4 at nt 1325
alignment score=0 , category=0 , p=0.0680712184898578
Cleavage Site 1325
All Other Sites
Reads
0
50
100
150
200
250
0
500
1000
1500
AT1G27370.4 position (nts)

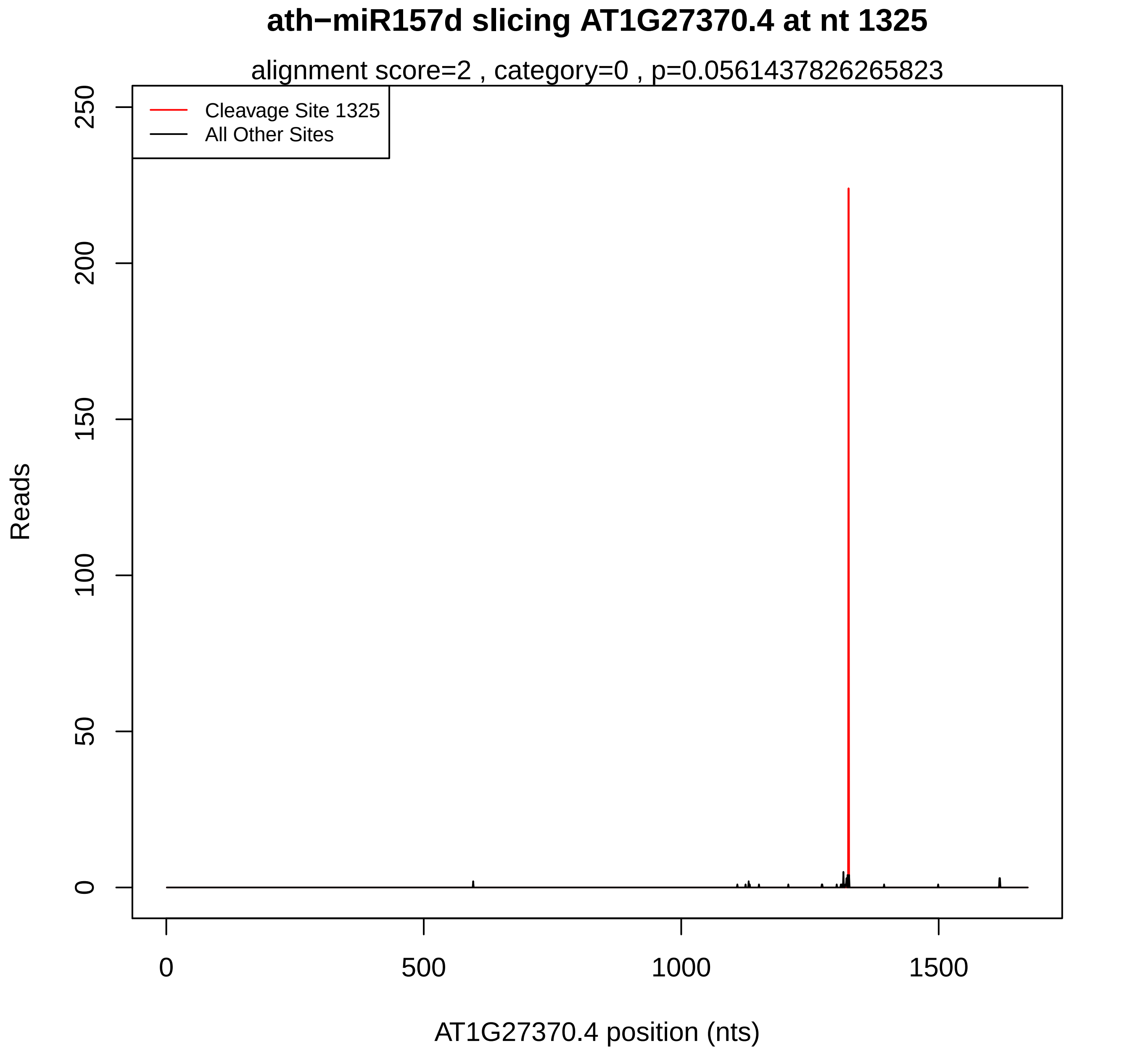

ath−miR157d slicing AT1G27370.4 at nt 1325
alignment score=2 , category=0 , p=0.0561437826265823
Cleavage Site 1325
All Other Sites
Reads
0
50
100
150
200
250
0
500
1000
1500
AT1G27370.4 position (nts)

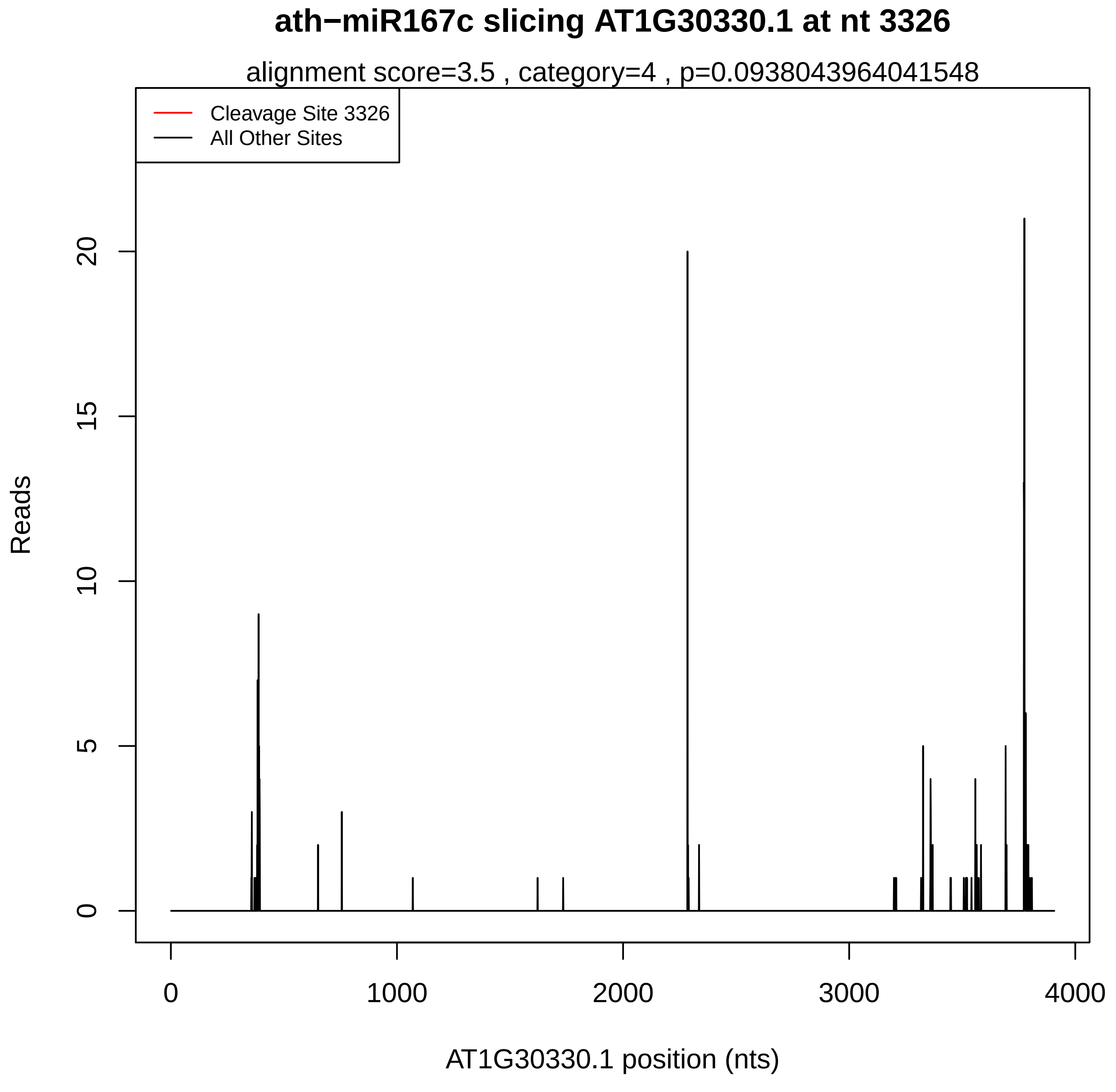
ath−miR167c slicing AT1G30330.1 at nt 3326
alignment score=3.5 , category=4 , p=0.0938043964041548
Cleavage Site 3326
All Other Sites
Reads
0
5
10
15
20
0
1000
2000
3000
4000
AT1G30330.1 position (nts)

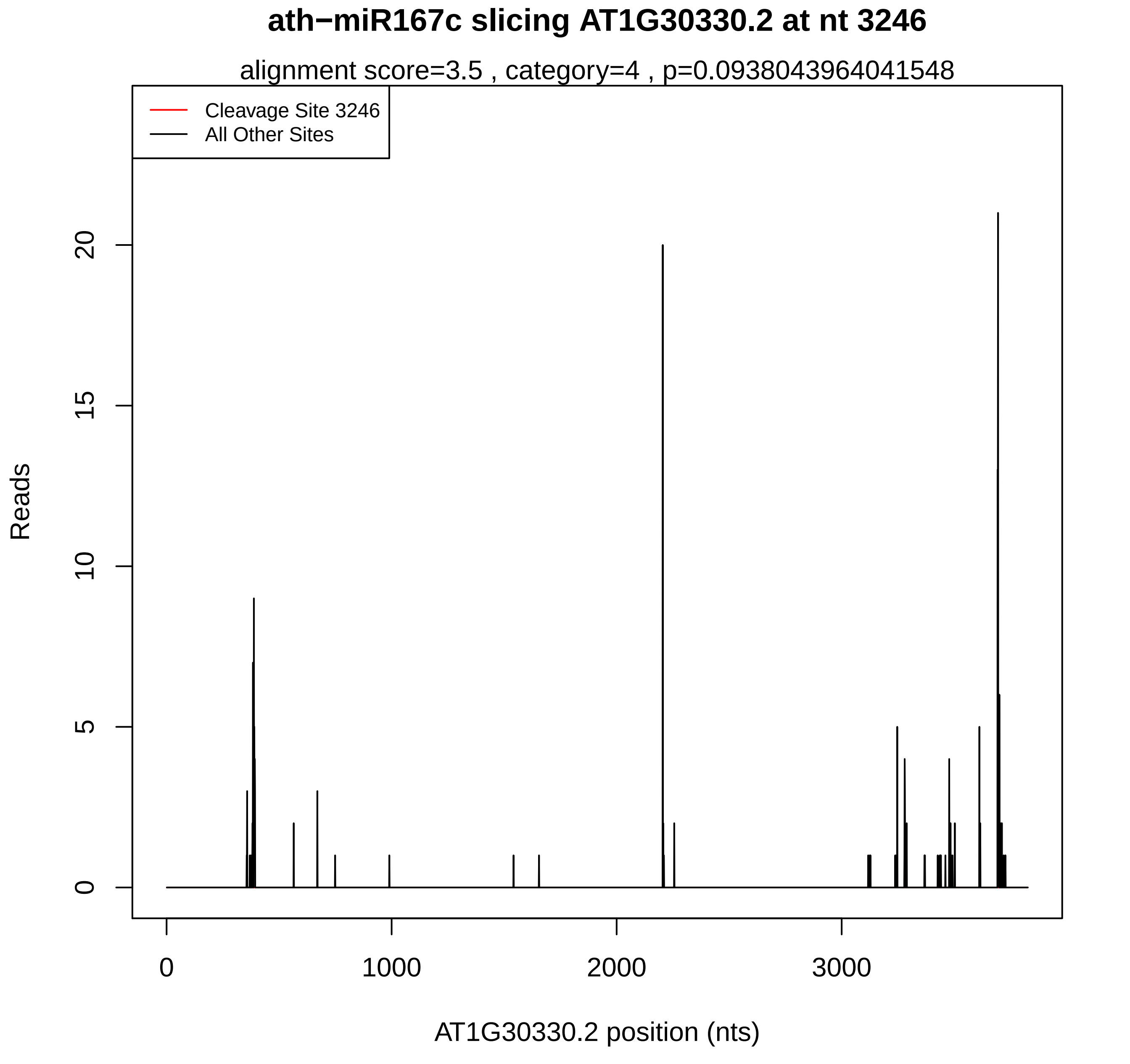

ath−miR167c slicing AT1G30330.2 at nt 3246
alignment score=3.5 , category=4 , p=0.0938043964041548
Cleavage Site 3246
All Other Sites
Reads
0
5
10
15
20
0
1000
2000
3000
AT1G30330.2 position (nts)

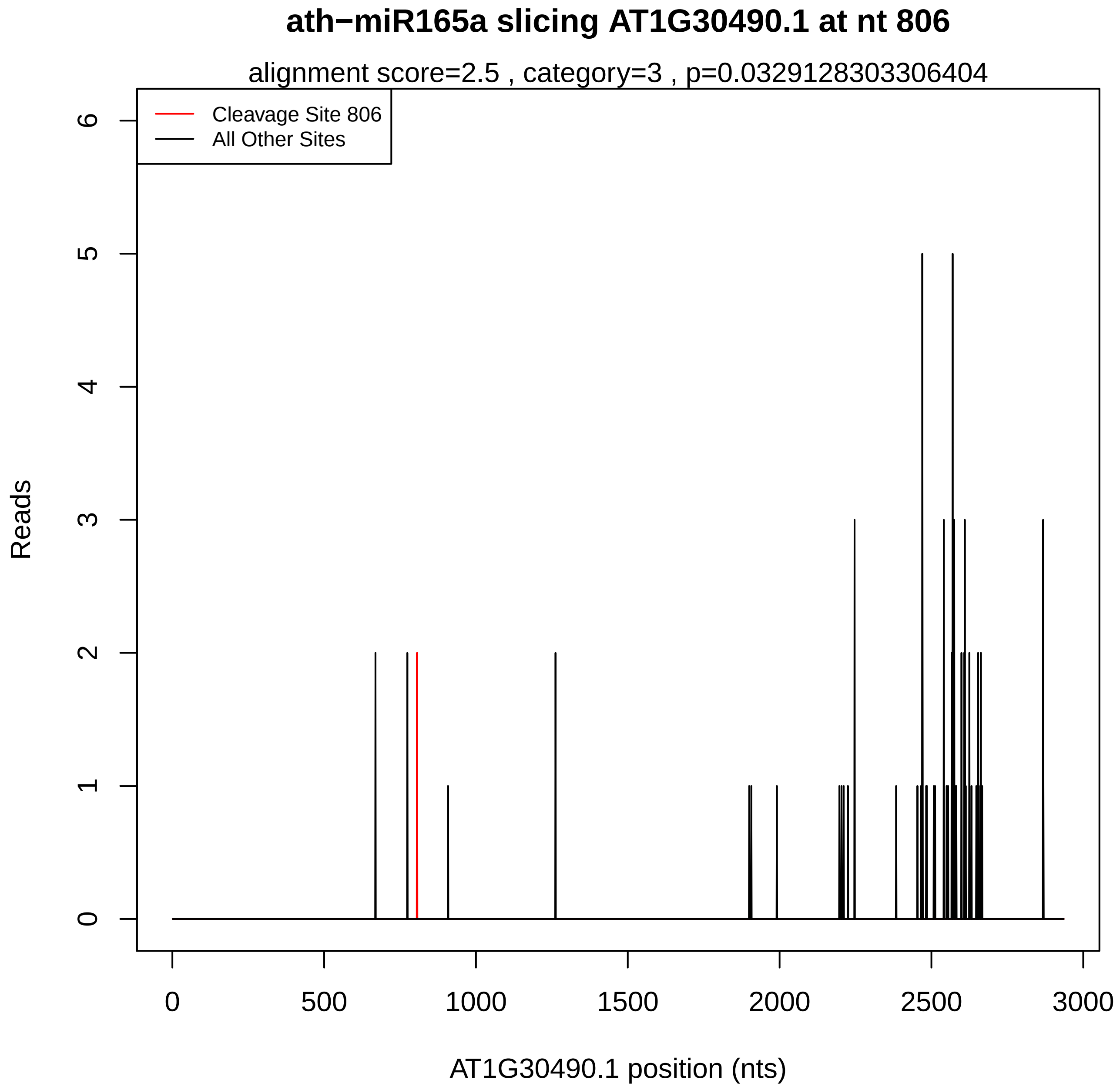
ath−miR165a slicing AT1G30490.1 at nt 806
alignment score=2.5 , category=3 , p=0.0329128303306404
Cleavage Site 806
All Other Sites
Reads
0
1
2
3
4
5
6
0
500
1000
1500
2000
2500
3000
AT1G30490.1 position (nts)

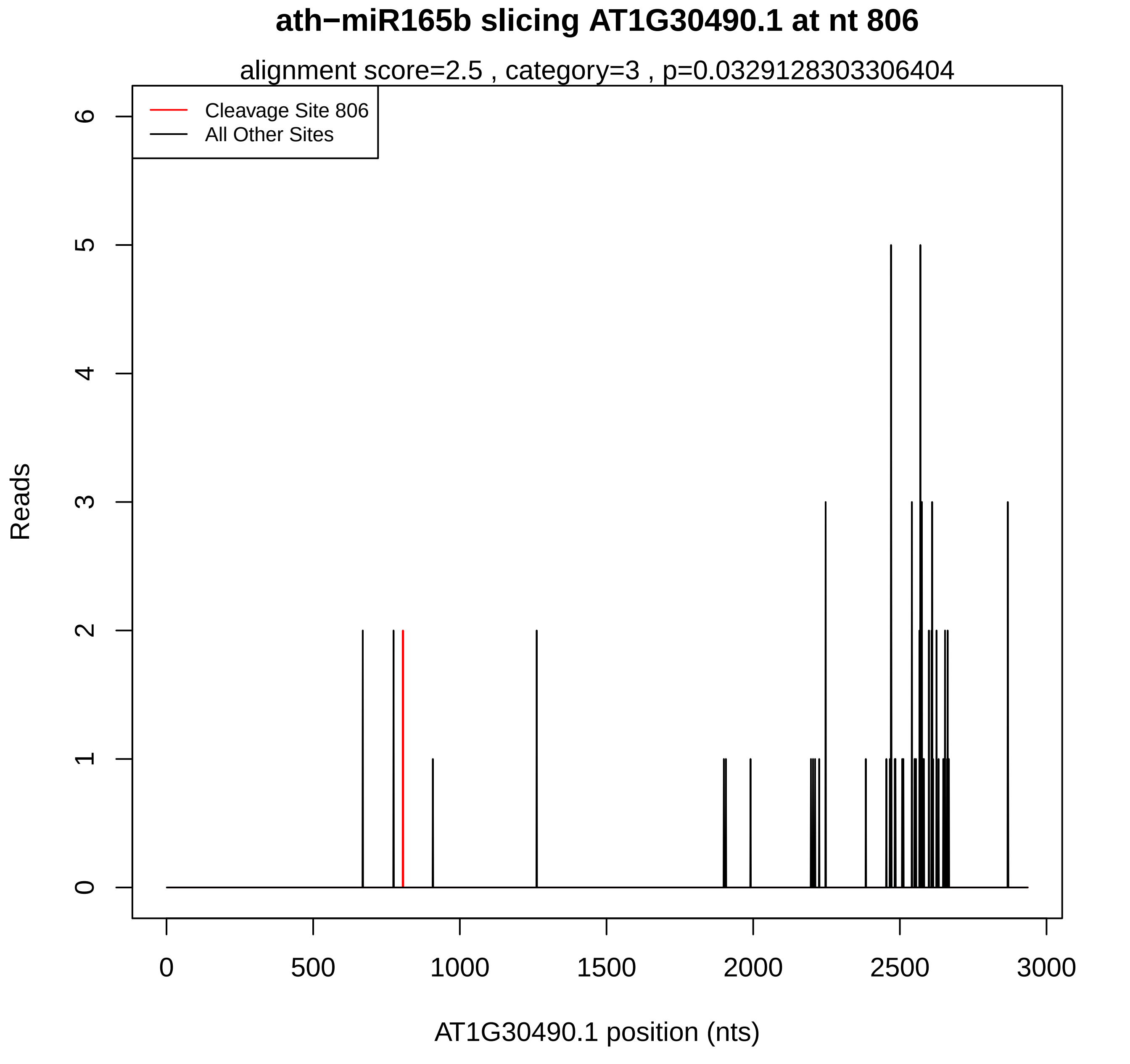
ath−miR165b slicing AT1G30490.1 at nt 806
alignment score=2.5 , category=3 , p=0.0329128303306404
Cleavage Site 806
All Other Sites
Reads
0
1
2
3
4
5
6
0
500
1000
1500
2000
2500
3000
AT1G30490.1 position (nts)

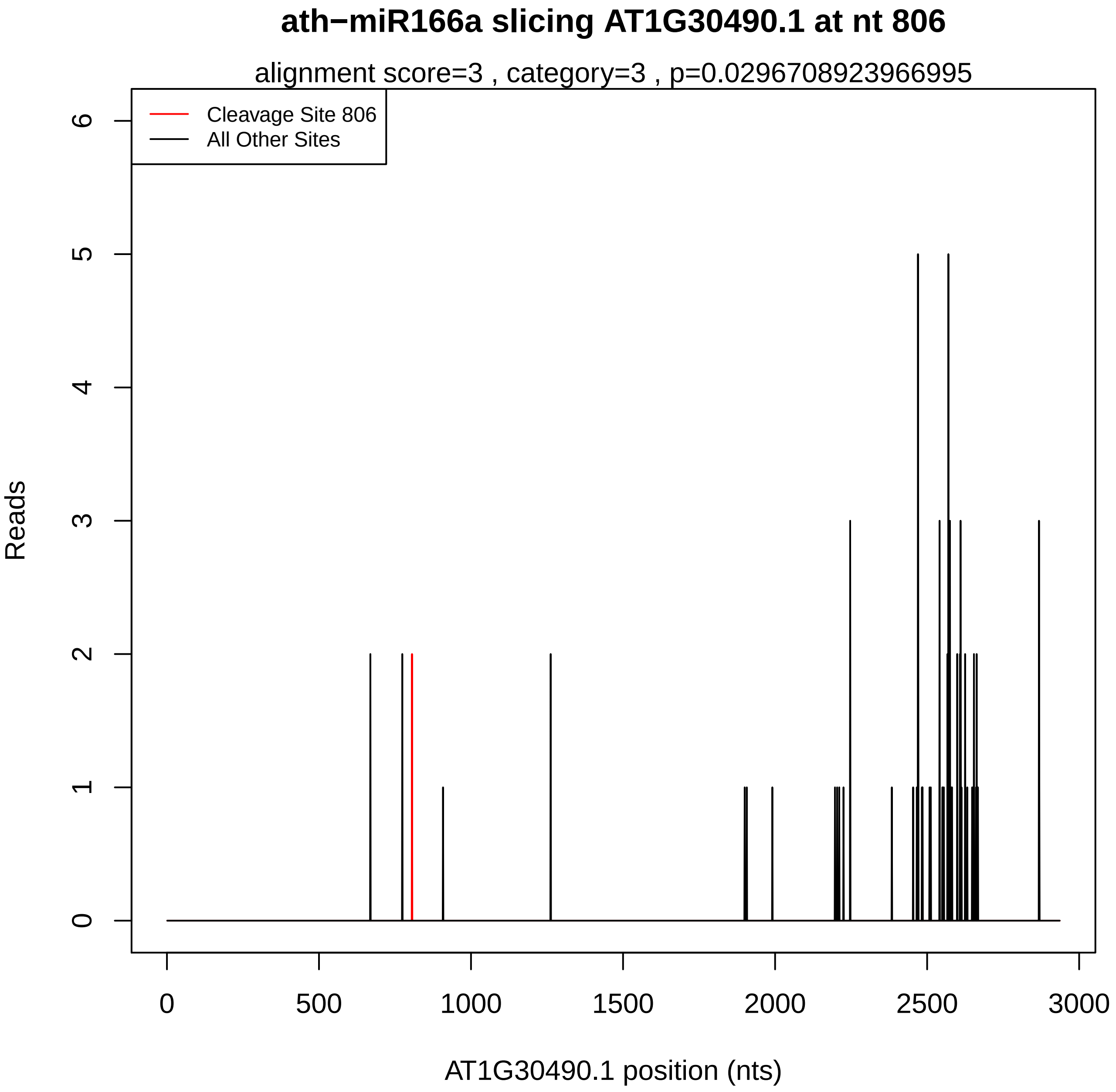
ath−miR166a slicing AT1G30490.1 at nt 806
alignment score=3 , category=3 , p=0.0296708923966995
Cleavage Site 806
All Other Sites
Reads
0
1
2
3
4
5
6
0
500
1000
1500
2000
2500
3000
AT1G30490.1 position (nts)

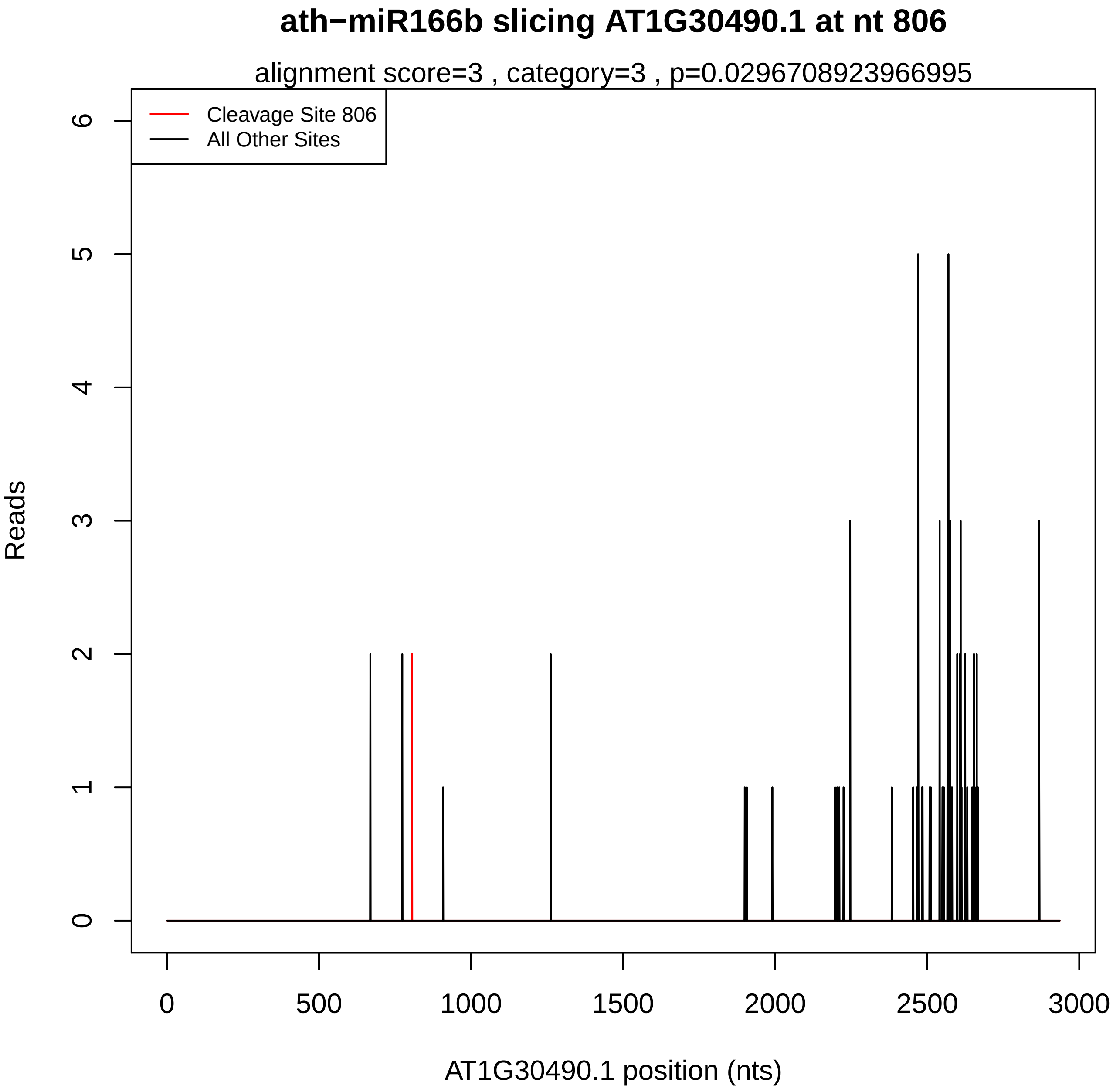

ath−miR166b slicing AT1G30490.1 at nt 806
alignment score=3 , category=3 , p=0.0296708923966995
Cleavage Site 806
All Other Sites
Reads
0
1
2
3
4
5
6
0
500
1000
1500
2000
2500
3000
AT1G30490.1 position (nts)

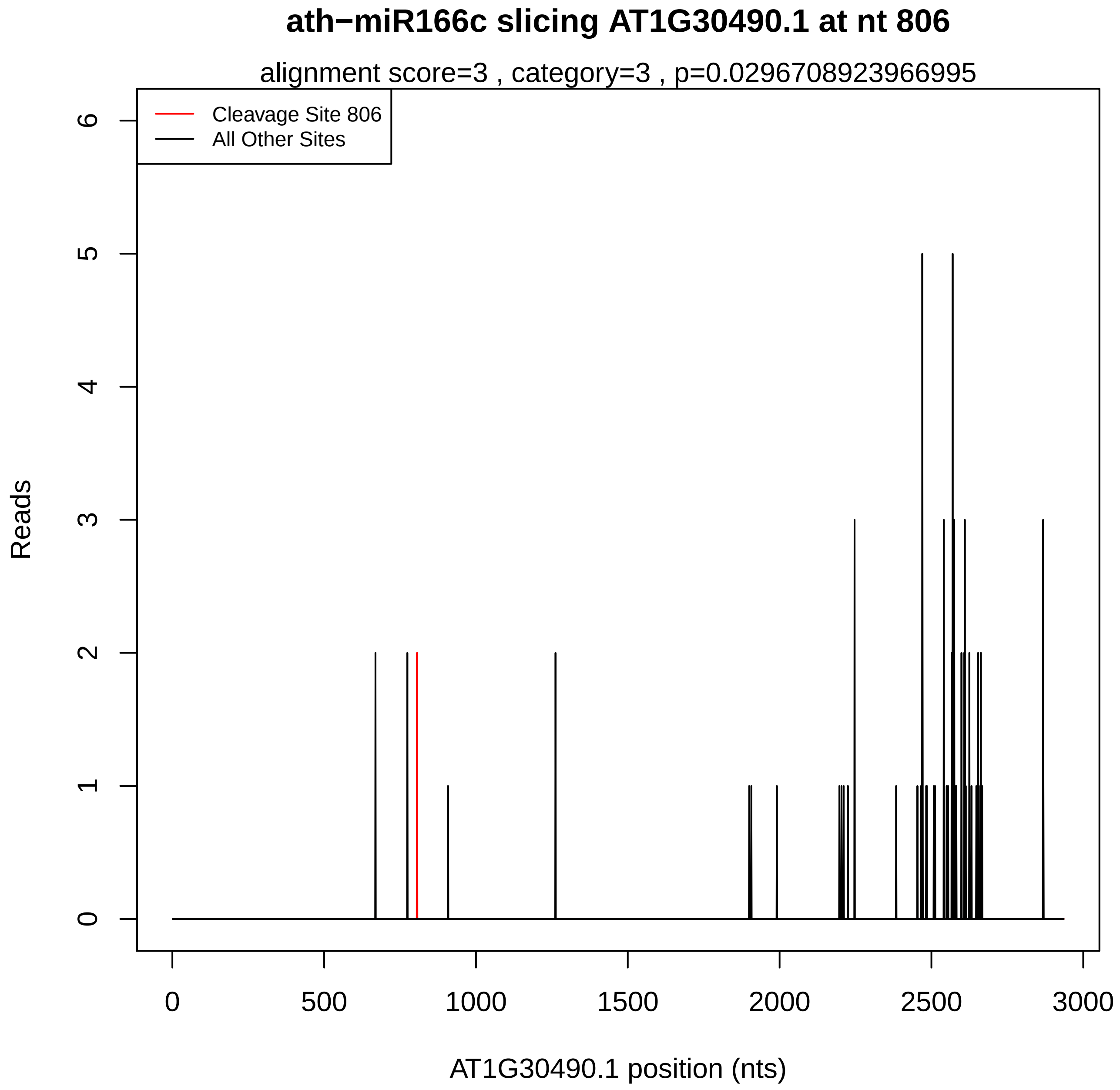

ath−miR166c slicing AT1G30490.1 at nt 806
alignment score=3 , category=3 , p=0.0296708923966995
Cleavage Site 806
All Other Sites
Reads
0
1
2
3
4
5
6
0
500
1000
1500
2000
2500
3000
AT1G30490.1 position (nts)

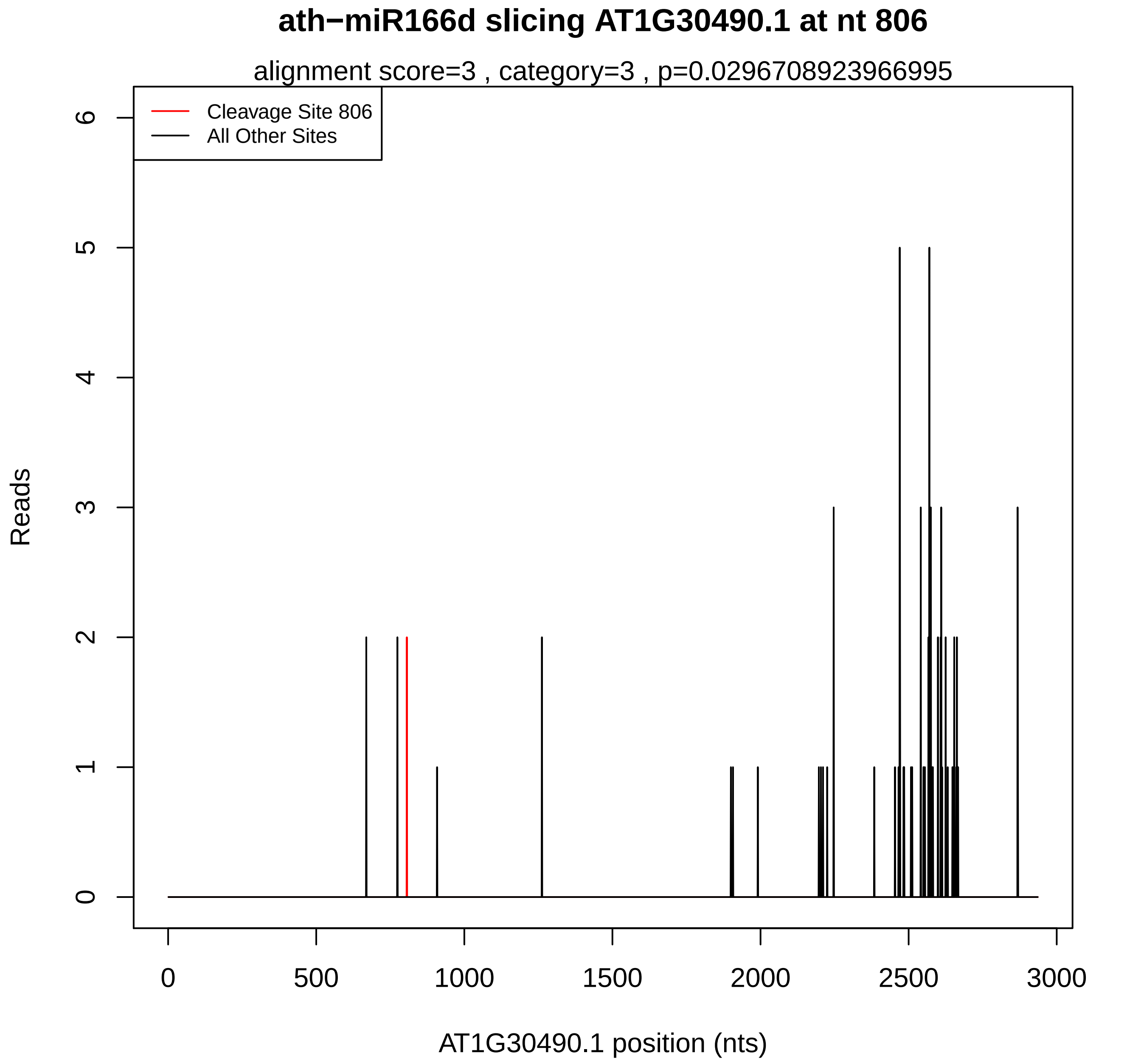

ath−miR166d slicing AT1G30490.1 at nt 806
alignment score=3 , category=3 , p=0.0296708923966995
Cleavage Site 806
All Other Sites
Reads
0
1
2
3
4
5
6
0
500
1000
1500
2000
2500
3000
AT1G30490.1 position (nts)

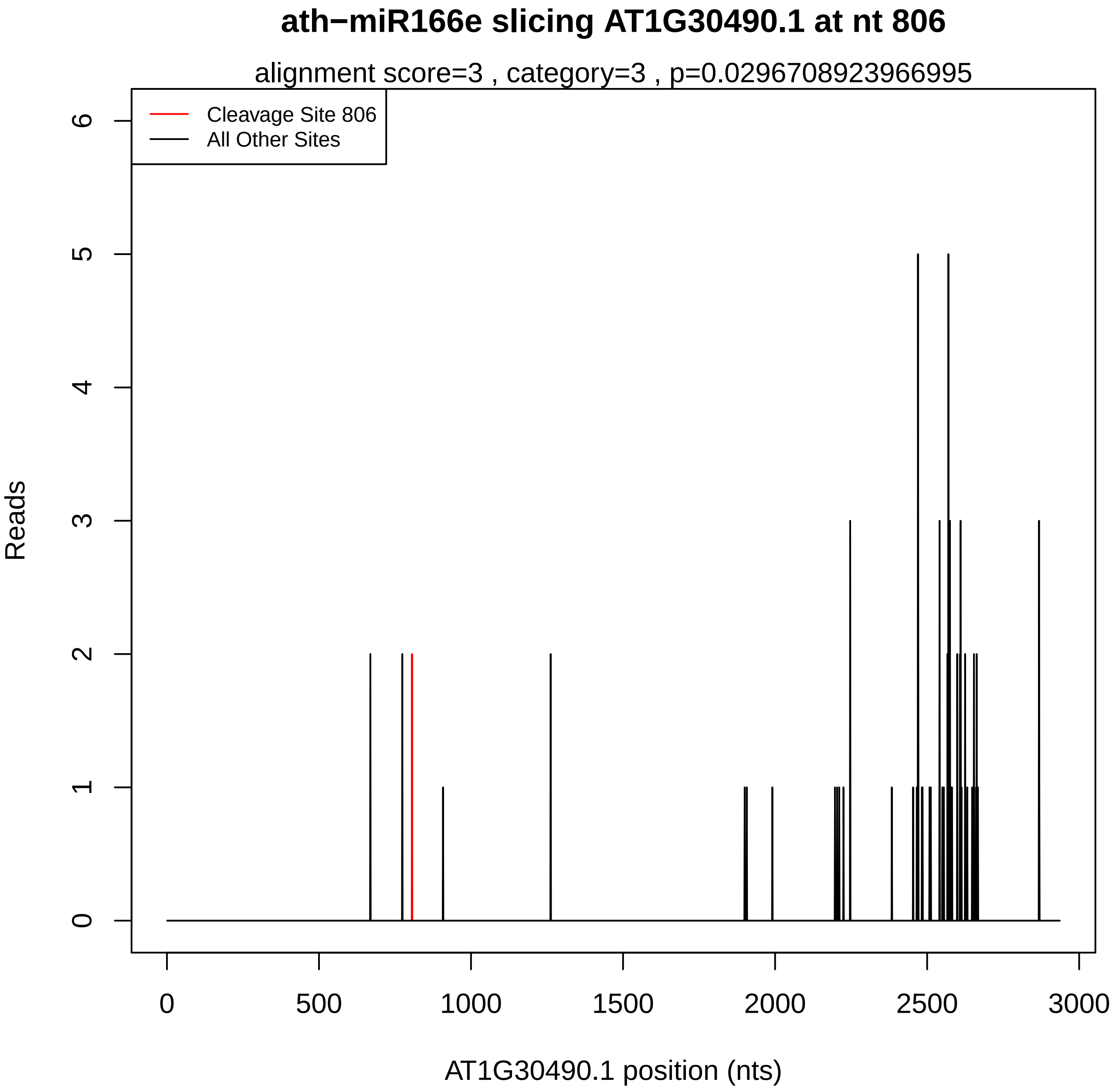
ath−miR166e slicing AT1G30490.1 at nt 806
alignment score=3 , category=3 , p=0.0296708923966995
Cleavage Site 806
All Other Sites
Reads
0
1
2
3
4
5
6
0
500
1000
1500
2000
2500
3000
AT1G30490.1 position (nts)

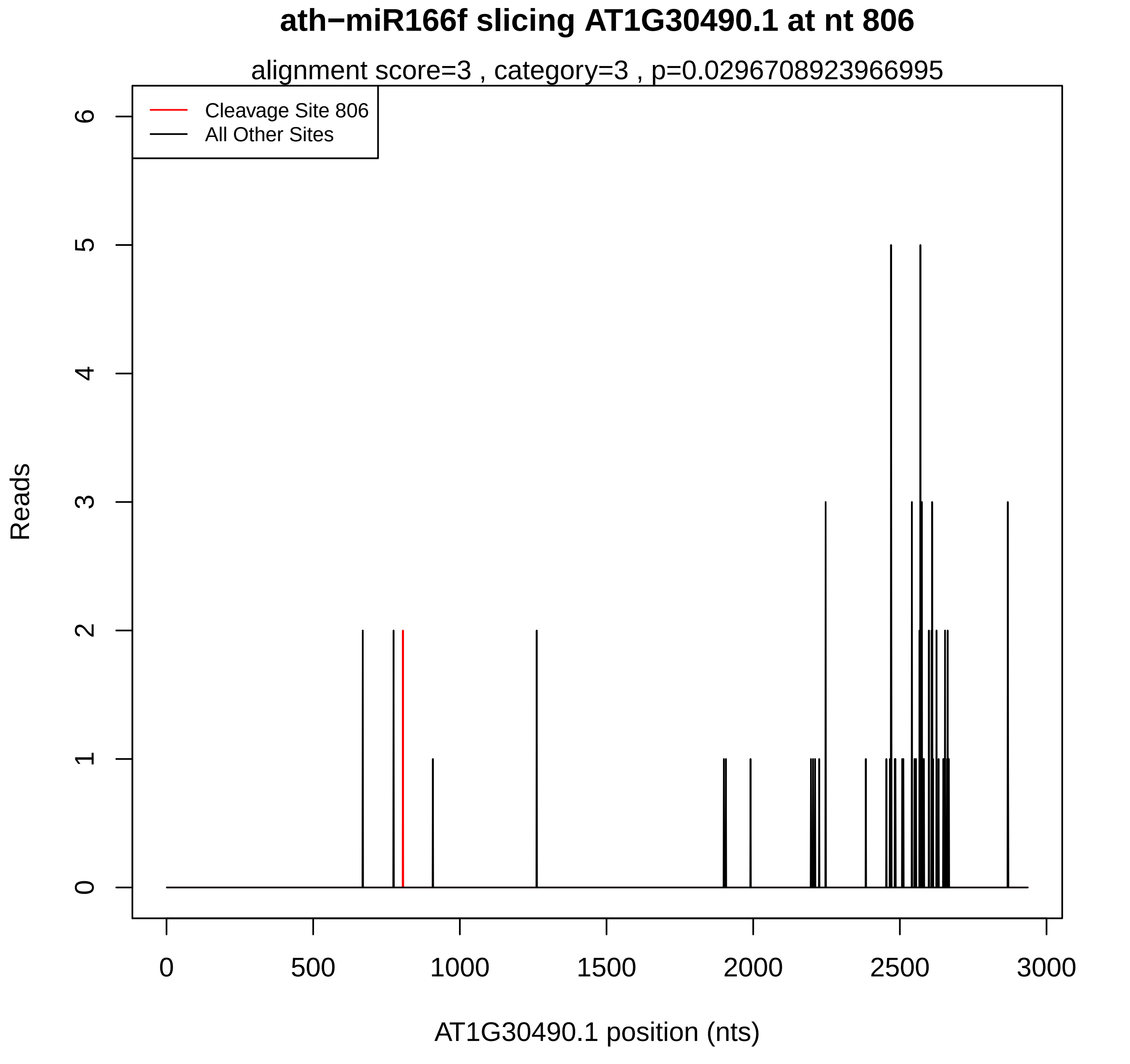
ath−miR166f slicing AT1G30490.1 at nt 806
alignment score=3 , category=3 , p=0.0296708923966995
Cleavage Site 806
All Other Sites
Reads
0
1
2
3
4
5
6
0
500
1000
1500
2000
2500
3000
AT1G30490.1 position (nts)

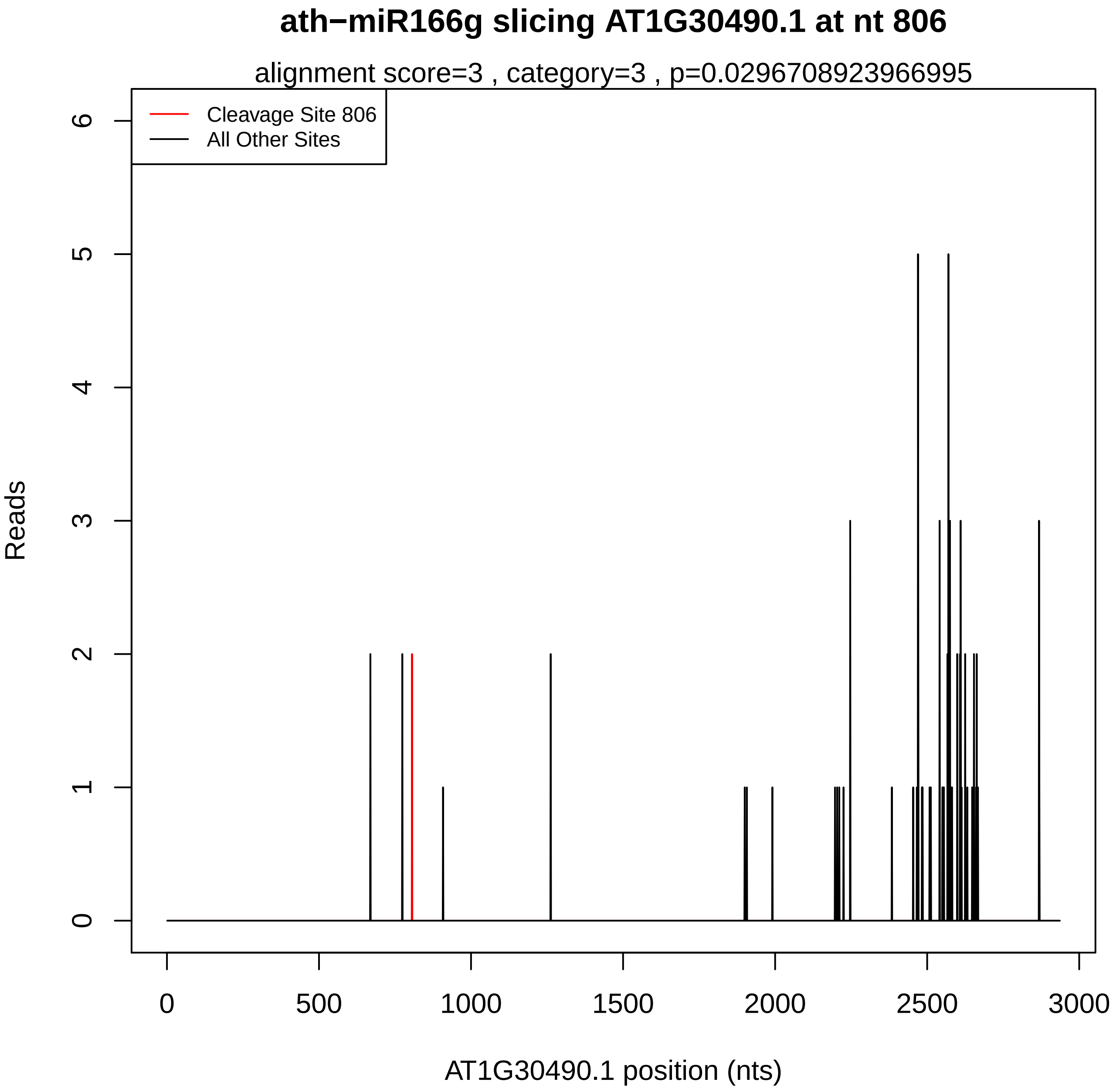
ath−miR166g slicing AT1G30490.1 at nt 806
alignment score=3 , category=3 , p=0.0296708923966995
Cleavage Site 806
All Other Sites
Reads
0
1
2
3
4
5
6
0
500
1000
1500
2000
2500
3000
AT1G30490.1 position (nts)

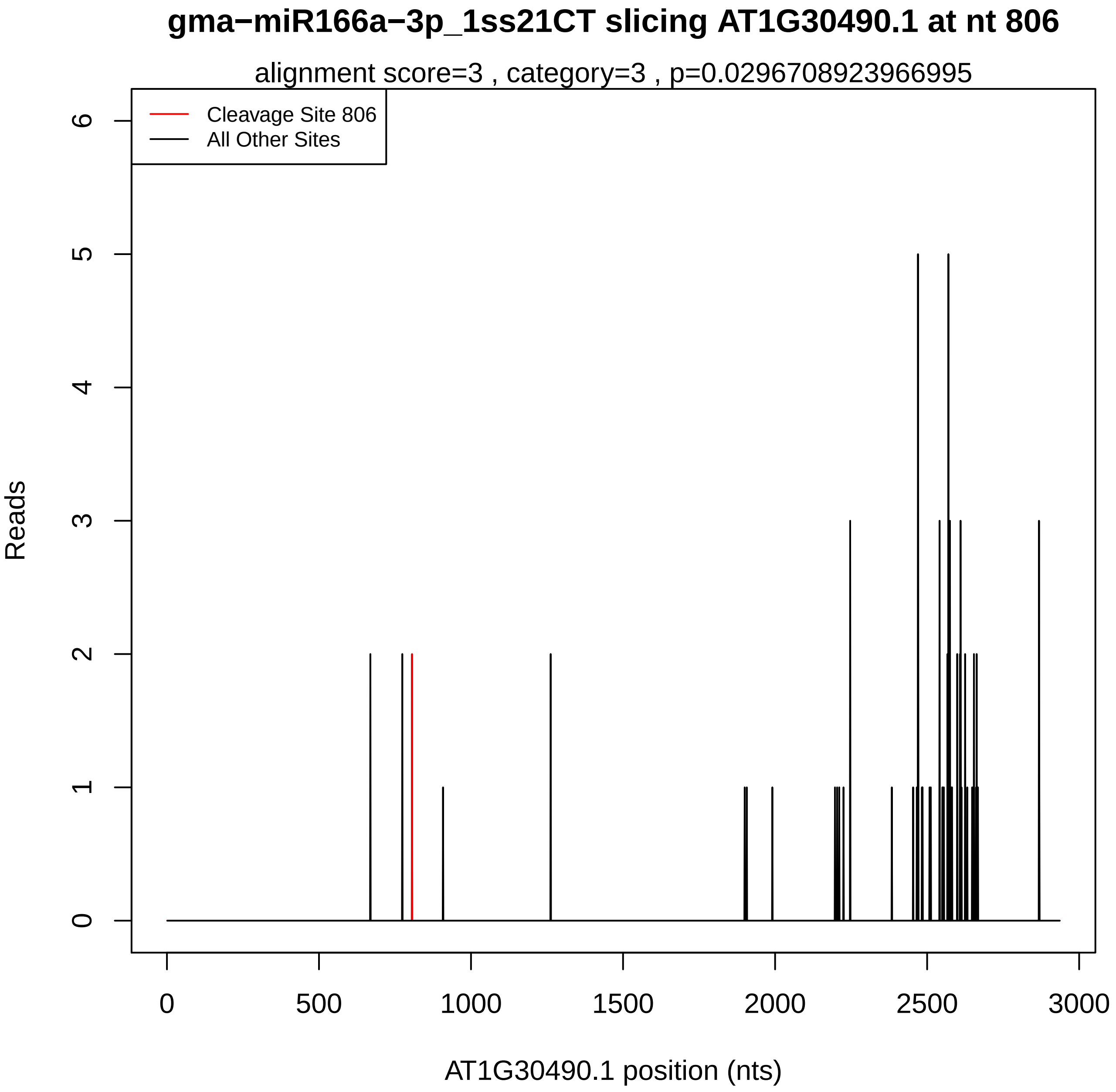
gma−miR166a−3p_1ss21CT slicing AT1G30490.1 at nt 806
alignment score=3 , category=3 , p=0.0296708923966995
Cleavage Site 806
All Other Sites
Reads
0
1
2
3
4
5
6
0
500
1000
1500
2000
2500
3000
AT1G30490.1 position (nts)

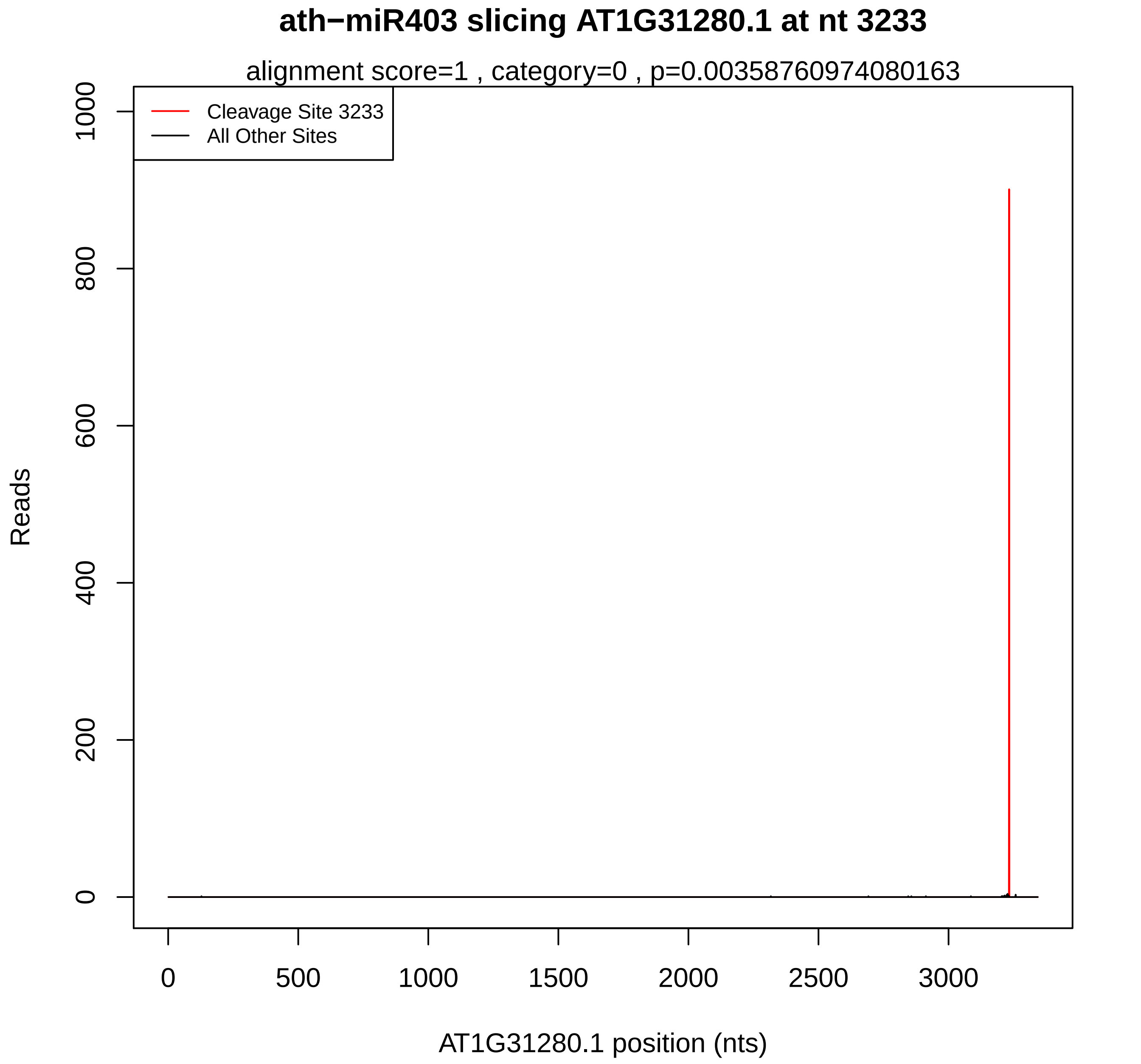

ath−miR403 slicing AT1G31280.1 at nt 3233
alignment score=1 , category=0 , p=0.00358760974080163
Cleavage Site 3233
All Other Sites
Reads
0
200
400
600
800
1000
0
500
1000
1500
2000
2500
3000
AT1G31280.1 position (nts)

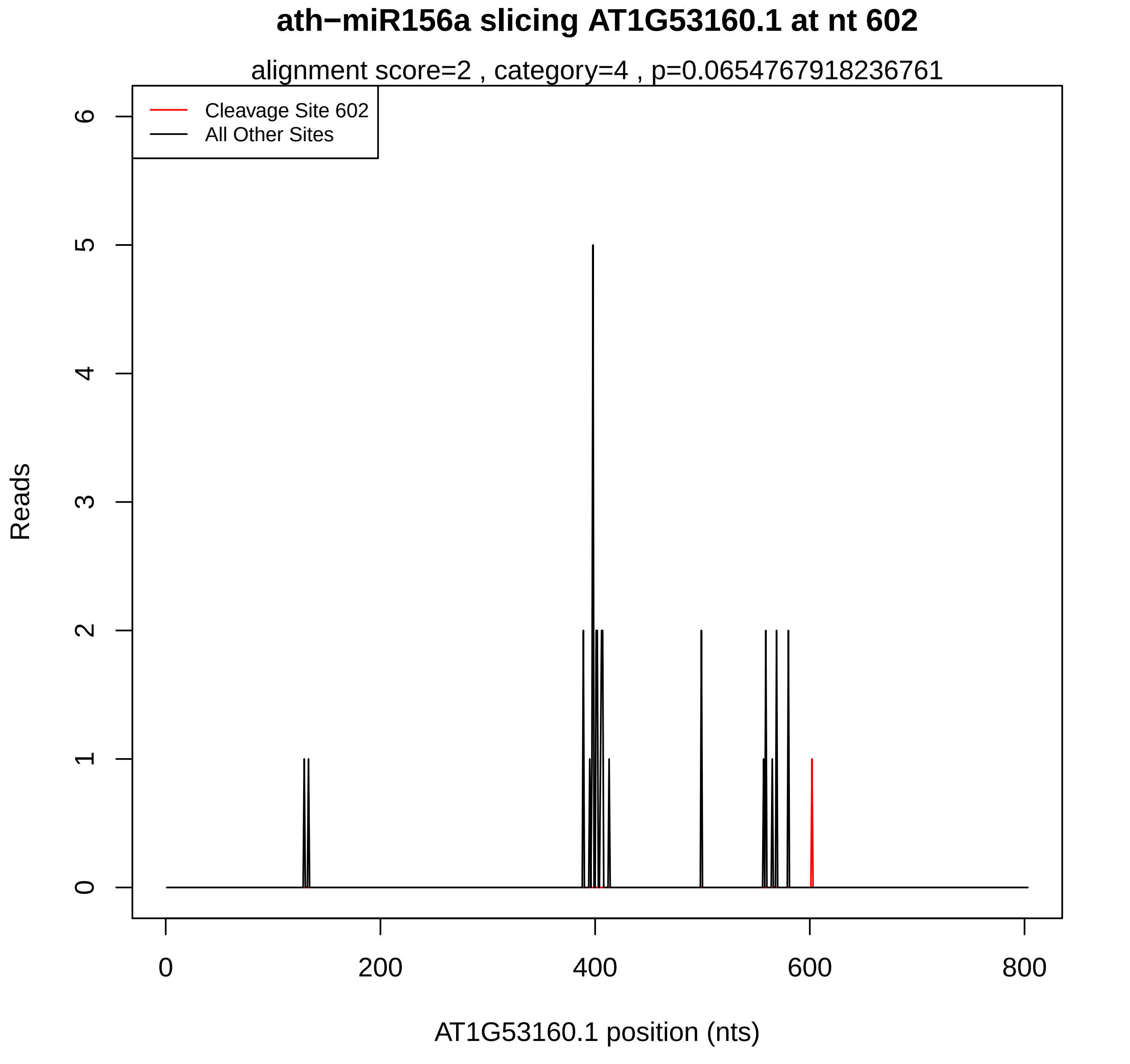

ath−miR156a slicing AT1G53160.1 at nt 602
alignment score=2 , category=4 , p=0.0654767918236761
Cleavage Site 602
All Other Sites
Reads
0
1
2
3
4
5
6
0
200
400
600
800
AT1G53160.1 position (nts)

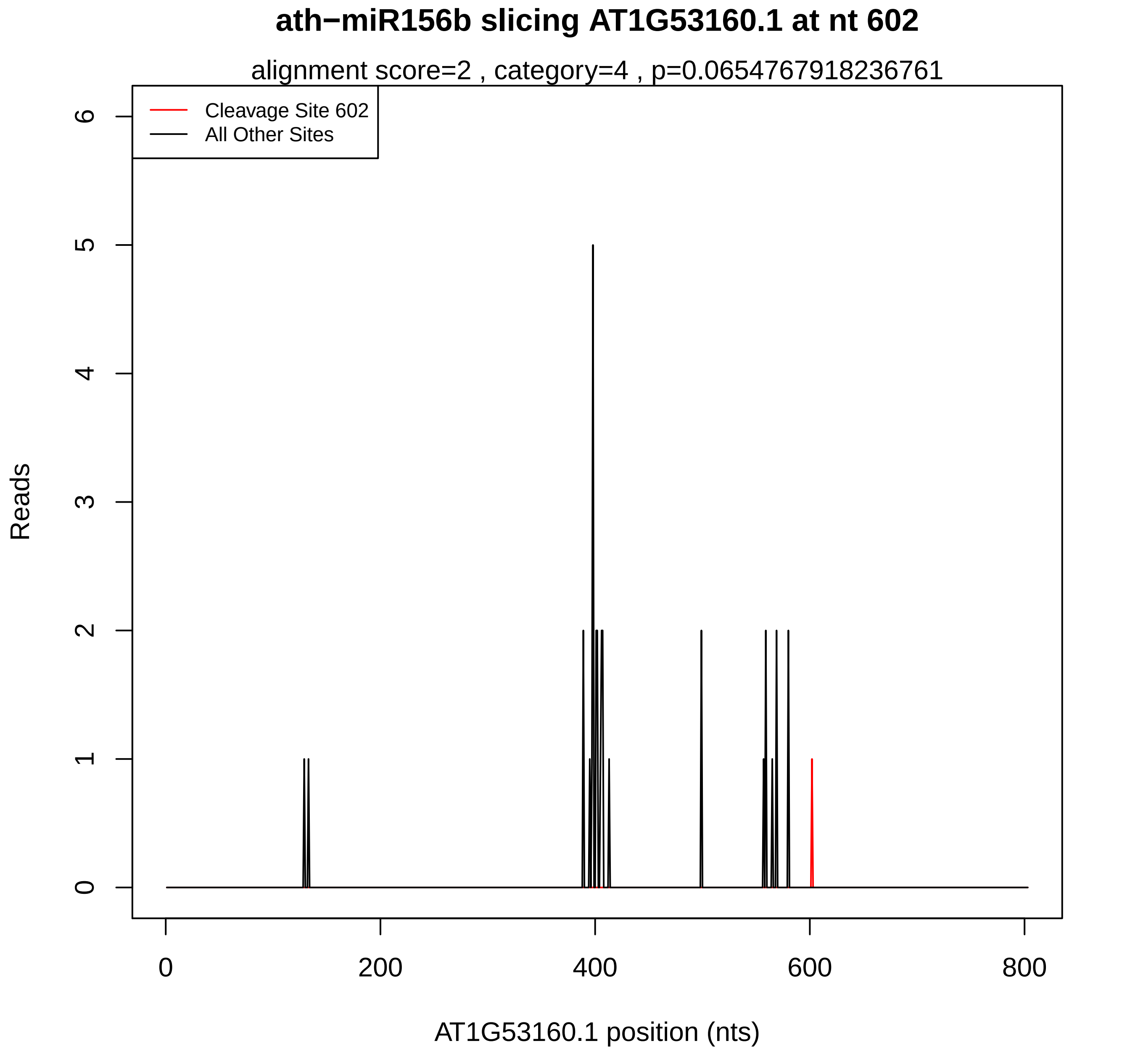
ath−miR156b slicing AT1G53160.1 at nt 602
alignment score=2 , category=4 , p=0.0654767918236761
Cleavage Site 602
All Other Sites
Reads
0
1
2
3
4
5
6
0
200
400
600
800
AT1G53160.1 position (nts)

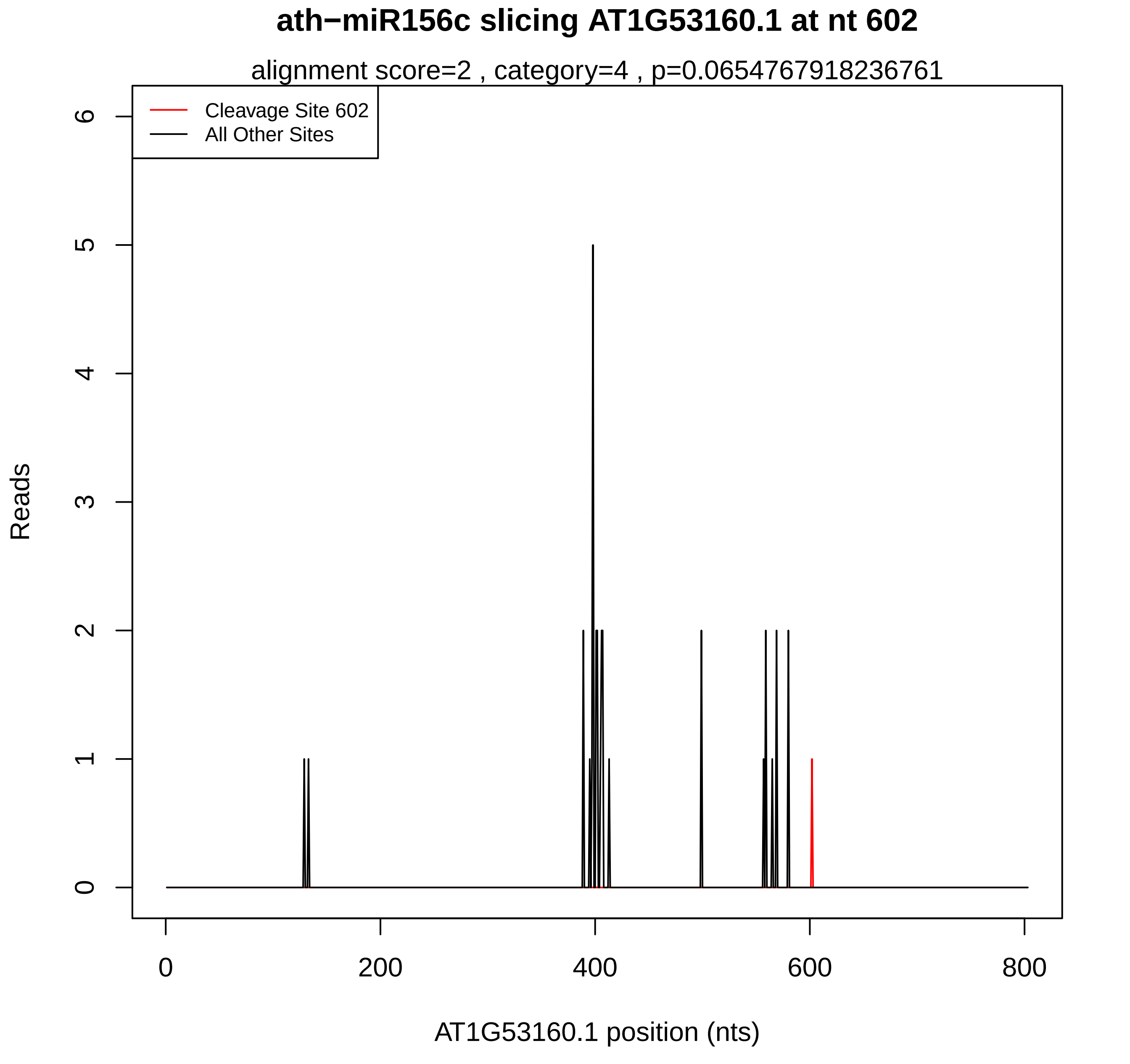
ath−miR156c slicing AT1G53160.1 at nt 602
alignment score=2 , category=4 , p=0.0654767918236761
Cleavage Site 602
All Other Sites
Reads
0
1
2
3
4
5
6
0
200
400
600
800
AT1G53160.1 position (nts)

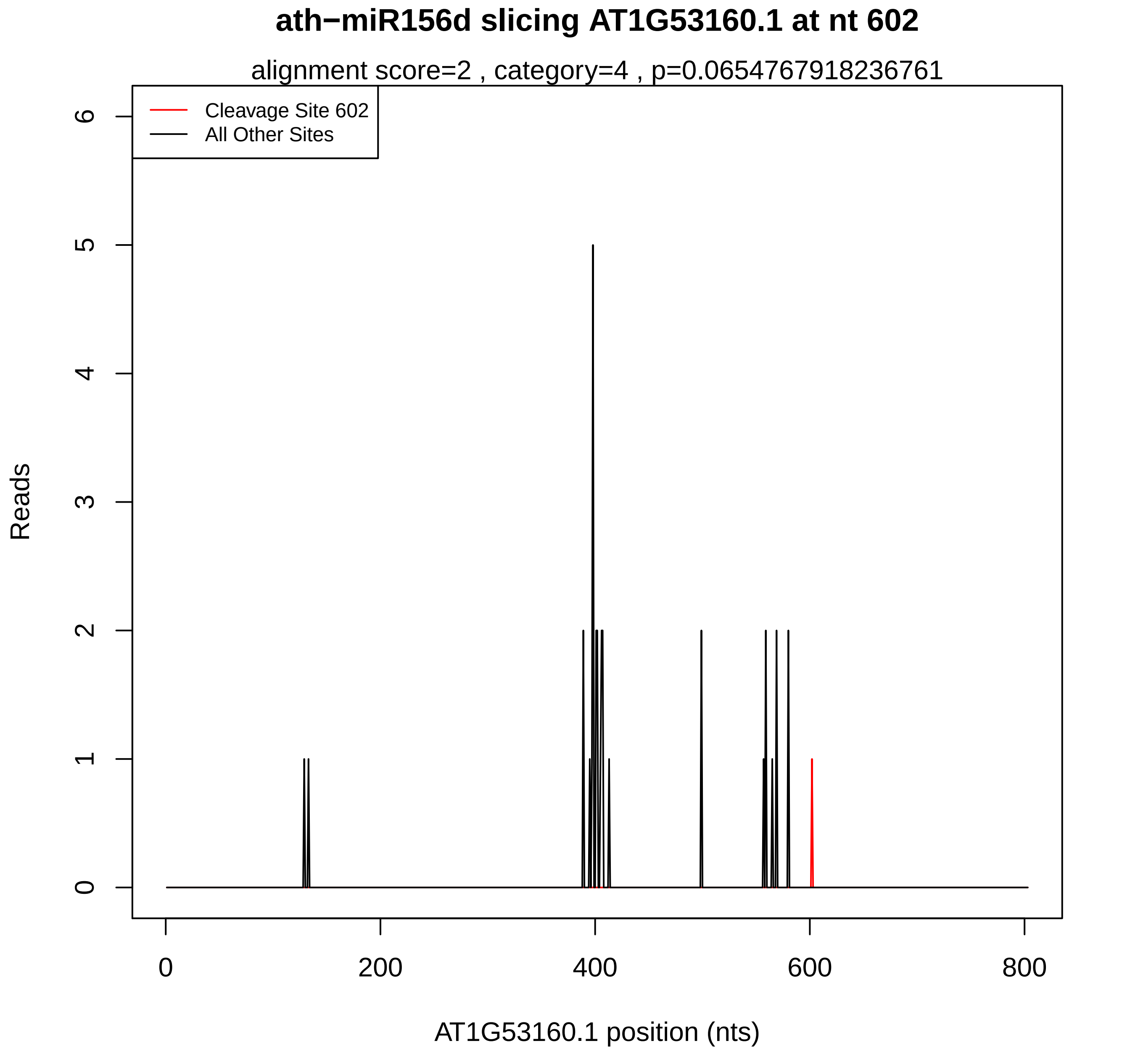
ath−miR156d slicing AT1G53160.1 at nt 602
alignment score=2 , category=4 , p=0.0654767918236761
Cleavage Site 602
All Other Sites
Reads
AT1G53160.1 position (nts)
0
1
2
3
4
5
6
0
200
400
600
800

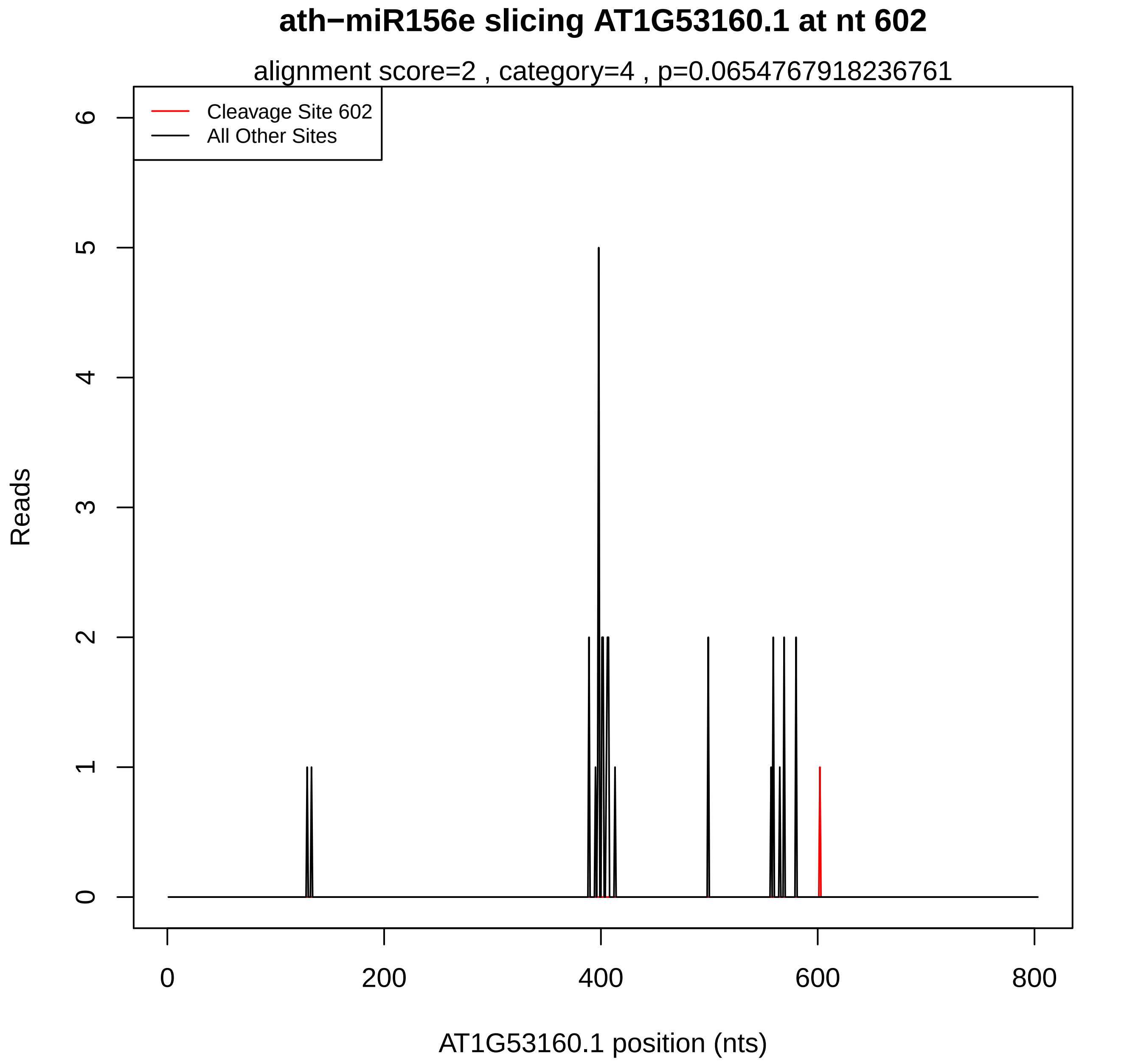
ath−miR156e slicing AT1G53160.1 at nt 602
alignment score=2 , category=4 , p=0.0654767918236761
Cleavage Site 602
All Other Sites
Reads
0
1
2
3
4
5
6
0
200
400
600
800
AT1G53160.1 position (nts)

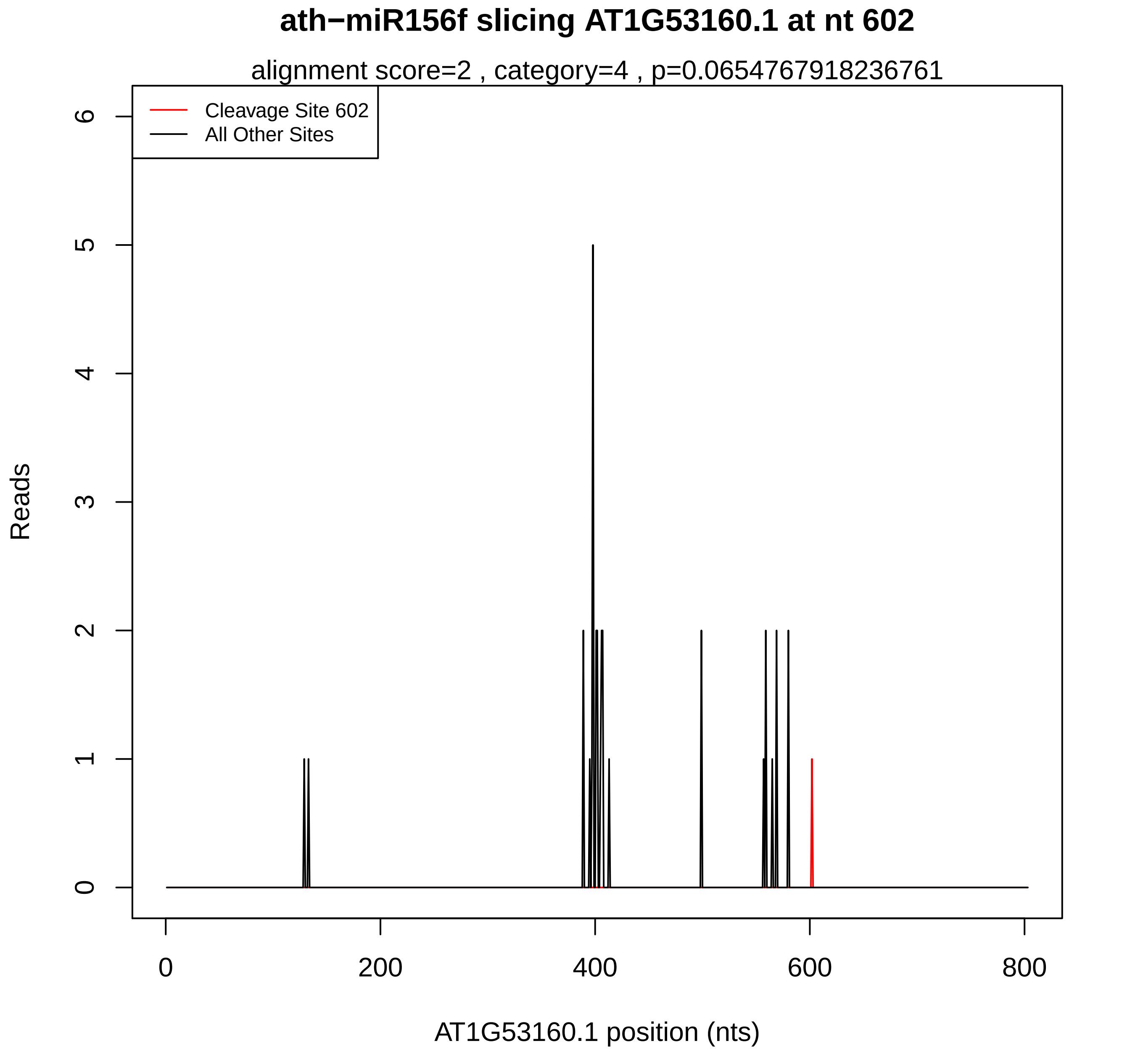
ath−miR156f slicing AT1G53160.1 at nt 602
alignment score=2 , category=4 , p=0.0654767918236761
Cleavage Site 602
All Other Sites
Reads
AT1G53160.1 position (nts)
0
1
2
3
4
5
6
0
200
400
600
800

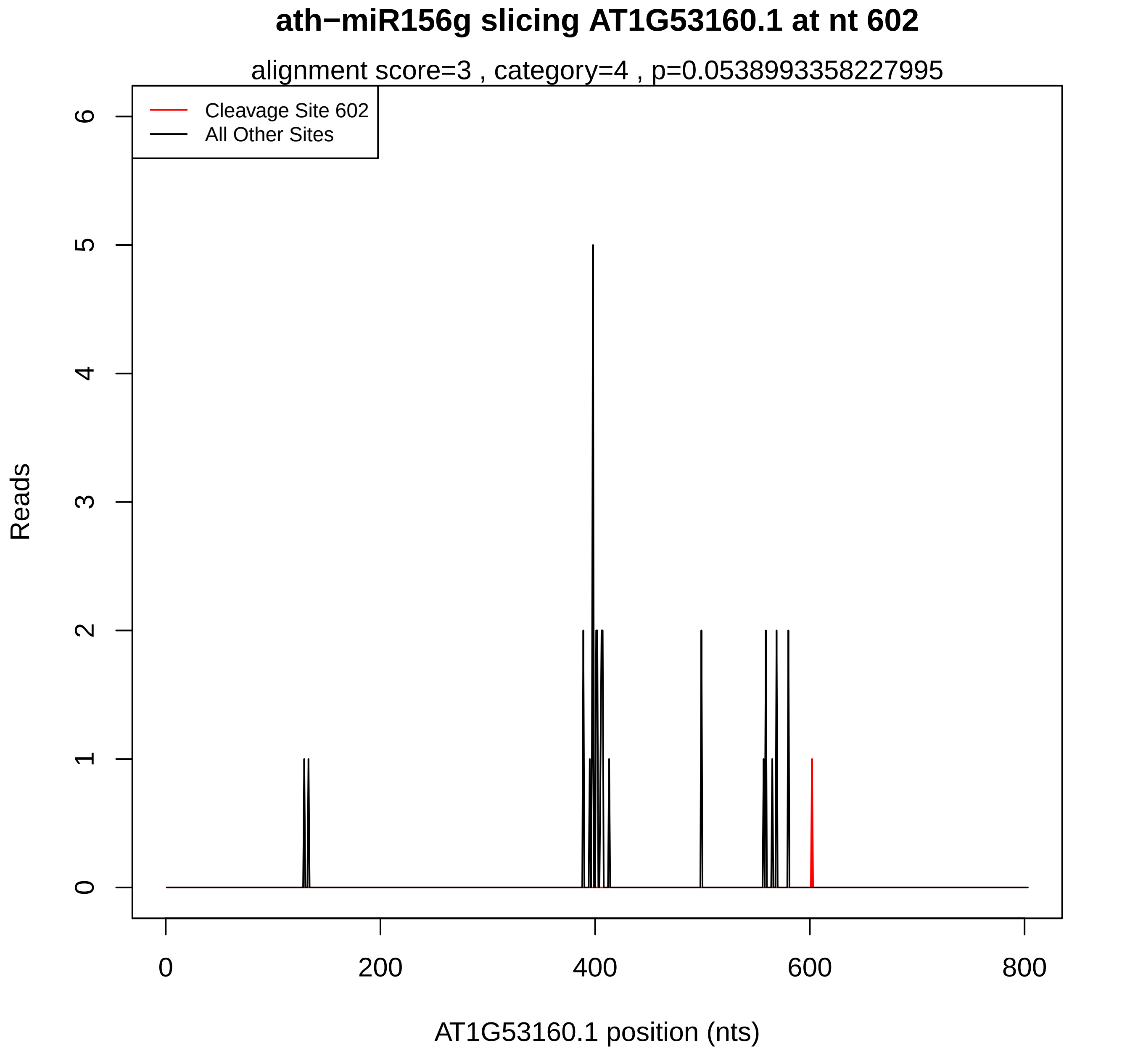
ath−miR156g slicing AT1G53160.1 at nt 602
alignment score=3 , category=4 , p=0.0538993358227995
Cleavage Site 602
All Other Sites
Reads
0
1
2
3
4
5
6
0
200
400
600
800
AT1G53160.1 position (nts)

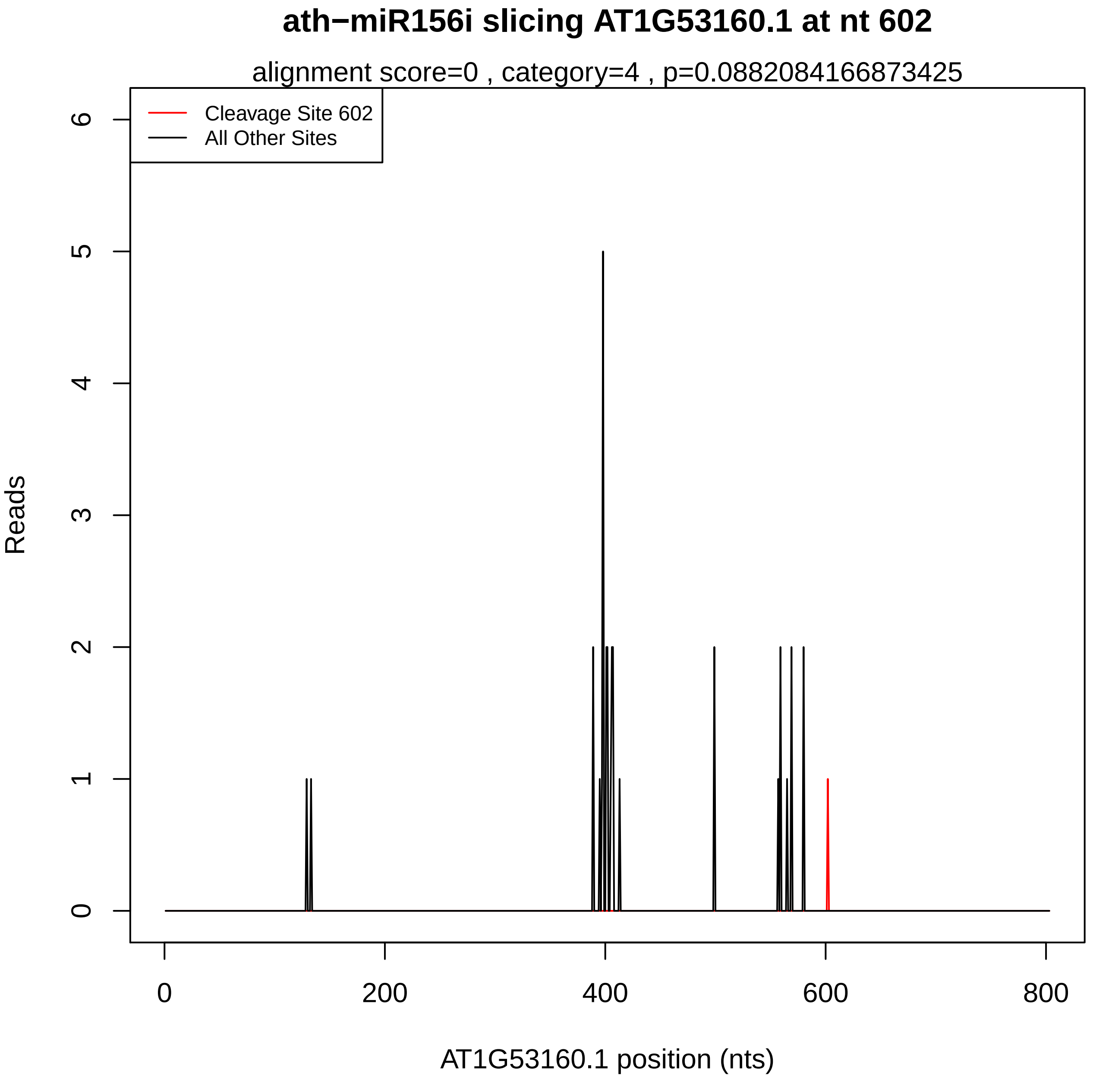
ath−miR156i slicing AT1G53160.1 at nt 602
alignment score=0 , category=4 , p=0.0882084166873425
Cleavage Site 602
All Other Sites
Reads
0
1
2
3
4
5
6
0
200
400
600
800
AT1G53160.1 position (nts)

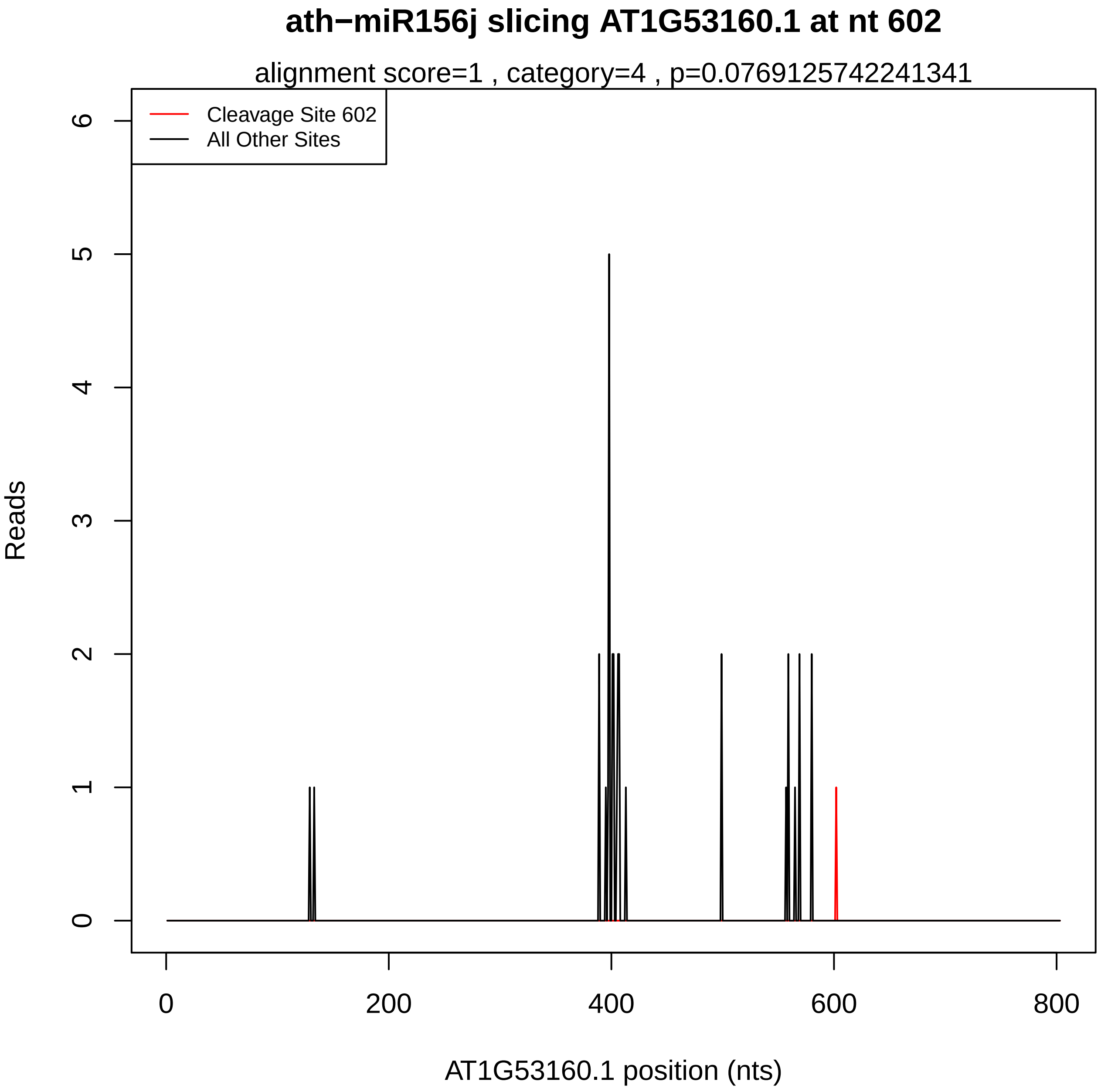

ath−miR156j slicing AT1G53160.1 at nt 602
alignment score=1 , category=4 , p=0.0769125742241341
Cleavage Site 602
All Other Sites
Reads
0
1
2
3
4
5
6
0
200
400
600
800
AT1G53160.1 position (nts)

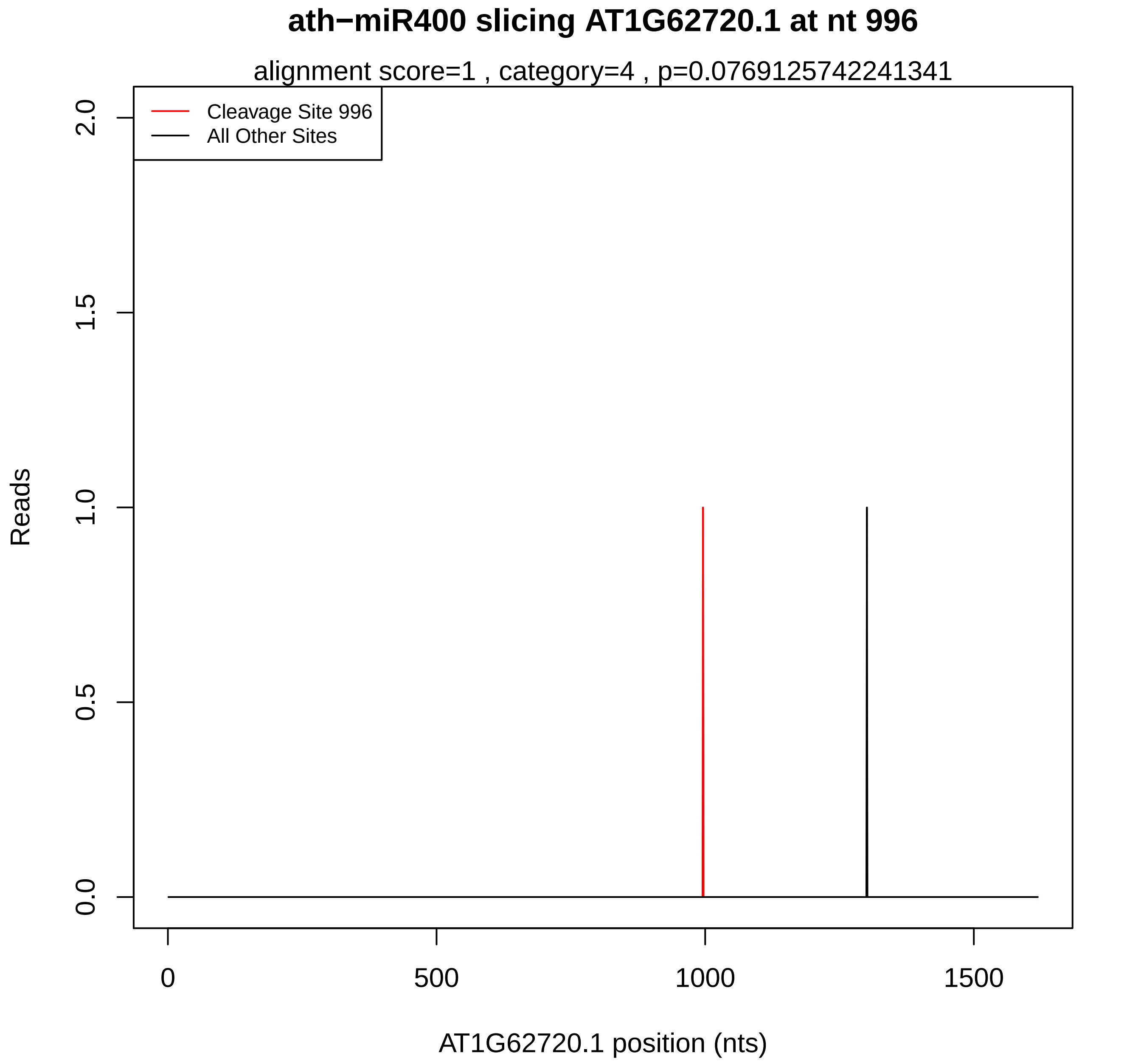
ath−miR400 slicing AT1G62720.1 at nt 996
alignment score=1 , category=4 , p=0.0769125742241341
Cleavage Site 996
All Other Sites
Reads
0.0
0.5
1.0
1.5
2.0
0
500
1000
1500
AT1G62720.1 position (nts)

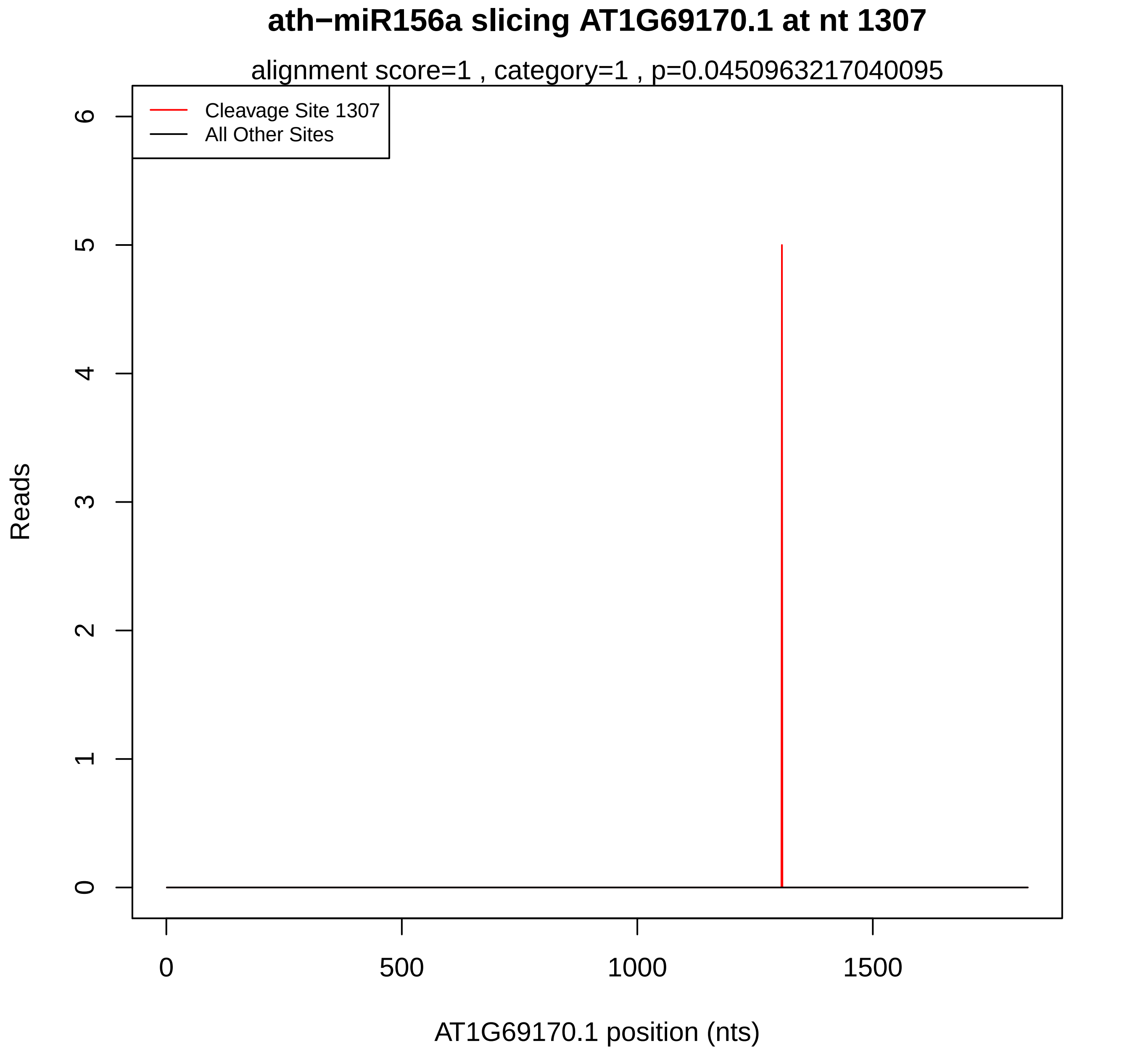
ath−miR156a slicing AT1G69170.1 at nt 1307
alignment score=1 , category=1 , p=0.0450963217040095
Cleavage Site 1307
All Other Sites
Reads
0
1
2
3
4
5
6
0
500
1000
1500
AT1G69170.1 position (nts)

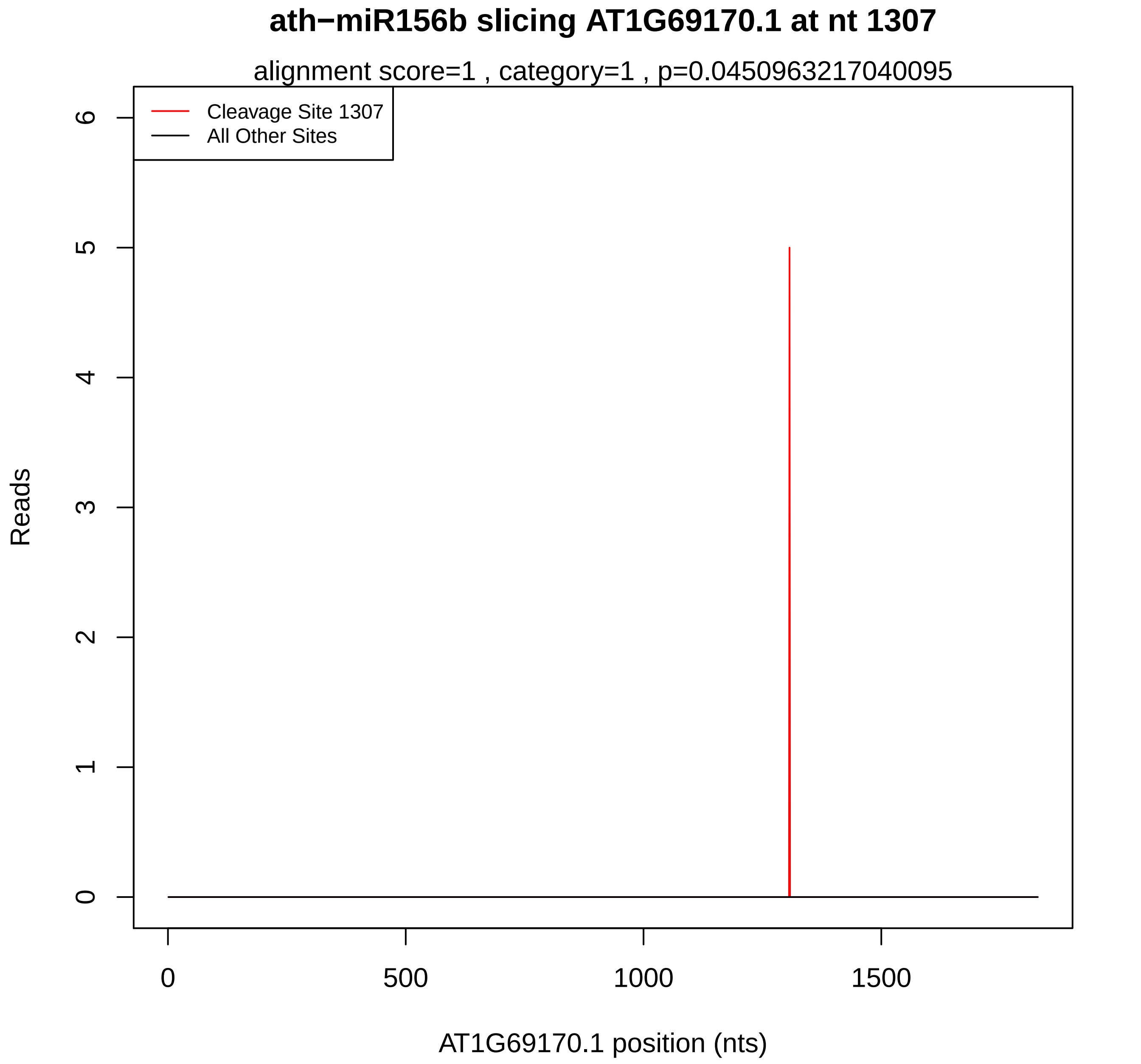
ath−miR156b slicing AT1G69170.1 at nt 1307
alignment score=1 , category=1 , p=0.0450963217040095
Cleavage Site 1307
All Other Sites
Reads
0
1
2
3
4
5
6
0
500
1000
1500
AT1G69170.1 position (nts)

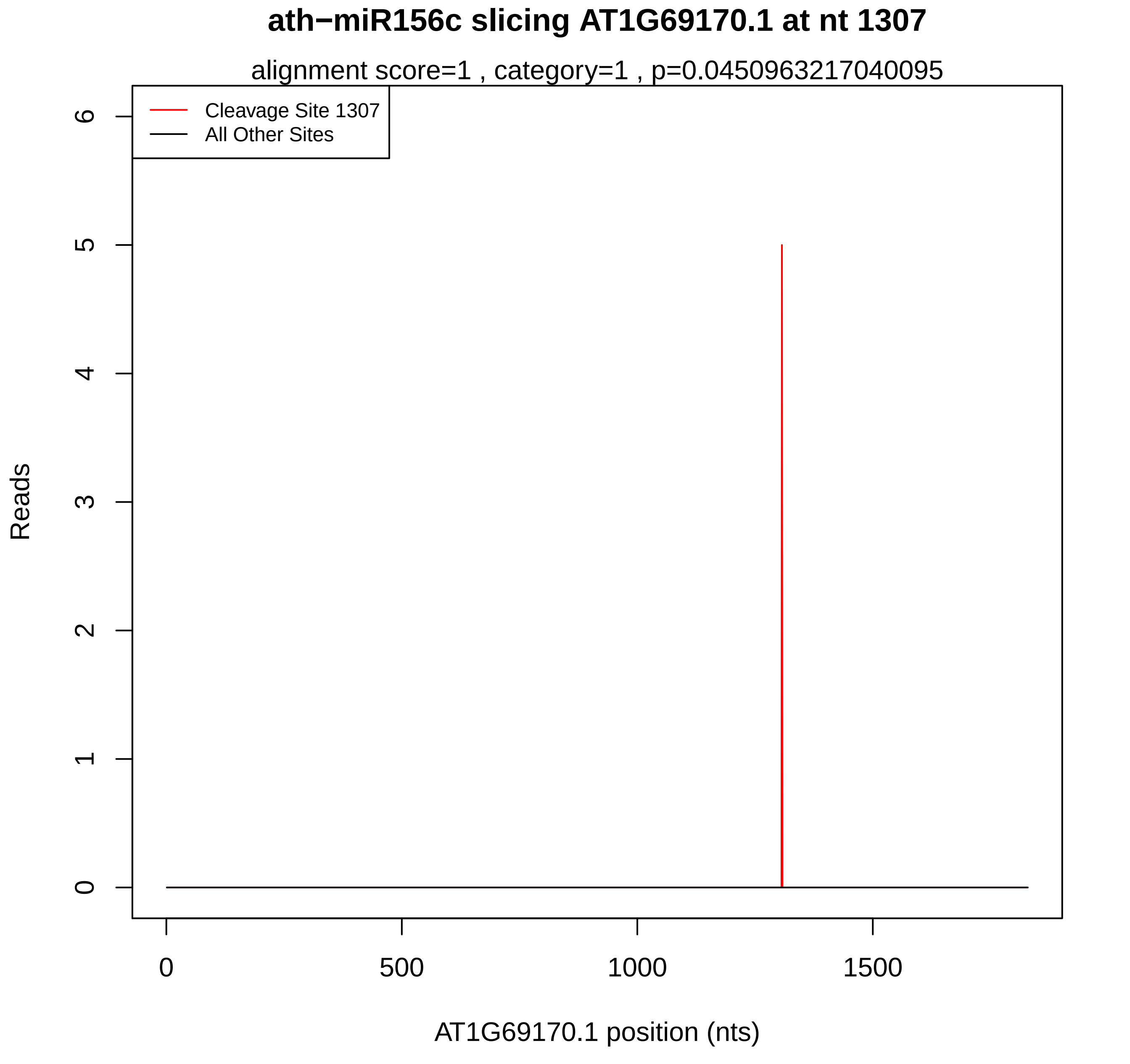
ath−miR156c slicing AT1G69170.1 at nt 1307
alignment score=1 , category=1 , p=0.0450963217040095
Cleavage Site 1307
All Other Sites
Reads
0
1
2
3
4
5
6
0
500
1000
1500
AT1G69170.1 position (nts)

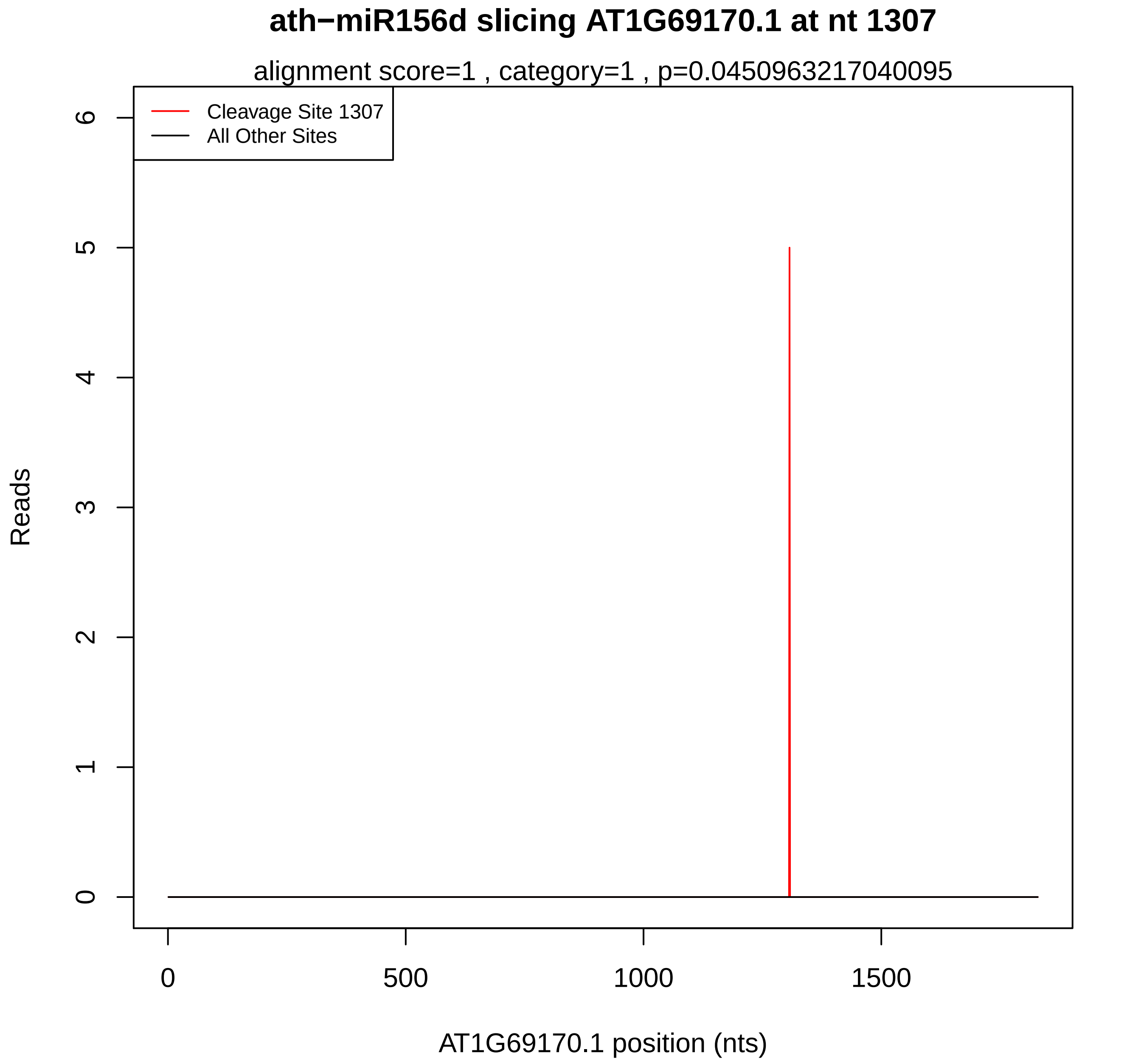

ath−miR156d slicing AT1G69170.1 at nt 1307
alignment score=1 , category=1 , p=0.0450963217040095
Cleavage Site 1307
All Other Sites
Reads
0
1
2
3
4
5
6
0
500
1000
1500
AT1G69170.1 position (nts)

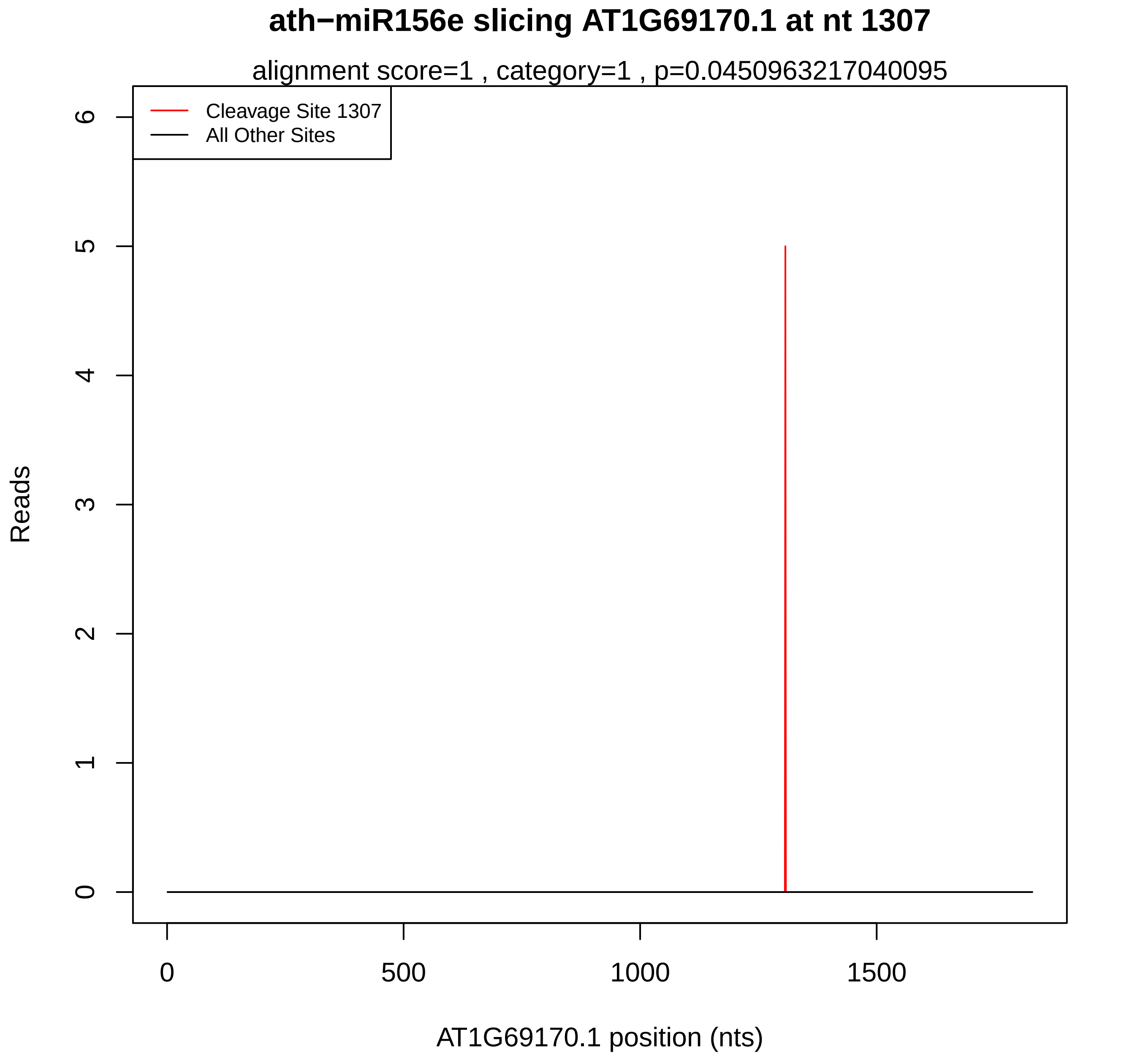
ath−miR156e slicing AT1G69170.1 at nt 1307
alignment score=1 , category=1 , p=0.0450963217040095
Cleavage Site 1307
All Other Sites
Reads
0
1
2
3
4
5
6
0
500
1000
1500
AT1G69170.1 position (nts)

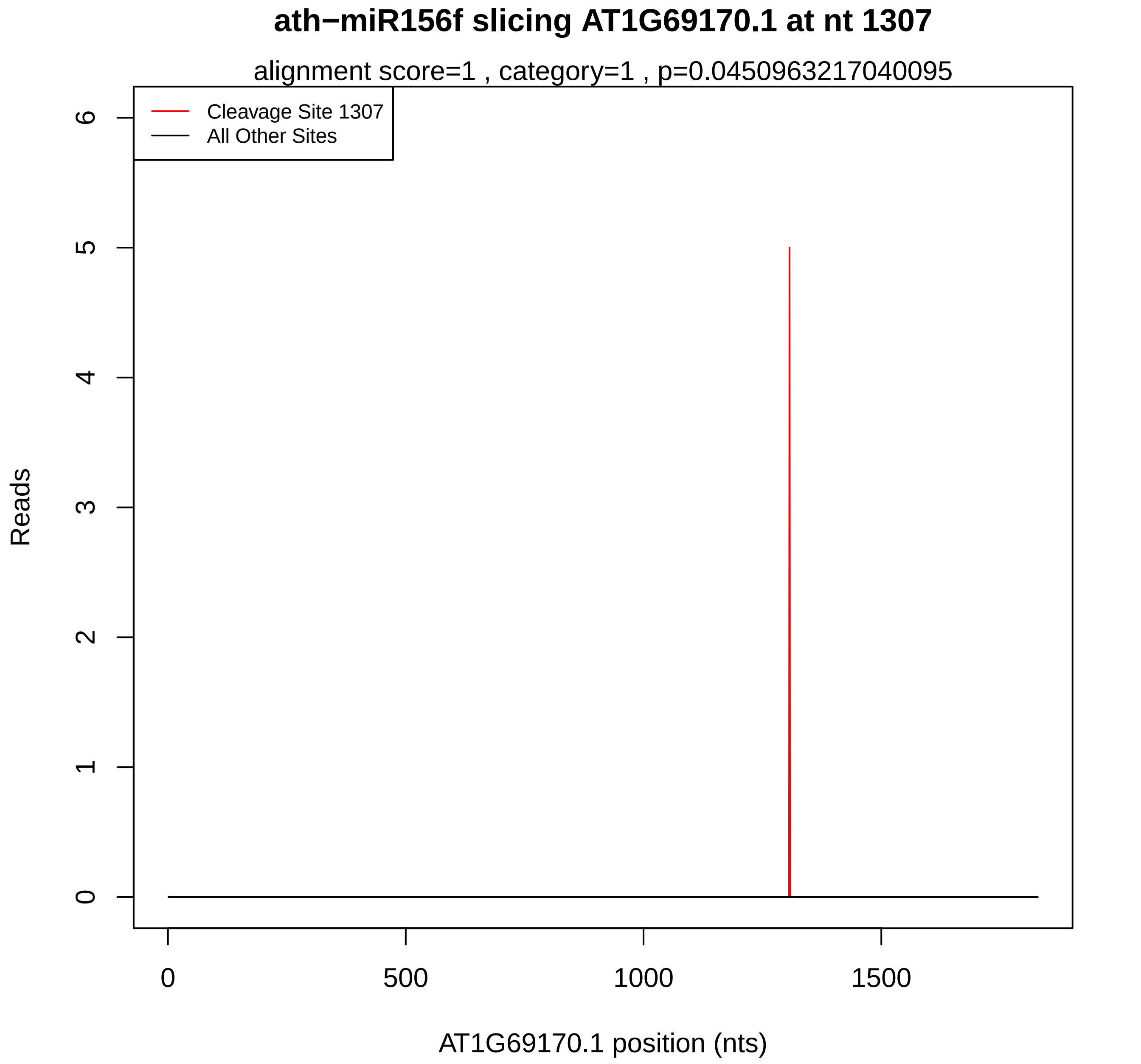
ath−miR156f slicing AT1G69170.1 at nt 1307
alignment score=1 , category=1 , p=0.0450963217040095
Cleavage Site 1307
All Other Sites
Reads
0
1
2
3
4
5
6
0
500
1000
1500
AT1G69170.1 position (nts)

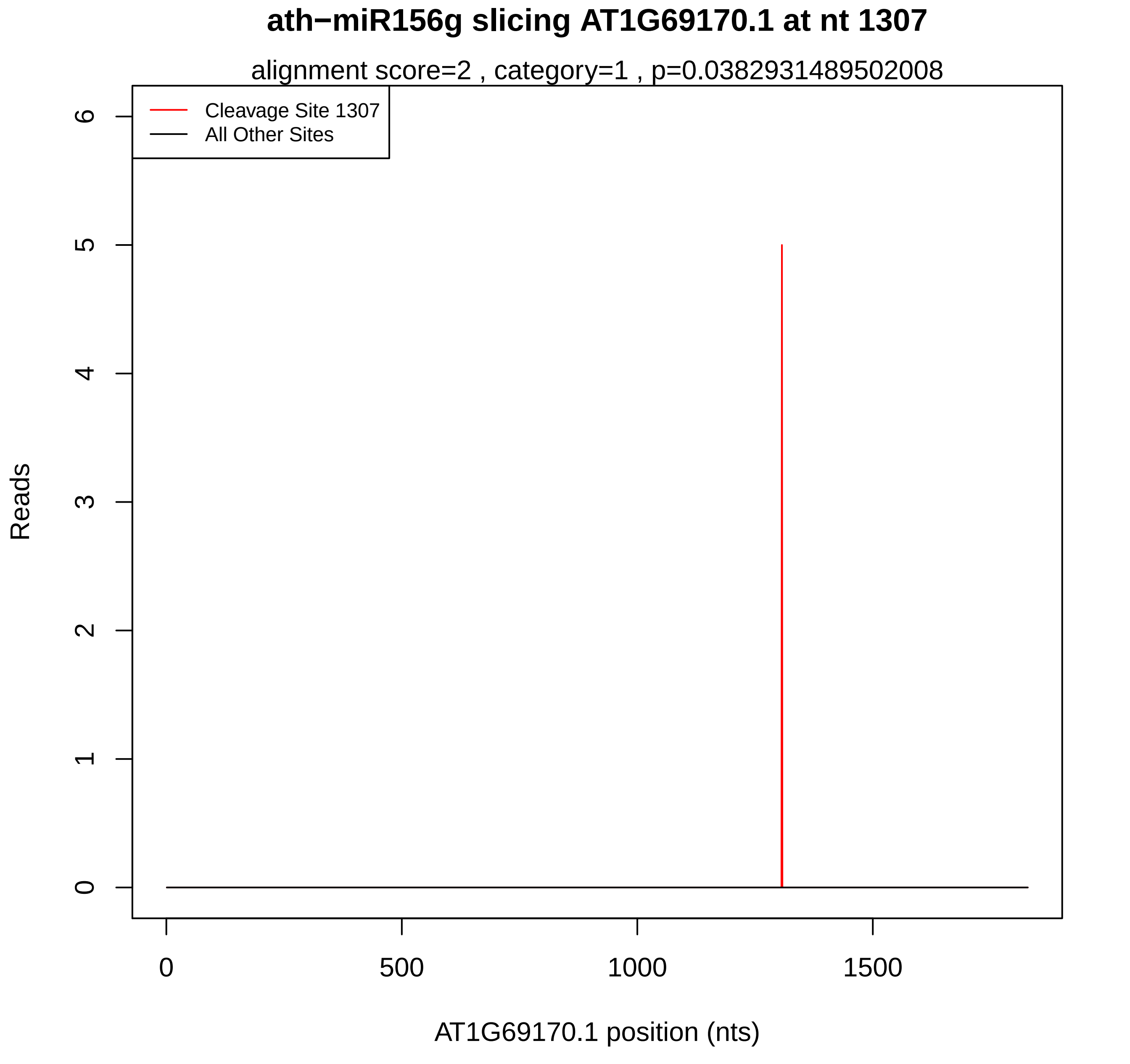

ath−miR156g slicing AT1G69170.1 at nt 1307
alignment score=2 , category=1 , p=0.0382931489502008
Cleavage Site 1307
All Other Sites
Reads
0
1
2
3
4
5
6
0
500
1000
1500
AT1G69170.1 position (nts)

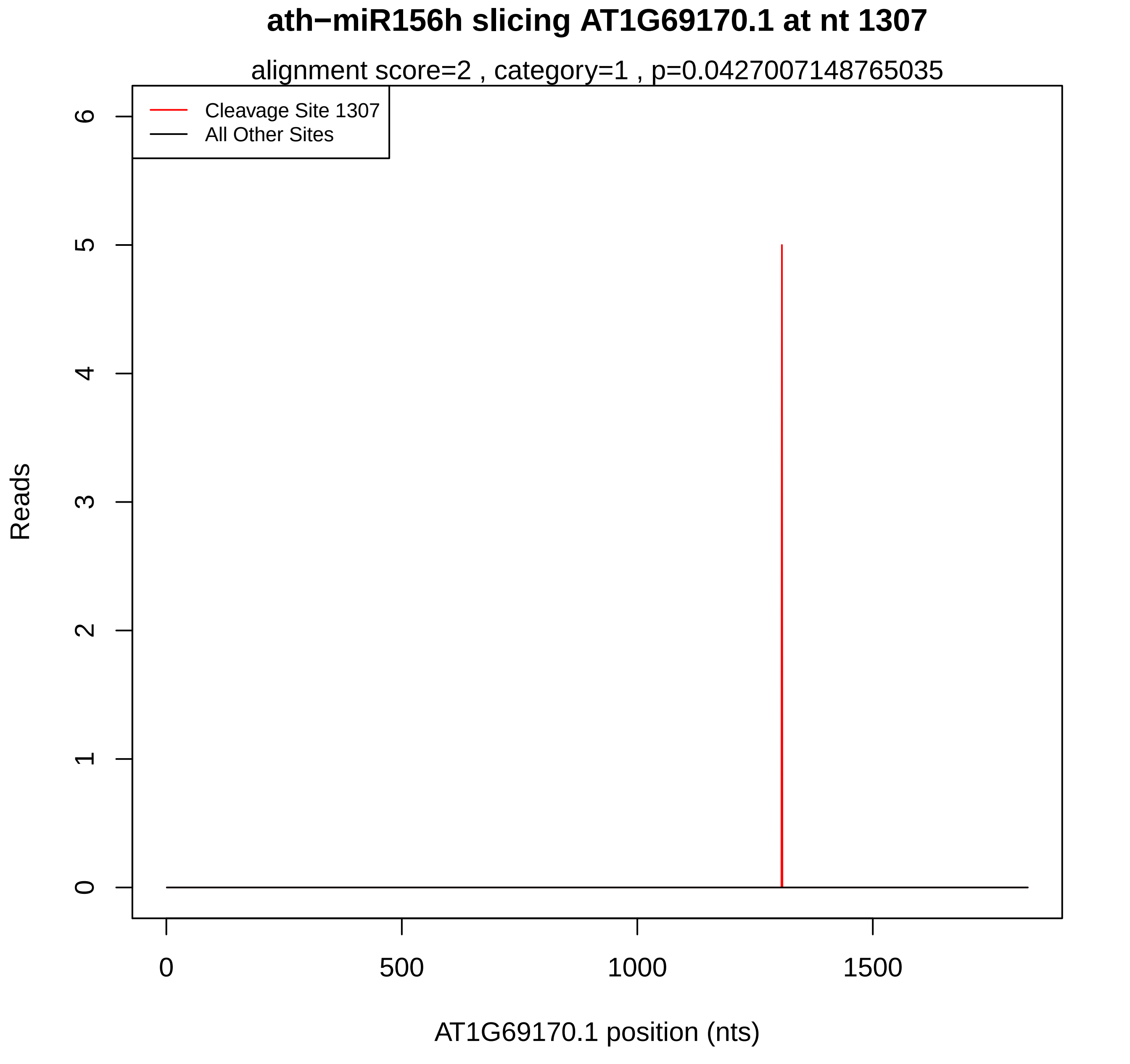
ath−miR156h slicing AT1G69170.1 at nt 1307
alignment score=2 , category=1 , p=0.0427007148765035
Cleavage Site 1307
All Other Sites
Reads
0
1
2
3
4
5
6
0
500
1000
1500
AT1G69170.1 position (nts)

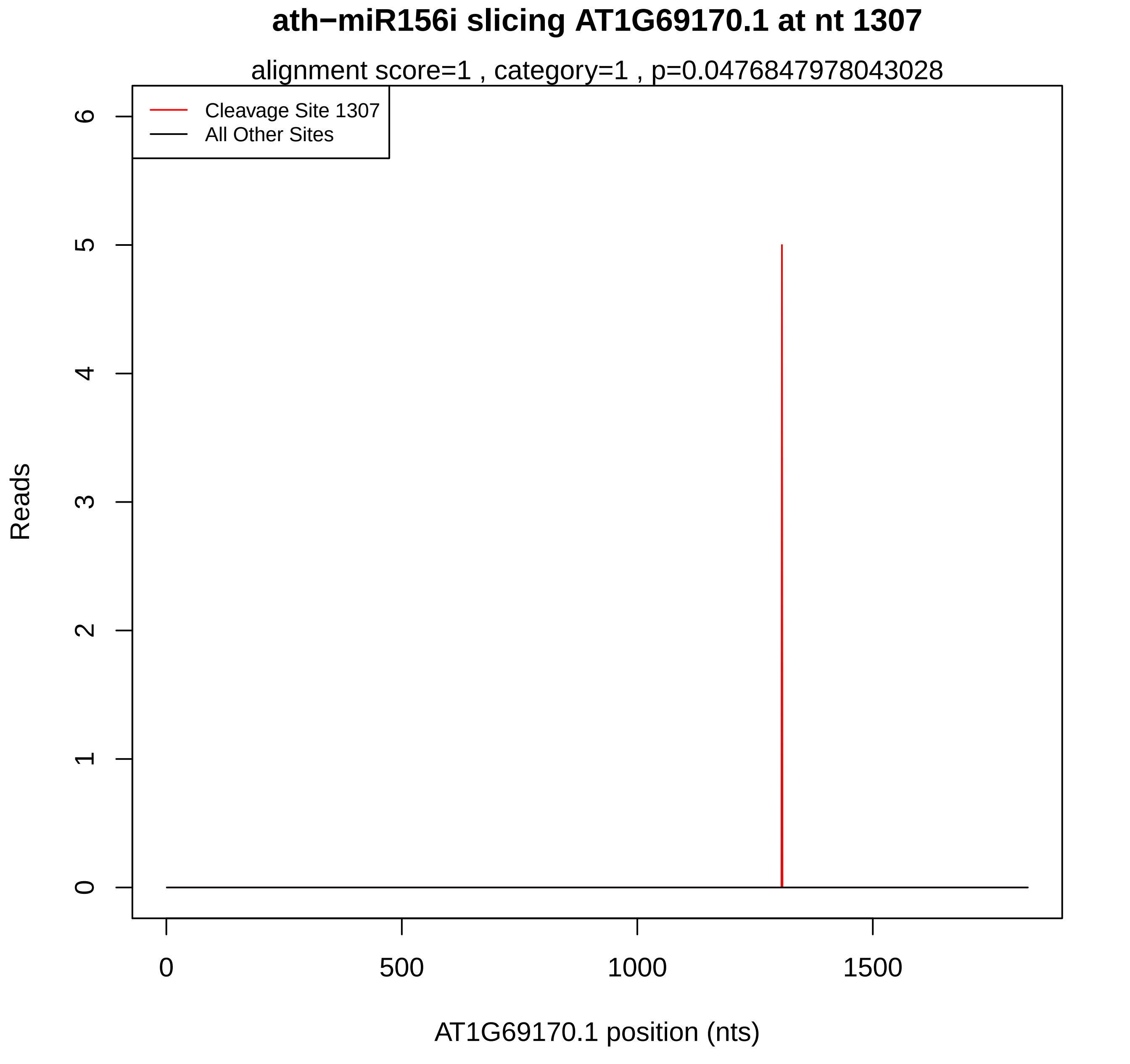
ath−miR156i slicing AT1G69170.1 at nt 1307
alignment score=1 , category=1 , p=0.0476847978043028
Cleavage Site 1307
All Other Sites
Reads
0
1
2
3
4
5
6
0
500
1000
1500
AT1G69170.1 position (nts)

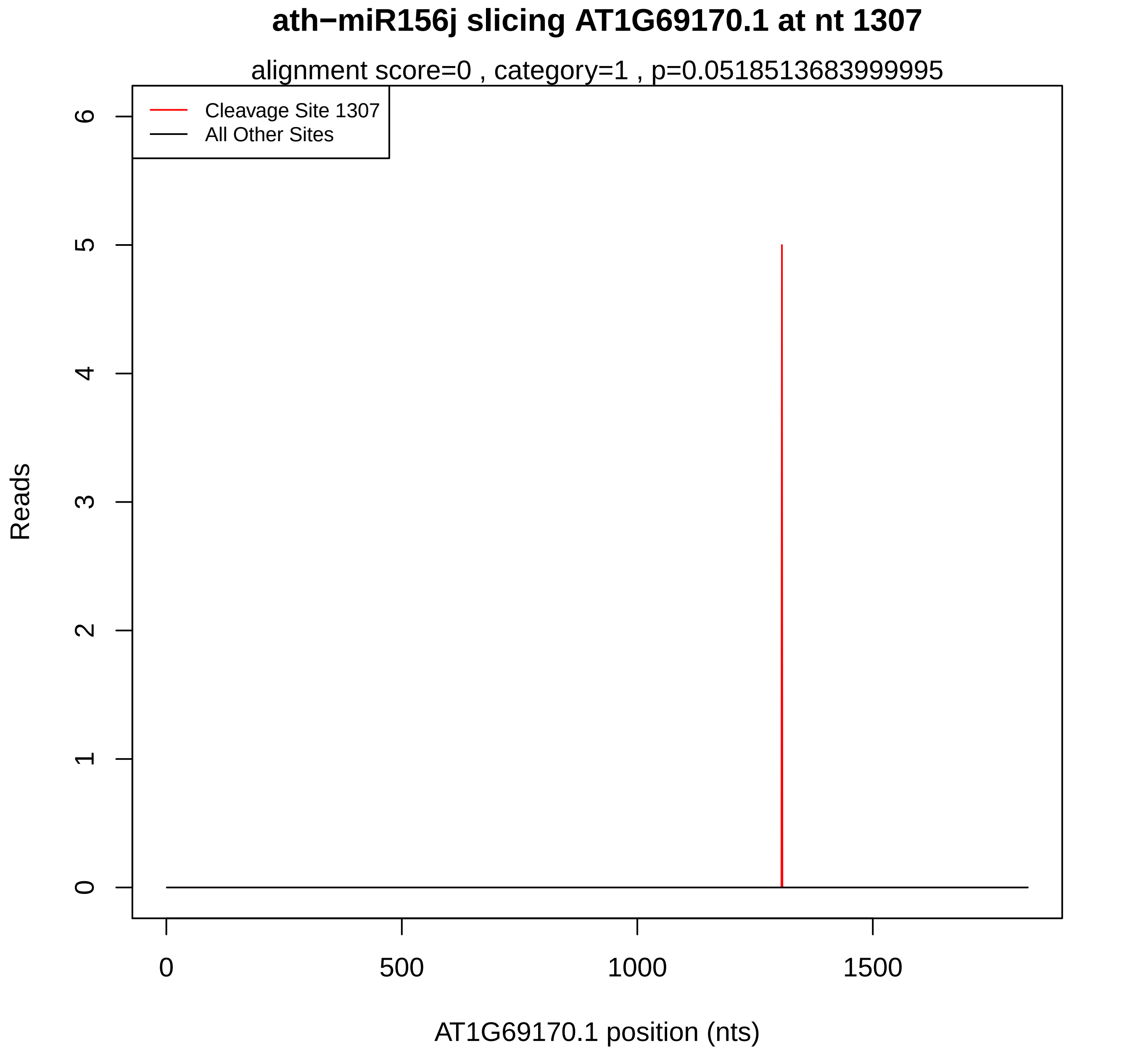

ath−miR156j slicing AT1G69170.1 at nt 1307
alignment score=0 , category=1 , p=0.0518513683999995
Cleavage Site 1307
All Other Sites
Reads
0
1
2
3
4
5
6
0
500
1000
1500
AT1G69170.1 position (nts)

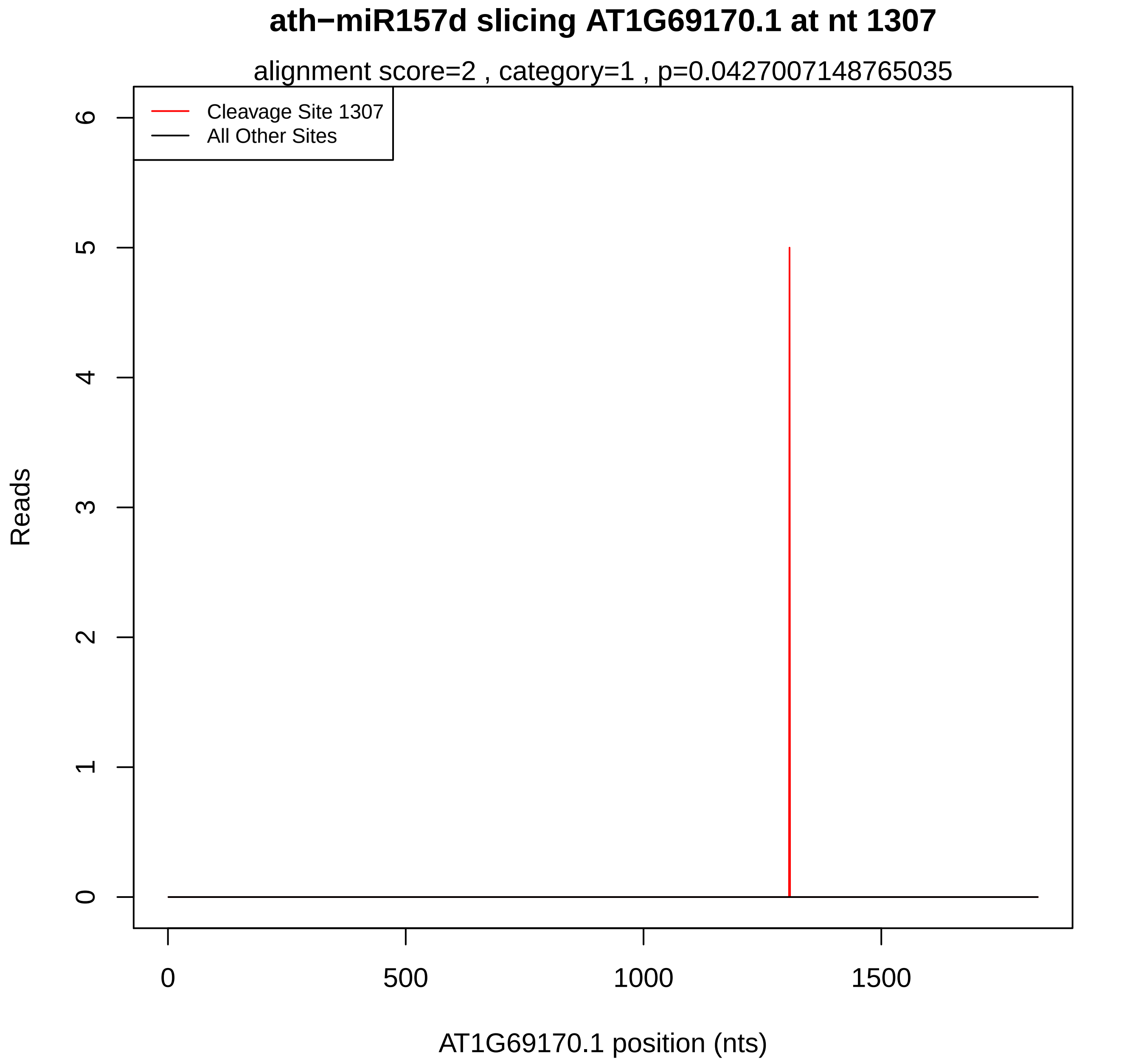
ath−miR157d slicing AT1G69170.1 at nt 1307
alignment score=2 , category=1 , p=0.0427007148765035
Cleavage Site 1307
All Other Sites
Reads
0
1
2
3
4
5
6
0
500
1000
1500
AT1G69170.1 position (nts)

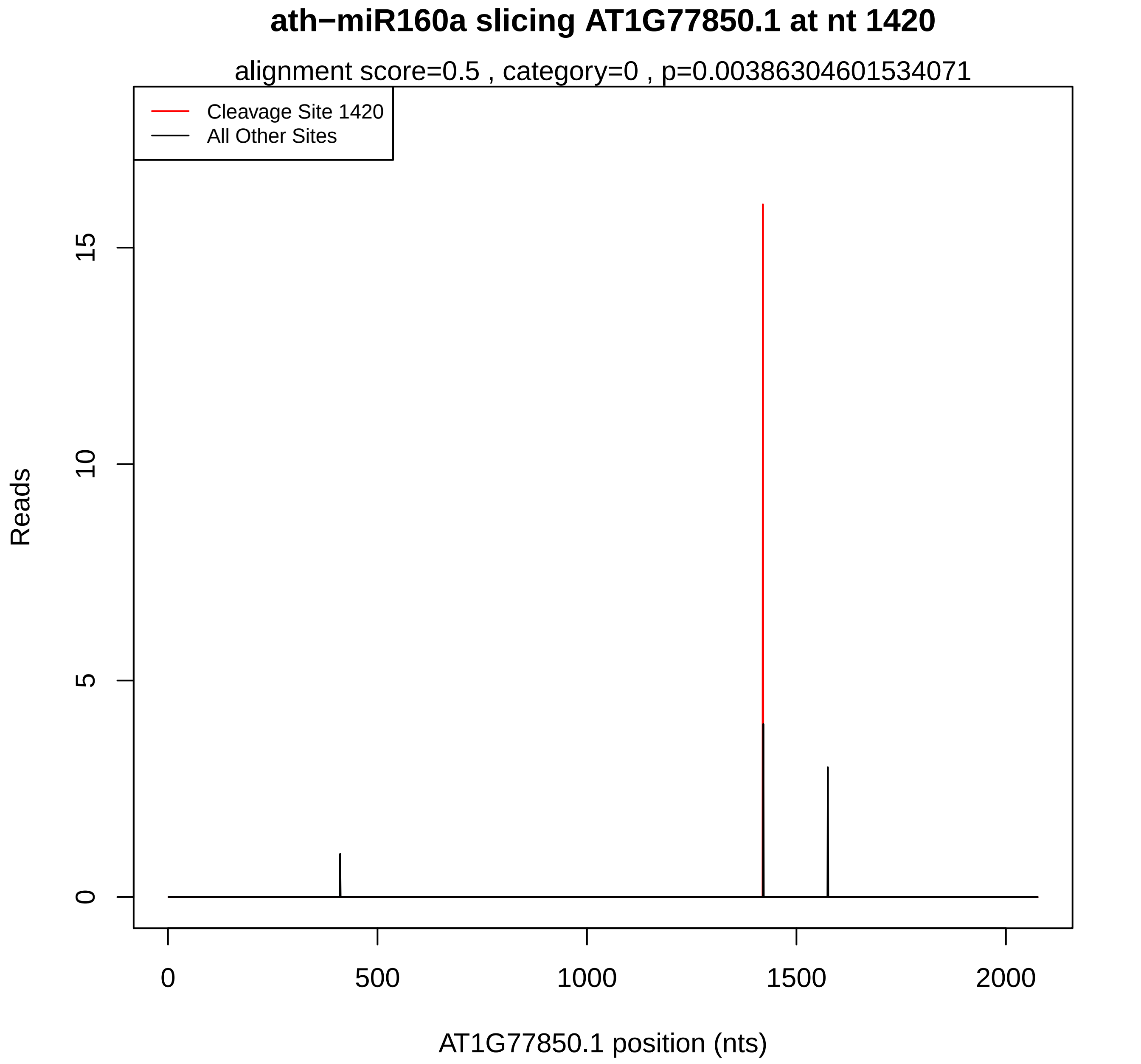

ath−miR160a slicing AT1G77850.1 at nt 1420
alignment score=0.5 , category=0 , p=0.00386304601534071
Cleavage Site 1420
All Other Sites
Reads
0
5
10
15
0
500
1000
1500
2000
AT1G77850.1 position (nts)

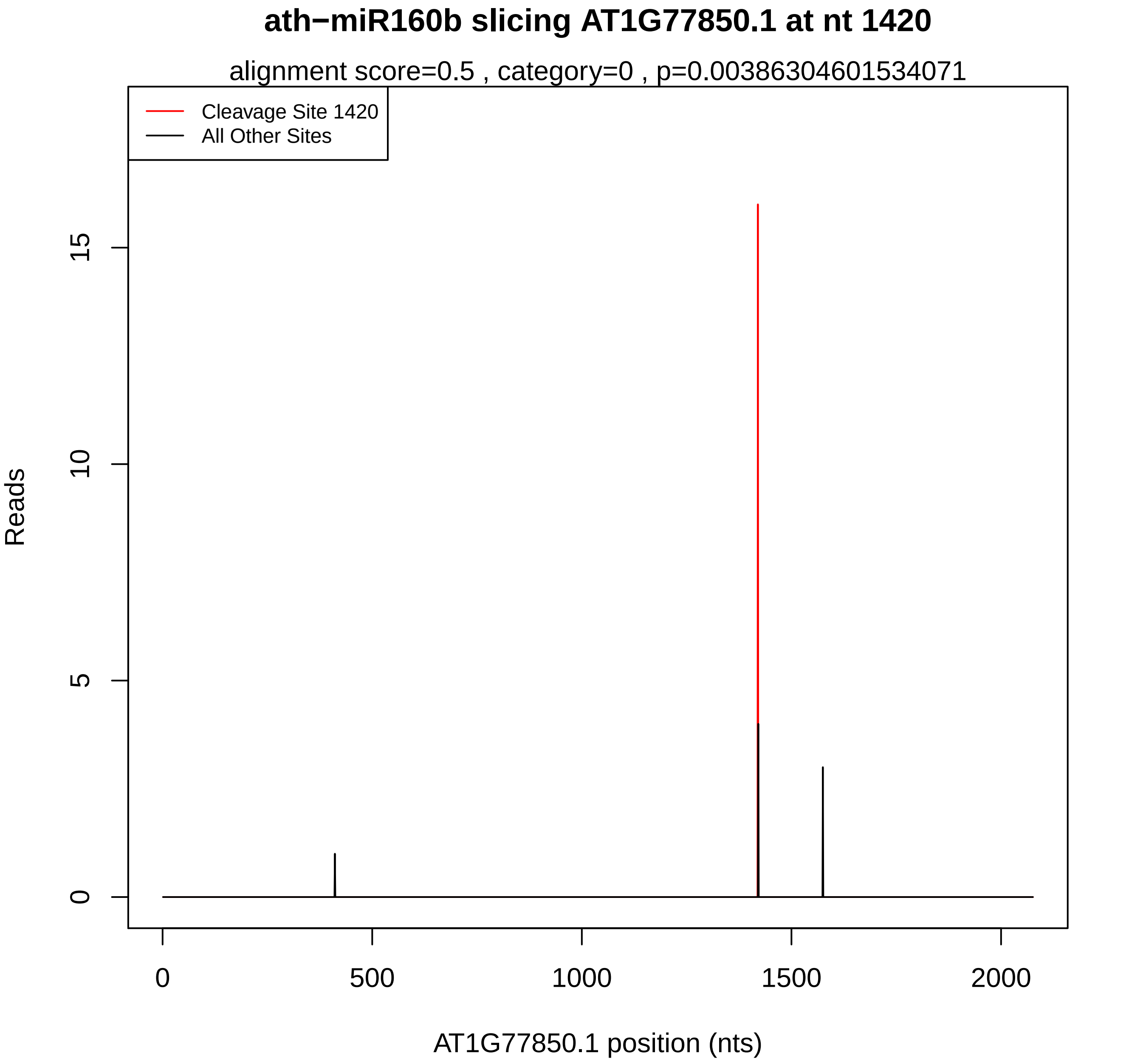
ath−miR160b slicing AT1G77850.1 at nt 1420
alignment score=0.5 , category=0 , p=0.00386304601534071
Cleavage Site 1420
All Other Sites
Reads
0
5
10
15
0
500
1000
1500
2000
AT1G77850.1 position (nts)

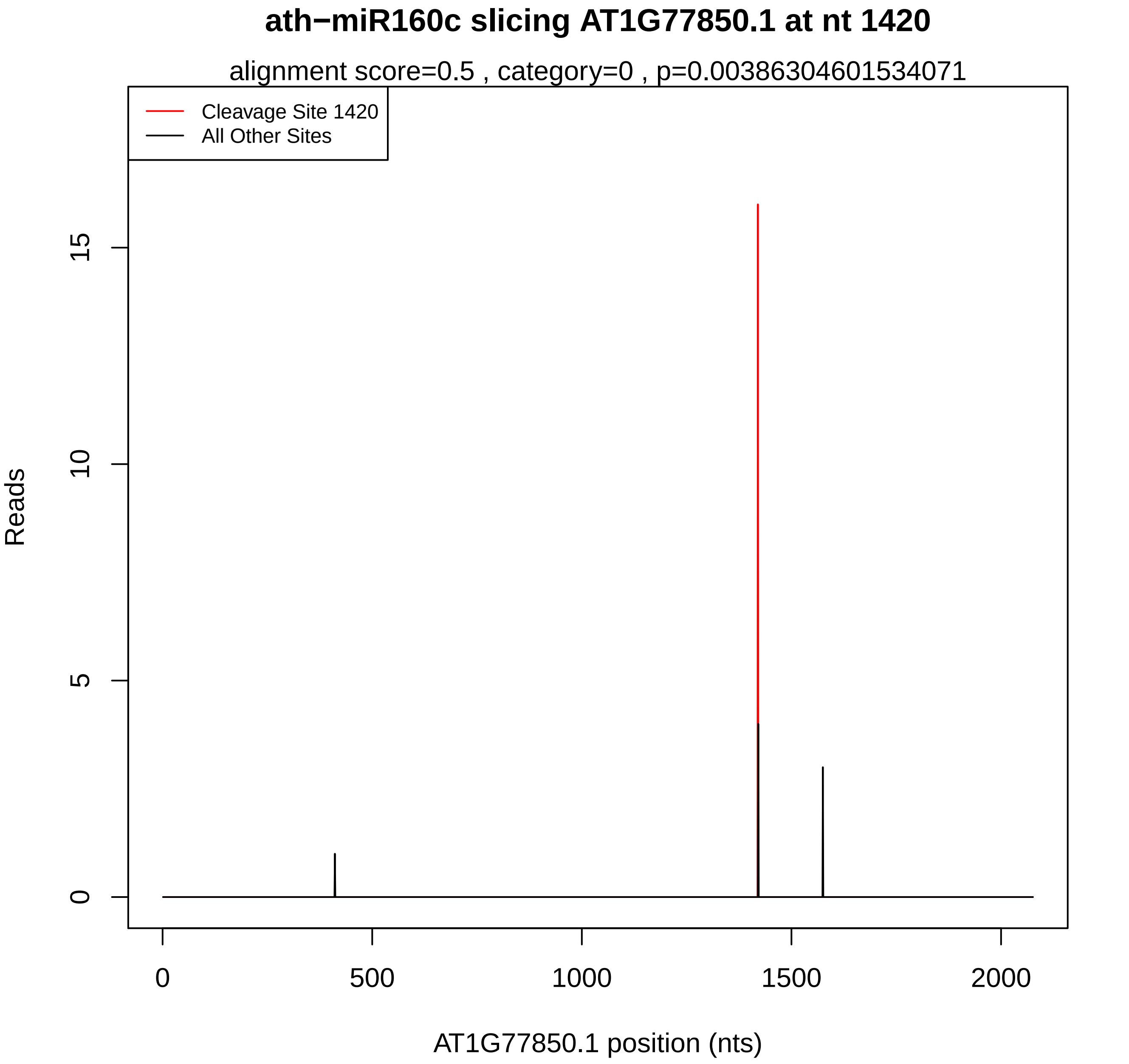

ath−miR160c slicing AT1G77850.1 at nt 1420
alignment score=0.5 , category=0 , p=0.00386304601534071
Cleavage Site 1420
All Other Sites
Reads
0
5
10
15
0
500
1000
1500
2000
AT1G77850.1 position (nts)

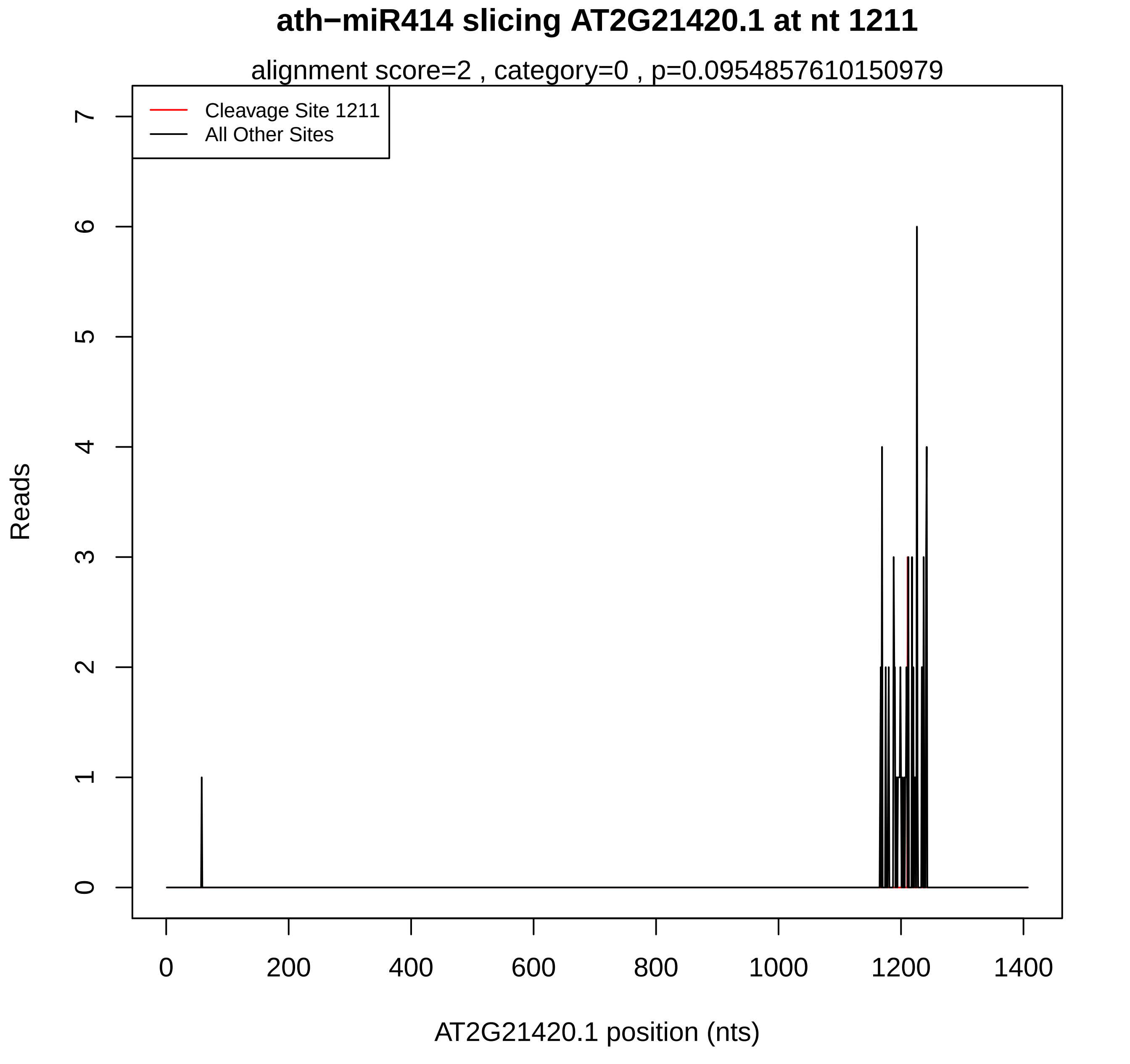
ath−miR414 slicing AT2G21420.1 at nt 1211
alignment score=2 , category=0 , p=0.0954857610150979
Cleavage Site 1211
All Other Sites
Reads
0
1
2
3
4
5
6
7
0
200
400
600
800
1000
1200
1400
AT2G21420.1 position (nts)

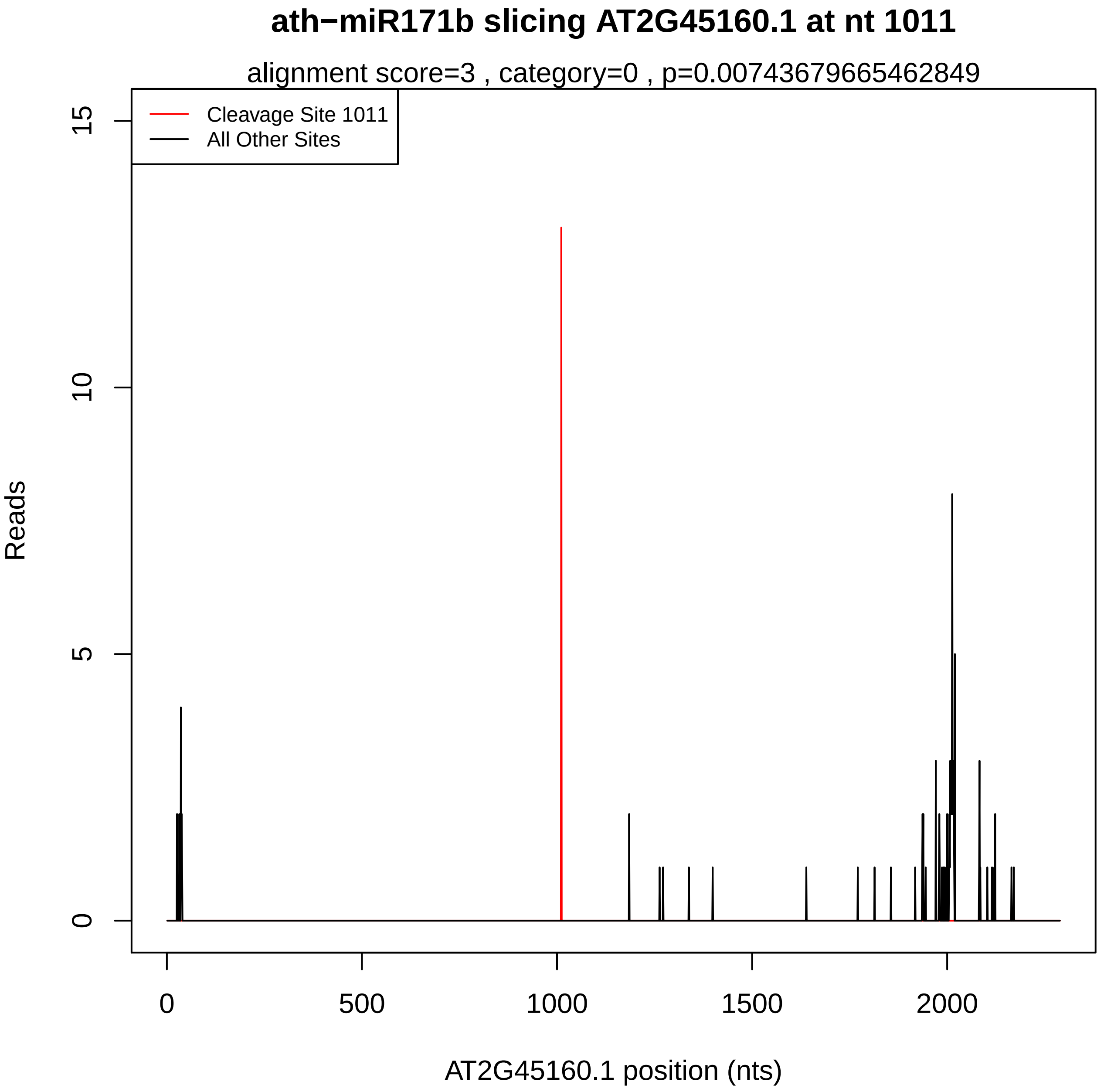
ath−miR171b slicing AT2G45160.1 at nt 1011
alignment score=3 , category=0 , p=0.00743679665462849
Cleavage Site 1011
All Other Sites
Reads
0
5
10
15
0
500
1000
1500
2000
AT2G45160.1 position (nts)

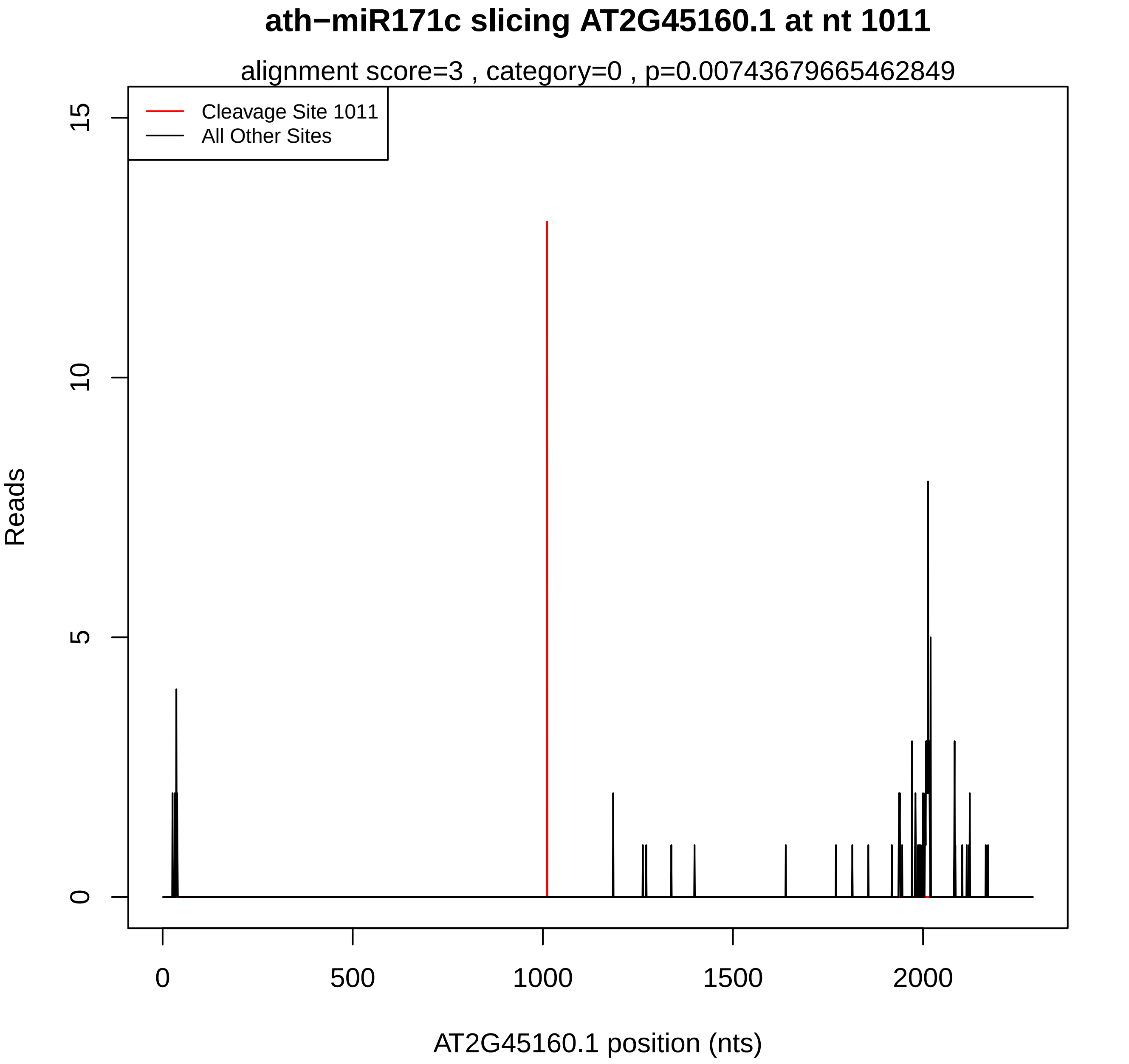

ath−miR171c slicing AT2G45160.1 at nt 1011
alignment score=3 , category=0 , p=0.00743679665462849
Cleavage Site 1011
All Other Sites
Reads
0
5
10
15
0
500
1000
1500
2000
AT2G45160.1 position (nts)

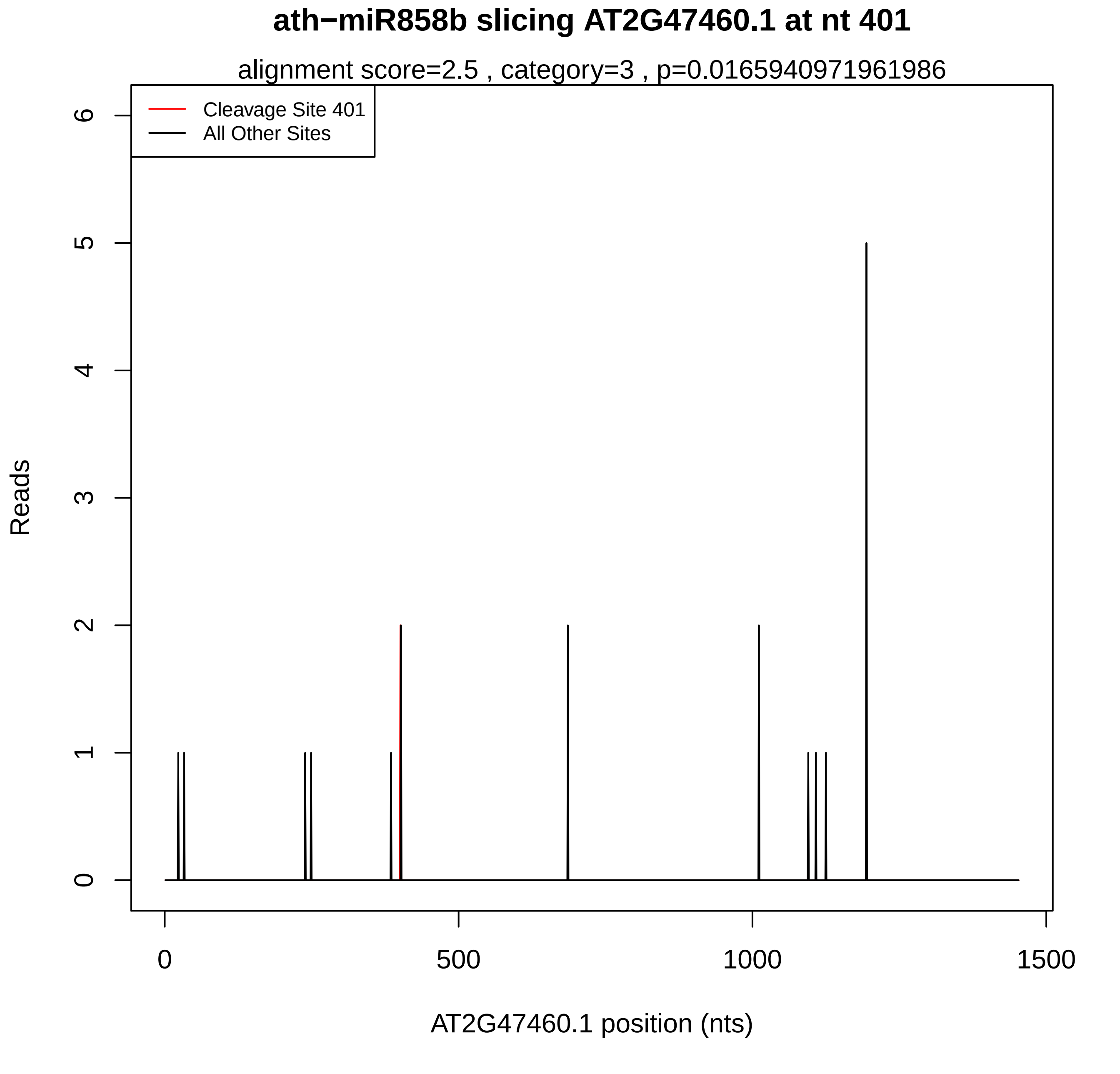
ath−miR858b slicing AT2G47460.1 at nt 401
alignment score=2.5 , category=3 , p=0.0165940971961986
Cleavage Site 401
All Other Sites
Reads
0
1
2
3
4
5
6
0
500
1000
1500
AT2G47460.1 position (nts)

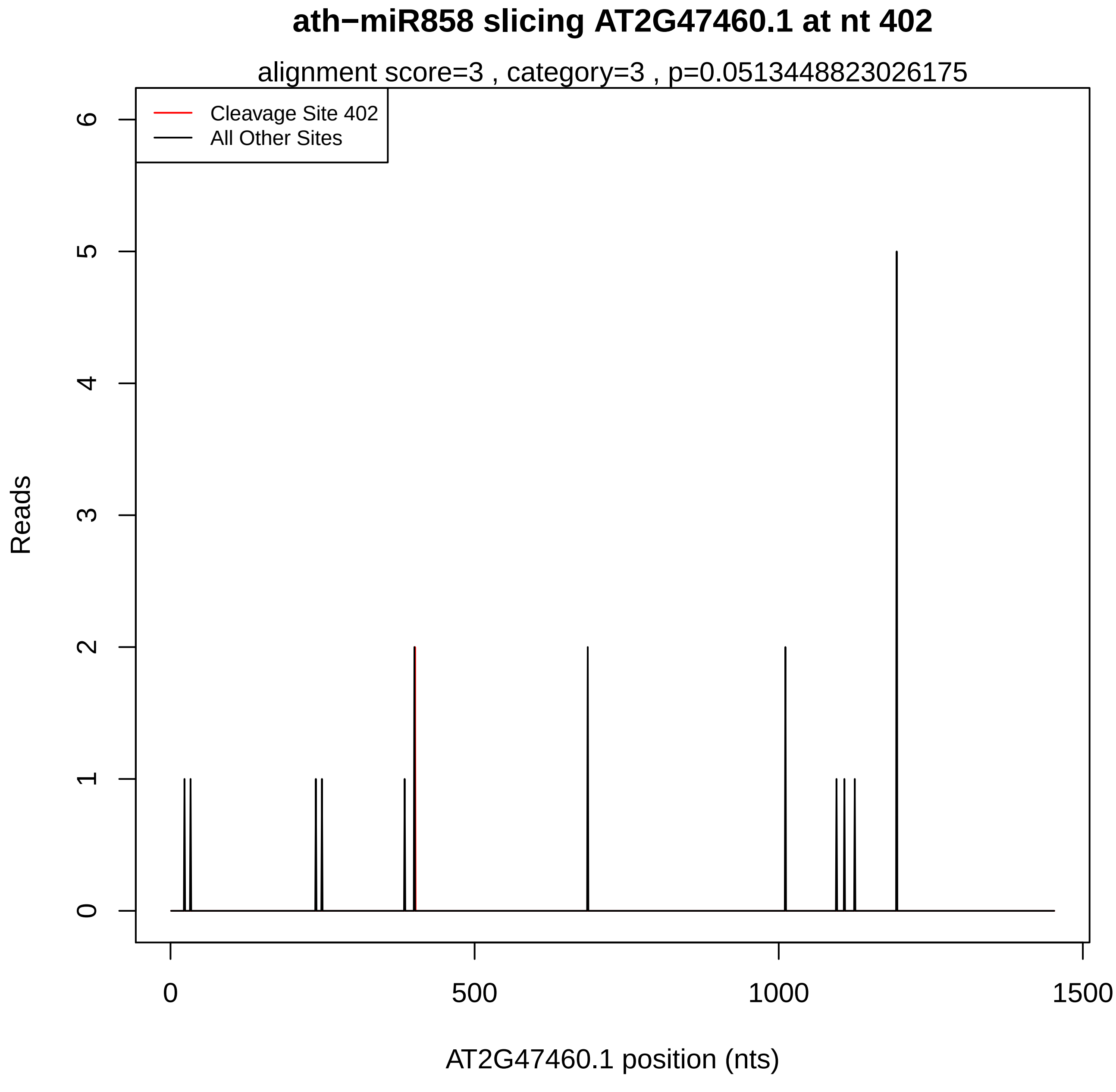
ath−miR858 slicing AT2G47460.1 at nt 402
alignment score=3 , category=3 , p=0.0513448823026175
Cleavage Site 402
All Other Sites
Reads
0
1
2
3
4
5
6
0
500
1000
1500
AT2G47460.1 position (nts)

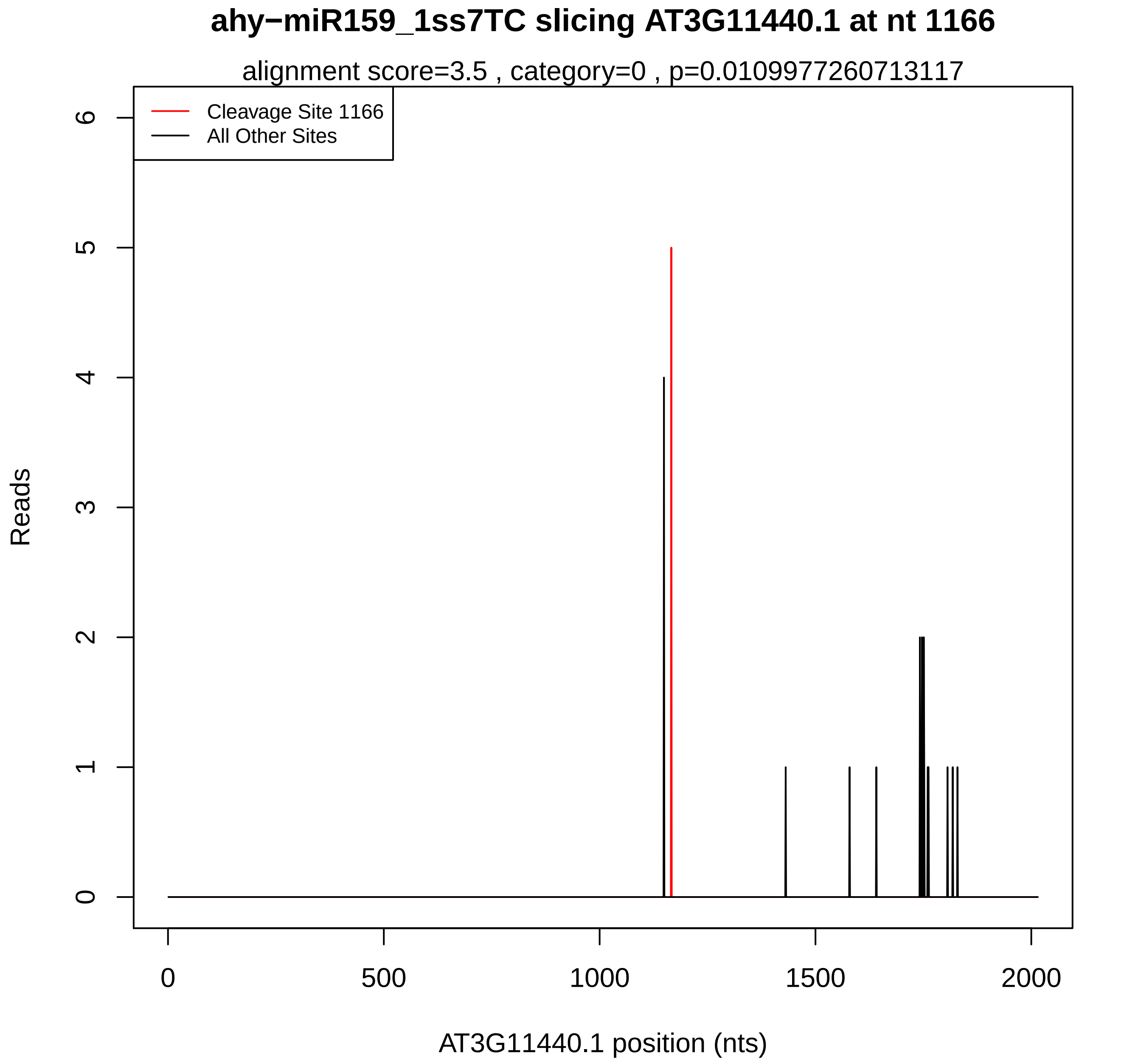
ahy−miR159_1ss7TC slicing AT3G11440.1 at nt 1166
alignment score=3.5 , category=0 , p=0.0109977260713117
Cleavage Site 1166
All Other Sites
Reads
0
1
2
3
4
5
6
0
500
1000
1500
2000
AT3G11440.1 position (nts)

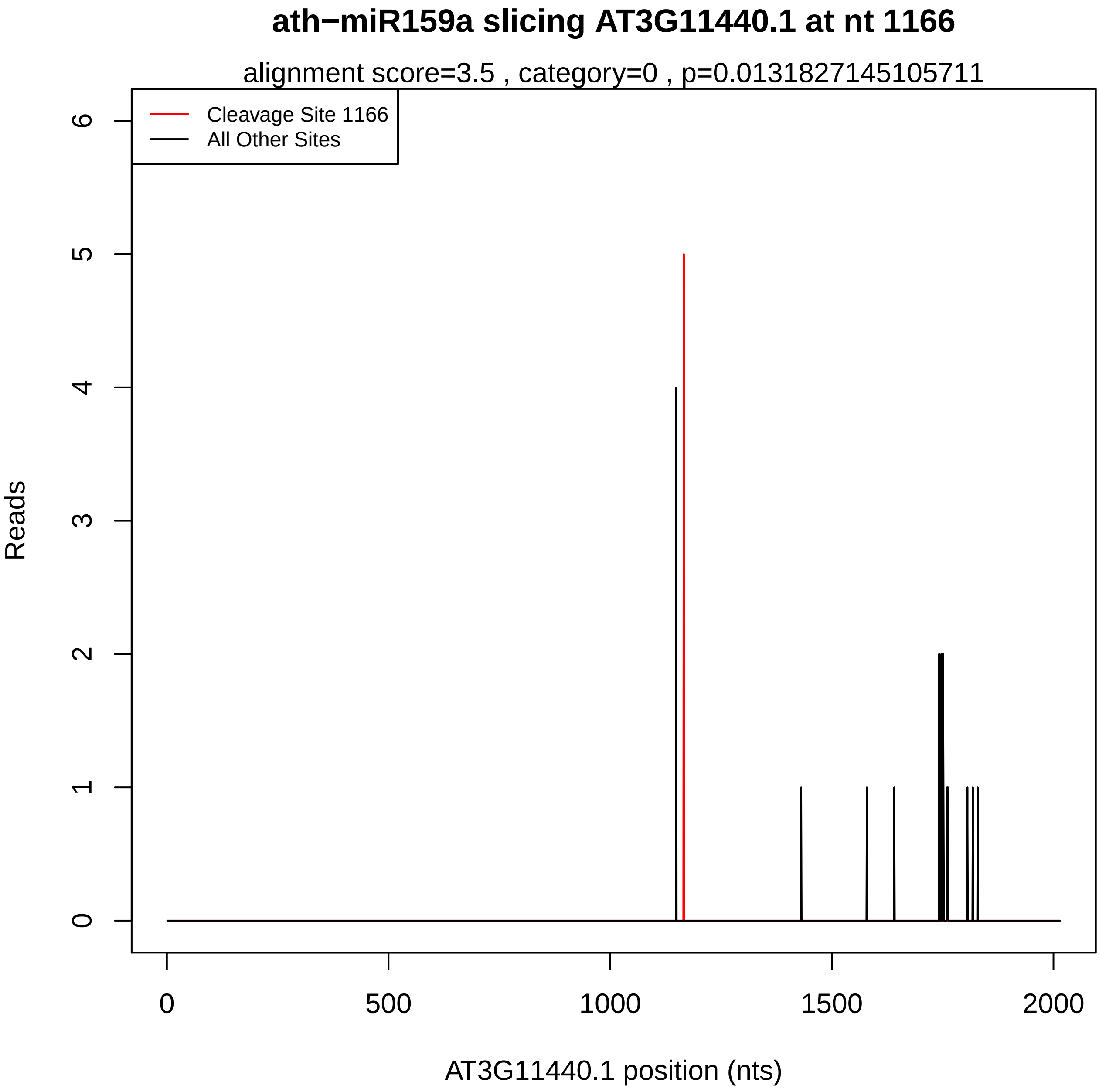
ath−miR159a slicing AT3G11440.1 at nt 1166
alignment score=3.5 , category=0 , p=0.0131827145105711
Cleavage Site 1166
All Other Sites
Reads
0
1
2
3
4
5
6
0
500
1000
1500
2000
AT3G11440.1 position (nts)

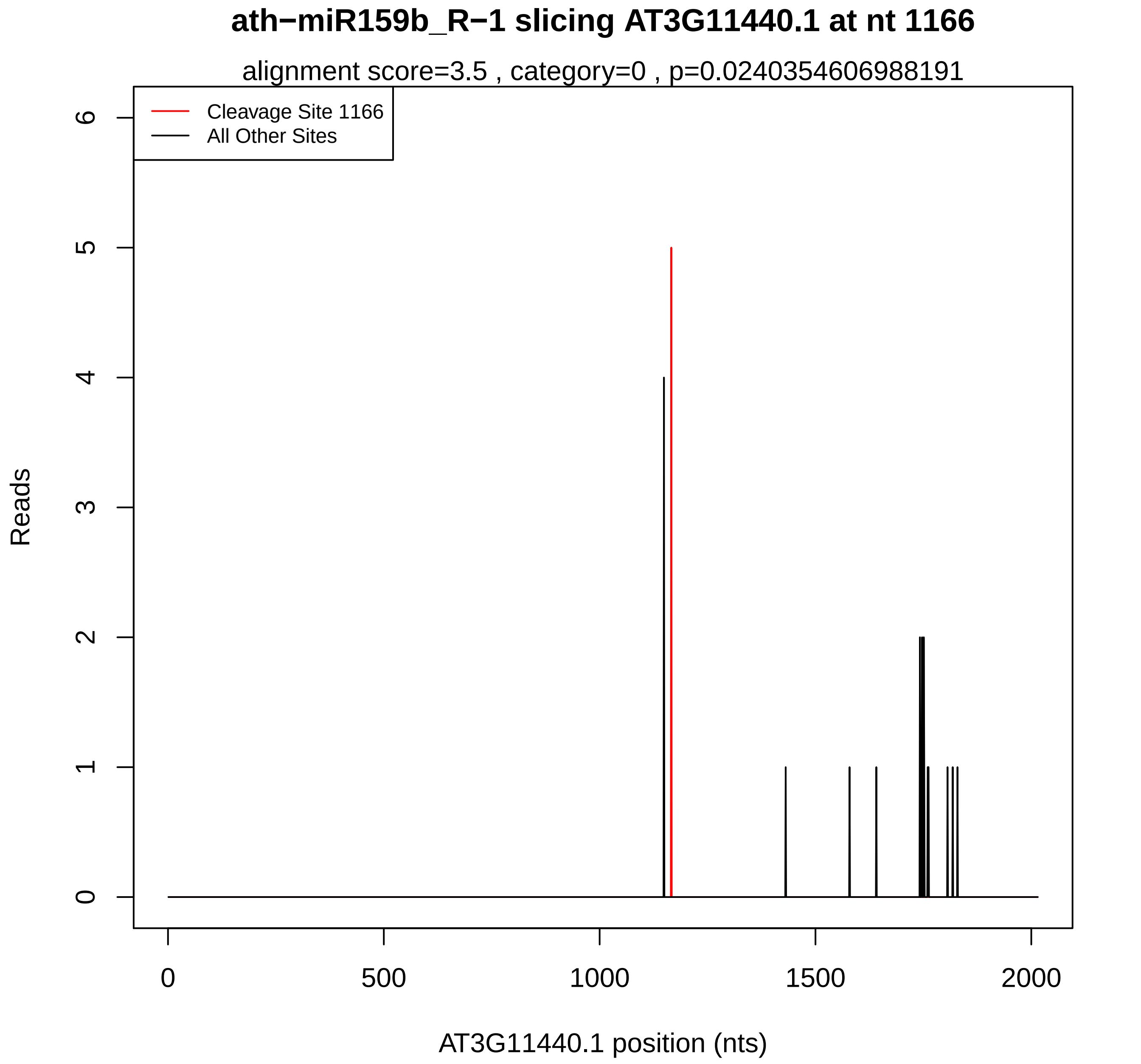
ath−miR159b_R−1 slicing AT3G11440.1 at nt 1166
alignment score=3.5 , category=0 , p=0.0240354606988191
Cleavage Site 1166
All Other Sites
Reads
0
1
2
3
4
5
6
0
500
1000
1500
2000
AT3G11440.1 position (nts)

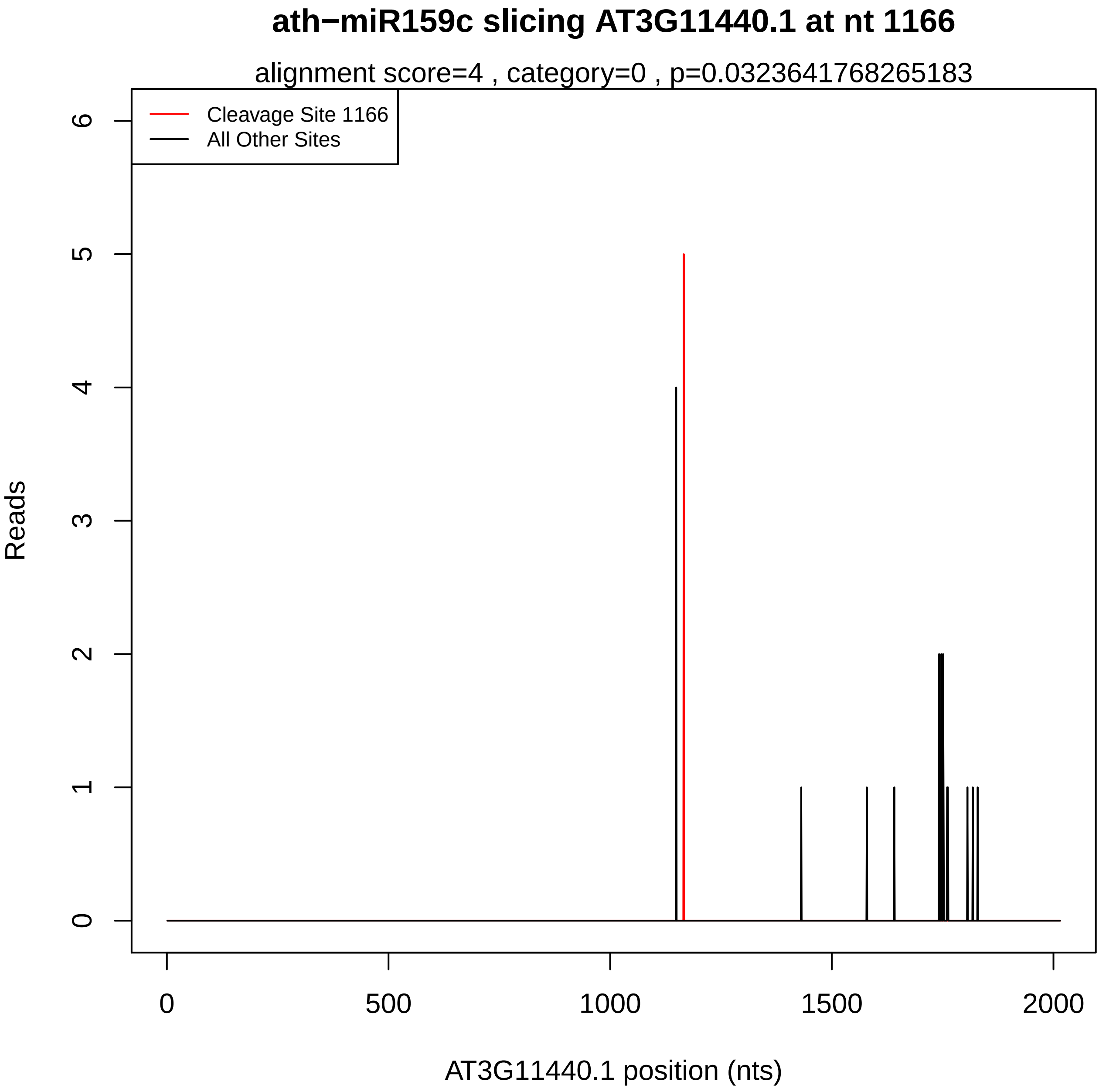
ath−miR159c slicing AT3G11440.1 at nt 1166
alignment score=4 , category=0 , p=0.0323641768265183
Cleavage Site 1166
All Other Sites
Reads
0
1
2
3
4
5
6
0
500
1000
1500
2000
AT3G11440.1 position (nts)

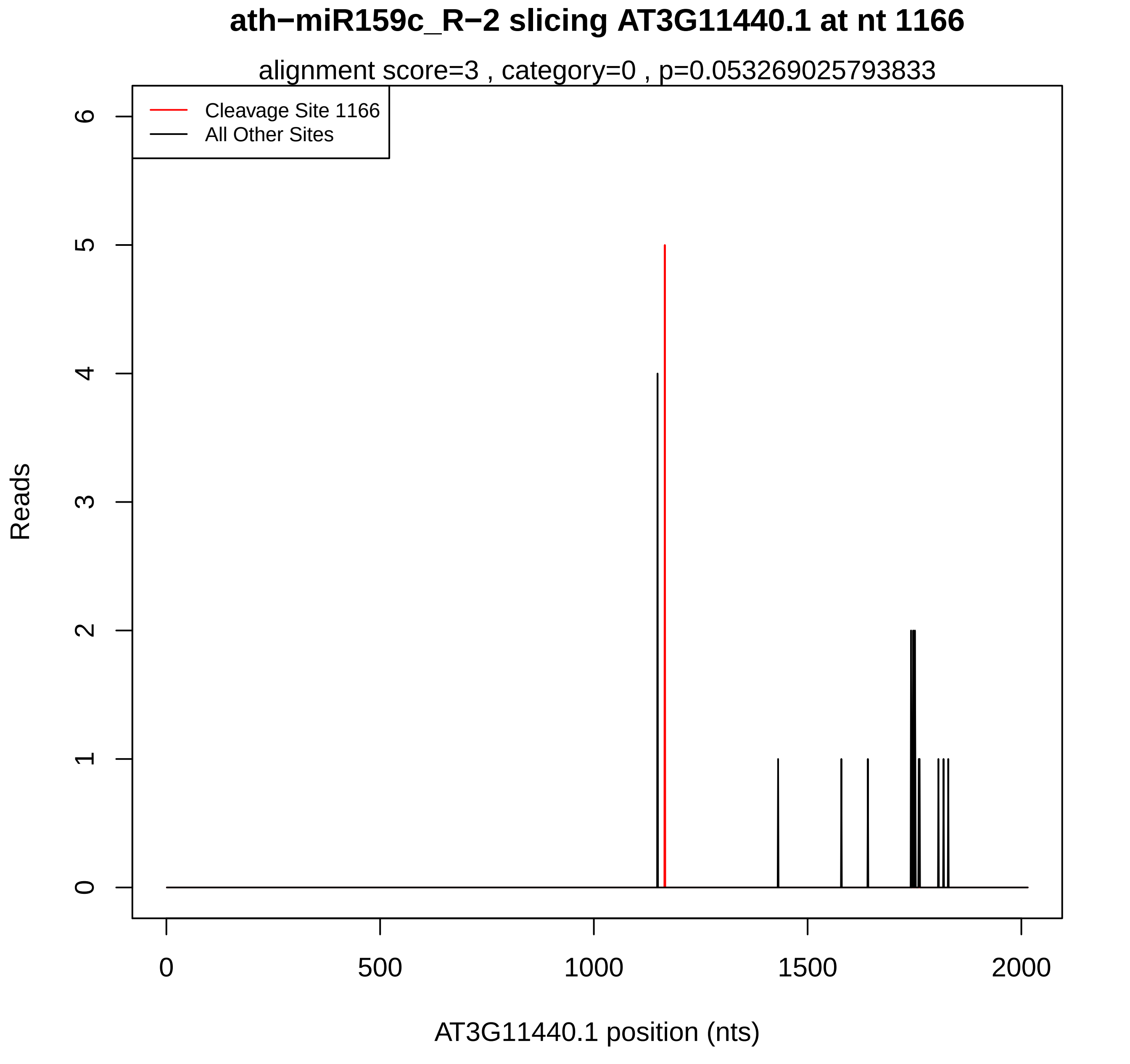
ath−miR159c_R−2 slicing AT3G11440.1 at nt 1166
alignment score=3 , category=0 , p=0.053269025793833
Cleavage Site 1166
All Other Sites
Reads
0
1
2
3
4
5
6
0
500
1000
1500
2000
AT3G11440.1 position (nts)

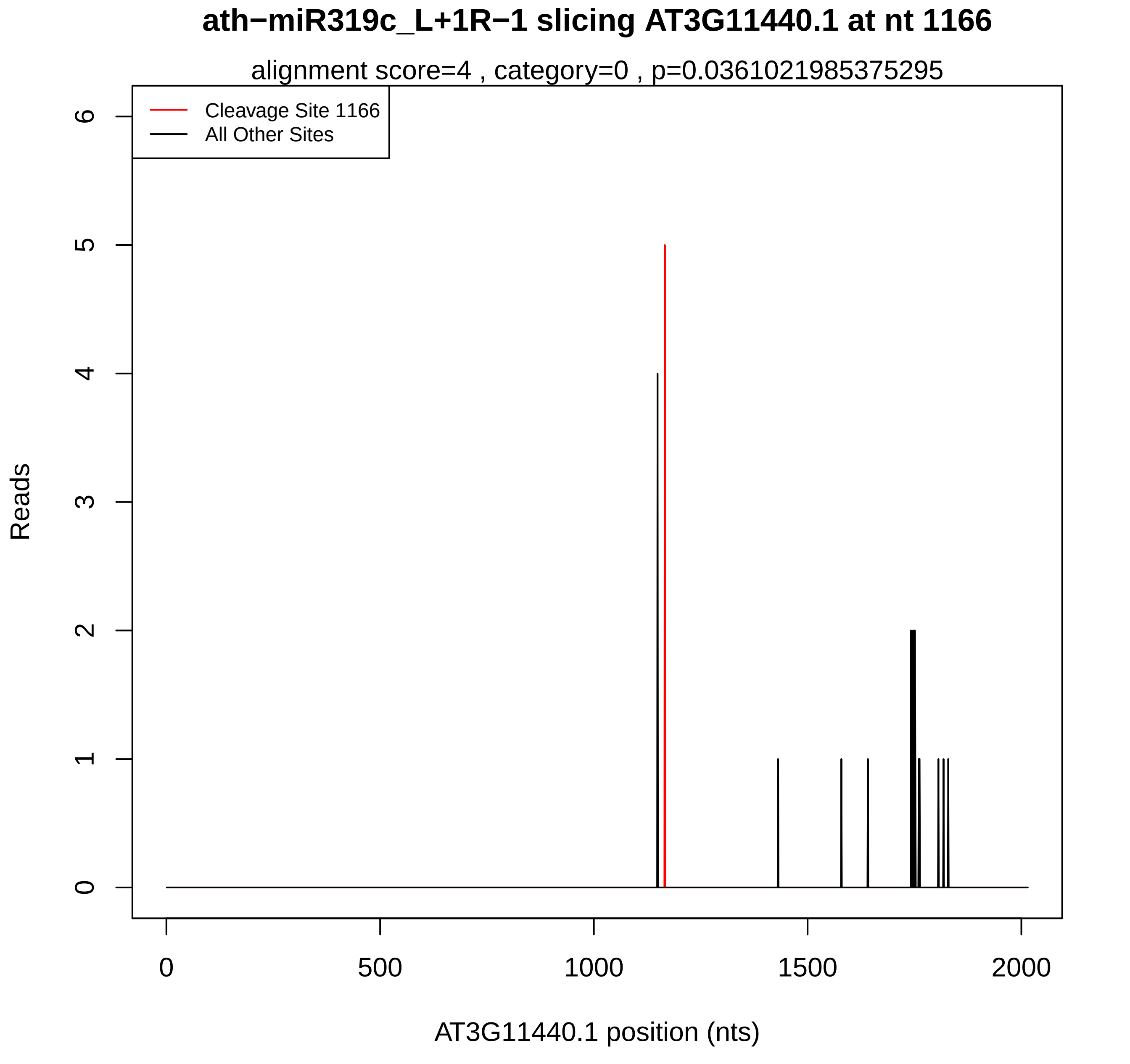

ath−miR319c_L+1R−1 slicing AT3G11440.1 at nt 1166
alignment score=4 , category=0 , p=0.0361021985375295
Cleavage Site 1166
All Other Sites
Reads
0
1
2
3
4
5
6
0
500
1000
1500
2000
AT3G11440.1 position (nts)

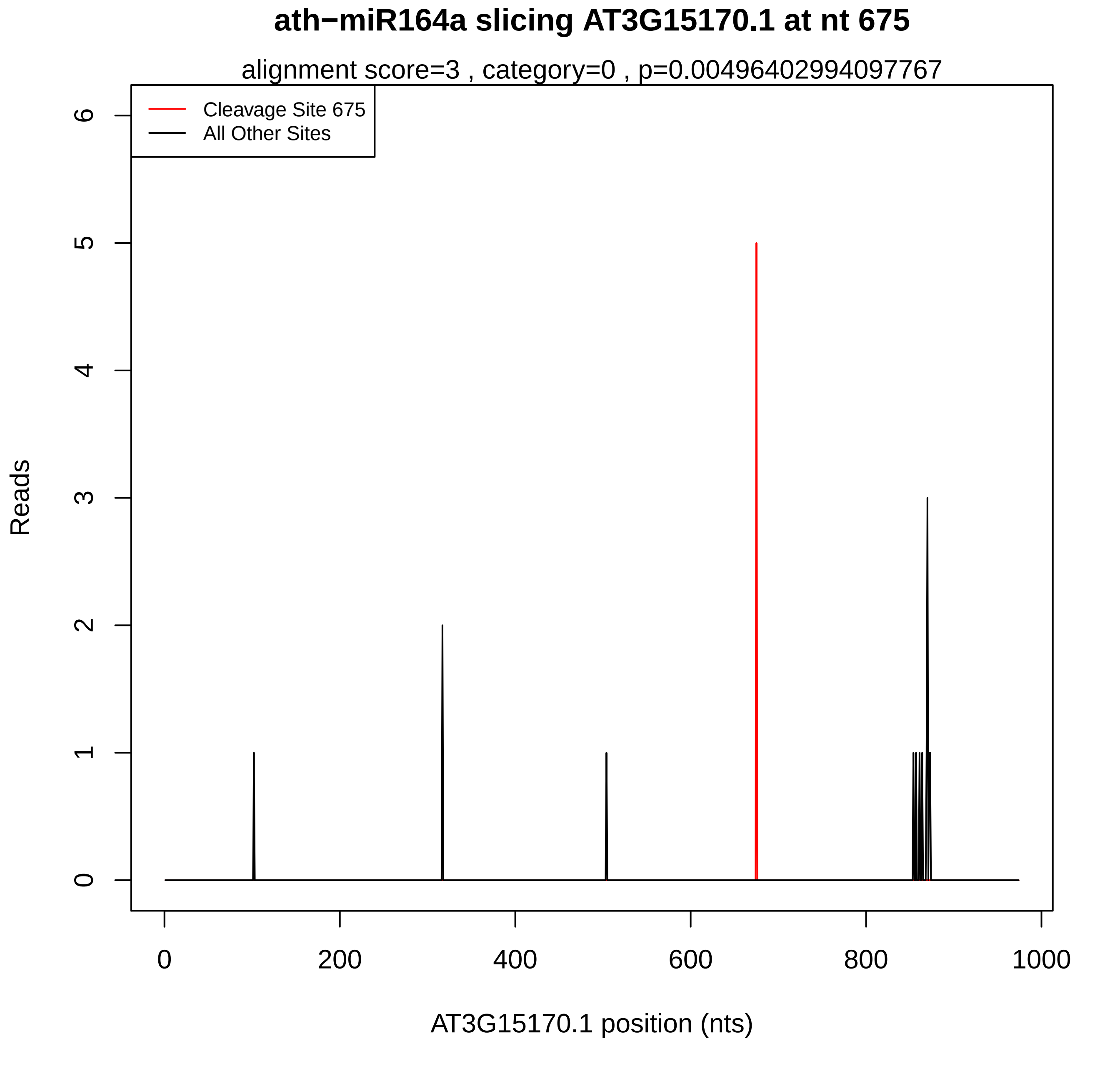

ath−miR164a slicing AT3G15170.1 at nt 675
alignment score=3 , category=0 , p=0.00496402994097767
Cleavage Site 675
All Other Sites
Reads
0
1
2
3
4
5
6
0
200
400
600
800
1000
AT3G15170.1 position (nts)

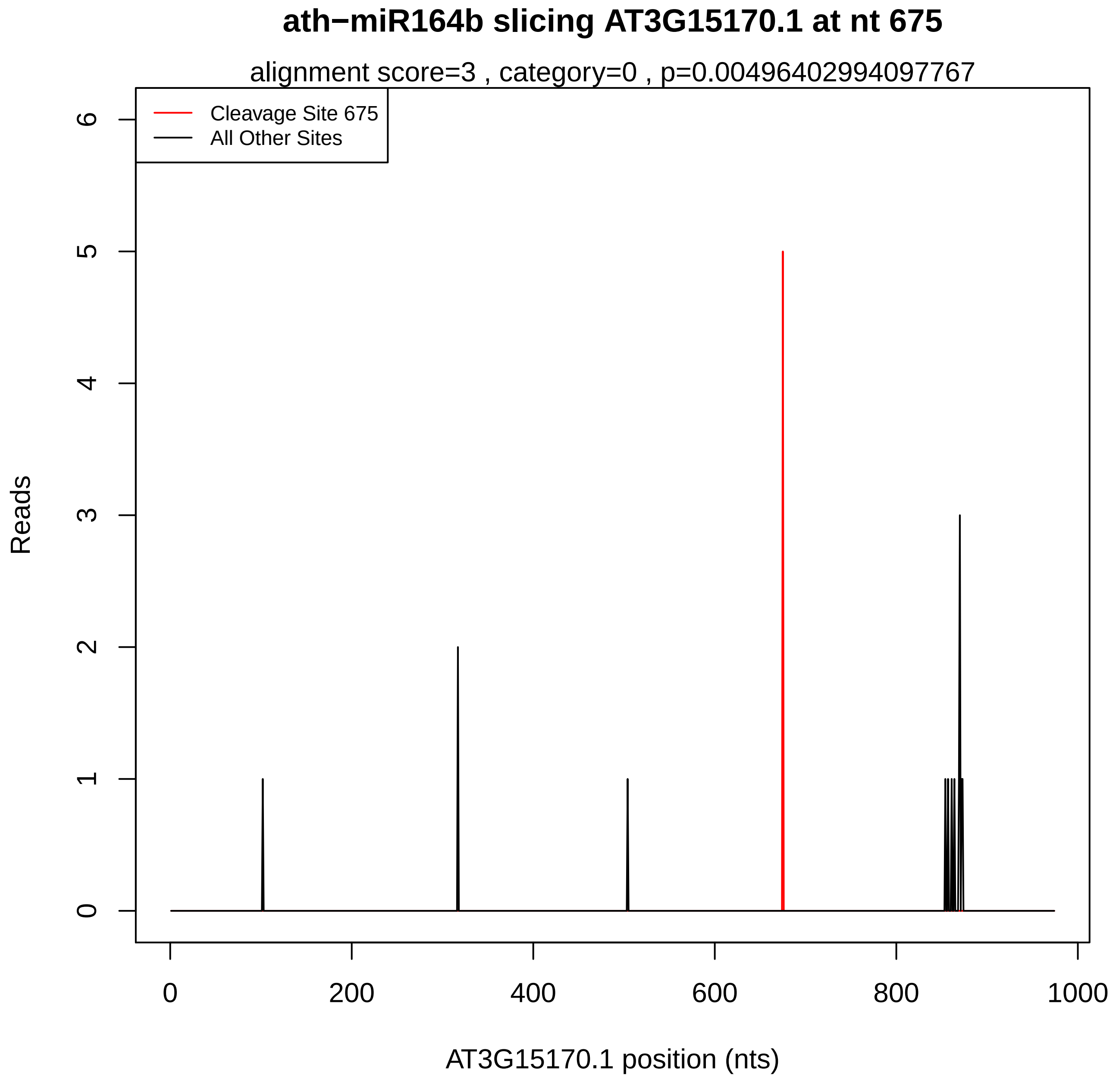
ath−miR164b slicing AT3G15170.1 at nt 675
alignment score=3 , category=0 , p=0.00496402994097767
Cleavage Site 675
All Other Sites
Reads
0
1
2
3
4
5
6
0
200
400
600
800
1000
AT3G15170.1 position (nts)

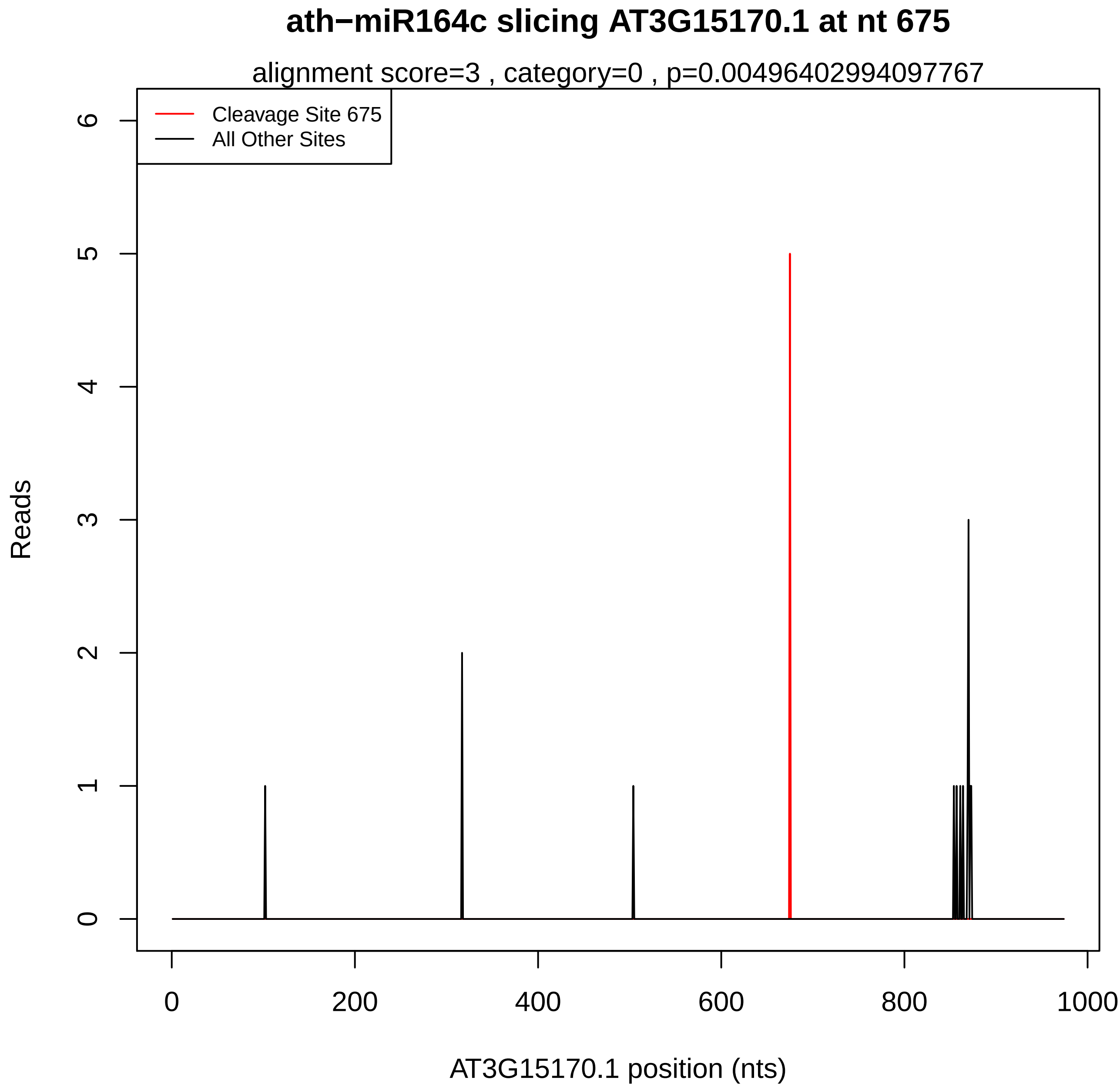

ath−miR164c slicing AT3G15170.1 at nt 675
alignment score=3 , category=0 , p=0.00496402994097767
Cleavage Site 675
All Other Sites
Reads
0
1
2
3
4
5
6
0
200
400
600
800
1000
AT3G15170.1 position (nts)

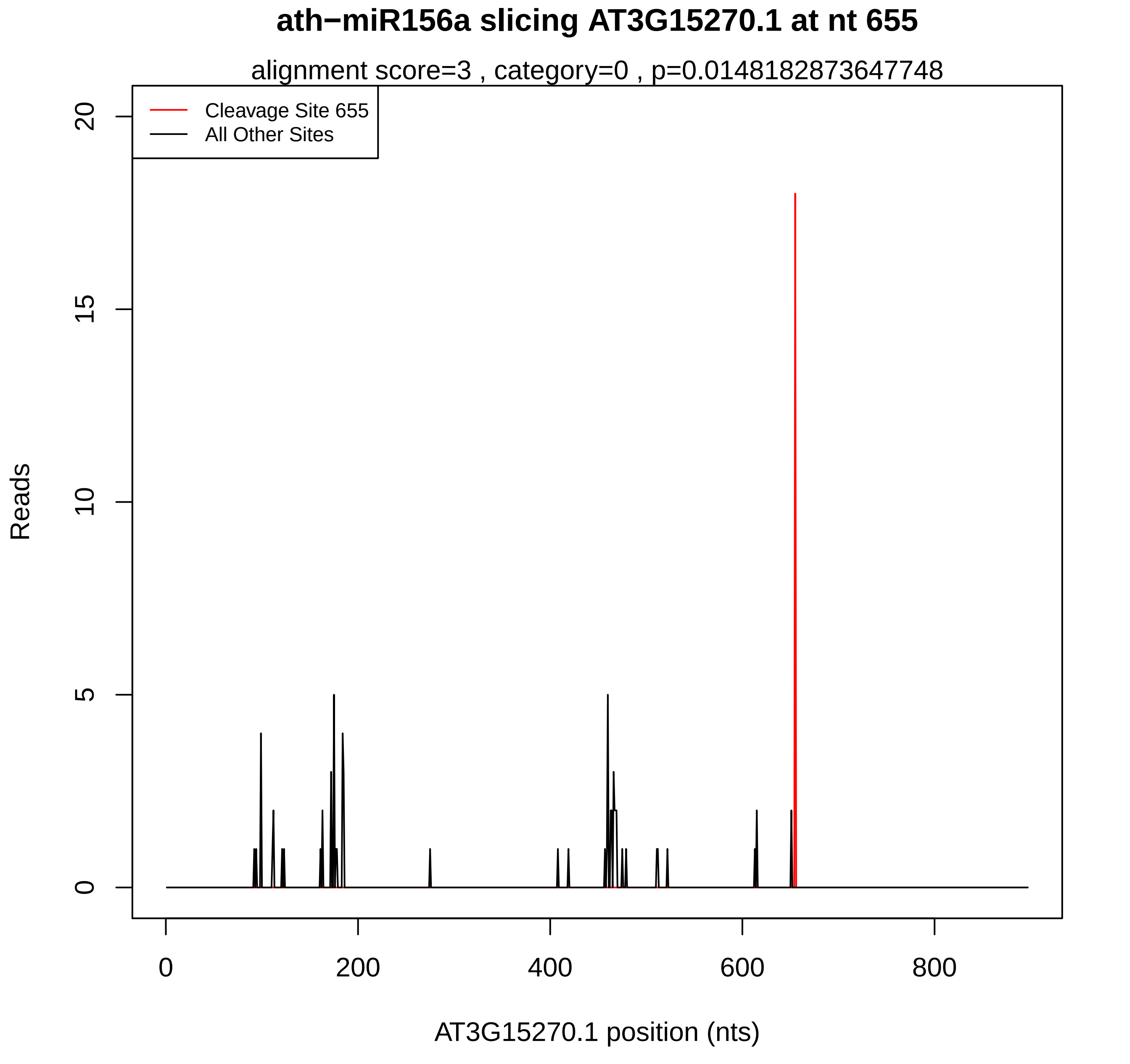
ath−miR156a slicing AT3G15270.1 at nt 655
alignment score=3 , category=0 , p=0.0148182873647748
Cleavage Site 655
All Other Sites
Reads
0
5
10
15
20
0
200
400
600
800
AT3G15270.1 position (nts)

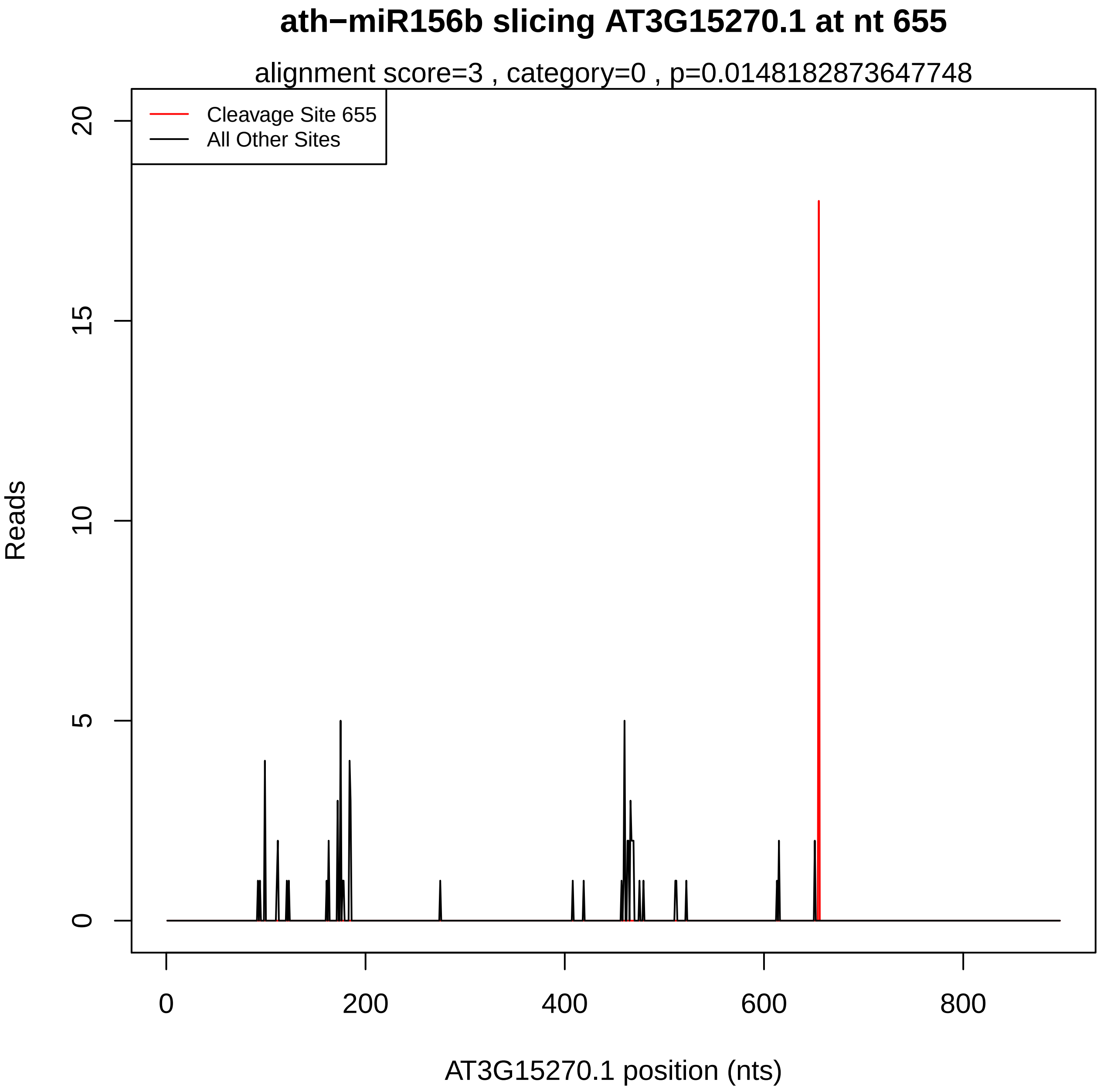
ath−miR156b slicing AT3G15270.1 at nt 655
alignment score=3 , category=0 , p=0.0148182873647748
Cleavage Site 655
All Other Sites
Reads
0
5
10
15
20
0
200
400
600
800
AT3G15270.1 position (nts)

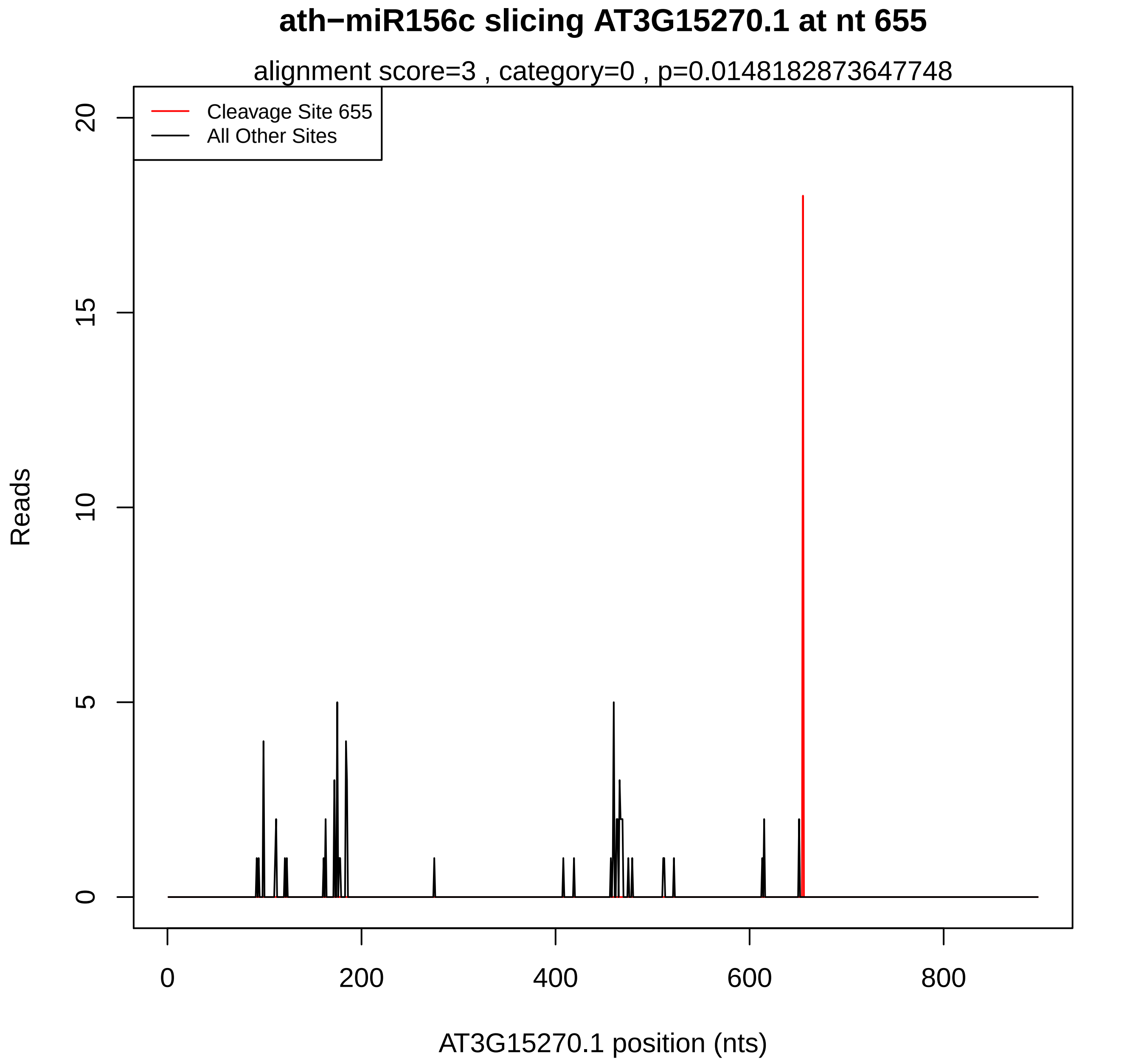
ath−miR156c slicing AT3G15270.1 at nt 655
alignment score=3 , category=0 , p=0.0148182873647748
Cleavage Site 655
All Other Sites
Reads
0
5
10
15
20
0
200
400
600
800
AT3G15270.1 position (nts)

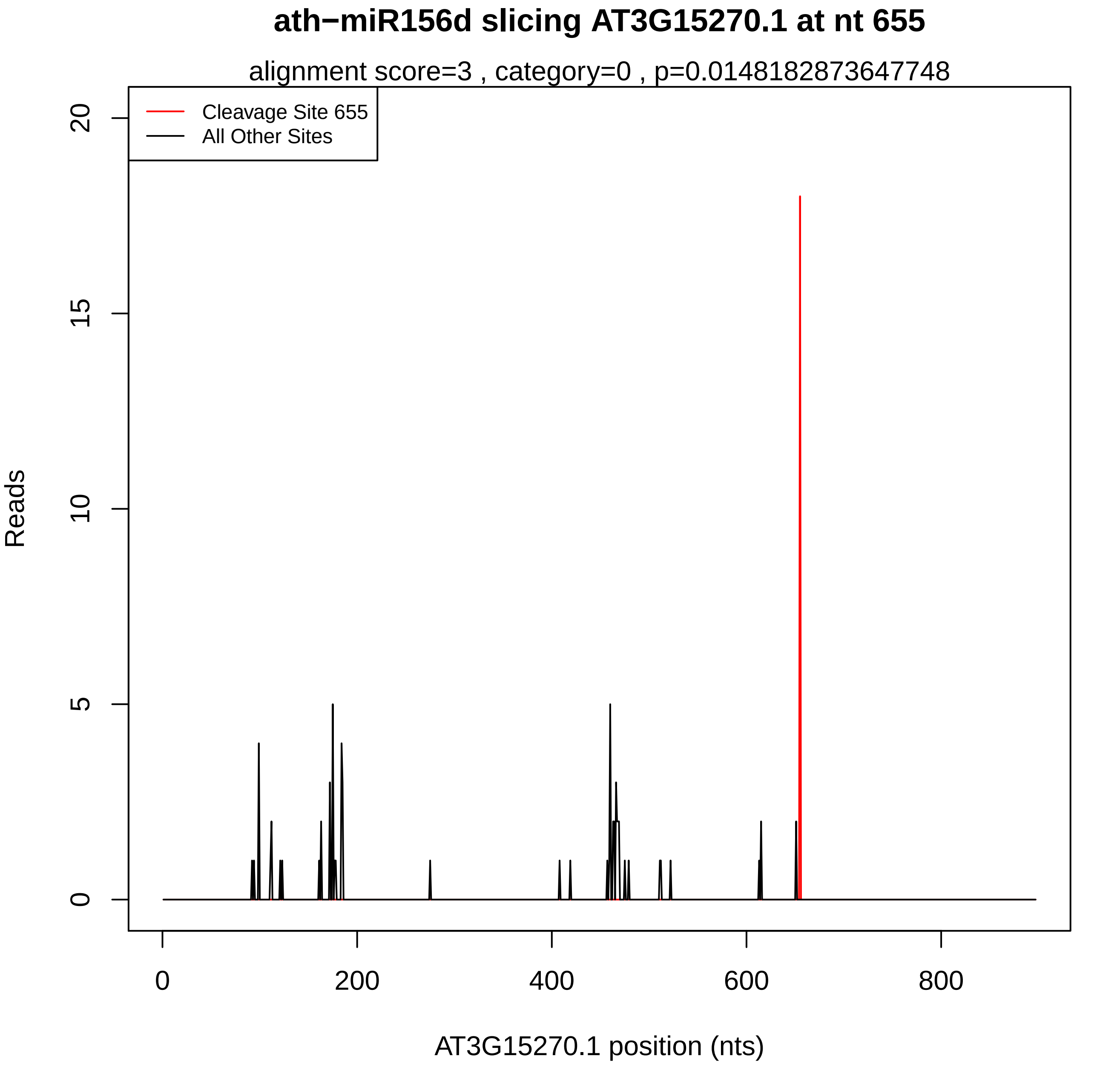
ath−miR156d slicing AT3G15270.1 at nt 655
alignment score=3 , category=0 , p=0.0148182873647748
Cleavage Site 655
All Other Sites
Reads
0
5
10
15
20
0
200
400
600
800
AT3G15270.1 position (nts)

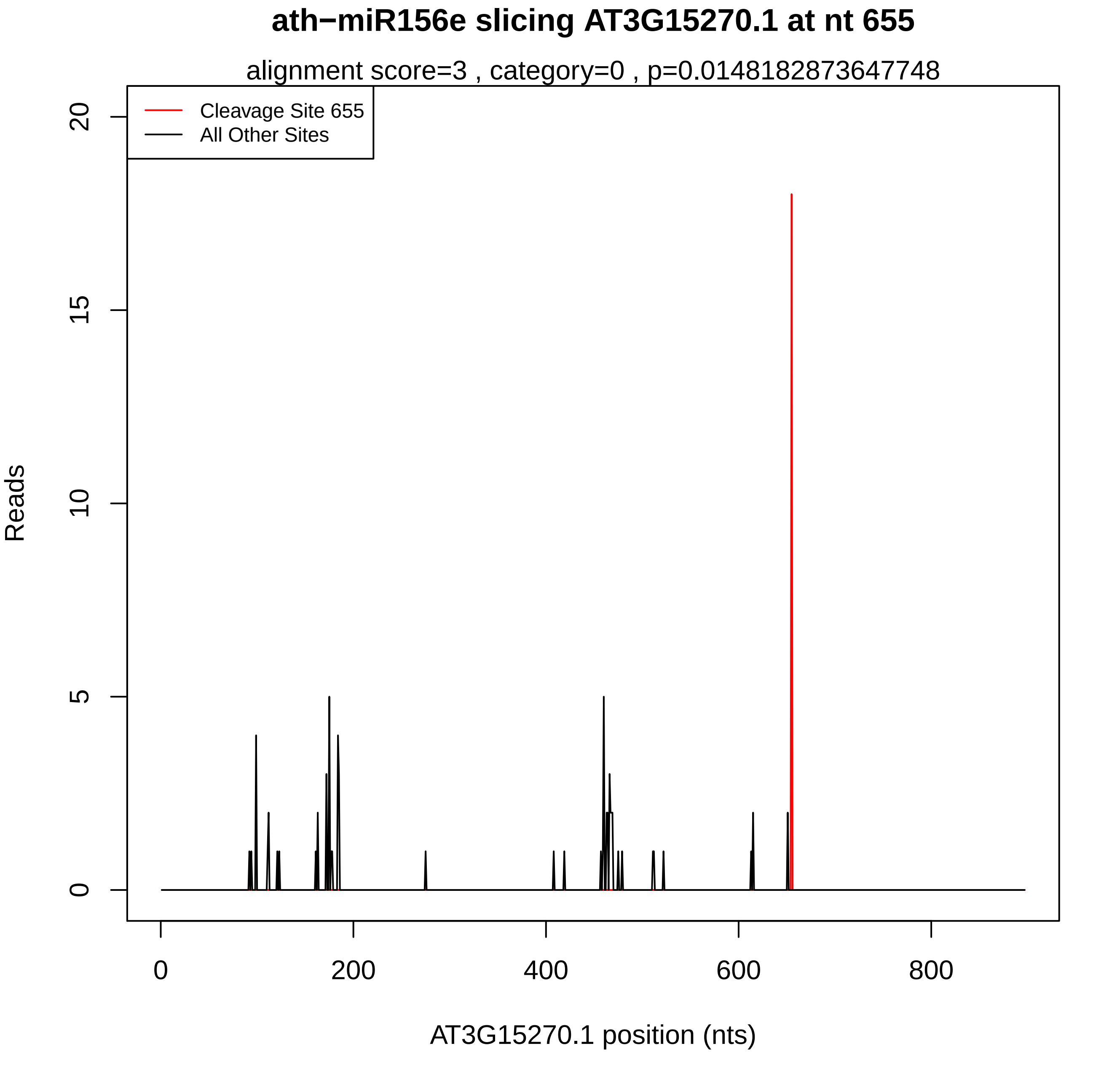
ath−miR156e slicing AT3G15270.1 at nt 655
alignment score=3 , category=0 , p=0.0148182873647748
Cleavage Site 655
All Other Sites
Reads
0
5
10
15
20
0
200
400
600
800
AT3G15270.1 position (nts)

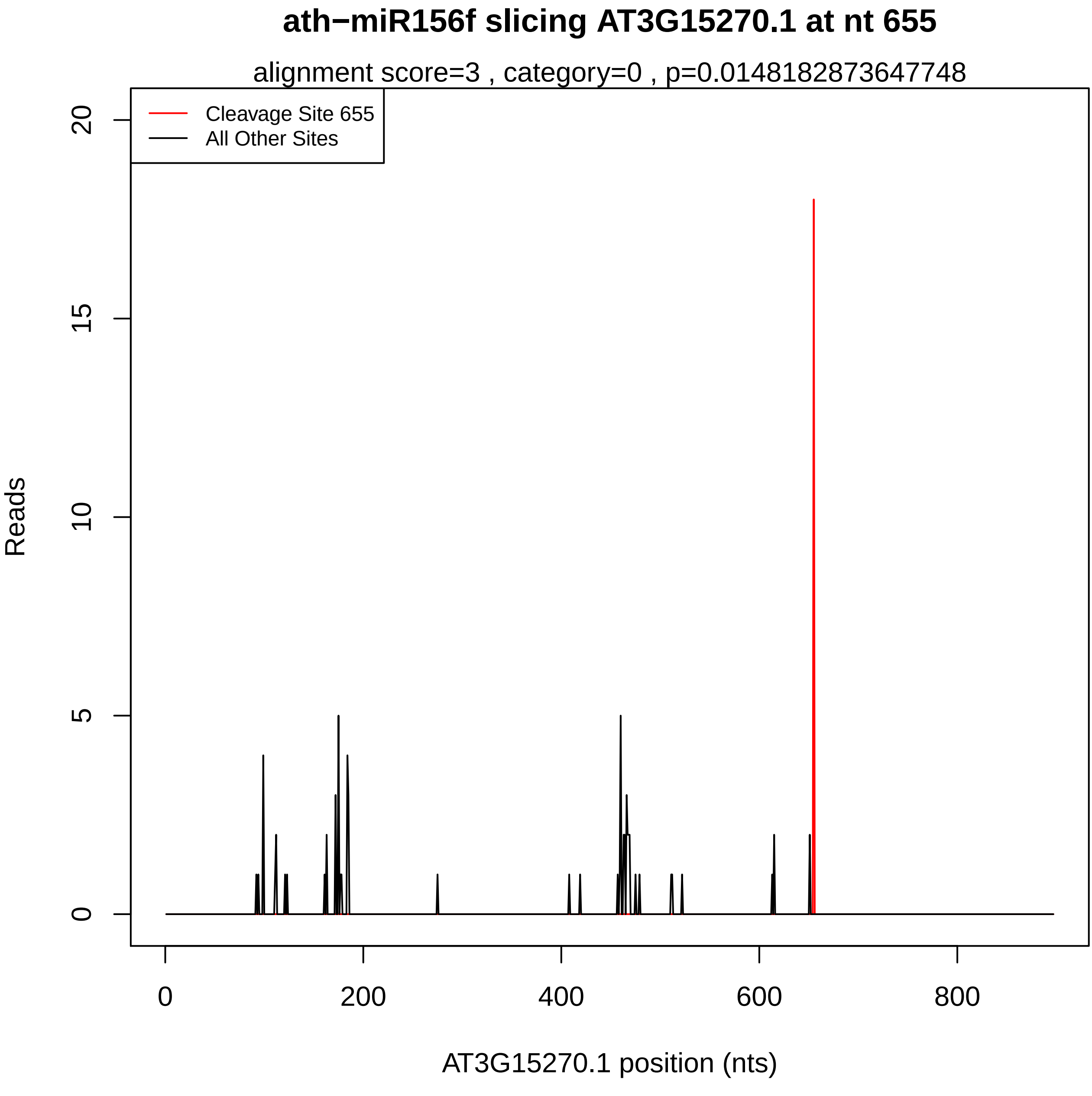
ath−miR156f slicing AT3G15270.1 at nt 655
alignment score=3 , category=0 , p=0.0148182873647748
Cleavage Site 655
All Other Sites
Reads
0
5
10
15
20
0
200
400
600
800
AT3G15270.1 position (nts)

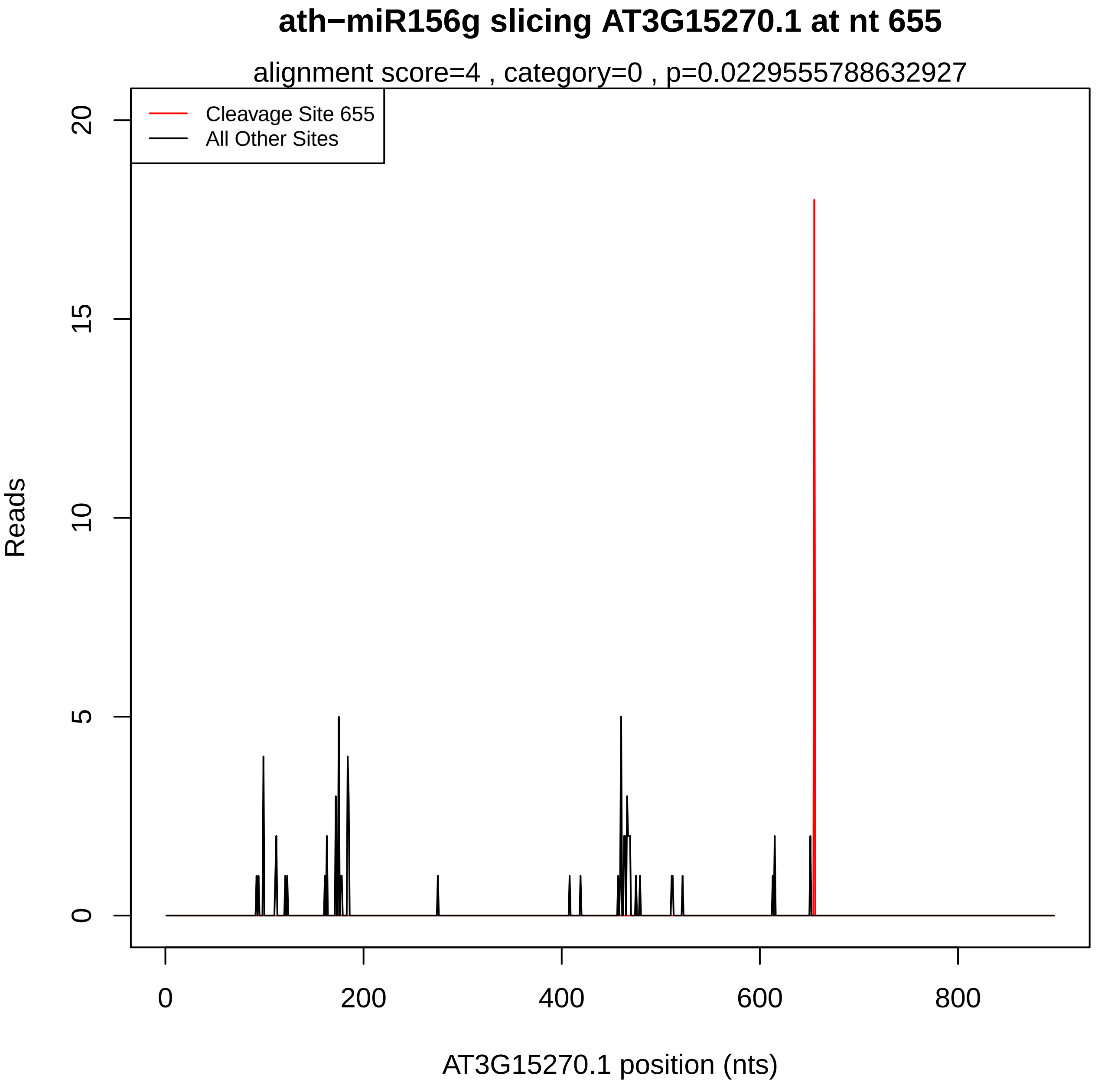
ath−miR156g slicing AT3G15270.1 at nt 655
alignment score=4 , category=0 , p=0.0229555788632927
Cleavage Site 655
All Other Sites
Reads
0
5
10
15
20
0
200
400
600
800
AT3G15270.1 position (nts)

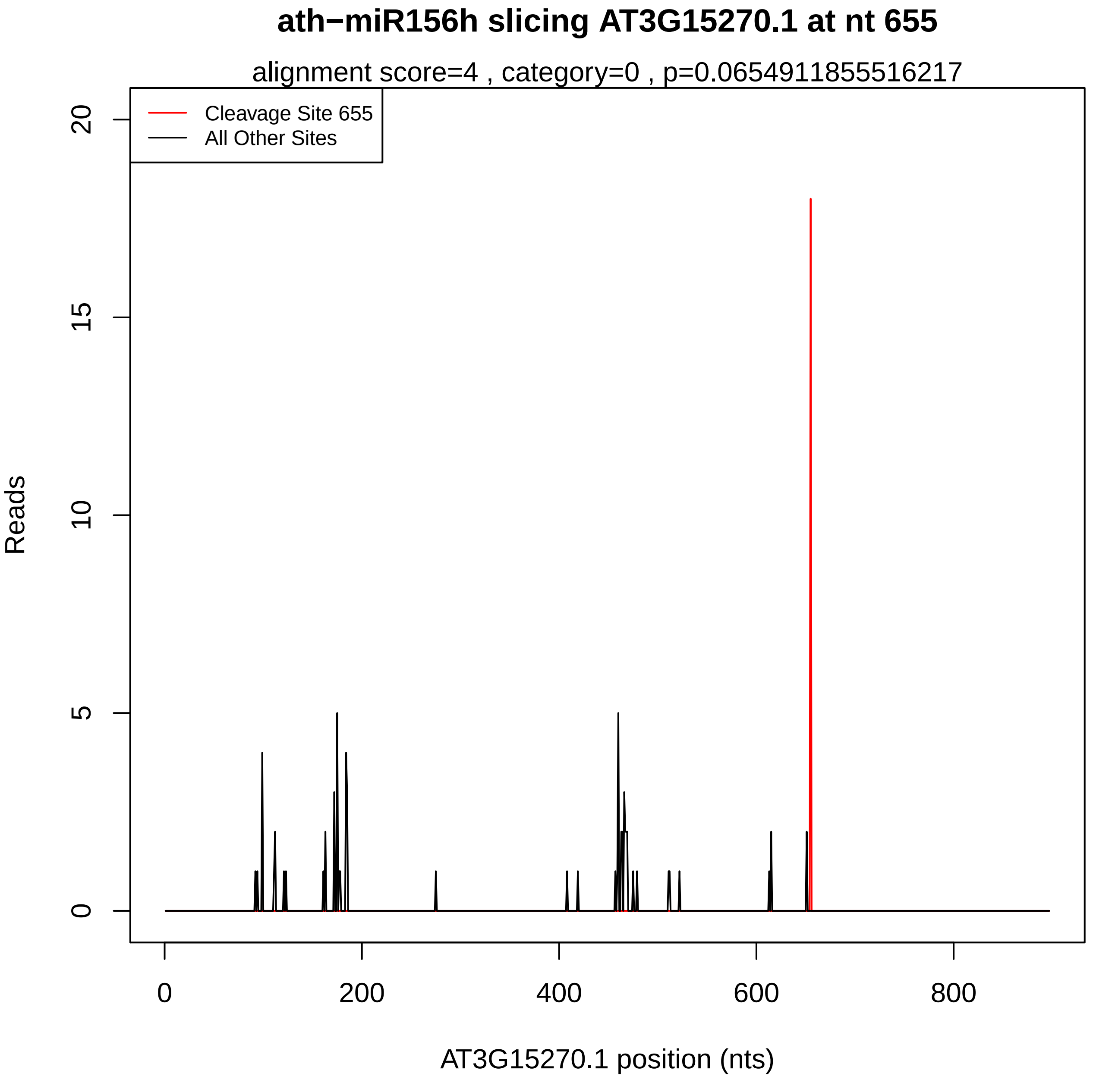
ath−miR156h slicing AT3G15270.1 at nt 655
alignment score=4 , category=0 , p=0.0654911855516217
Cleavage Site 655
All Other Sites
Reads
0
5
10
15
20
0
200
400
600
800
AT3G15270.1 position (nts)

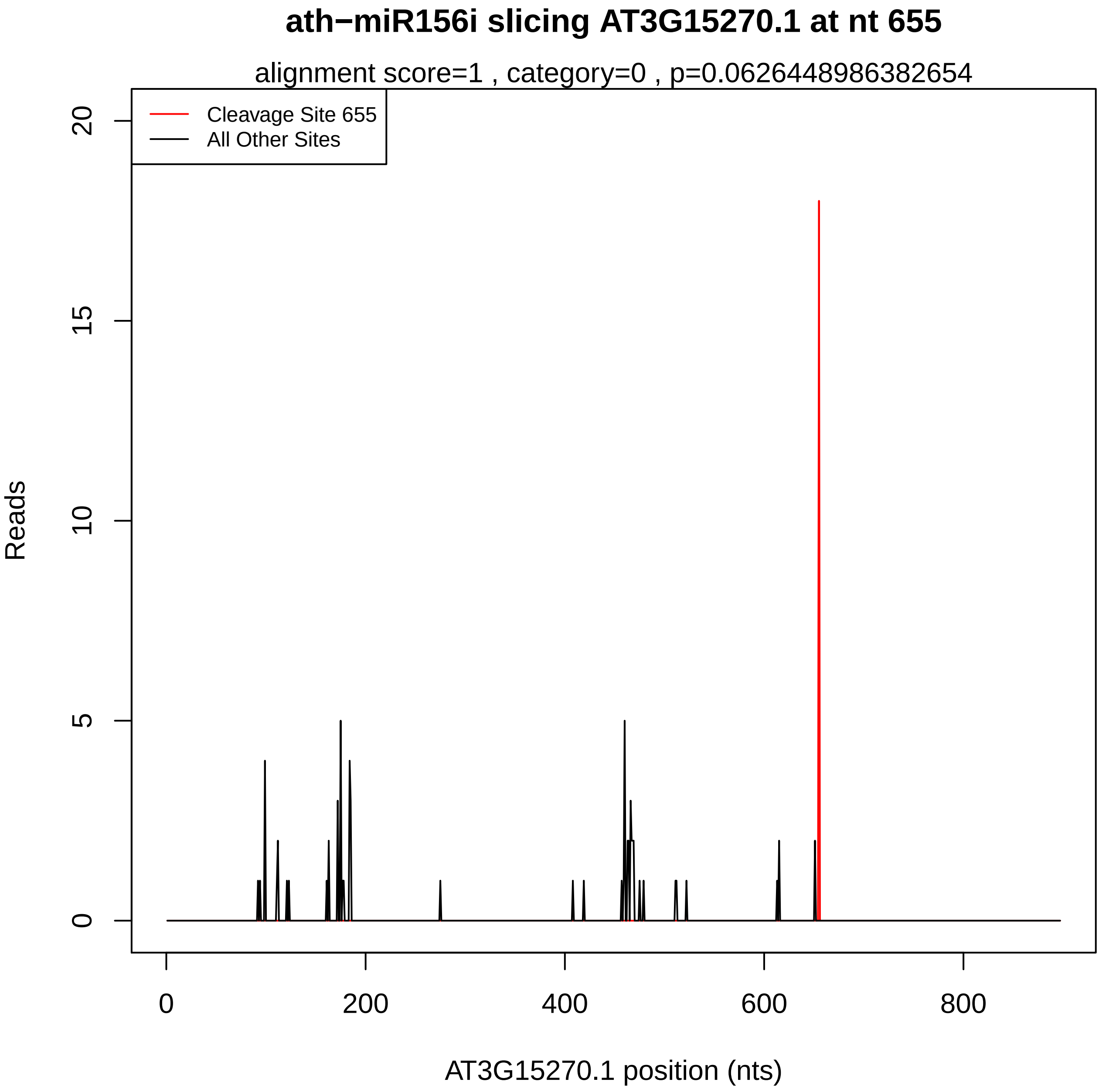
ath−miR156i slicing AT3G15270.1 at nt 655
alignment score=1 , category=0 , p=0.0626448986382654
Cleavage Site 655
All Other Sites
Reads
0
5
10
15
20
0
200
400
600
800
AT3G15270.1 position (nts)

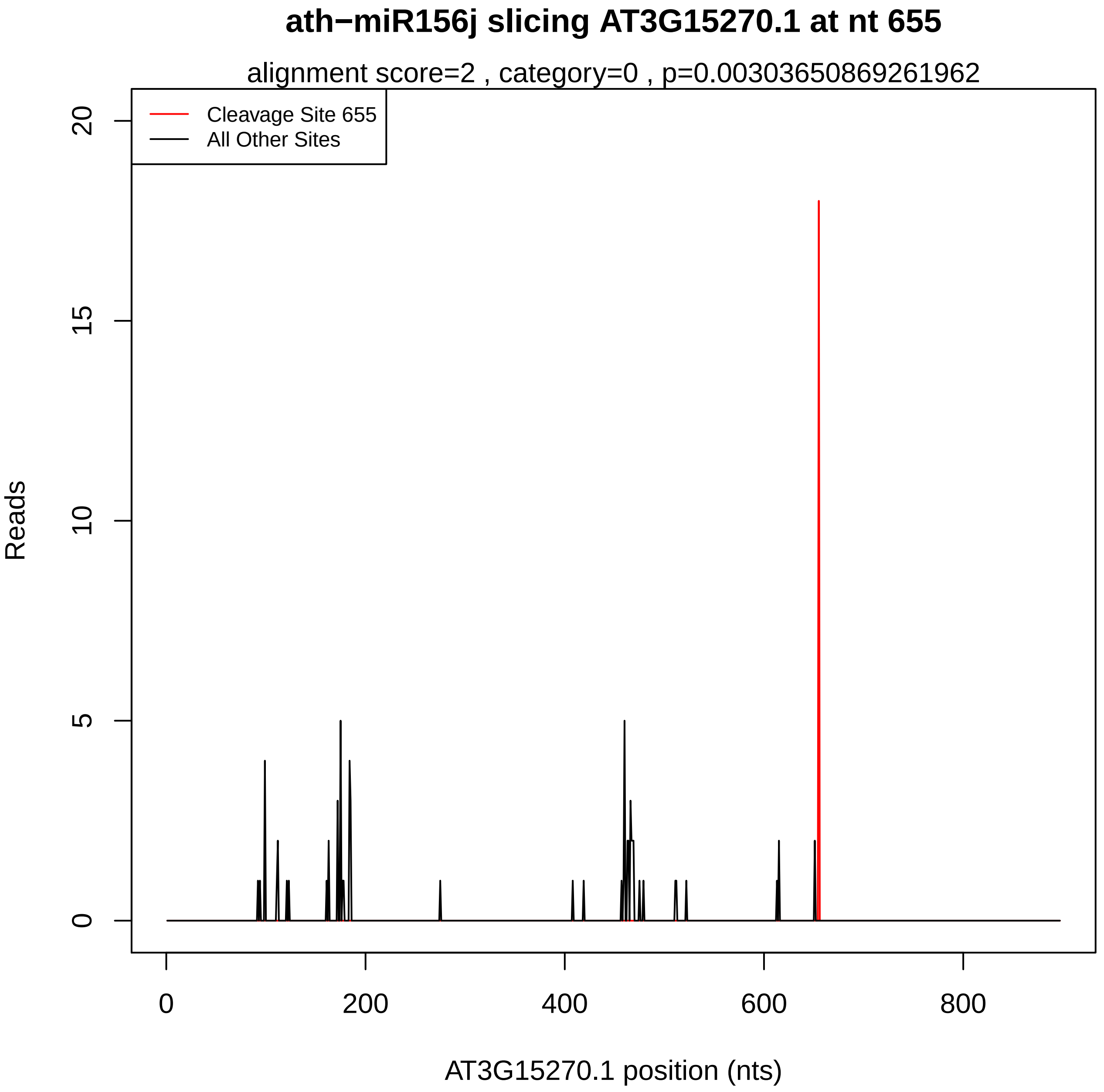

ath−miR156j slicing AT3G15270.1 at nt 655
alignment score=2 , category=0 , p=0.00303650869261962
Cleavage Site 655
All Other Sites
Reads
0
5
10
15
20
0
200
400
600
800
AT3G15270.1 position (nts)

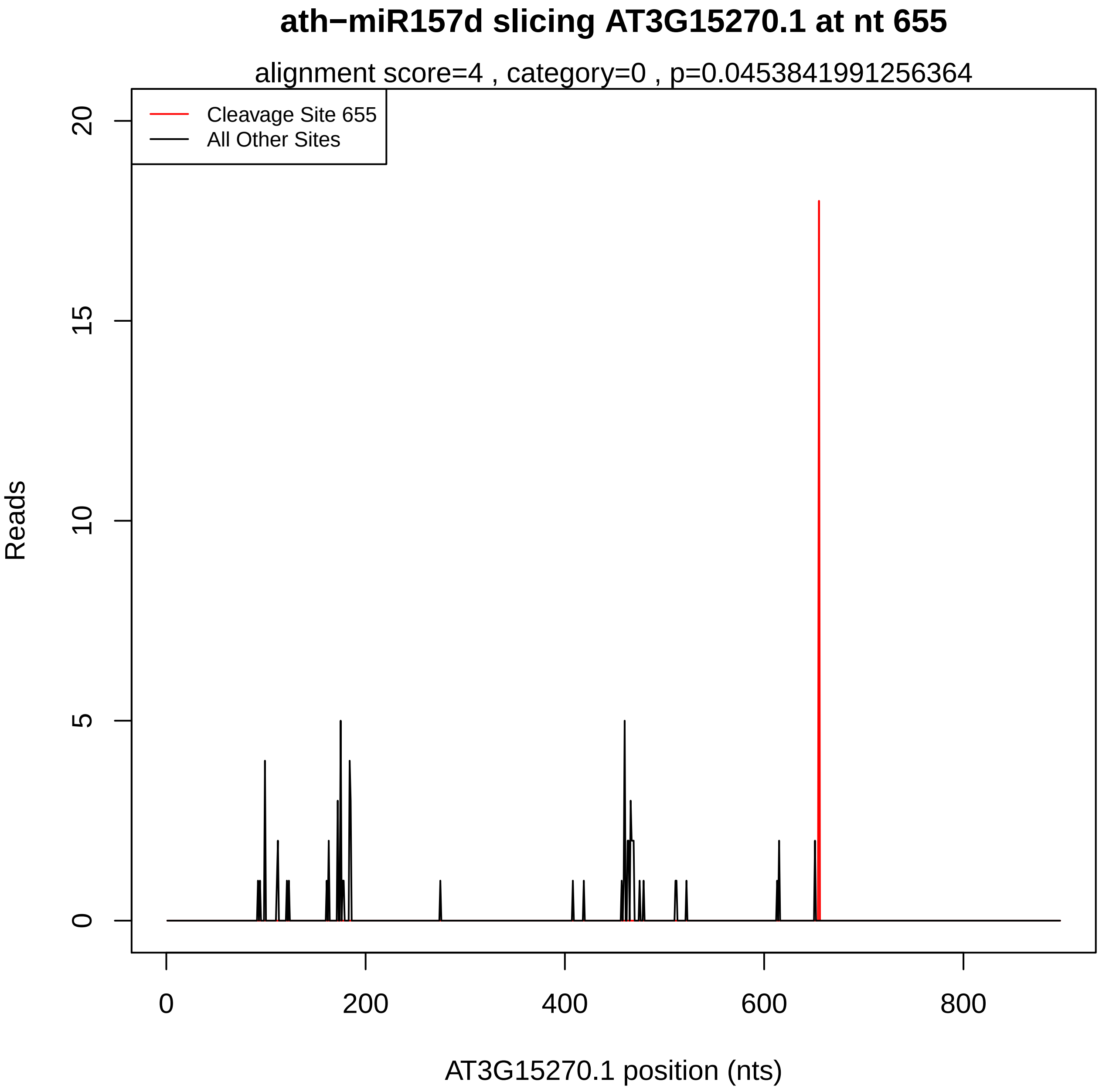

ath−miR157d slicing AT3G15270.1 at nt 655
alignment score=4 , category=0 , p=0.0453841991256364
Cleavage Site 655
All Other Sites
Reads
0
5
10
15
20
0
200
400
600
800
AT3G15270.1 position (nts)

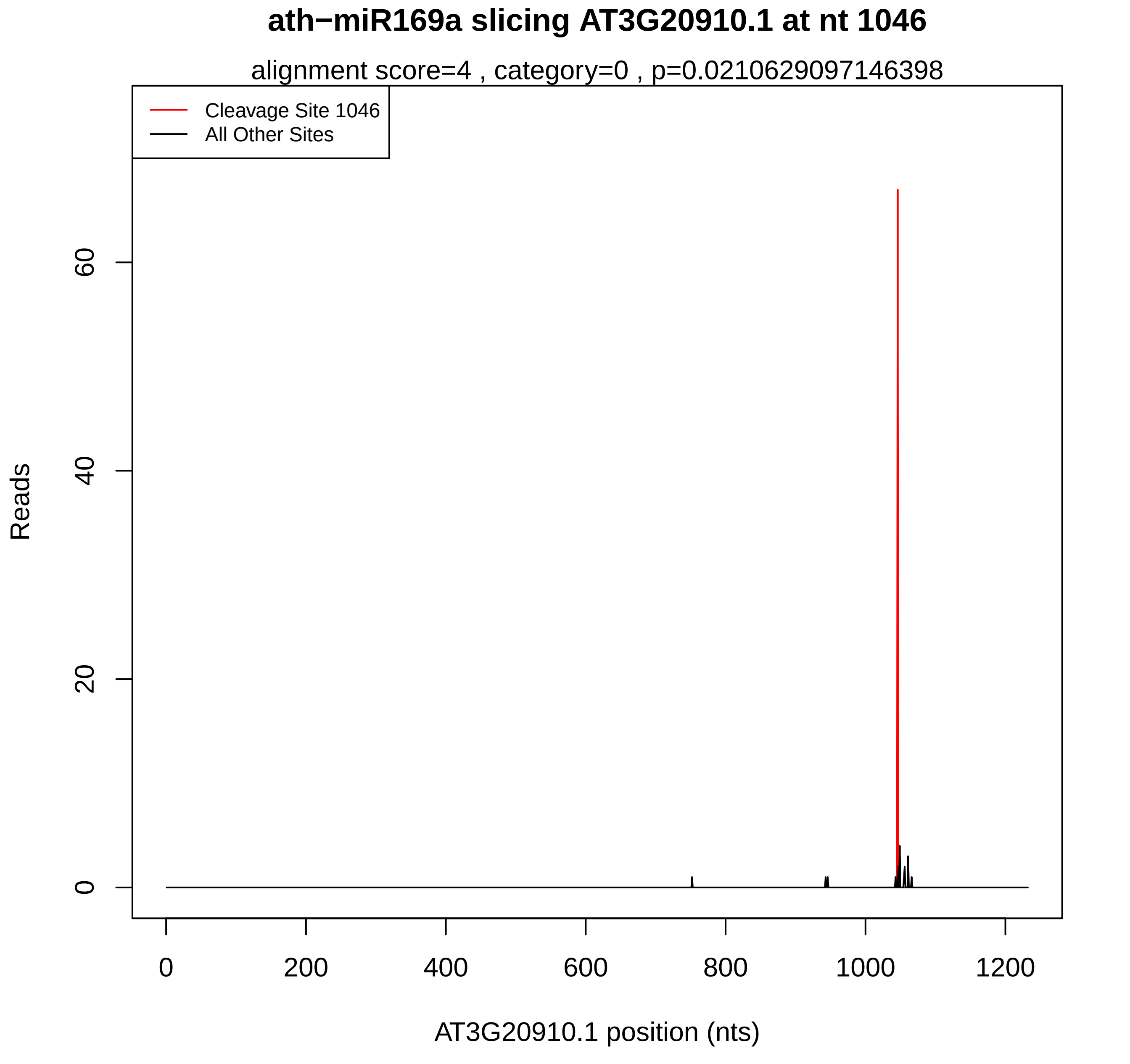
ath−miR169a slicing AT3G20910.1 at nt 1046
alignment score=4 , category=0 , p=0.0210629097146398
Cleavage Site 1046
All Other Sites
Reads
0
20
40
60
0
200
400
600
800
1000
1200
AT3G20910.1 position (nts)

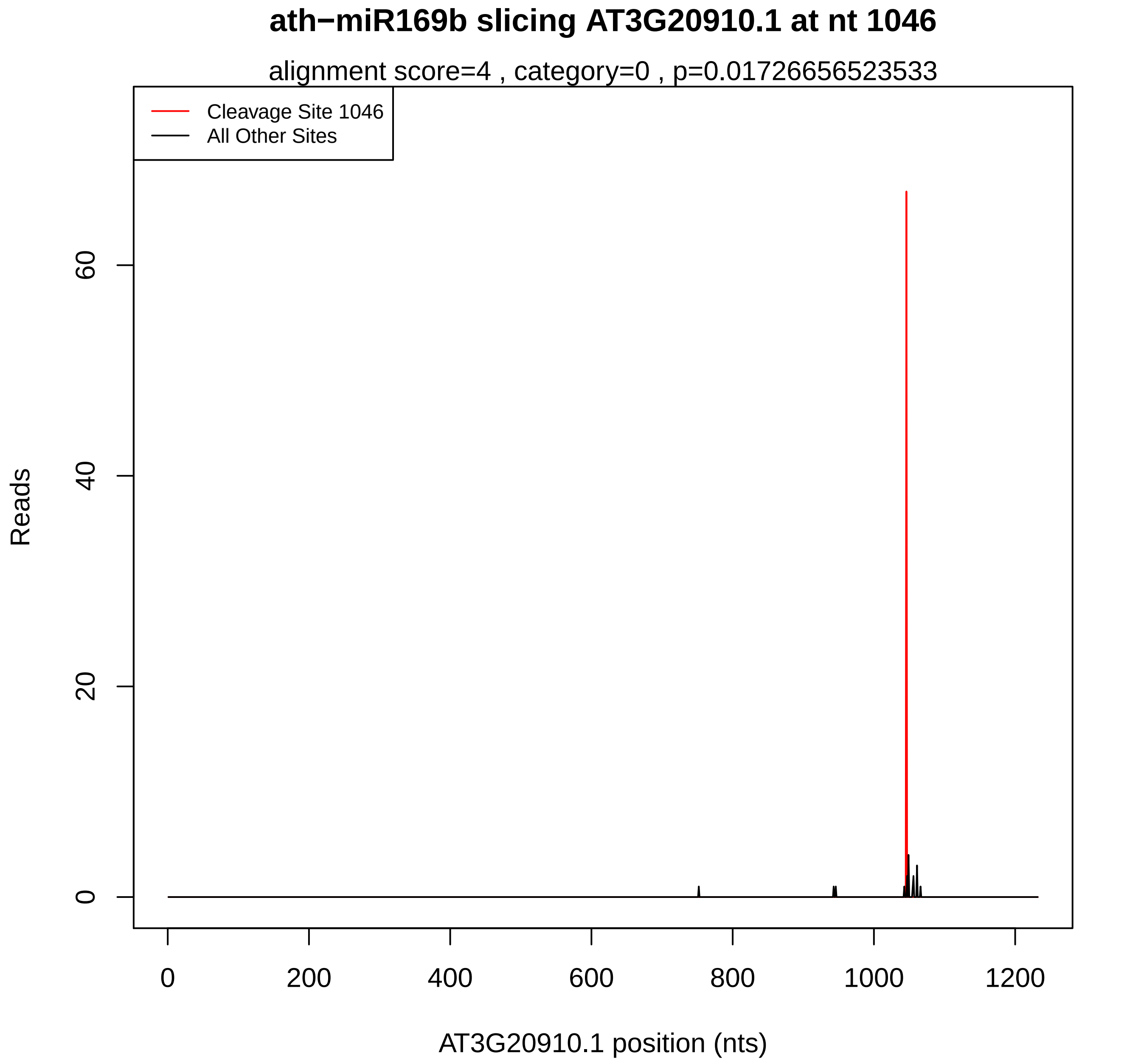
ath−miR169b slicing AT3G20910.1 at nt 1046
alignment score=4 , category=0 , p=0.01726656523533
Cleavage Site 1046
All Other Sites
Reads
0
20
40
60
0
200
400
600
800
1000
1200
AT3G20910.1 position (nts)

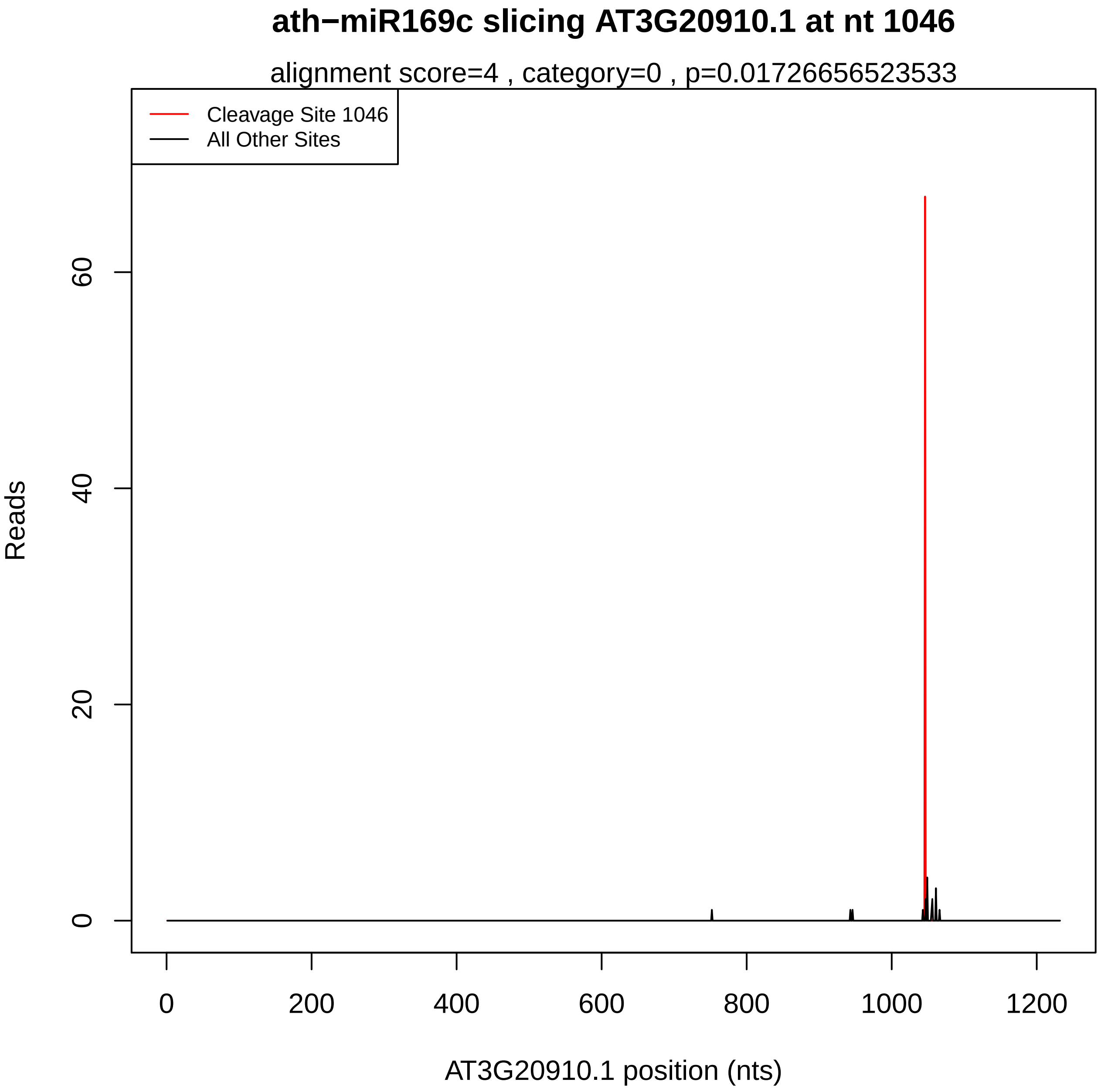

ath−miR169c slicing AT3G20910.1 at nt 1046
alignment score=4 , category=0 , p=0.01726656523533
Cleavage Site 1046
All Other Sites
Reads
0
20
40
60
0
200
400
600
800
1000
1200
AT3G20910.1 position (nts)

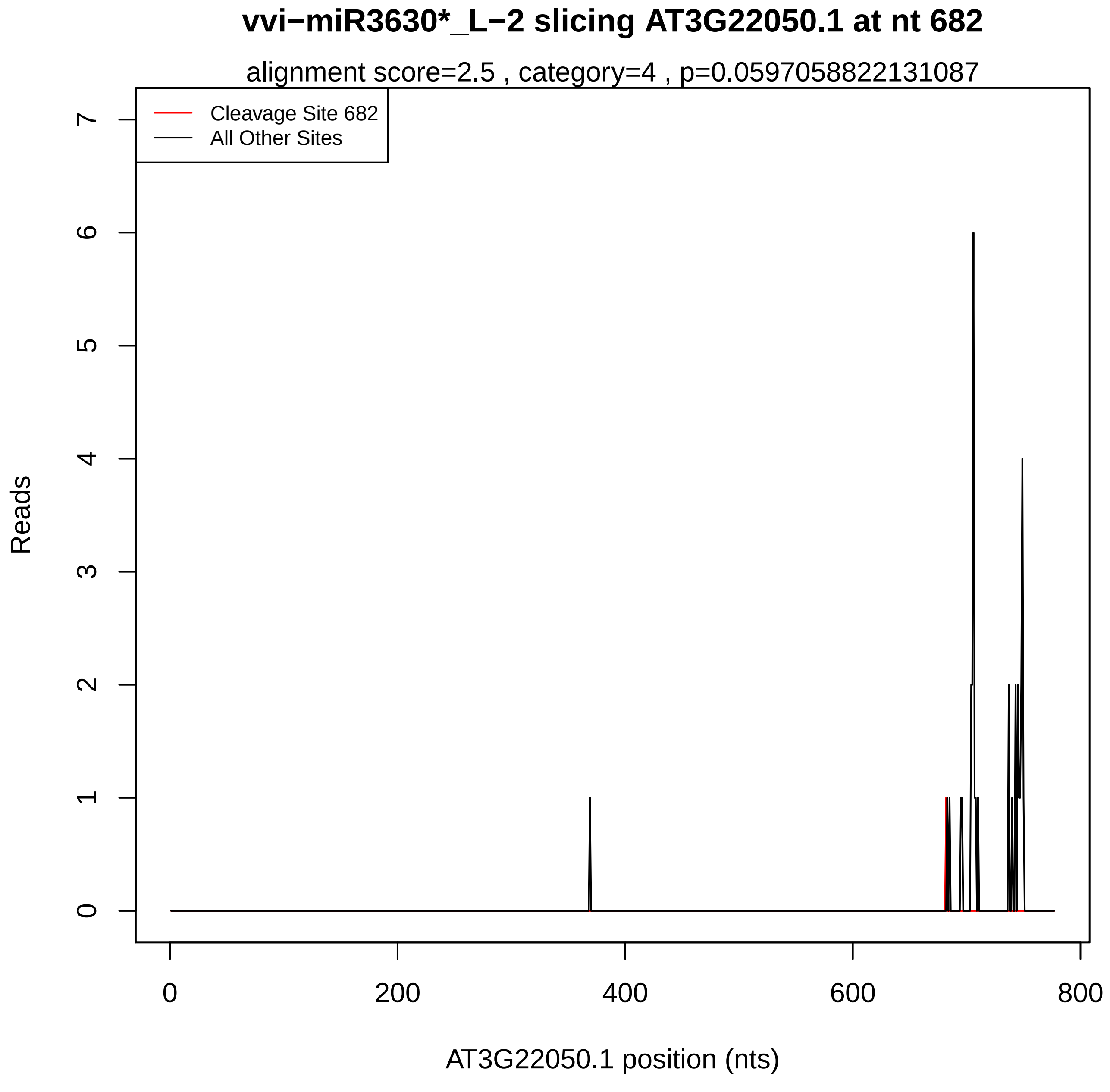

vvi−miR3630*_L−2 slicing AT3G22050.1 at nt 682
alignment score=2.5 , category=4 , p=0.0597058822131087
Cleavage Site 682
All Other Sites
Reads
0
1
2
3
4
5
6
7
0
200
400
600
800
AT3G22050.1 position (nts)

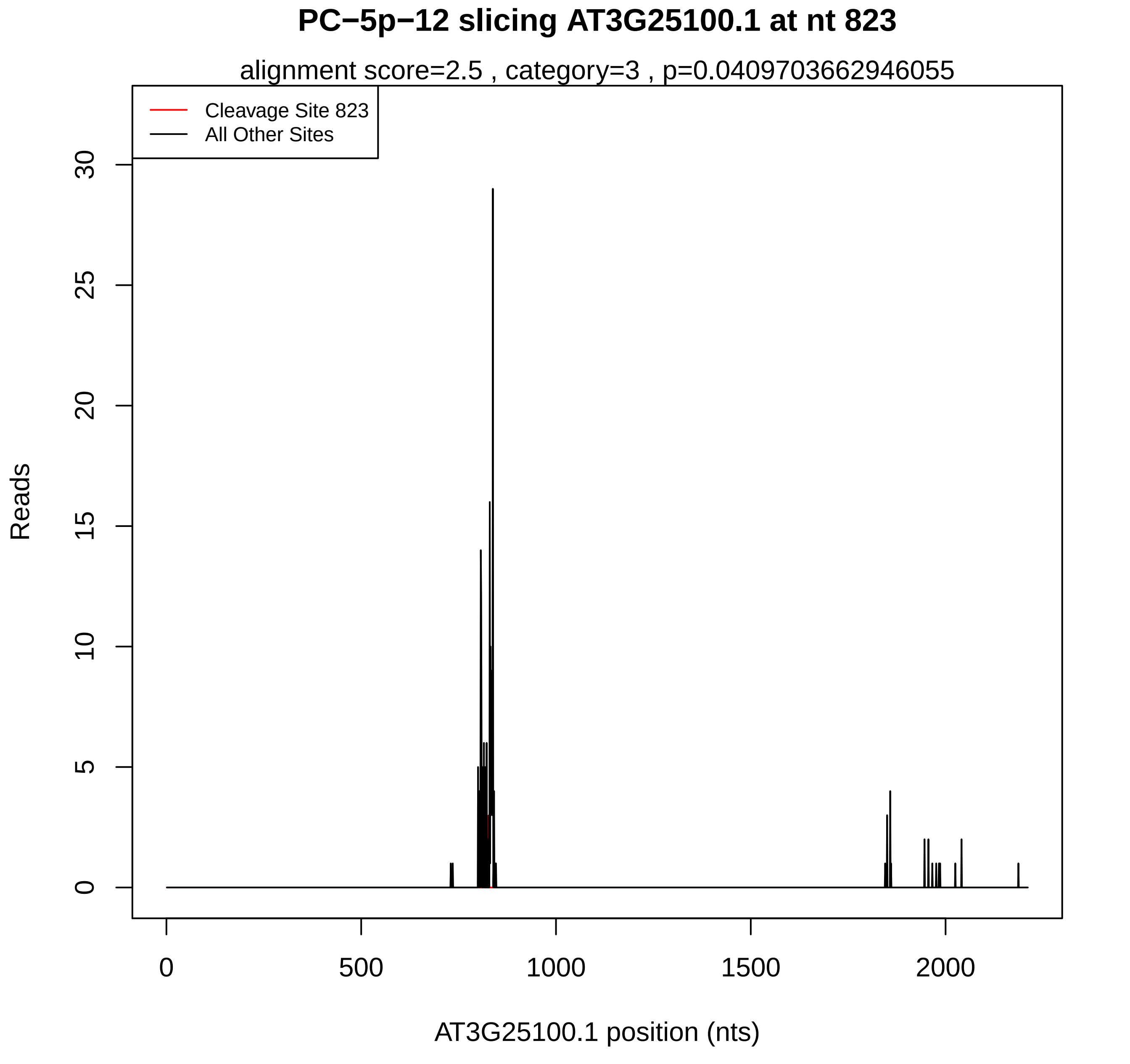
PC−5p−12 slicing AT3G25100.1 at nt 823
alignment score=2.5 , category=3 , p=0.0409703662946055
Cleavage Site 823
All Other Sites
Reads
0
5
10
15
20
25
30
0
500
1000
1500
2000
AT3G25100.1 position (nts)

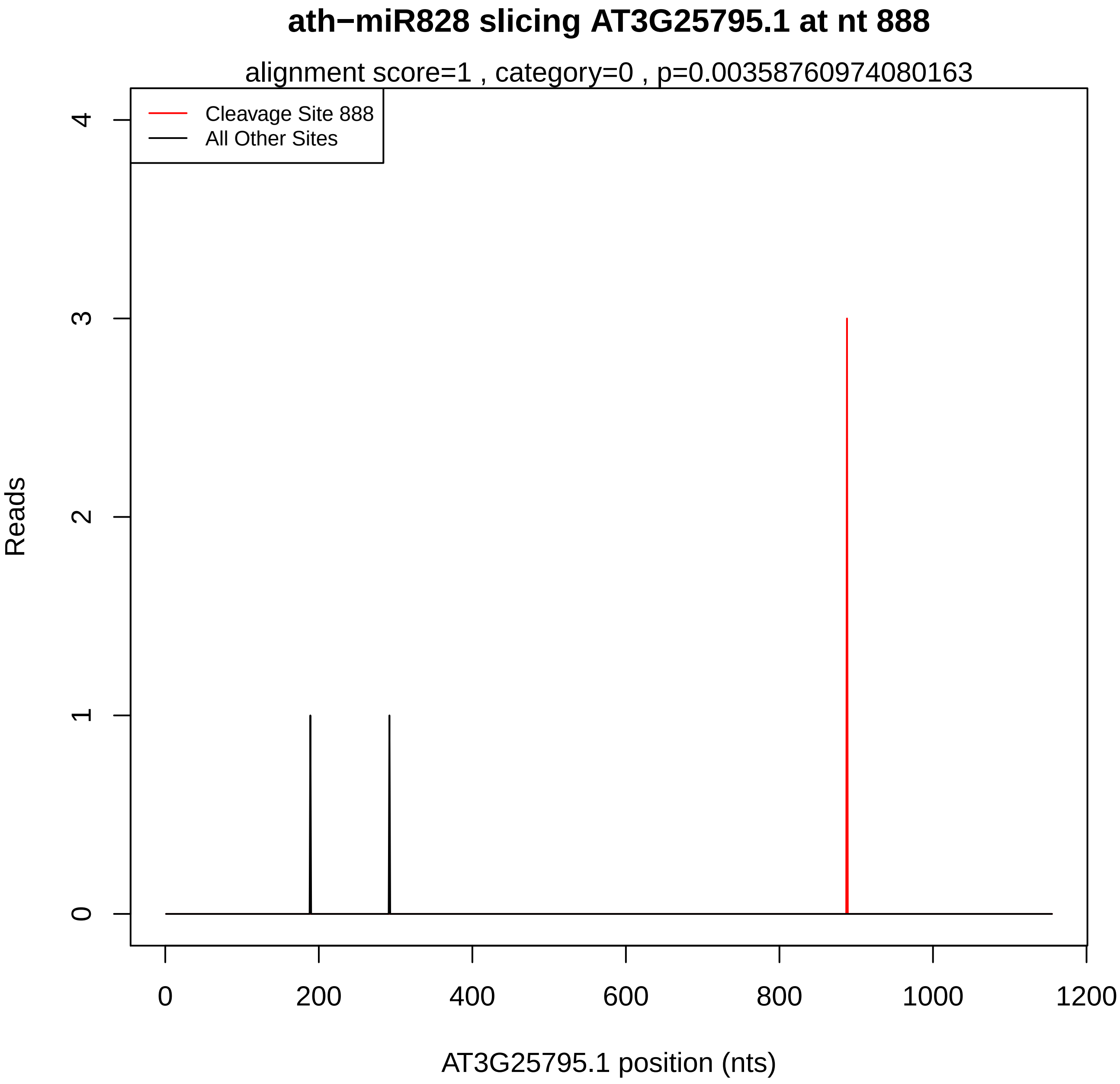

ath−miR828 slicing AT3G25795.1 at nt 888
alignment score=1 , category=0 , p=0.00358760974080163
Cleavage Site 888
All Other Sites
Reads
0
1
2
3
4
0
200
400
600
800
1000
1200
AT3G25795.1 position (nts)

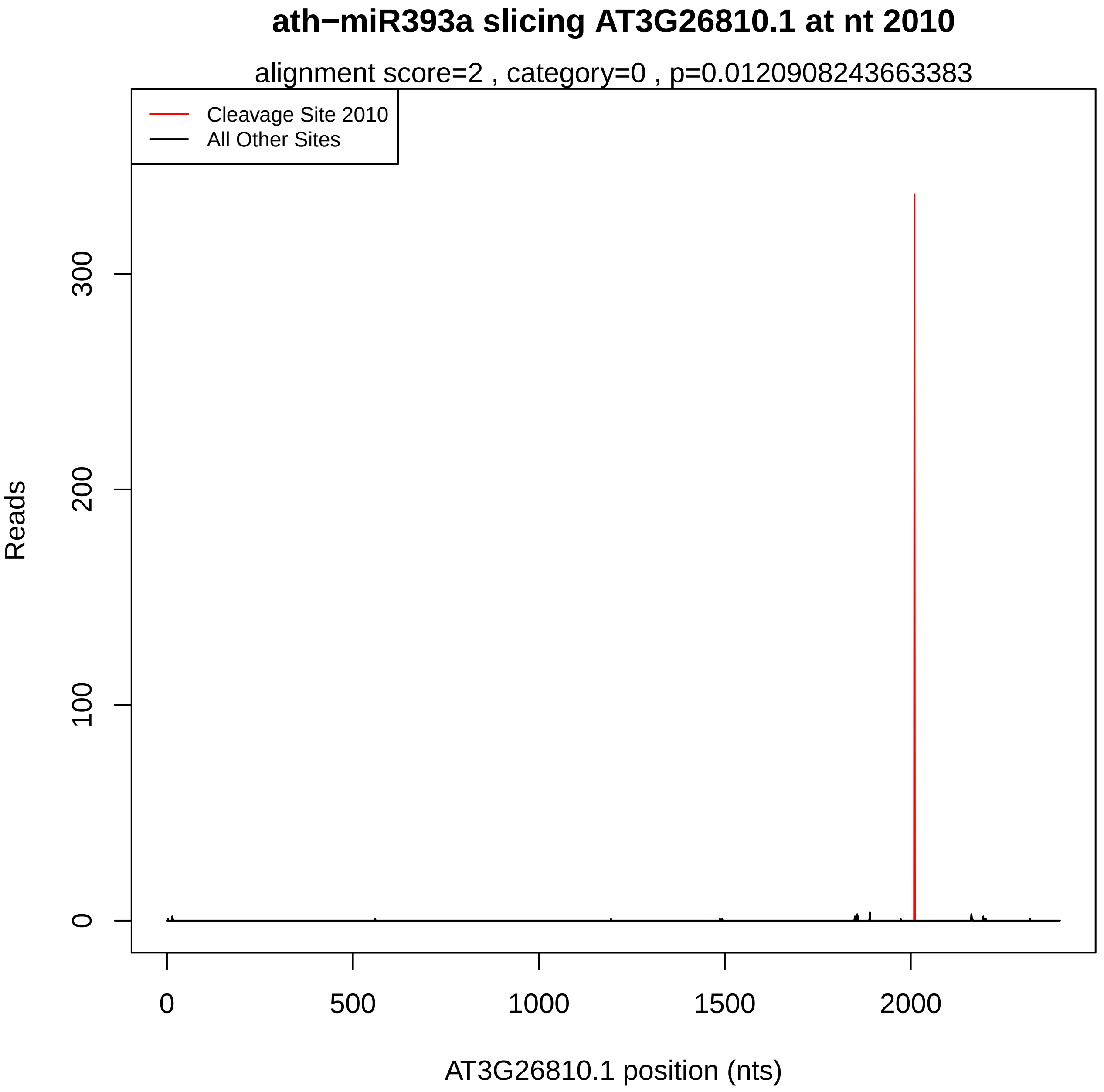
ath−miR393a slicing AT3G26810.1 at nt 2010
alignment score=2 , category=0 , p=0.0120908243663383
Cleavage Site 2010
All Other Sites
Reads
0
100
200
300
0
500
1000
1500
2000
AT3G26810.1 position (nts)

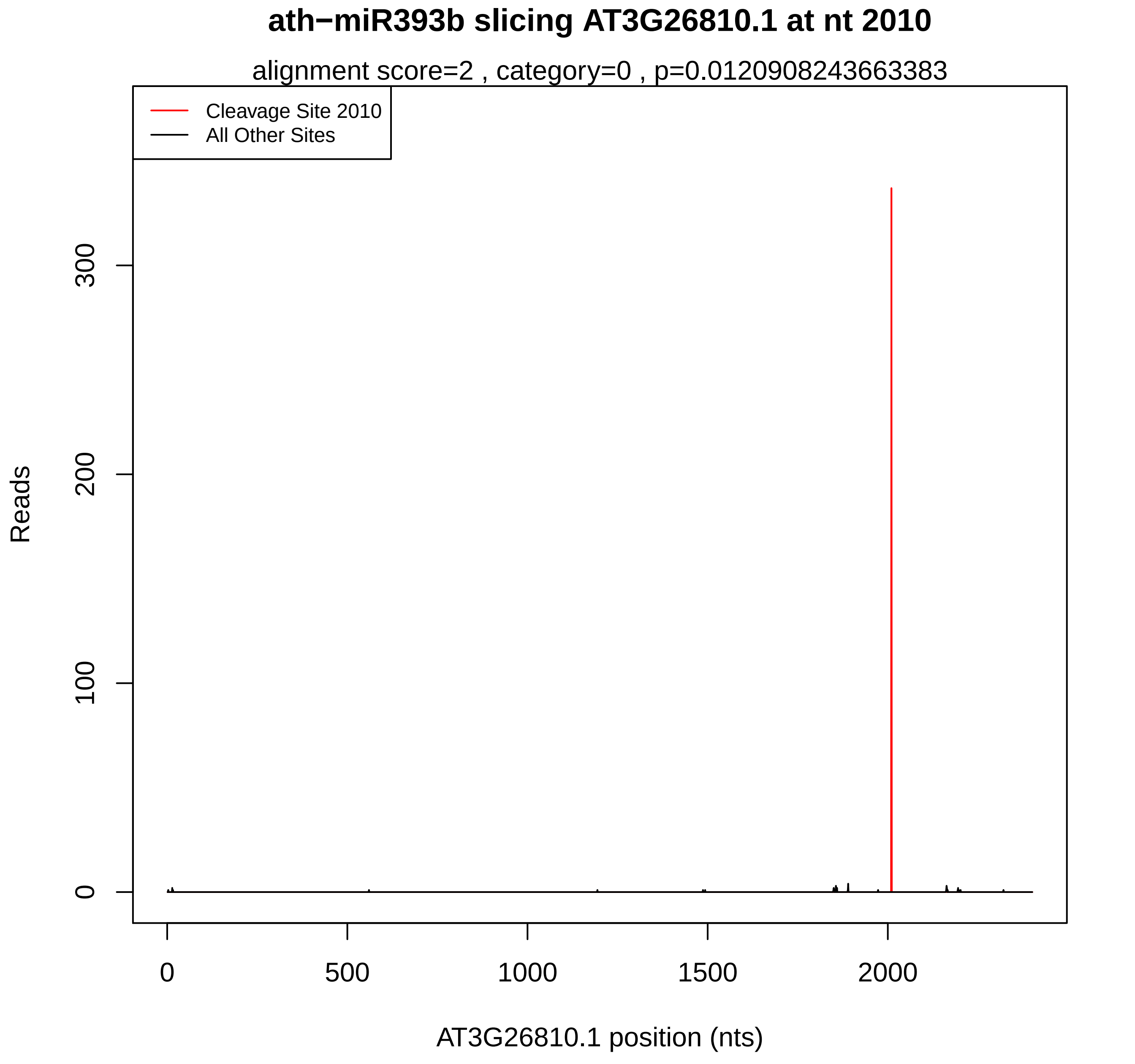
ath−miR393b slicing AT3G26810.1 at nt 2010
alignment score=2 , category=0 , p=0.0120908243663383
Cleavage Site 2010
All Other Sites
Reads
0
100
200
300
0
500
1000
1500
2000
AT3G26810.1 position (nts)

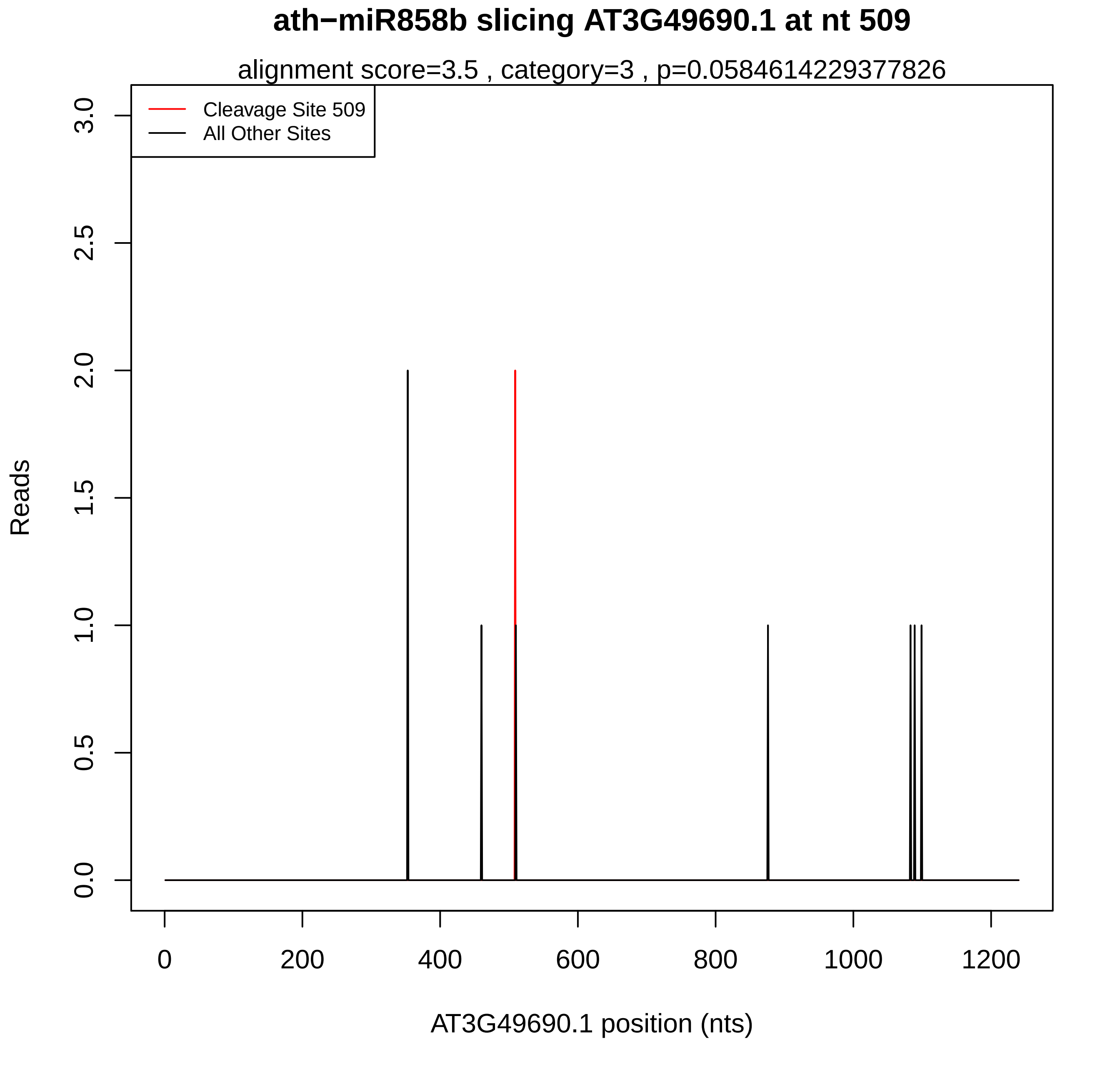
ath−miR858b slicing AT3G49690.1 at nt 509
alignment score=3.5 , category=3 , p=0.0584614229377826
Cleavage Site 509
All Other Sites
Reads
0.0
0.5
1.0
1.5
2.0
2.5
3.0
0
200
400
600
800
1000
1200
AT3G49690.1 position (nts)

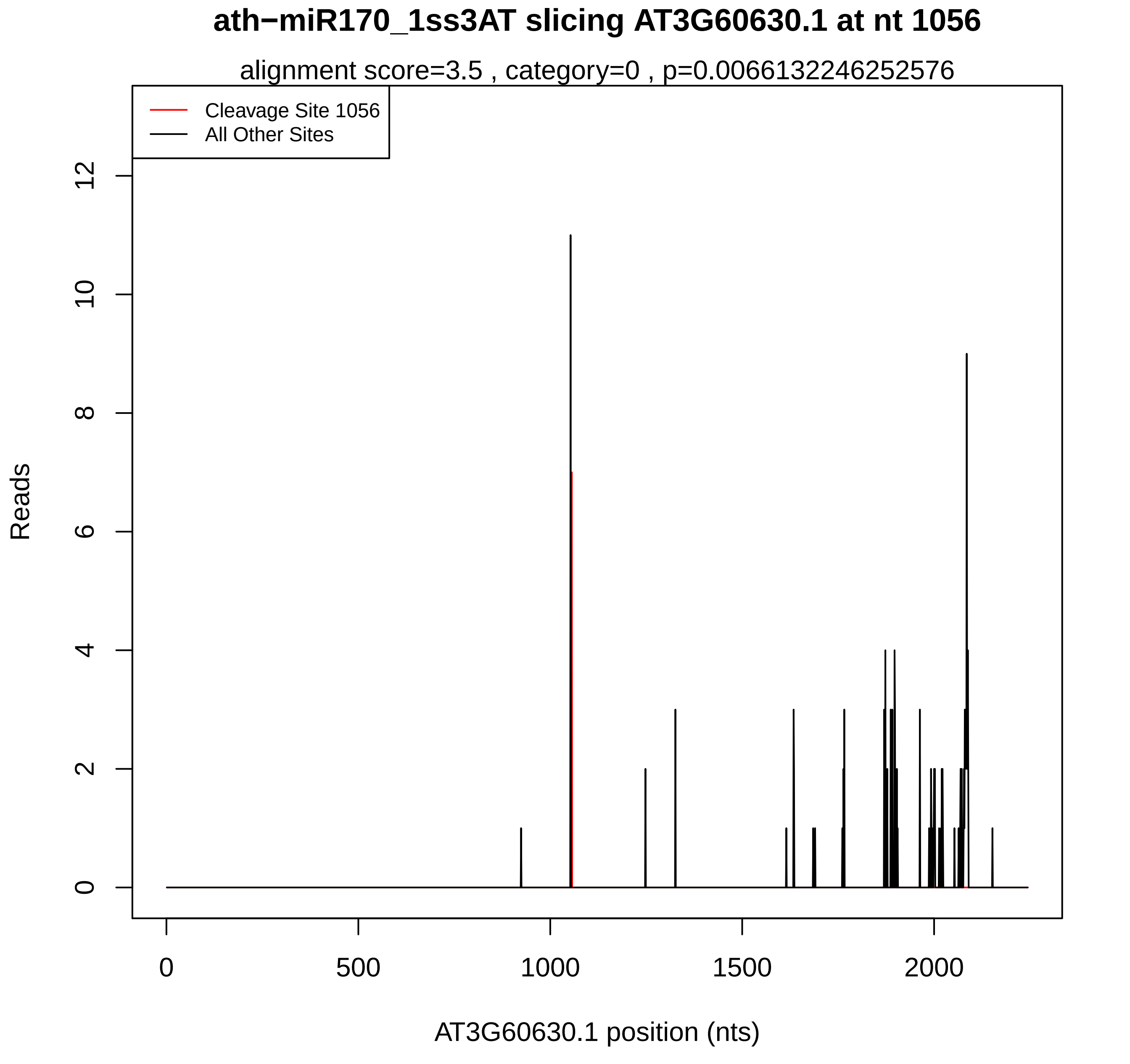

ath−miR170_1ss3AT slicing AT3G60630.1 at nt 1056
alignment score=3.5 , category=0 , p=0.0066132246252576
Cleavage Site 1056
All Other Sites
Reads
0
2
4
6
8
10
12
0
500
1000
1500
2000
AT3G60630.1 position (nts)

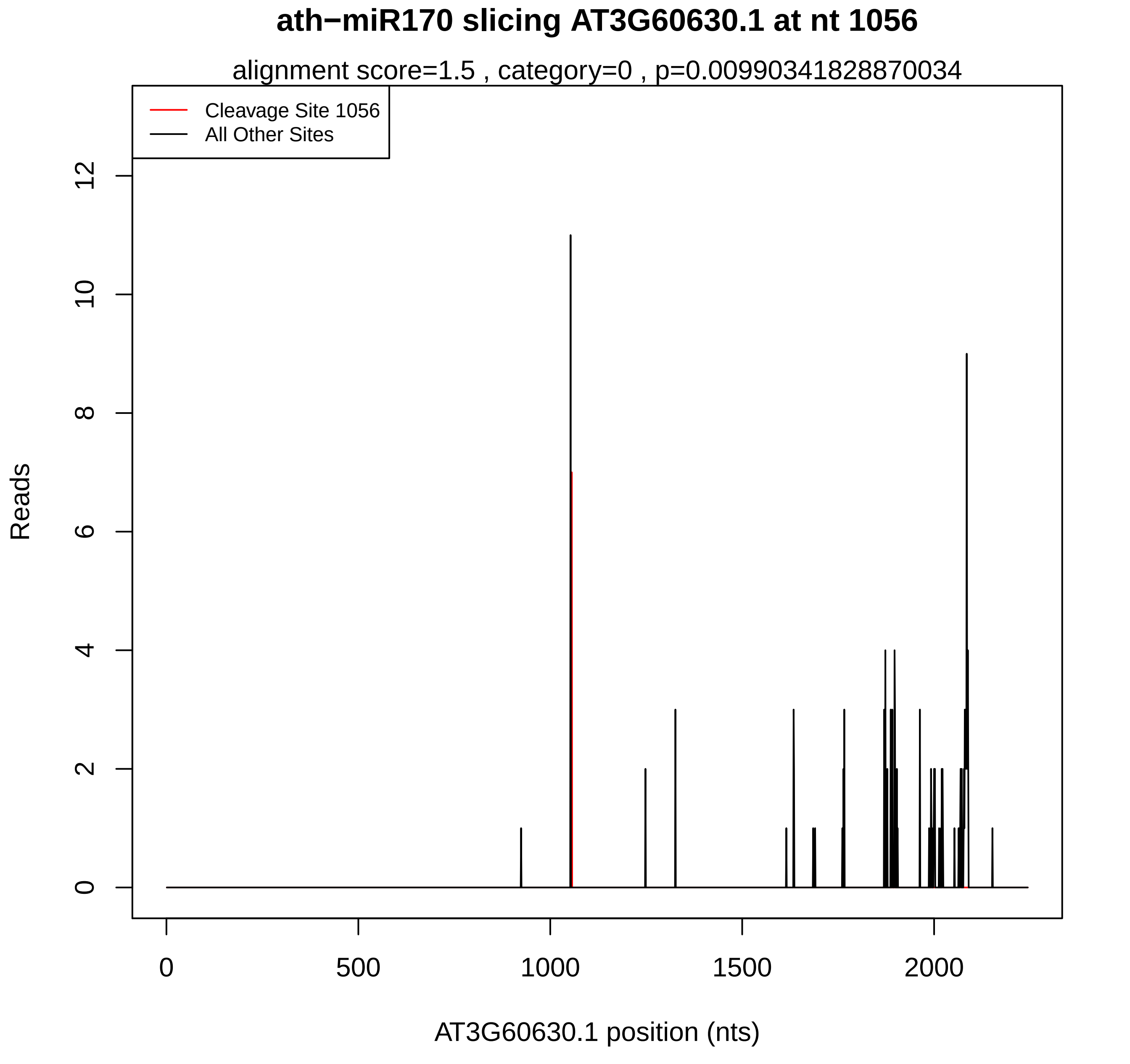
ath−miR170 slicing AT3G60630.1 at nt 1056
alignment score=1.5 , category=0 , p=0.00990341828870034
Cleavage Site 1056
All Other Sites
Reads
0
2
4
6
8
10
12
0
500
1000
1500
2000
AT3G60630.1 position (nts)

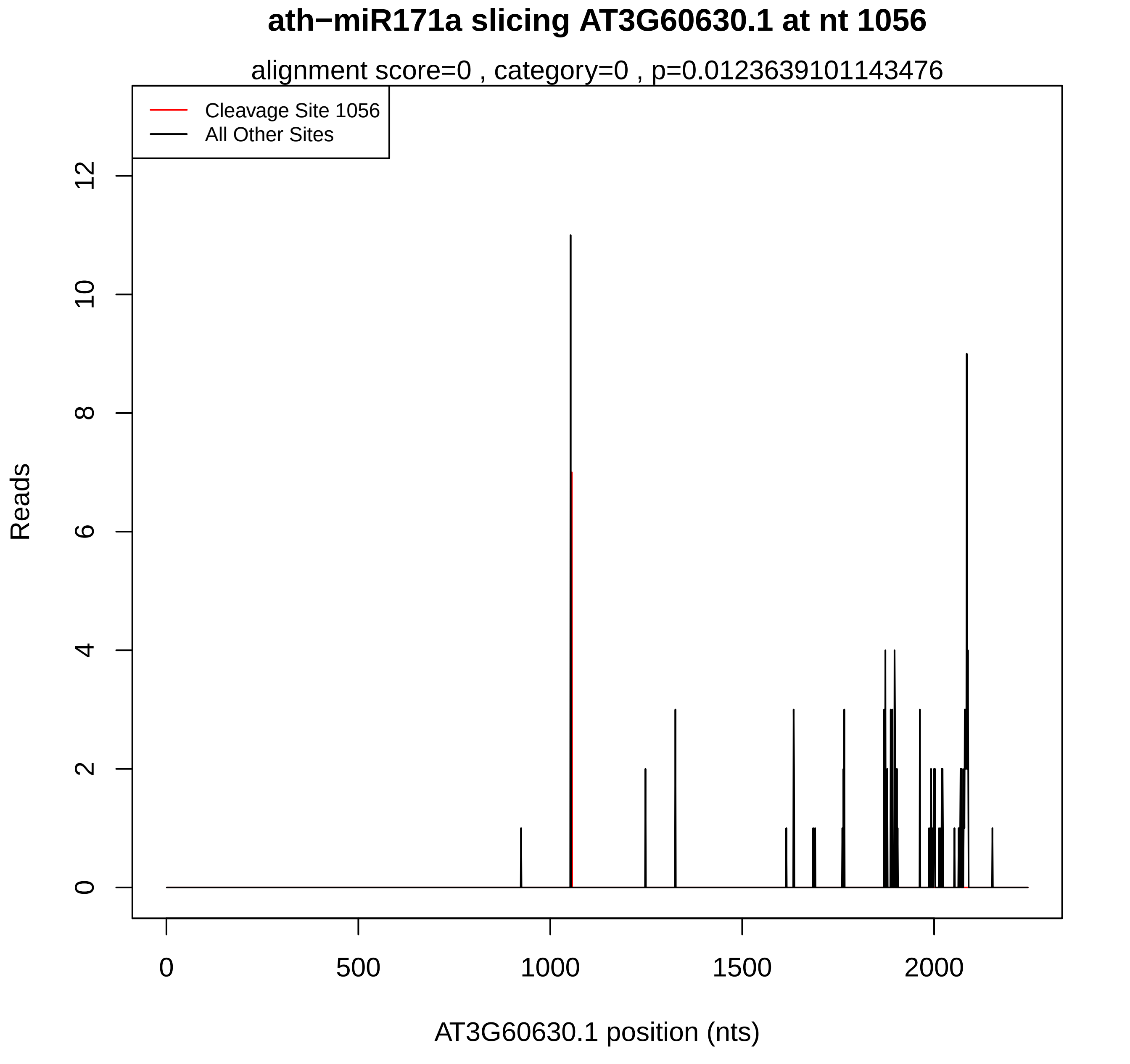

ath−miR171a slicing AT3G60630.1 at nt 1056
alignment score=0 , category=0 , p=0.0123639101143476
Cleavage Site 1056
All Other Sites
Reads
0
2
4
6
8
10
12
0
500
1000
1500
2000
AT3G60630.1 position (nts)

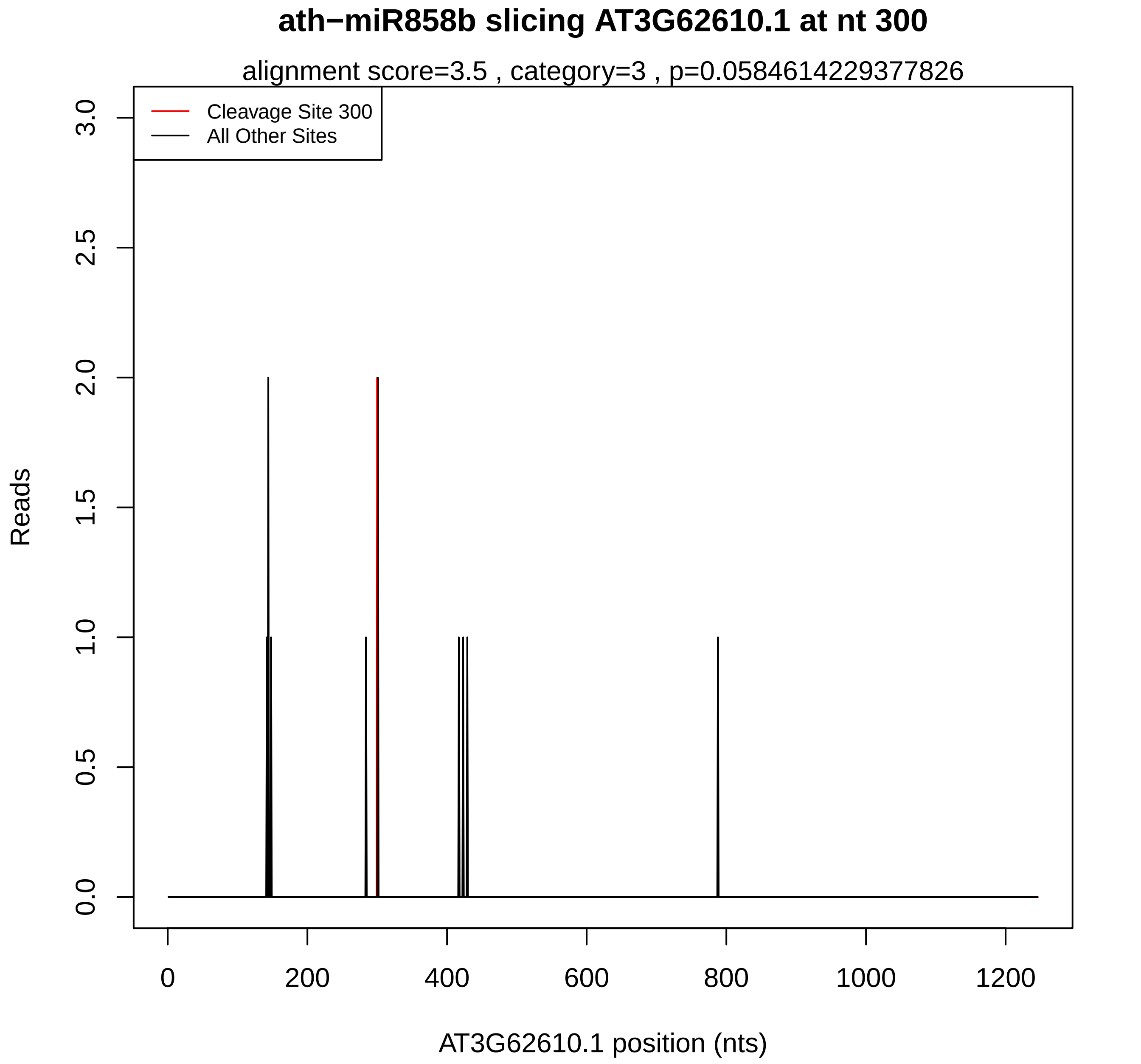
ath−miR858b slicing AT3G62610.1 at nt 300
alignment score=3.5 , category=3 , p=0.0584614229377826
Cleavage Site 300
All Other Sites
Reads
0.0
0.5
1.0
1.5
2.0
2.5
3.0
0
200
400
600
800
1000
1200
AT3G62610.1 position (nts)

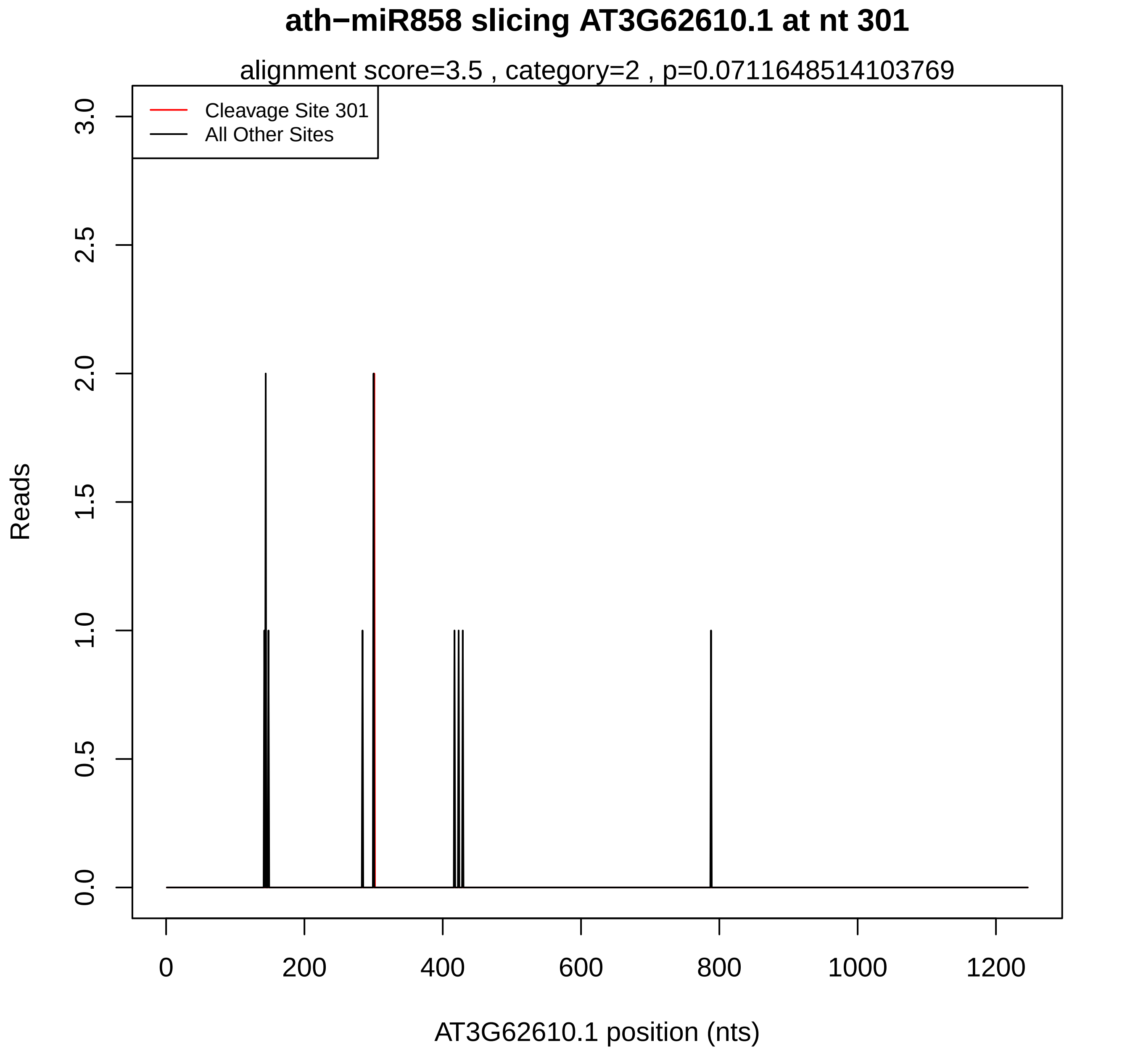

ath−miR858 slicing AT3G62610.1 at nt 301
alignment score=3.5 , category=2 , p=0.0711648514103769
Cleavage Site 301
All Other Sites
Reads
0.0
0.5
1.0
1.5
2.0
2.5
3.0
0
200
400
600
800
1000
1200
AT3G62610.1 position (nts)

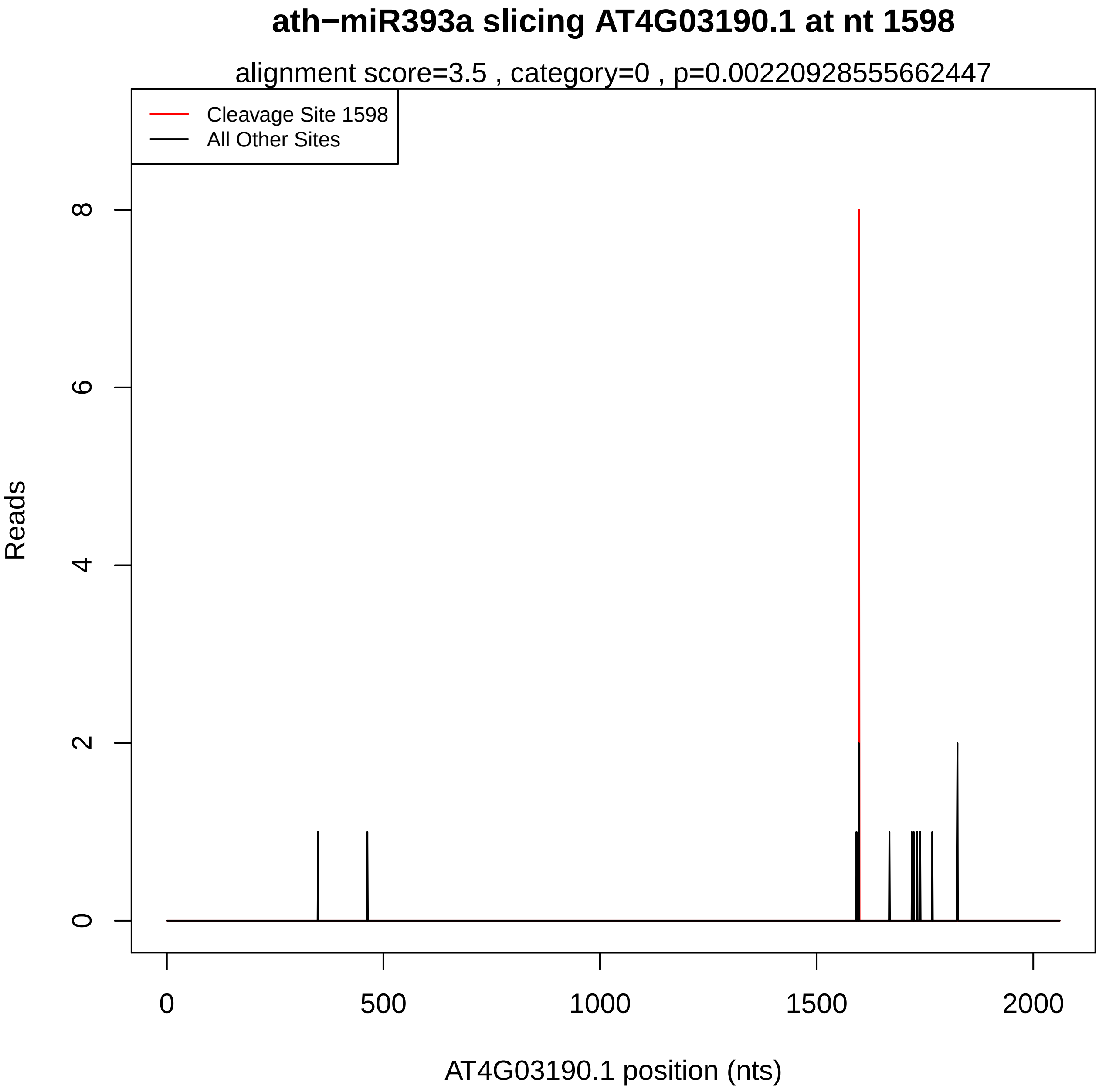

ath−miR393a slicing AT4G03190.1 at nt 1598
alignment score=3.5 , category=0 , p=0.00220928555662447
Cleavage Site 1598
All Other Sites
Reads
0
2
4
6
8
0
500
1000
1500
2000
AT4G03190.1 position (nts)

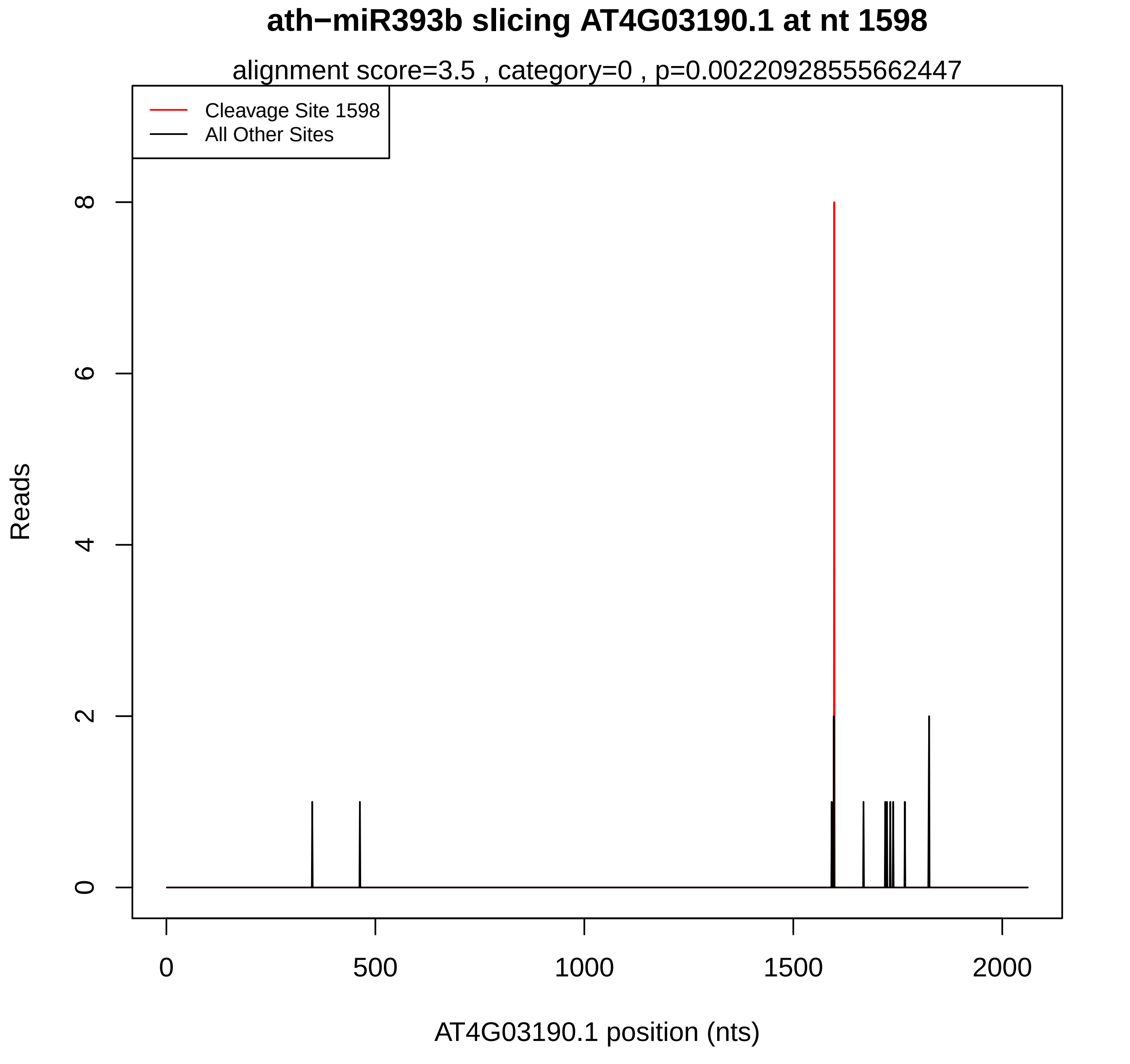
ath−miR393b slicing AT4G03190.1 at nt 1598
alignment score=3.5 , category=0 , p=0.00220928555662447
Cleavage Site 1598
All Other Sites
Reads
0
2
4
6
8
0
500
1000
1500
2000
AT4G03190.1 position (nts)

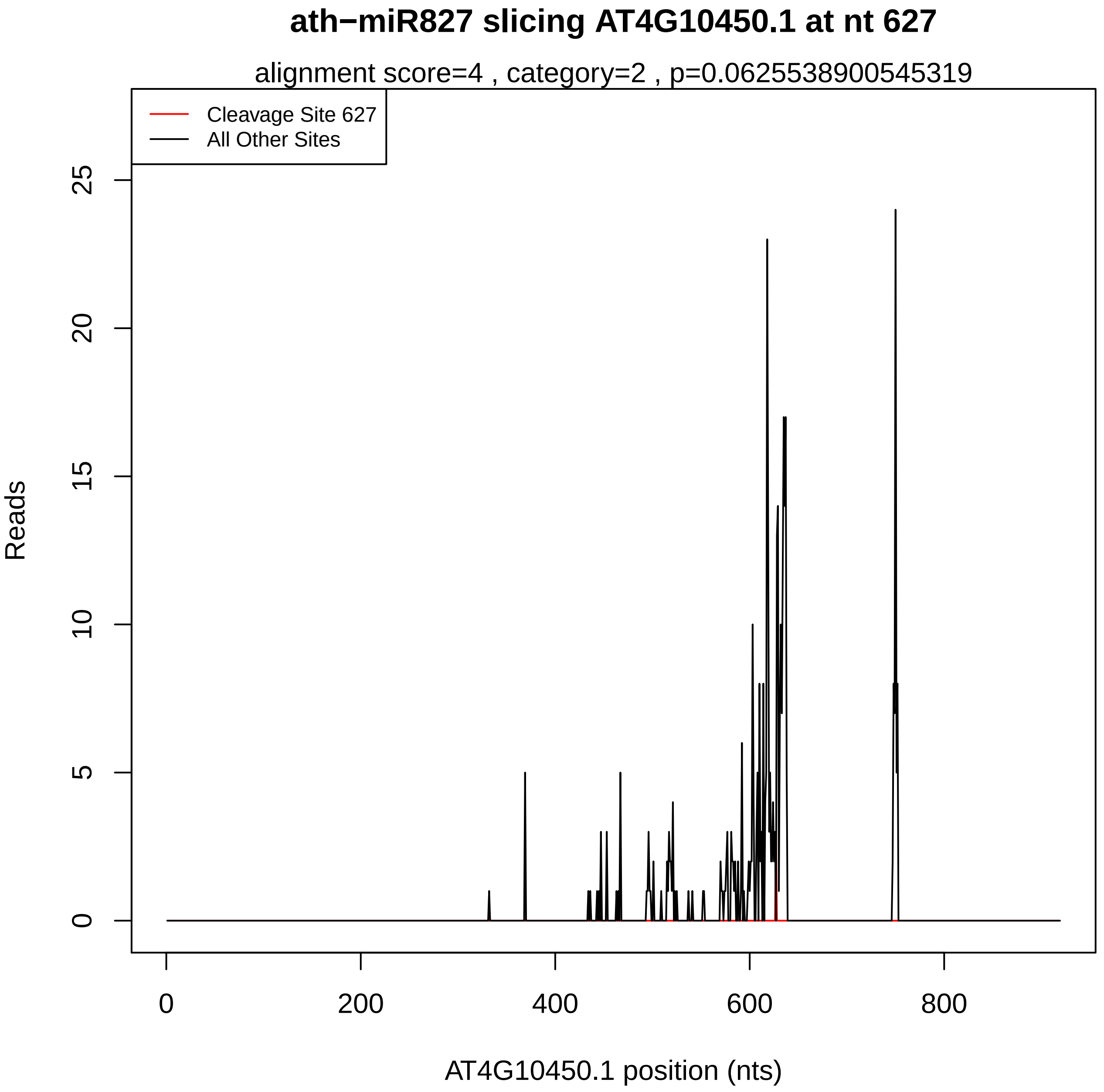
ath−miR827 slicing AT4G10450.1 at nt 627
alignment score=4 , category=2 , p=0.0625538900545319
Cleavage Site 627
All Other Sites
Reads
0
5
10
15
20
25
0
200
400
600
800
AT4G10450.1 position (nts)

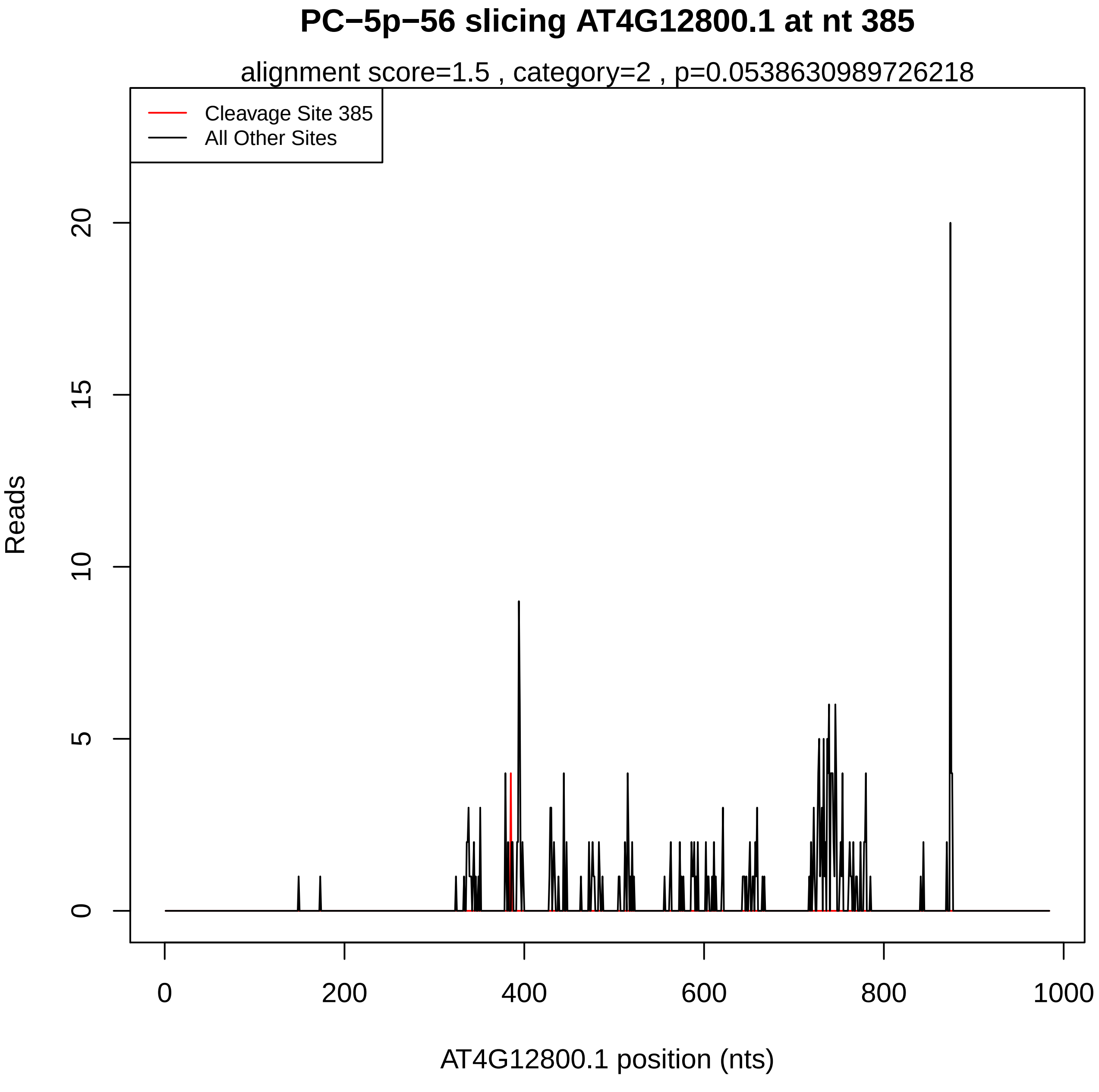
PC−5p−56 slicing AT4G12800.1 at nt 385
alignment score=1.5 , category=2 , p=0.0538630989726218
Cleavage Site 385
All Other Sites
Reads
0
5
10
15
20
0
200
400
600
800
1000
AT4G12800.1 position (nts)

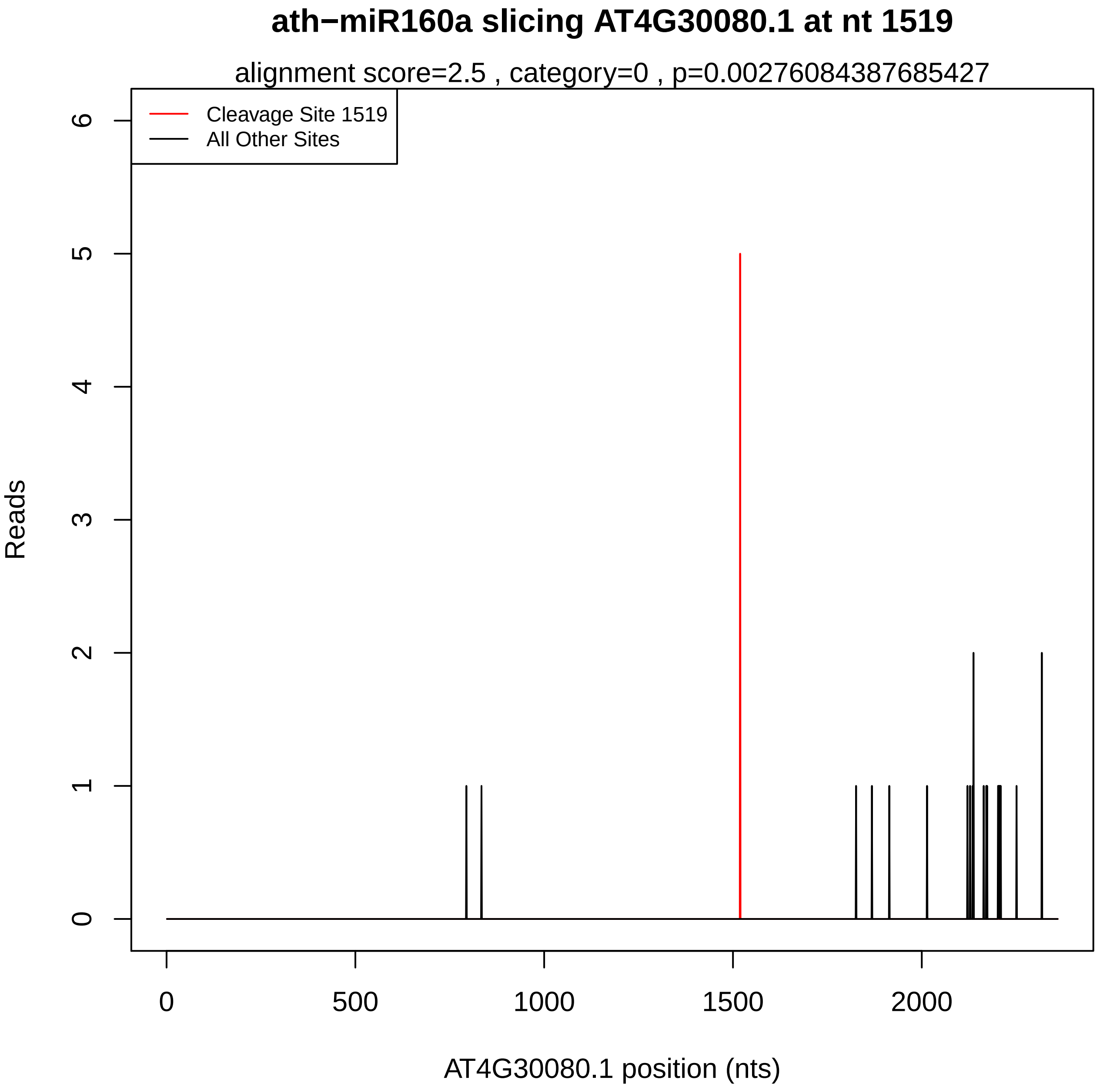
ath−miR160a slicing AT4G30080.1 at nt 1519
alignment score=2.5 , category=0 , p=0.00276084387685427
Cleavage Site 1519
All Other Sites
Reads
0
1
2
3
4
5
6
0
500
1000
1500
2000
AT4G30080.1 position (nts)

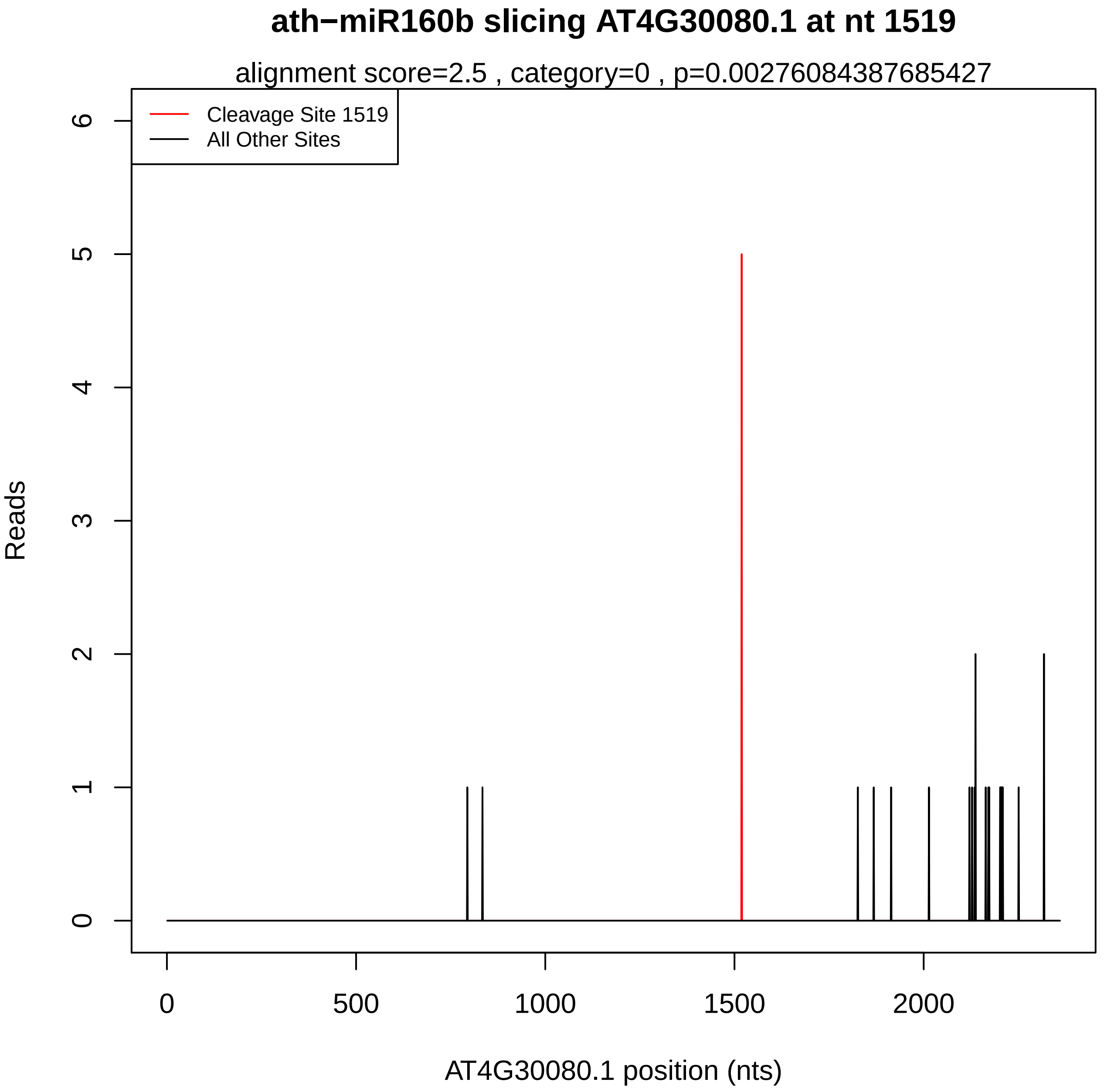
ath−miR160b slicing AT4G30080.1 at nt 1519
alignment score=2.5 , category=0 , p=0.00276084387685427
Cleavage Site 1519
All Other Sites
Reads
0
1
2
3
4
5
6
0
500
1000
1500
2000
AT4G30080.1 position (nts)

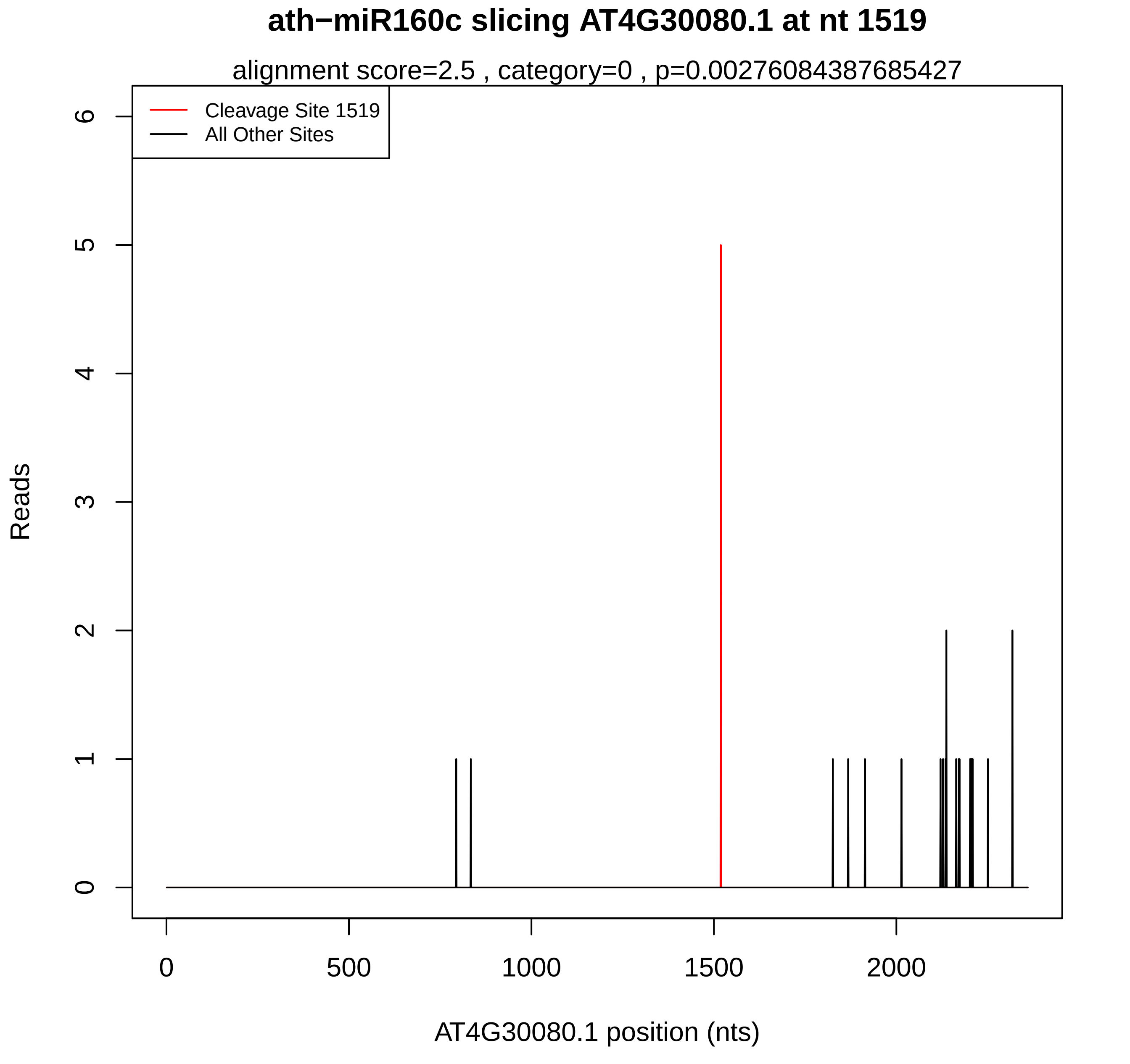

ath−miR160c slicing AT4G30080.1 at nt 1519
alignment score=2.5 , category=0 , p=0.00276084387685427
Cleavage Site 1519
All Other Sites
Reads
0
1
2
3
4
5
6
0
500
1000
1500
2000
AT4G30080.1 position (nts)

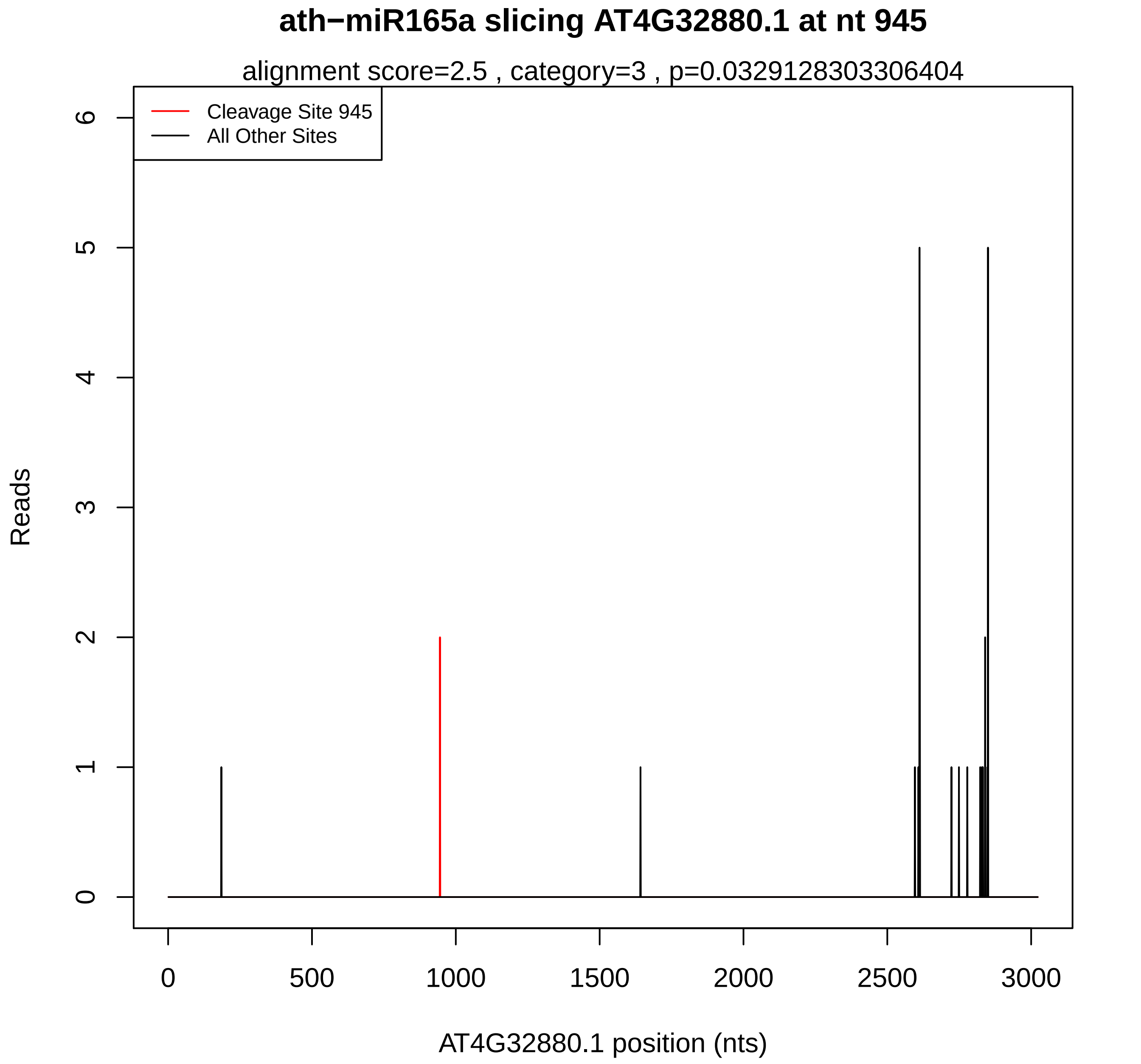

ath−miR165a slicing AT4G32880.1 at nt 945
alignment score=2.5 , category=3 , p=0.0329128303306404
Cleavage Site 945
All Other Sites
Reads
0
1
2
3
4
5
6
0
500
1000
1500
2000
2500
3000
AT4G32880.1 position (nts)

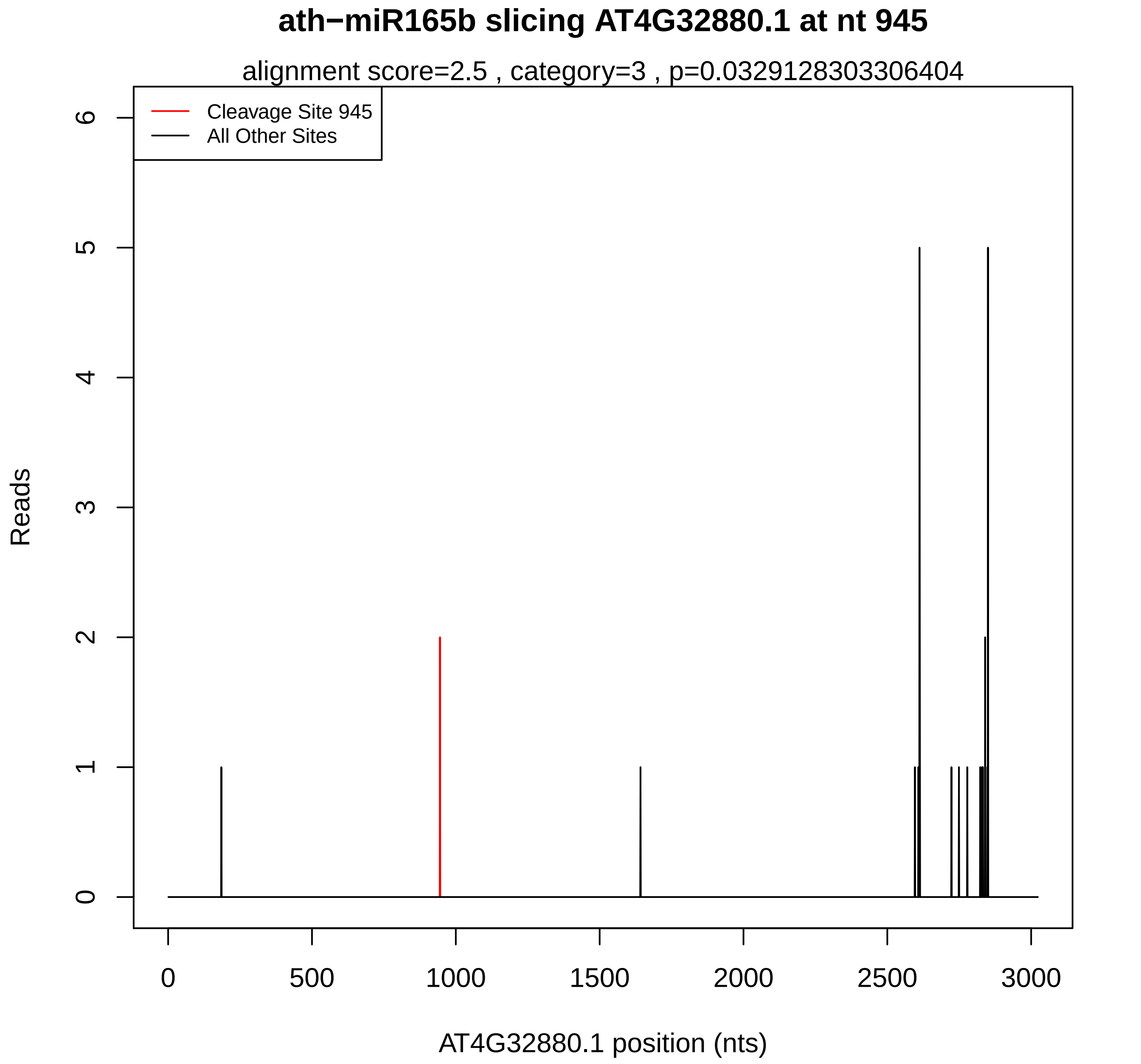
ath−miR165b slicing AT4G32880.1 at nt 945
alignment score=2.5 , category=3 , p=0.0329128303306404
Cleavage Site 945
All Other Sites
Reads
0
1
2
3
4
5
6
0
500
1000
1500
2000
2500
3000
AT4G32880.1 position (nts)

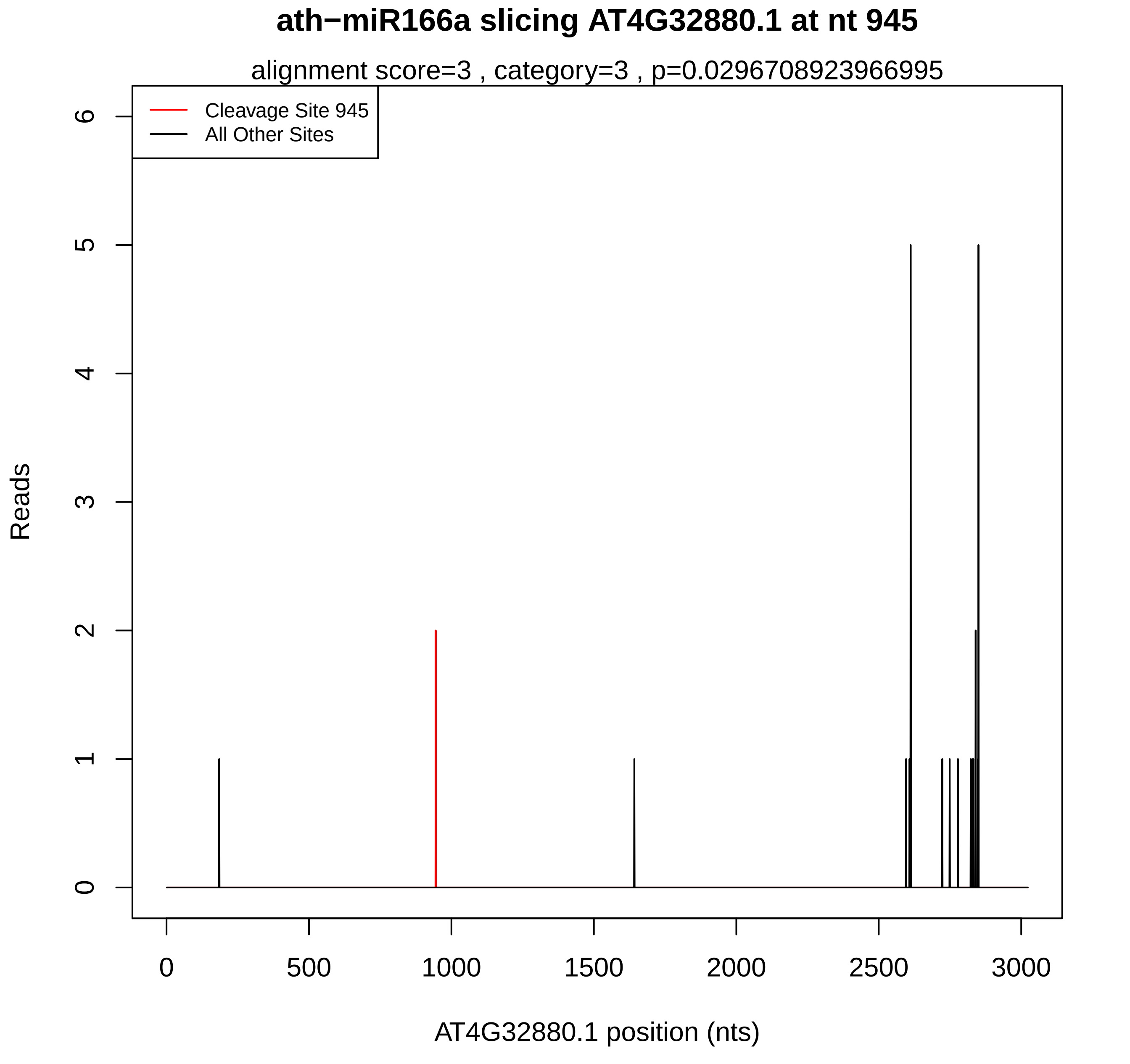

ath−miR166a slicing AT4G32880.1 at nt 945
alignment score=3 , category=3 , p=0.0296708923966995
Cleavage Site 945
All Other Sites
Reads
0
1
2
3
4
5
6
0
500
1000
1500
2000
2500
3000
AT4G32880.1 position (nts)

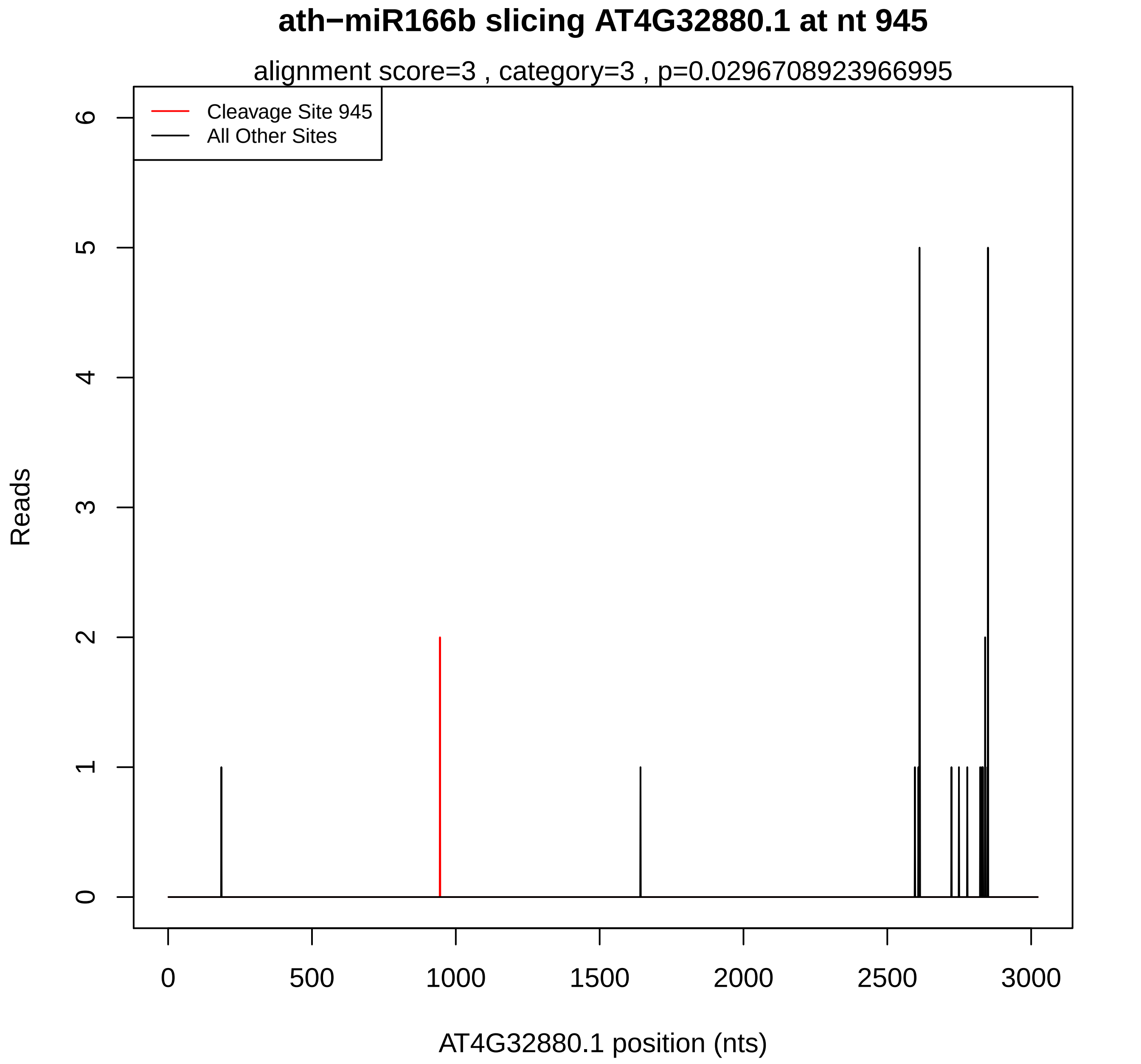
ath−miR166b slicing AT4G32880.1 at nt 945
alignment score=3 , category=3 , p=0.0296708923966995
Cleavage Site 945
All Other Sites
Reads
0
1
2
3
4
5
6
0
500
1000
1500
2000
2500
3000
AT4G32880.1 position (nts)

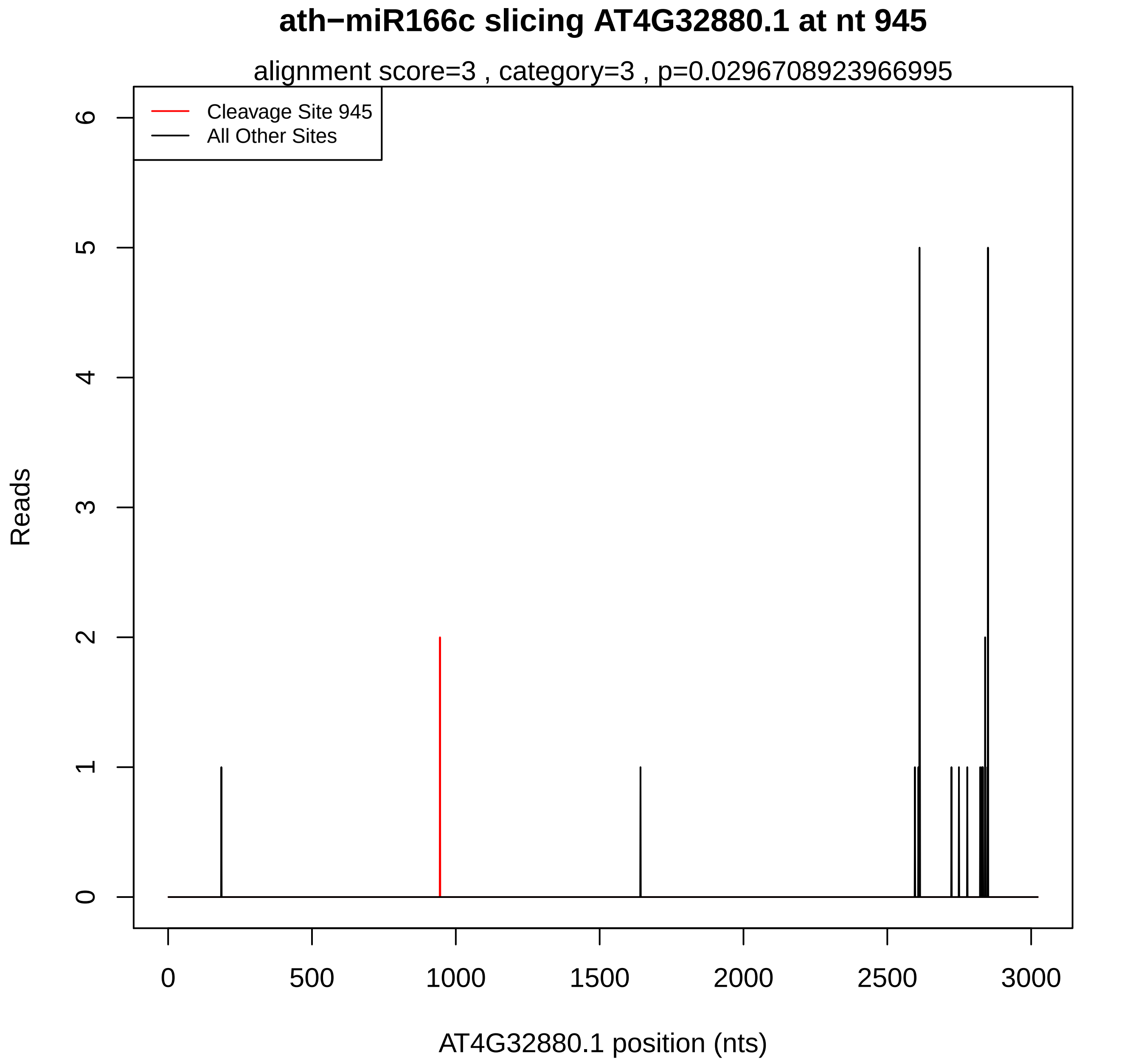
ath−miR166c slicing AT4G32880.1 at nt 945
alignment score=3 , category=3 , p=0.0296708923966995
Cleavage Site 945
All Other Sites
Reads
0
1
2
3
4
5
6
0
500
1000
1500
2000
2500
3000
AT4G32880.1 position (nts)

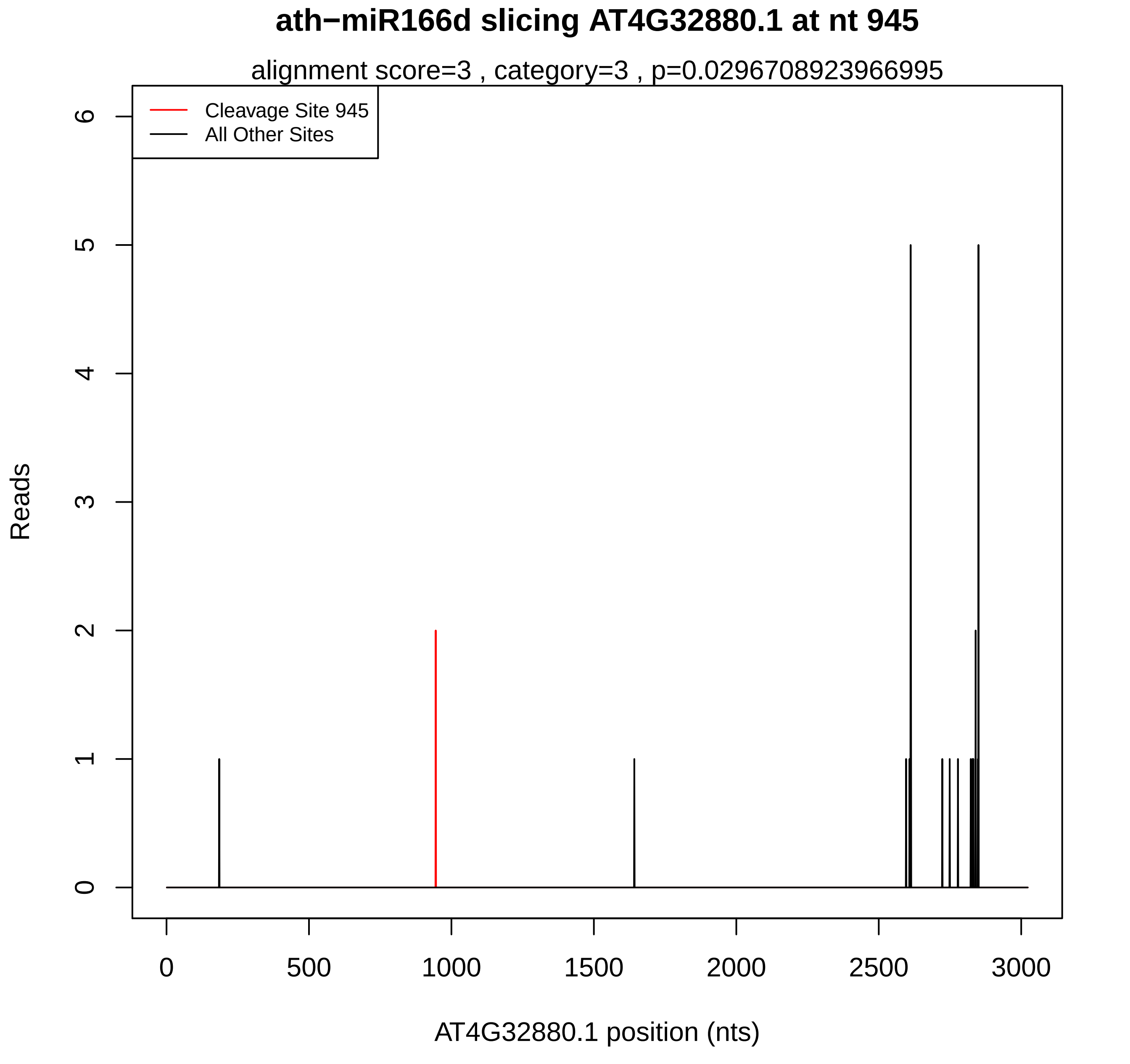

ath−miR166d slicing AT4G32880.1 at nt 945
alignment score=3 , category=3 , p=0.0296708923966995
Cleavage Site 945
All Other Sites
Reads
0
1
2
3
4
5
6
0
500
1000
1500
2000
2500
3000
AT4G32880.1 position (nts)

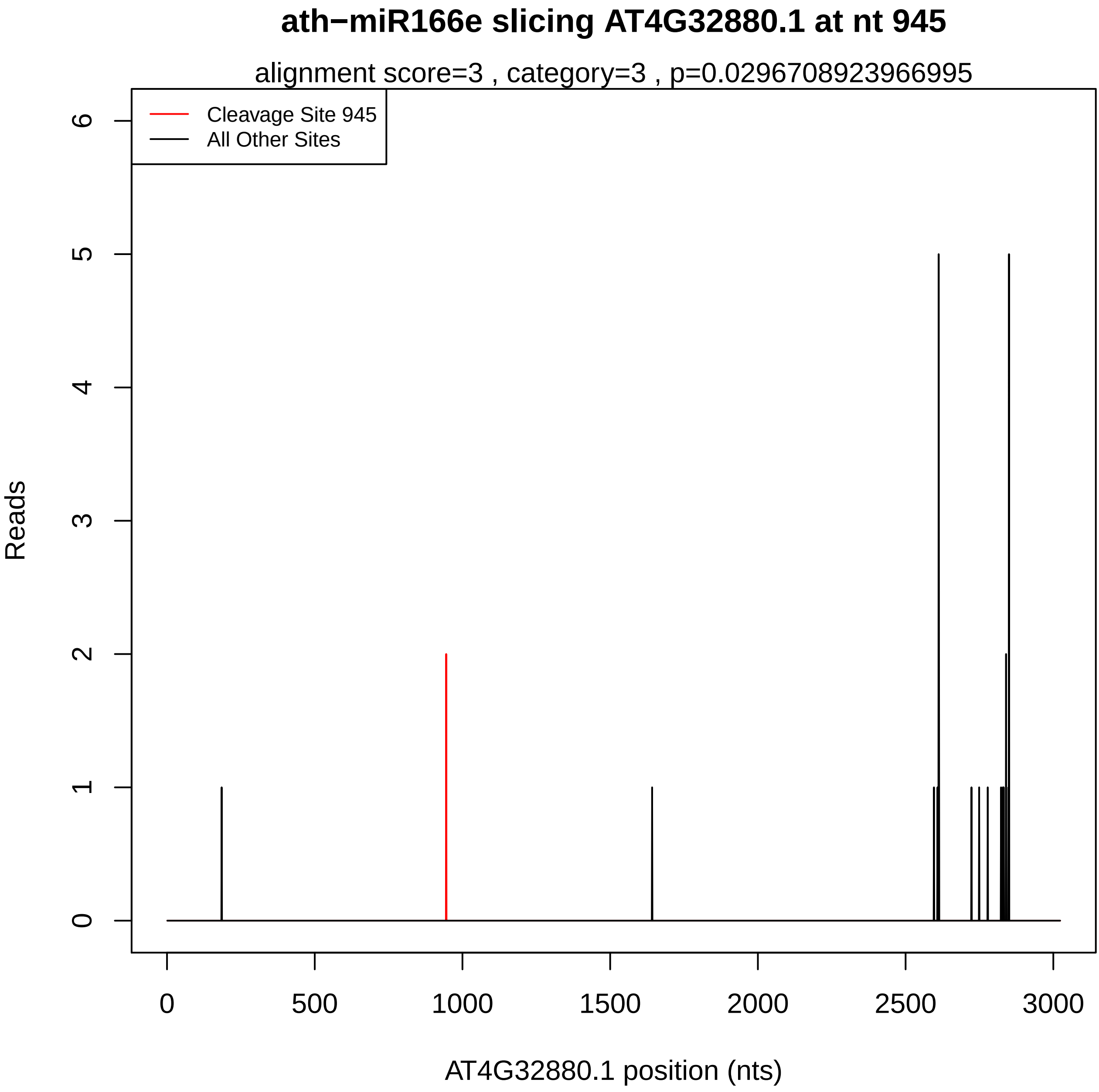
ath−miR166e slicing AT4G32880.1 at nt 945
alignment score=3 , category=3 , p=0.0296708923966995
Cleavage Site 945
All Other Sites
Reads
0
1
2
3
4
5
6
0
500
1000
1500
2000
2500
3000
AT4G32880.1 position (nts)

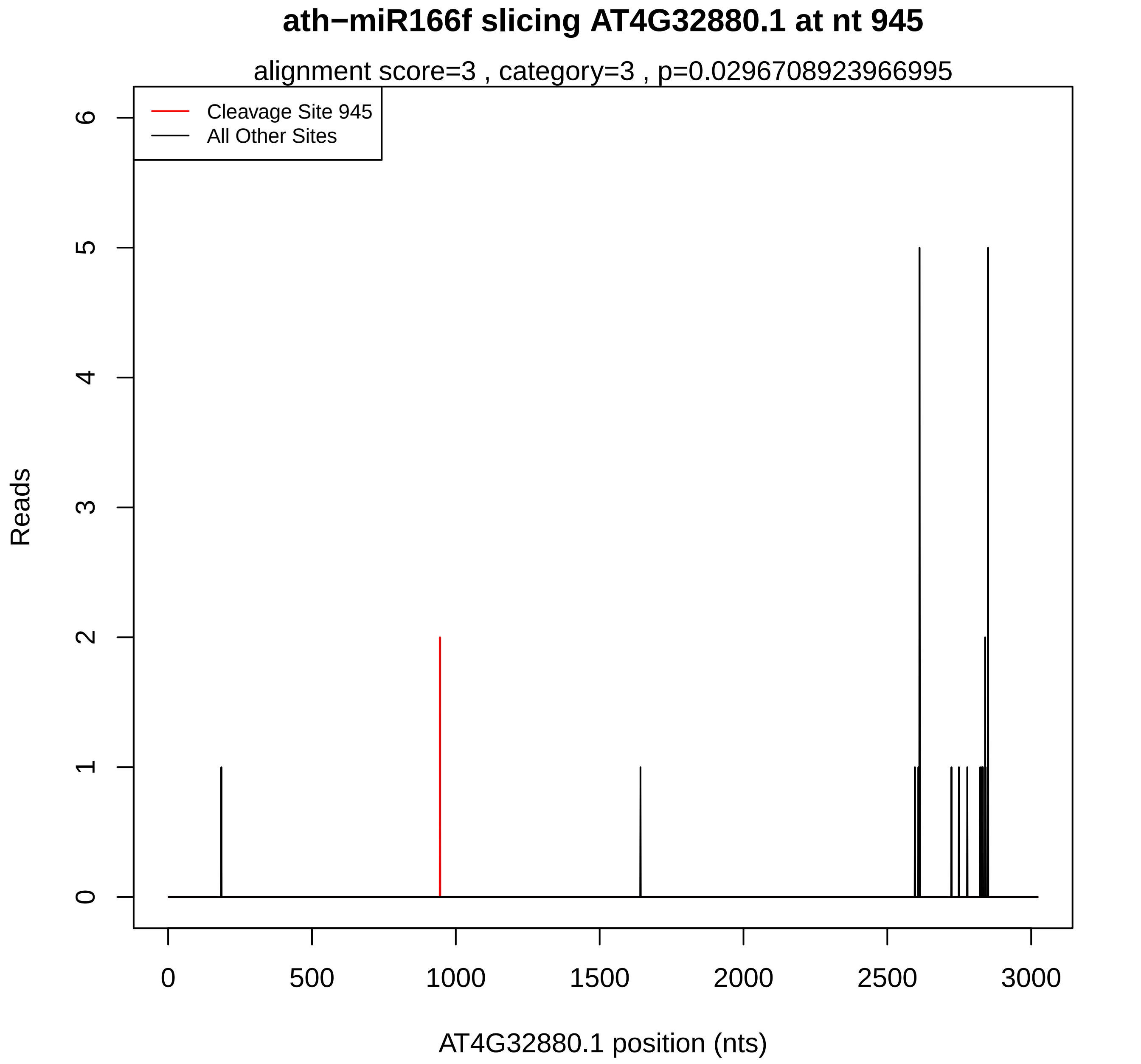
ath−miR166f slicing AT4G32880.1 at nt 945
alignment score=3 , category=3 , p=0.0296708923966995
Cleavage Site 945
All Other Sites
Reads
0
1
2
3
4
5
6
0
500
1000
1500
2000
2500
3000
AT4G32880.1 position (nts)

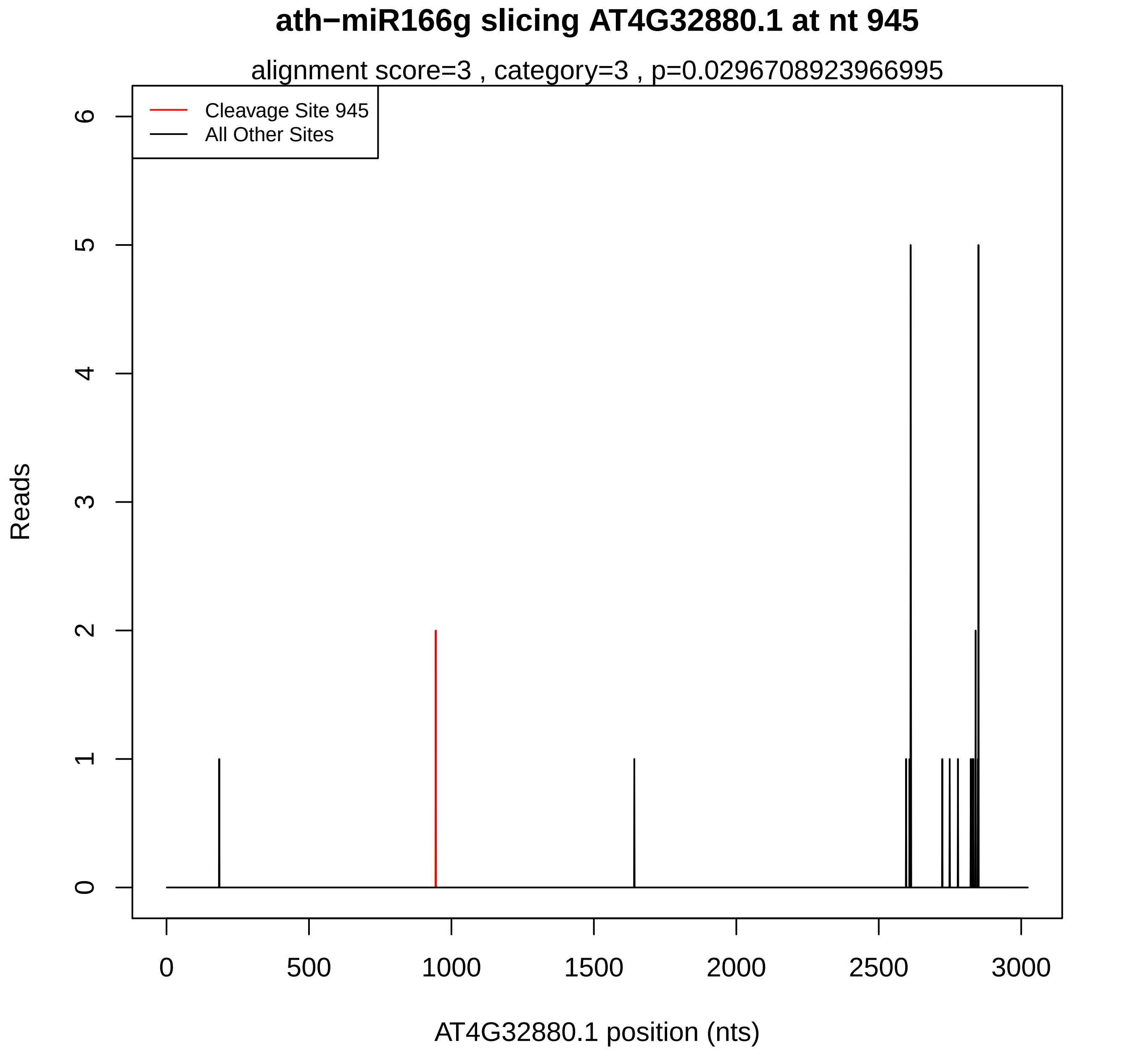
ath−miR166g slicing AT4G32880.1 at nt 945
alignment score=3 , category=3 , p=0.0296708923966995
Cleavage Site 945
All Other Sites
Reads
0
1
2
3
4
5
6
0
500
1000
1500
2000
2500
3000
AT4G32880.1 position (nts)

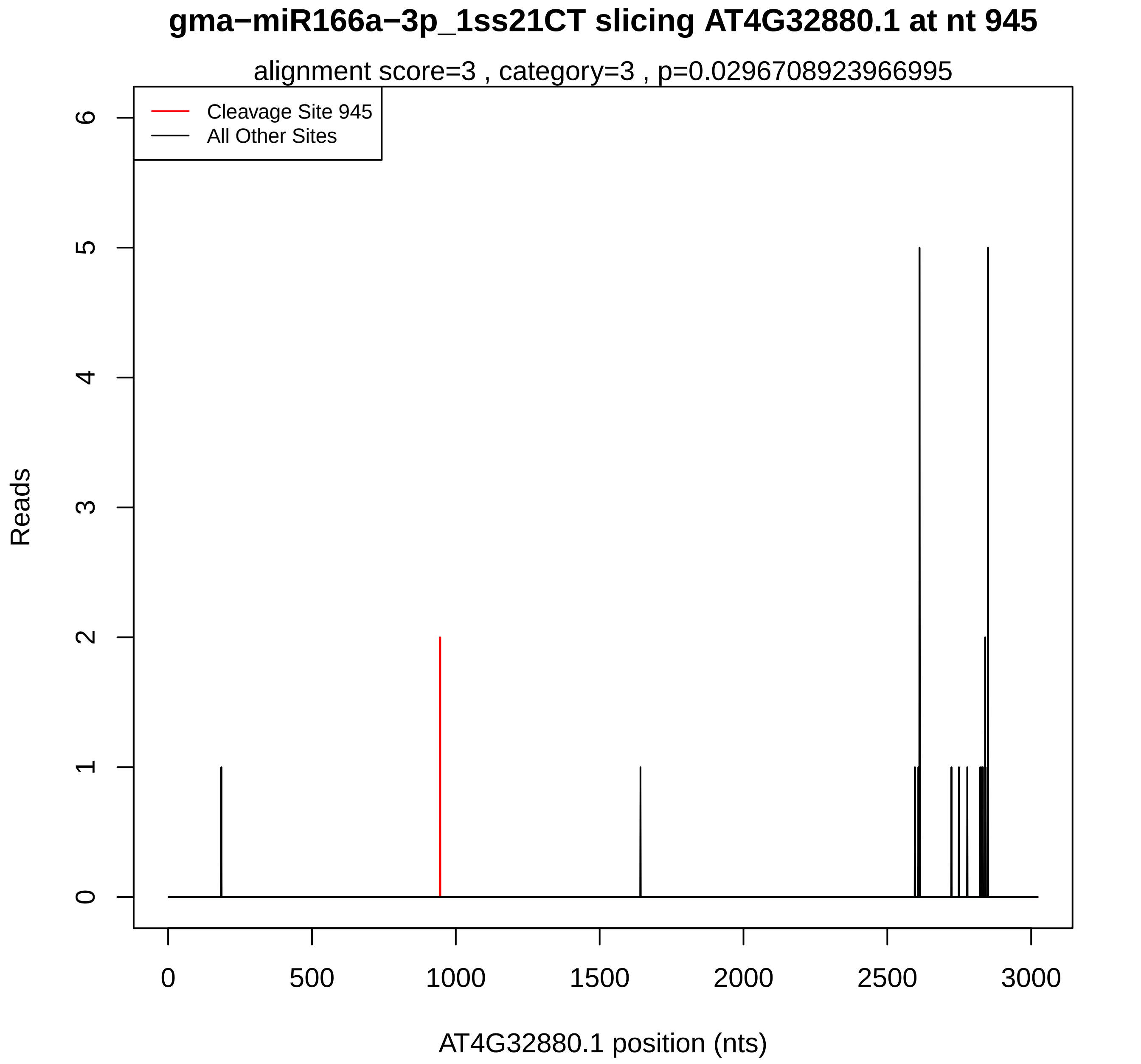
gma−miR166a−3p_1ss21CT slicing AT4G32880.1 at nt 945
alignment score=3 , category=3 , p=0.0296708923966995
Cleavage Site 945
All Other Sites
Reads
0
1
2
3
4
5
6
0
500
1000
1500
2000
2500
3000
AT4G32880.1 position (nts)

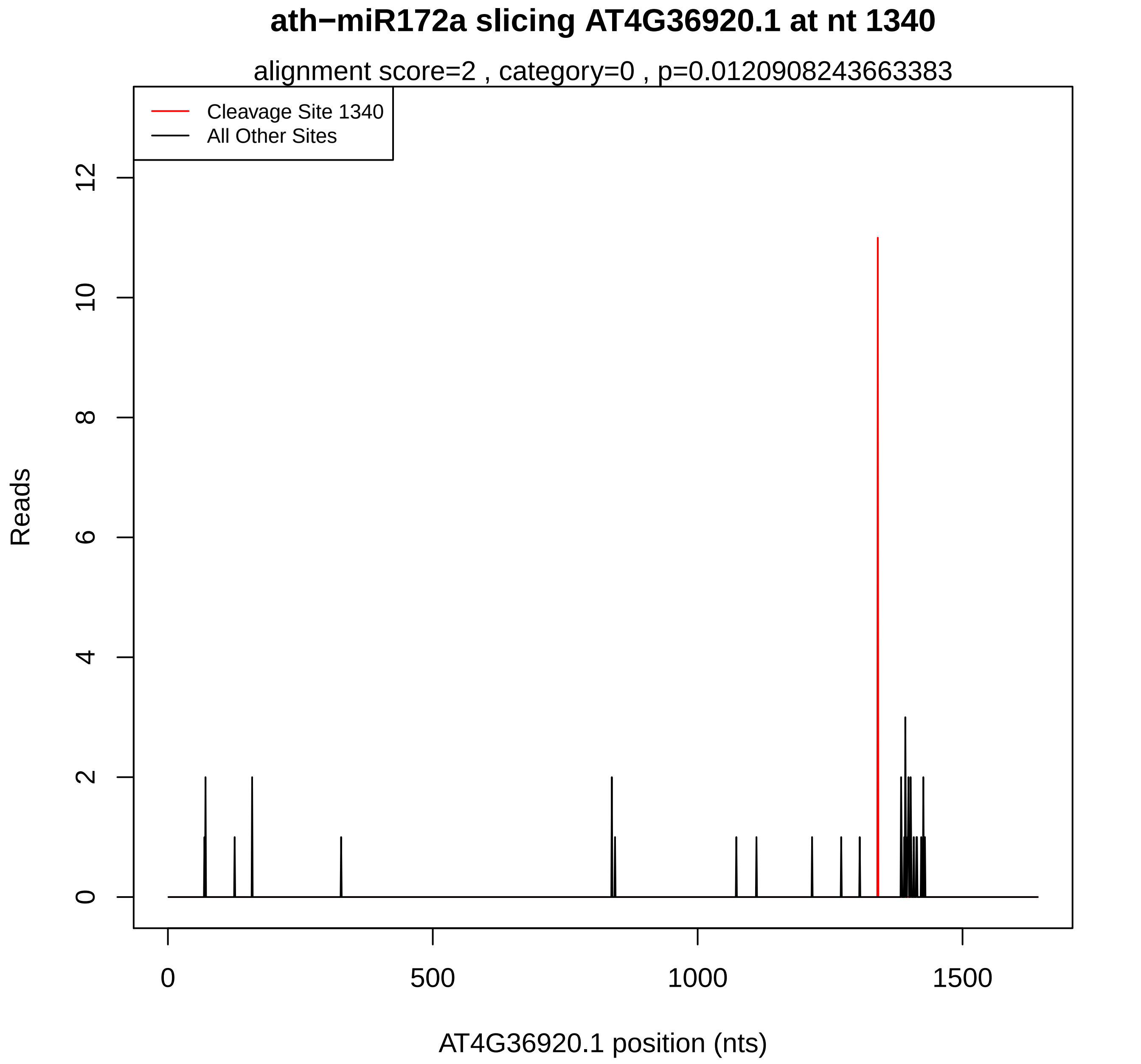

ath−miR172a slicing AT4G36920.1 at nt 1340
alignment score=2 , category=0 , p=0.0120908243663383
Cleavage Site 1340
All Other Sites
Reads
0
2
4
6
8
10
12
0
500
1000
1500
AT4G36920.1 position (nts)

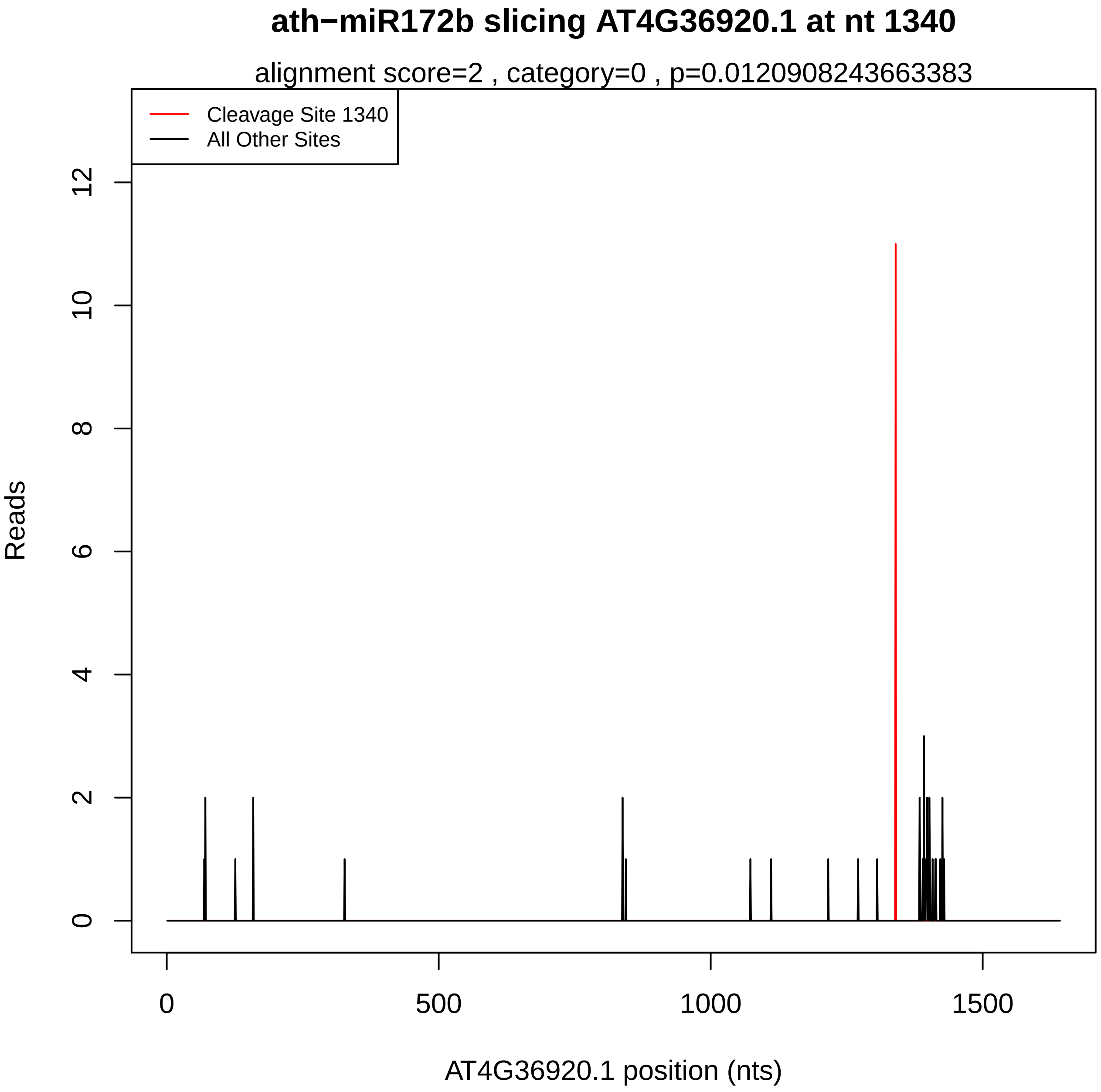

ath−miR172b slicing AT4G36920.1 at nt 1340
alignment score=2 , category=0 , p=0.0120908243663383
Cleavage Site 1340
All Other Sites
Reads
0
2
4
6
8
10
12
0
500
1000
1500
AT4G36920.1 position (nts)

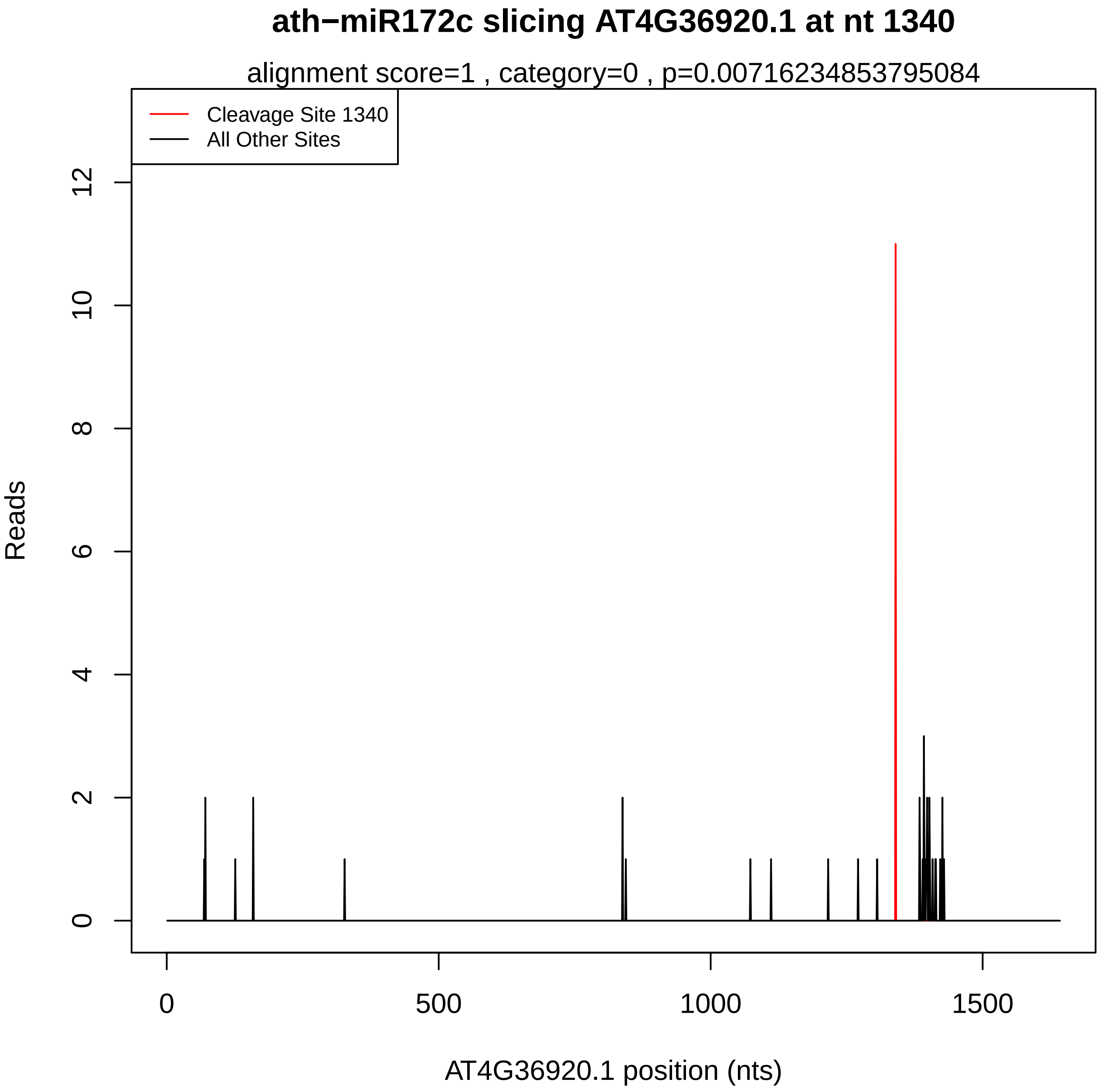

ath−miR172c slicing AT4G36920.1 at nt 1340
alignment score=1 , category=0 , p=0.00716234853795084
Cleavage Site 1340
All Other Sites
Reads
0
2
4
6
8
10
12
0
500
1000
1500
AT4G36920.1 position (nts)

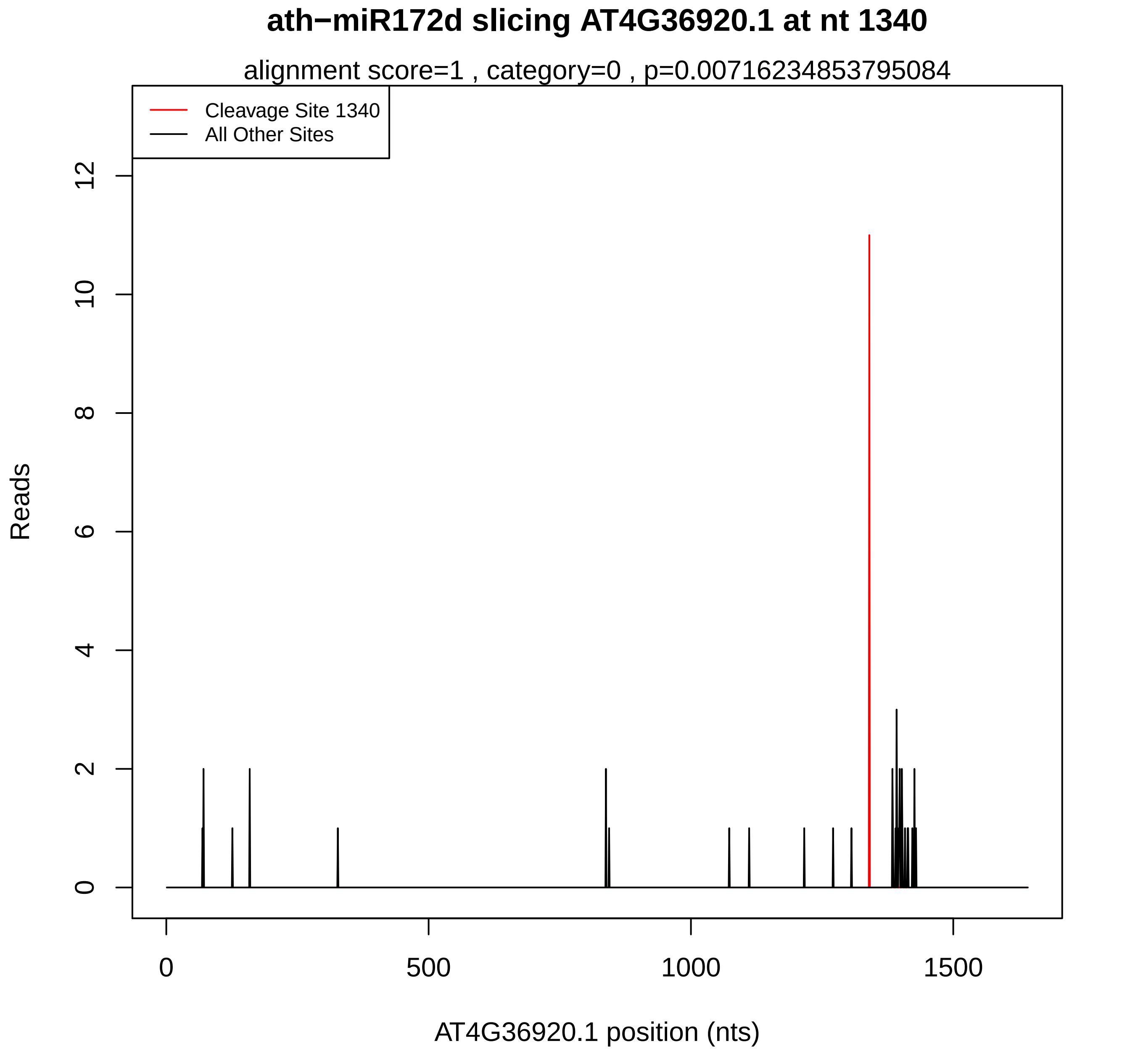

ath−miR172d slicing AT4G36920.1 at nt 1340
alignment score=1 , category=0 , p=0.00716234853795084
Cleavage Site 1340
All Other Sites
Reads
0
2
4
6
8
10
12
0
500
1000
1500
AT4G36920.1 position (nts)

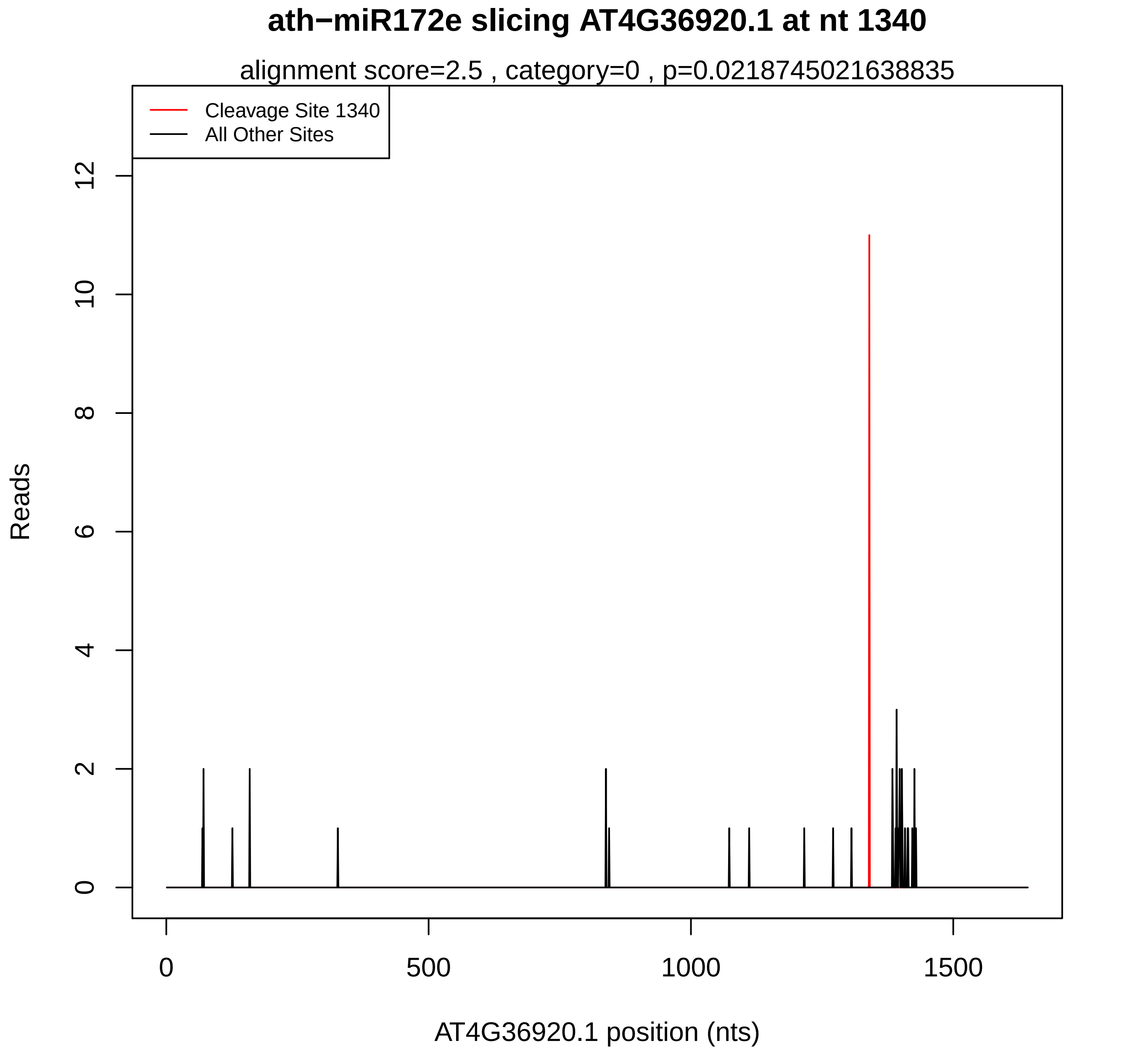
ath−miR172e slicing AT4G36920.1 at nt 1340
alignment score=2.5 , category=0 , p=0.0218745021638835
Cleavage Site 1340
All Other Sites
Reads
0
2
4
6
8
10
12
0
500
1000
1500
AT4G36920.1 position (nts)

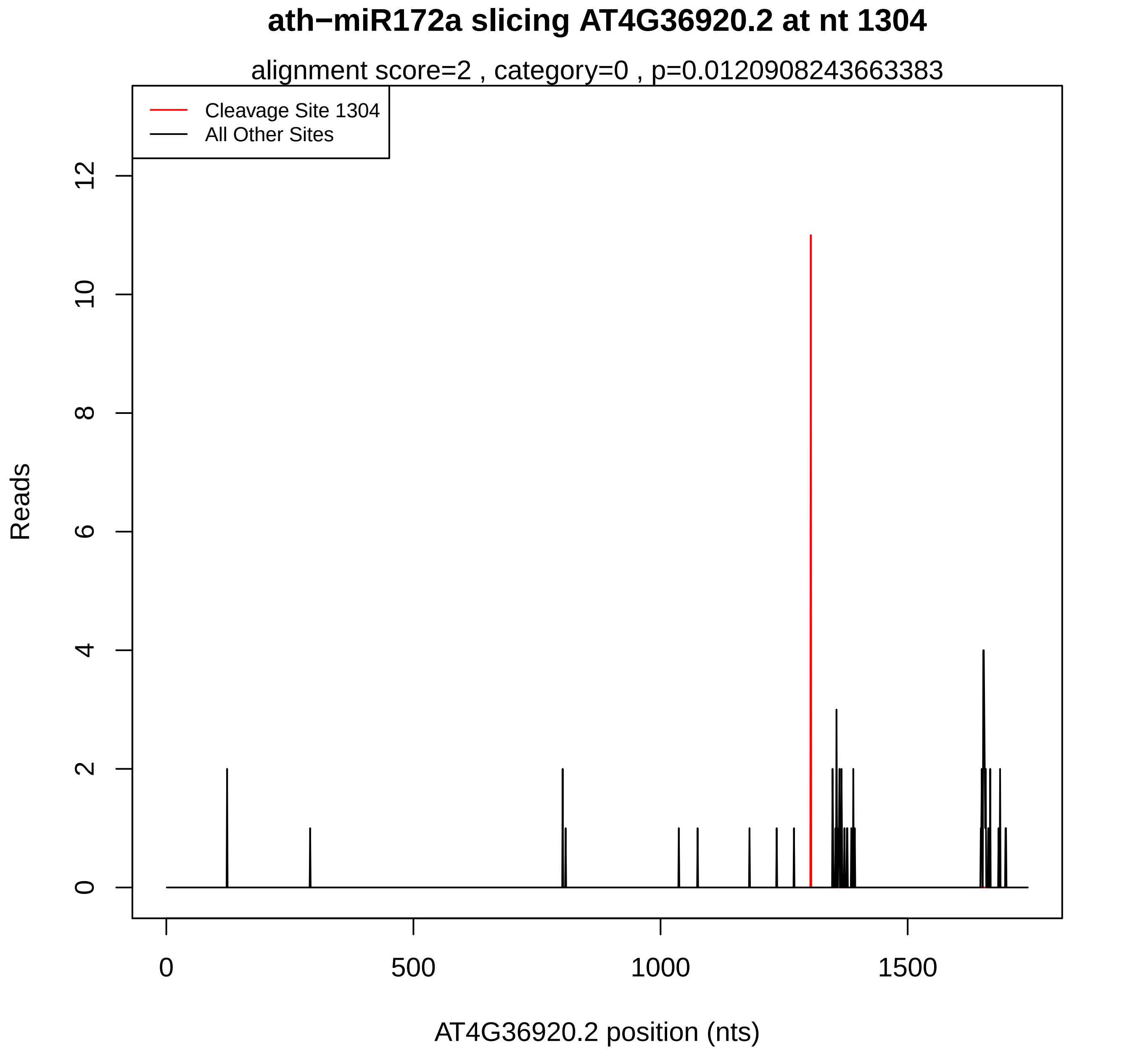
ath−miR172a slicing AT4G36920.2 at nt 1304
alignment score=2 , category=0 , p=0.0120908243663383
Cleavage Site 1304
All Other Sites
Reads
0
2
4
6
8
10
12
0
500
1000
1500
AT4G36920.2 position (nts)

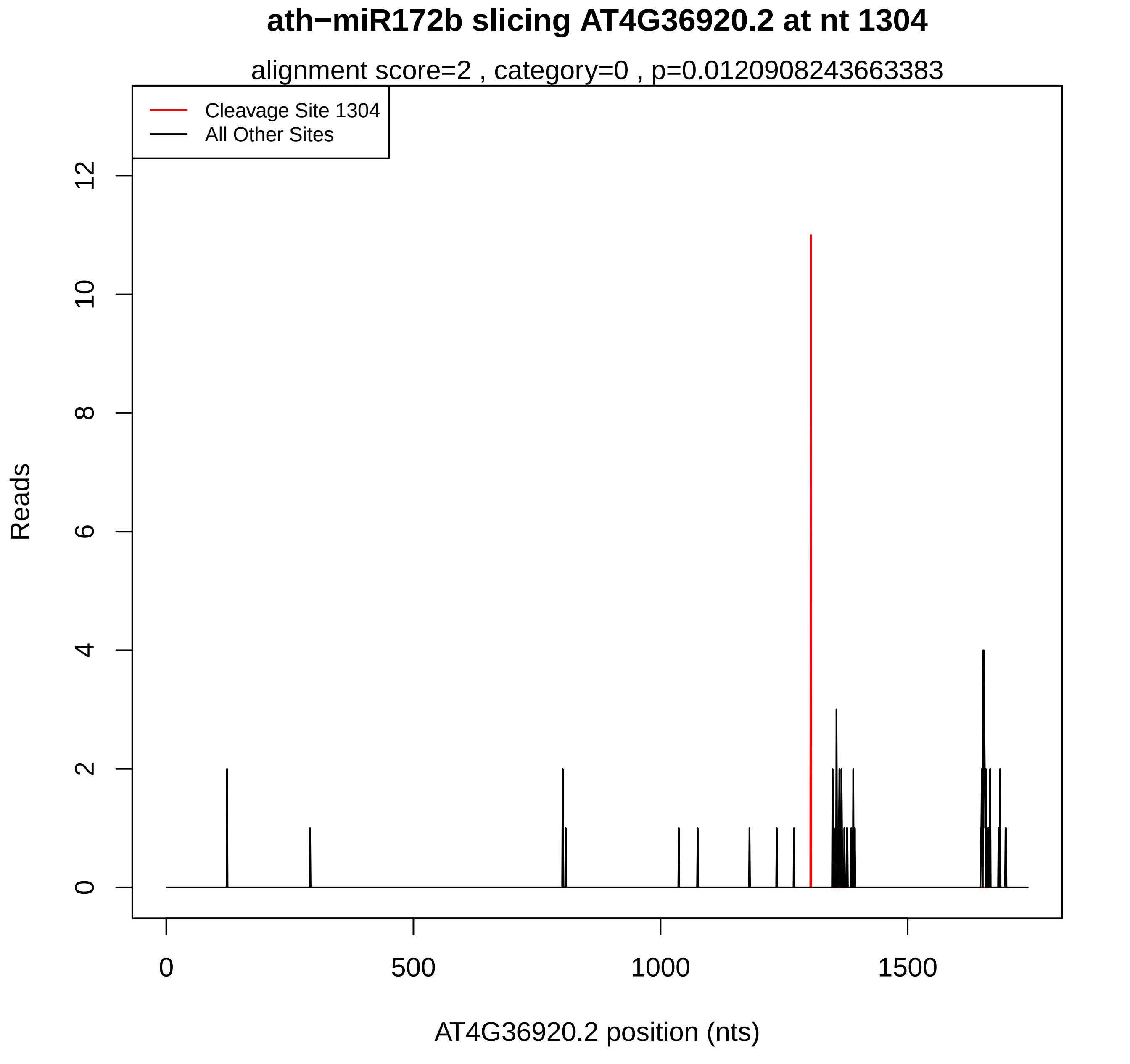
ath−miR172b slicing AT4G36920.2 at nt 1304
alignment score=2 , category=0 , p=0.0120908243663383
Cleavage Site 1304
All Other Sites
Reads
0
2
4
6
8
10
12
0
500
1000
1500
AT4G36920.2 position (nts)

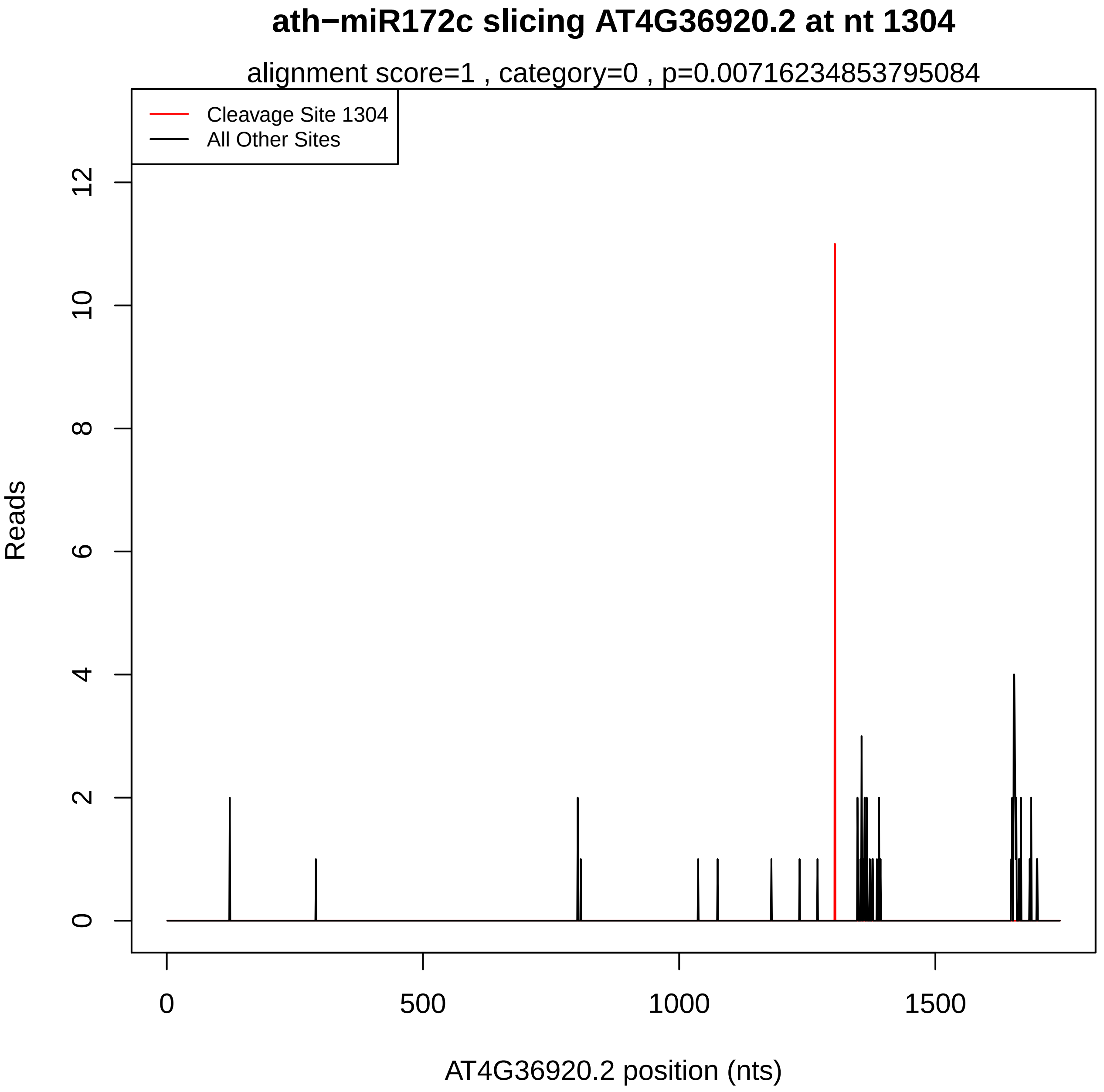
ath−miR172c slicing AT4G36920.2 at nt 1304
alignment score=1 , category=0 , p=0.00716234853795084
Cleavage Site 1304
All Other Sites
Reads
0
2
4
6
8
10
12
0
500
1000
1500
AT4G36920.2 position (nts)

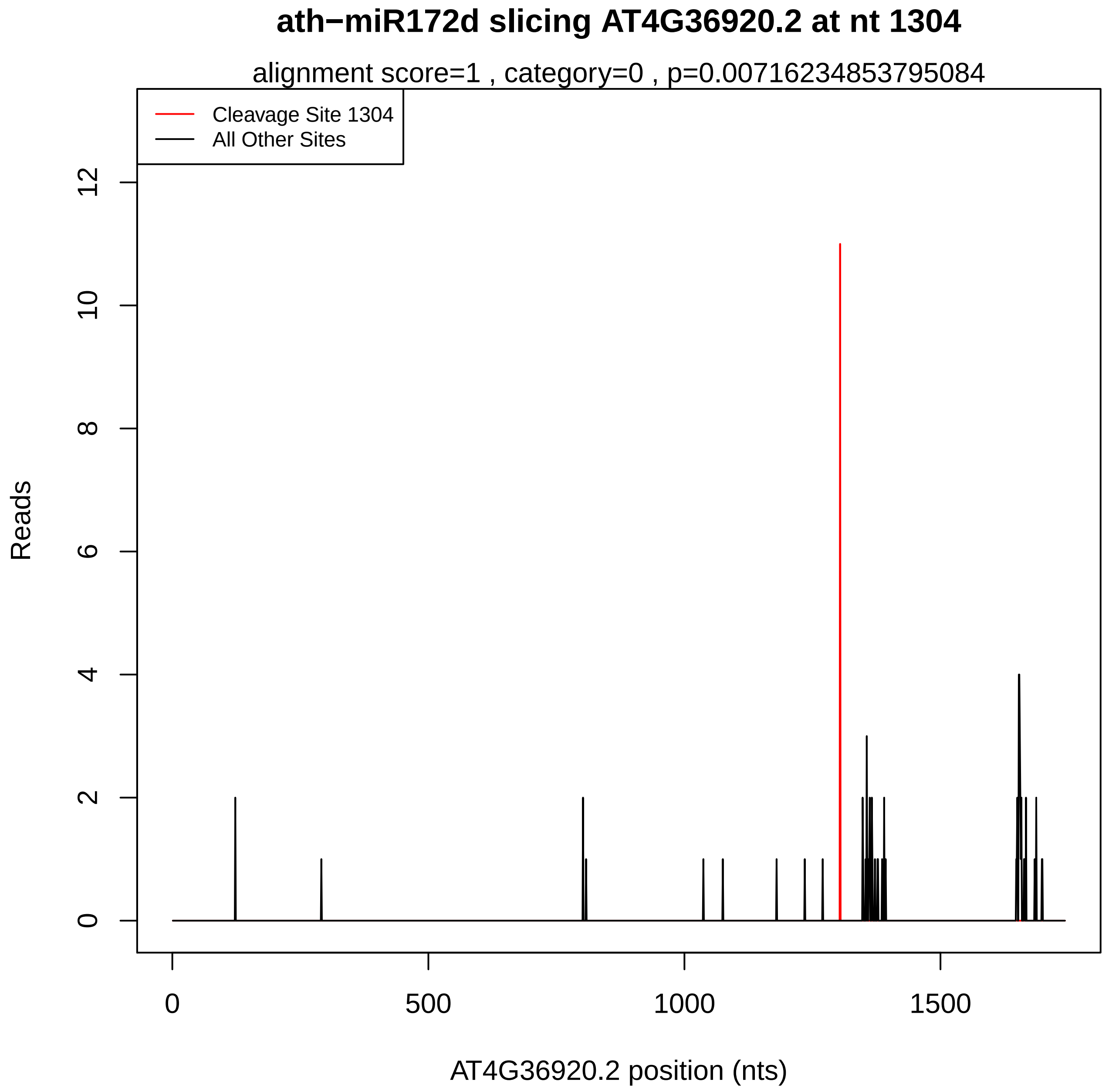

ath−miR172d slicing AT4G36920.2 at nt 1304
alignment score=1 , category=0 , p=0.00716234853795084
Cleavage Site 1304
All Other Sites
Reads
0
2
4
6
8
10
12
0
500
1000
1500
AT4G36920.2 position (nts)

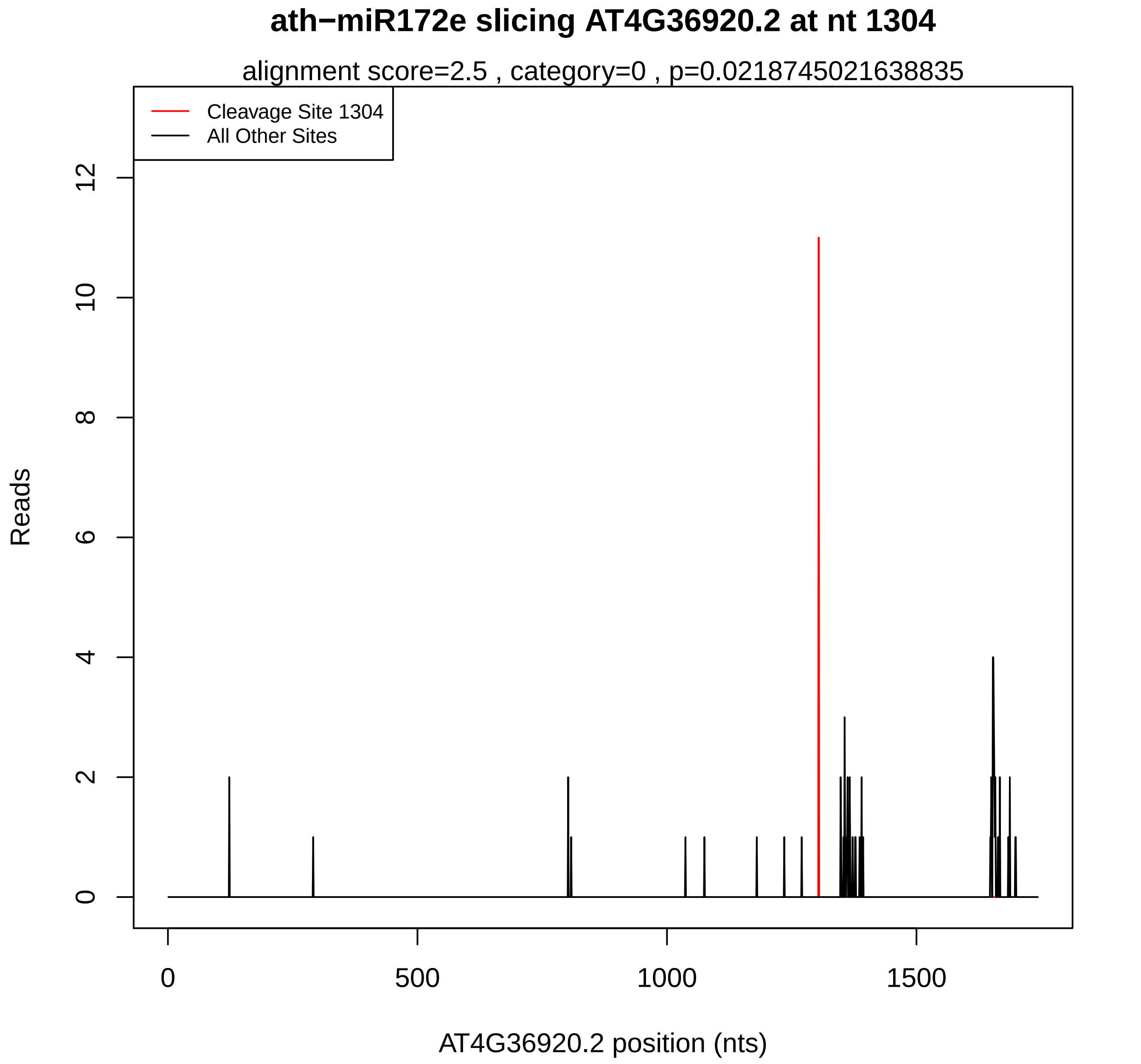

ath−miR172e slicing AT4G36920.2 at nt 1304
alignment score=2.5 , category=0 , p=0.0218745021638835
Cleavage Site 1304
All Other Sites
Reads
0
2
4
6
8
10
12
0
500
1000
1500
AT4G36920.2 position (nts)

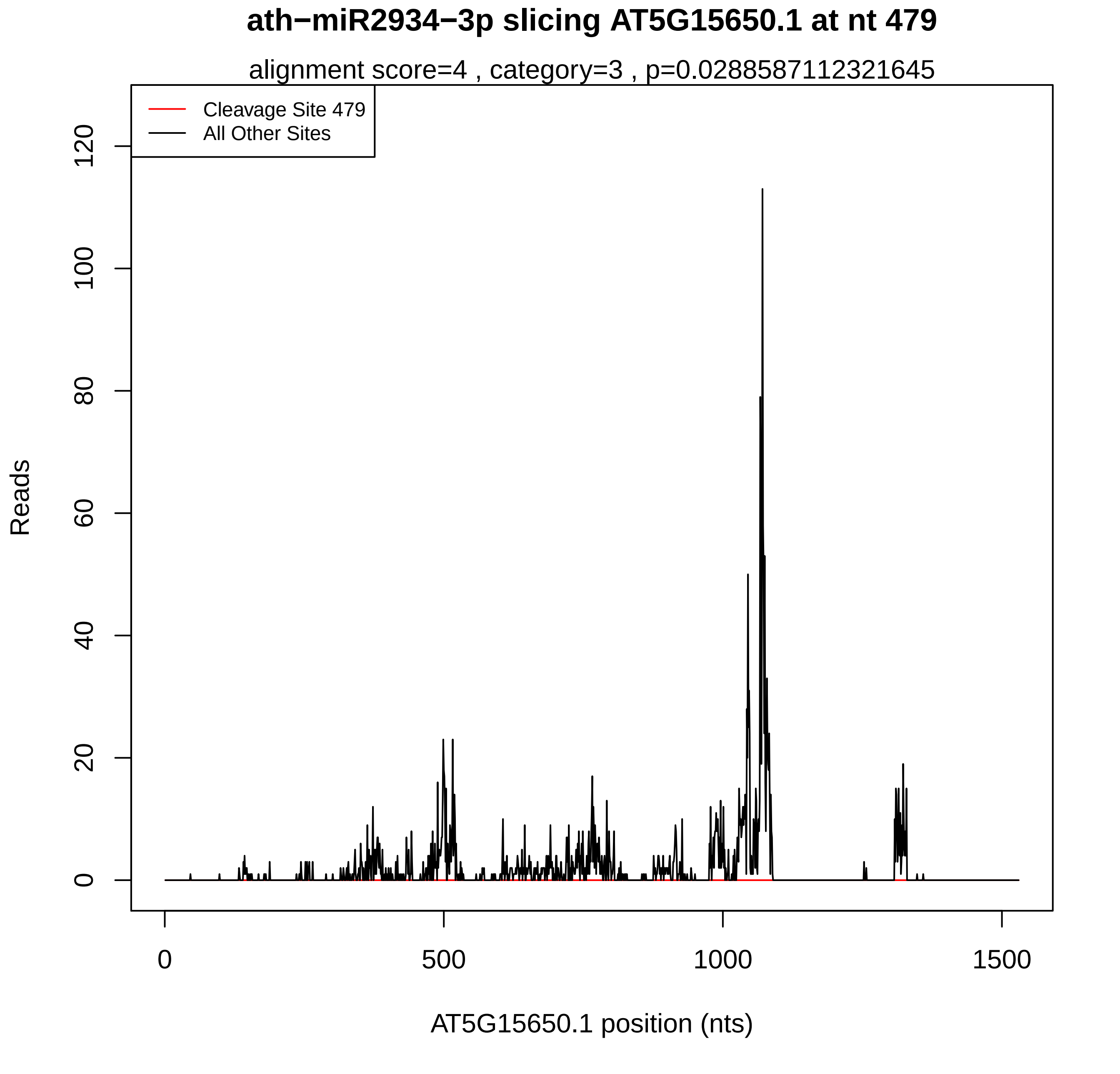

ath−miR2934−3p slicing AT5G15650.1 at nt 479
alignment score=4 , category=3 , p=0.0288587112321645
Cleavage Site 479
All Other Sites
Reads
0
20
40
60
80
100
120
0
500
1000
1500
AT5G15650.1 position (nts)

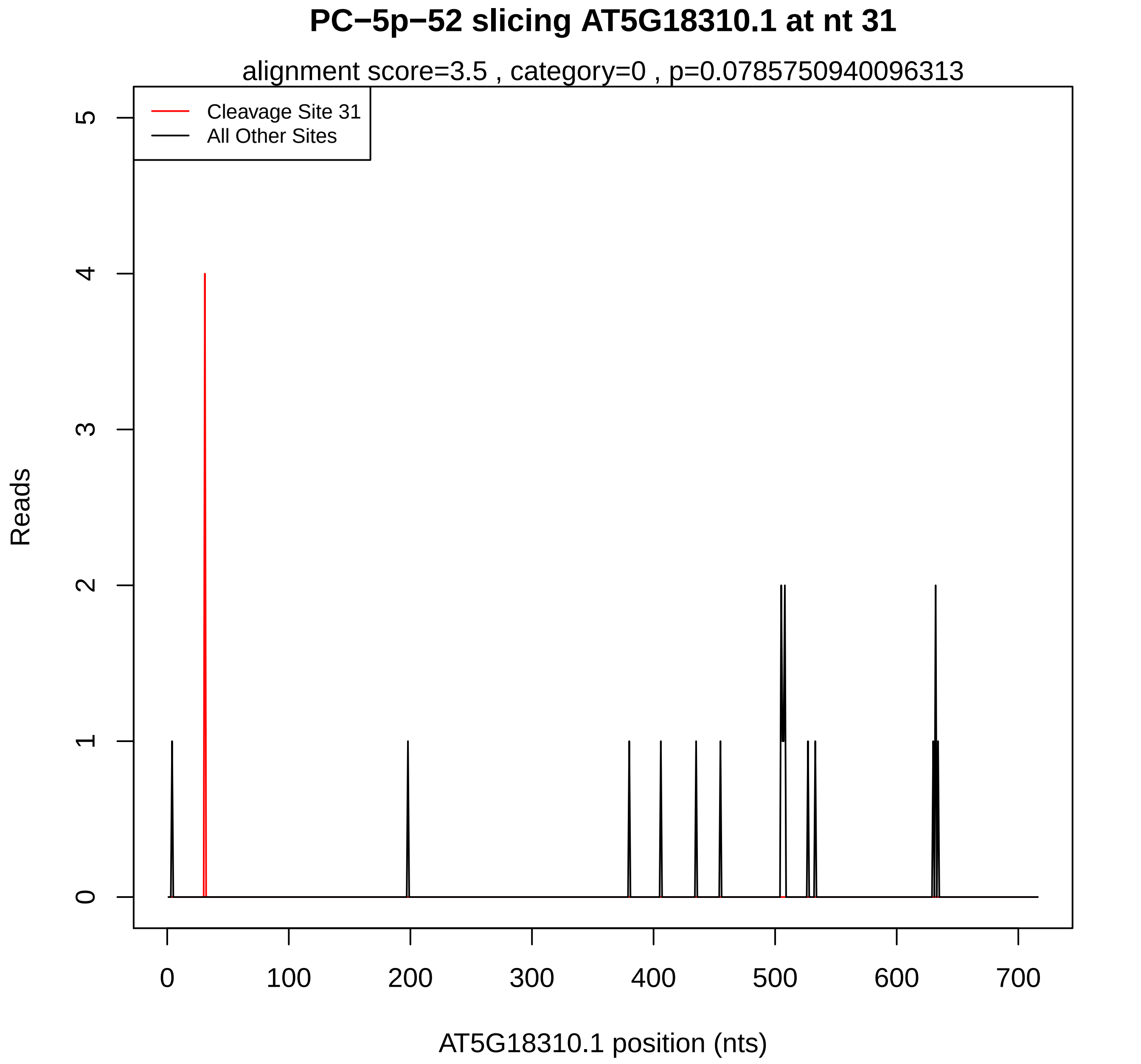

PC−5p−52 slicing AT5G18310.1 at nt 31
alignment score=3.5 , category=0 , p=0.0785750940096313
Cleavage Site 31
All Other Sites
Reads
0
1
2
3
4
5
0
100
200
300
400
500
600
700
AT5G18310.1 position (nts)

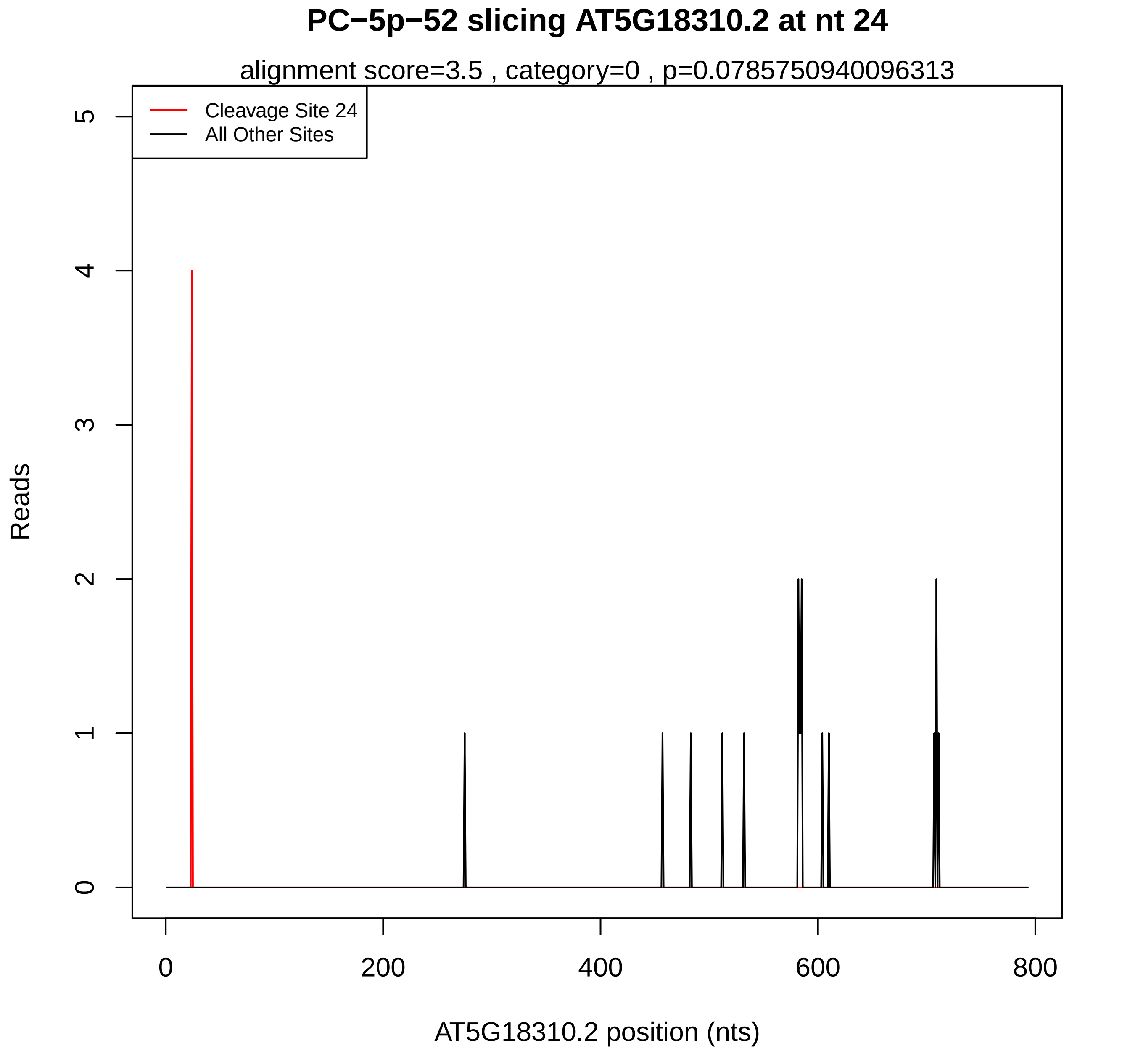

PC−5p−52 slicing AT5G18310.2 at nt 24
alignment score=3.5 , category=0 , p=0.0785750940096313
Cleavage Site 24
All Other Sites
Reads
0
1
2
3
4
5
0
200
400
600
800
AT5G18310.2 position (nts)

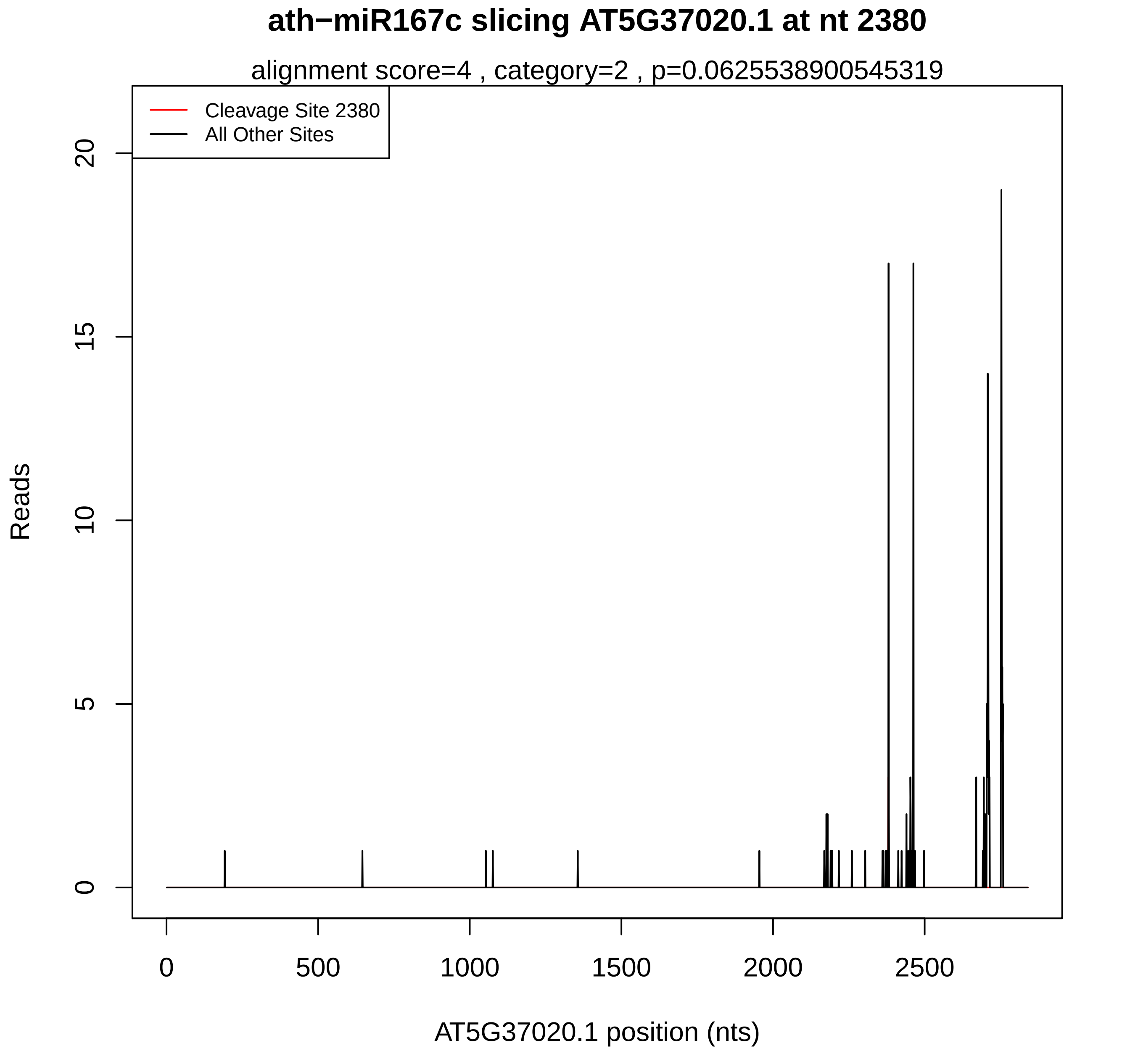

ath−miR167c slicing AT5G37020.1 at nt 2380
alignment score=4 , category=2 , p=0.0625538900545319
Cleavage Site 2380
All Other Sites
Reads
0
5
10
15
20
0
500
1000
1500
2000
2500
AT5G37020.1 position (nts)

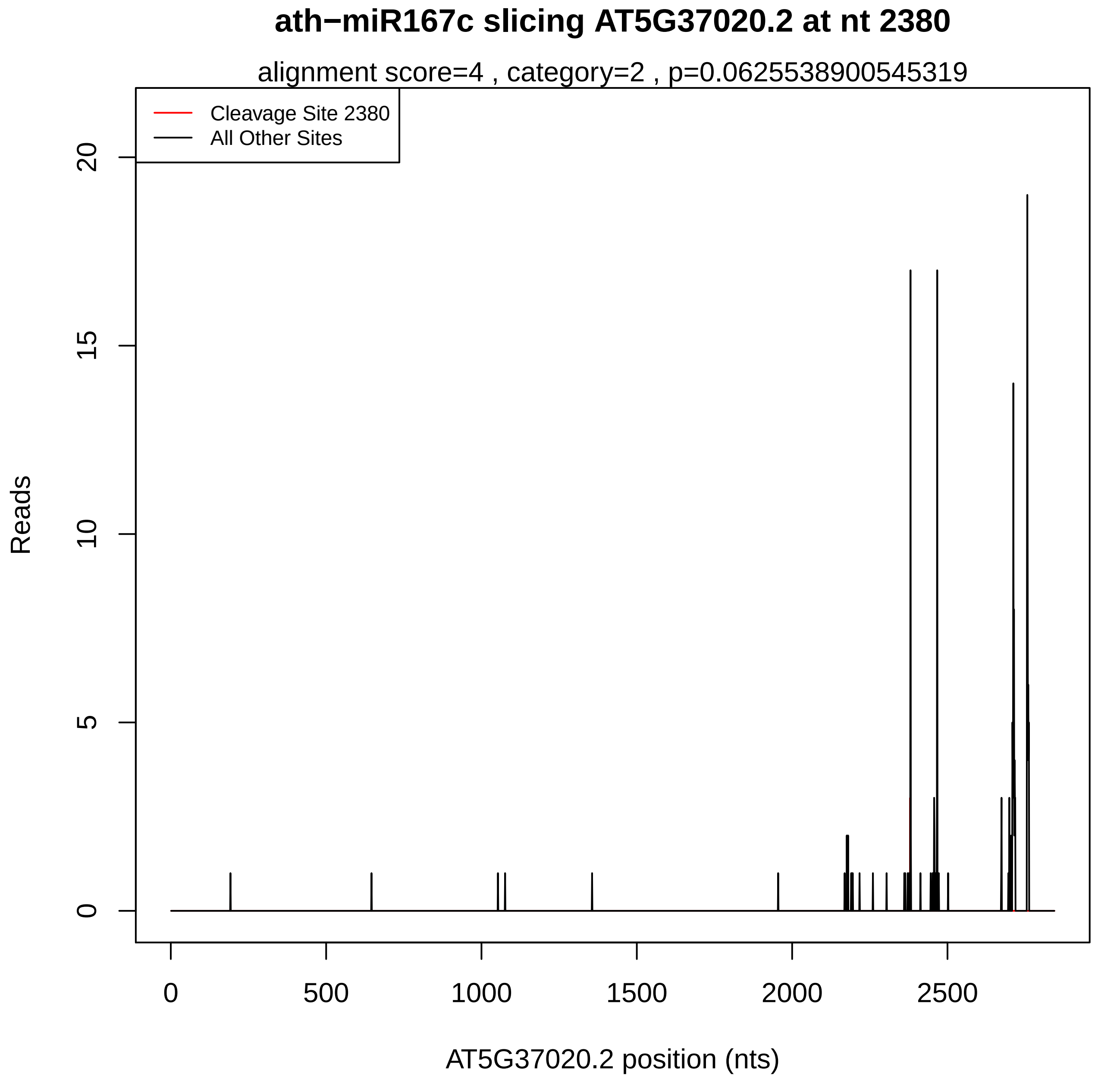
ath−miR167c slicing AT5G37020.2 at nt 2380
alignment score=4 , category=2 , p=0.0625538900545319
Cleavage Site 2380
All Other Sites
Reads
0
5
10
15
20
0
500
1000
1500
2000
2500
AT5G37020.2 position (nts)

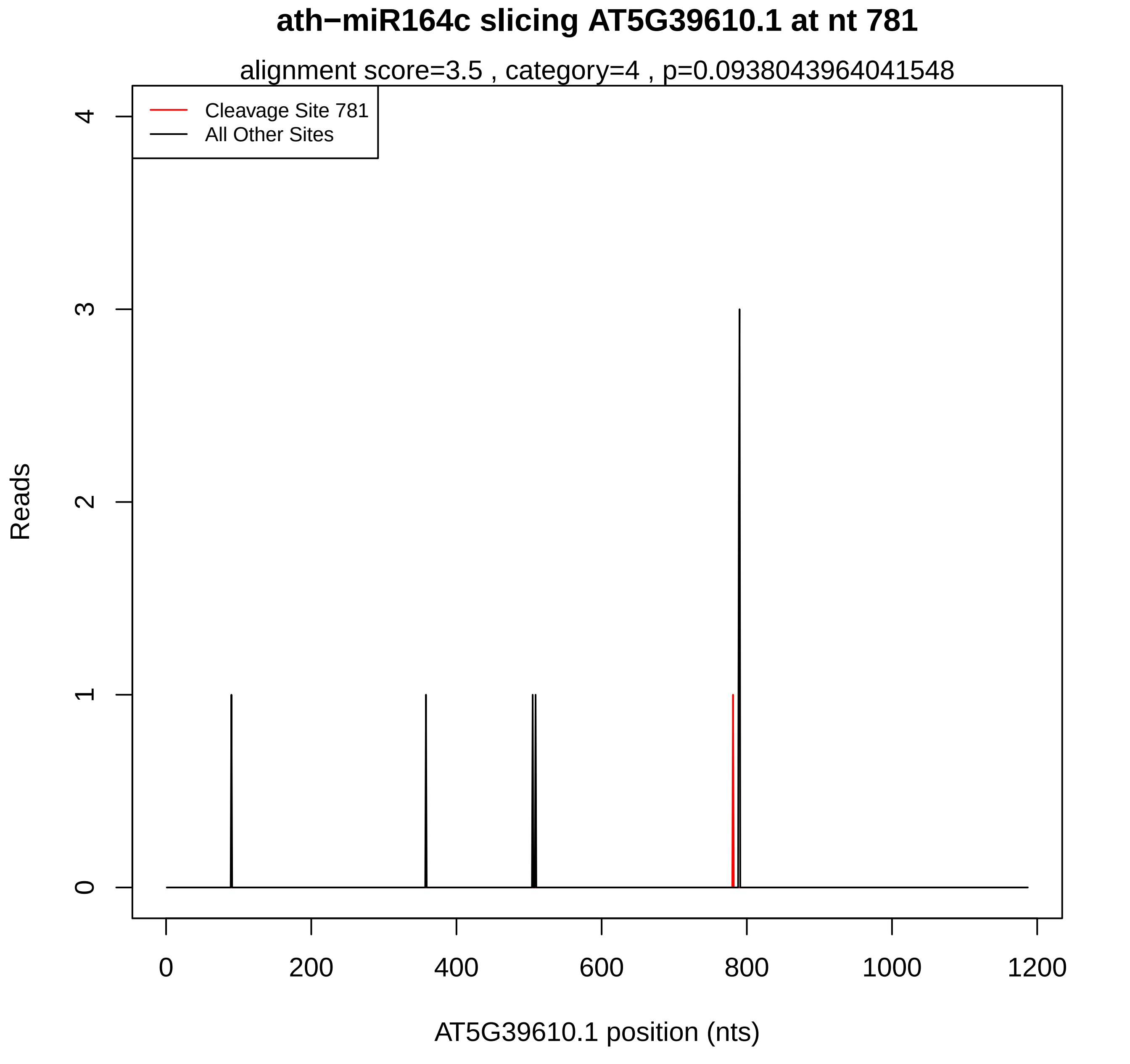
ath−miR164c slicing AT5G39610.1 at nt 781
alignment score=3.5 , category=4 , p=0.0938043964041548
Cleavage Site 781
All Other Sites
Reads
0
1
2
3
4
0
200
400
600
800
1000
1200
AT5G39610.1 position (nts)

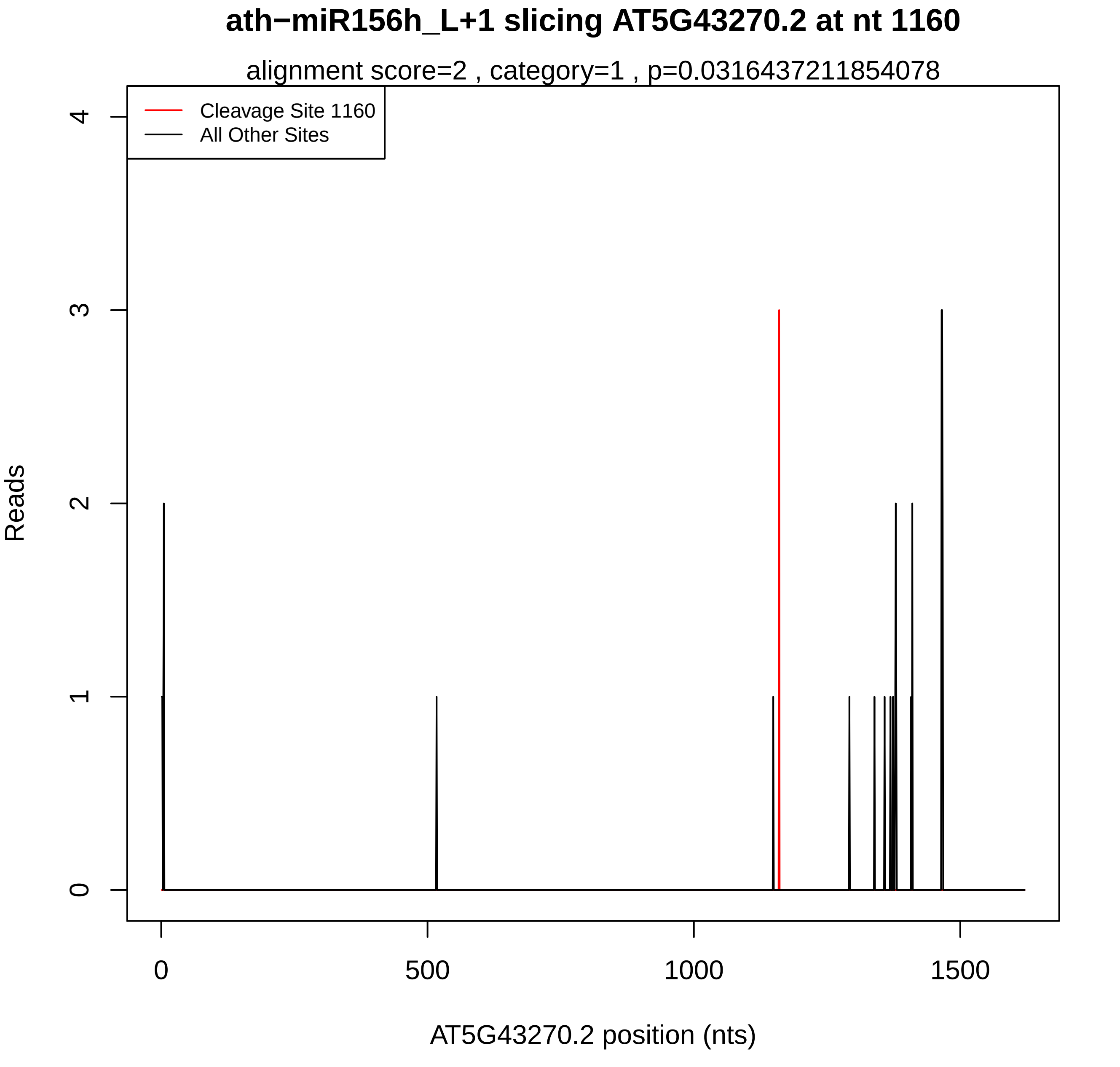
ath−miR156h_L+1 slicing AT5G43270.2 at nt 1160
alignment score=2 , category=1 , p=0.0316437211854078
Cleavage Site 1160
All Other Sites
Reads
0
1
2
3
4
0
500
1000
1500
AT5G43270.2 position (nts)

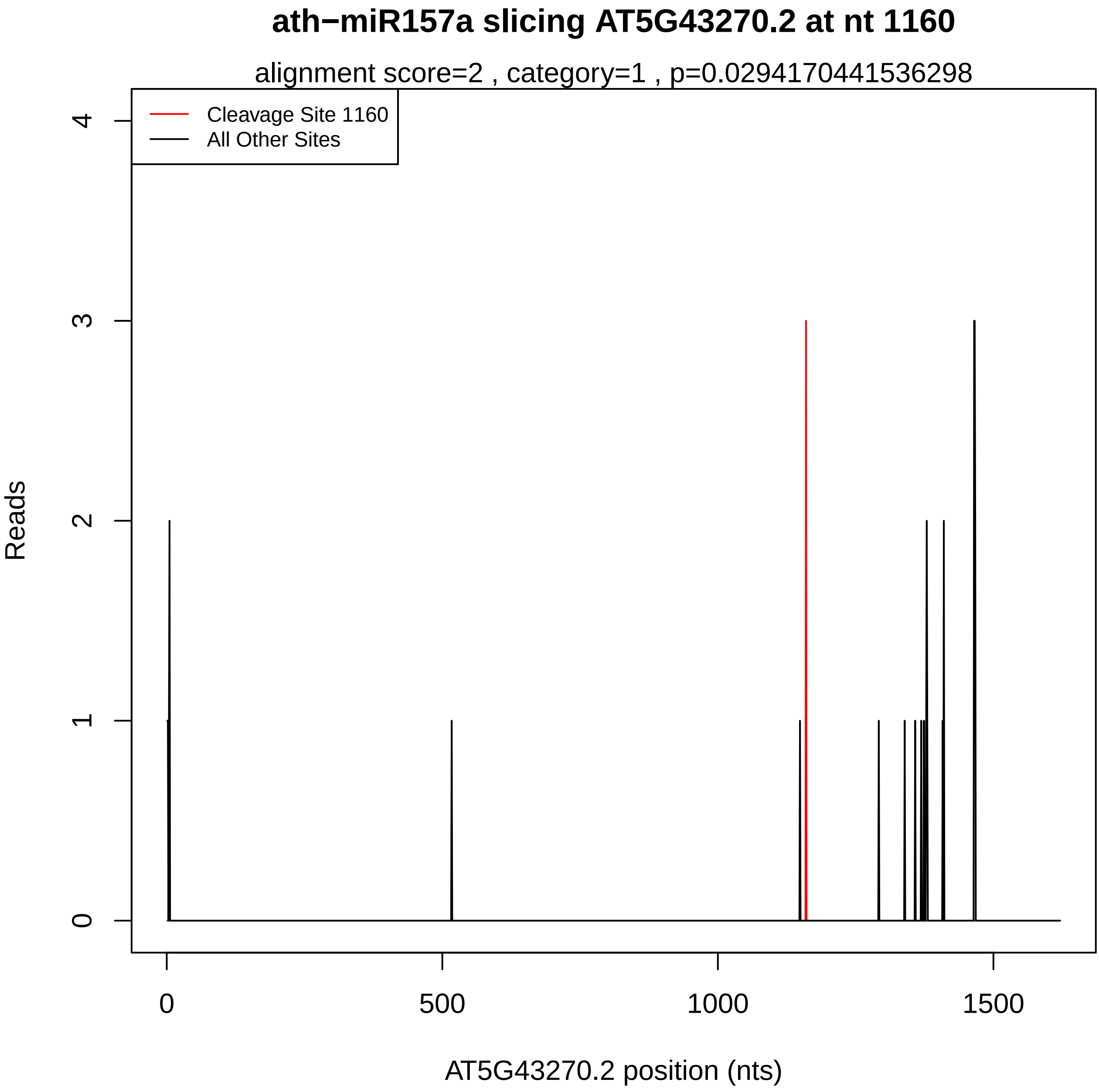
ath−miR157a slicing AT5G43270.2 at nt 1160
alignment score=2 , category=1 , p=0.0294170441536298
Cleavage Site 1160
All Other Sites
Reads
0
1
2
3
4
0
500
1000
1500
AT5G43270.2 position (nts)

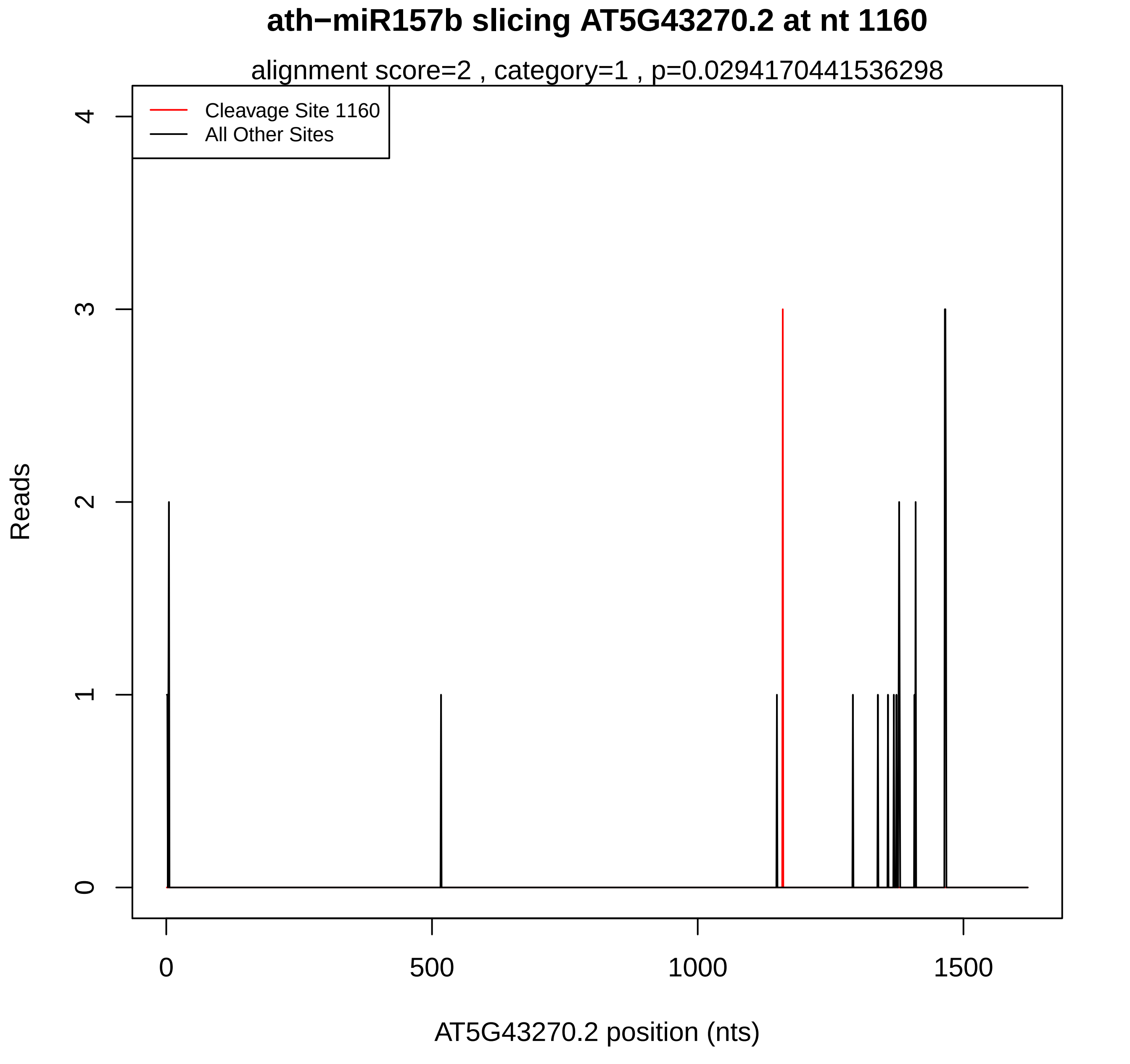

ath−miR157b slicing AT5G43270.2 at nt 1160
alignment score=2 , category=1 , p=0.0294170441536298
Cleavage Site 1160
All Other Sites
Reads
0
1
2
3
4
0
500
1000
1500
AT5G43270.2 position (nts)

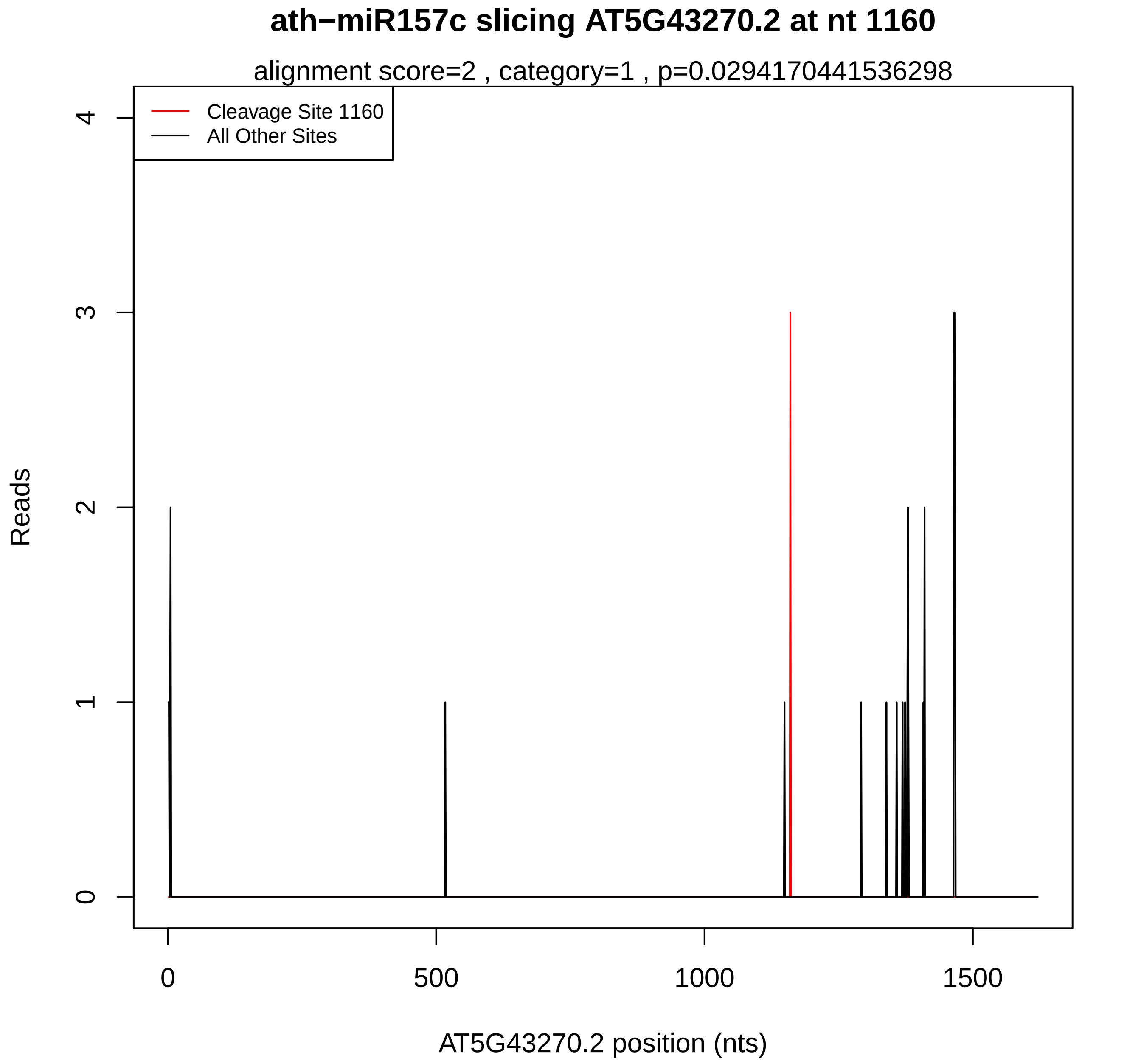

ath−miR157c slicing AT5G43270.2 at nt 1160
alignment score=2 , category=1 , p=0.0294170441536298
Cleavage Site 1160
All Other Sites
Reads
AT5G43270.2 position (nts)
0
1
2
3
4
0
500
1000
1500

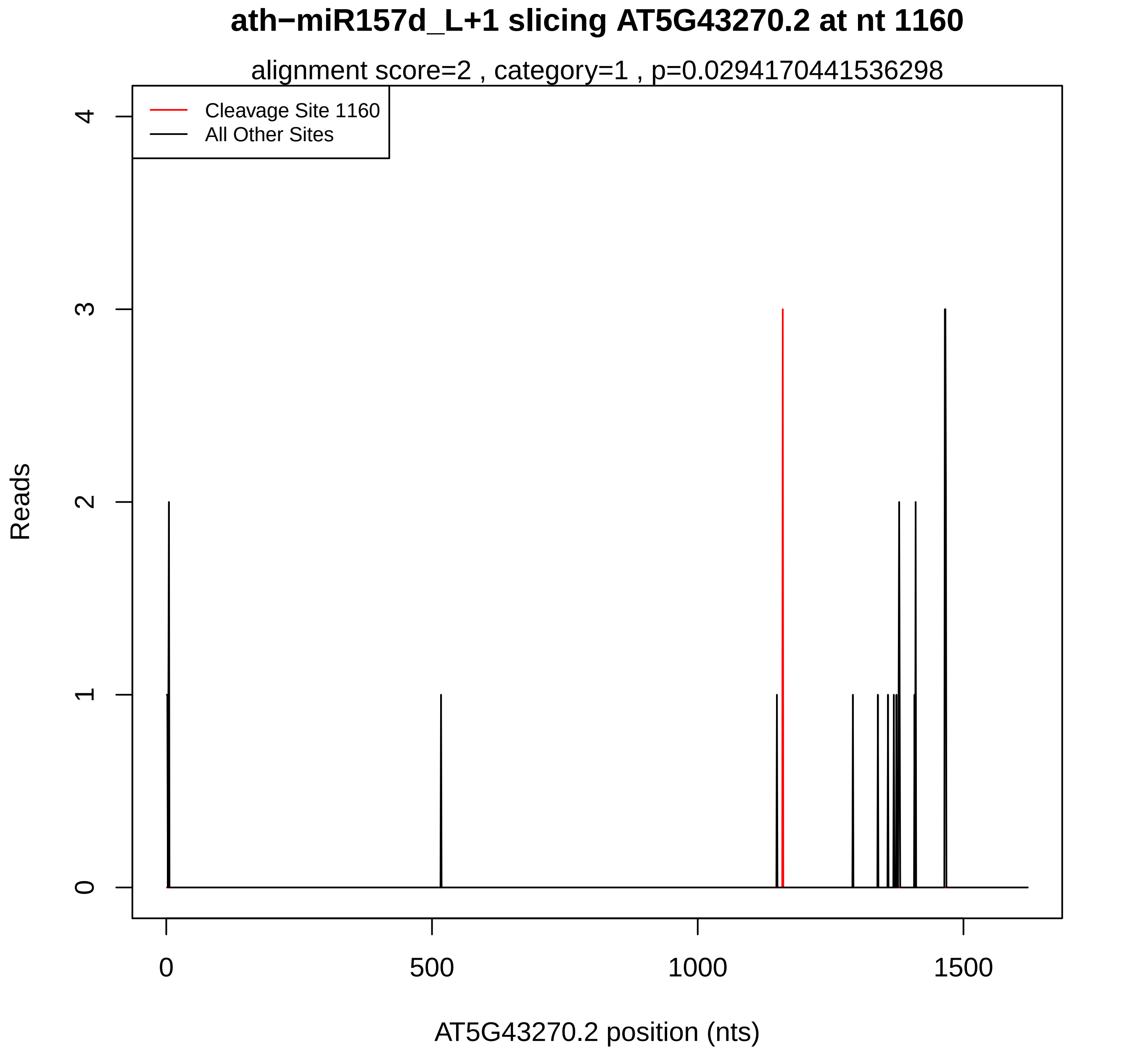

ath−miR157d_L+1 slicing AT5G43270.2 at nt 1160
alignment score=2 , category=1 , p=0.0294170441536298
Cleavage Site 1160
All Other Sites
Reads
0
1
2
3
4
0
500
1000
1500
AT5G43270.2 position (nts)

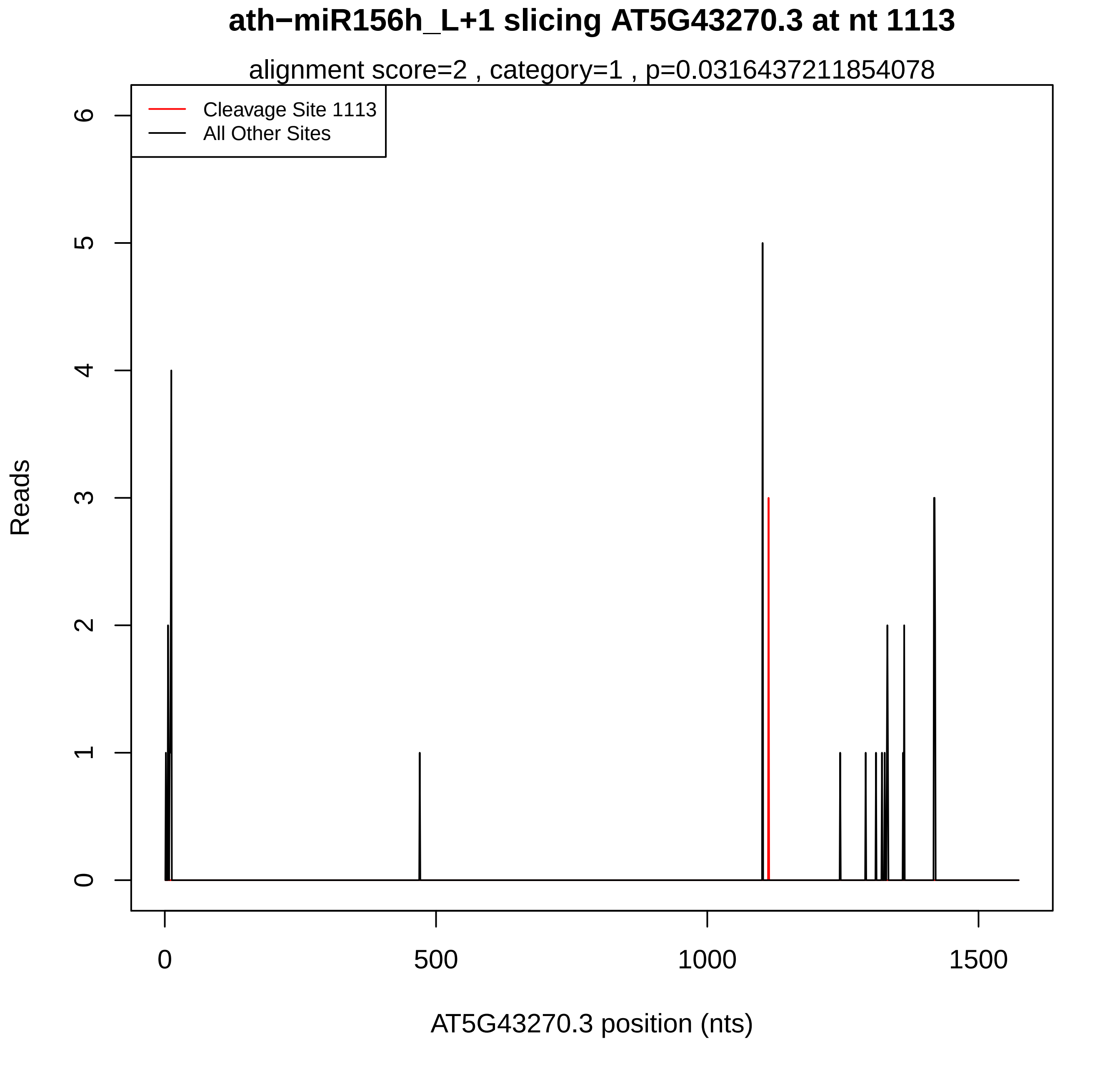

ath−miR156h_L+1 slicing AT5G43270.3 at nt 1113
alignment score=2 , category=1 , p=0.0316437211854078
Cleavage Site 1113
All Other Sites
Reads
AT5G43270.3 position (nts)
0
1
2
3
4
5
6
0
500
1000
1500

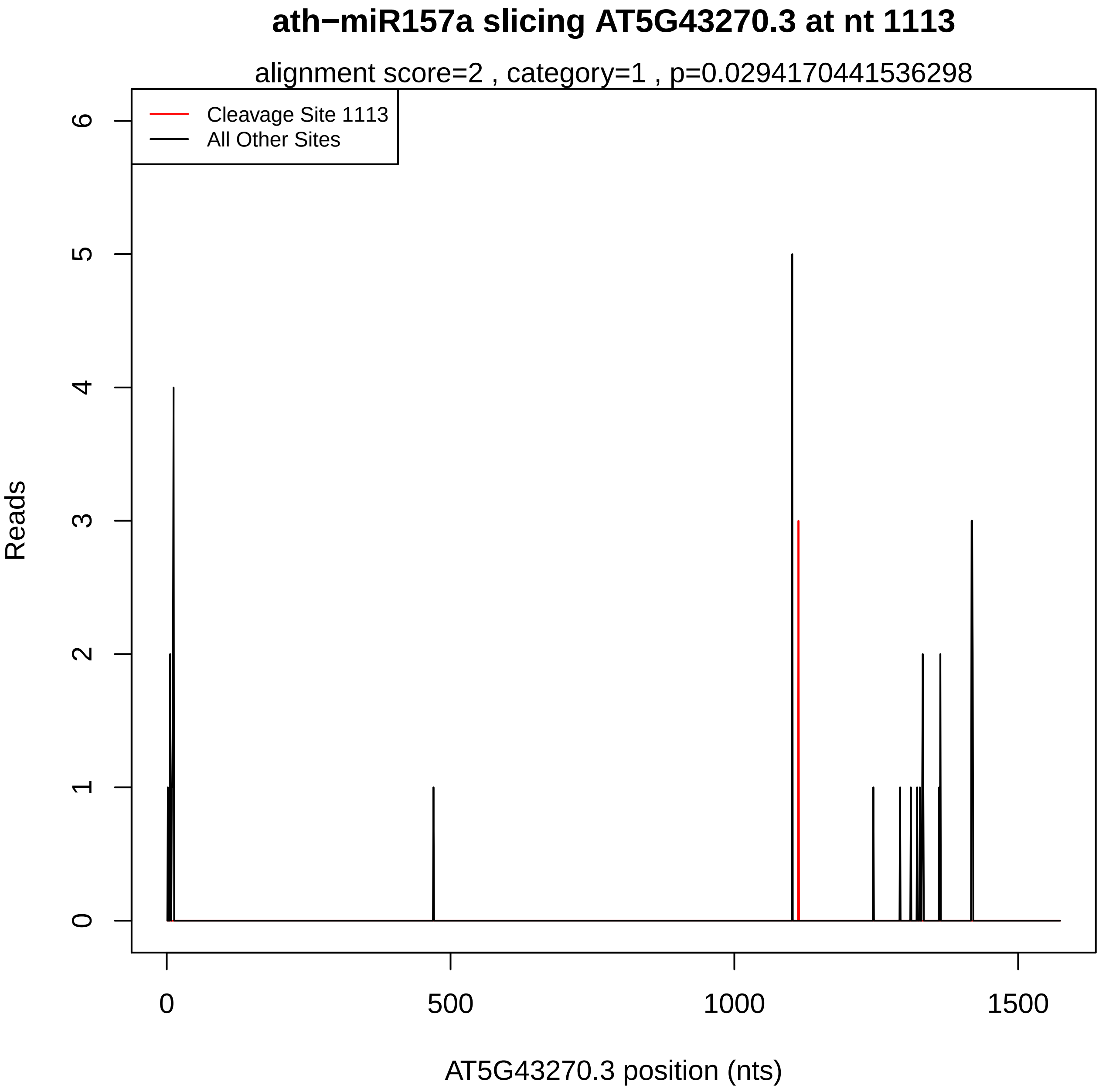

ath−miR157a slicing AT5G43270.3 at nt 1113
alignment score=2 , category=1 , p=0.0294170441536298
Cleavage Site 1113
All Other Sites
Reads
0
1
2
3
4
5
6
0
500
1000
1500
AT5G43270.3 position (nts)

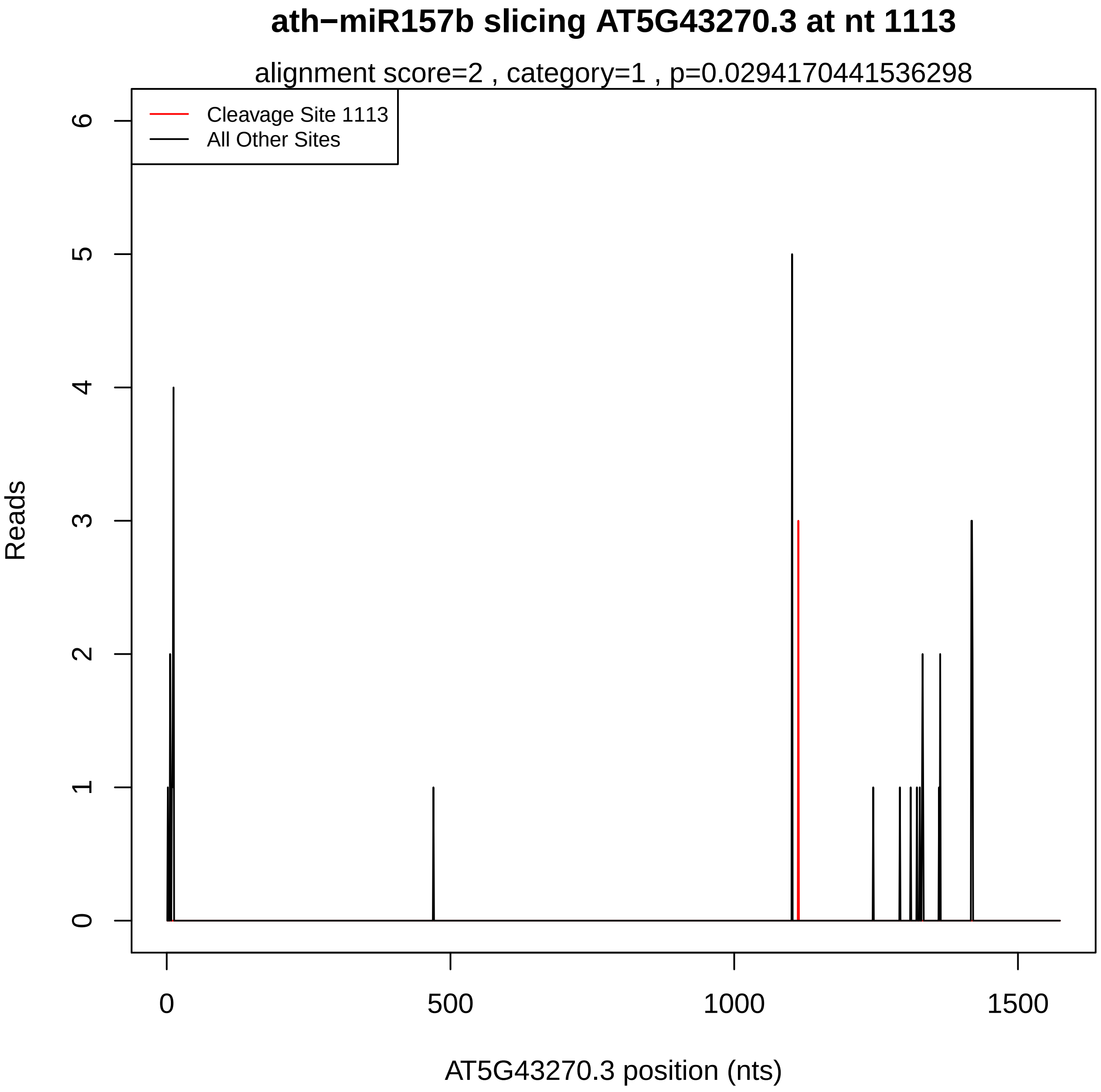
ath−miR157b slicing AT5G43270.3 at nt 1113
alignment score=2 , category=1 , p=0.0294170441536298
Cleavage Site 1113
All Other Sites
Reads
0
1
2
3
4
5
6
0
500
1000
1500
AT5G43270.3 position (nts)

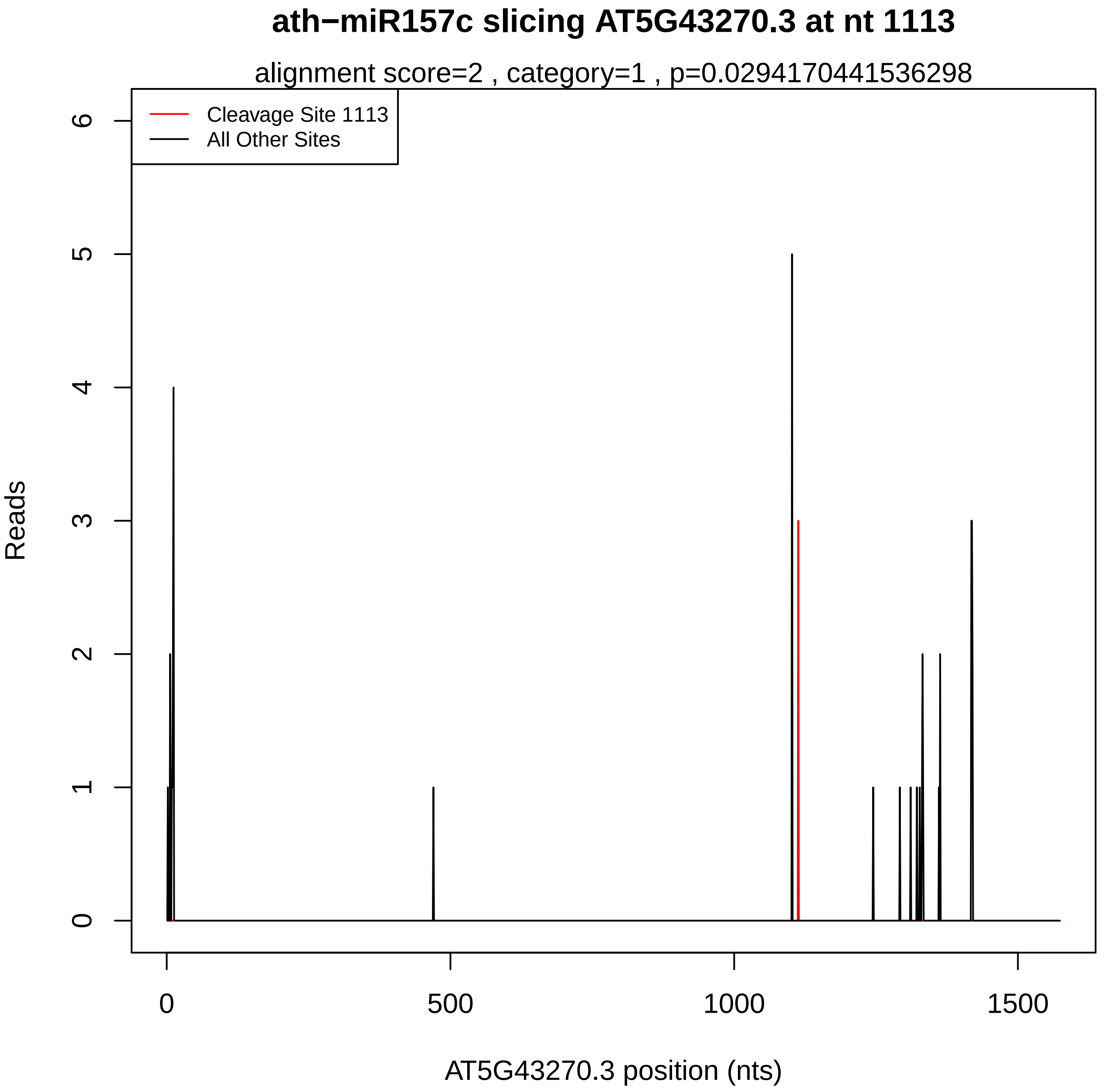
ath−miR157c slicing AT5G43270.3 at nt 1113
alignment score=2 , category=1 , p=0.0294170441536298
Cleavage Site 1113
All Other Sites
Reads
0
1
2
3
4
5
6
0
500
1000
1500
AT5G43270.3 position (nts)

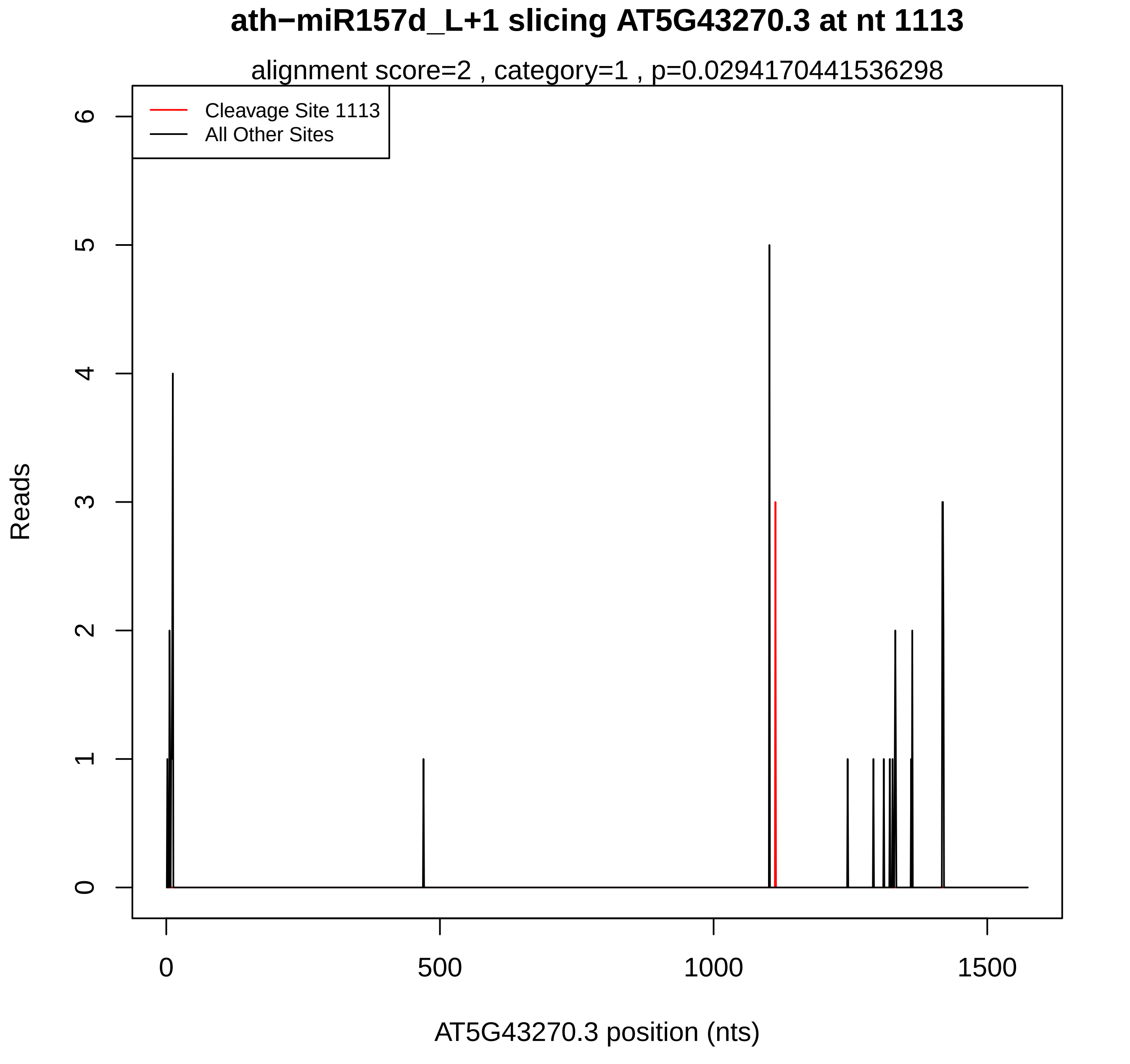

ath−miR157d_L+1 slicing AT5G43270.3 at nt 1113
alignment score=2 , category=1 , p=0.0294170441536298
Cleavage Site 1113
All Other Sites
Reads
0
1
2
3
4
5
6
0
500
1000
1500
AT5G43270.3 position (nts)

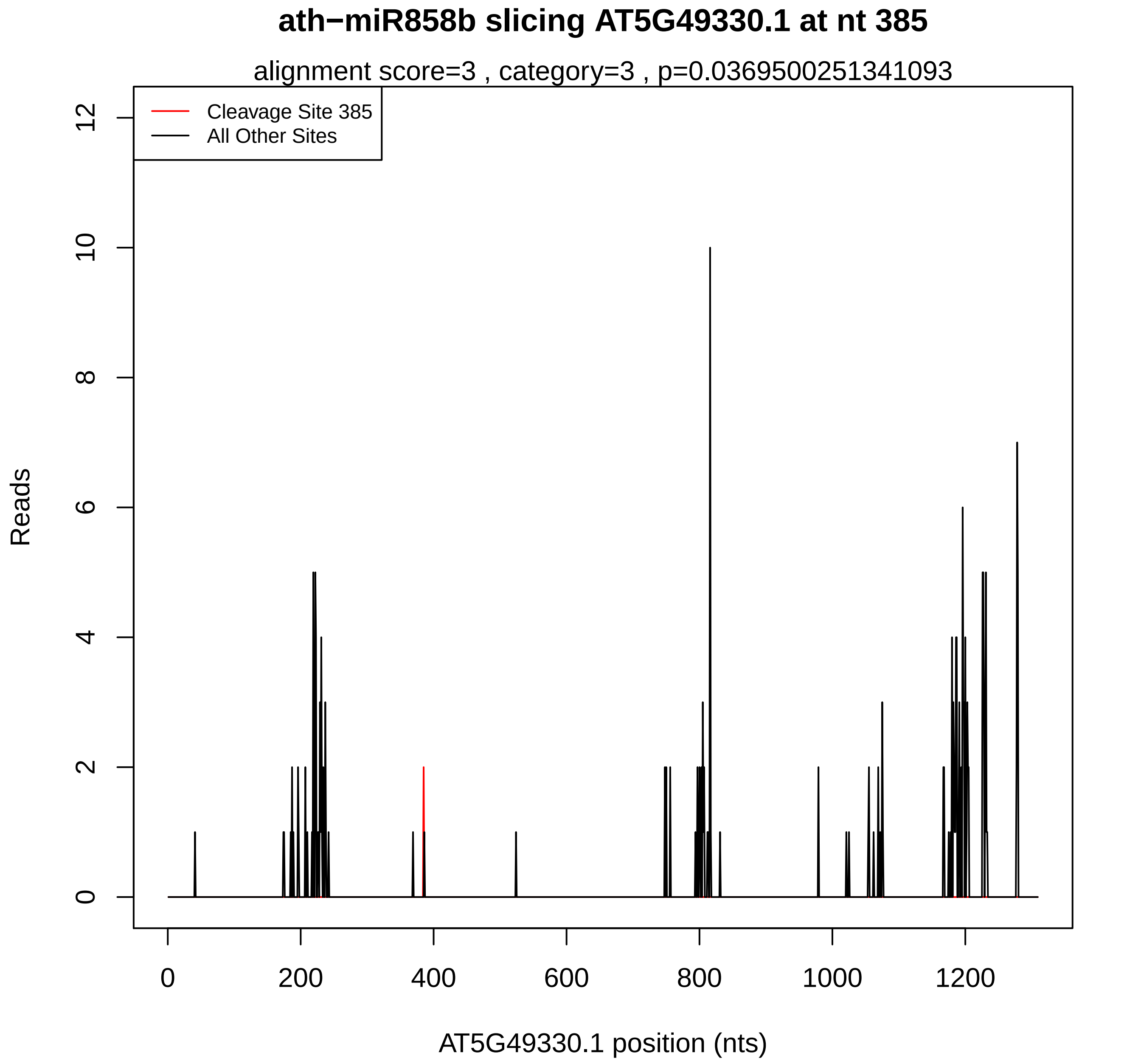
ath−miR858b slicing AT5G49330.1 at nt 385
alignment score=3 , category=3 , p=0.0369500251341093
Cleavage Site 385
All Other Sites
Reads
0
2
4
6
8
10
12
0
200
400
600
800
1000
1200
AT5G49330.1 position (nts)

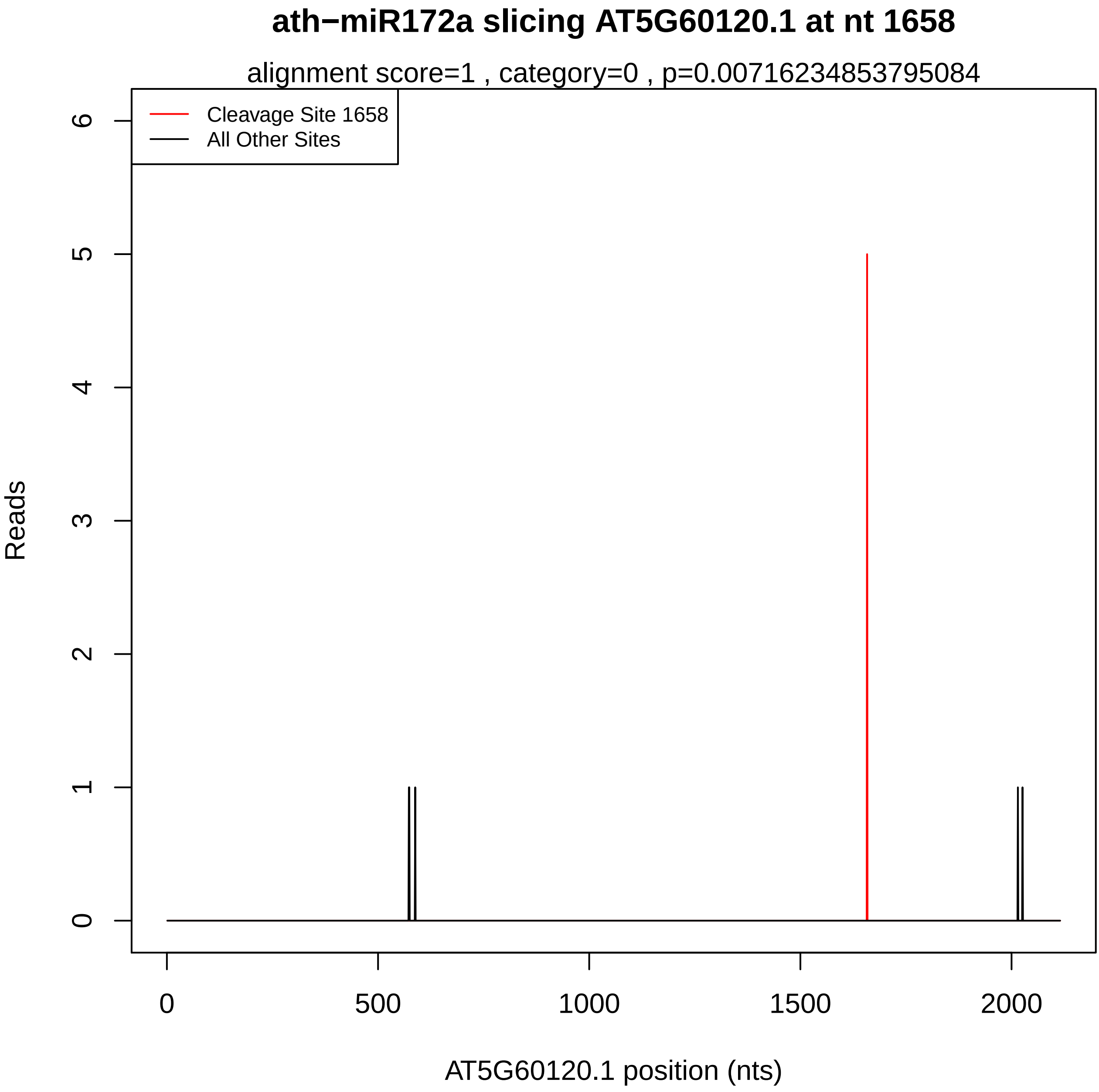
ath−miR172a slicing AT5G60120.1 at nt 1658
alignment score=1 , category=0 , p=0.00716234853795084
Cleavage Site 1658
All Other Sites
Reads
0
1
2
3
4
5
6
0
500
1000
1500
2000
AT5G60120.1 position (nts)

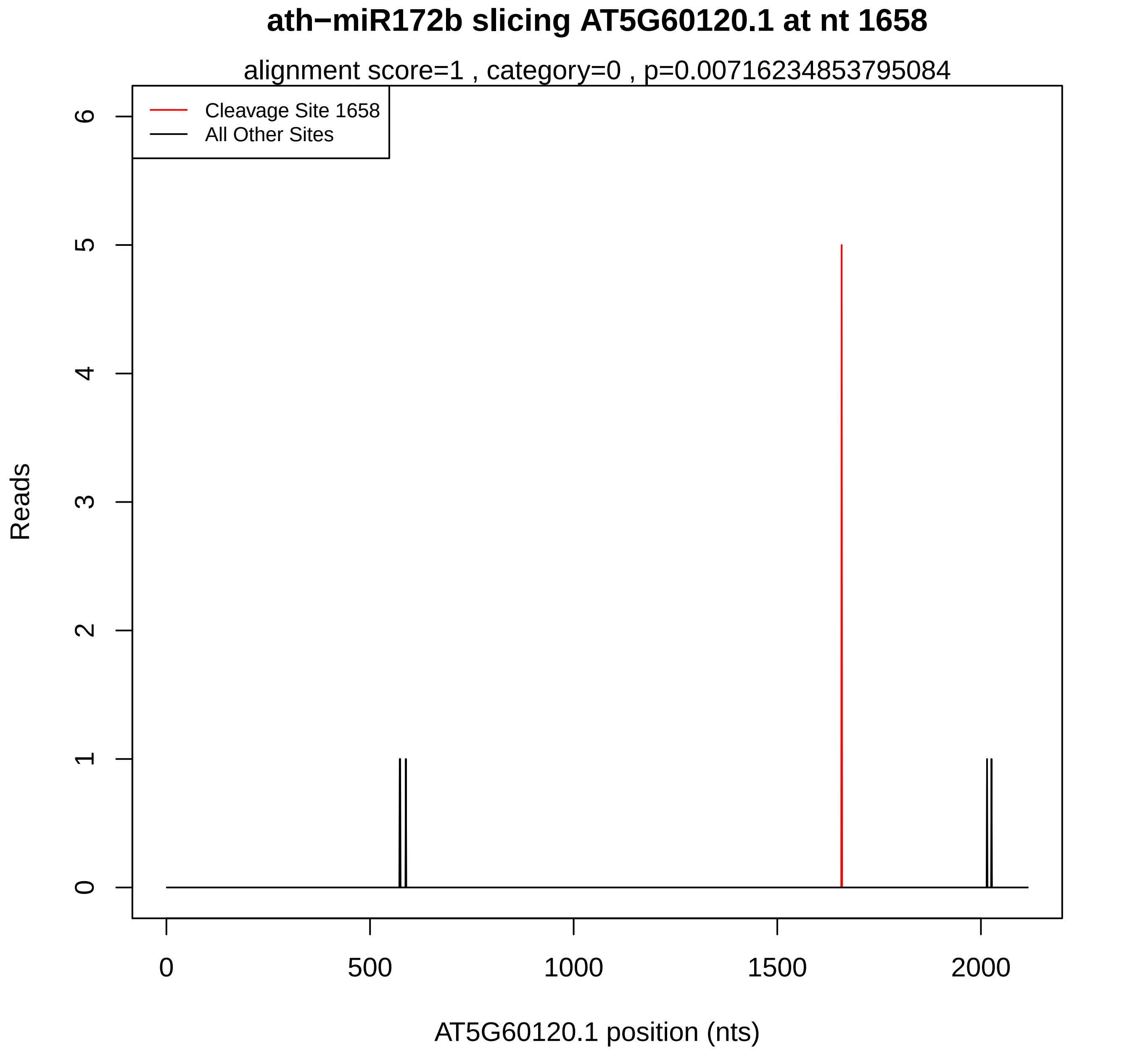
ath−miR172b slicing AT5G60120.1 at nt 1658
alignment score=1 , category=0 , p=0.00716234853795084
Cleavage Site 1658
All Other Sites
Reads
0
1
2
3
4
5
6
0
500
1000
1500
2000
AT5G60120.1 position (nts)

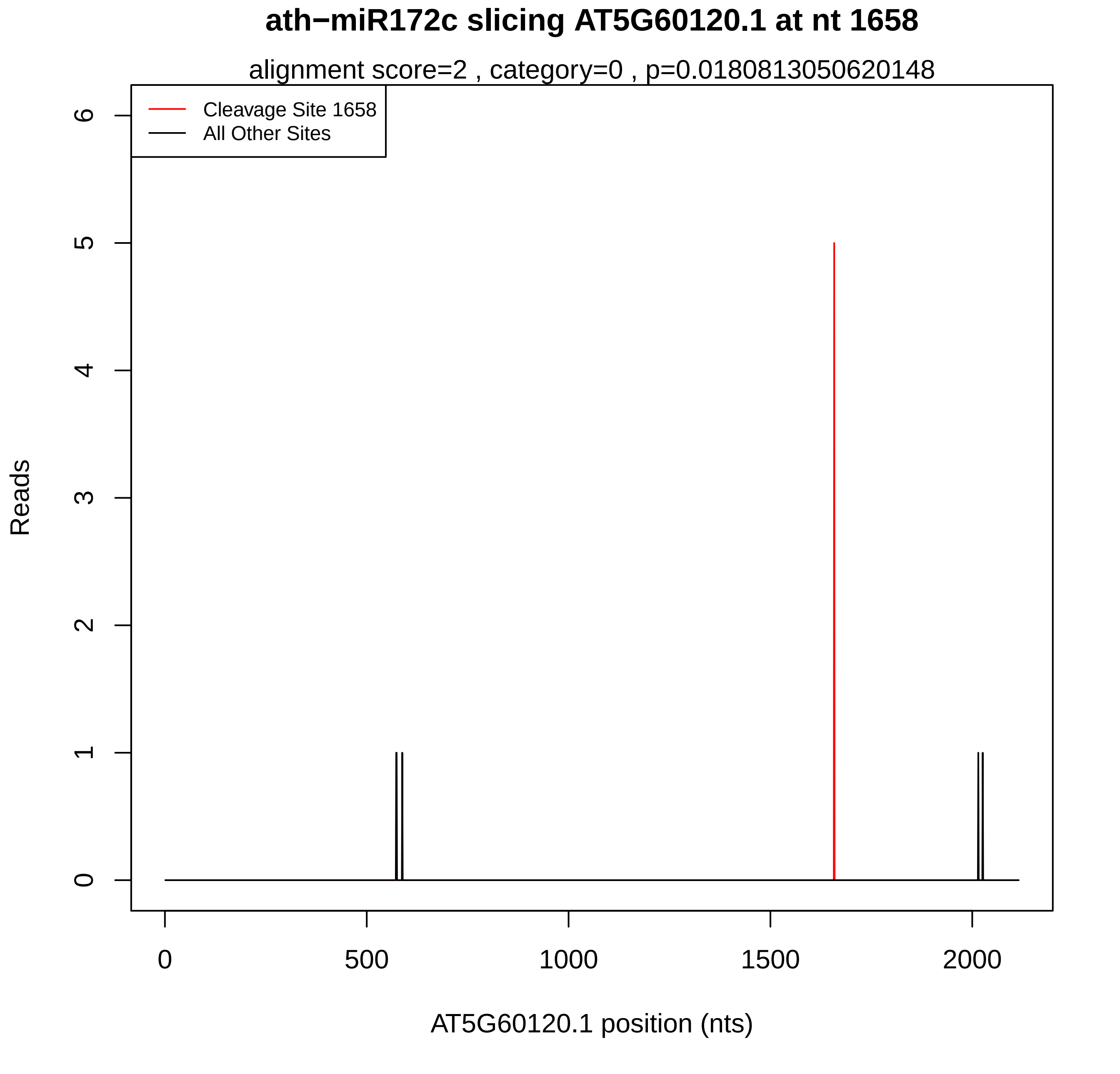
ath−miR172c slicing AT5G60120.1 at nt 1658
alignment score=2 , category=0 , p=0.0180813050620148
Cleavage Site 1658
All Other Sites
Reads
0
1
2
3
4
5
6
0
500
1000
1500
2000
AT5G60120.1 position (nts)

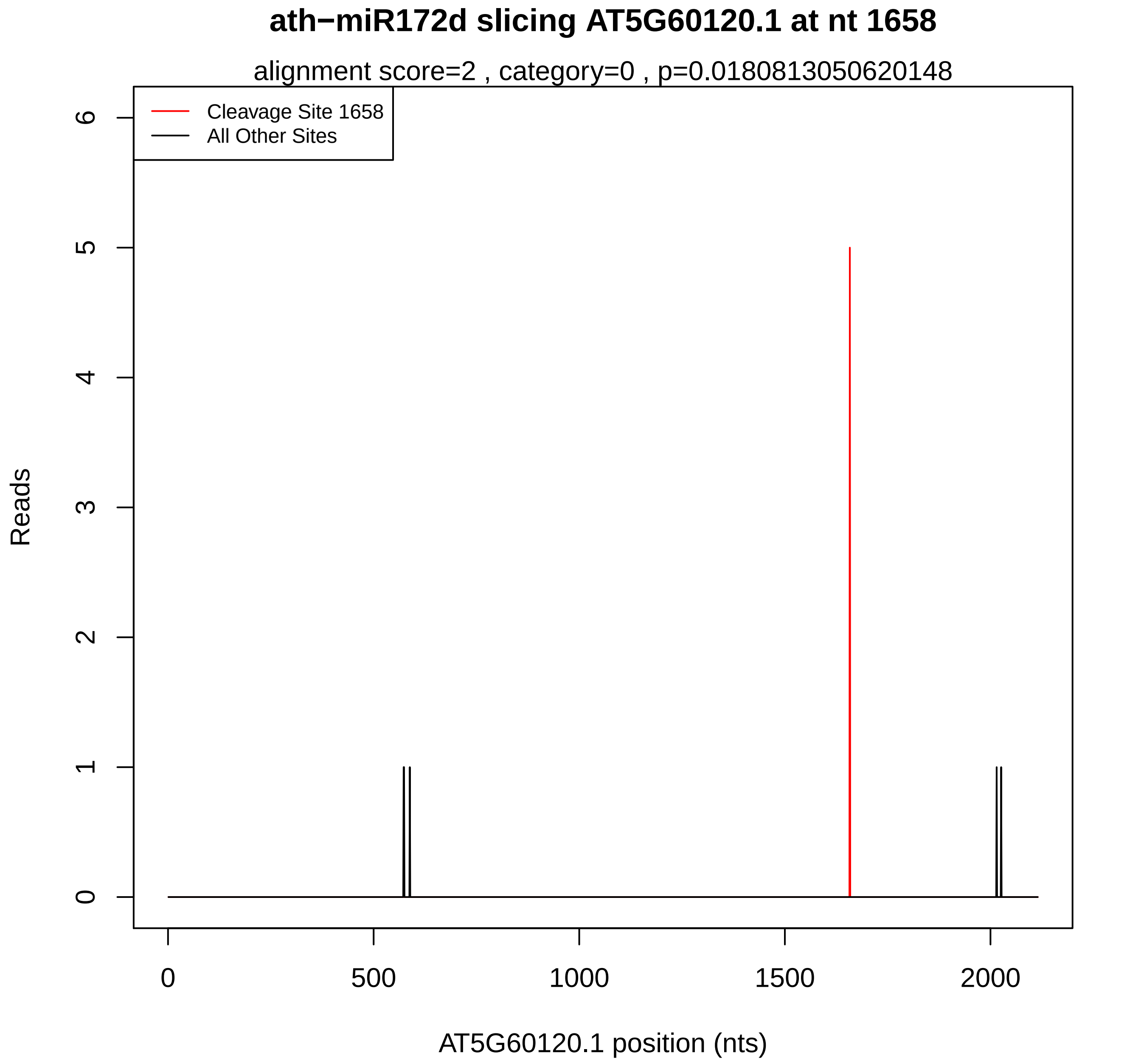

ath−miR172d slicing AT5G60120.1 at nt 1658
alignment score=2 , category=0 , p=0.0180813050620148
Cleavage Site 1658
All Other Sites
Reads
0
1
2
3
4
5
6
0
500
1000
1500
2000
AT5G60120.1 position (nts)

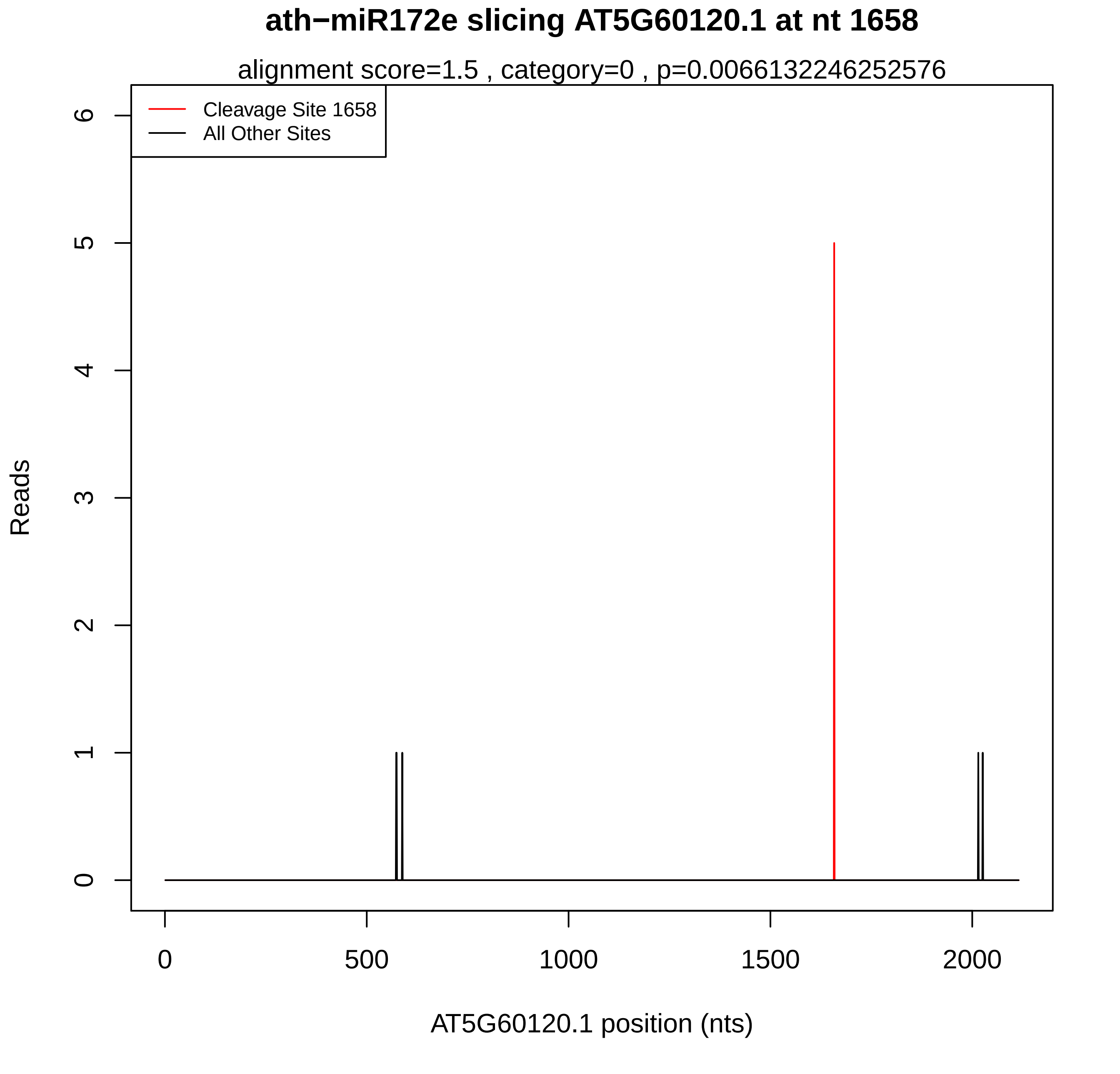
ath−miR172e slicing AT5G60120.1 at nt 1658
alignment score=1.5 , category=0 , p=0.0066132246252576
Cleavage Site 1658
All Other Sites
Reads
0
1
2
3
4
5
6
0
500
1000
1500
2000
AT5G60120.1 position (nts)

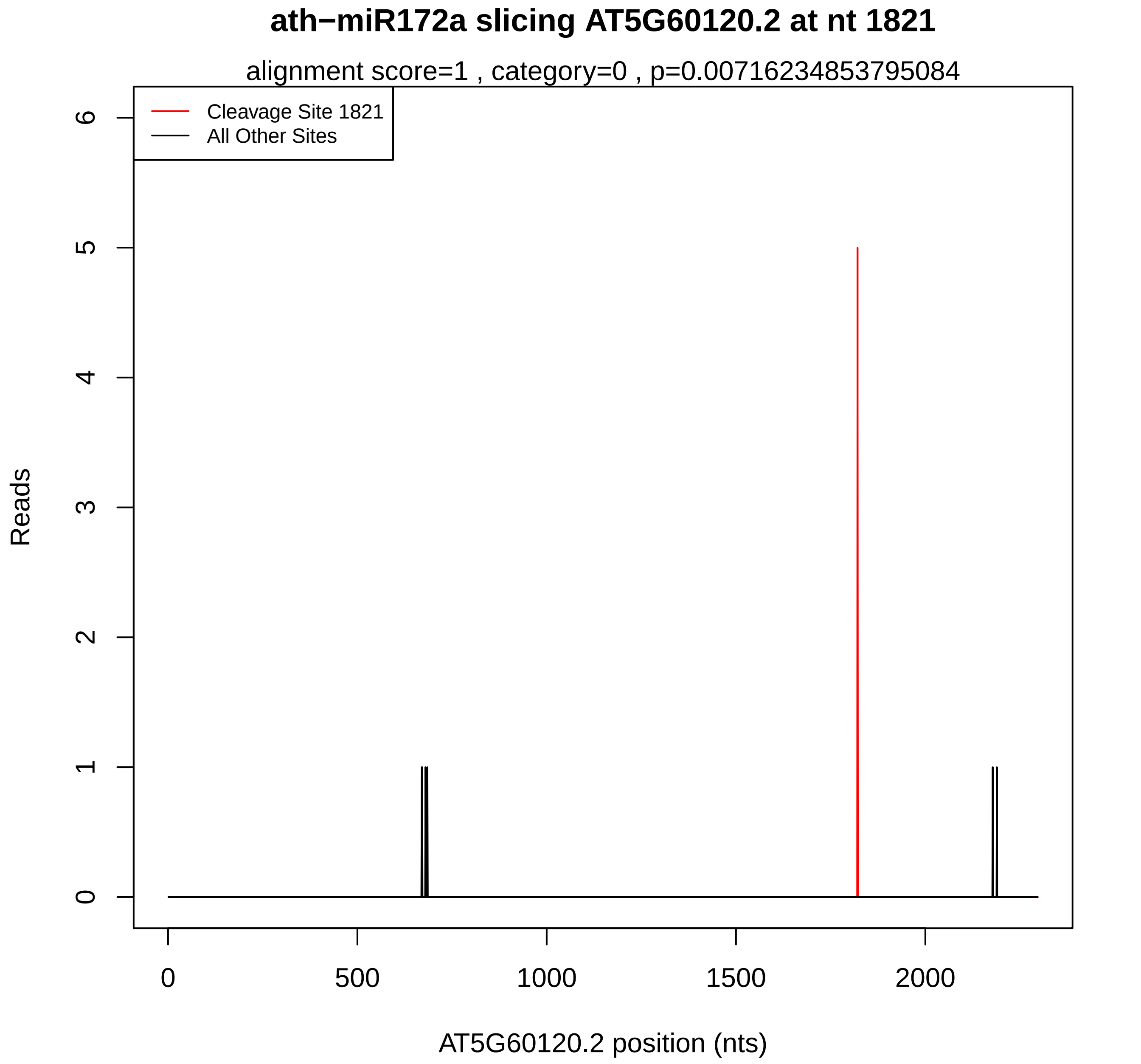
ath−miR172a slicing AT5G60120.2 at nt 1821
alignment score=1 , category=0 , p=0.00716234853795084
Cleavage Site 1821
All Other Sites
Reads
0
1
2
3
4
5
6
0
500
1000
1500
2000
AT5G60120.2 position (nts)

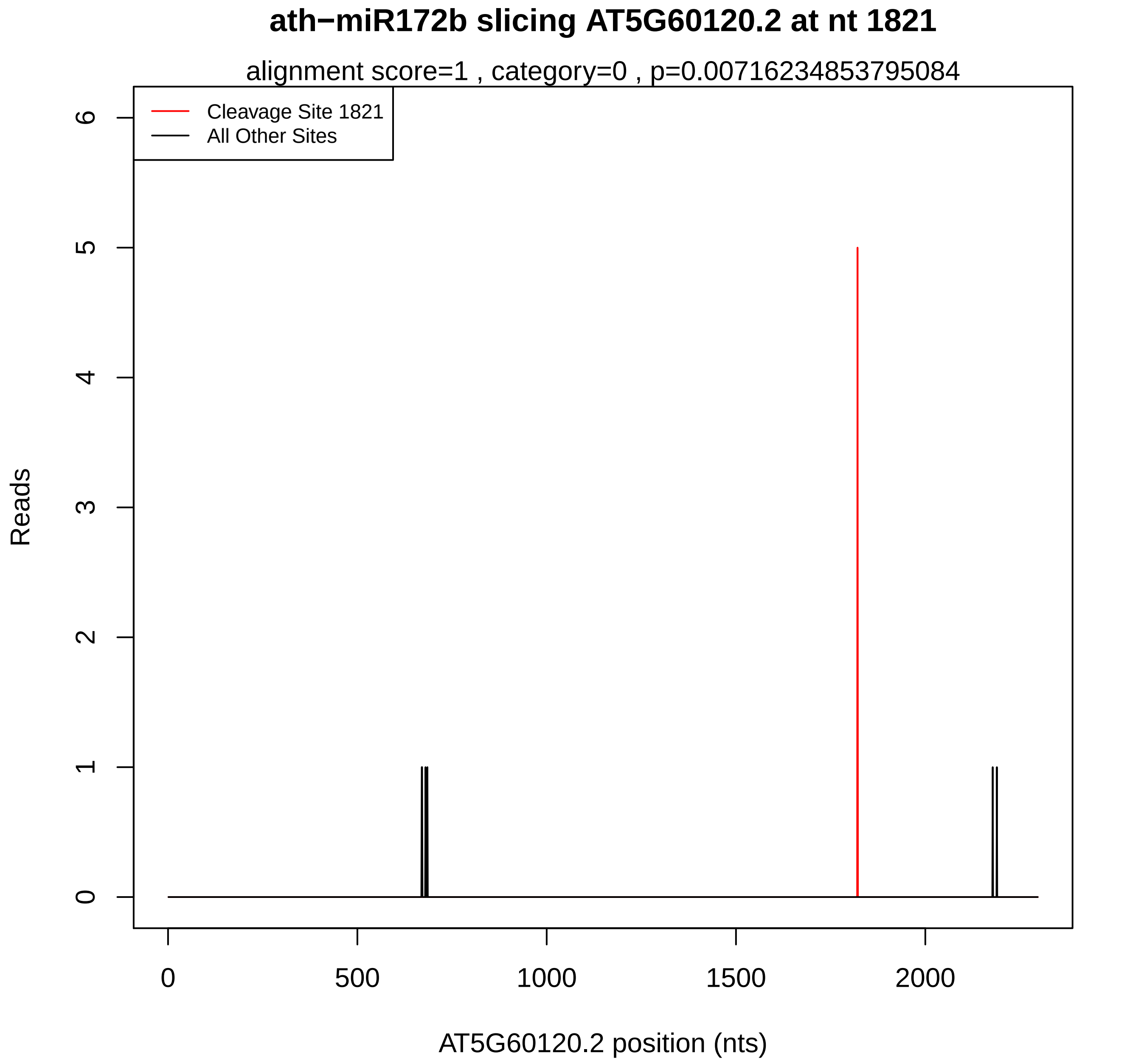
ath−miR172b slicing AT5G60120.2 at nt 1821
alignment score=1 , category=0 , p=0.00716234853795084
Cleavage Site 1821
All Other Sites
Reads
0
1
2
3
4
5
6
0
500
1000
1500
2000
AT5G60120.2 position (nts)

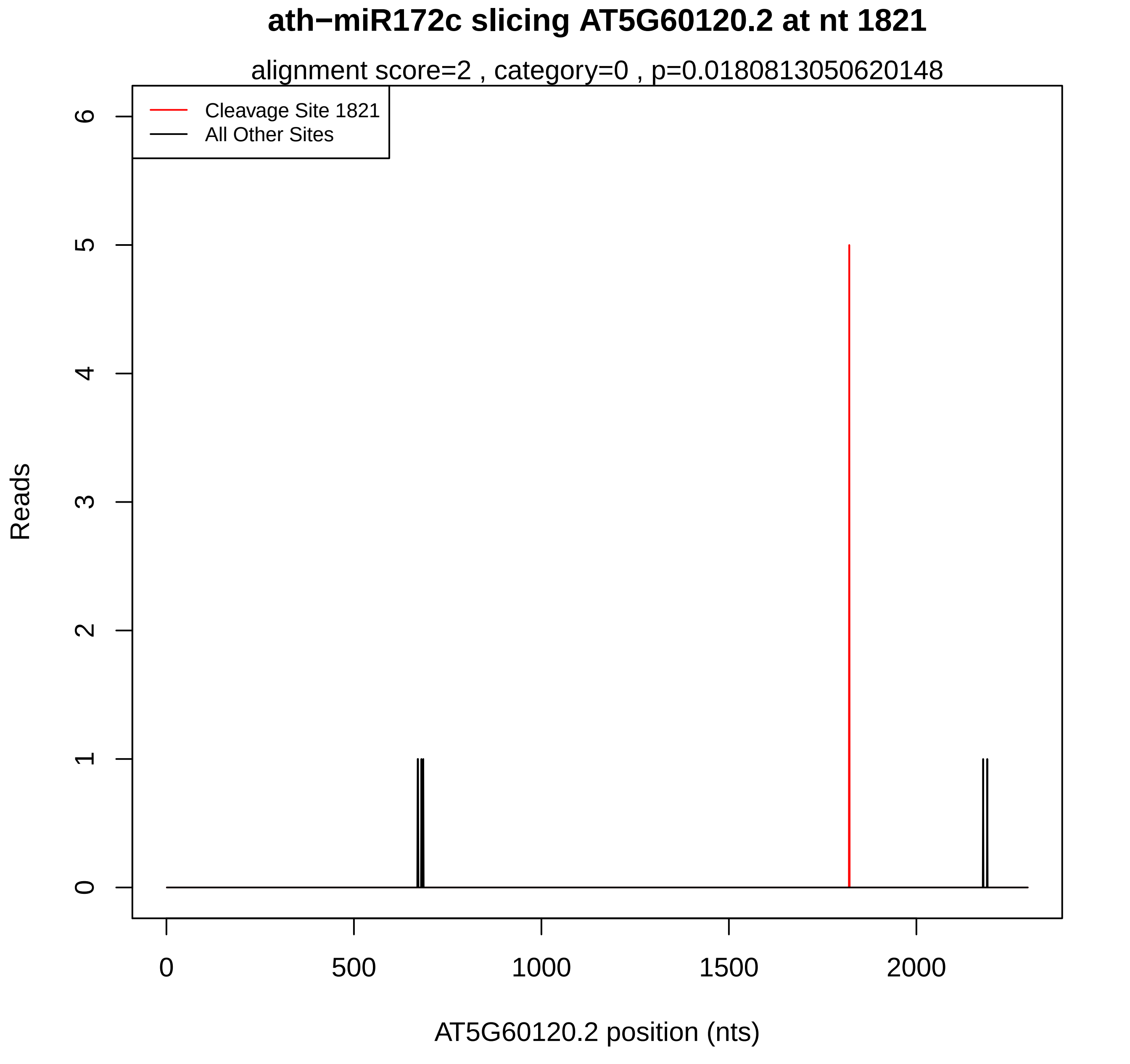
ath−miR172c slicing AT5G60120.2 at nt 1821
alignment score=2 , category=0 , p=0.0180813050620148
Cleavage Site 1821
All Other Sites
Reads
0
1
2
3
4
5
6
0
500
1000
1500
2000
AT5G60120.2 position (nts)

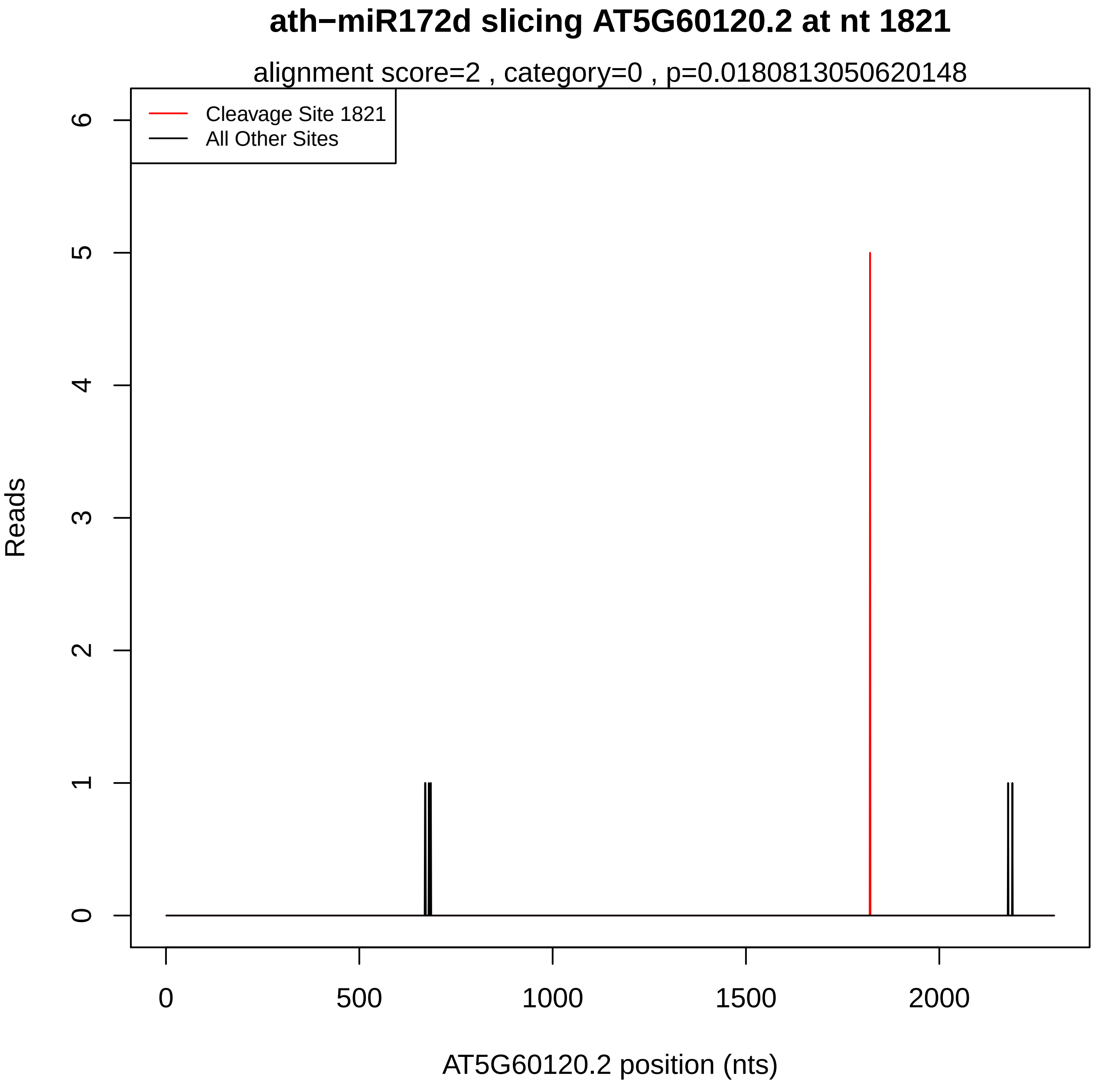

ath−miR172d slicing AT5G60120.2 at nt 1821
alignment score=2 , category=0 , p=0.0180813050620148
Cleavage Site 1821
All Other Sites
Reads
0
1
2
3
4
5
6
0
500
1000
1500
2000
AT5G60120.2 position (nts)

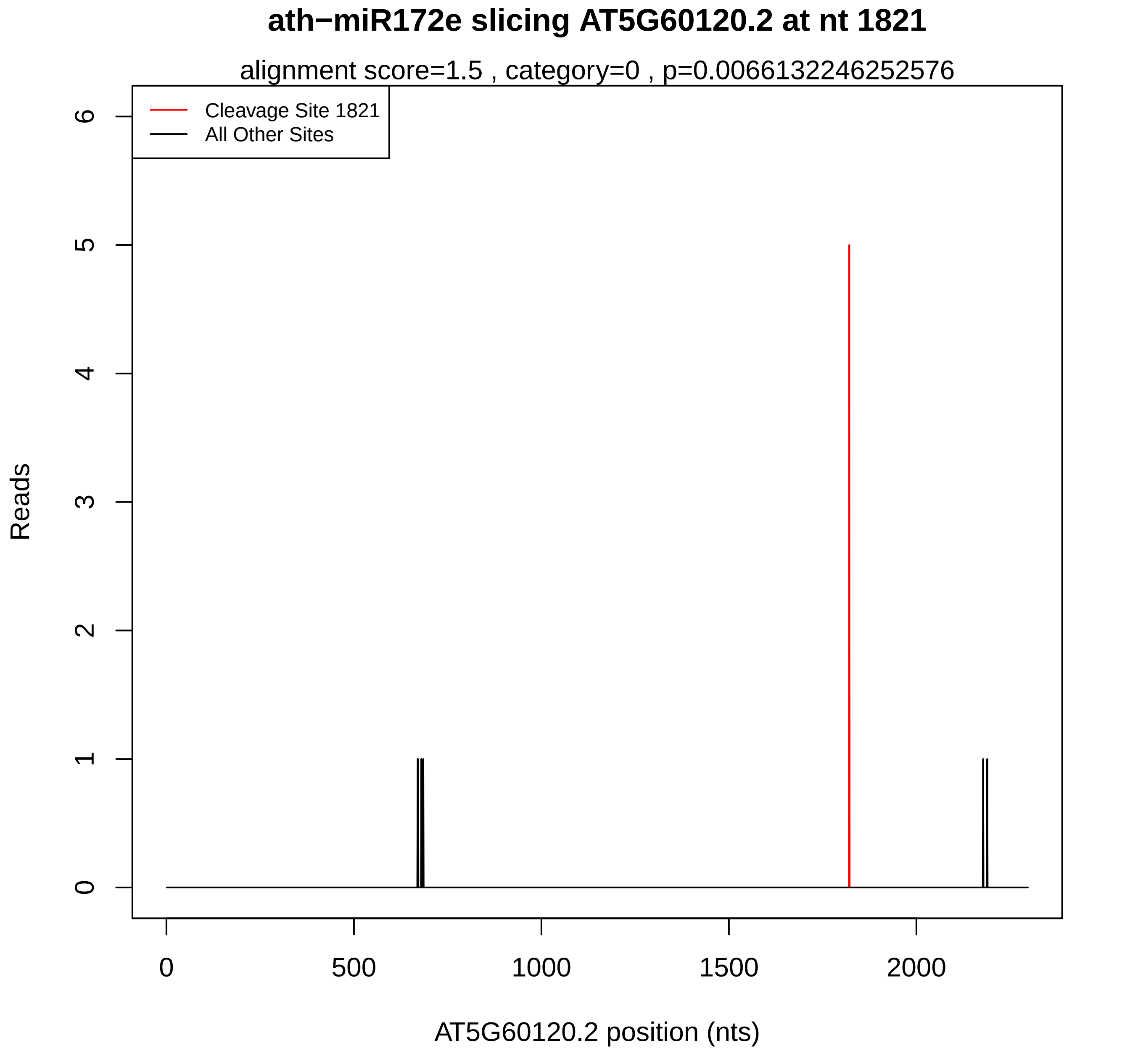
ath−miR172e slicing AT5G60120.2 at nt 1821
alignment score=1.5 , category=0 , p=0.0066132246252576
Cleavage Site 1821
All Other Sites
Reads
0
1
2
3
4
5
6
0
500
1000
1500
2000
AT5G60120.2 position (nts)

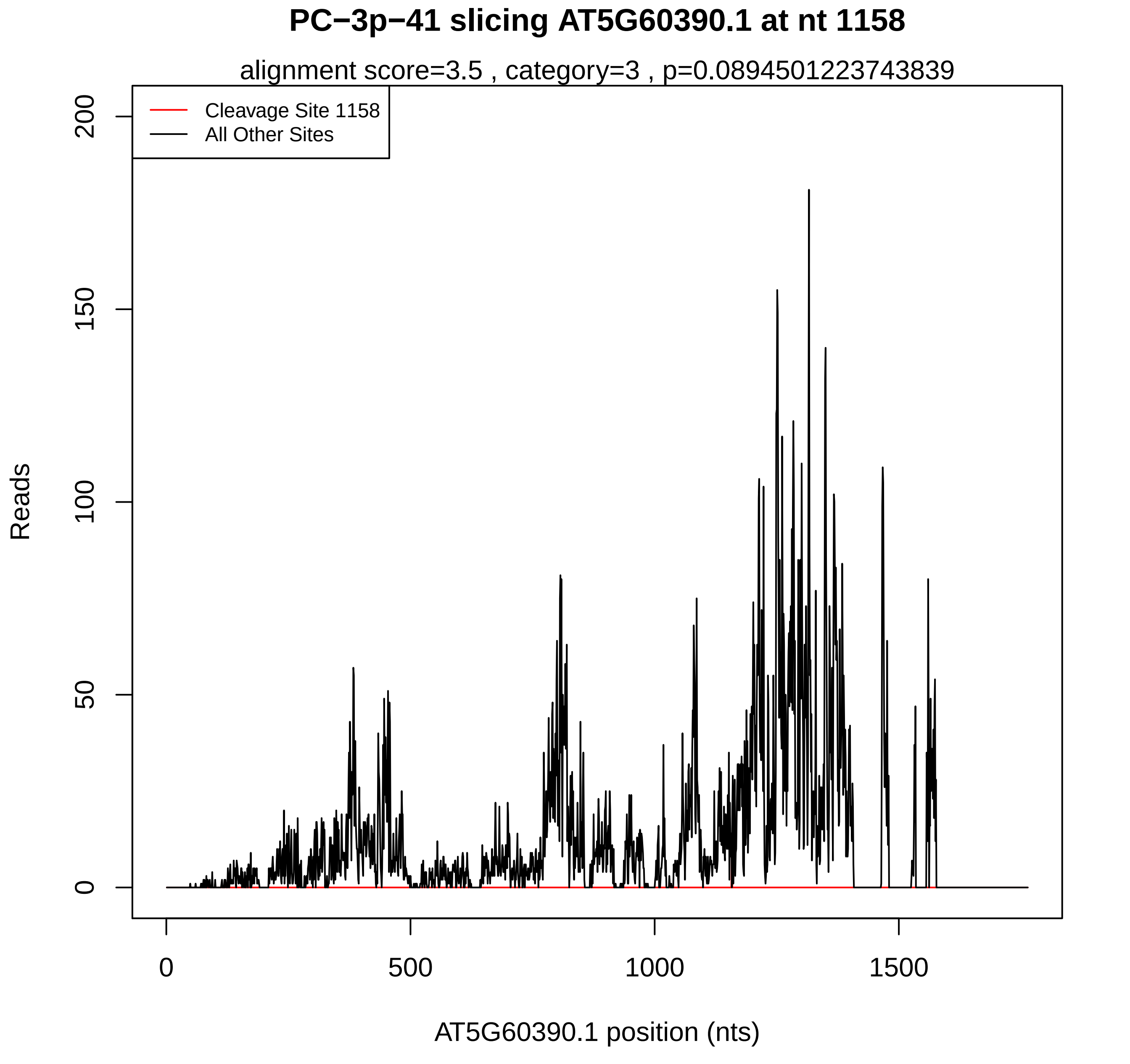
PC−3p−41 slicing AT5G60390.1 at nt 1158
alignment score=3.5 , category=3 , p=0.0894501223743839
Cleavage Site 1158
All Other Sites
Reads
0
50
100
150
200
0
500
1000
1500
AT5G60390.1 position (nts)

PC−3p−41 slicing AT5G60390.2 at nt 1158

alignment score=3.5 , category=3 , p=0.0894501223743839

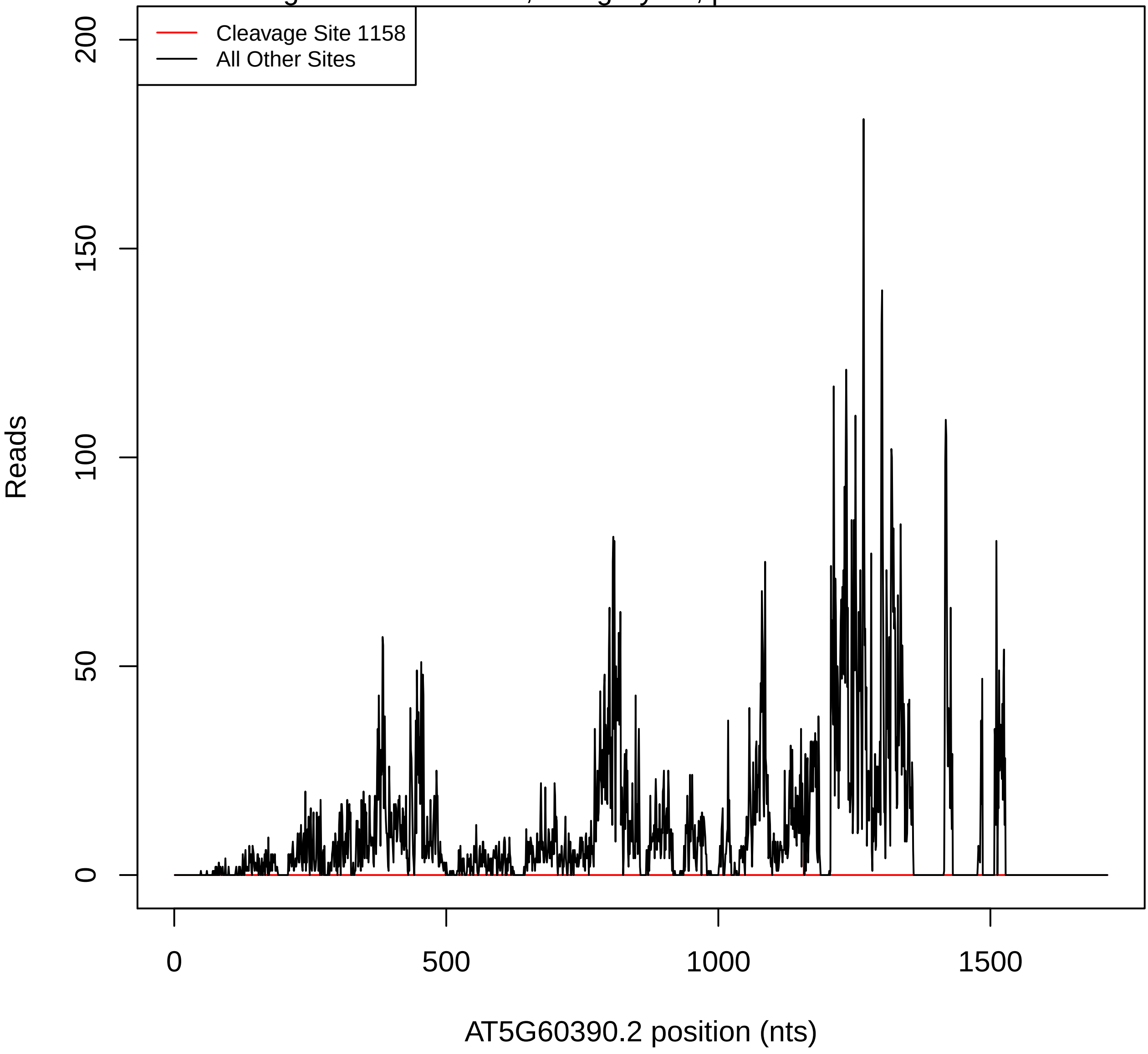

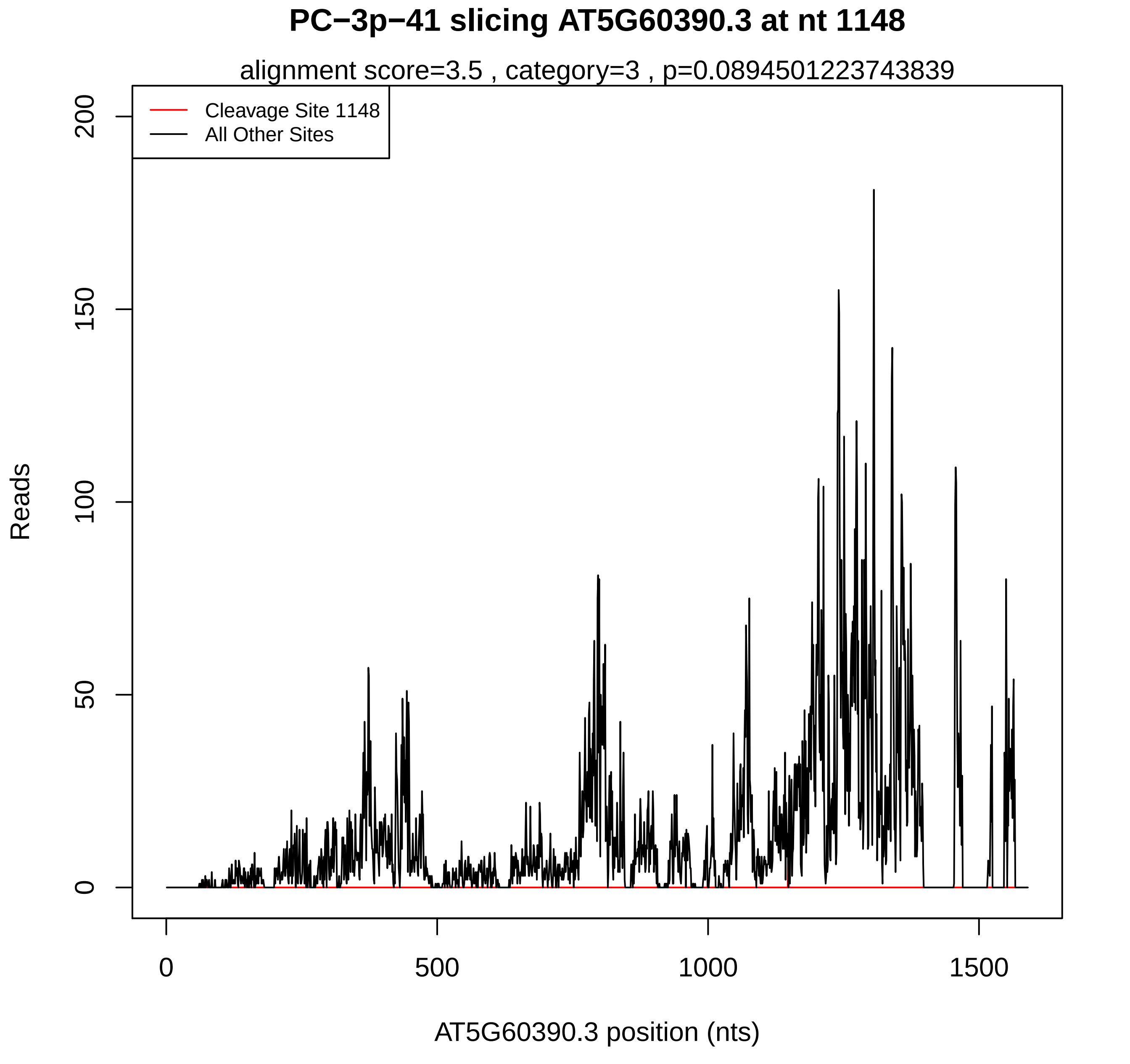

PC−3p−41 slicing AT5G60390.3 at nt 1148
alignment score=3.5 , category=3 , p=0.0894501223743839
Cleavage Site 1148
All Other Sites
Reads
0
50
100
150
200
0
500
1000
1500
AT5G60390.3 position (nts)

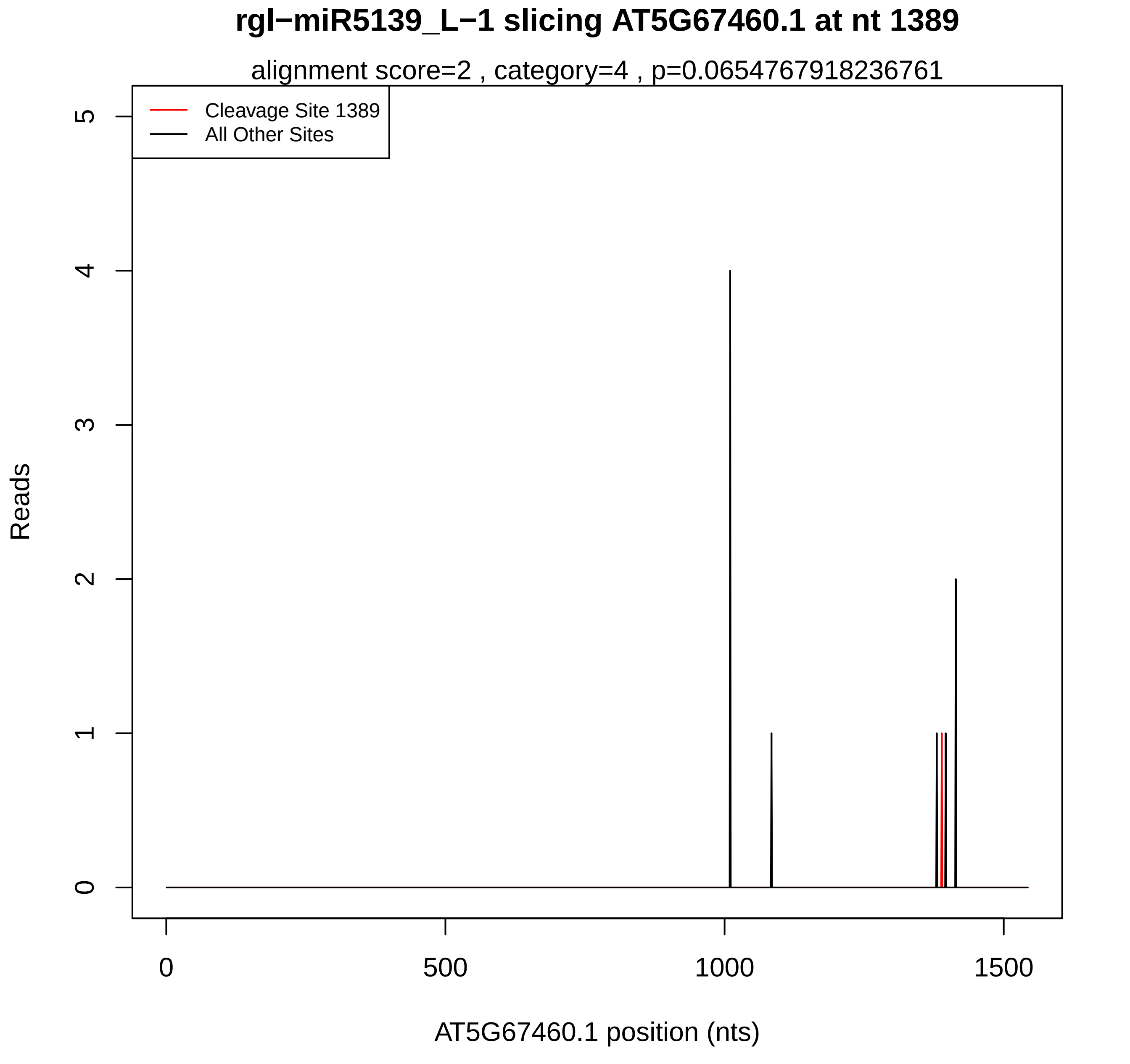
rgl−miR5139_L−1 slicing AT5G67460.1 at nt 1389
alignment score=2 , category=4 , p=0.0654767918236761
Cleavage Site 1389
All Other Sites
Reads
0
1
2
3
4
5
0
500
1000
1500
AT5G67460.1 position (nts)
