# Supplementary material for: Identification of miRNAs and their targets using high-throughput sequencing and degradome analysis in cytoplasmic male-sterile and its maintainer fertile lines of brassica juncea
Source: BMC Genomics. 2013 Jan 16;14:9. doi: 10.1186/1471-2164-14-9 (PMC3553062; doi:10.1186/1471-2164-14-9)
Supplement: Additional file 4 — Figure S3. Target plot (t-plot) of representative validated miRNAs target in CMS of Brassica juncea. [file 1471-2164-14-9-S4.pdf]

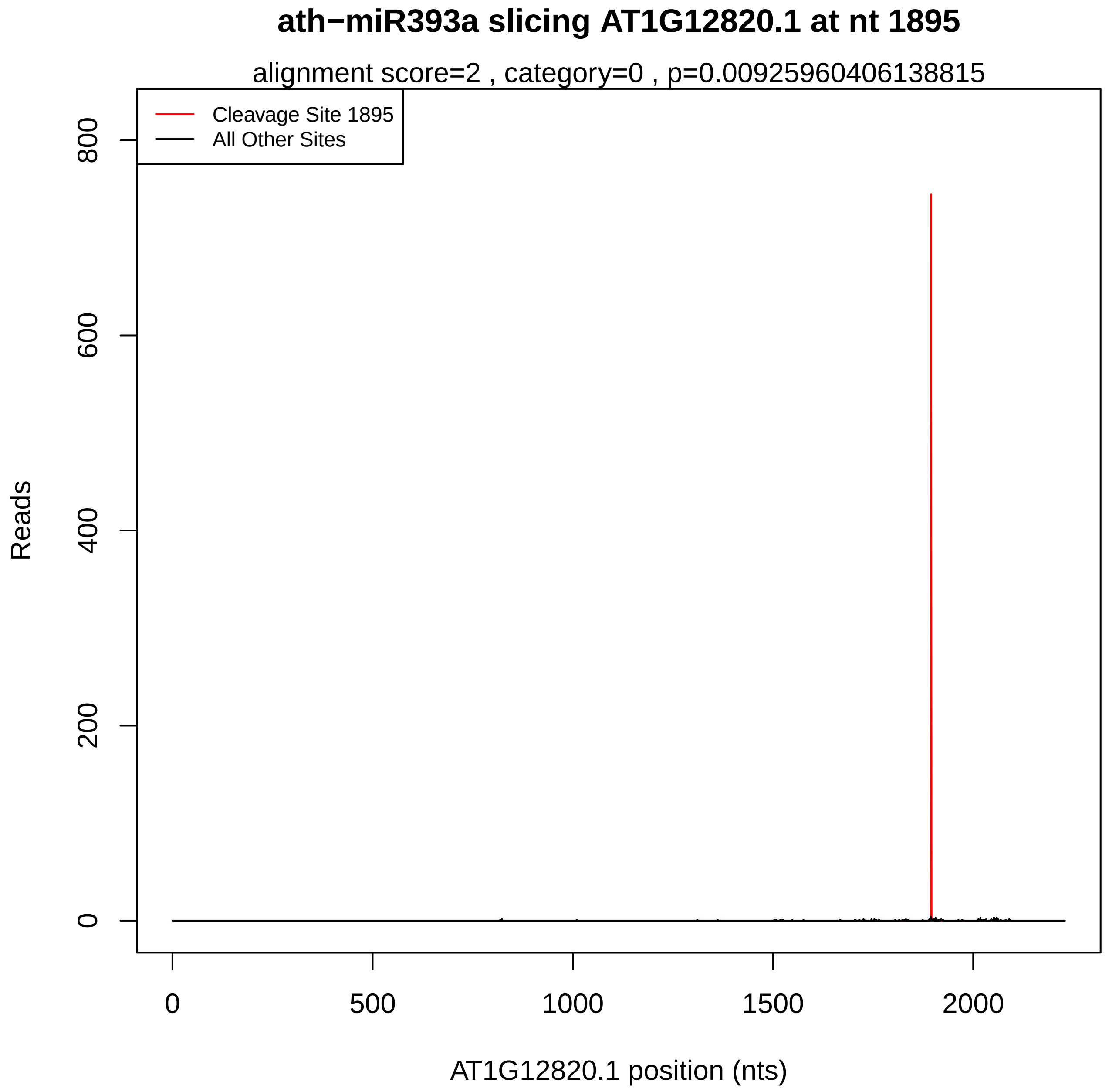

ath−miR393a slicing AT1G12820.1 at nt 1895
alignment score=2 , category=0 , p=0.00925960406138815
Cleavage Site 1895
All Other Sites
Reads
0
200
400
600
800
0
500
1000
1500
2000
AT1G12820.1 position (nts)

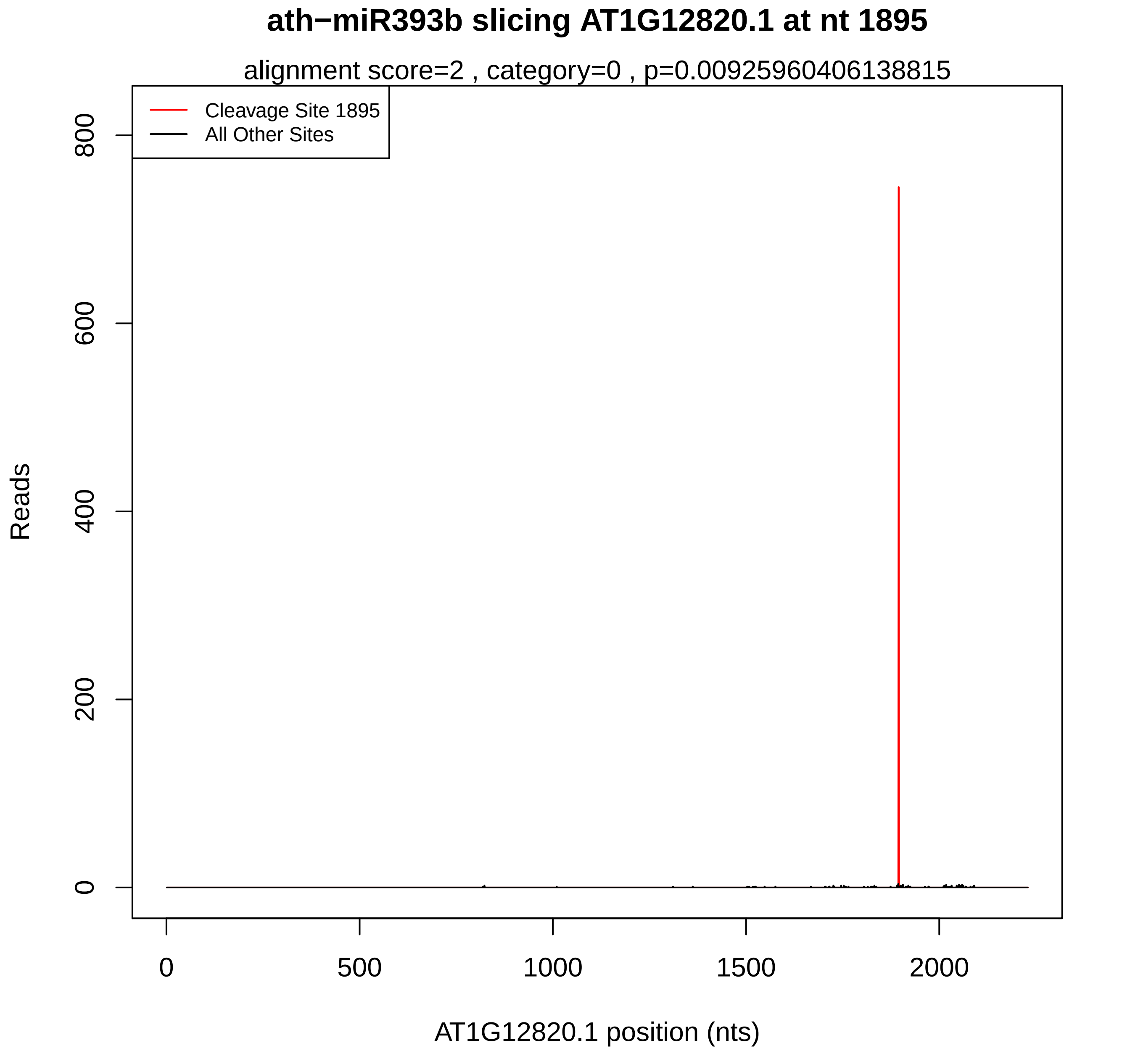
ath−miR393b slicing AT1G12820.1 at nt 1895
alignment score=2 , category=0 , p=0.00925960406138815
Cleavage Site 1895
All Other Sites
Reads
0
200
400
600
800
0
500
1000
1500
2000
AT1G12820.1 position (nts)

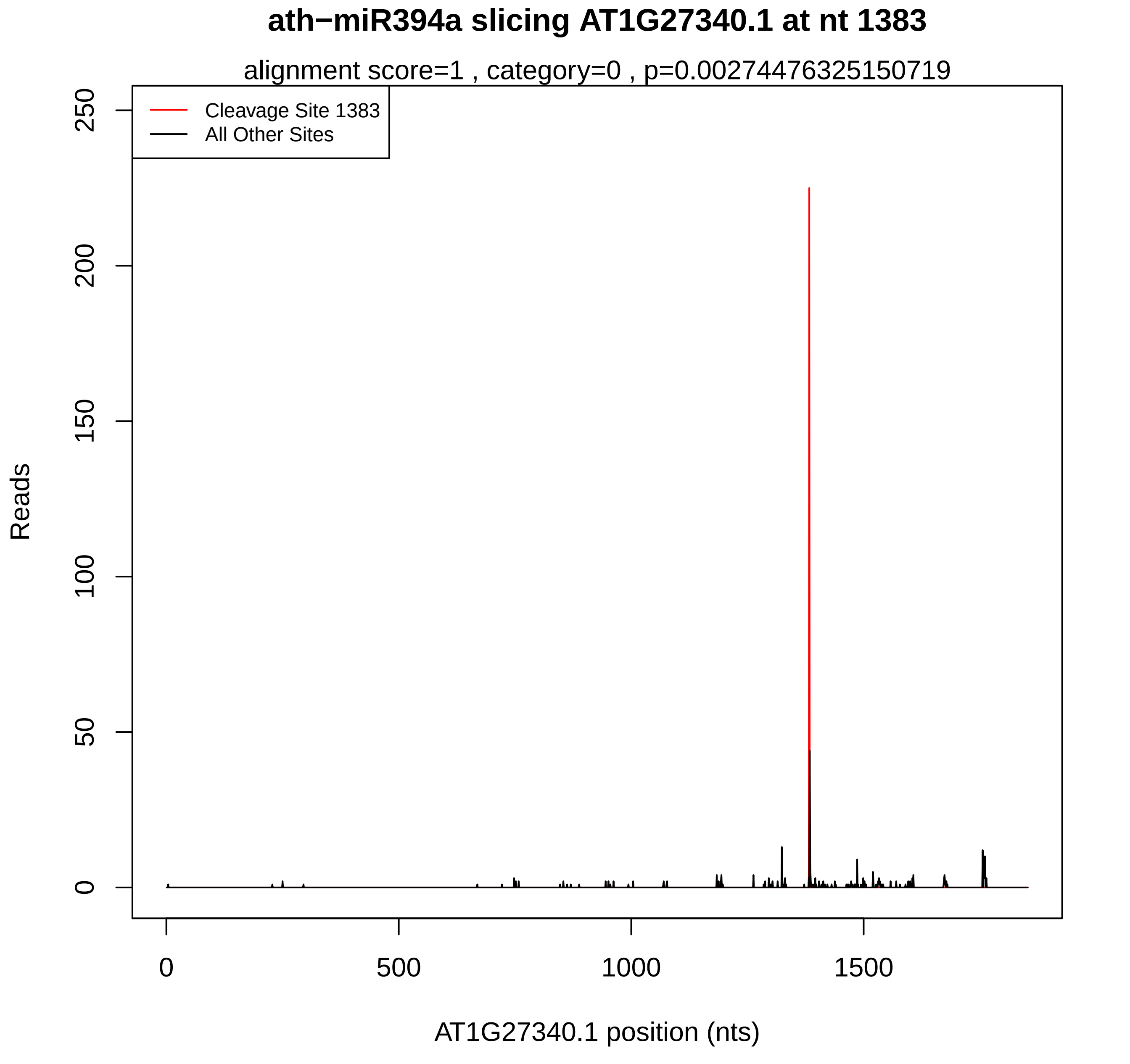
ath−miR394a slicing AT1G27340.1 at nt 1383
alignment score=1 , category=0 , p=0.00274476325150719
Cleavage Site 1383
All Other Sites
Reads
0
50
100
150
200
250
0
500
1000
1500
AT1G27340.1 position (nts)

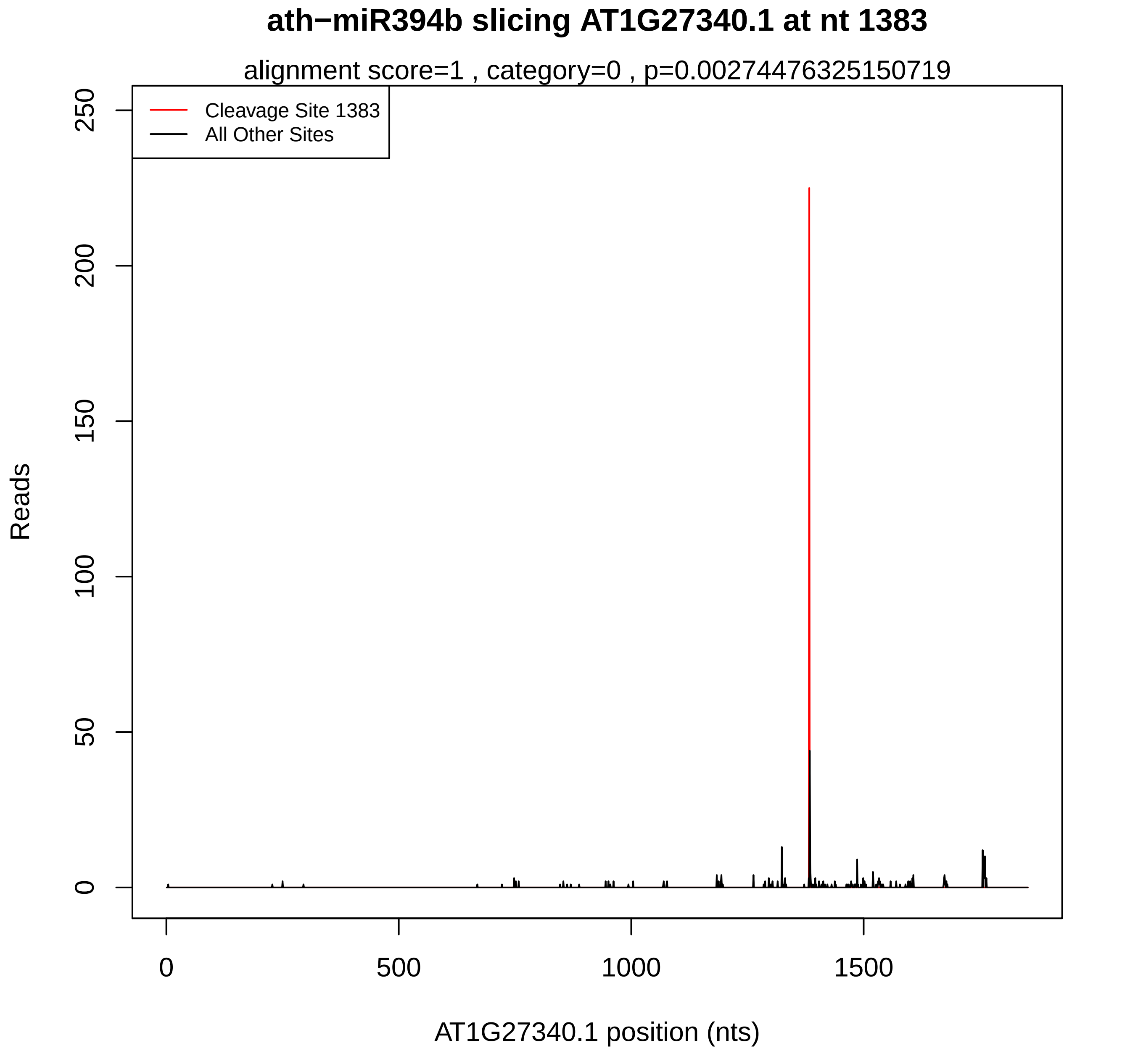

ath−miR394b slicing AT1G27340.1 at nt 1383
alignment score=1 , category=0 , p=0.00274476325150719
Cleavage Site 1383
All Other Sites
Reads
0
50
100
150
200
250
0
500
1000
1500
AT1G27340.1 position (nts)

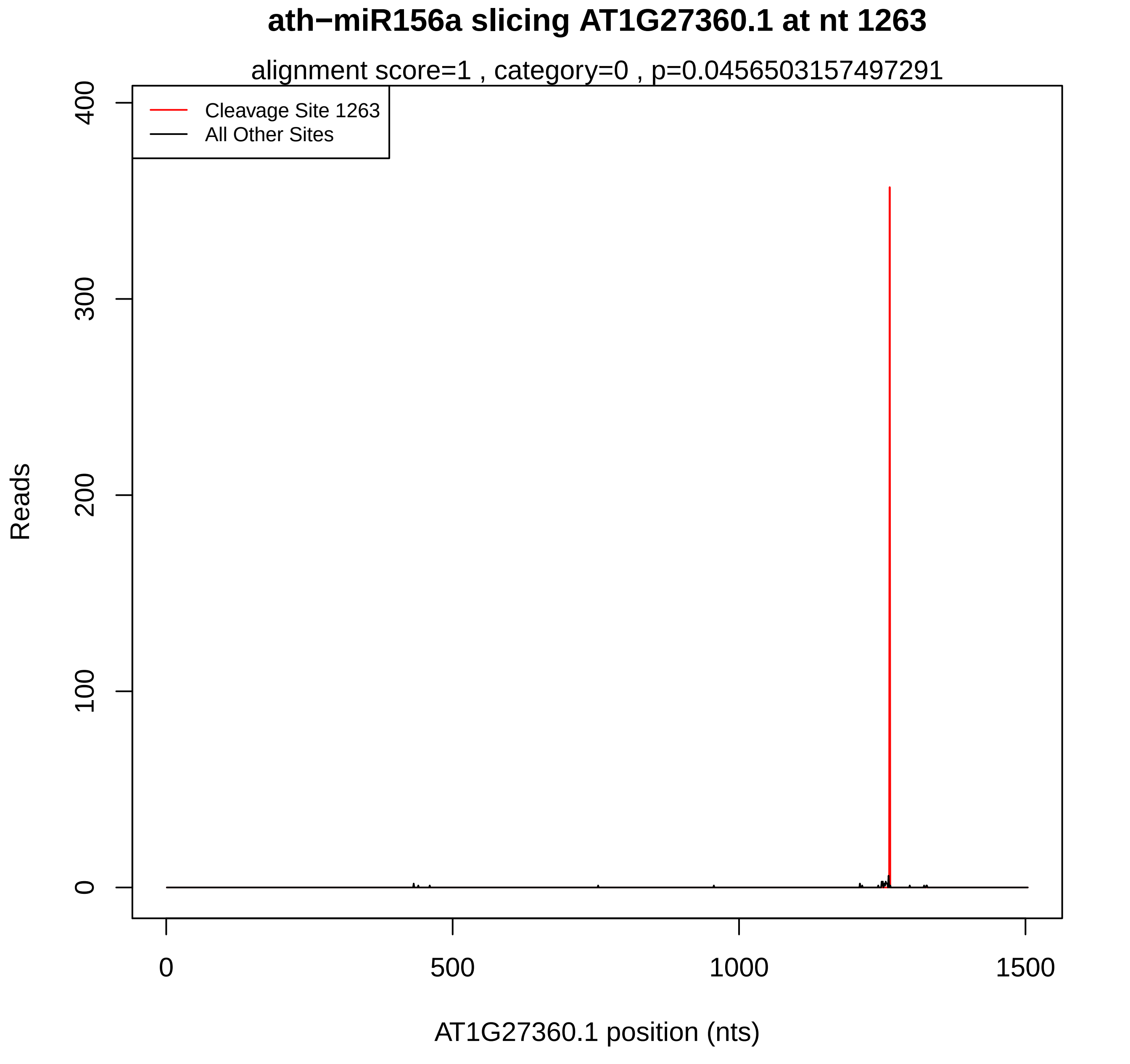
ath−miR156a slicing AT1G27360.1 at nt 1263
alignment score=1 , category=0 , p=0.0456503157497291
Cleavage Site 1263
All Other Sites
Reads
0
100
200
300
400
0
500
1000
1500
AT1G27360.1 position (nts)

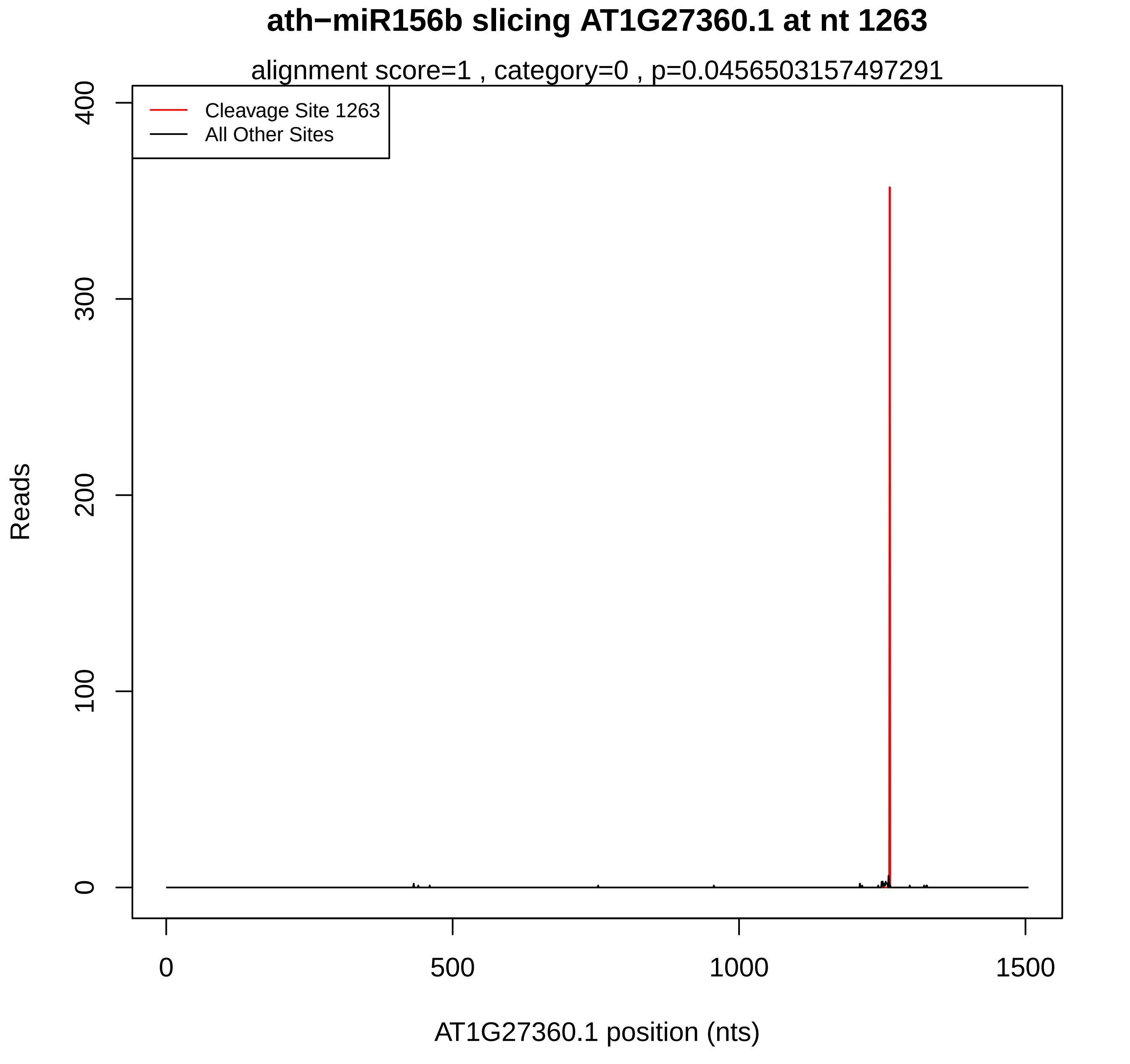
ath−miR156b slicing AT1G27360.1 at nt 1263
alignment score=1 , category=0 , p=0.0456503157497291
Cleavage Site 1263
All Other Sites
Reads
0
100
200
300
400
0
500
1000
1500
AT1G27360.1 position (nts)

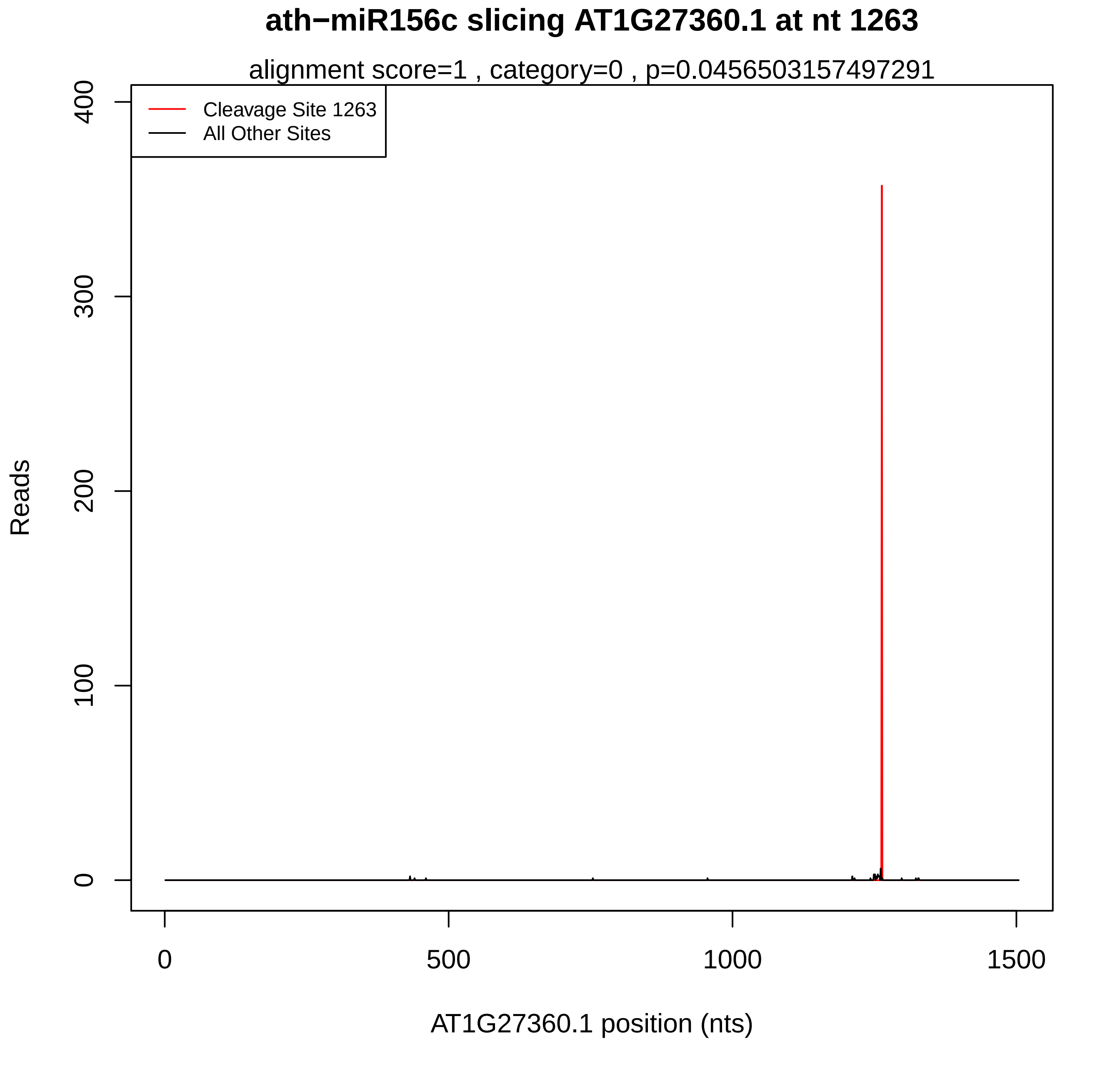
ath−miR156c slicing AT1G27360.1 at nt 1263
alignment score=1 , category=0 , p=0.0456503157497291
Cleavage Site 1263
All Other Sites
Reads
0
100
200
300
400
0
500
1000
1500
AT1G27360.1 position (nts)

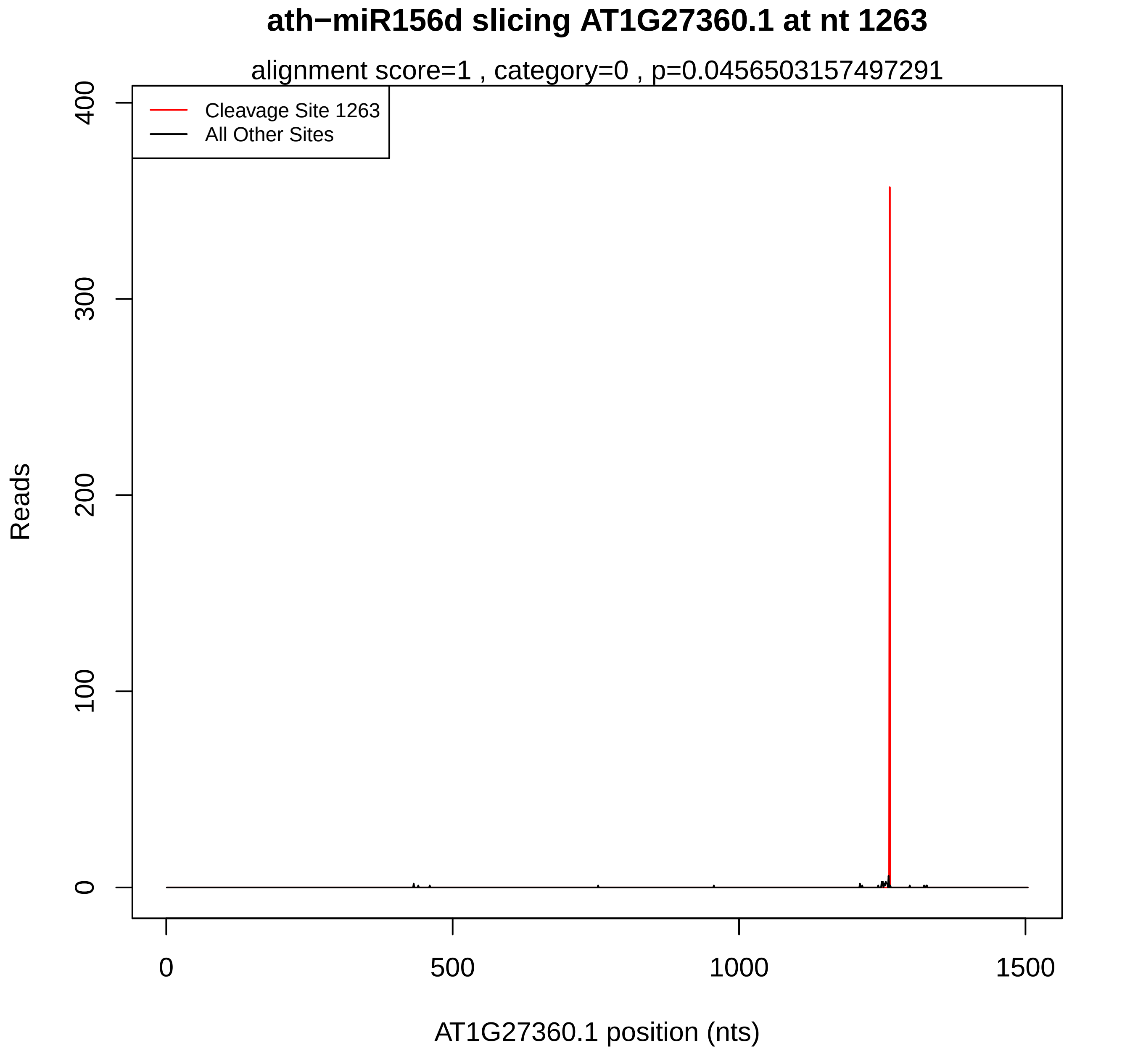
ath−miR156d slicing AT1G27360.1 at nt 1263
alignment score=1 , category=0 , p=0.0456503157497291
Cleavage Site 1263
All Other Sites
Reads
0
100
200
300
400
0
500
1000
1500
AT1G27360.1 position (nts)

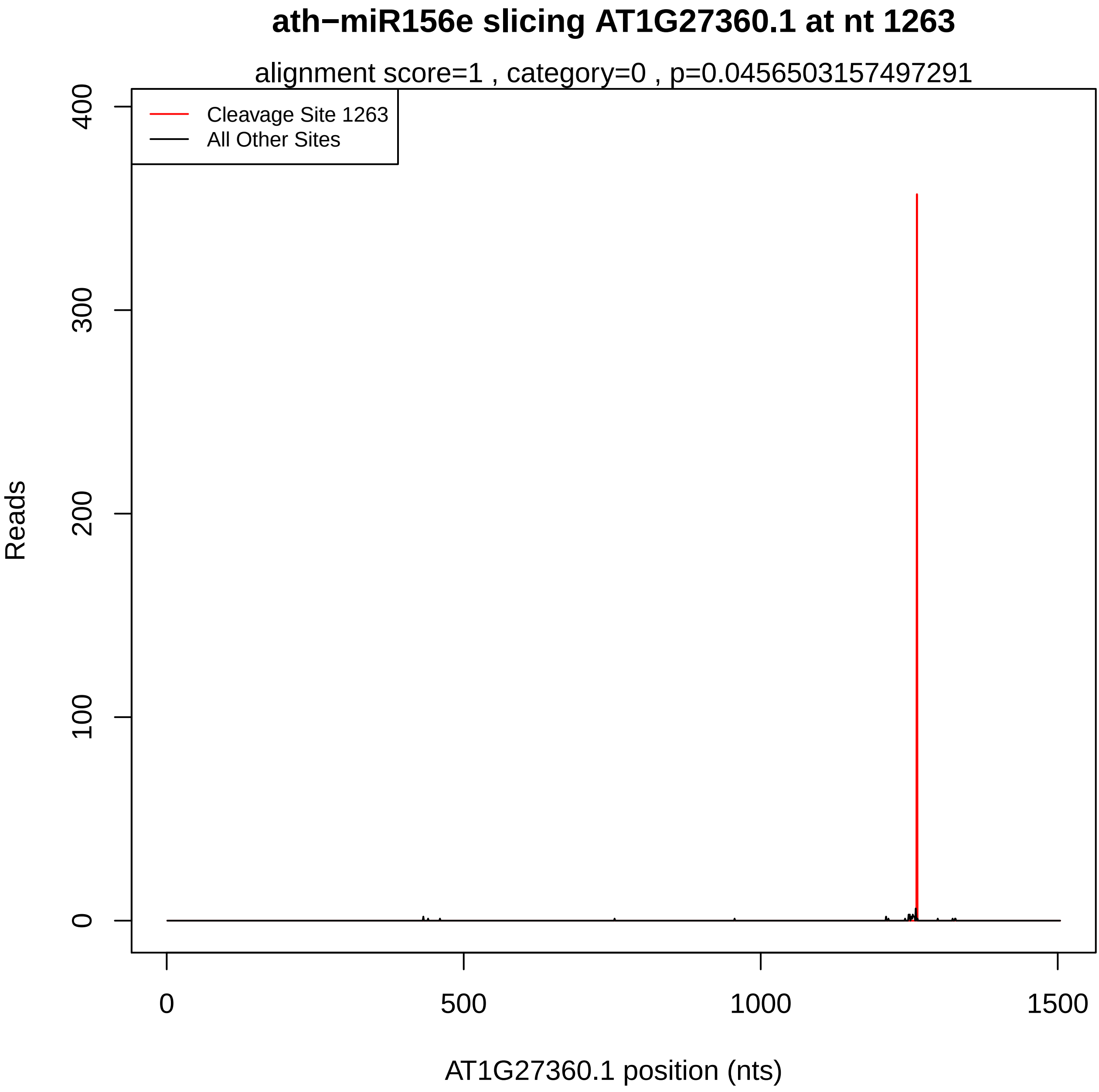
ath−miR156e slicing AT1G27360.1 at nt 1263
alignment score=1 , category=0 , p=0.0456503157497291
Cleavage Site 1263
All Other Sites
Reads
0
100
200
300
400
0
500
1000
1500
AT1G27360.1 position (nts)

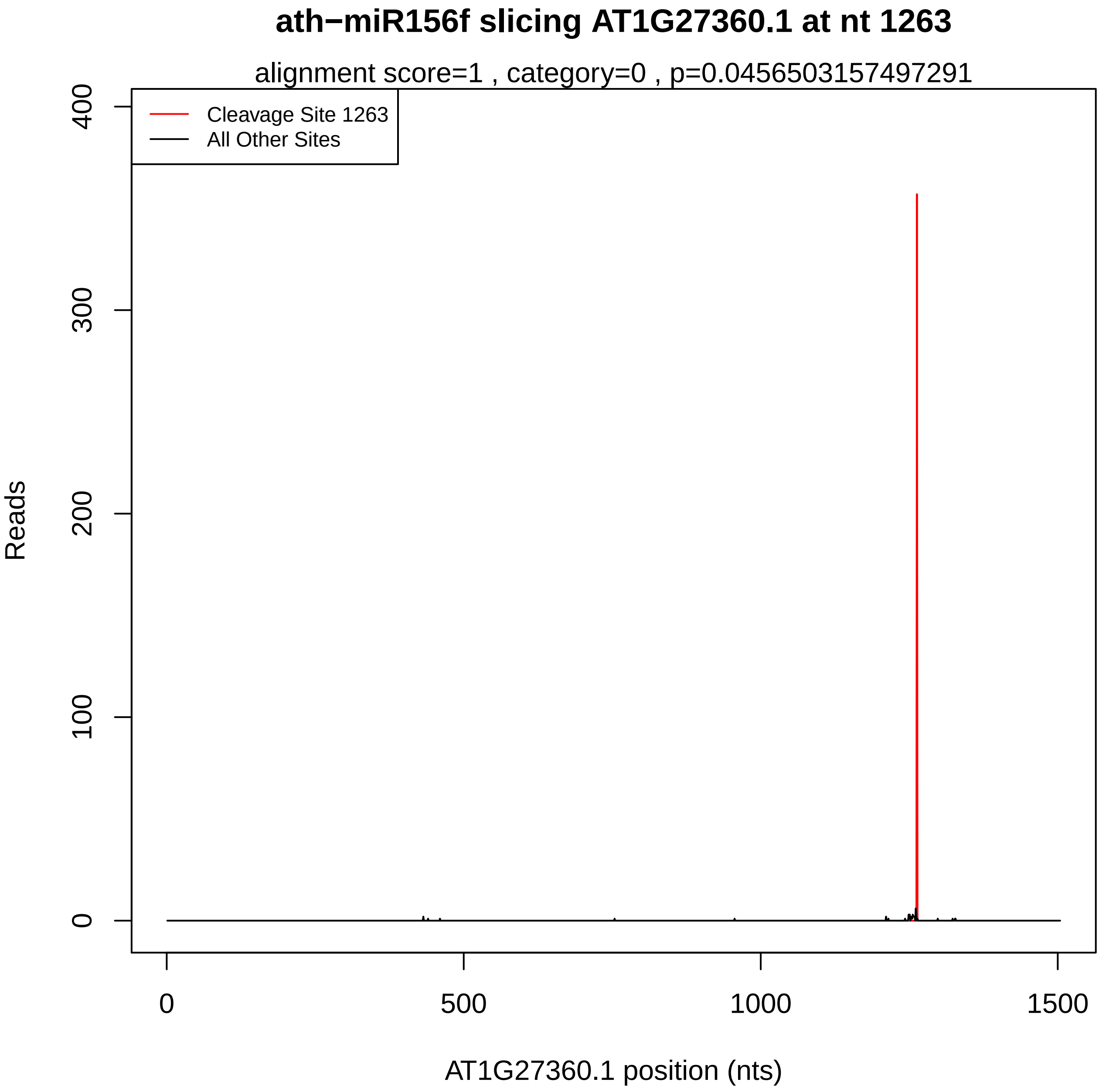

ath−miR156f slicing AT1G27360.1 at nt 1263
alignment score=1 , category=0 , p=0.0456503157497291
Cleavage Site 1263
All Other Sites
Reads
0
100
200
300
400
0
500
1000
1500
AT1G27360.1 position (nts)

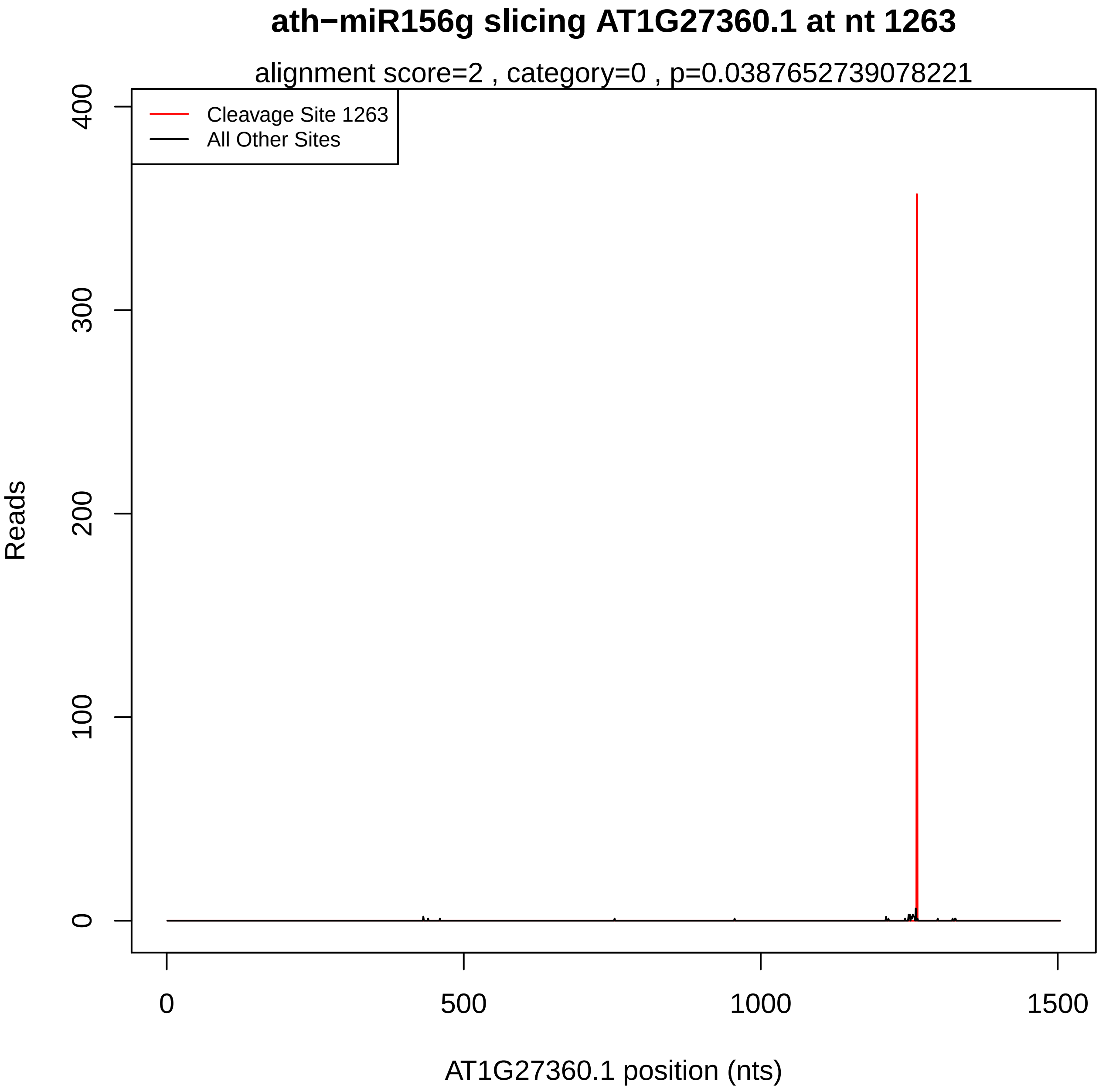

ath−miR156g slicing AT1G27360.1 at nt 1263
alignment score=2 , category=0 , p=0.0387652739078221
Cleavage Site 1263
All Other Sites
Reads
0
100
200
300
400
0
500
1000
1500
AT1G27360.1 position (nts)

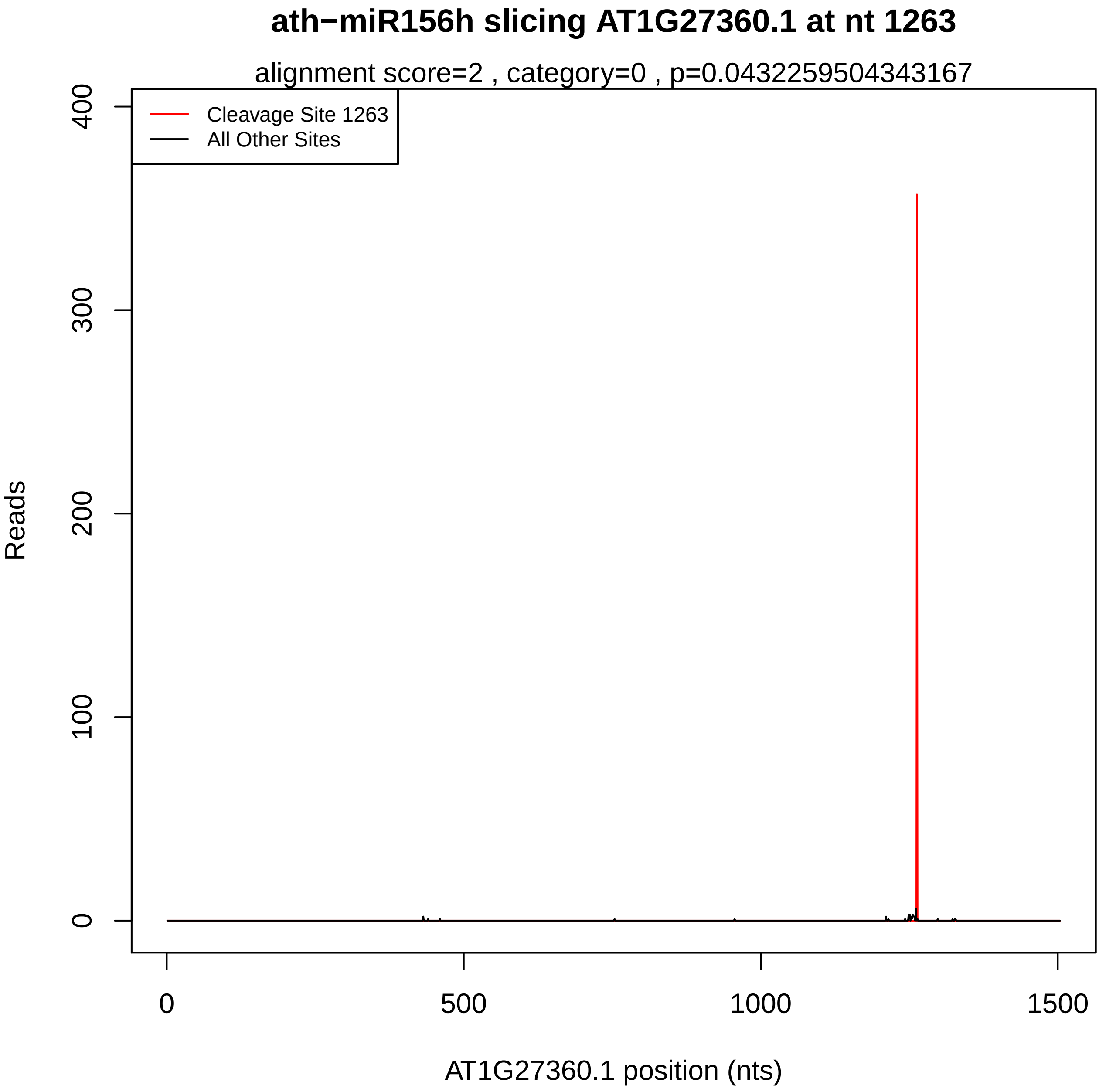
ath−miR156h slicing AT1G27360.1 at nt 1263
alignment score=2 , category=0 , p=0.0432259504343167
Cleavage Site 1263
All Other Sites
Reads
0
100
200
300
400
0
500
1000
1500
AT1G27360.1 position (nts)

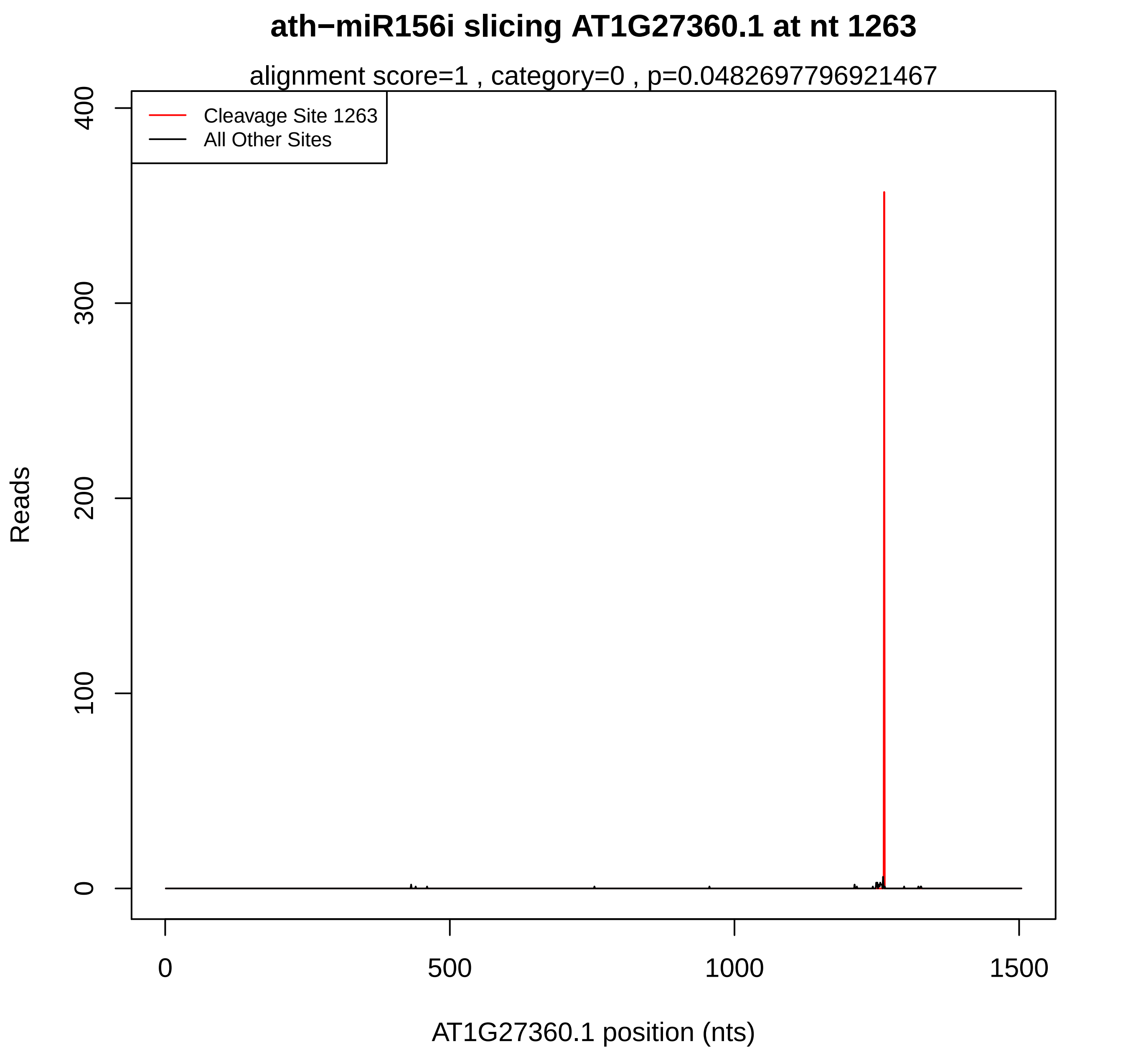
ath−miR156i slicing AT1G27360.1 at nt 1263
alignment score=1 , category=0 , p=0.0482697796921467
Cleavage Site 1263
All Other Sites
Reads
0
100
200
300
400
0
500
1000
1500
AT1G27360.1 position (nts)

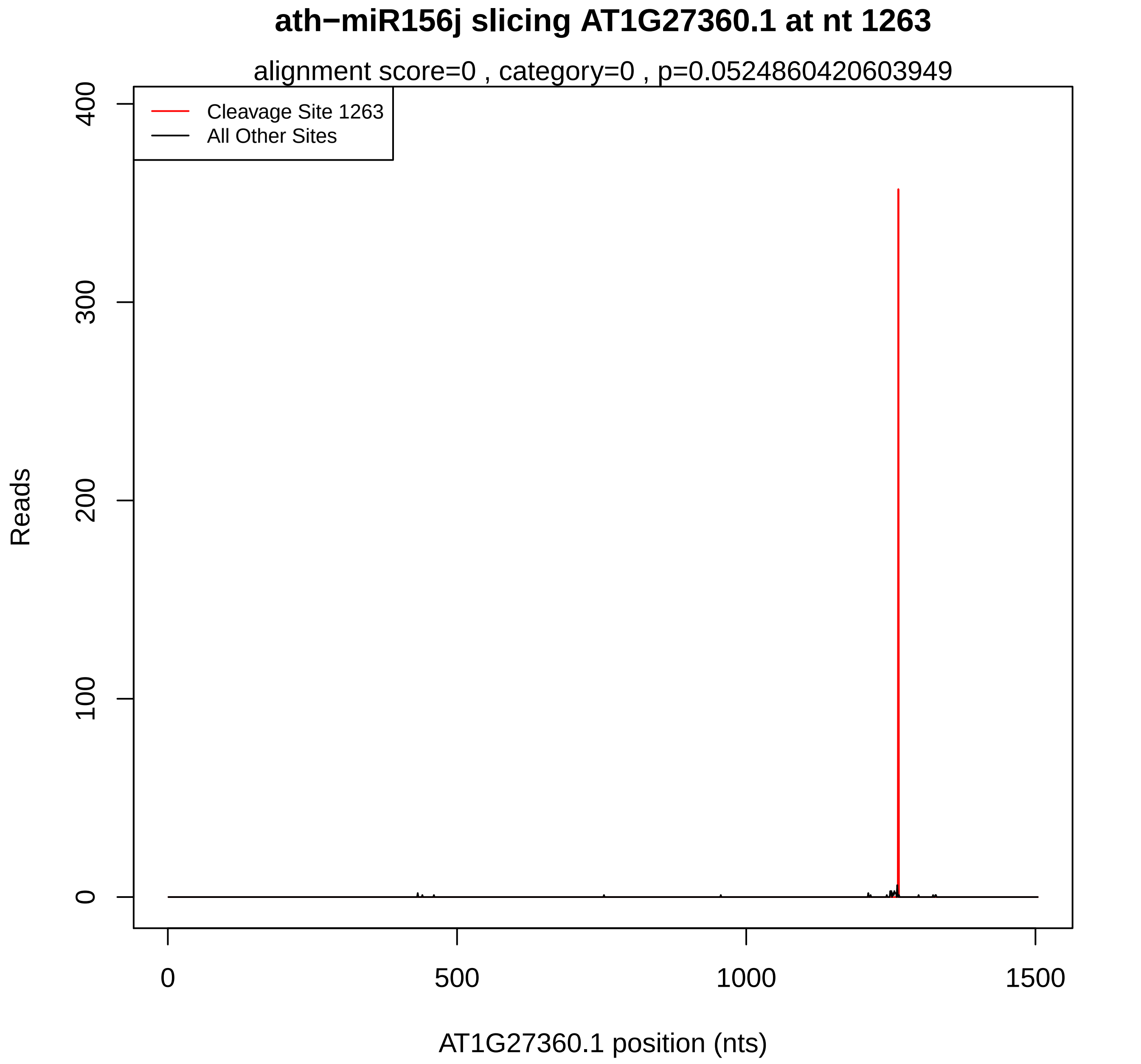
ath−miR156j slicing AT1G27360.1 at nt 1263
alignment score=0 , category=0 , p=0.0524860420603949
Cleavage Site 1263
All Other Sites
Reads
0
100
200
300
400
0
500
1000
1500
AT1G27360.1 position (nts)

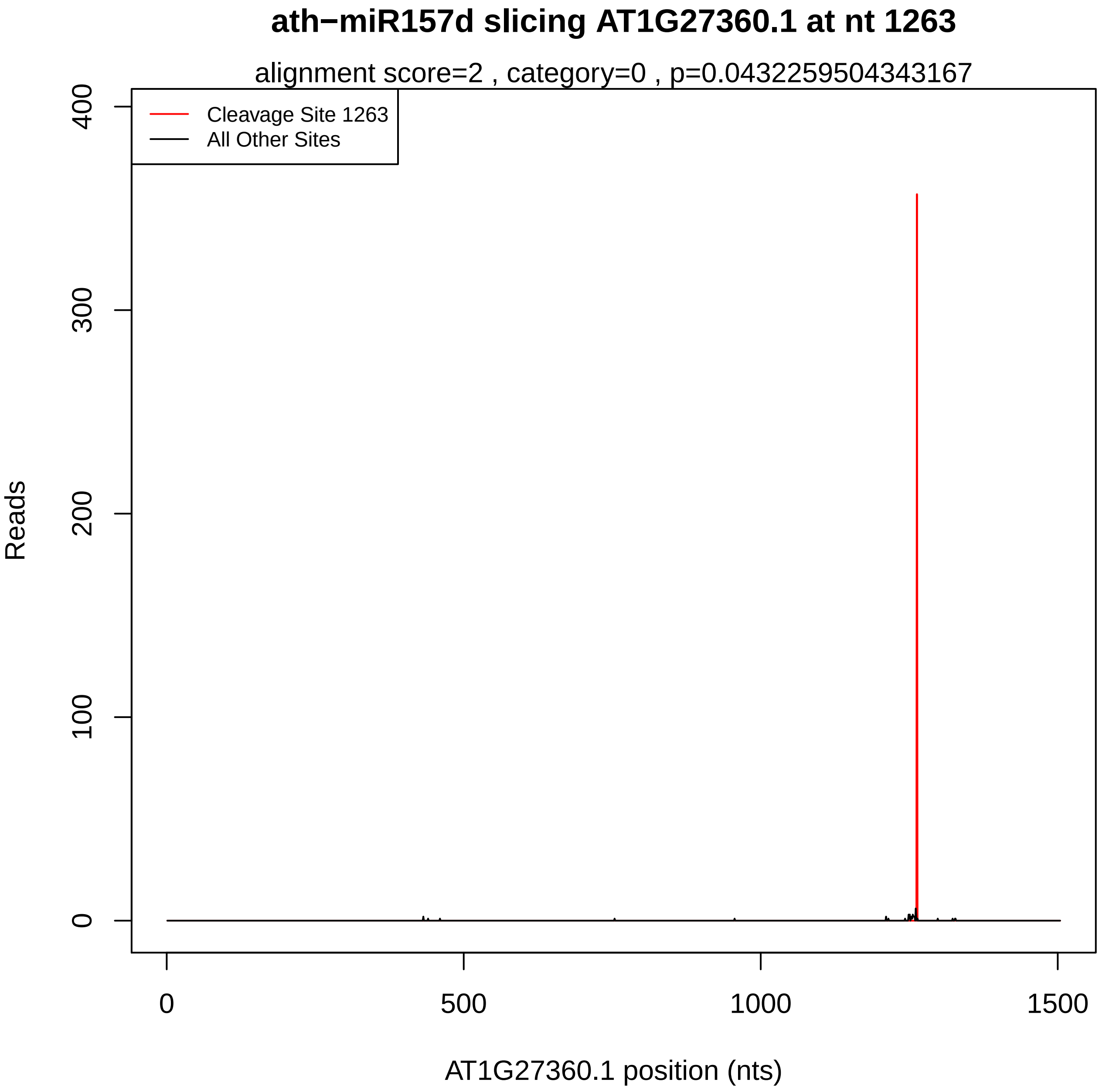
ath−miR157d slicing AT1G27360.1 at nt 1263
alignment score=2 , category=0 , p=0.0432259504343167
Cleavage Site 1263
All Other Sites
Reads
0
100
200
300
400
0
500
1000
1500
AT1G27360.1 position (nts)

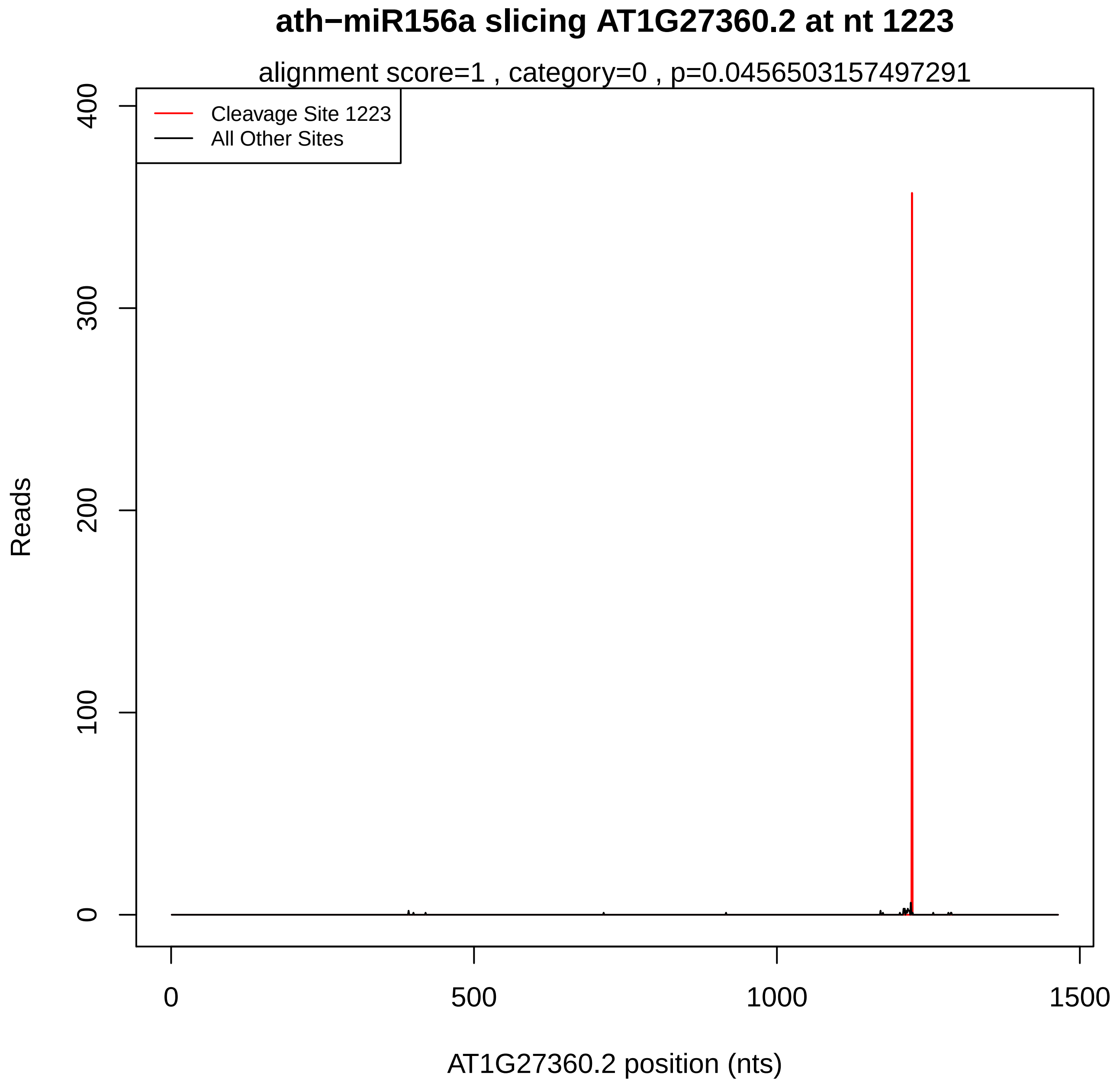
ath−miR156a slicing AT1G27360.2 at nt 1223
alignment score=1 , category=0 , p=0.0456503157497291
Cleavage Site 1223
All Other Sites
Reads
0
100
200
300
400
0
500
1000
1500
AT1G27360.2 position (nts)

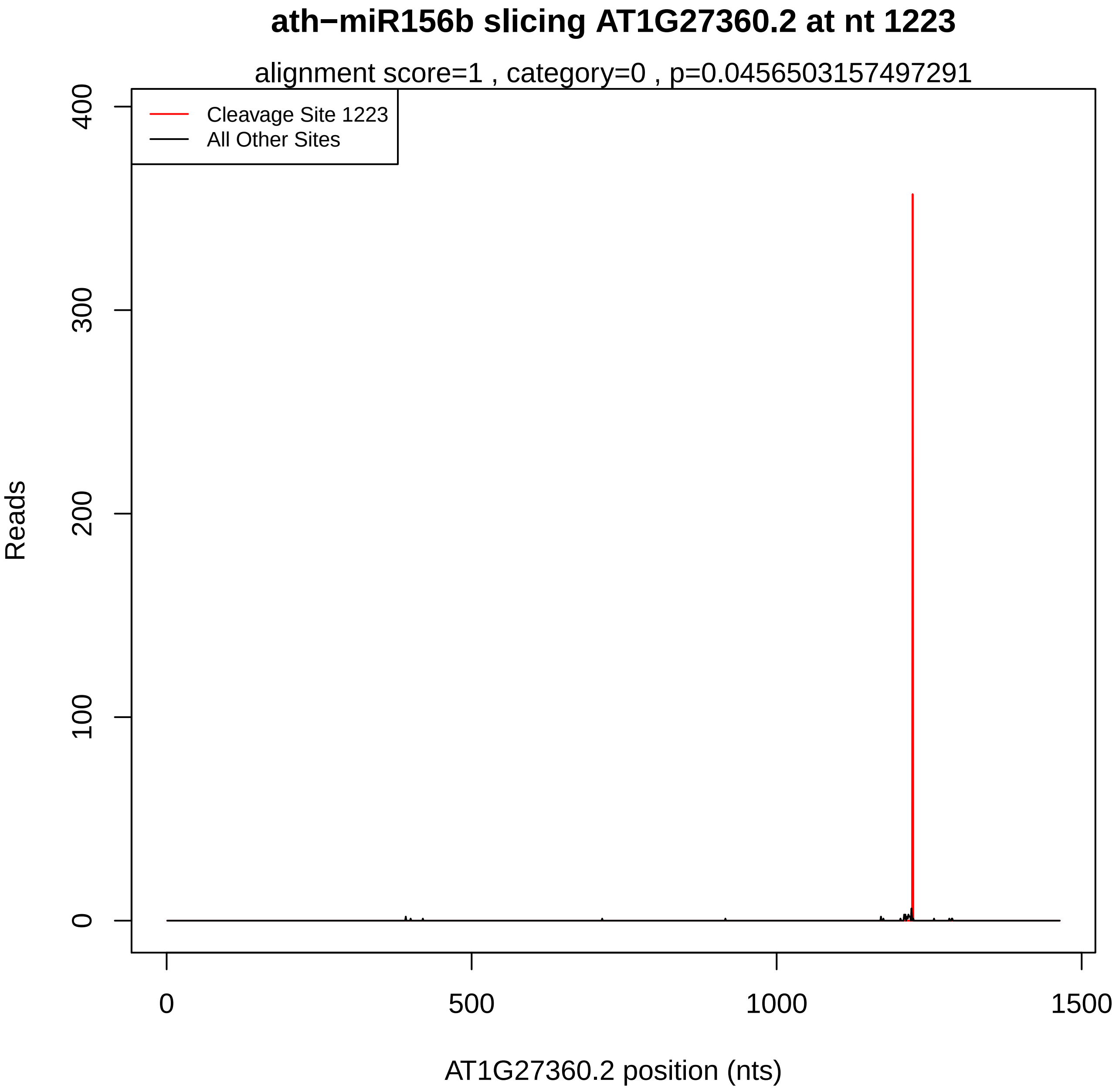
ath−miR156b slicing AT1G27360.2 at nt 1223
alignment score=1 , category=0 , p=0.0456503157497291
Cleavage Site 1223
All Other Sites
Reads
0
100
200
300
400
0
500
1000
1500
AT1G27360.2 position (nts)

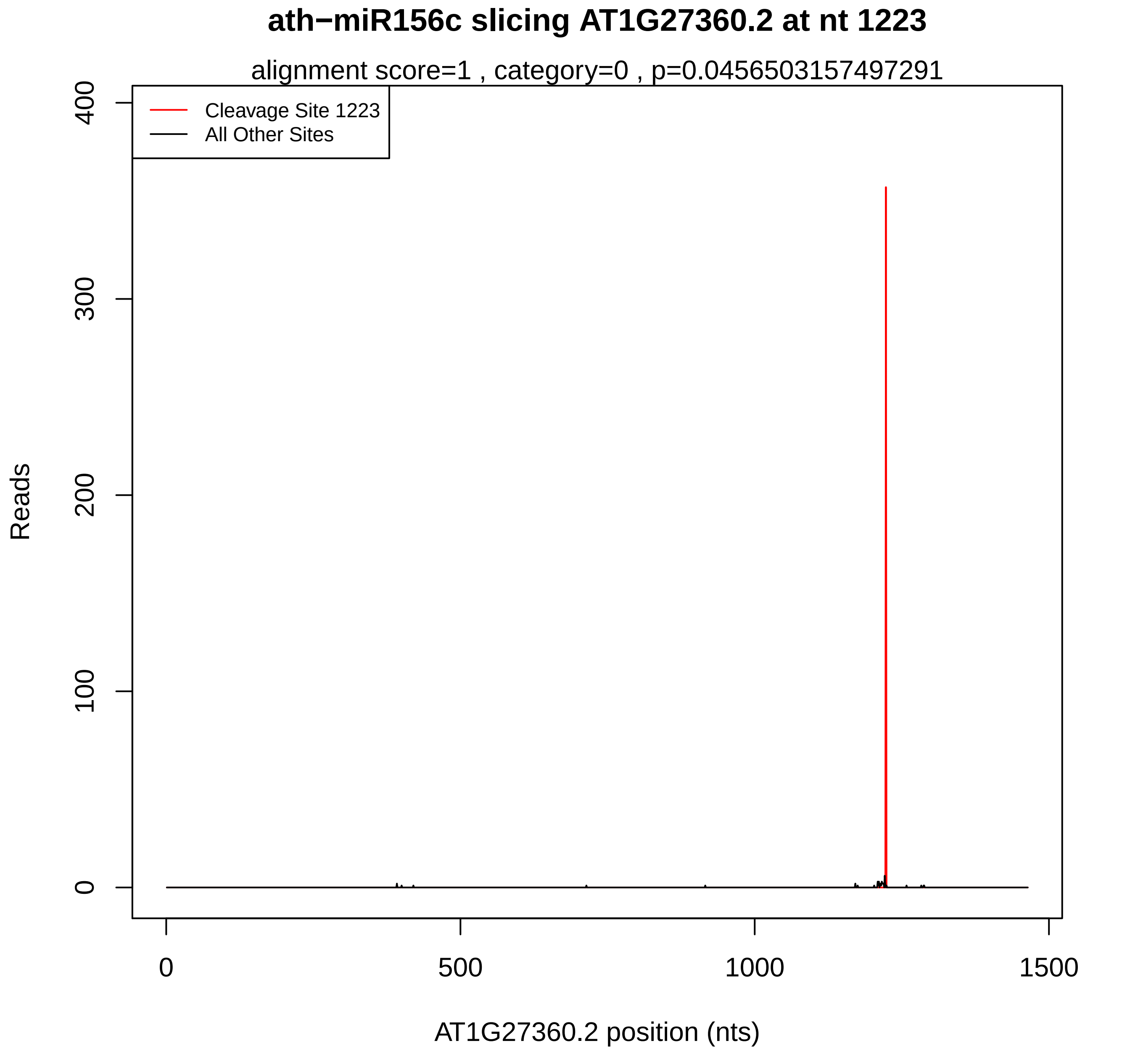
ath−miR156c slicing AT1G27360.2 at nt 1223
alignment score=1 , category=0 , p=0.0456503157497291
Cleavage Site 1223
All Other Sites
Reads
0
100
200
300
400
0
500
1000
1500
AT1G27360.2 position (nts)

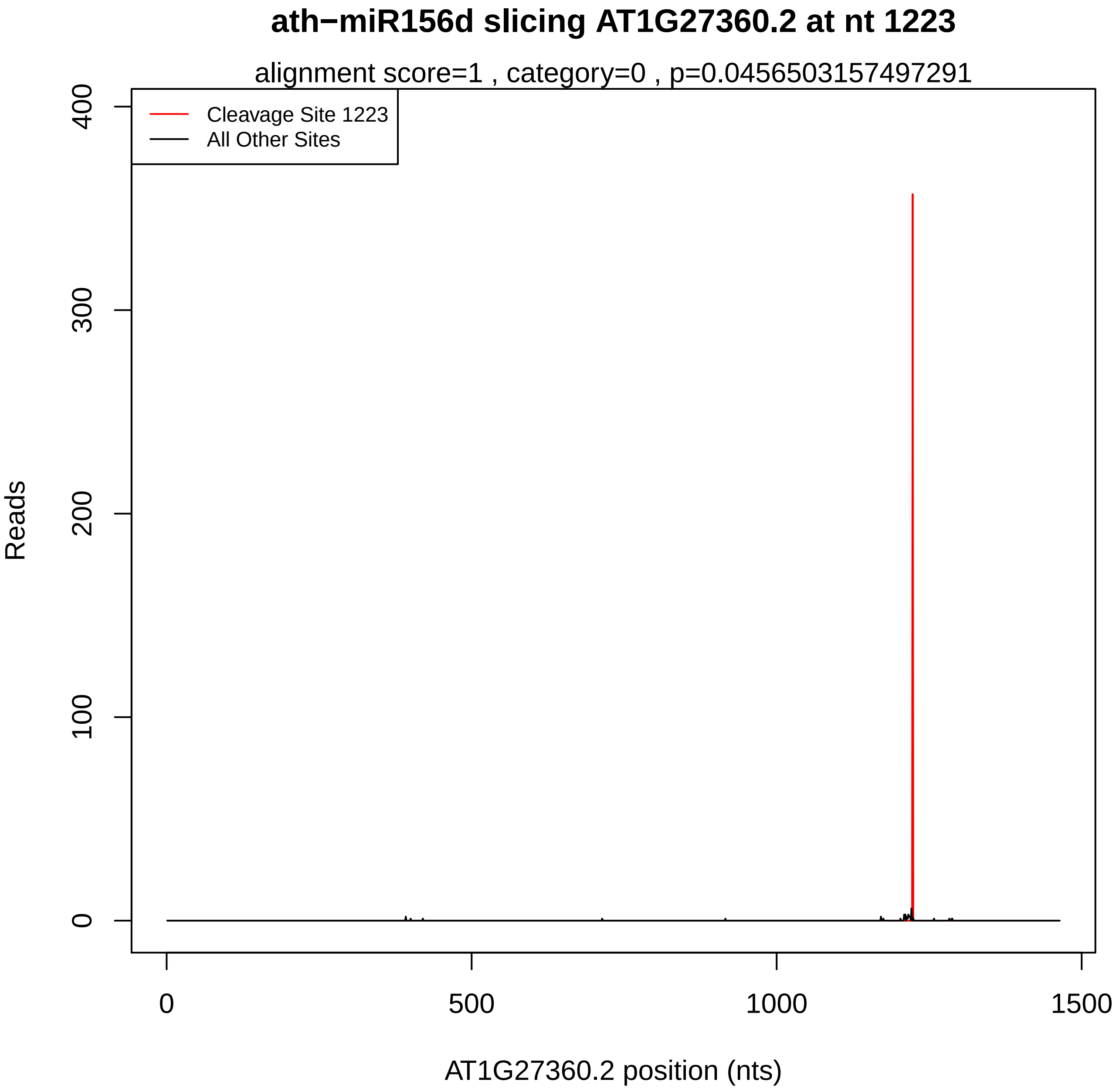
ath−miR156d slicing AT1G27360.2 at nt 1223
alignment score=1 , category=0 , p=0.0456503157497291
Cleavage Site 1223
All Other Sites
Reads
0
100
200
300
400
0
500
1000
1500
AT1G27360.2 position (nts)

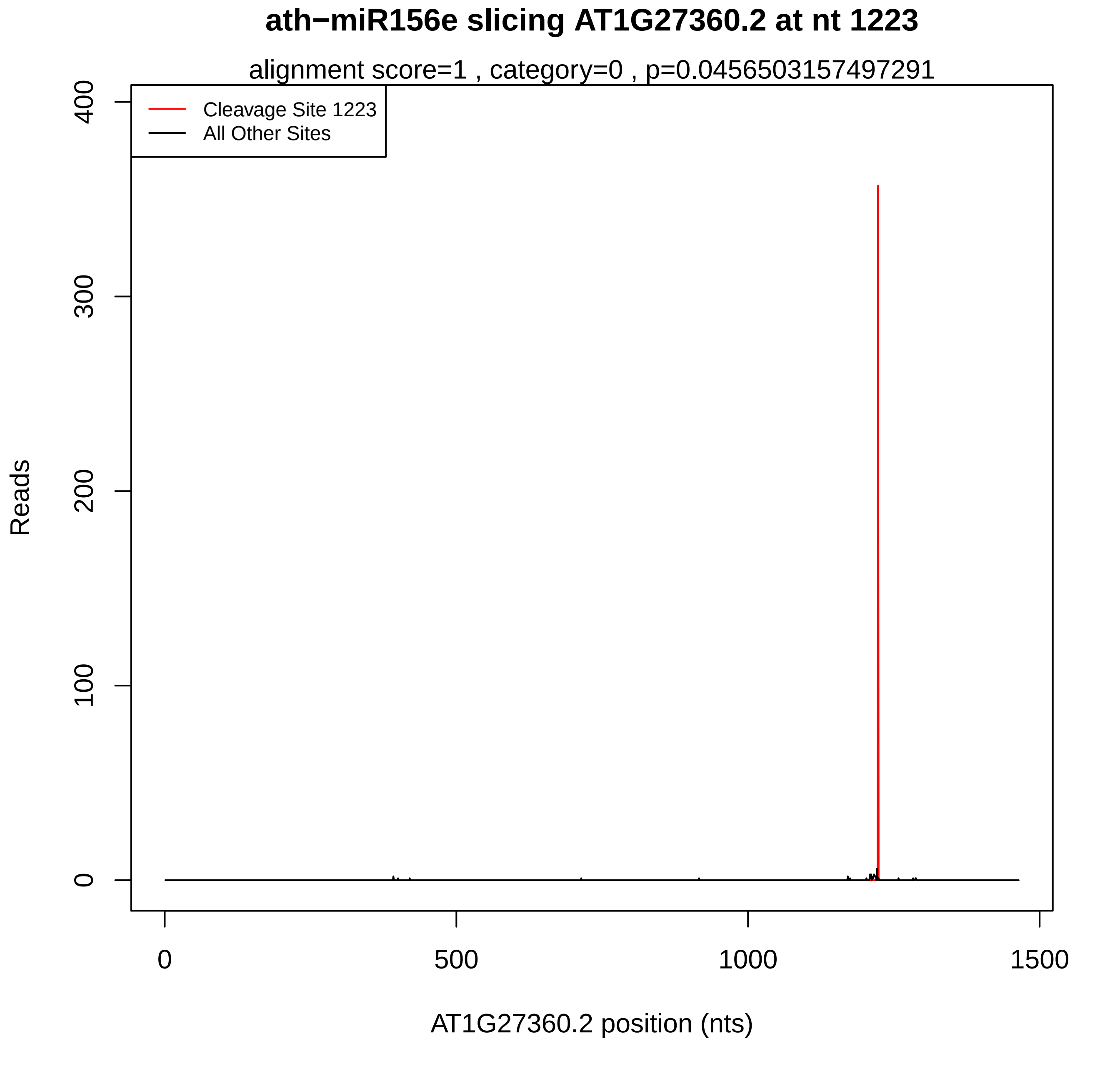
ath−miR156e slicing AT1G27360.2 at nt 1223
alignment score=1 , category=0 , p=0.0456503157497291
Cleavage Site 1223
All Other Sites
Reads
0
100
200
300
400
0
500
1000
1500
AT1G27360.2 position (nts)

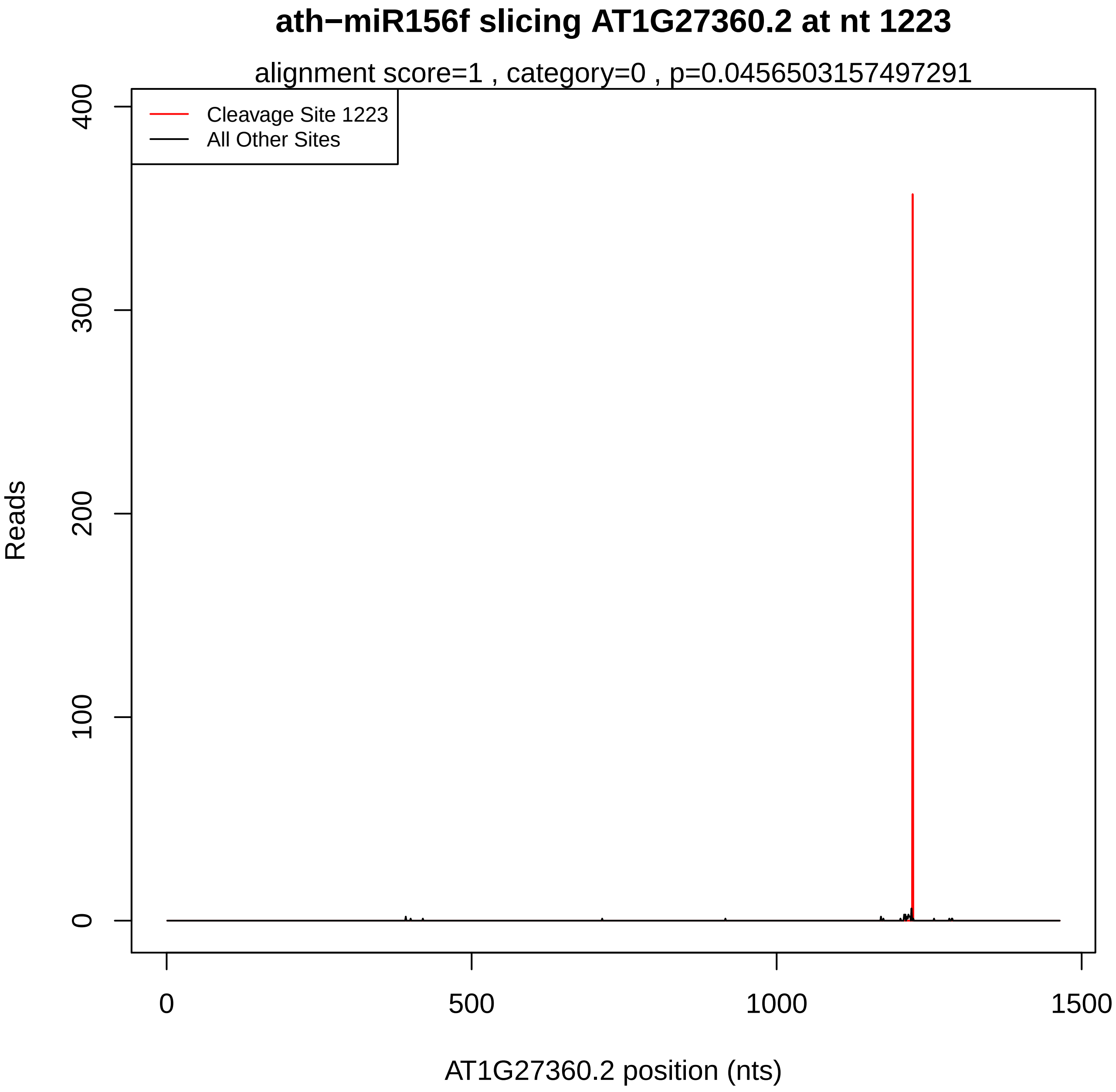
ath−miR156f slicing AT1G27360.2 at nt 1223
alignment score=1 , category=0 , p=0.0456503157497291
Cleavage Site 1223
All Other Sites
Reads
0
100
200
300
400
0
500
1000
1500
AT1G27360.2 position (nts)

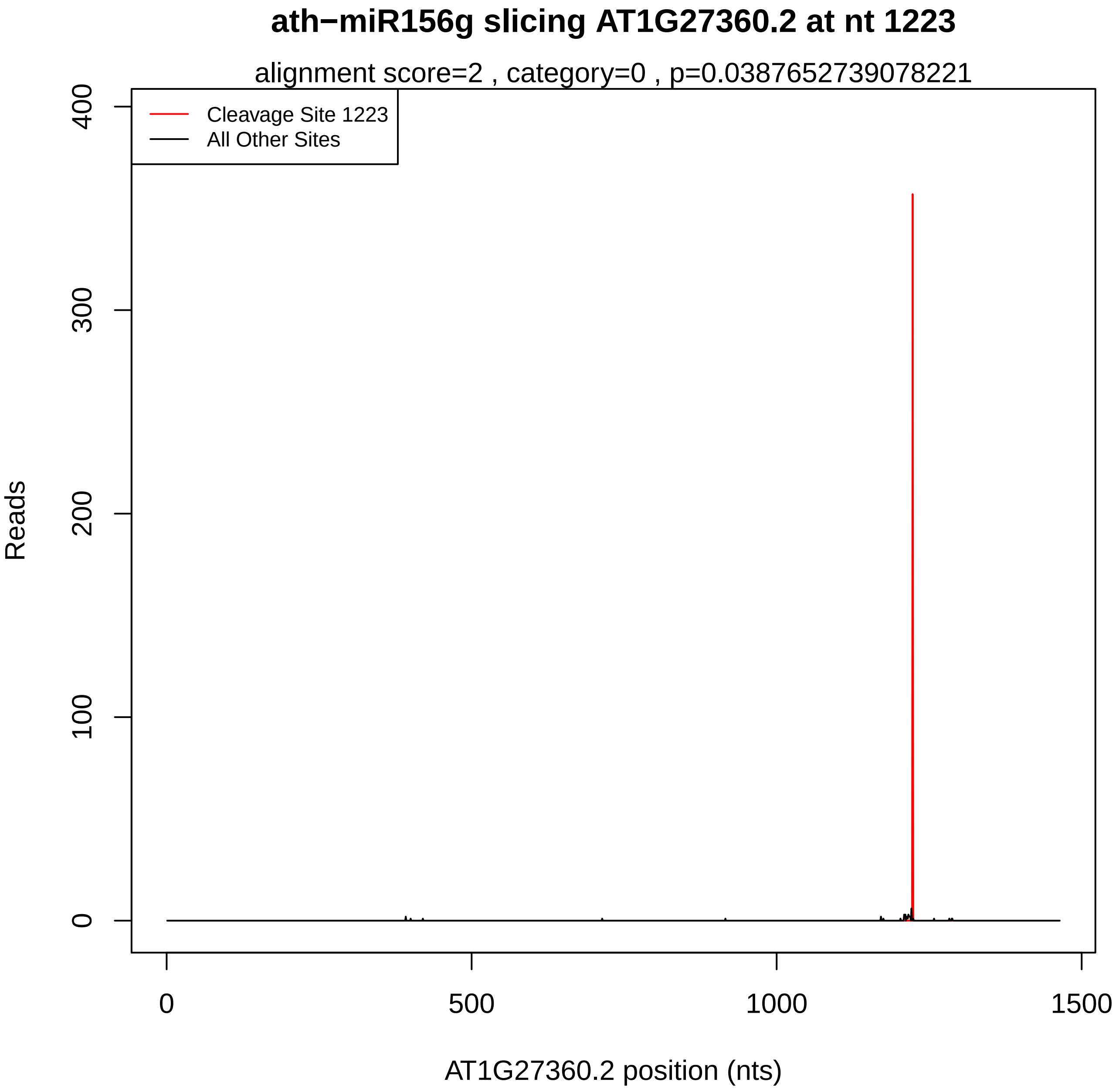
ath−miR156g slicing AT1G27360.2 at nt 1223
alignment score=2 , category=0 , p=0.0387652739078221
Cleavage Site 1223
All Other Sites
Reads
0
100
200
300
400
0
500
1000
1500
AT1G27360.2 position (nts)

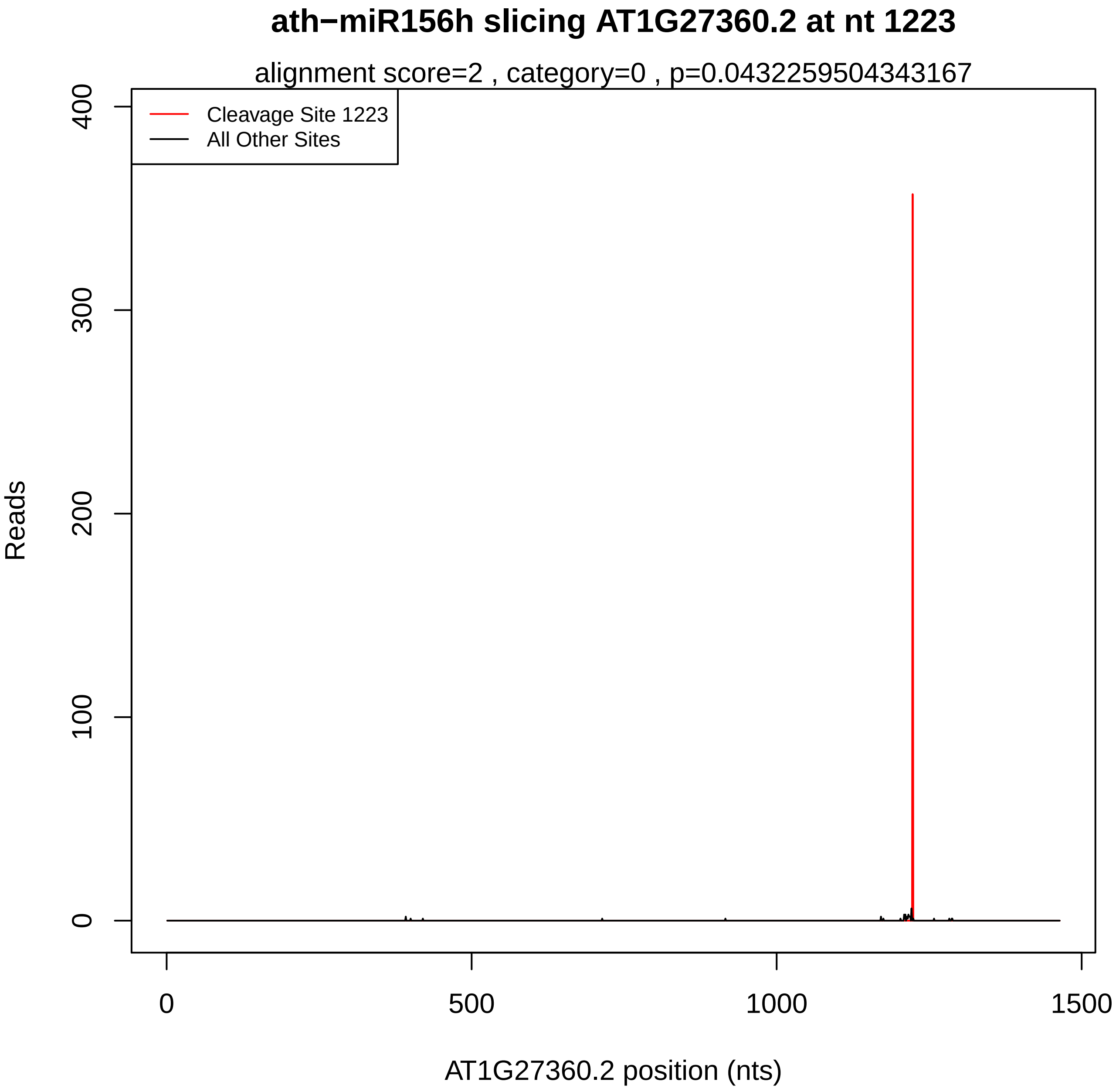
ath−miR156h slicing AT1G27360.2 at nt 1223
alignment score=2 , category=0 , p=0.0432259504343167
Cleavage Site 1223
All Other Sites
Reads
0
100
200
300
400
0
500
1000
1500
AT1G27360.2 position (nts)

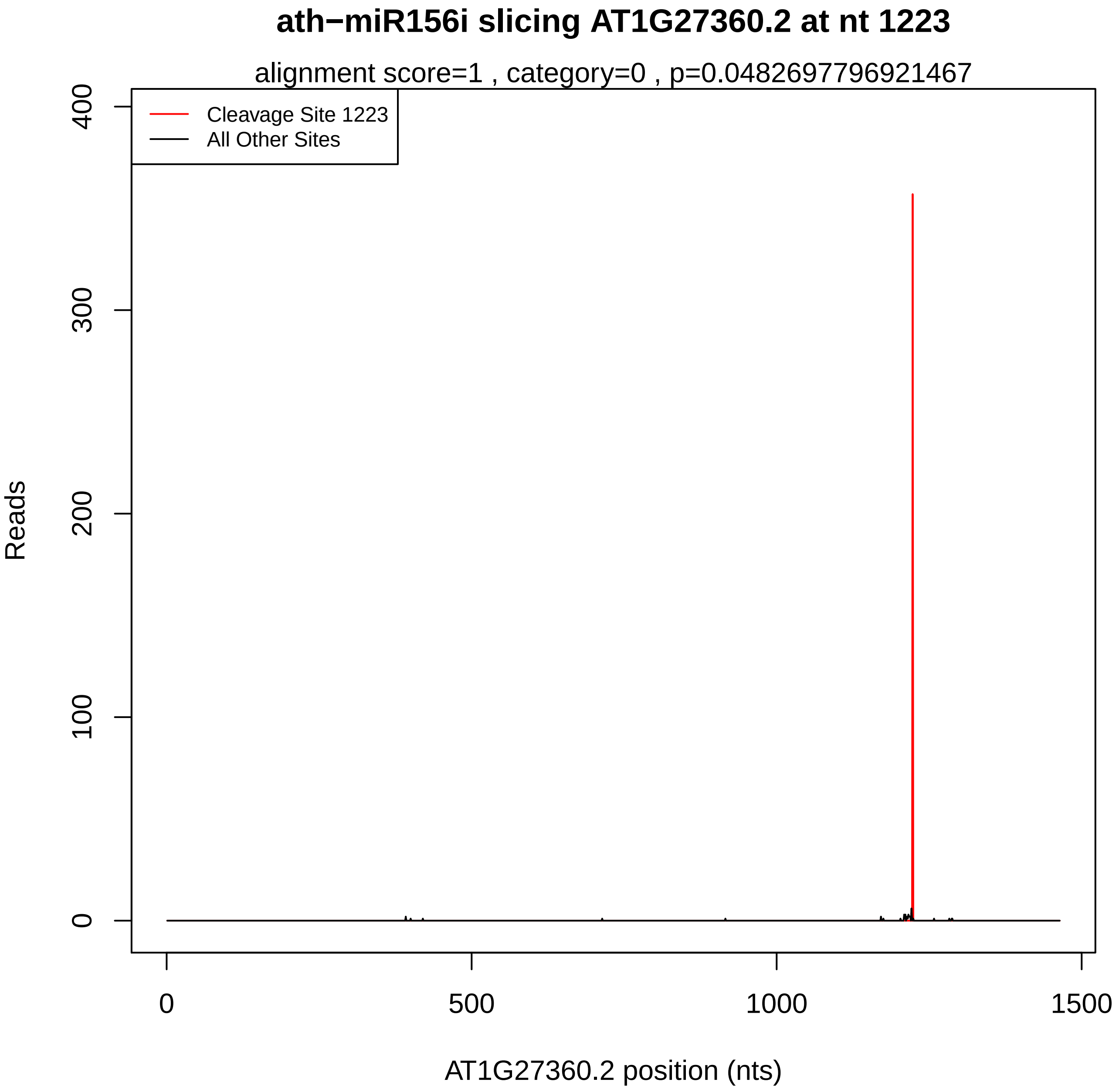

ath−miR156i slicing AT1G27360.2 at nt 1223
alignment score=1 , category=0 , p=0.0482697796921467
Cleavage Site 1223
All Other Sites
Reads
0
100
200
300
400
500
1000
1500
AT1G27360.2 position (nts)

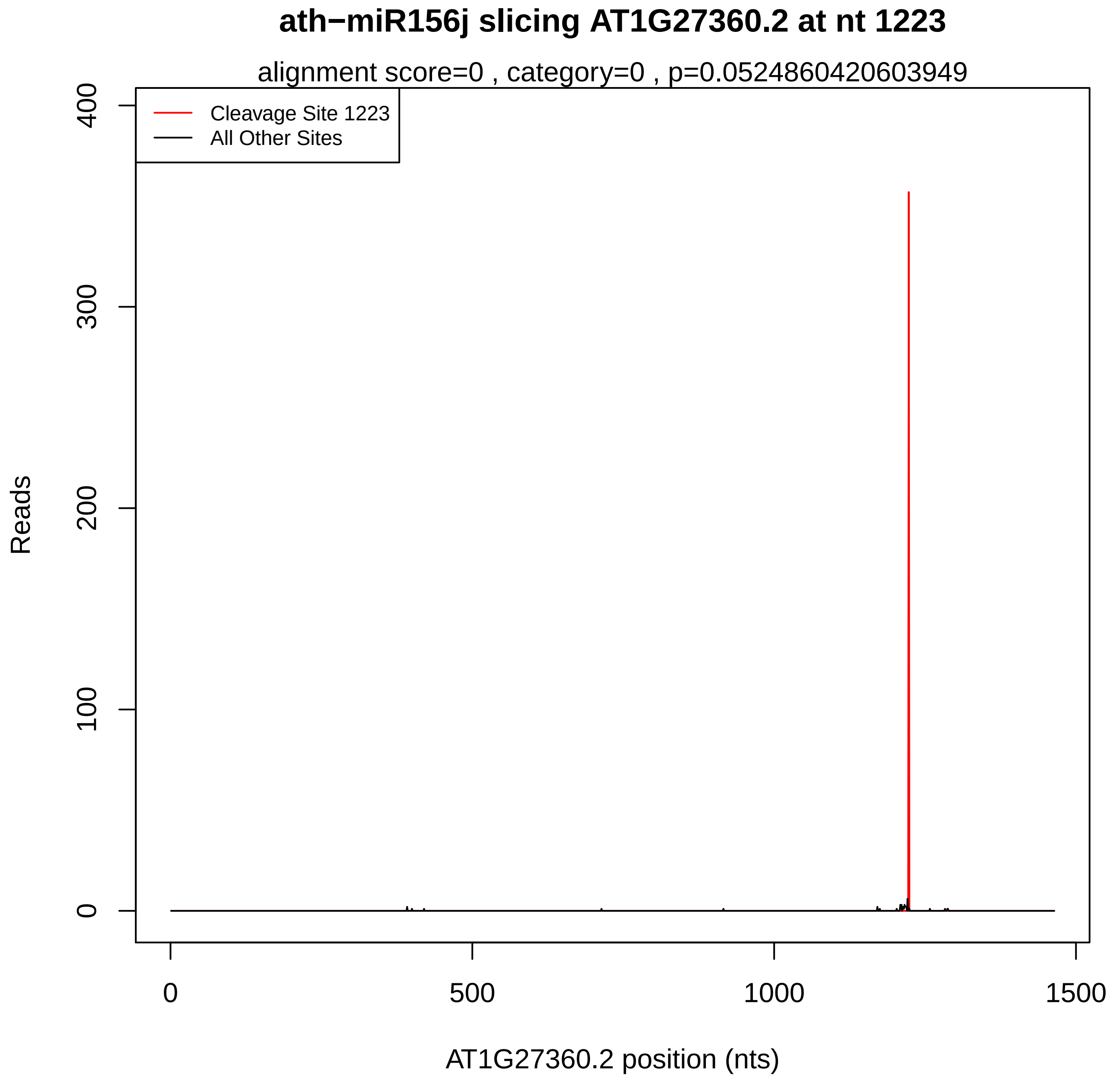

ath−miR156j slicing AT1G27360.2 at nt 1223
alignment score=0 , category=0 , p=0.0524860420603949
Cleavage Site 1223
All Other Sites
Reads
0
100
200
300
400
0
500
1000
1500
AT1G27360.2 position (nts)

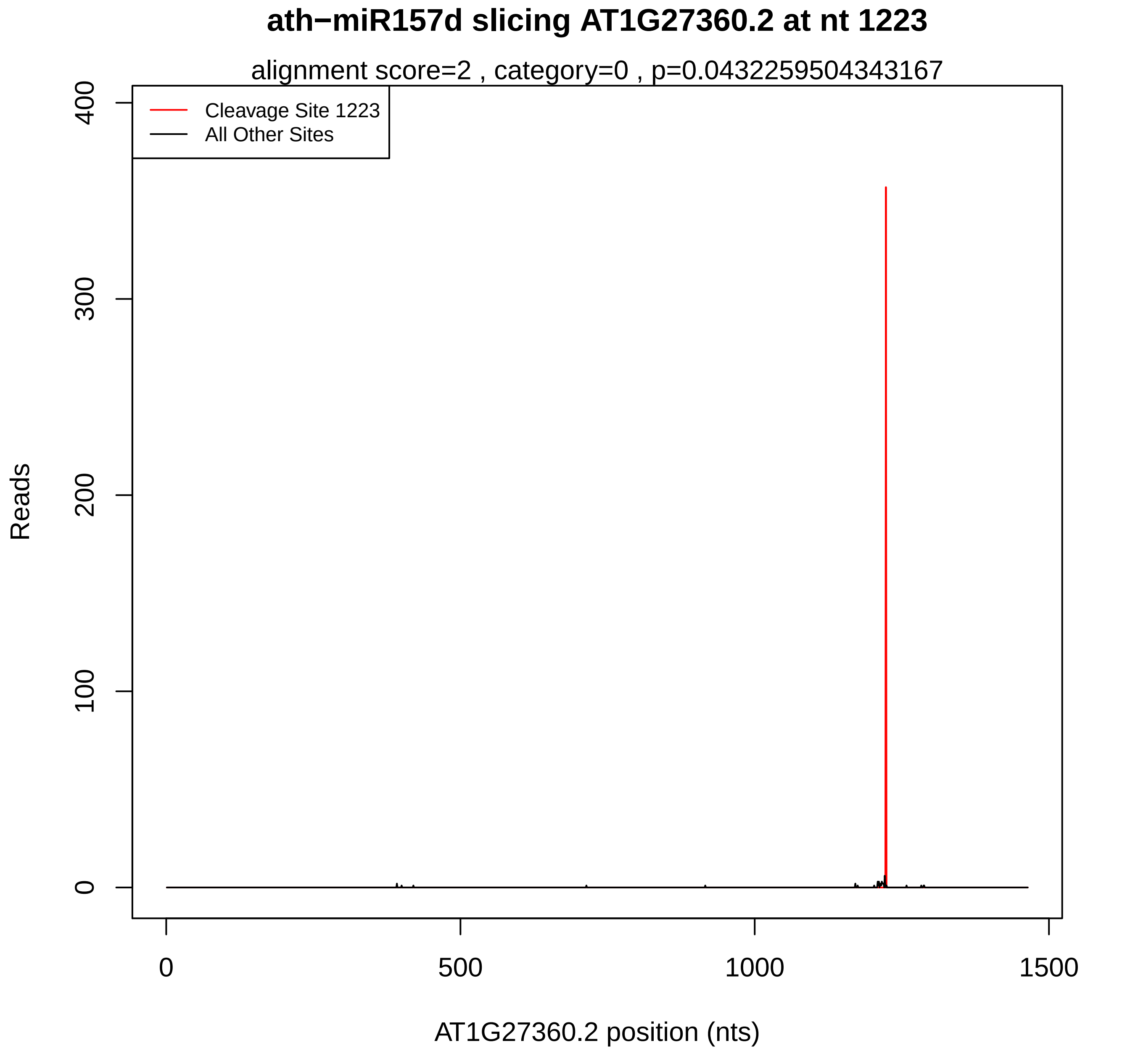
ath−miR157d slicing AT1G27360.2 at nt 1223
alignment score=2 , category=0 , p=0.0432259504343167
Cleavage Site 1223
All Other Sites
Reads
0
100
200
300
400
0
500
1000
1500
AT1G27360.2 position (nts)

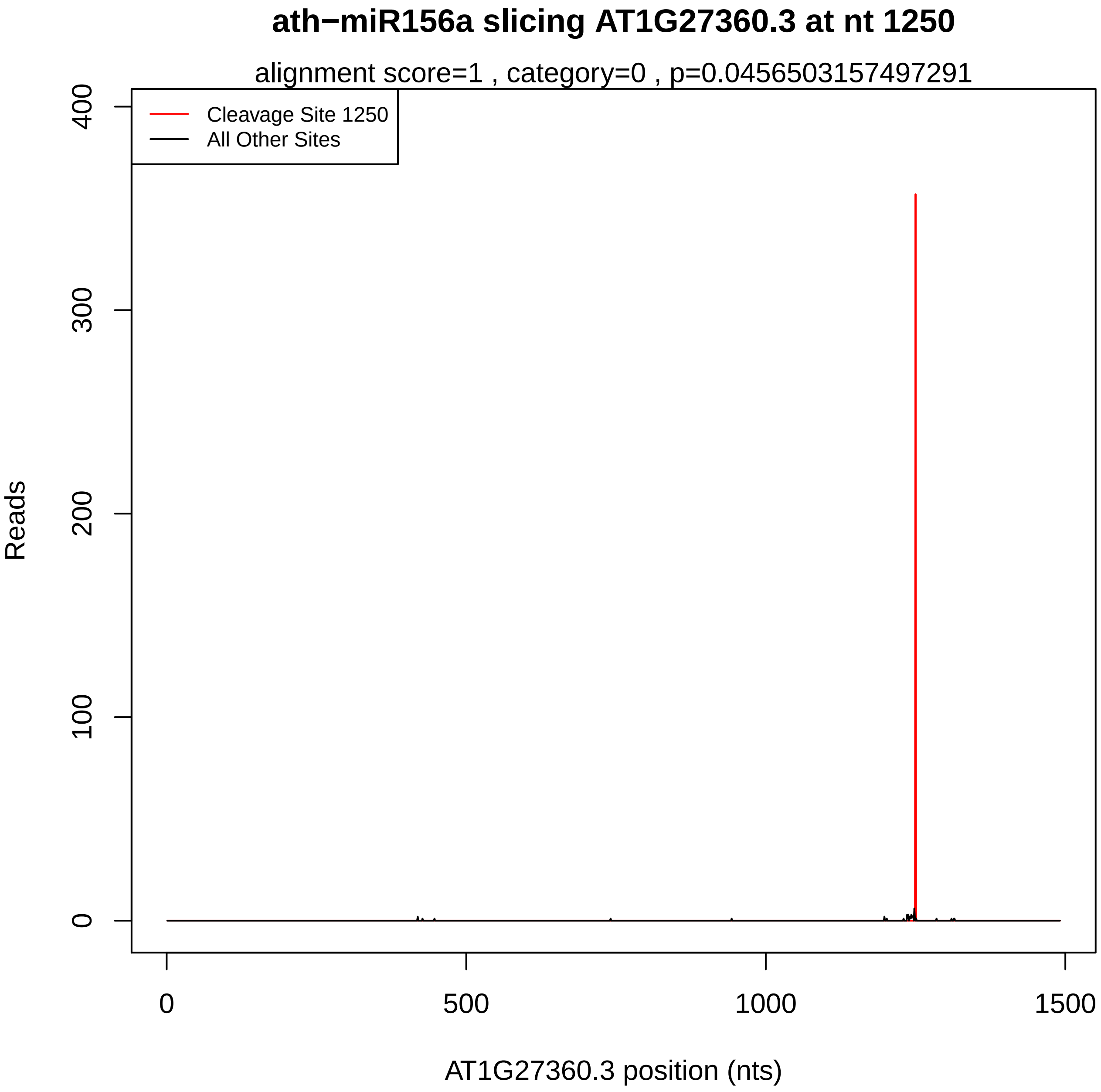
ath−miR156a slicing AT1G27360.3 at nt 1250
alignment score=1 , category=0 , p=0.0456503157497291
Cleavage Site 1250
All Other Sites
Reads
0
100
200
300
400
0
500
1000
1500
AT1G27360.3 position (nts)

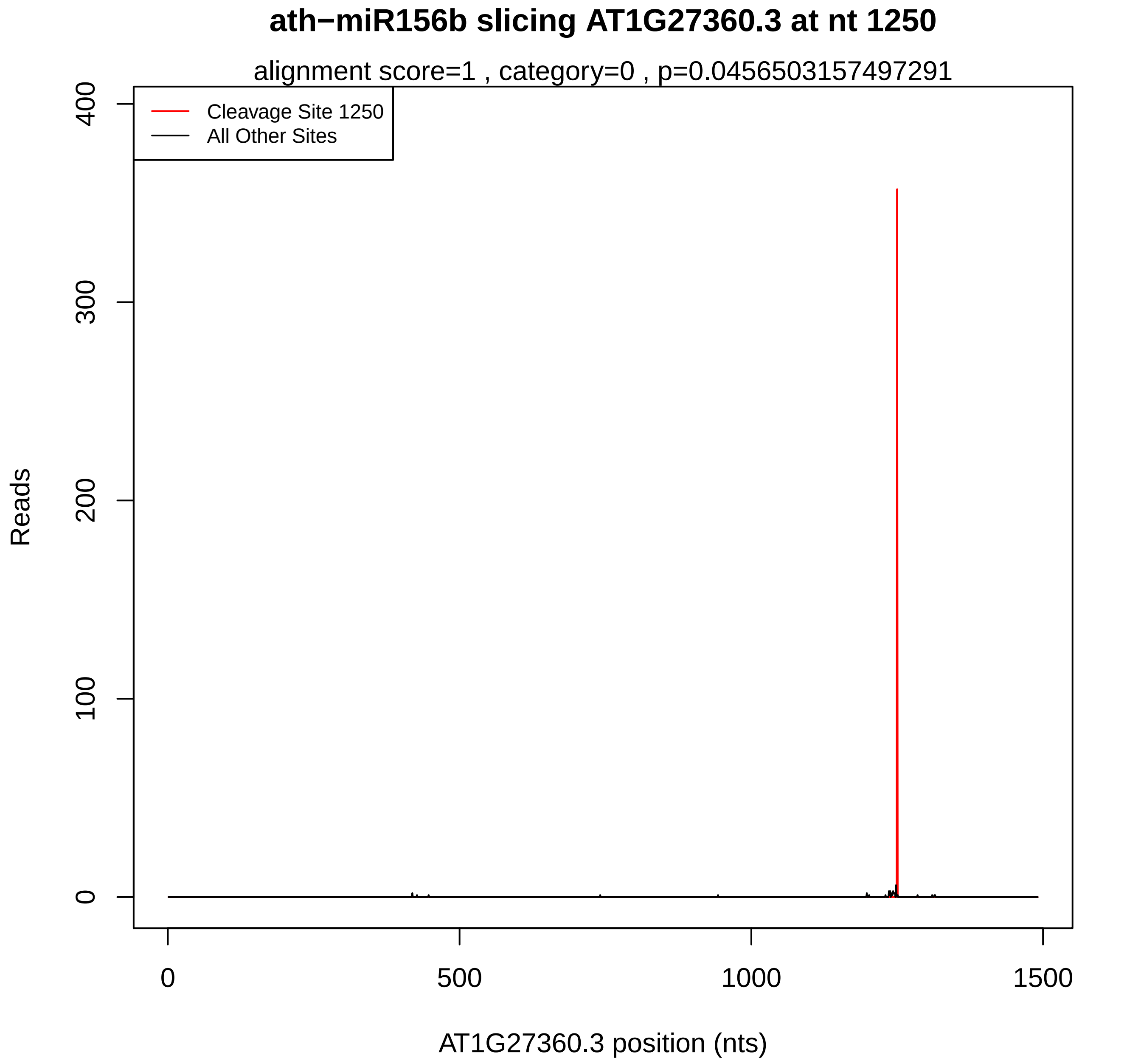
ath−miR156b slicing AT1G27360.3 at nt 1250
alignment score=1 , category=0 , p=0.0456503157497291
Cleavage Site 1250
All Other Sites
Reads
0
100
200
300
400
0
500
1000
1500
AT1G27360.3 position (nts)

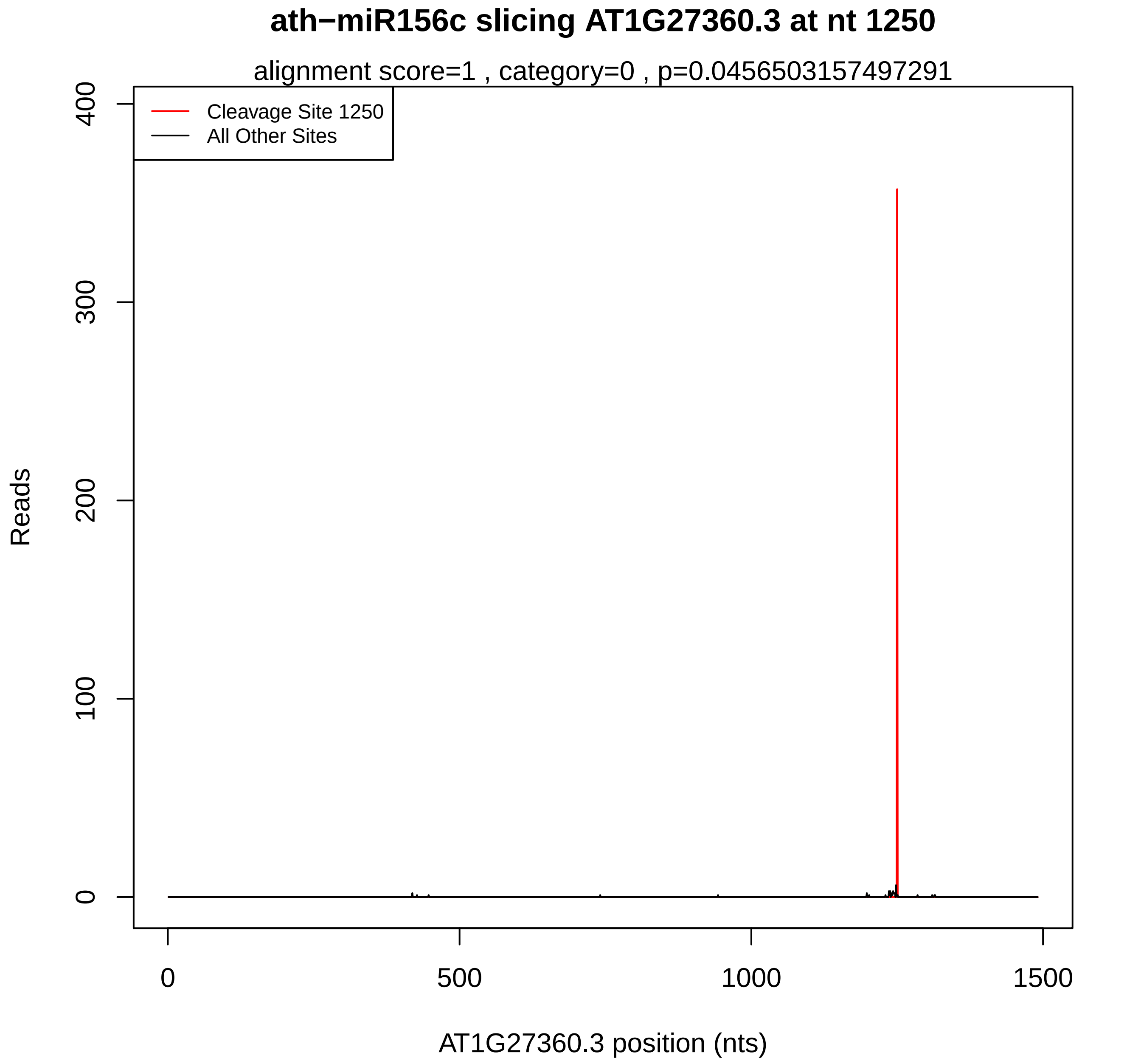

ath−miR156c slicing AT1G27360.3 at nt 1250
alignment score=1 , category=0 , p=0.0456503157497291
Cleavage Site 1250
All Other Sites
Reads
0
100
200
300
400
0
500
1000
1500
AT1G27360.3 position (nts)

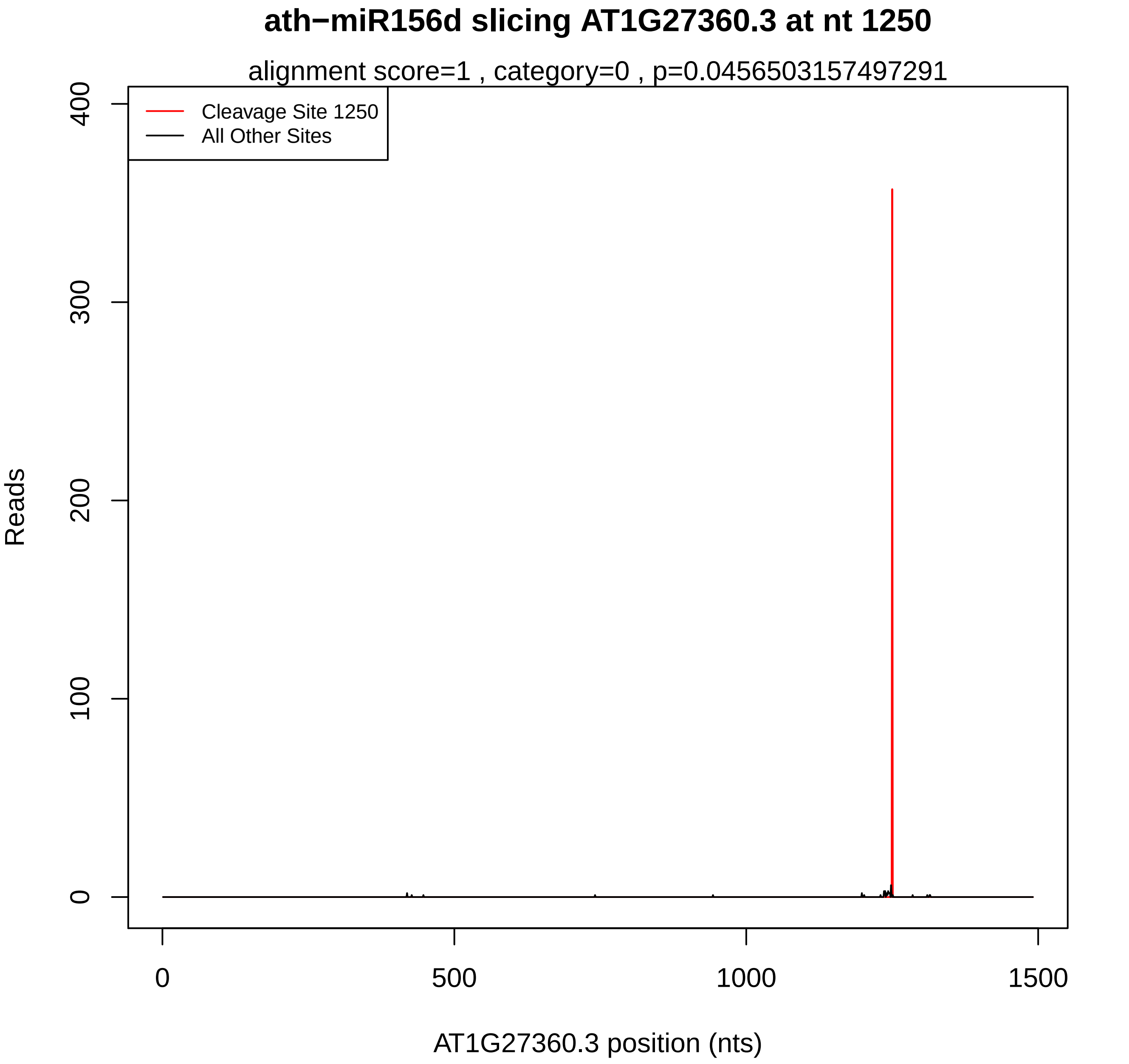
ath−miR156d slicing AT1G27360.3 at nt 1250
alignment score=1 , category=0 , p=0.0456503157497291
Cleavage Site 1250
All Other Sites
Reads
0
100
200
300
400
0
500
1000
1500
AT1G27360.3 position (nts)

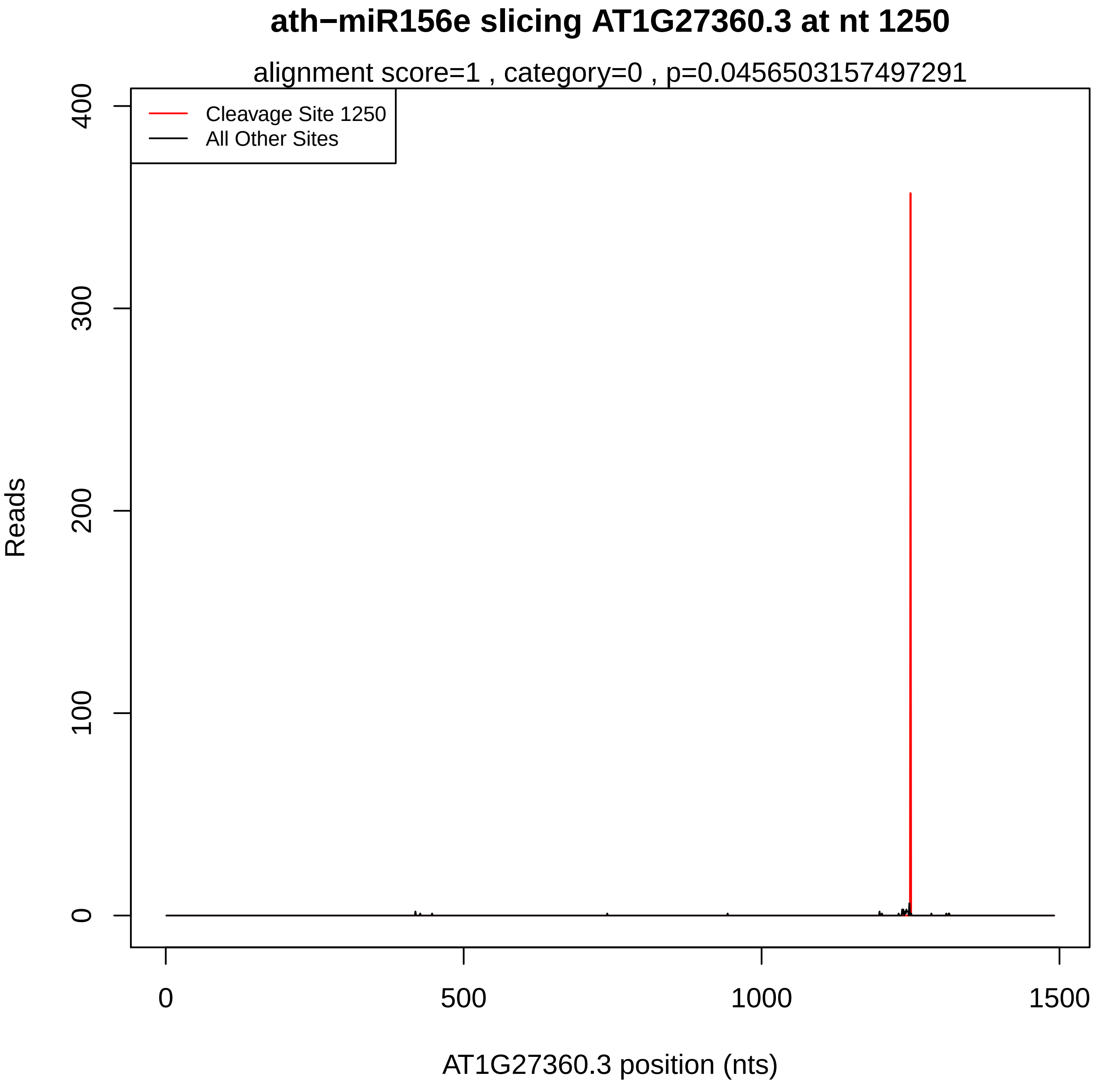
ath−miR156e slicing AT1G27360.3 at nt 1250
alignment score=1 , category=0 , p=0.0456503157497291
Cleavage Site 1250
All Other Sites
Reads
0
100
200
300
400
0
500
1000
1500
AT1G27360.3 position (nts)

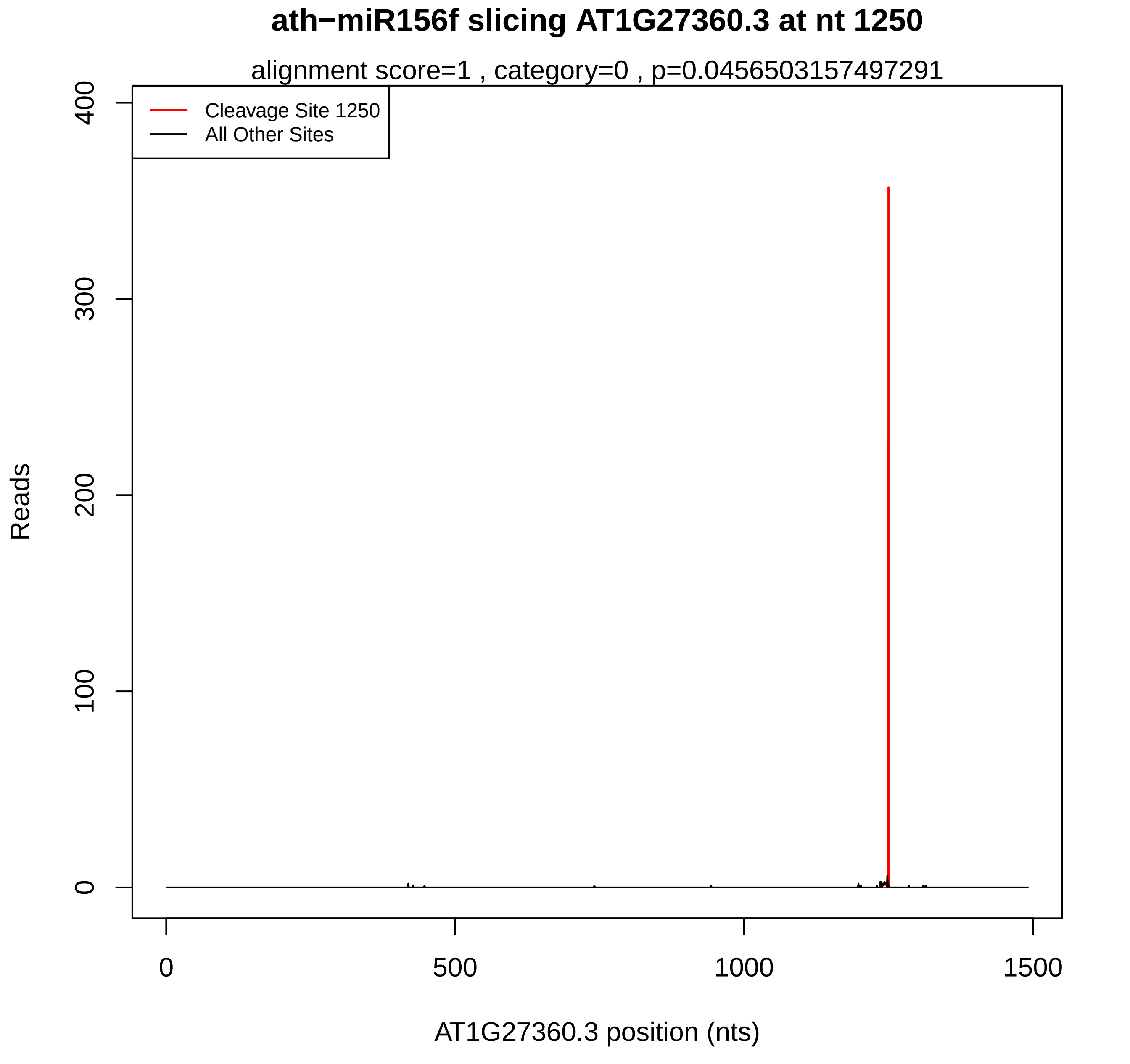
ath−miR156f slicing AT1G27360.3 at nt 1250
alignment score=1 , category=0 , p=0.0456503157497291
Cleavage Site 1250
All Other Sites
Reads
0
100
200
300
400
0
500
1000
1500
AT1G27360.3 position (nts)

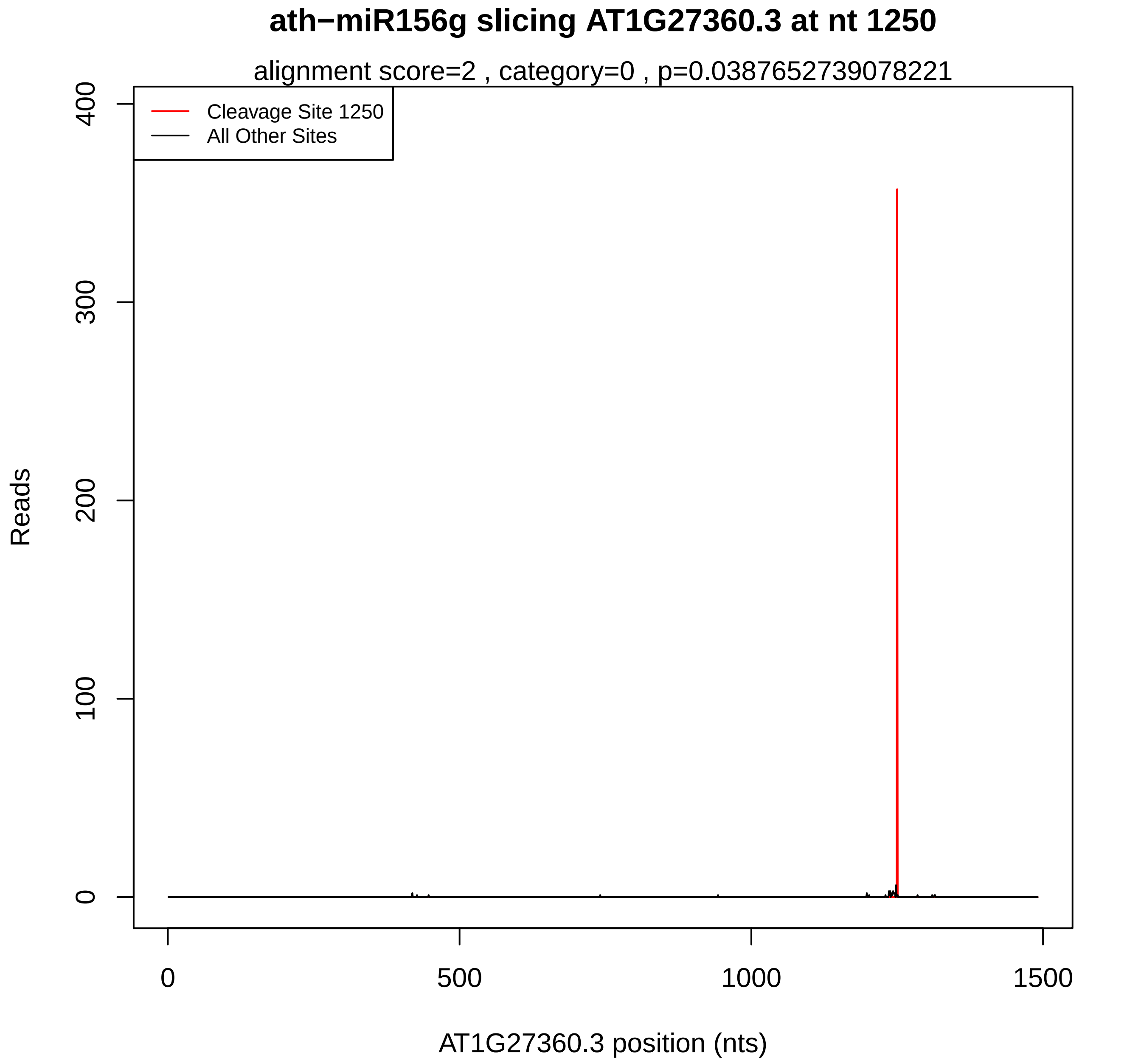
ath−miR156g slicing AT1G27360.3 at nt 1250
alignment score=2 , category=0 , p=0.0387652739078221
Cleavage Site 1250
All Other Sites
Reads
0
100
200
300
400
0
500
1000
1500
AT1G27360.3 position (nts)

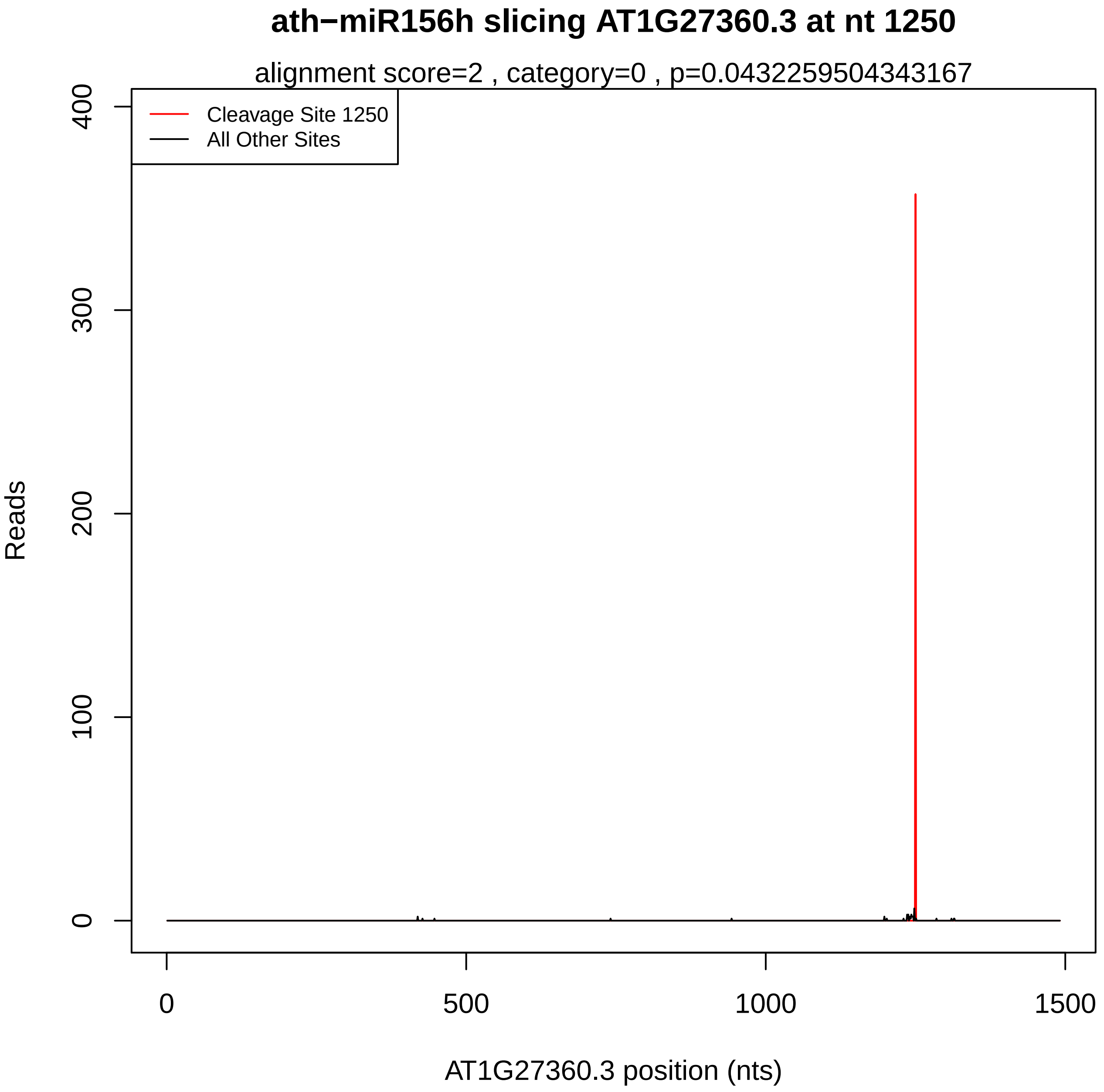
ath−miR156h slicing AT1G27360.3 at nt 1250
alignment score=2 , category=0 , p=0.0432259504343167
Cleavage Site 1250
All Other Sites
Reads
0
100
200
300
400
0
500
1000
1500
AT1G27360.3 position (nts)

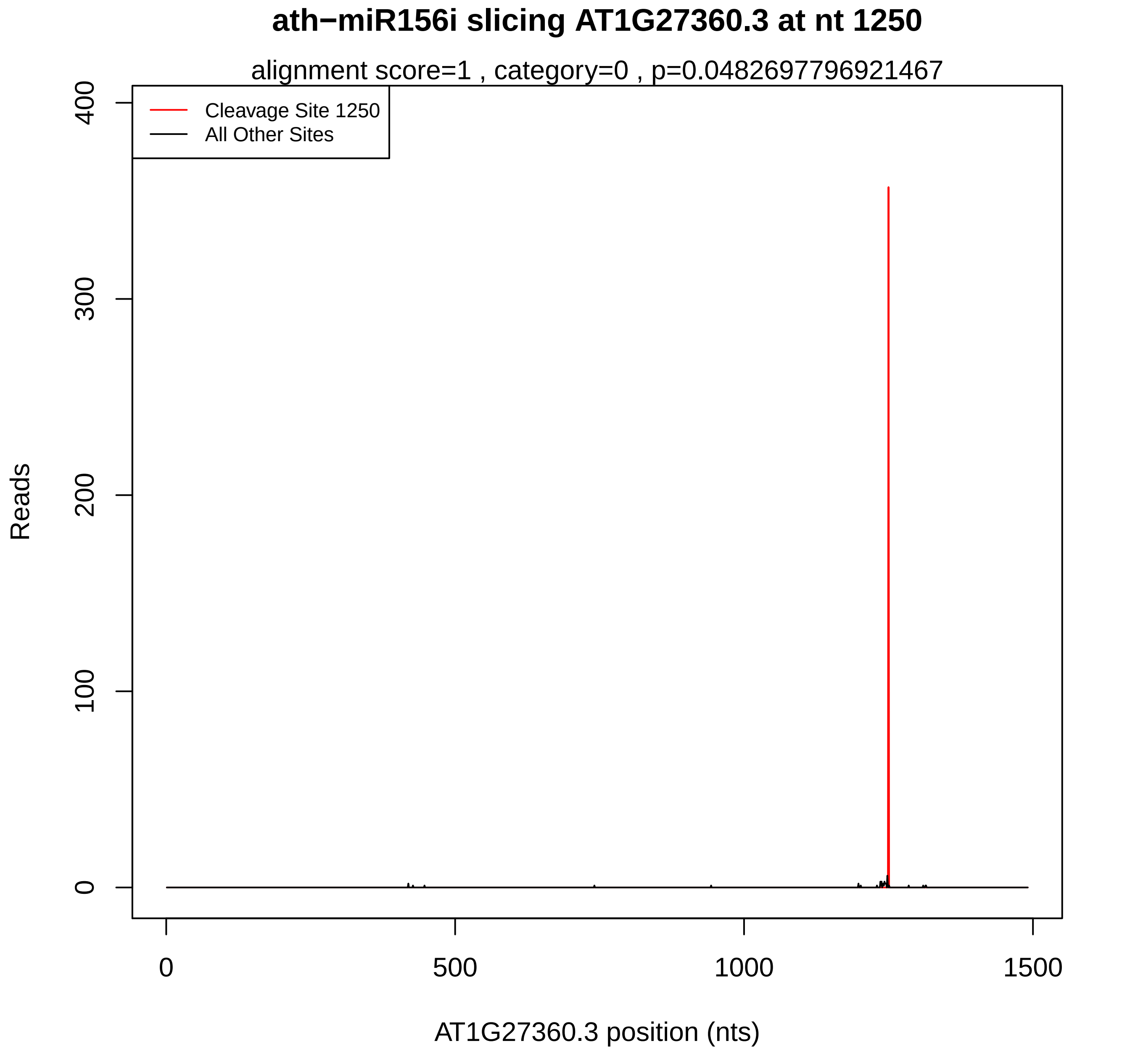
ath−miR156i slicing AT1G27360.3 at nt 1250
alignment score=1 , category=0 , p=0.0482697796921467
Cleavage Site 1250
All Other Sites
Reads
0
100
200
300
400
0
500
1000
1500
AT1G27360.3 position (nts)

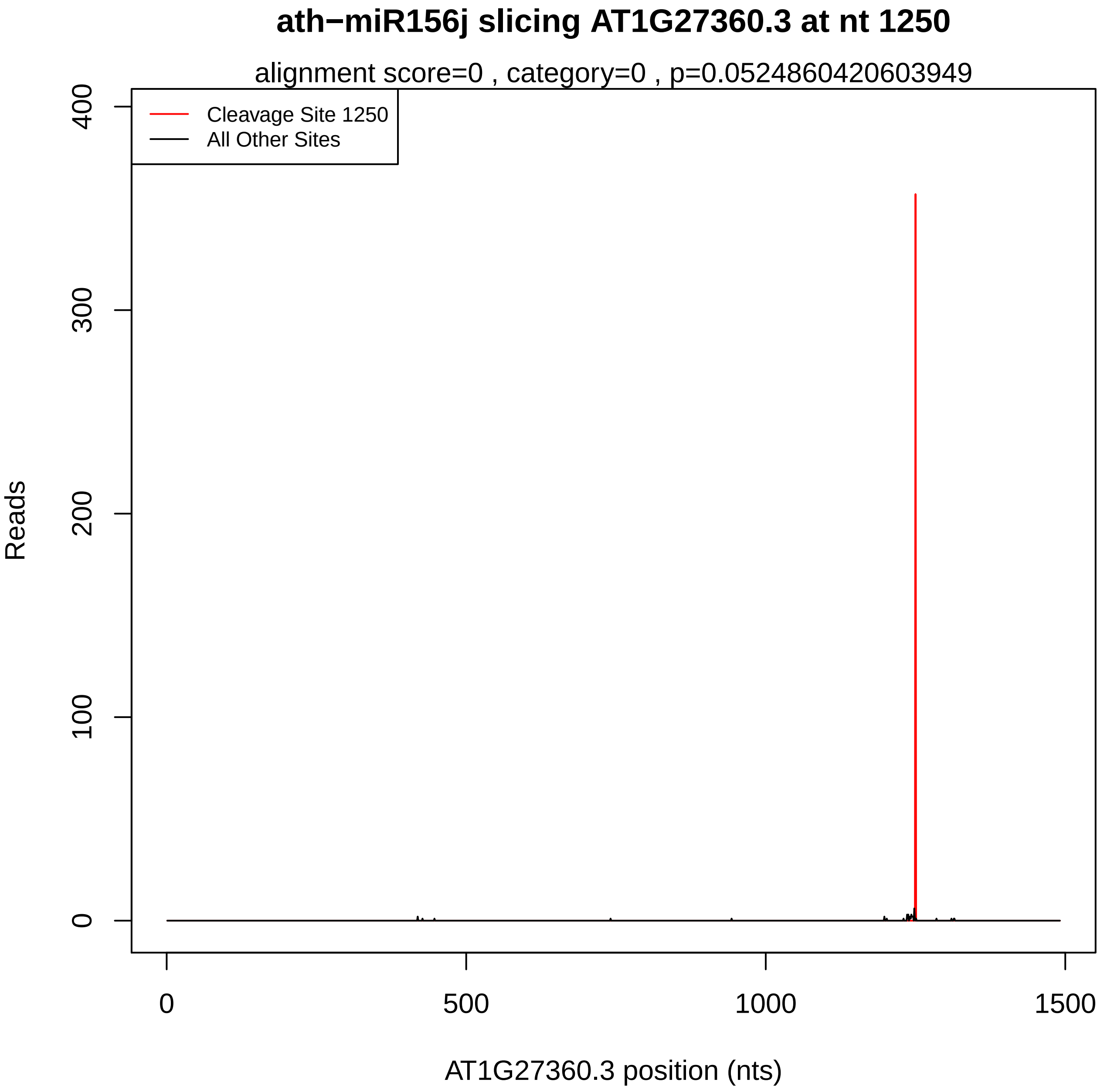
ath−miR156j slicing AT1G27360.3 at nt 1250
alignment score=0 , category=0 , p=0.0524860420603949
Cleavage Site 1250
All Other Sites
Reads
0
100
200
300
400
0
500
1000
1500
AT1G27360.3 position (nts)

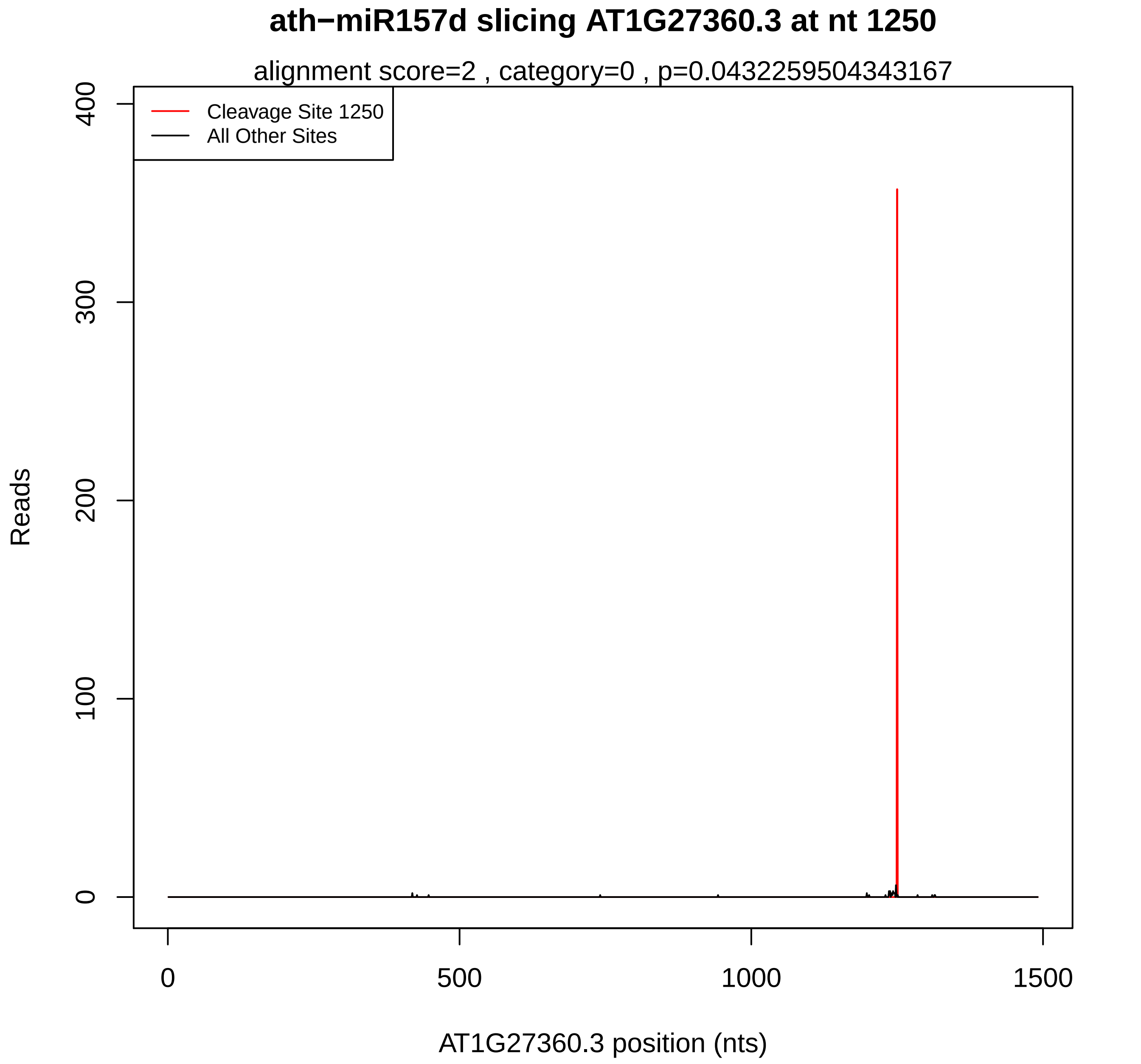

ath−miR157d slicing AT1G27360.3 at nt 1250
alignment score=2 , category=0 , p=0.0432259504343167
Cleavage Site 1250
All Other Sites
Reads
0
100
200
300
400
0
500
1000
1500
AT1G27360.3 position (nts)

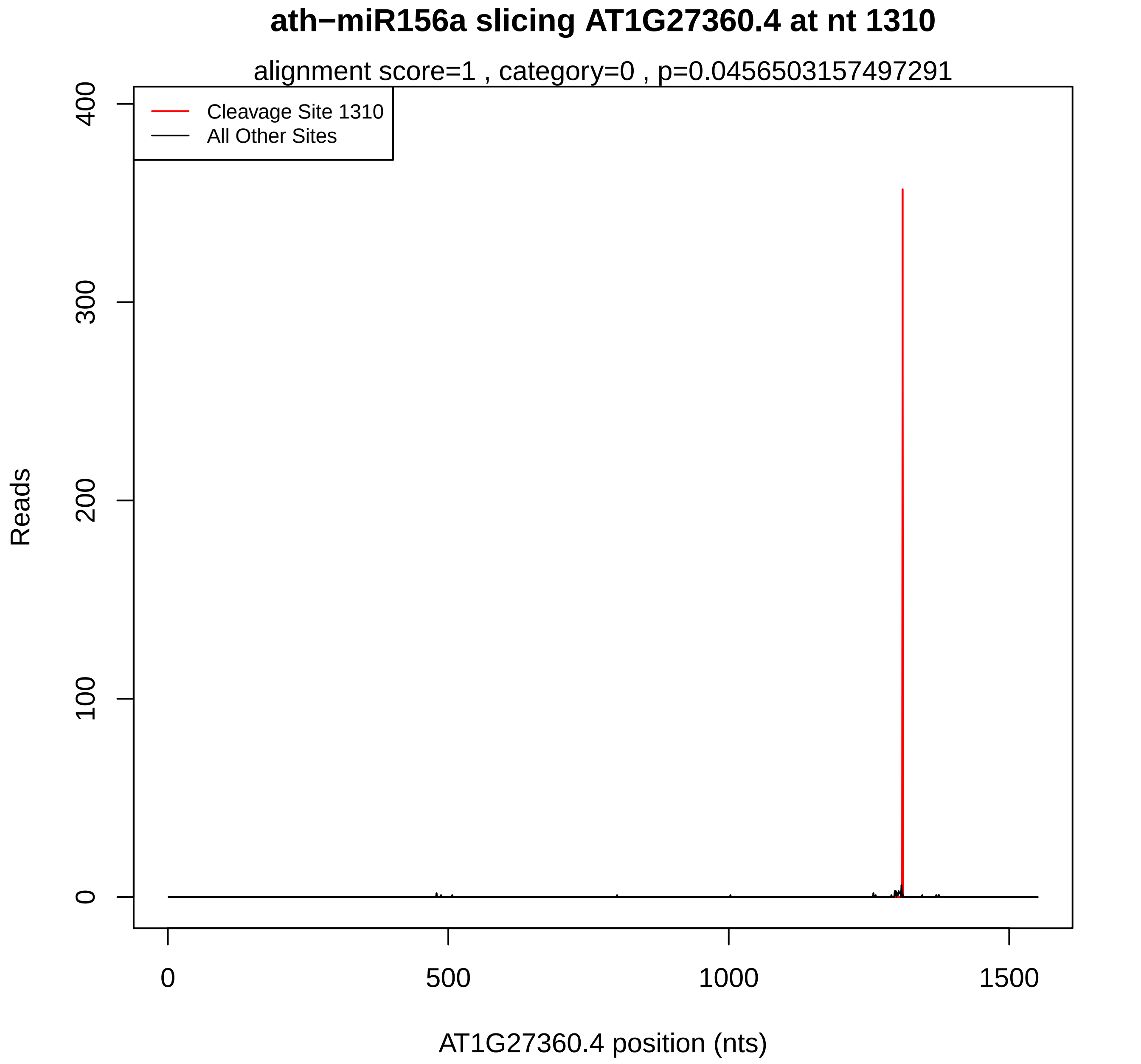

ath−miR156a slicing AT1G27360.4 at nt 1310
alignment score=1 , category=0 , p=0.0456503157497291
Cleavage Site 1310
All Other Sites
Reads
0
100
200
300
400
0
500
1000
1500
AT1G27360.4 position (nts)

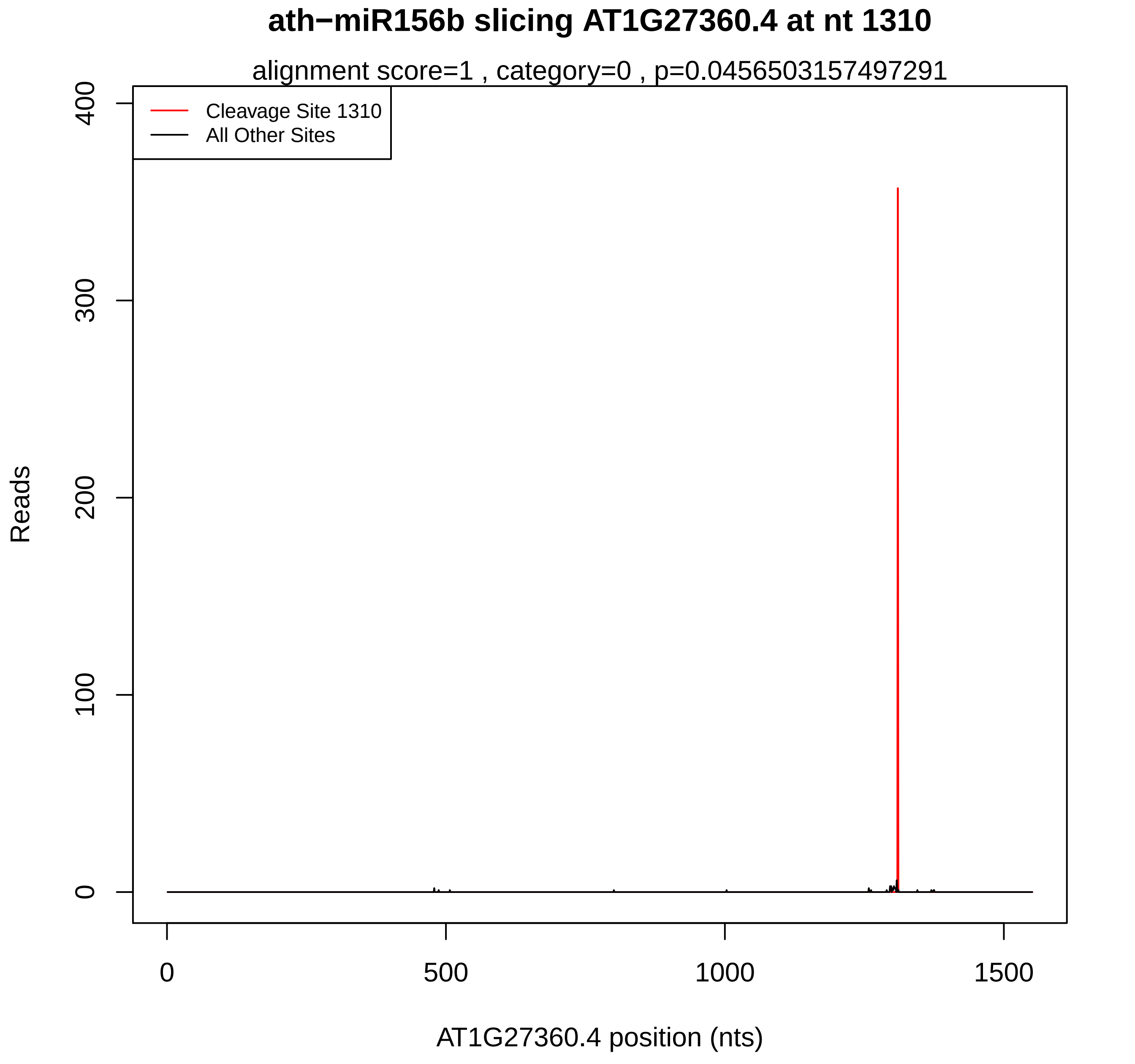

ath−miR156b slicing AT1G27360.4 at nt 1310
alignment score=1 , category=0 , p=0.0456503157497291
Cleavage Site 1310
All Other Sites
Reads
0
100
200
300
400
0
500
1000
1500
AT1G27360.4 position (nts)

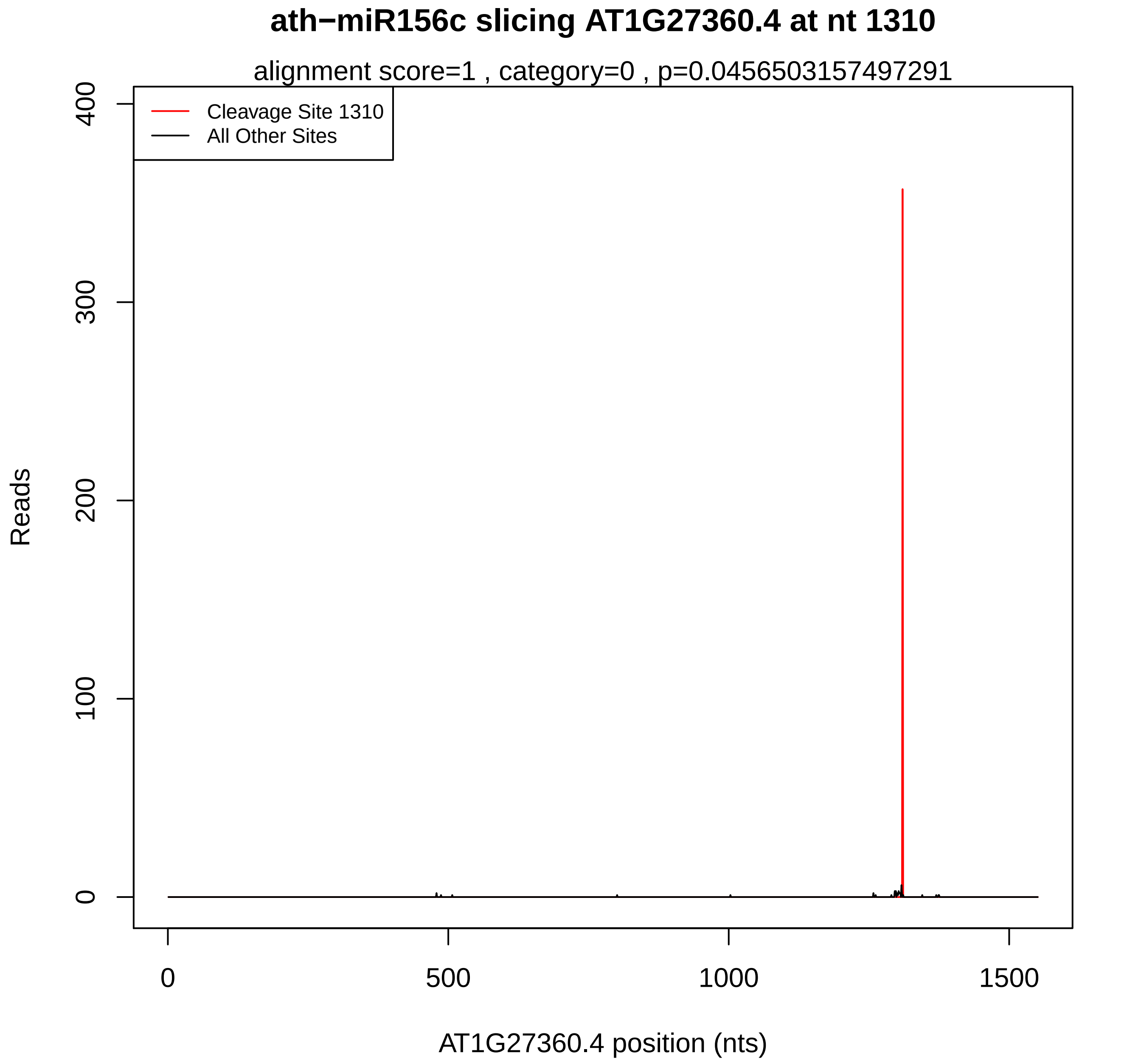
ath−miR156c slicing AT1G27360.4 at nt 1310
alignment score=1 , category=0 , p=0.0456503157497291
Cleavage Site 1310
All Other Sites
Reads
0
100
200
300
400
0
500
1000
1500
AT1G27360.4 position (nts)

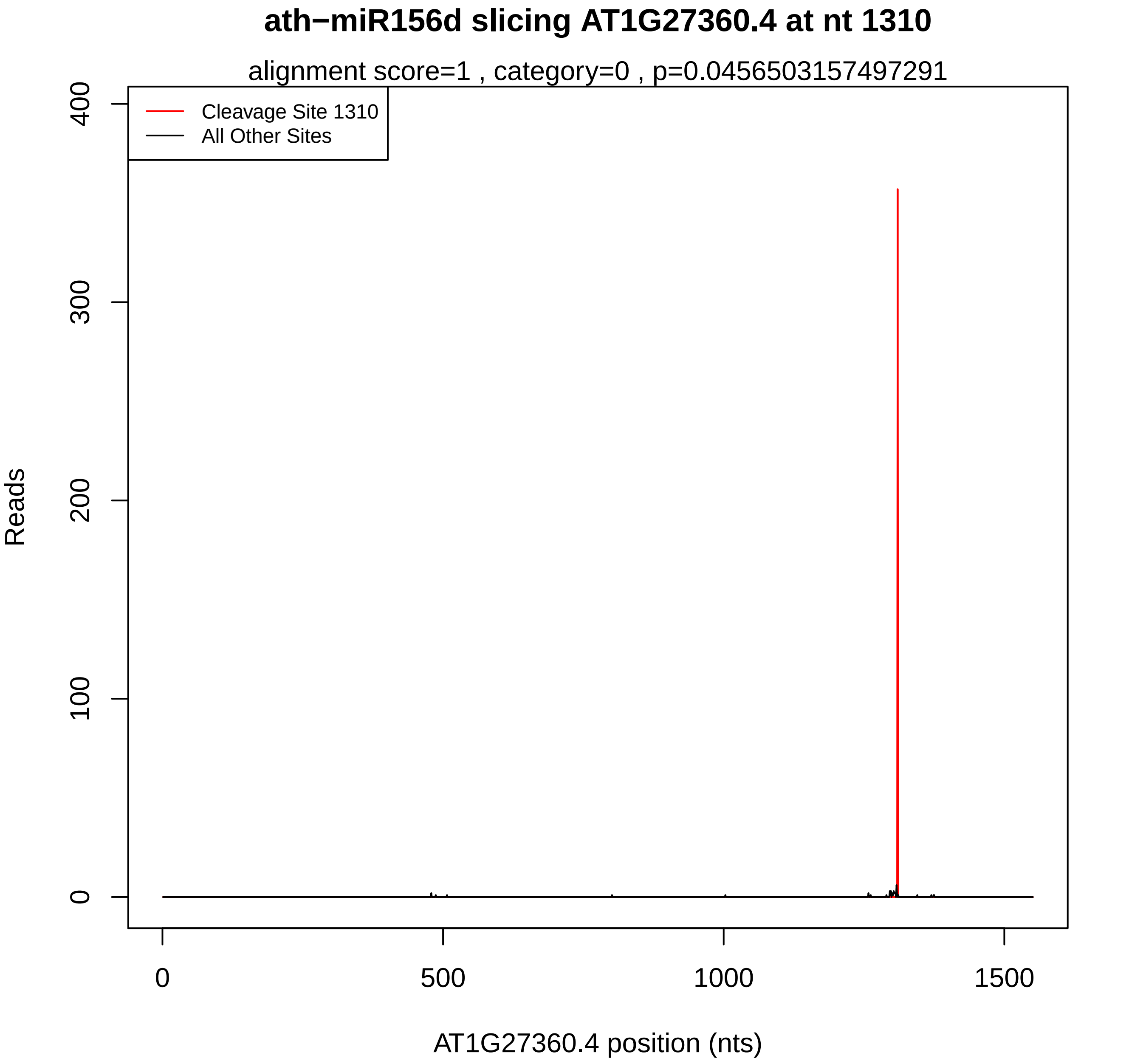
ath−miR156d slicing AT1G27360.4 at nt 1310
alignment score=1 , category=0 , p=0.0456503157497291
Cleavage Site 1310
All Other Sites
Reads
0
100
200
300
400
0
500
1000
1500
AT1G27360.4 position (nts)

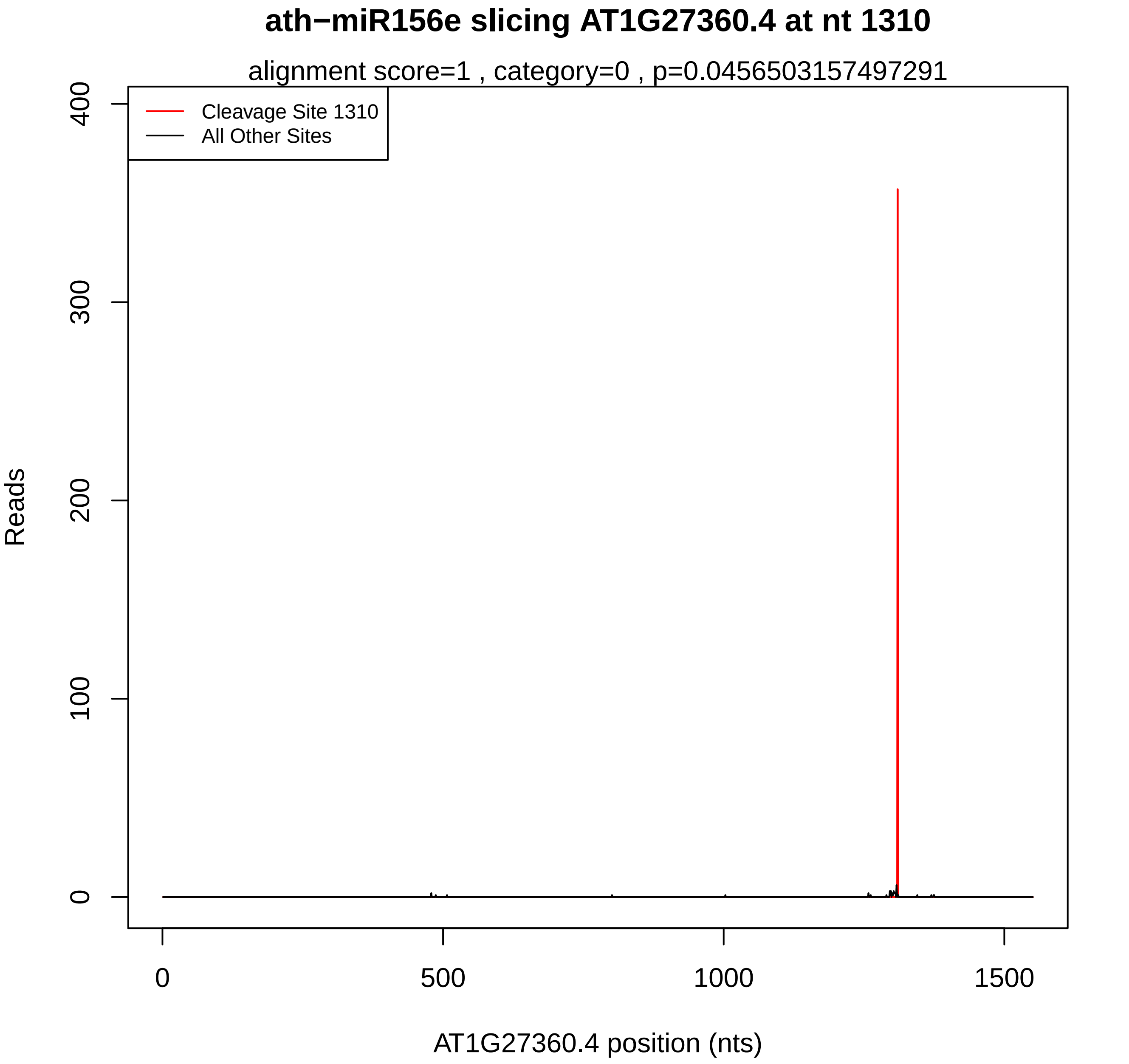
ath−miR156e slicing AT1G27360.4 at nt 1310
alignment score=1 , category=0 , p=0.0456503157497291
Cleavage Site 1310
All Other Sites
Reads
0
100
200
300
400
0
500
1000
1500
AT1G27360.4 position (nts)

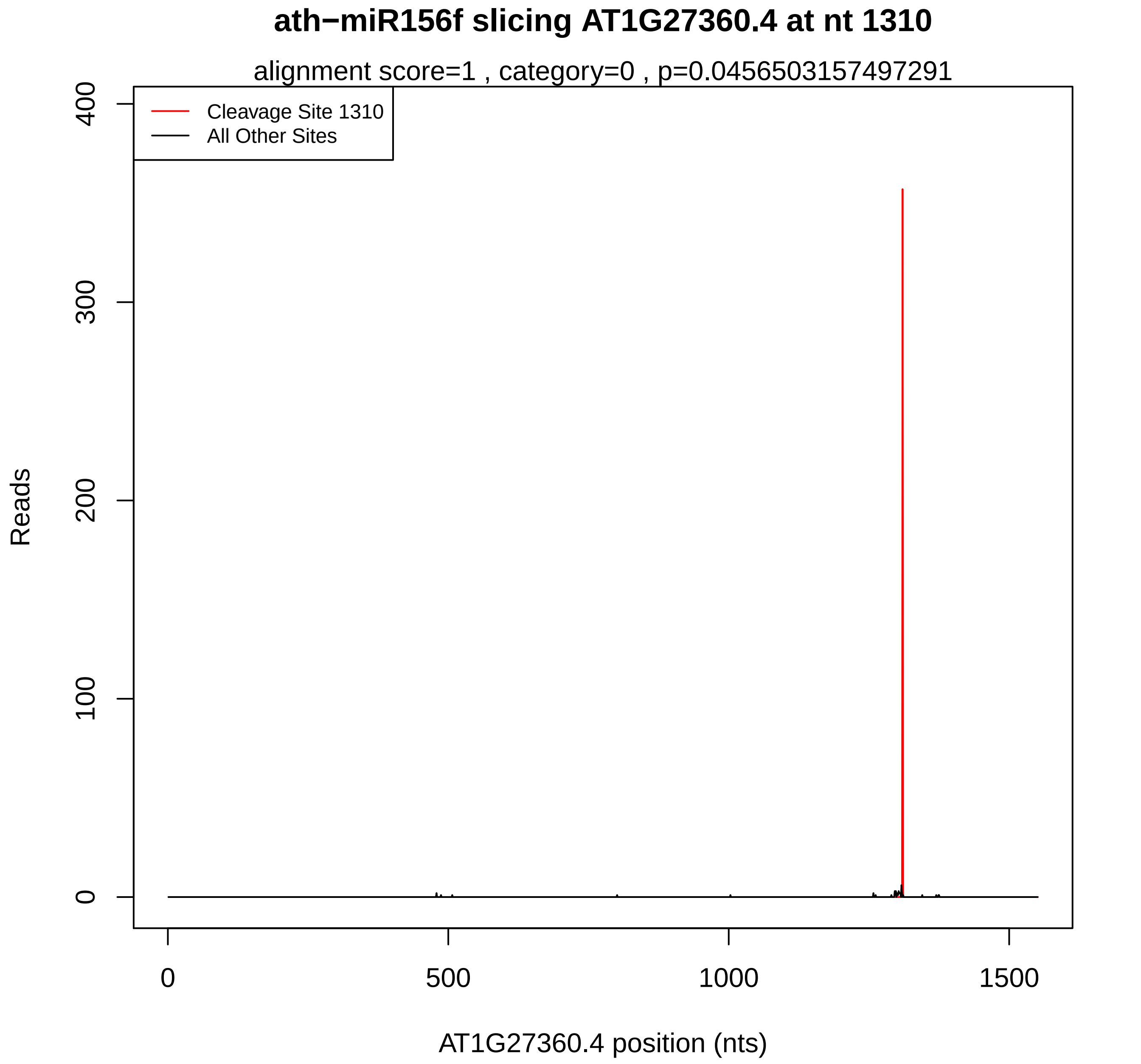
ath−miR156f slicing AT1G27360.4 at nt 1310
alignment score=1 , category=0 , p=0.0456503157497291
Cleavage Site 1310
All Other Sites
Reads
0
100
200
300
400
0
500
1000
1500
AT1G27360.4 position (nts)

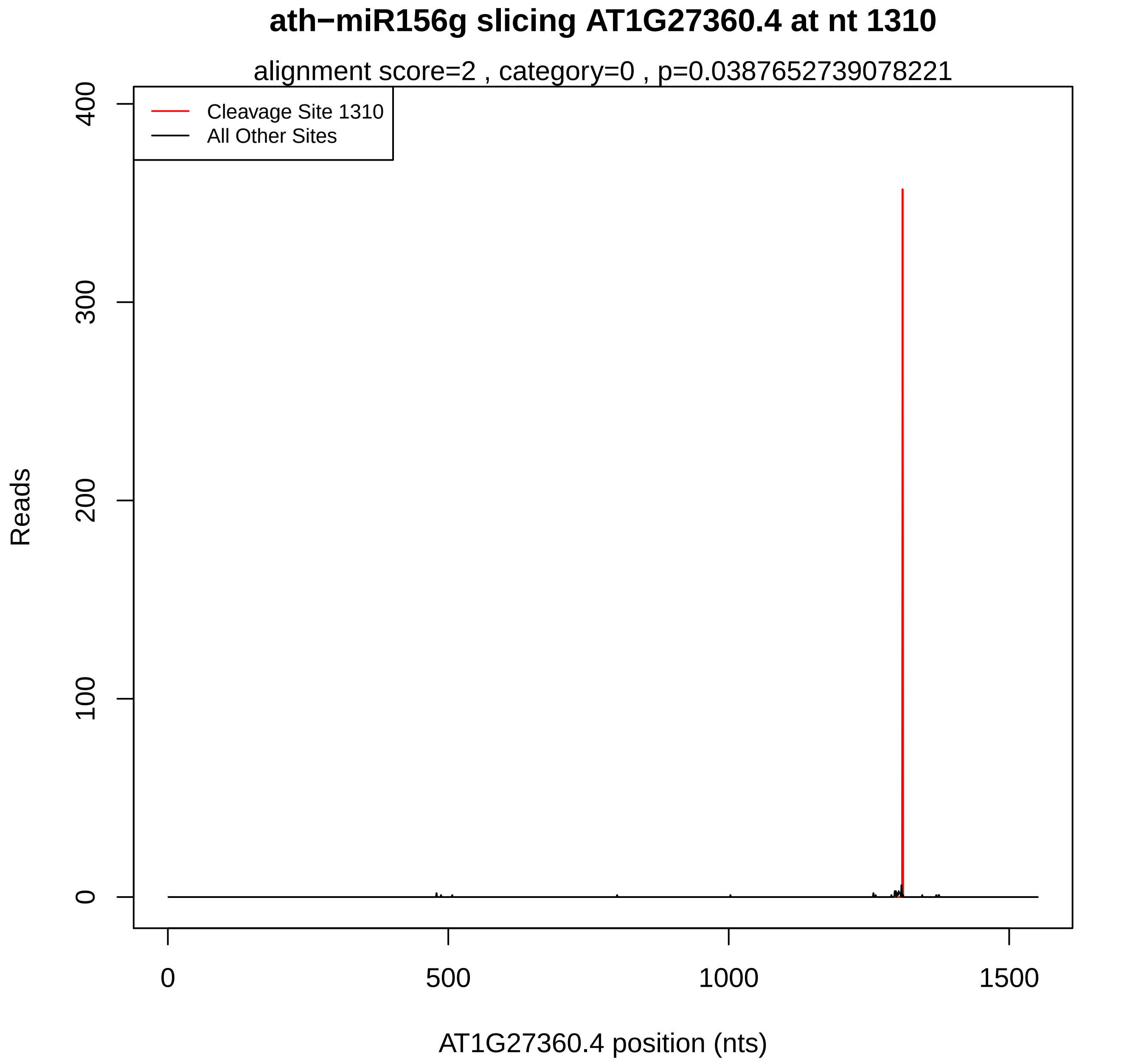
ath−miR156g slicing AT1G27360.4 at nt 1310
alignment score=2 , category=0 , p=0.0387652739078221
Cleavage Site 1310
All Other Sites
Reads
0
100
200
300
400
0
500
1000
1500
AT1G27360.4 position (nts)

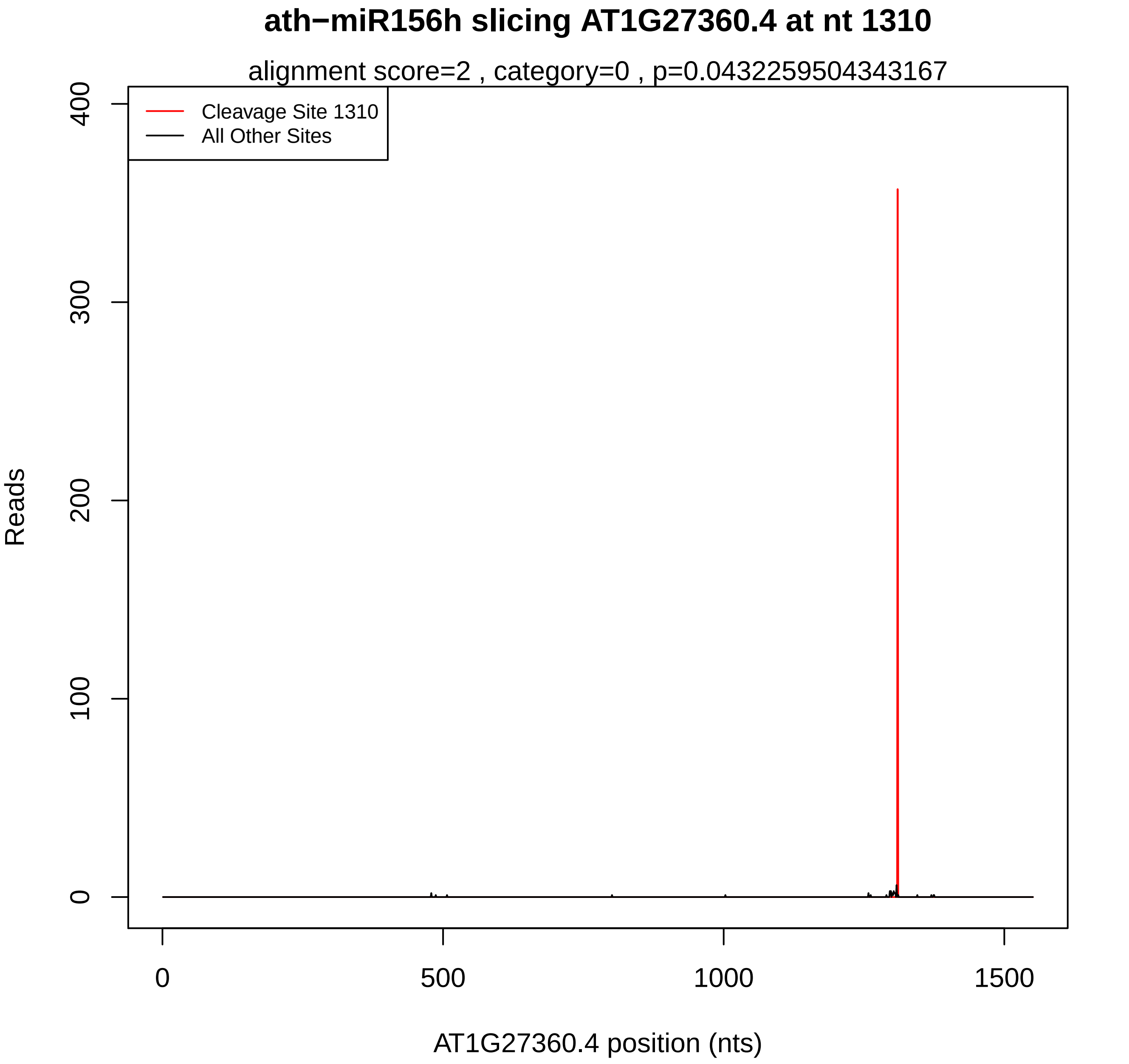
ath−miR156h slicing AT1G27360.4 at nt 1310
alignment score=2 , category=0 , p=0.0432259504343167
Cleavage Site 1310
All Other Sites
Reads
0
100
200
300
400
0
500
1000
1500
AT1G27360.4 position (nts)

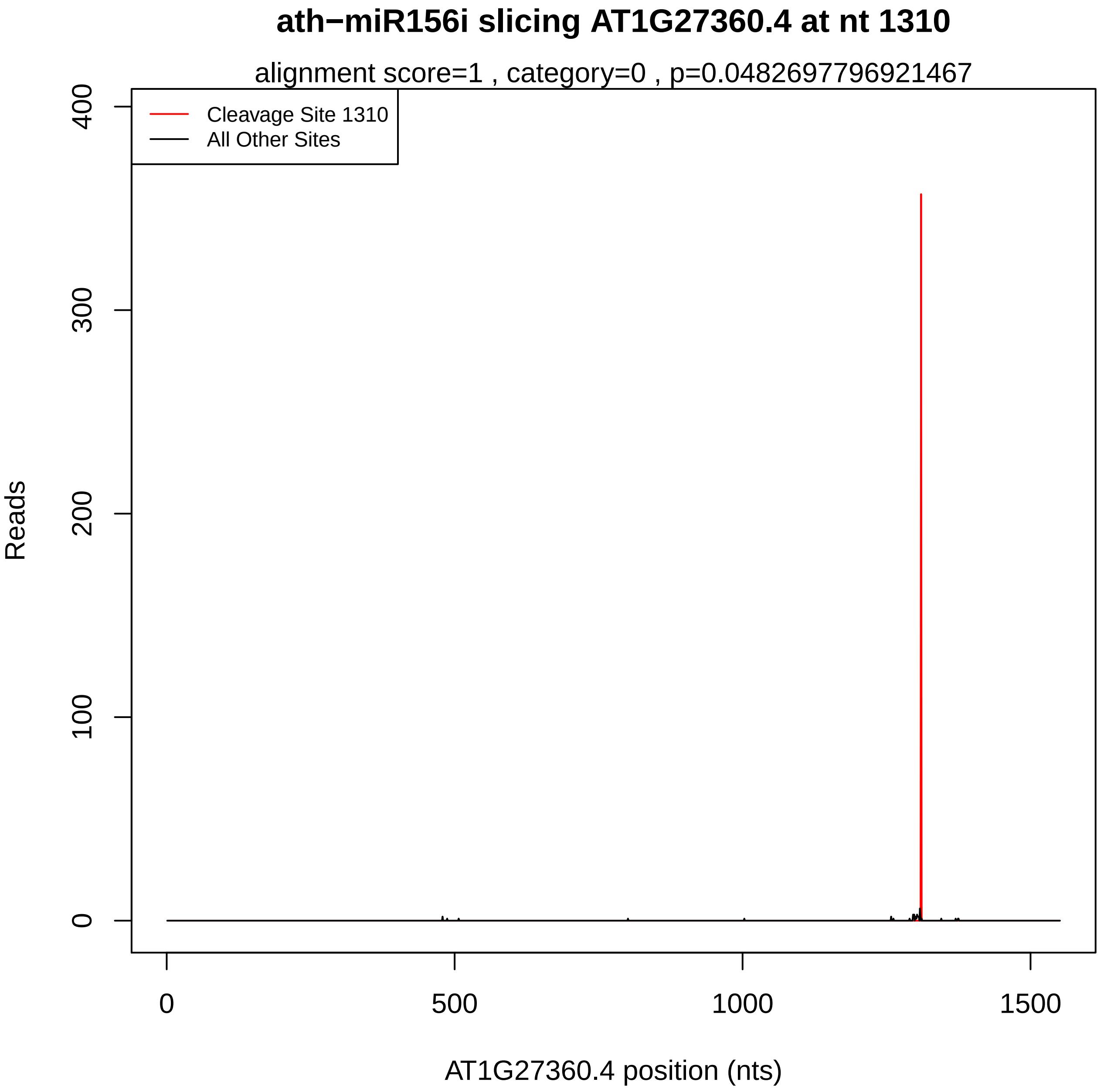

ath−miR156i slicing AT1G27360.4 at nt 1310
alignment score=1 , category=0 , p=0.0482697796921467
Cleavage Site 1310
All Other Sites
Reads
0
100
200
300
400
0
500
1000
1500
AT1G27360.4 position (nts)

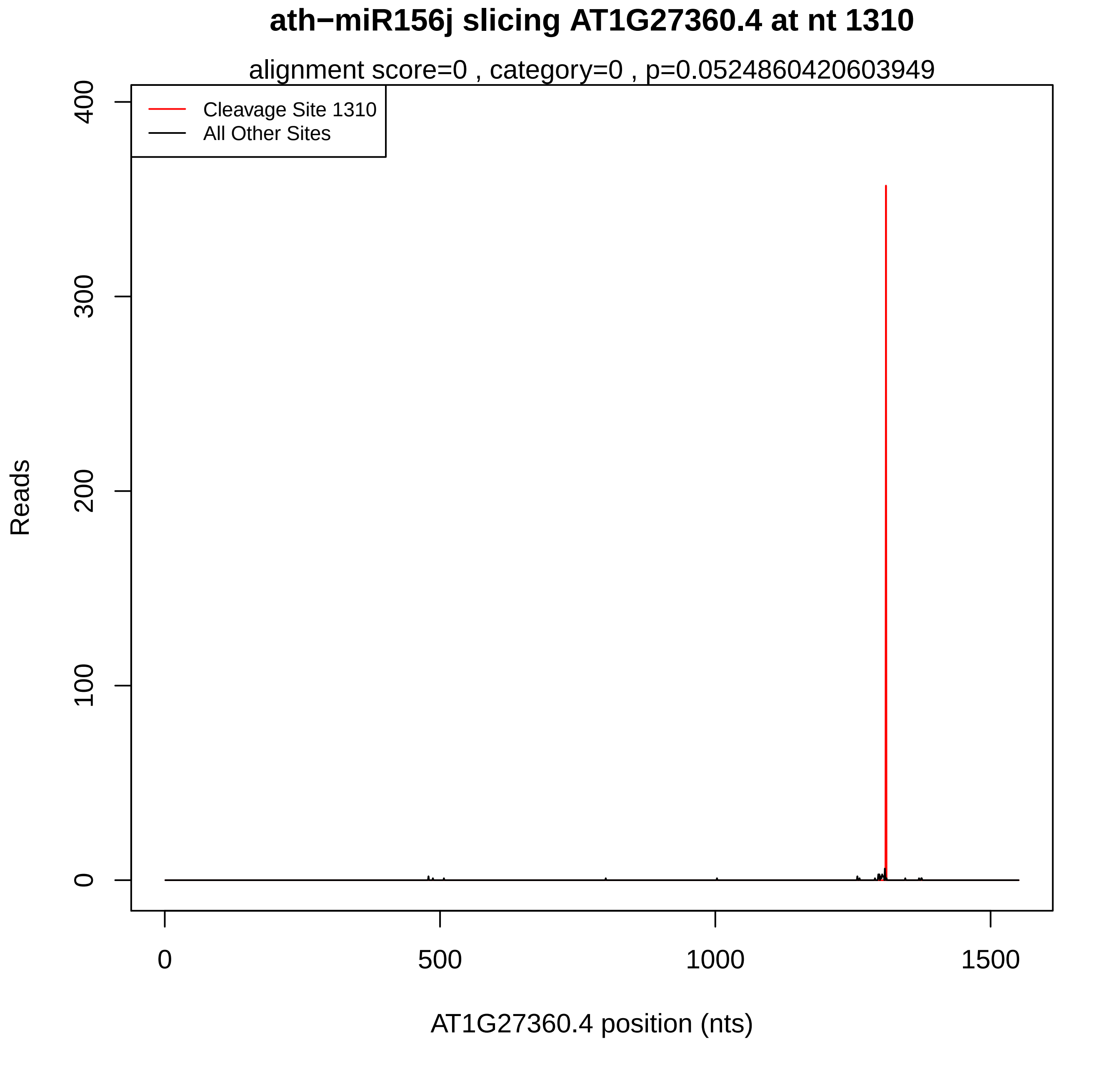
ath−miR156j slicing AT1G27360.4 at nt 1310
alignment score=0 , category=0 , p=0.0524860420603949
Cleavage Site 1310
All Other Sites
Reads
0
100
200
300
400
0
500
1000
1500
AT1G27360.4 position (nts)

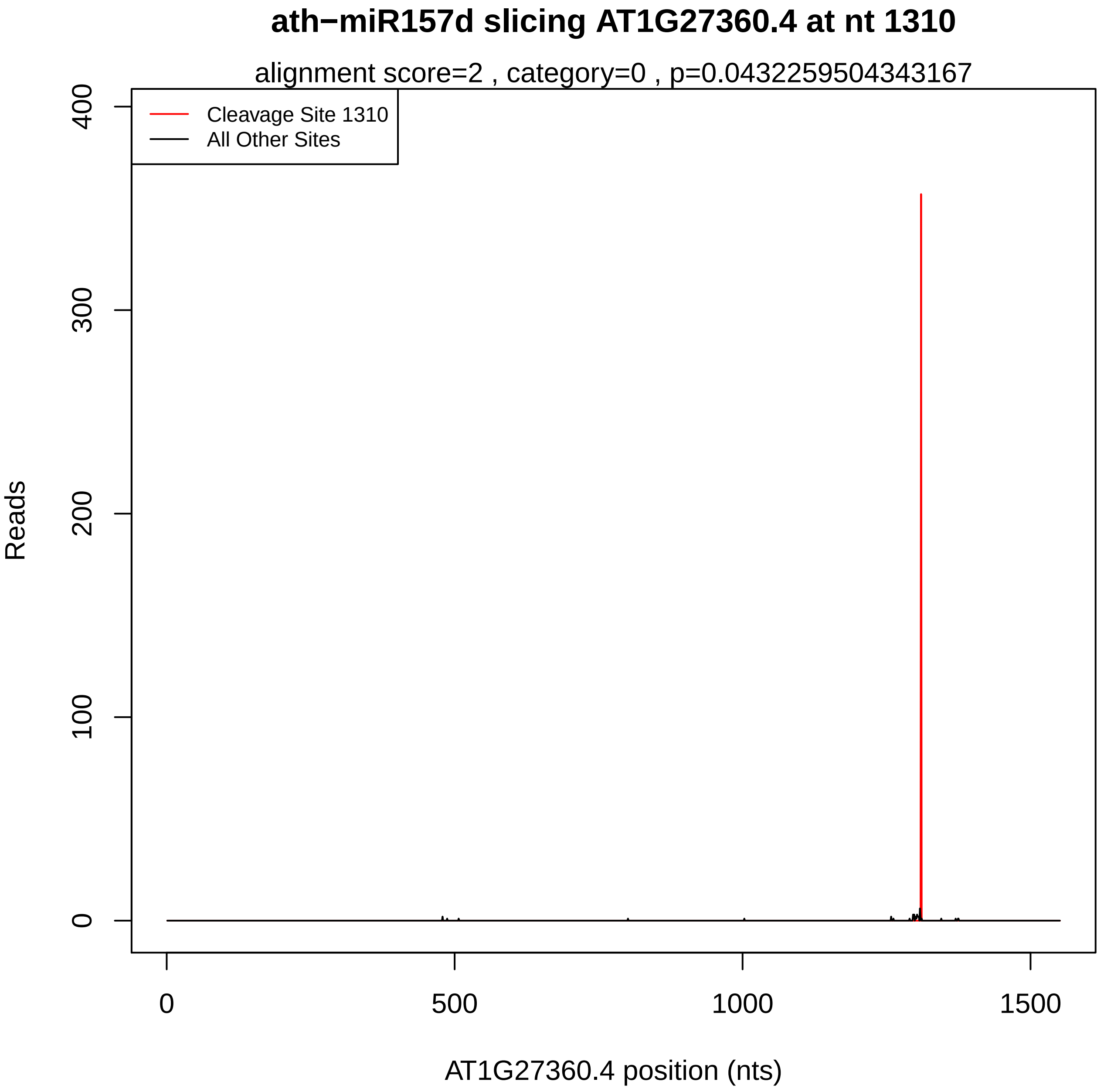
ath−miR157d slicing AT1G27360.4 at nt 1310
alignment score=2 , category=0 , p=0.0432259504343167
Cleavage Site 1310
All Other Sites
Reads
0
100
200
300
400
0
500
1000
1500
AT1G27360.4 position (nts)

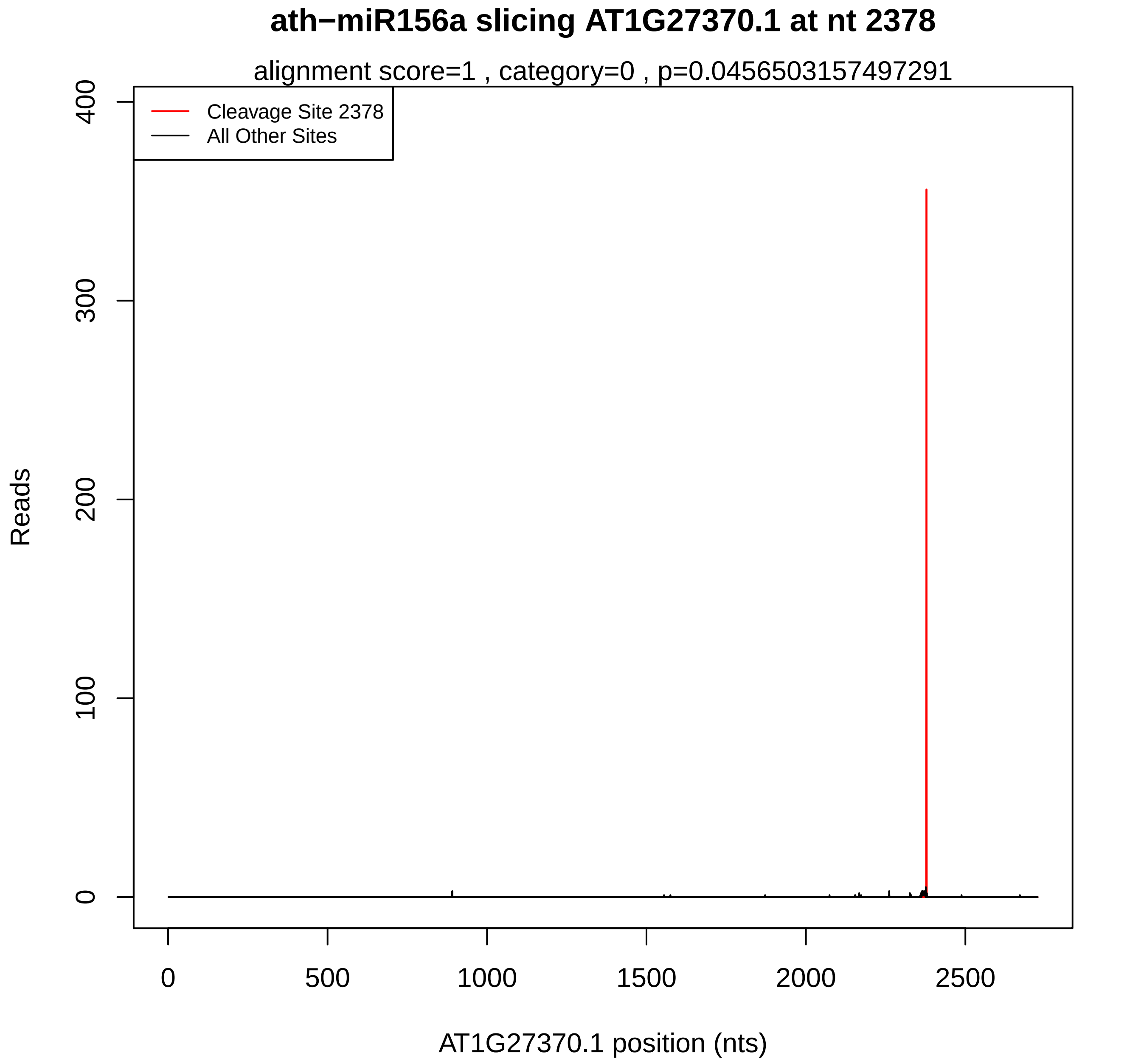

ath−miR156a slicing AT1G27370.1 at nt 2378
alignment score=1 , category=0 , p=0.0456503157497291
Cleavage Site 2378
All Other Sites
Reads
0
100
200
300
400
0
500
1000
1500
2000
2500
AT1G27370.1 position (nts)

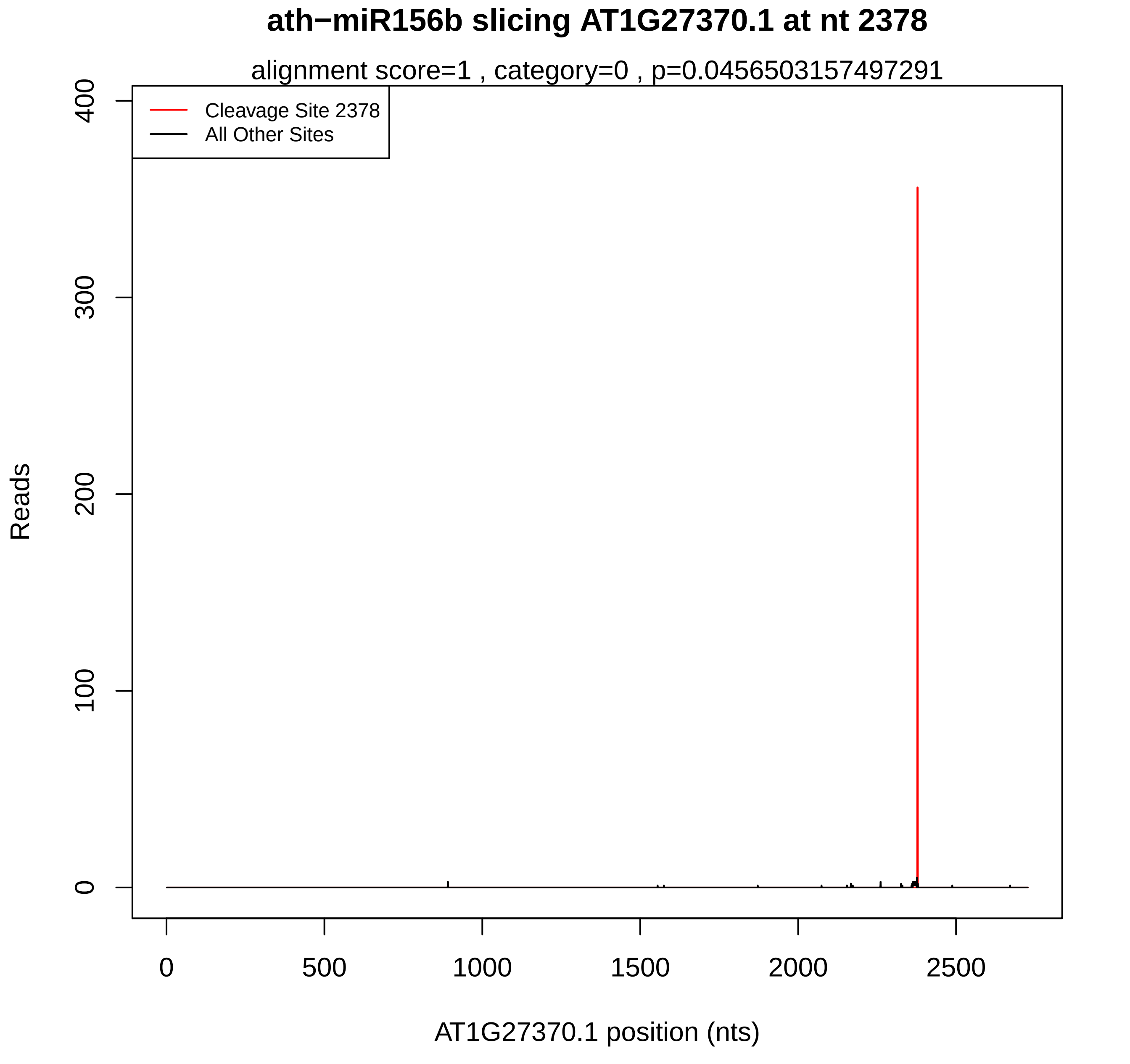

ath−miR156b slicing AT1G27370.1 at nt 2378
alignment score=1 , category=0 , p=0.0456503157497291
Cleavage Site 2378
All Other Sites
Reads
0
100
200
300
400
0
500
1000
1500
2000
2500
AT1G27370.1 position (nts)

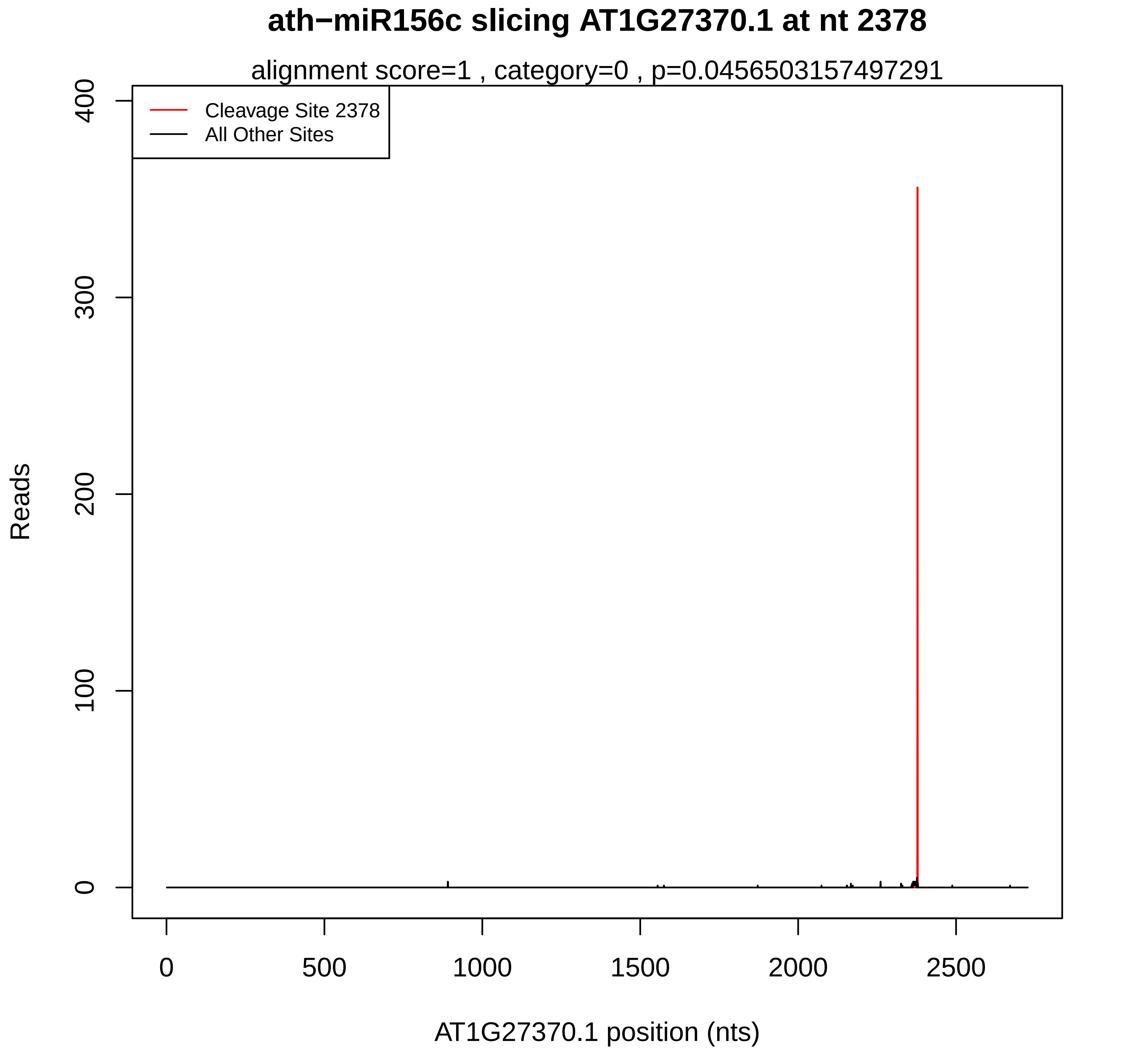

ath−miR156c slicing AT1G27370.1 at nt 2378
alignment score=1 , category=0 , p=0.0456503157497291
Cleavage Site 2378
All Other Sites
Reads
0
100
200
300
400
0
500
1000
1500
2000
2500
AT1G27370.1 position (nts)

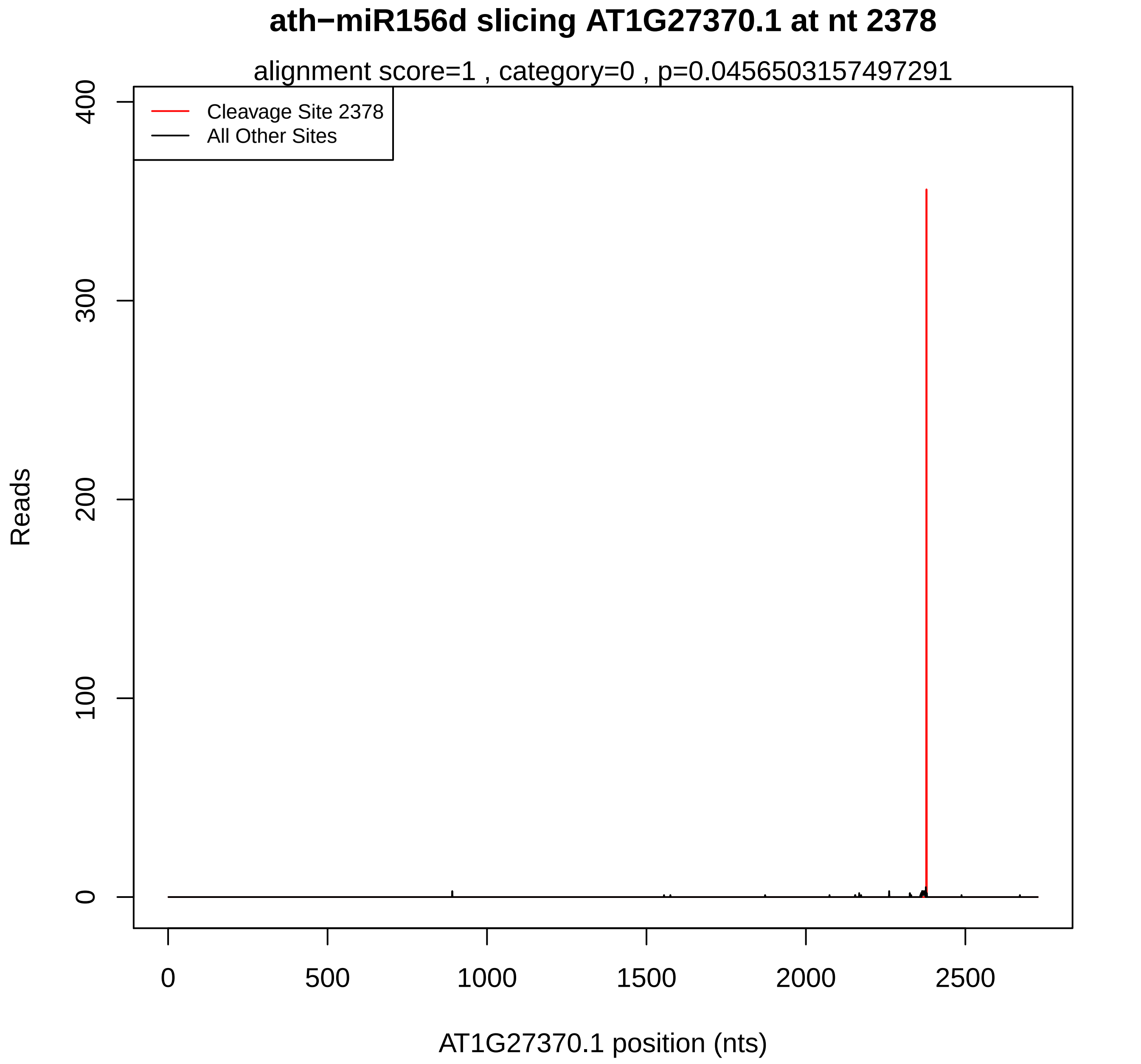
ath−miR156d slicing AT1G27370.1 at nt 2378
alignment score=1 , category=0 , p=0.0456503157497291
Cleavage Site 2378
All Other Sites
Reads
0
100
200
300
400
0
500
1000
1500
2000
2500
AT1G27370.1 position (nts)

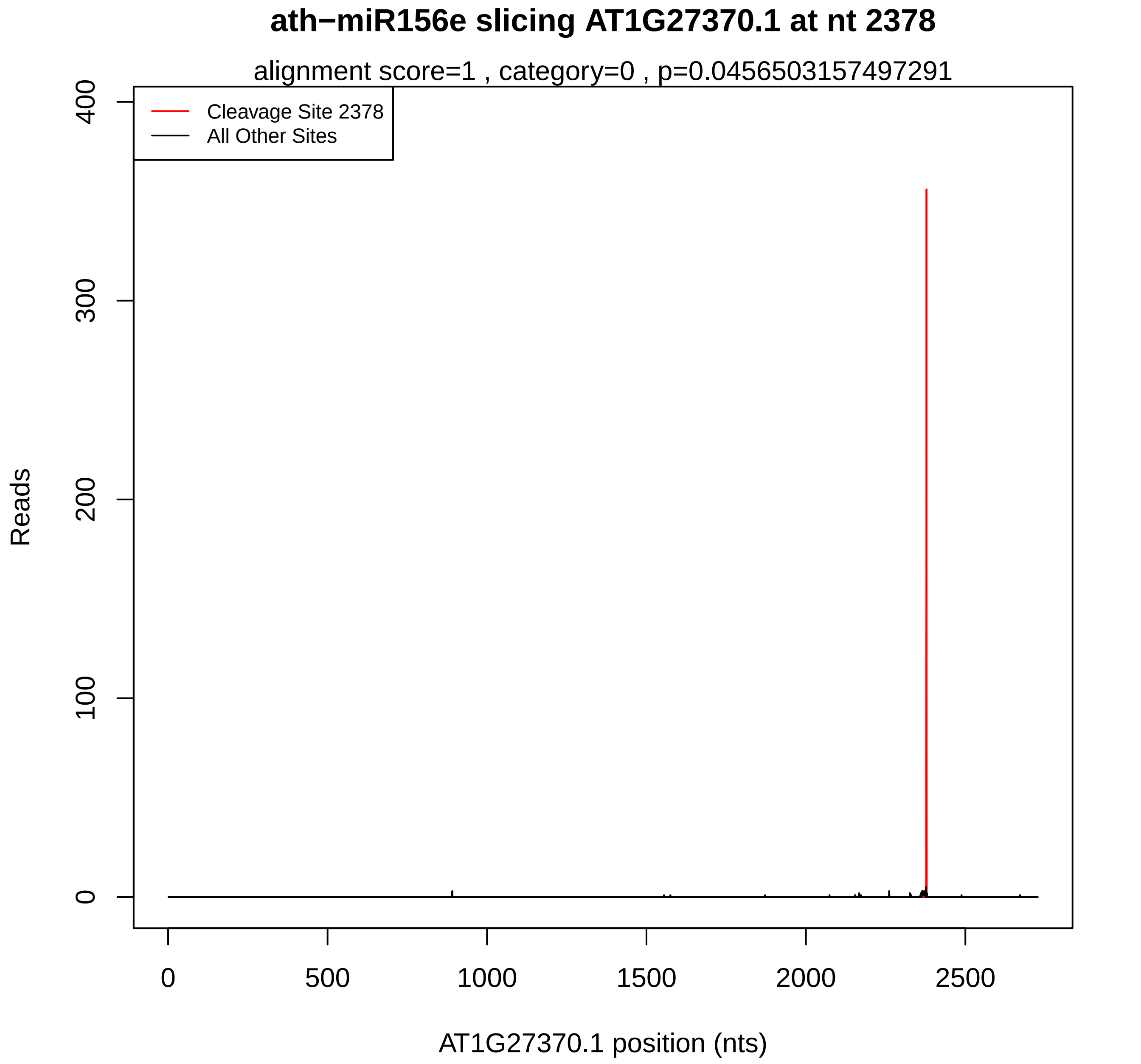

ath−miR156e slicing AT1G27370.1 at nt 2378
alignment score=1 , category=0 , p=0.0456503157497291
Cleavage Site 2378
All Other Sites
Reads
0
100
200
300
400
0
500
1000
1500
2000
2500
AT1G27370.1 position (nts)

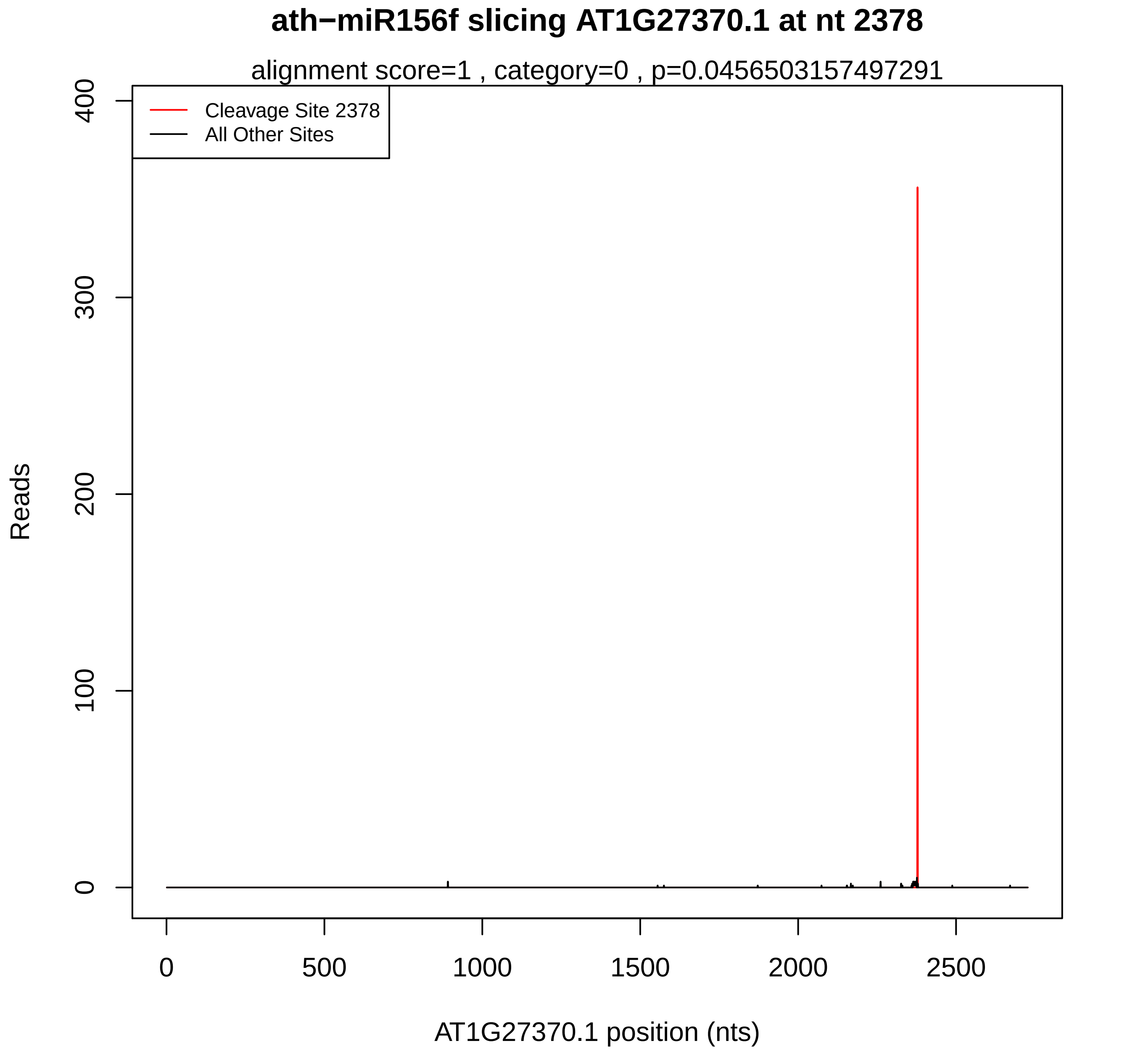

ath−miR156f slicing AT1G27370.1 at nt 2378
alignment score=1 , category=0 , p=0.0456503157497291
Cleavage Site 2378
All Other Sites
Reads
0
100
200
300
400
0
500
1000
1500
2000
2500
AT1G27370.1 position (nts)

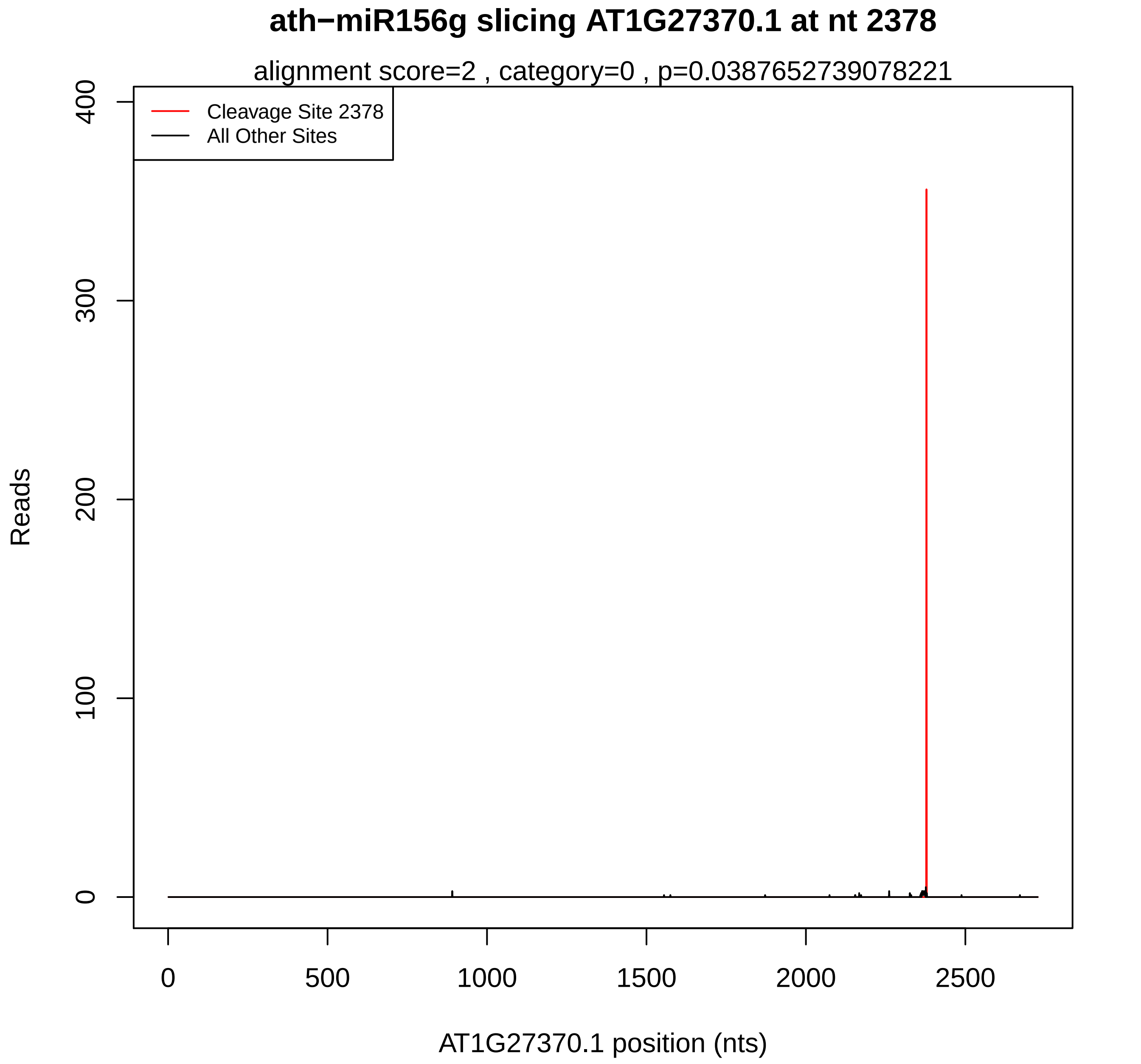
ath−miR156g slicing AT1G27370.1 at nt 2378
alignment score=2 , category=0 , p=0.0387652739078221
Cleavage Site 2378
All Other Sites
Reads
0
100
200
300
400
0
500
1000
1500
2000
2500
AT1G27370.1 position (nts)

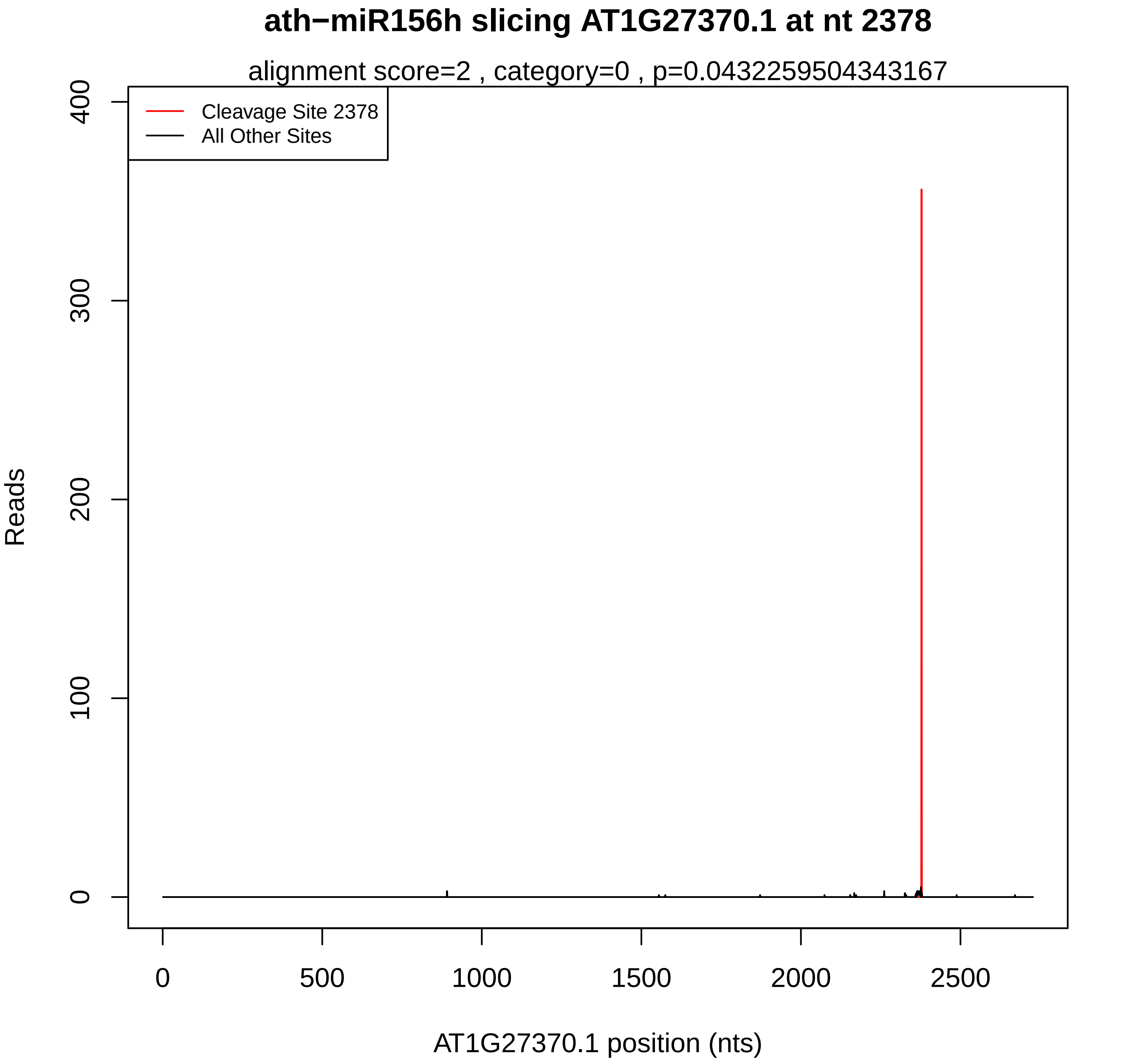
ath−miR156h slicing AT1G27370.1 at nt 2378
alignment score=2 , category=0 , p=0.0432259504343167
Cleavage Site 2378
All Other Sites
Reads
0
100
200
300
400
0
500
1000
1500
2000
2500
AT1G27370.1 position (nts)

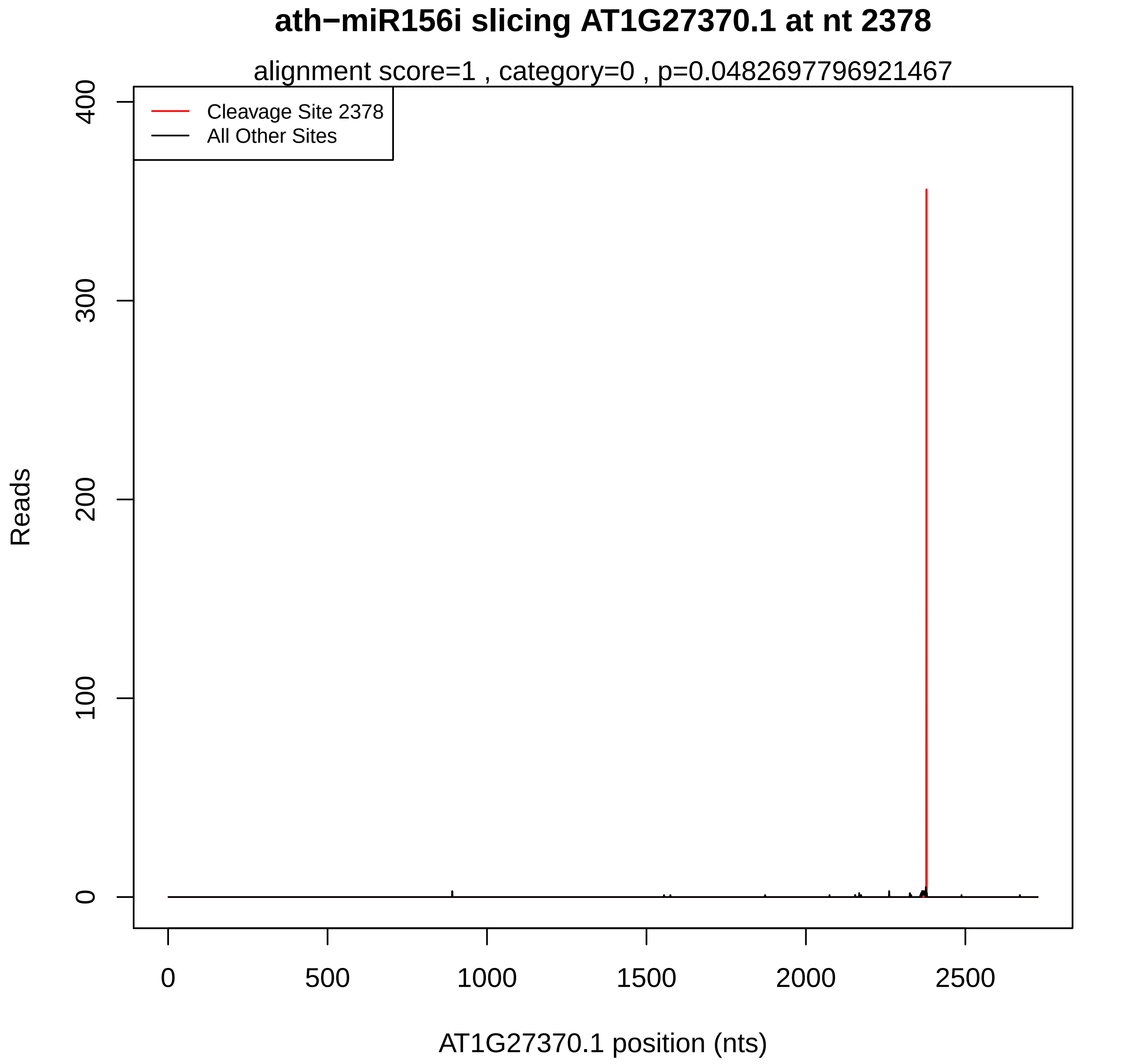
ath−miR156i slicing AT1G27370.1 at nt 2378
alignment score=1 , category=0 , p=0.0482697796921467
Cleavage Site 2378
All Other Sites
Reads
0
100
200
300
400
0
500
1000
1500
2000
2500
AT1G27370.1 position (nts)

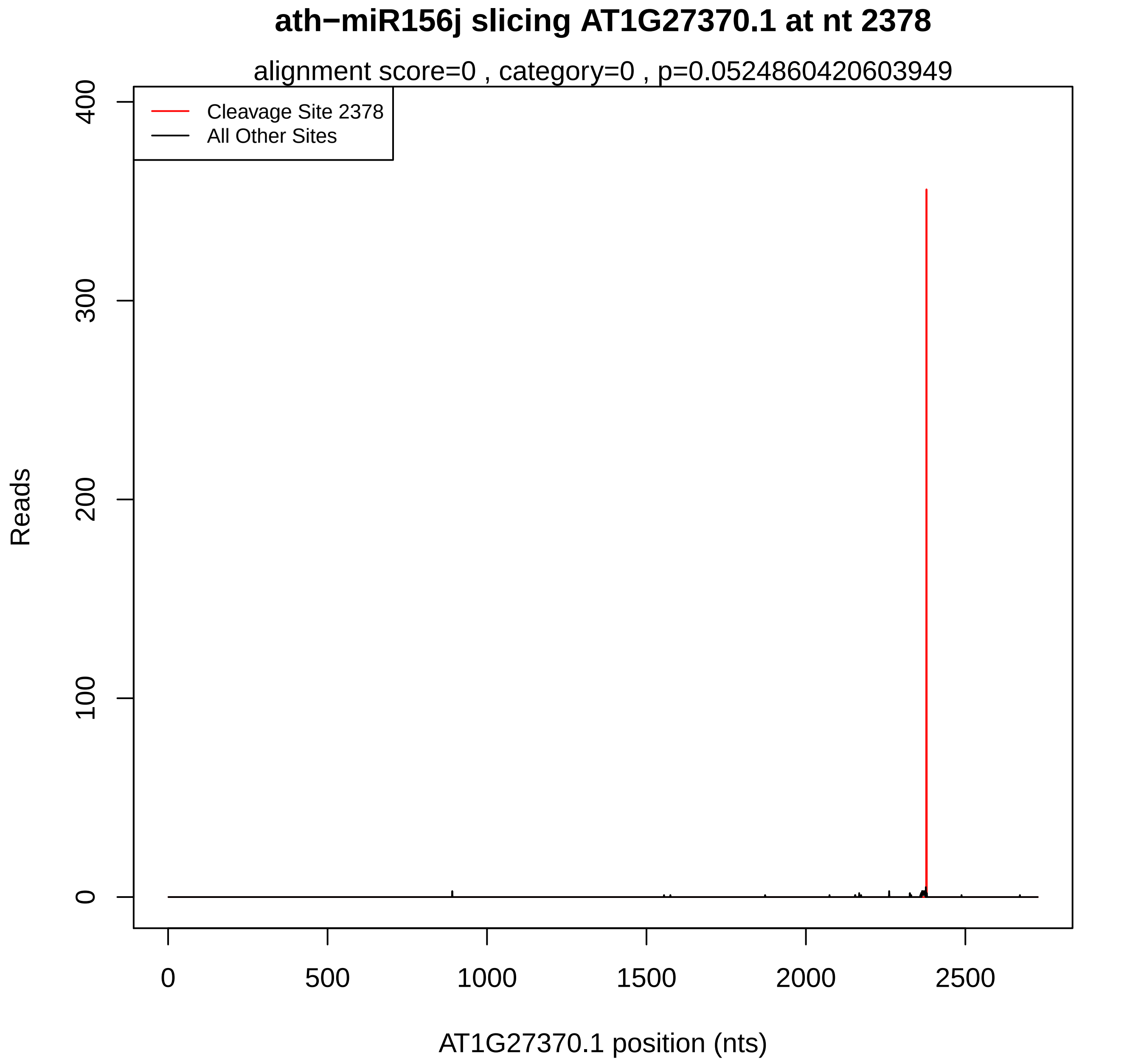
ath−miR156j slicing AT1G27370.1 at nt 2378
alignment score=0 , category=0 , p=0.0524860420603949
Cleavage Site 2378
All Other Sites
Reads
0
100
200
300
400
0
500
1000
1500
2000
2500
AT1G27370.1 position (nts)

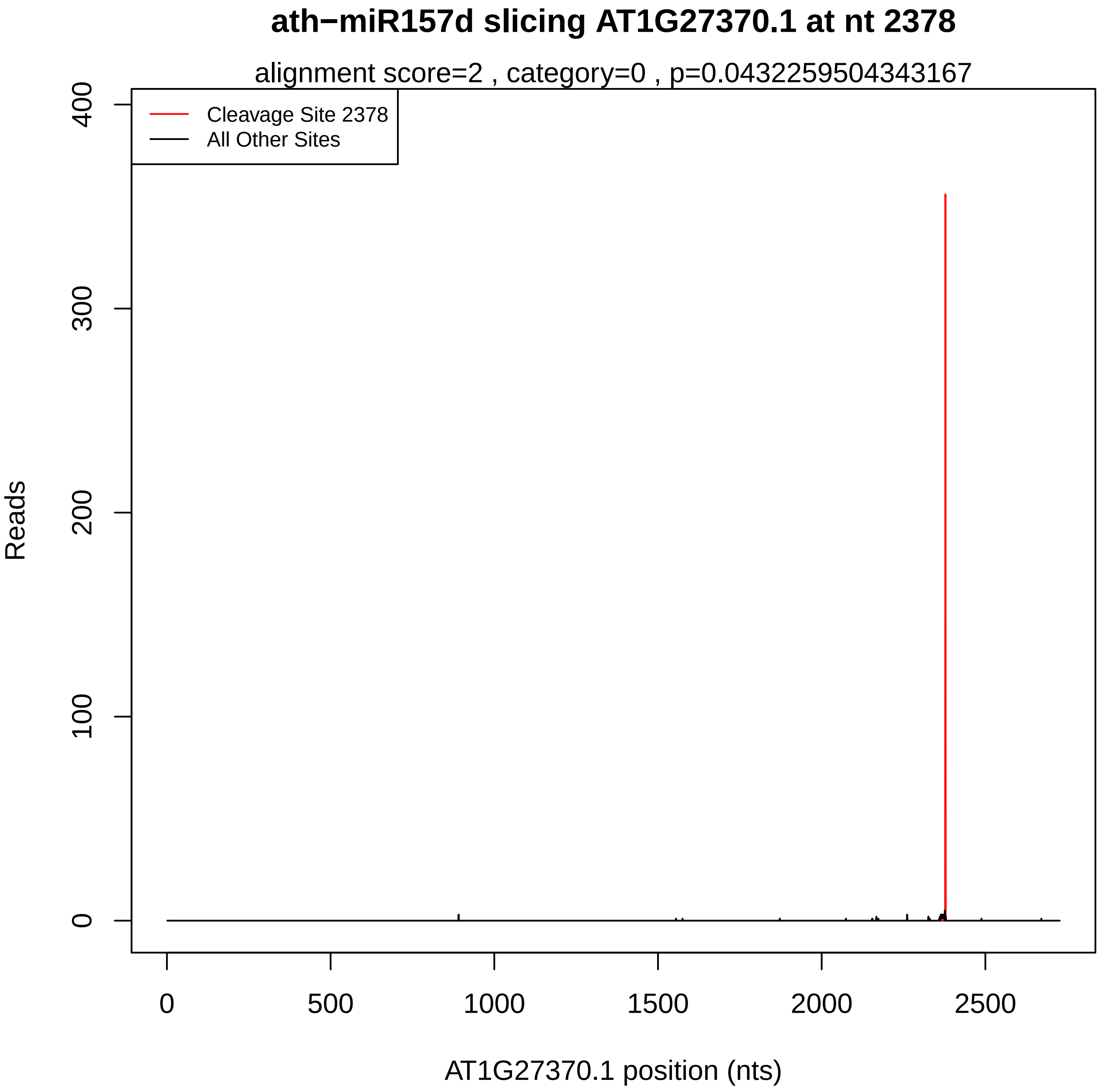

ath−miR157d slicing AT1G27370.1 at nt 2378
alignment score=2 , category=0 , p=0.0432259504343167
Cleavage Site 2378
All Other Sites
Reads
0
100
200
300
400
0
500
1000
1500
2000
2500
AT1G27370.1 position (nts)

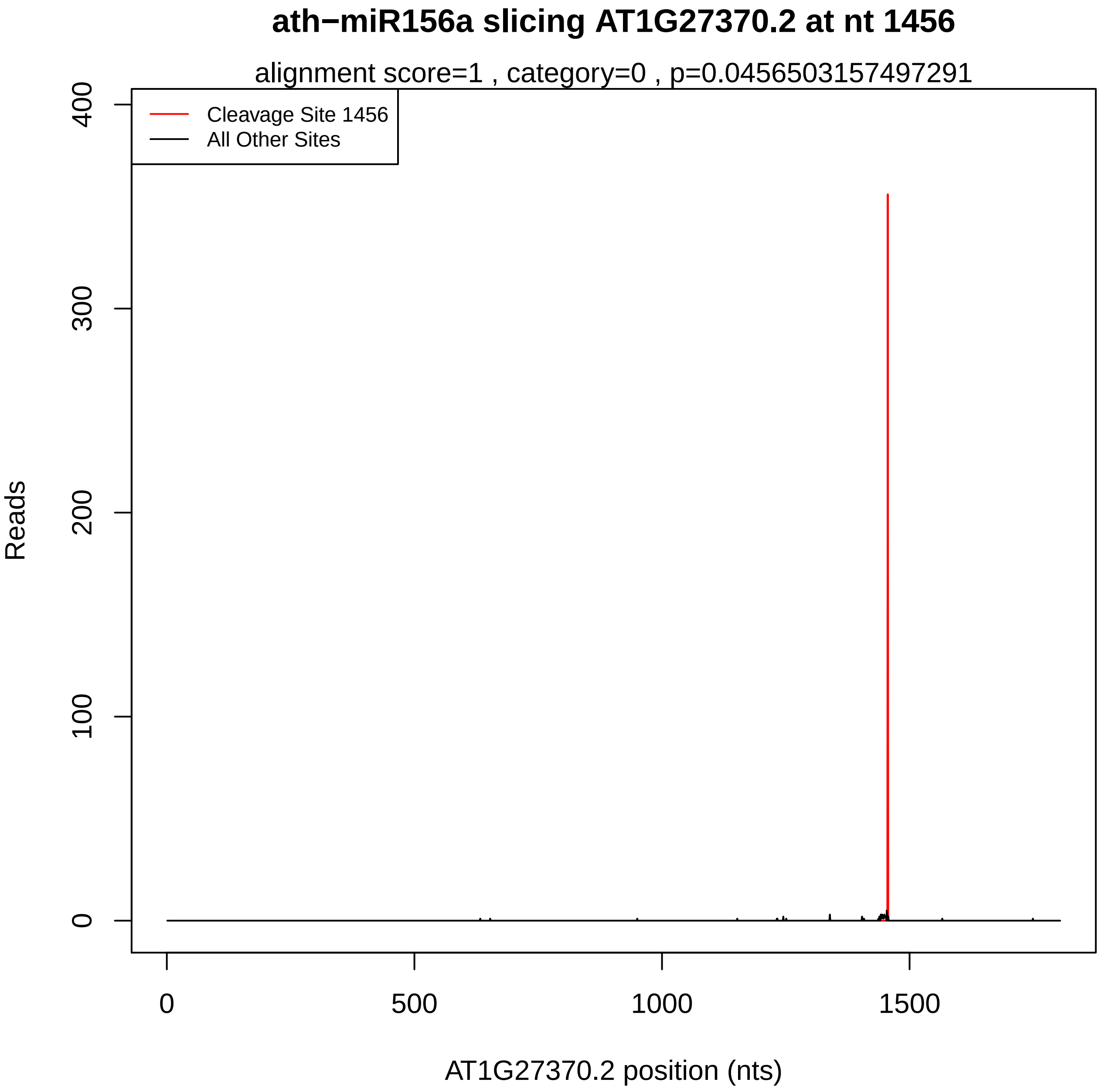
ath−miR156a slicing AT1G27370.2 at nt 1456
alignment score=1 , category=0 , p=0.0456503157497291
Cleavage Site 1456
All Other Sites
Reads
0
100
200
300
400
0
500
1000
1500
AT1G27370.2 position (nts)

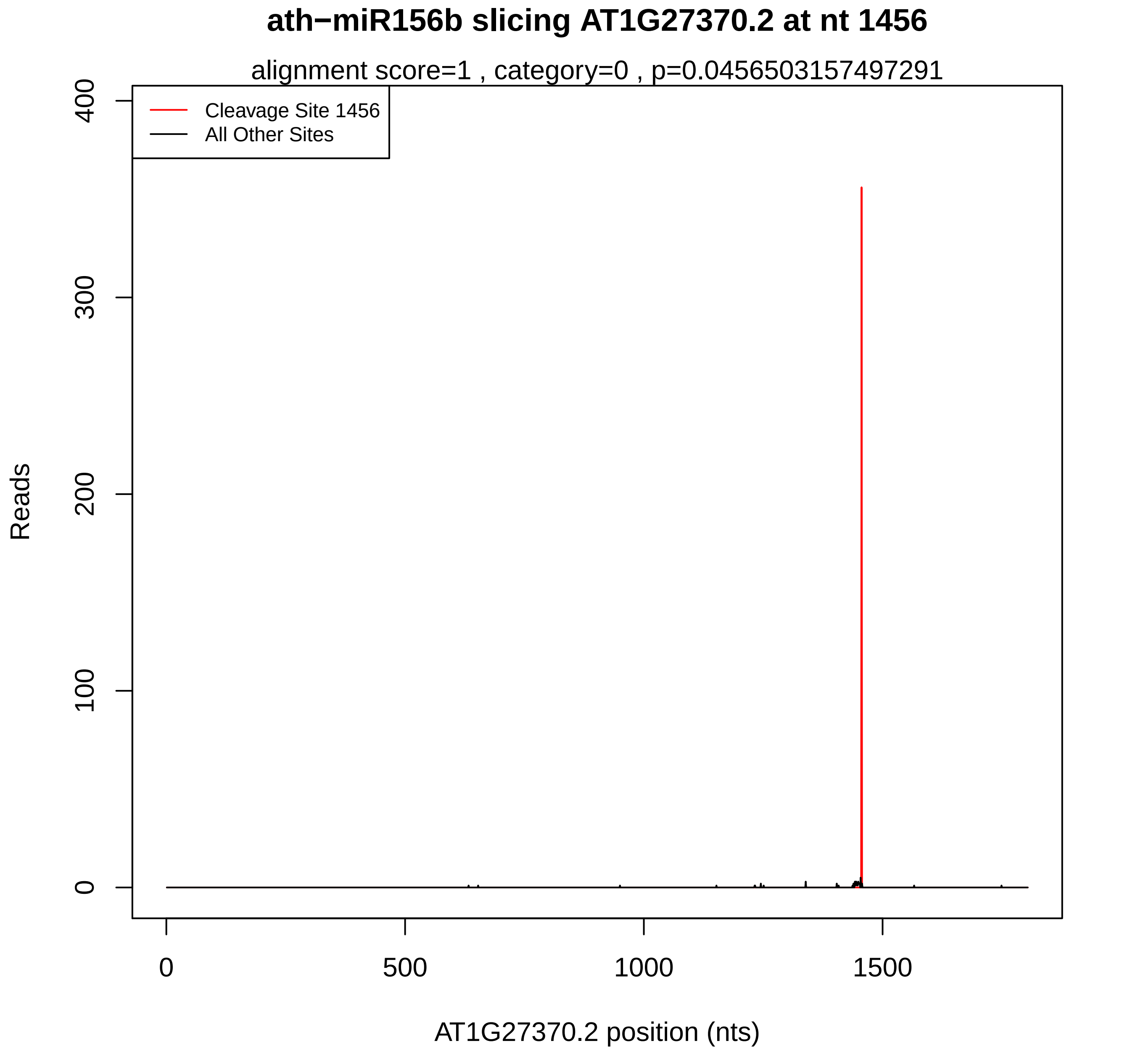

ath−miR156b slicing AT1G27370.2 at nt 1456
alignment score=1 , category=0 , p=0.0456503157497291
Cleavage Site 1456
All Other Sites
Reads
0
100
200
300
400
0
500
1000
1500
AT1G27370.2 position (nts)

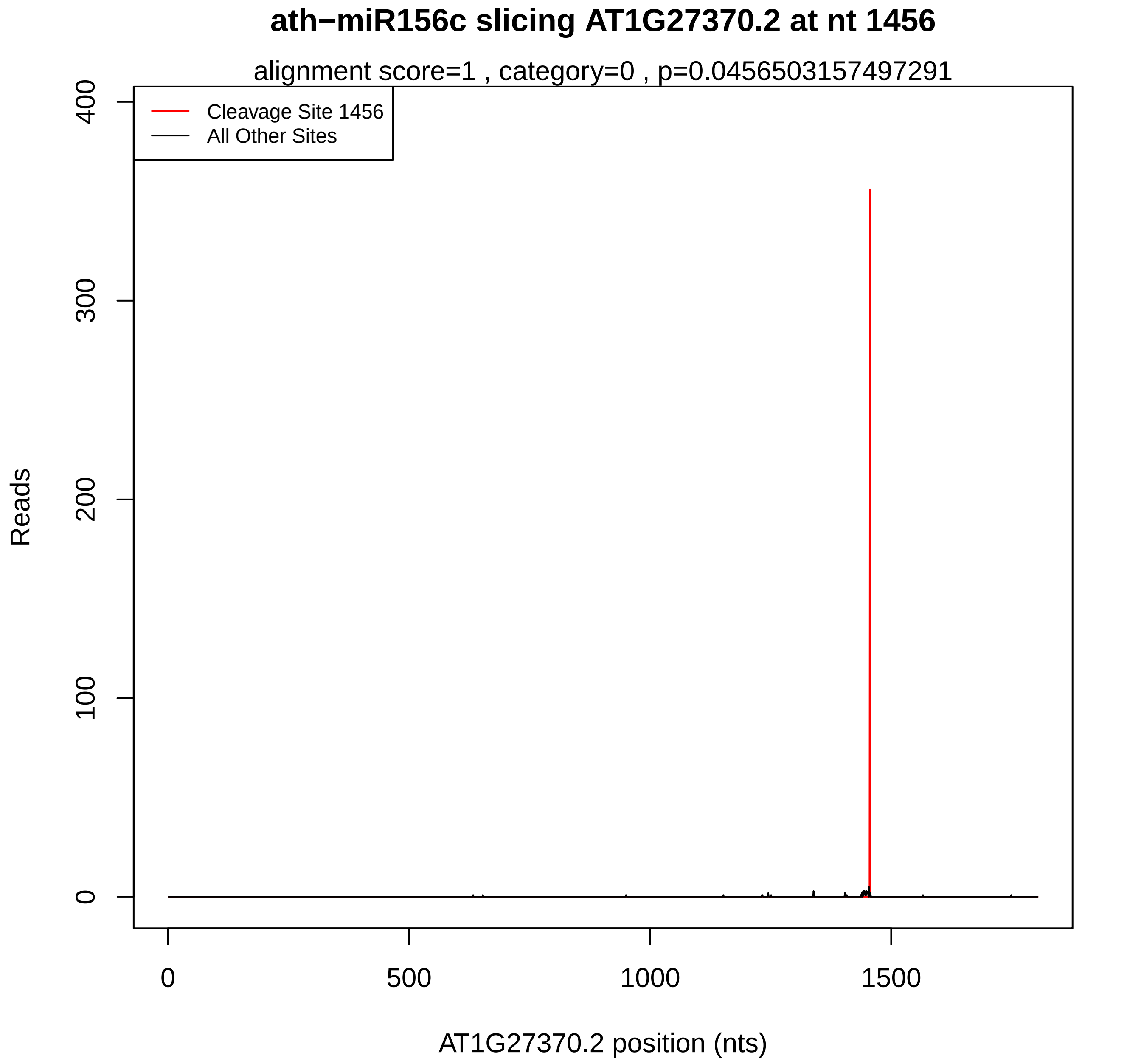
ath−miR156c slicing AT1G27370.2 at nt 1456
alignment score=1 , category=0 , p=0.0456503157497291
Cleavage Site 1456
All Other Sites
Reads
0
100
200
300
400
0
500
1000
1500
AT1G27370.2 position (nts)

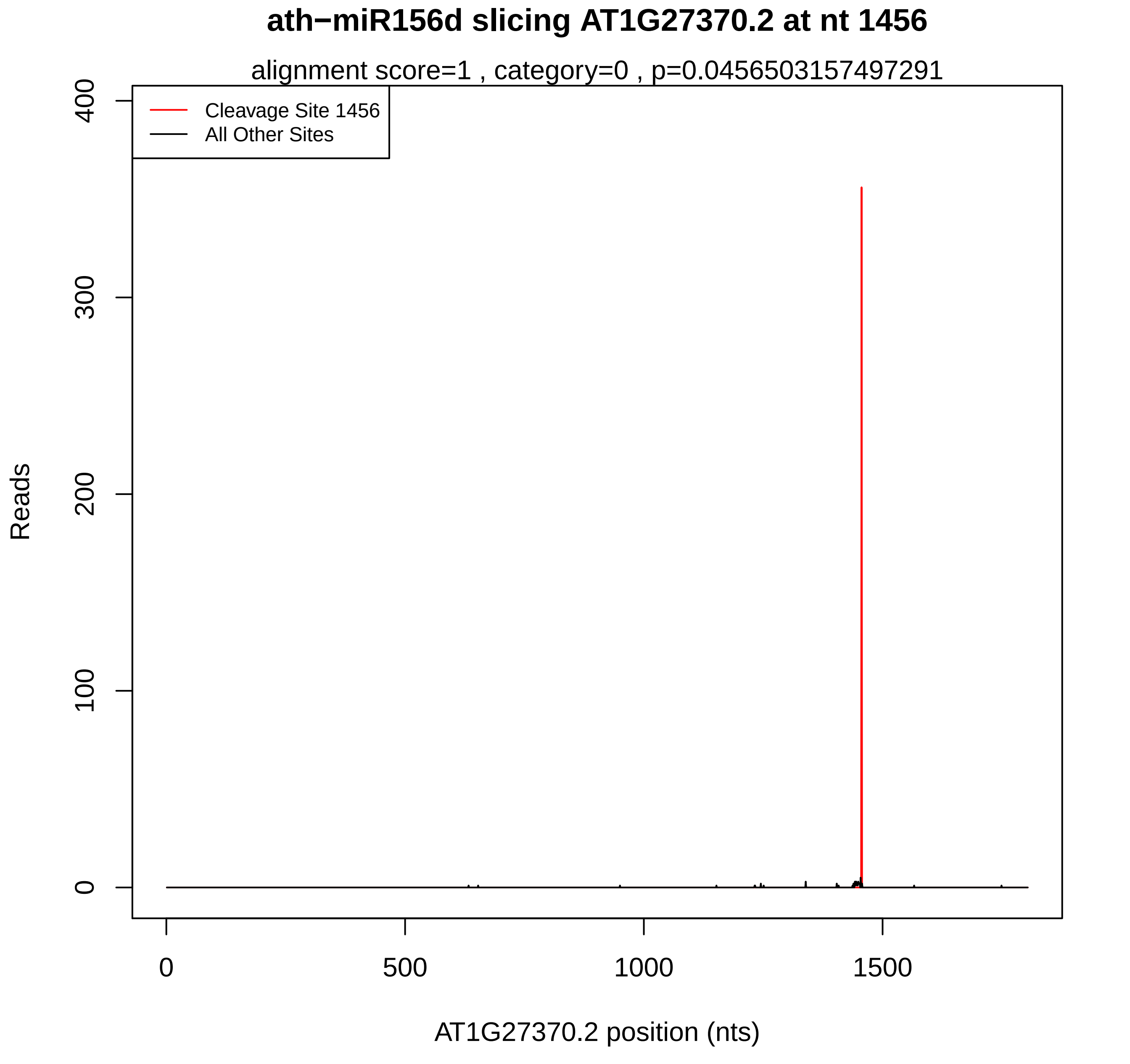
ath−miR156d slicing AT1G27370.2 at nt 1456
alignment score=1 , category=0 , p=0.0456503157497291
Cleavage Site 1456
All Other Sites
Reads
0
100
200
300
400
0
500
1000
1500
AT1G27370.2 position (nts)

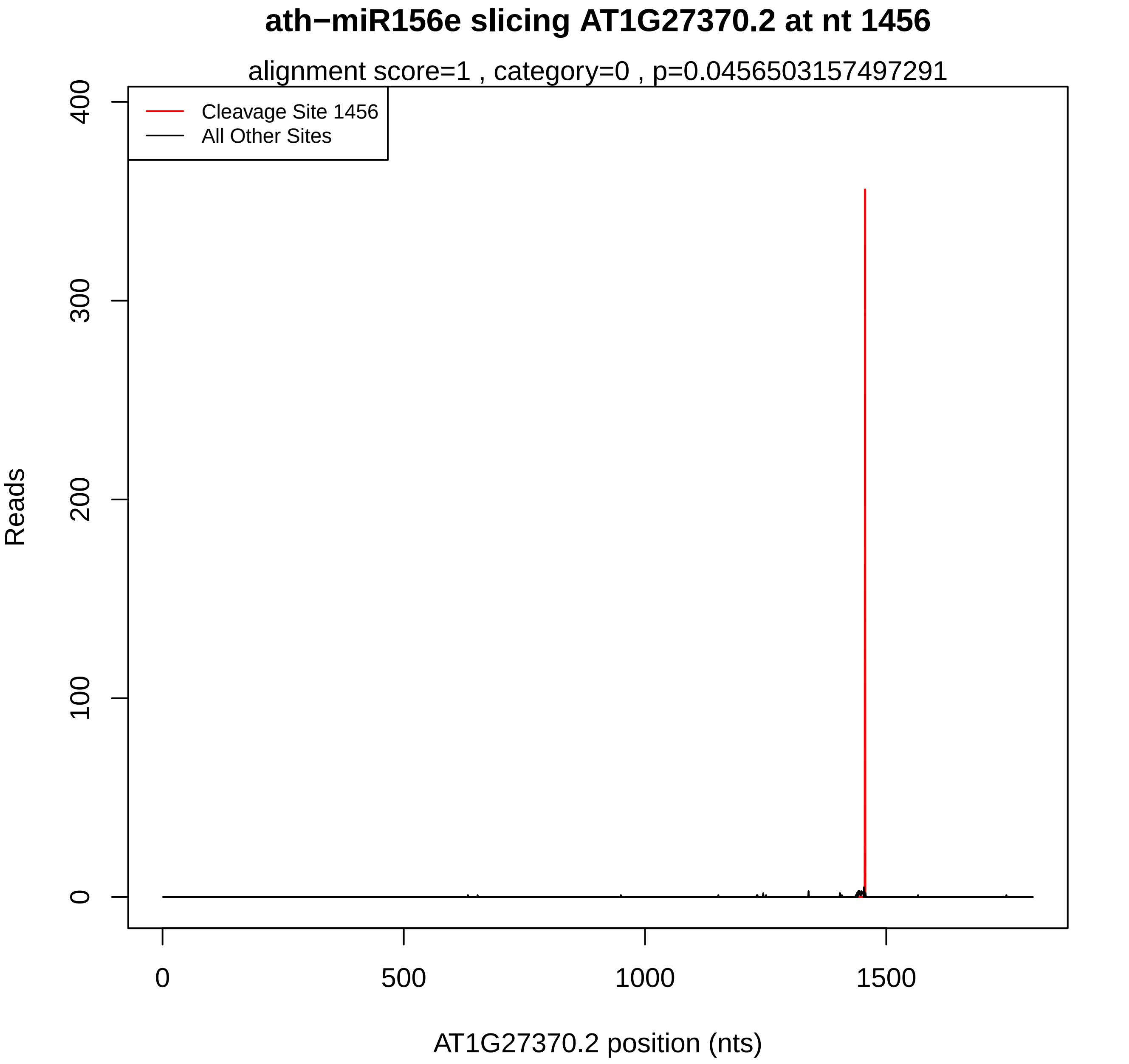
ath−miR156e slicing AT1G27370.2 at nt 1456
alignment score=1 , category=0 , p=0.0456503157497291
Cleavage Site 1456
All Other Sites
Reads
0
100
200
300
400
0
500
1000
1500
AT1G27370.2 position (nts)

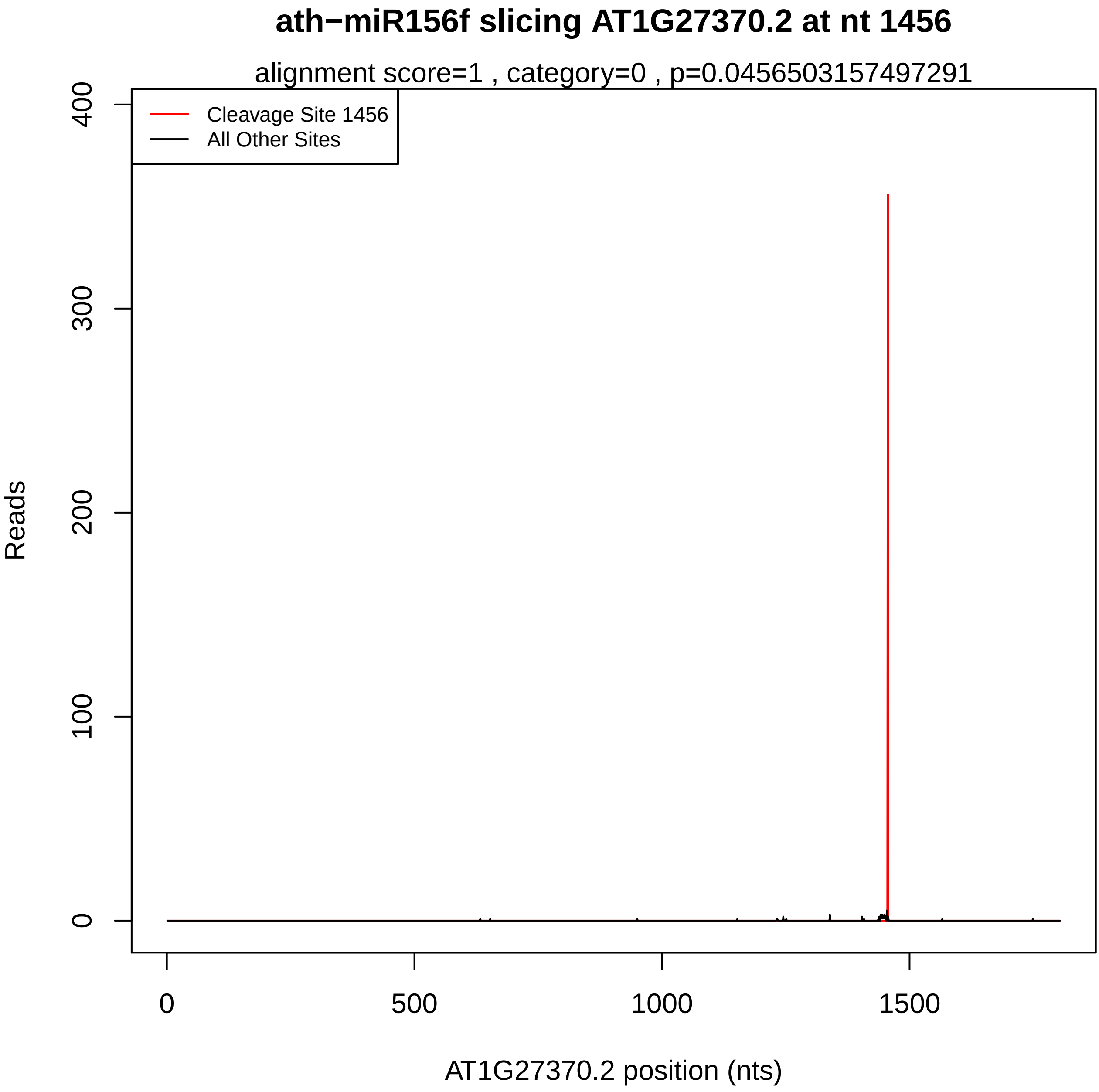
ath−miR156f slicing AT1G27370.2 at nt 1456
alignment score=1 , category=0 , p=0.0456503157497291
Cleavage Site 1456
All Other Sites
Reads
0
100
200
300
400
0
500
1000
1500
AT1G27370.2 position (nts)

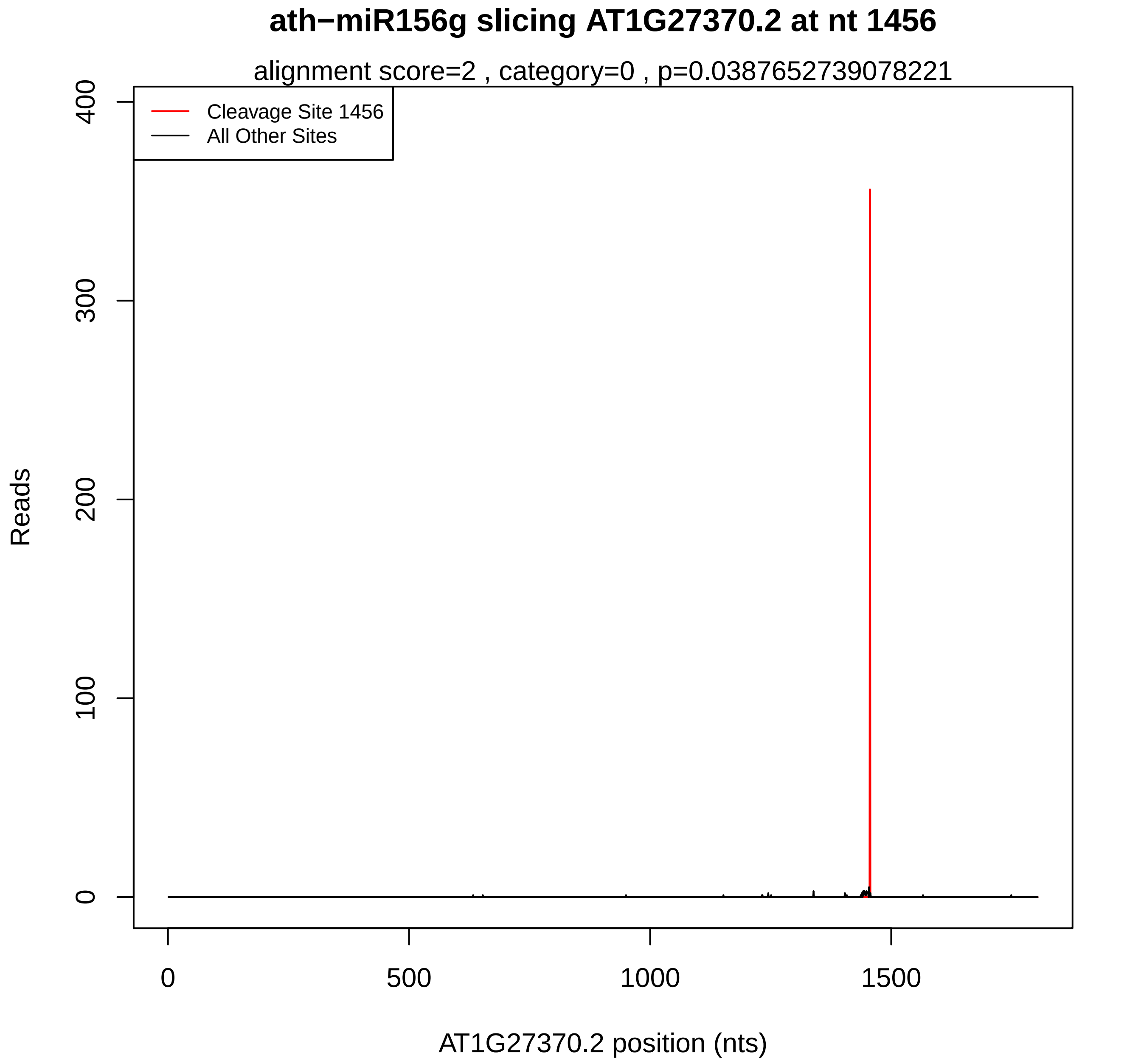
ath−miR156g slicing AT1G27370.2 at nt 1456
alignment score=2 , category=0 , p=0.0387652739078221
Cleavage Site 1456
All Other Sites
Reads
0
100
200
300
400
0
500
1000
1500
AT1G27370.2 position (nts)

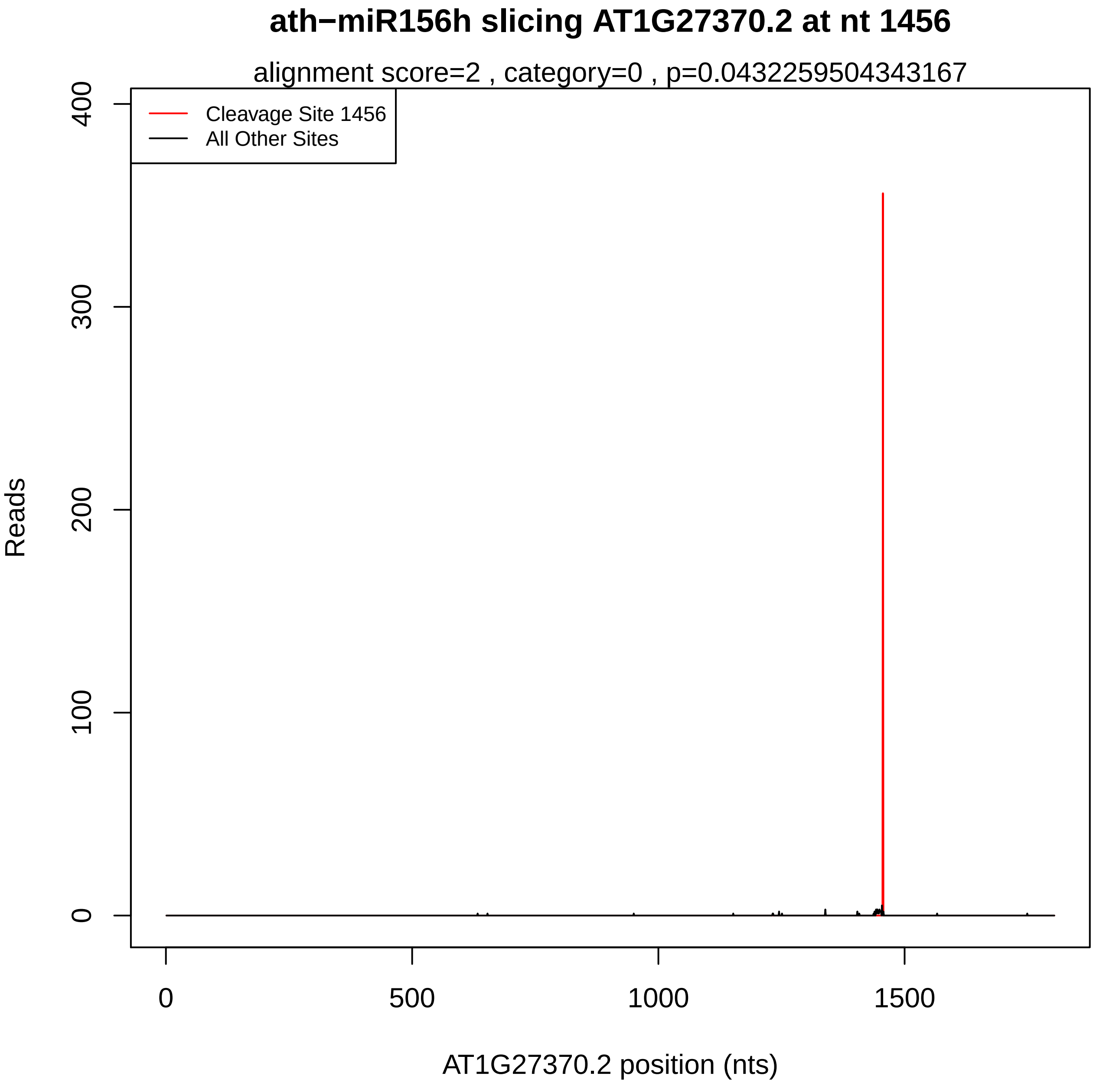

ath−miR156h slicing AT1G27370.2 at nt 1456
alignment score=2 , category=0 , p=0.0432259504343167
Cleavage Site 1456
All Other Sites
Reads
0
100
200
300
400
0
500
1000
1500
AT1G27370.2 position (nts)

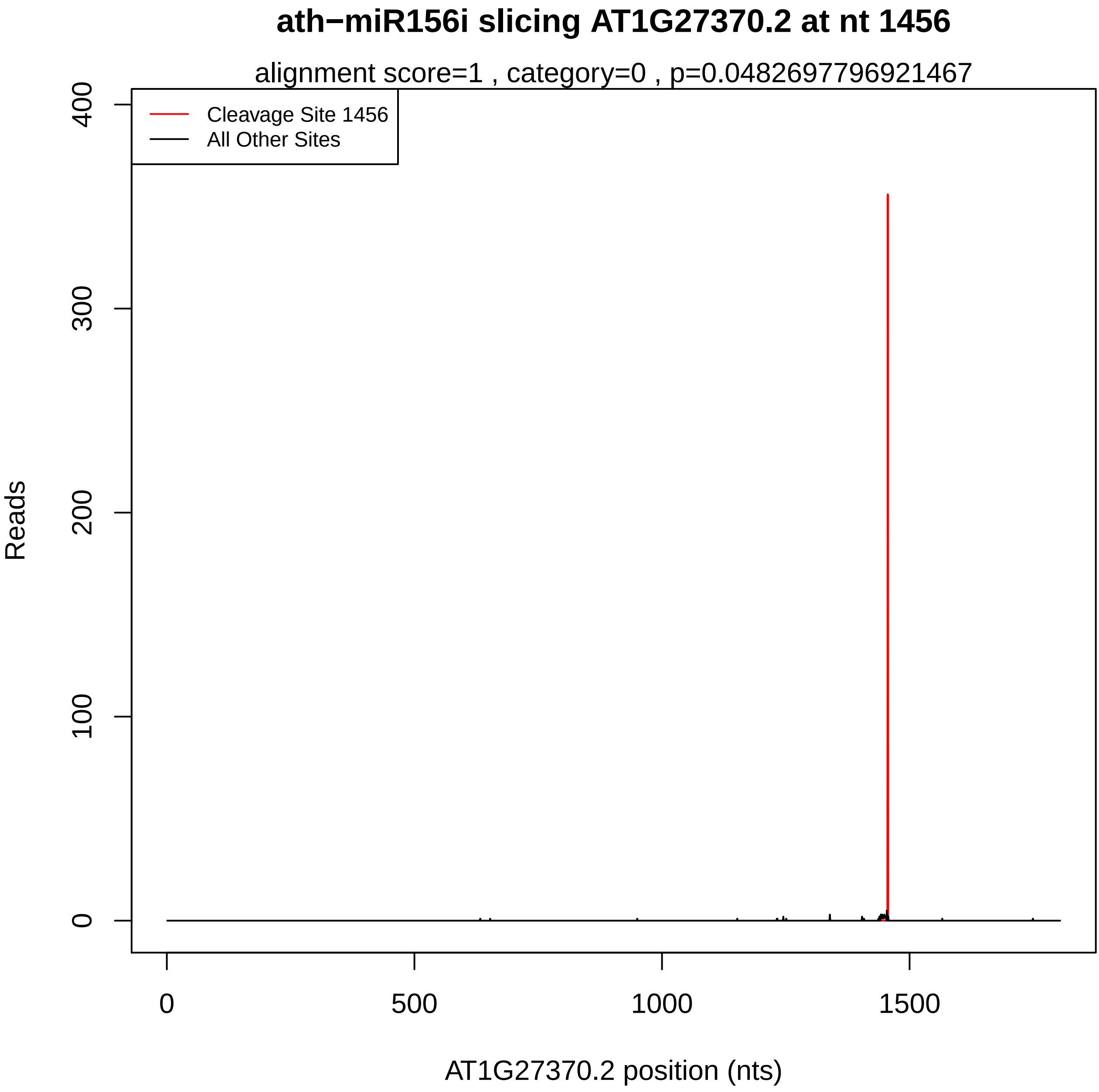
ath−miR156i slicing AT1G27370.2 at nt 1456
alignment score=1 , category=0 , p=0.0482697796921467
Cleavage Site 1456
All Other Sites
Reads
0
100
200
300
400
0
500
1000
1500
AT1G27370.2 position (nts)

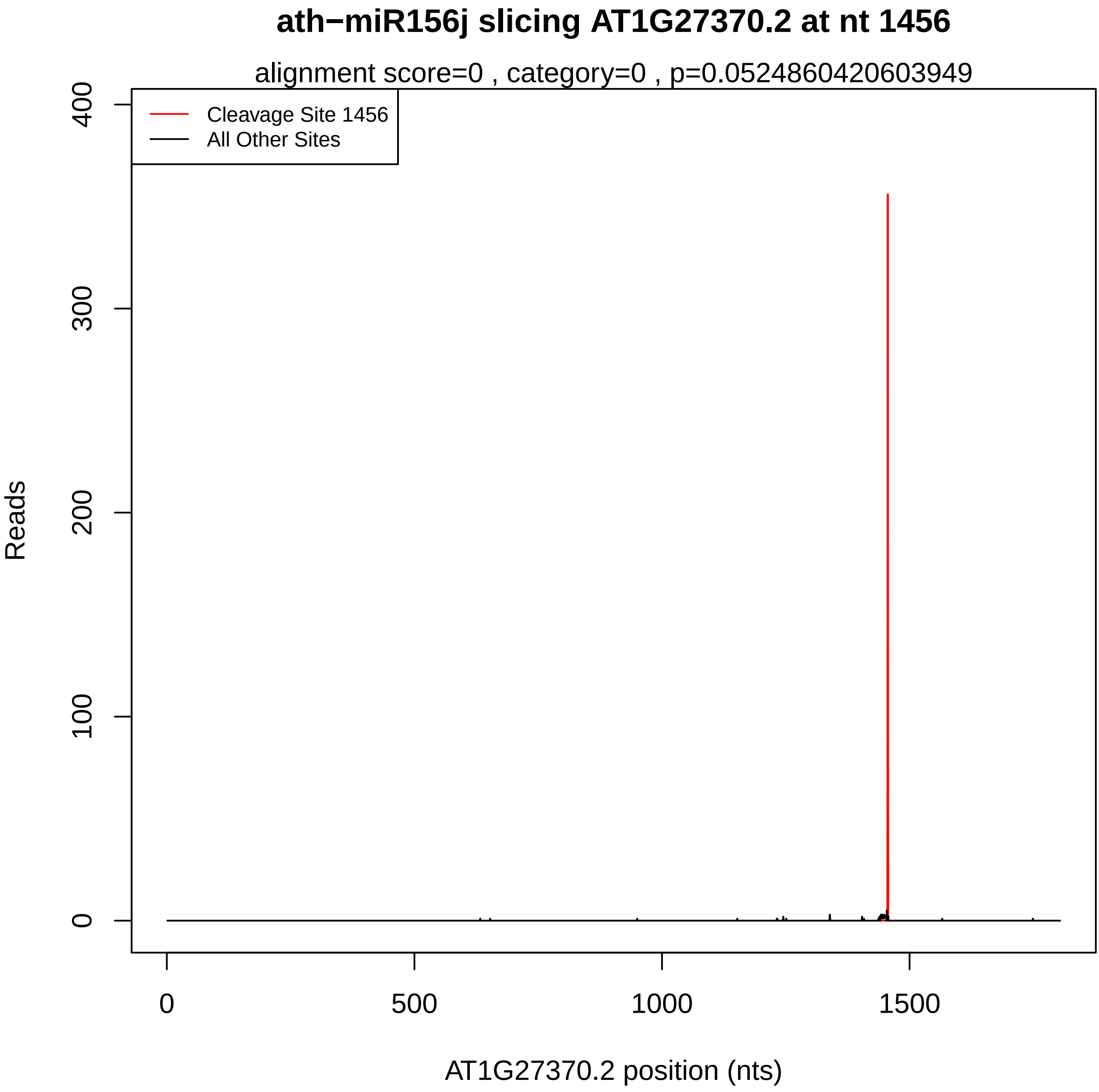
ath−miR156j slicing AT1G27370.2 at nt 1456
alignment score=0 , category=0 , p=0.0524860420603949
Cleavage Site 1456
All Other Sites
Reads
0
100
200
300
400
0
500
1000
1500
AT1G27370.2 position (nts)

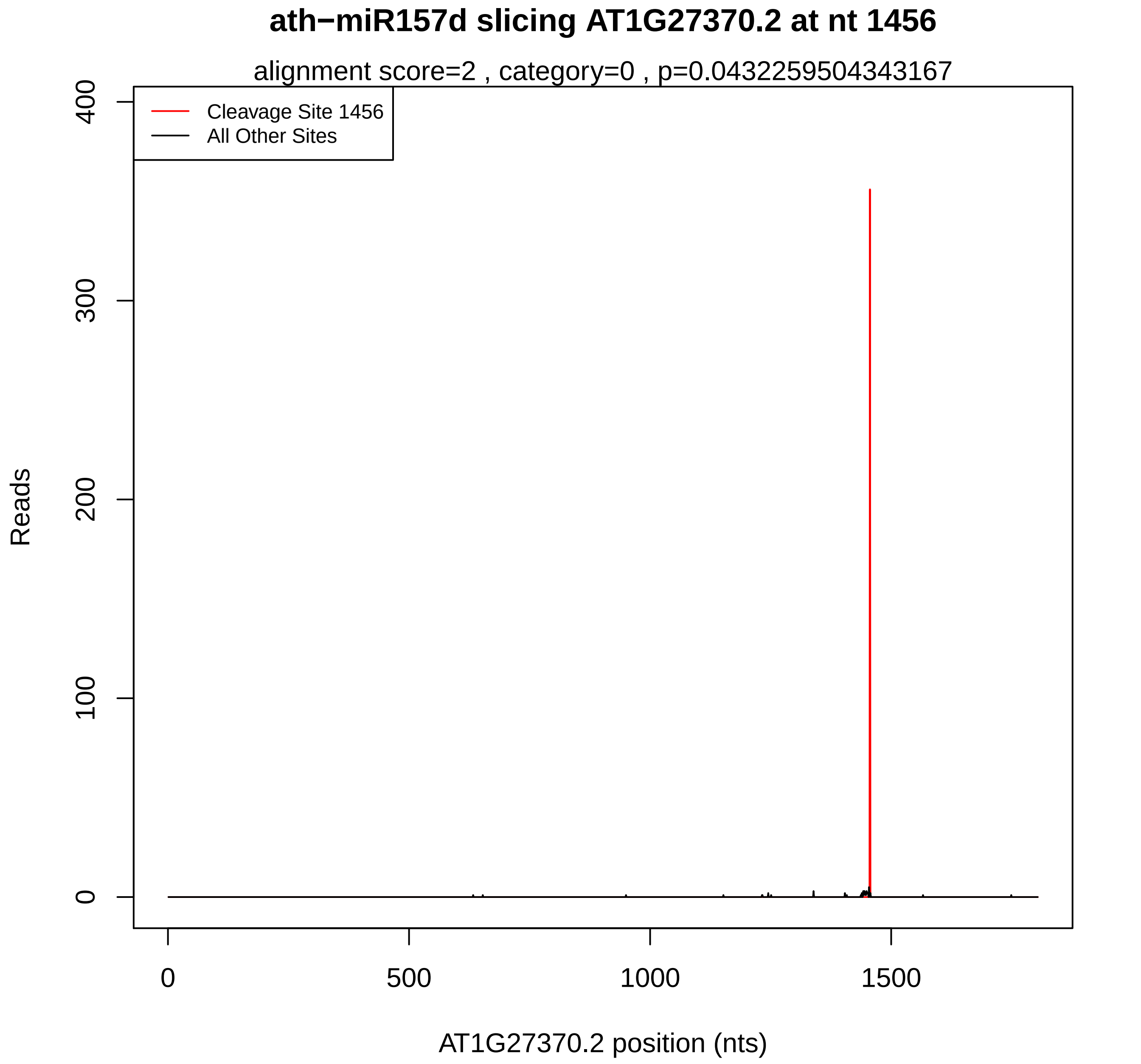
ath−miR157d slicing AT1G27370.2 at nt 1456
alignment score=2 , category=0 , p=0.0432259504343167
Cleavage Site 1456
All Other Sites
Reads
0
100
200
300
400
0
500
1000
1500
AT1G27370.2 position (nts)

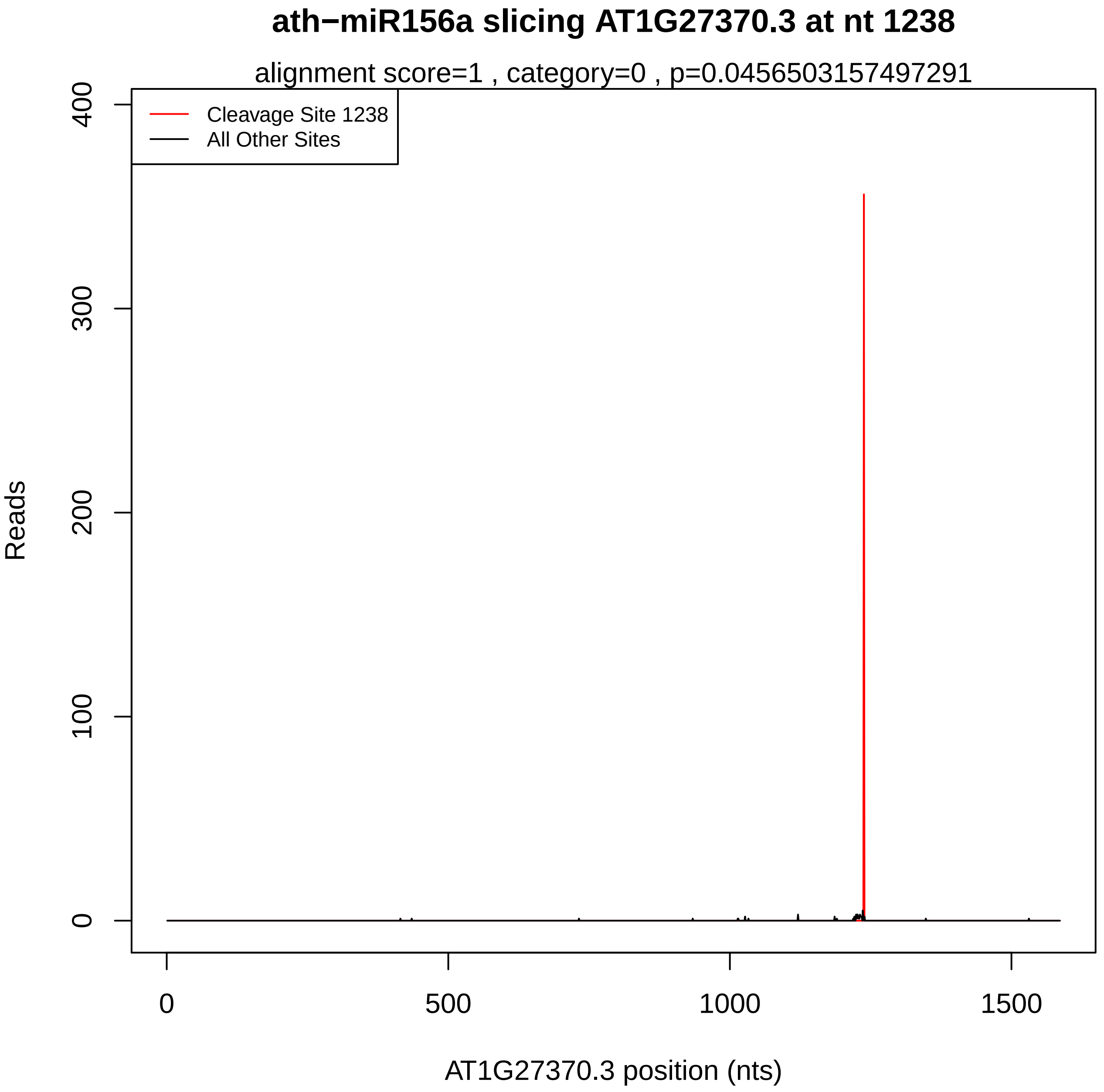

ath−miR156a slicing AT1G27370.3 at nt 1238
alignment score=1 , category=0 , p=0.0456503157497291
Cleavage Site 1238
All Other Sites
Reads
0
100
200
300
400
0
500
1000
1500
AT1G27370.3 position (nts)

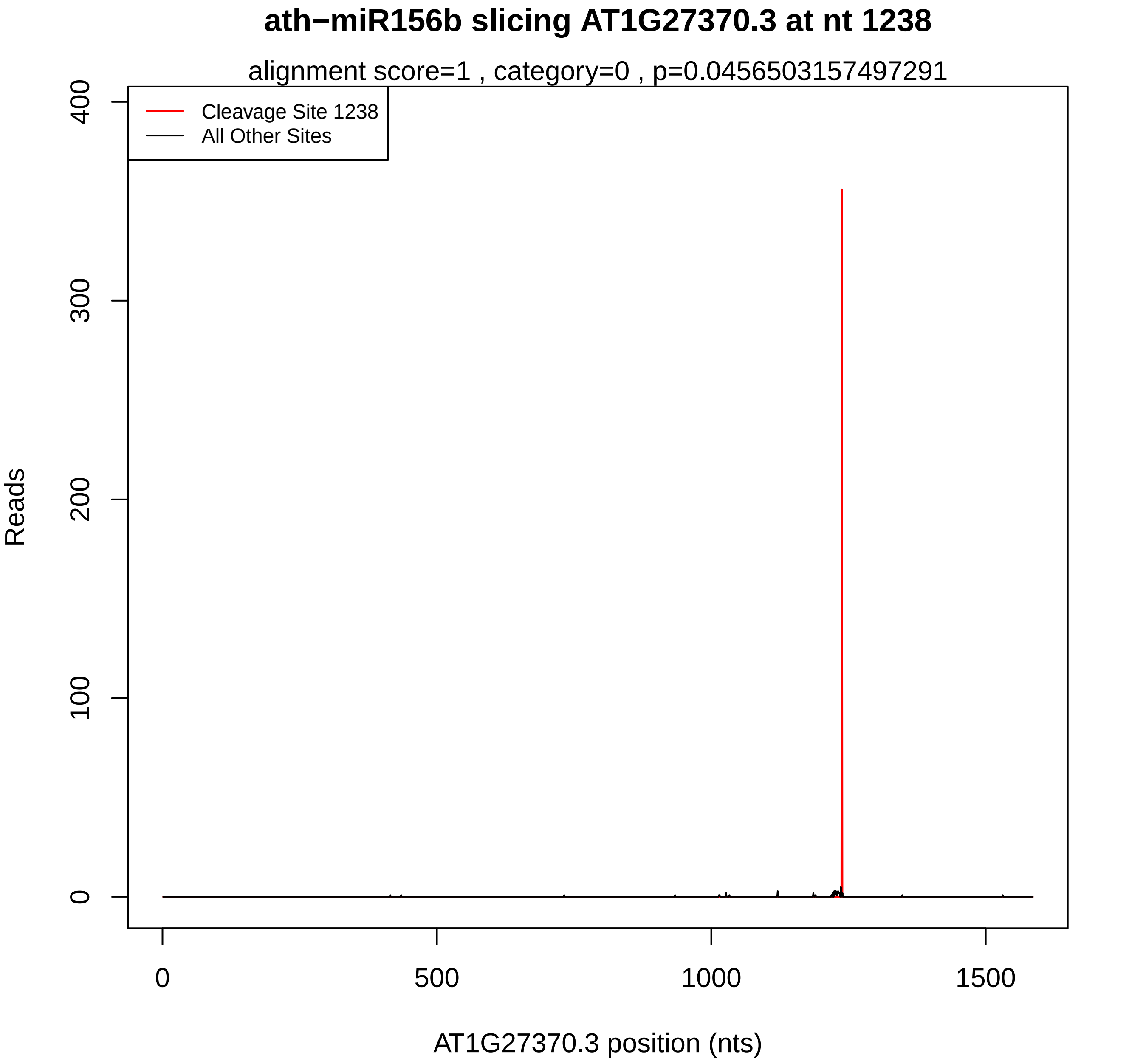
ath−miR156b slicing AT1G27370.3 at nt 1238
alignment score=1 , category=0 , p=0.0456503157497291
Cleavage Site 1238
All Other Sites
Reads
0
100
200
300
400
0
500
1000
1500
AT1G27370.3 position (nts)

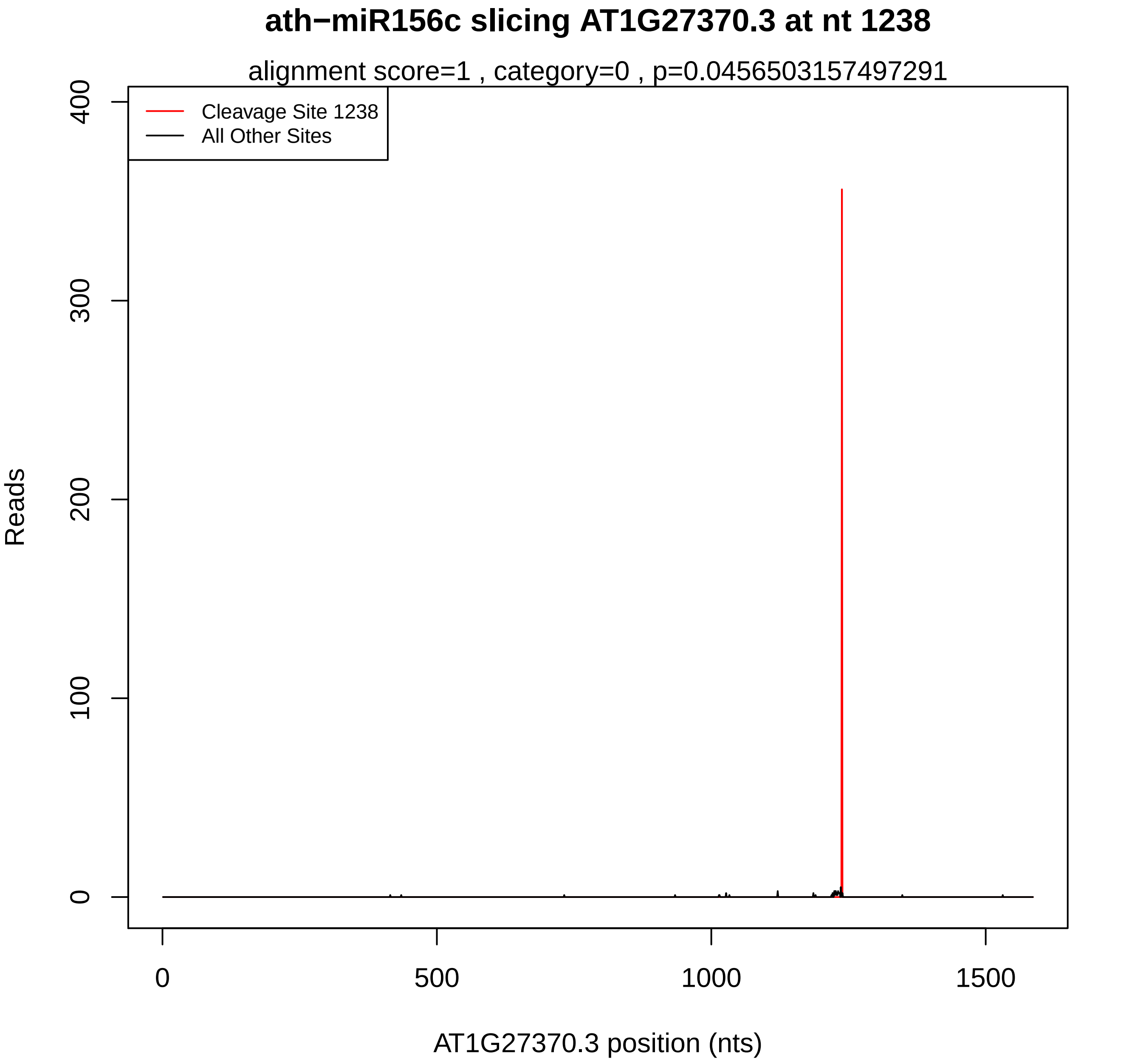

ath−miR156c slicing AT1G27370.3 at nt 1238
alignment score=1 , category=0 , p=0.0456503157497291
Cleavage Site 1238
All Other Sites
Reads
0
100
200
300
400
0
500
1000
1500
AT1G27370.3 position (nts)

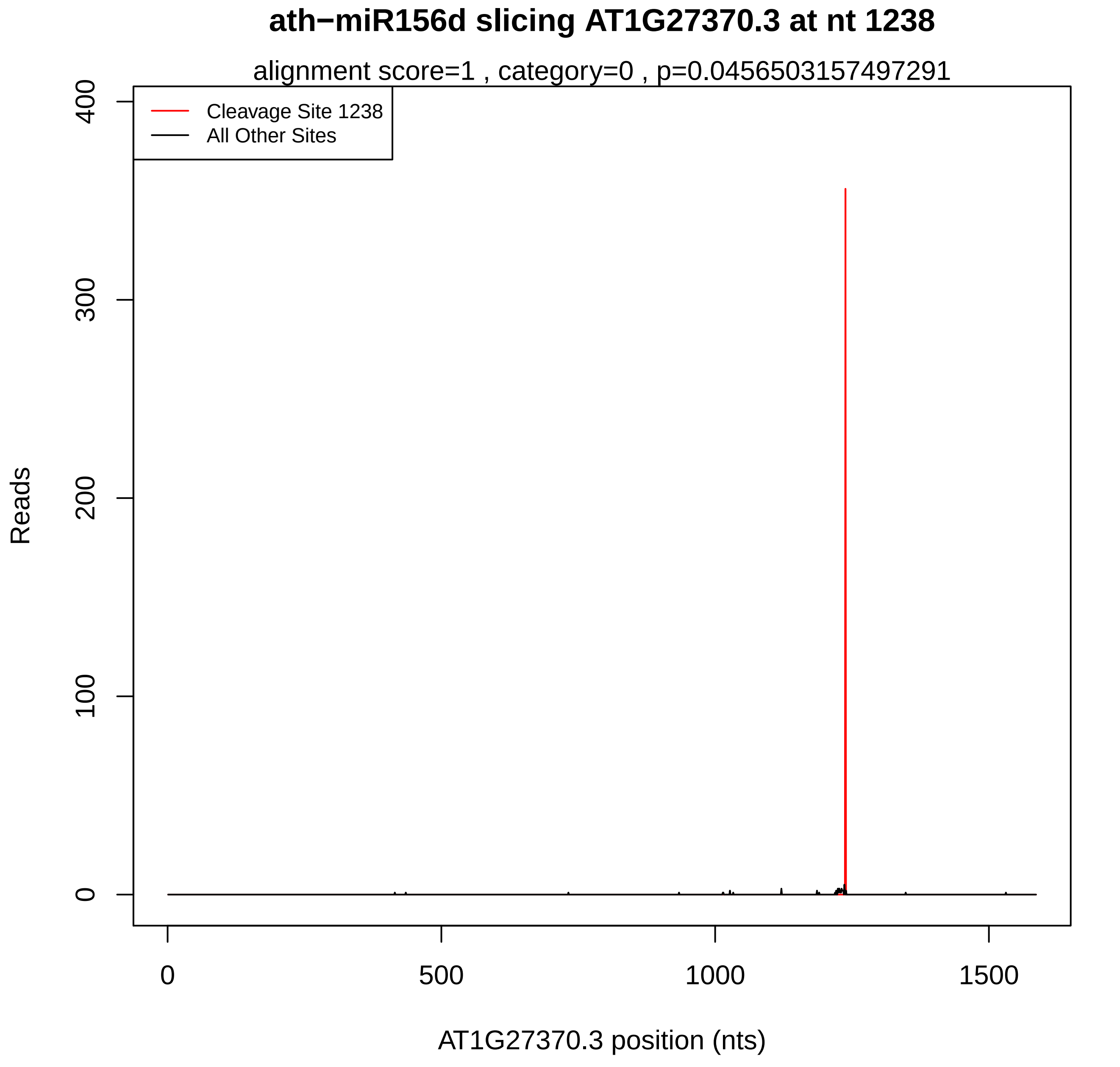

ath−miR156d slicing AT1G27370.3 at nt 1238
alignment score=1 , category=0 , p=0.0456503157497291
Cleavage Site 1238
All Other Sites
Reads
0
100
200
300
400
0
500
1000
1500
AT1G27370.3 position (nts)

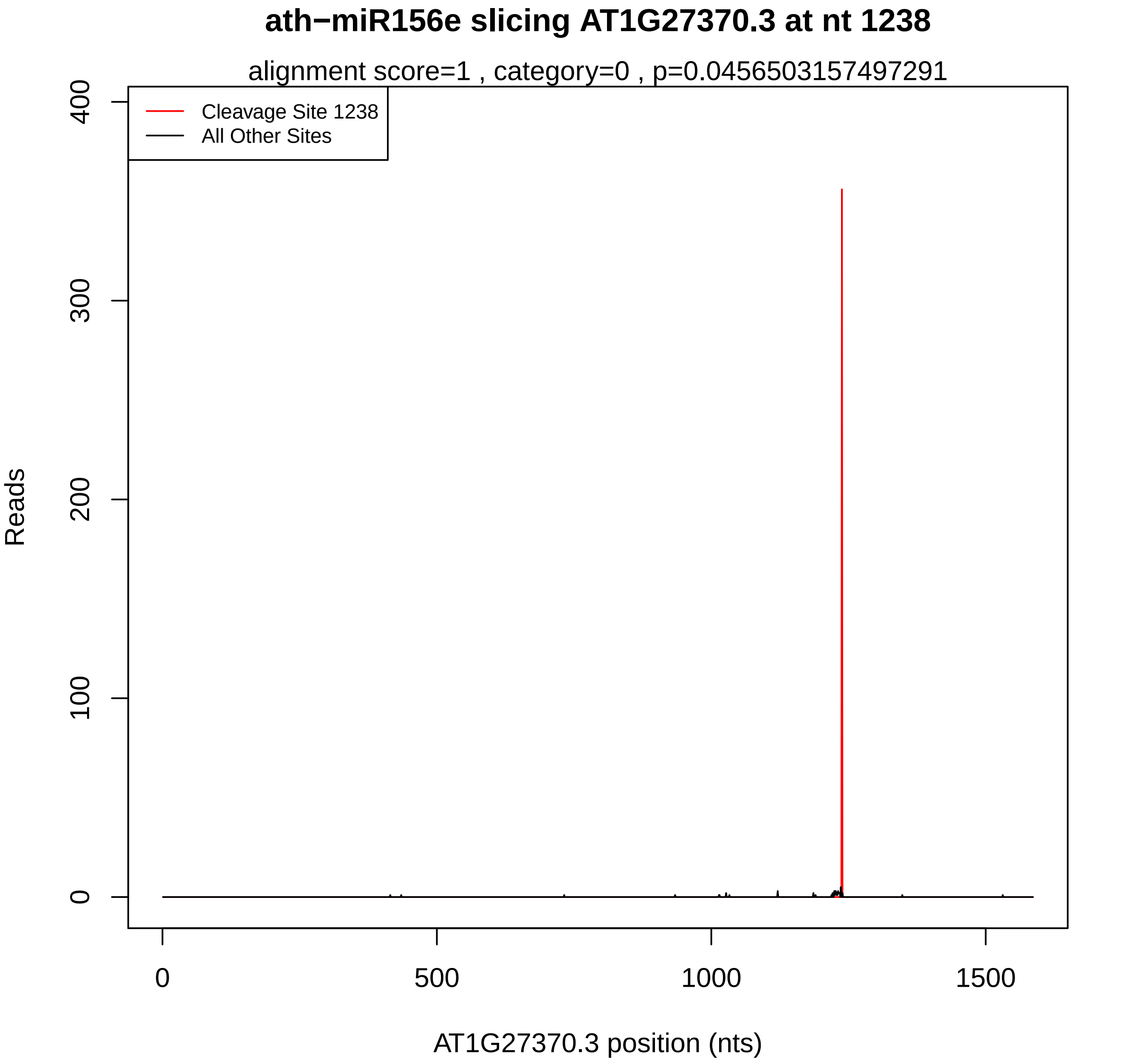
ath−miR156e slicing AT1G27370.3 at nt 1238
alignment score=1 , category=0 , p=0.0456503157497291
Cleavage Site 1238
All Other Sites
Reads
0
100
200
300
400
0
500
1000
1500
AT1G27370.3 position (nts)

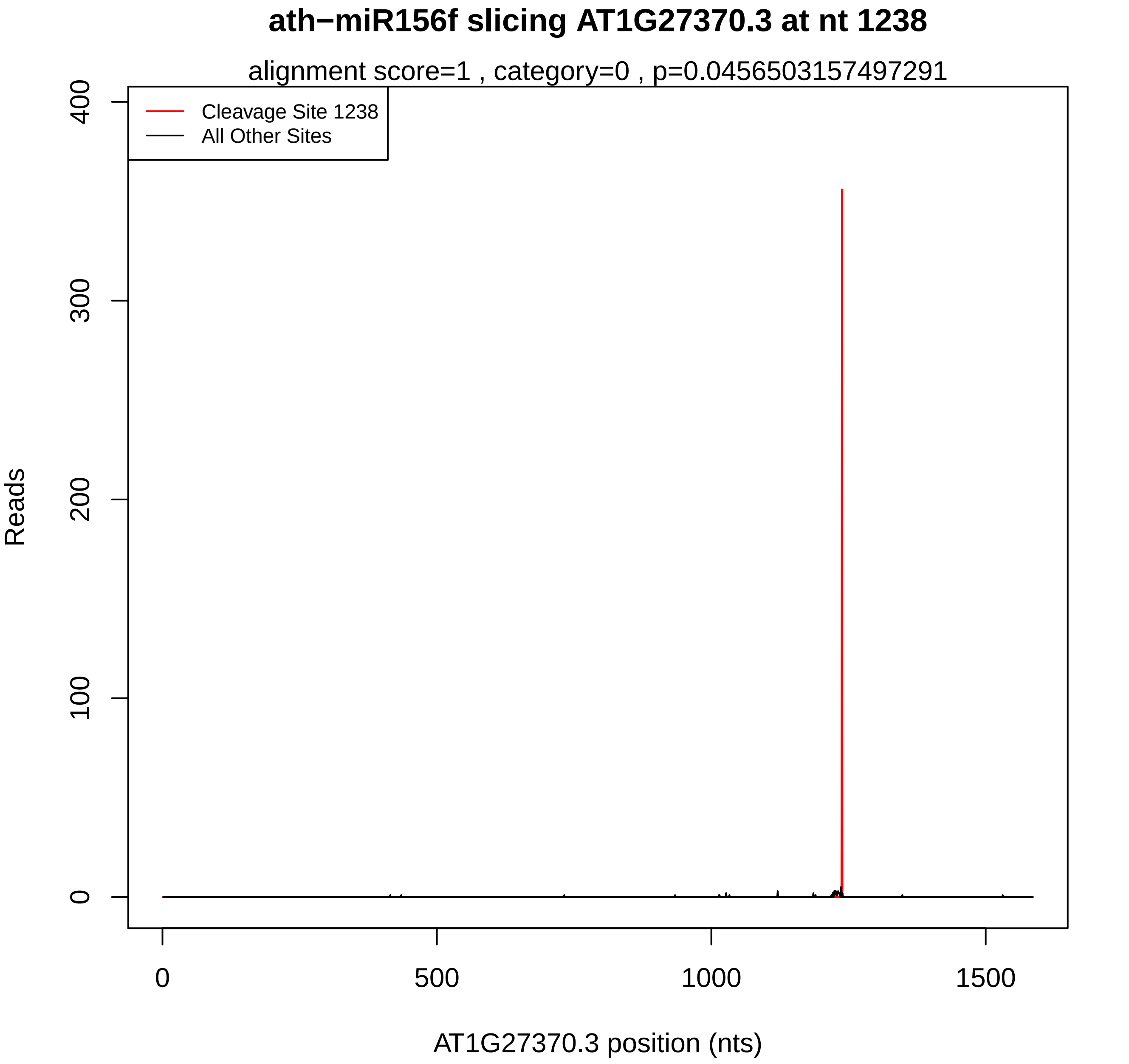

ath−miR156f slicing AT1G27370.3 at nt 1238
alignment score=1 , category=0 , p=0.0456503157497291
Cleavage Site 1238
All Other Sites
Reads
0
100
200
300
400
0
500
1000
1500
AT1G27370.3 position (nts)

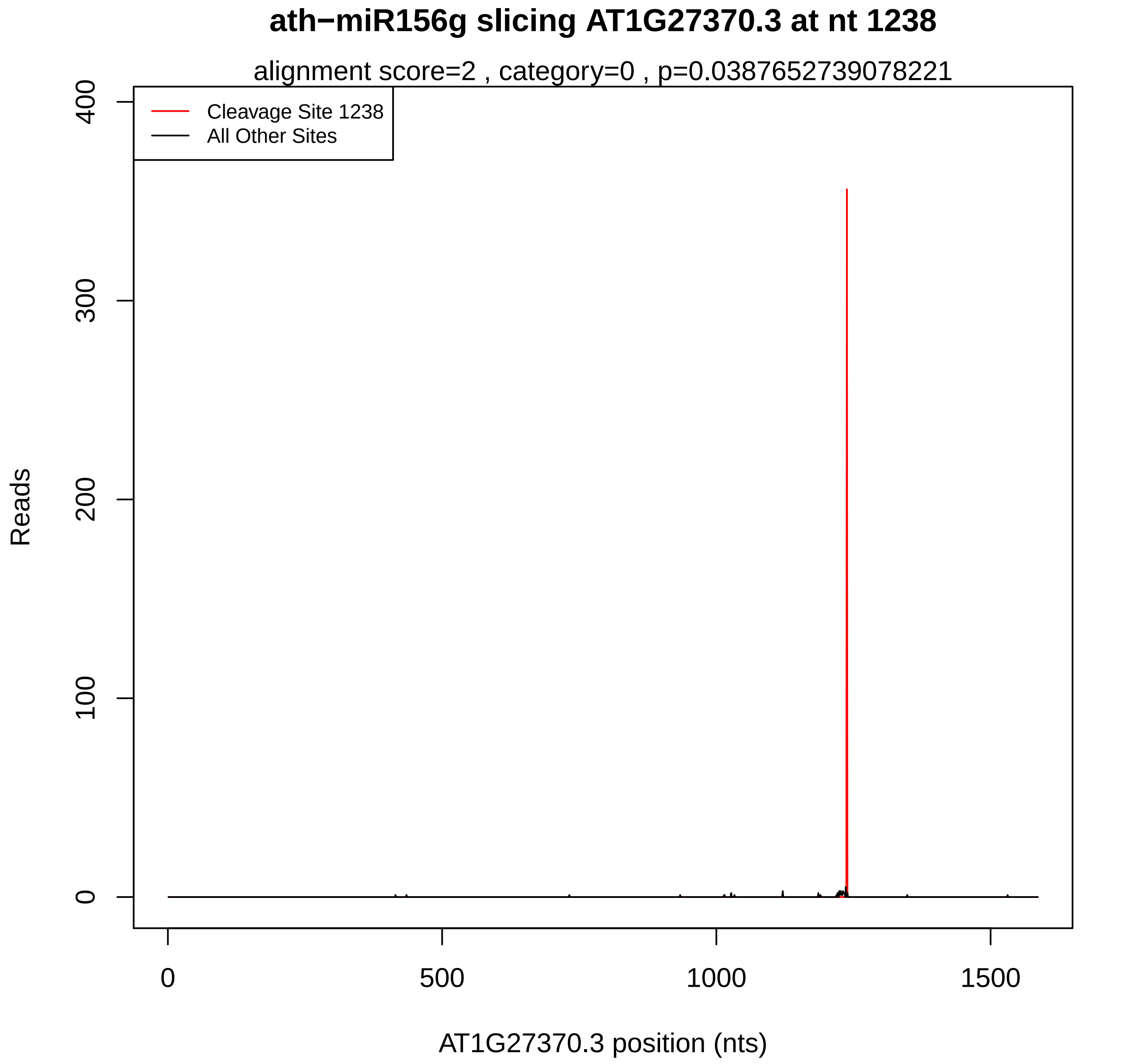

ath−miR156g slicing AT1G27370.3 at nt 1238
alignment score=2 , category=0 , p=0.0387652739078221
Cleavage Site 1238
All Other Sites
Reads
0
100
200
300
400
0
500
1000
1500
AT1G27370.3 position (nts)

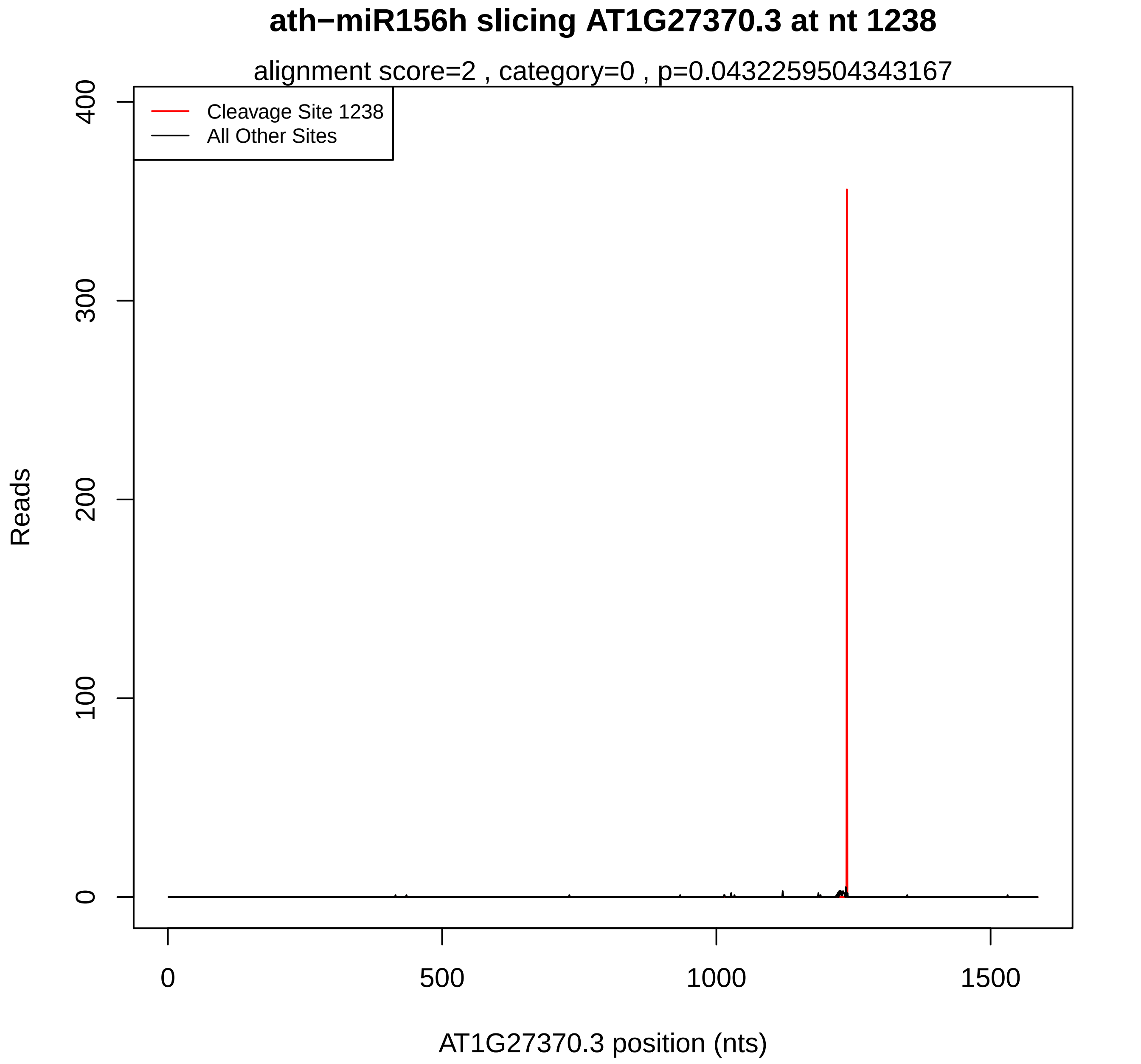
ath−miR156h slicing AT1G27370.3 at nt 1238
alignment score=2 , category=0 , p=0.0432259504343167
Cleavage Site 1238
All Other Sites
Reads
0
100
200
300
400
0
500
1000
1500
AT1G27370.3 position (nts)

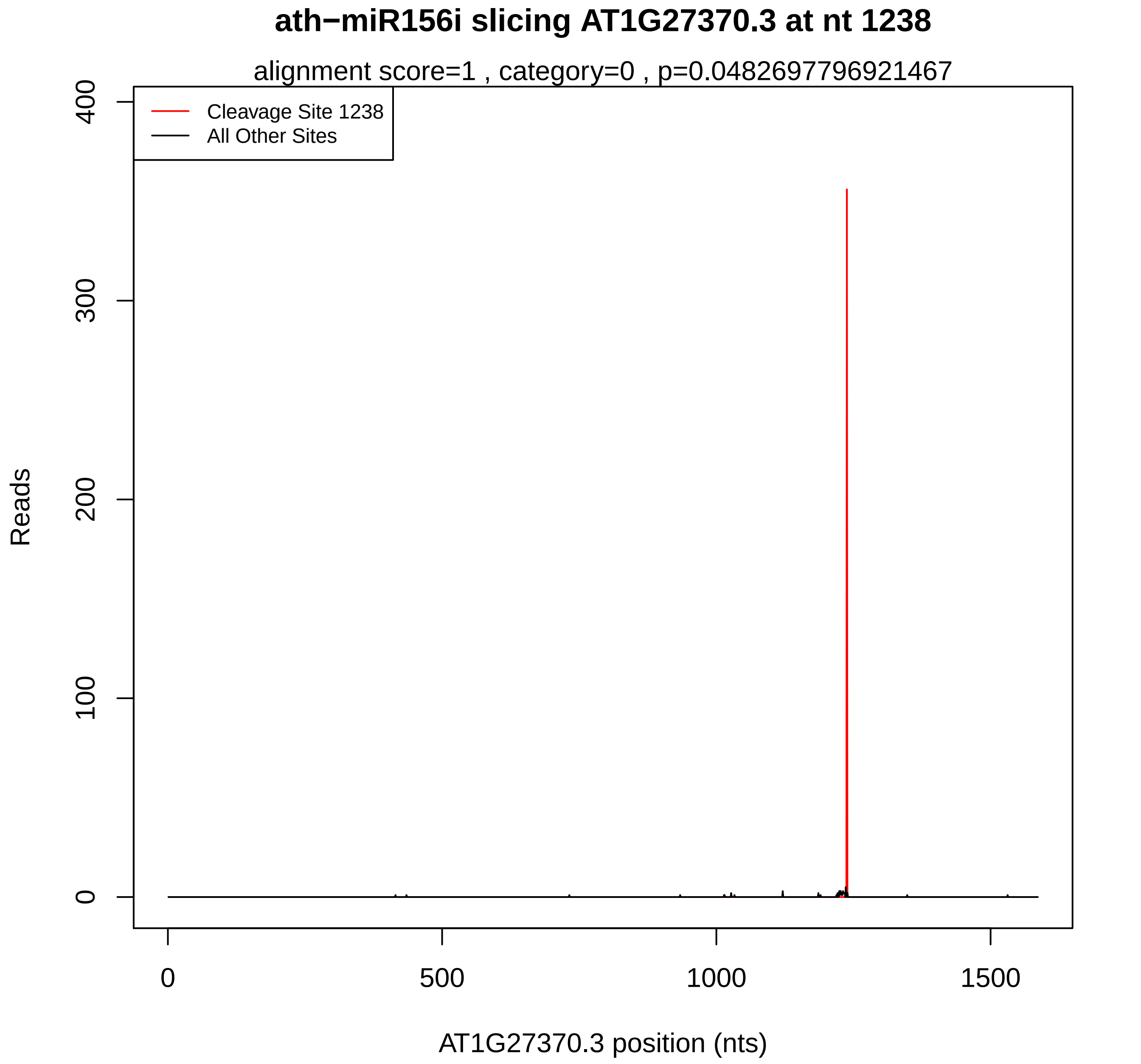
ath−miR156i slicing AT1G27370.3 at nt 1238
alignment score=1 , category=0 , p=0.0482697796921467
Cleavage Site 1238
All Other Sites
Reads
0
100
200
300
400
0
500
1000
1500
AT1G27370.3 position (nts)

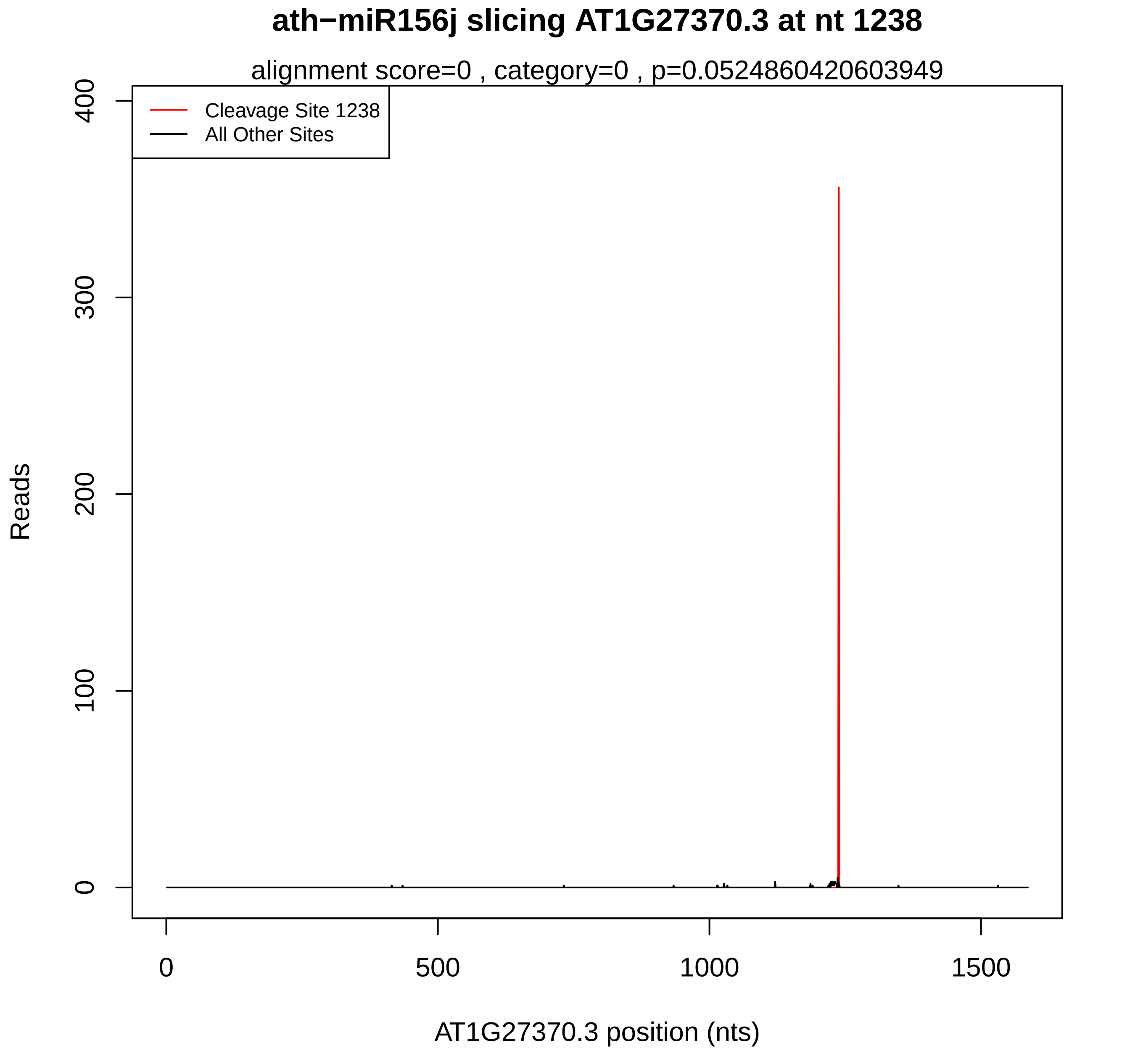

ath−miR156j slicing AT1G27370.3 at nt 1238
alignment score=0 , category=0 , p=0.0524860420603949
Cleavage Site 1238
All Other Sites
Reads
0
100
200
300
400
0
500
1000
1500
AT1G27370.3 position (nts)

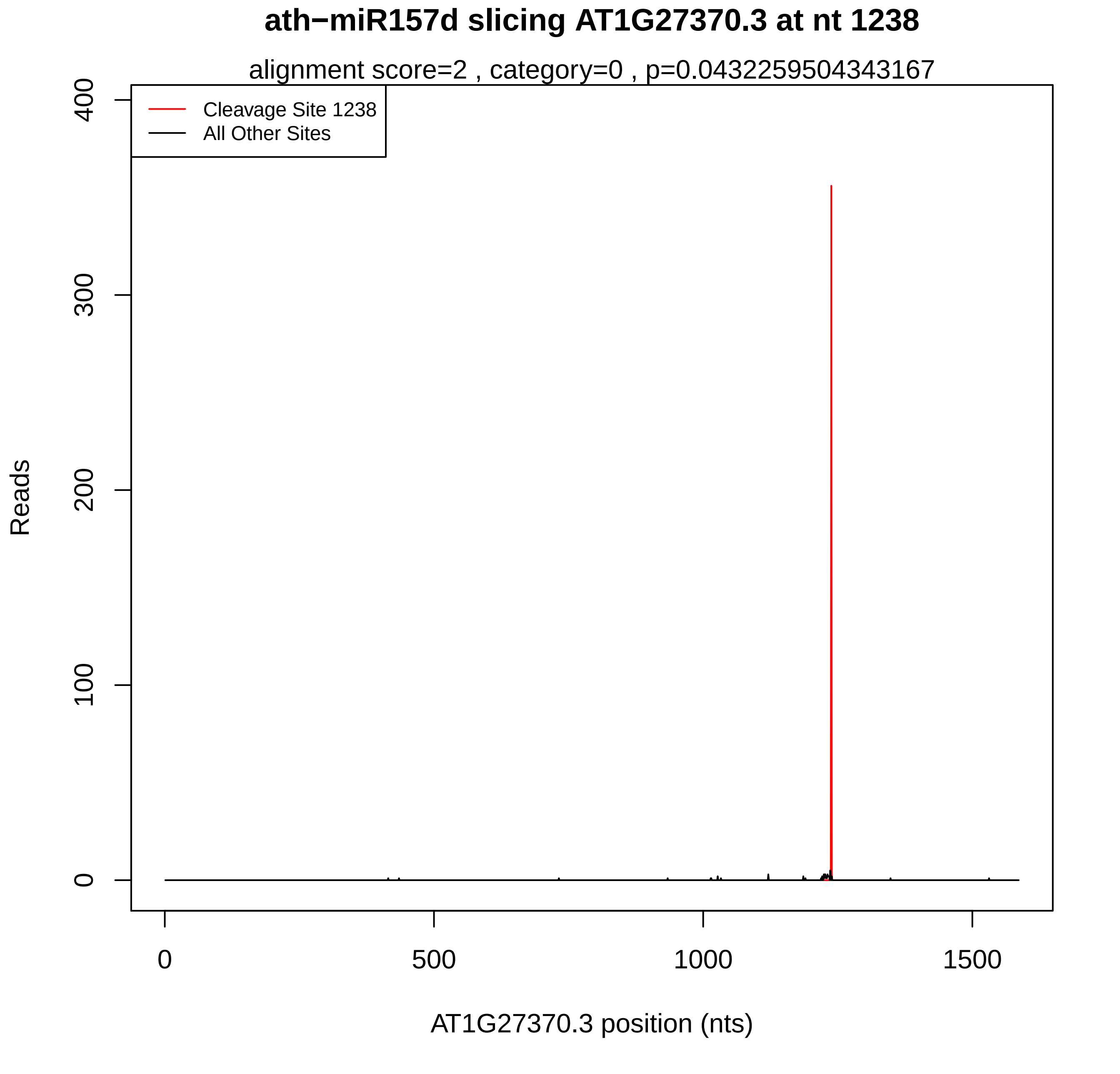
ath−miR157d slicing AT1G27370.3 at nt 1238
alignment score=2 , category=0 , p=0.0432259504343167
Cleavage Site 1238
All Other Sites
Reads
0
100
200
300
400
0
500
1000
1500
AT1G27370.3 position (nts)

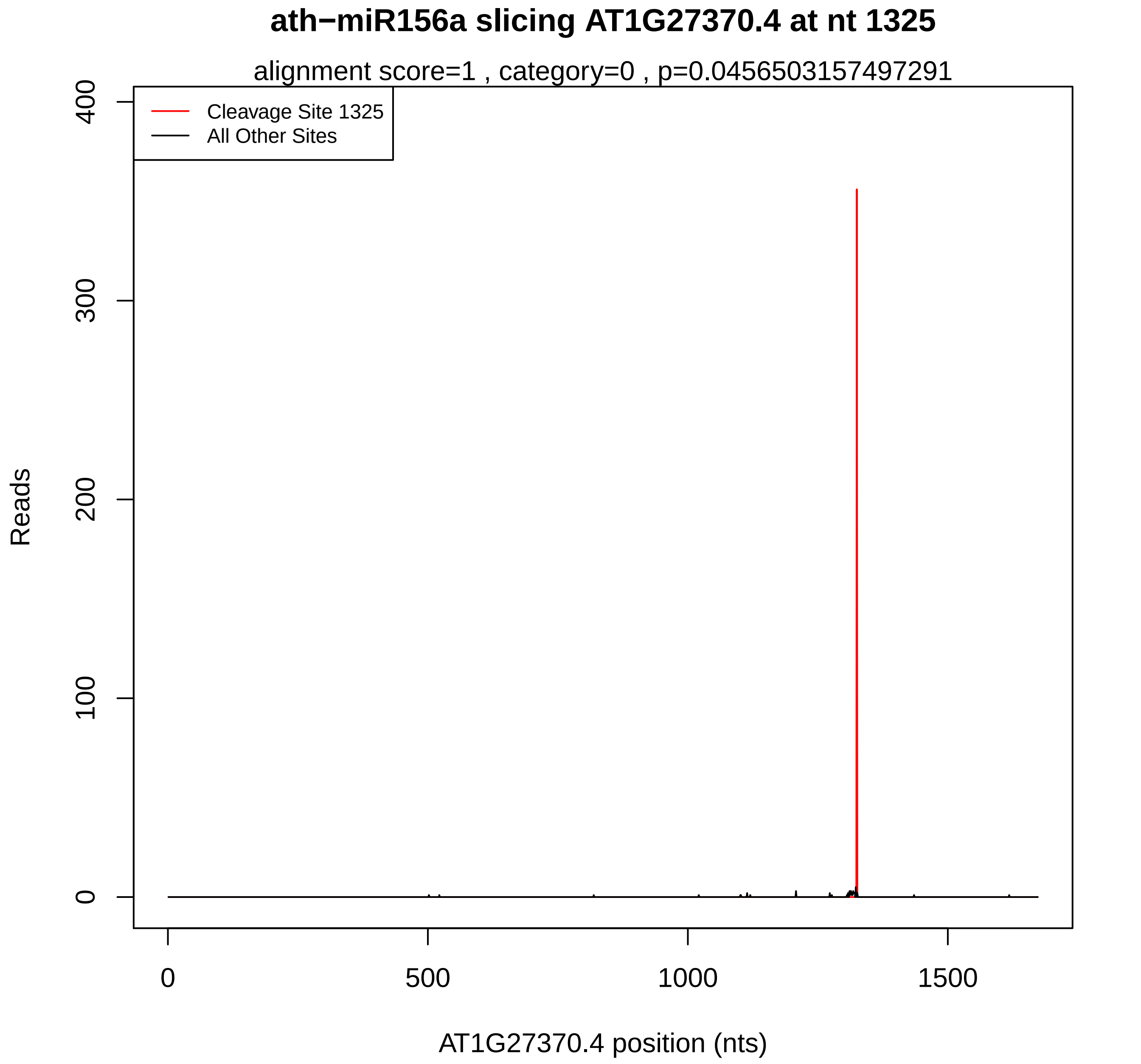
ath−miR156a slicing AT1G27370.4 at nt 1325
alignment score=1 , category=0 , p=0.0456503157497291
Cleavage Site 1325
All Other Sites
Reads
0
100
200
300
400
0
500
1000
1500
AT1G27370.4 position (nts)

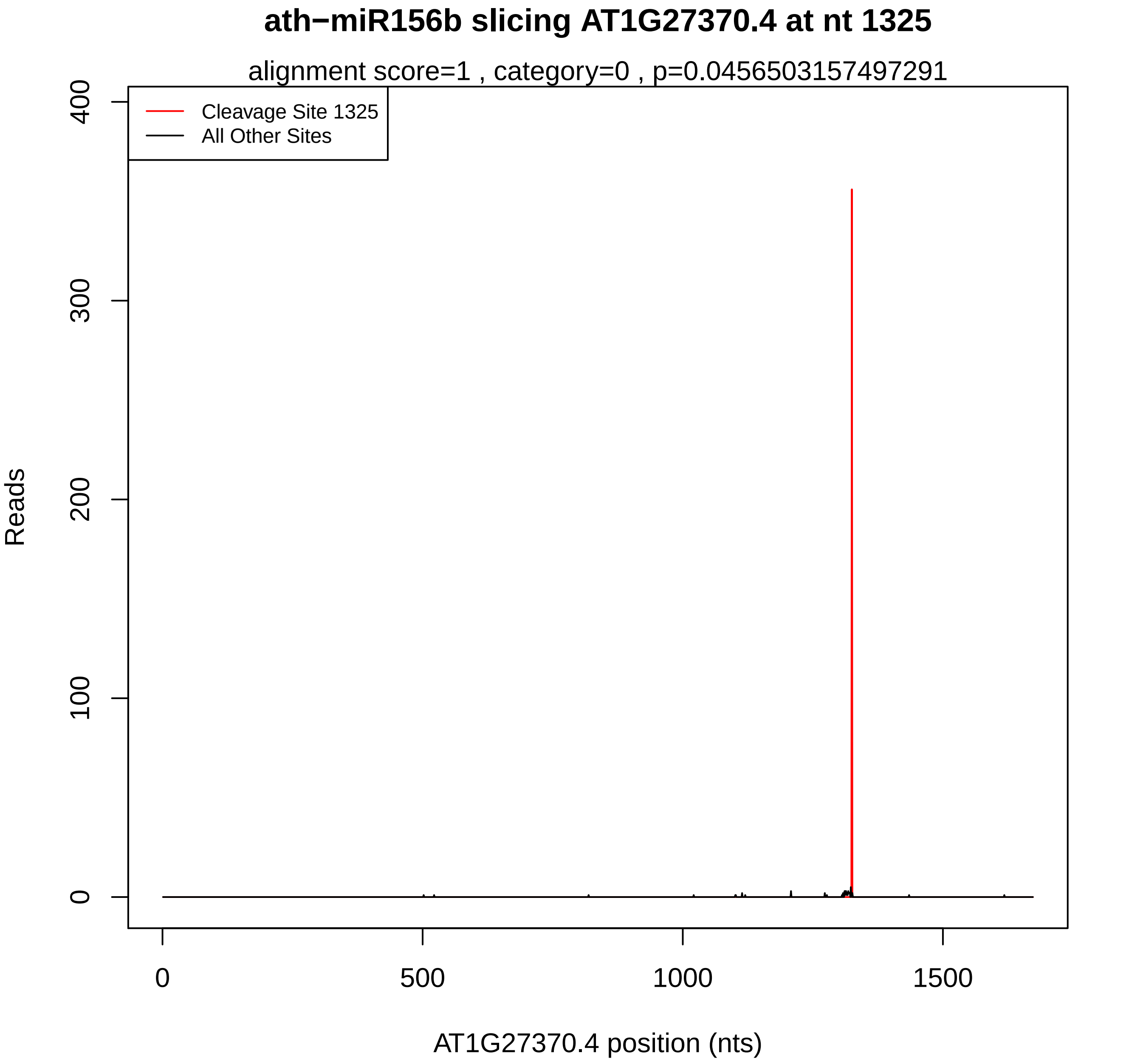
ath−miR156b slicing AT1G27370.4 at nt 1325
alignment score=1 , category=0 , p=0.0456503157497291
Cleavage Site 1325
All Other Sites
Reads
0
100
200
300
400
0
500
1000
1500
AT1G27370.4 position (nts)

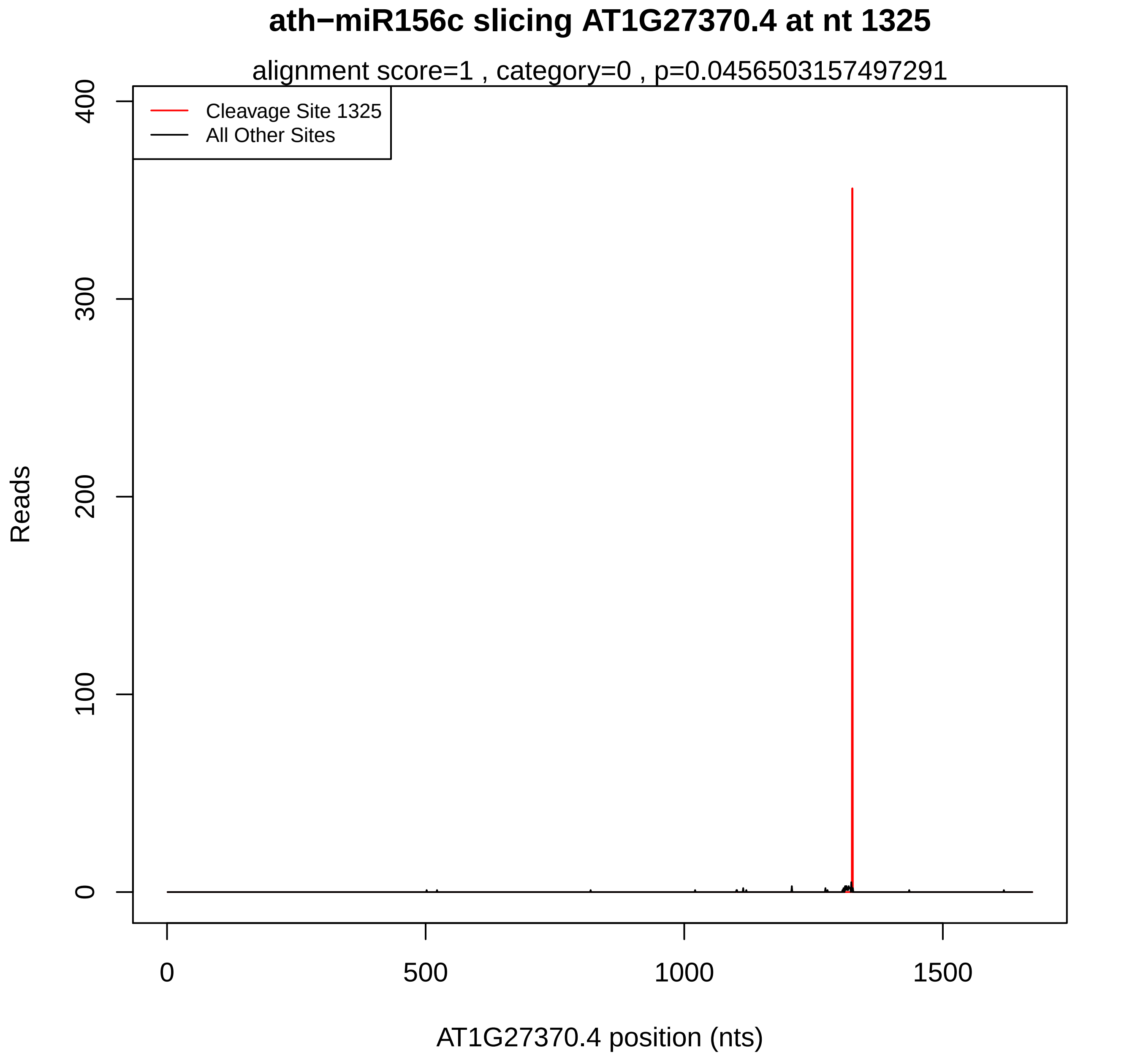
ath−miR156c slicing AT1G27370.4 at nt 1325
alignment score=1 , category=0 , p=0.0456503157497291
Cleavage Site 1325
All Other Sites
Reads
0
100
200
300
400
0
500
1000
1500
AT1G27370.4 position (nts)

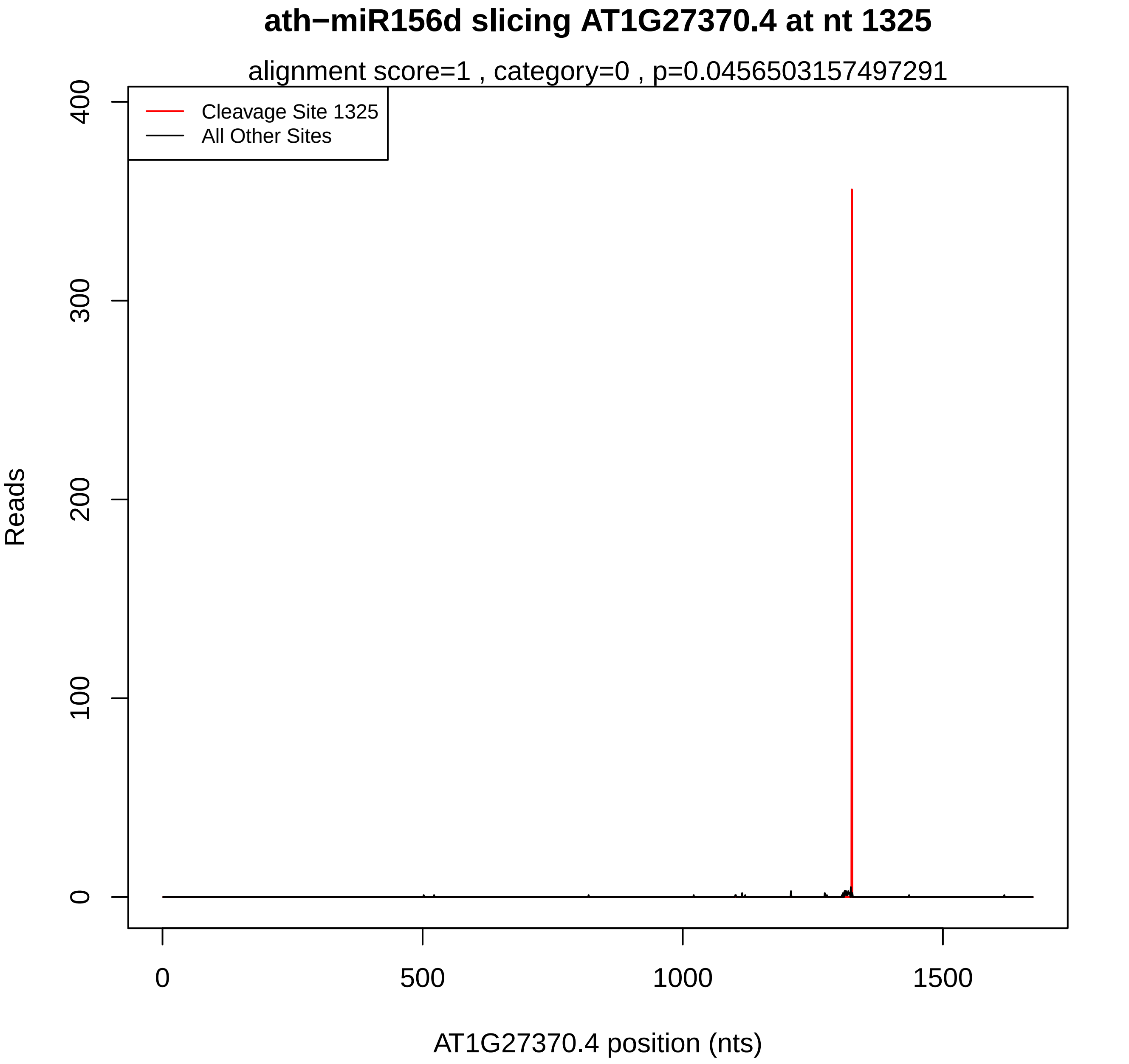
ath−miR156d slicing AT1G27370.4 at nt 1325
alignment score=1 , category=0 , p=0.0456503157497291
Cleavage Site 1325
All Other Sites
Reads
0
100
200
300
400
0
500
1000
1500
AT1G27370.4 position (nts)

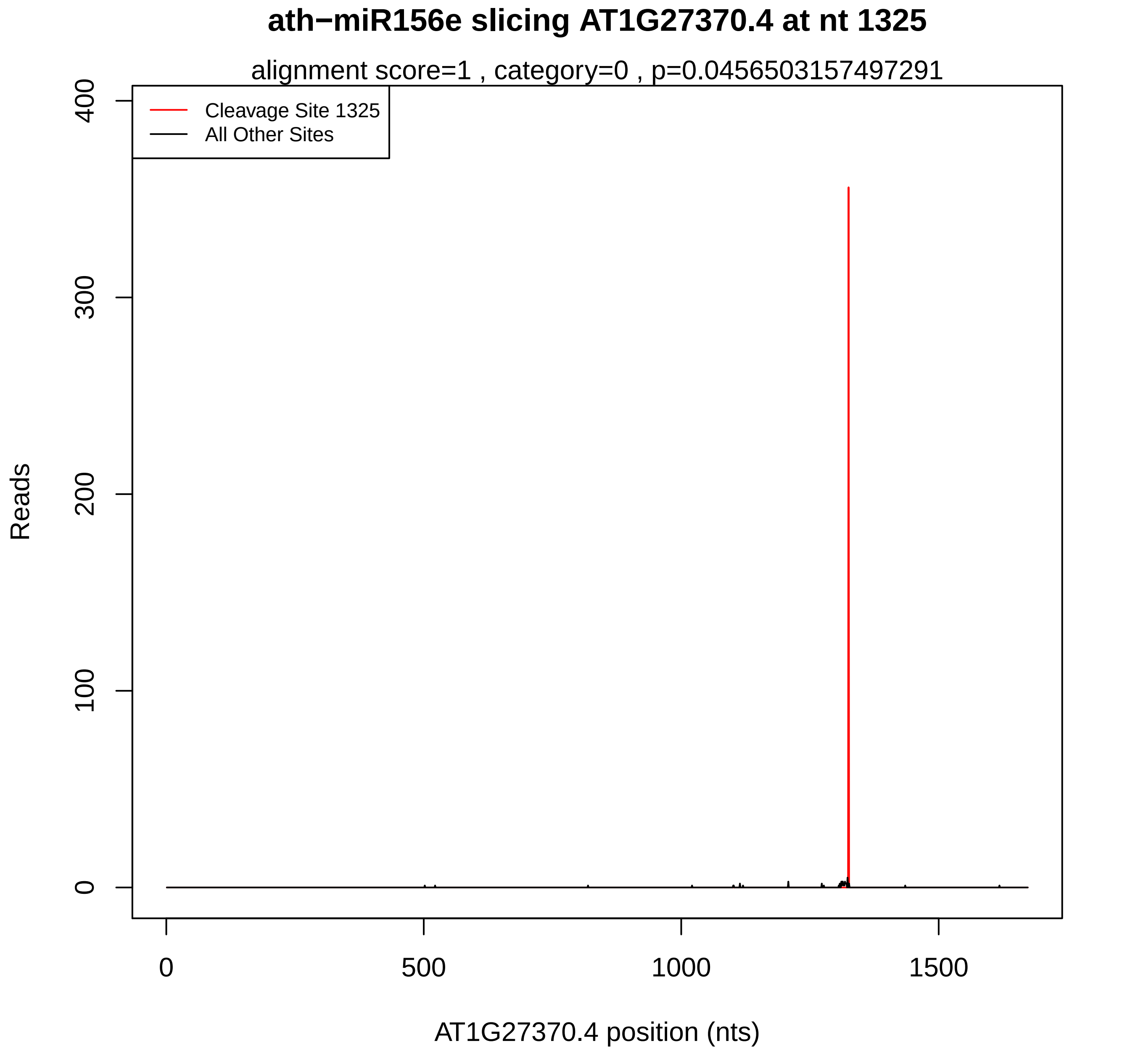
ath−miR156e slicing AT1G27370.4 at nt 1325
alignment score=1 , category=0 , p=0.0456503157497291
Cleavage Site 1325
All Other Sites
Reads
0
100
200
300
400
0
500
1000
1500
AT1G27370.4 position (nts)

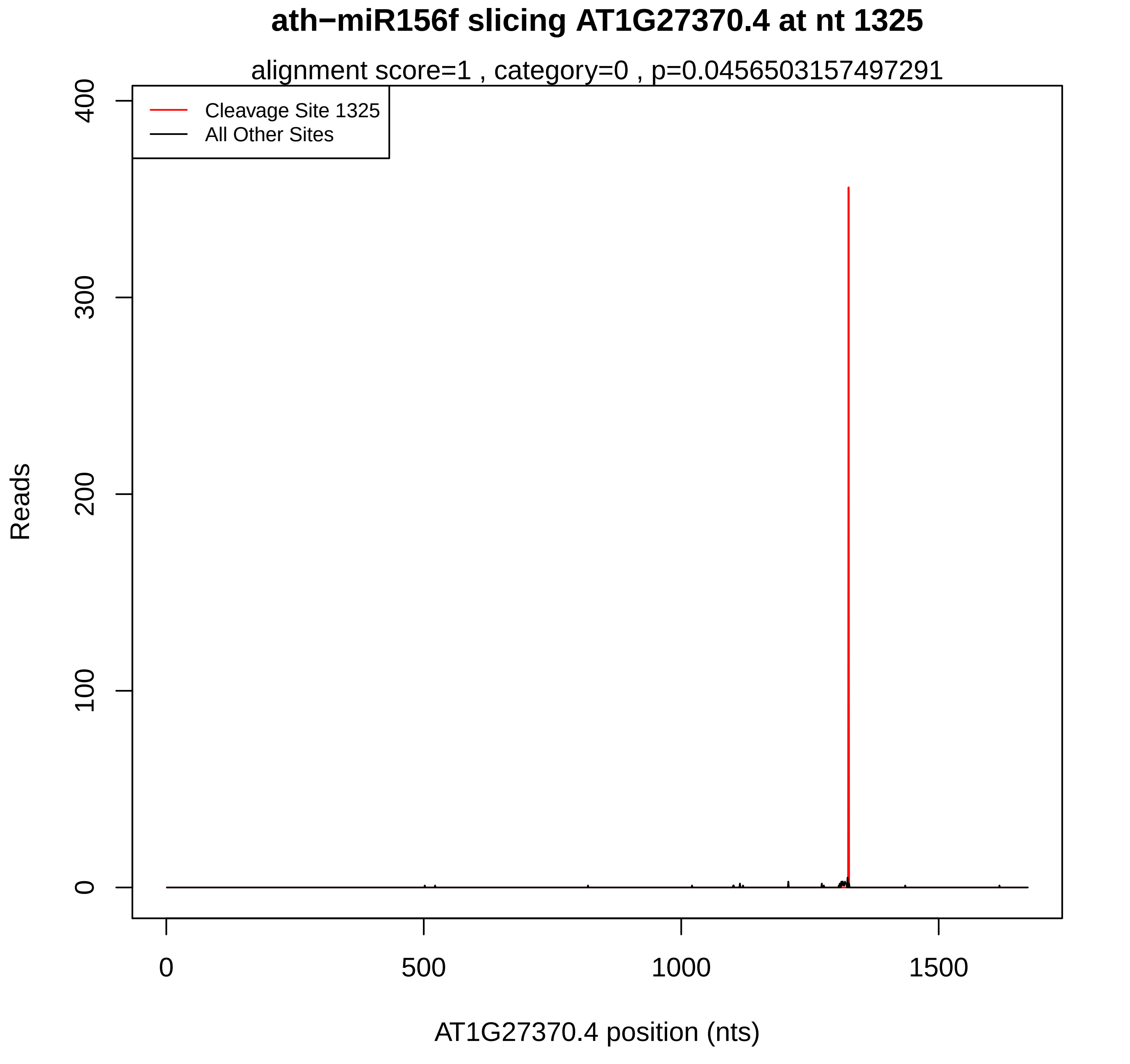

ath−miR156f slicing AT1G27370.4 at nt 1325
alignment score=1 , category=0 , p=0.0456503157497291
Cleavage Site 1325
All Other Sites
Reads
0
100
200
300
400
0
500
1000
1500
AT1G27370.4 position (nts)

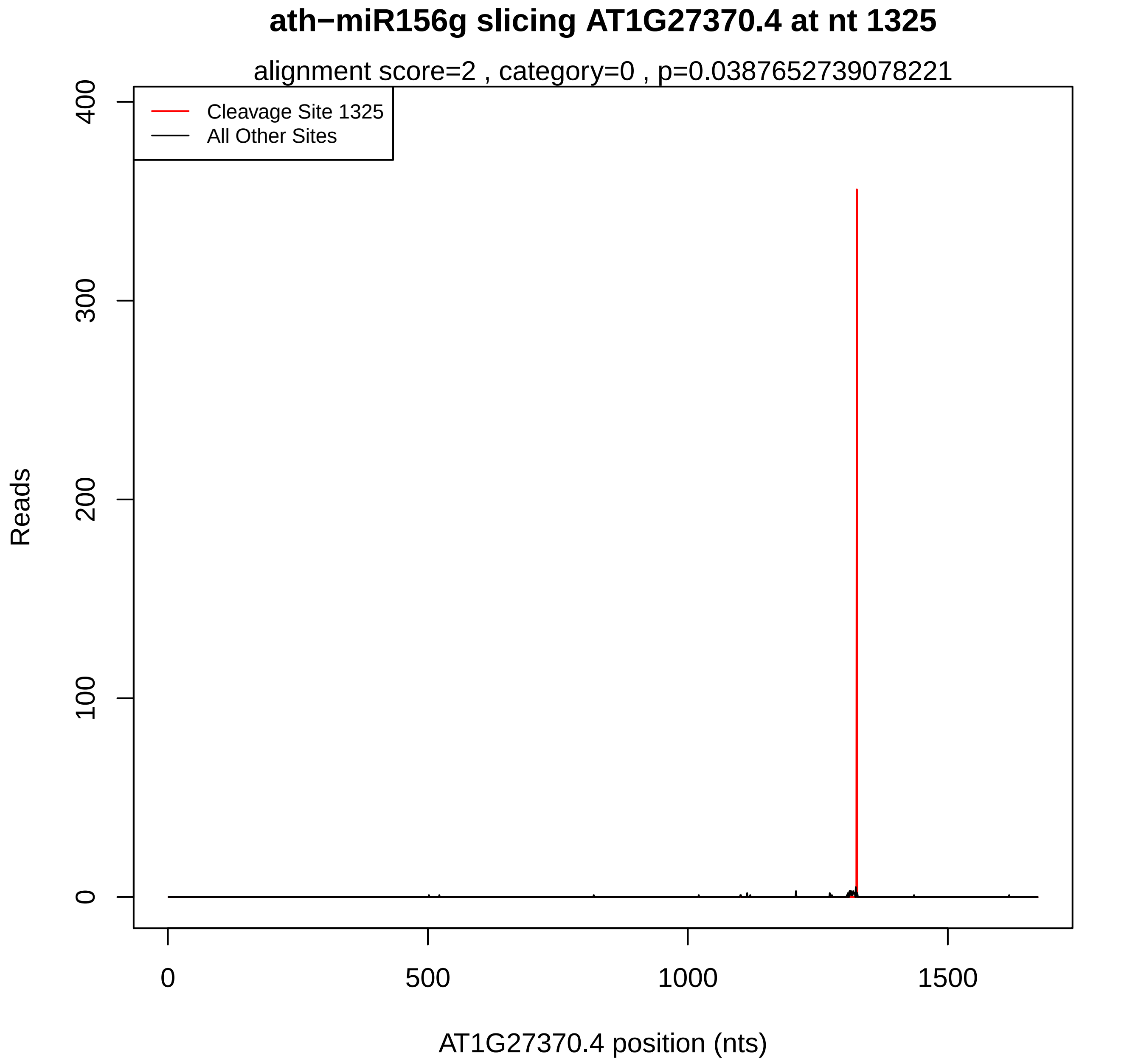

ath−miR156g slicing AT1G27370.4 at nt 1325
alignment score=2 , category=0 , p=0.0387652739078221
Cleavage Site 1325
All Other Sites
Reads
0
100
200
300
400
0
500
1000
1500
AT1G27370.4 position (nts)

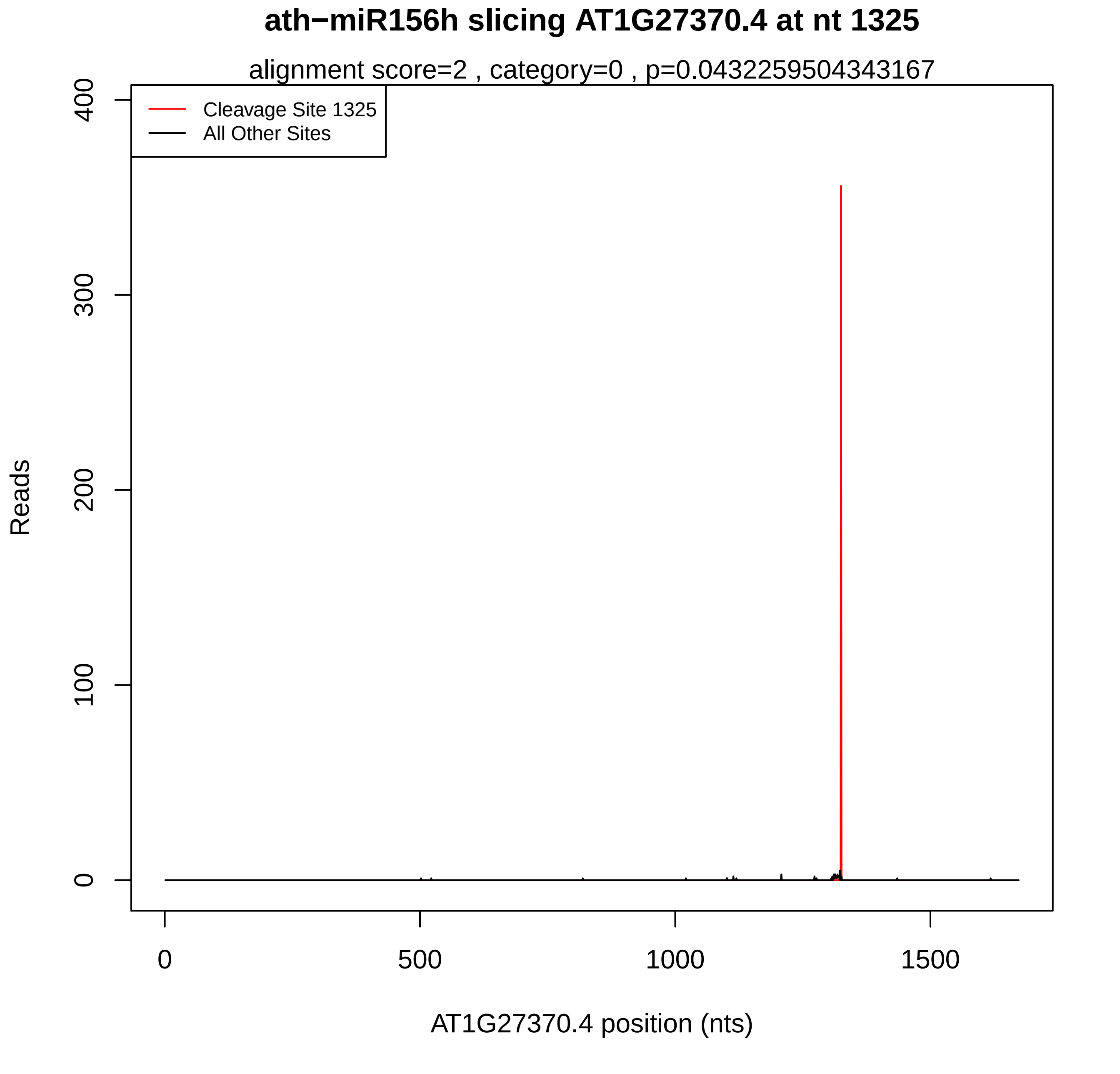

ath−miR156h slicing AT1G27370.4 at nt 1325
alignment score=2 , category=0 , p=0.0432259504343167
Cleavage Site 1325
All Other Sites
Reads
0
100
200
300
400
0
500
1000
1500
AT1G27370.4 position (nts)

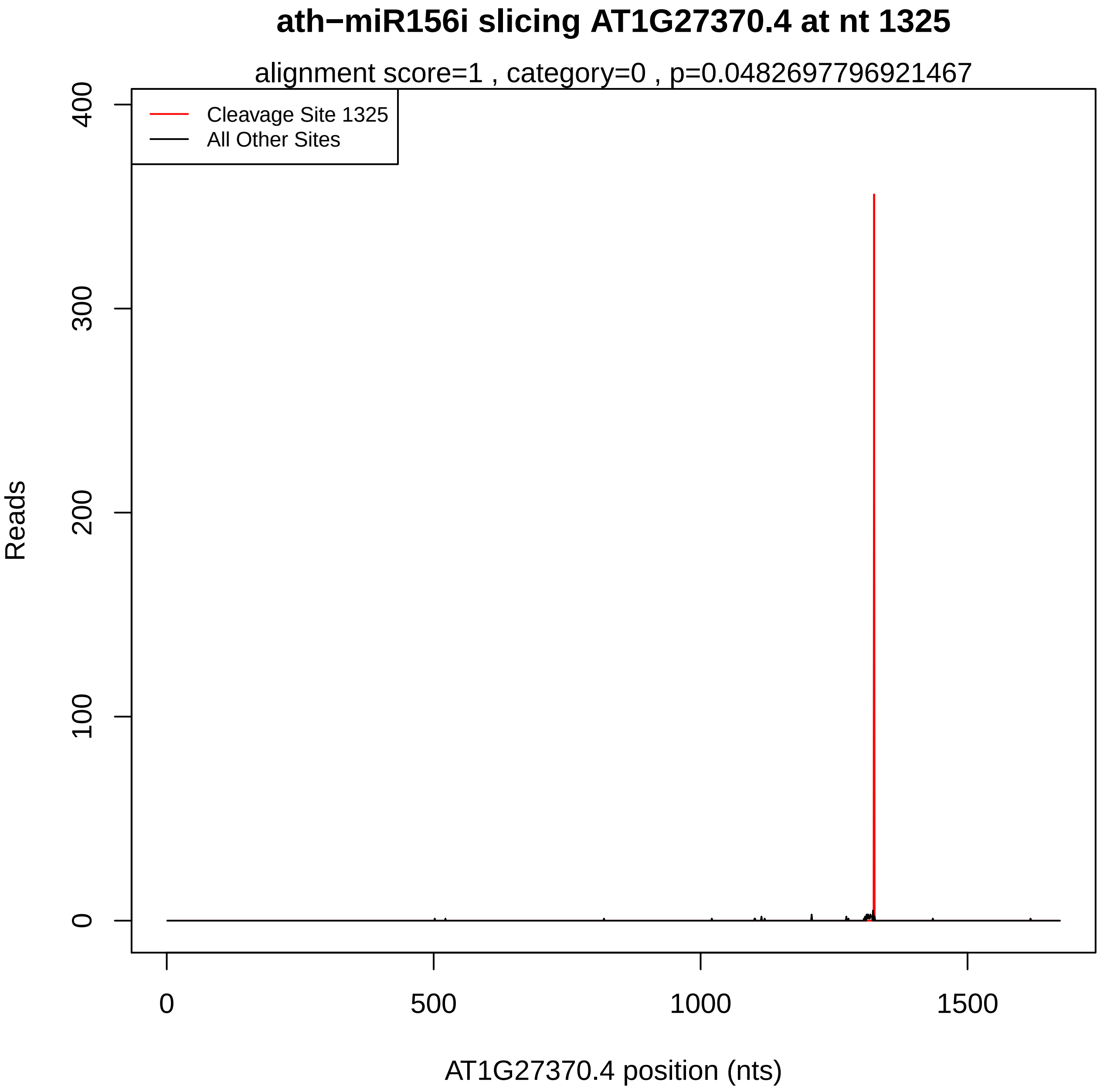
ath−miR156i slicing AT1G27370.4 at nt 1325
alignment score=1 , category=0 , p=0.0482697796921467
Cleavage Site 1325
All Other Sites
Reads
0
100
200
300
400
0
500
1000
1500
AT1G27370.4 position (nts)

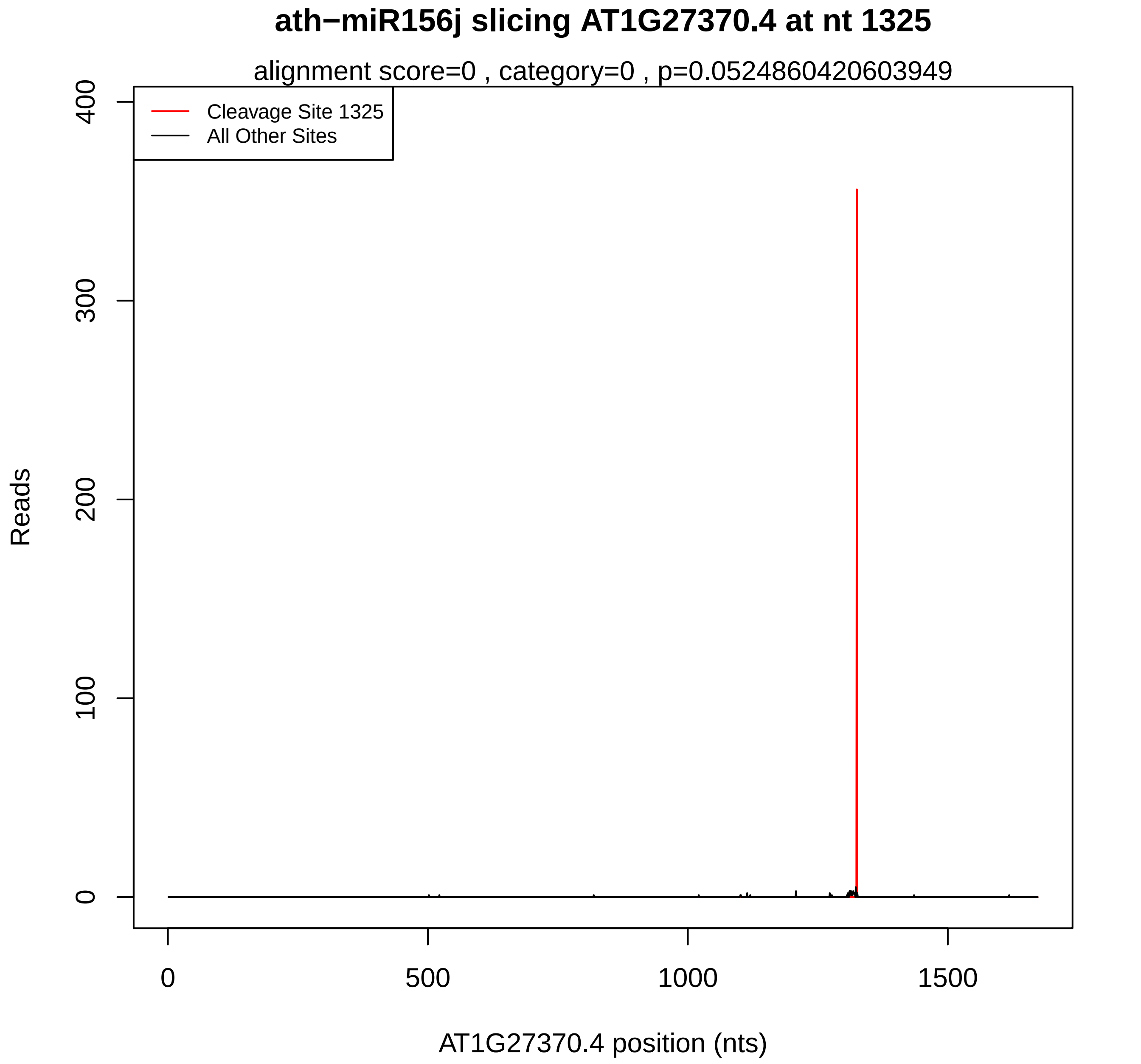
ath−miR156j slicing AT1G27370.4 at nt 1325
alignment score=0 , category=0 , p=0.0524860420603949
Cleavage Site 1325
All Other Sites
Reads
0
100
200
300
400
0
500
1000
1500
AT1G27370.4 position (nts)

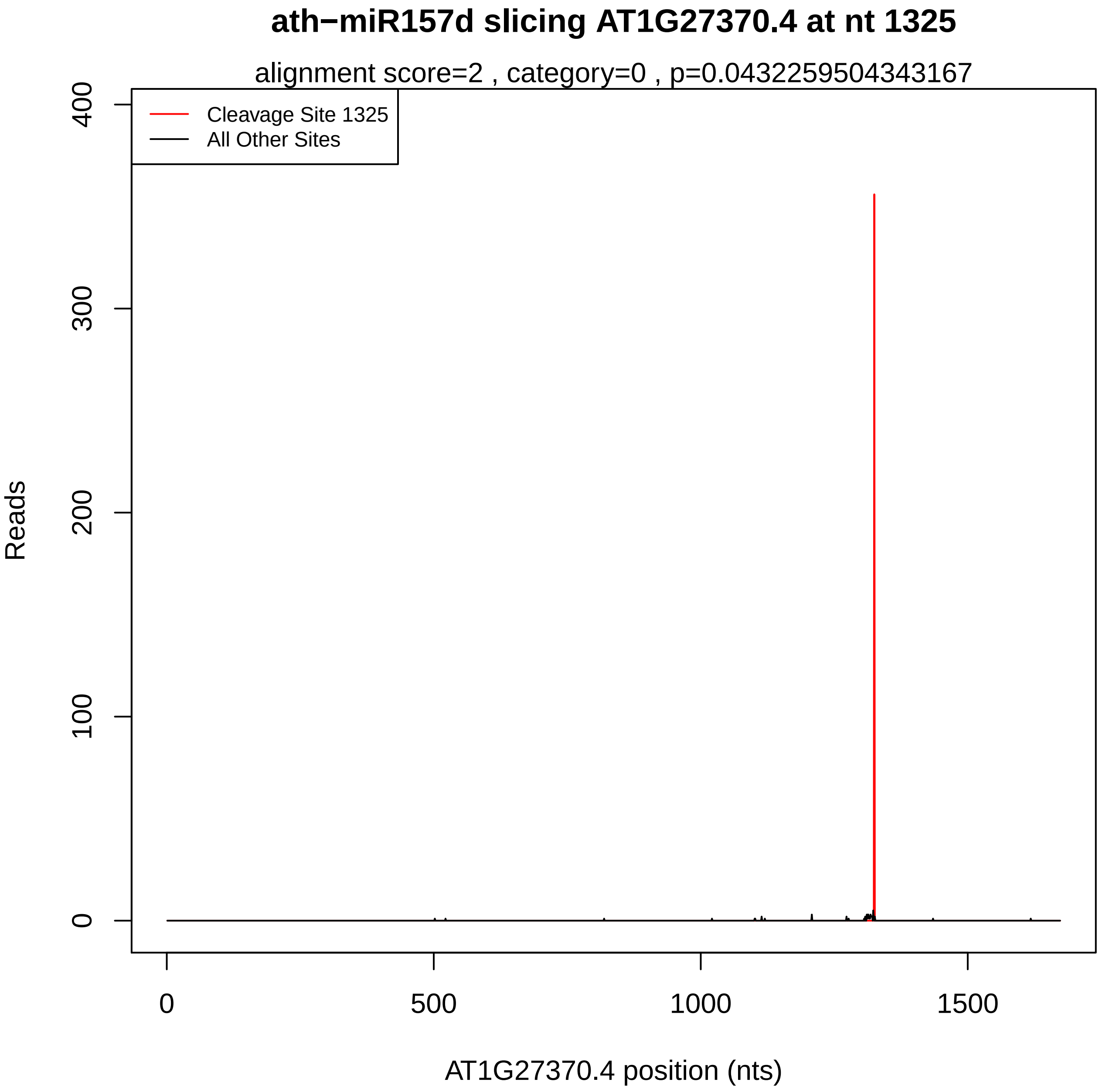

ath−miR157d slicing AT1G27370.4 at nt 1325
alignment score=2 , category=0 , p=0.0432259504343167
Cleavage Site 1325
All Other Sites
Reads
0
100
200
300
400
0
500
1000
1500
AT1G27370.4 position (nts)

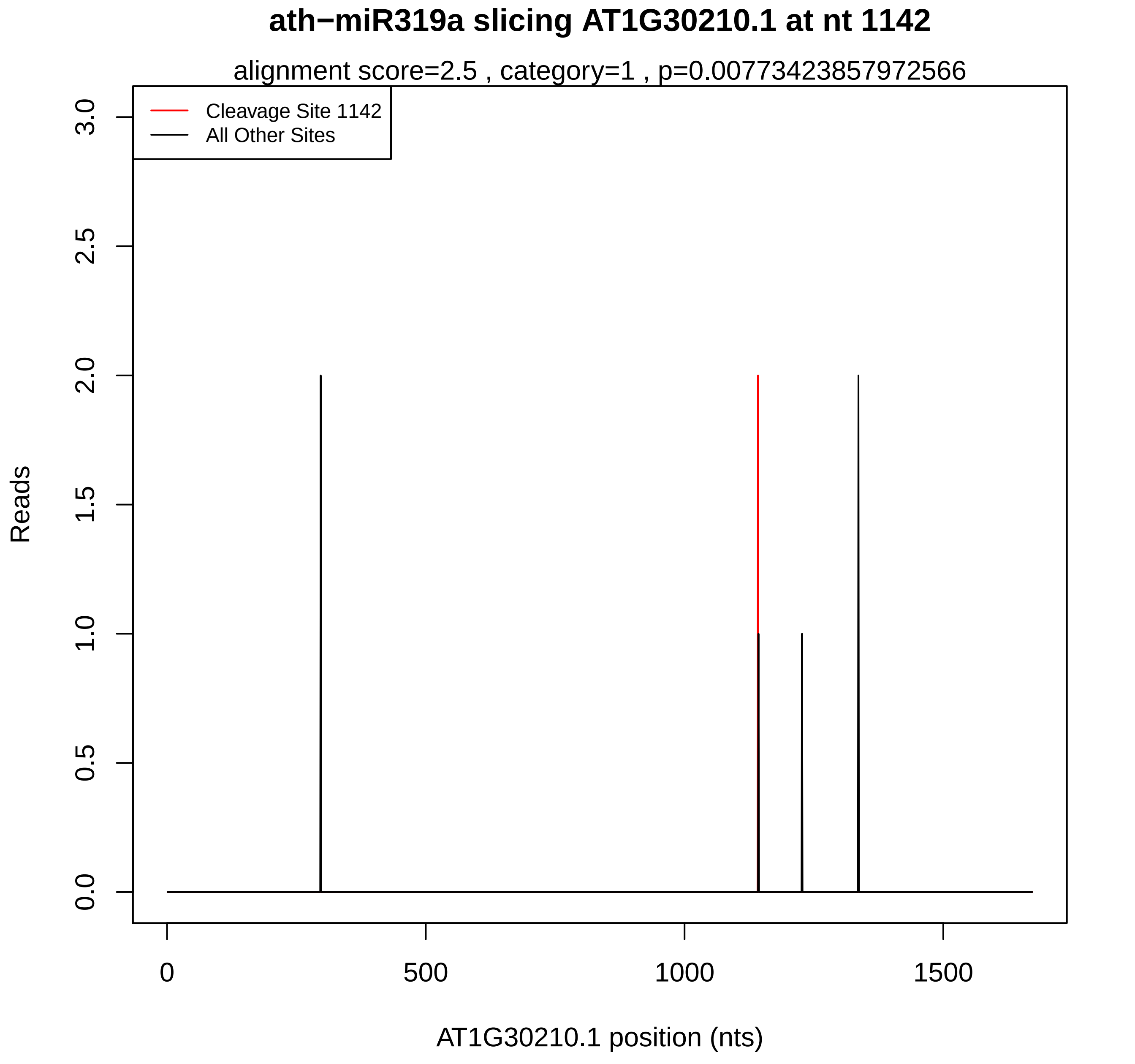
ath−miR319a slicing AT1G30210.1 at nt 1142
alignment score=2.5 , category=1 , p=0.00773423857972566
Cleavage Site 1142
All Other Sites
Reads
0.0
0.5
1.0
1.5
2.0
2.5
3.0
0
500
1000
1500
AT1G30210.1 position (nts)

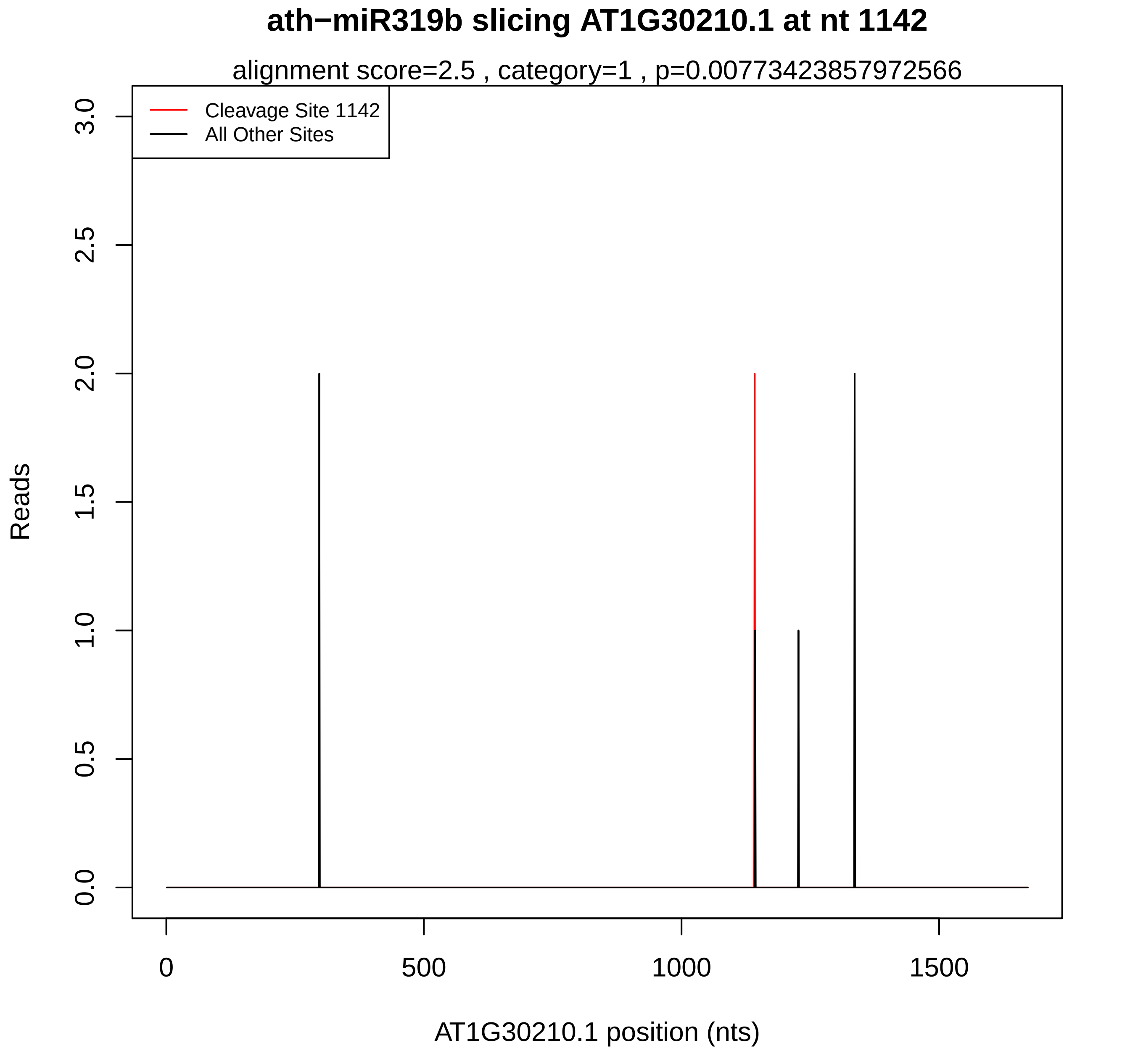

ath−miR319b slicing AT1G30210.1 at nt 1142
alignment score=2.5 , category=1 , p=0.00773423857972566
Cleavage Site 1142
All Other Sites
Reads
0.0
0.5
1.0
1.5
2.0
2.5
3.0
0
500
1000
1500
AT1G30210.1 position (nts)

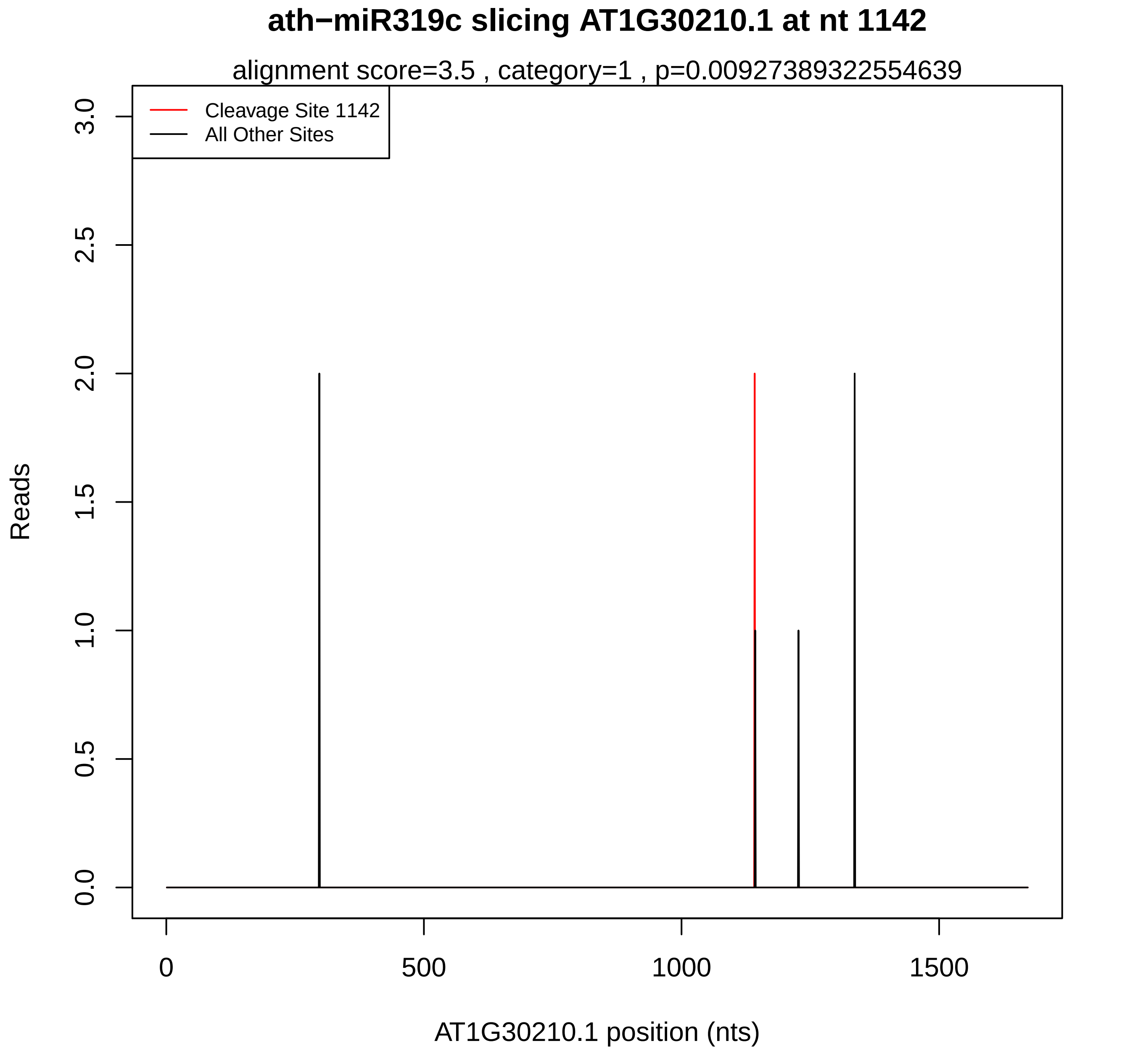

ath−miR319c slicing AT1G30210.1 at nt 1142
alignment score=3.5 , category=1 , p=0.00927389322554639
Cleavage Site 1142
All Other Sites
Reads
0.0
0.5
1.0
1.5
2.0
2.5
3.0
0
500
1000
1500
AT1G30210.1 position (nts)

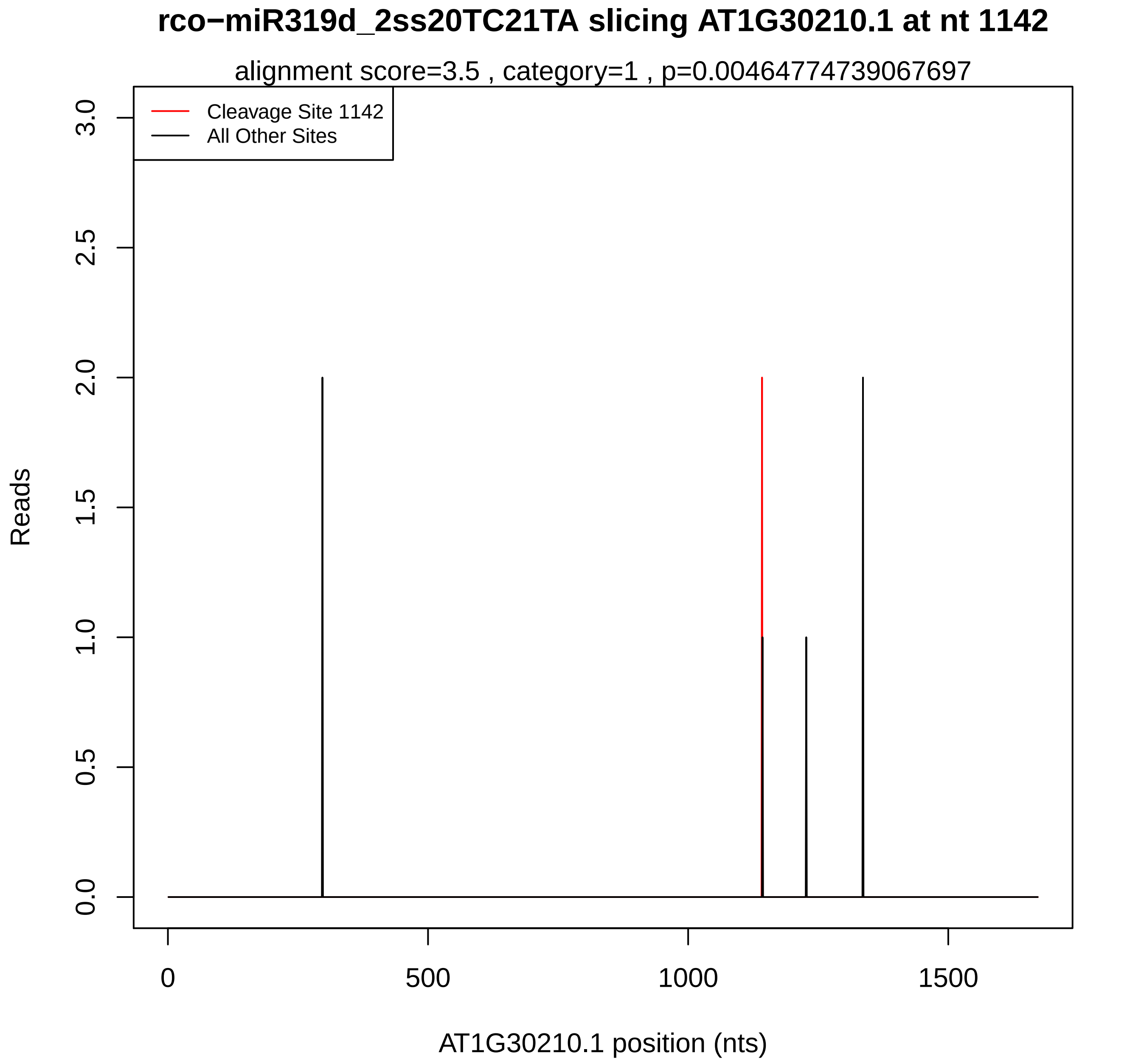
rco−miR319d_2ss20TC21TA slicing AT1G30210.1 at nt 1142
alignment score=3.5 , category=1 , p=0.00464774739067697
Cleavage Site 1142
All Other Sites
Reads
0.0
0.5
1.0
1.5
2.0
2.5
3.0
0
500
1000
1500
AT1G30210.1 position (nts)

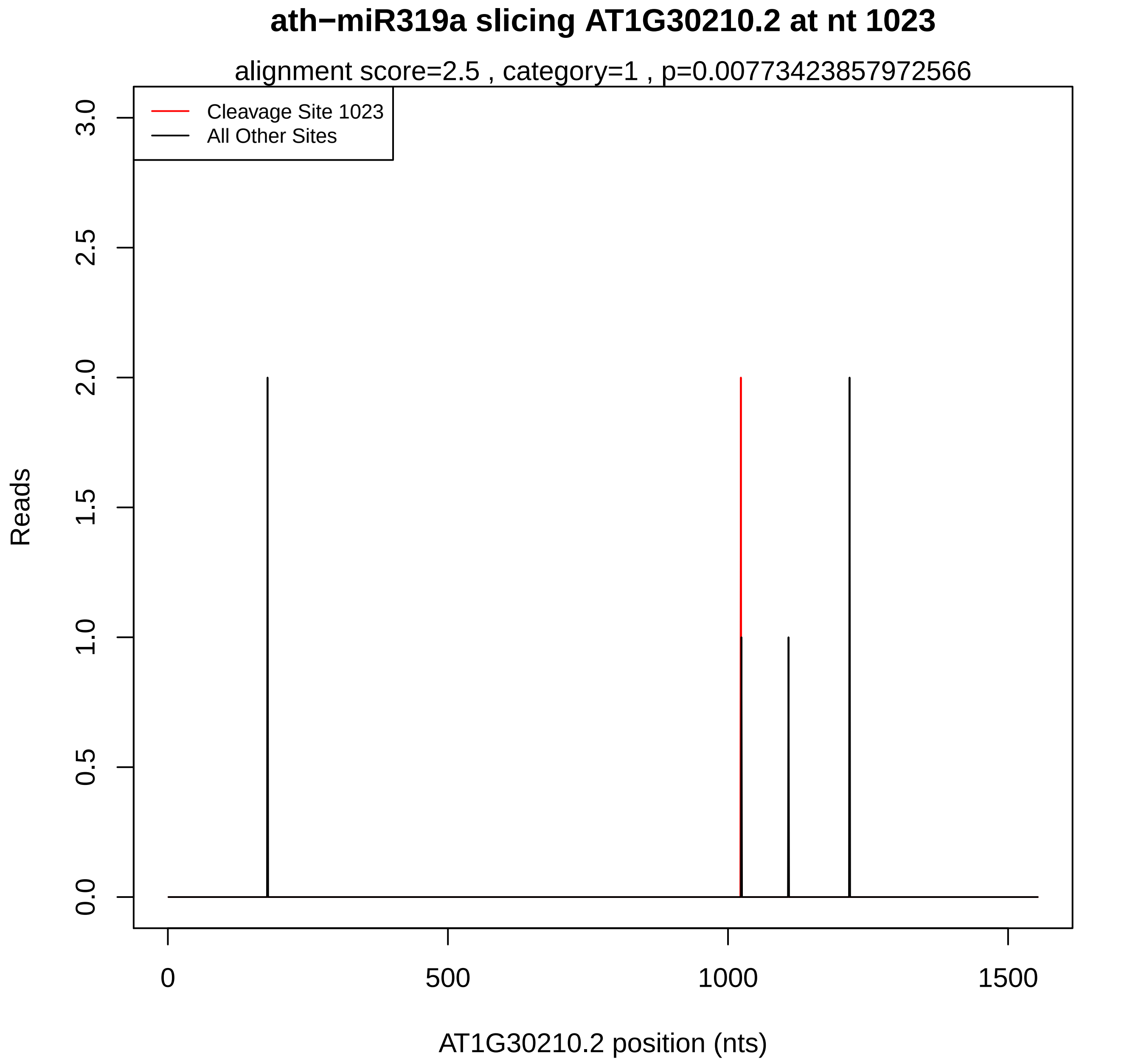
ath−miR319a slicing AT1G30210.2 at nt 1023
alignment score=2.5 , category=1 , p=0.00773423857972566
Cleavage Site 1023
All Other Sites
Reads
0.0
0.5
1.0
1.5
2.0
2.5
3.0
0
500
1000
1500
AT1G30210.2 position (nts)

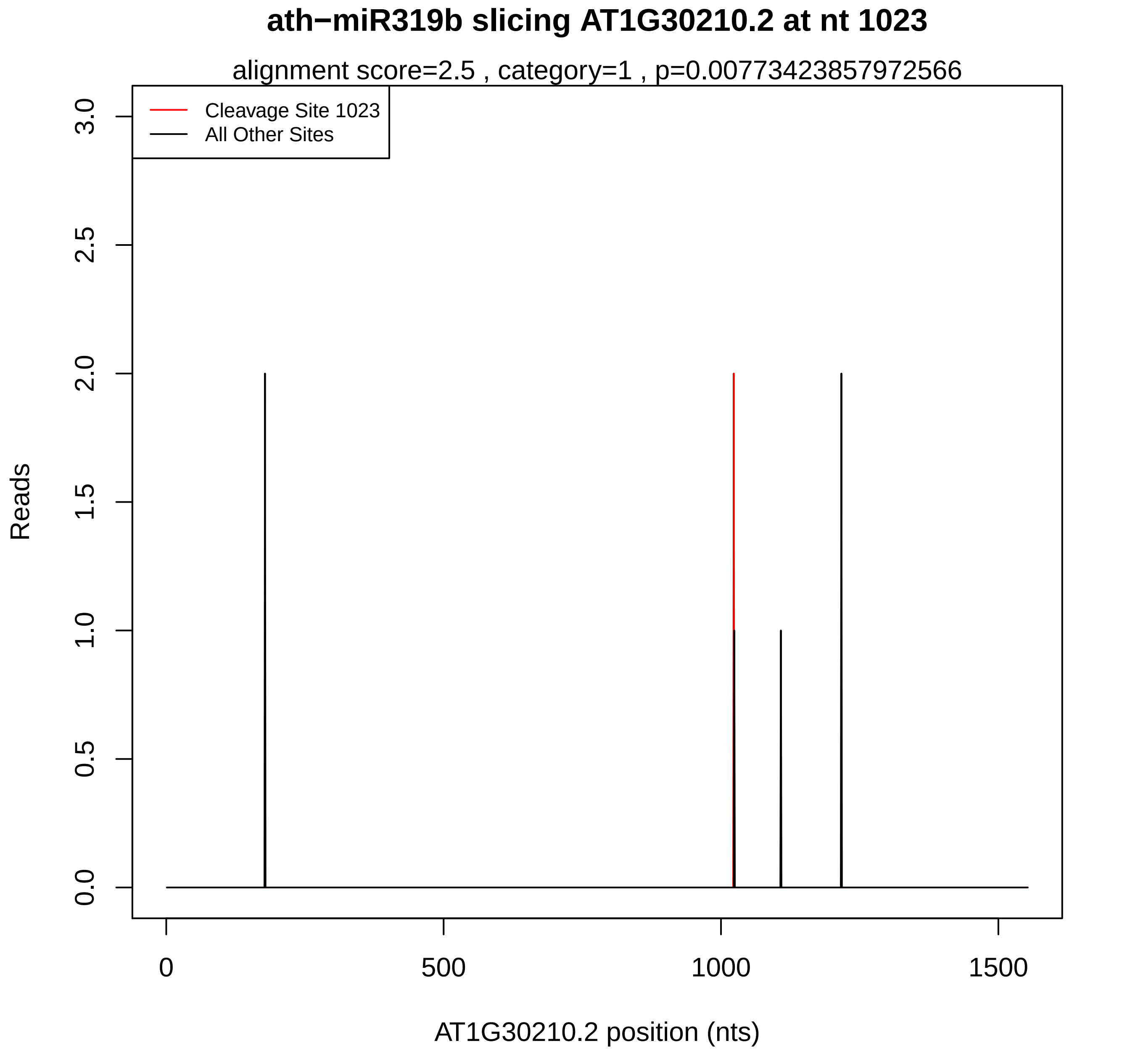

ath−miR319b slicing AT1G30210.2 at nt 1023
alignment score=2.5 , category=1 , p=0.00773423857972566
Cleavage Site 1023
All Other Sites
Reads
0.0
0.5
1.0
1.5
2.0
2.5
3.0
0
500
1000
1500
AT1G30210.2 position (nts)

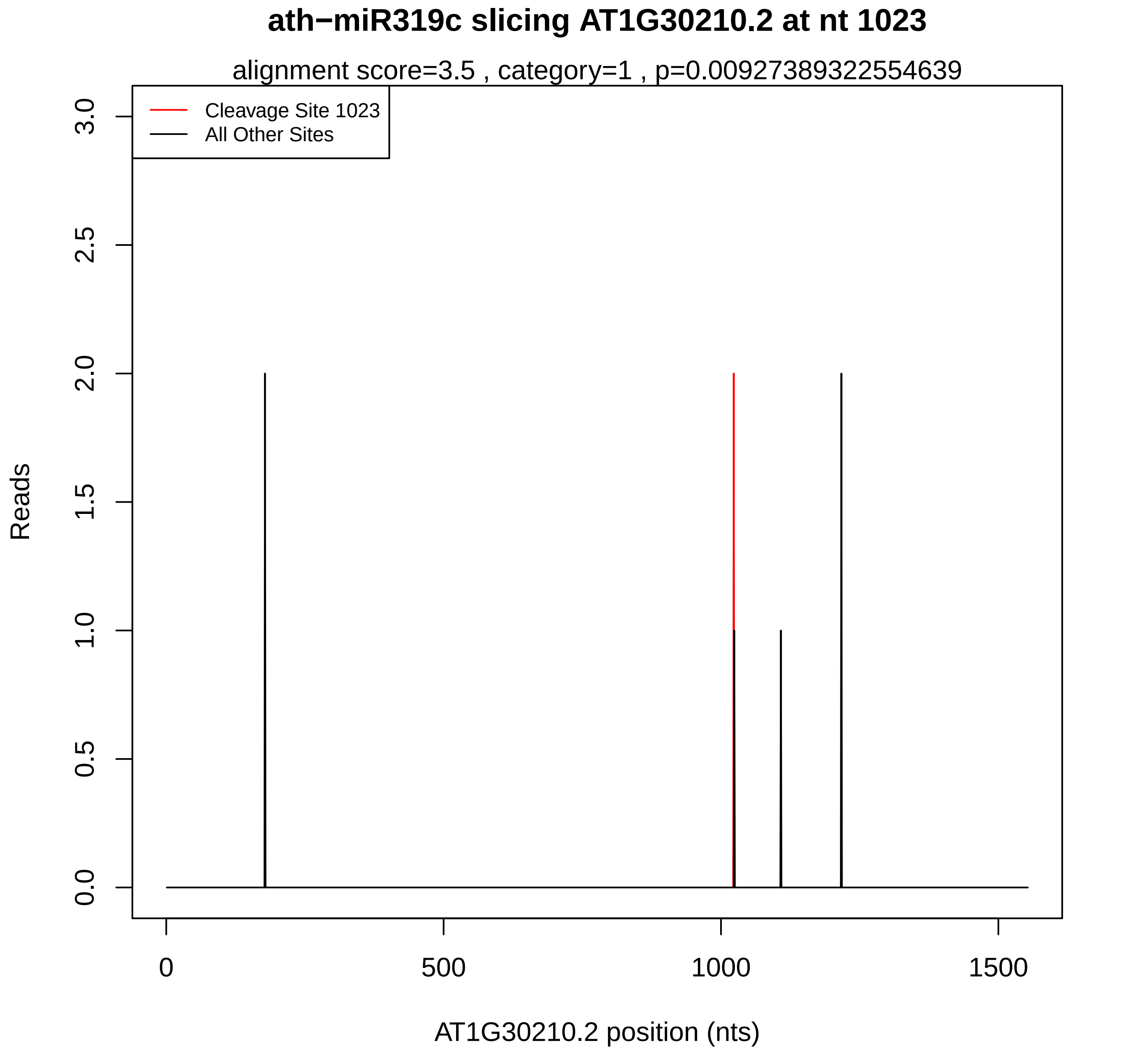

ath−miR319c slicing AT1G30210.2 at nt 1023
alignment score=3.5 , category=1 , p=0.00927389322554639
Cleavage Site 1023
All Other Sites
Reads
0.0
0.5
1.0
1.5
2.0
2.5
3.0
0
500
1000
1500
AT1G30210.2 position (nts)

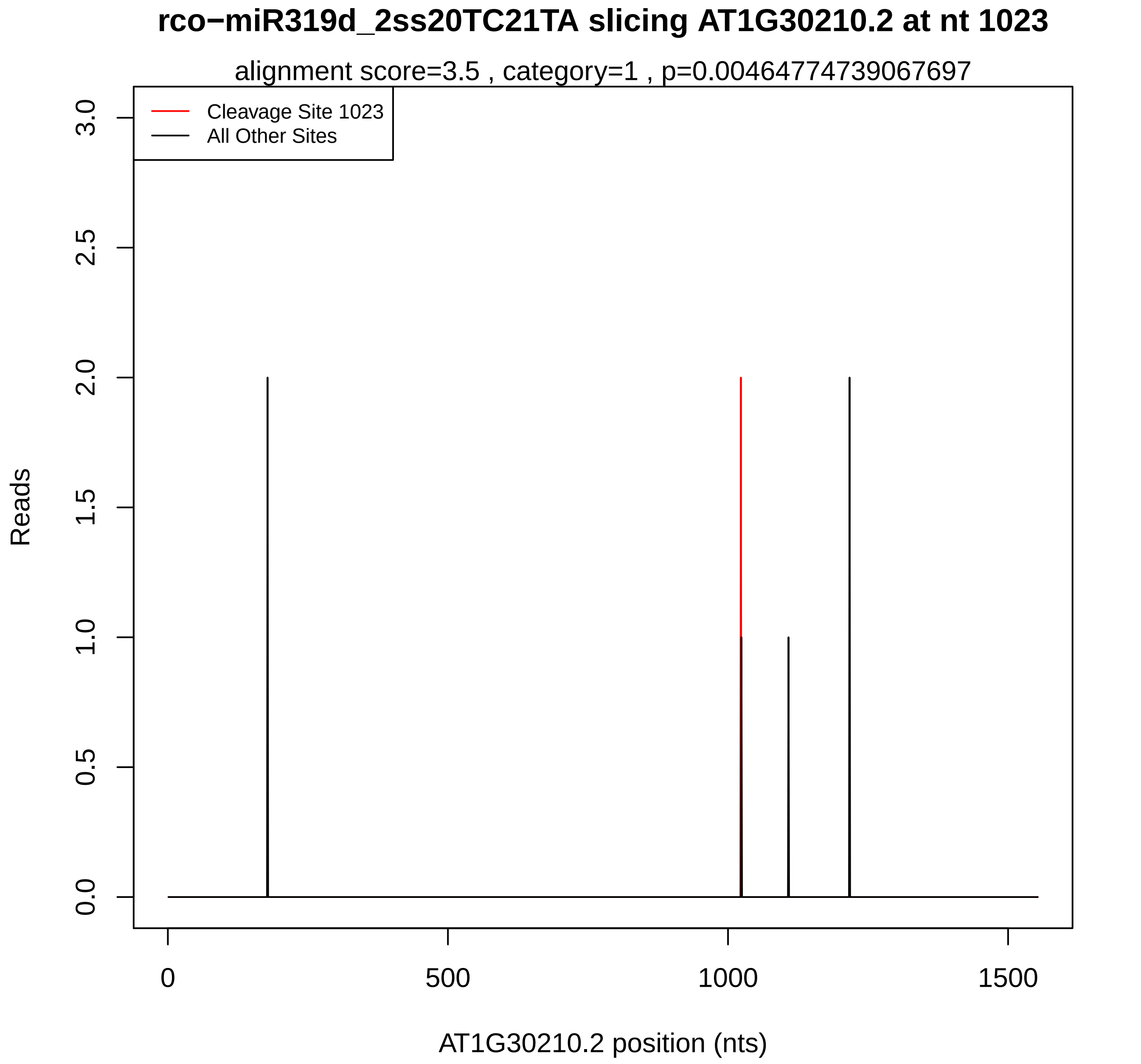
rco−miR319d_2ss20TC21TA slicing AT1G30210.2 at nt 1023
alignment score=3.5 , category=1 , p=0.00464774739067697
Cleavage Site 1023
All Other Sites
Reads
0.0
0.5
1.0
1.5
2.0
2.5
3.0
0
500
1000
1500
AT1G30210.2 position (nts)

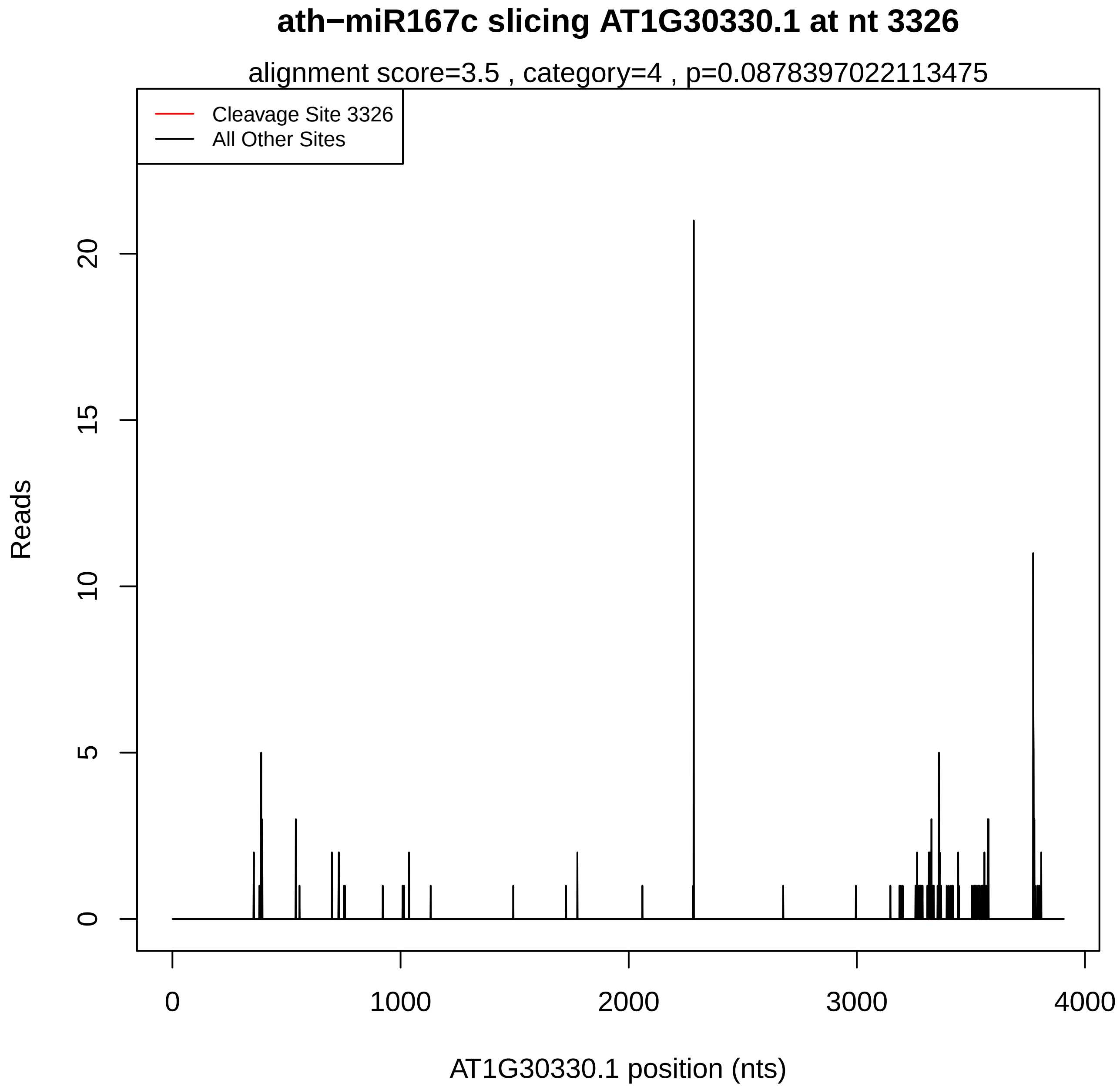
ath−miR167c slicing AT1G30330.1 at nt 3326
alignment score=3.5 , category=4 , p=0.0878397022113475
Cleavage Site 3326
All Other Sites
Reads
0
5
10
15
20
0
1000
2000
3000
4000
AT1G30330.1 position (nts)

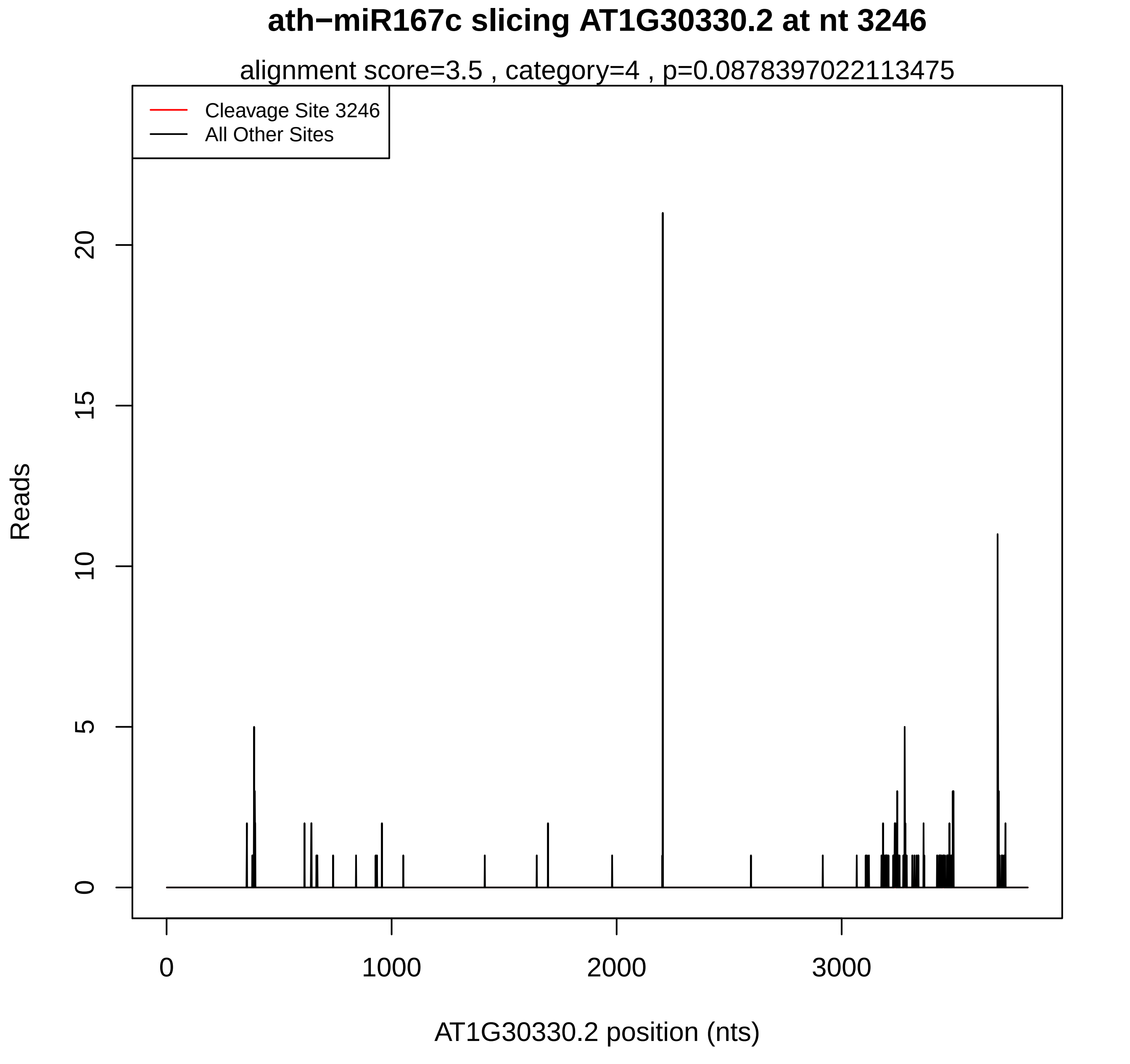
ath−miR167c slicing AT1G30330.2 at nt 3246
alignment score=3.5 , category=4 , p=0.0878397022113475
Cleavage Site 3246
All Other Sites
Reads
0
5
10
15
20
0
1000
2000
3000
AT1G30330.2 position (nts)

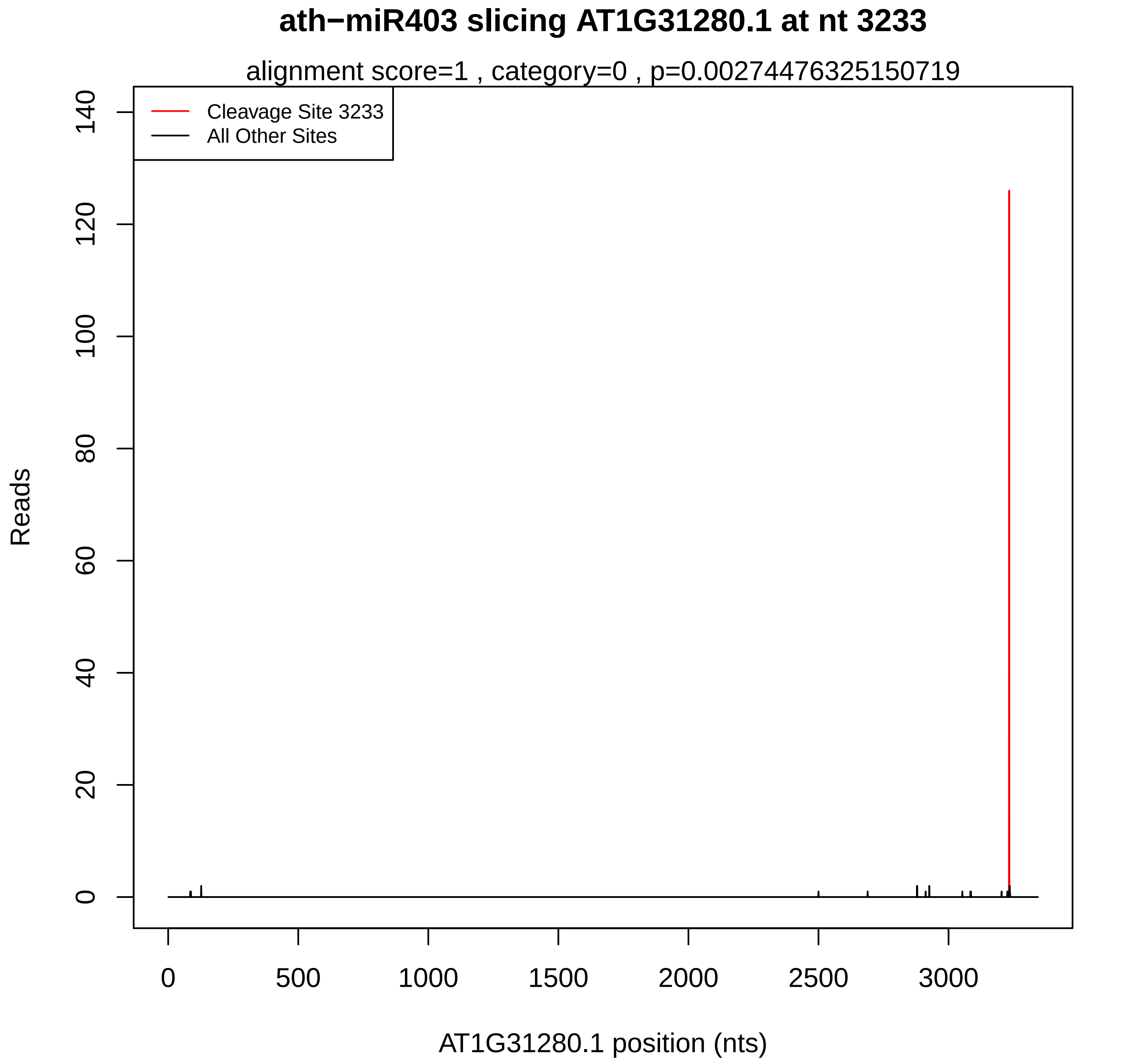
ath−miR403 slicing AT1G31280.1 at nt 3233
alignment score=1 , category=0 , p=0.00274476325150719
Cleavage Site 3233
All Other Sites
Reads
0
20
40
60
80
100
120
140
0
500
1000
1500
2000
2500
3000
AT1G31280.1 position (nts)

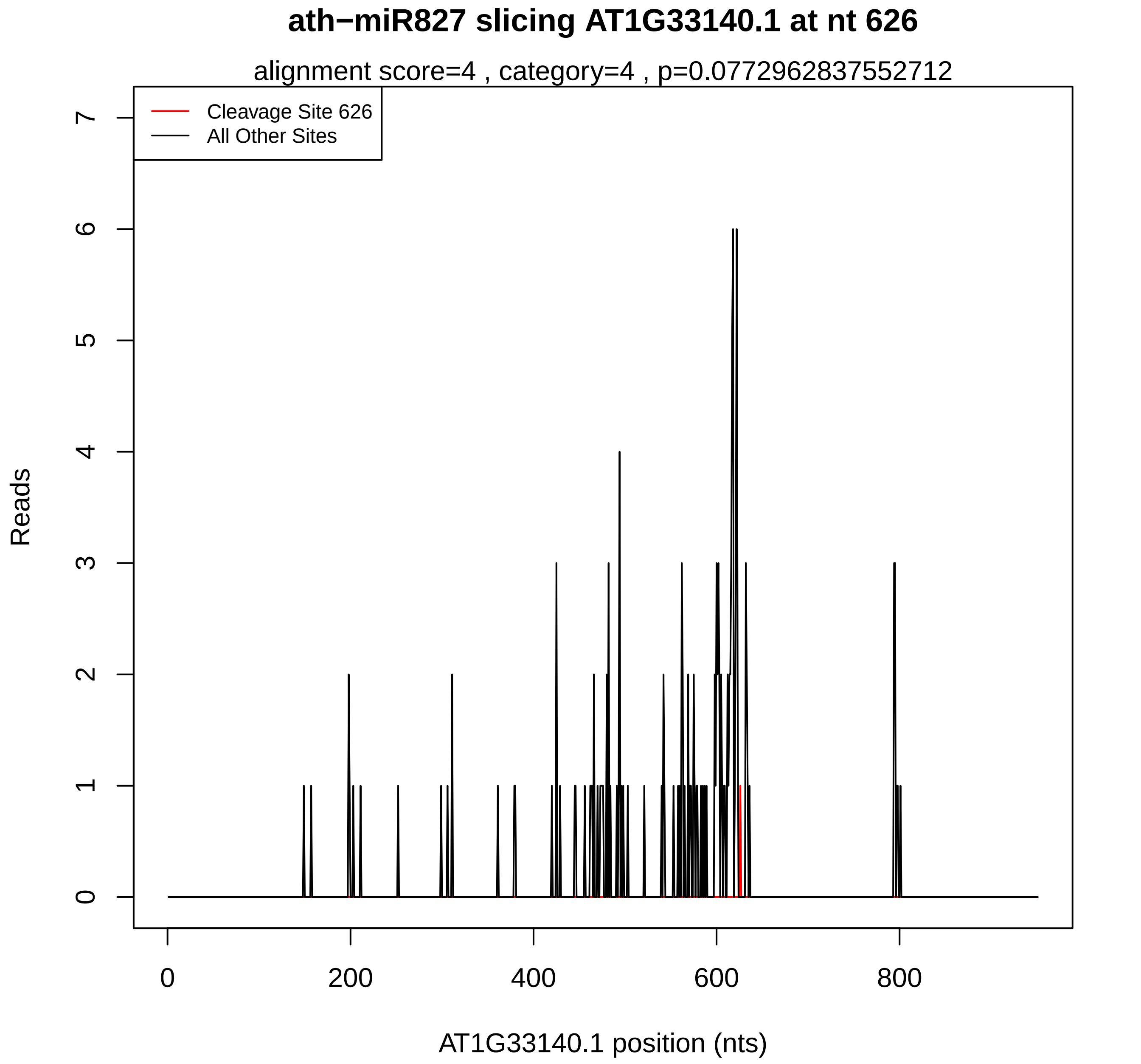

ath−miR827 slicing AT1G33140.1 at nt 626
alignment score=4 , category=4 , p=0.0772962837552712
Cleavage Site 626
All Other Sites
Reads
0
1
2
3
4
5
6
7
0
200
400
600
800
AT1G33140.1 position (nts)

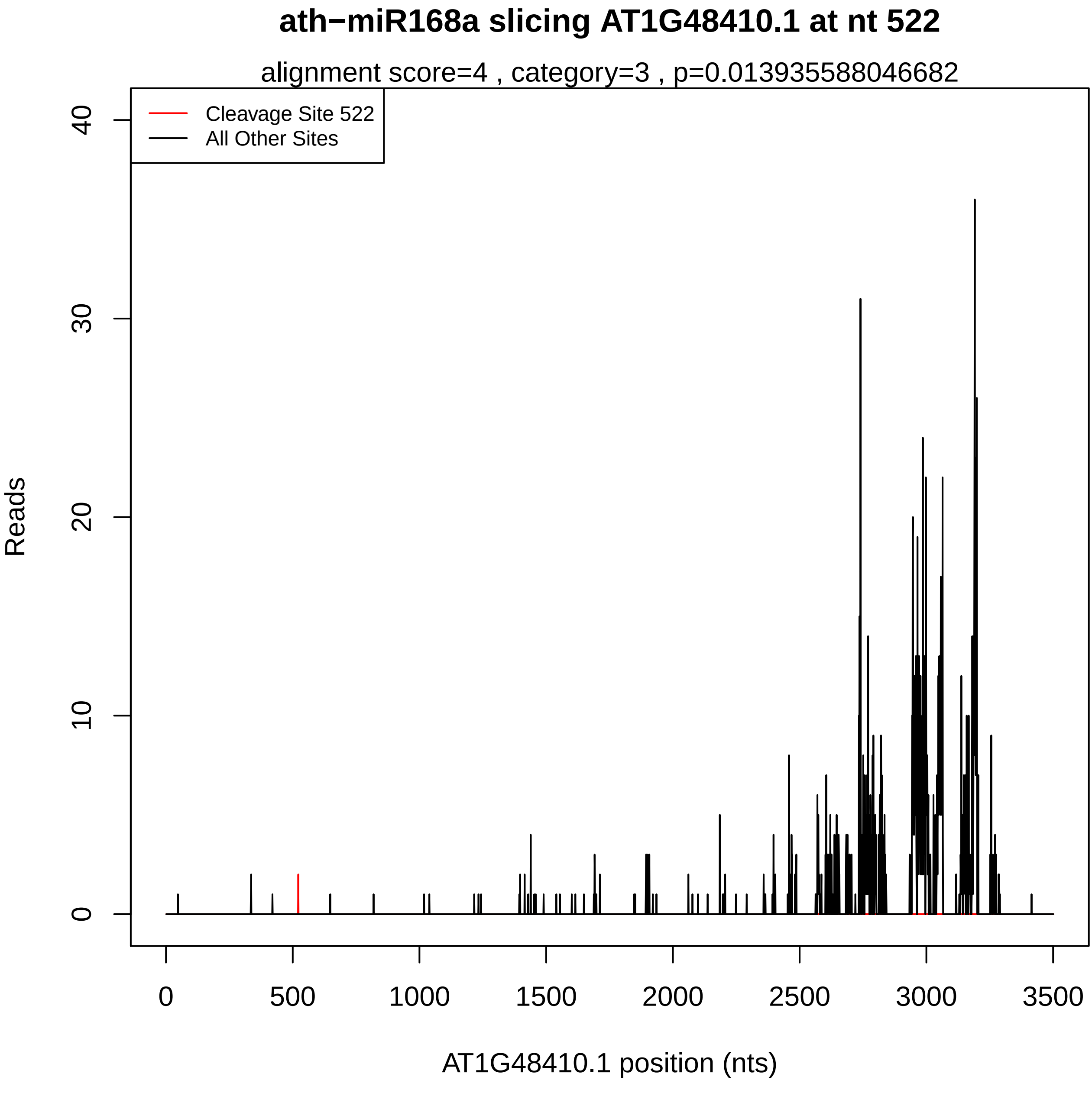

ath−miR168a slicing AT1G48410.1 at nt 522
alignment score=4 , category=3 , p=0.013935588046682
Cleavage Site 522
All Other Sites
Reads
AT1G48410.1 position (nts)
0
10
20
30
40
0
500
1000
1500
2000
2500
3000
3500

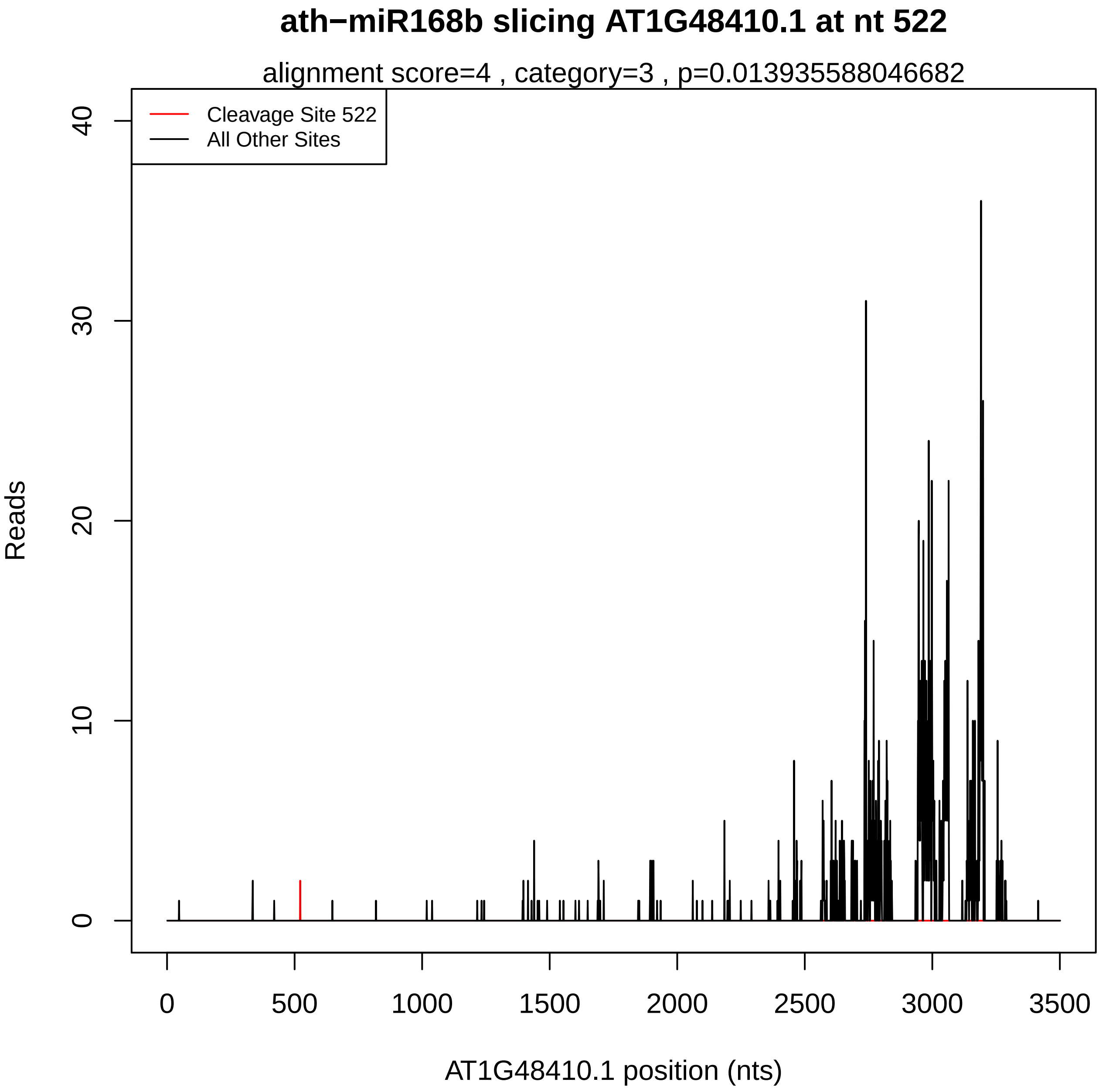
ath−miR168b slicing AT1G48410.1 at nt 522
alignment score=4 , category=3 , p=0.013935588046682
Cleavage Site 522
All Other Sites
Reads
0
10
20
30
40
0
500
1000
1500
2000
2500
3000
3500
AT1G48410.1 position (nts)

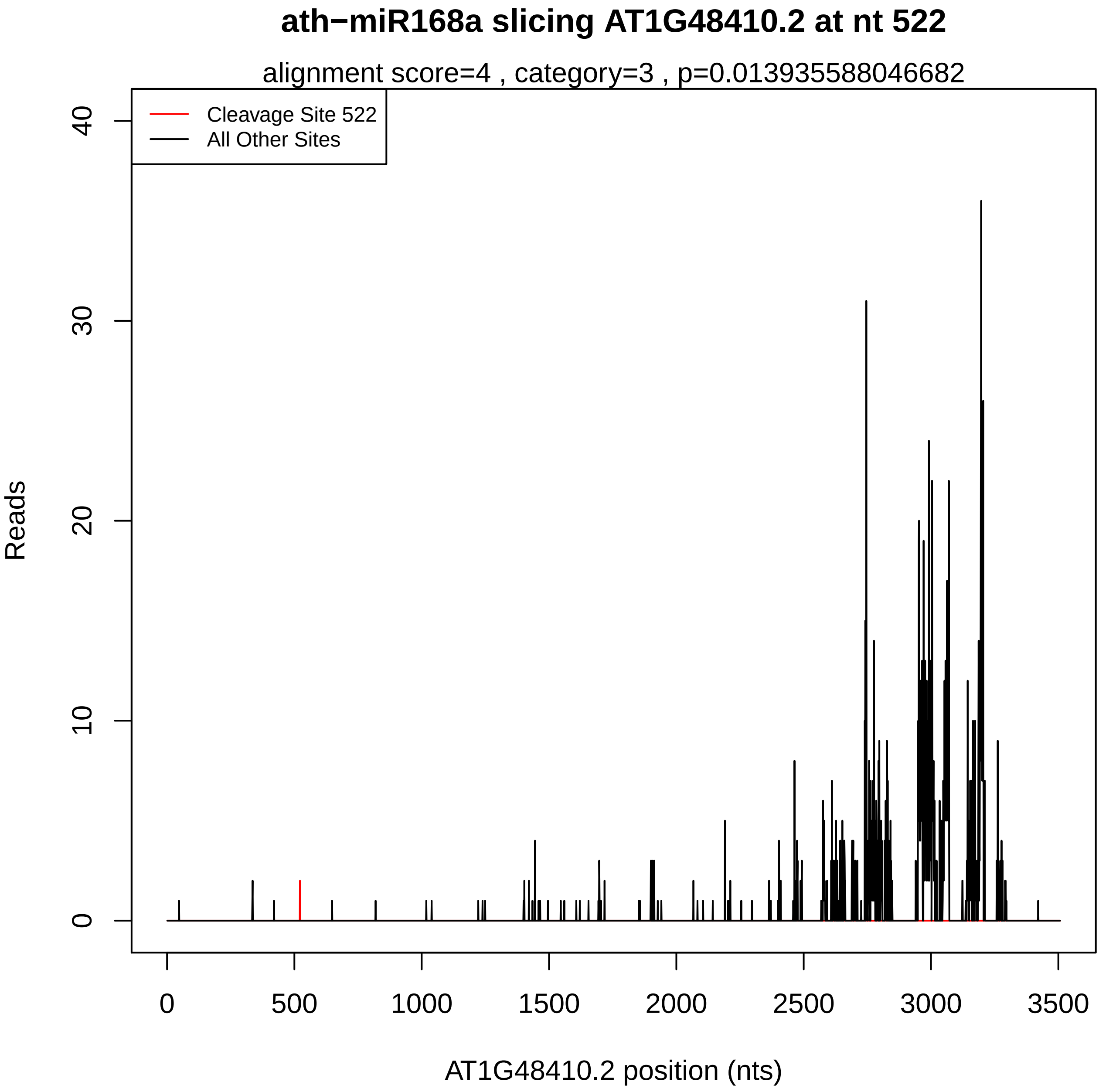
ath−miR168a slicing AT1G48410.2 at nt 522
alignment score=4 , category=3 , p=0.013935588046682
Cleavage Site 522
All Other Sites
Reads
0
10
20
30
40
0
500
1000
1500
2000
2500
3000
3500
AT1G48410.2 position (nts)

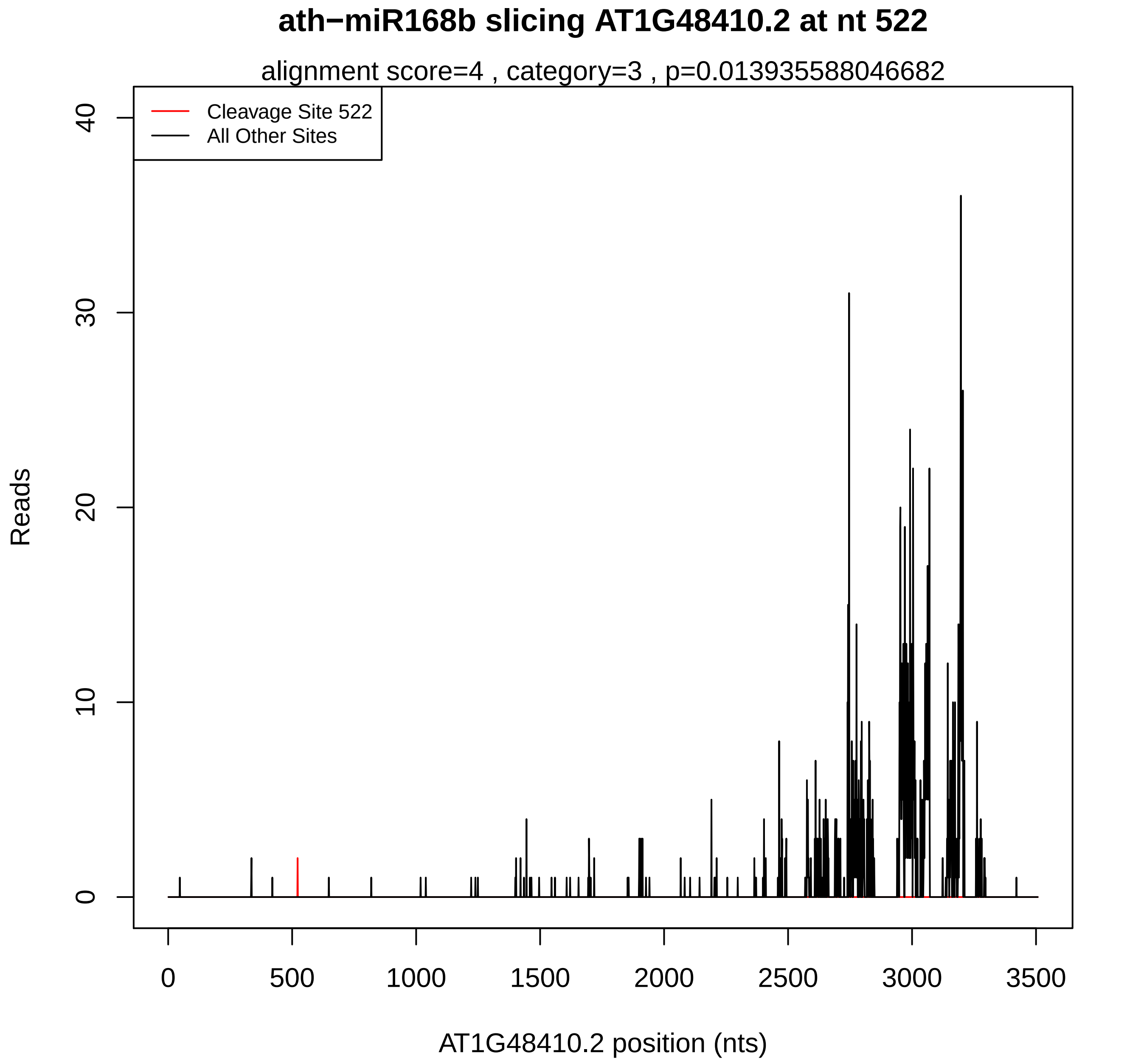
ath−miR168b slicing AT1G48410.2 at nt 522
alignment score=4 , category=3 , p=0.013935588046682
Cleavage Site 522
All Other Sites
Reads
0
10
20
30
40
0
500
1000
1500
2000
2500
3000
3500
AT1G48410.2 position (nts)

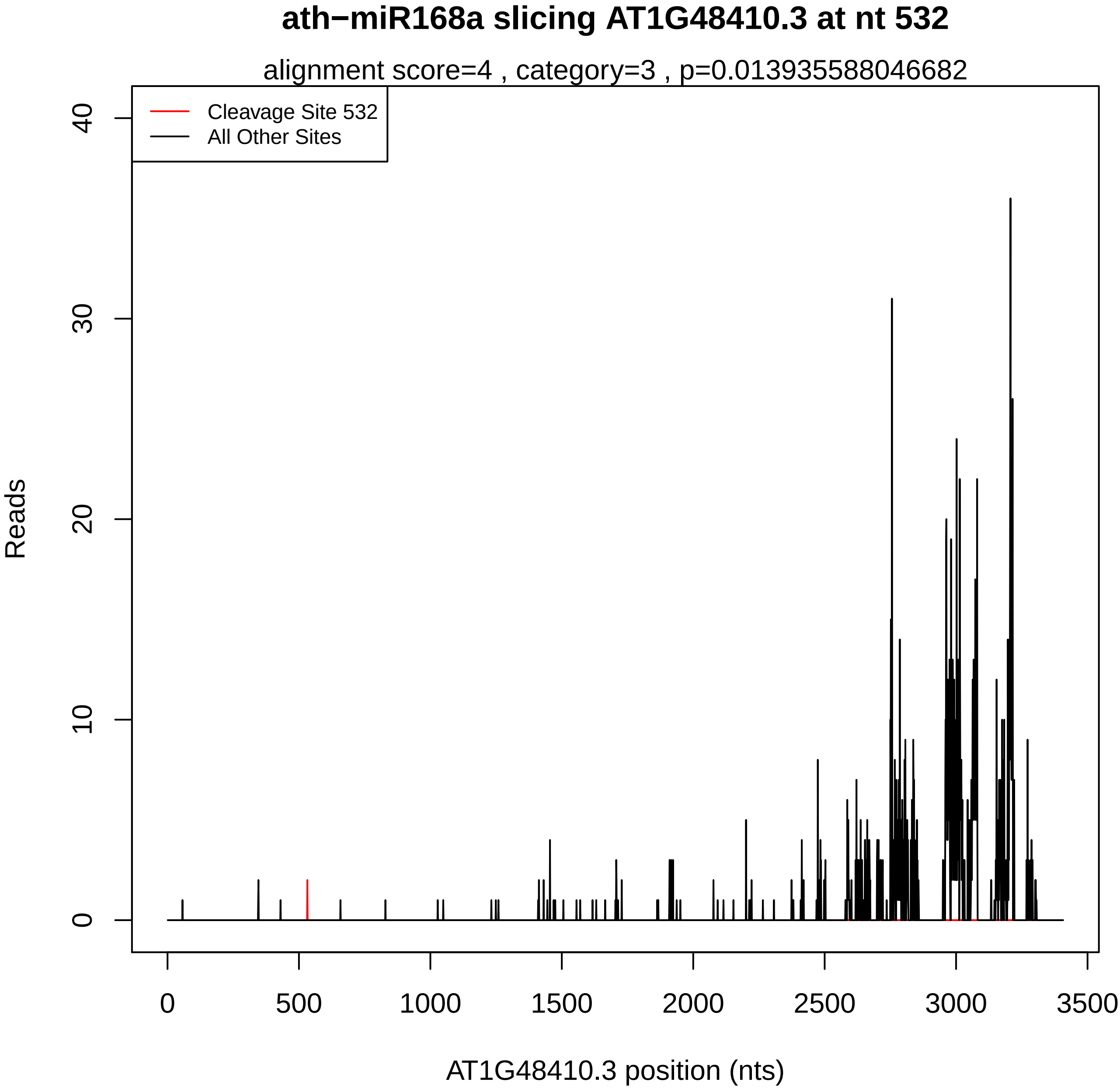

ath−miR168a slicing AT1G48410.3 at nt 532
alignment score=4 , category=3 , p=0.013935588046682
Cleavage Site 532
All Other Sites
Reads
0
10
20
30
40
0
500
1000
1500
2000
2500
3000
3500
AT1G48410.3 position (nts)

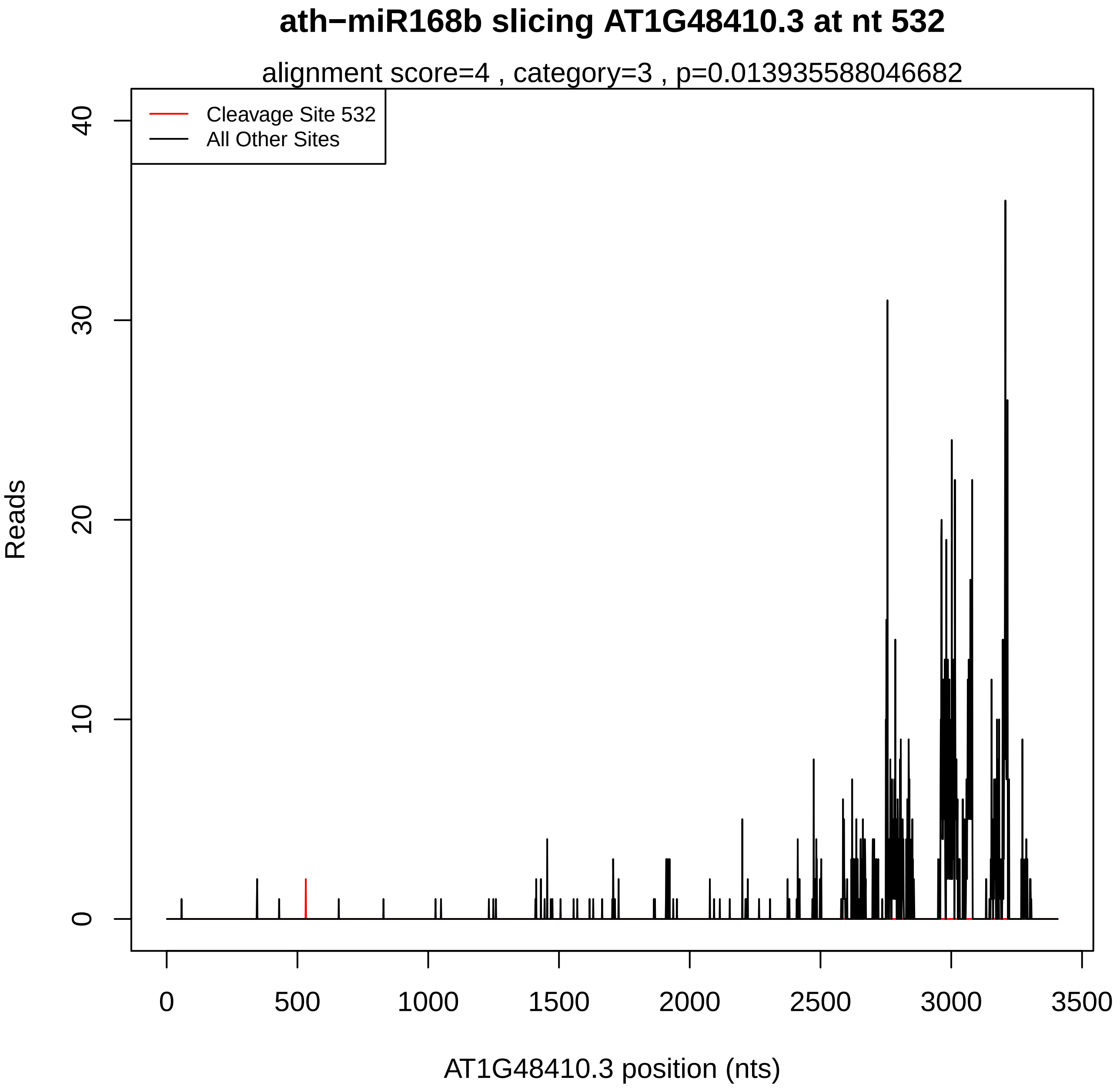
ath−miR168b slicing AT1G48410.3 at nt 532
alignment score=4 , category=3 , p=0.013935588046682
Cleavage Site 532
All Other Sites
Reads
0
10
20
30
40
0
500
1000
1500
2000
2500
3000
3500
AT1G48410.3 position (nts)

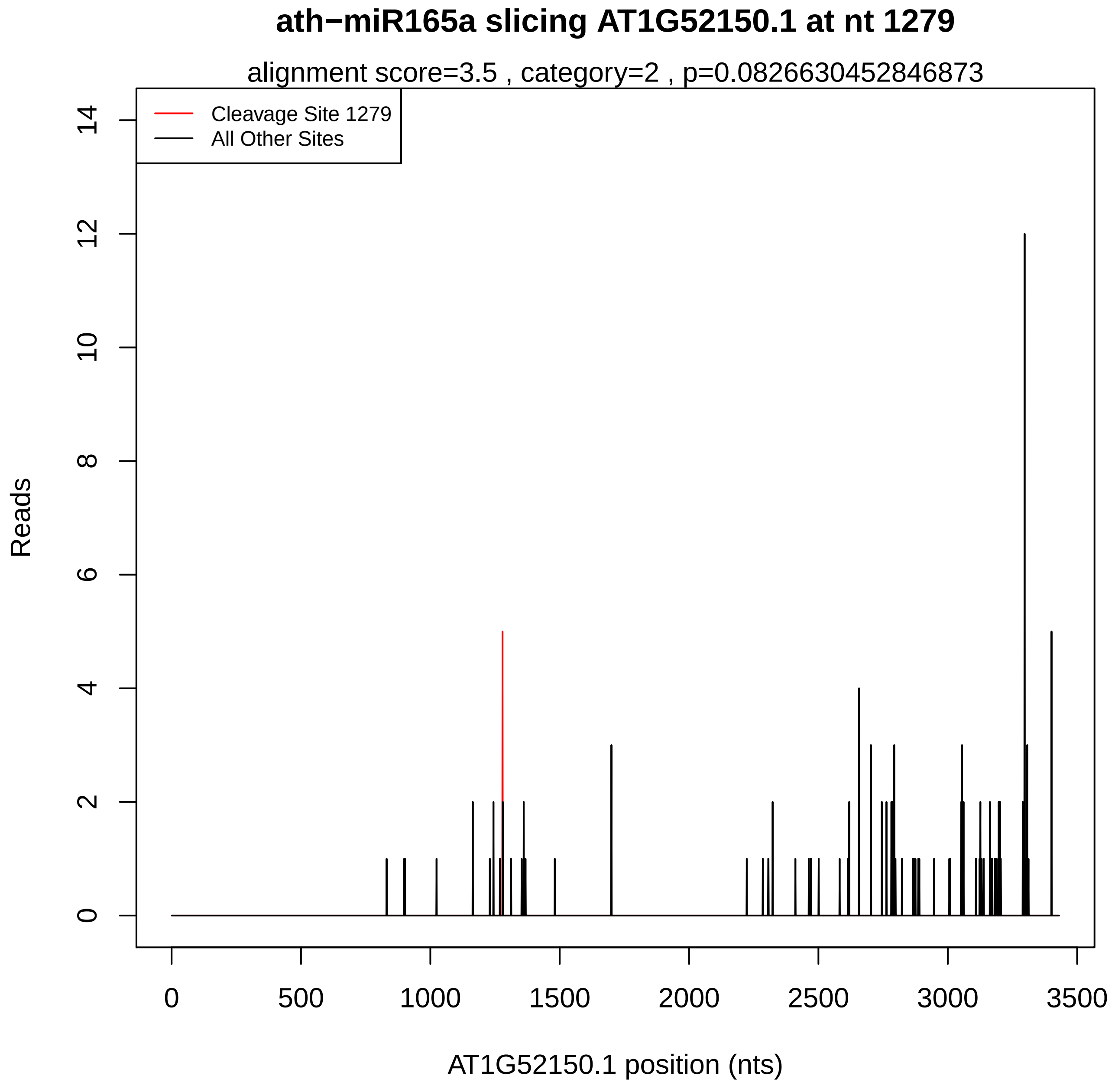
ath−miR165a slicing AT1G52150.1 at nt 1279
alignment score=3.5 , category=2 , p=0.0826630452846873
Cleavage Site 1279
All Other Sites
Reads
0
2
4
6
8
10
12
14
0
500
1000
1500
2000
2500
3000
3500
AT1G52150.1 position (nts)

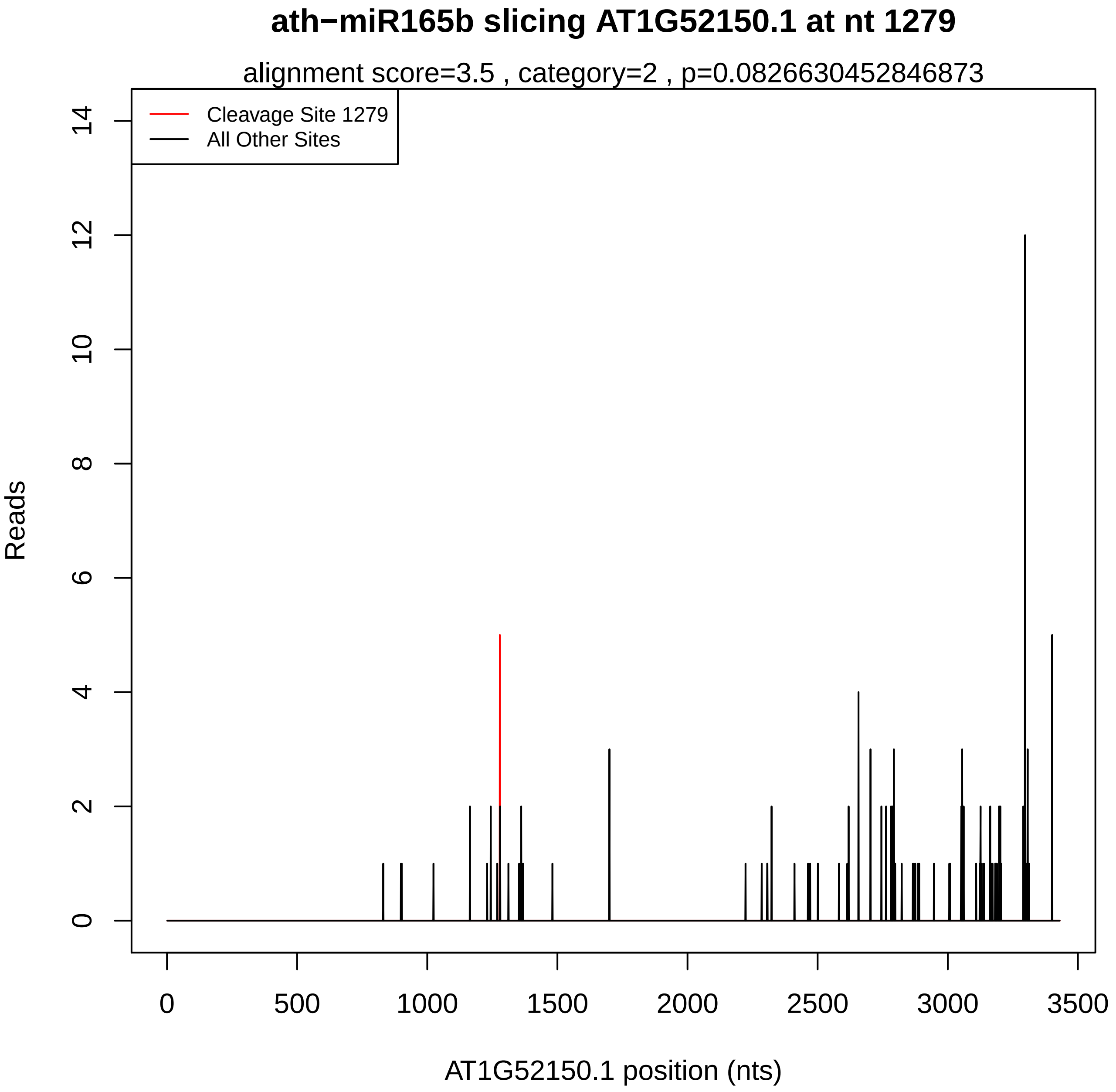

ath−miR165b slicing AT1G52150.1 at nt 1279
alignment score=3.5 , category=2 , p=0.0826630452846873
Cleavage Site 1279
All Other Sites
Reads
0
2
4
6
8
10
12
14
0
500
1000
1500
2000
2500
3000
3500
AT1G52150.1 position (nts)

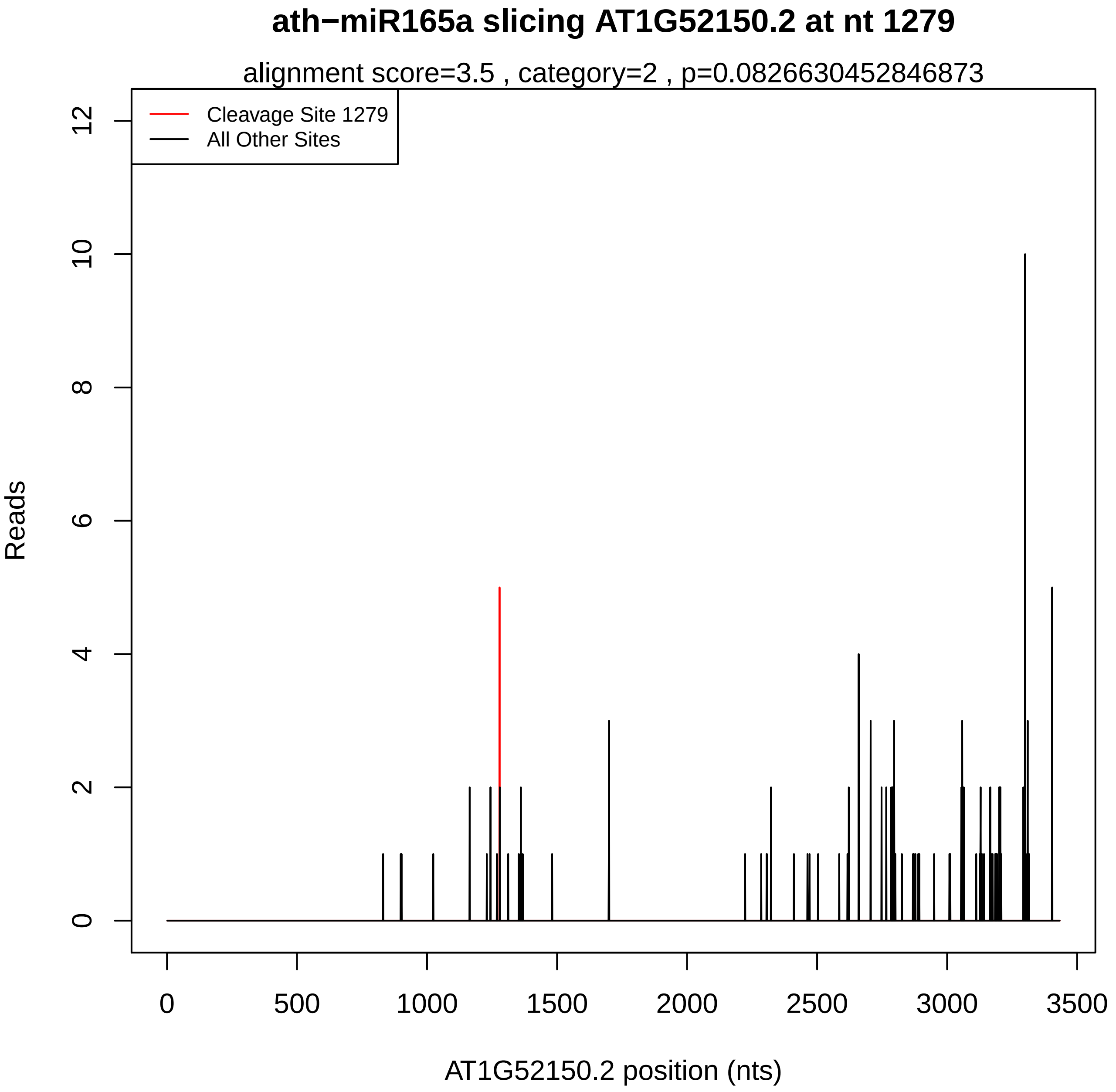
ath−miR165a slicing AT1G52150.2 at nt 1279
alignment score=3.5 , category=2 , p=0.0826630452846873
Cleavage Site 1279
All Other Sites
Reads
0
2
4
6
8
10
12
0
500
1000
1500
2000
2500
3000
3500
AT1G52150.2 position (nts)

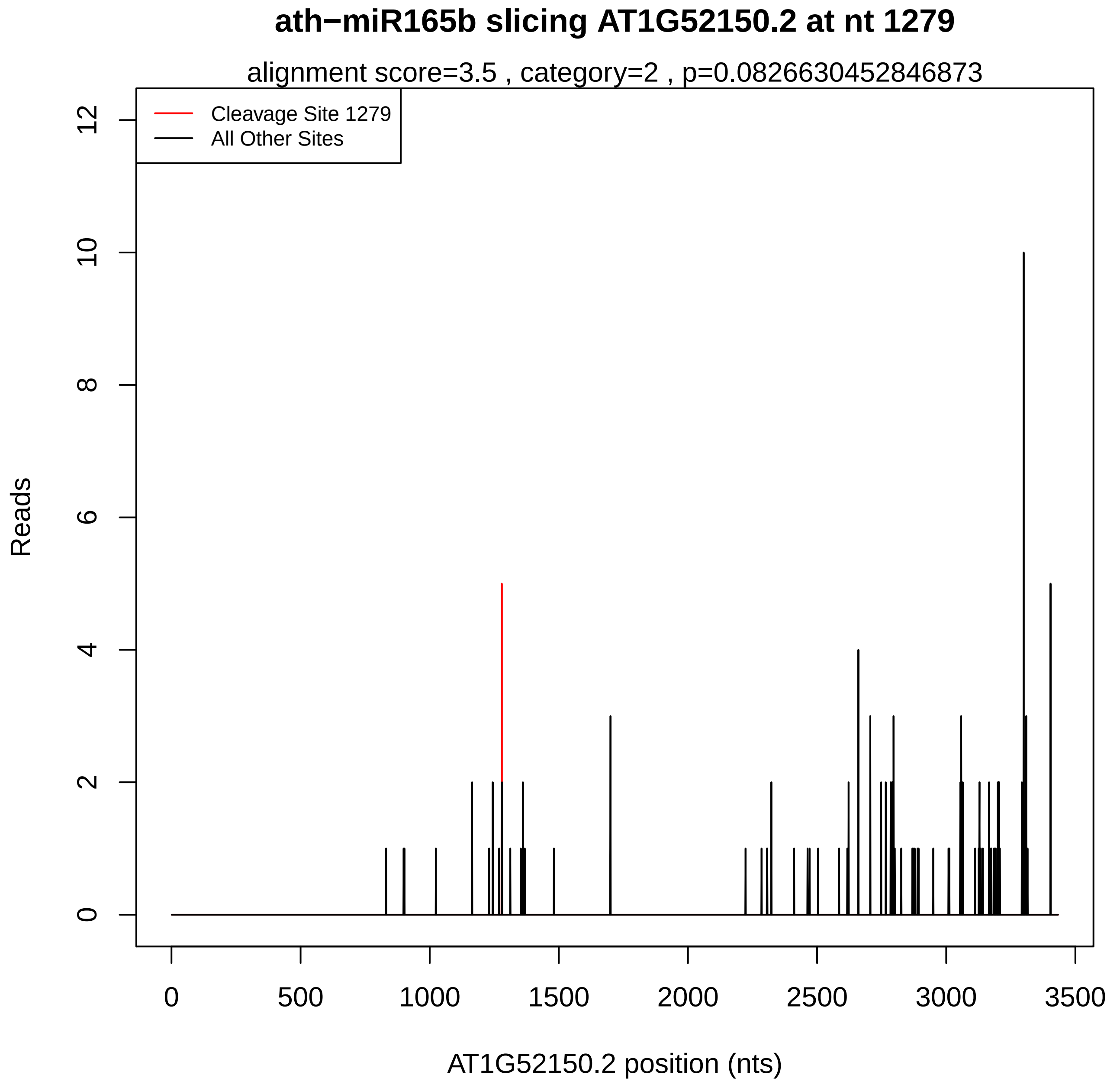

ath−miR165b slicing AT1G52150.2 at nt 1279
alignment score=3.5 , category=2 , p=0.0826630452846873
Cleavage Site 1279
All Other Sites
Reads
0
2
4
6
8
10
12
0
500
1000
1500
2000
2500
3000
3500
AT1G52150.2 position (nts)

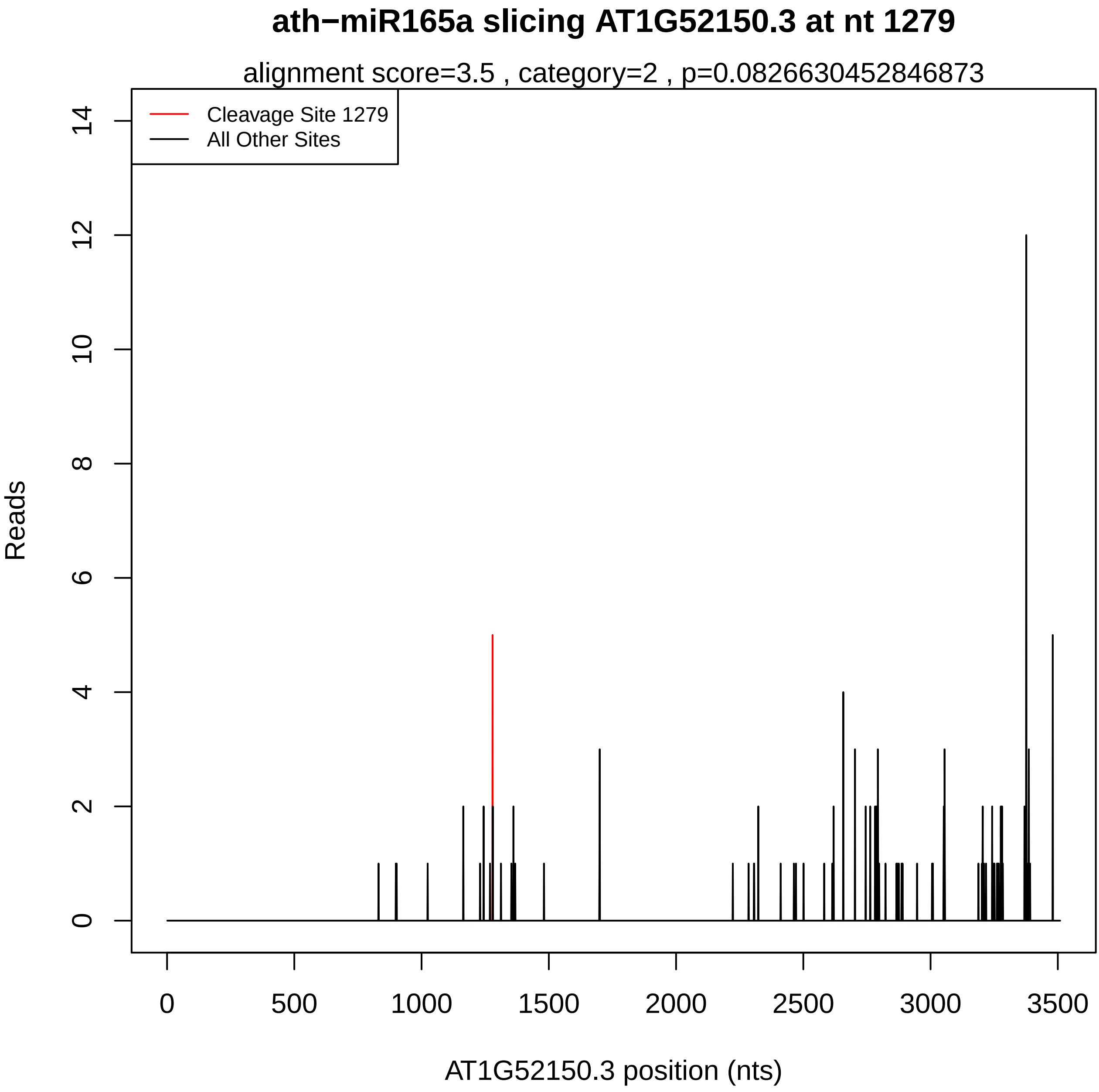
ath−miR165a slicing AT1G52150.3 at nt 1279
alignment score=3.5 , category=2 , p=0.0826630452846873
Cleavage Site 1279
All Other Sites
Reads
0
2
4
6
8
10
12
14
0
500
1000
1500
2000
2500
3000
3500
AT1G52150.3 position (nts)

ath−miR165b slicing AT1G52150.3 at nt 1279

alignment score=3.5 , category=2 , p=0.0826630452846873

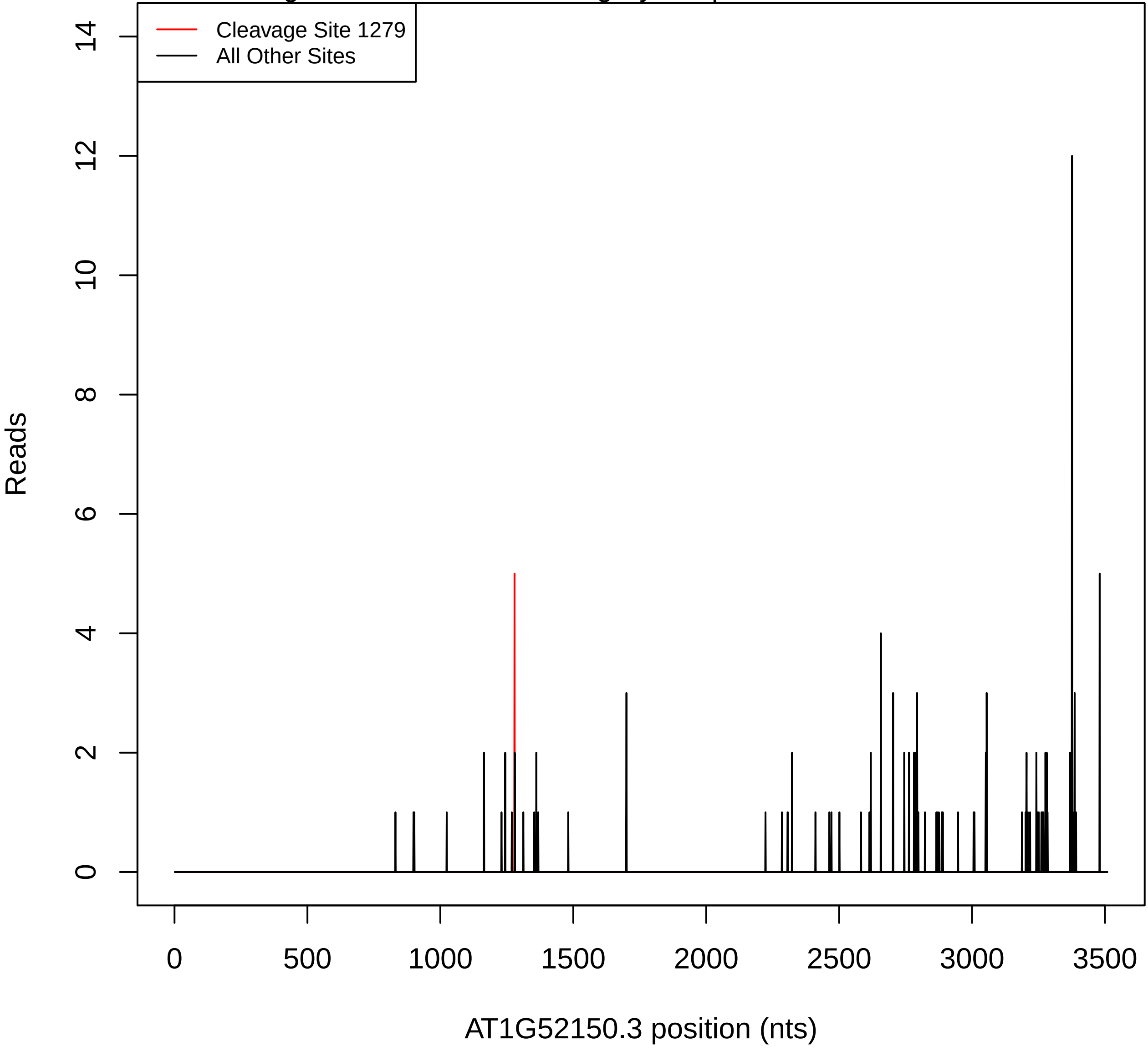

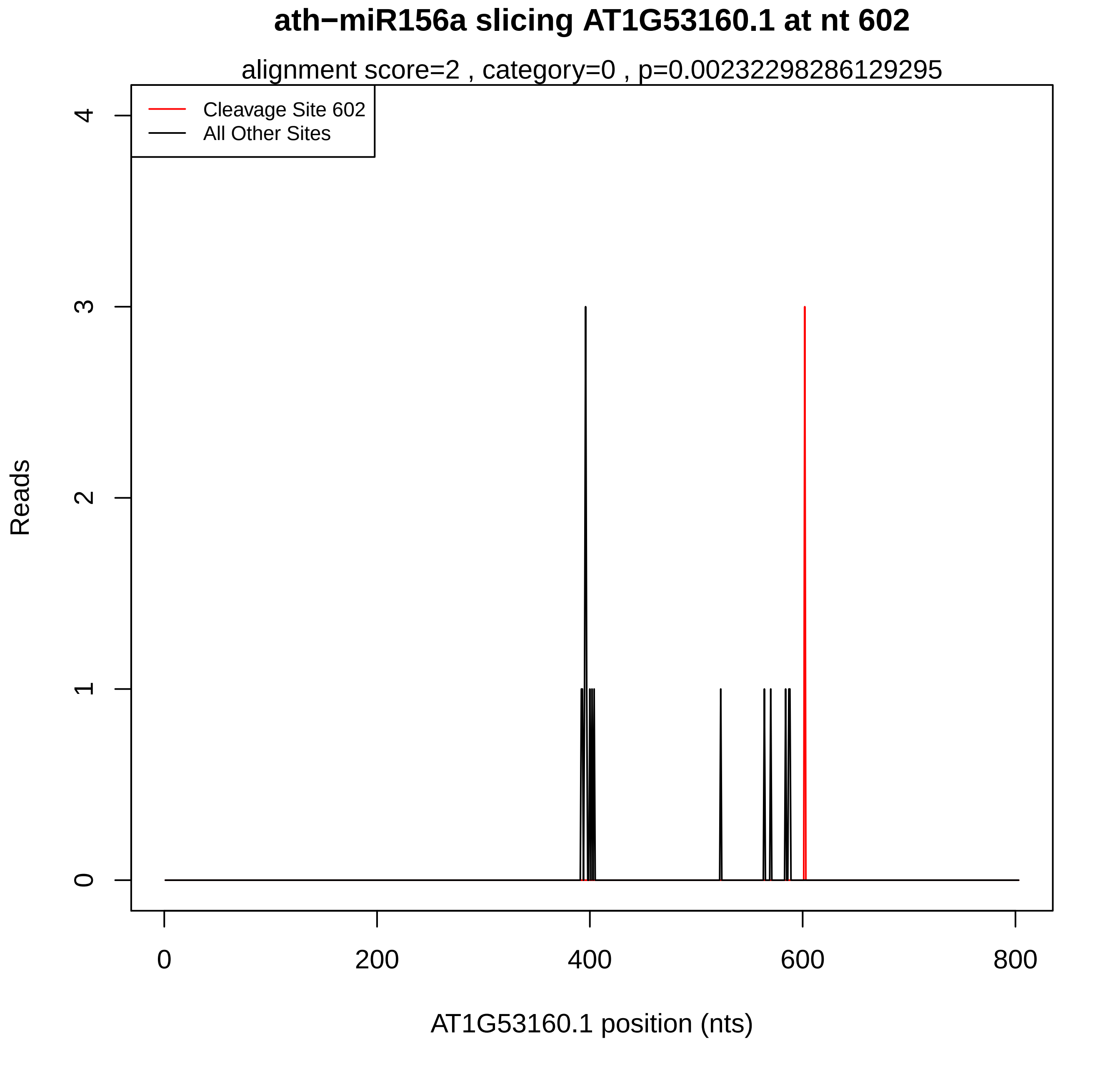
ath−miR156a slicing AT1G53160.1 at nt 602
alignment score=2 , category=0 , p=0.00232298286129295
Cleavage Site 602
All Other Sites
Reads
0
1
2
3
4
0
200
400
600
800
AT1G53160.1 position (nts)

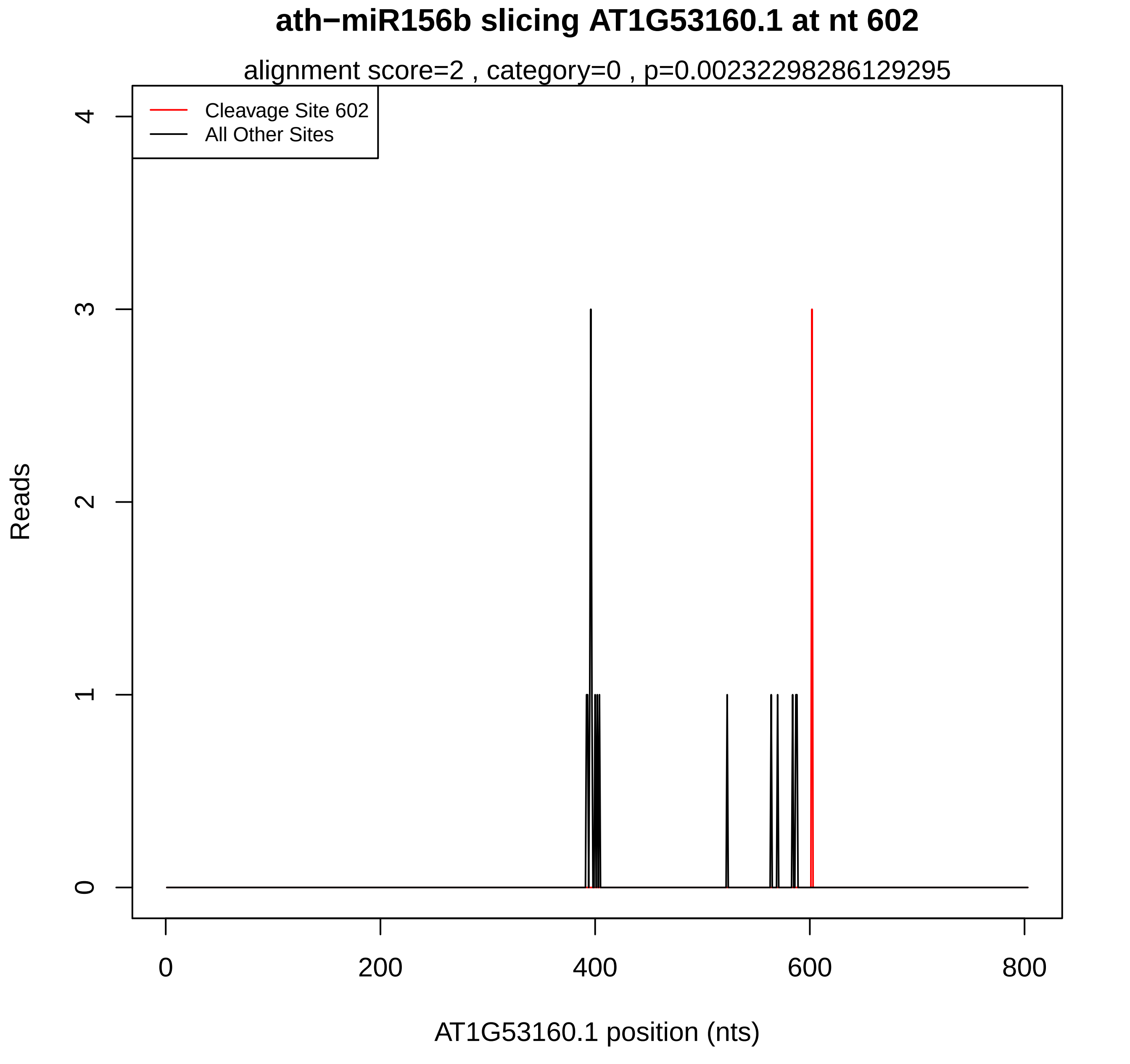

ath−miR156b slicing AT1G53160.1 at nt 602
alignment score=2 , category=0 , p=0.00232298286129295
Cleavage Site 602
All Other Sites
Reads
0
1
2
3
4
0
200
400
600
800
AT1G53160.1 position (nts)

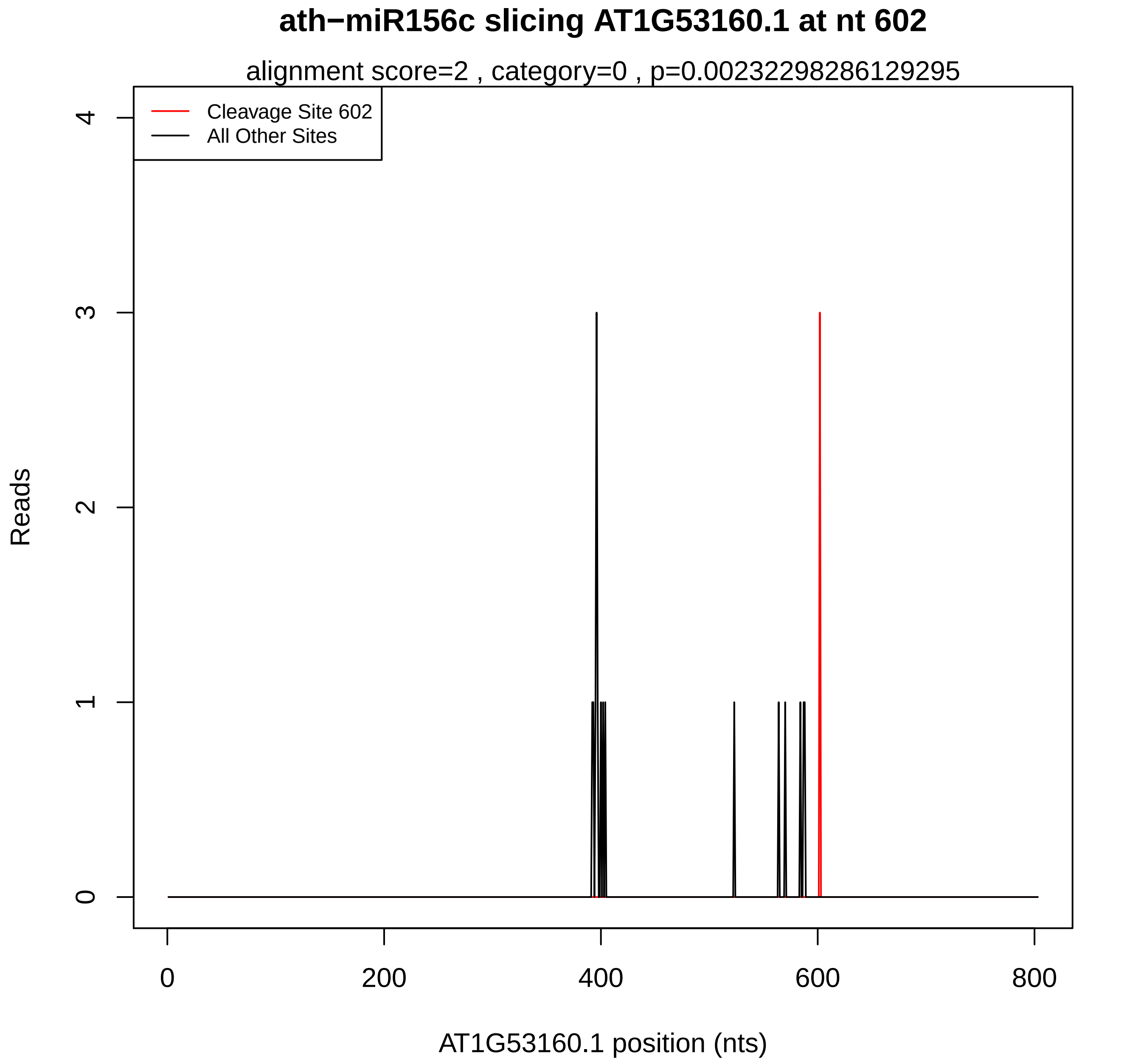
ath−miR156c slicing AT1G53160.1 at nt 602
alignment score=2 , category=0 , p=0.00232298286129295
Cleavage Site 602
All Other Sites
Reads
0
1
2
3
4
0
200
400
600
800
AT1G53160.1 position (nts)

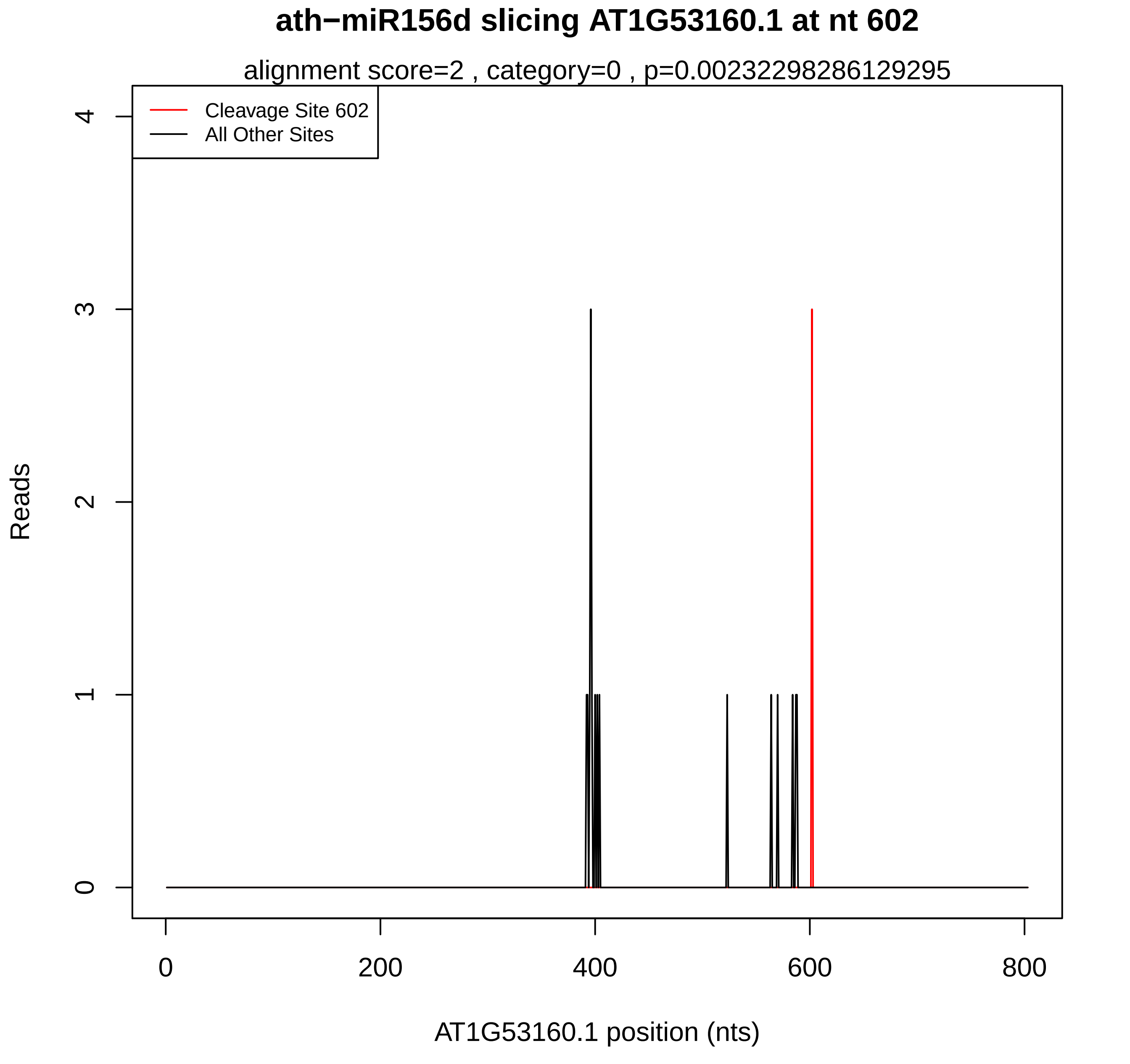
ath−miR156d slicing AT1G53160.1 at nt 602
alignment score=2 , category=0 , p=0.00232298286129295
Cleavage Site 602
All Other Sites
Reads
0
1
2
3
4
0
200
400
600
800
AT1G53160.1 position (nts)

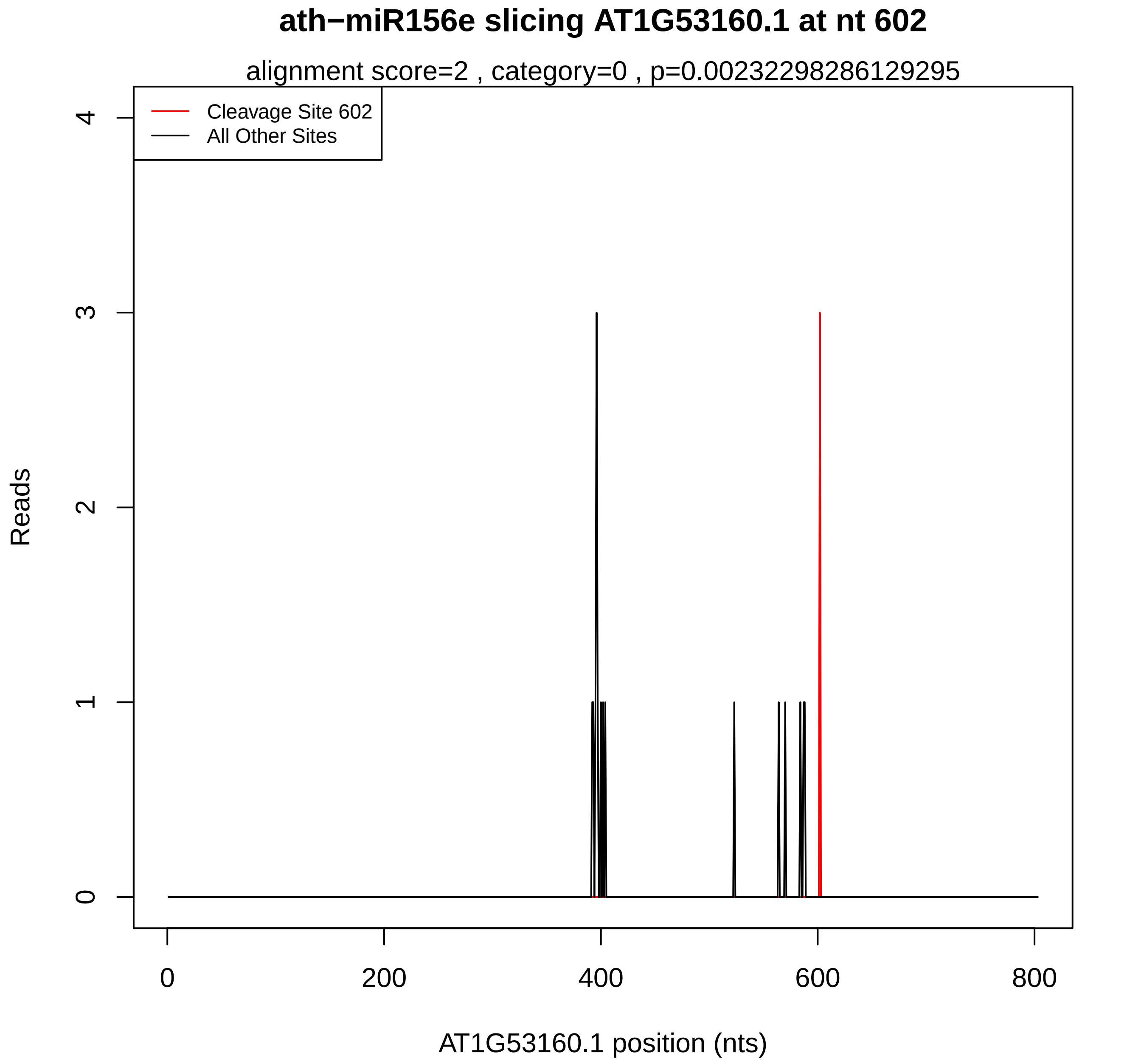

ath−miR156e slicing AT1G53160.1 at nt 602
alignment score=2 , category=0 , p=0.00232298286129295
Cleavage Site 602
All Other Sites
Reads
0
1
2
3
4
0
200
400
600
800
AT1G53160.1 position (nts)

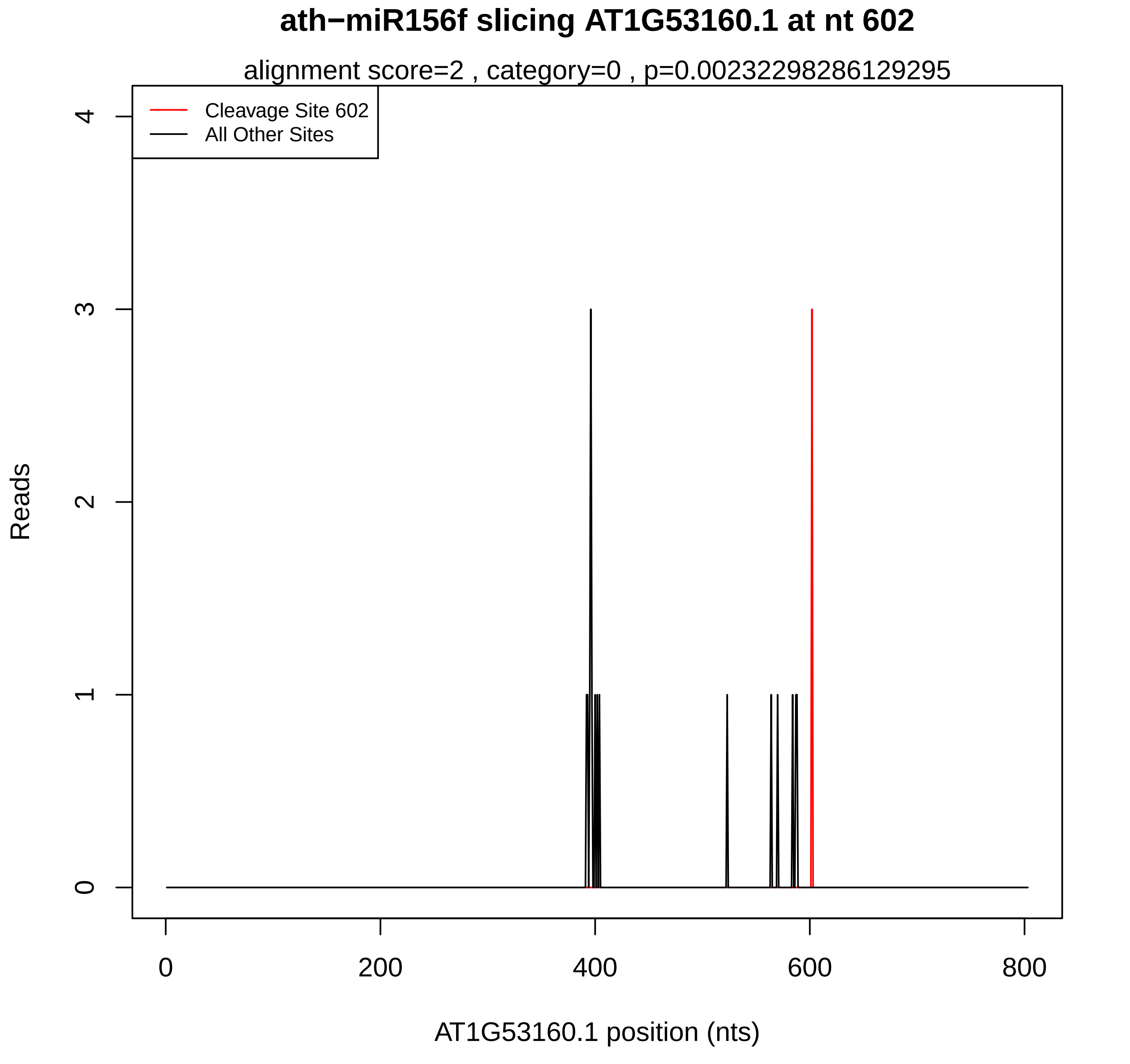

ath−miR156f slicing AT1G53160.1 at nt 602
alignment score=2 , category=0 , p=0.00232298286129295
Cleavage Site 602
All Other Sites
Reads
0
1
2
3
4
0
200
400
600
800
AT1G53160.1 position (nts)

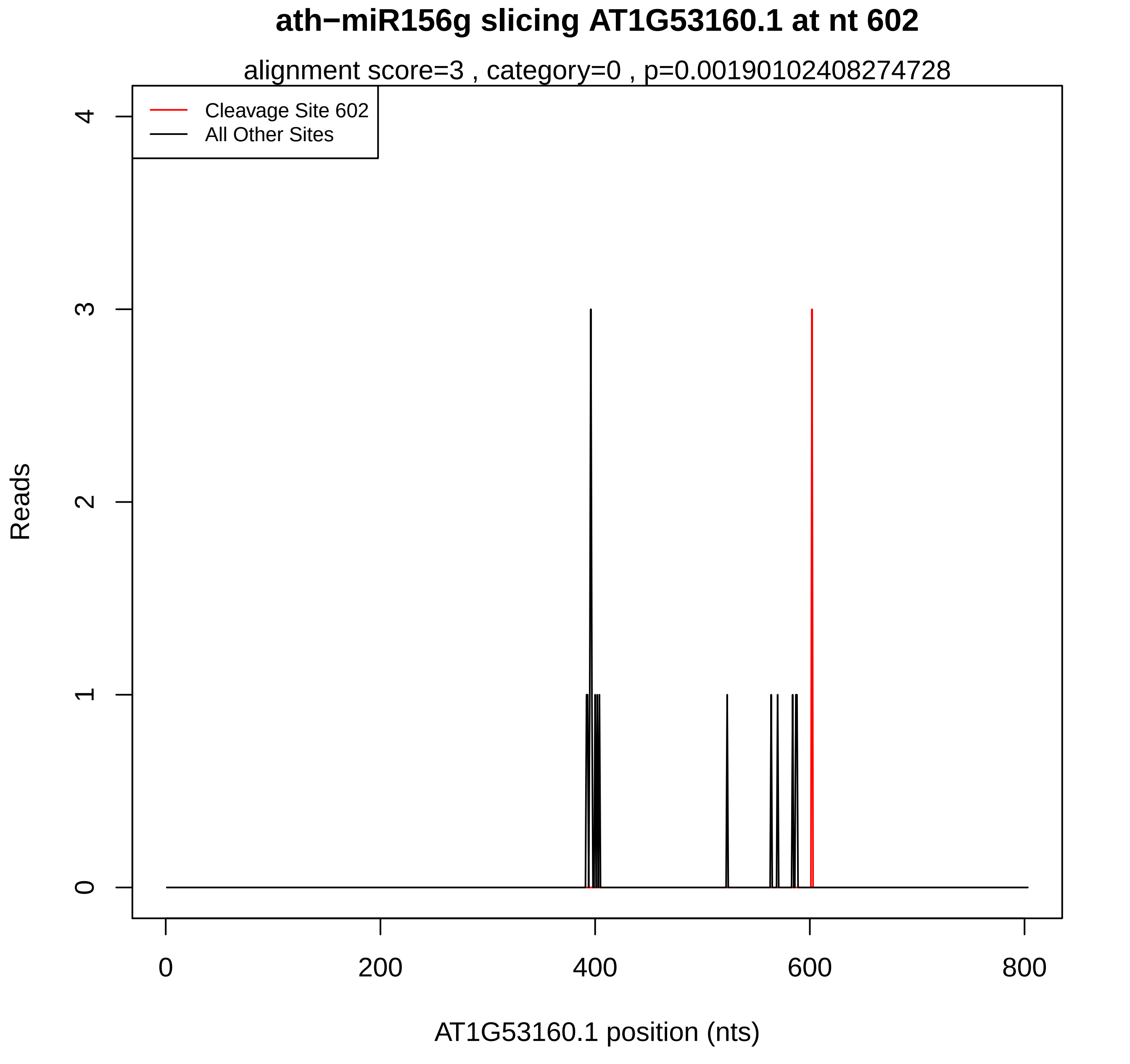
ath−miR156g slicing AT1G53160.1 at nt 602
alignment score=3 , category=0 , p=0.00190102408274728
Cleavage Site 602
All Other Sites
Reads
0
1
2
3
4
0
200
400
600
800
AT1G53160.1 position (nts)

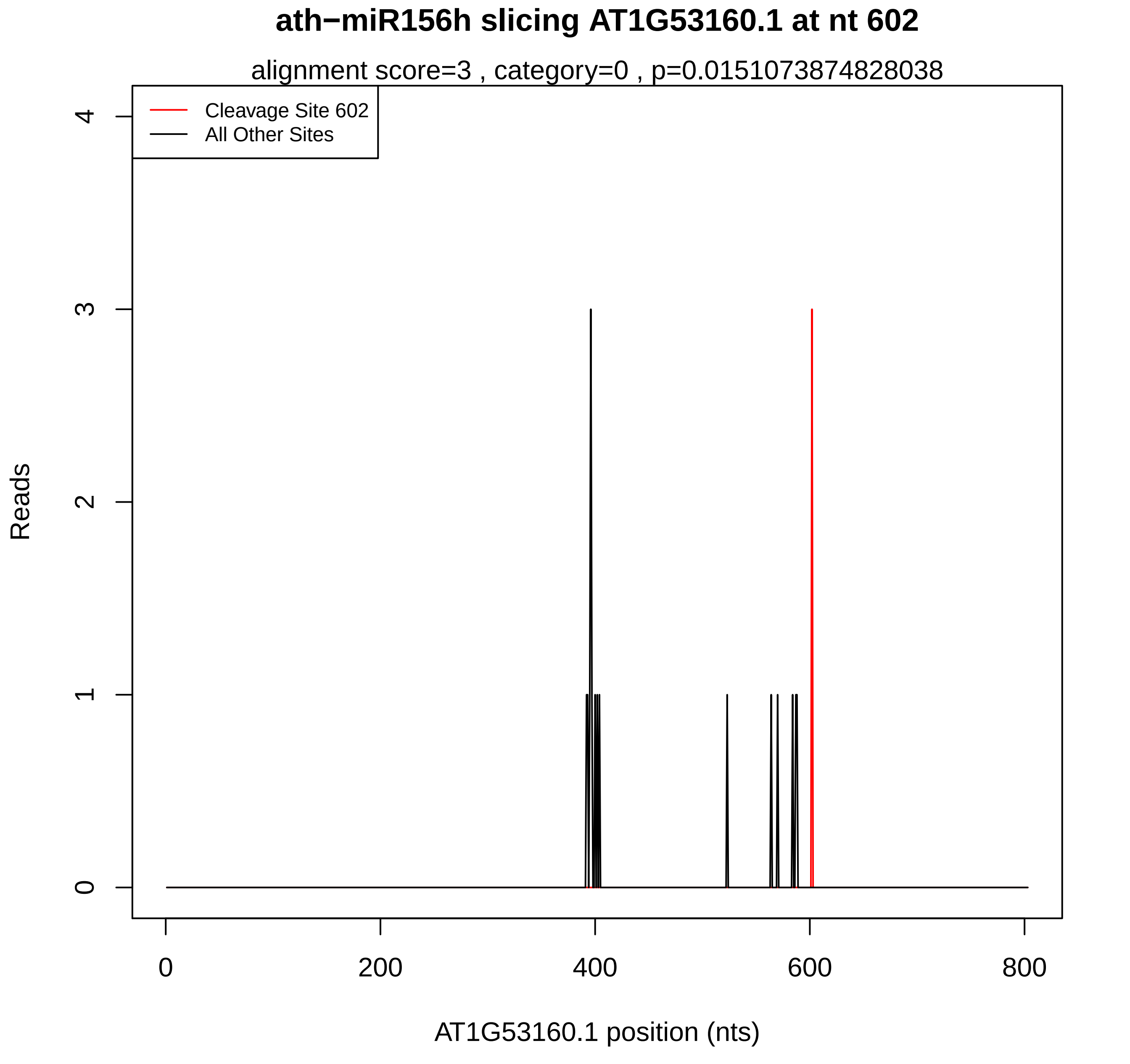

ath−miR156h slicing AT1G53160.1 at nt 602
alignment score=3 , category=0 , p=0.0151073874828038
Cleavage Site 602
All Other Sites
Reads
0
1
2
3
4
0
200
400
600
800
AT1G53160.1 position (nts)

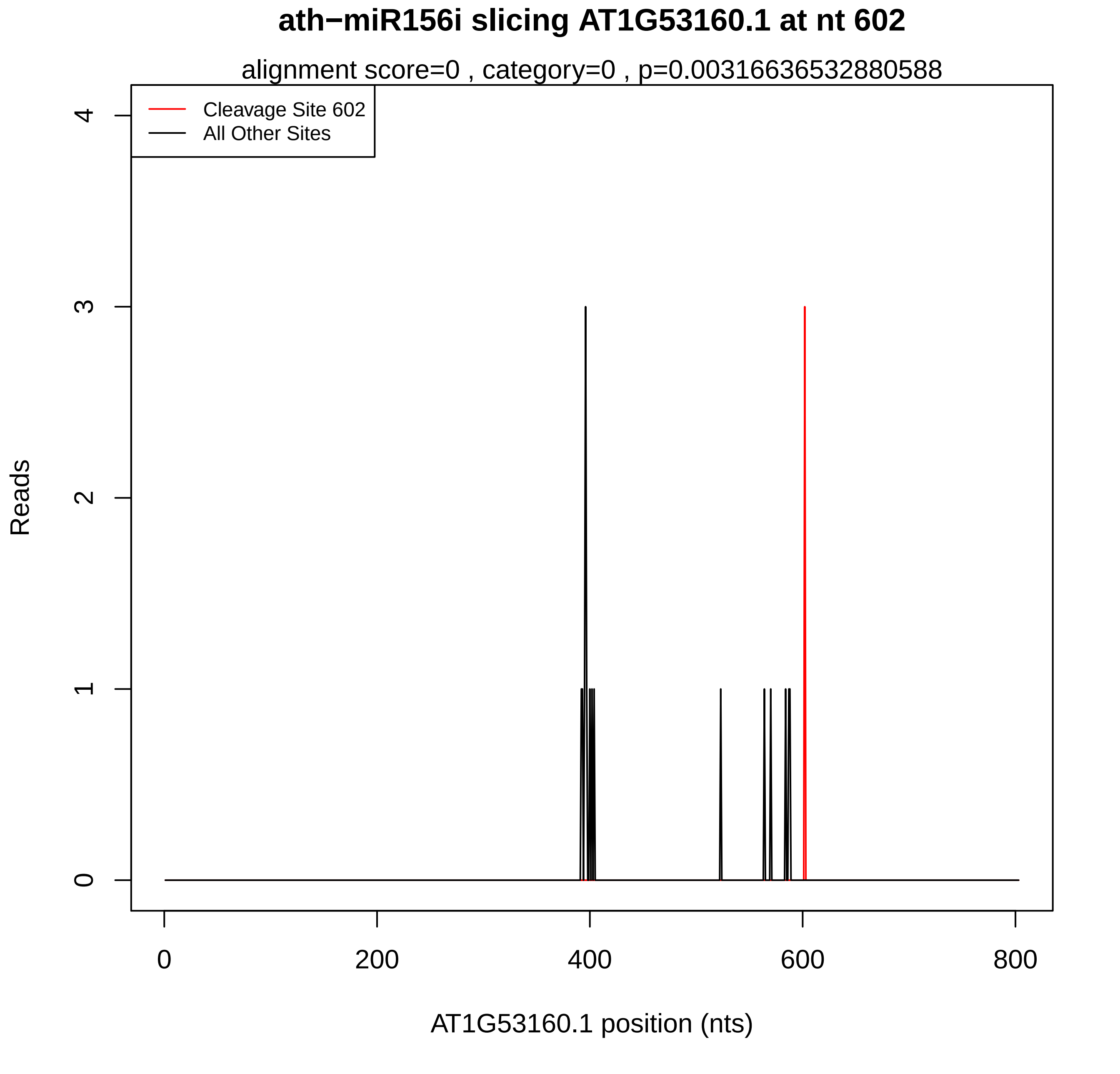

ath−miR156i slicing AT1G53160.1 at nt 602
alignment score=0 , category=0 , p=0.00316636532880588
Cleavage Site 602
All Other Sites
Reads
0
1
2
3
4
0
200
400
600
800
AT1G53160.1 position (nts)

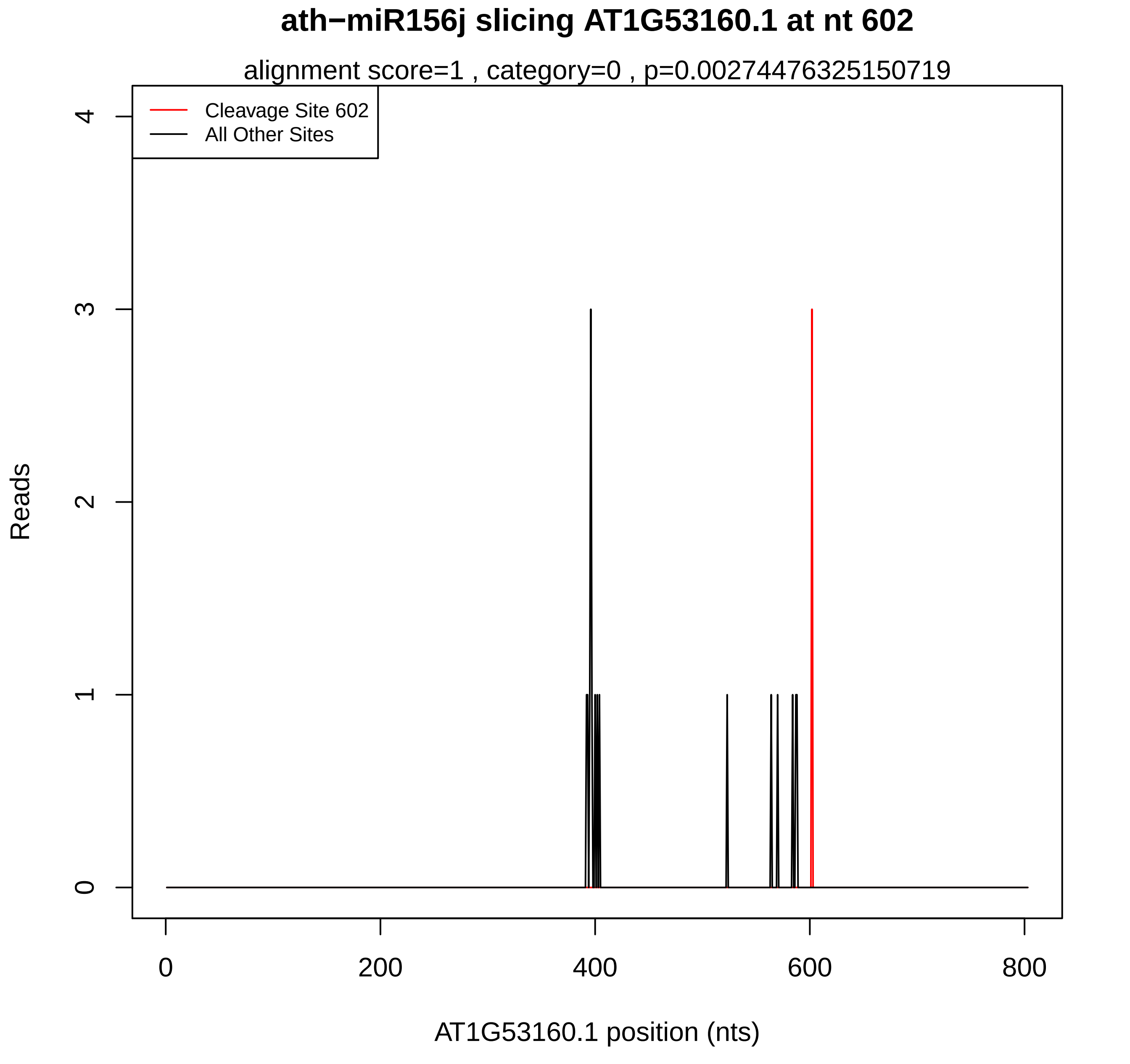
ath−miR156j slicing AT1G53160.1 at nt 602
alignment score=1 , category=0 , p=0.00274476325150719
Cleavage Site 602
All Other Sites
Reads
0
1
2
3
4
0
200
400
600
800
AT1G53160.1 position (nts)

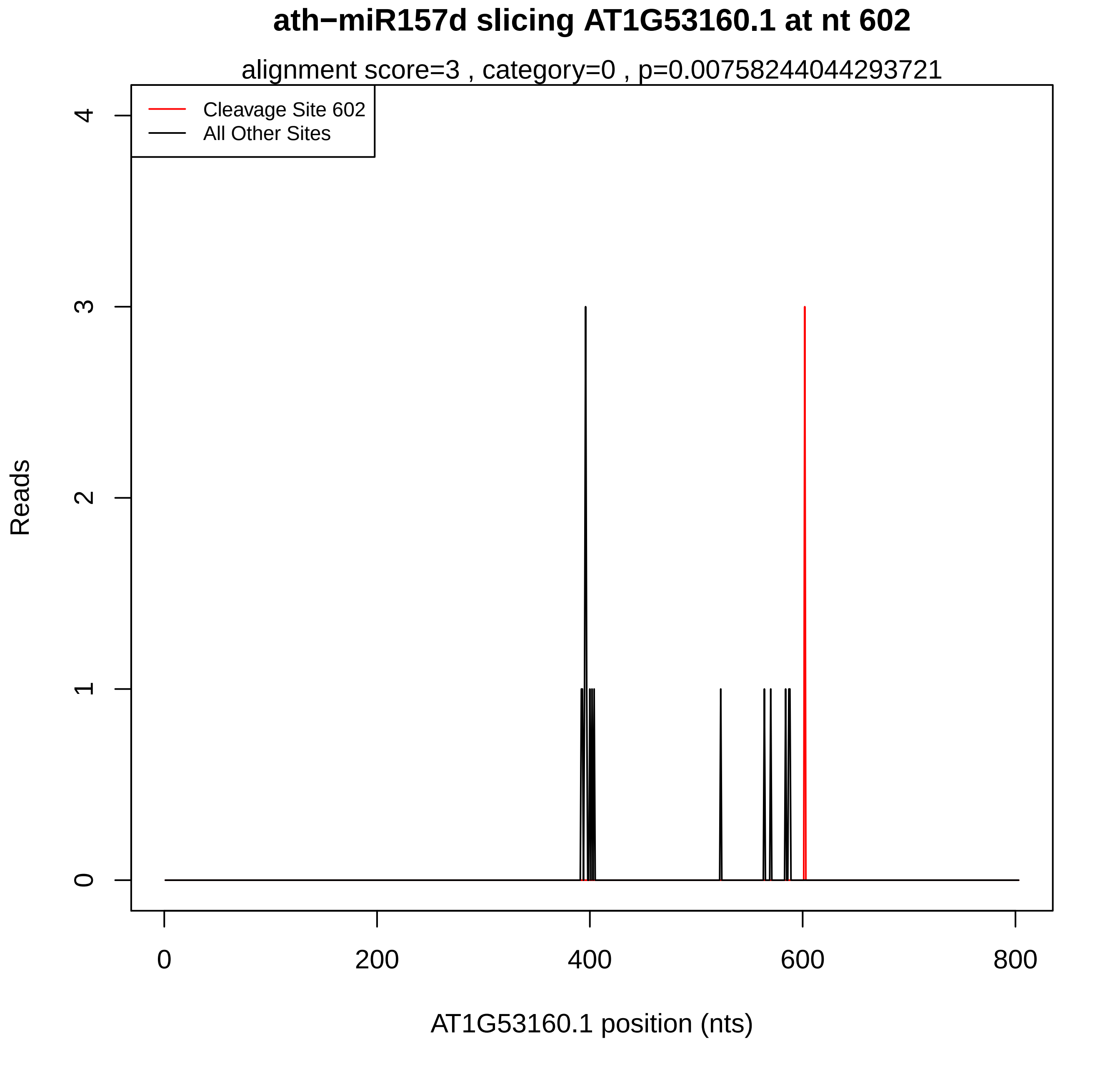
ath−miR157d slicing AT1G53160.1 at nt 602
alignment score=3 , category=0 , p=0.00758244044293721
Cleavage Site 602
All Other Sites
Reads
0
1
2
3
4
0
200
400
600
800
AT1G53160.1 position (nts)

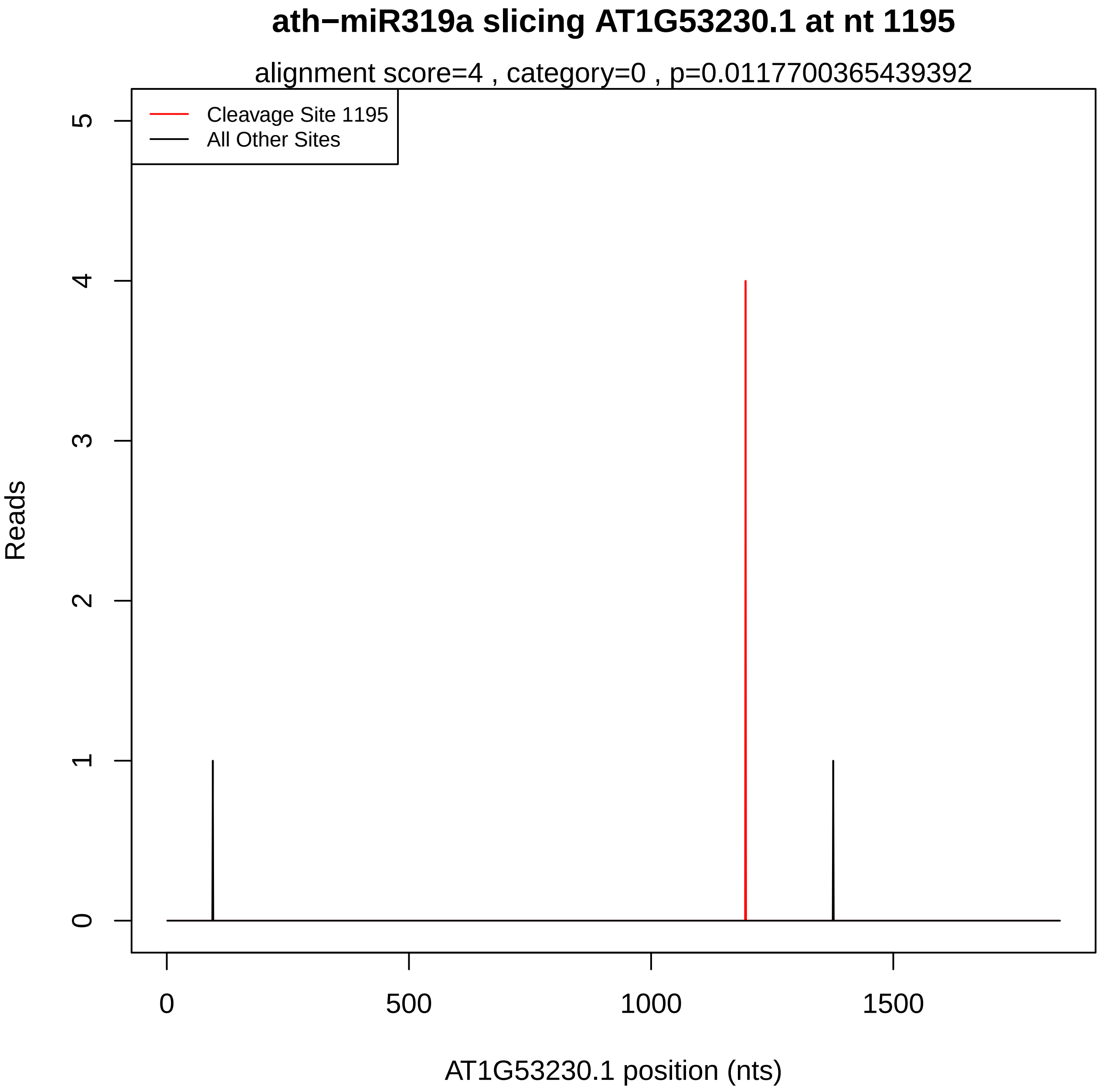
ath−miR319a slicing AT1G53230.1 at nt 1195
alignment score=4 , category=0 , p=0.0117700365439392
Cleavage Site 1195
All Other Sites
Reads
0
1
2
3
4
5
0
500
1000
1500
AT1G53230.1 position (nts)

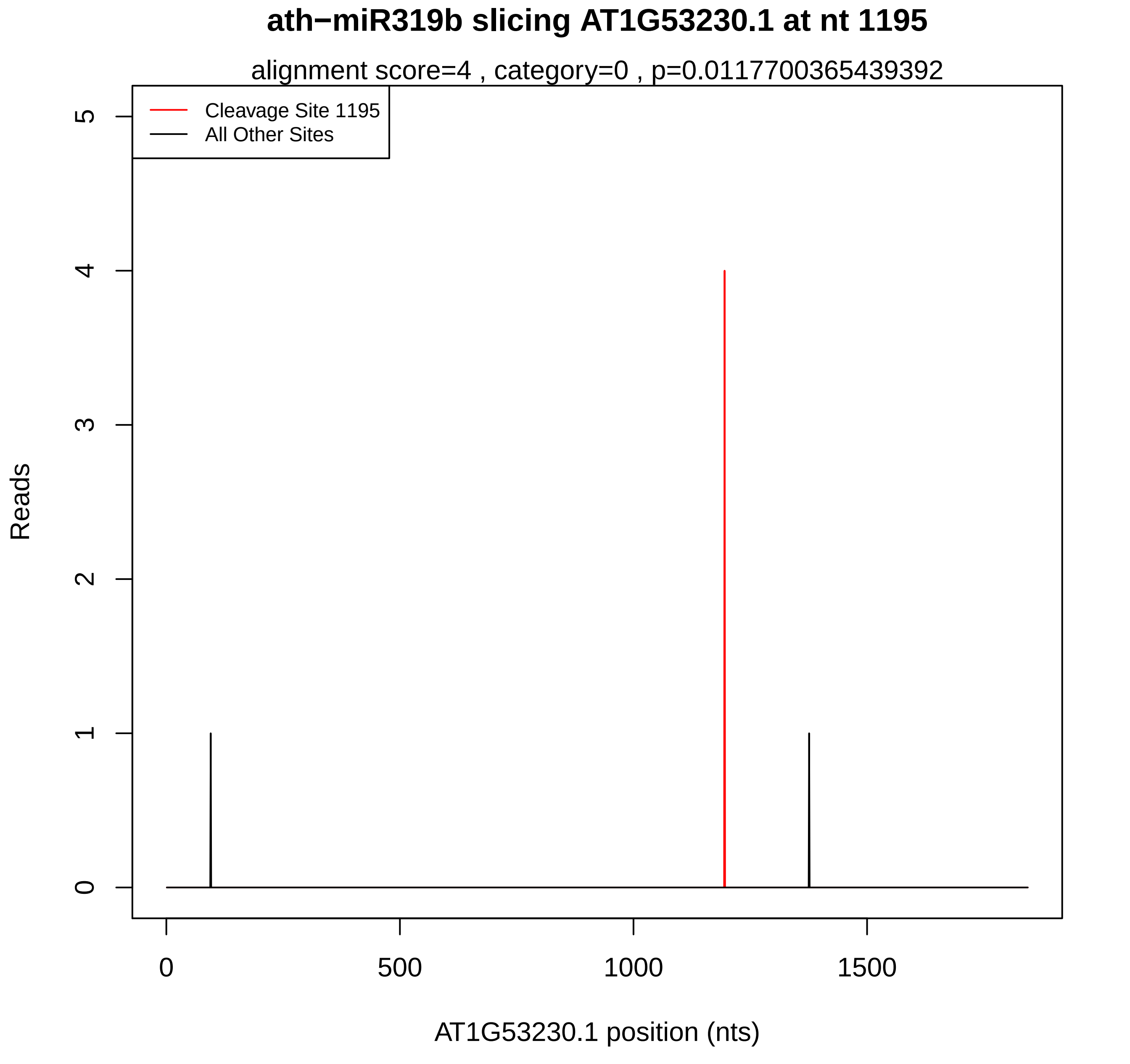
ath−miR319b slicing AT1G53230.1 at nt 1195
alignment score=4 , category=0 , p=0.0117700365439392
Cleavage Site 1195
All Other Sites
Reads
0
1
2
3
4
5
0
500
1000
1500
AT1G53230.1 position (nts)

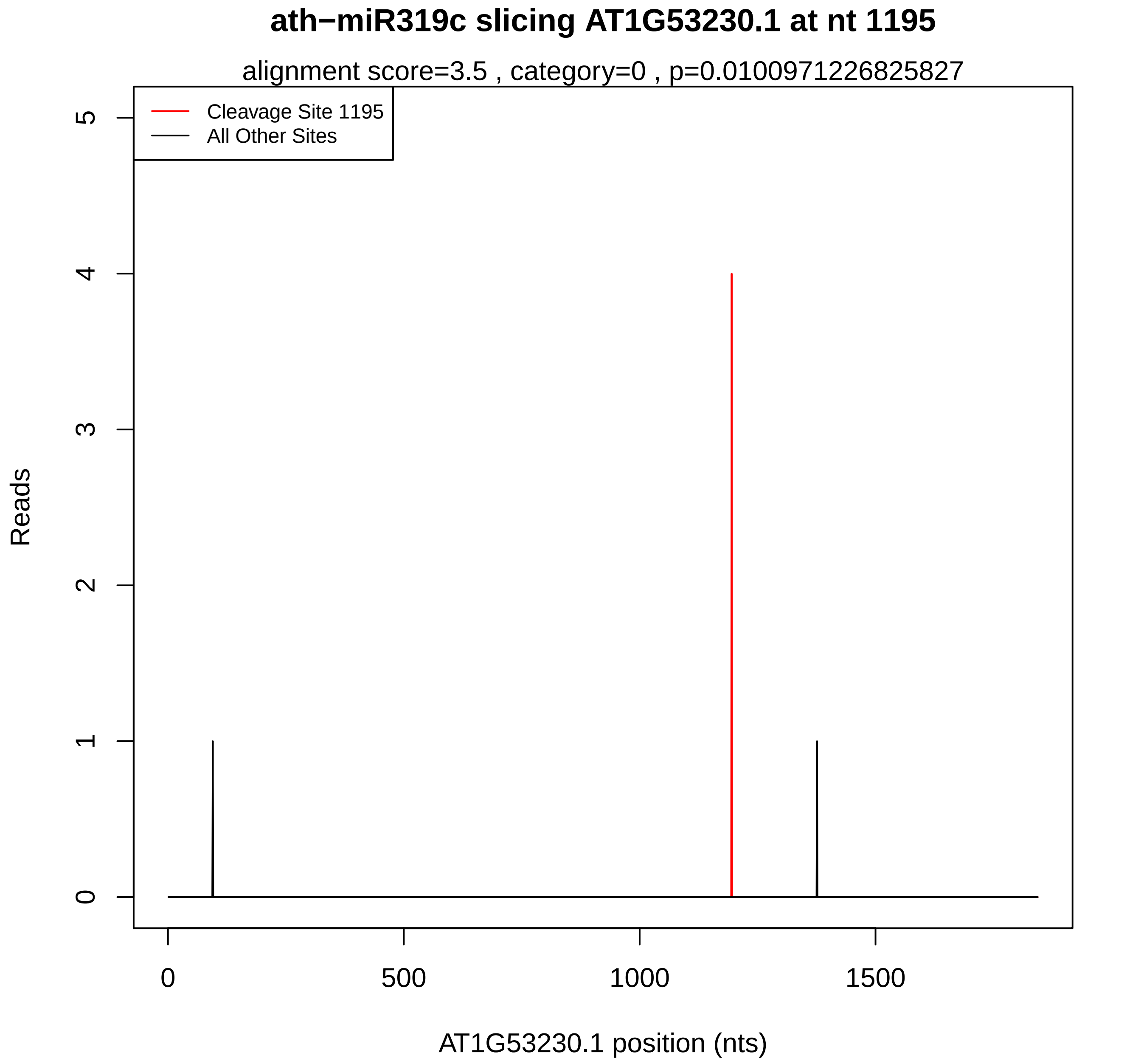

ath−miR319c slicing AT1G53230.1 at nt 1195
alignment score=3.5 , category=0 , p=0.0100971226825827
Cleavage Site 1195
All Other Sites
Reads
0
1
2
3
4
5
0
500
1000
1500
AT1G53230.1 position (nts)

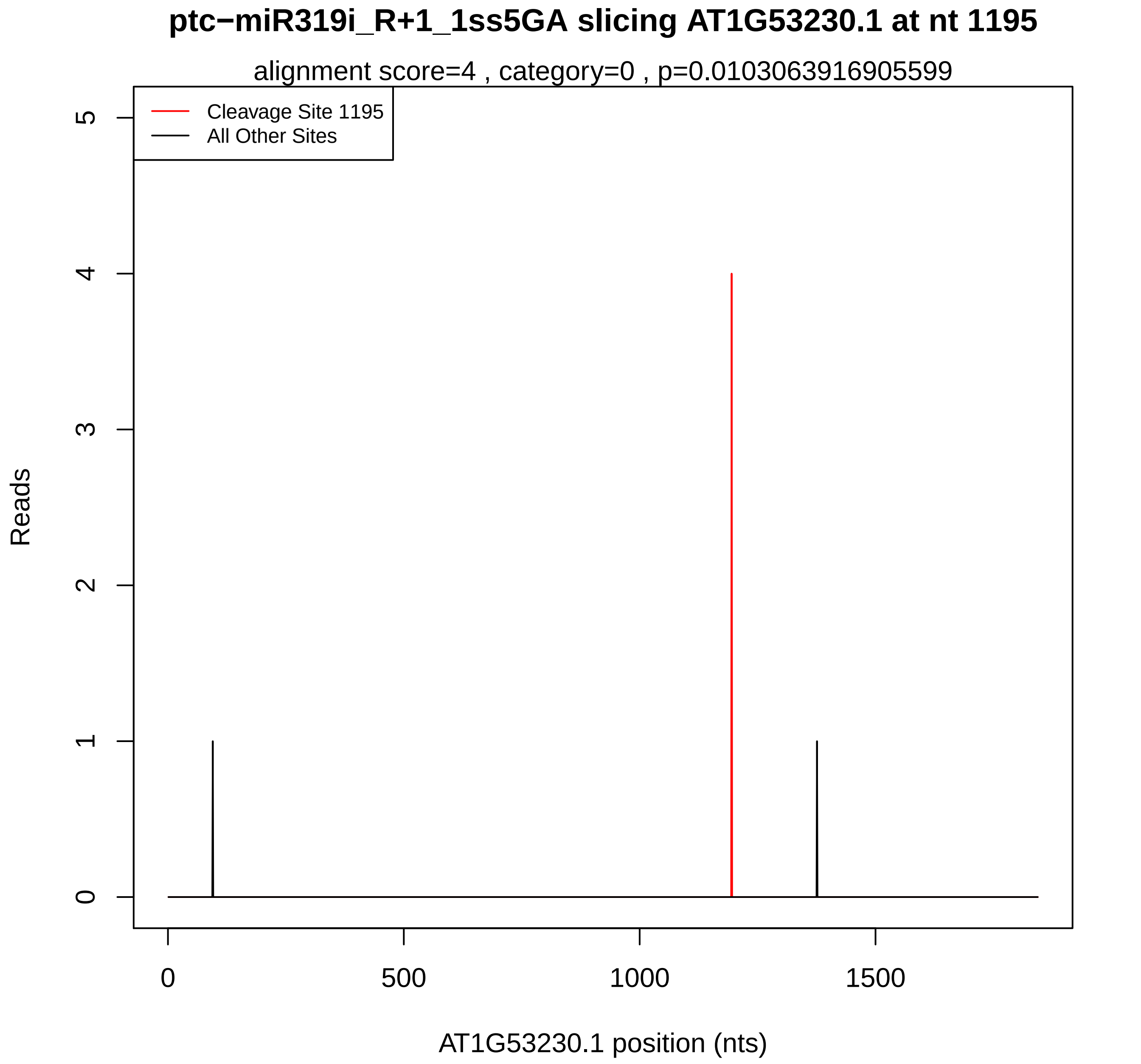

ptc−miR319i_R+1_1ss5GA slicing AT1G53230.1 at nt 1195
alignment score=4 , category=0 , p=0.0103063916905599
Cleavage Site 1195
All Other Sites
Reads
0
1
2
3
4
5
0
500
1000
1500
AT1G53230.1 position (nts)

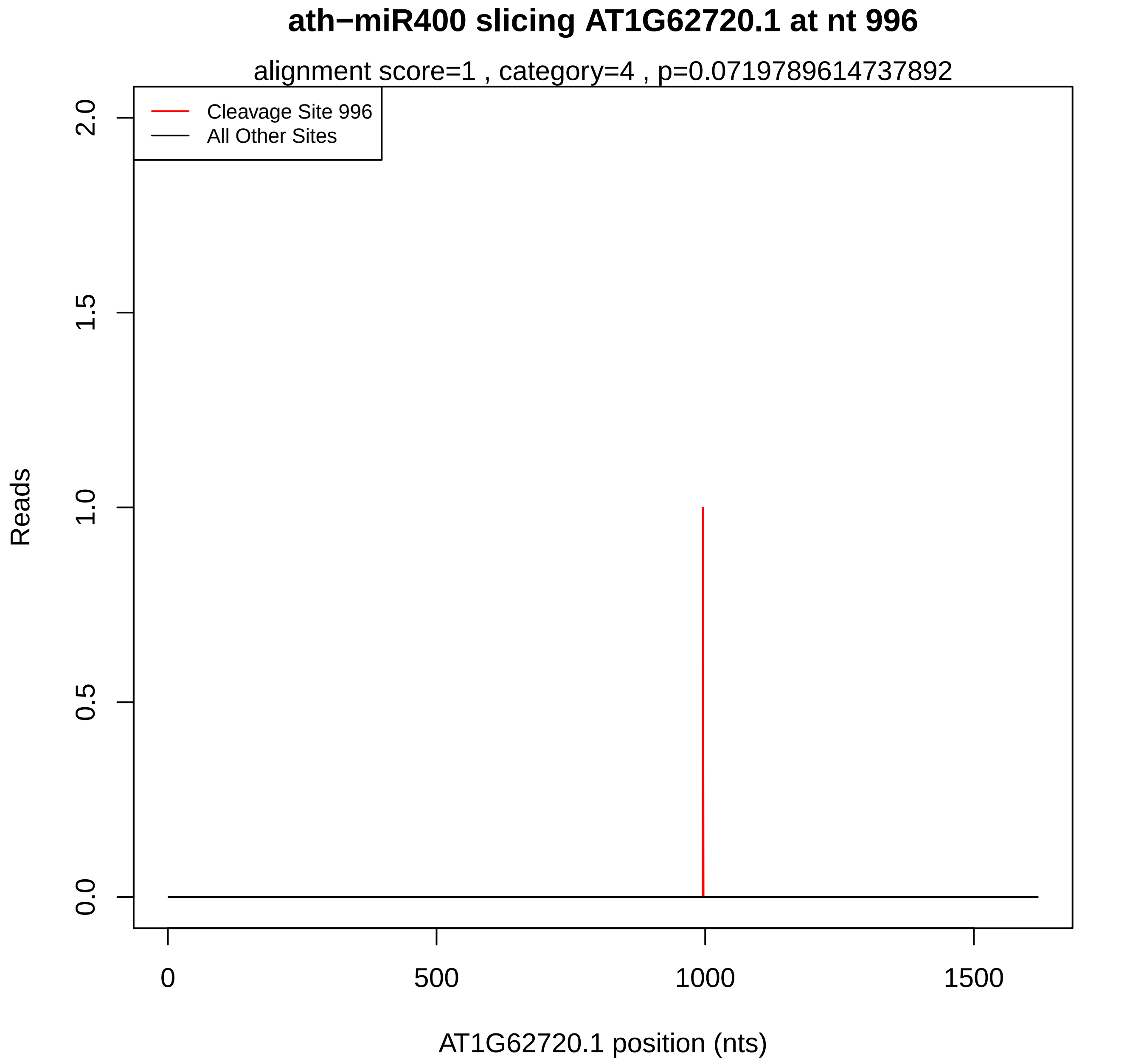
ath−miR400 slicing AT1G62720.1 at nt 996
alignment score=1 , category=4 , p=0.0719789614737892
Cleavage Site 996
All Other Sites
Reads
0.0
0.5
1.0
1.5
2.0
0
500
1000
1500
AT1G62720.1 position (nts)

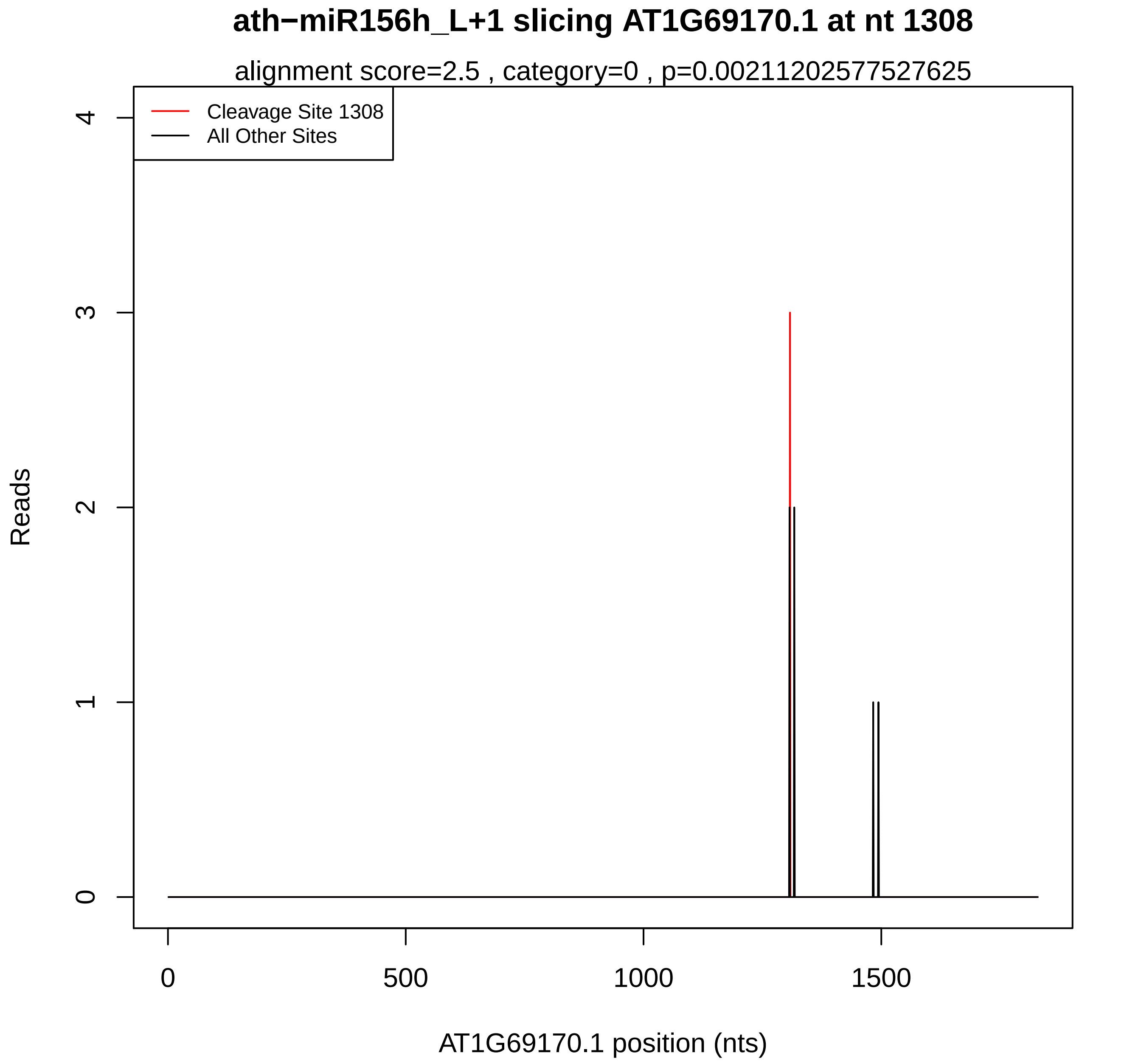

ath−miR156h_L+1 slicing AT1G69170.1 at nt 1308
alignment score=2.5 , category=0 , p=0.00211202577527625
Cleavage Site 1308
All Other Sites
Reads
0
1
2
3
4
0
500
1000
1500
AT1G69170.1 position (nts)

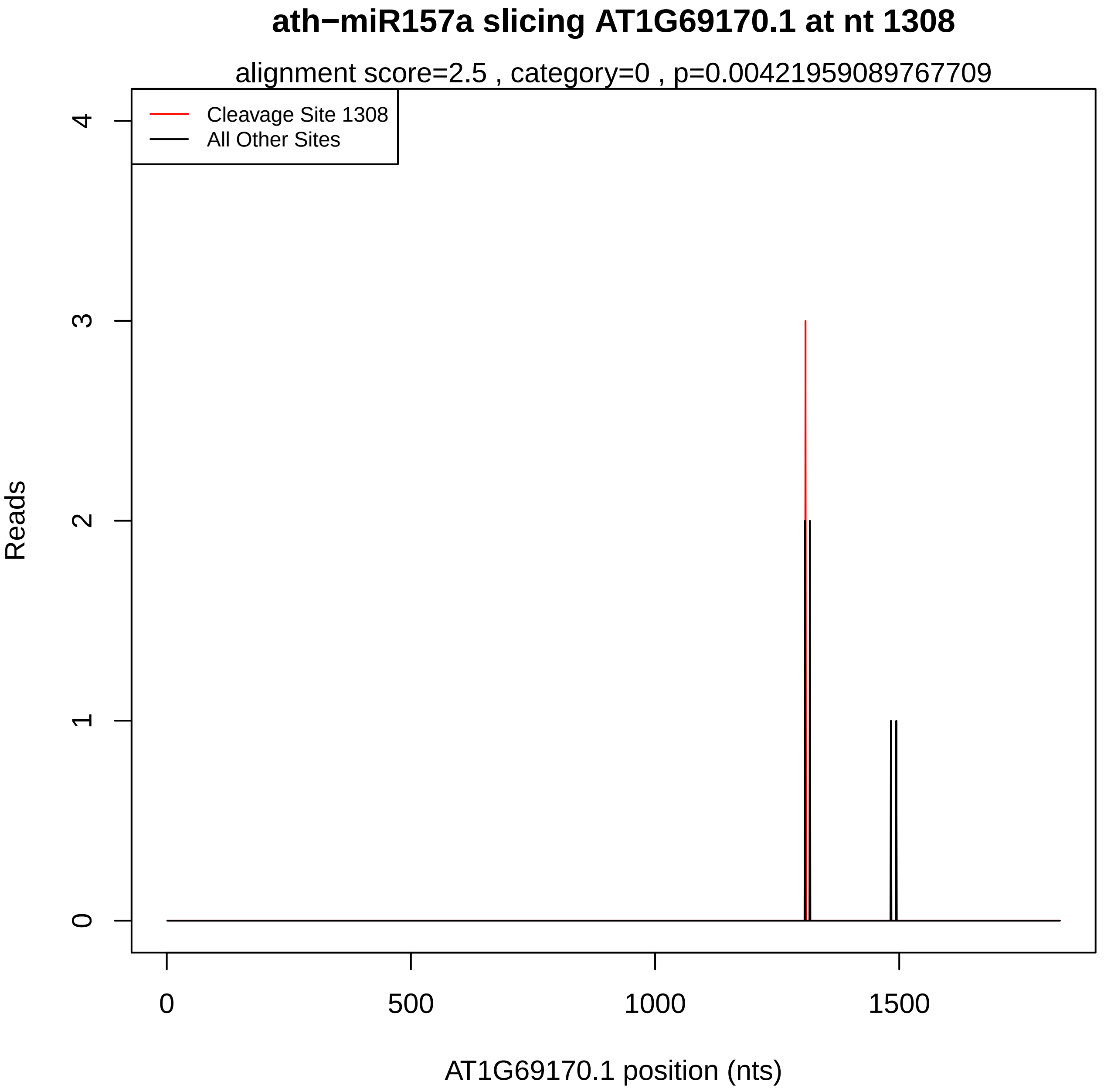
ath−miR157a slicing AT1G69170.1 at nt 1308
alignment score=2.5 , category=0 , p=0.00421959089767709
Cleavage Site 1308
All Other Sites
Reads
0
1
2
3
4
0
500
1000
1500
AT1G69170.1 position (nts)

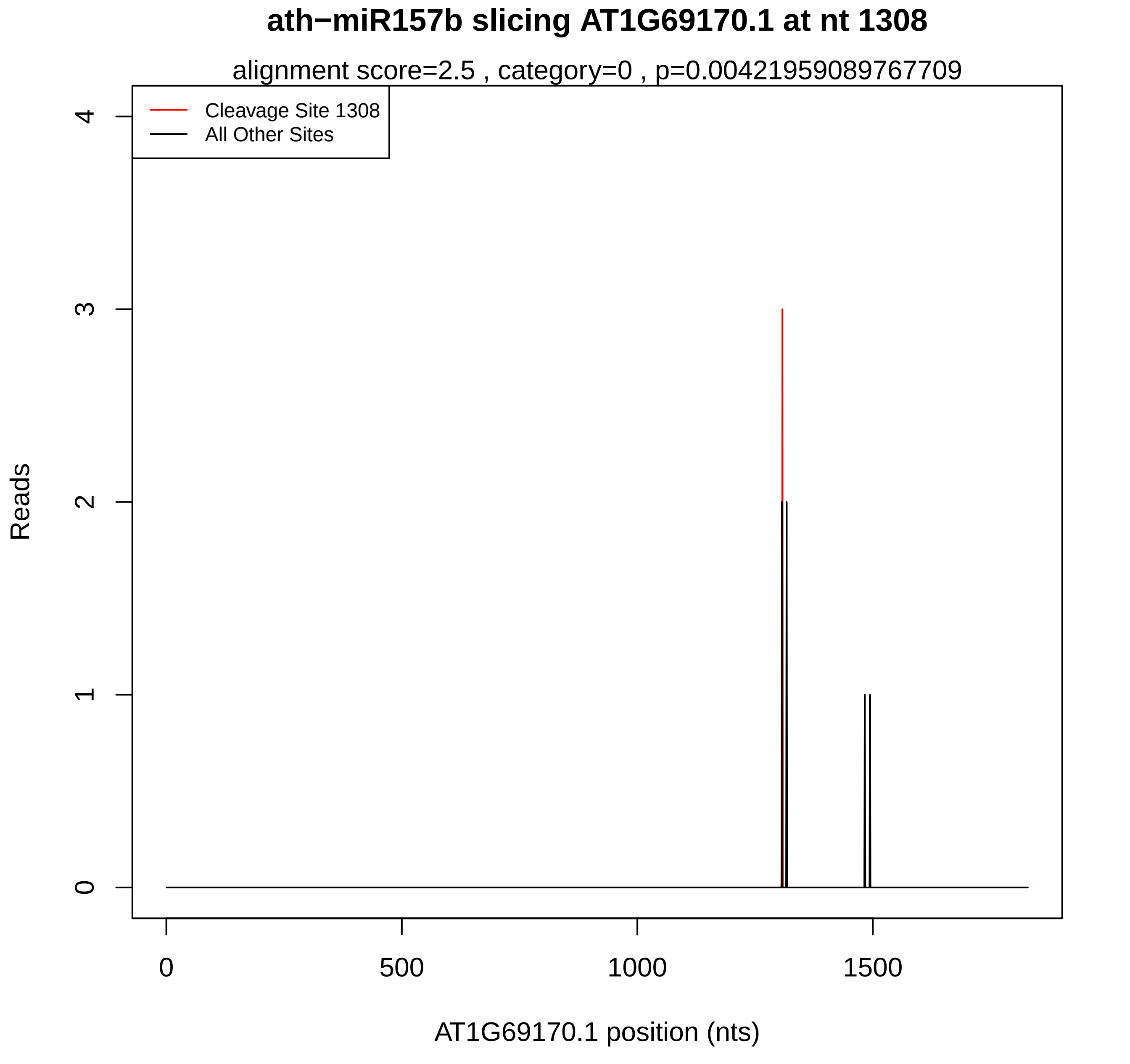
ath−miR157b slicing AT1G69170.1 at nt 1308
alignment score=2.5 , category=0 , p=0.00421959089767709
Cleavage Site 1308
All Other Sites
Reads
0
1
2
3
4
0
500
1000
1500
AT1G69170.1 position (nts)

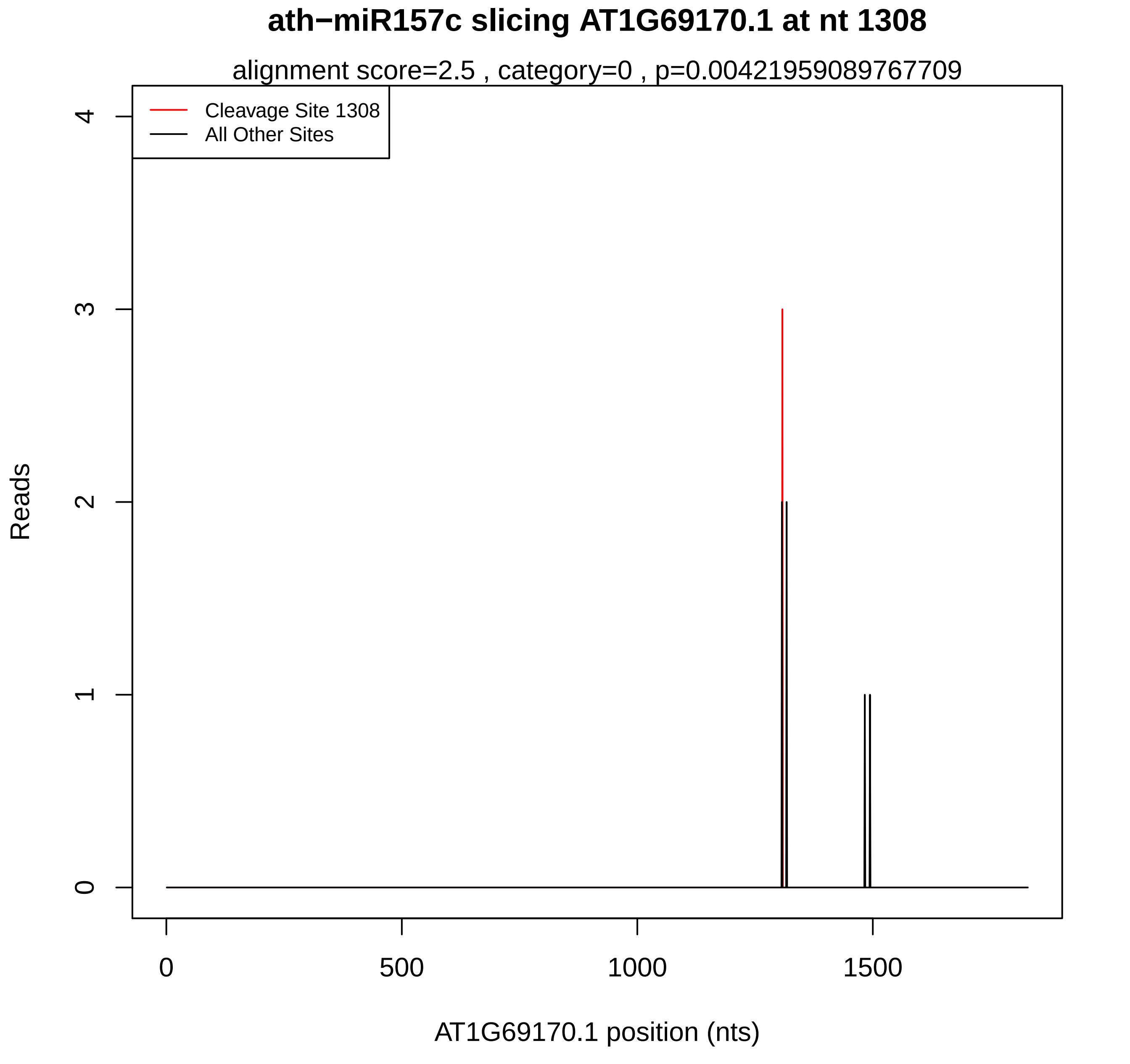

ath−miR157c slicing AT1G69170.1 at nt 1308
alignment score=2.5 , category=0 , p=0.00421959089767709
Cleavage Site 1308
All Other Sites
Reads
0
1
2
3
4
0
500
1000
1500
AT1G69170.1 position (nts)

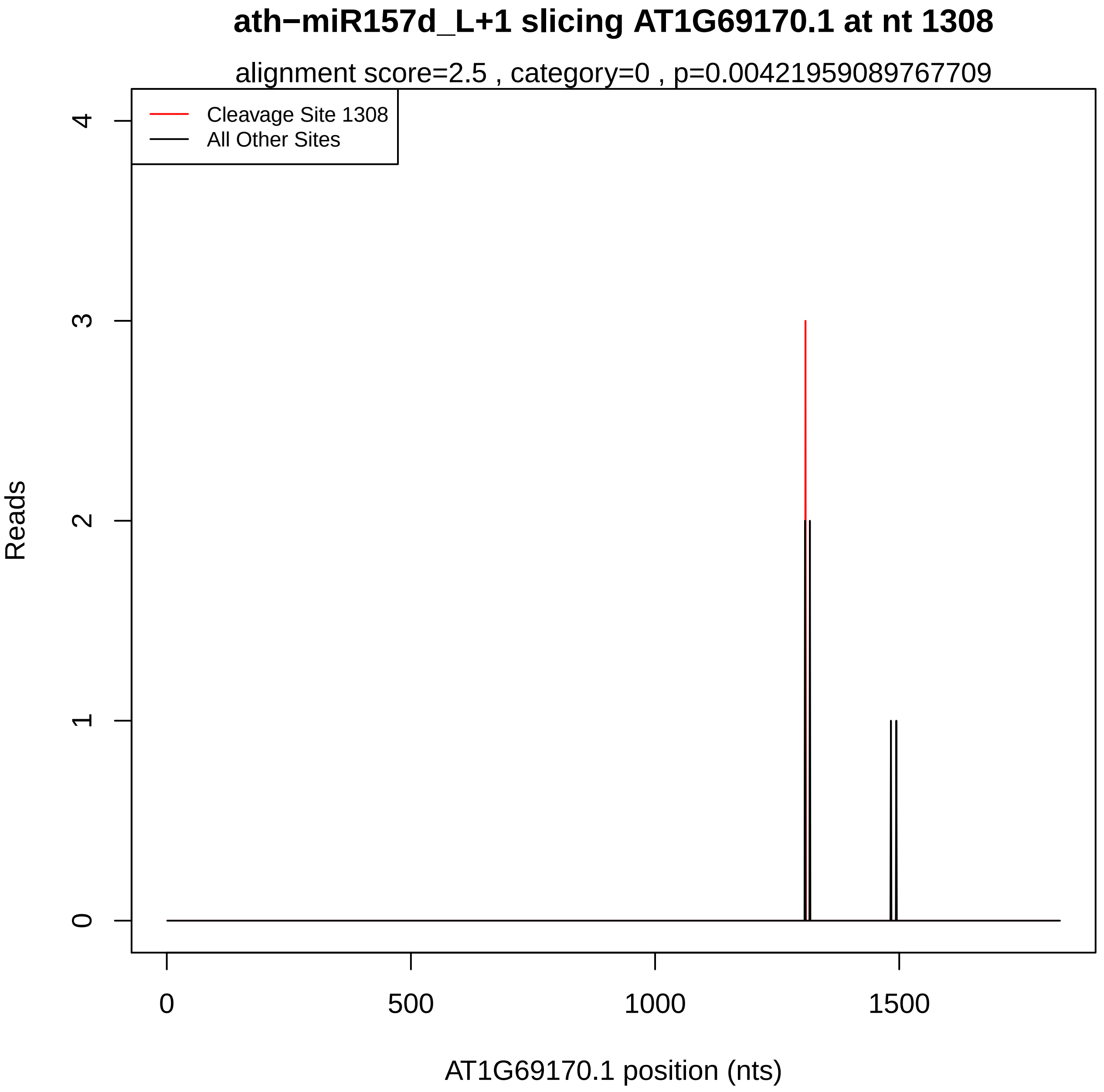

ath−miR157d_L+1 slicing AT1G69170.1 at nt 1308
alignment score=2.5 , category=0 , p=0.00421959089767709
Cleavage Site 1308
All Other Sites
Reads
0
1
2
3
4
0
500
1000
1500
AT1G69170.1 position (nts)

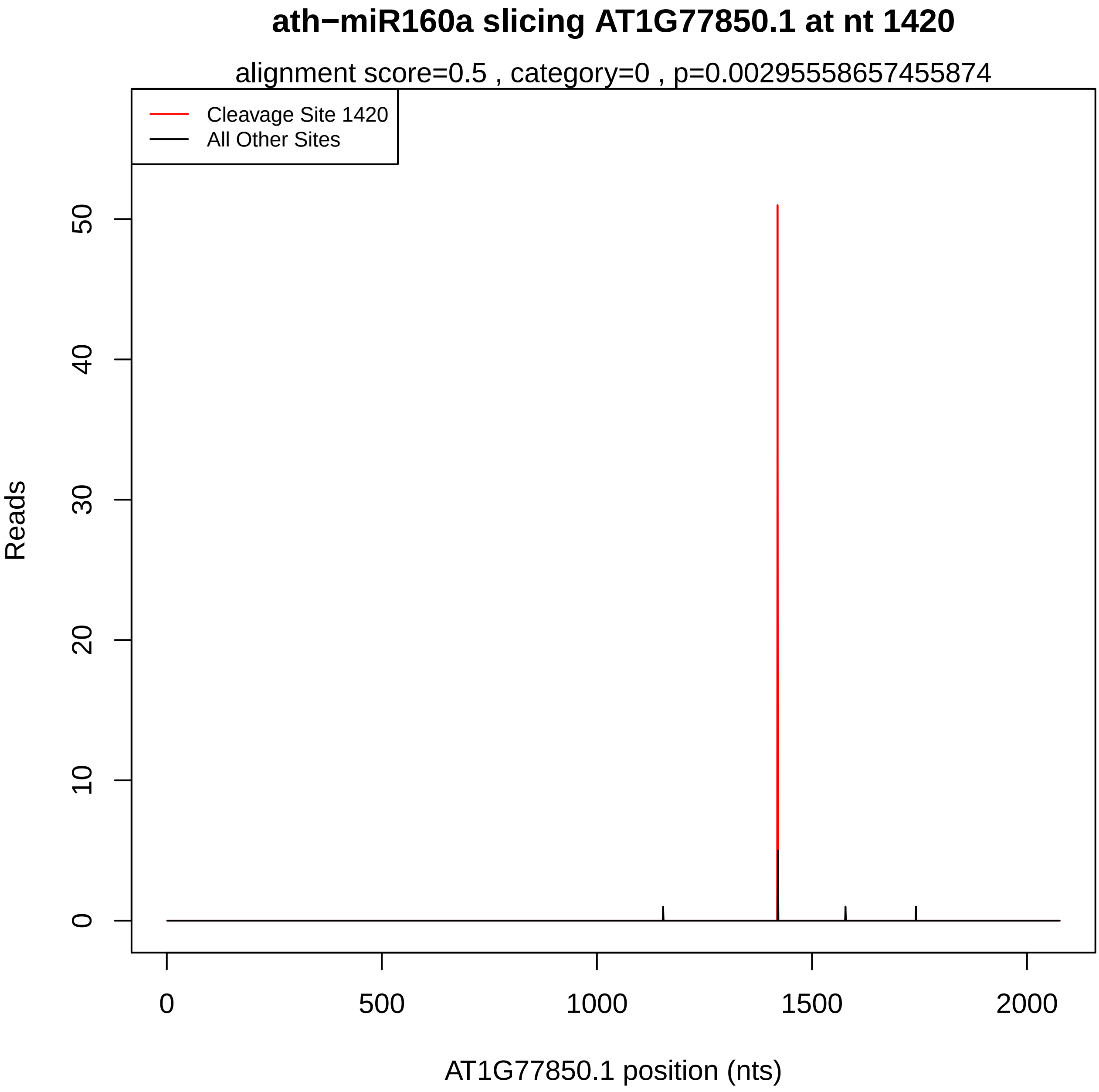

ath−miR160a slicing AT1G77850.1 at nt 1420
alignment score=0.5 , category=0 , p=0.00295558657455874
Cleavage Site 1420
All Other Sites
Reads
0
10
20
30
40
50
0
500
1000
1500
2000
AT1G77850.1 position (nts)

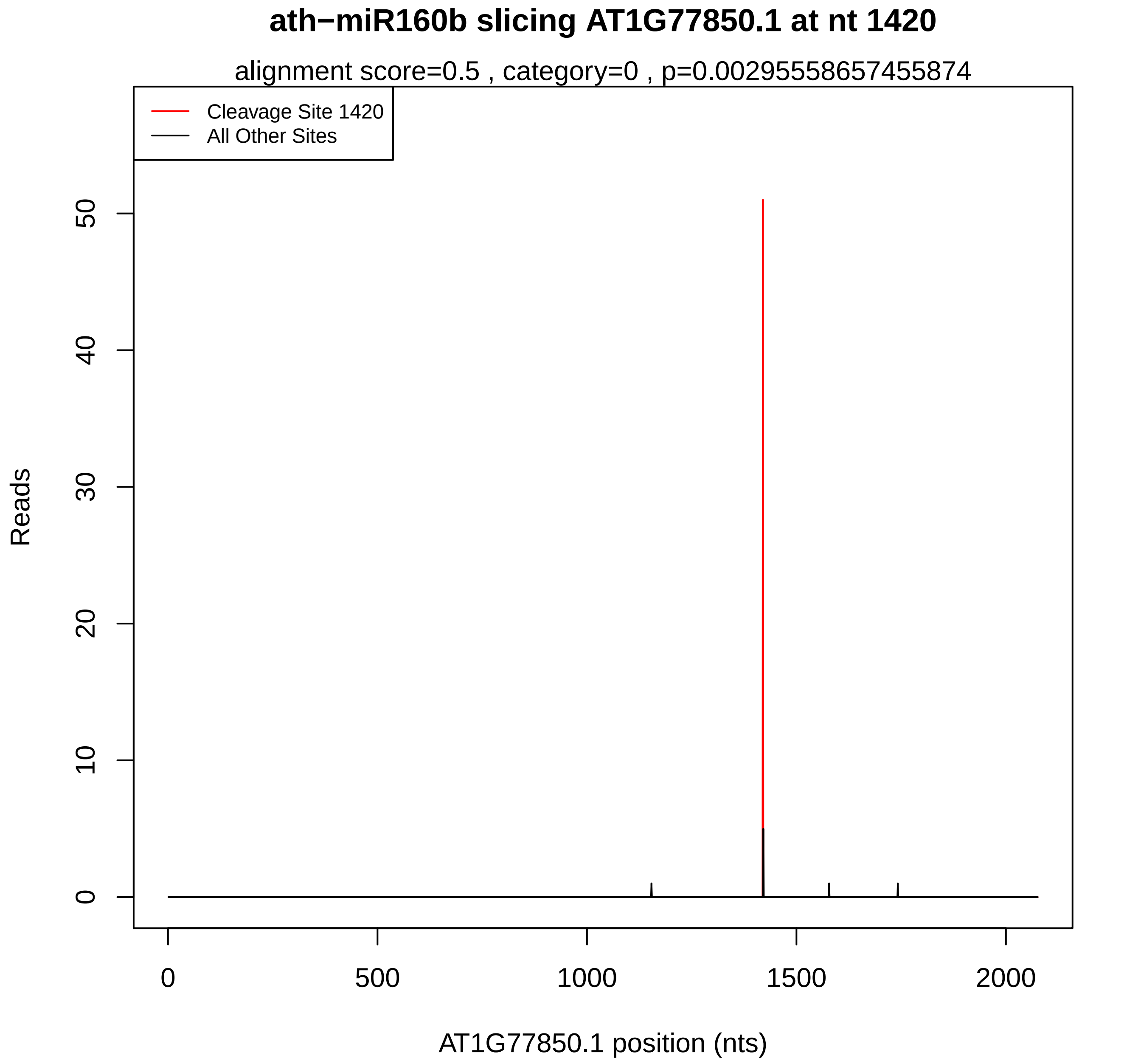
ath−miR160b slicing AT1G77850.1 at nt 1420
alignment score=0.5 , category=0 , p=0.00295558657455874
Cleavage Site 1420
All Other Sites
Reads
0
10
20
30
40
50
0
500
1000
1500
2000
AT1G77850.1 position (nts)

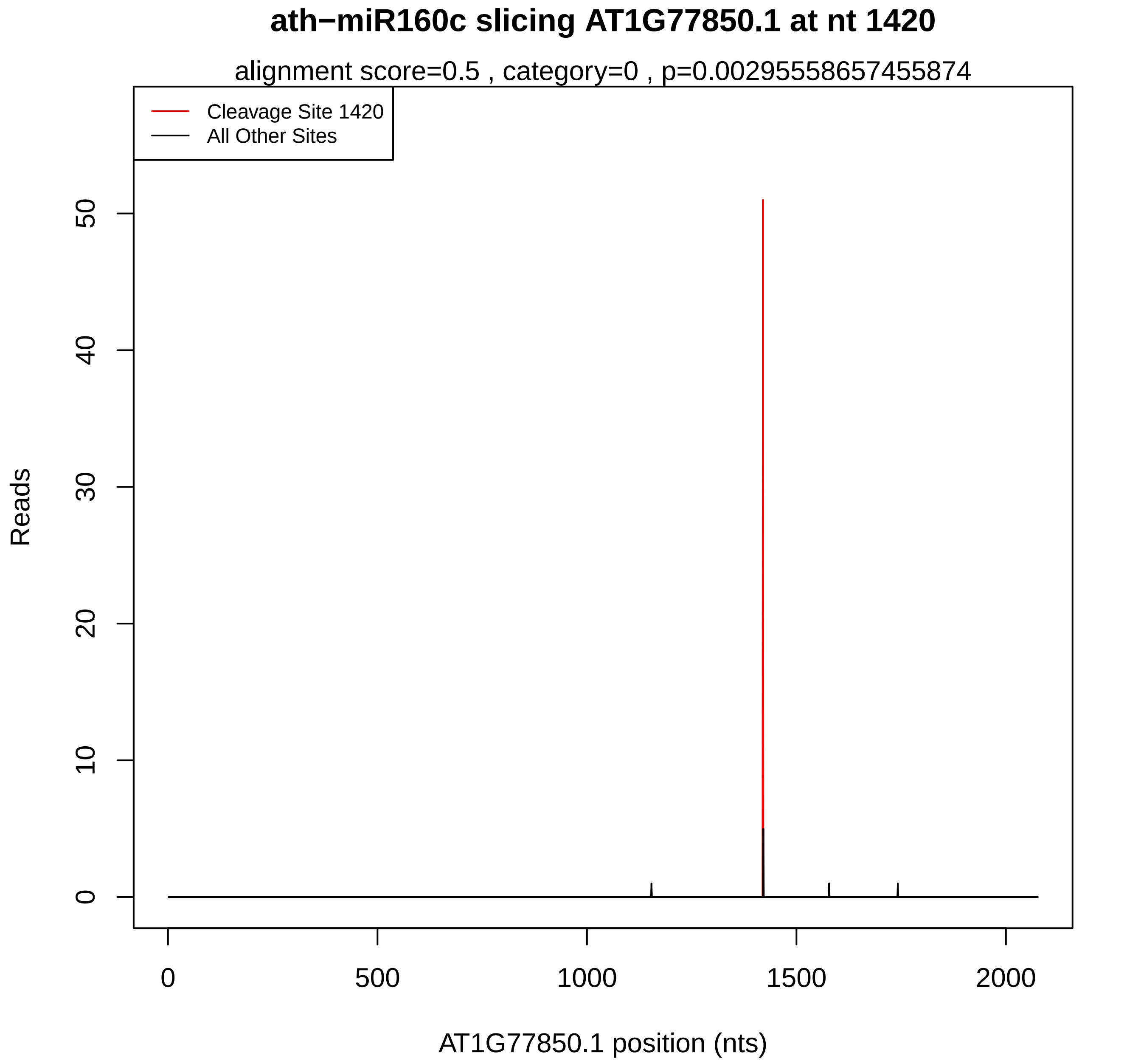
ath−miR160c slicing AT1G77850.1 at nt 1420
alignment score=0.5 , category=0 , p=0.00295558657455874
Cleavage Site 1420
All Other Sites
Reads
0
10
20
30
40
50
0
500
1000
1500
2000
AT1G77850.1 position (nts)

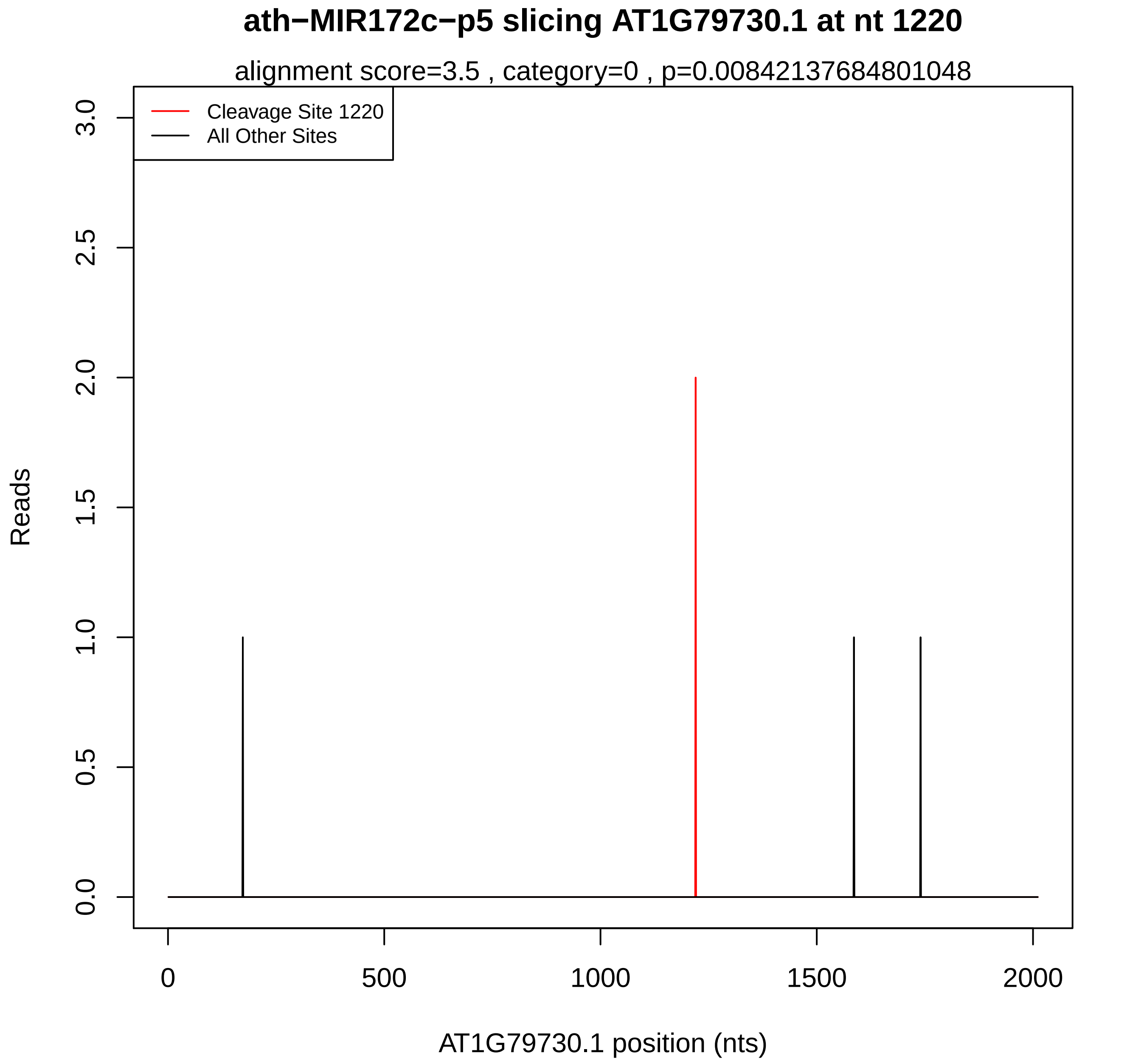
ath−MIR172c−p5 slicing AT1G79730.1 at nt 1220
alignment score=3.5 , category=0 , p=0.00842137684801048
Cleavage Site 1220
All Other Sites
Reads
0.0
0.5
1.0
1.5
2.0
2.5
3.0
0
500
1000
1500
2000
AT1G79730.1 position (nts)

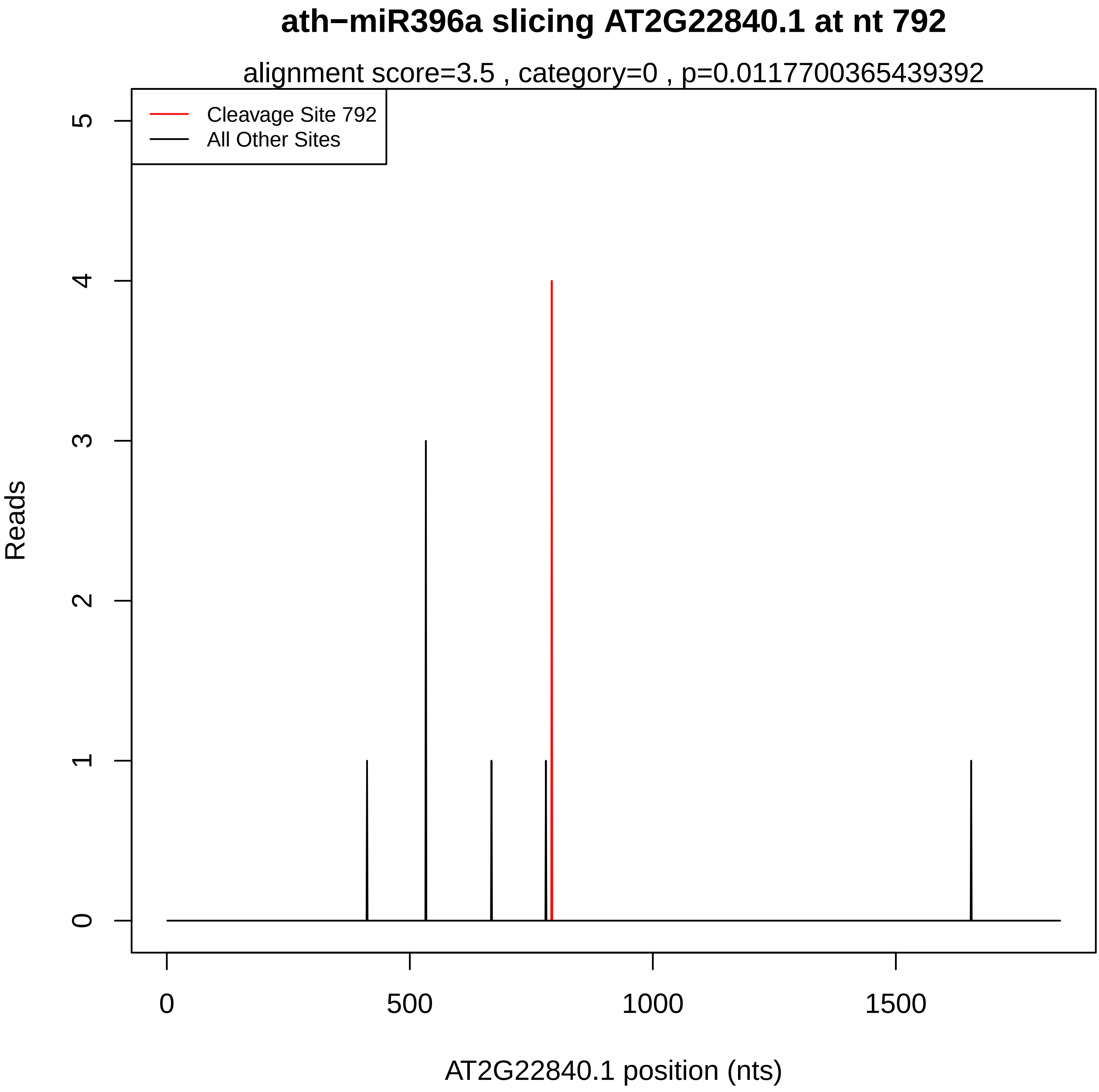

ath−miR396a slicing AT2G22840.1 at nt 792
alignment score=3.5 , category=0 , p=0.0117700365439392
Cleavage Site 792
All Other Sites
Reads
0
1
2
3
4
5
0
500
1000
1500
AT2G22840.1 position (nts)

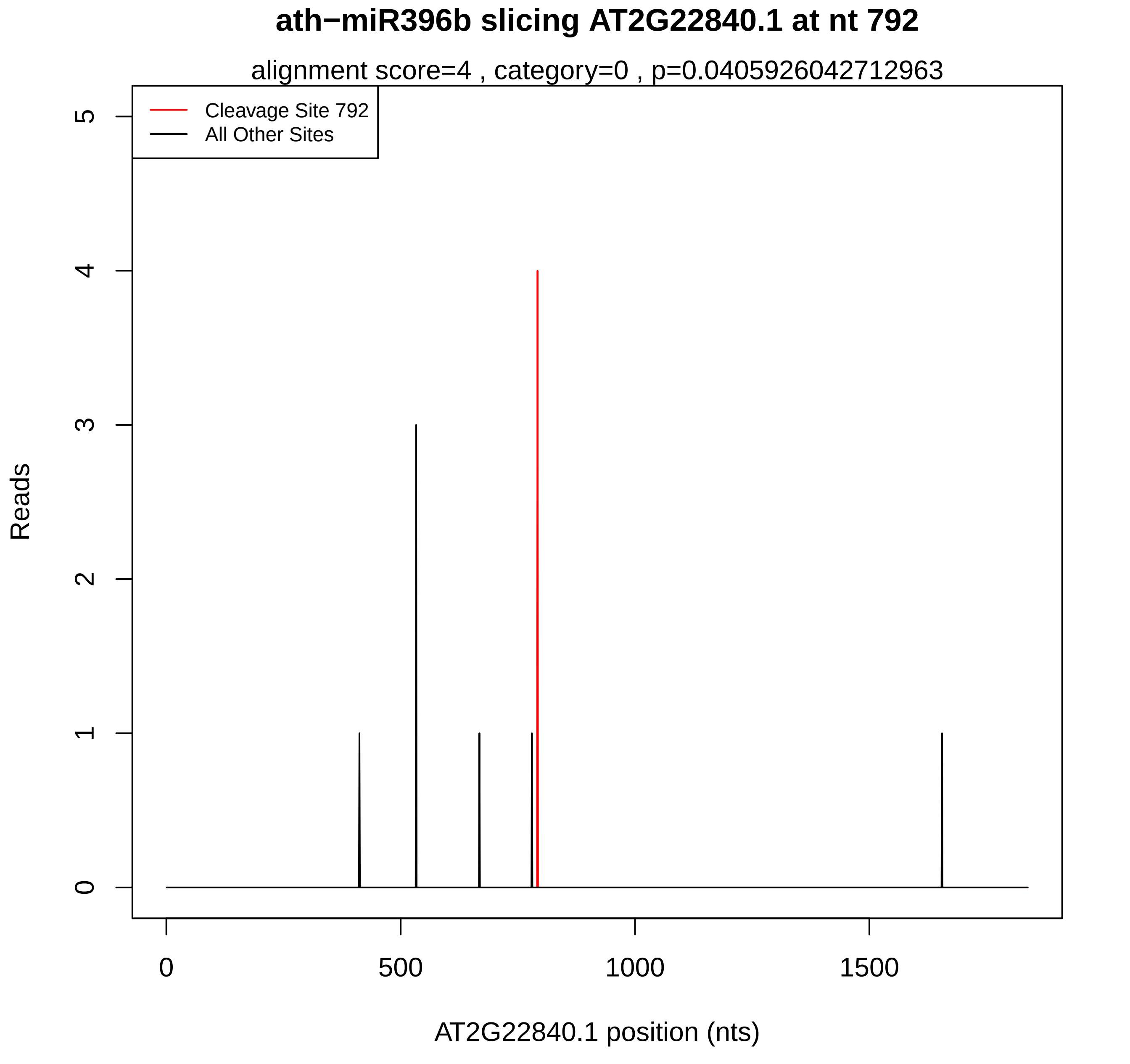

ath−miR396b slicing AT2G22840.1 at nt 792
alignment score=4 , category=0 , p=0.0405926042712963
Cleavage Site 792
All Other Sites
Reads
0
1
2
3
4
5
0
500
1000
1500
AT2G22840.1 position (nts)

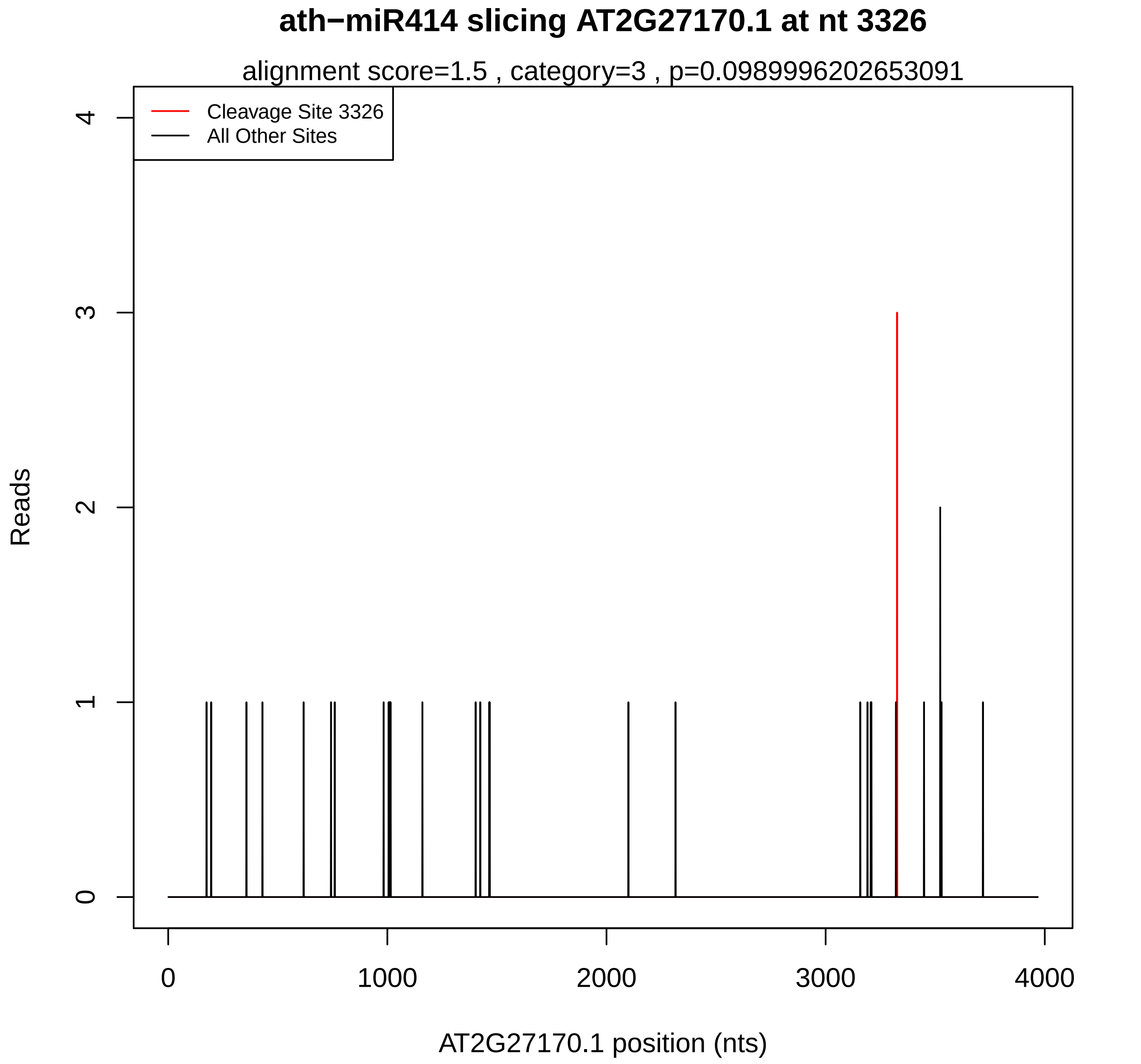

ath−miR414 slicing AT2G27170.1 at nt 3326
alignment score=1.5 , category=3 , p=0.0989996202653091
Cleavage Site 3326
All Other Sites
Reads
0
1
2
3
4
0
1000
2000
3000
4000
AT2G27170.1 position (nts)

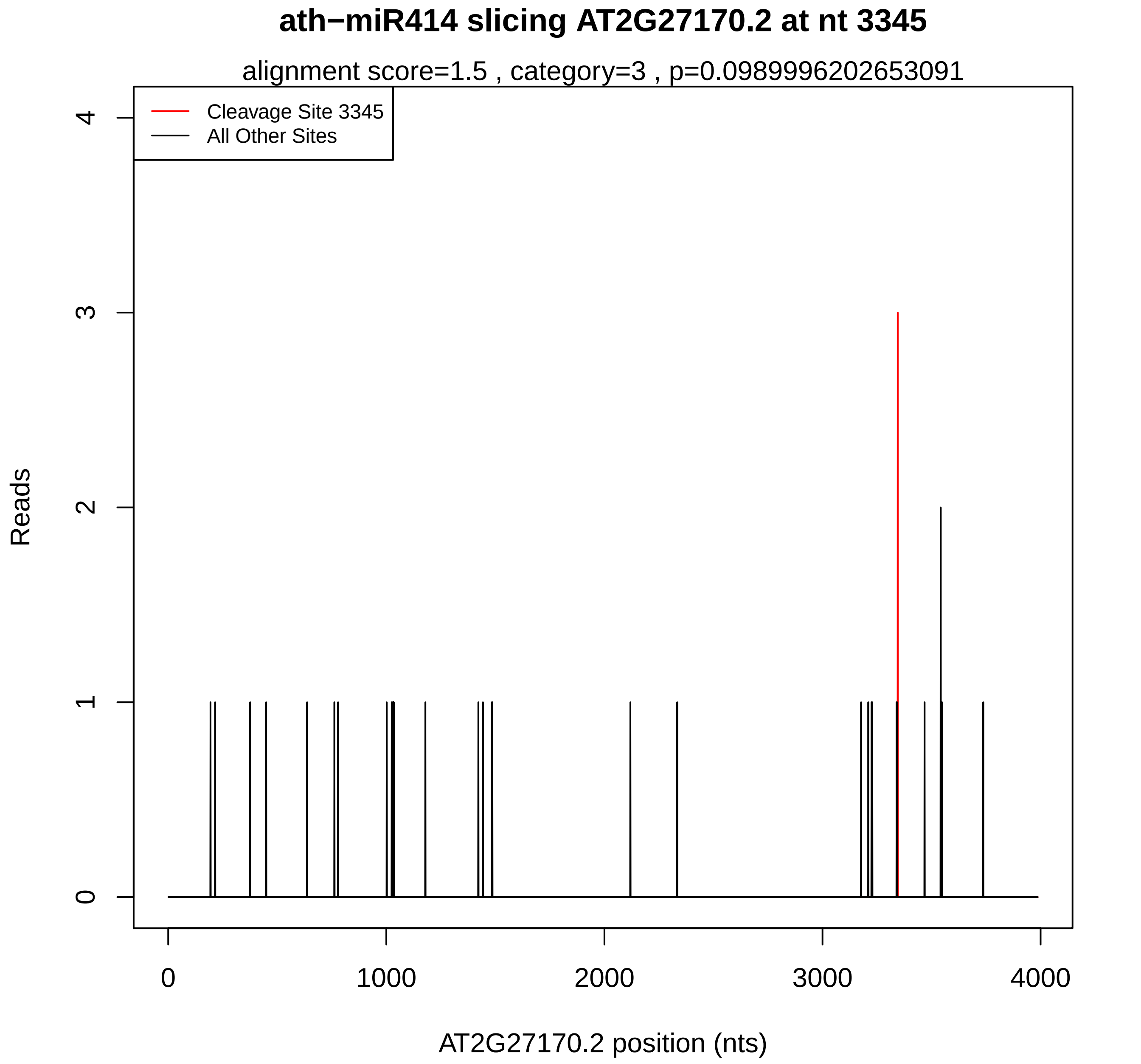
ath−miR414 slicing AT2G27170.2 at nt 3345
alignment score=1.5 , category=3 , p=0.0989996202653091
Cleavage Site 3345
All Other Sites
Reads
0
1
2
3
4
0
1000
2000
3000
4000
AT2G27170.2 position (nts)

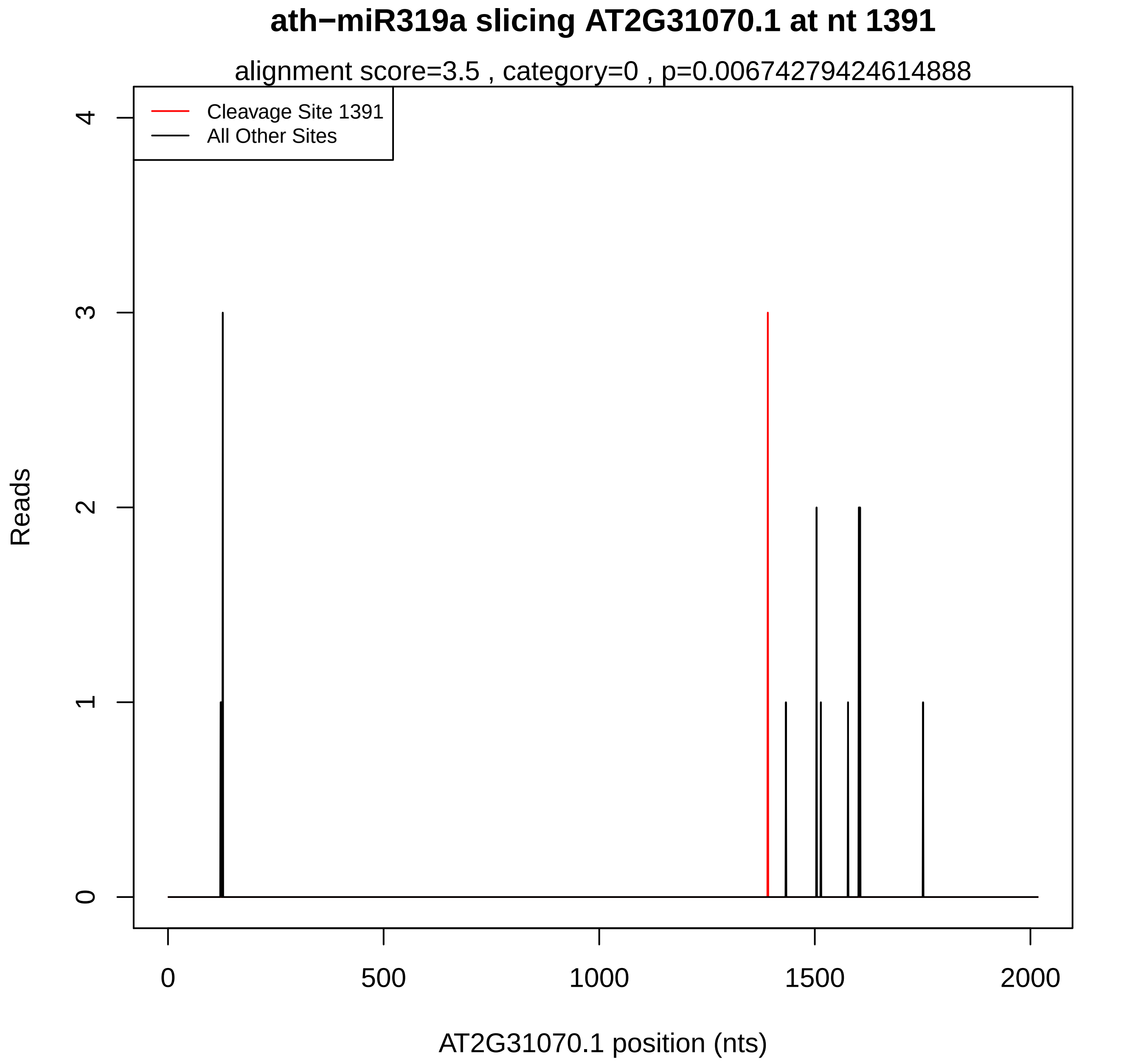

ath−miR319a slicing AT2G31070.1 at nt 1391
alignment score=3.5 , category=0 , p=0.00674279424614888
Cleavage Site 1391
All Other Sites
Reads
0
1
2
3
4
0
500
1000
1500
2000
AT2G31070.1 position (nts)

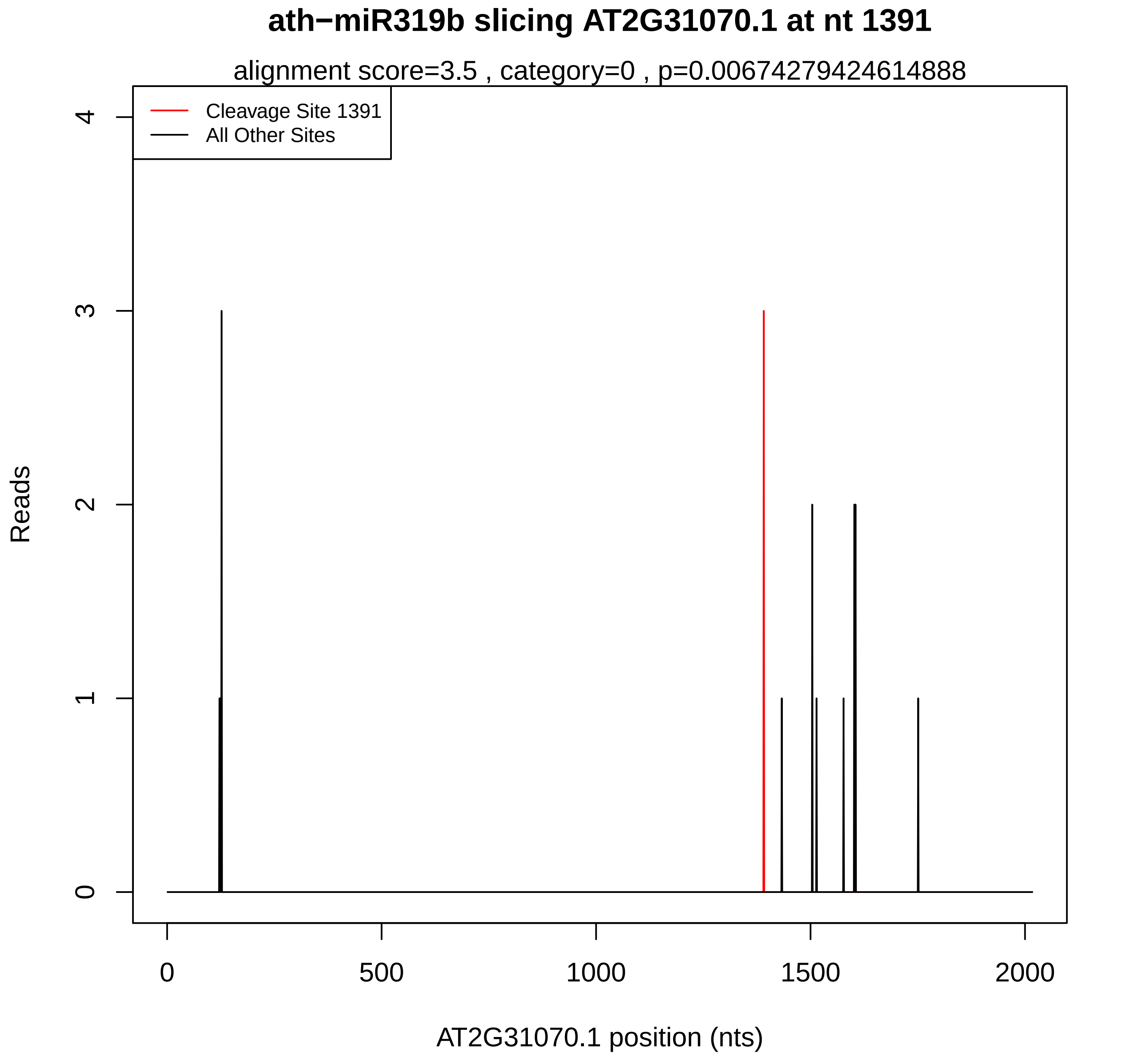
ath−miR319b slicing AT2G31070.1 at nt 1391
alignment score=3.5 , category=0 , p=0.00674279424614888
Cleavage Site 1391
All Other Sites
Reads
0
1
2
3
4
0
500
1000
1500
2000
AT2G31070.1 position (nts)

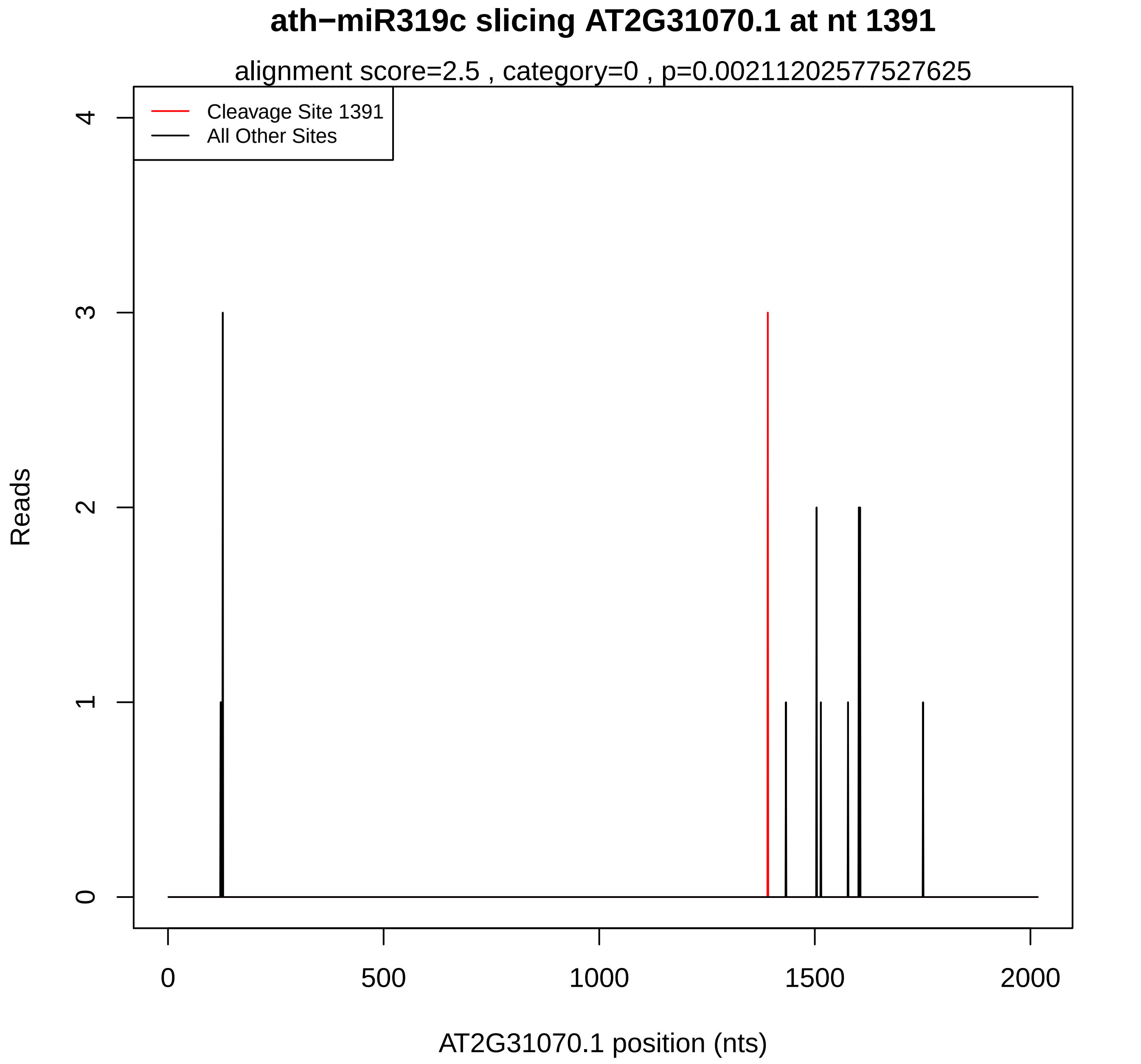
ath−miR319c slicing AT2G31070.1 at nt 1391
alignment score=2.5 , category=0 , p=0.00211202577527625
Cleavage Site 1391
All Other Sites
Reads
0
1
2
3
4
0
500
1000
1500
2000
AT2G31070.1 position (nts)

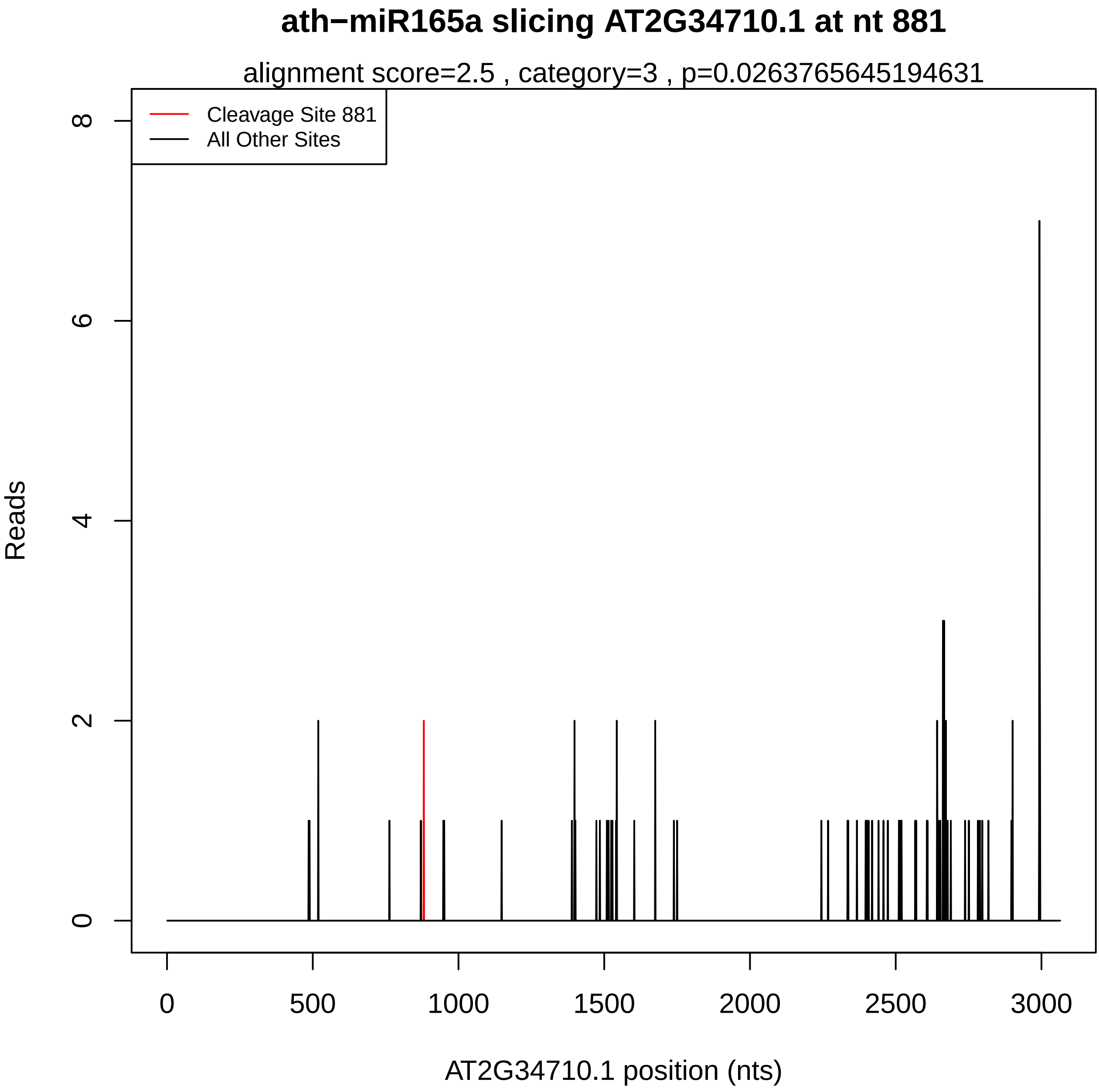
ath−miR165a slicing AT2G34710.1 at nt 881
alignment score=2.5 , category=3 , p=0.0263765645194631
Cleavage Site 881
All Other Sites
Reads
0
2
4
6
8
0
500
1000
1500
2000
2500
3000
AT2G34710.1 position (nts)

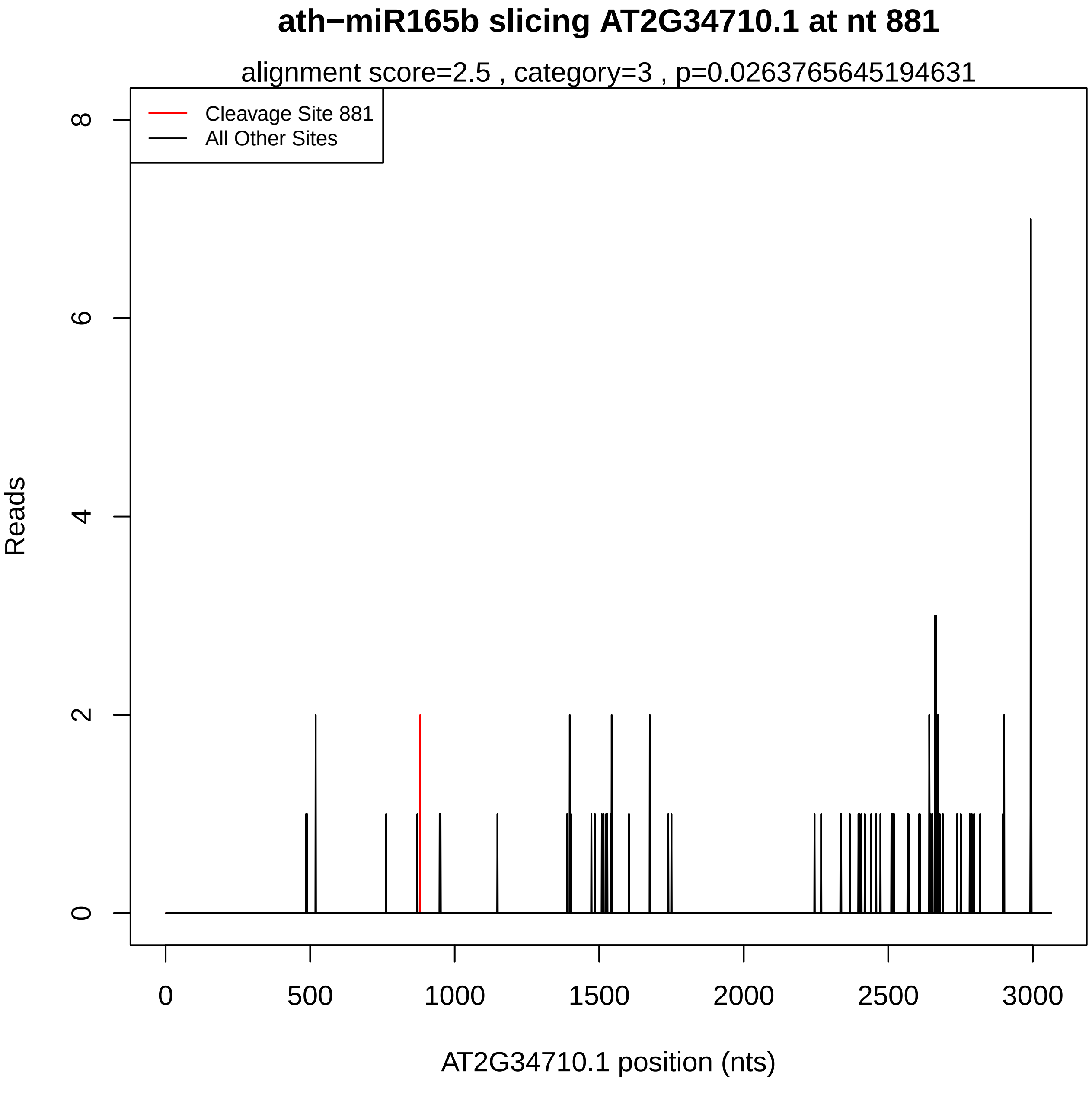
ath−miR165b slicing AT2G34710.1 at nt 881
alignment score=2.5 , category=3 , p=0.0263765645194631
Cleavage Site 881
All Other Sites
Reads
0
2
4
6
8
0
500
1000
1500
2000
2500
3000
AT2G34710.1 position (nts)

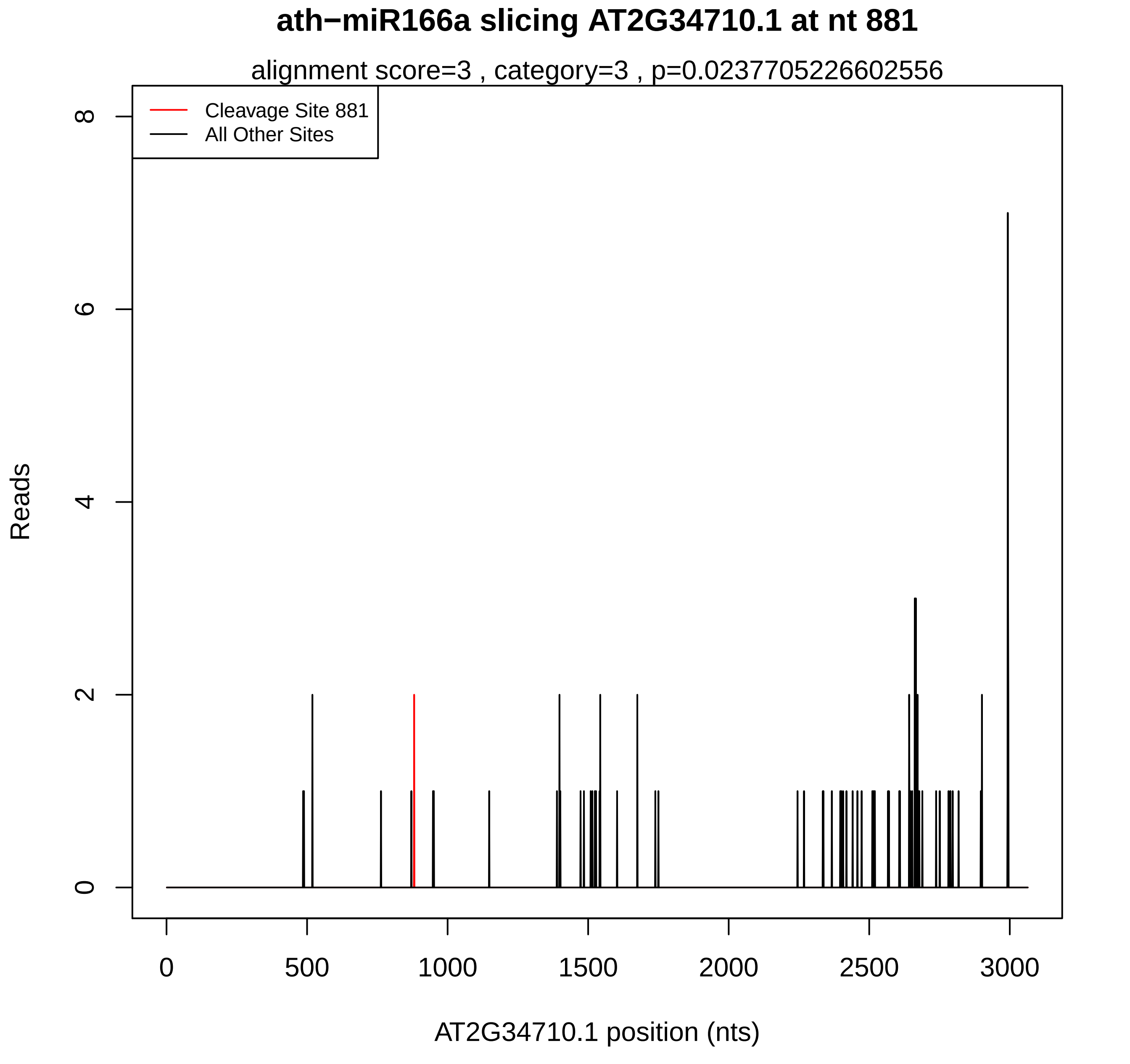

ath−miR166a slicing AT2G34710.1 at nt 881
alignment score=3 , category=3 , p=0.0237705226602556
Cleavage Site 881
All Other Sites
Reads
0
2
4
6
8
0
500
1000
1500
2000
2500
3000
AT2G34710.1 position (nts)

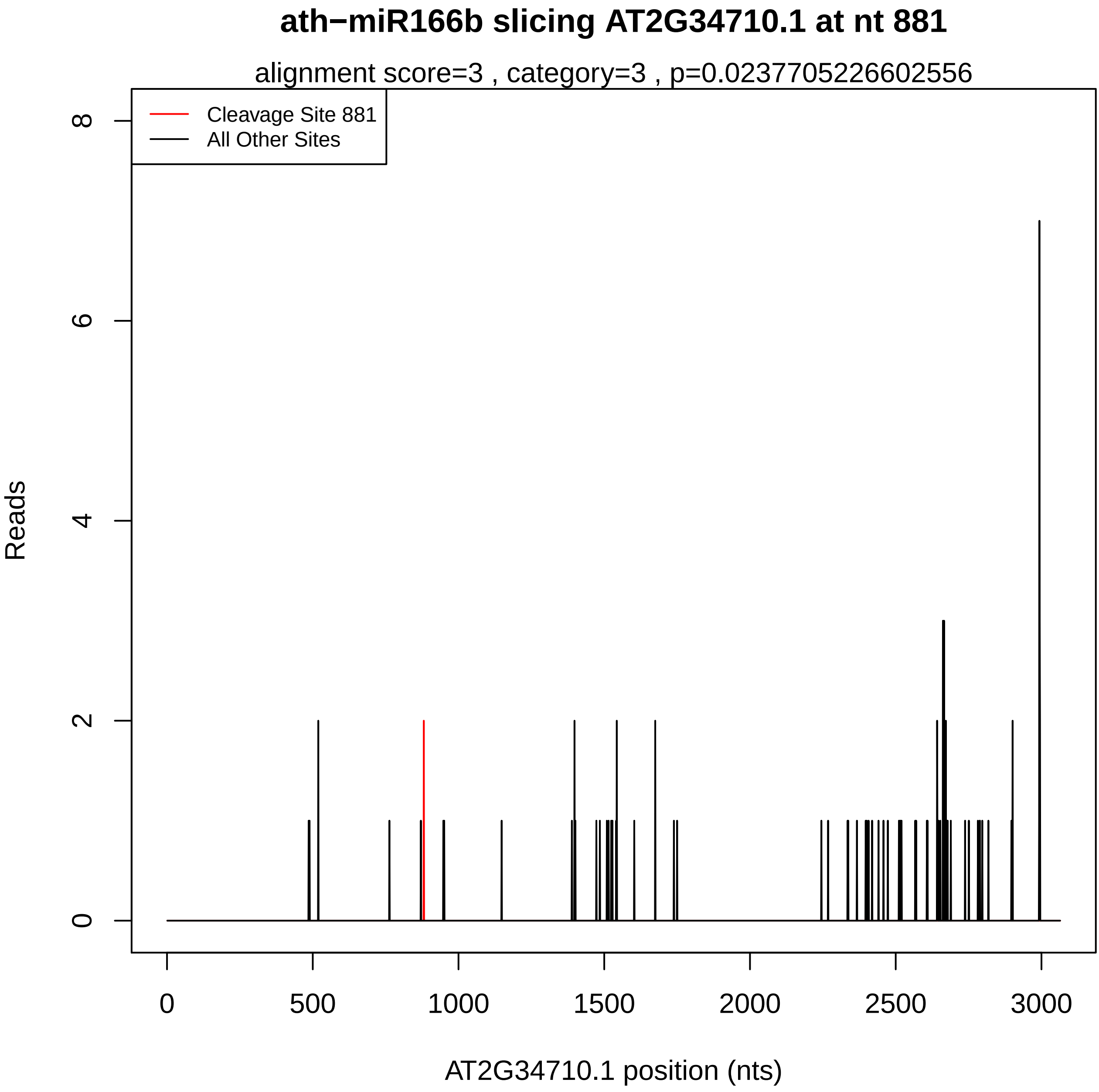

ath−miR166b slicing AT2G34710.1 at nt 881
alignment score=3 , category=3 , p=0.0237705226602556
Cleavage Site 881
All Other Sites
Reads
0
2
4
6
8
0
500
1000
1500
2000
2500
3000
AT2G34710.1 position (nts)

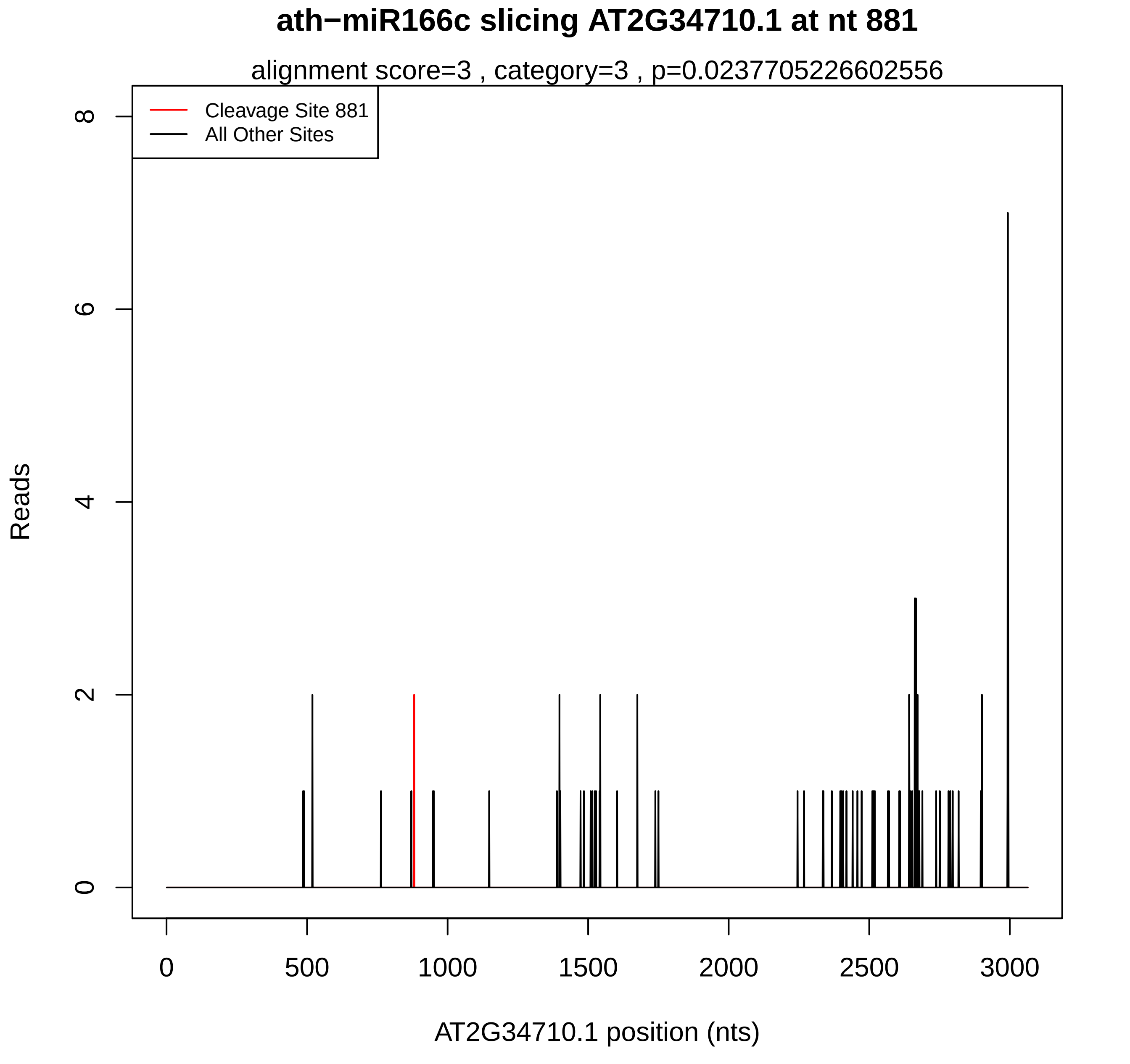
ath−miR166c slicing AT2G34710.1 at nt 881
alignment score=3 , category=3 , p=0.0237705226602556
Cleavage Site 881
All Other Sites
Reads
0
2
4
6
8
0
500
1000
1500
2000
2500
3000
AT2G34710.1 position (nts)

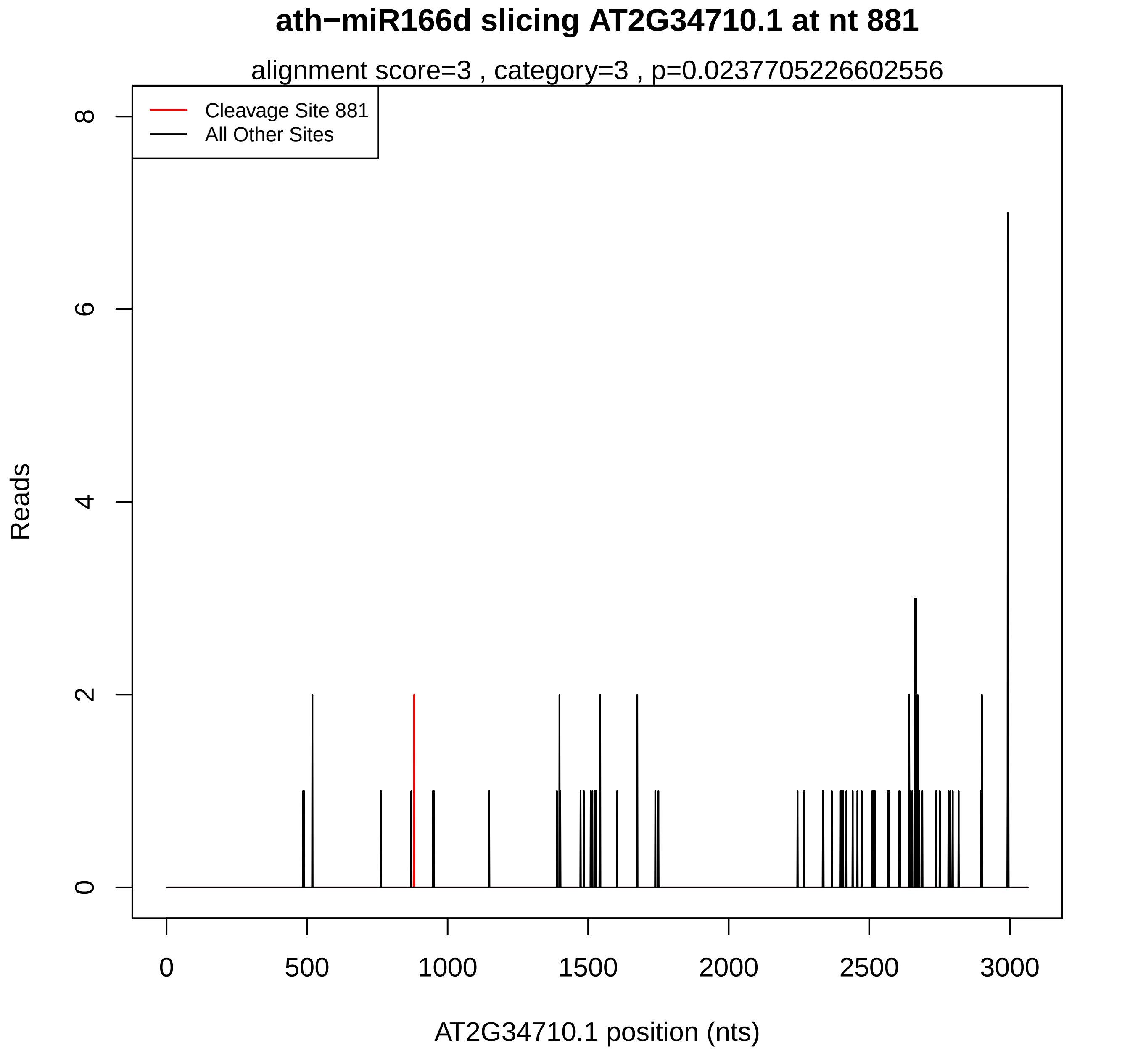

ath−miR166d slicing AT2G34710.1 at nt 881
alignment score=3 , category=3 , p=0.0237705226602556
Cleavage Site 881
All Other Sites
Reads
0
2
4
6
8
0
500
1000
1500
2000
2500
3000
AT2G34710.1 position (nts)

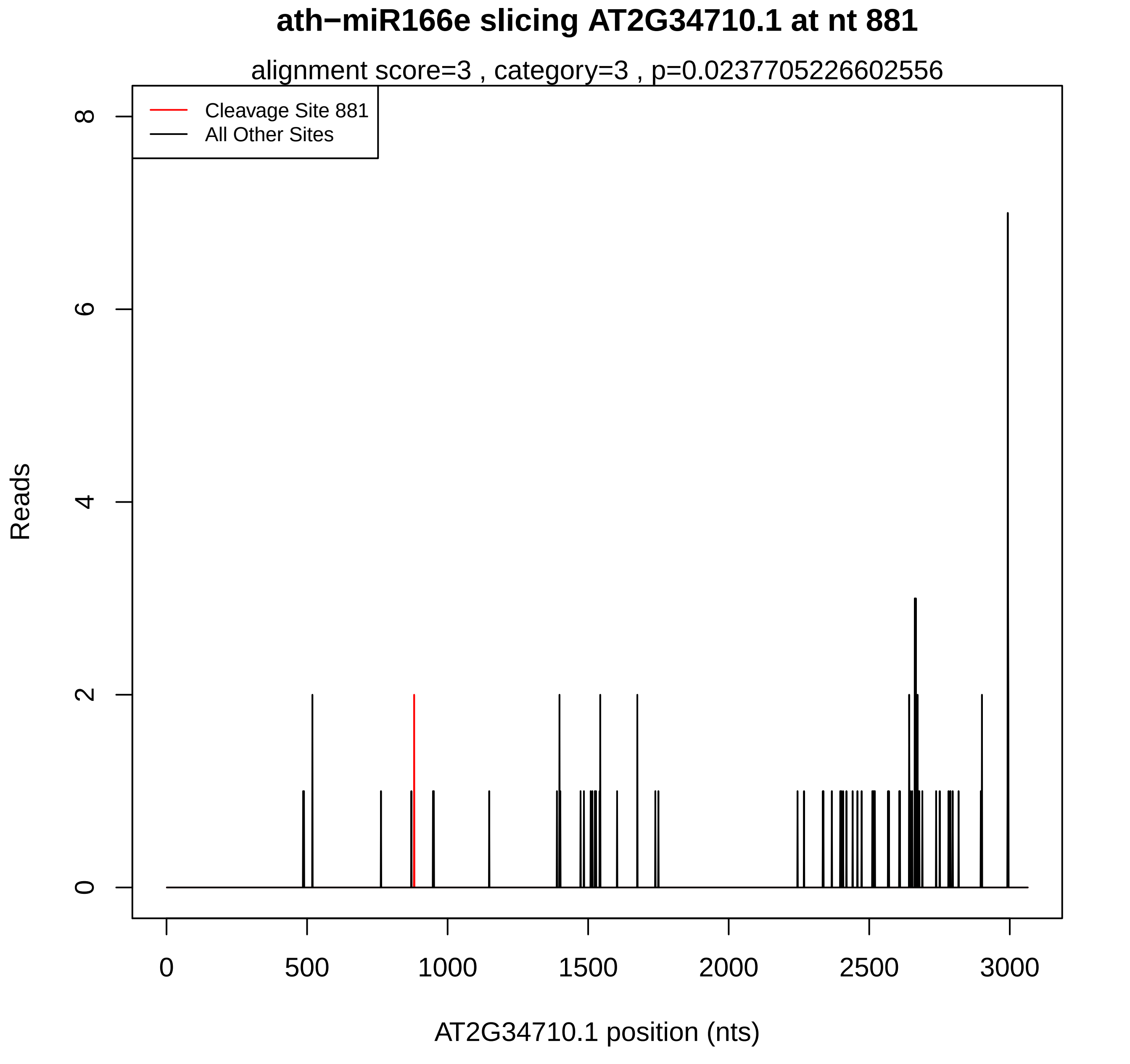
ath−miR166e slicing AT2G34710.1 at nt 881
alignment score=3 , category=3 , p=0.0237705226602556
Cleavage Site 881
All Other Sites
Reads
0
2
4
6
8
0
500
1000
1500
2000
2500
3000
AT2G34710.1 position (nts)

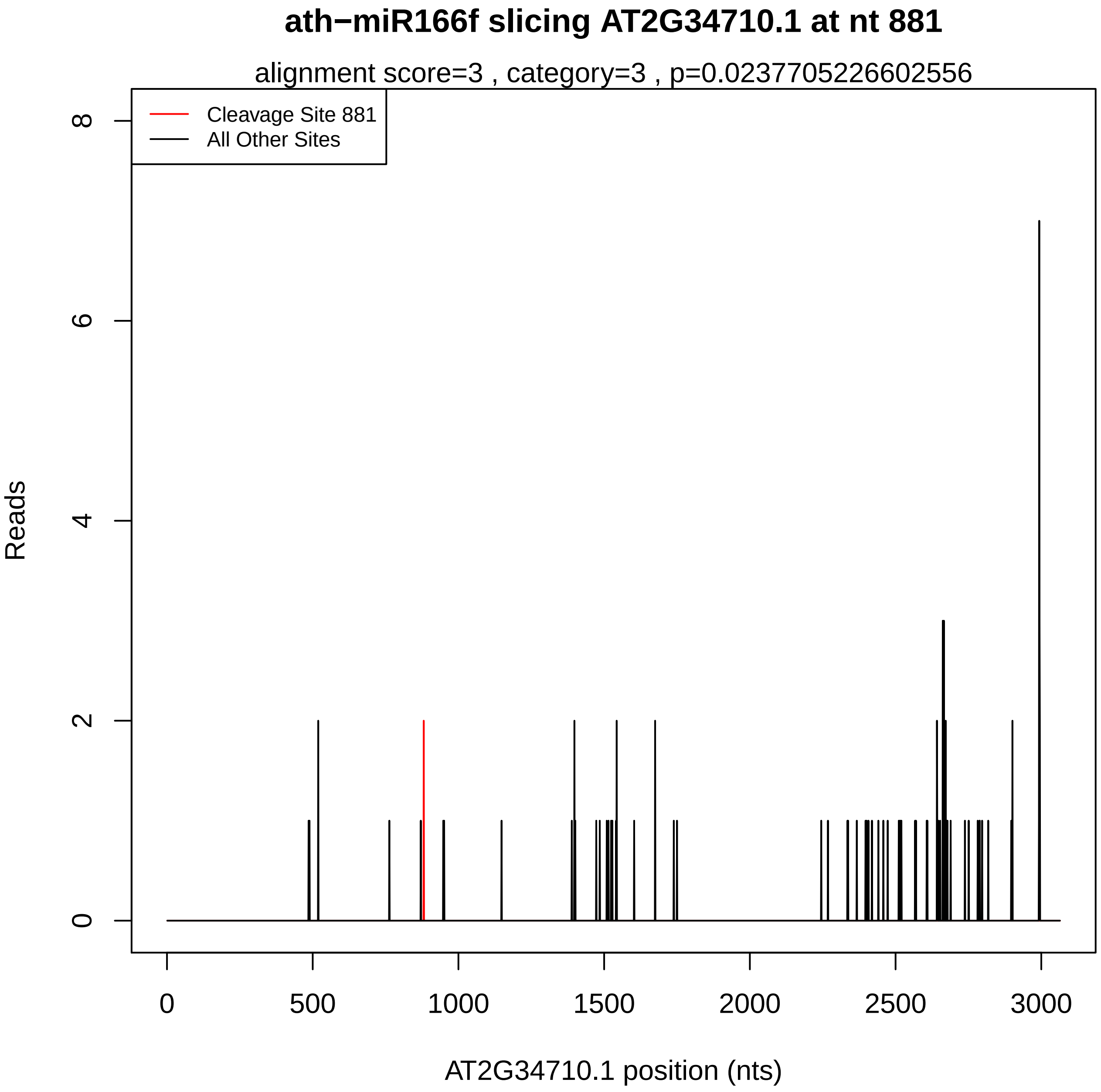
ath−miR166f slicing AT2G34710.1 at nt 881
alignment score=3 , category=3 , p=0.0237705226602556
Cleavage Site 881
All Other Sites
Reads
0
2
4
6
8
0
500
1000
1500
2000
2500
3000
AT2G34710.1 position (nts)

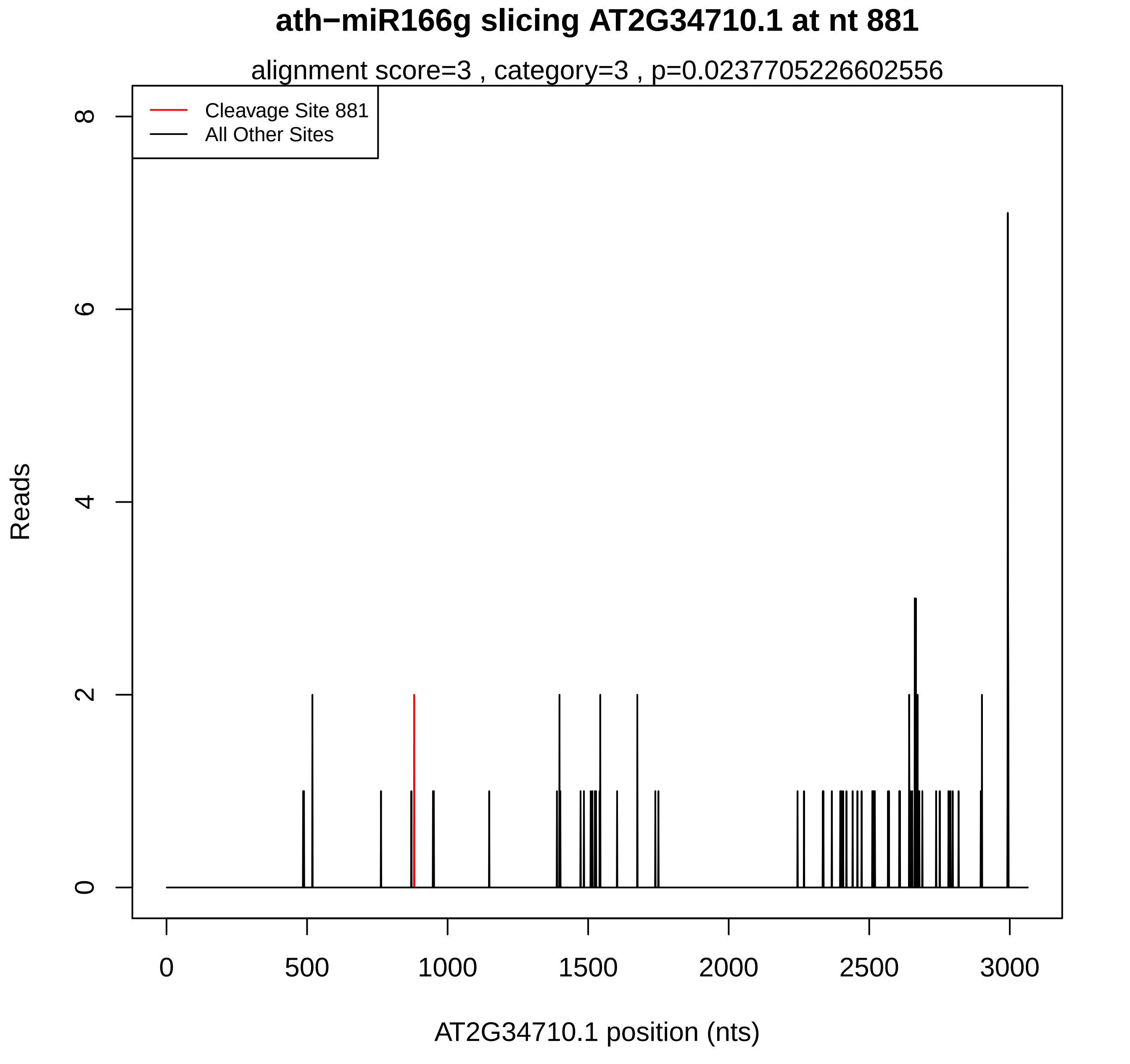
ath−miR166g slicing AT2G34710.1 at nt 881
alignment score=3 , category=3 , p=0.0237705226602556
Cleavage Site 881
All Other Sites
Reads
0
2
4
6
8
0
500
1000
1500
2000
2500
3000
AT2G34710.1 position (nts)

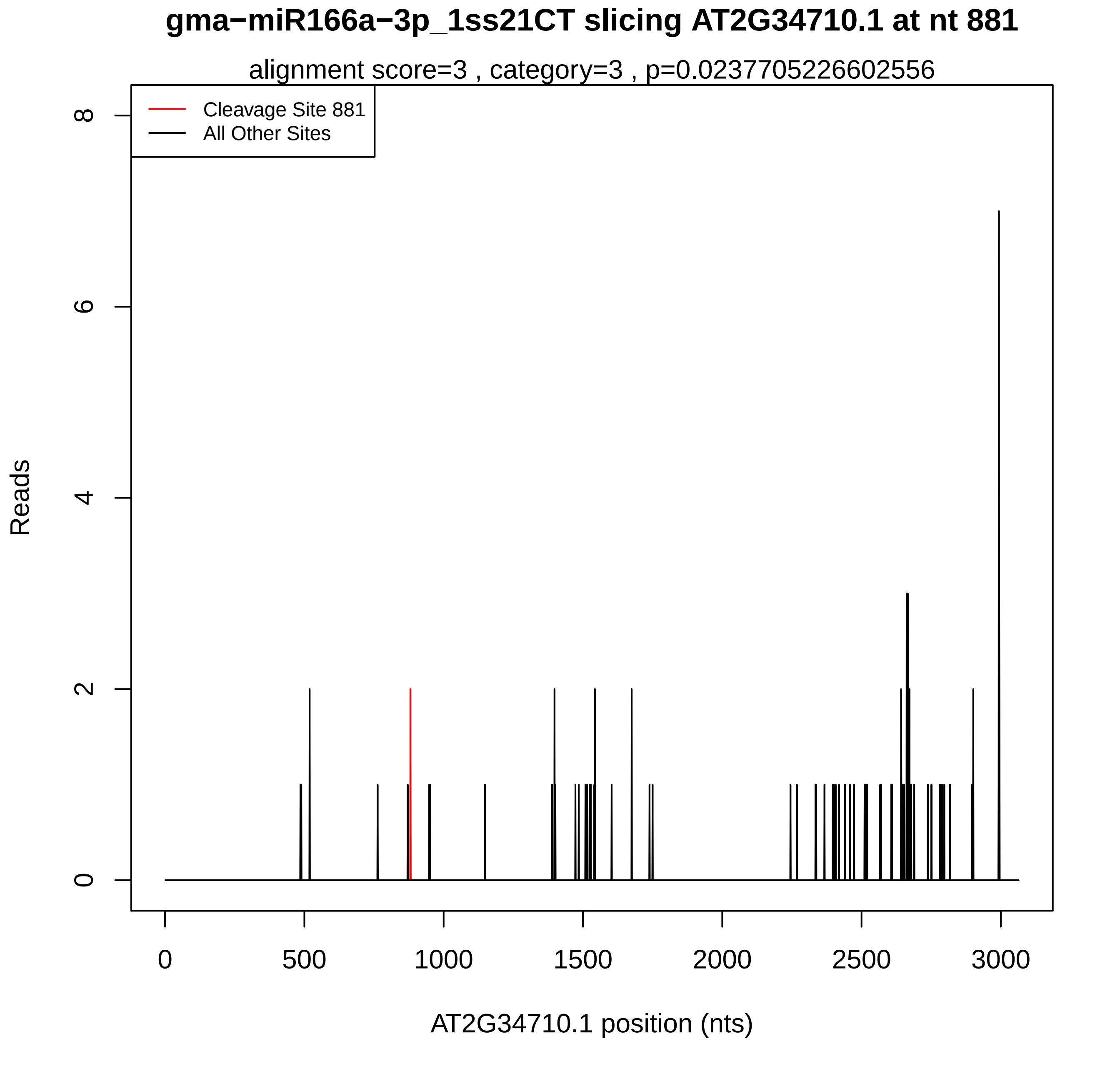
gma−miR166a−3p_1ss21CT slicing AT2G34710.1 at nt 881
alignment score=3 , category=3 , p=0.0237705226602556
Cleavage Site 881
All Other Sites
Reads
0
2
4
6
8
0
500
1000
1500
2000
2500
3000
AT2G34710.1 position (nts)

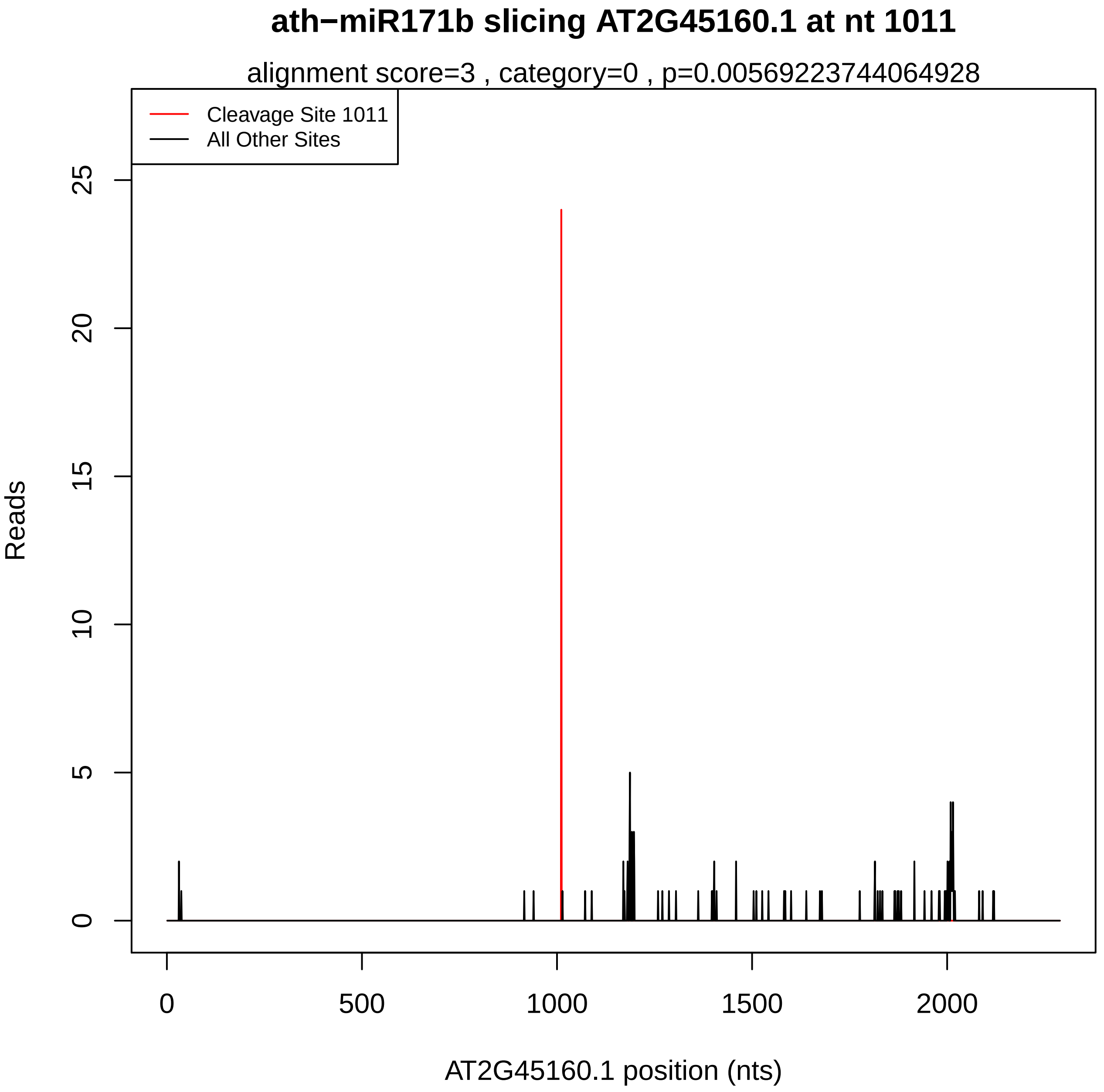

ath−miR171b slicing AT2G45160.1 at nt 1011
alignment score=3 , category=0 , p=0.00569223744064928
Cleavage Site 1011
All Other Sites
Reads
0
5
10
15
20
25
0
500
1000
1500
2000
AT2G45160.1 position (nts)

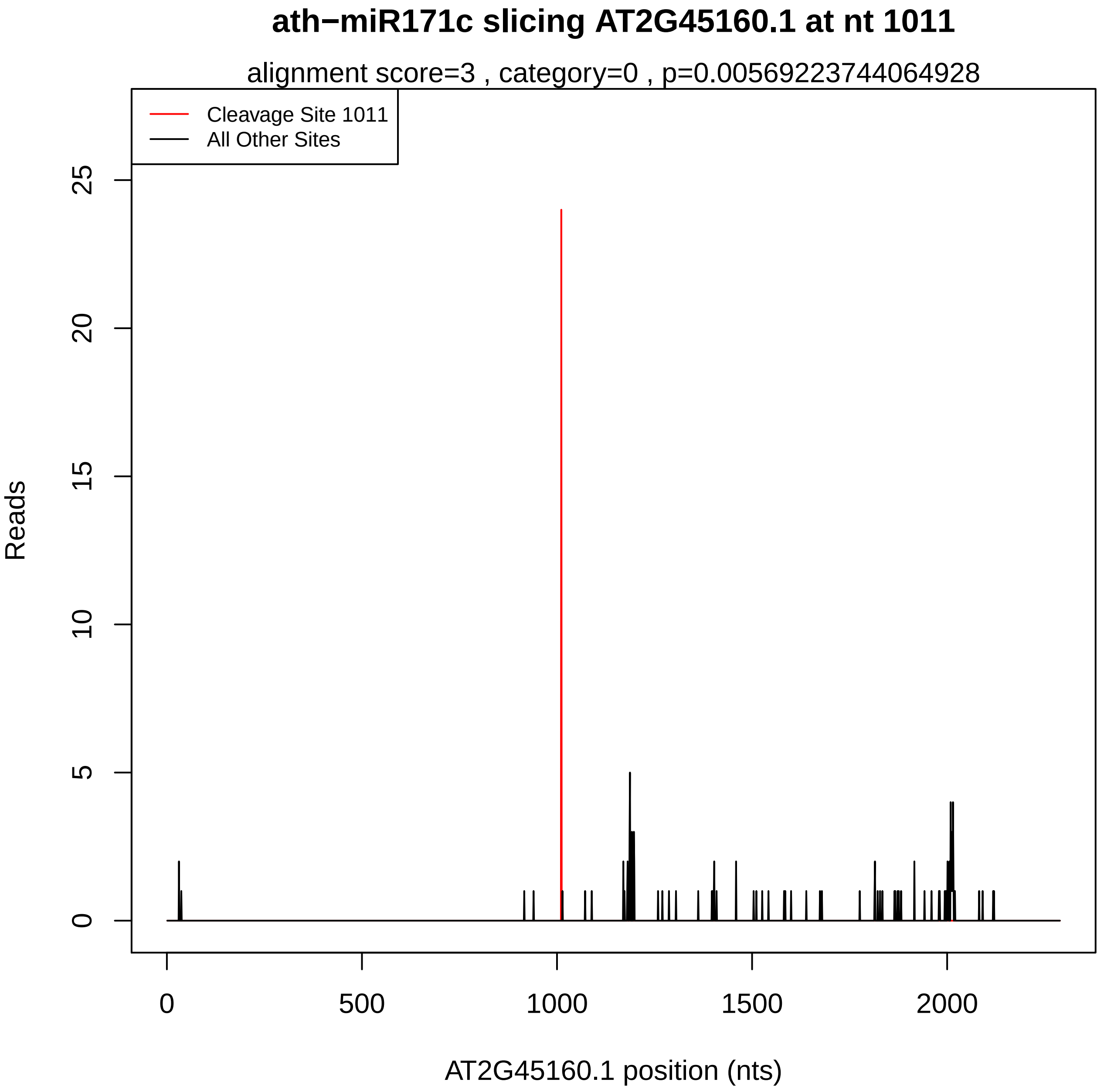
ath−miR171c slicing AT2G45160.1 at nt 1011
alignment score=3 , category=0 , p=0.00569223744064928
Cleavage Site 1011
All Other Sites
Reads
0
5
10
15
20
25
0
500
1000
1500
2000
AT2G45160.1 position (nts)

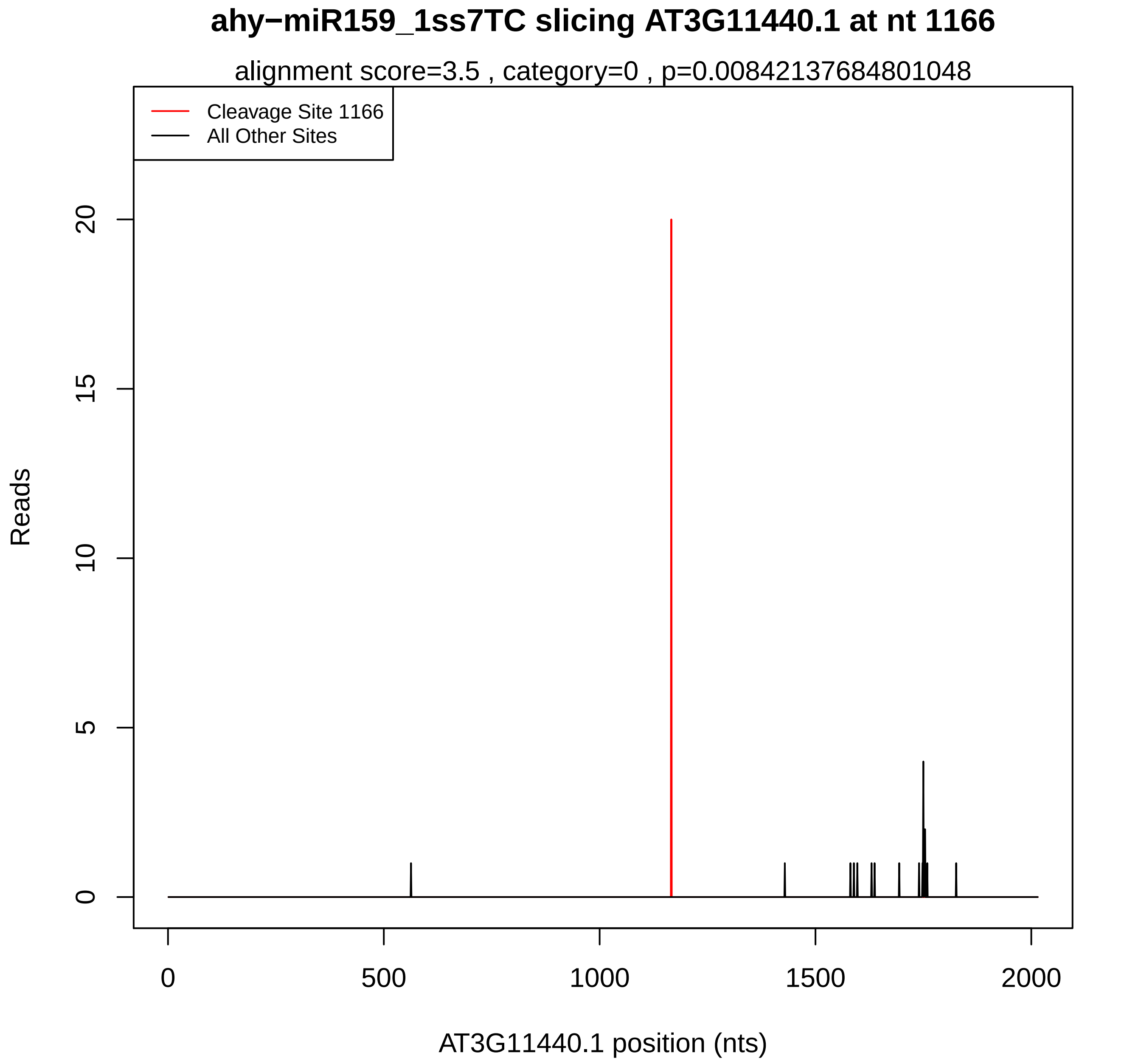
ahy−miR159_1ss7TC slicing AT3G11440.1 at nt 1166
alignment score=3.5 , category=0 , p=0.00842137684801048
Cleavage Site 1166
All Other Sites
Reads
0
5
10
15
20
0
500
1000
1500
2000
AT3G11440.1 position (nts)

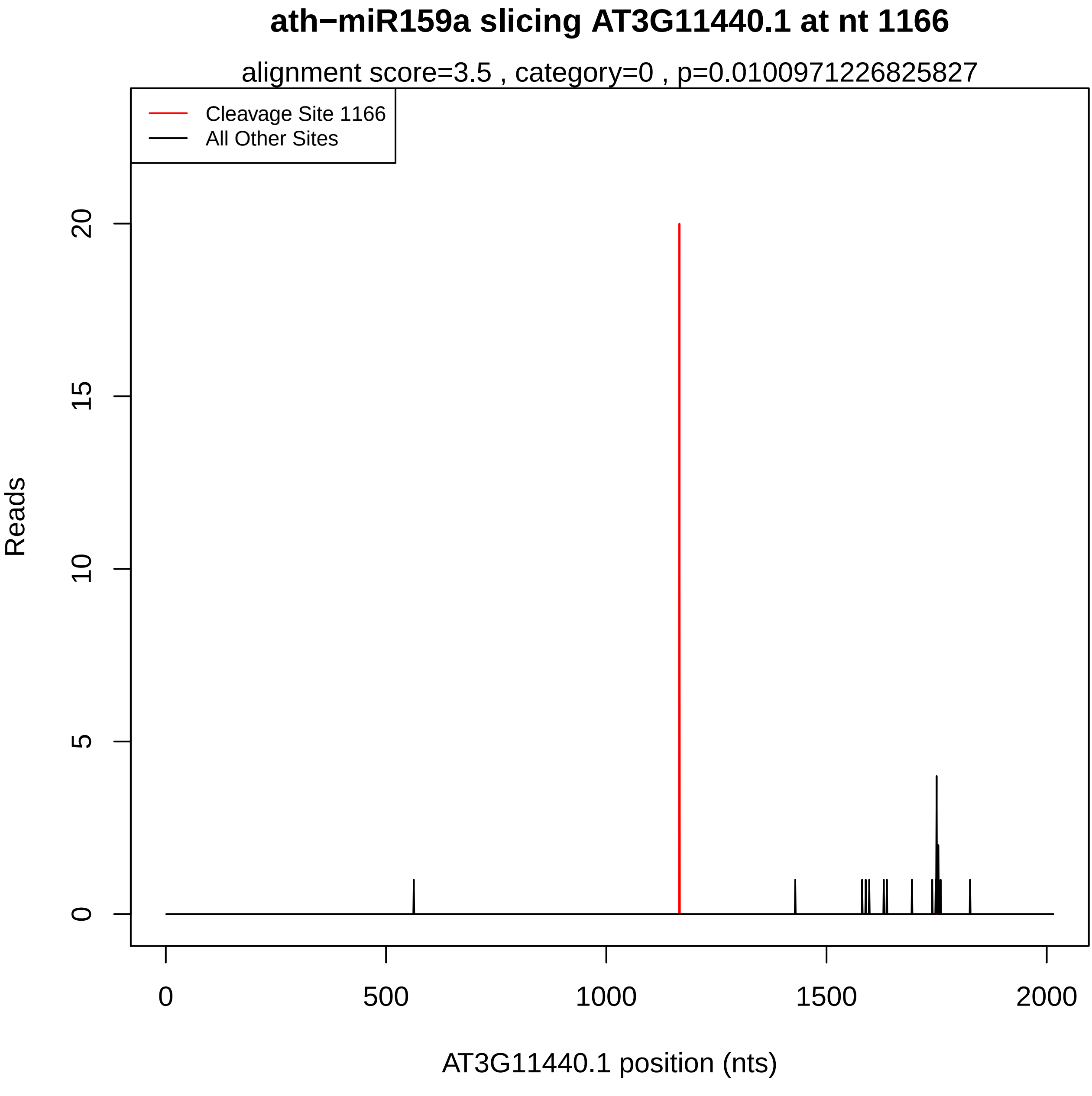
ath−miR159a slicing AT3G11440.1 at nt 1166
alignment score=3.5 , category=0 , p=0.0100971226825827
Cleavage Site 1166
All Other Sites
Reads
0
5
10
15
20
0
500
1000
1500
2000
AT3G11440.1 position (nts)

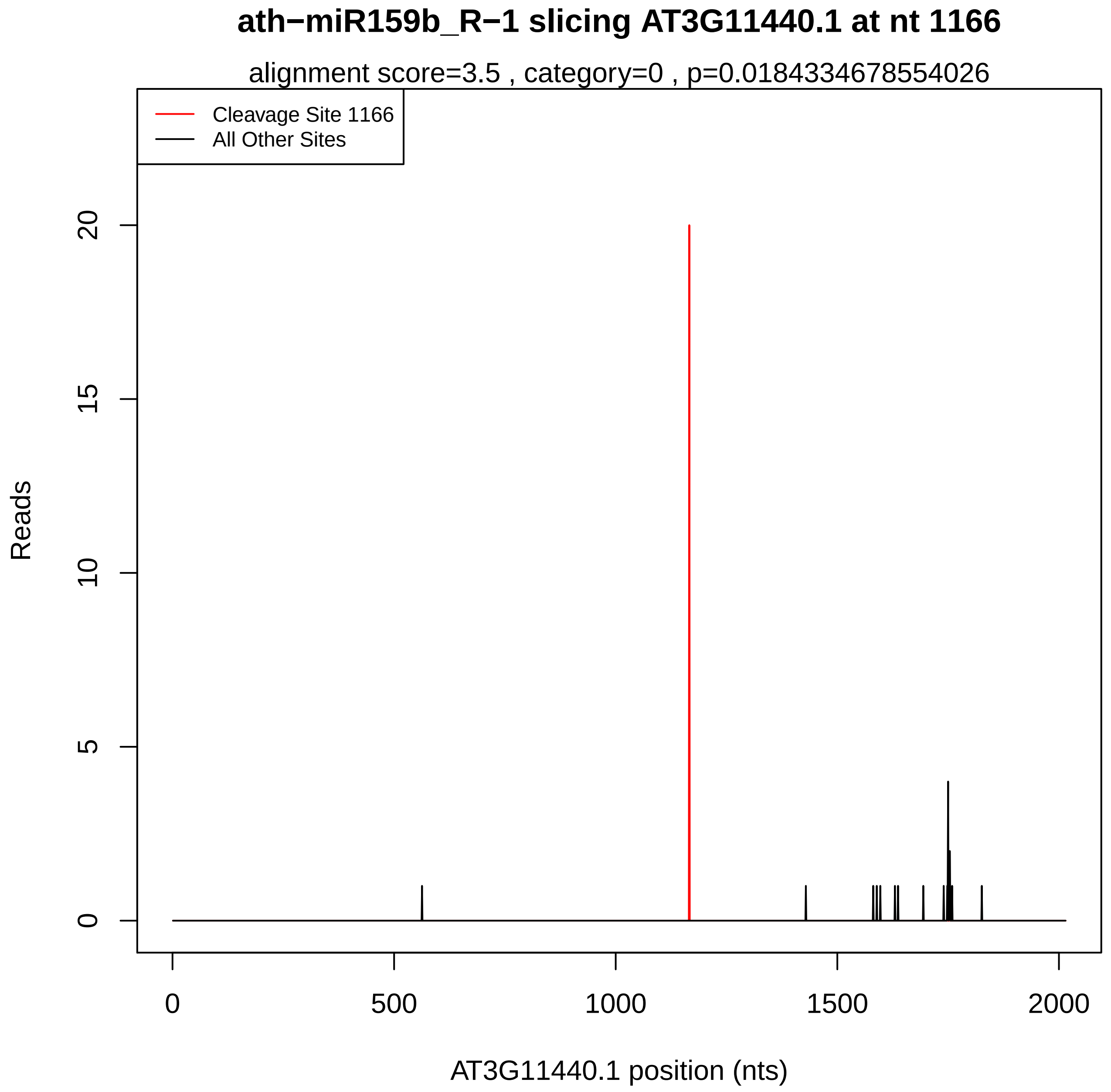
ath−miR159b_R−1 slicing AT3G11440.1 at nt 1166
alignment score=3.5 , category=0 , p=0.0184334678554026
Cleavage Site 1166
All Other Sites
Reads
0
5
10
15
20
0
500
1000
1500
2000
AT3G11440.1 position (nts)

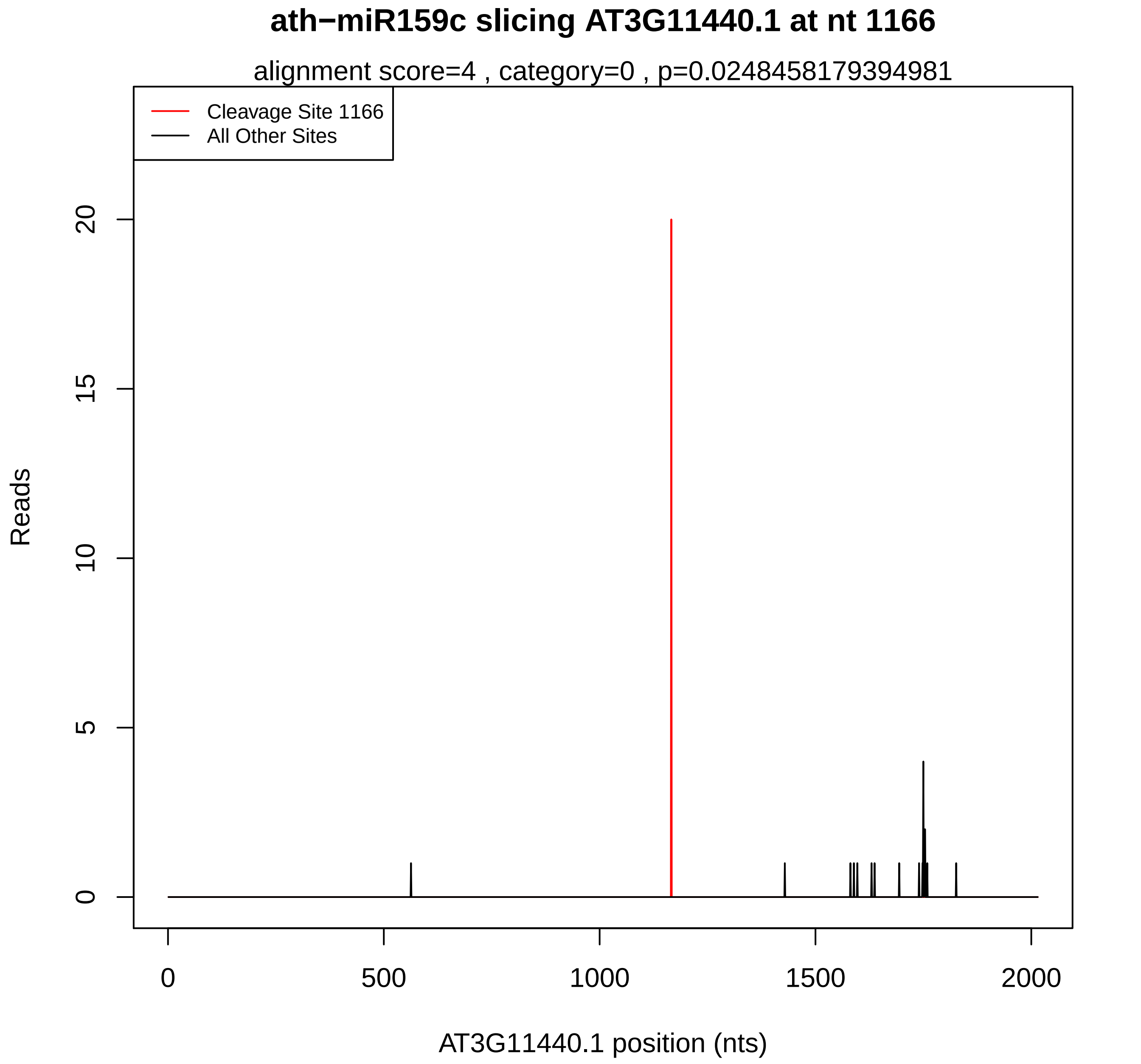

ath−miR159c slicing AT3G11440.1 at nt 1166
alignment score=4 , category=0 , p=0.0248458179394981
Cleavage Site 1166
All Other Sites
Reads
0
5
10
15
20
0
500
1000
1500
2000
AT3G11440.1 position (nts)

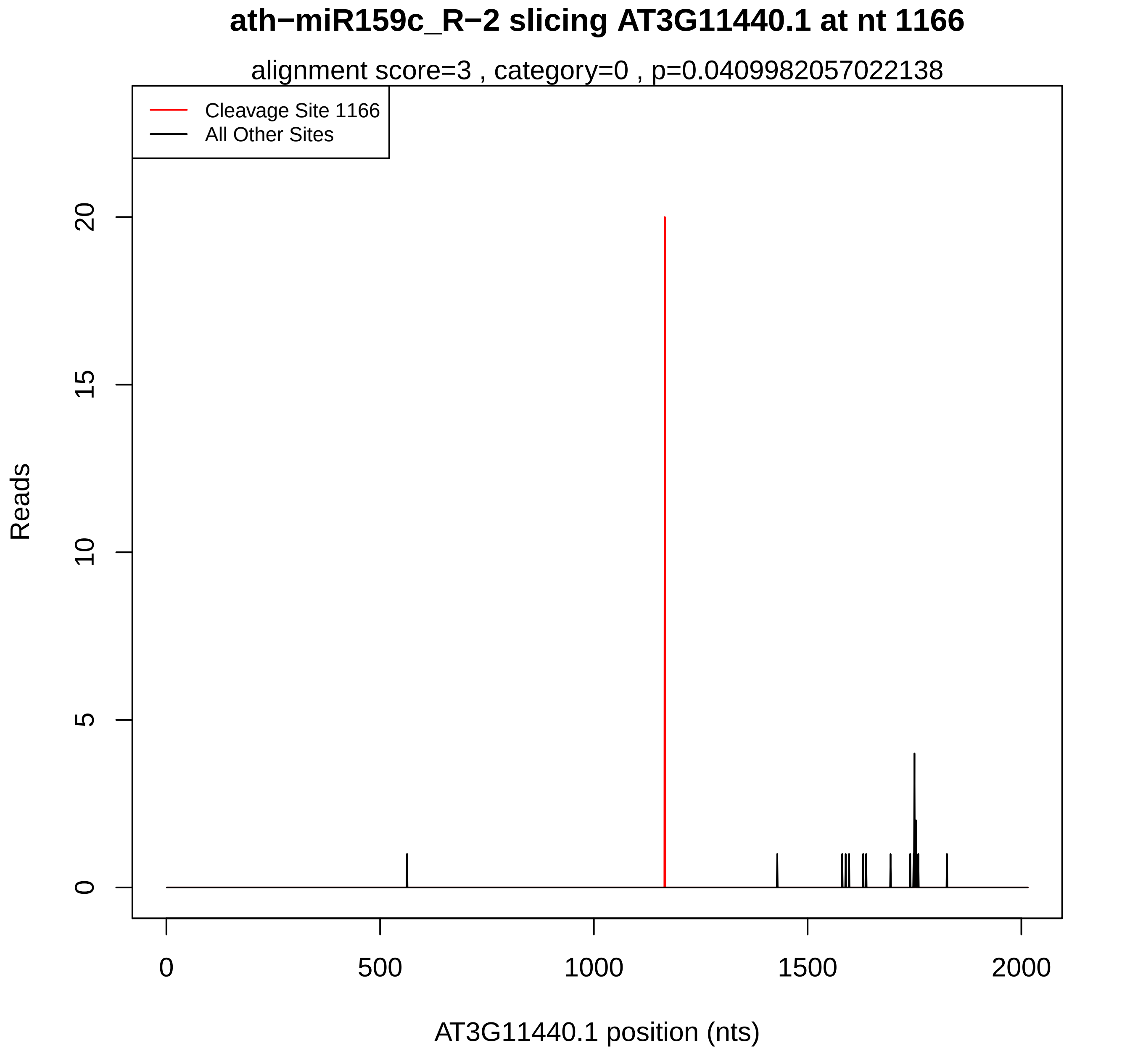
ath−miR159c_R−2 slicing AT3G11440.1 at nt 1166
alignment score=3 , category=0 , p=0.0409982057022138
Cleavage Site 1166
All Other Sites
Reads
0
5
10
15
20
0
500
1000
1500
2000
AT3G11440.1 position (nts)

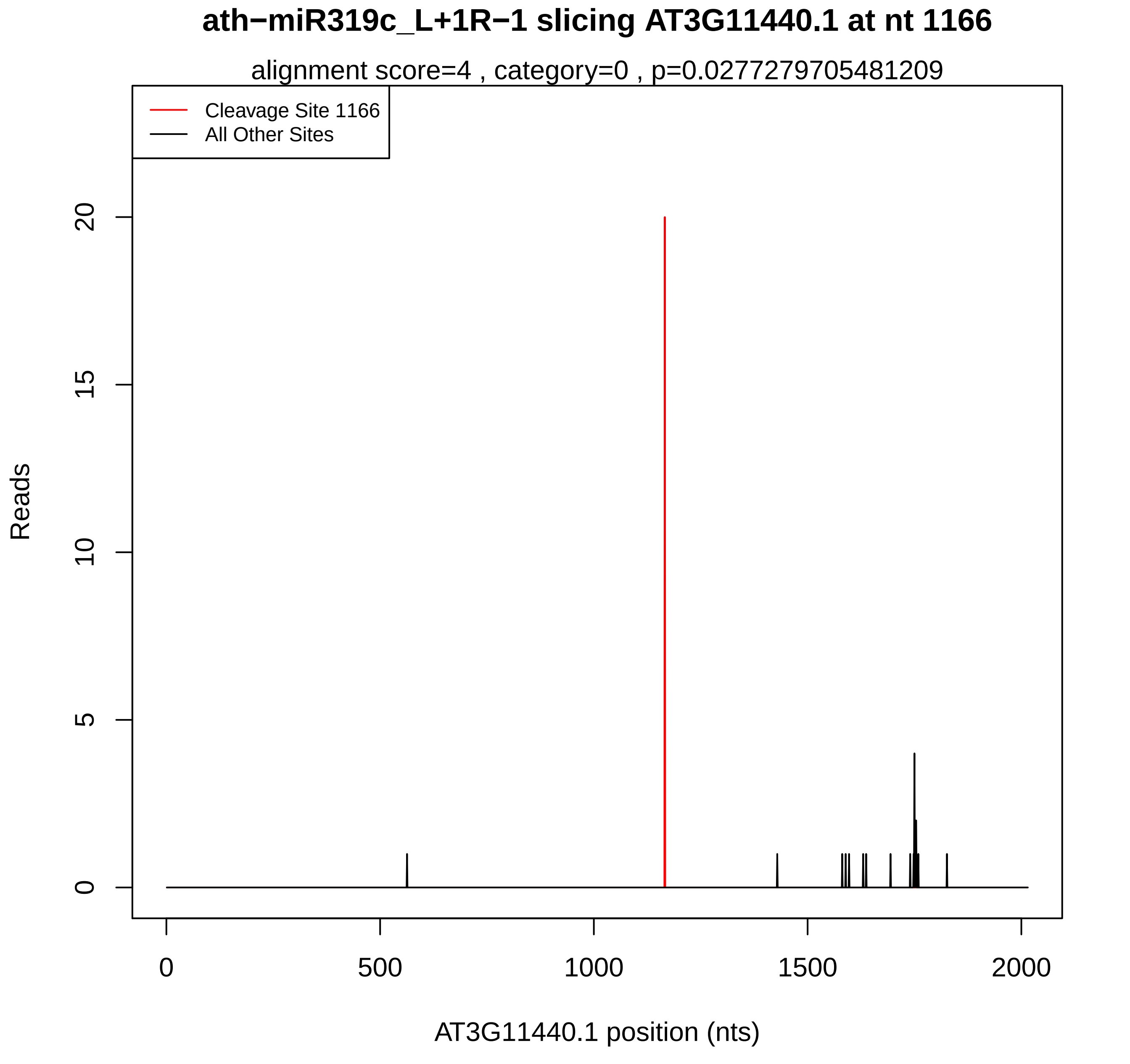

ath−miR319c_L+1R−1 slicing AT3G11440.1 at nt 1166
alignment score=4 , category=0 , p=0.0277279705481209
Cleavage Site 1166
All Other Sites
Reads
0
5
10
15
20
0
500
1000
1500
2000
AT3G11440.1 position (nts)

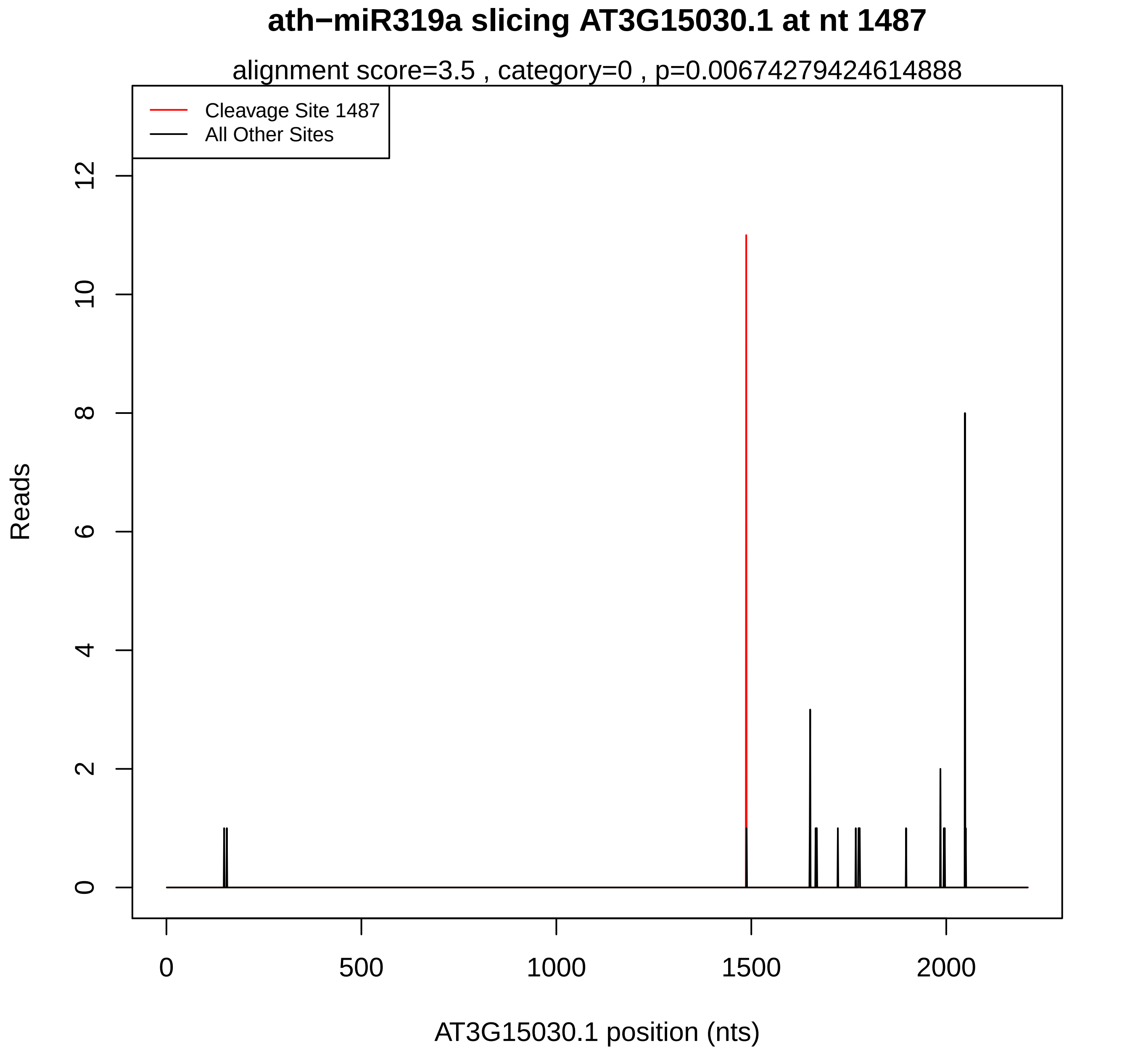
ath−miR319a slicing AT3G15030.1 at nt 1487
alignment score=3.5 , category=0 , p=0.00674279424614888
Cleavage Site 1487
All Other Sites
Reads
0
2
4
6
8
10
12
0
500
1000
1500
2000
AT3G15030.1 position (nts)

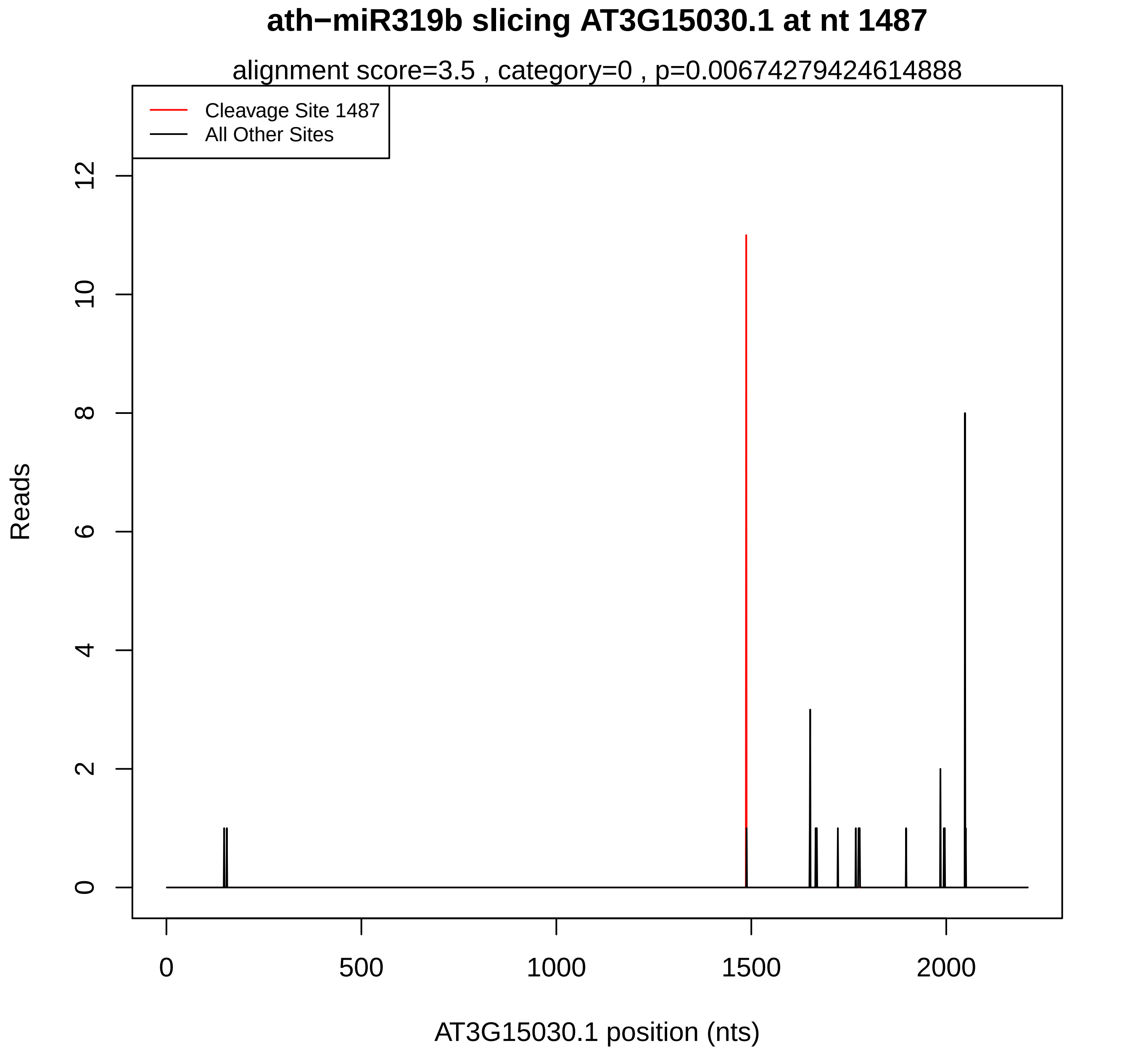

ath−miR319b slicing AT3G15030.1 at nt 1487
alignment score=3.5 , category=0 , p=0.00674279424614888
Cleavage Site 1487
All Other Sites
Reads
0
2
4
6
8
10
12
0
500
1000
1500
2000
AT3G15030.1 position (nts)

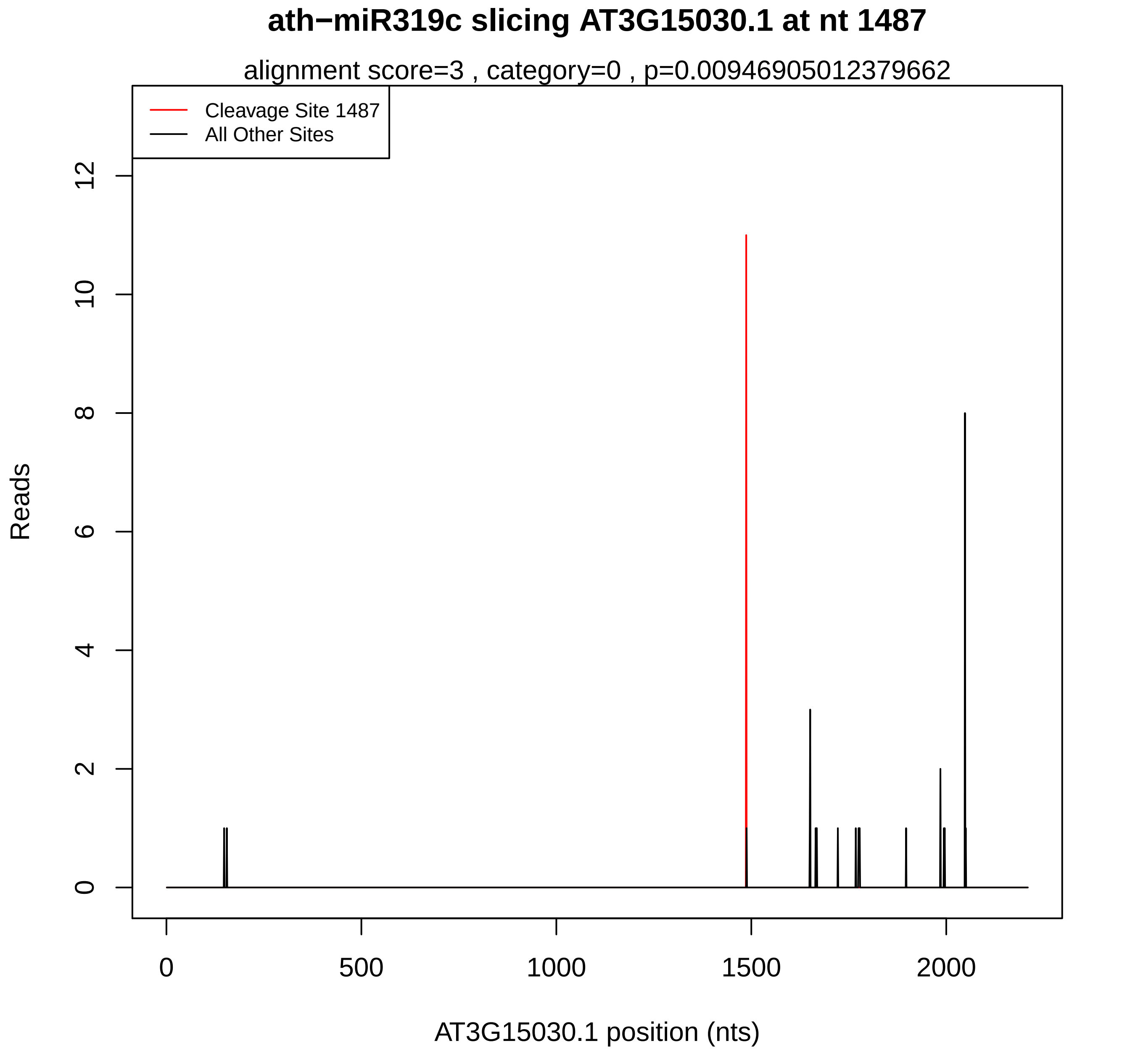
ath−miR319c slicing AT3G15030.1 at nt 1487
alignment score=3 , category=0 , p=0.00946905012379662
Cleavage Site 1487
All Other Sites
Reads
0
2
4
6
8
10
12
0
500
1000
1500
2000
AT3G15030.1 position (nts)

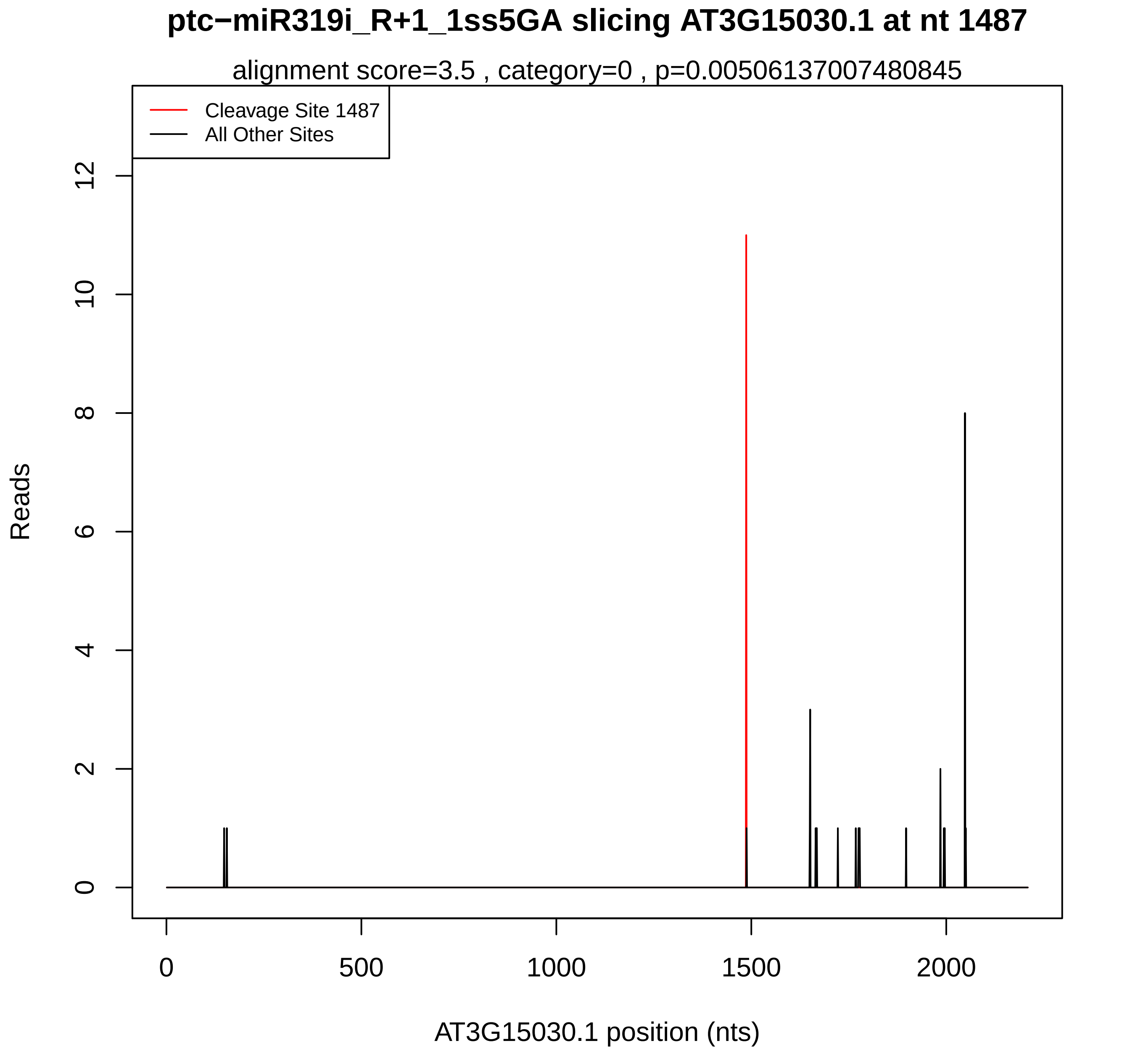

ptc−miR319i_R+1_1ss5GA slicing AT3G15030.1 at nt 1487
alignment score=3.5 , category=0 , p=0.00506137007480845
Cleavage Site 1487
All Other Sites
Reads
0
2
4
6
8
10
12
0
500
1000
1500
2000
AT3G15030.1 position (nts)

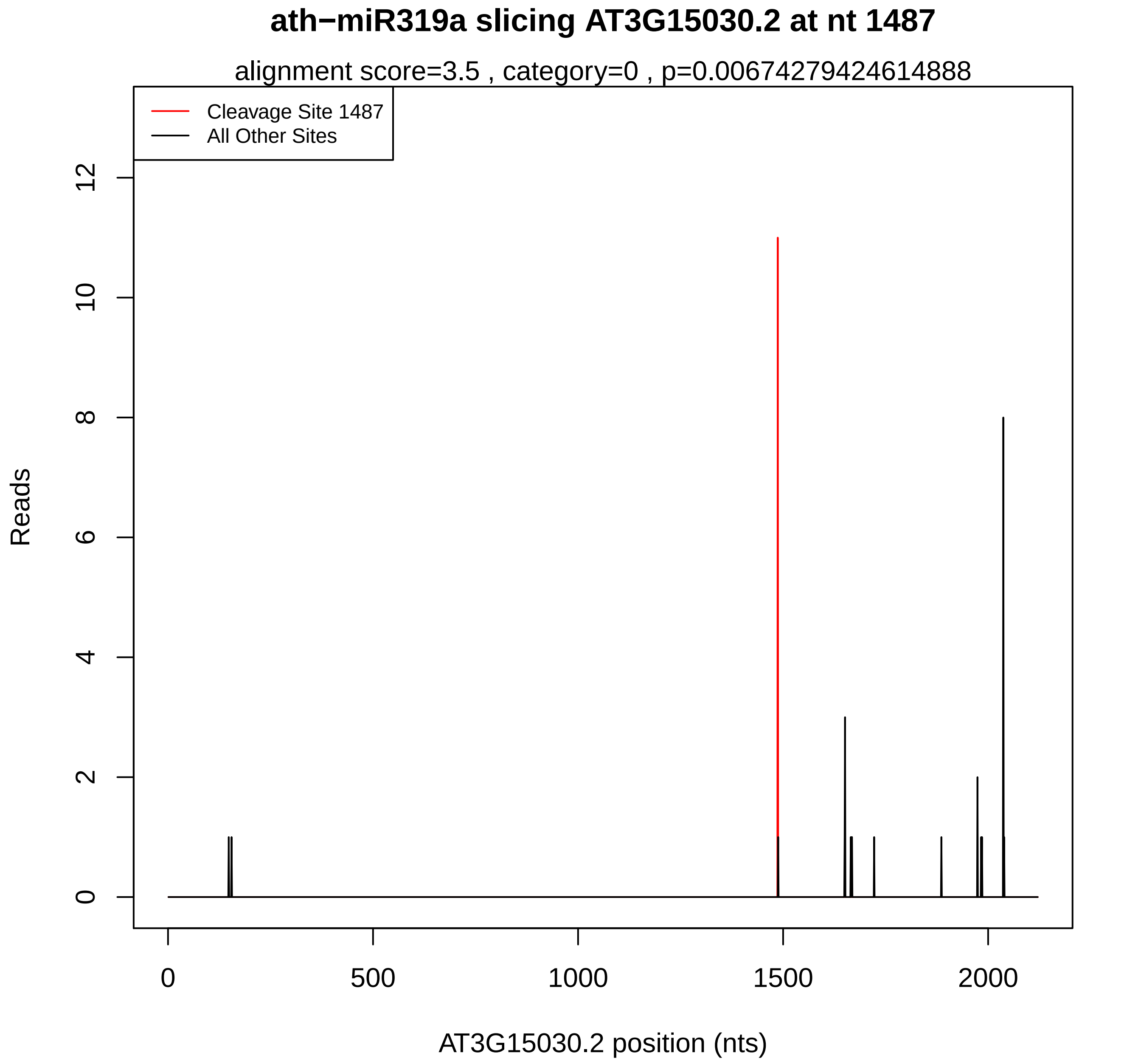

ath−miR319a slicing AT3G15030.2 at nt 1487
alignment score=3.5 , category=0 , p=0.00674279424614888
Cleavage Site 1487
All Other Sites
Reads
0
2
4
6
8
10
12
0
500
1000
1500
2000
AT3G15030.2 position (nts)

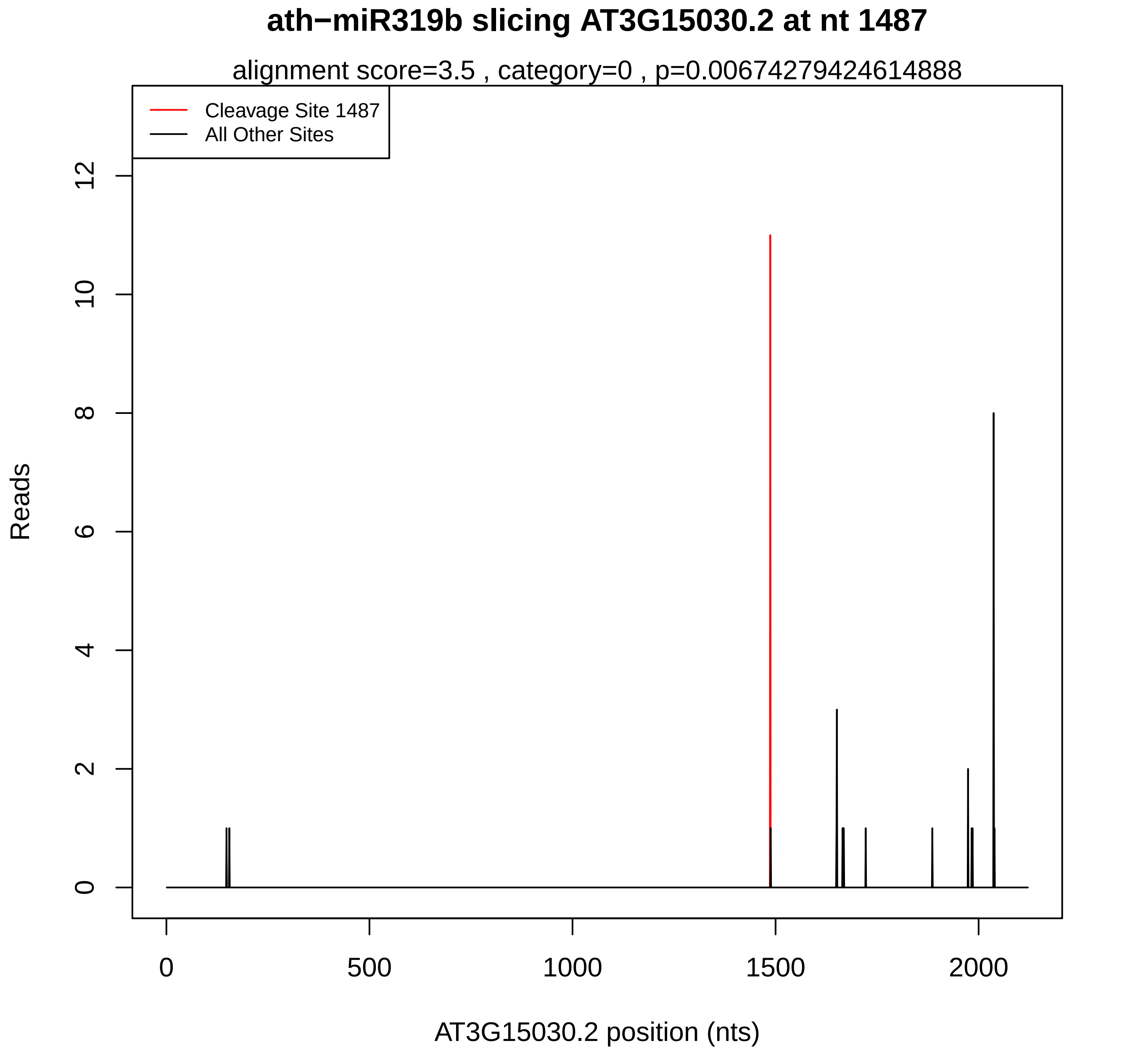

ath−miR319b slicing AT3G15030.2 at nt 1487
alignment score=3.5 , category=0 , p=0.00674279424614888
Cleavage Site 1487
All Other Sites
Reads
0
2
4
6
8
10
12
0
500
1000
1500
2000
AT3G15030.2 position (nts)

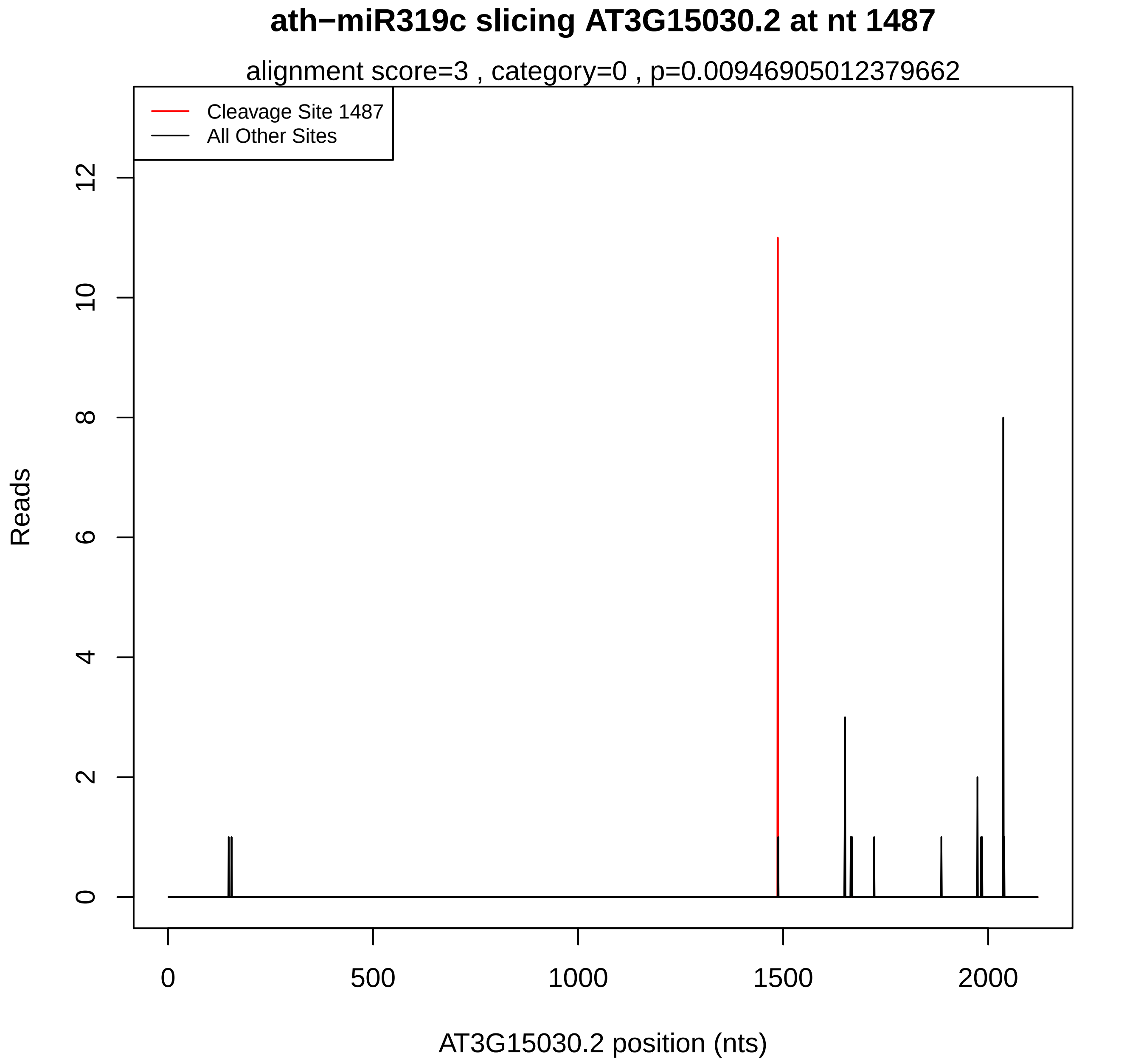

ath−miR319c slicing AT3G15030.2 at nt 1487
alignment score=3 , category=0 , p=0.00946905012379662
Cleavage Site 1487
All Other Sites
Reads
0
2
4
6
8
10
12
0
500
1000
1500
2000
AT3G15030.2 position (nts)

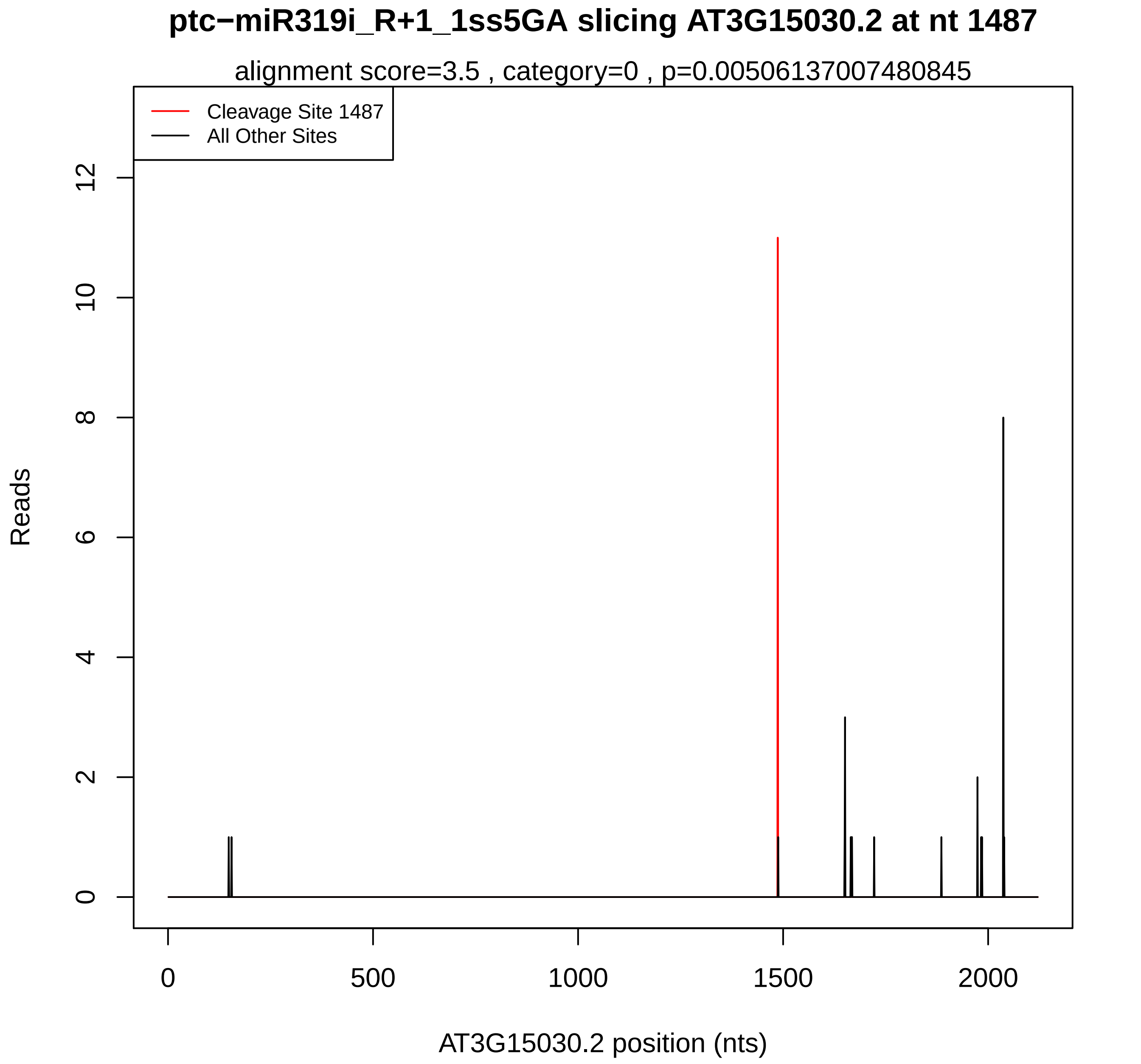

ptc−miR319i_R+1_1ss5GA slicing AT3G15030.2 at nt 1487
alignment score=3.5 , category=0 , p=0.00506137007480845
Cleavage Site 1487
All Other Sites
Reads
0
2
4
6
8
10
12
0
500
1000
1500
2000
AT3G15030.2 position (nts)

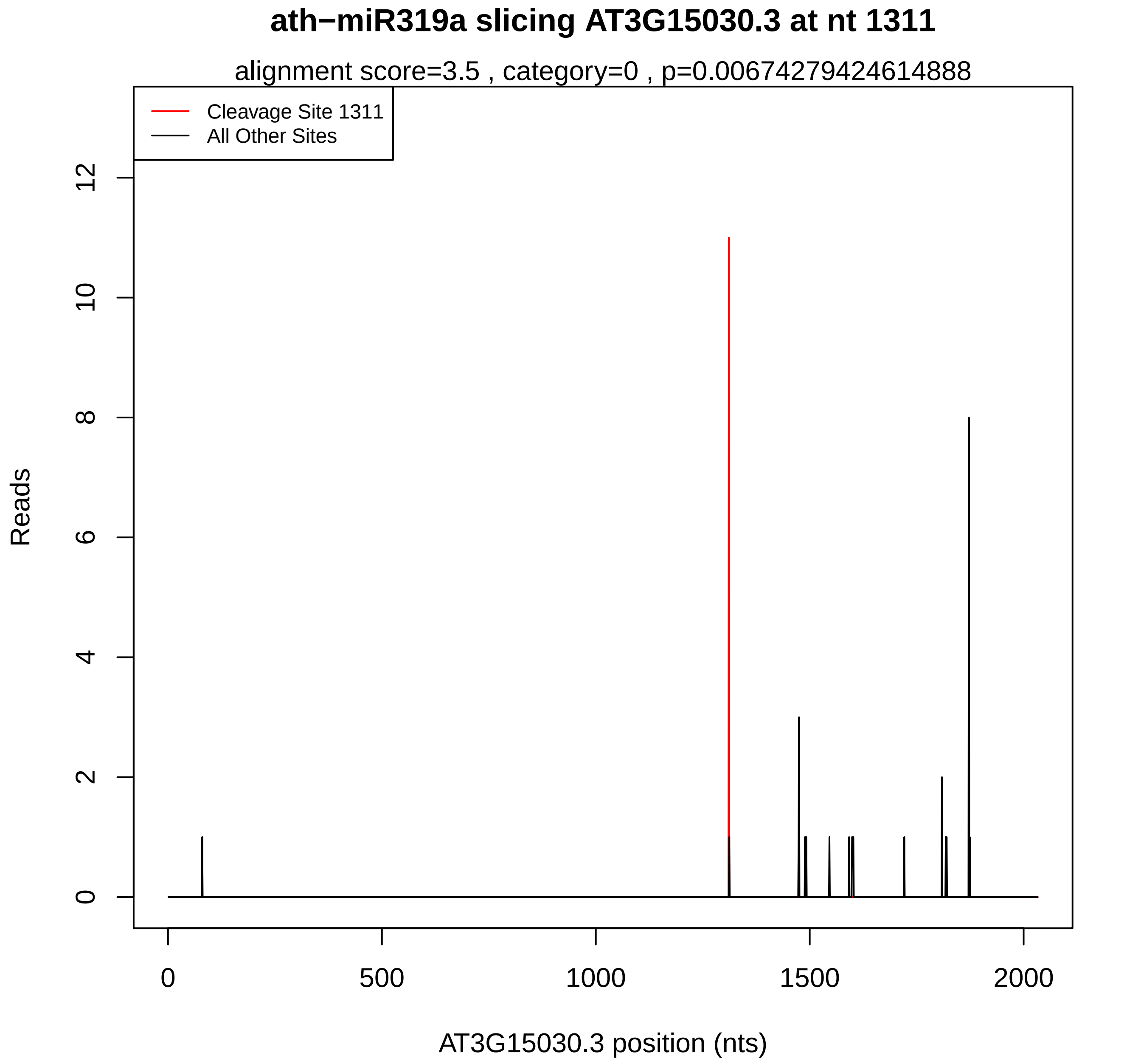

ath−miR319a slicing AT3G15030.3 at nt 1311
alignment score=3.5 , category=0 , p=0.00674279424614888
Cleavage Site 1311
All Other Sites
Reads
0
2
4
6
8
10
12
0
500
1000
1500
2000
AT3G15030.3 position (nts)

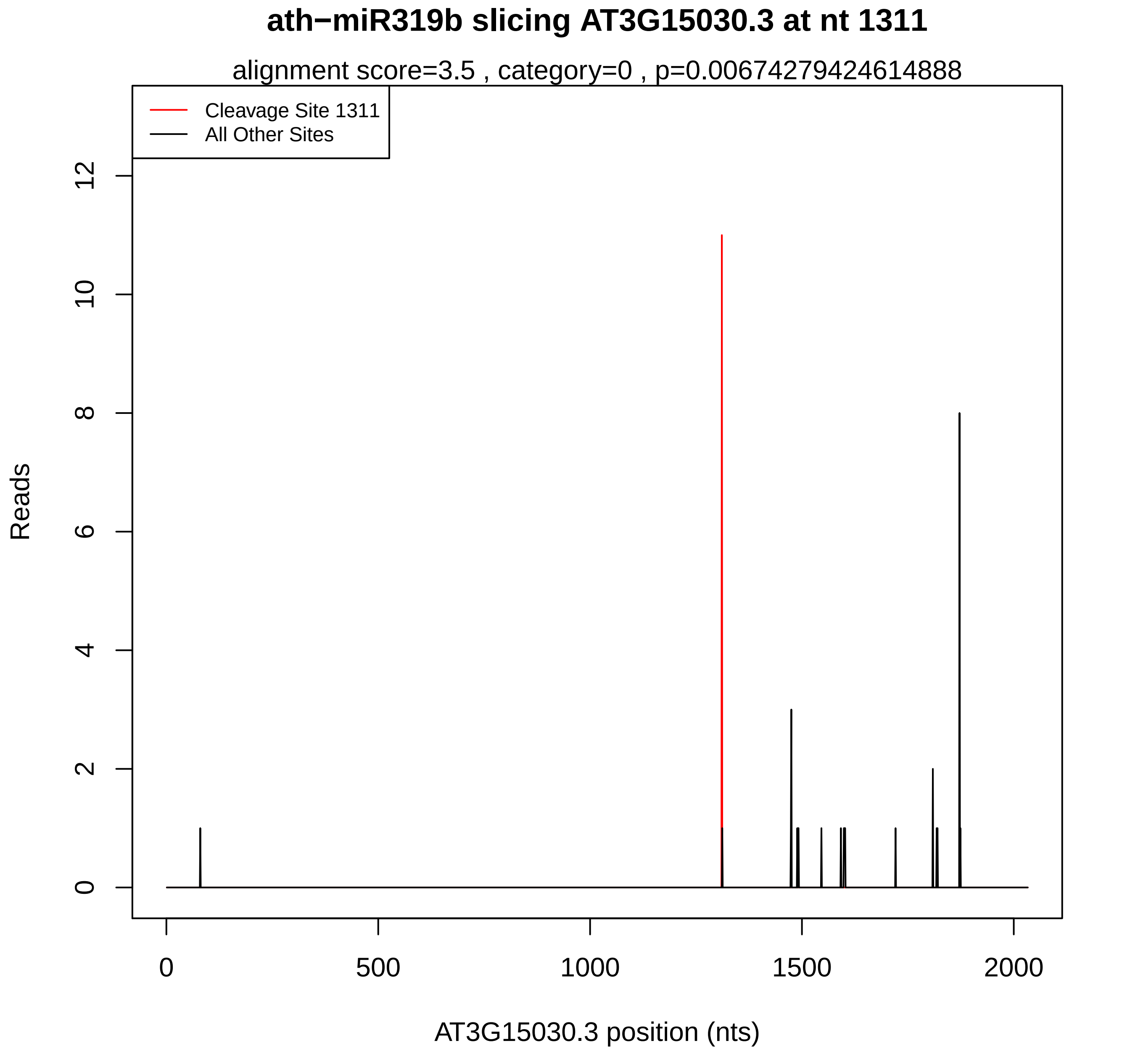

ath−miR319b slicing AT3G15030.3 at nt 1311
alignment score=3.5 , category=0 , p=0.00674279424614888
Cleavage Site 1311
All Other Sites
Reads
0
2
4
6
8
10
12
0
500
1000
1500
2000
AT3G15030.3 position (nts)

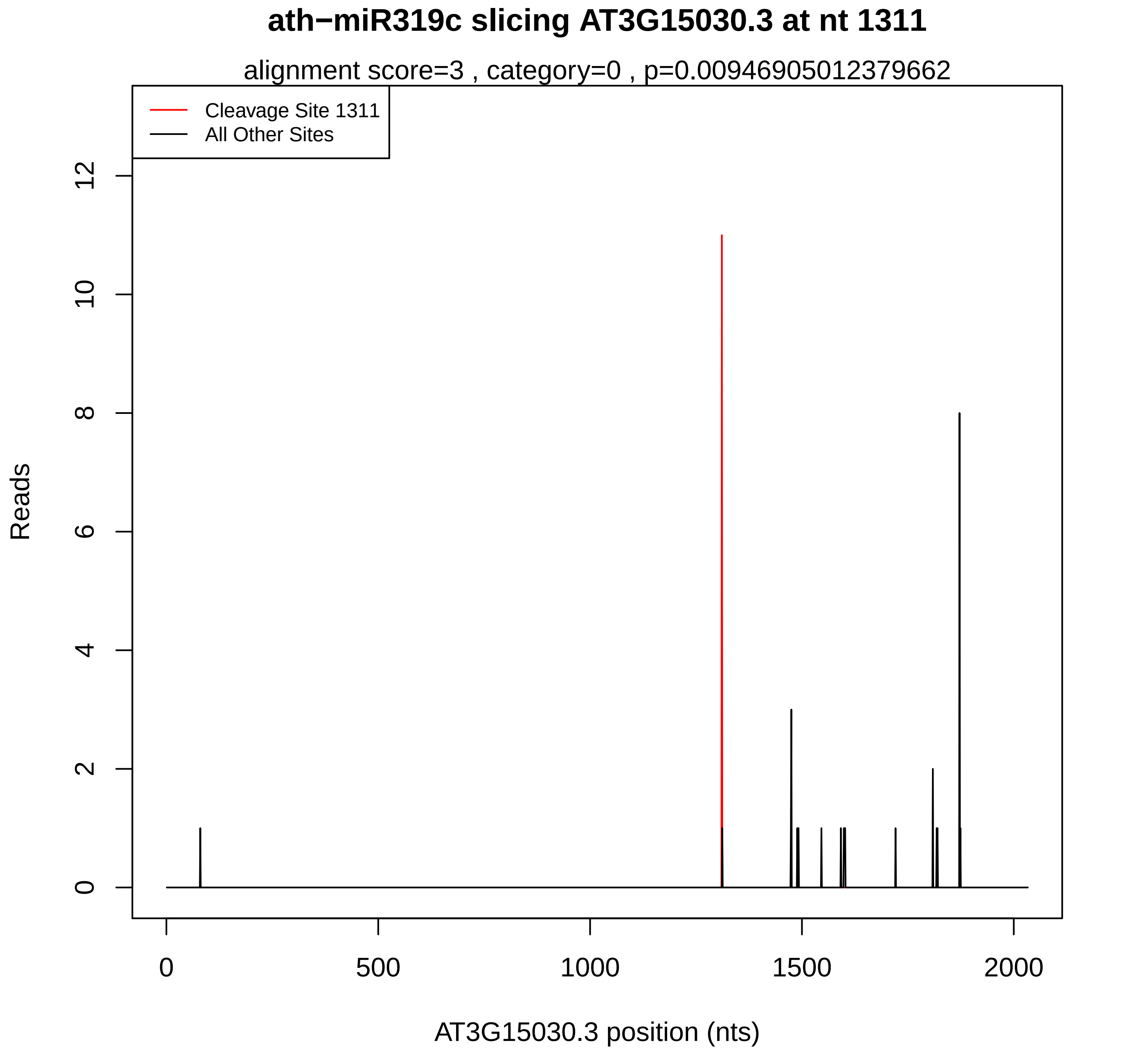

ath−miR319c slicing AT3G15030.3 at nt 1311
alignment score=3 , category=0 , p=0.00946905012379662
Cleavage Site 1311
All Other Sites
Reads
0
2
4
6
8
10
12
0
500
1000
1500
2000
AT3G15030.3 position (nts)

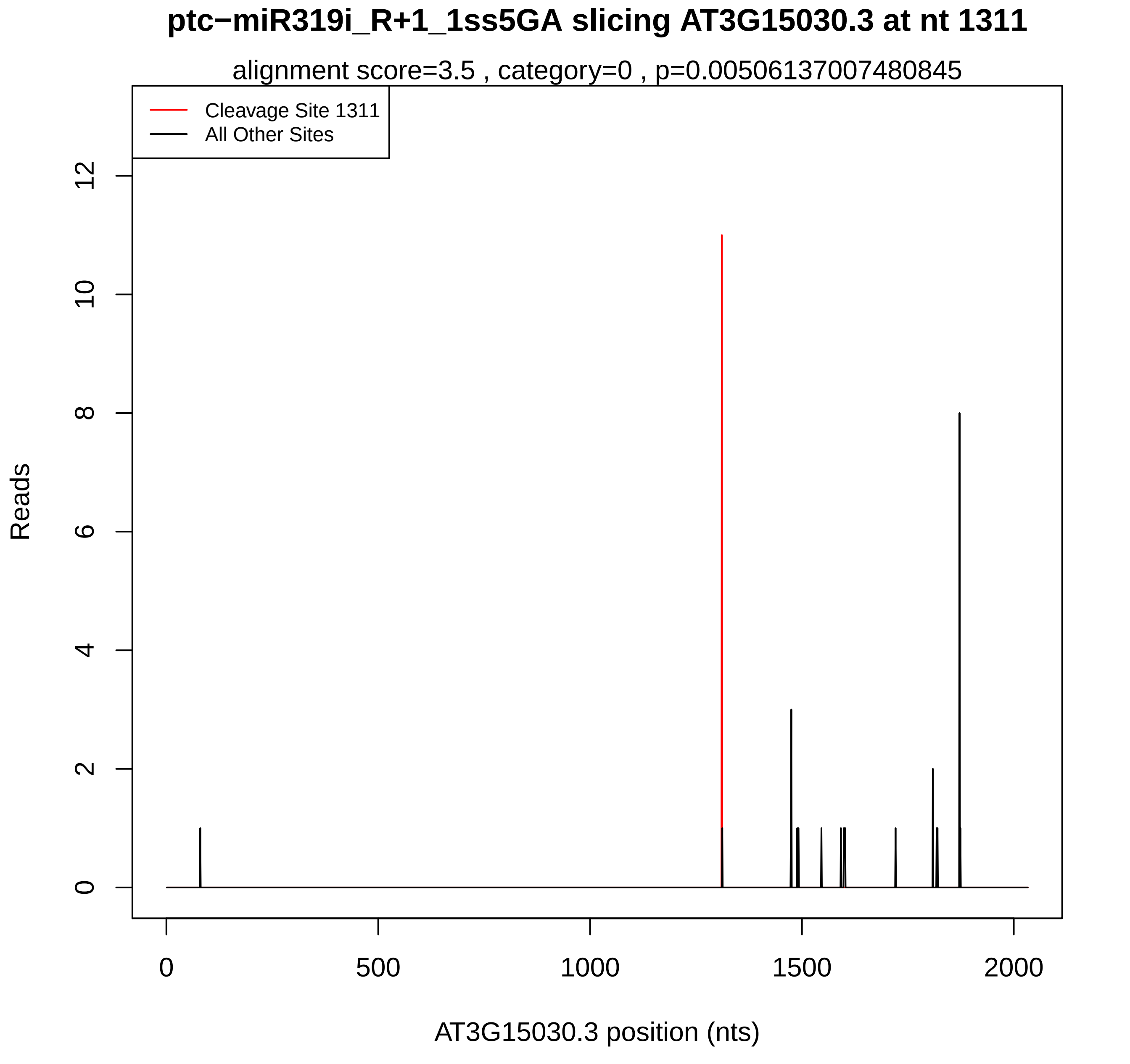
ptc−miR319i_R+1_1ss5GA slicing AT3G15030.3 at nt 1311
alignment score=3.5 , category=0 , p=0.00506137007480845
Cleavage Site 1311
All Other Sites
Reads
0
2
4
6
8
10
12
0
500
1000
1500
2000
AT3G15030.3 position (nts)

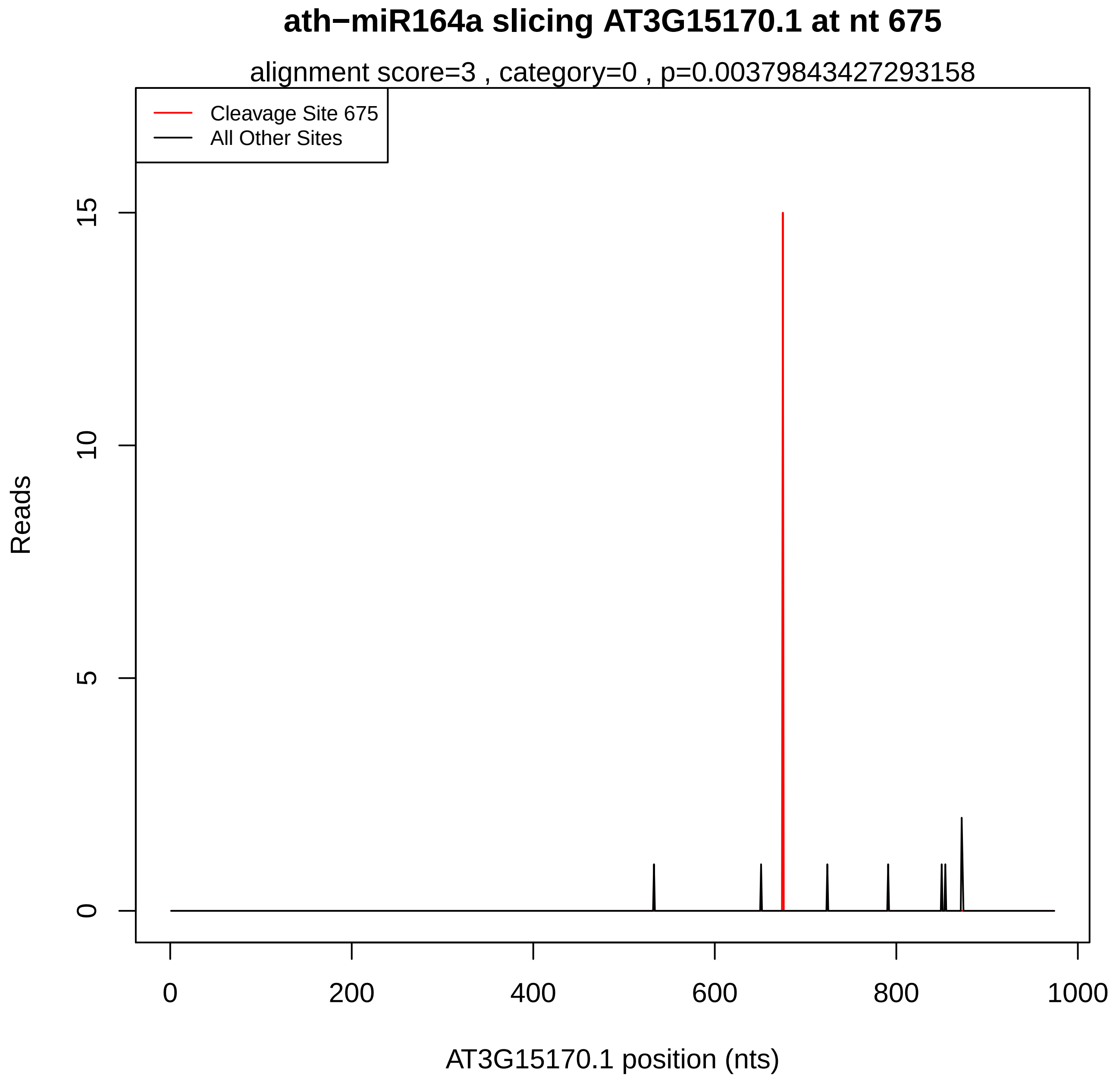
ath−miR164a slicing AT3G15170.1 at nt 675
alignment score=3 , category=0 , p=0.00379843427293158
Cleavage Site 675
All Other Sites
Reads
0
5
10
15
0
200
400
600
800
1000
AT3G15170.1 position (nts)

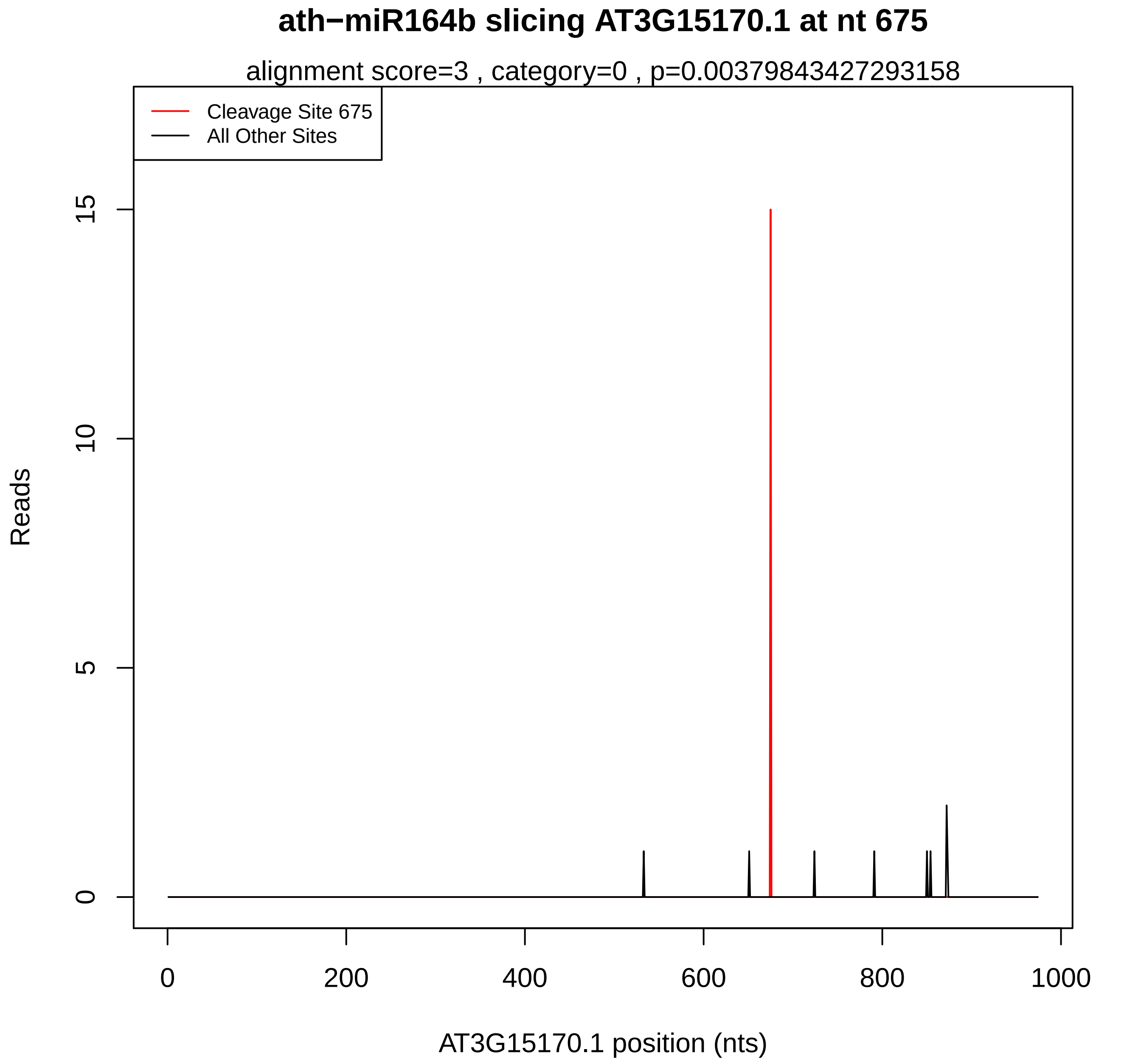

ath−miR164b slicing AT3G15170.1 at nt 675
alignment score=3 , category=0 , p=0.00379843427293158
Cleavage Site 675
All Other Sites
Reads
0
5
10
15
0
200
400
600
800
1000
AT3G15170.1 position (nts)

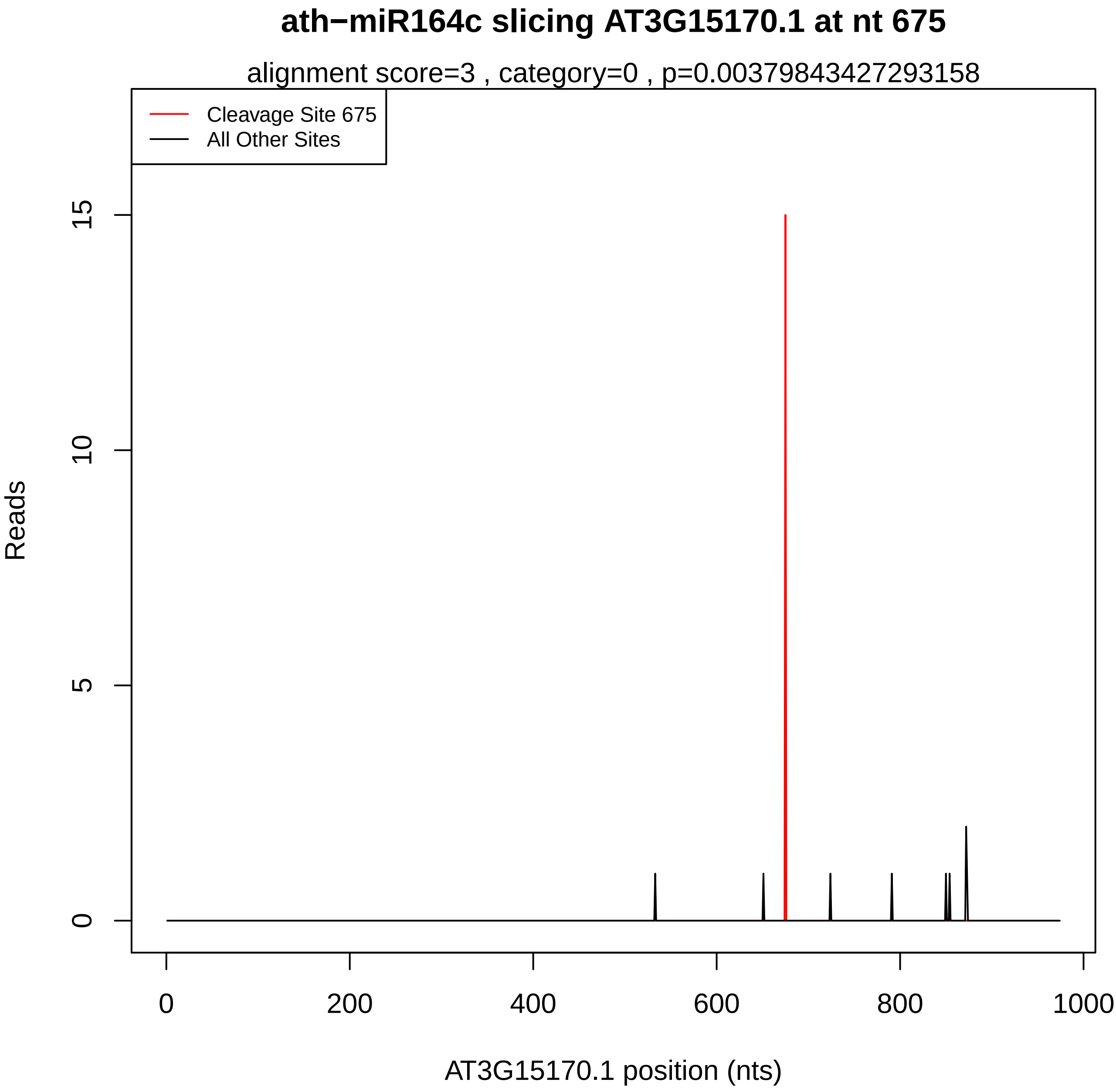

ath−miR164c slicing AT3G15170.1 at nt 675
alignment score=3 , category=0 , p=0.00379843427293158
Cleavage Site 675
All Other Sites
Reads
0
5
10
15
0
200
400
600
800
1000
AT3G15170.1 position (nts)

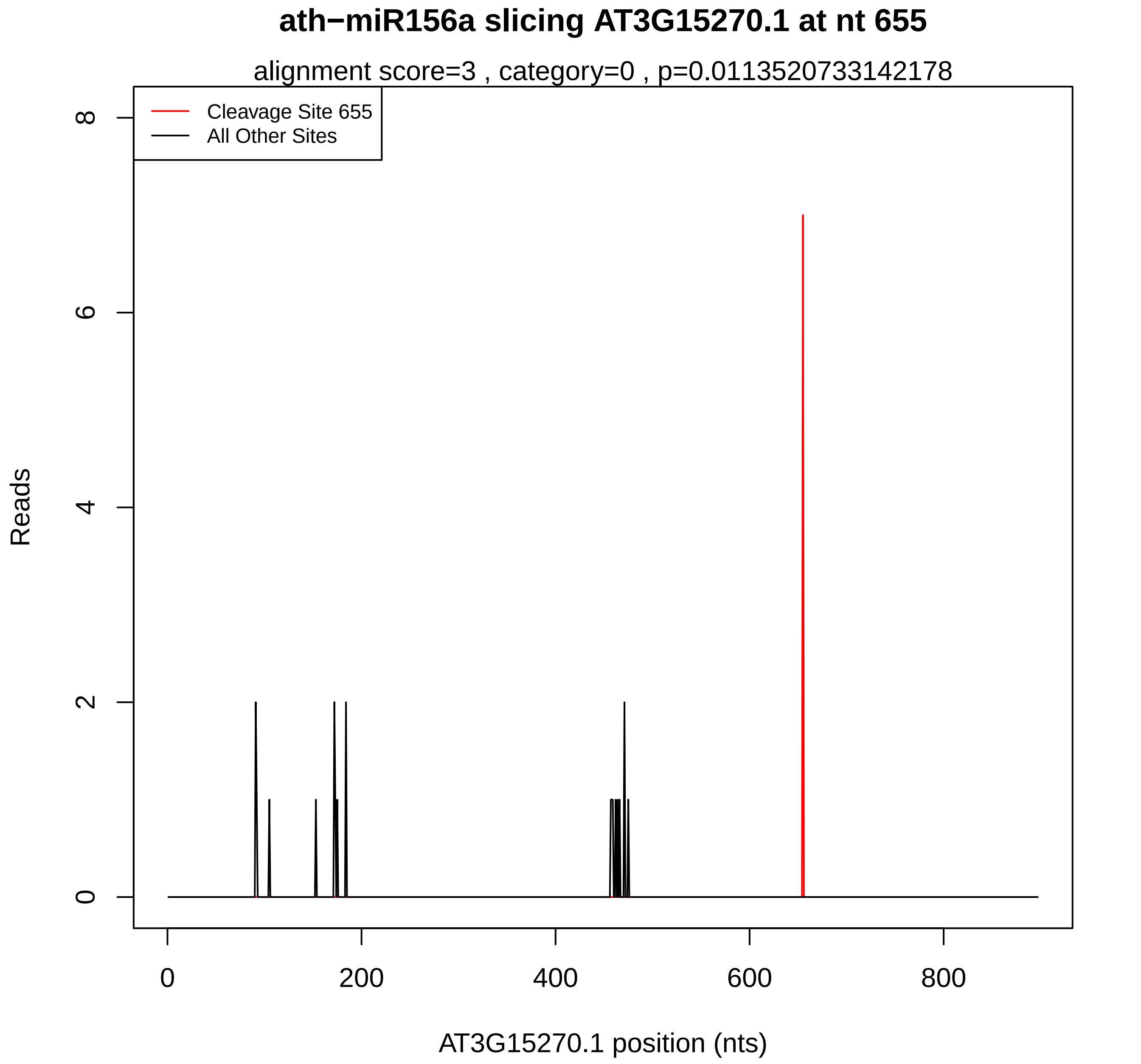
ath−miR156a slicing AT3G15270.1 at nt 655
alignment score=3 , category=0 , p=0.0113520733142178
Cleavage Site 655
All Other Sites
Reads
0
2
4
6
8
0
200
400
600
800
AT3G15270.1 position (nts)

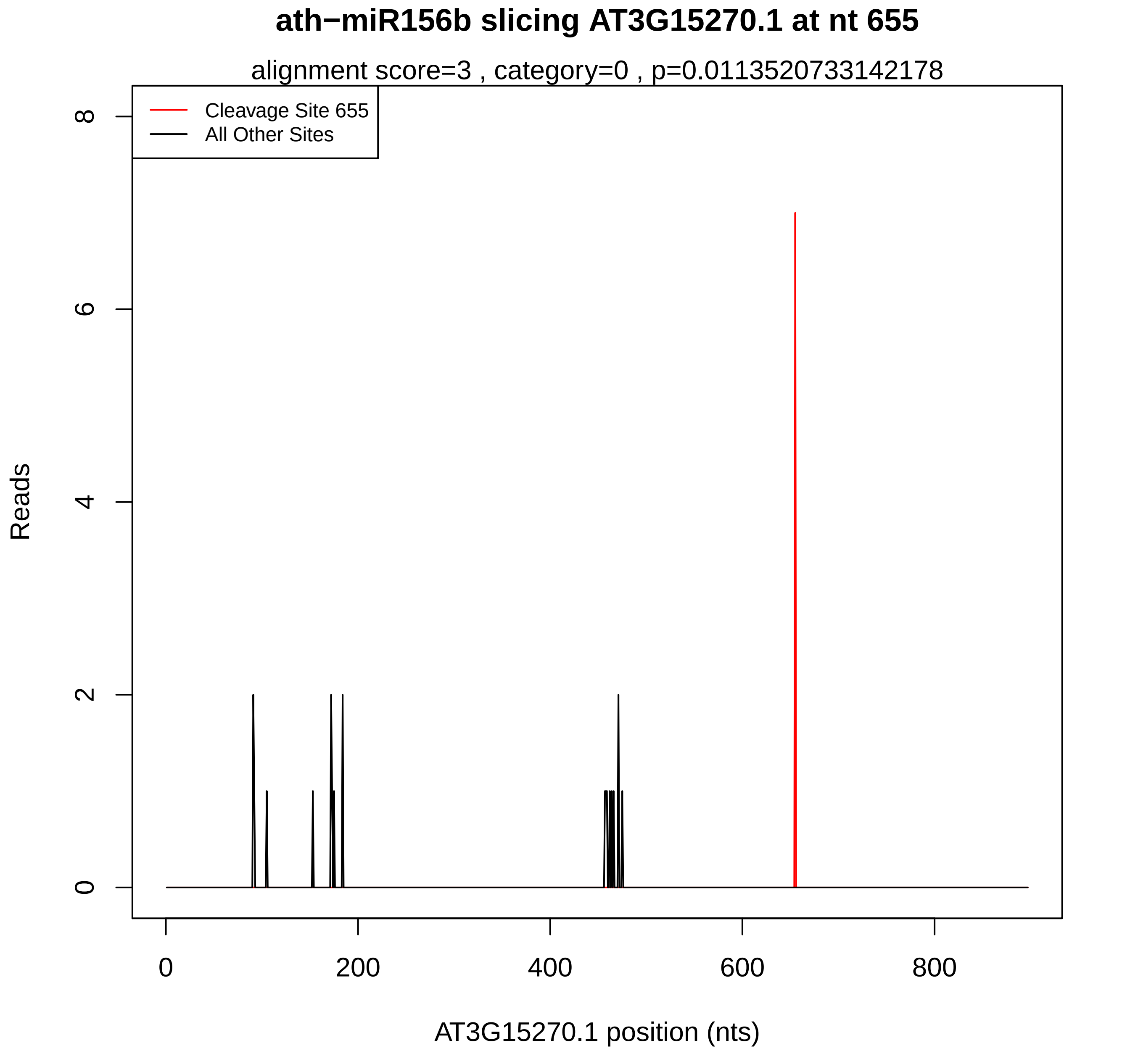
ath−miR156b slicing AT3G15270.1 at nt 655
alignment score=3 , category=0 , p=0.0113520733142178
Cleavage Site 655
All Other Sites
Reads
0
2
4
6
8
0
200
400
600
800
AT3G15270.1 position (nts)

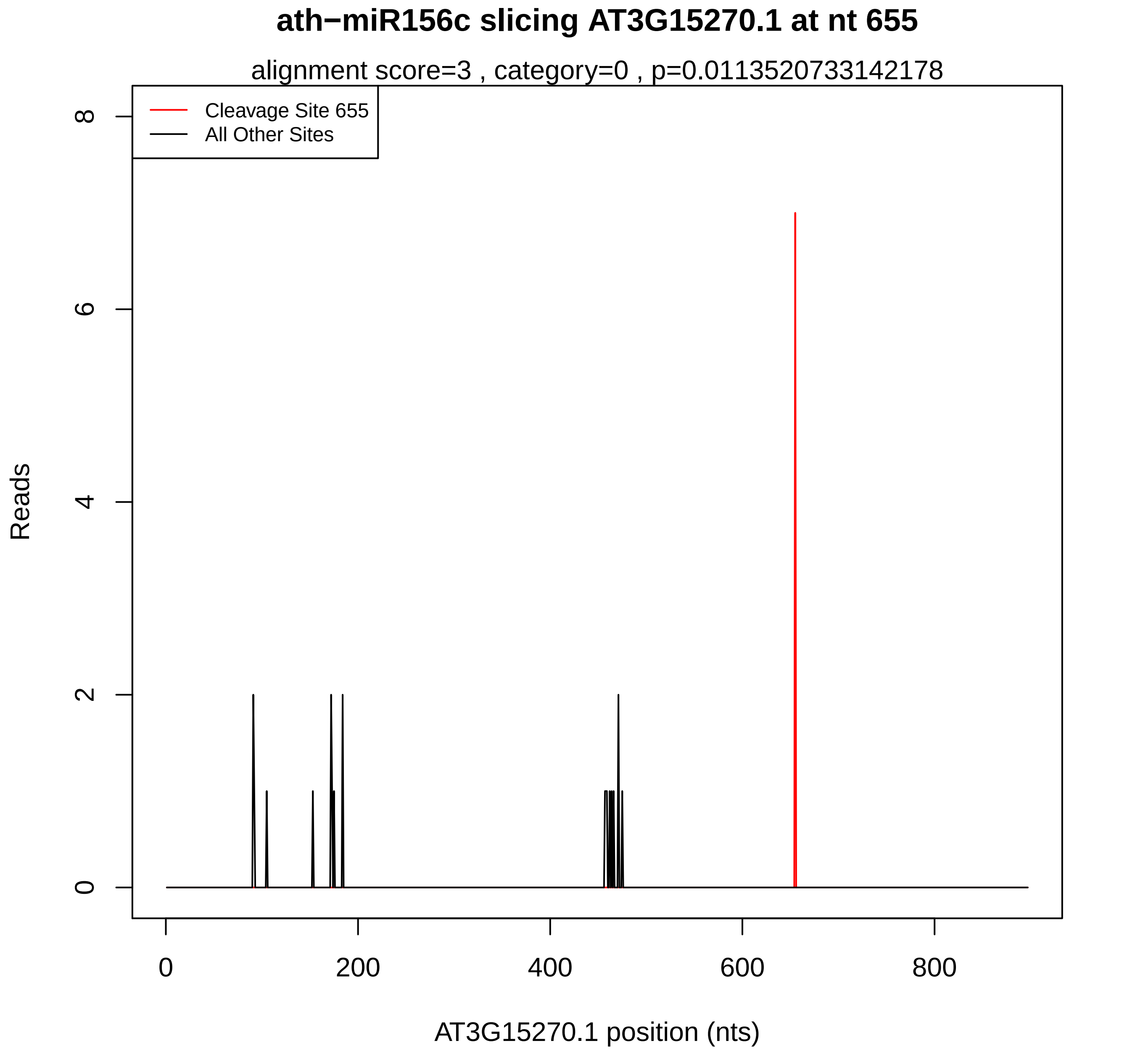
ath−miR156c slicing AT3G15270.1 at nt 655
alignment score=3 , category=0 , p=0.0113520733142178
Cleavage Site 655
All Other Sites
Reads
0
2
4
6
8
0
200
400
600
800
AT3G15270.1 position (nts)

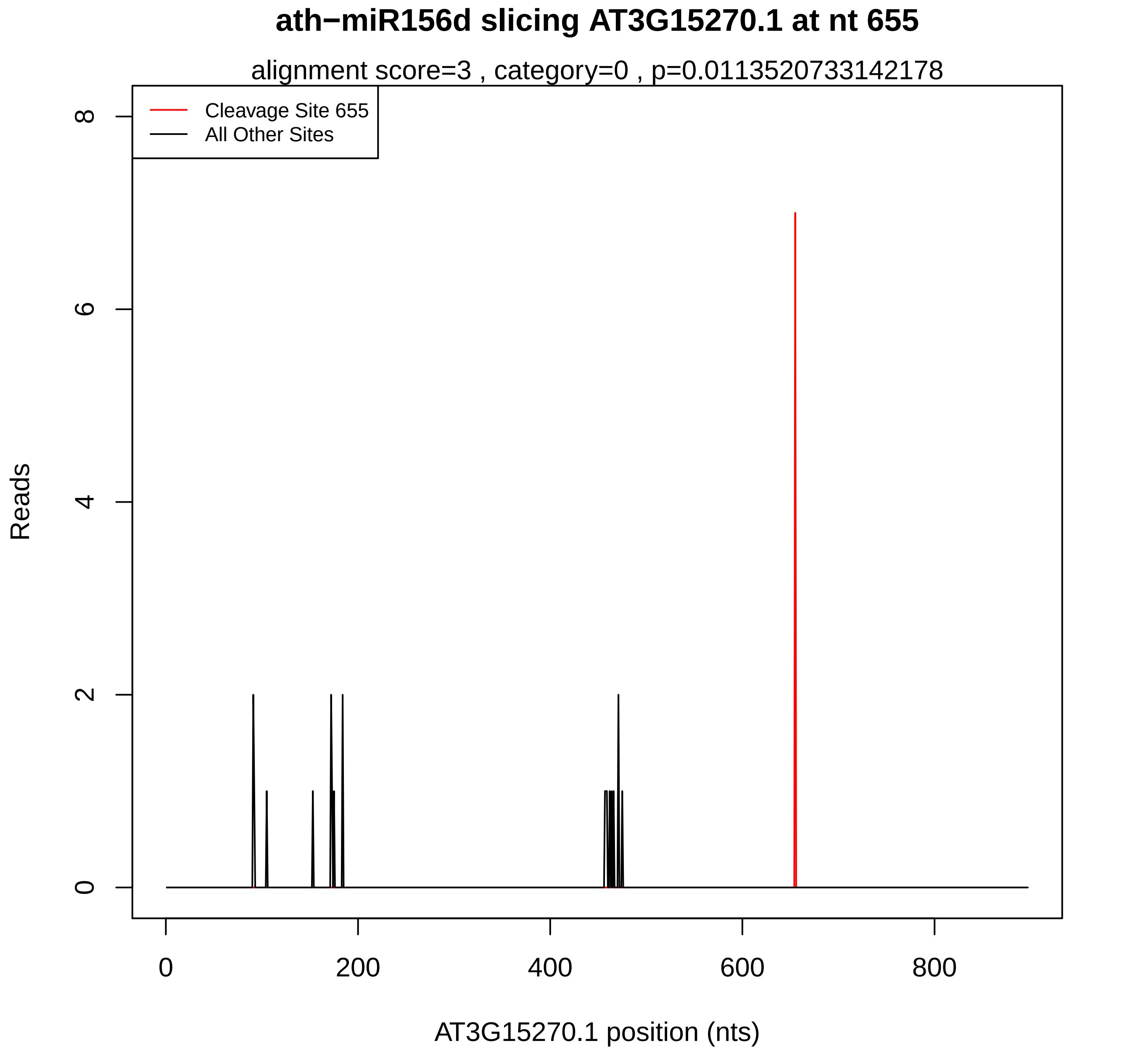
ath−miR156d slicing AT3G15270.1 at nt 655
alignment score=3 , category=0 , p=0.0113520733142178
Cleavage Site 655
All Other Sites
Reads
0
2
4
6
8
0
200
400
600
800
AT3G15270.1 position (nts)

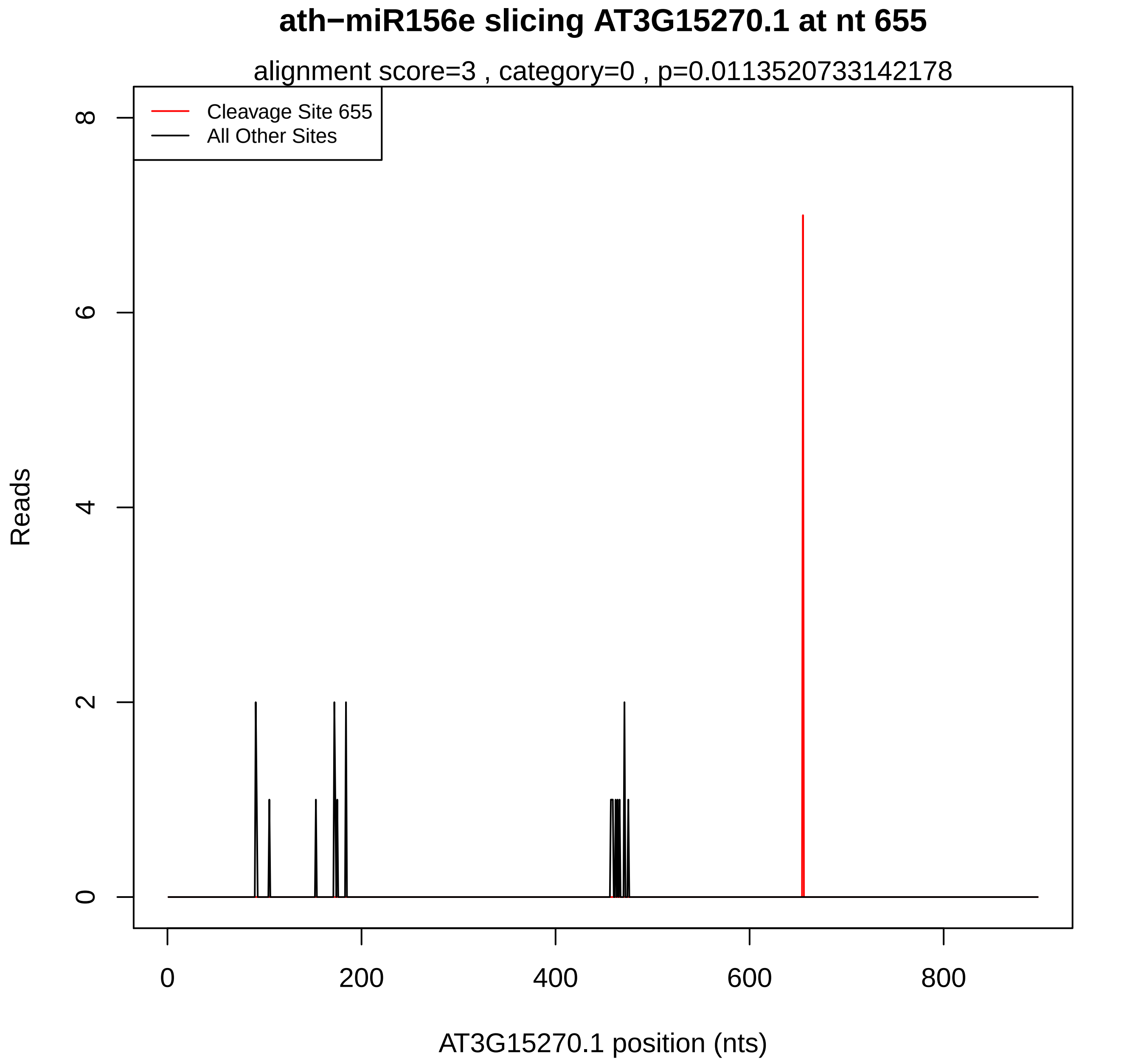

ath−miR156e slicing AT3G15270.1 at nt 655
alignment score=3 , category=0 , p=0.0113520733142178
Cleavage Site 655
All Other Sites
Reads
0
2
4
6
8
0
200
400
600
800
AT3G15270.1 position (nts)

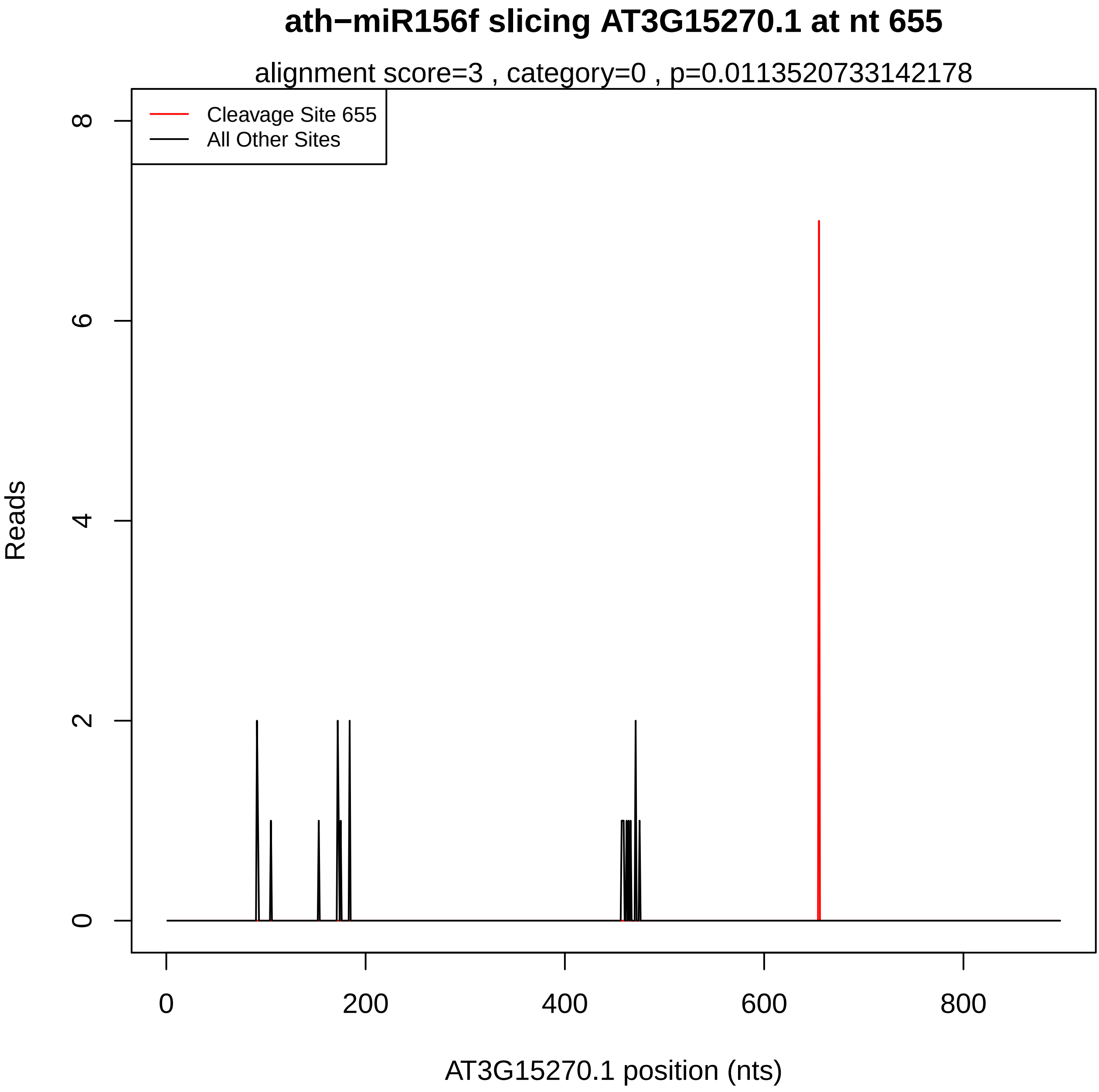
ath−miR156f slicing AT3G15270.1 at nt 655
alignment score=3 , category=0 , p=0.0113520733142178
Cleavage Site 655
All Other Sites
Reads
0
2
4
6
8
0
200
400
600
800
AT3G15270.1 position (nts)

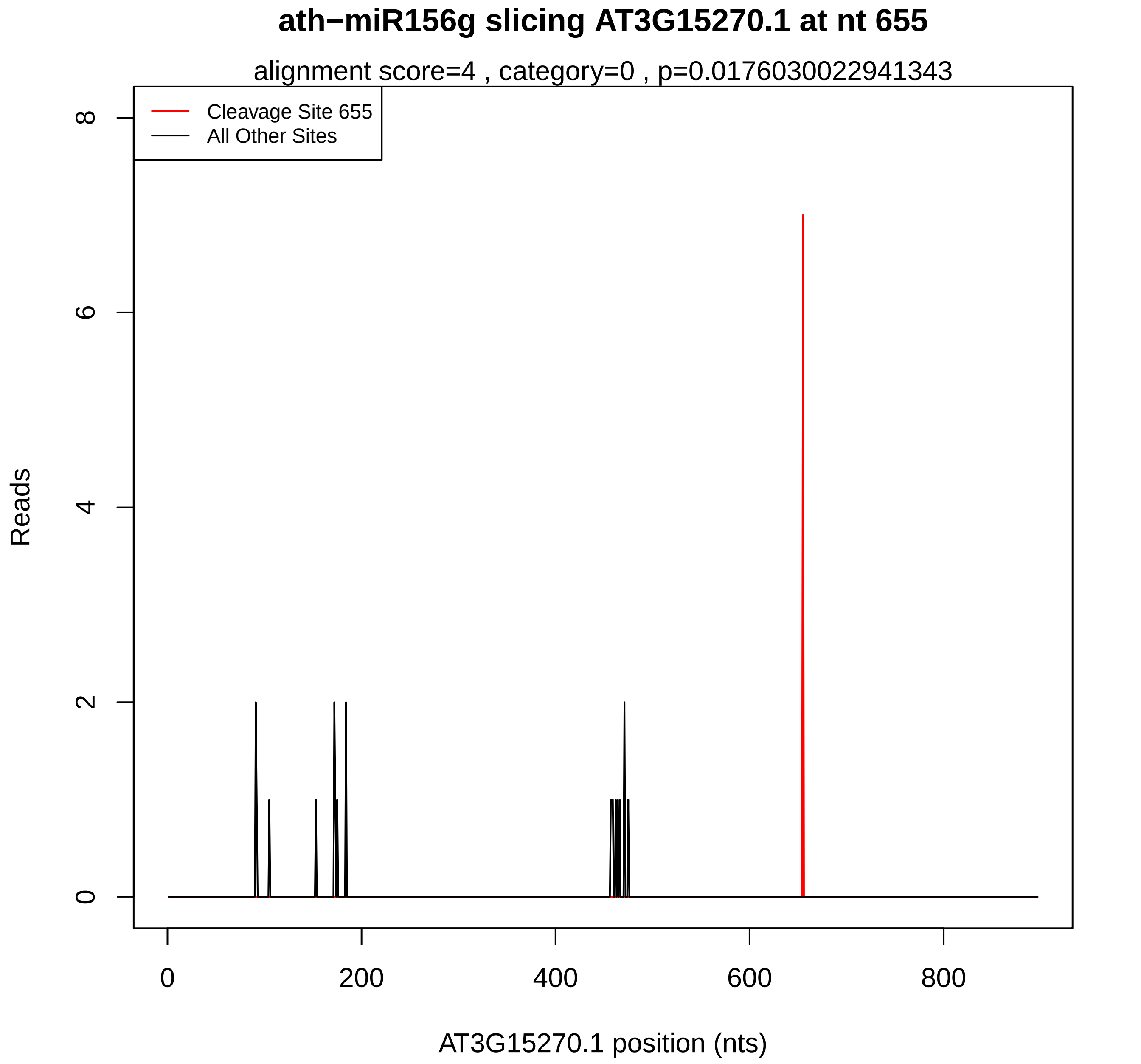
ath−miR156g slicing AT3G15270.1 at nt 655
alignment score=4 , category=0 , p=0.0176030022941343
Cleavage Site 655
All Other Sites
Reads
0
2
4
6
8
0
200
400
600
800
AT3G15270.1 position (nts)

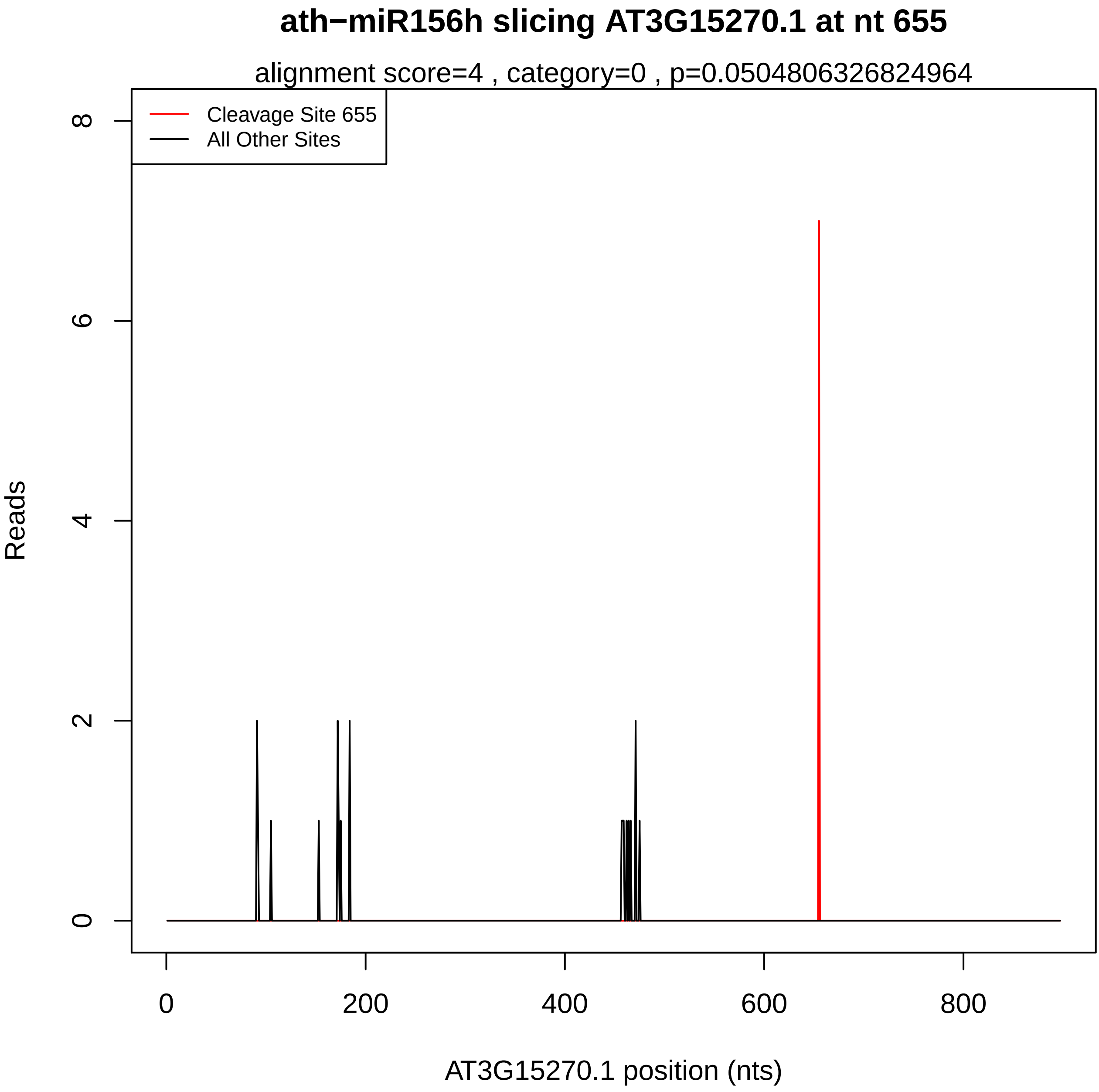

ath−miR156h slicing AT3G15270.1 at nt 655
alignment score=4 , category=0 , p=0.0504806326824964
Cleavage Site 655
All Other Sites
Reads
0
2
4
6
8
0
200
400
600
800
AT3G15270.1 position (nts)

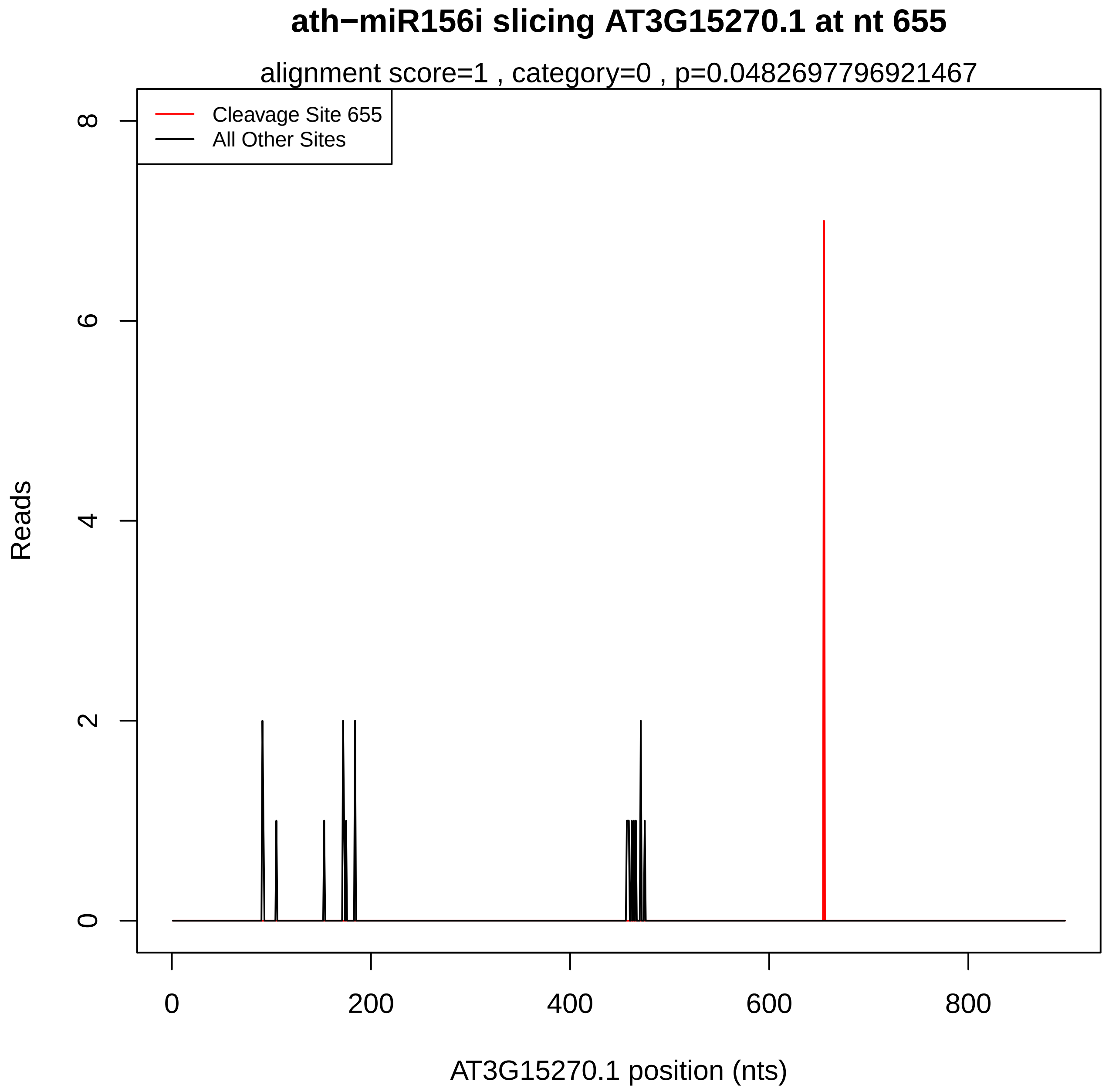

ath−miR156i slicing AT3G15270.1 at nt 655
alignment score=1 , category=0 , p=0.0482697796921467
Cleavage Site 655
All Other Sites
Reads
0
2
4
6
8
0
200
400
600
800
AT3G15270.1 position (nts)

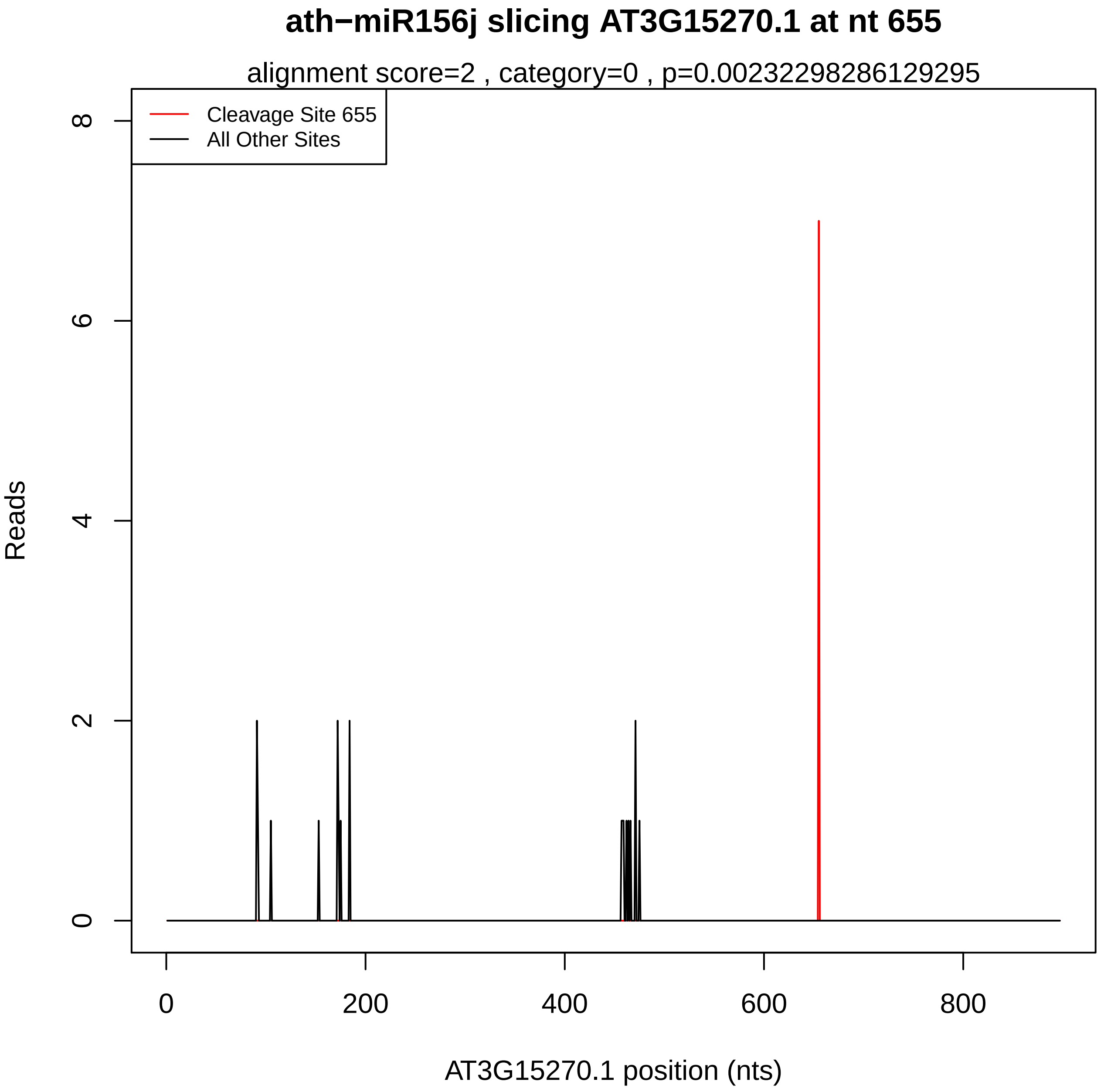
ath−miR156j slicing AT3G15270.1 at nt 655
alignment score=2 , category=0 , p=0.00232298286129295
Cleavage Site 655
All Other Sites
Reads
0
2
4
6
8
0
200
400
600
800
AT3G15270.1 position (nts)

ath−miR157d slicing AT3G15270.1 at nt 655

alignment score=4 , category=0 , p=0.0348961388985012

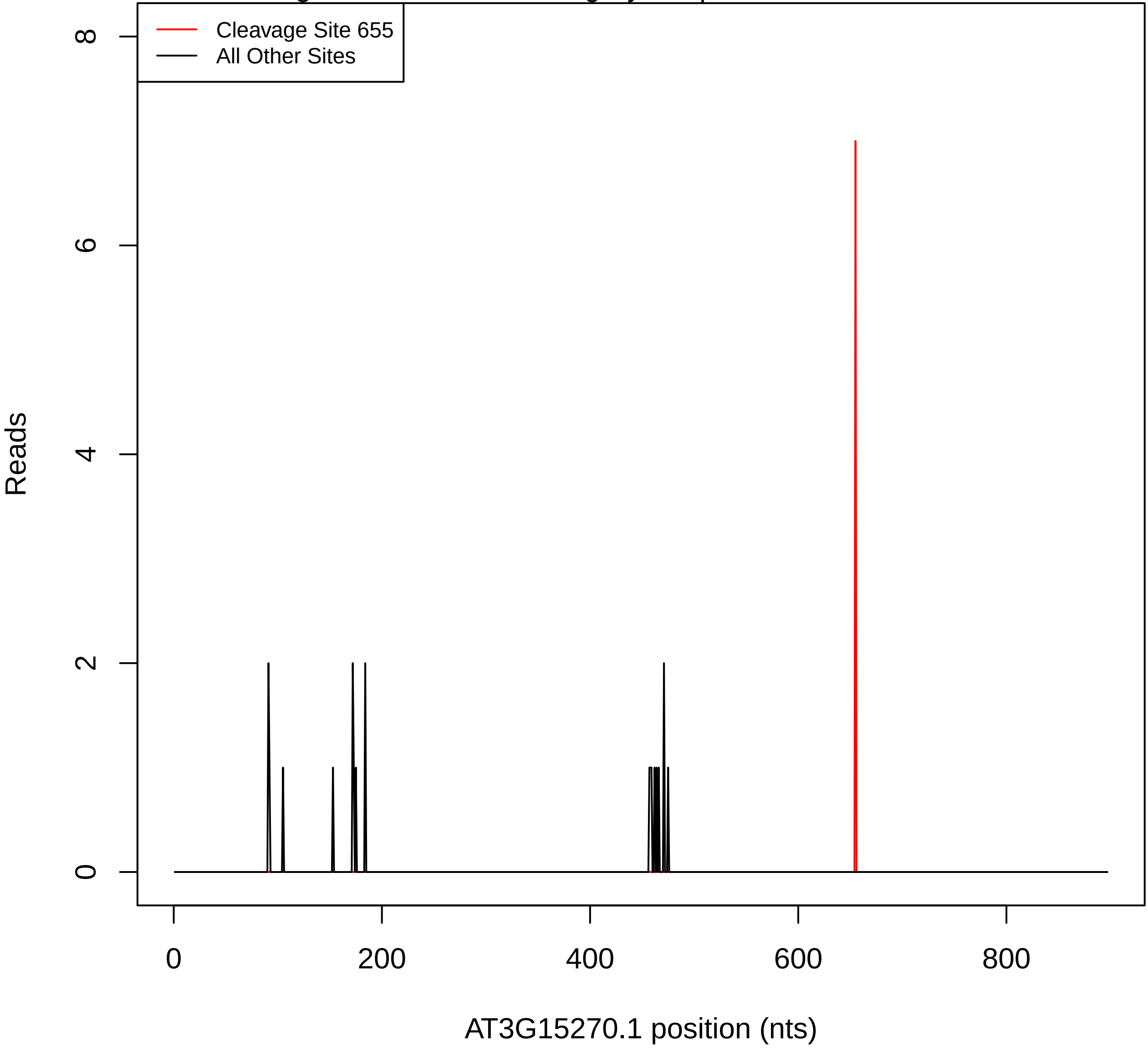

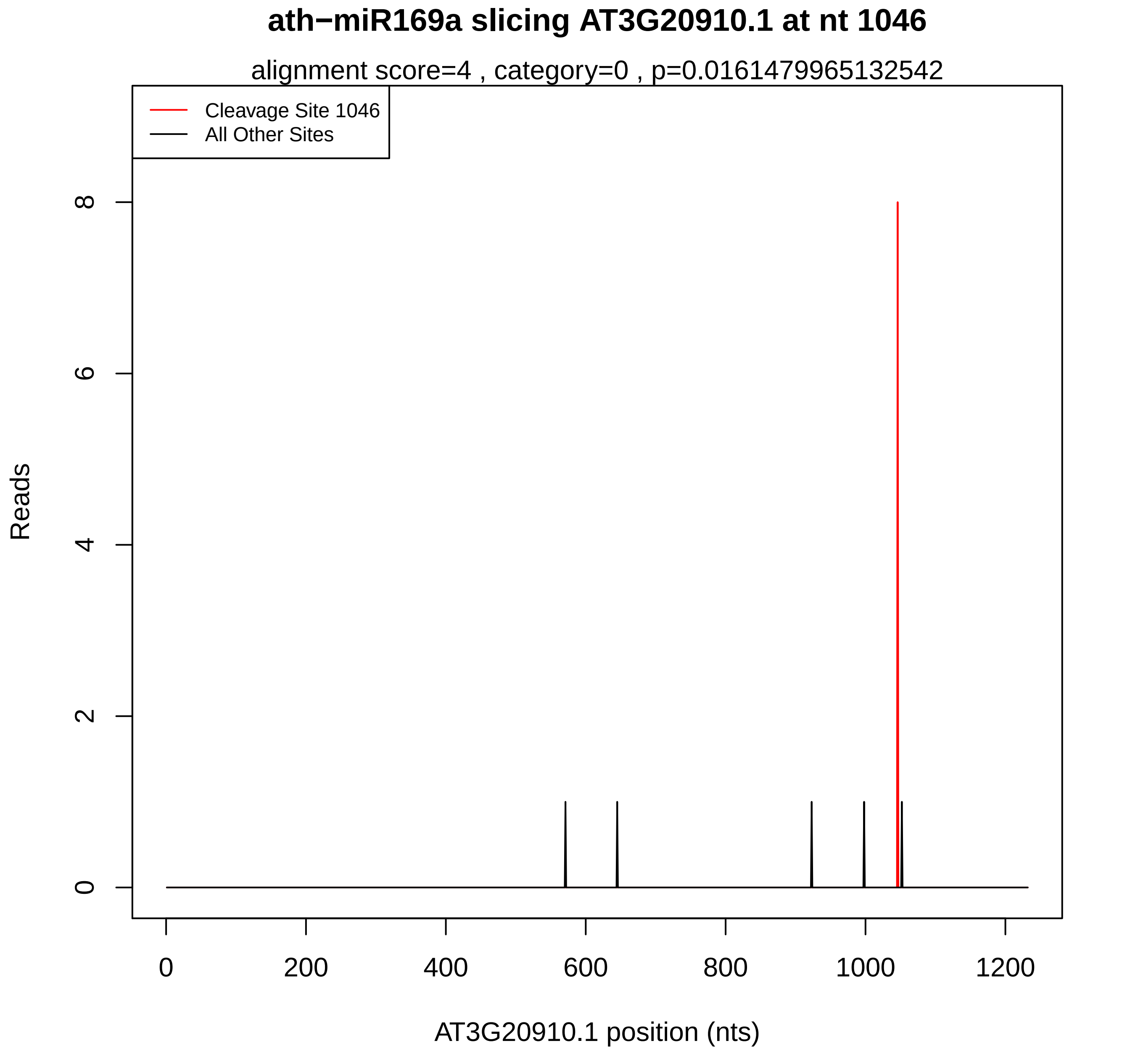

ath−miR169a slicing AT3G20910.1 at nt 1046
alignment score=4 , category=0 , p=0.0161479965132542
Cleavage Site 1046
All Other Sites
Reads
0
2
4
6
8
0
200
400
600
800
1000
1200
AT3G20910.1 position (nts)

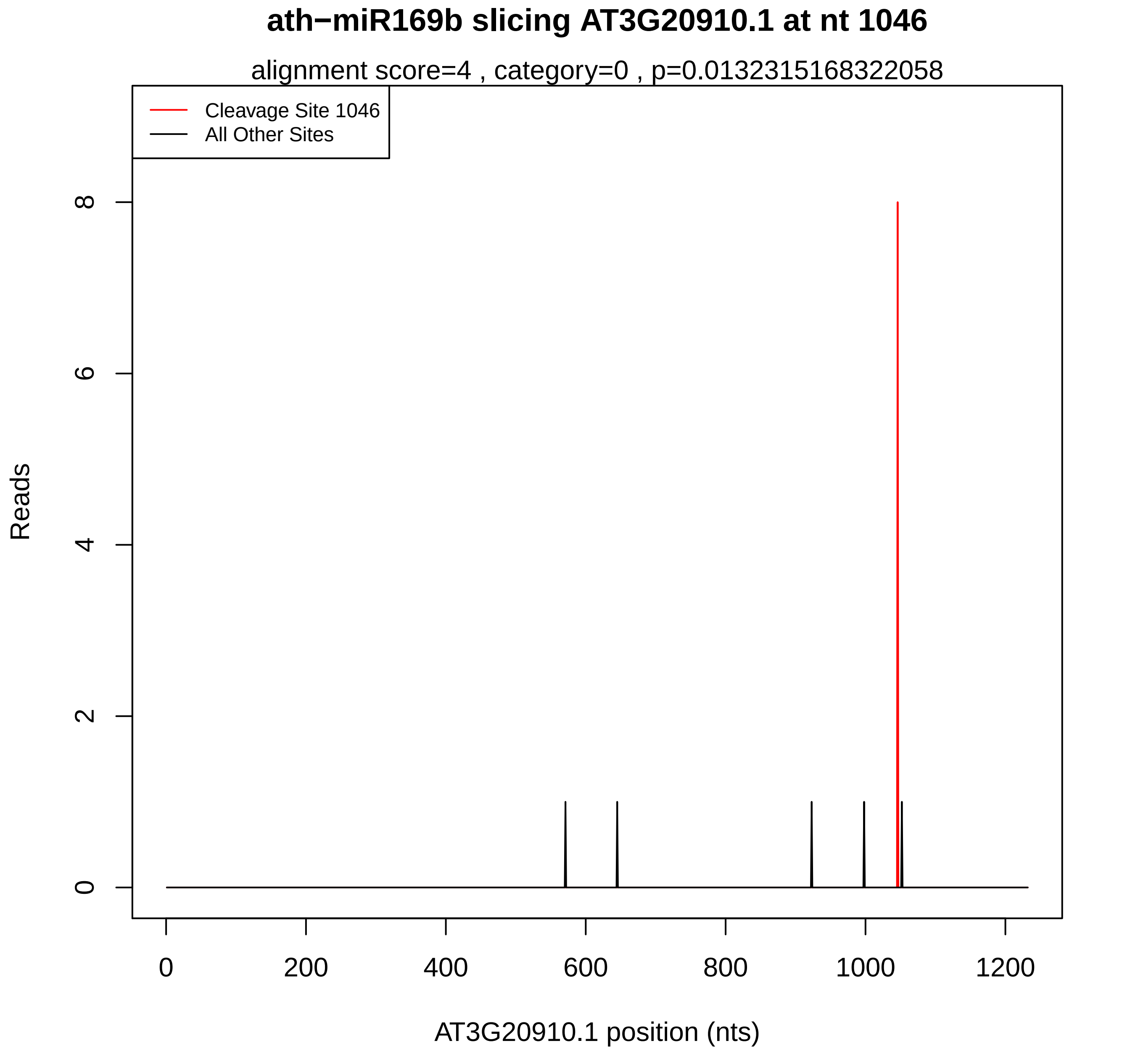
ath−miR169b slicing AT3G20910.1 at nt 1046
alignment score=4 , category=0 , p=0.0132315168322058
Cleavage Site 1046
All Other Sites
Reads
0
2
4
6
8
0
200
400
600
800
1000
1200
AT3G20910.1 position (nts)

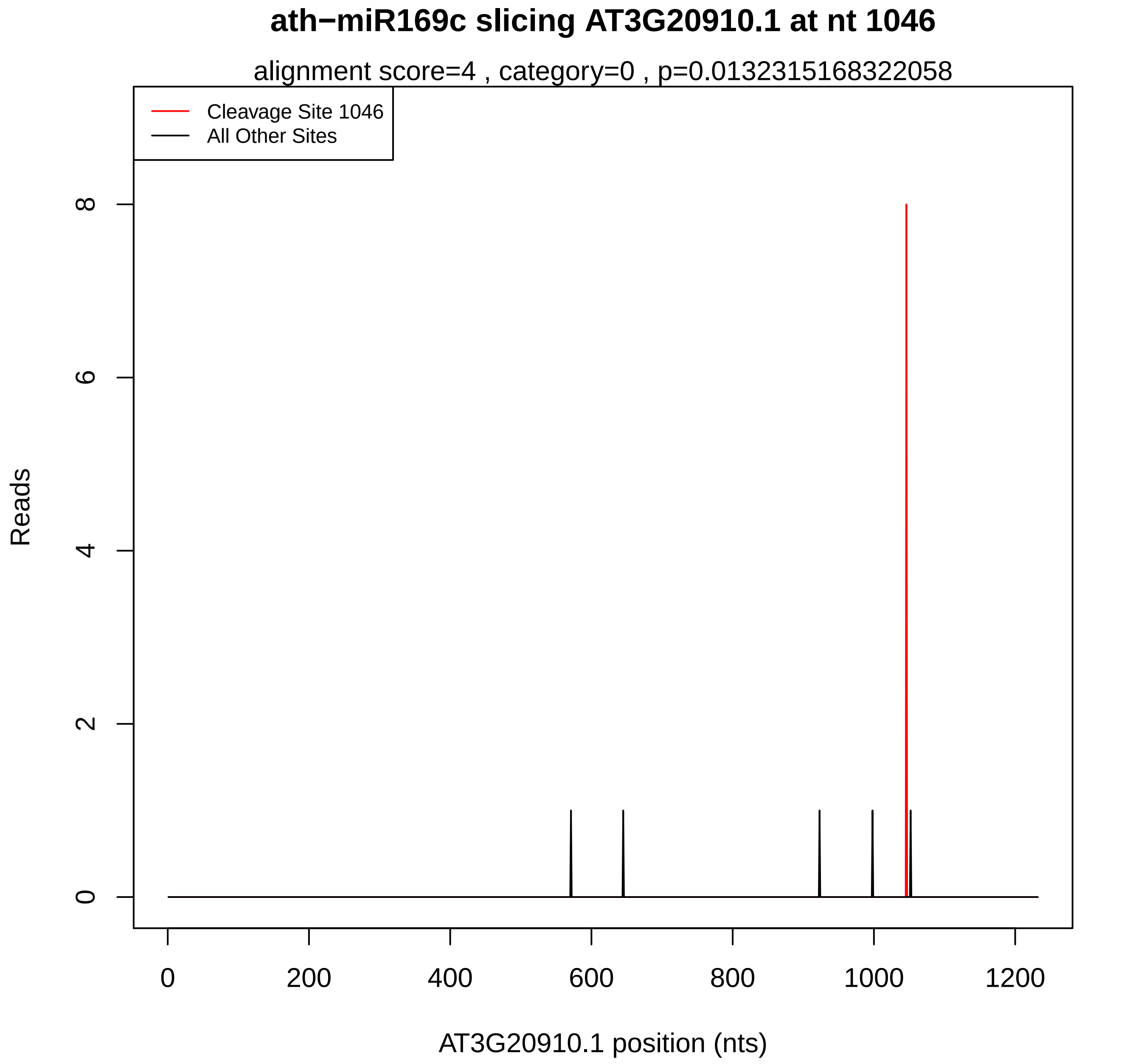

ath−miR169c slicing AT3G20910.1 at nt 1046
alignment score=4 , category=0 , p=0.0132315168322058
Cleavage Site 1046
All Other Sites
Reads
0
2
4
6
8
0
200
400
600
800
1000
1200
AT3G20910.1 position (nts)

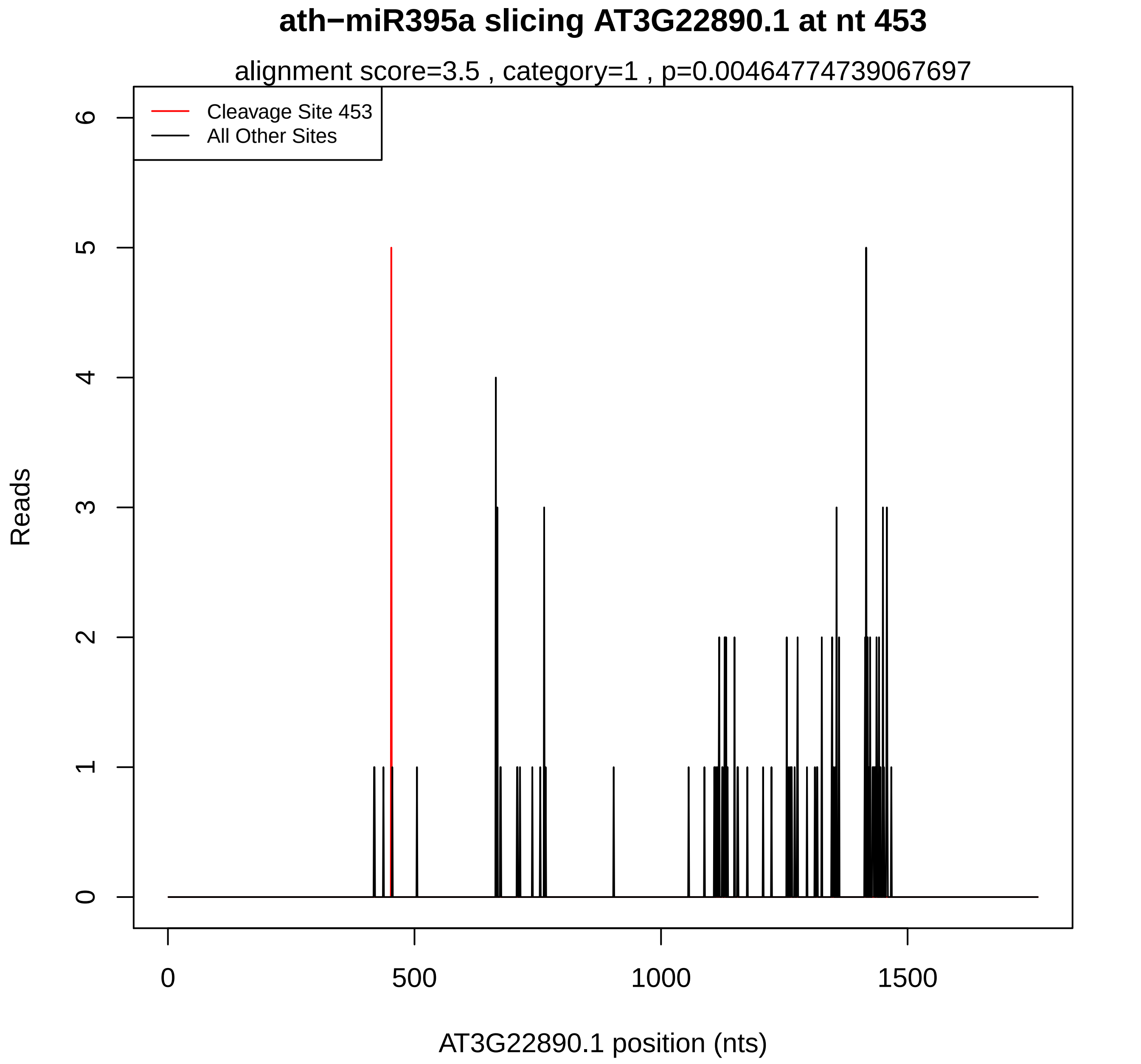

ath−miR395a slicing AT3G22890.1 at nt 453
alignment score=3.5 , category=1 , p=0.00464774739067697
Cleavage Site 453
All Other Sites
Reads
0
1
2
3
4
5
6
0
500
1000
1500
AT3G22890.1 position (nts)

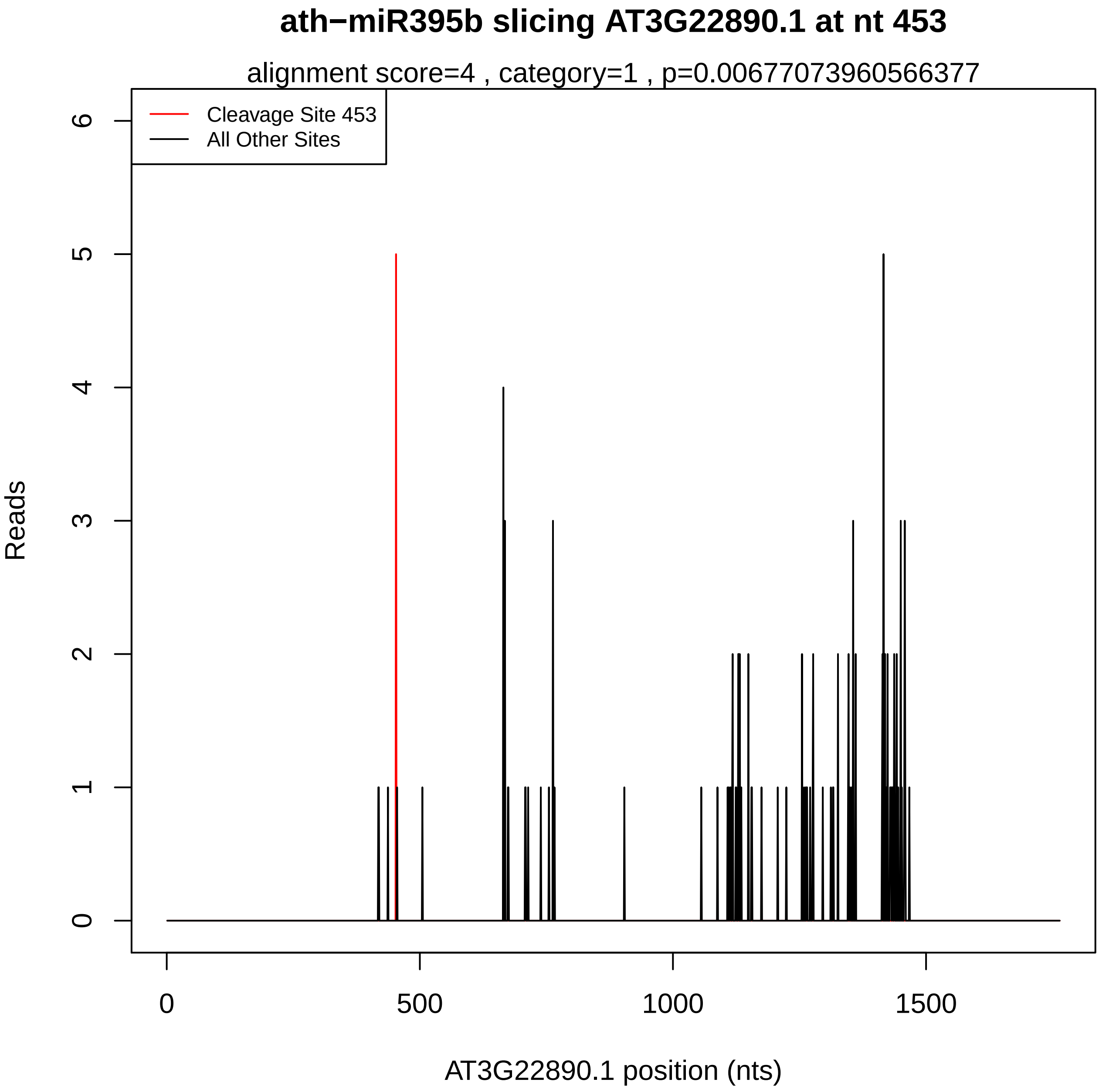

ath−miR395b slicing AT3G22890.1 at nt 453
alignment score=4 , category=1 , p=0.00677073960566377
Cleavage Site 453
All Other Sites
Reads
0
1
2
3
4
5
6
0
500
1000
1500
AT3G22890.1 position (nts)

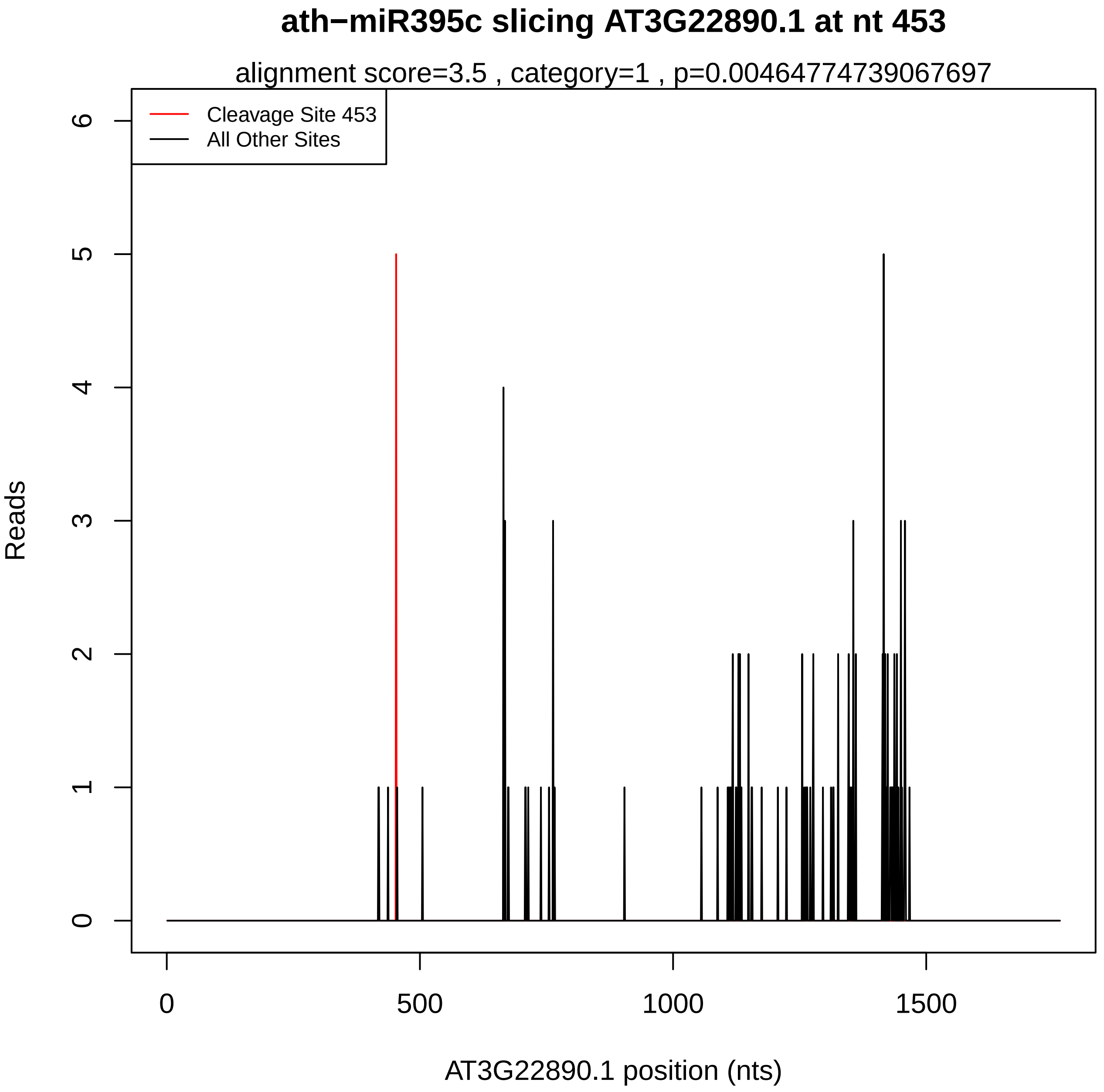

ath−miR395c slicing AT3G22890.1 at nt 453
alignment score=3.5 , category=1 , p=0.00464774739067697
Cleavage Site 453
All Other Sites
Reads
0
1
2
3
4
5
6
0
500
1000
1500
AT3G22890.1 position (nts)

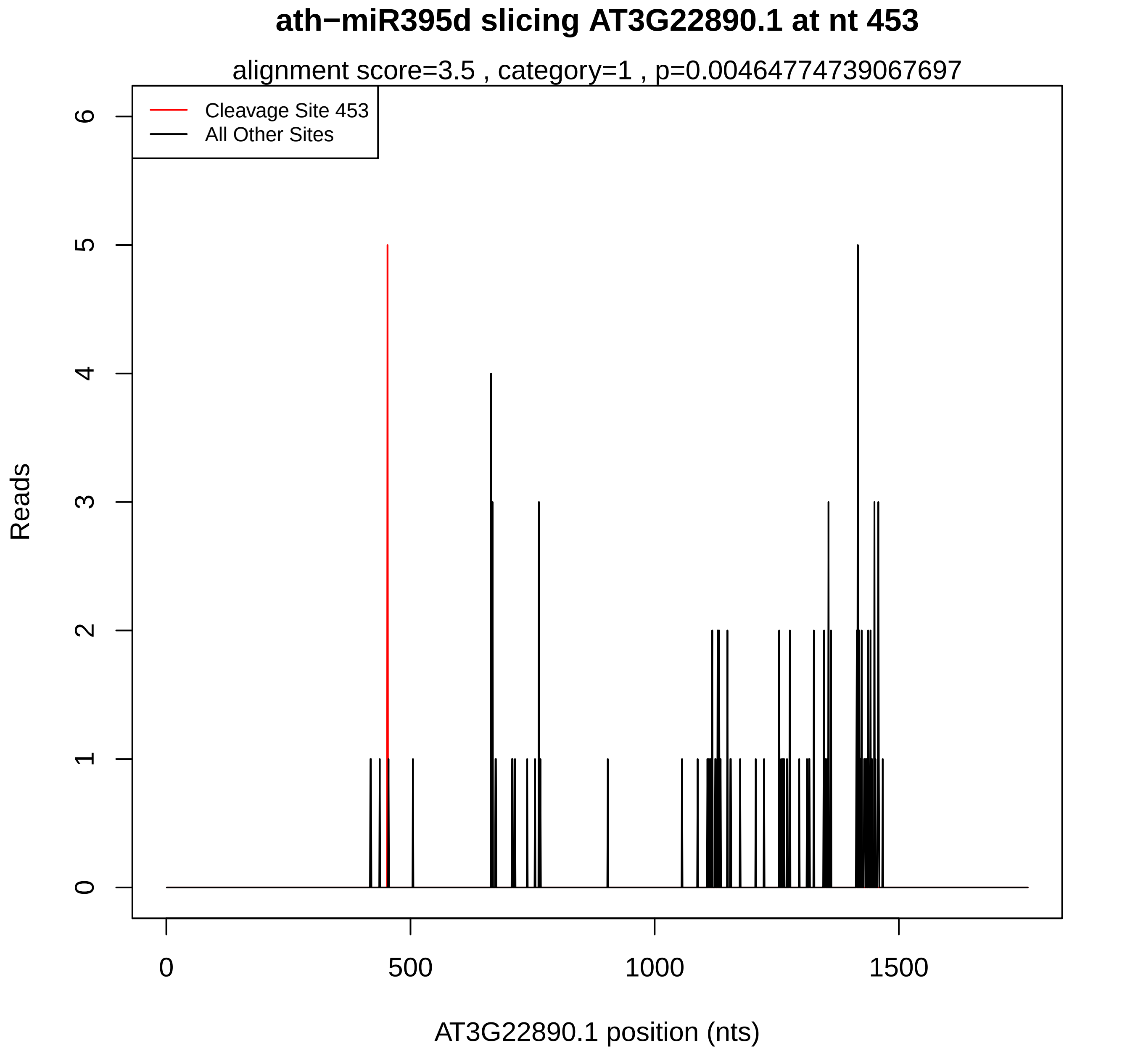
ath−miR395d slicing AT3G22890.1 at nt 453
alignment score=3.5 , category=1 , p=0.00464774739067697
Cleavage Site 453
All Other Sites
Reads
0
1
2
3
4
5
6
0
500
1000
1500
AT3G22890.1 position (nts)

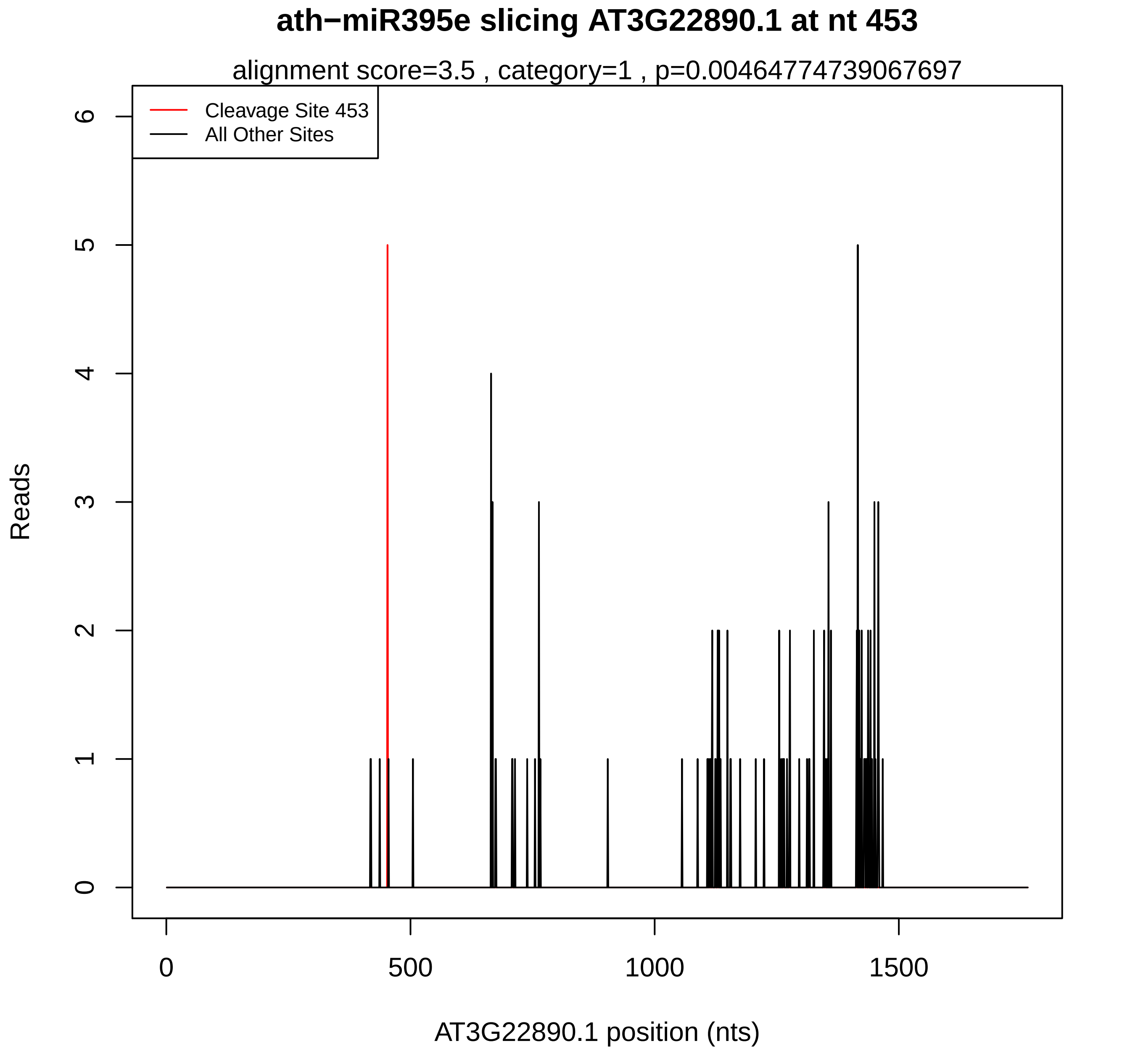

ath−miR395e slicing AT3G22890.1 at nt 453
alignment score=3.5 , category=1 , p=0.00464774739067697
Cleavage Site 453
All Other Sites
Reads
0
1
2
3
4
5
6
0
500
1000
1500
AT3G22890.1 position (nts)

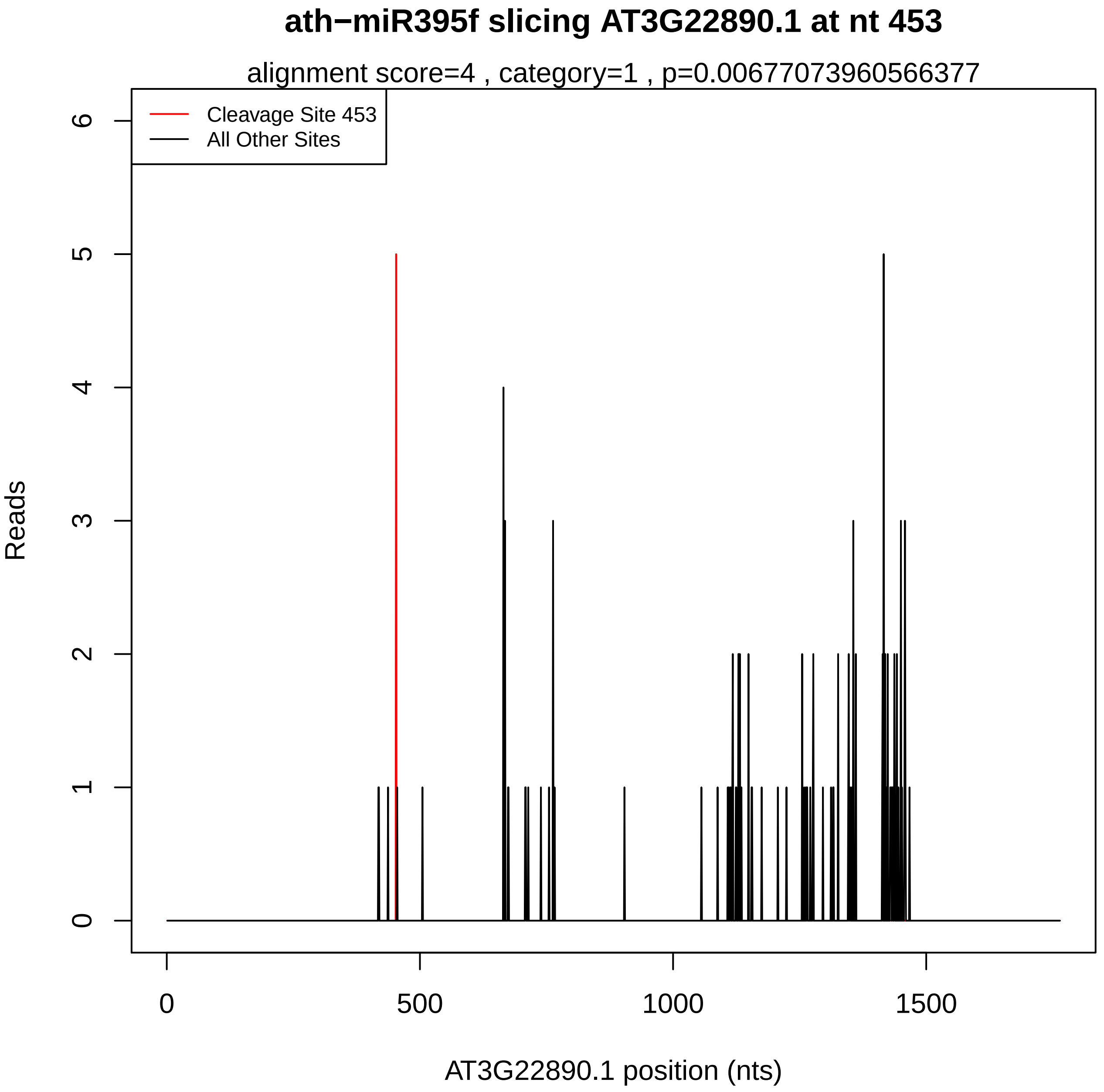

ath−miR395f slicing AT3G22890.1 at nt 453
alignment score=4 , category=1 , p=0.00677073960566377
Cleavage Site 453
All Other Sites
Reads
0
1
2
3
4
5
6
0
500
1000
1500
AT3G22890.1 position (nts)

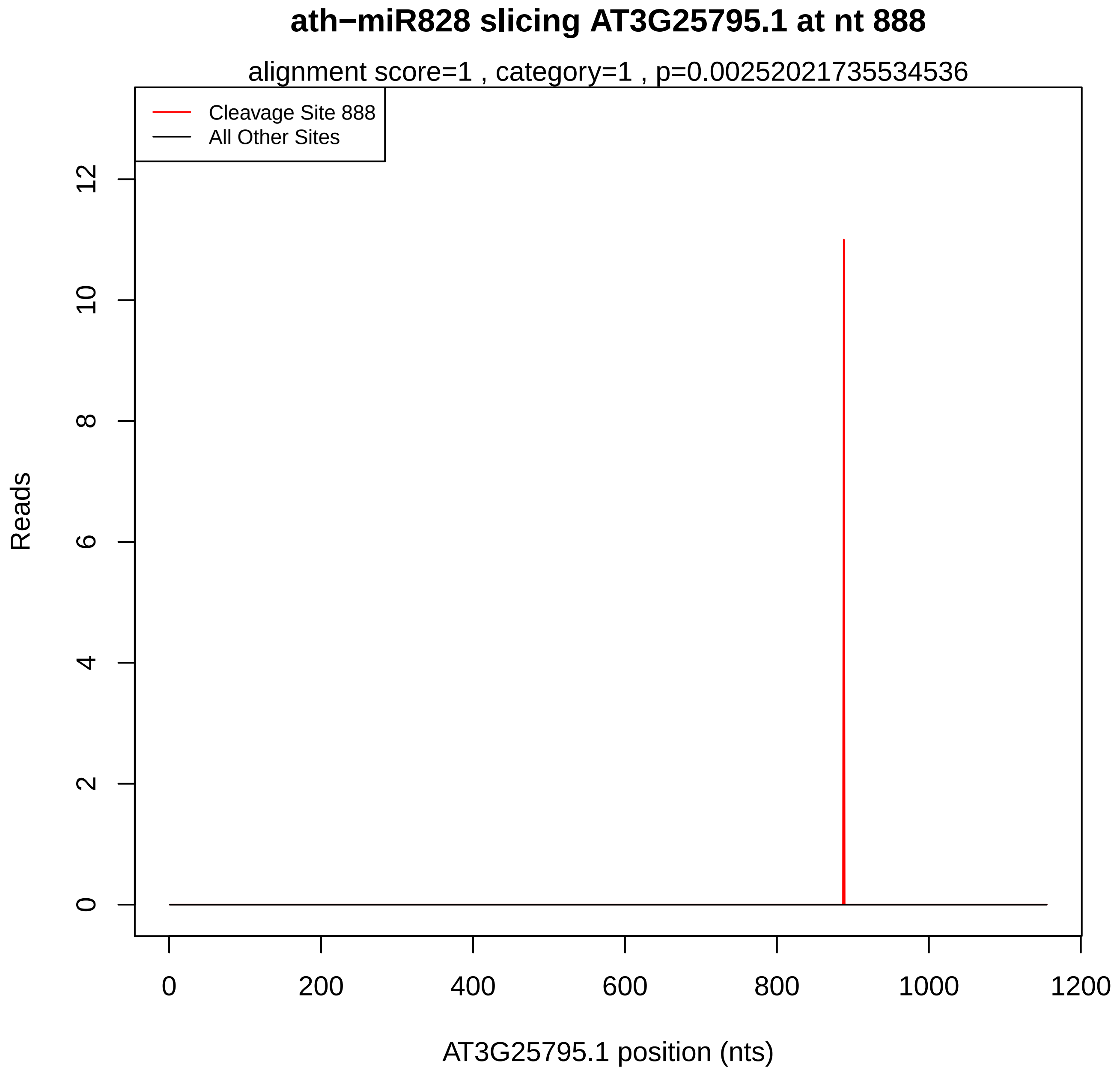

ath−miR828 slicing AT3G25795.1 at nt 888
alignment score=1 , category=1 , p=0.00252021735534536
Cleavage Site 888
All Other Sites
Reads
0
2
4
6
8
10
12
0
200
400
600
800
1000
1200
AT3G25795.1 position (nts)

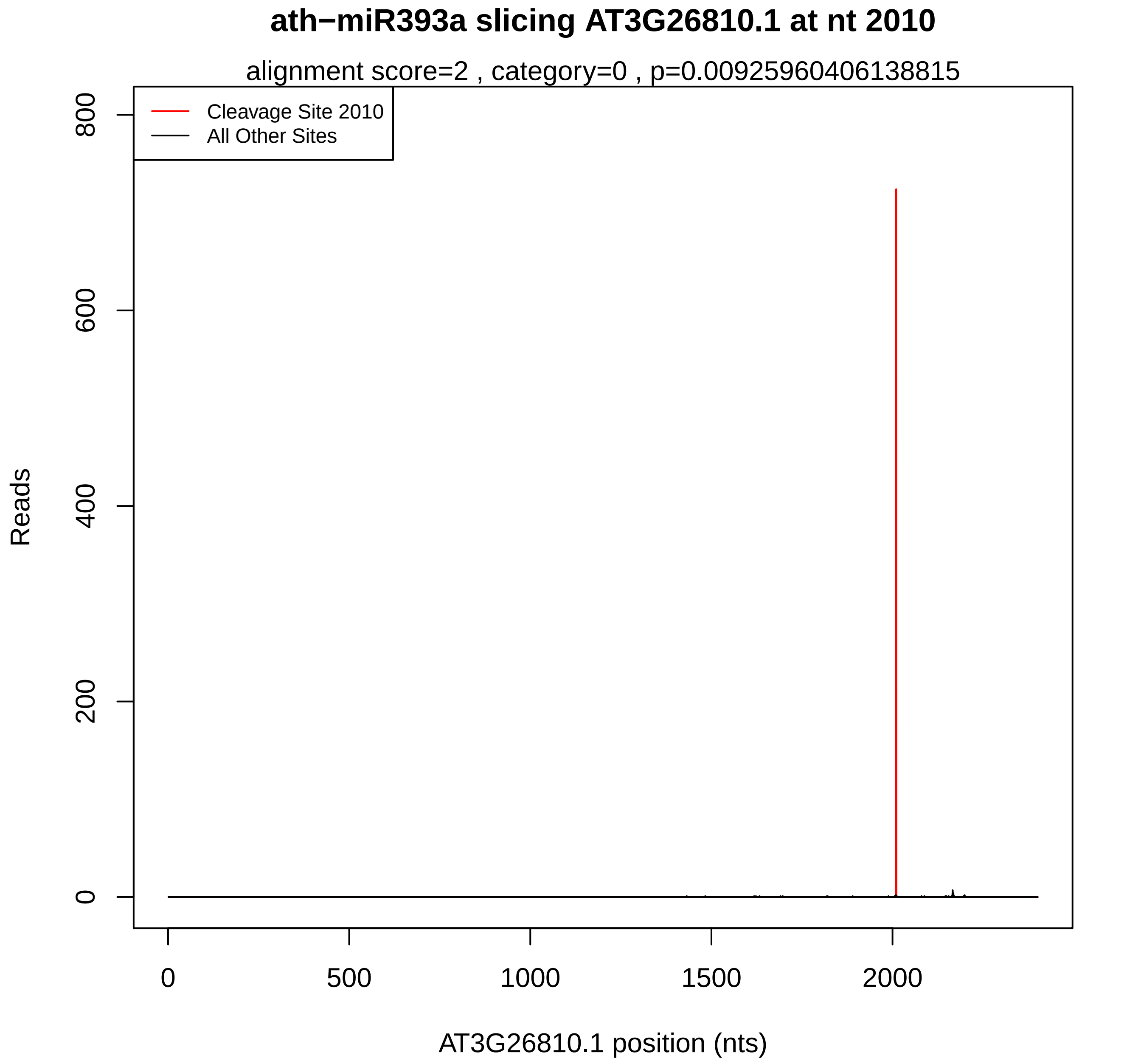
ath−miR393a slicing AT3G26810.1 at nt 2010
alignment score=2 , category=0 , p=0.00925960406138815
Cleavage Site 2010
All Other Sites
Reads
0
200
400
600
800
0
500
1000
1500
2000
AT3G26810.1 position (nts)

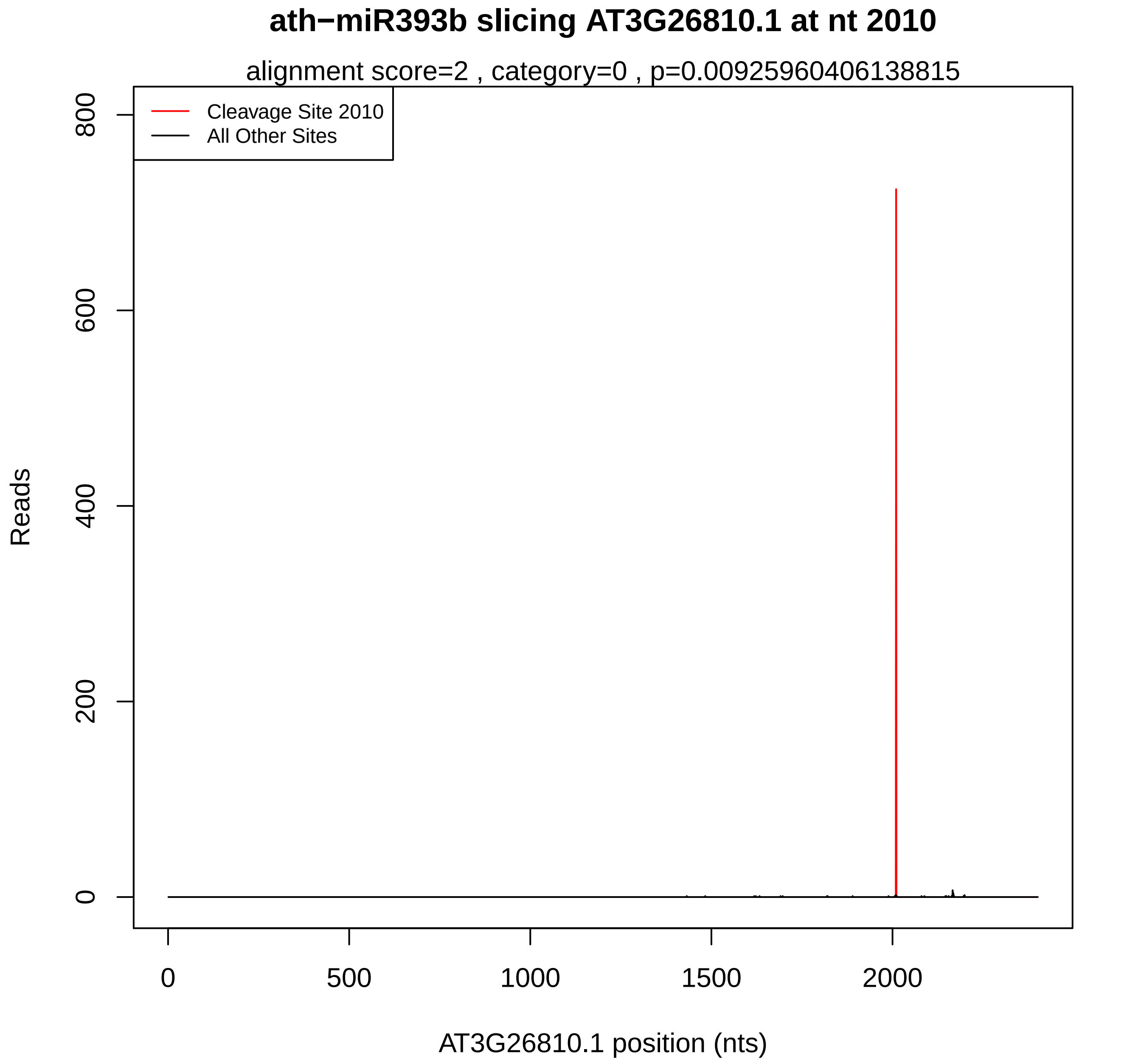

ath−miR393b slicing AT3G26810.1 at nt 2010
alignment score=2 , category=0 , p=0.00925960406138815
Cleavage Site 2010
All Other Sites
Reads
0
200
400
600
800
0
500
1000
1500
2000
AT3G26810.1 position (nts)

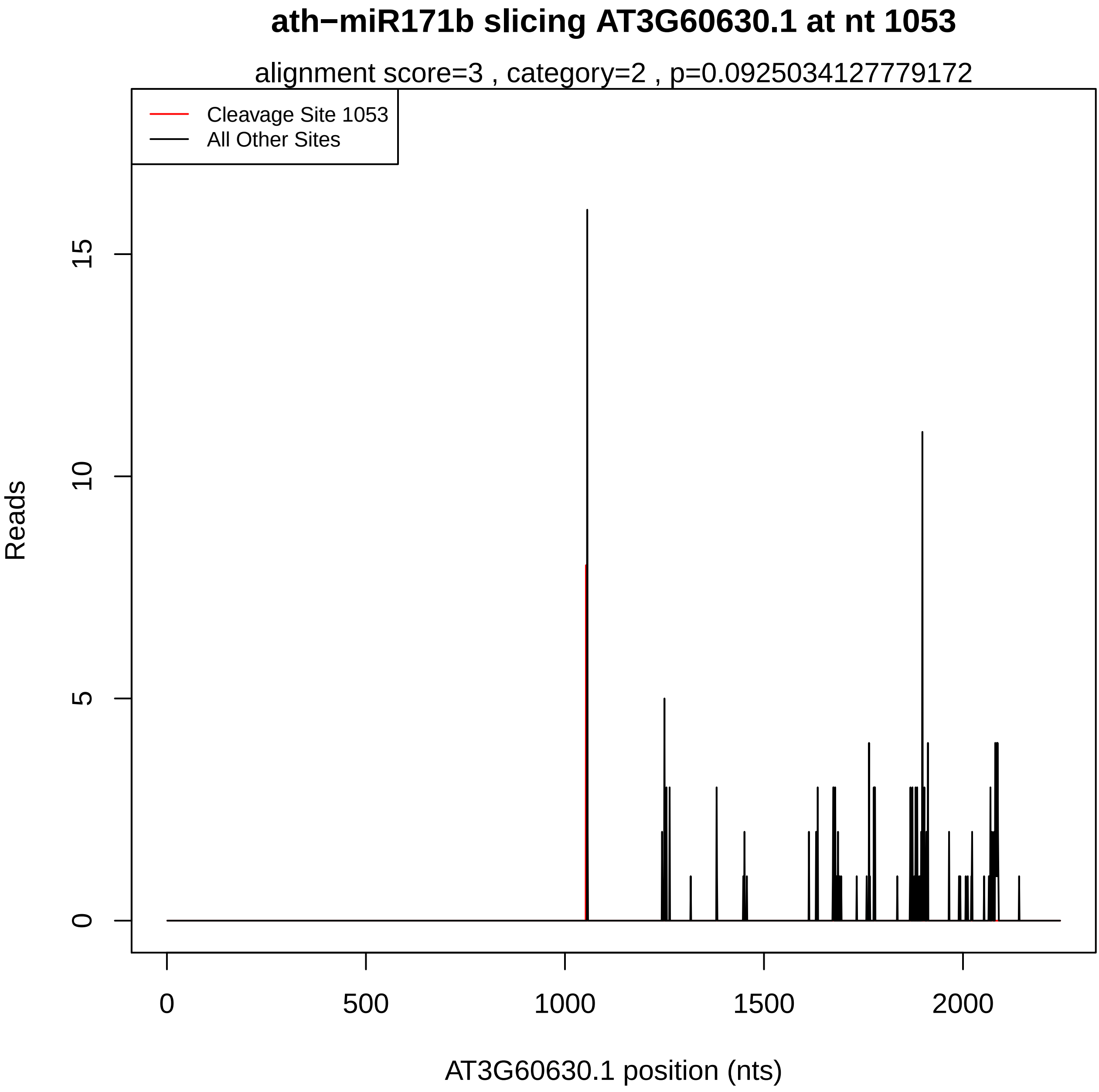
ath−miR171b slicing AT3G60630.1 at nt 1053
alignment score=3 , category=2 , p=0.0925034127779172
Cleavage Site 1053
All Other Sites
Reads
0
5
10
15
0
500
1000
1500
2000
AT3G60630.1 position (nts)

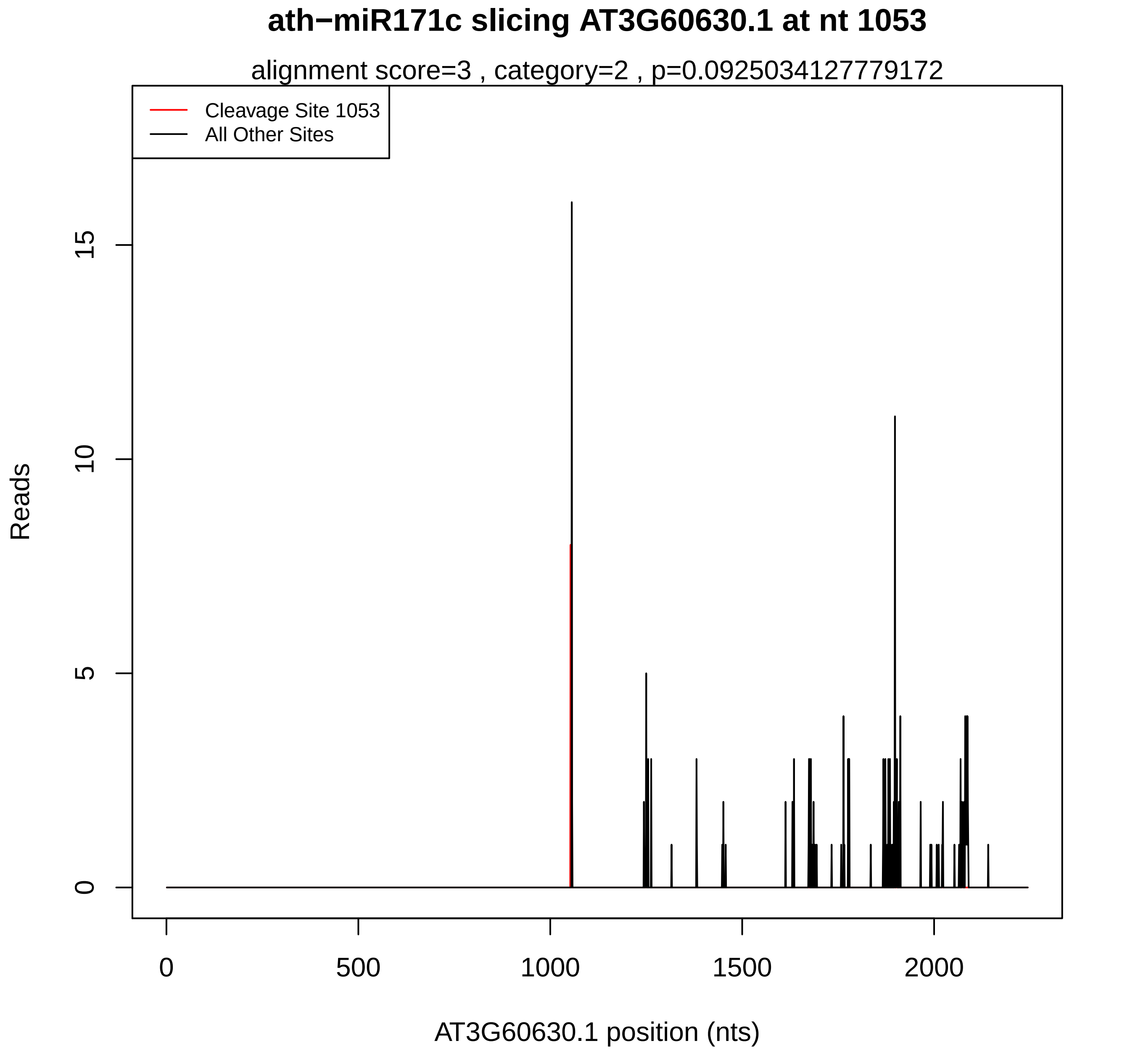
ath−miR171c slicing AT3G60630.1 at nt 1053
alignment score=3 , category=2 , p=0.0925034127779172
Cleavage Site 1053
All Other Sites
Reads
0
5
10
15
0
500
1000
1500
2000
AT3G60630.1 position (nts)

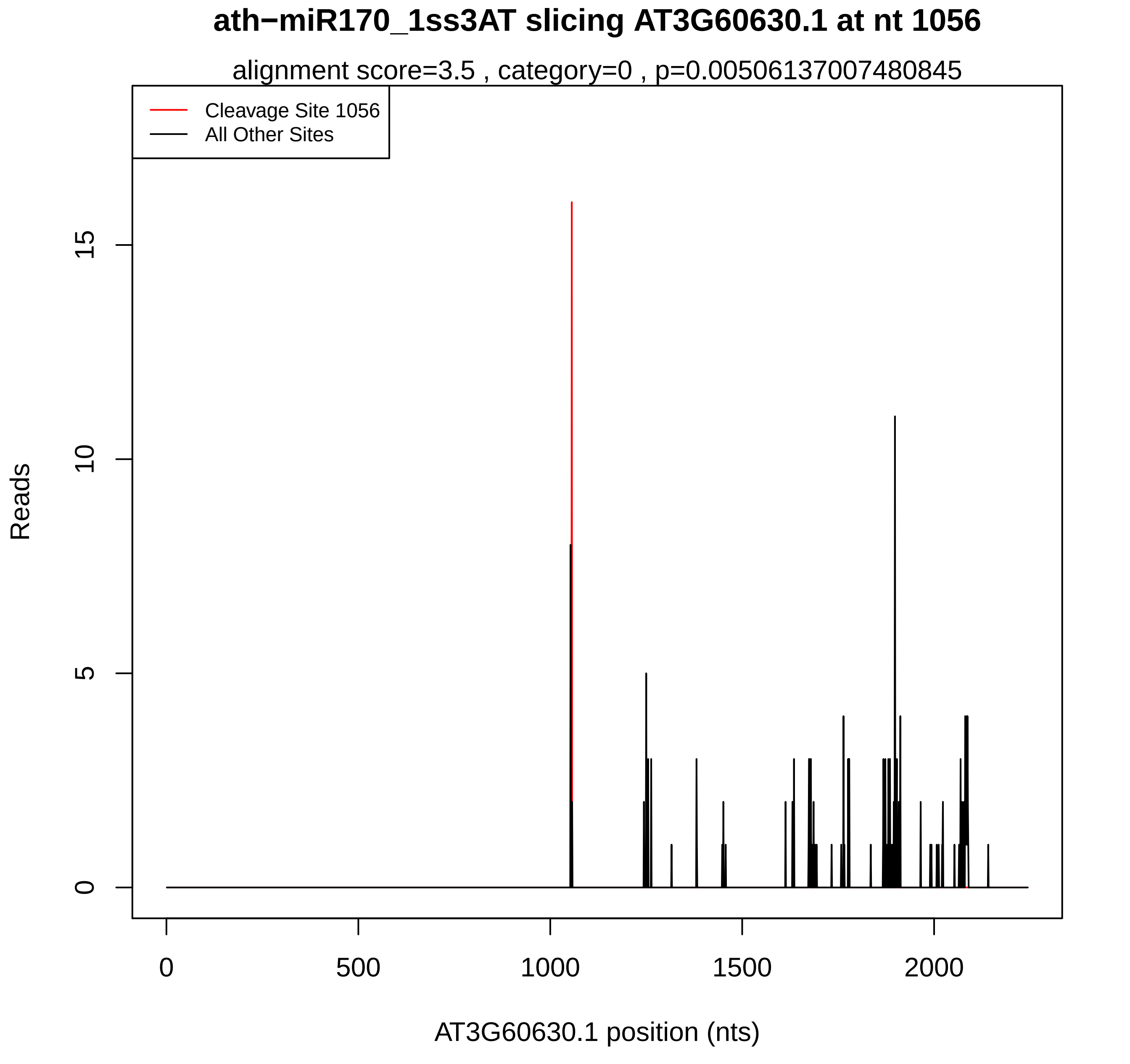
ath−miR170_1ss3AT slicing AT3G60630.1 at nt 1056
alignment score=3.5 , category=0 , p=0.00506137007480845
Cleavage Site 1056
All Other Sites
Reads
0
5
10
15
0
500
1000
1500
2000
AT3G60630.1 position (nts)

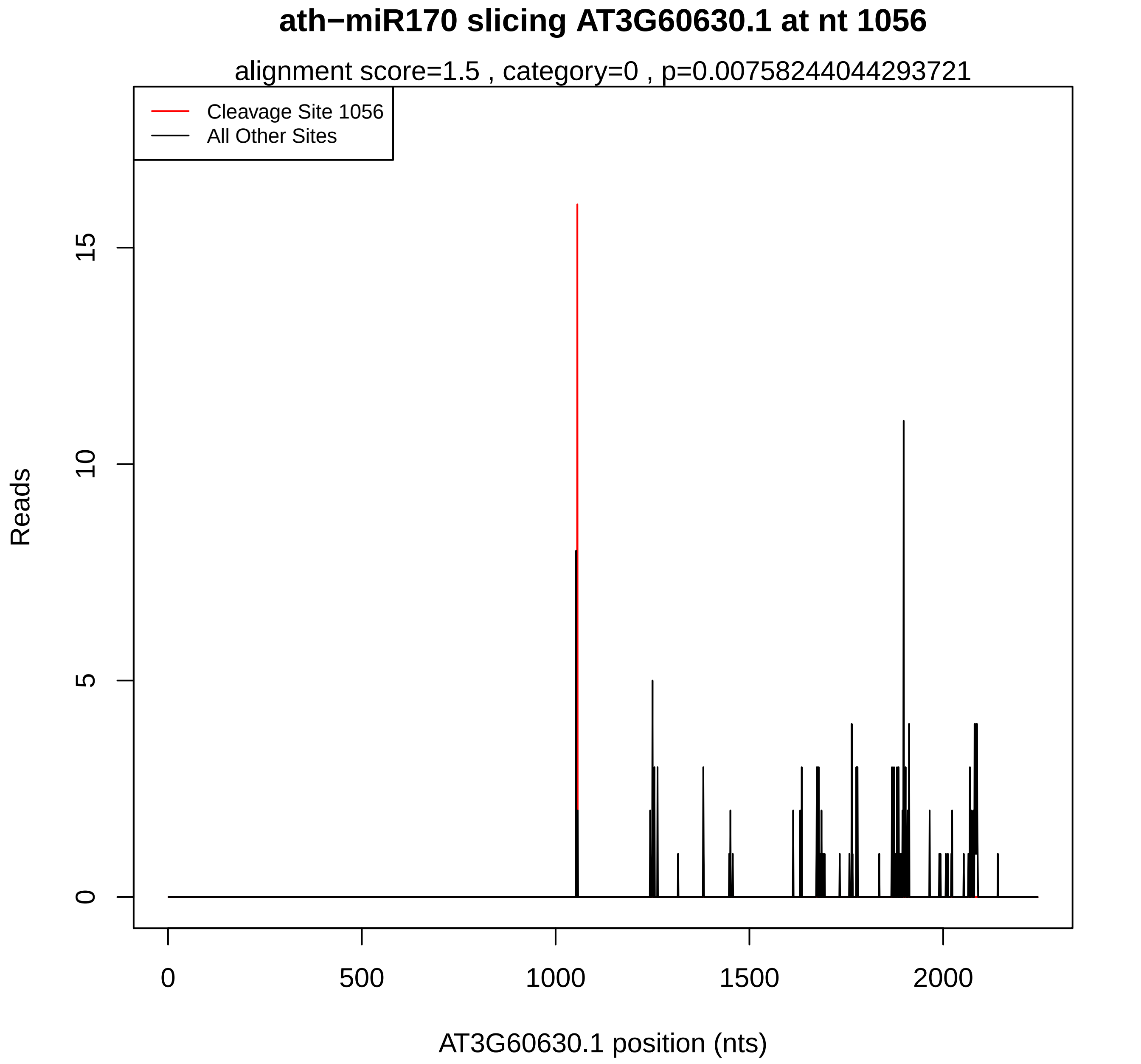
ath−miR170 slicing AT3G60630.1 at nt 1056
alignment score=1.5 , category=0 , p=0.00758244044293721
Cleavage Site 1056
All Other Sites
Reads
0
5
10
15
0
500
1000
1500
2000
AT3G60630.1 position (nts)

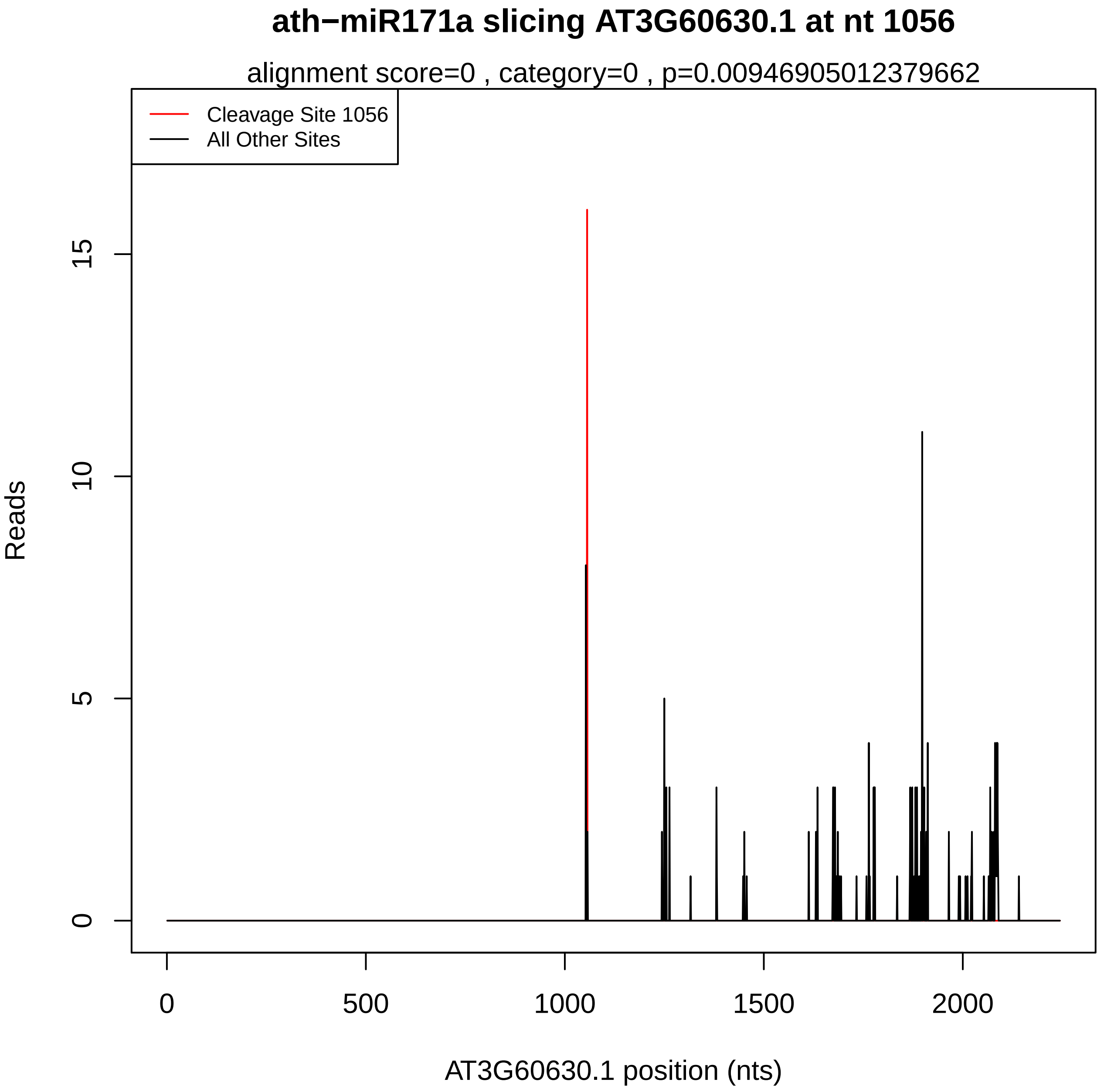

ath−miR171a slicing AT3G60630.1 at nt 1056
alignment score=0 , category=0 , p=0.00946905012379662
Cleavage Site 1056
All Other Sites
Reads
0
5
10
15
0
500
1000
1500
2000
AT3G60630.1 position (nts)

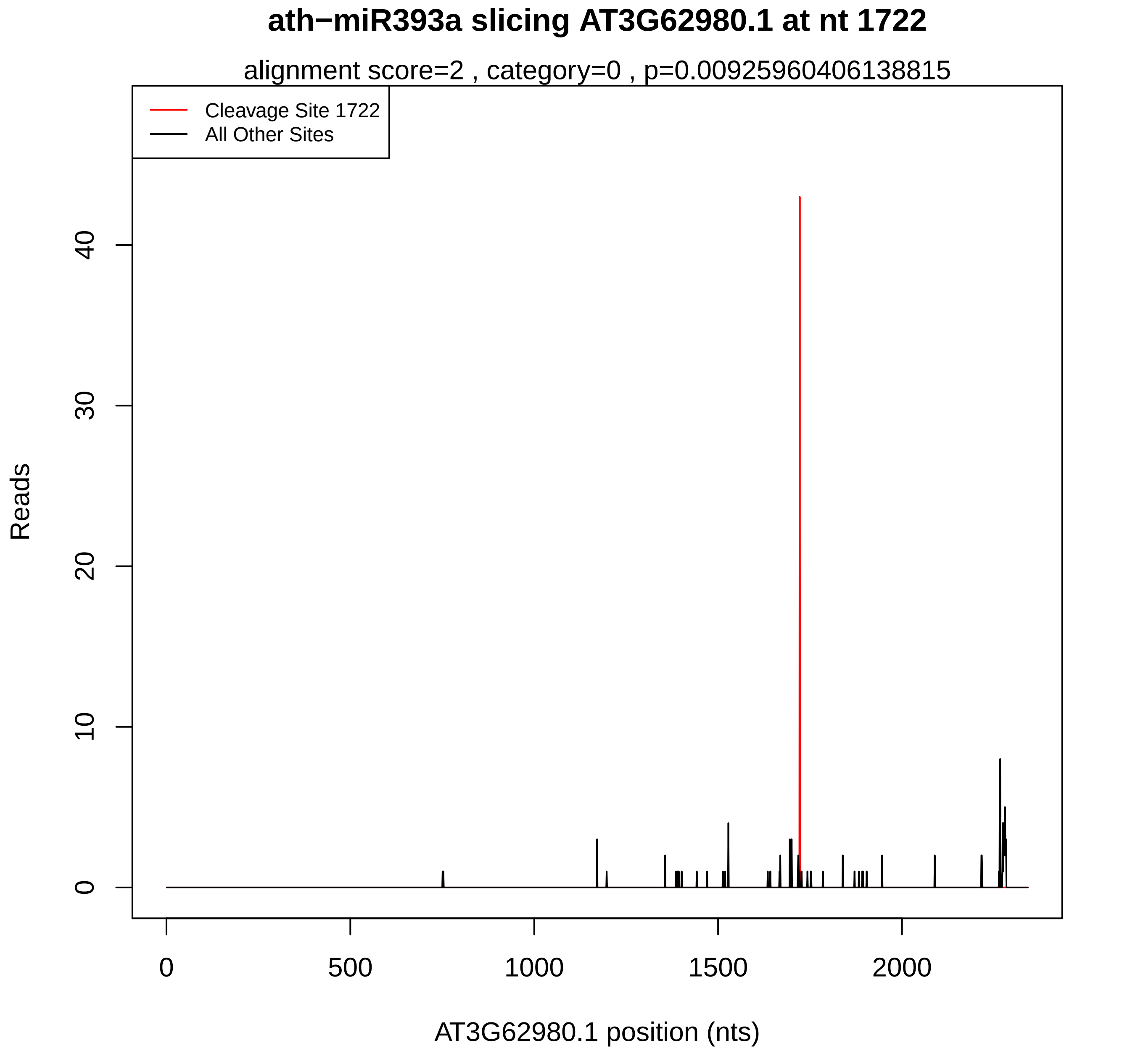
ath−miR393a slicing AT3G62980.1 at nt 1722
alignment score=2 , category=0 , p=0.00925960406138815
Cleavage Site 1722
All Other Sites
Reads
0
10
20
30
40
0
500
1000
1500
2000
AT3G62980.1 position (nts)

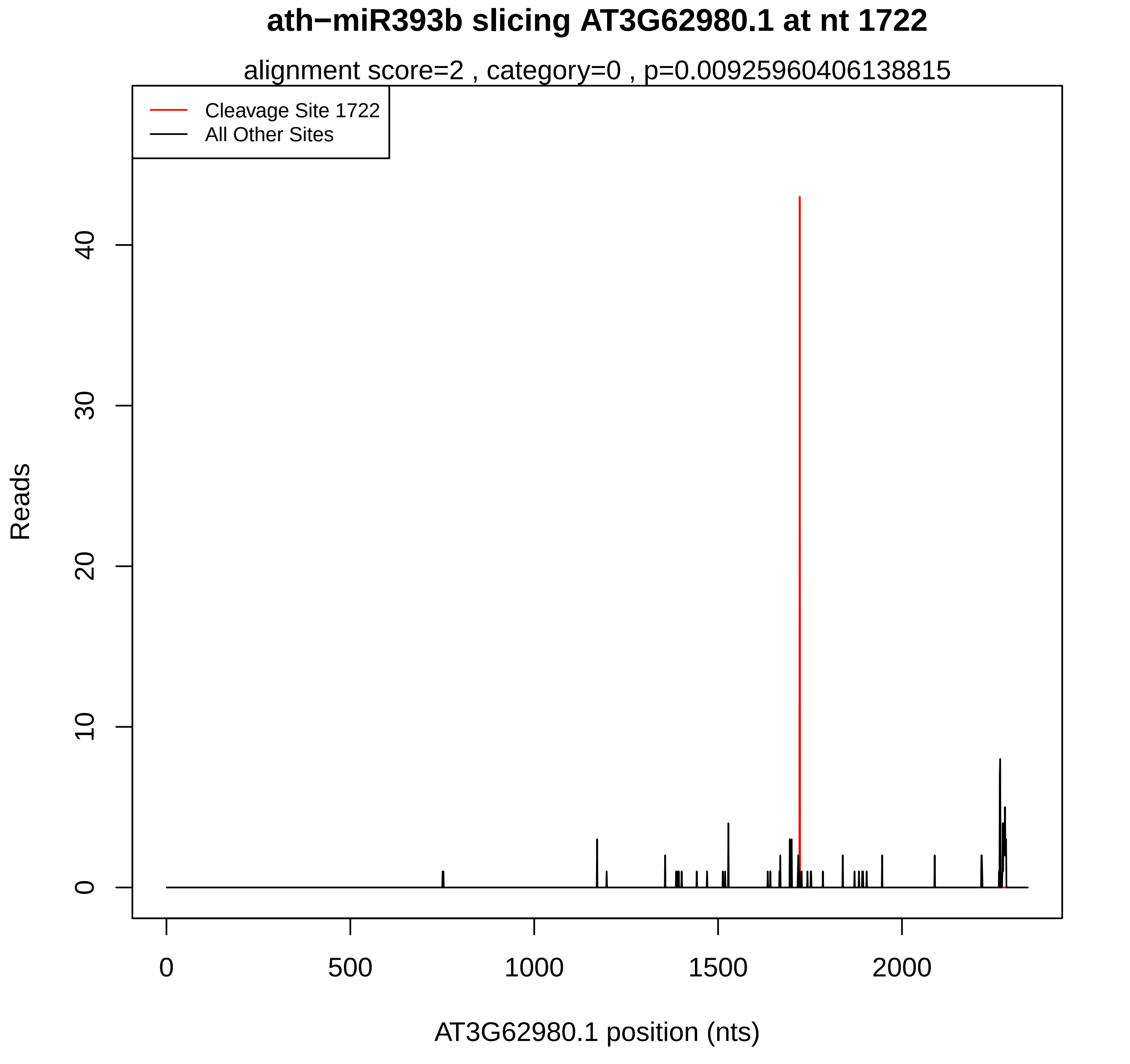
ath−miR393b slicing AT3G62980.1 at nt 1722
alignment score=2 , category=0 , p=0.00925960406138815
Cleavage Site 1722
All Other Sites
Reads
0
10
20
30
40
0
500
1000
1500
2000
AT3G62980.1 position (nts)

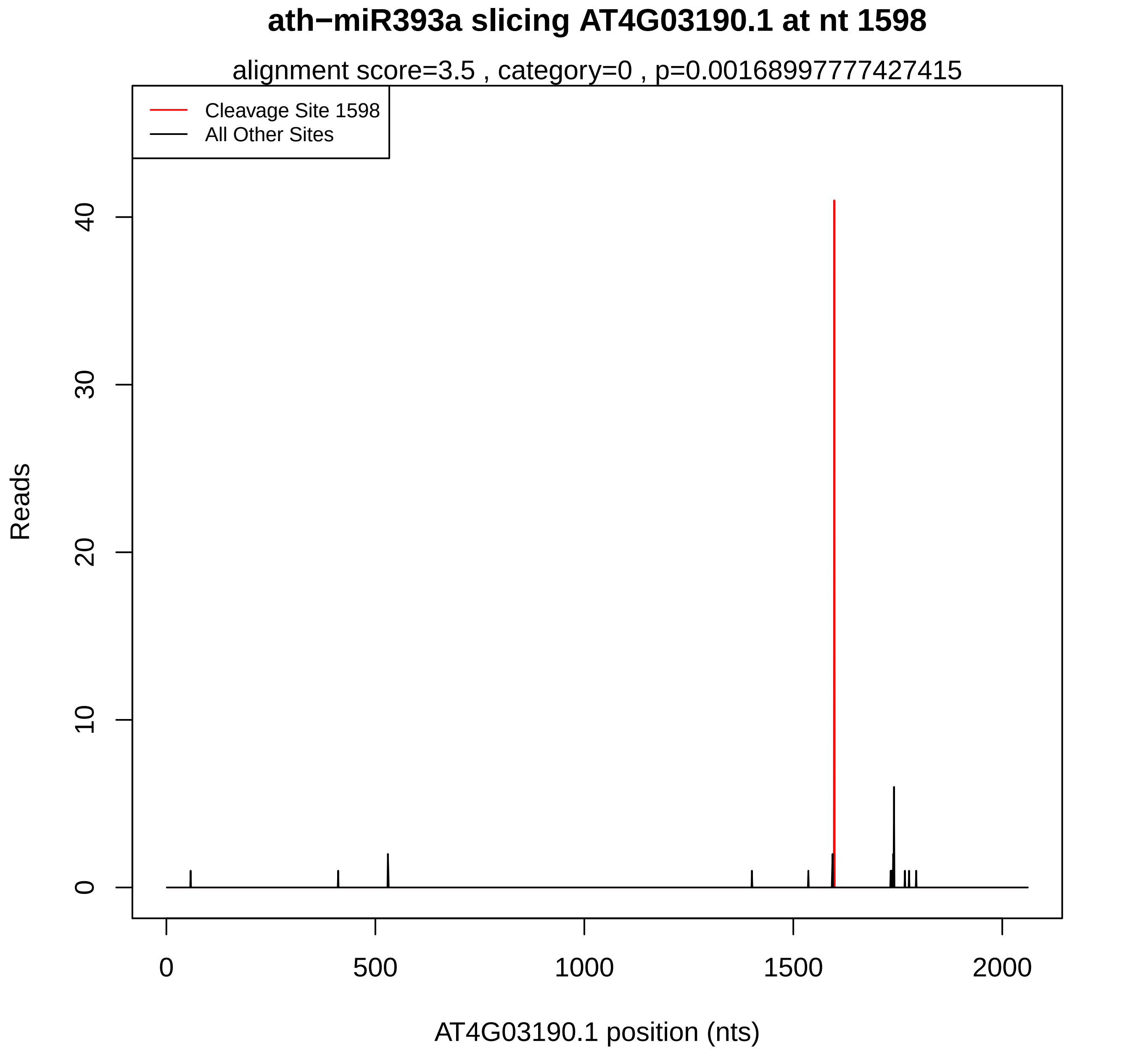

ath−miR393a slicing AT4G03190.1 at nt 1598
alignment score=3.5 , category=0 , p=0.00168997777427415
Cleavage Site 1598
All Other Sites
Reads
0
10
20
30
40
0
500
1000
1500
2000
AT4G03190.1 position (nts)

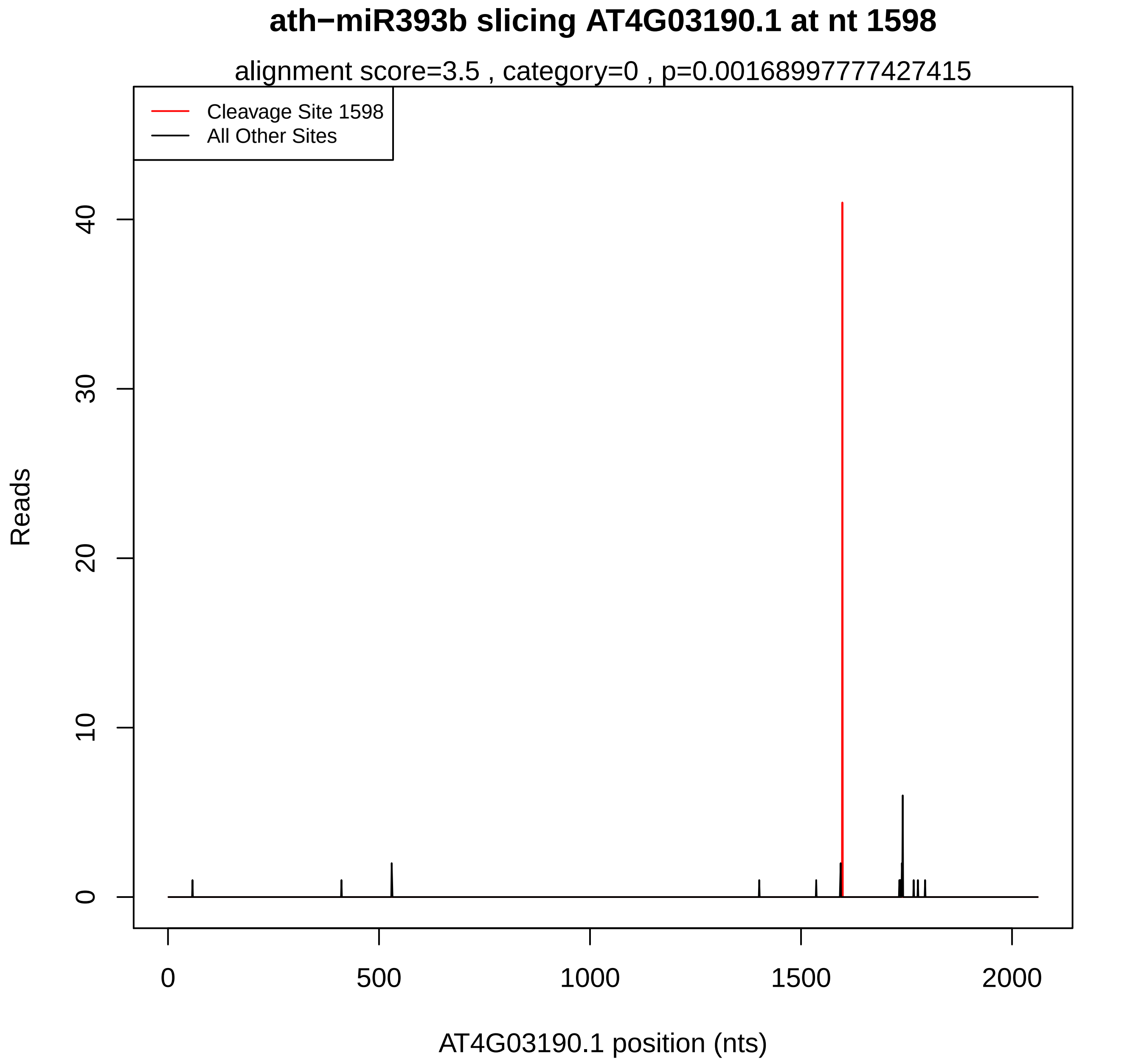

ath−miR393b slicing AT4G03190.1 at nt 1598
alignment score=3.5 , category=0 , p=0.00168997777427415
Cleavage Site 1598
All Other Sites
Reads
0
10
20
30
40
0
500
1000
1500
2000
AT4G03190.1 position (nts)

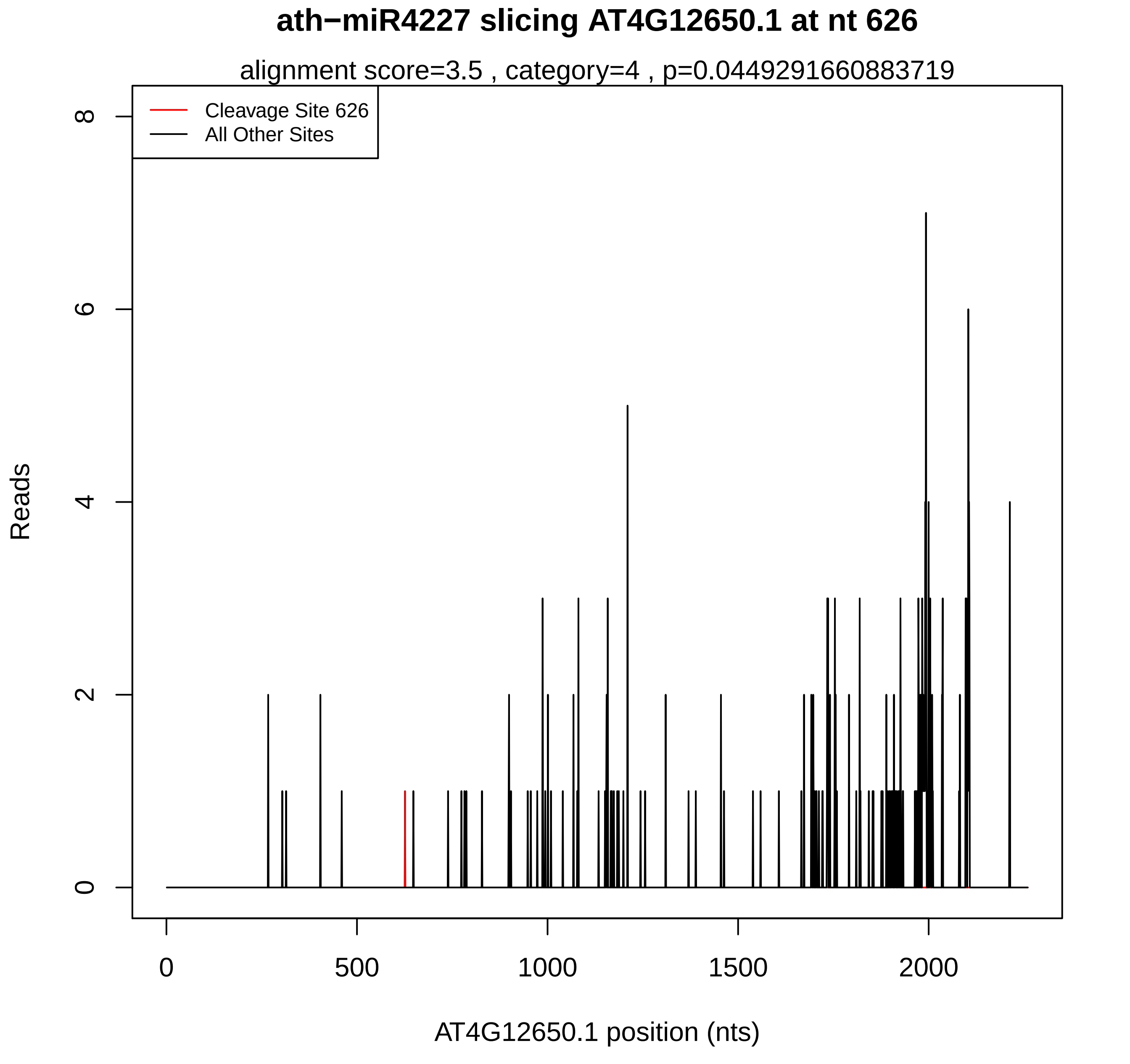
ath−miR4227 slicing AT4G12650.1 at nt 626
alignment score=3.5 , category=4 , p=0.0449291660883719
Cleavage Site 626
All Other Sites
Reads
0
2
4
6
8
0
500
1000
1500
2000
AT4G12650.1 position (nts)

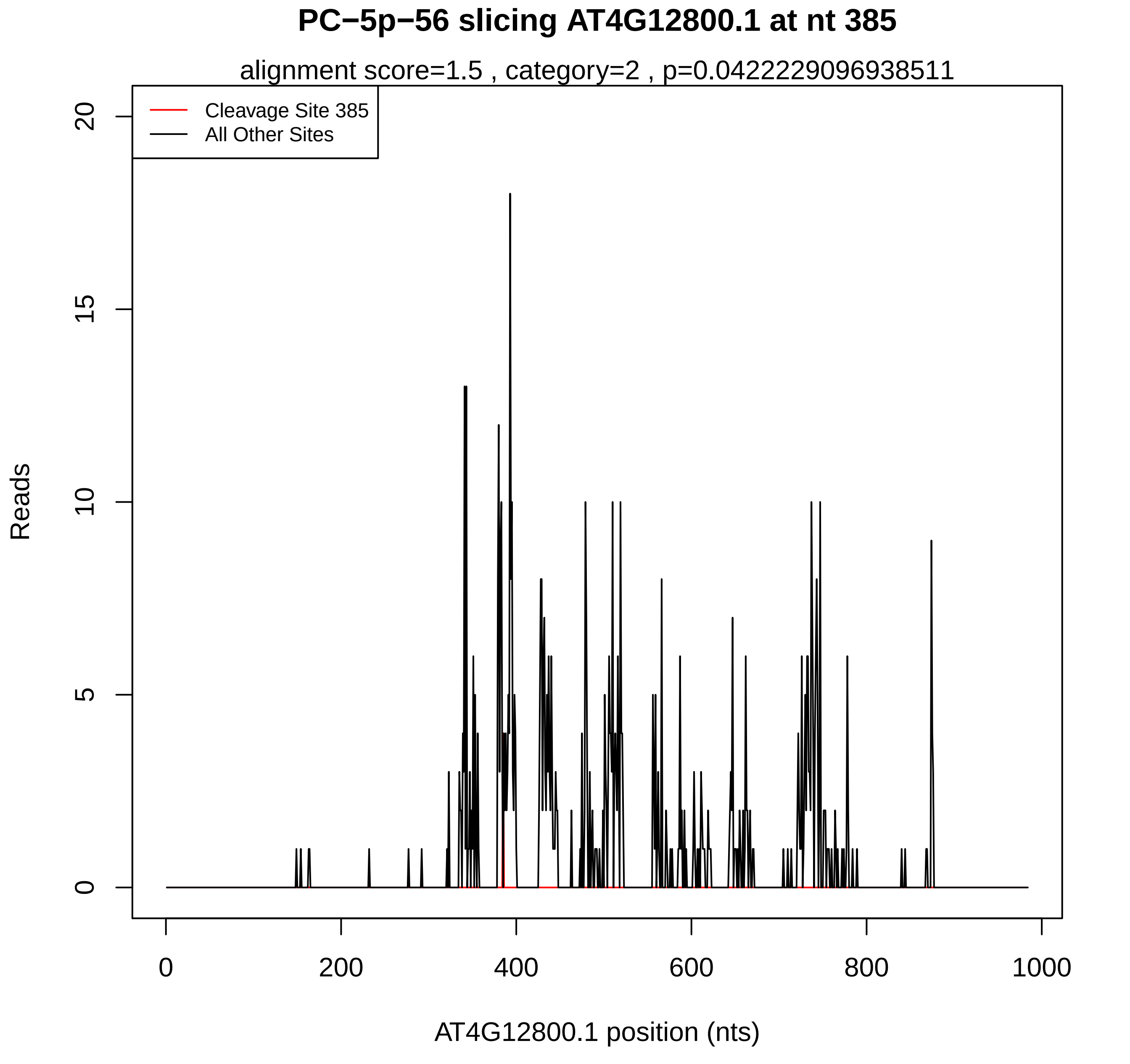

PC−5p−56 slicing AT4G12800.1 at nt 385
alignment score=1.5 , category=2 , p=0.0422229096938511
Cleavage Site 385
All Other Sites
Reads
0
5
10
15
20
0
200
400
600
800
1000
AT4G12800.1 position (nts)

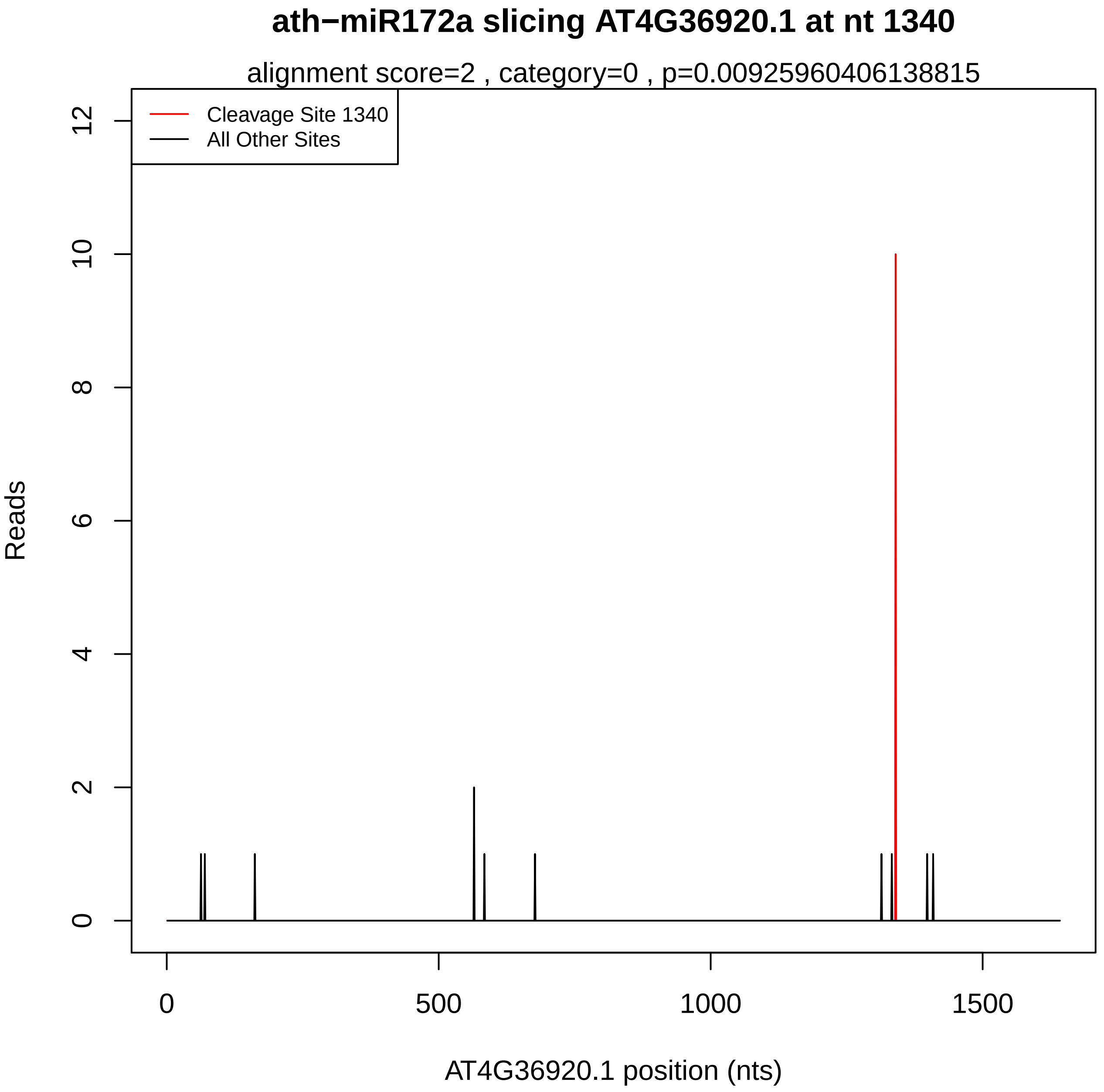
ath−miR172a slicing AT4G36920.1 at nt 1340
alignment score=2 , category=0 , p=0.00925960406138815
Cleavage Site 1340
All Other Sites
Reads
0
2
4
6
8
10
12
0
500
1000
1500
AT4G36920.1 position (nts)

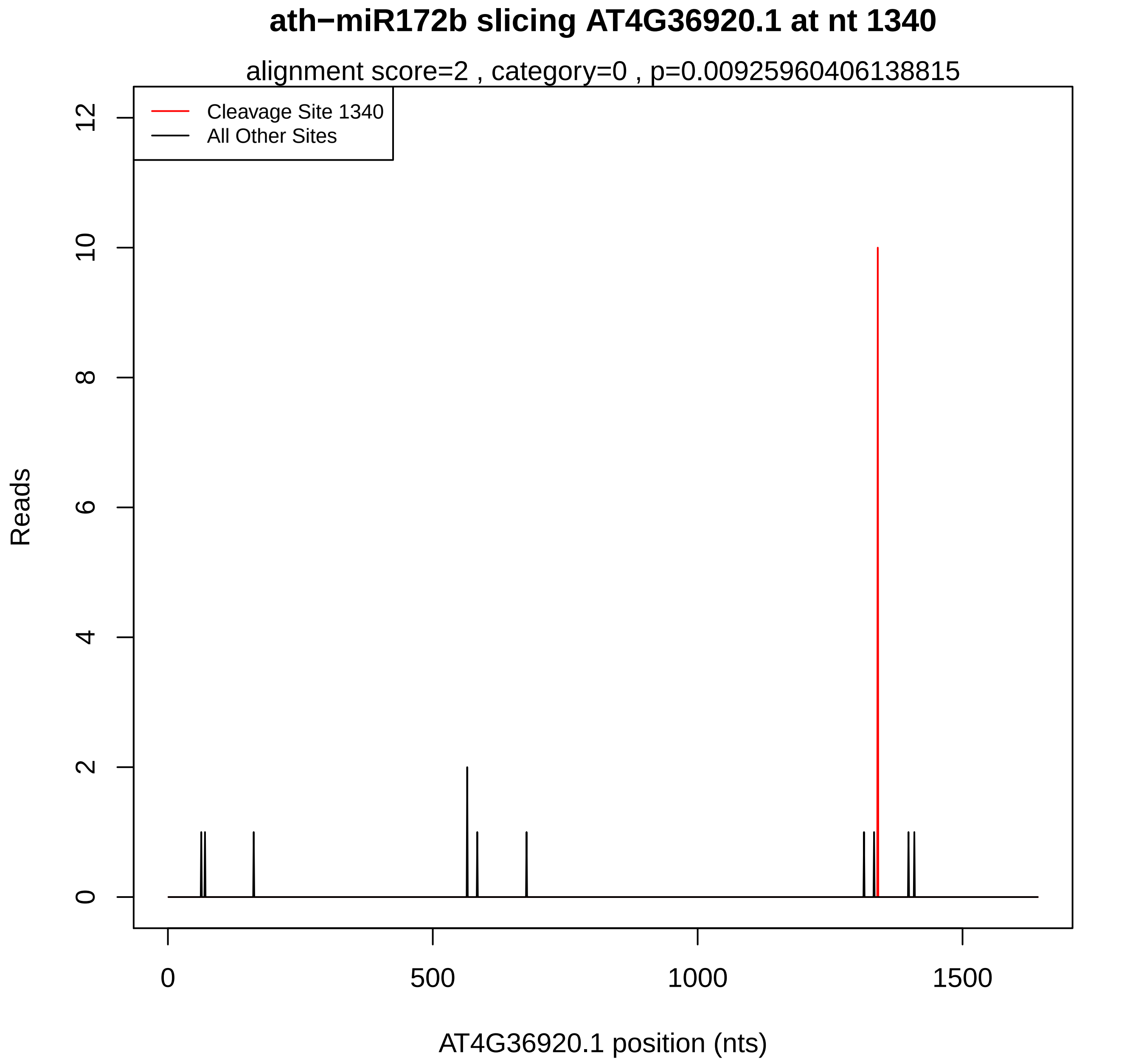
ath−miR172b slicing AT4G36920.1 at nt 1340
alignment score=2 , category=0 , p=0.00925960406138815
Cleavage Site 1340
All Other Sites
Reads
0
2
4
6
8
10
12
0
500
1000
1500
AT4G36920.1 position (nts)

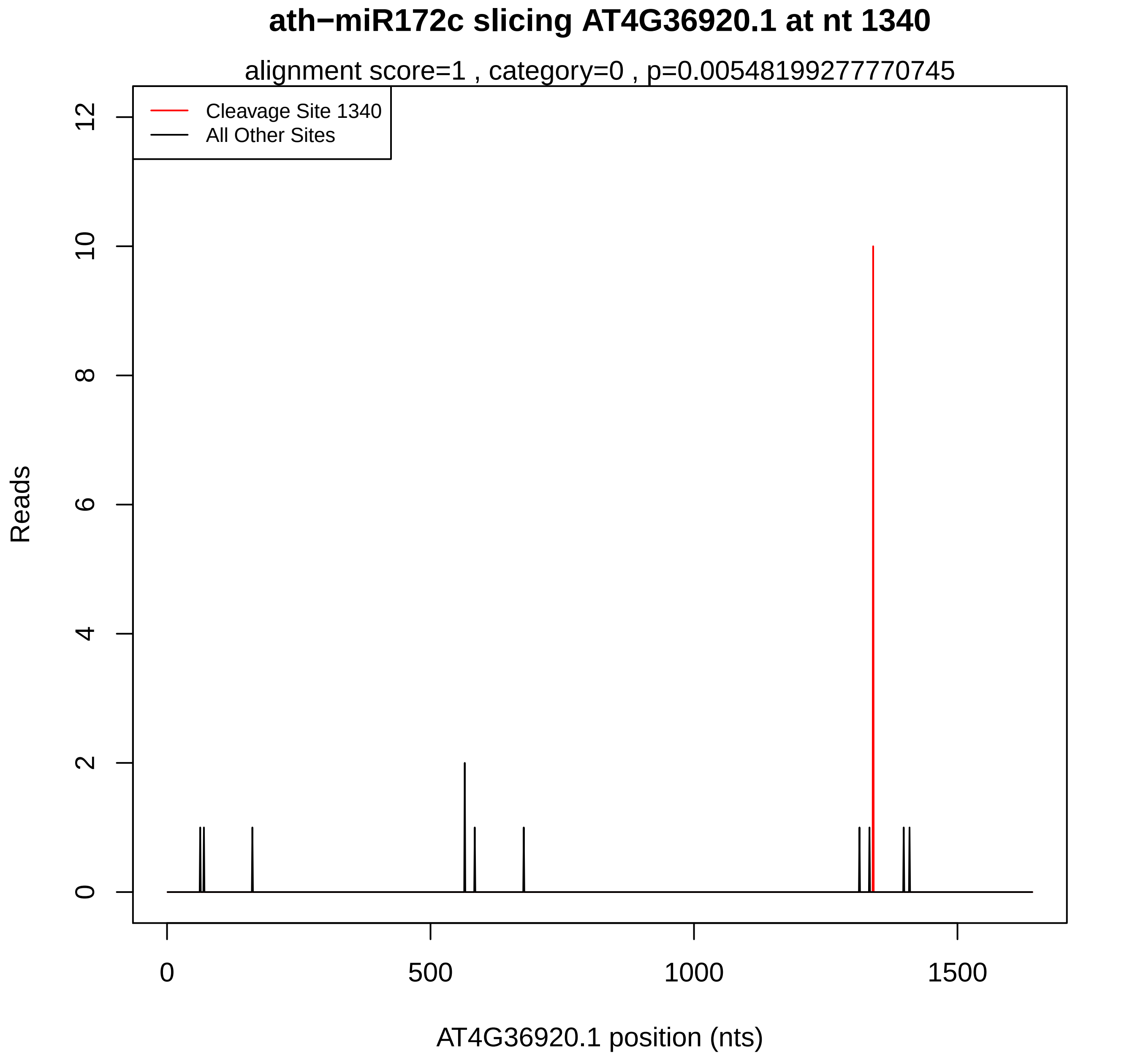
ath−miR172c slicing AT4G36920.1 at nt 1340
alignment score=1 , category=0 , p=0.00548199277770745
Cleavage Site 1340
All Other Sites
Reads
0
2
4
6
8
10
12
0
500
1000
1500
AT4G36920.1 position (nts)

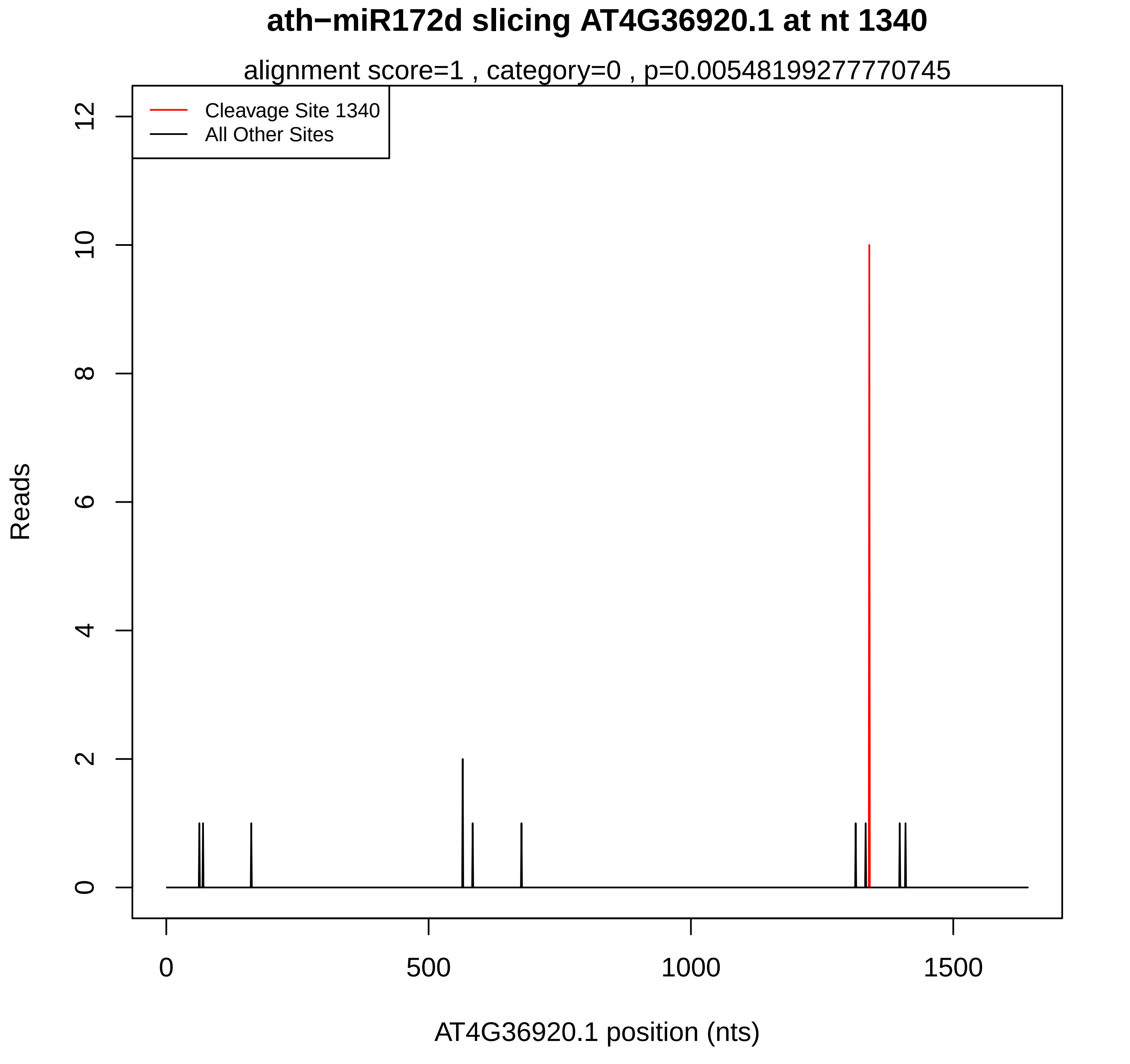

ath−miR172d slicing AT4G36920.1 at nt 1340
alignment score=1 , category=0 , p=0.00548199277770745
Cleavage Site 1340
All Other Sites
Reads
0
2
4
6
8
10
12
0
500
1000
1500
AT4G36920.1 position (nts)

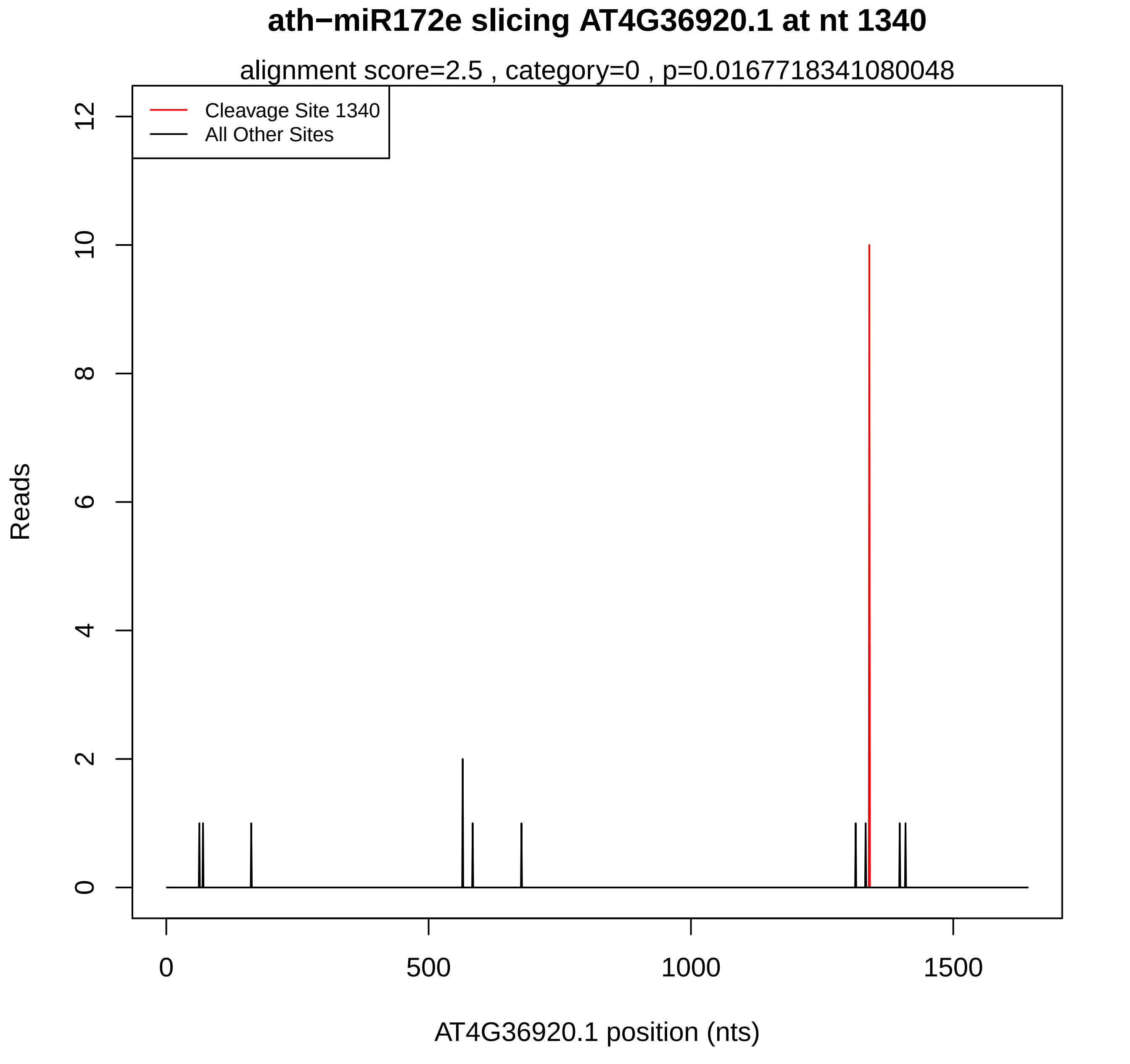

ath−miR172e slicing AT4G36920.1 at nt 1340
alignment score=2.5 , category=0 , p=0.0167718341080048
Cleavage Site 1340
All Other Sites
Reads
0
2
4
6
8
10
12
0
500
1000
1500
AT4G36920.1 position (nts)

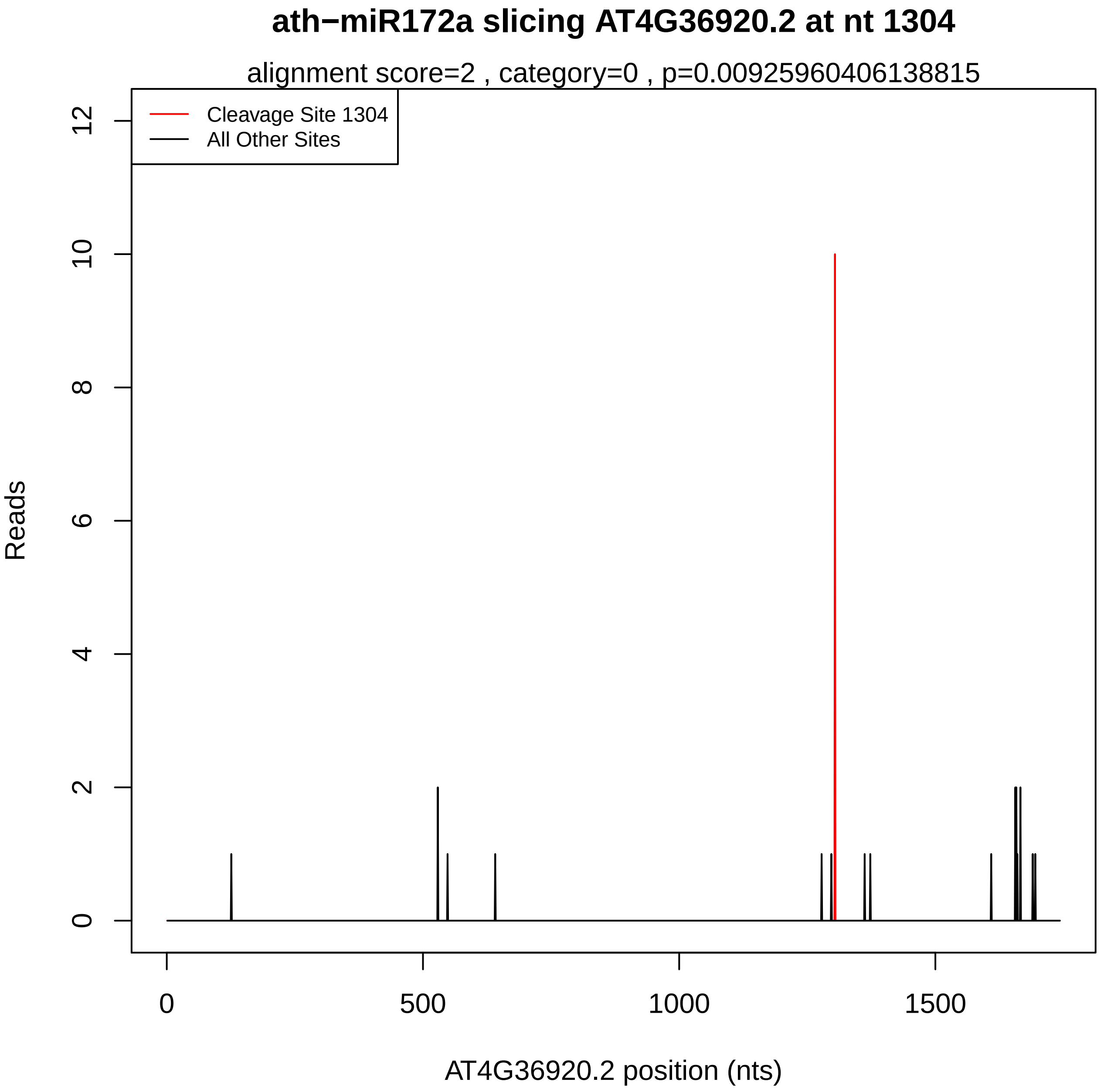
ath−miR172a slicing AT4G36920.2 at nt 1304
alignment score=2 , category=0 , p=0.00925960406138815
Cleavage Site 1304
All Other Sites
Reads
0
2
4
6
8
10
12
0
500
1000
1500
AT4G36920.2 position (nts)

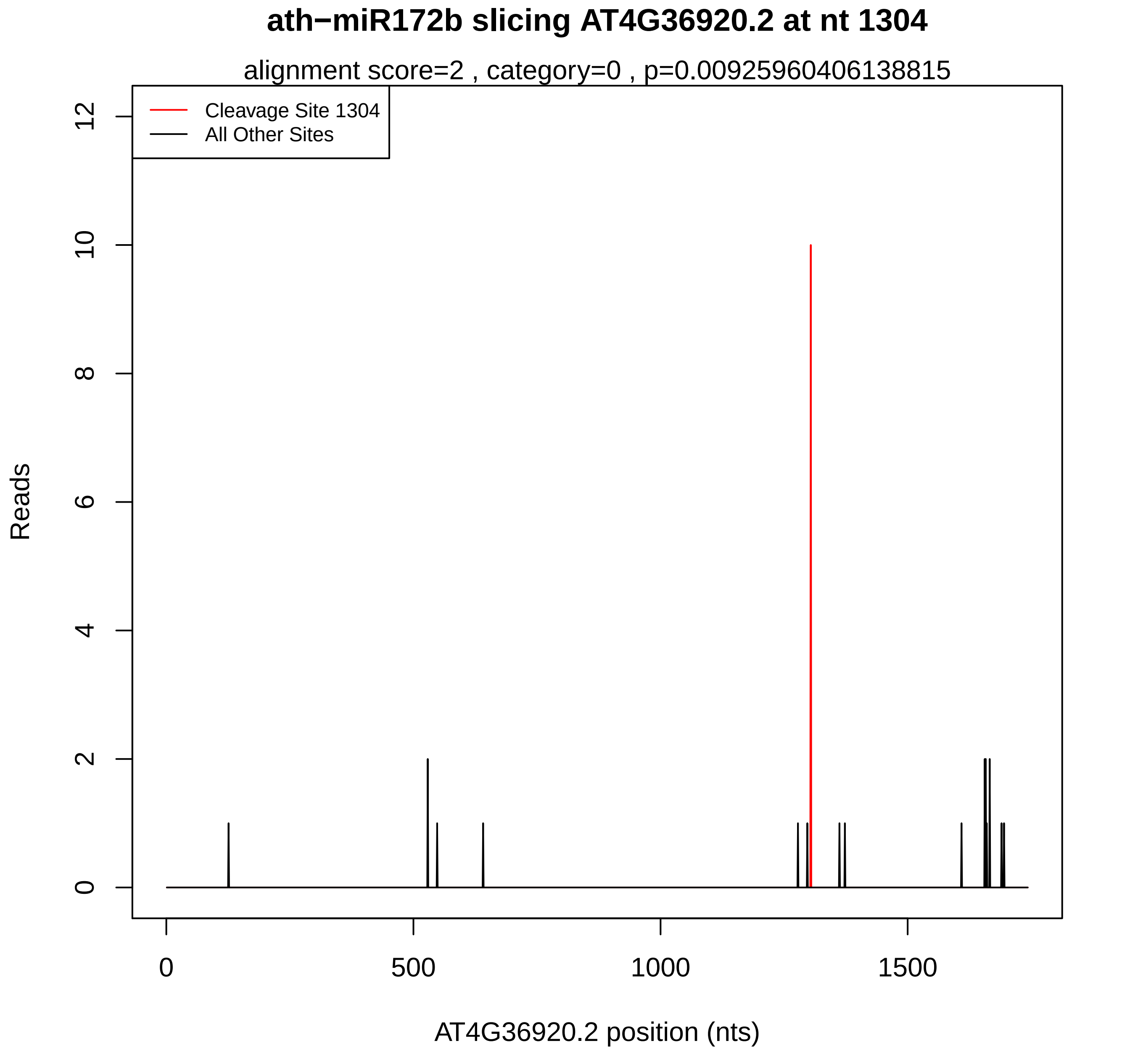

ath−miR172b slicing AT4G36920.2 at nt 1304
alignment score=2 , category=0 , p=0.00925960406138815
Cleavage Site 1304
All Other Sites
Reads
0
2
4
6
8
10
12
0
500
1000
1500
AT4G36920.2 position (nts)

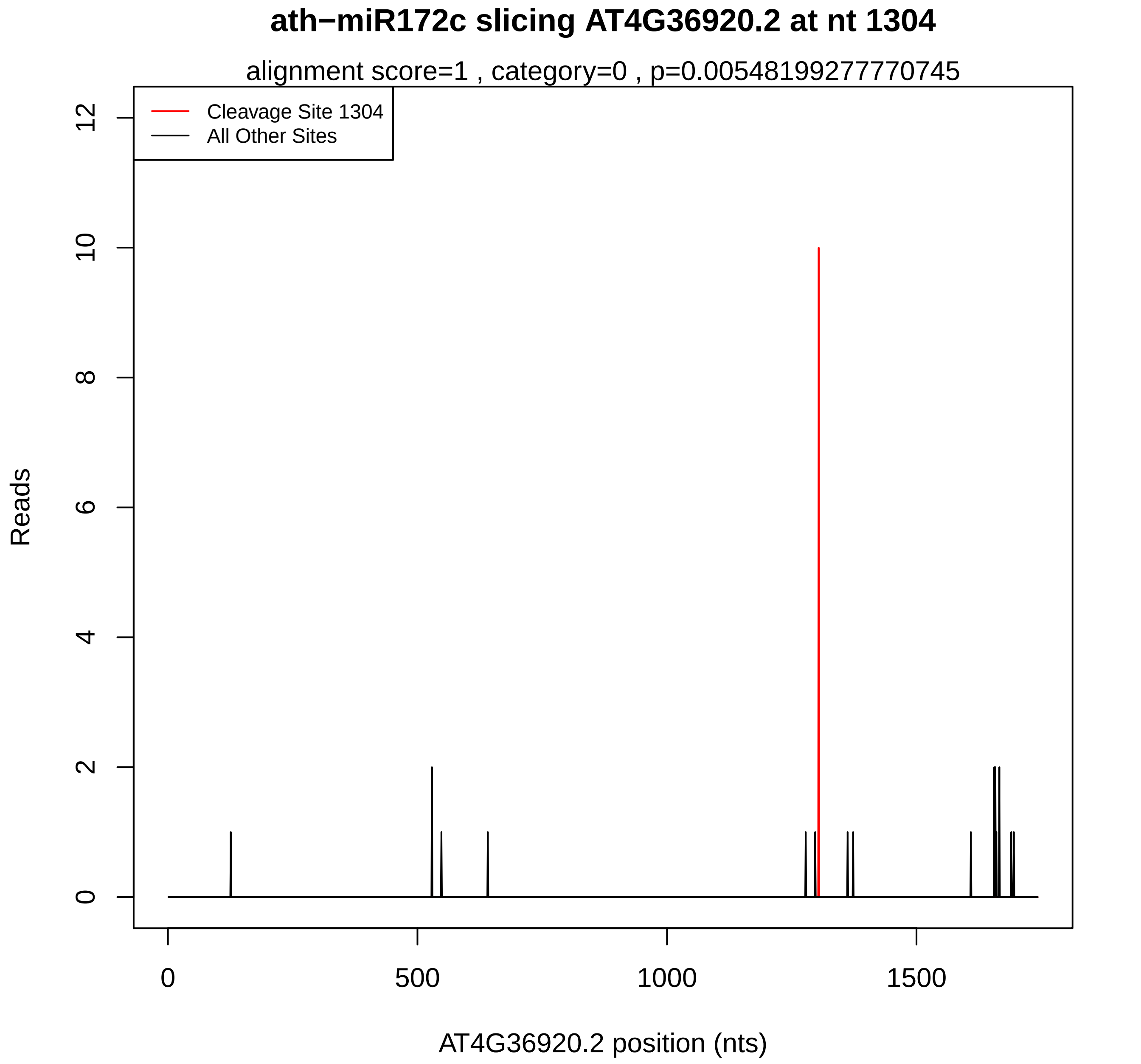

ath−miR172c slicing AT4G36920.2 at nt 1304
alignment score=1 , category=0 , p=0.00548199277770745
Cleavage Site 1304
All Other Sites
Reads
0
2
4
6
8
10
12
0
500
1000
1500
AT4G36920.2 position (nts)

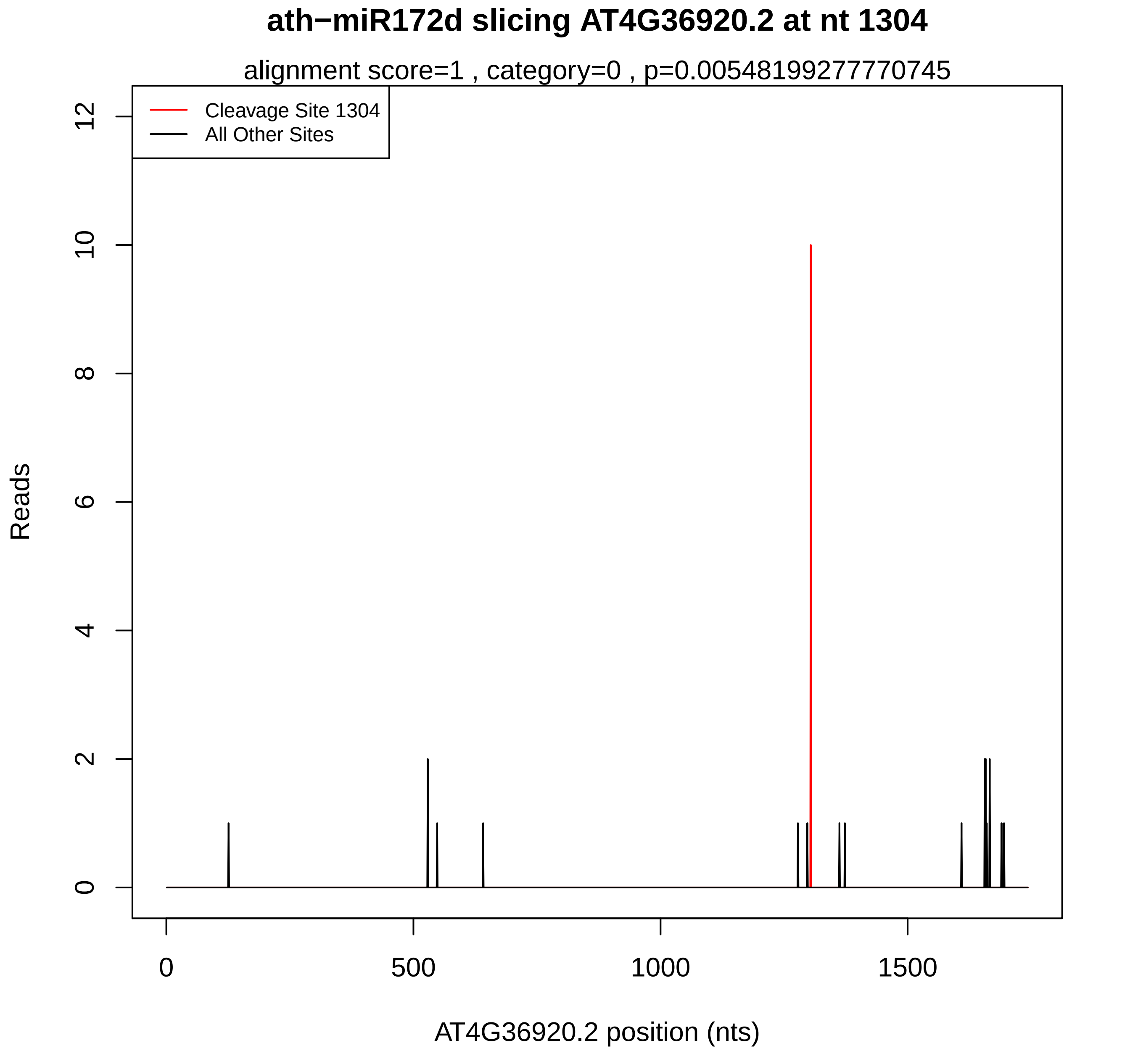

ath−miR172d slicing AT4G36920.2 at nt 1304
alignment score=1 , category=0 , p=0.00548199277770745
Cleavage Site 1304
All Other Sites
Reads
0
2
4
6
8
10
12
0
500
1000
1500
AT4G36920.2 position (nts)

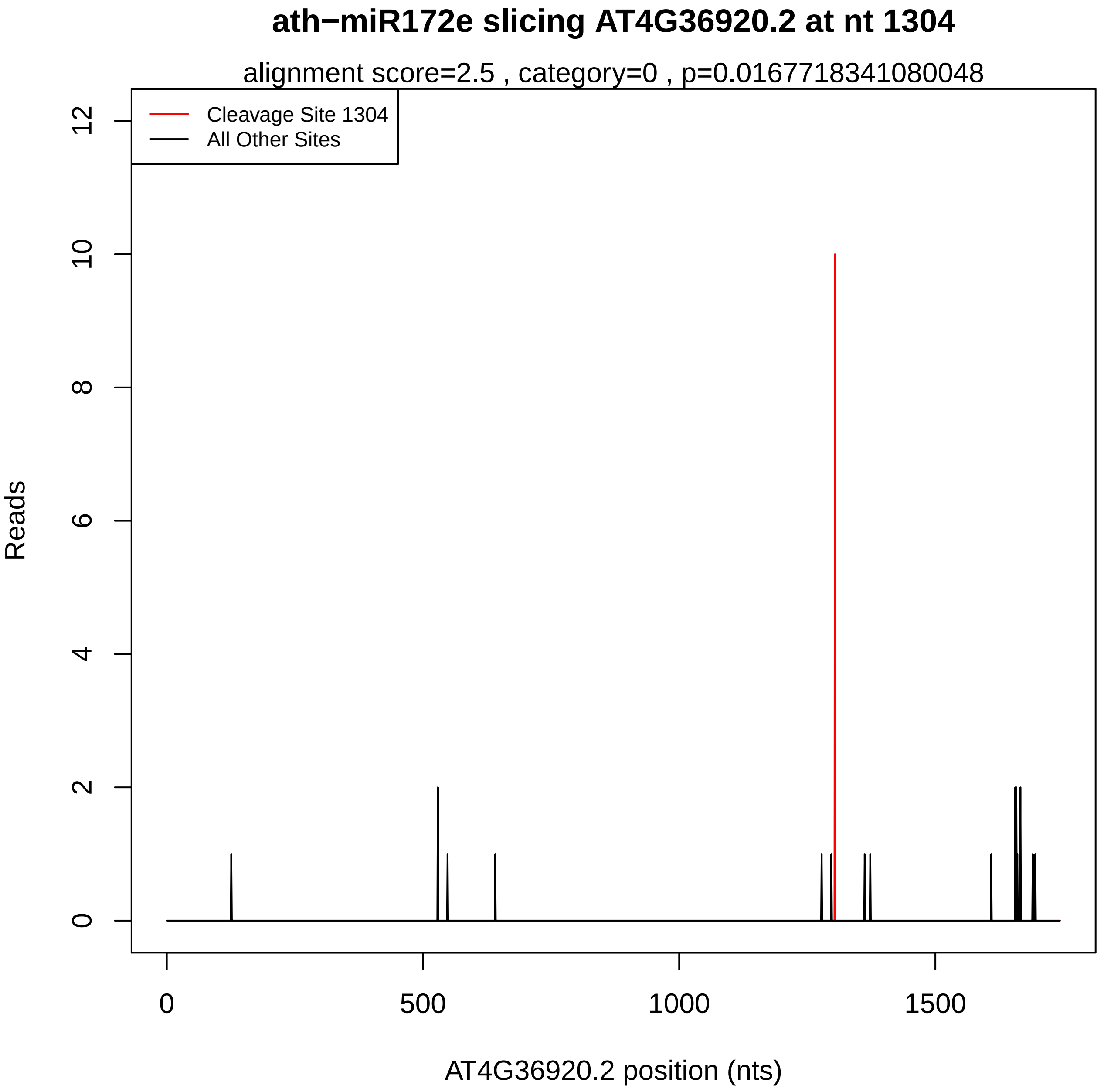
ath−miR172e slicing AT4G36920.2 at nt 1304
alignment score=2.5 , category=0 , p=0.0167718341080048
Cleavage Site 1304
All Other Sites
Reads
0
2
4
6
8
10
12
0
500
1000
1500
AT4G36920.2 position (nts)

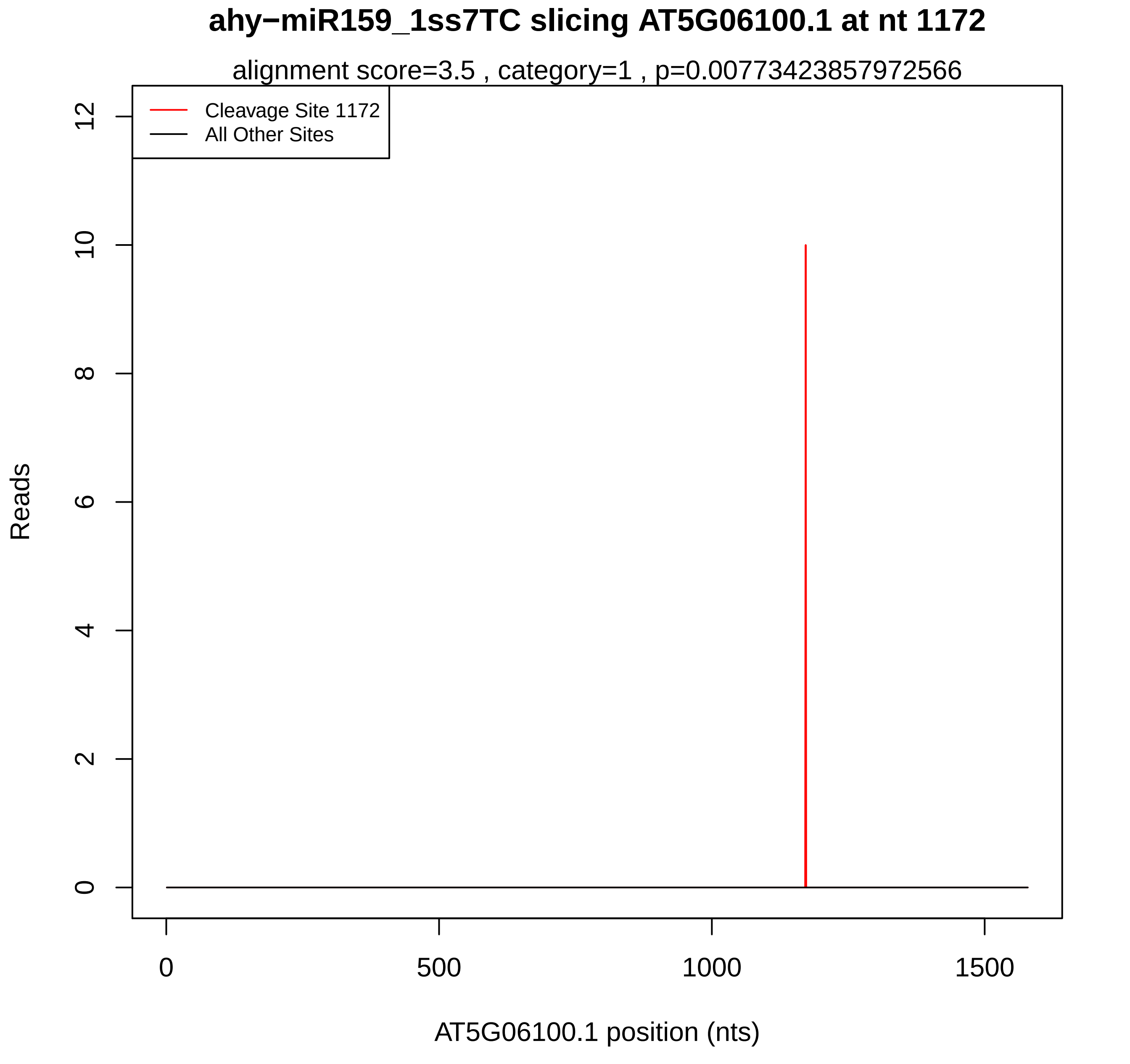
ahy−miR159_1ss7TC slicing AT5G06100.1 at nt 1172
alignment score=3.5 , category=1 , p=0.00773423857972566
Cleavage Site 1172
All Other Sites
Reads
0
2
4
6
8
10
12
0
500
1000
1500
AT5G06100.1 position (nts)

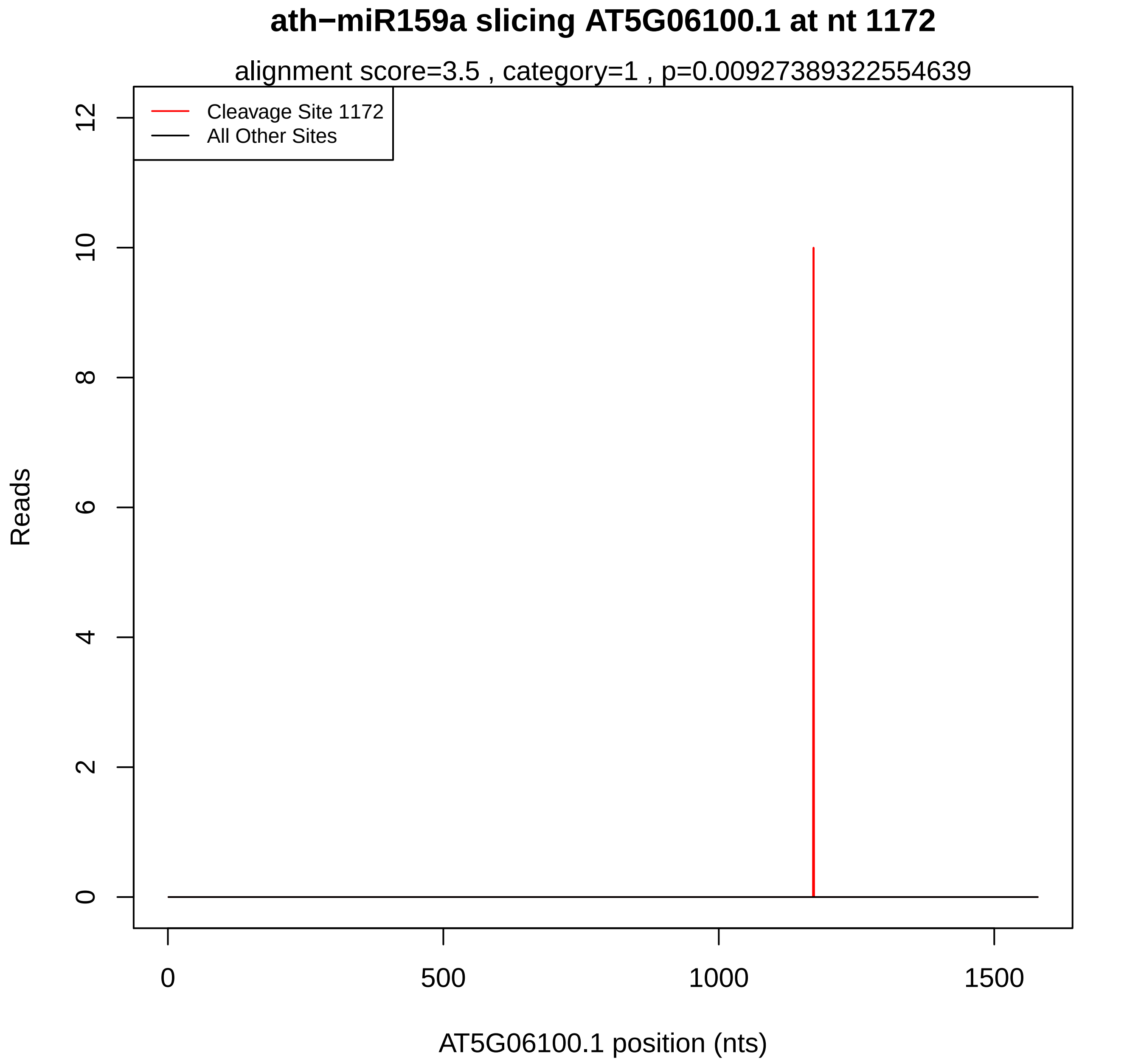

ath−miR159a slicing AT5G06100.1 at nt 1172
alignment score=3.5 , category=1 , p=0.00927389322554639
Cleavage Site 1172
All Other Sites
Reads
0
2
4
6
8
10
12
0
500
1000
1500
AT5G06100.1 position (nts)

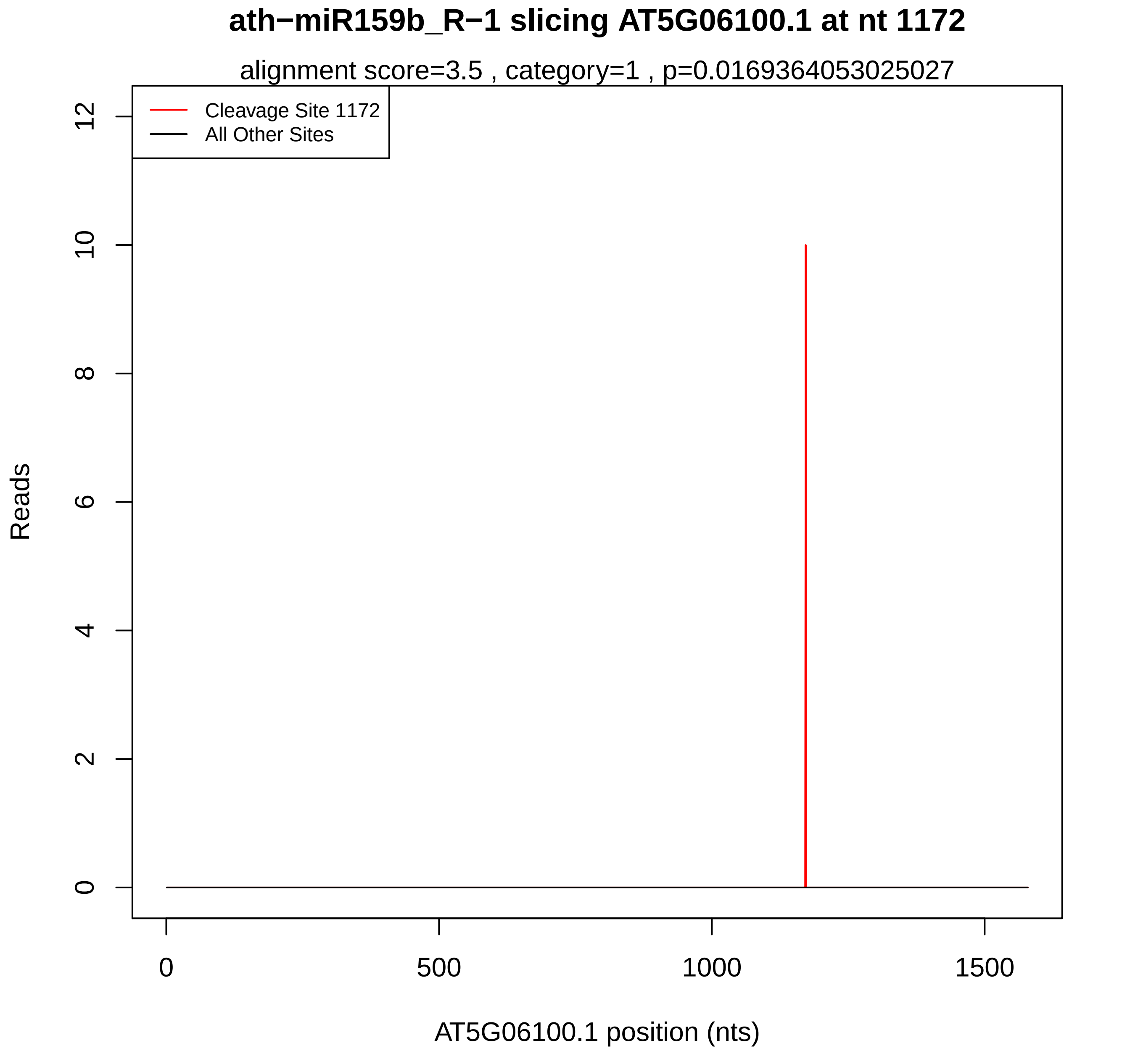
ath−miR159b_R−1 slicing AT5G06100.1 at nt 1172
alignment score=3.5 , category=1 , p=0.0169364053025027
Cleavage Site 1172
All Other Sites
Reads
0
2
4
6
8
10
12
0
500
1000
1500
AT5G06100.1 position (nts)

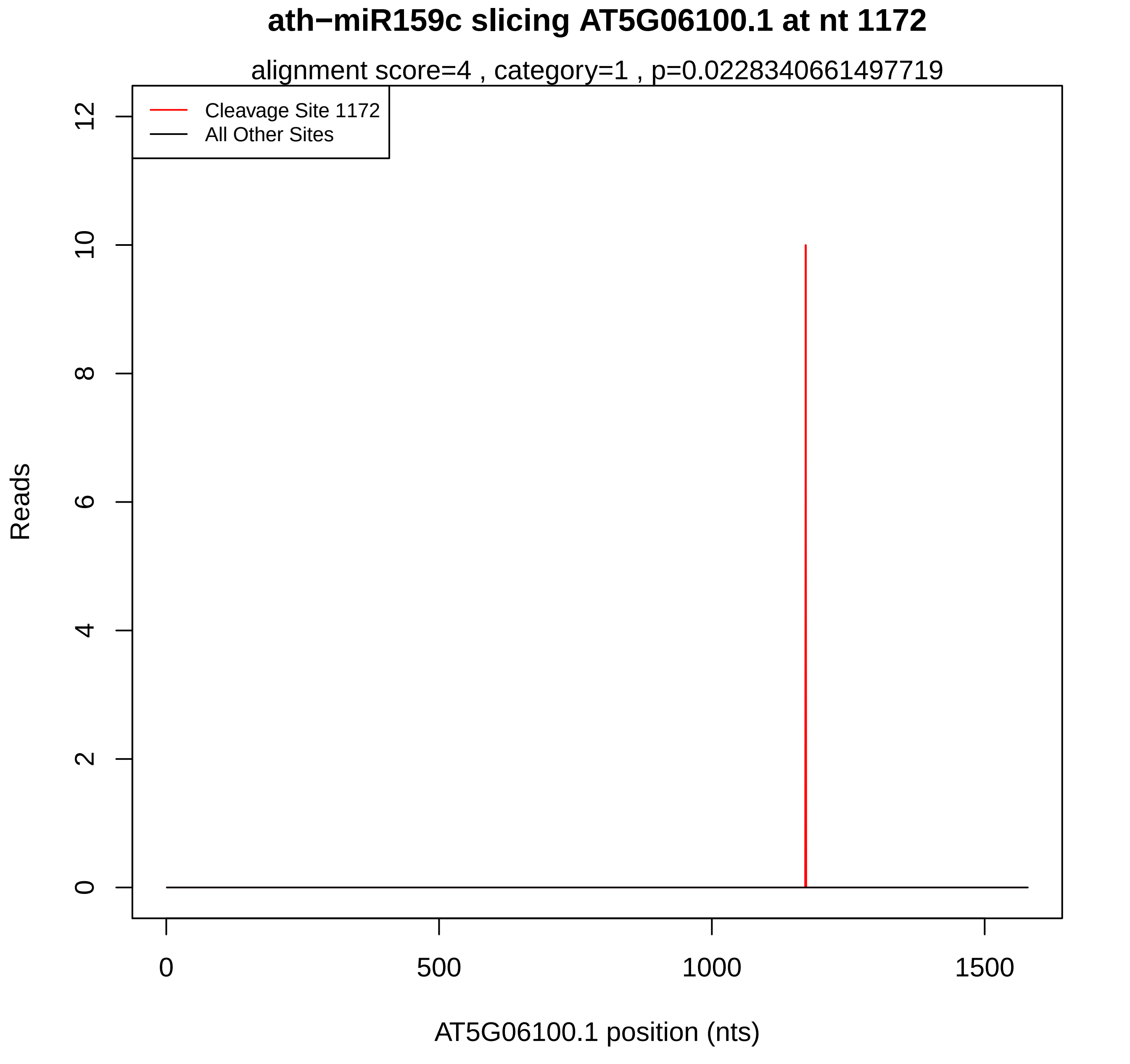
ath−miR159c slicing AT5G06100.1 at nt 1172
alignment score=4 , category=1 , p=0.0228340661497719
Cleavage Site 1172
All Other Sites
Reads
0
2
4
6
8
10
12
0
500
1000
1500
AT5G06100.1 position (nts)

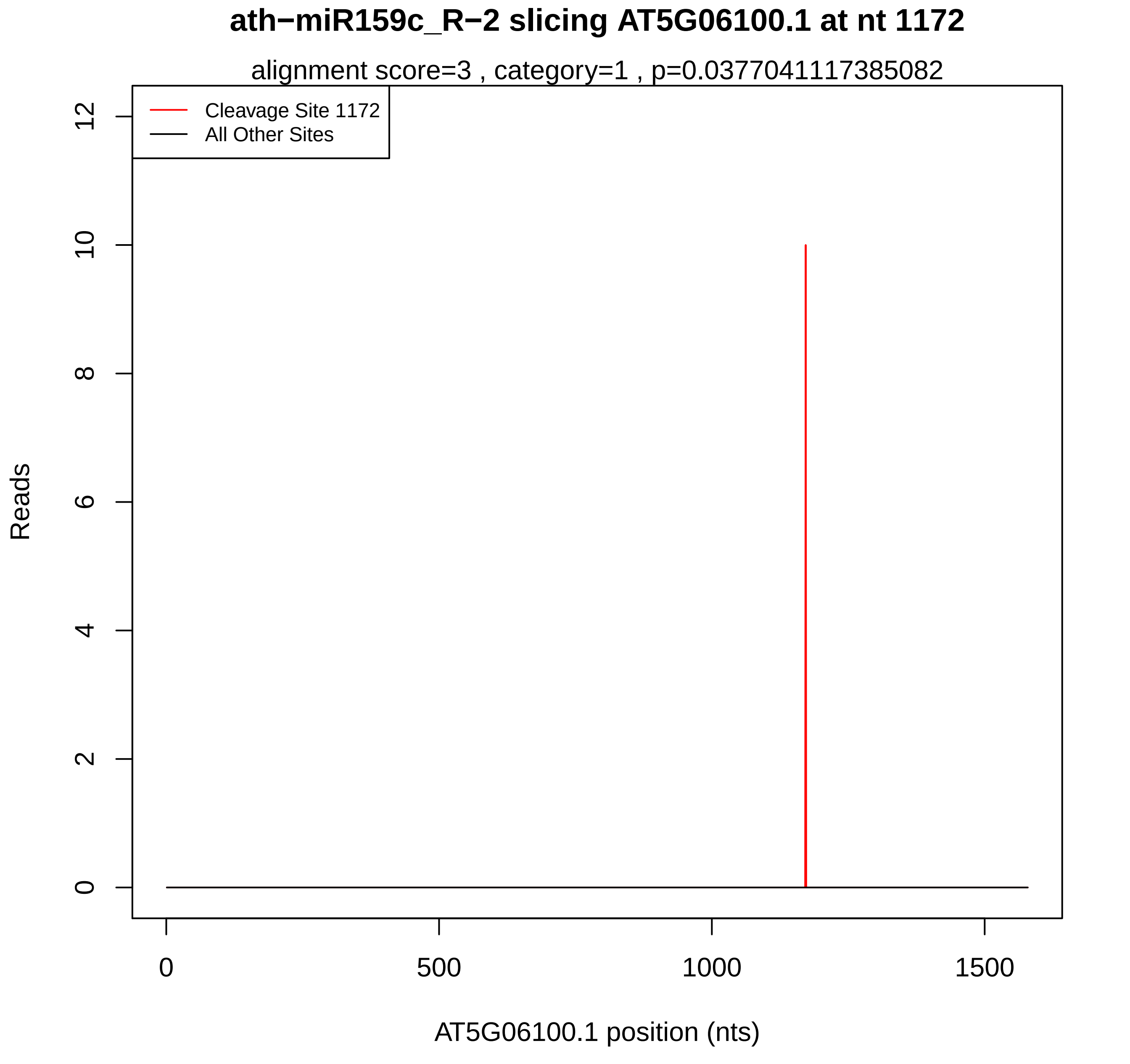

ath−miR159c_R−2 slicing AT5G06100.1 at nt 1172
alignment score=3 , category=1 , p=0.0377041117385082
Cleavage Site 1172
All Other Sites
Reads
0
2
4
6
8
10
12
0
500
1000
1500
AT5G06100.1 position (nts)

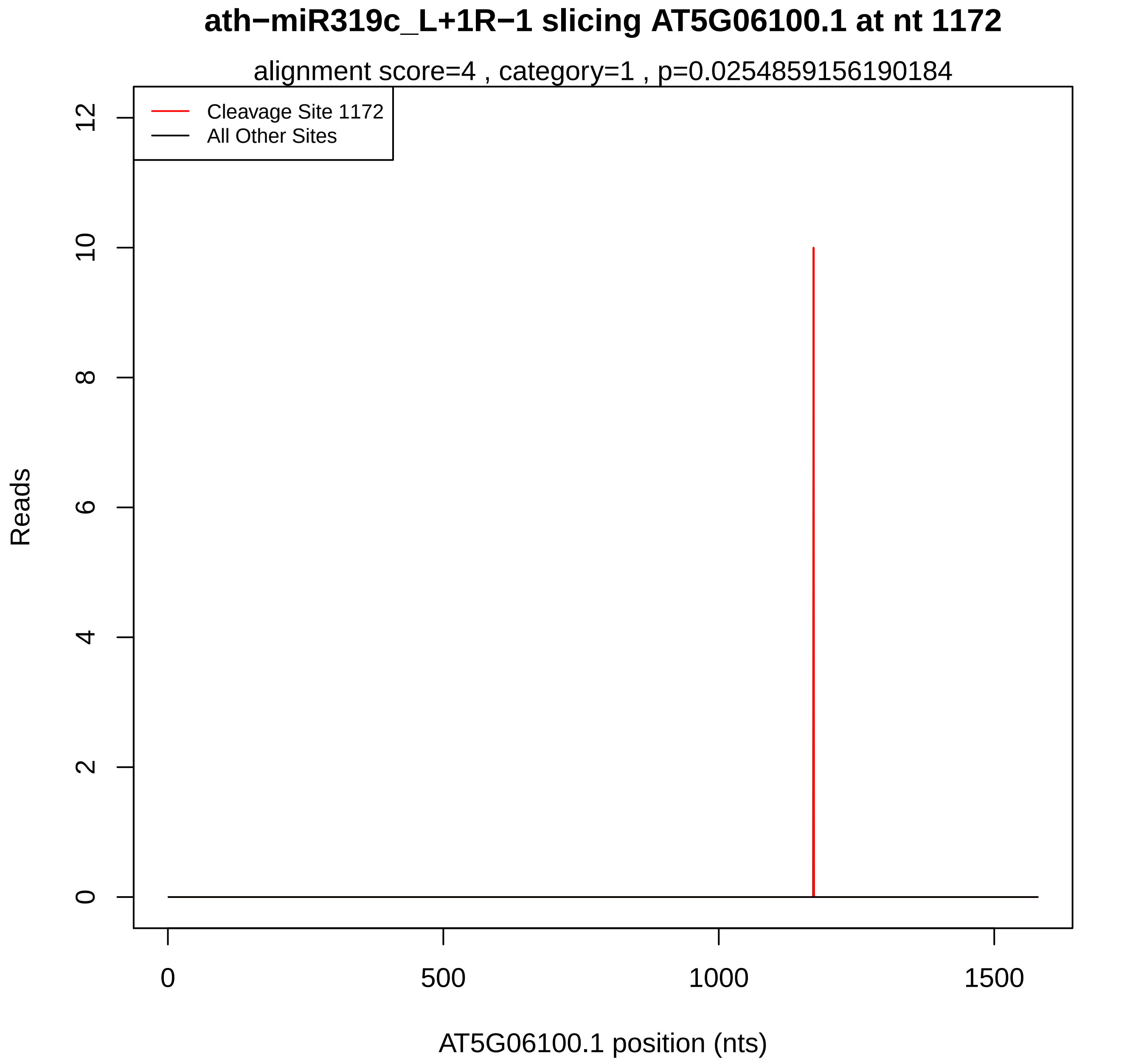
ath−miR319c_L+1R−1 slicing AT5G06100.1 at nt 1172
alignment score=4 , category=1 , p=0.0254859156190184
Cleavage Site 1172
All Other Sites
Reads
0
2
4
6
8
10
12
0
500
1000
1500
AT5G06100.1 position (nts)

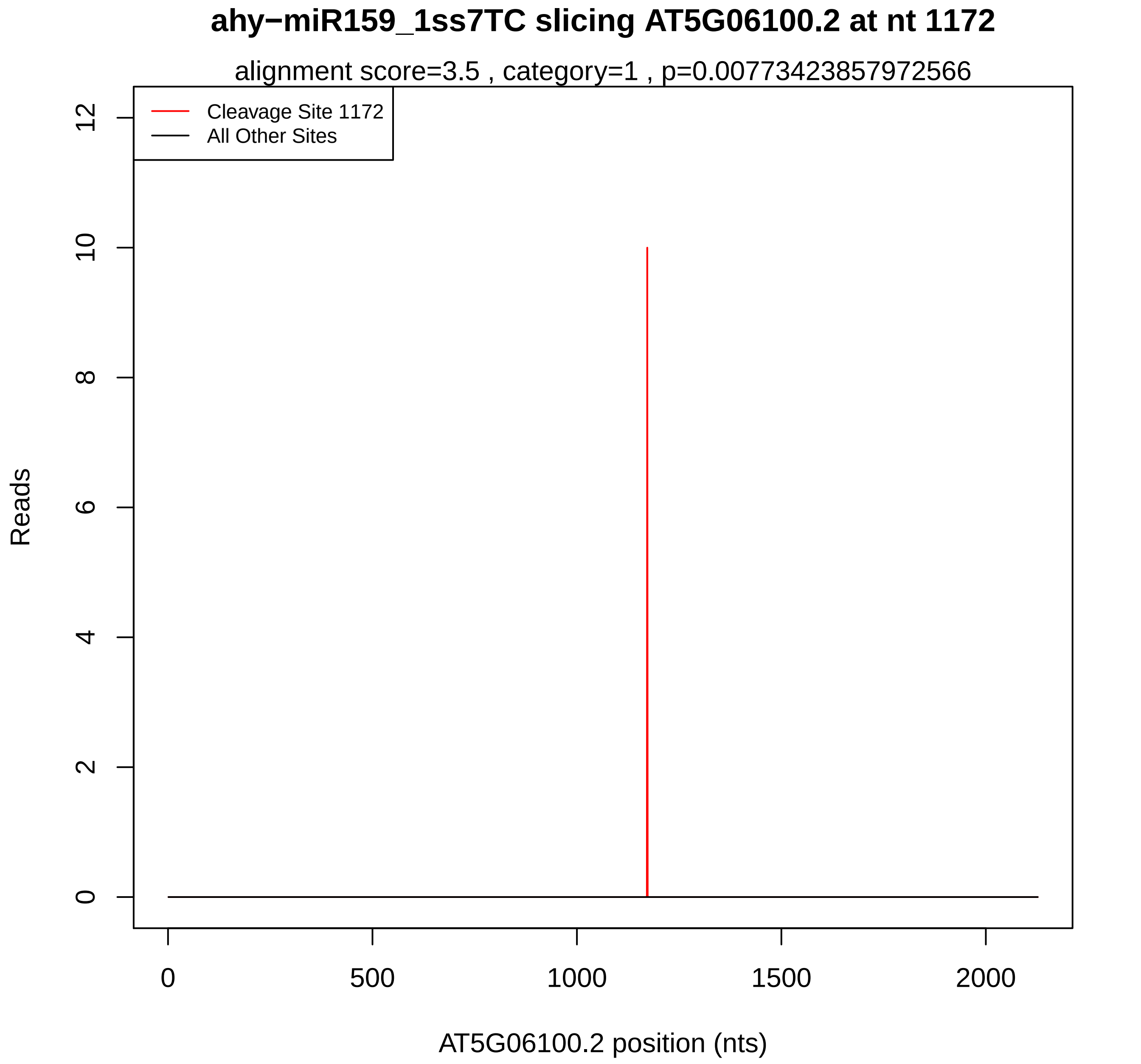
ahy−miR159_1ss7TC slicing AT5G06100.2 at nt 1172
alignment score=3.5 , category=1 , p=0.00773423857972566
Cleavage Site 1172
All Other Sites
Reads
0
2
4
6
8
10
12
0
500
1000
1500
2000
AT5G06100.2 position (nts)

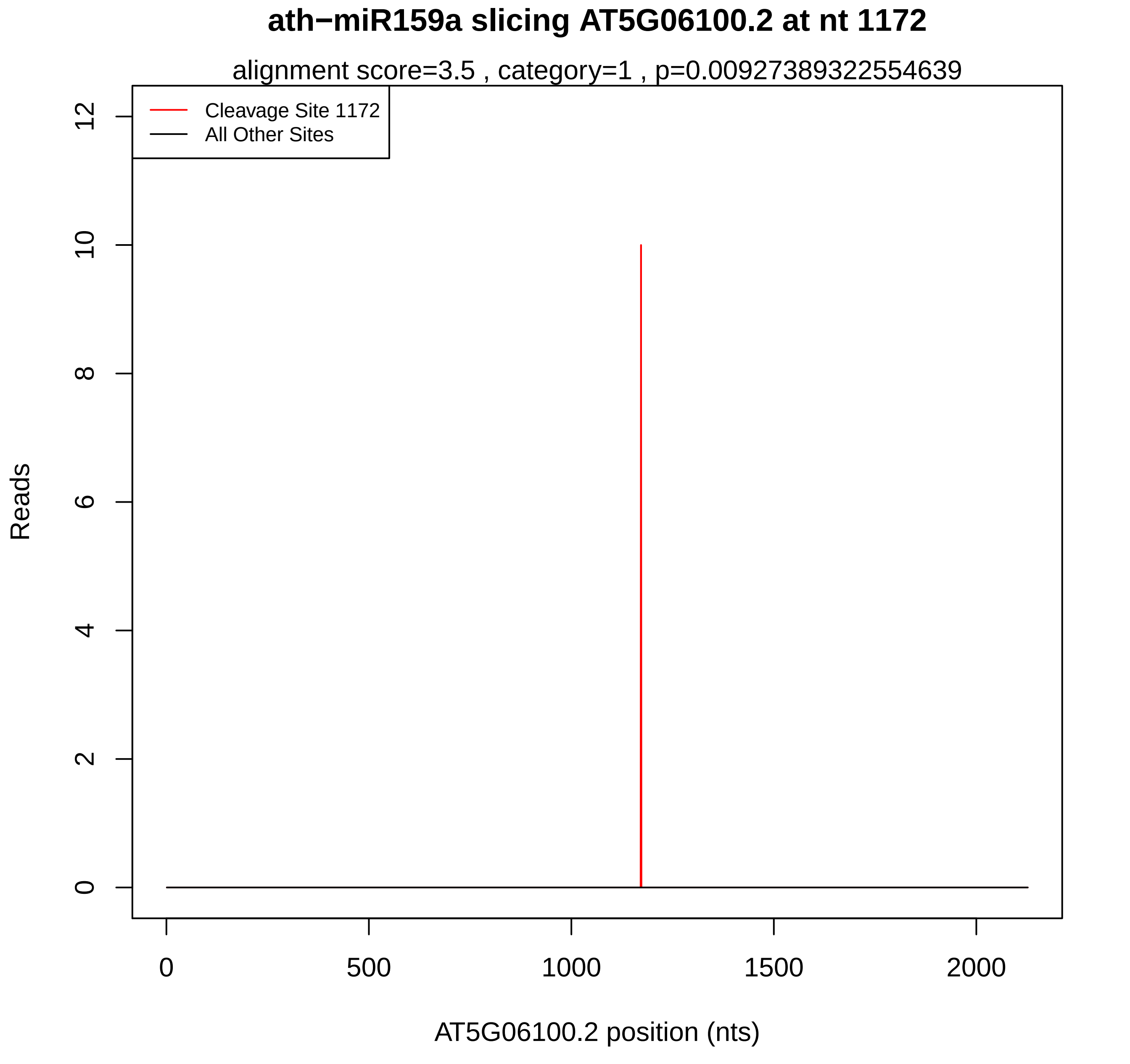

ath−miR159a slicing AT5G06100.2 at nt 1172
alignment score=3.5 , category=1 , p=0.00927389322554639
Cleavage Site 1172
All Other Sites
Reads
0
2
4
6
8
10
12
0
500
1000
1500
2000
AT5G06100.2 position (nts)

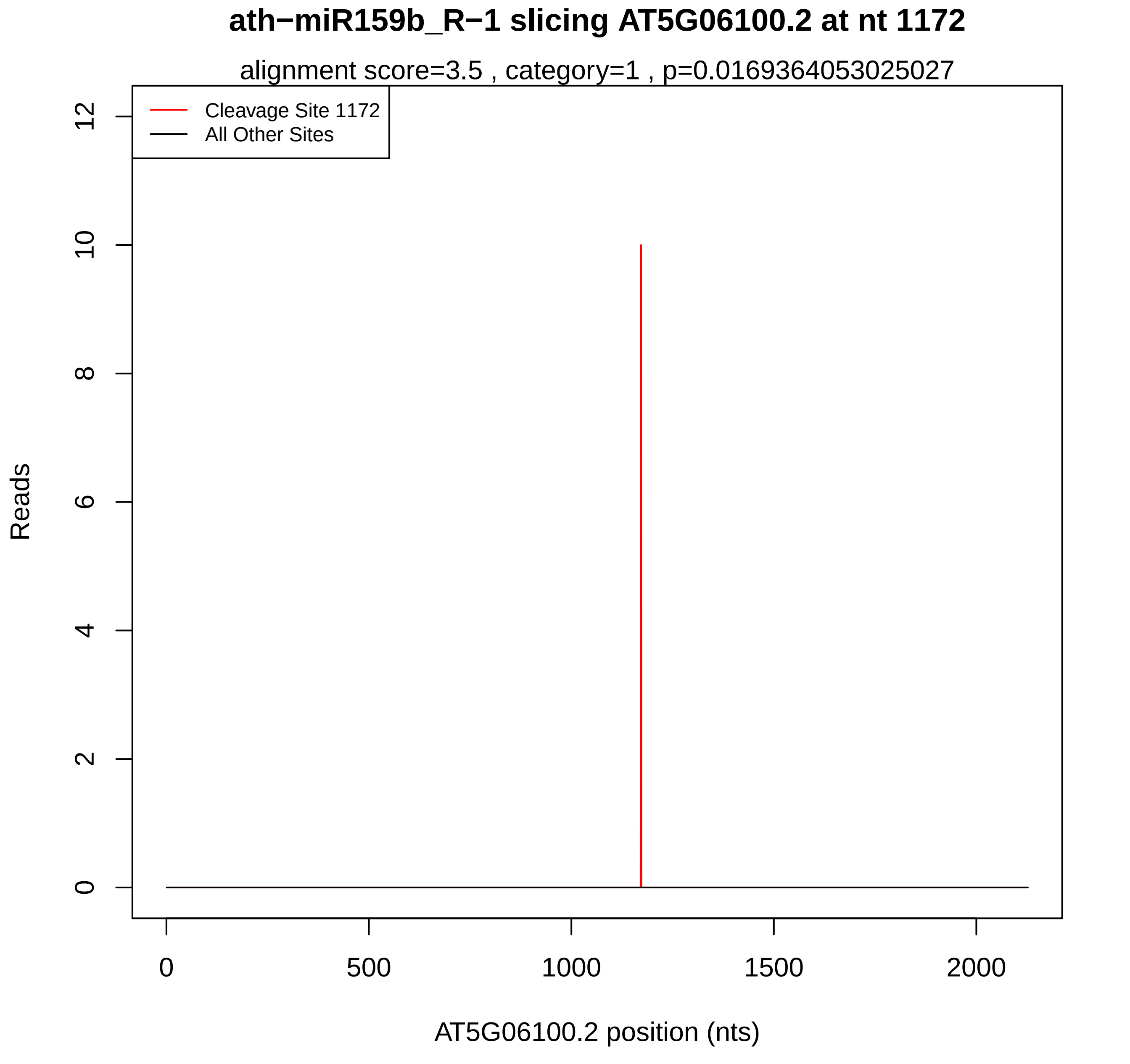
ath−miR159b_R−1 slicing AT5G06100.2 at nt 1172
alignment score=3.5 , category=1 , p=0.0169364053025027
Cleavage Site 1172
All Other Sites
Reads
0
2
4
6
8
10
12
0
500
1000
1500
2000
AT5G06100.2 position (nts)

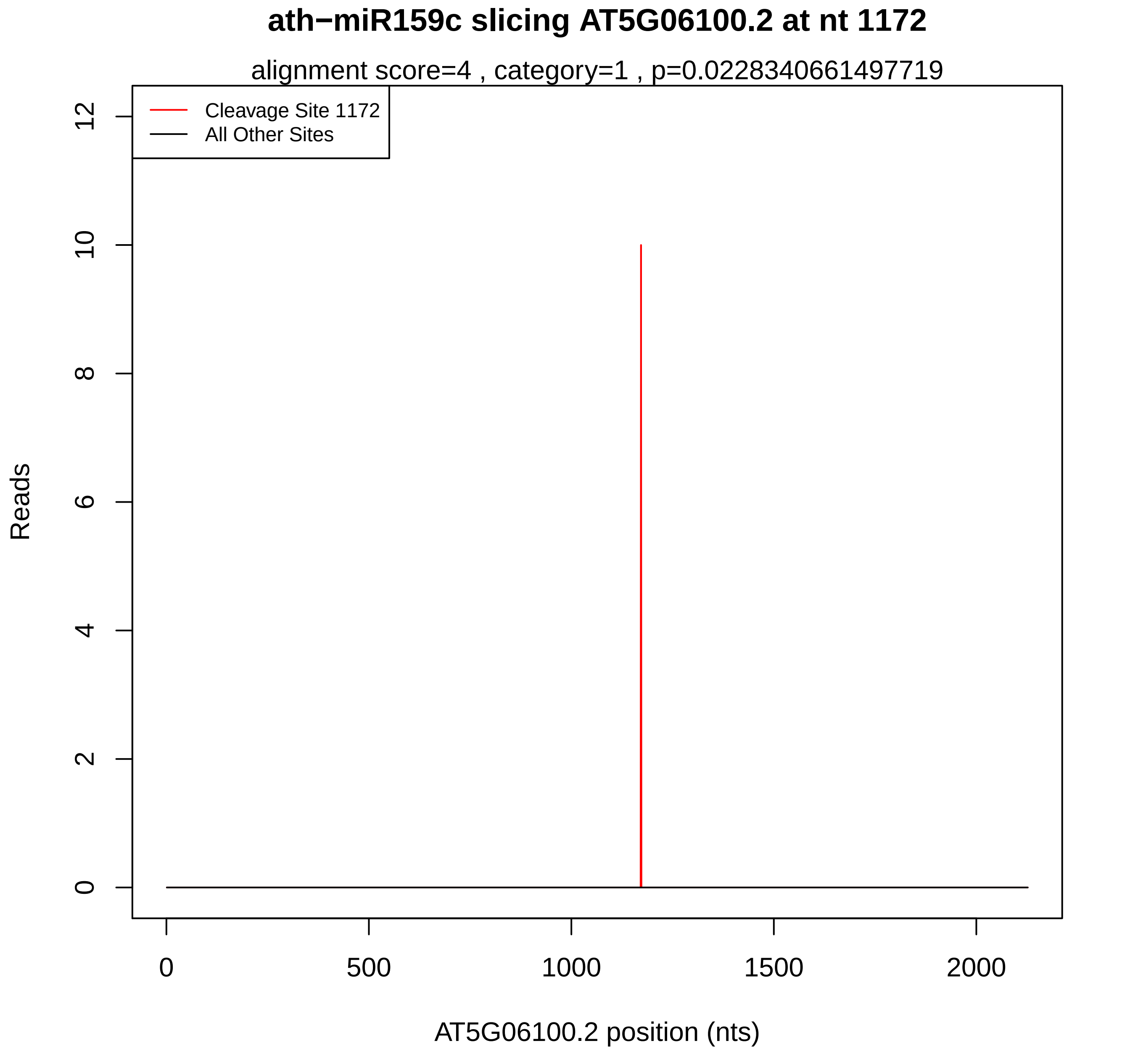
ath−miR159c slicing AT5G06100.2 at nt 1172
alignment score=4 , category=1 , p=0.0228340661497719
Cleavage Site 1172
All Other Sites
Reads
0
2
4
6
8
10
12
0
500
1000
1500
2000
AT5G06100.2 position (nts)

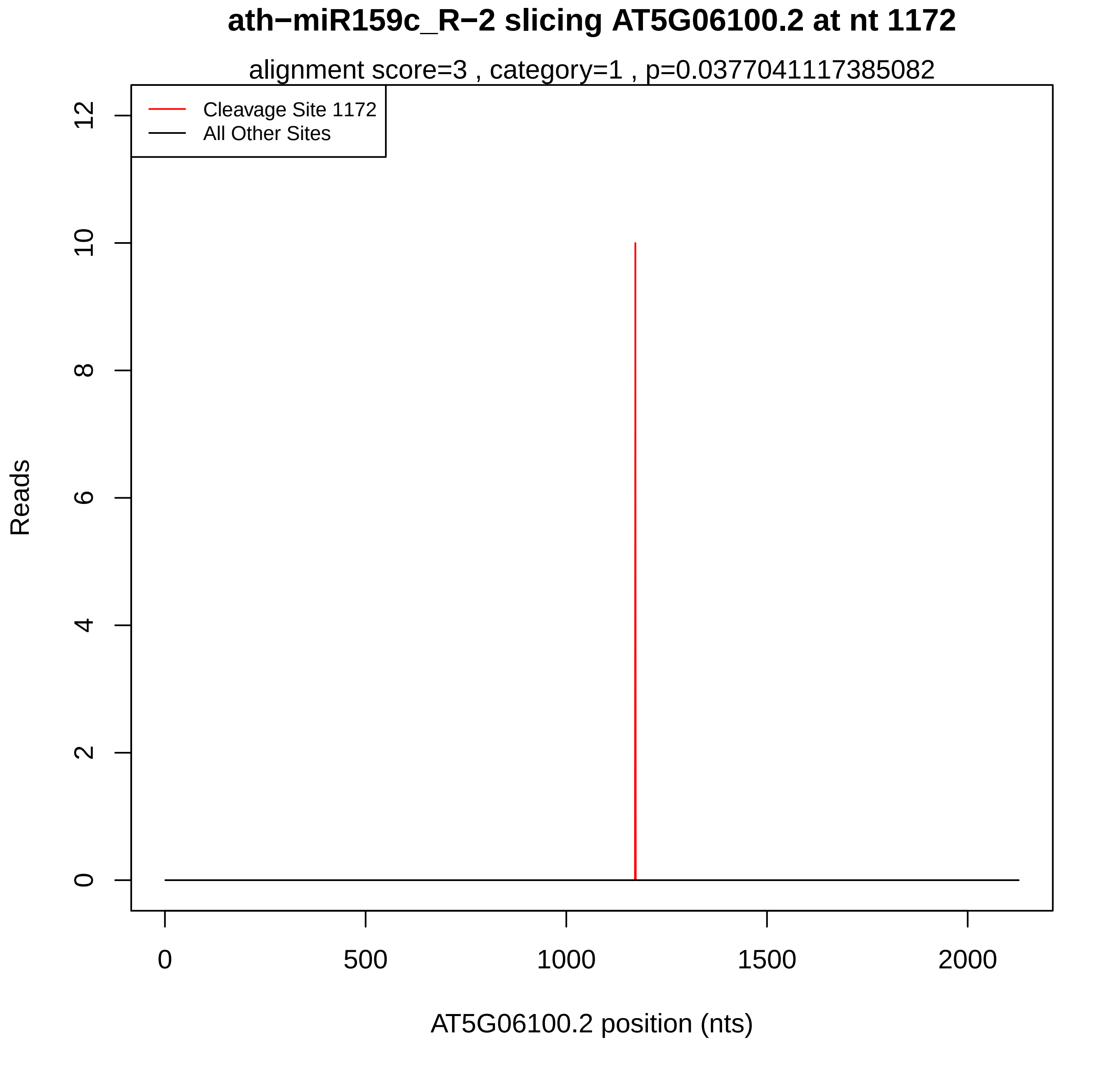

ath−miR159c_R−2 slicing AT5G06100.2 at nt 1172
alignment score=3 , category=1 , p=0.0377041117385082
Cleavage Site 1172
All Other Sites
Reads
0
2
4
6
8
10
12
0
500
1000
1500
2000
AT5G06100.2 position (nts)

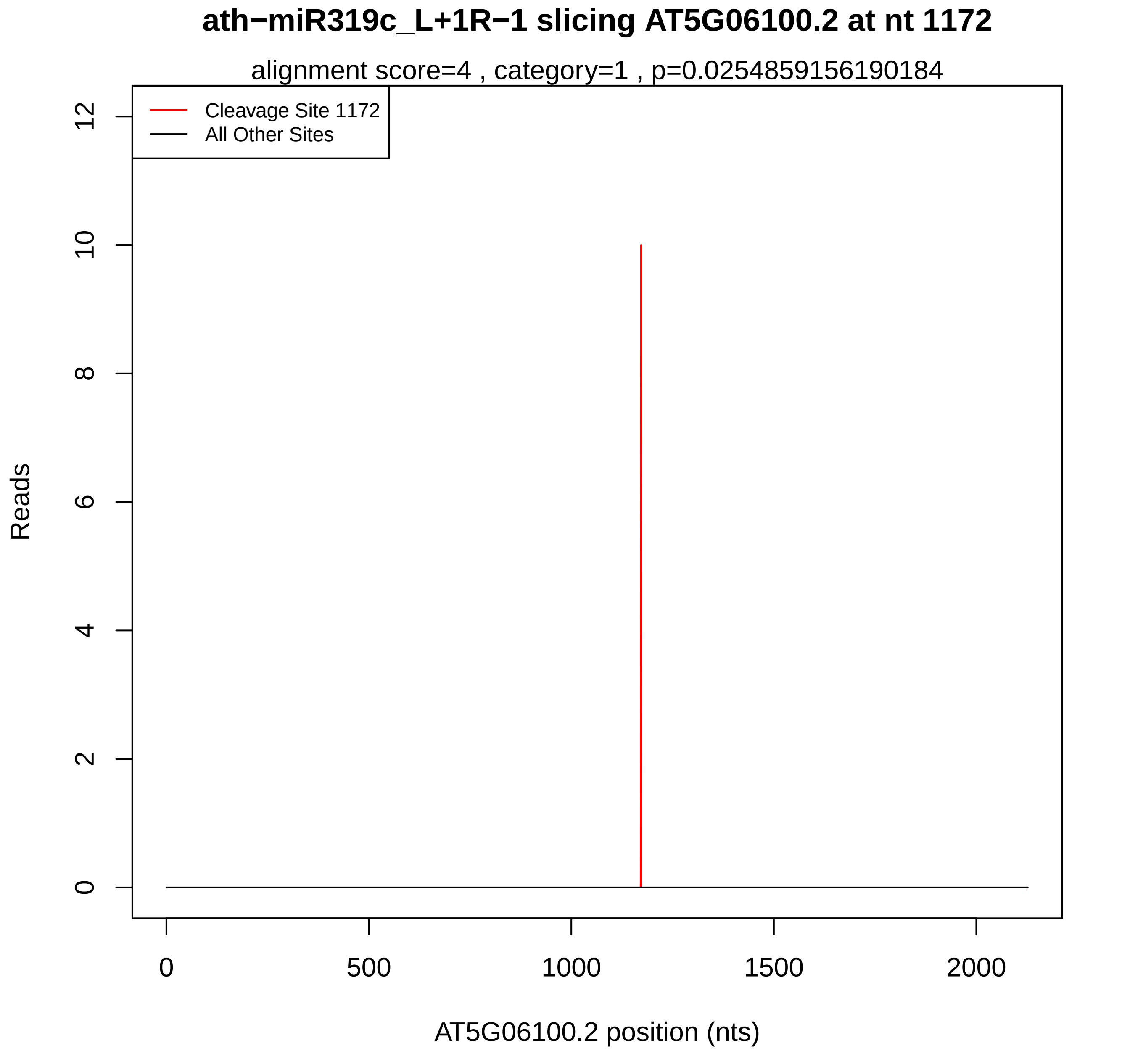
ath−miR319c_L+1R−1 slicing AT5G06100.2 at nt 1172
alignment score=4 , category=1 , p=0.0254859156190184
Cleavage Site 1172
All Other Sites
Reads
0
2
4
6
8
10
12
0
500
1000
1500
2000
AT5G06100.2 position (nts)

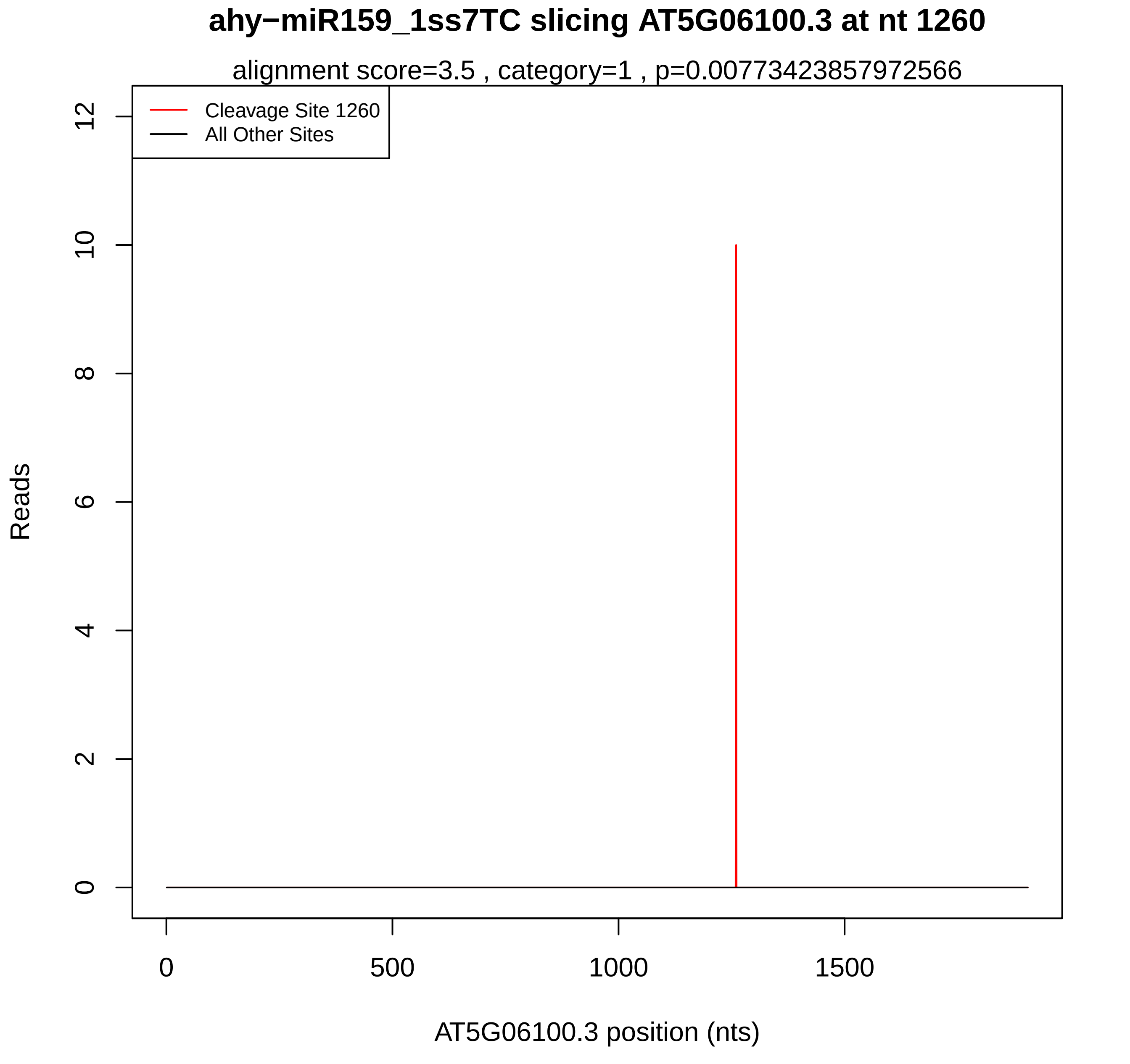

ahy−miR159_1ss7TC slicing AT5G06100.3 at nt 1260
alignment score=3.5 , category=1 , p=0.00773423857972566
Cleavage Site 1260
All Other Sites
Reads
0
2
4
6
8
10
12
0
500
1000
1500
AT5G06100.3 position (nts)

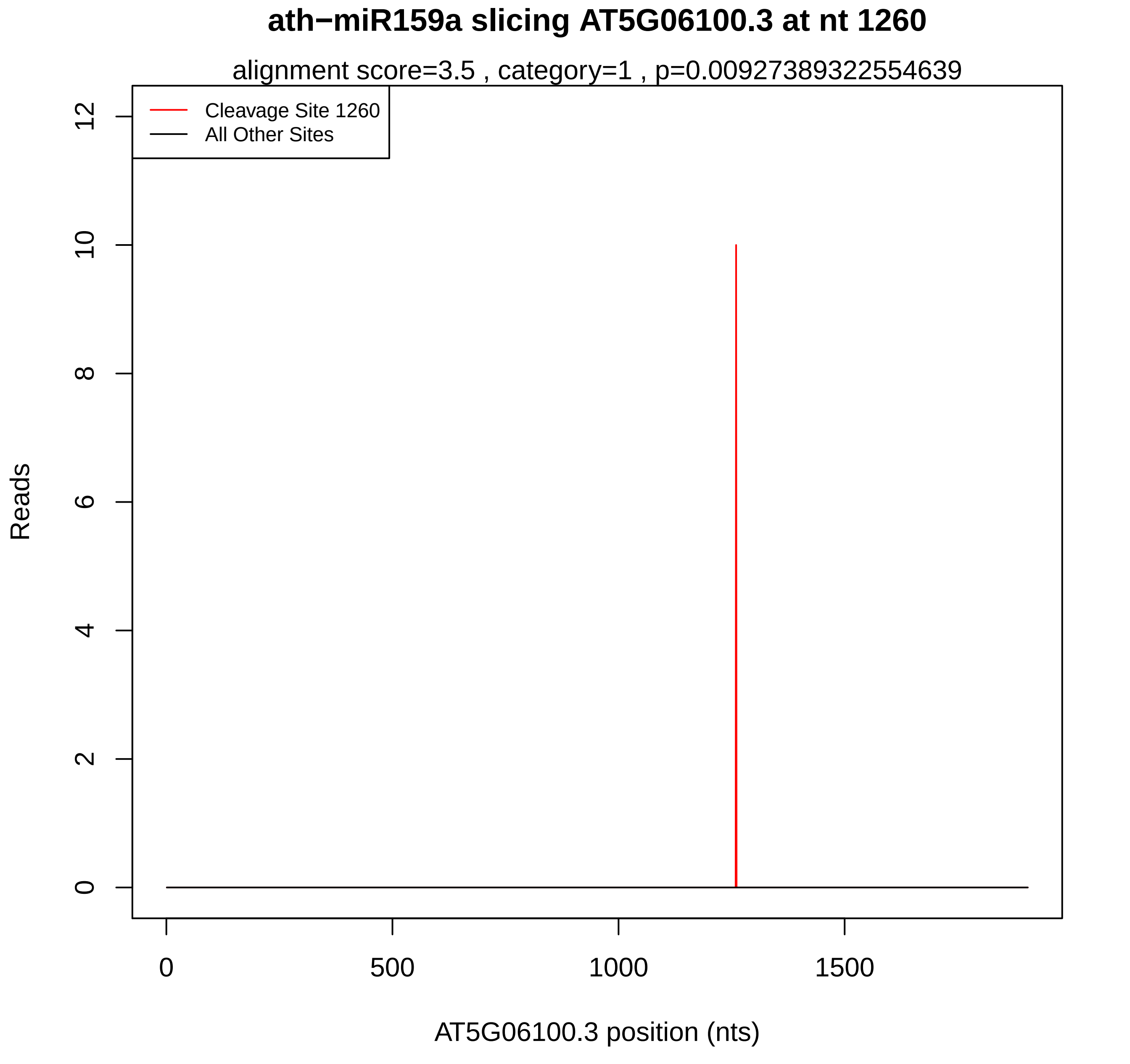
ath−miR159a slicing AT5G06100.3 at nt 1260
alignment score=3.5 , category=1 , p=0.00927389322554639
Cleavage Site 1260
All Other Sites
Reads
0
2
4
6
8
10
12
0
500
1000
1500
AT5G06100.3 position (nts)

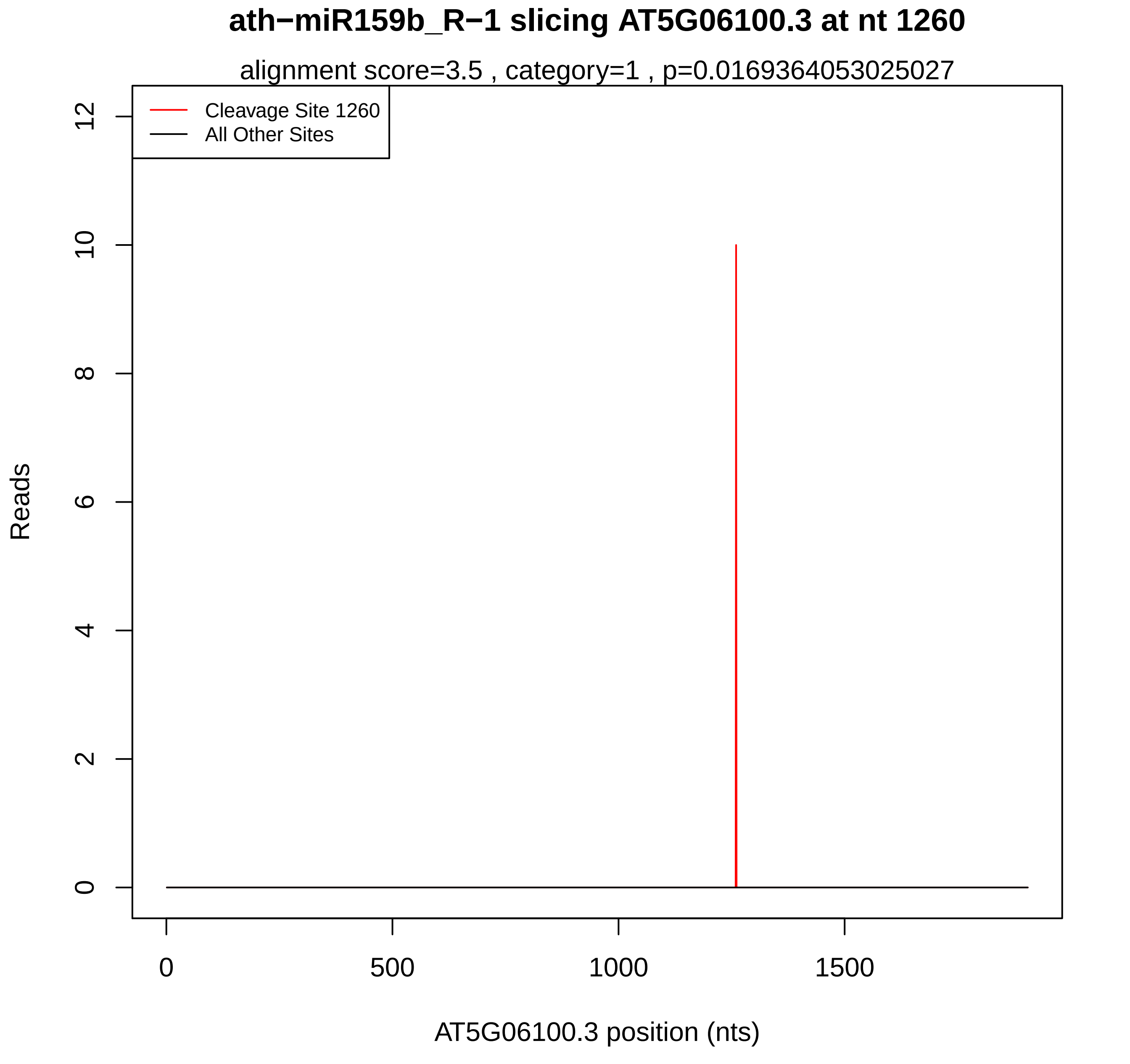
ath−miR159b_R−1 slicing AT5G06100.3 at nt 1260
alignment score=3.5 , category=1 , p=0.0169364053025027
Cleavage Site 1260
All Other Sites
Reads
0
2
4
6
8
10
12
0
500
1000
1500
AT5G06100.3 position (nts)

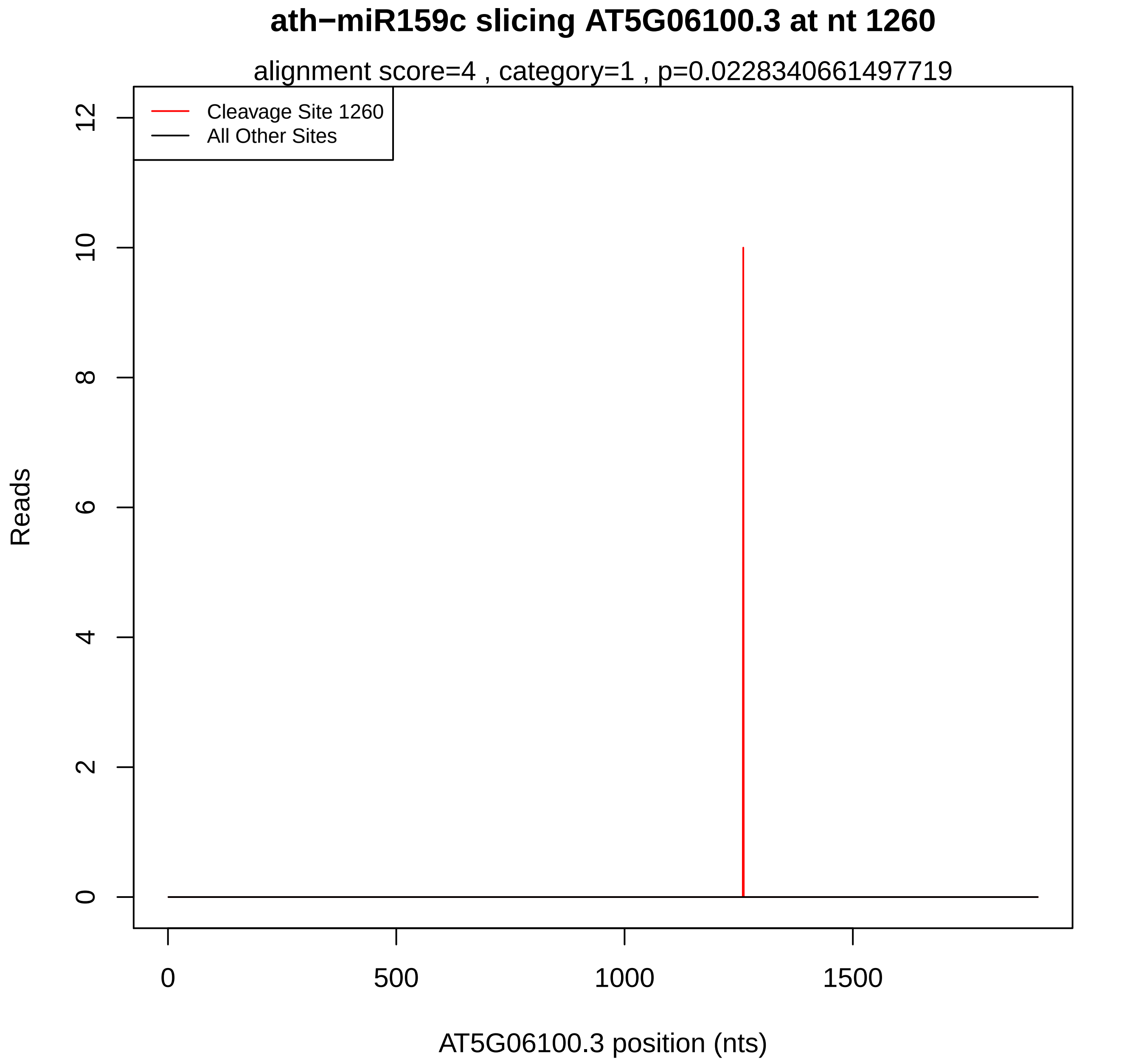

ath−miR159c slicing AT5G06100.3 at nt 1260
alignment score=4 , category=1 , p=0.0228340661497719
Cleavage Site 1260
All Other Sites
Reads
0
2
4
6
8
10
12
0
500
1000
1500
AT5G06100.3 position (nts)

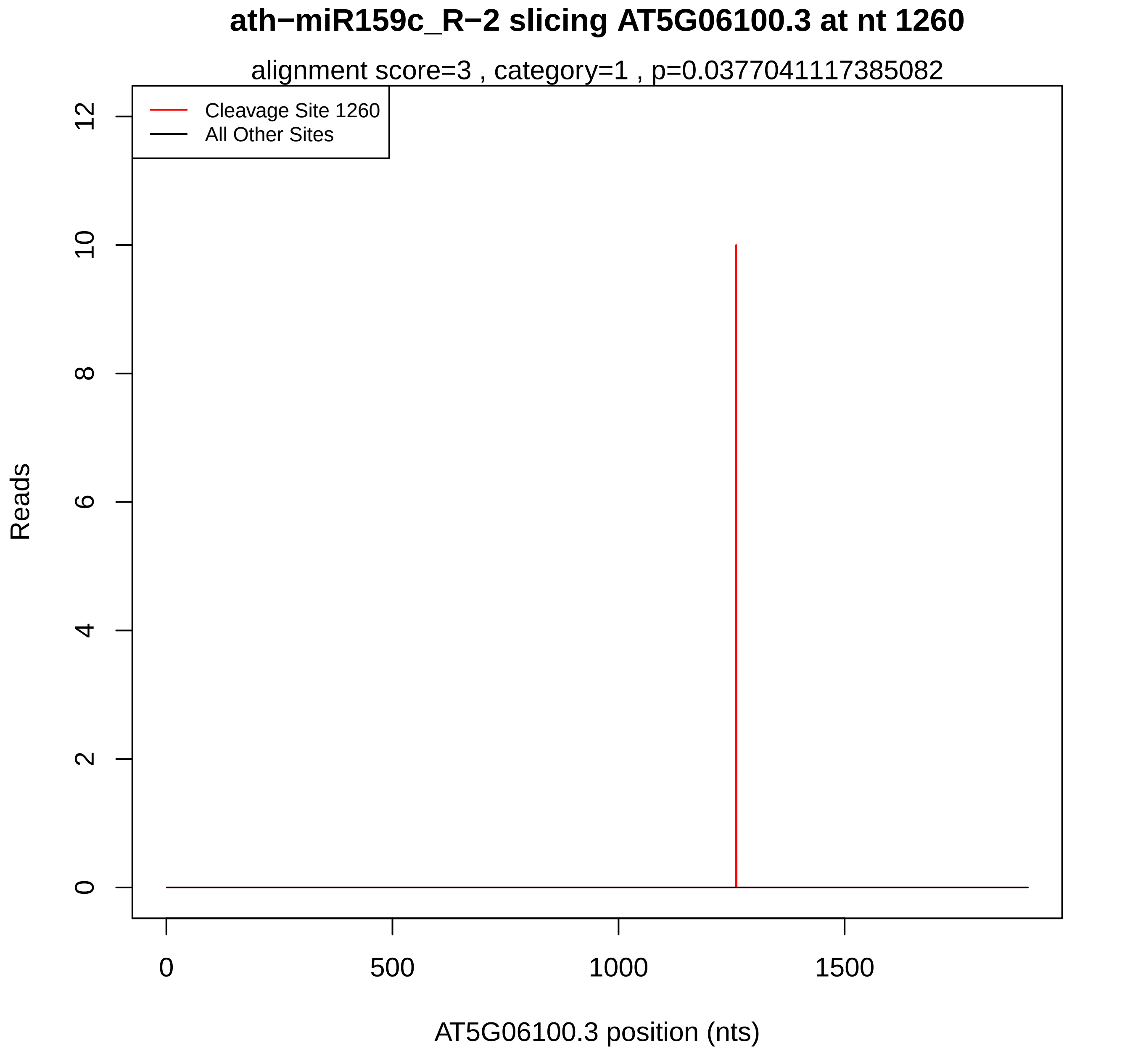
ath−miR159c_R−2 slicing AT5G06100.3 at nt 1260
alignment score=3 , category=1 , p=0.0377041117385082
Cleavage Site 1260
All Other Sites
Reads
0
2
4
6
8
10
12
0
500
1000
1500
AT5G06100.3 position (nts)

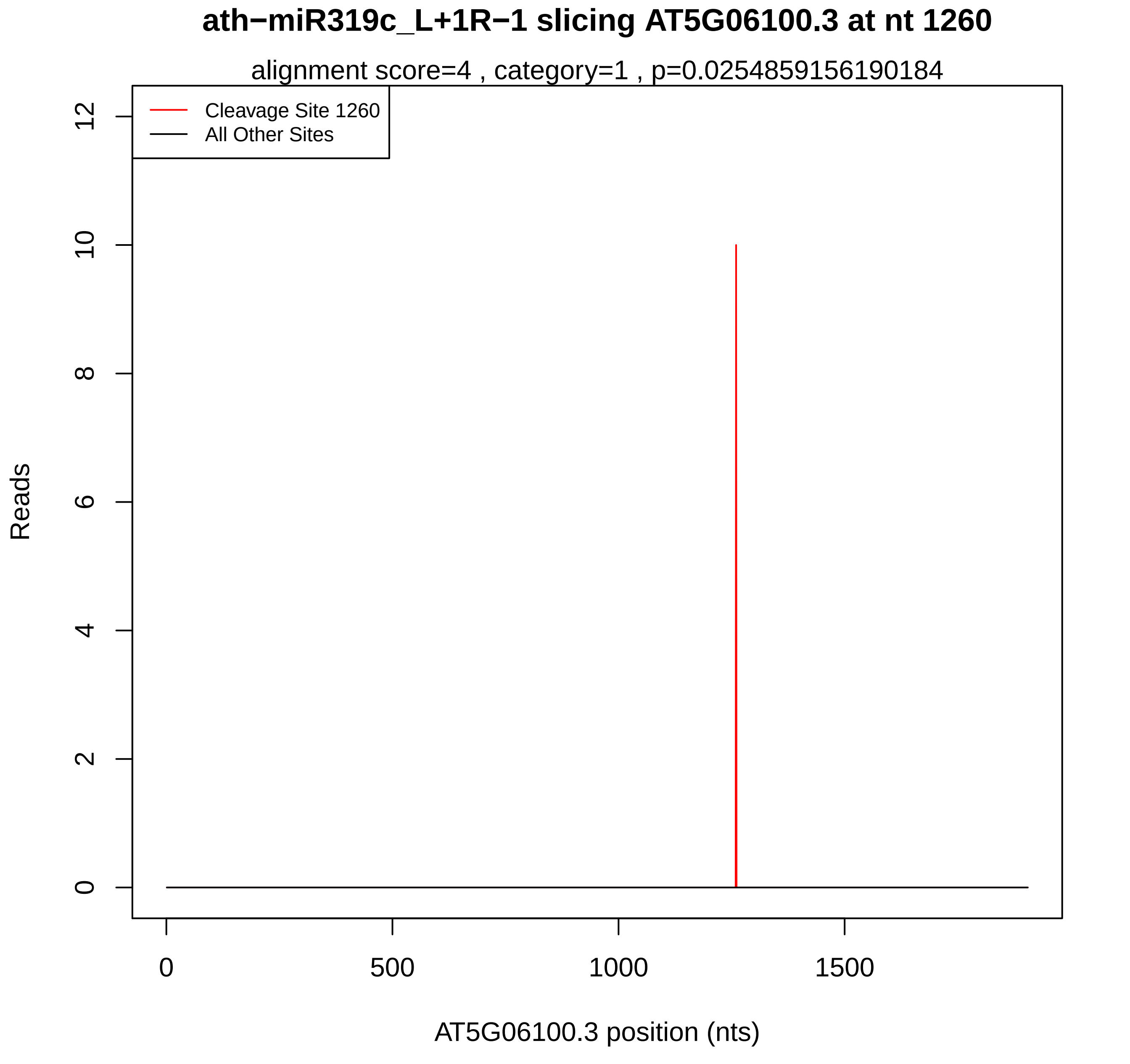
ath−miR319c_L+1R−1 slicing AT5G06100.3 at nt 1260
alignment score=4 , category=1 , p=0.0254859156190184
Cleavage Site 1260
All Other Sites
Reads
0
2
4
6
8
10
12
0
500
1000
1500
AT5G06100.3 position (nts)

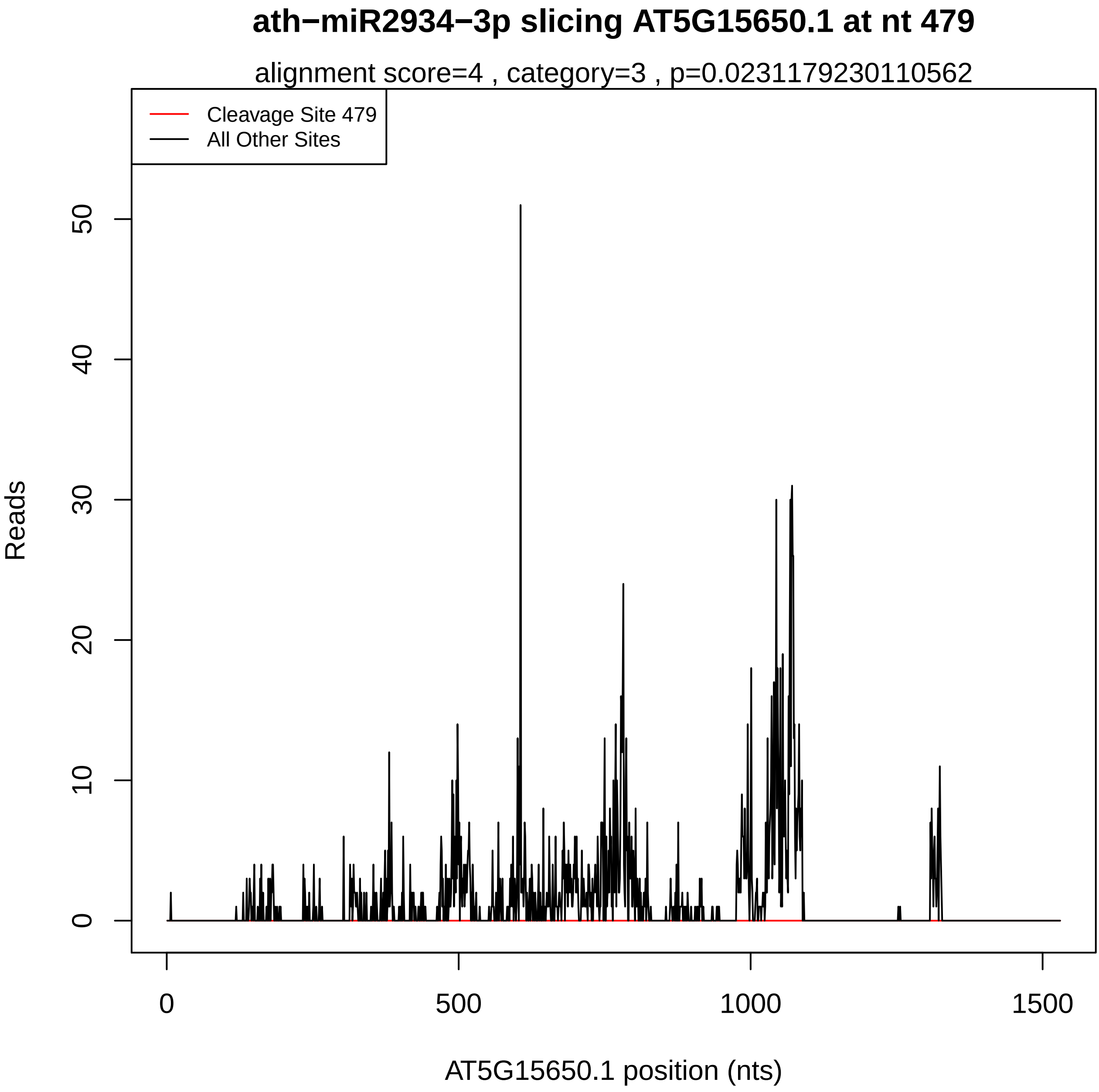

ath−miR2934−3p slicing AT5G15650.1 at nt 479
alignment score=4 , category=3 , p=0.0231179230110562
Cleavage Site 479
All Other Sites
Reads
0
10
20
30
40
50
0
500
1000
1500
AT5G15650.1 position (nts)

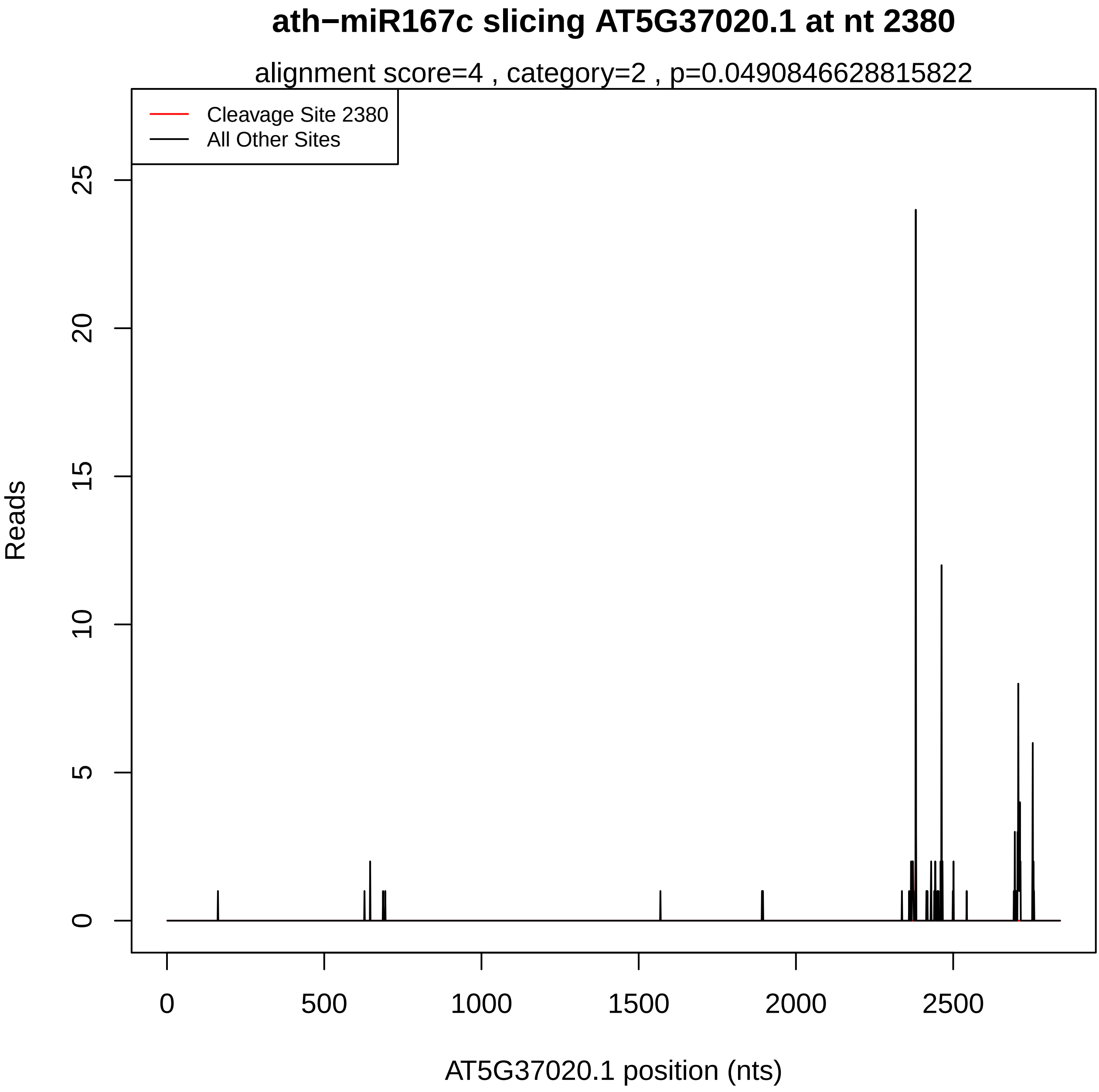

ath−miR167c slicing AT5G37020.1 at nt 2380
alignment score=4 , category=2 , p=0.0490846628815822
Cleavage Site 2380
All Other Sites
Reads
0
5
10
15
20
25
0
500
1000
1500
2000
2500
AT5G37020.1 position (nts)

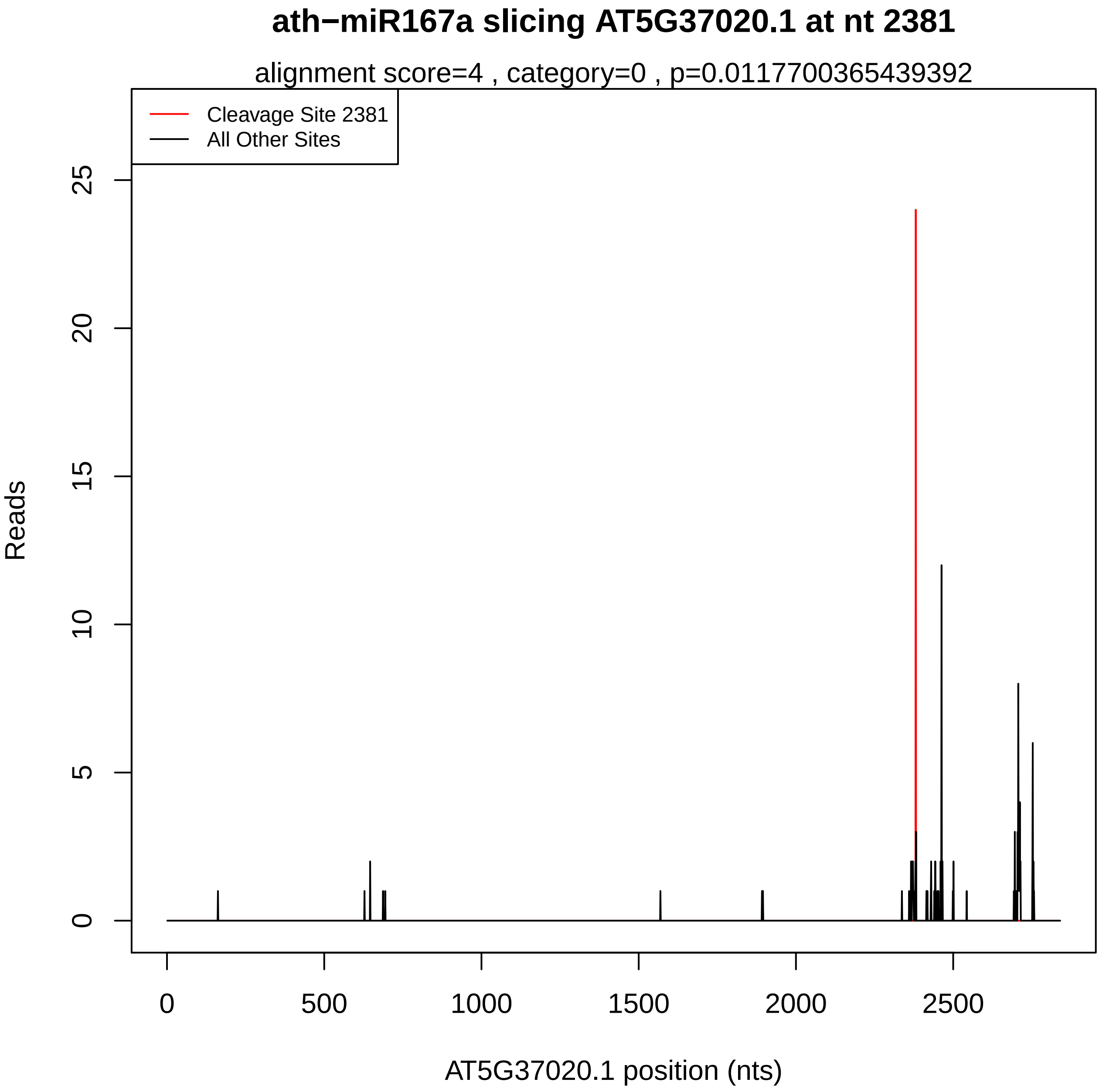

ath−miR167a slicing AT5G37020.1 at nt 2381
alignment score=4 , category=0 , p=0.0117700365439392
Cleavage Site 2381
All Other Sites
Reads
0
5
10
15
20
25
0
500
1000
1500
2000
2500
AT5G37020.1 position (nts)

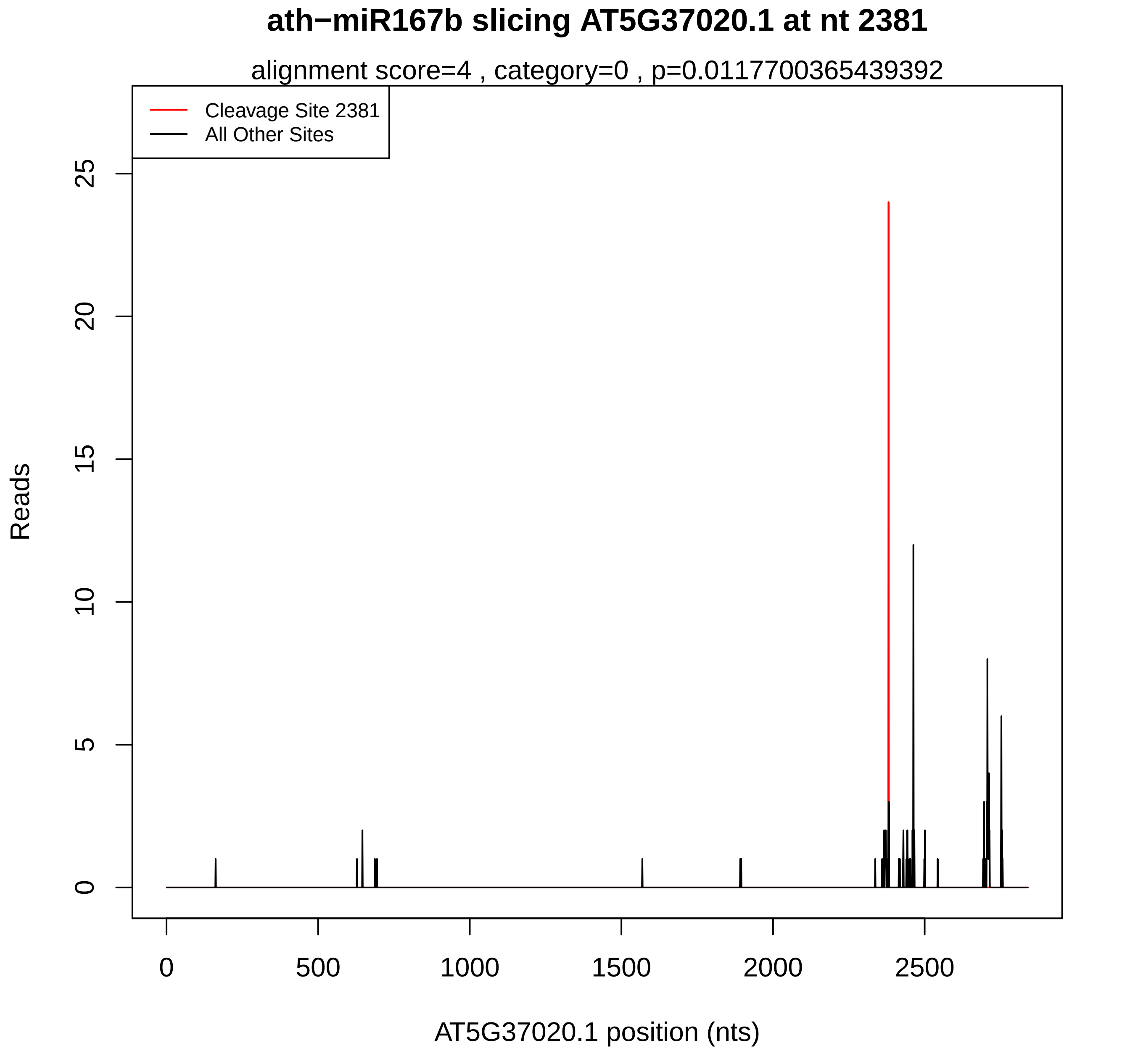
ath−miR167b slicing AT5G37020.1 at nt 2381
alignment score=4 , category=0 , p=0.0117700365439392
Cleavage Site 2381
All Other Sites
Reads
0
5
10
15
20
25
0
500
1000
1500
2000
2500
AT5G37020.1 position (nts)

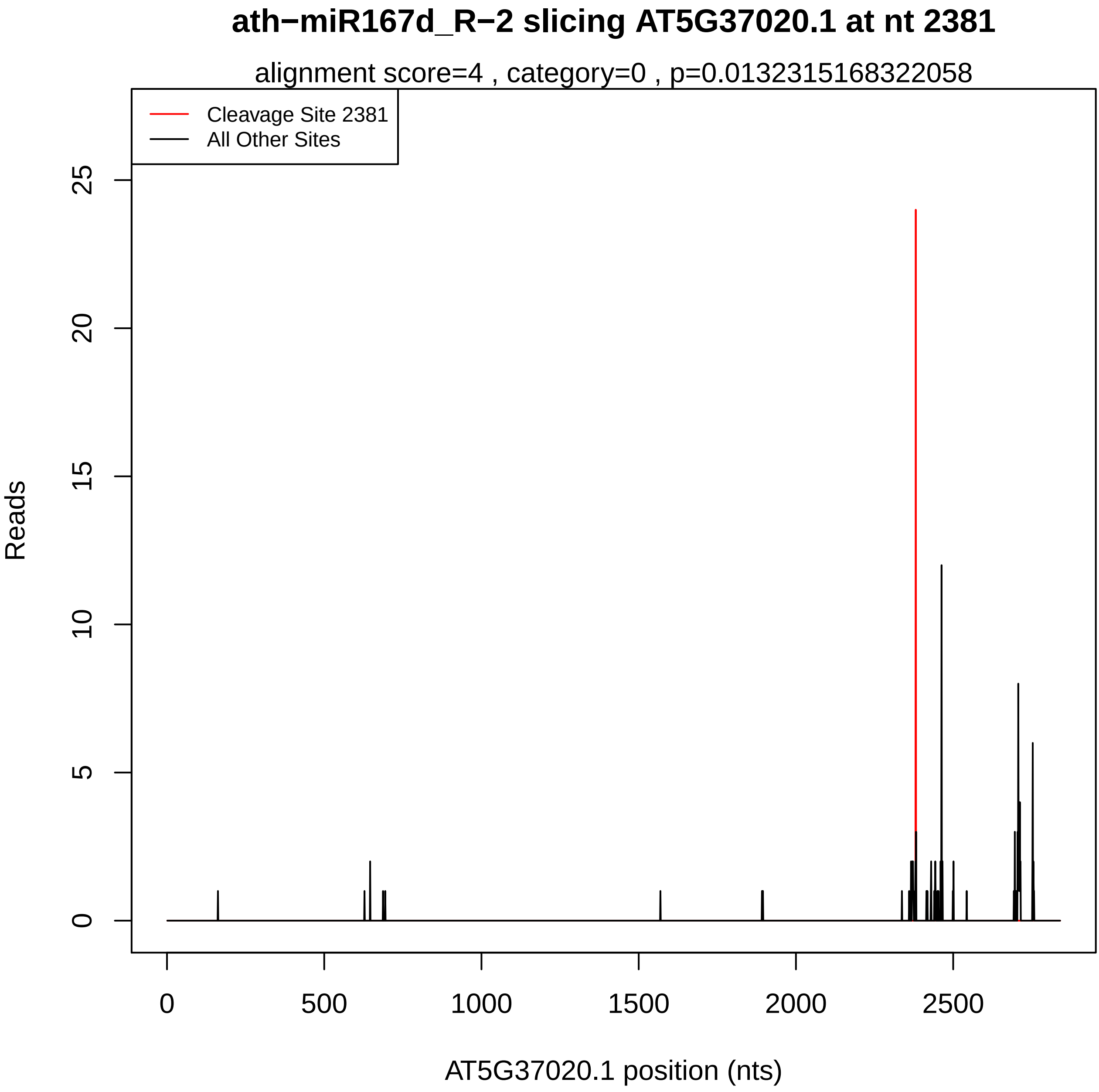
ath−miR167d_R−2 slicing AT5G37020.1 at nt 2381
alignment score=4 , category=0 , p=0.0132315168322058
Cleavage Site 2381
All Other Sites
Reads
AT5G37020.1 position (nts)
0
5
10
15
20
25
0
500
1000
1500
2000
2500

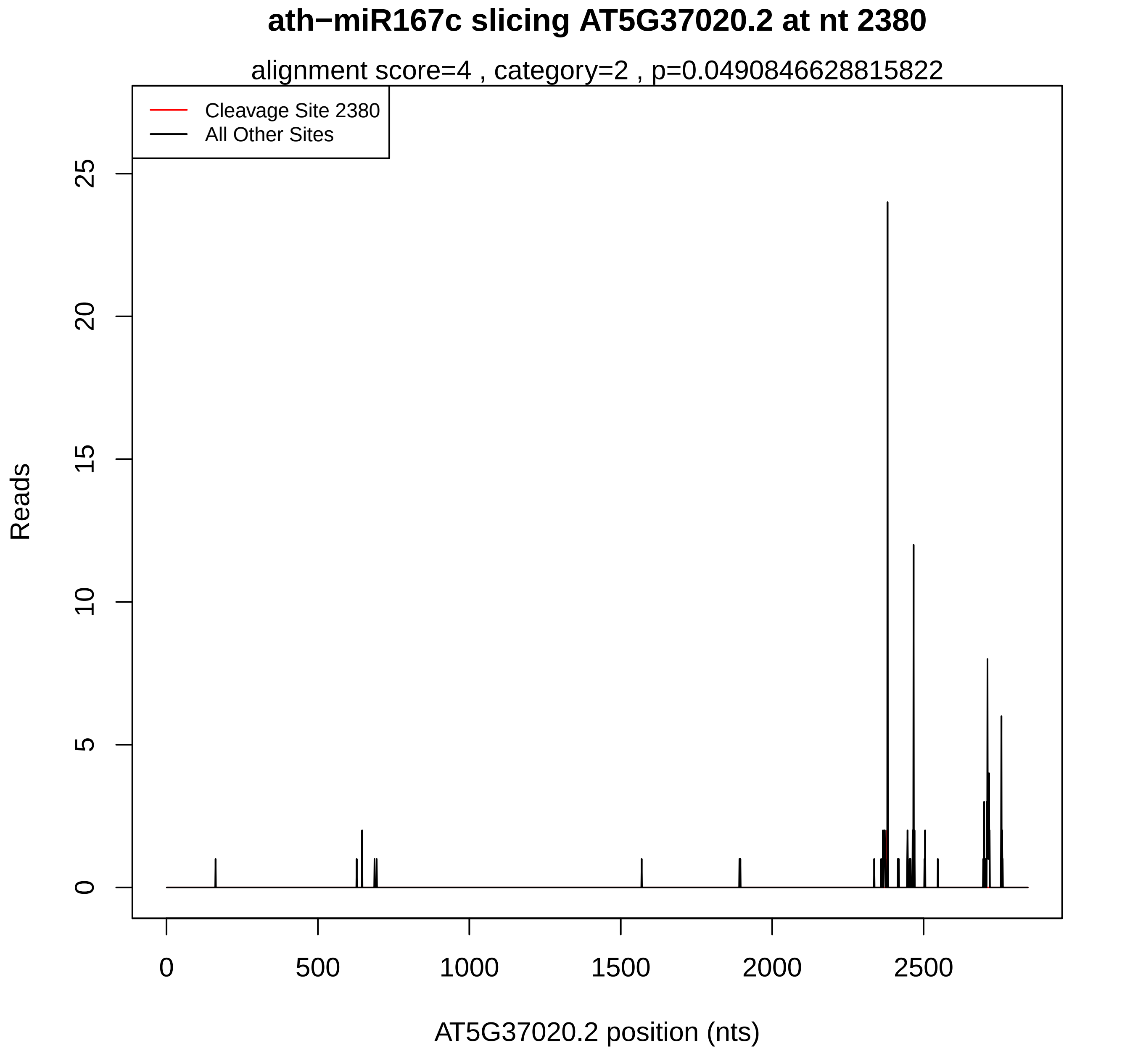
ath−miR167c slicing AT5G37020.2 at nt 2380
alignment score=4 , category=2 , p=0.0490846628815822
Cleavage Site 2380
All Other Sites
Reads
0
5
10
15
20
25
0
500
1000
1500
2000
2500
AT5G37020.2 position (nts)

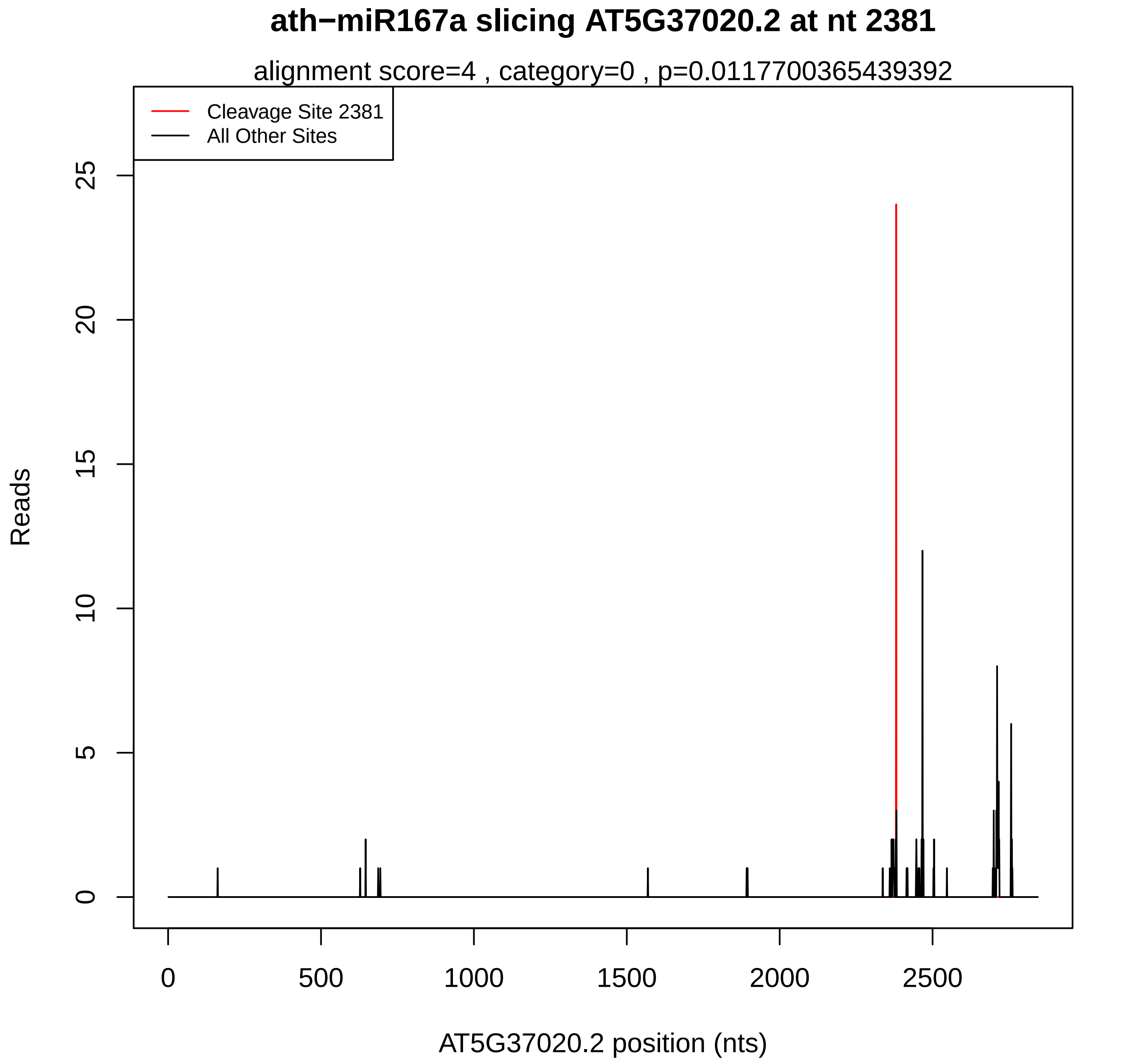

ath−miR167a slicing AT5G37020.2 at nt 2381
alignment score=4 , category=0 , p=0.0117700365439392
Cleavage Site 2381
All Other Sites
Reads
0
5
10
15
20
25
0
500
1000
1500
2000
2500
AT5G37020.2 position (nts)

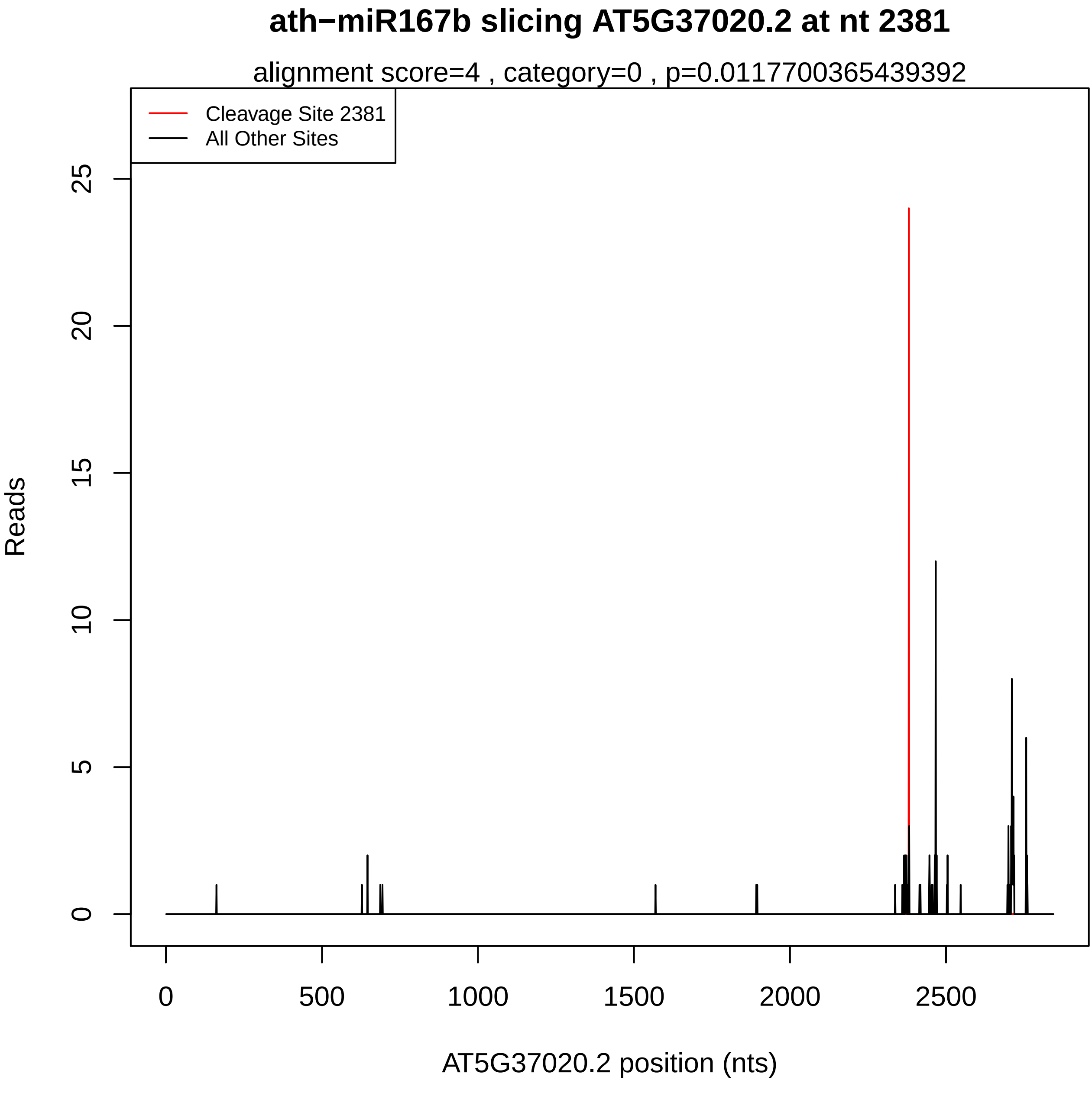
ath−miR167b slicing AT5G37020.2 at nt 2381
alignment score=4 , category=0 , p=0.0117700365439392
Cleavage Site 2381
All Other Sites
Reads
0
5
10
15
20
25
0
500
1000
1500
2000
2500
AT5G37020.2 position (nts)

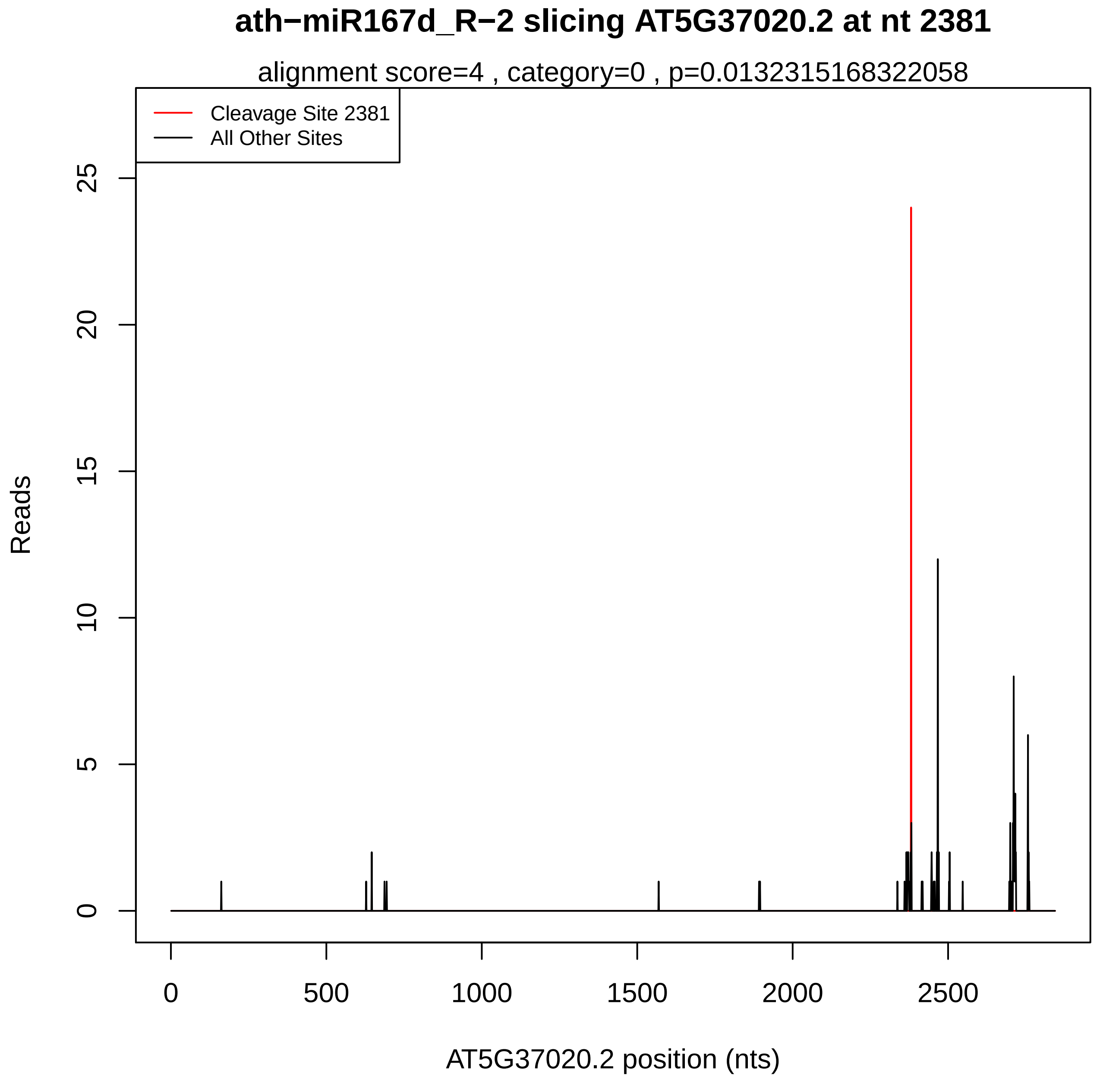
ath−miR167d_R−2 slicing AT5G37020.2 at nt 2381
alignment score=4 , category=0 , p=0.0132315168322058
Cleavage Site 2381
All Other Sites
Reads
0
5
10
15
20
25
0
500
1000
1500
2000
2500
AT5G37020.2 position (nts)

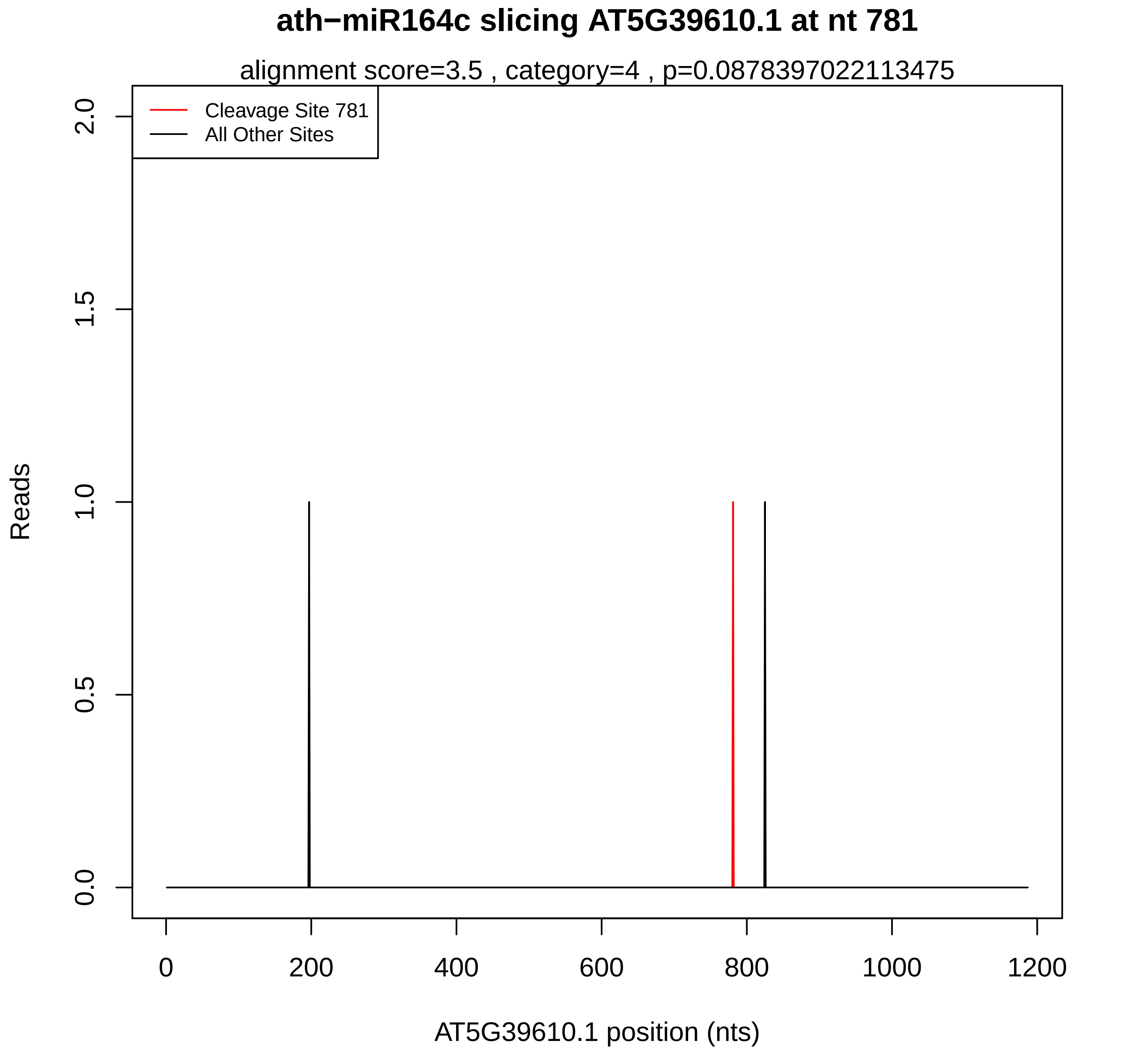
ath−miR164c slicing AT5G39610.1 at nt 781
alignment score=3.5 , category=4 , p=0.0878397022113475
Cleavage Site 781
All Other Sites
Reads
0.0
0.5
1.0
1.5
2.0
0
200
400
600
800
1000
1200
AT5G39610.1 position (nts)

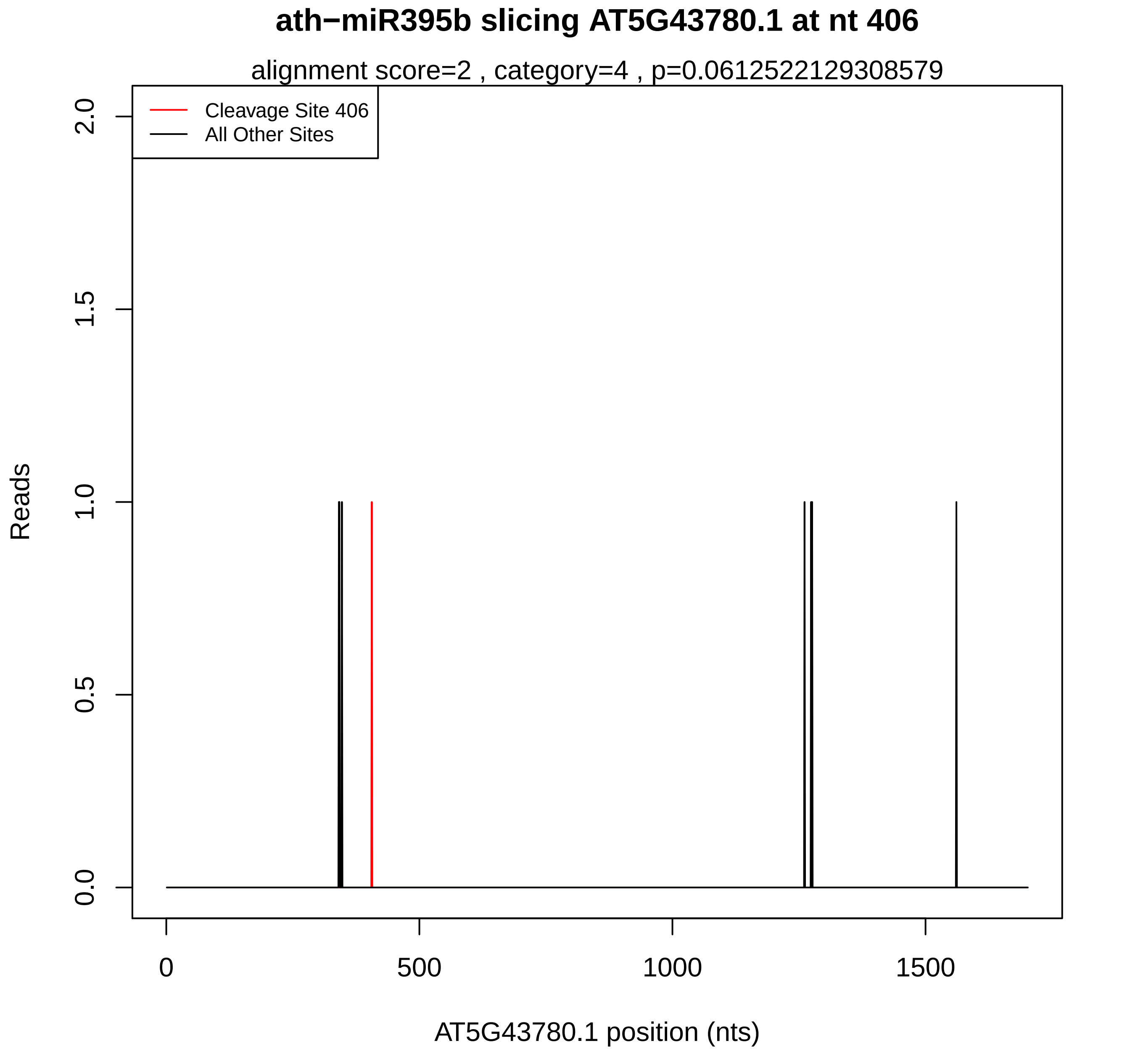

ath−miR395b slicing AT5G43780.1 at nt 406
alignment score=2 , category=4 , p=0.0612522129308579
Cleavage Site 406
All Other Sites
Reads
0.0
0.5
1.0
1.5
2.0
0
500
1000
1500
AT5G43780.1 position (nts)

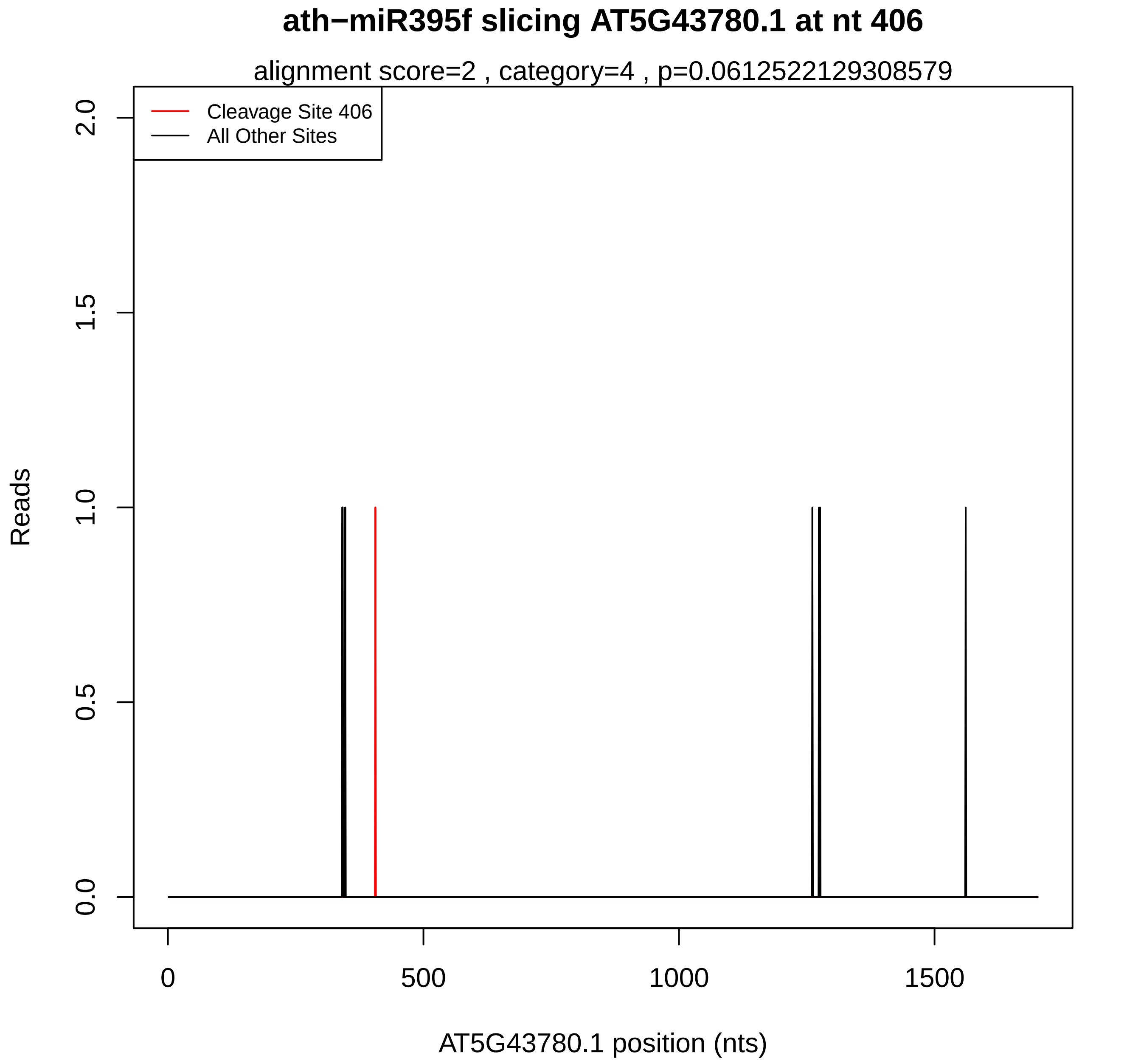

ath−miR395f slicing AT5G43780.1 at nt 406
alignment score=2 , category=4 , p=0.0612522129308579
Cleavage Site 406
All Other Sites
Reads
0.0
0.5
1.0
1.5
2.0
0
500
1000
1500
AT5G43780.1 position (nts)

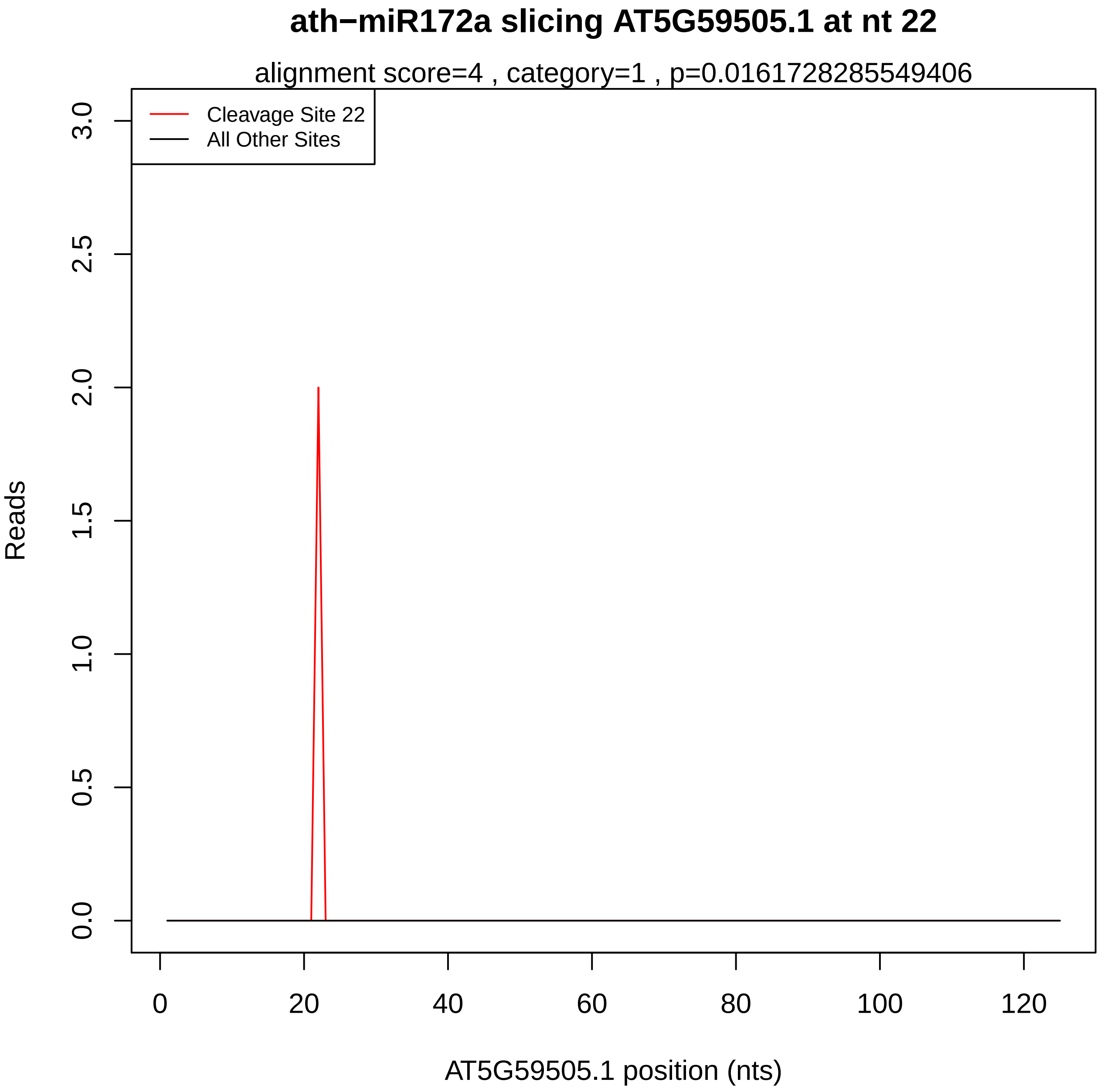
ath−miR172a slicing AT5G59505.1 at nt 22
alignment score=4 , category=1 , p=0.0161728285549406
Cleavage Site 22
All Other Sites
Reads
0.0
0.5
1.0
1.5
2.0
2.5
3.0
0
20
40
60
80
100
120
AT5G59505.1 position (nts)

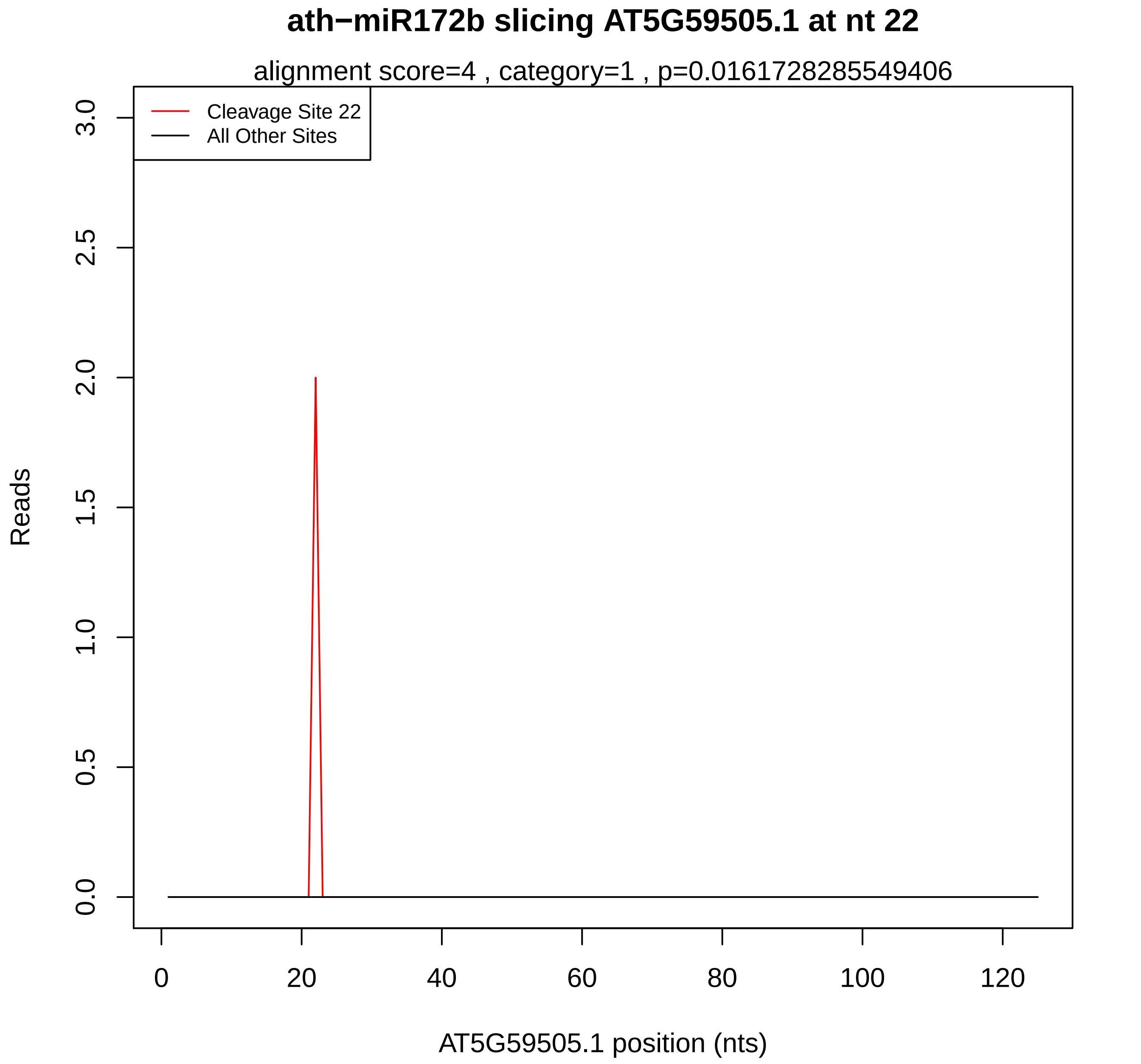
ath−miR172b slicing AT5G59505.1 at nt 22
alignment score=4 , category=1 , p=0.0161728285549406
Cleavage Site 22
All Other Sites
Reads
0.0
0.5
1.0
1.5
2.0
2.5
3.0
0
20
40
60
80
100
120
AT5G59505.1 position (nts)

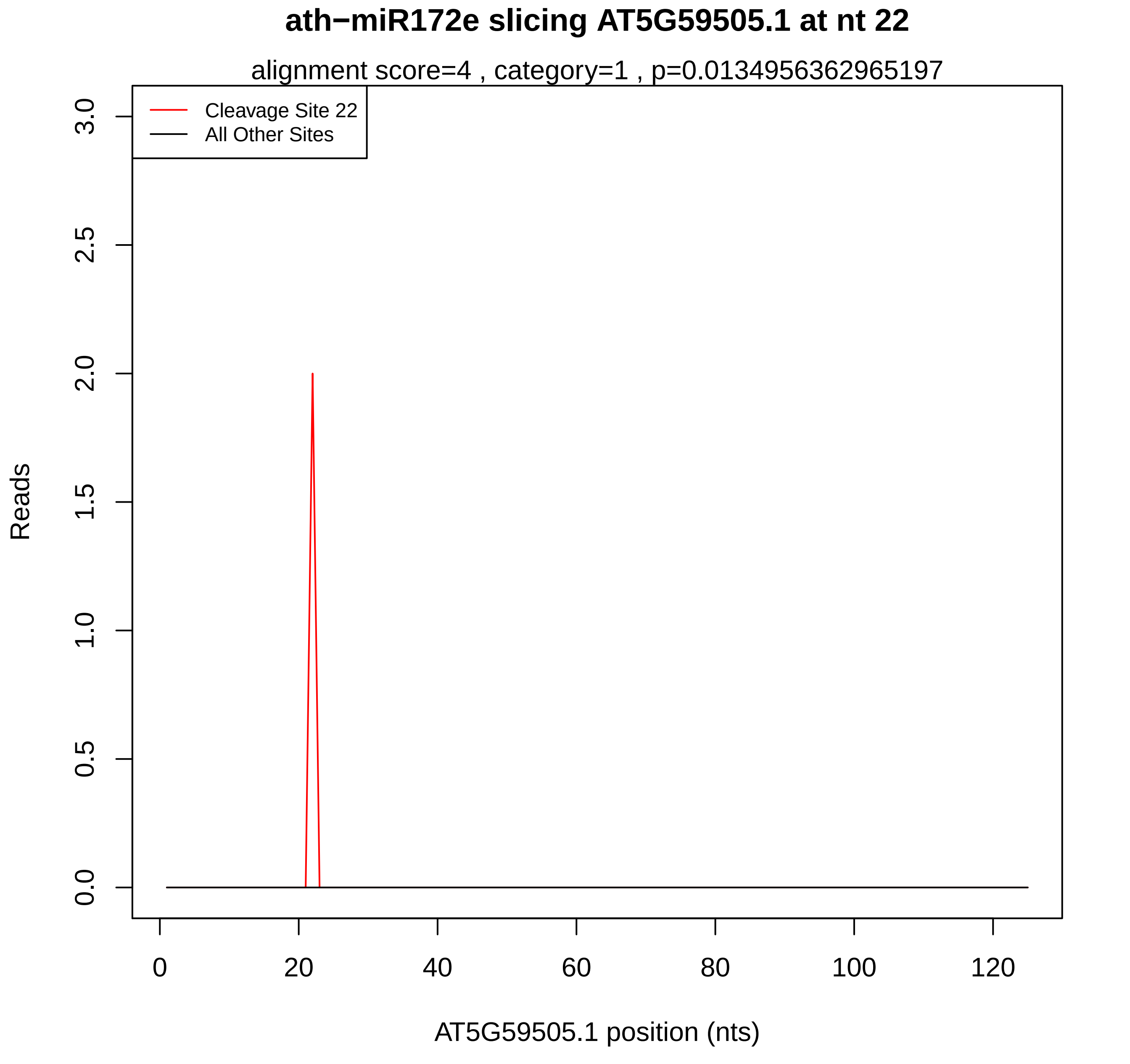
ath−miR172e slicing AT5G59505.1 at nt 22
alignment score=4 , category=1 , p=0.0134956362965197
Cleavage Site 22
All Other Sites
Reads
0.0
0.5
1.0
1.5
2.0
2.5
3.0
0
20
40
60
80
100
120
AT5G59505.1 position (nts)

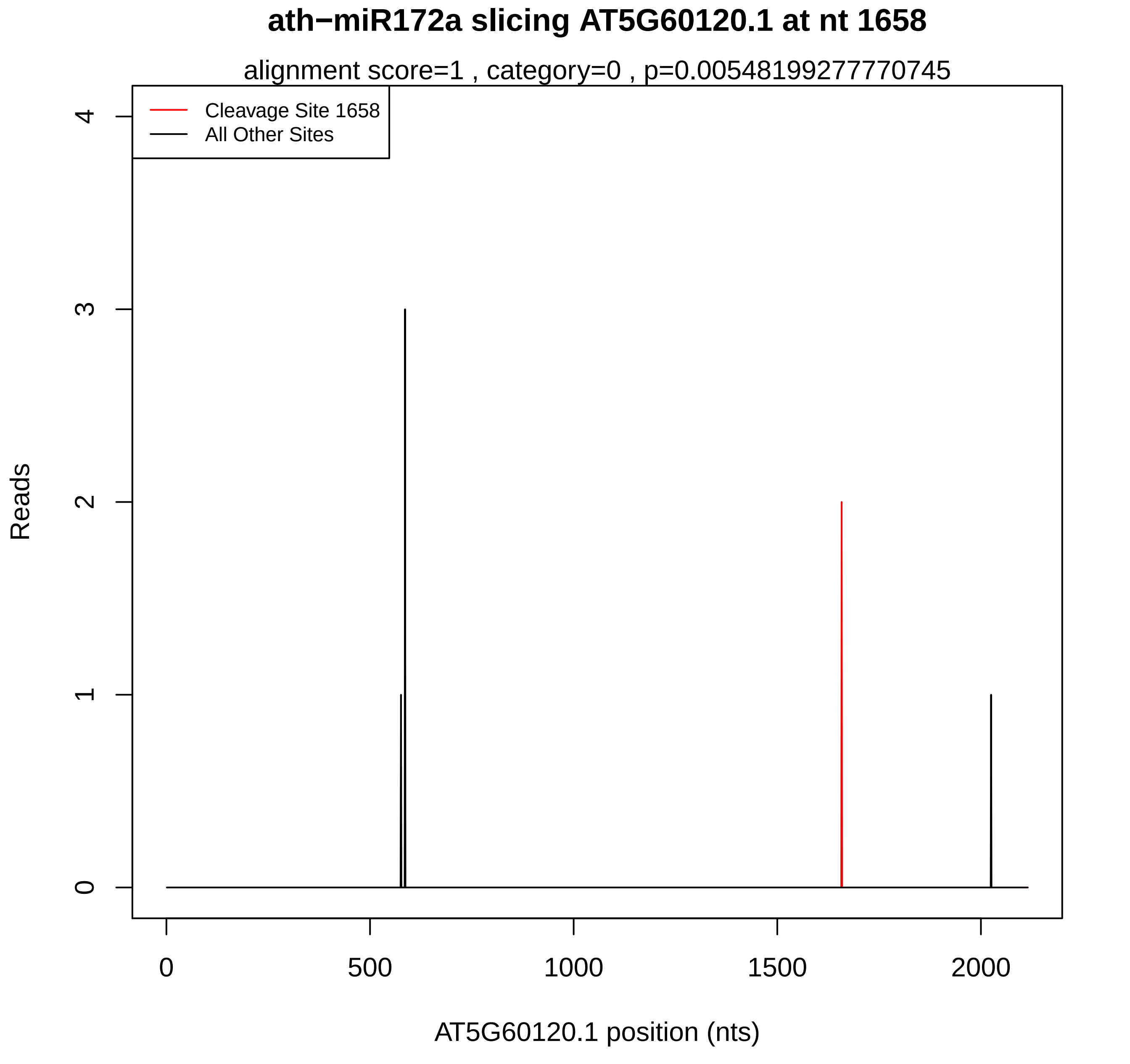
ath−miR172a slicing AT5G60120.1 at nt 1658
alignment score=1 , category=0 , p=0.00548199277770745
Cleavage Site 1658
All Other Sites
Reads
0
1
2
3
4
0
500
1000
1500
2000
AT5G60120.1 position (nts)

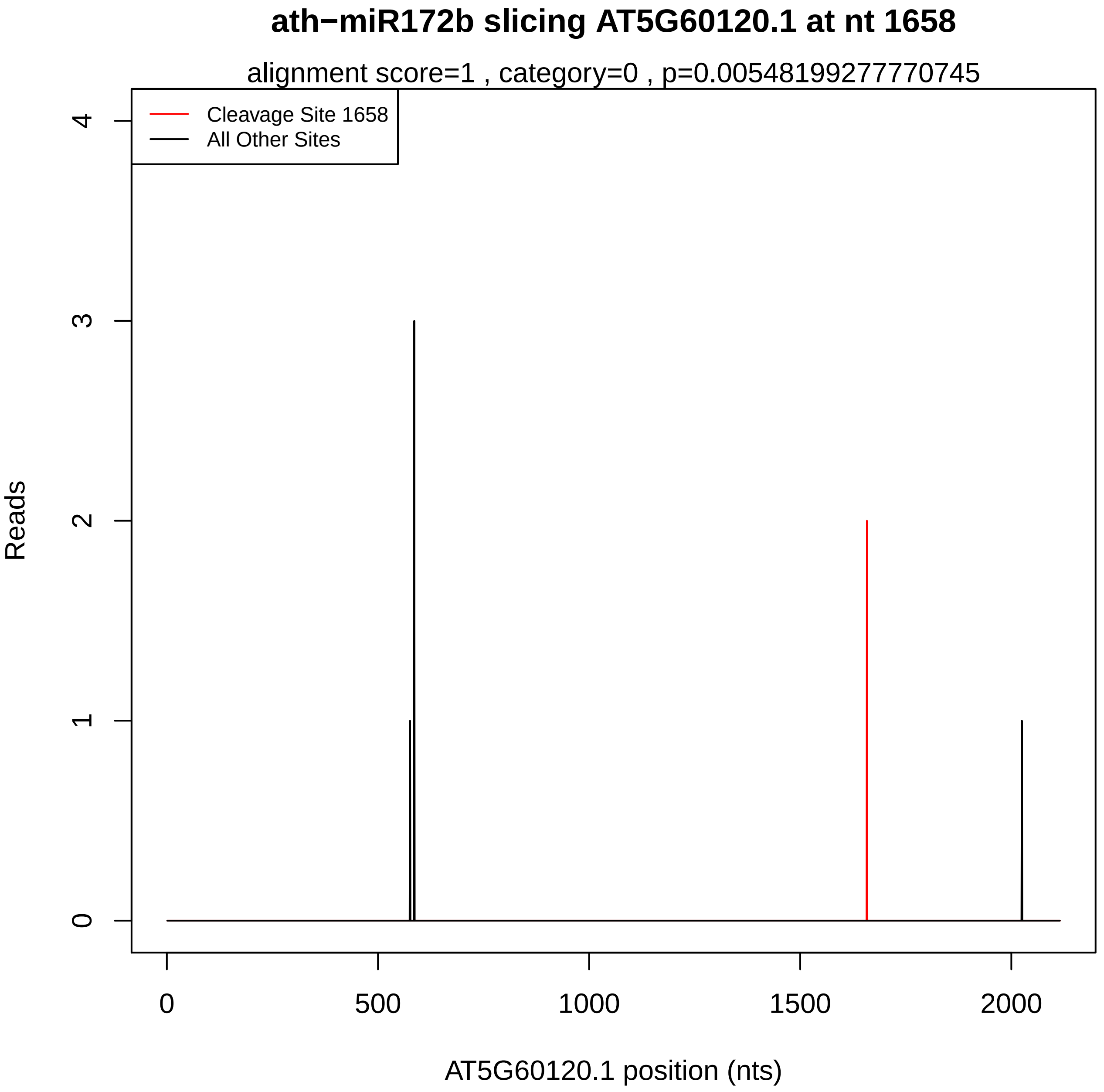
ath−miR172b slicing AT5G60120.1 at nt 1658
alignment score=1 , category=0 , p=0.00548199277770745
Cleavage Site 1658
All Other Sites
Reads
0
1
2
3
4
0
500
1000
1500
2000
AT5G60120.1 position (nts)

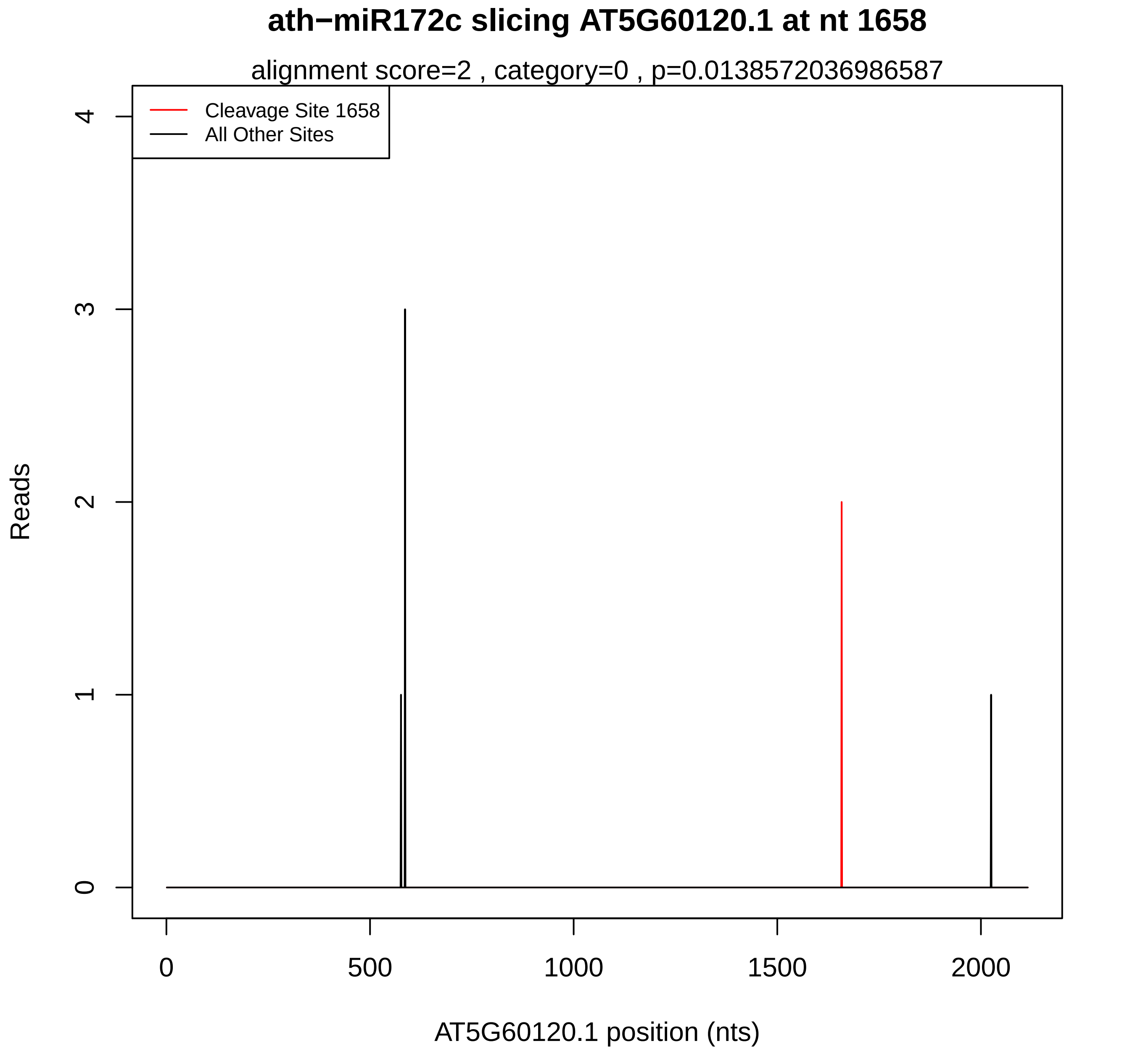
ath−miR172c slicing AT5G60120.1 at nt 1658
alignment score=2 , category=0 , p=0.0138572036986587
Cleavage Site 1658
All Other Sites
Reads
0
1
2
3
4
0
500
1000
1500
2000
AT5G60120.1 position (nts)

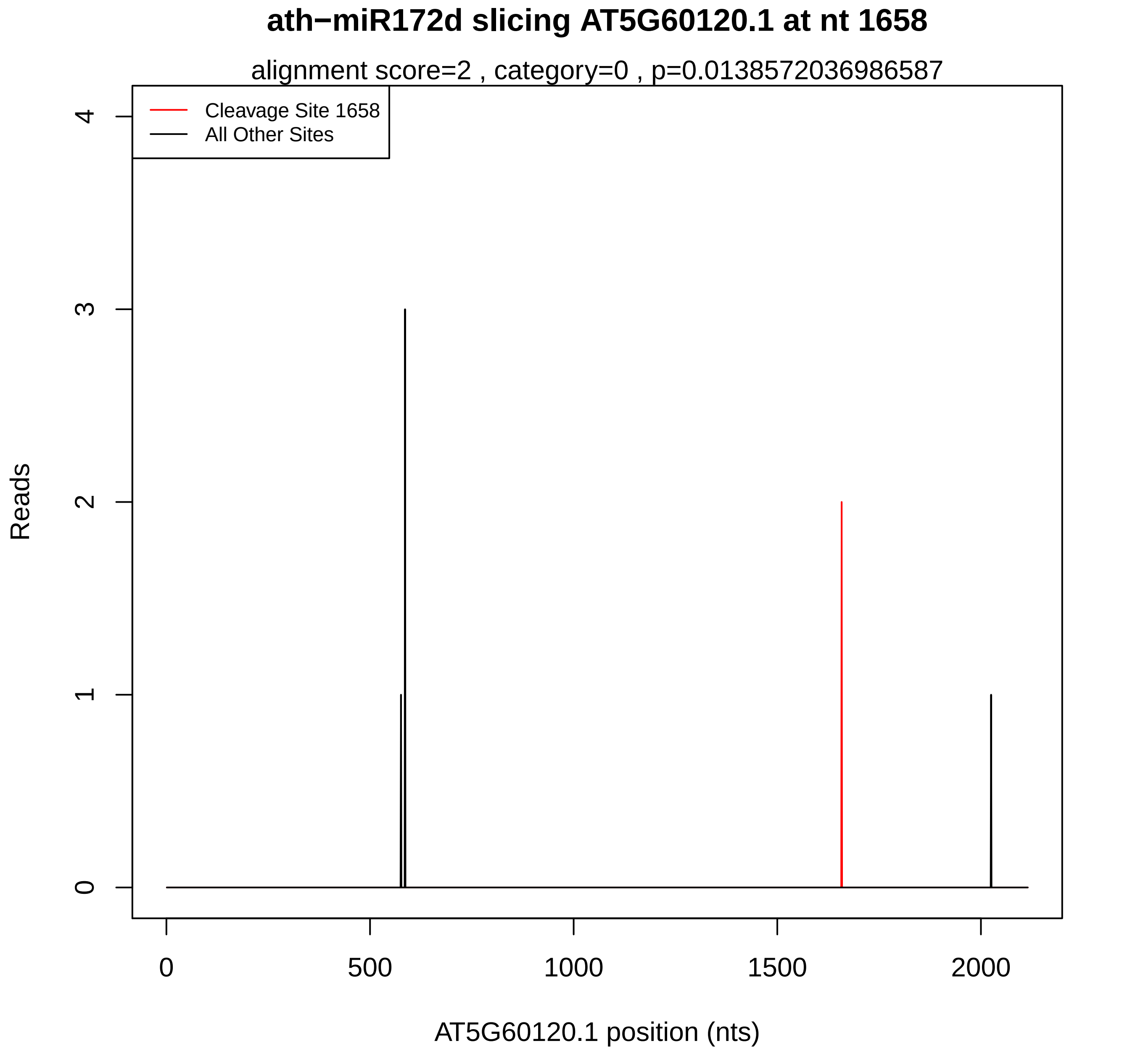
ath−miR172d slicing AT5G60120.1 at nt 1658
alignment score=2 , category=0 , p=0.0138572036986587
Cleavage Site 1658
All Other Sites
Reads
0
1
2
3
4
0
500
1000
1500
2000
AT5G60120.1 position (nts)

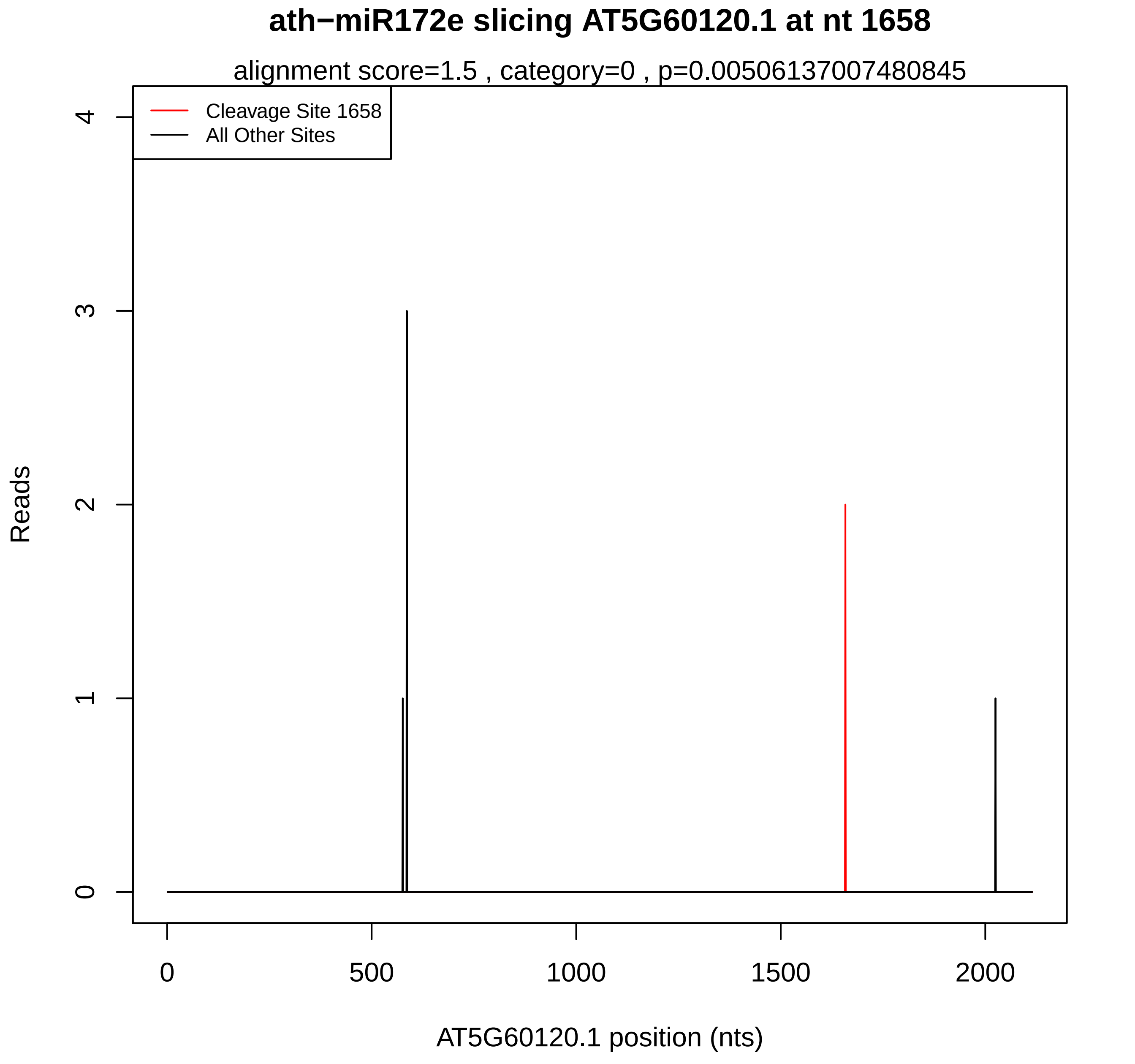
ath−miR172e slicing AT5G60120.1 at nt 1658
alignment score=1.5 , category=0 , p=0.00506137007480845
Cleavage Site 1658
All Other Sites
Reads
0
1
2
3
4
0
500
1000
1500
2000
AT5G60120.1 position (nts)

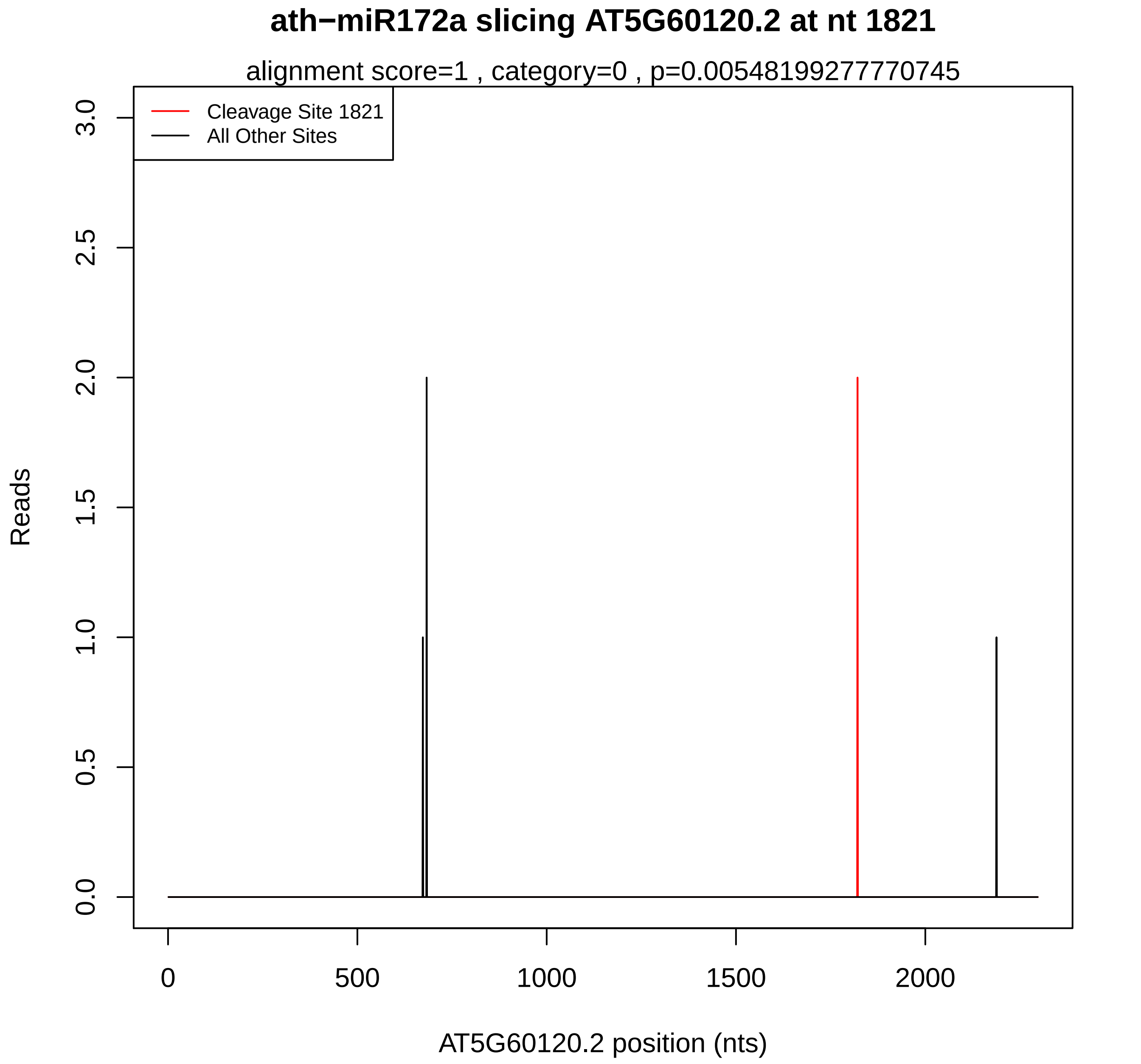

ath−miR172a slicing AT5G60120.2 at nt 1821
alignment score=1 , category=0 , p=0.00548199277770745
Cleavage Site 1821
All Other Sites
Reads
0.0
0.5
1.0
1.5
2.0
2.5
3.0
0
500
1000
1500
2000
AT5G60120.2 position (nts)

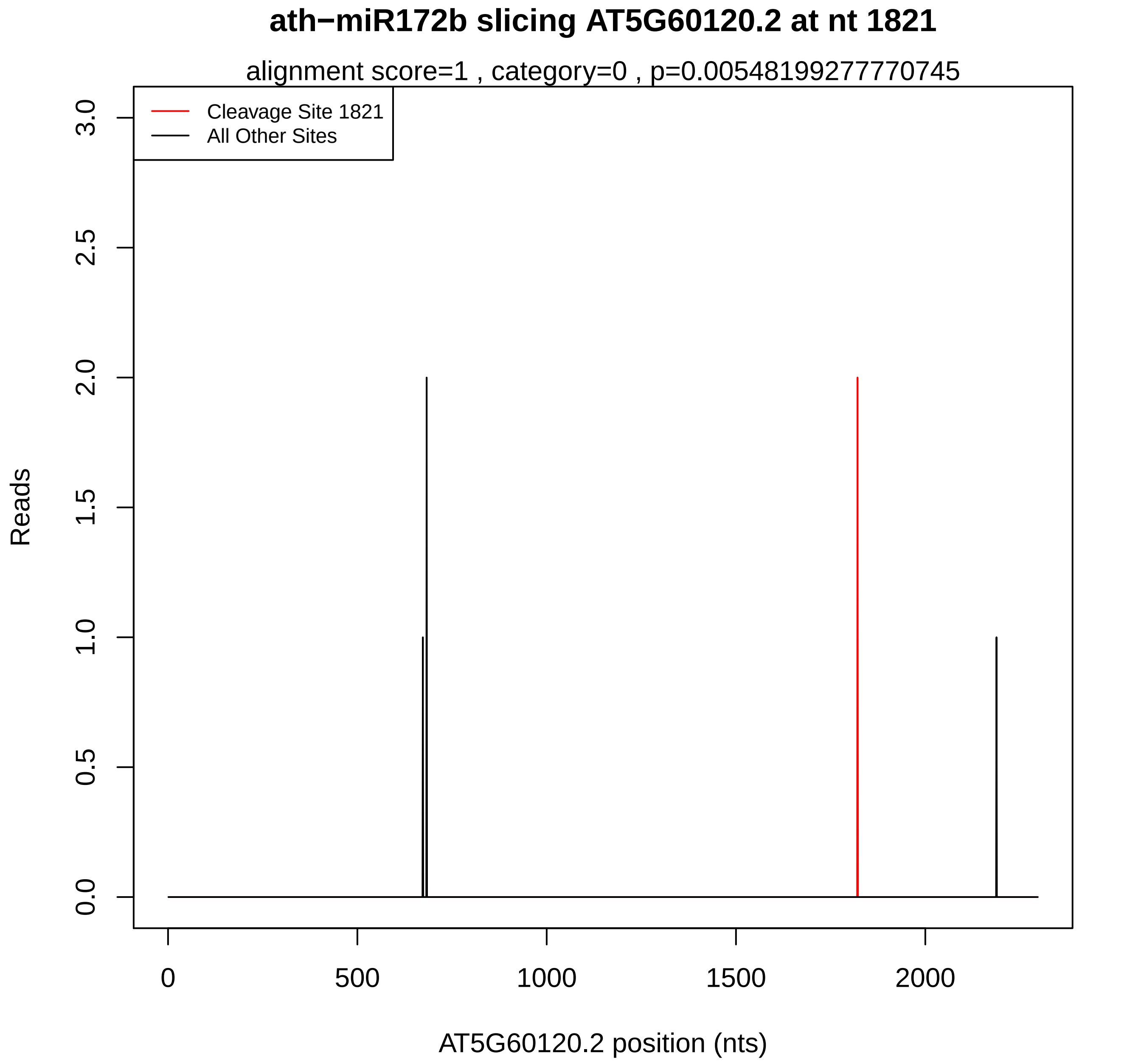
ath−miR172b slicing AT5G60120.2 at nt 1821
alignment score=1 , category=0 , p=0.00548199277770745
Cleavage Site 1821
All Other Sites
Reads
0.0
0.5
1.0
1.5
2.0
2.5
3.0
0
500
1000
1500
2000
AT5G60120.2 position (nts)

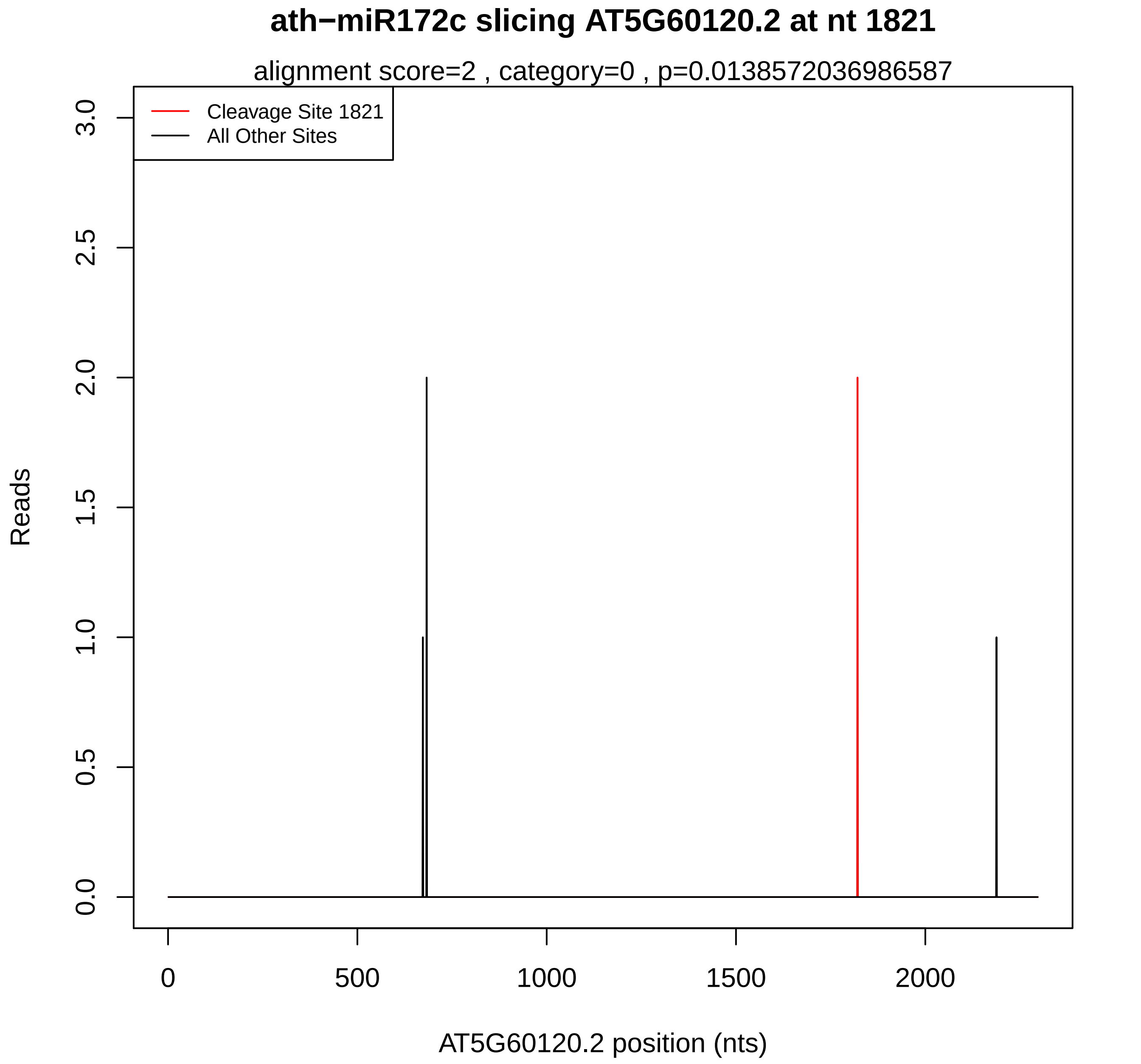

ath−miR172c slicing AT5G60120.2 at nt 1821
alignment score=2 , category=0 , p=0.0138572036986587
Cleavage Site 1821
All Other Sites
Reads
0.0
0.5
1.0
1.5
2.0
2.5
3.0
0
500
1000
1500
2000
AT5G60120.2 position (nts)

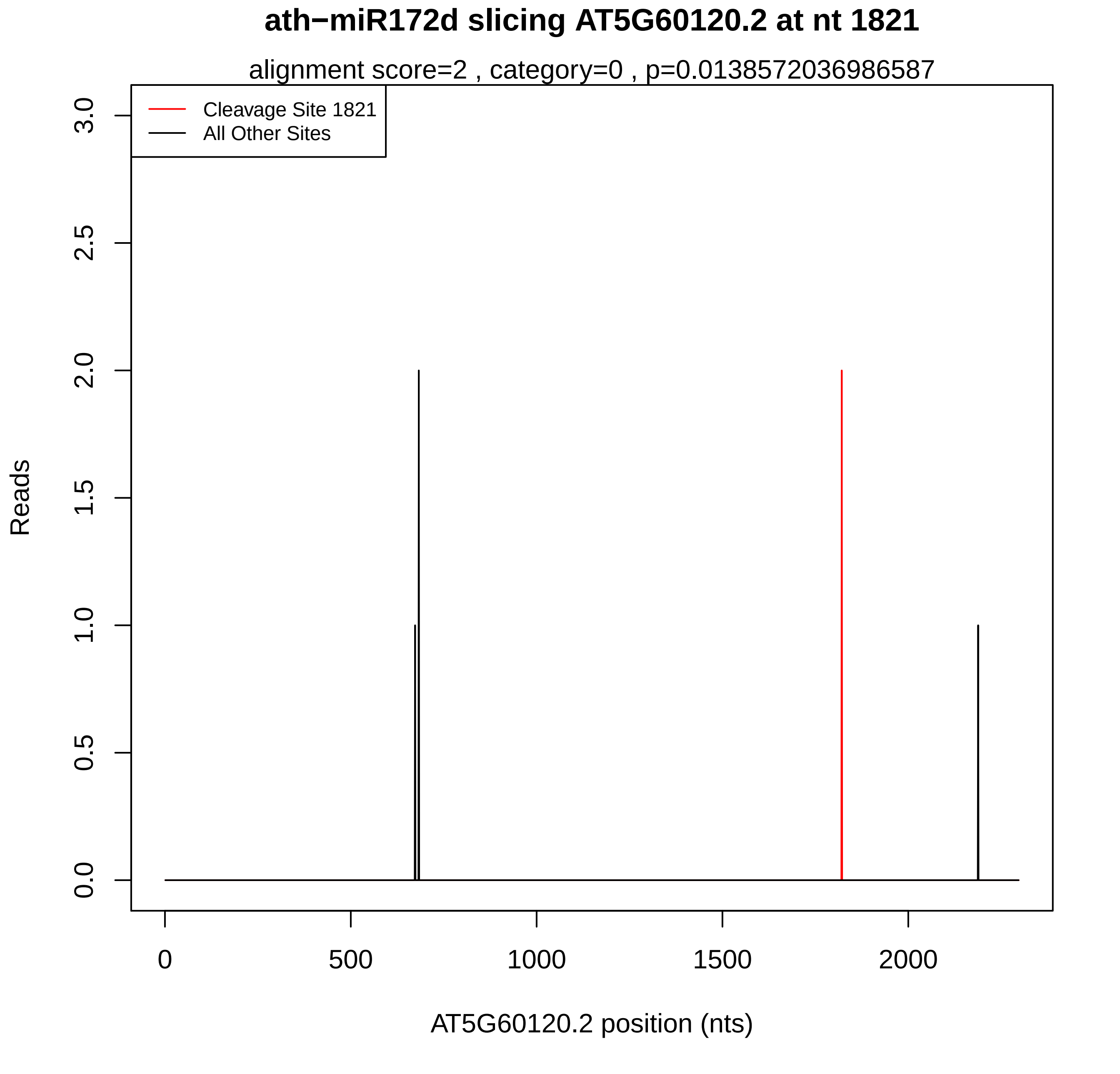
ath−miR172d slicing AT5G60120.2 at nt 1821
alignment score=2 , category=0 , p=0.0138572036986587
Cleavage Site 1821
All Other Sites
Reads
0.0
0.5
1.0
1.5
2.0
2.5
3.0
0
500
1000
1500
2000
AT5G60120.2 position (nts)

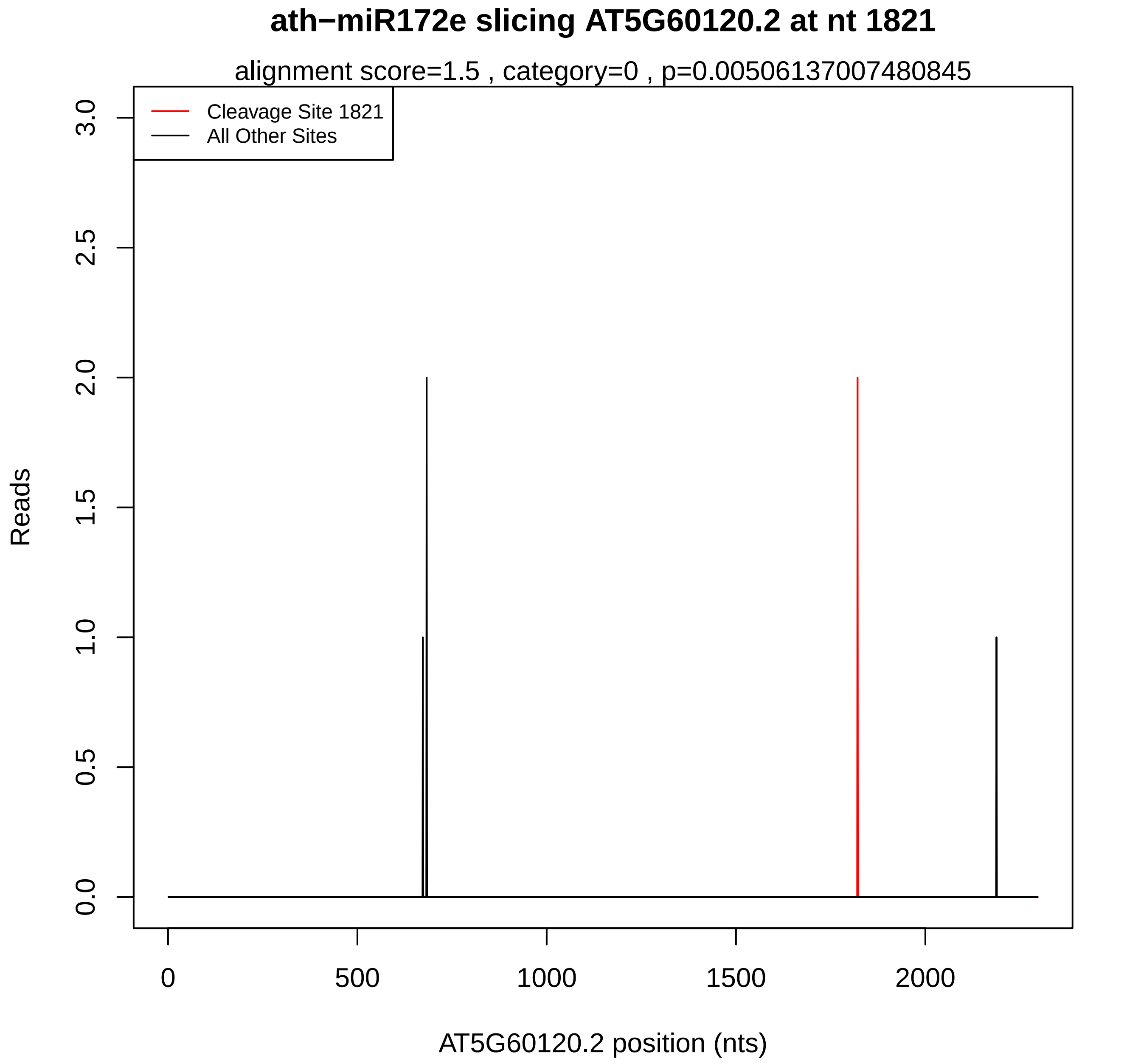

ath−miR172e slicing AT5G60120.2 at nt 1821
alignment score=1.5 , category=0 , p=0.00506137007480845
Cleavage Site 1821
All Other Sites
Reads
0.0
0.5
1.0
1.5
2.0
2.5
3.0
0
500
1000
1500
2000
AT5G60120.2 position (nts)
